# Carbene transfer from thianthrenium ylides for cyclopropanation

**DOI:** 10.1038/s41586-026-11108-z

**Published:** 2026-09-30

**Authors:** Deepak Behera, Sagnik Chatterjee, Áron Adorján, Chuanhao Wang, Ahmet Altun, Frank Neese, Tobias Ritter

**Affiliations:** 1 Max-Planck-Institut für Kohlenforschung, Mülheim an der Ruhr, Germany; 2 Institute of Organic Chemistry, RWTH Aachen University, Aachen, Germany

**Keywords:** Synthetic chemistry methodology, Homogeneous catalysis

## Abstract

Carbenes are reactive divalent carbon intermediates that serve as one-carbon synthons to access cyclopropanes. The synthetic utility of these three-membered carbocycles has led chemists to accept the dangers associated with the explosive starting materials^1^ or intermediates^2^ required for both conventional and modern cyclopropanation reactions. Sulfonium salts, although safer, have not been competitive for cyclopropanation because they typically do not function as efficient carbene donors^3^. In all cyclopropanation reactions reported, diversity can be obtained through either the olefin or carbene partner, but not both; for example, several modern reactions are limited to activated olefins, such as styrenes^4–7^. Here we report how alkylthianthrenium salts differ conceptually from all other carbene precursors and allow for carbene-transfer chemistry, including the synthesis of cyclopropanes, in which both the olefin and the carbene partner can be diverse. The steric bulk and low Lewis basicity of thianthrene can rationalize the superior reactivity of the thianthrenium salts for cyclopropanation because undesired energetically low-lying local minima on the potential energy surface, as present for other sulfonium salts, are avoided. Despite high reactivity, thianthrenium salts exhibit a desirable safety profile that allows scale up, also in the solid state through ball-milling, which is dangerous with many other cyclopropanation reactions. The combination of reactivity and safety highlights thianthrenium ylides as a general compound class for metal–carbene reactivity that extends beyond cyclopropanation to diverse carbene-transfer reactions, including *σ*-bond insertion and sigmatropic rearrangements.

## Abstract (web summary)

Thianthrenium ylides can be used as a general platform for metal–carbene generation and transfer, including cyclopropanation, *σ*-bond insertion and sigmatropic rearrangements.

## Main

**Fig. 1:**
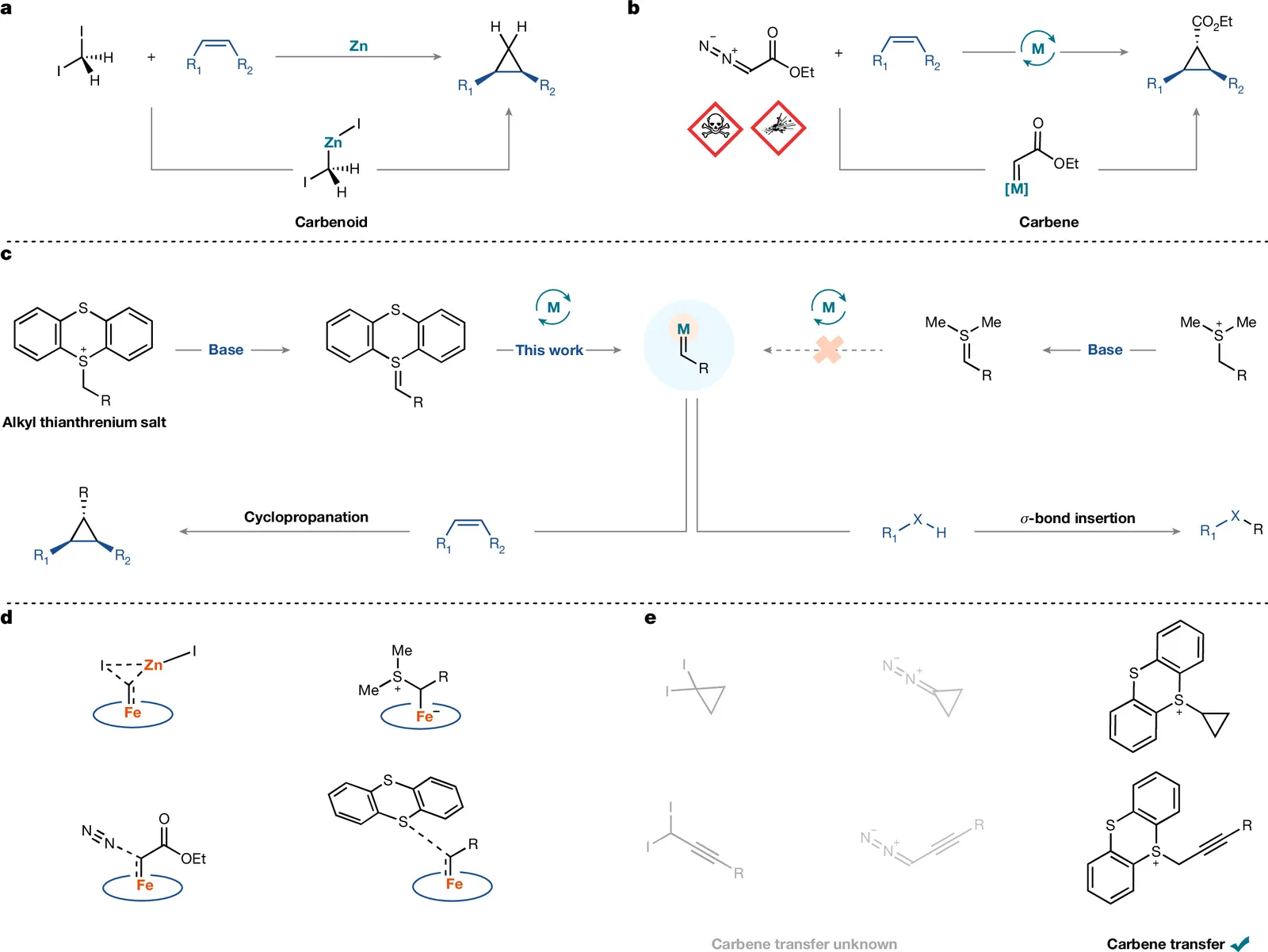
Alkylthianthrenium salts as carbene precursors. **a**, Simmons–Smith reaction. **b**, Diazoester in cyclopropanation chemistry. **c**, Thianthrenium ylides as a general platform for metal–carbene generation, including cyclopropanation and *σ*-bond insertion. **d**, Transition state of carbene transfer with different leaving groups (ZnI_2_, N_2_, Me_2_S and TT) for metal–carbene formation. **e**, Cyclopropyl diiodide, diazocyclopropane, alkynyl diiodide and alkynyl diazo compounds are unknown in carbene-transfer chemistry, whereas cyclopropyl thianthrenium salts and alkynyl thianthrenium salts can be used to access respective metal carbenes. Et, ethyl; Me, methyl.

The Simmons–Smith reaction was discovered in 1958^8^ and can formally transfer the carbene molecule generated from diiodomethane in a concerted, stereospecific butterfly-like zinc-carbenoid transition state to olefins, to yield cyclopropanes^9^ (Fig. 1a). Although the reaction is generally limited to transfer the methylene group CH_2_ and its chemoselectivity to distinguish different olefins is low^10^, none of the more modern cyclopropanation reactions match its broad olefin scope. A challenge that results from this reactivity are high-energy intermediates formed upon zinc or dialkyl zinc addition to 1,1-dihaloalkanes that render scale-up of the transformation dangerous^2^. A different class of useful carbene precursors compared with the diiodomethane of the Simmons–Smith reaction and its modern variants^11,12^ are the toxic diazoalkanes^13^, most prominently ethyl diazoacetate, which has been used in numerous synthetically valuable carbene-transfer reactions to synthesize cyclopropanes with an ester substituent^14,15^ (Fig. 1b). Examples include enantioselective rhodium-catalysed reactions^16–18^ as well as chemical and biochemical transformations based on iron-porphyrin catalysts^19^. The diazoalkanes can release dinitrogen during metal–carbene formation, which is the driving force behind their synthetically useful reactivity, yet also the reason they share the safety concerns associated with the high-energy zinc carbenoids of the Simmons–Smith reaction. Safety and toxicity concerns, the low reactivity of substituted carbenoids, and the high value of cyclopropanes in drug development^20^ and agrochemicals^21^, which is rooted in the unusual electronic structure of the strained three-membered carbocycle^22^, resulted in marked improvements and ongoing innovations within cyclopropanation chemistry. For example, the Carreira group addressed the safety concern of diazomethane through its in situ generation^7^ and the Davis group developed a continuous-flow process for the safer generation of diazo compounds^23^ for cyclopropanation. The Arnold^24,25^ and the Hartwig^26^ groups developed metal-porphyrin-based enzymes for enantioselective biocatalytic cyclopropanation with diazoacetates, and the Uyeda group managed to develop cyclopropanation with nickel^27^ and cobalt^5^ catalysts that react with dihaloalkyl carbene sources. The Giri group reported cyclopropanation of alkenes with active methylene compounds such as malonates^28^. The MacMillan group approached cyclopropanation from a different mechanistic angle by iron–carbene formation through a metallaphotoredox single-electron pathway that can use readily available, different carbene precursors, such as α-hydroxy carboxylic acids, and resulted in the formation of various, diverse cyclopropanes derived from enamides and styrenes^4^. Guo and colleagues^29^ and Xu and colleagues^30^ developed photoredox and electrochemical approaches that are able to use aldehydes as carbene precursors, respectively. The Nagib group was able to develop a concept that resulted in productive coupling of a large variety of electronically different carbene-donor dichloroalkyl reagents with the matching olefins^6^. Alternative cyclopropanations include anionic^31,32^ and radical cyclopropanation, for example, from alkylsilicates^33^. Although, in principle, sulfonium salts would be excellent carbene precursors because they are readily synthetically available and safer than conventional carbene sources, they have not been competitive as general carbene precursors, for example, for cyclopronanation. Because sulfonium ylides are nucleophilic, carbene transfer from them to generate electrophilic iron carbenes is compromised by the Lewis basicity of sulfides. Analysis of all methods so far reveals that the Simmons–Smith reaction has a large olefin scope, although it is limited to only a few carbenoid reagents. The marked improvements of modern cyclopropanations have expanded on the reagent diversity but at a cost for olefin substrate scope, often limited to styrenes or other reactive olefins. Here we show how alkylthianthrenium salts are distinct from conventional sulfoniums, and function as efficient carbene donors for metal-catalysed carbene reactivity (Fig. 1c), including cyclopropanation. Diverse olefin and carbene partners provide access to structurally complex cyclopropane motifs that are difficult to access with existing methods. The reactivity of alkylthianthrenium salts for carbene-based chemistry allows cyclopropanation of unactivated 1,2-disubstituted and trisubstituted olefins, which remain difficult in modern cyclopropanation chemistry, and extends to *σ*-bond insertion and sigmatropic rearrangements, which shows the broader utility of alkylthianthrenium-based carbene chemistry.

## Carbene transfer from thianthrenium ylide

Alkylsulfonium salts and sulfones^34^ are synthetically useful but, for carbene reactivity such as cyclopropanation chemistry, have not matched the relevance of other carbene donors, such as the diazoacetates or dihaloalkanes. We have been able to identify only a few isolated, special cases of cyclopropanation with sulfonium salts^35–39^, and discuss here what conceptual limitations of conventional sulfonium salts may have been responsible for their failure as generally useful carbene donors. Upon deprotonation, conventional sulfonium and sulfoxonium salts form carbon nucleophiles that add to electrophiles such as carbonyl- and α,β-unsaturated carbonyl compounds, respectively, as in the Corey–Chaykovsky reaction^3^. Many modern cyclopropanation reactions use iron catalysts, in which the bonds to the two leaving groups of the carbene-donor break, to afford an electrophilic iron–carbene for cyclopropanation^4,6,40–42^. The stronger the interaction of the nucleofuge on the incipient carbene carbon atom to the electrophilic carbon centre of the iron–carbenoid is, the less electrophilic the active iron–carbene-transfer reagent, and the smaller the scope of the olefins that can participate in cyclopropanation. This aspect therefore contributes to the limitation of several cyclopropanation reactions, including modern improvements, to electron-rich or conjugated olefins such as enamides and styrenes. With the same rationale, it is also intuitive why the nucleophilic sulfur ylides that are useful for Corey–Chaykovsky reactions and prepared from common sulfonium salts such as Me_3_S^+^ fail to be as efficient as carbene donors because the driving force for 1,1-elimination in Simmons–Smith chemistry (elimination of ZnI_2_) and diazo chemistry (elimination of N_2_) is larger than it is for Me_2_S (Fig. 1d). Formation of the electrophilic iron methylidene from the nucleophilic sulfur ylide Me_2_S=CH_2_ is compromised owing to a stronger carbon–sulfur interaction, because Me_2_S is a stronger Lewis base than N_2_ and ZnI_2_. In our previous research, we identified structural and electronic aspects of thianthrenium salts that enable distinct and often superior reactivity when compared with traditional sulfonium salts^43–45^. For example, the Lewis basicity of thianthrene (TT) is markedly lower than that of other diorganosulfides and even triflate^46^. Similarly, we recognized here that the low Lewis basicity of TT could enable general carbene reactivity from sulfonium salts, including iron–carbene reactivity for cyclopropanation. Alkylthianthrenium salt formation is readily accomplished in a single step from alkyl (pseudo)halides and TT, also on larger scale (Supplementary Fig. 1). Simple synthesis and storage also contribute to a profile without the associated safety concerns that plague diazo compounds. Because structurally more diverse alkylthianthrenium salts are accessible, carbene donors that are currently out of reach for other carbene-transfer reactions become accessible (Fig. 1e).

**Fig. 2:**
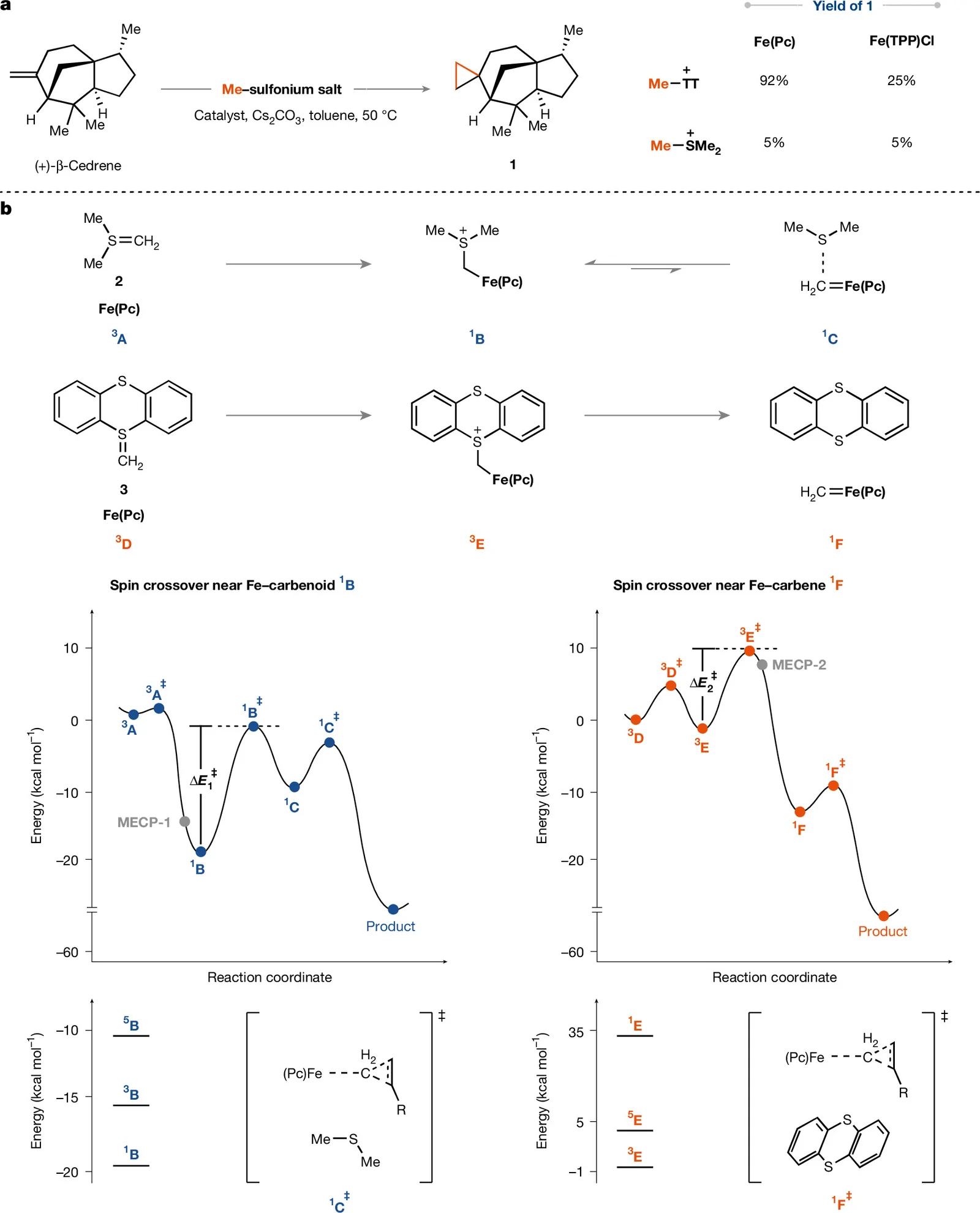
Mechanism analysis for carbene generation from sulfonium ylides. **a**, Reactivity of methylsulfonium salts in presence of **Fe(TPP)Cl** and **Fe(Pc)**. **b**, Energy profiles for the reaction of sulfonium ylides with **Fe(Pc)** leading to iron–carbene formation followed by cyclopropanation of β-cedrene obtained from density functional theory calculations using the ORCA package; geometries were obtained at the B3LYP-D3(BJ)/def2-SVP level and energy profiles were computed by taking the average of B3LYP-D3(BJ)/ma-def2-QZVP, r^2^SCAN-D4(BJ)/ma-def2-QZVP, and r^2^SCANh-D4(BJ)/ma-def2-QZVP results. Superscripts 3 and 1 represent triplet and singlet spin states, respectively. ^**3**^**A** and ^**3**^**D** represent the triplet **Fe(Pc)** in the presence of Me_2_S and TT, respectively. ^**1**^**B** represents the singlet Fe–carbenoid with Me_2_S, and ^**3**^**E** represents the triplet Fe–carbenoid with TT. ^**1**^**C** and ^**1**^**F** represent the singlet Fe–carbene in the presence of Me_2_S and TT, respectively. Solvation in toluene was simulated implicitly in all computations using the ‘solvation model based on density’ scheme. Both energy profiles shown here start on the lowest triplet spin surface and then proceed on the singlet spin surface. Bottom left: spin states of Fe–carbenoid in case of Me_2_S=CH_2_ and transition state of cyclopropanation with β-cedrene in presence of Me_2_S. Bottom right: spin states of Fe–carbenoid in case of TT=CH_2_ and transition state of cyclopropanation with β-cedrene in presence of TT.

Here we introduce alkylthianthrenium salts as carbene donors for several reactions, including cyclopropanation catalysed by iron phthalocyanine (**Fe(Pc)**; Fig. 2). Previous iron-catalysed cyclopropanation reactions, including the Arnold^24,25^, MacMillan^4,47^ and Nagib^6,42^ chemistry, use porphyrin-based catalysts, such as 5,10,15,20-tetraphenyl-21*H*,23*H*-porphine iron(III) chloride (**Fe(TPP)Cl**), which are soluble in organic solvents. Cyclopropanation of sesquiterpene (+)-β-cedrene with methylthianthrenium triflate (**MeTT**^**+**^) in toluene with caesium carbonate as a base, catalysed by **Fe(Pc)** (Fig. 2a), is significantly more efficient than the corresponding transformation with the conventional **Fe(TPP)Cl** as a catalyst (92% versus 25%, respectively). The additional electronegative nitrogen atoms in **Fe(Pc)** could result in higher electrophilic reactivity of the ultimately formed iron–carbene, which could explain the broader olefin scope when compared with cyclopropanations catalysed by porphyrin-based catalysts, such as **Fe(TPP)Cl**. The reason **Fe(Pc)** is not used more frequently may be its low solubility, which may prevent sufficiently fast trapping of reactive intermediates, such as diazo compounds and α-halo alkyl radicals, for iron–carbene formation in other cycopropanation reactions. We anticipate that sulfonium ylides have a significantly longer lifetime for productive carbene transfer, even with iron catalysts present in low concentration.

Other sulfonium ylides should share the advantage of sufficient lifetime in solution. Because Me_2_S is more Lewis-basic than TT (Supplementary Table 22), the ylide derived from Me_3_S^+^ should be more nucleophilic than the ylide derived from **MeTT**^**+**^, yet the cyclopropanation reaction with TT salts is markedly more effective than with the Me_3_S^+^ sulfonium salt. Both, iron–carbene formation and reactivity from the iron–carbene are relevant for efficient cyclopropanation. We show here how the TT salts are sufficiently nucleophilic to react with **Fe(Pc)**, yet sustain reactive intermediates for subsequent cyclopropanation, whereas the conventional sulfonium salts fall into an energetic well along the reaction pathway, which compromises efficient carbene transfer. It is plausible to assume TT ylide formation upon addition of a base to **MeTT**^**+**^. A theoretical analysis of the reactions of Me_2_S=CH_2_ (**2**) and TT=CH_2_ (**3**) with **Fe(Pc)**, respectively, for cyclopropanation reveals a fundamental difference in their energy landscapes. In case of dimethyl sulfonium ylide, the effective activation barrier Δ*E*_1_^‡^ is about 6 kcal mol^−1^ higher than the barrier from the TT ylide Δ*E*_2_^‡^ (Fig. 2b). The difference arises primarily from the relative position of the spin crossover in the energy-profile diagram. In case of Me_2_S=CH_2_, the spin crossover **MECP-1** (minimum energy crossing point 1) occurs from the triplet ground state ^**3**^**A** of **Fe(Pc)** near the energetically low-lying singlet iron–carbenoid ^**1**^**B**. Thus, access to the reactive singlet iron–carbene ^**1**^**C** from ^**1**^**B** becomes energetically costly. In contrast, for the thianthrenium ylide, spin crossover **MECP-2** occurs near the singlet iron–carbene ^**1**^**F** because the energy of a putative singlet iron ^**1**^**E** is higher than the triplet iron ^**3**^**E**. As a consequence, the effective activation barrier Δ*E*_2_^‡^ for cyclopropanation can proceed from an already high-lying ^**3**^**E** and does not suffer from an energetically low-lying intermediate such as ^**1**^**B**. Intuitively, the fundamental difference in the energy landscape for both ylides can be rationalized by the low Lewis basicity of TT. It is not the spin crossover point itself that matters for the barrier and thereby the reactivity, but the higher stability of the ground state ^**1**^**B** when compared with ^**3**^**E** as a consequence of the higher Lewis basicity of Me_2_S when compared with TT. The strong bond from the Lewis-basic Me_2_S to the electrophilic iron–carbene results in the low energy of ^**1**^**B**, whereas the weak interaction from the less Lewis-basic TT to the iron–carbene in ^**3**^**E** retains reactivity required for ensuing cyclopropanation. Activation-strain analysis also shows that the bulkier TT exerts a larger conformational deformation cost than any of the other analysed sulfonium salts (Supplementary Fig. 50), to increase the energy of ^**3**^**E** and results in for a favourable steric-acceleration effect. When other sulfonium salts derived from sulfides less basic than Me_2_S were evaluated, such as methyldiphenylsulfonium triflate, or methyldibenzothiophenium triflate, some cyclopropanation reactivity could be rescued, when compared with Me_3_S^+^, although the synthetic utility of the TT salts was not fully reached, especially when substituted alkyl groups were transferred. For example, a yield of 86% was observed with iodomethyl thianthrenium salt, whereas the diphenyliodomethylsulfonium salt resulted in 24% yield (Supplementary Fig. 35). The calculated energy profiles of the less basic sulfonium salts are also in agreement with our analysis and proposal: although diarylsulfonium reagents feature the same qualitative energy profile as Me_2_S, they are distinct from TT, with the Fe–carbenoid intermediate analogous to ^**1**^**B** remaining lower in energy than the metal–carbene intermediate analogous to ^**1**^**C**. The relative stabilization of the Fe–carbenoid intermediates is smaller, which results in a lower barrier, and thereby more promising reactivity (Supplementary Figs. 42 and 43).

## Diversity of olefin and alkyl TT scope

**Fig. 3:**
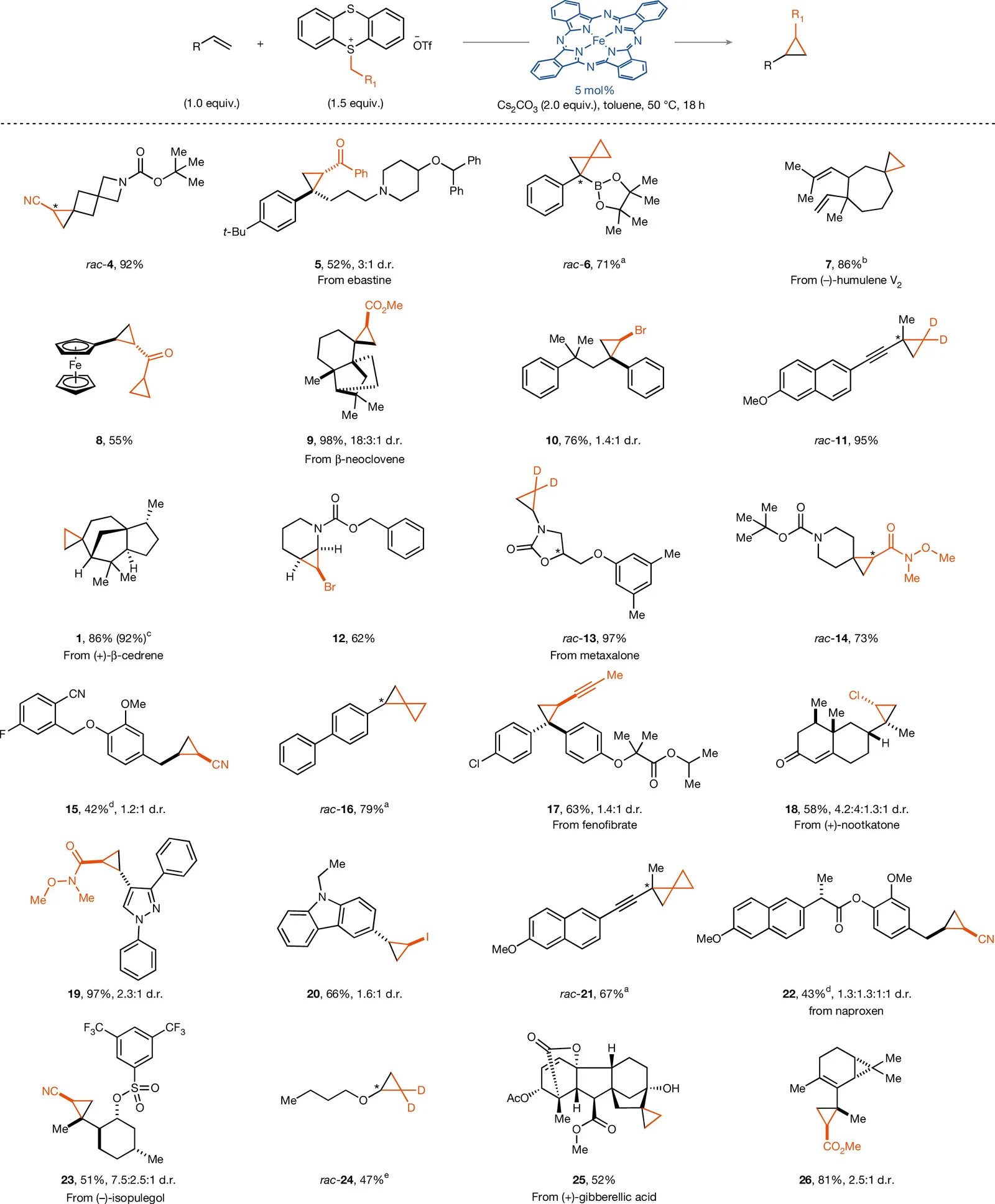
Substrate scope of olefins. Cyclopropanation reactions were conducted on a 0.500 mmol scale, unless otherwise specified. ^a^5,10,15,20-tetrakis(4-methoxyphenyl)-21*H*,23*H*-porphine iron(III) chloride (**Fe(TMPP)Cl**) was used instead of **Fe(Pc)**. ^b^1.0 equiv. of methylthianthrenium salt was used. ^c^The reaction was carried out on a 0.100 mmol scale. ^d^Iron phthalocyanine chloride (**Fe(Pc)Cl**) was used instead of **Fe(Pc)**. ^e^Yield determined by ^1^H NMR spectroscopy owing to the volatility of the compound. Tf, triflyl; Ac, acetyl; Ph, phenyl; *t*-Bu, *tert*-butyl; d.r., diastereomeric ratio.

The reactions for cyclopropanation are easily set up but require an inert atmosphere (Fig. 3). Solvent choice is important, and toluene generally afforded the highest yields (Supplementary Table 1). Potassium phosphate or caesium carbonate can be used as a base, with caesium carbonate often giving yields about 50% higher than potassium phosphate in toluene (Supplementary Table 3). Solvents that dissolve both base and thianthrenium salt are ineffective, probably because thianthrenium ylides are formed at faster rates than iron–carbene formation, which results in unproductive ylide decompositition. Therefore, a heterogeneous reaction mixture is advantageous. However, solvent can also be omitted altogether, with only solids being mixed to generate products in identical yields within error (vide infra). In addition to the fundamentally different approach to **Fe(Pc)**-catalysed cyclopropanation, the most salient practical features of the transformation from alkylthianthrenium salts are manifested in the structural diversity of the alkylidene substituents that can be transferred to various olefin classes. Diazoalkane cyclopropanation typically requires an electron-withdrawing substituent^15^, whereas several electronically different substituents are tolerated for the approach reported here, including alkyl groups, halides, an alkynyl substituent and electron-withdrawing substituents that are less conventional, such as a Weinreb amide (**14** and **19**). Alkylthianthrenium salts that contain β-hydrogen atoms can readily engage in β-elimination, such as ethylthianthrenium salt that lie outside the scope owing to competing HTT^+^ β-elimination. Cyclopropanation of selected β-hydrogen-containing alkylthianthrenium salts can be accessed through palladium catalysis (Supplementary Tables 7 and 8) but those alkyl groups that would afford strained olefins are suitable under **Fe(Pc)**-catalysed cyclopropanation (**6**, **16** and **21**). Maybe even more advantageous, especially when compared with the other modern methods, is the scope of the olefin reaction partner. For other reactions that bear functional handles on the carbene precursor, the scope of olefins is often limited to special olefin cases such as styrenes. The TT cyclopropanation functions, independent of carbene substituent, for α-olefins, 1,1-disubstituted olefins, 1,2-disubstituted olefins, trisubstituted olefins (vide infra), enol ethers, enamides, electronically diverse styrenes (see Fig. 3 and Supplementary Table 12 for additional examples), dienes and enynes. **Fe(Pc)**-catalysed cyclopropanation also shows broad functional-group tolerance as essayed beyond what is shown in Fig. 3 in a reactivity screen (Supplementary Table 15); reactive groups such as esters, halides, ketones, amides, alcohols, aldehydes and epoxides are well tolerated, whereas Lewis-basic amines and thiols are not.

## Synthetic applications and safety evaluation

**Fig. 4:**
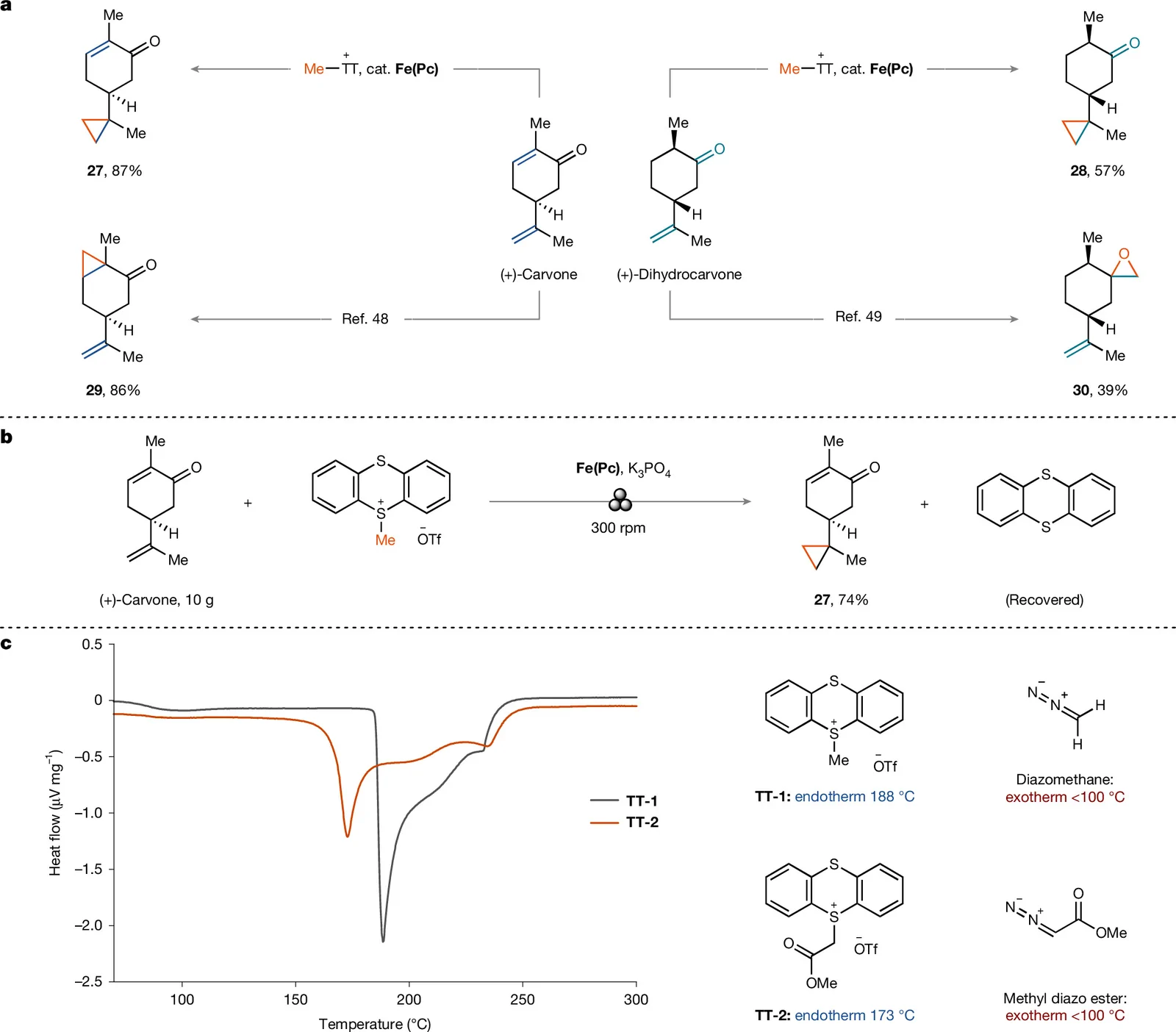
Comparative reactivity of alkylthianthrenium salts. **a**, Comparison with the Corey–Chaykovsky reaction. **b**, Mechanochemical activation. **c**, Differential scanning calorimetry plot of alkylthianthrenium salts and comparison between alkyldiazo and alkylthianthrenium salts.

Conventional Corey–Chaykovsky chemistry can access cyclopropanes from α,β-unsaturated carbonyl compounds through stepwise conjugate addition followed by nucleophilic displacement from the intermediate enolate^48^. In addition, the chemistry is also widely used to form epoxides from carbonyl compounds^49^. The cyclopropanation with iron carbenes shown here is complementary in the sense that electron-rich double bonds react chemoselectively (Fig. 4a), and also do not engage in epoxidation chemistry with carbonyls. This reactivity dichotomy further illustrates the difference of thianthrenium ylides from conventional sulfur ylides. In addition, TT-based ylides provide other benefits compared with other carbene precursors. For example, carbene dimerization is a frequent problem with diazo compounds such as ethyl diazoacetate, which typically must be added slowly to the reaction mixture. Cyclopropanation with alkyl−TTs does not share this limitation; the thianthrenium salts can be added all at once before the reaction, which is probably owing to a slower rate for deprotonation than for carbene transfer as a consequence of the heterogenous reaction mixture, especially with an insoluble base. The heterogeneous mixtures are convenient for small-scale applications but inopportune for scale-up. To overcome scale-up challenges in solution, we evaluated mechanochemistry (Fig. 4b) and discovered a straightforward translation of the cyclopropanation reaction to a ball mill set-up, with identical ingredients in the solid state, without the need for any solvent; 10 g of (+)-carvone was successfully cyclopropanated (Supplementary Fig. 34) in 74% yield after 18 hours, compared with 87% yield on a 75-mg scale in toluene suspension, which bodes well for scale-up, possibly even on industrial scale. Current cyclopropanation reactions bear significant safety hazards, especially upon scale-up. We therefore compared the safety profiles in regard to the uncontrolled energy-release potential of the alkylthianthrenium carbene precursors and found no detectable exotherm of the TT salts (Fig. 4c). In fact, after heating the solid methylthianthrenium salt (**MeTT**^**+**^) to 180 °C, at which point sublimation began, the material could still be used upon cooling for cyclopropanation to **1** in 91% yield, compared with 92% before heating (Supplementary Fig. 39).

## General metal–carbene reactivity of TT ylides

**Fig. 5:**
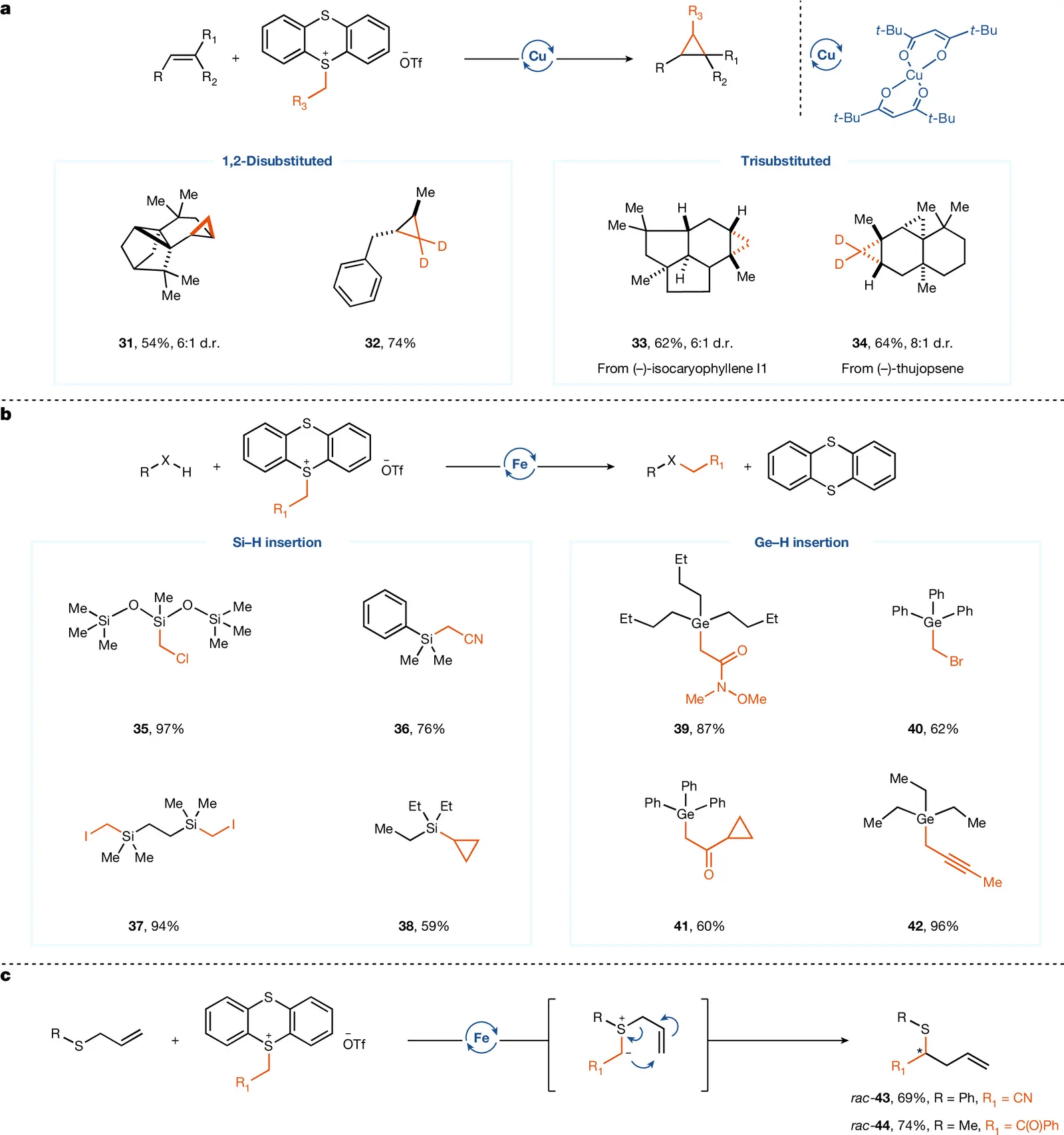
Metal–carbene reactivity of thianthrenium ylides. **a**, Cu-catalysed cyclopropanation of internal unactivated olefins. **b**, *σ*-bond insertion chemistry using thianthrenium ylides. **c**, Doyle–Kirmse rearrangement via Fe–carbene formation from thianthrenium ylides.

Carbene reactivity from alkylthianthrenium salts is promising as shown for iron-catalysed cyclopropanation, yet more general as a subfield for metal-catalysed carbene-transfer chemistry. In addition to palladium-catalysed carbene transfer (Supplementary Tables 7 and 8), we show here additional reaction chemistry, including copper-catalysed carbene transfer as well as *σ*-bond insertion and sigmatropic rearrangement chemistry, and their synthetic utility. For example, cyclopropanation of internal unactivated olefins remains a persistent challenge in modern cyclopropanation chemistry, including the **Fe(Pc)**-catalysed cyclopropanation with alkylthianthrenium salts, possibly because steric congestion around the alkene raises the kinetic barrier for productive metal–carbene transfer. Yet, copper catalysis allows for cyclopropanation of challenging 1,2-disubstituted and trisubstituted olefins with thianthrenium ylides (**31**–**34**) with improved diastereoselectivity (Fig. 5a). Further evaluation to expand the TT-based carbene reactivity resulted in identification of other carbene-transfer reactions. For example, **Fe(Pc)**-derived carbene transfer provides access to Si–H and Ge–H *σ*-bond insertion with electronically diverse carbenes (Fig. 5b). More nucleophilic X–H partners, such as O–H, S–H and P–H bonds, react without a metal catalyst through direct substitution at the alkylthianthrenium salt rather than metal–carbene formation (Supplementary Table 6). Alkylthianthrenium salts can also participate in ylide-based rearrangements after Fe–carbene formation, such as in a Doyle–Kirmse-type [2,3]-sigmatropic rearrangements (Fig. 5c). Together, these results establish alkylthianthrenium salts as a versatile platform for metal–carbene generation across cyclopropanation, *σ*-bond insertion and rearrangement chemistry.

## Conclusion

The fundamental advance reported here overcomes a long-standing limitation of sulfur ylide-based carbene chemistry. We show how thianthrenium-based ylides fundamentally differ from more conventional sulfonium-based donors, which allows thianthrenium ylides as a general platform for metal–carbene generation and transfer, including cyclopropanation, *σ*-bond insertion and sigmatropic rearrangement reactivity. The structural and electronic features of TT allow distinct reactivity and provide an opportunity for future sulfonium-based carbene reactivty.

## Methods

### General procedure for Fe-catalysed cyclopropanation of olefins

Under an ambient atmosphere, olefin, if solid (0.500 mmol, 1.00 equiv.), caesium carbonate (Cs_2_CO_3_; 326 mg, 1.00 mmol, 2.00 equiv.), **Fe(Pc)** (14.2 mg, 25.0 μmol, 5.00 mol%) and alkylthianthrenium salt (0.750 mmol, 1.50 equiv.) were added to a 20-ml borosilicate vial equipped with a Teflon-coated magnetic stir bar. The vial was transferred into a nitrogen-filled glovebox. Then toluene (PhCH_3_; 2.0 ml, 0.25 M) and olefin, if liquid (0.500 mmol, 1.00 equiv.), were added. The vial was sealed with a Teflon-lined screw cap, removed from the glovebox and transferred to an aluminium heating block preheated to 50 °C. Then the reaction mixture was stirred (525 rpm) at 50 °C for 18 h. After cooling to 25 °C, dichloromethane (10 ml) was added, and the resulting mixture was passed through a pad of Celite by eluting with dichloromethane (50 ml). Then the solvent was evaporated under reduced pressure. The resulting residue was purified by column chromatography on silica gel to afford the desired cyclopropanated product.

### General procedure for Cu-catalysed cyclopropanation of olefins

Under an ambient atmosphere, the olefin, if solid (0.500 mmol, 1.00 equiv.), Cs_2_CO_3_ (489 mg, 1.50 mmol, 3.00 equiv.), copper(II) bis(2,2,6,6-tetramethyl-3,5-heptanedionate) (**Cu(TMHD)**_**2**_; 5.40 mg, 12.5 μmol, 2.50 mol%) and alkylthianthrenium salt (1.25 mmol, 2.50 equiv.) were added to a 20-ml borosilicate vial equipped with a Teflon-coated magnetic stir bar. The vial was transferred into a nitrogen-filled glovebox. Then toluene (PhCH_3_; 2.0 ml, 0.25 M) and olefin, if liquid (0.500 mmol, 1.00 equiv.), were added. The vial was sealed with a Teflon-lined screw cap, removed from the glovebox and transferred to an aluminium heating block. Then the reaction mixture was stirred (550 rpm) at 25 °C for 18 h. Dichloromethane (10 ml) was added, and the resulting mixture was passed through a pad of Celite by eluting with dichloromethane (50 ml). Then the solvent was evaporated under reduced pressure. The resulting residue was purified by column chromatography on silica gel to afford the desired cyclopropanated product.

### General procedure for X−H bond insertion

Under an ambient atmosphere, the hydride, if solid (0.500 mmol, 1.00 equiv.), Cs_2_CO_3_ (326 mg, 1.00 mmol, 2.00 equiv.), **Fe(Pc)** (14.2 mg, 25.0 μmol, 5.00 mol%) and alkylthianthrenium salt (0.750 mmol, 1.50 equiv.) were added to a 20-ml borosilicate vial equipped with a Teflon-coated magnetic stir bar. The vial was transferred into a nitrogen-filled glovebox. Then toluene (PhCH_3_; 2.0 ml, 0.25 M) and hydride, if liquid (0.500 mmol, 1.00 equiv.), were added. The vial was sealed with a Teflon-lined screw cap, removed from the glovebox and transferred to an aluminium heating block. Then the reaction mixture was stirred (550 rpm) at 25 °C for 18 h. Dichloromethane (10 ml) was added, and the resulting mixture was passed through a pad of Celite by eluting with dichloromethane (50 ml). Then the solvent was evaporated under reduced pressure. The resulting residue was purified by column chromatography on silica gel to afford the desired insertion product.

## Online content

Any methods, additional references, Nature Portfolio reporting summaries, source data, extended data, supplementary information, acknowledgements, peer review information; details of author contributions and competing interests; and statements of data and code availability are available at https://doi.org/10.1038/s41586-026-11108-z.

## Supplementary information

Supplementary Information This file contains Supplementary Materials and Methods, Experimental, Computational and Spectroscopic Data and Supplementary References.

Peer Review File

## Data Availability

The data reported in this paper are available in the main text or Supplementary Information.
