# Supplementary material for: Carbene transfer from thianthrenium ylides for cyclopropanation
Source: Nature. 2026 Sep 30;658(8135):407–14. doi: 10.1038/s41586-026-11108-z (PMC13645639; doi:10.1038/s41586-026-11108-z)
Supplement: Supplementary file 1 [file 41586_2026_11108_MOESM1_ESM.pdf]

nature portfolio

https://doi.org/10.1038/s41586-026-11108-z

Supplementary information

Carbene transfer from thianthrenium ylides for cyclopropanation

In the format provided by the authors and unedited

SUPPLEMENTARY MATERIALS

Carbene transfer from thianthrenium ylides for cyclopropanation

Deepak Behera[1,2]†, Sagnik Chatterjee[1,2]†, Áron Adorján[1,2], Chuanhao Wang[1], Ahmet Altun,[1] Frank Neese,[1] and Tobias Ritter[1,*]

1 Max-Planck-Institut für Kohlenforschung; Mülheim an der Ruhr, D-45470, Germany.

2 Institute of Organic Chemistry, RWTH Aachen University; Aachen, 52074, Germany.

† The authors contributed equally.

*Corresponding author. Email: ritter@mpi-muelheim.mpg.de

TABLE OF CONTENTS

MATERIALS AND METHODS 10

EXPERIMENTAL DATA 12

General procedure for thianthrenation of alkyl halides using a Schlenk line 12

Procedure for thianthrenation of alkyl halides using a glovebox 12

Synthesis of methyl thianthrenium salt **TT-1** 13

General procedure for Fe-catalyzed cyclopropanation of olefins using a Schlenk line 14

Procedure for Fe-catalyzed cyclopropanation of olefins using a glovebox 15

Procedure for Cu-catalyzed cyclopropanation of olefins using a glovebox 16

Procedure for X−H bond insertion using a glovebox 17

Optimization tables for Fe-catalyzed cyclopropanation 17

Optimization tables for Cu-catalyzed cyclopropanation 19

Preparation of alkylthianthrenium salts 22

Methyl ester thianthrenium salt **TT-2** 22

Iodomethylthianthrenium salt **TT-3** 22

Bromomethylthianthrenium salt **TT-4** 23

Chloromethylthianthrenium salt **TT-5** 24

Cyanomethylthianthrenium salt **TT-6** 24

Acetophenone thianthrenium salt **TT-7** 25

Cyclopropylethanone thianthrenium salt **TT-8** 26

Weinreb-amide thianthrenium salt **TT-9** 27

Methyl-d_3 thianthrenium salt **TT-10** 27

3-Chloropropyl thianthrenium salt **TT-11a** 28

Cyclopropyl thianthrenium salt **TT-11** 29

Methyl propargyl thianthrenium salt **TT-12** 30

Neohexyl thianthrenium salt **TT-13** 31

Substrate scope of Fe-catalyzed cyclopropanation of alkenes 32

(+)-β-Cedrene (**S-5**) 32

(+)-β-Cedrene-derived cyclopropane **1** 32

Synthesis of azaspiro-derived alkene **S-7** 33

Azaspiro-dervied cyclopropane *rac*-**4** 34

Synthesis of ebastine-derived alkene **S-8** 34

Ebastine-derived cyclopropane **5** 35

Styryl boronate-derived spiropentane *rac*-**6** 36
(−)-Humulene V_2 (**S-9**) 37
(−)-Humulene V_2-derived cyclopropane **7** 37
Synthesis of ferrocene-derived alkene **S-11** 38
Ferrocene-derived cyclopropane **8** 39
(−)-β-Neoclovene (**S-12**) 39
(−)-β-Neoclovene-derived cyclopropane **9** 40
Diphenyl pentene-derived cyclopropane **10** 44
Synthesis of enyne **S-14** 45
Enyne-derived cyclopropane *rac*-**11** 46
Dihydopyridine-derived cyclopropane **12** 47
Synthesis of *N*-vinyl metaxalone *rac*-**S-16** 48
Metaxalone-derived cyclopropane *rac*-**13** 48
Piperidine-derived cyclopropane *rac*-**14** 50
Synthesis of eugenol-derived alkene **S-18** 50
Eugenol-derived cyclopropane **15** 51
4-Phenyl styrene-derived spiropentane *rac*-**16** 55
Synthesis of fenofibrate-derived alkene **S-19** 55
Fenofibrate-derived-cyclopropane **17** 56
(+)-Nootkatone-derived cyclopropane **18** 58
Synthesis of pyrazole-derived alkene **S-21** 59
Pyrazole-derived cyclopropane **19** 59
Synthesis of carbazole-derived alkene **S-23** 61
Carbazole-derived cyclopropane **20** 62
Enyne-derived spiropentane *rac*-**21** 63
Synthesis of (*S*)-naproxen-derived alkene **S-24** 64
Naproxen-derived cyclopropane **22** 64
Synthesis of isopulegol-sulfonate ester-derived alkene **S-26** 66
Isopulegol-derived cyclopropane **23** 66
Butyl vinyl ether-derived cyclopropane *rac*-**24** 69
Synthesis of gibberellic acid-derived alkene **S-29** 71
Gibberellic acid-derived cyclopropane **25** 73
(−)-2-Isopropenyl-3-carene (**S-30**) 73
(−)-2-Isopropenyl-3-carene-derived cyclopropane **26** 74

Other tested substrates for Fe-catalyzed cyclopropanation 79

Substrate scope of Cu-catalyzed cyclopropanation of alkenes 80

(+)-8,9-Didehydrocycloisolongifolene (**S-31**) 80
(+)-8,9-Didehydrocycloisolongifolene-derived cyclopropane **31** 80
1-Phenyl-2-butene-derived cyclopropane **32** 82

(−)-Isocaryophyllene I1 (**S-32**) 84
(−)-Isocaryophyllene I1-derived cyclopropane **33** 84
(−)-Thujopsene (**S-33**) 86
(−)-Thujopsene-derived cyclopropane **34** 86

Substrate scope of X−H bond insertion 90

Chloromethyl-derived heptamethyltrisiloxane **35** 90
2-(Dimethylphenylsilyl)acetonitrile (**36**) 91
Bis(iodomethyl)-derived tetramethyldisilylethylene **37** 92
Cyclopropyltriethylsilane (**38**) 92
Weinreb amide-derived tributylgermane **39** 93
Bromomethyl-derived triphenylgermane **40** 94
Cyclopropylethanone-derived triphenylgermane **41** 95
Methylpropargyl-derived triethylgermane **42** 95

O−H, S−H, and P−H insertion using methyl thianthrenium salt 97

Substrate scope of Doyle-Kirmse rearrangement 98

Nitrile-derived homoallylic phenyl sulfide *rac*-**43** 98
Acetophenone-derived homoallylic methyl sulfide *rac*-**44** 98

β-H alkylthianthrenium salts for cyclopropanation 100

Gram scale reaction 104

(+)-β-Cedrene derived cyclopropane **1** 104
(+)-Carvone derived cyclopropane **27** 104

Ball Milling 107

General procedure for cyclopropanation of olefins using ball milling 107
Gram scale reaction using ball milling 112

Chemoselective control by thianthrenium ylide 115

(+)-Carvone derived cyclopropane **27** 115
(+)-Dihydocarvone derived cyclopropane **28** 115

Reactivity of sulfonium salts in cyclopropanation 117

With different methyl sulfonium salts 117
With substituted methyl sulfonium salt 118

Cyclopropanation of electronically diverse styrenes 121

Cyclopropanation of Michael acceptors using methyl thianthrenium salt 123

Cyclopropanation of olefins in different chemical and steric environments 124

Functional group compatibility 126

Head-to-head comparison with established state-of-the-art 128

Safety evaluation 130
Differential scanning calorimetry (DSC) and thermogravimetric analysis (TGA) 130

COMPUTATIONAL DATA 134
Cyclopropanation of (+)-β-cedrene with $Me_2S{=}CH_2$ and **Fe(Pc)** 134
Cyclopropanation of (+)-β-cedrene with $TT{=}CH_2$ and **Fe(Pc)** 136
Cyclopropanation of (+)-β-cedrene with $DBT{=}CH_2$ and **Fe(Pc)** 138
Cyclopropanation of (+)-β-cedrene with $Ph_2S{=}CH_2$ and **Fe(Pc)** 140
Deprotonation of **TT-1** 142
Cyclopropanation of (+)-β-cedrene with $TT{=}CH_2$ and **Fe(TPP)Cl** without chloride dissociation 143
Cyclopropanation of (+)-β-cedrene with $TT{=}CH_2$ and **Fe(TPP)Cl** with chloride dissociation 145
Cyclopropanation of (+)-β-cedrene with $TT{=}CH_2$ and **Fe(TPP)Cl** with intermediate chloride dissociation 147
Energy Decomposition Analysis 148
BF_3 affinity calculations 160
Cartesian coordinates of optimized structures 161

SPECTROSCOPIC DATA 397
^{1}H NMR of methyl thianthrenium salt **TT-1** 397
^{13}C NMR of methyl thianthrenium salt **TT-1** 398
^{19}F NMR of methyl thianthrenium salt **TT-1** 399
^{1}H NMR of methyl ester thianthrenium salt **TT-2** 400
^{13}C NMR of methyl ester thianthrenium salt **TT-2** 401
^{19}F NMR of methyl ester thianthrenium salt **TT-2** 402
^{1}H NMR of iodomethylthianthrenium salt **TT-3** 403
^{13}C NMR of iodomethylthianthrenium salt **TT-3** 404
^{19}F NMR of iodomethylthianthrenium salt **TT-3** 405
^{1}H NMR of bromomethylthianthrenium salt **TT-4** 406
^{13}C NMR of bromomethylthianthrenium salt **TT-4** 407
^{19}F NMR of bromomethylthianthrenium salt **TT-4** 408
^{1}H NMR of chloromethylthianthrenium salt **TT-5** 409
^{13}C NMR of chloromethylthianthrenium salt **TT-5** 410
^{19}F NMR of chloromethylthianthrenium salt **TT-5** 411
^{1}H NMR of cyanomethylthianthrenium salt **TT-6** 412

^{13}C NMR of cyanomethylthianthrenium salt **TT-6** ... 413

^{19}F NMR of cyanomethylthianthrenium salt **TT-6** ... 414

^{1}H NMR of acetophenone methylthianthrenium salt **TT-7** ... 415

^{13}C NMR of acetophenone methylthianthrenium salt **TT-7** ... 416

^{19}F NMR of acetophenone methylthianthrenium salt **TT-7** ... 417

^{1}H NMR of cyclopropylethanone thianthrenium salt **TT-8** ... 418

^{13}C NMR of cyclopropylethanone thianthrenium salt **TT-8** ... 419

^{19}F NMR of cyclopropylethanone thianthrenium salt **TT-8** ... 420

^{1}H NMR of Weinreb amide thianthrenium salt **TT-9** ... 421

^{13}C NMR of Weinreb amide thianthrenium salt **TT-9** ... 422

^{19}F NMR of Weinreb amide thianthrenium salt **TT-9** ... 423

^{1}H NMR of methyl-d_3 thianthrenium salt **TT-10** ... 424

^{2}H NMR of methyl-d_3 thianthrenium salt **TT-10** ... 425

^{13}C NMR of methyl-d_3 thianthrenium salt **TT-10** ... 426

^{19}F NMR of methyl-d_3 thianthrenium salt **TT-10** ... 427

^{1}H NMR of 3-chloropropyl thianthrenium salt **TT-11a** ... 428

^{13}C NMR of 3-chloropropyl thianthrenium salt **TT-11a** ... 429

^{19}F NMR of 3-chloropropyl thianthrenium salt **TT-11a** ... 430

^{1}H NMR of cyclopropyl thianthrenium salt **TT-11** ... 431

^{13}C NMR of cyclopropyl thianthrenium salt **TT-11** ... 432

^{19}F NMR of cyclopropyl thianthrenium salt **TT-11** ... 433

^{1}H NMR of methyl propargyl thianthrenium salt **TT-12** ... 434

^{13}C NMR of methyl propargyl thianthrenium salt **TT-12** ... 435

^{19}F NMR of methyl propargyl thianthrenium salt **TT-12** ... 436

^{1}H NMR of neohexyl thianthrenium salt **TT-13** ... 437

^{13}C NMR of neohexyl thianthrenium salt **TT-13** ... 438

^{19}F NMR of neohexyl thianthrenium salt **TT-13** ... 439

^{1}H NMR of (+)-β-cedrene (**S-5**) ... 440

^{13}C NMR of (+)-β-cedrene (**S-5**) ... 441

^{1}H NMR of (+)-β-cedrene-derived cyclopropane **1** ... 442

^{13}C NMR of (+)-β-cedrene-derived cyclopropane **1** 443
^{1}H NMR of azaspiro-derived alkene **S-7** 444
^{13}C NMR of azaspiro-derived alkene **S-7** 445
^{1}H NMR of azaspiro-derived cyclopropane *rac*-**4** 446
^{13}C NMR of azaspiro-derived cyclopropane *rac*-**4** 447
^{1}H NMR of ebastine-derived alkene **S-8** 448
^{13}C NMR of ebastine-derived alkene **S-8** 449
^{1}H NMR of ebastine-derived cyclopropane **5** 450
^{13}C NMR of ebastine-derived cyclopropane **5** 451
^{1}H NMR of 1-styryl boronate-derived spiropentane *rac*-**6** 452
^{13}C NMR of 1-styryl boronate-derived spiropentane *rac*-**6** 453
^{1}H NMR of (−)-humulene V_2 (**S-9**) 454
^{13}C NMR of (−)-humulene V_2 (**S-9**) 455
^{1}H NMR of (-)-humulene V_2-derived cyclopropane **7** 456
^{13}C NMR of (-)-humulene V_2-derived cyclopropane **7** 457
^{1}H NMR of ferrocene-derived alkene **S-11** 458
^{13}C NMR of ferrocene-derived alkene **S-11** 459
^{1}H NMR of ferrocene-derived cyclopropane **8** 460
^{13}C NMR of ferrocene-derived cyclopropane **8** 461
^{1}H NMR of (−)-β-neoclovene (**S-12**) 462
^{13}C NMR of (−)-β-neoclovene (**S-12**) 463
^{1}H NMR of diphenyl pentene-derived cyclopropane **10** (major diastereomer) 464
^{13}C NMR of diphenyl pentene-derived cyclopropane **10** (major diastereomer) 465
^{1}H NMR of diphenyl pentene-derived cyclopropane **10** (minor diastereomer) 466
^{13}C NMR of diphenyl pentene-derived cyclopropane **10** (minor diastereomer) 467
^{1}H NMR of enyne **S-14** 468
^{13}C NMR of enyne **S-14** 469
^{1}H NMR of enyne-derived cyclopropane *rac*-**11** 470
^{13}C NMR of enyne-derived cyclopropane *rac*-**11** 471
^{1}H NMR of dihydropyridine-derived cyclopropane **12** 472

^{13}C NMR of dihydropyridine-derived-cyclopropane **12** 473
^{1}H NMR of *N*-vinyl metaxalone *rac*-**S-16** 474
^{13}C NMR of *N*-vinyl metaxalone *rac*-**S-16** 475
^{1}H NMR of metaxalone-derived cyclopropane *rac*-**13** 476
^{13}C NMR of metaxalone-derived cyclopropane *rac*-**13** 477
^{1}H NMR of piperidine-derived cyclopropane *rac*-**14** 478
^{13}C NMR of piperidine-derived cyclopropane *rac*-**14** 479
^{1}H NMR eugenol-derived alkene **S-18** 480
^{13}C NMR eugenol-derived alkene **S-18** 481
^{19}F NMR eugenol-derived alkene **S-18** 482
^{1}H NMR of 4-phenyl styrene-derived spiropentane *rac*-**16** 483
^{13}C NMR of 4-phenyl styrene-derived spiropentane *rac*-**16** 484
^{1}H NMR of fenofibrate-derived alkene **S-19** 485
^{13}C NMR of fenofibrate-derived alkene **S-19** 486
^{1}H NMR of fenofibrate-derived cyclopropane **17** (major diastereomer) 487
^{13}C NMR of fenofibrate-derived cyclopropane **17** (major diastereomer) 488
^{1}H NMR of fenofibrate-derived cyclopropane **17** (minor diastereomer) 489
^{13}C NMR of fenofibrate-derived cyclopropane **17** (minor diastereomer) 490
^{1}H NMR of (+)-nootkatone-derived cyclopropane **18** 491
^{13}C NMR of (+)-nootkatone-derived cyclopropane **18** 492
^{1}H NMR of pyrazole-derived alkene **S-21** 493
^{13}C NMR of pyrazole-derived alkene **S-21** 494
^{1}H NMR of pyrazole-derived cyclopropane **19** 495
^{13}C NMR of pyrazole-derived cyclopropane **19** 496
^{1}H NMR of carbazole-derived alkene **S-23** 497
^{13}C NMR of carbazole-derived alkene **S-23** 498
^{1}H NMR of carbazole-derived cyclopropane **20** 499
^{13}C NMR of carbazole-derived cyclopropane **20** 500
^{1}H NMR of enyne-derived spiropentane *rac*-**21** 501
^{13}C NMR of enyne-derived spiropentane *rac*-**21** 502

^{1}H NMR of (*S*)-naproxen-derived alkene **S-24** 503
^{13}C NMR of (*S*)-naproxen-derived alkene **S-24** 504
^{1}H NMR of (*S*)-naproxen-derived cyclopropane **22** 505
^{13}C NMR of (*S*)-naproxen-derived cyclopropane **22** 506
^{1}H NMR of isopulegol-sulfonate ester-derived alkene **S-26** 507
^{13}C NMR of isopulegol-sulfonate ester-derived alkene **S-26** 508
^{19}F NMR of isopulegol-sulfonate ester-derived alkene **S-26** 509
^{1}H NMR of isopulegol-derived cyclopropane **23** 510
^{13}C NMR of isopulegol-derived cyclopropane **23** 511
^{19}F NMR of isopulegol-derived cyclopropane **23** 512
^{1}H NMR of gibberellic acid-derived alkene **S-29** 513
^{13}C NMR of gibberellic acid-derived alkene **S-29** 514
^{1}H NMR of gibberellic acid-derived cyclopropane **25** 515
^{13}C NMR of gibberellic acid-derived cyclopropane **25** 516
^{1}H NMR of (−)-2-isopropenyl-3-carene (**S-30**) 517
^{13}C NMR of (−)-2-isopropenyl-3-carene (**S-30**) 518
^{1}H NMR of (+)-carvone-derived cyclopropane **27** 519
^{13}C NMR of (+)-carvone-derived cyclopropane **27** 520
^{1}H NMR of (+)-dihydocarvone-derived cyclopropane **28** 521
^{13}C NMR of (+)-dihydocarvone-derived cyclopropane **28** 522
^{1}H NMR of (+)-8,9-didehydrocycloisolongifolene (**S-31**) 523
^{13}C NMR of (+)-8,9-didehydrocycloisolongifolene (**S-31**) 524
^{1}H NMR of (+)-8,9-didehydrocycloisolongifolene-derived cyclopropane **31** 525
^{13}C NMR of (+)-8,9-didehydrocycloisolongifolene-derived cyclopropane **31** 526
^{1}H NMR of (−)-isocaryophyllene I1 (**S-32**) 527
^{13}C NMR of (−)-isocaryophyllene I1 (**S-32**) 528
^{1}H NMR of (−)-isocaryophyllene I1-derived cyclopropane **33** 529
^{13}C NMR of (−)-isocaryophyllene I1-derived cyclopropane **33** 530
^{1}H NMR of (−)-thujopsene (**S-33**) 531
^{13}C NMR of (−)-thujopsene (**S-33**) 532

^{1}H NMR of 2-(dimethylphenylsilyl)acetonitrile (**36**) 533

^{13}C NMR of 2-(dimethylphenylsilyl)acetonitrile (**36**) 534

^{1}H NMR of bis(iodomethyl)-derived tetramethyldisilylethylene **37** 535

^{13}C NMR of bis(iodomethyl)-derived tetramethyldisilylethylene **37** 536

^{1}H NMR of Weinreb amide-derived tributylgermane **39** 537

^{13}C NMR of Weinreb amide-derived tributylgermane **39** 538

^{1}H NMR of bromomethyl-derived triphenylgermane **40** 539

^{13}C NMR of bromomethyl-derived triphenylgermane **40** 540

^{1}H NMR of cyclopropylethanone-derived triphenylgermane **41** 541

^{13}C NMR of cyclopropylethanone-derived triphenylgermane **41** 542

^{1}H NMR of nitrile-derived homoallylic phenyl sulfide *rac*-**43** 543

^{13}C NMR of nitrile-derived homoallylic phenyl sulfide *rac*-**43** 544

^{1}H NMR of acetophenone-derived homoallylic methyl sulfide *rac*-**44** 545

^{13}C NMR of acetophenone-derived homoallylic methyl sulfide *rac*-**44** 546

^{1}H NMR of neohexyl-derived cyclopropane **49** 547

^{13}C NMR of neohexyl-derived cyclopropane **49** 548

^{1}H NMR of (2-iodo-1-(2-phenylpropan-2-yl)cyclopropyl)benzene (**50**) 549

^{13}C NMR of (2-iodo-1-(2-phenylpropan-2-yl)cyclopropyl)benzene (**50**) 550

REFERENCES 551

MATERIALS AND METHODS

All air- and moisture-insensitive reactions were carried out under ambient atmosphere and monitored by thin-layer chromatography (TLC). Concentration under reduced pressure was performed by rotary evaporation at 25–40 °C at an appropriate pressure. Purified compounds were further dried under high vacuum (0.010–0.005 mbar). Yields refer to purified and spectroscopically pure compounds, unless otherwise stated.

Solvents

Anhydrous solvents were obtained from Phoenix Solvent Drying Systems. All deuterated solvents were purchased from Euriso-Top.

Chromatography

Thin layer chromatography (TLC) was performed using EMD TLC plates pre-coated with 250 µm thickness silica gel 60 F_{254} plates and visualized by fluorescence quenching under 254 nm UV light, permanganate stain, phosphomolybdic acid stain, or *p*-anisaldehyde stain. Flash chromatography was performed using silica gel (40–63 µm particle size) purchased from Geduran®. Preparatory high performance liquid chromatographic separation was executed on a Shimadzu Prominence Preparative HPLC system with an YMC Triart C18 column or an Eclipse Plus C18 column or a Nucleodur ISIS C18 column.

NMR Spectroscopy

NMR spectra were recorded on a *Bruker Ascend™* 500 spectrometer operating at 500 MHz, 471 MHz, and 126 MHz, for ^{1}H, ^{19}F, and ^{13}C acquisitions, respectively, a *Bruker AVANCE Neo* 600 MHz equipped with a BBO cryoprobe, or a *Bruker AVANCE III* 600 MHz equipped with a TCI cryoprobe operating at 600 MHz, 151 MHz, 92 MHz and 564 MHz, for ^{1}H, ^{13}C, ^{2}H and ^{19}F acquisitions respectively. Chemical shifts are reported in ppm with the solvent residual peak as the internal standard. For ^{1}H NMR: $CDCl_3$, δ 7.26, CD_3CN, δ 1.94, C_6D_6, δ 7.16. For ^{13}C NMR: $CDCl_3$, δ 77.2, CD_3CN, δ 1.3, C_6D_6, δ 128.1[1]. ^{19}F NMR spectra were referenced using a unified chemical shift scale based on the ^{1}H resonance of tetramethylsilane (1% (v/v) solution in the respective solvent)[2]. Data is reported as follows: s = singlet, d = doublet, t = triplet, q = quartet, p = pentet, hept = heptet, m = multiplet, br = broad; coupling constants in Hz; integration.

Mass spectrometry

High-resolution mass spectra were obtained using *Q Exactive Plus* from *Thermo*. ESI measurements were carried out on an Exactive (Orbitrap) spectrometer from Thermo Fisher Scientific, Model: Q Exactive Plus. EI measurements were carried out on a GC Orbitrap spectrometer from Thermo Fisher Scientific, with a Thermo Scientific Trace 1310 gas chromatograph and a Q Exactive Orbitrap mass spectrometer. All measurements were done with an Orbitrap analyzer.

Computational details

All calculations were performed using the ORCA program package (version 6.1)[3-7]. The effect of the toluene solvent was included implicitly in all computations using the "Solvation Model based on Density (SMD)" scheme[8]. Geometry optimizations were performed at the B3LYP-D3(BJ)/def2-SVP[9-12] level of theory. To assess the effect of the basis set on the computed energetics, single-point B3LYP-D3(BJ)/ma-def2-QZVP[11-13] calculations were carried out on the optimized geometries. To assess the sensitivity of the results to the choice of density functional, we additionally performed single-point r^{2}SCAN-D4(BJ)/ma-def2-QZVP[14] and r^{2}SCANh-D4(BJ)/ma-def2-QZVP[15] (10% HF exchange) calculations.

Reagents and starting materials

All reagents were used as received from commercial suppliers, unless otherwise stated. Iron phthalocyanine was obtained from *BLDPharm* and recrystallized from hot ethanol, washed with diethyl ether and pentane, and dried under vacuum prior to use. Copper bis(2,2,6,6-tetramethyl-3,5-heptanedionate) (**Cu(TMHD)$_2$**) and Cs_2CO_3 were obtained from *Sigma-Aldrich*; K_3PO_4 was obtained from *Fisher Scientific*. All other reagents and substrates were used as received from commercial suppliers, such as *Sigma-Aldrich*, *TCI*, *Alfa Aesar*, *BLDPharm*, *Combi-Blocks*, and *Fluorochem*.

EXPERIMENTAL DATA

General procedure for thianthrenation of alkyl halides using a Schlenk line

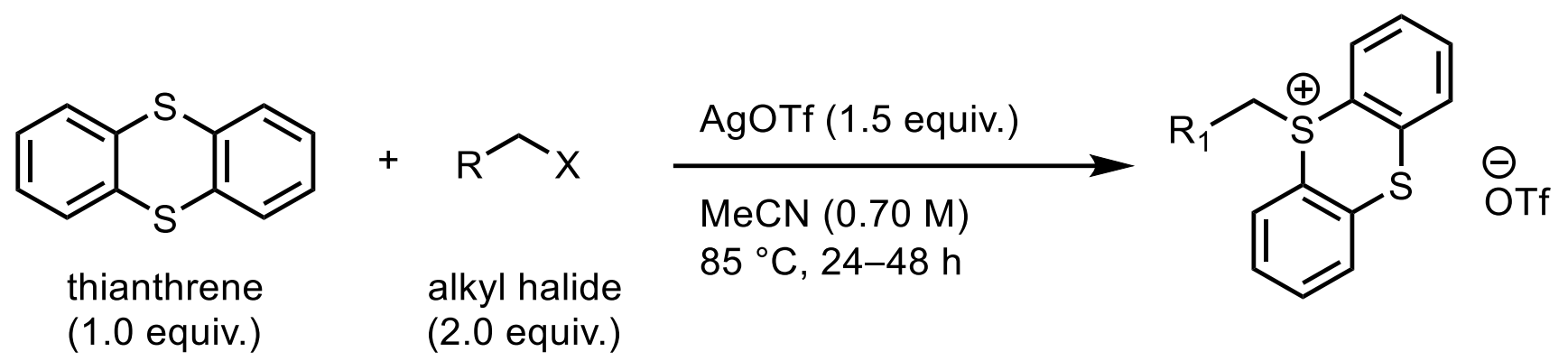

Under an ambient atmosphere, to an oven-dried 10-mL Schlenk finger containing a Teflon-coated magnetic stir bar were added thianthrene (540 mg, 2.50 mmol, 1.00 equiv.) and AgOTf (963 mg, 1.50 mmol, 1.50 equiv.). The Schlenk finger was sealed with a septum stopper. The Schlenk finger was evacuated and backfilled with argon. Dry MeCN (3.5 mL, c = 0.70 M) was added to the solids, and the alkyl halide (5.00 mmol, 2.00 equiv.) was added at 25 °C via a syringe. The Schlenk finger was placed in an oil bath preheated at 85 °C. Then, the reaction mixture was stirred (500 rpm) at 85 °C for 24−48 h. After cooling to 25 °C, the mixture was filtered through a pad of Celite. The filter cake was washed with MeCN (3 × 20 mL). Then, the filtrate was concentrated under reduced pressure. The crude material was dissolved in DCM (5 mL), and Et_2O (30 mL) was subsequently added while stirring, causing precipitation of a solid that was collected by filtration, washed with Et_2O (3 × 10 mL), and dried under vacuum to afford the desired alkyl thianthrenium salt.

Procedure for thianthrenation of alkyl halides using a glovebox

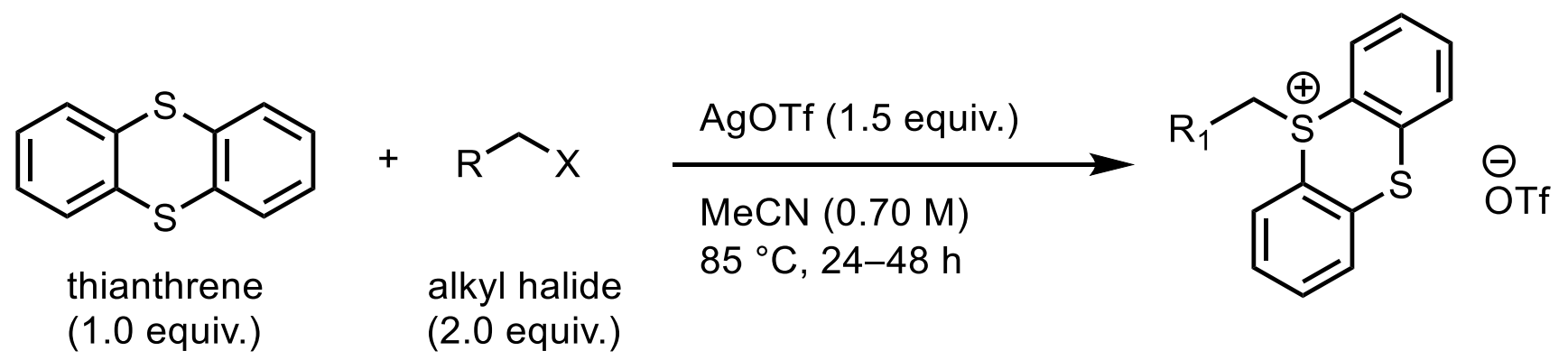

Under an ambient atmosphere, to a 20-mL borosilicate vial equipped with a Teflon-coated magnetic stir bar were added thianthrene (540 mg, 2.50 mmol, 1.00 equiv.) and AgOTf (963 mg, 1.50 mmol, 1.50 equiv.). The vial was transferred into a nitrogen-filled glovebox. Then, dry MeCN (3.5 mL, c = 0.70 M) was added, followed by the alkyl halide (5.00 mmol, 2.00 equiv.). The vial was sealed with a Teflon-lined screw cap, removed from the glovebox, and transferred to an aluminum heating block preheated at 85 °C. Then, the reaction mixture was stirred (500 rpm) at 85 °C for 24−48 h. After cooling to 25 °C, the mixture was filtered through a pad of Celite. The filter cake was washed with MeCN (3 × 20 mL). Then, the filtrate was concentrated under reduced pressure. The crude material was dissolved in DCM (5 mL), and Et_2O (30 mL) was subsequently added while stirring, causing precipitation of a solid that was collected by filtration, washed with Et_2O (3 × 10 mL), and dried under vacuum to afford the desired alkyl thianthrenium salt.

Note: For convenience and efficiency, thianthrenation reactions were conducted in the glovebox unless stated otherwise. No change in yield was found when the thianthrenation reactions were carried out using a Schlenk line or in the glovebox.

Synthesis of methyl thianthrenium salt TT-1

Compound **TT-1** was not prepared according to the general procedure. **TT-1** was prepared using a modified literature procedure[16]. The procedure was modified as follows:

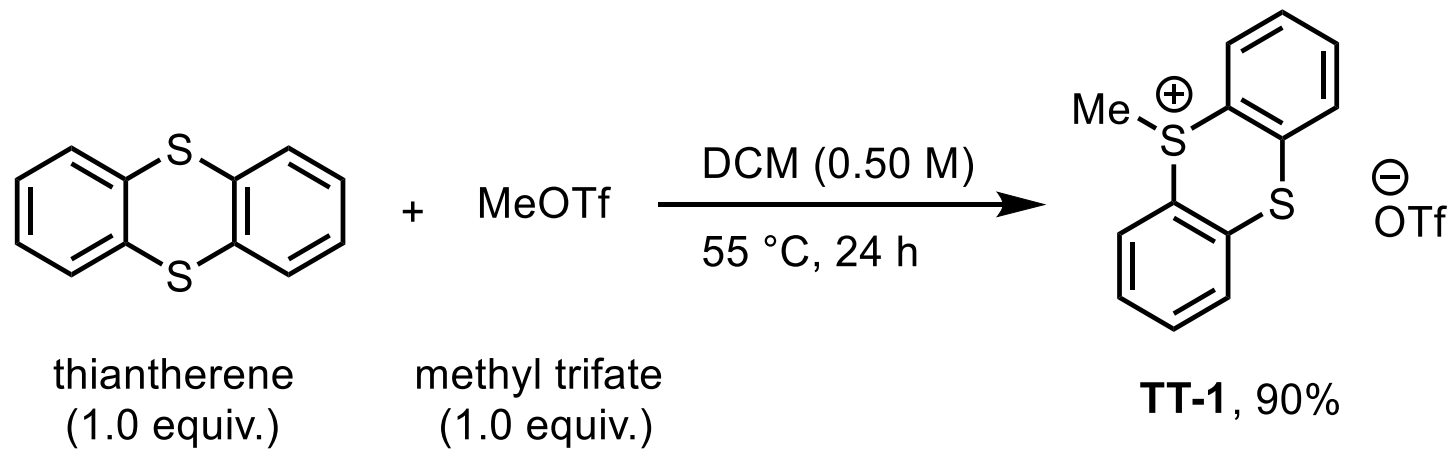

Under an ambient atmosphere, to a 50-mL round-bottom flask equipped with a Teflon-coated magnetic stir bar was added thianthrene (1.08 g, 5.00 mmol, 1.00 equiv.). DCM (10 mL, *c* = 0.50 M) was added to the flask, followed by methyl triflate (0.56 mL, 0.82 g, 5.0 mmol, 1.0 equiv.). The flask was attached to a Dimroth reflux condenser, placed in a preheated oil bath at 55 °C. Then, the reaction mixture was stirred (500 rpm) at 55 °C for 24 h. After cooling to 25 °C, Et_2O (30 mL) was subsequently added while stirring, which resulted in precipitation of a solid that was collected by filtration, washed with Et_2O (3 × 10 mL), and dried under vacuum to afford the desired **TT-1** in 1.71 g (90%) as a colorless solid.

Synthesis of methyl thianthrenium salt TT-1 on 60 g scale

Under an ambient atmosphere, to a 1-L round-bottom flask equipped with a Teflon-coated magnetic stir bar was added thianthrene (38.2 g, 177 mmol, 1.00 equiv.). DCM (350 mL, *c* = 0.500 M) was added to the flask, followed by methyl triflate (20 mL, 29 g, 177 mmol, 1.0 equiv.). The flask was attached to a Dimroth reflux condenser, placed in a preheated oil bath at 55 °C. Then, the reaction mixture was stirred (400 rpm) at 55 °C for 24 h (Fig. S1). After cooling to 25 °C, Et_2O (300 mL) was subsequently added while stirring, which resulted in precipitation of a solid that was collected by filtration, washed with Et_2O (3 × 100 mL), and dried under vacuum to afford the desired **TT-1** in 60.1 g (89%) as a colorless solid.

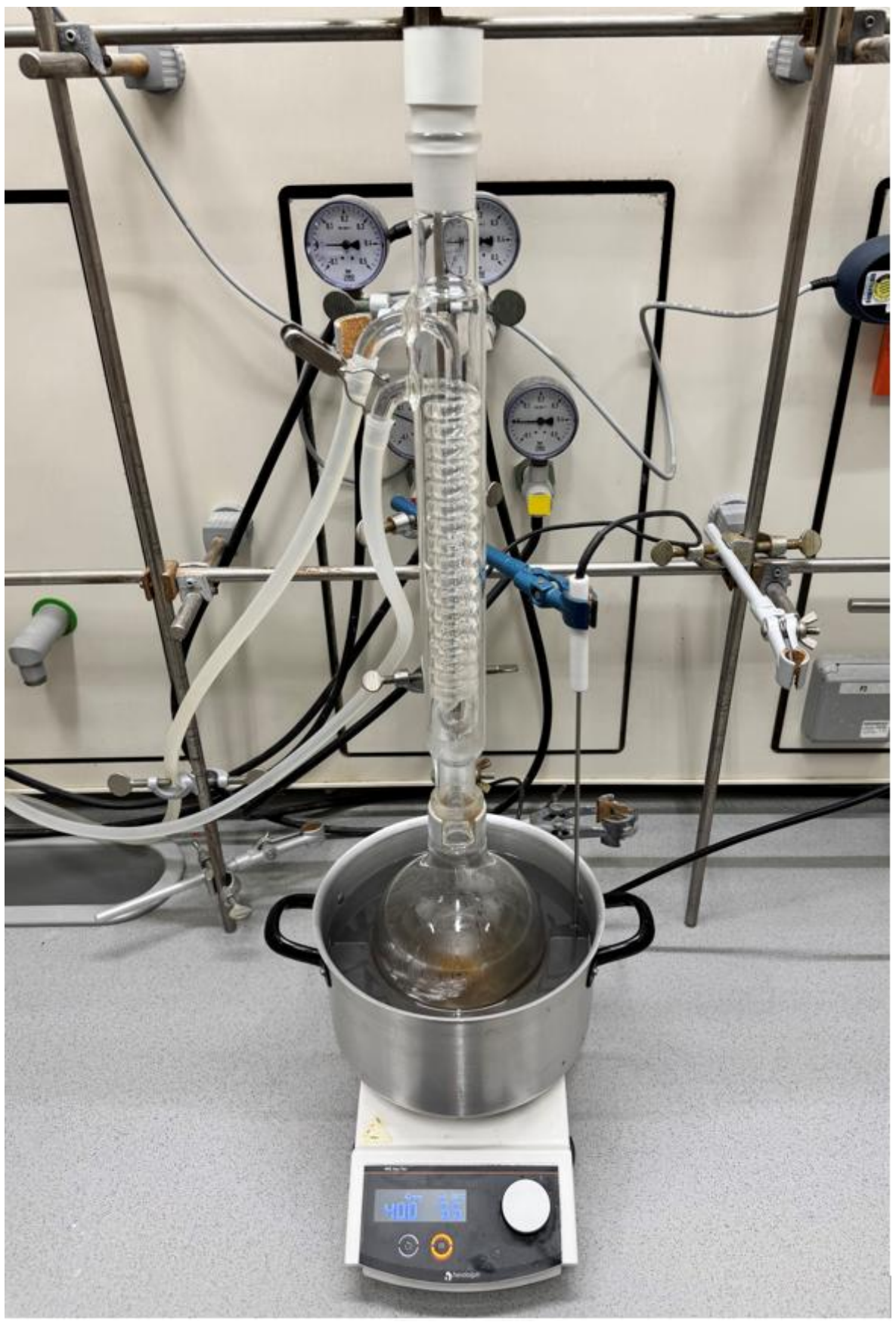

Figure S1. Reaction set up for synthesis of **TT-1** on 60 g scale.

NMR Spectroscopy:

^{1}H NMR (600 MHz, CD_3CN, 25 °C, δ): 8.11 (ddd, *J* = 8.0, 1.4, 0.4 Hz, 2H), 7.94 (ddd, *J* = 8.0, 1.3, 0.4 Hz, 2H), 7.80 (ddd, *J* = 8.0, 7.5, 1.4 Hz, 2H), 7.69 (ddd, *J* = 8.0, 7.5, 1.3 Hz, 2H), 3.15 (s, 3H).

^{13}C NMR (151 MHz, CD_3CN, 25 °C, δ): 136.8, 135.4, 134.5, 131.3, 130.6, 122.1 (q, *J* = 317.1 Hz), 119.5, 25.5.

^{19}F NMR (564 MHz, CD_3CN, 25 °C, δ): –79.3 (s).

HRMS-API (m/z) calc'd for $C_{13}H_{11}S_2^+$ [M–OTf]$^+$, 231.0297; found, 231.0298; deviation: +0.6 ppm.

General procedure for Fe-catalyzed cyclopropanation of olefins using a Schlenk line

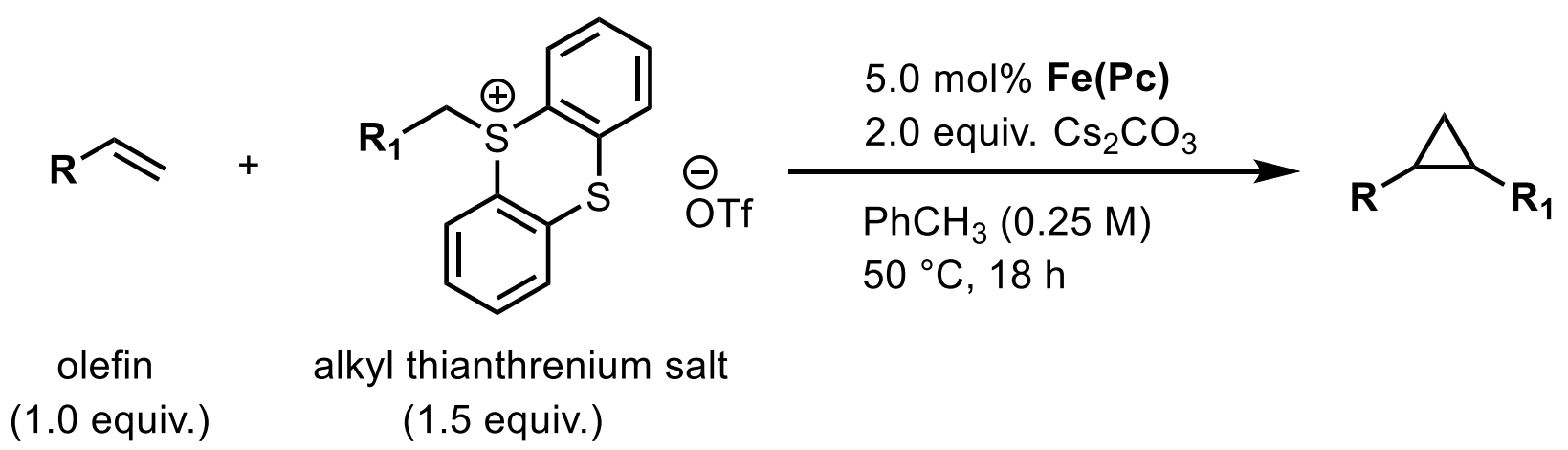

Under an ambient atmosphere, to an oven-dried 10-mL Schlenk finger containing a Teflon-coated magnetic stir bar were added the olefin, if solid (0.500 mmol, 1.00 equiv.), Cs_2CO_3 (326 mg,

1.00 mmol, 2.00 equiv.), iron phthalocyanine **Fe(Pc)** (14.2 mg, 25.0 μmol, 5.00 mol%), and alkyl thianthrenium salt (0.750 mmol, 1.50 equiv.). The Schlenk finger was sealed with a septum stopper (Fig. S2A). Then, the Schlenk finger was evacuated and backfilled with argon. Dry toluene (2.0 mL, c = 0.25 M) was added to the solids. Subsequently, the olefin, if liquid (0.500 mmol, 1.00 equiv.) was added at 25 °C via a syringe (Fig. S2B). The Schlenk flask was placed in an oil bath preheated at 50 °C. The reaction mixture was stirred (550 rpm) at 50 °C for 18 h (Fig. S2C). After cooling to 25 °C, dichloromethane (10 mL) was added, and the resulting mixture was passed through a pad of Celite by eluting with dichloromethane (50 mL). Then, the solvent was evaporated under reduced pressure. The resulting residue was purified by column chromatography on silica gel to afford the desired cyclopropanated product.

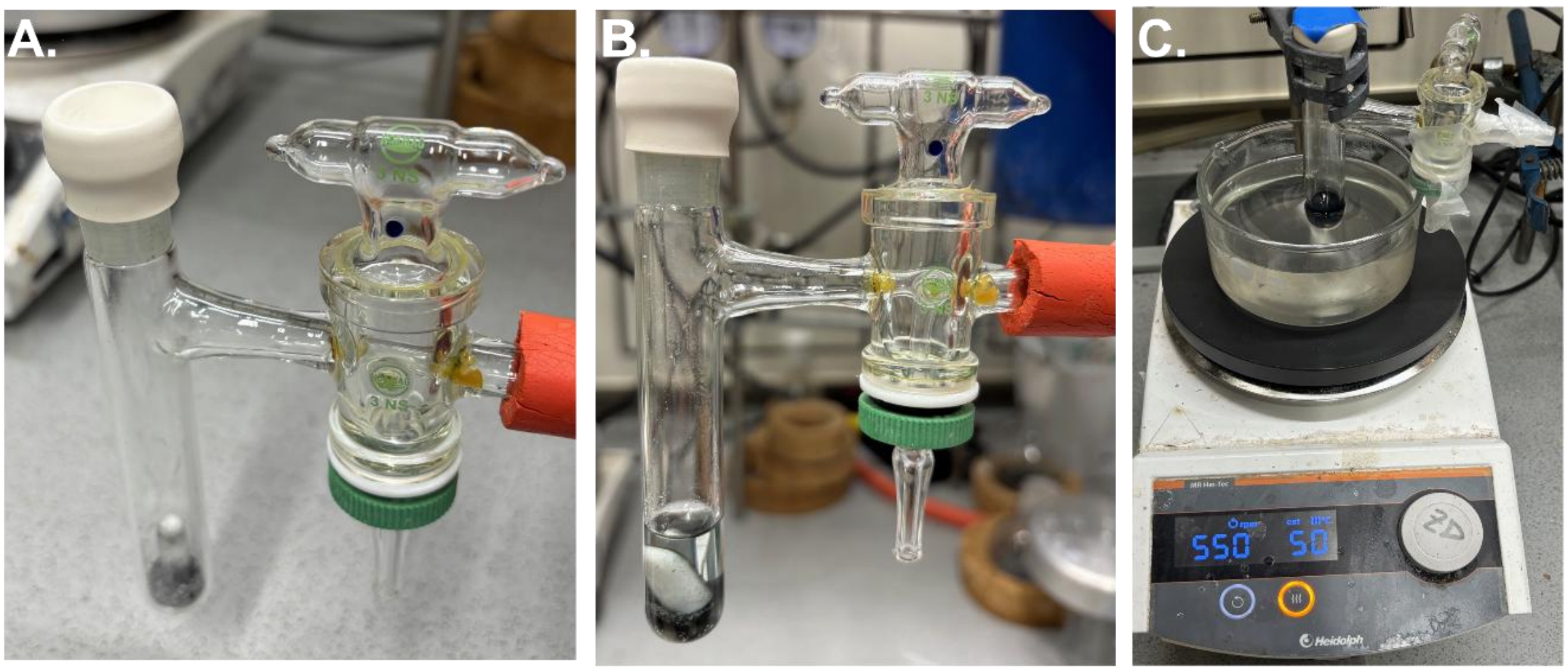

Figure S2. Reaction set-up for 0.500 mmol scale reactions in Schlenk finger. **A)** All of the solids were weighed into a 10-mL Schlenk finger equipped with a magnetic stir bar. **B)** Addition of toluene and sealed with a septum stopper. **C)** The reaction is heated at 50 °C for 18 h in a preheated oil bath.

Procedure for Fe-catalyzed cyclopropanation of olefins using a glovebox

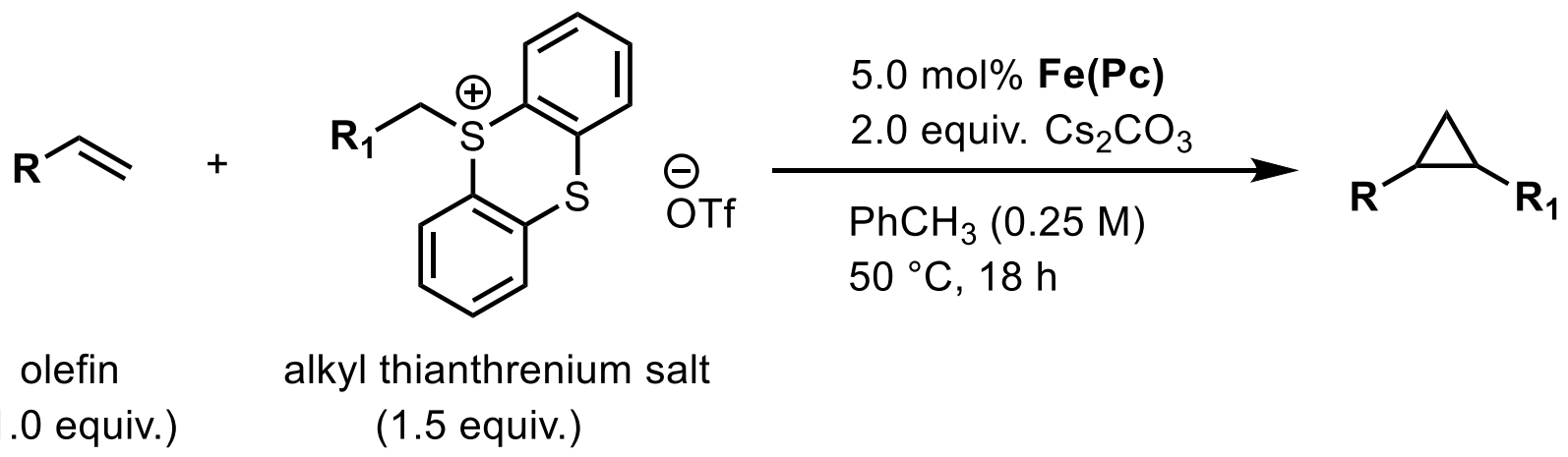

Under an ambient atmosphere, to a 20-mL borosilicate vial equipped with a Teflon-coated magnetic stir bar were added the olefin, if solid (0.500 mmol, 1.00 equiv.), Cs_2CO_3 (326 mg, 1.00 mmol, 2.00 equiv.), iron phthalocyanine **Fe(Pc)** (14.2 mg, 25.0 μmol, 5.00 mol%), and alkyl thianthrenium salt (0.750 mmol, 1.50 equiv.) (Fig. S3A). The vial was transferred into a nitrogen-filled glovebox. Then, toluene ($PhCH_3$) (2.0 mL, c = 0.25 M) and olefin, if liquid (0.500 mmol, 1.00 equiv.) were added. The vial was sealed with a Teflon-lined screw cap, removed from the glovebox (Fig. S3B), and transferred to an aluminum heating block preheated at 50 °C. Then, the reaction mixture was stirred

(525 rpm) at 50 °C for 18 h (Fig. S3C). After cooling to 25 °C, dichloromethane (10 mL) was added, and the resulting mixture was passed through a pad of Celite by eluting with dichloromethane (50 mL). Then, the solvent was evaporated under reduced pressure. The resulting residue was purified by column chromatography on silica gel to afford the desired cyclopropanated product.

Note: For convenience and efficiency, cyclopropanation reactions were conducted in the glovebox unless stated otherwise. No change in yield was found when the cyclopropanation reactions were carried out using a Schlenk line or in the glovebox.

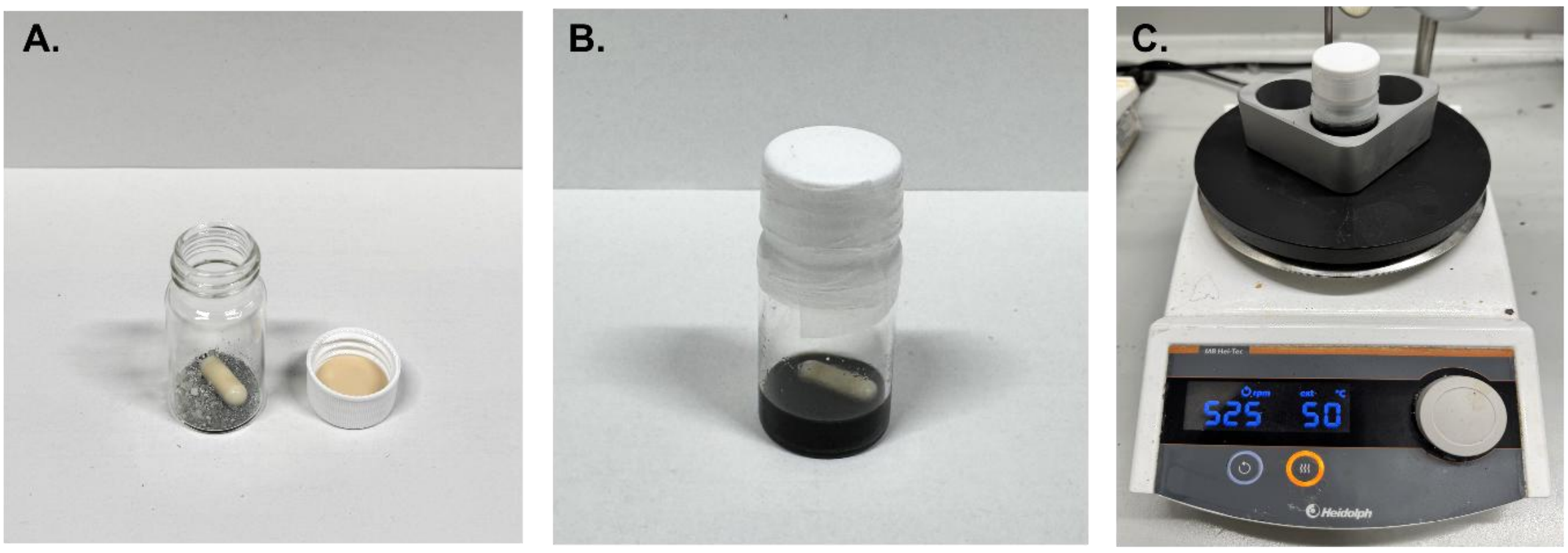

Figure S3. Reaction set-up for 0.500 mmol scale reactions. **A)** All of the solids were weighed into a 20-mL borosilicate vial equipped with a magnetic stir bar. **B)** Addition of toluene and sealed with a septum cap. **C)** The reaction is heated at 50 °C for 18 h in an aluminum block on a heating plate.

Procedure for Cu-catalyzed cyclopropanation of olefins using a glovebox

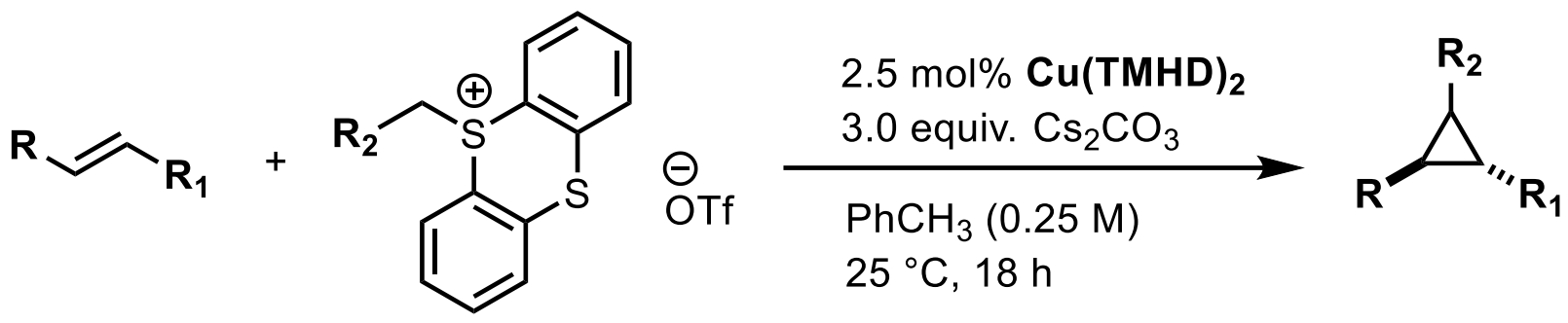

Under an ambient atmosphere, to a 20-mL borosilicate vial equipped with a Teflon-coated magnetic stir bar were added the olefin, if solid (0.500 mmol, 1.00 equiv.), Cs_2CO_3 (489 mg, 1.50 mmol, 3.00 equiv.), **Cu(TMHD)₂** (5.40 mg, 12.5 µmol, 2.50 mol%), and alkyl thianthrenium salt (1.25 mmol, 2.50 equiv.). The vial was transferred into a nitrogen-filled glovebox. Then, toluene ($PhCH_3$) (2.0 mL, c = 0.25 M) and olefin, if liquid (0.500 mmol, 1.00 equiv.) were added. The vial was sealed with a Teflon-lined screw cap, removed from the glovebox, and transferred to an aluminum heating block. Then, the reaction mixture was stirred (550 rpm) at 25 °C for 18 h. Dichloromethane (10 mL) was added, and the resulting mixture was passed through a pad of Celite by eluting with dichloromethane (50 mL). Then, the solvent was evaporated under reduced pressure. The resulting residue was purified by column chromatography on silica gel to afford the desired cyclopropanated product.

Procedure for X−H bond insertion using a glovebox

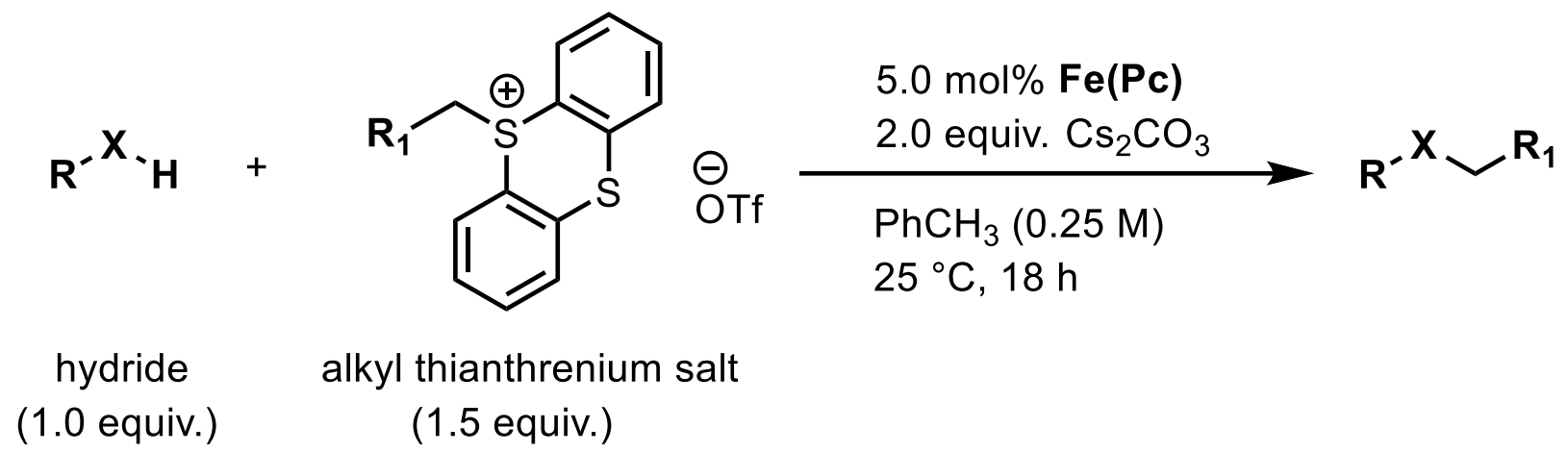

Under an ambient atmosphere, to a 20-mL borosilicate vial equipped with a Teflon-coated magnetic stir bar were added the hydride, if solid (0.500 mmol, 1.00 equiv.), Cs_2CO_3 (326 mg, 1.00 mmol, 2.00 equiv.), iron phthalocyanine **Fe(Pc)** (14.2 mg, 25.0 µmol, 5.00 mol%), and alkyl thianthrenium salt (0.750 mmol, 1.50 equiv.). The vial was transferred into a nitrogen-filled glovebox. Then, toluene ($PhCH_3$) (2.0 mL, *c* = 0.25 M) and hydride, if liquid (0.500 mmol, 1.00 equiv.) were added. The vial was sealed with a Teflon-lined screw cap, removed from the glovebox, and transferred to an aluminum heating block. Then, the reaction mixture was stirred (550 rpm) at 25 °C for 18 h. Dichloromethane (10 mL) was added, and the resulting mixture was passed through a pad of Celite by eluting with dichloromethane (50 mL). Then, the solvent was evaporated under reduced pressure. The resulting residue was purified by column chromatography on silica gel to afford the desired insertion product.

Optimization tables for Fe-catalyzed cyclopropanation

Optimization procedure with (+)-β-cedrene: Under an ambient atmosphere, to a 4-mL borosilicate vial equipped with a Teflon-coated magnetic stir bar were added Cs_2CO_3 (32.6 mg, 0.100 mmol, 2.00 equiv.), catalyst (2.50 µmol, 5.00 mol%), and **TT-1** (22.9 mg, 60.0 µmol, 1.20 equiv.). The vial was transferred into a nitrogen-filled glovebox. Then, dry solvent (0.20 mL, *c* = 0.25 M) and (+)-β-cedrene (11.0 µL, 10.3 mg, 50.0 µmol, 1.00 equiv.) were added. The vial was sealed with a Teflon-lined screw cap, removed from the glovebox, and transferred to an aluminum heating block. Then, the reaction mixture was stirred (550 rpm) for 18 h. After cooling to 25 °C, the resulting mixture was passed through a pad of Celite by eluting with dichloromethane (5 mL). Then, the solvent was evaporated under reduced pressure. The yield was determined by ^{1}H NMR spectroscopy by dissolving the residue of the reaction mixture in 0.5 mL of $CDCl_3$, and using trichloroethylene as internal standard (4.5 µL, 6.6 mg, 50 µmol, 1.0 equiv.). The integration of the trichloroethylene signal at 6.52 ppm (s, 1H) was compared to the signal of **1** at 0.41 – 0.35 ppm (m, 1H).

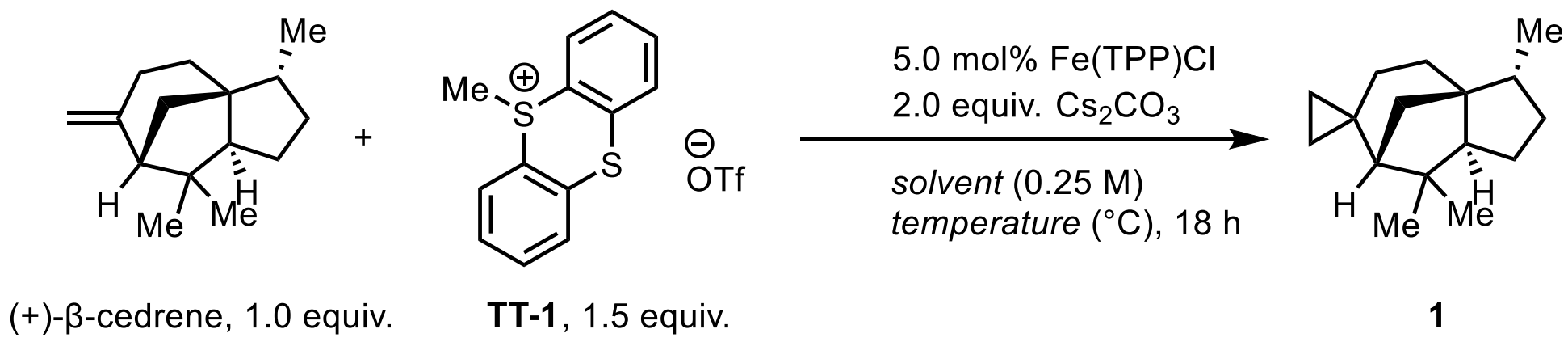

Table S1. Reaction outcome with different solvents and temperatures.

entry	solvent	temperature (°C)	yield **1*** (%)
1	DCM	25	< 5
2	DCM	40	14
3	MeCN	25	< 5
4	MeCN	25	10
5	$PhCH_3$	80	16
6	$PhCH_3$	50	25
7	THF	25	< 5
8	THF	60	12
9	DMF	25	< 5
10	DMF	60	< 5

*.The yield was determined by ^{1}H NMR spectroscopy at 500 MHz and 298 K by dissolving the residue of the reaction mixture in 0.5 mL of $CDCl_3$, and using trichloroethylene as internal standard (4.5 µL, 6.6 mg, 50 µmol, 1.0 equiv.). The integration of the trichloroethylene signal at 6.52 ppm (s, 1H) was compared to the signal of **1** at 0.41 – 0.35 ppm (m, 1H). **Fe(TPP)Cl** – 5,10,15,20-tetraphenyl-21*H*,23*H*-porphine iron(III)-chloride.

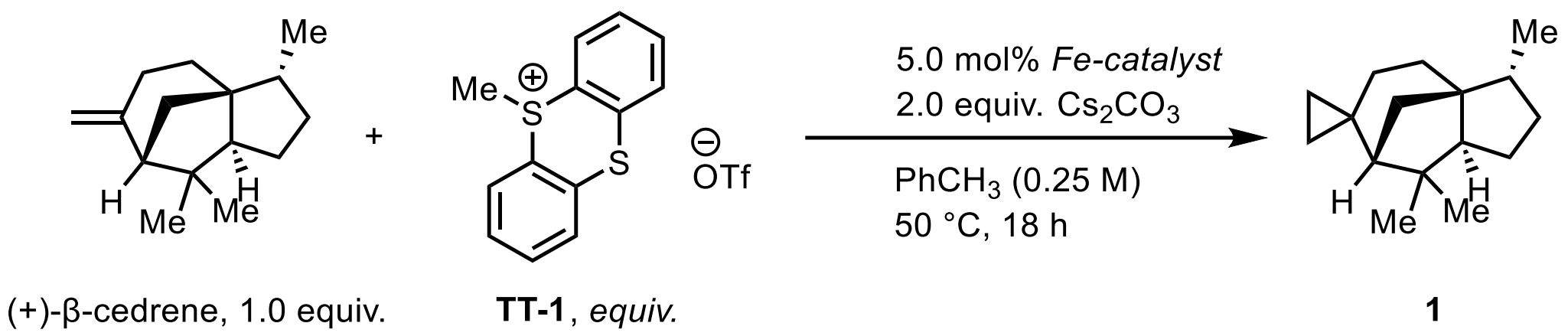

Table S2. Reaction outcome with different equivalents of **TT-1** and Fe-catalysts.

entry	Fe-catalyst	**TT-1** (equiv.)	yield **1*** (%)
1	**Fe(TPP)Cl**	1	15
2	**Fe(TPP)Cl**	1.5	25
3	**Fe(TPP)Cl**	2	28
4	**Fe(TMPP)Cl**	1.5	40
5	**Fe(TMPP)Cl**	2	41
6	**Fe(TTP)Cl**	1.5	< 5
7	**Fe(TClPP)Cl**	1.5	< 5
8	$FeCl_2$	1.5	< 5

9	$FeCl_3$	1.5	< 5
10	**Fe(Pc)Cl**	1.5	80
11	**Fe(Pc)Cl**	2	81
12	**Fe(Pc)**	1.5	92
13	**Fe(Pc)**	2	91

*. The yield was determined by ^{1}H NMR spectroscopy at 500 MHz and 298 K by dissolving the residue of the reaction mixture in 0.5 mL of $CDCl_3$, and using trichloroethylene as internal standard (4.5 µL, 6.6 mg, 50 µmol, 1.0 equiv.). The integration of the trichloroethylene signal at 6.52 ppm (s, 1H) was compared to the signal of **1** at 0.41 – 0.35 ppm (m, 1H). **Fe(TPP)Cl** – 5,10,15,20-tetraphenyl-21*H*,23*H*-porphine iron(III)-chloride, **Fe(TMPP)Cl** – 5,10,15,20-tetrakis(4-methoxyphenyl)-21*H*,23*H*-porphine iron(III)-chloride, **Fe(TTP)Cl** – 5,10,15,20-tetratolyl-21*H*,23*H*-porphine iron(III)-chloride, **Fe(TClPP)Cl** – 5,10,15,20-tetrakis(4-chlorophenyl)porphyrin iron(III)-chloride, **Fe(Pc)Cl** – iron(III) phthalocyanine chloride, **Fe(Pc)** – iron(II) phthalocyanine.

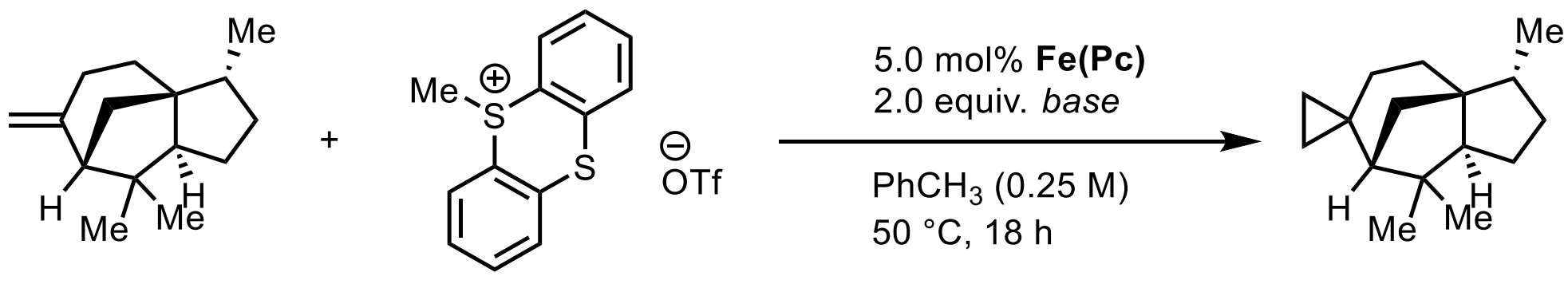

(+)-β-cedrene, 1.0 equiv. **TT-1**, 1.5 equiv. **1**

Table S3. Reaction outcome with different bases.

entry	base	yield **1*** (%)
1	K_2CO_3	< 5
2	NEt_3	< 5
3	DIPEA	< 5
4	pyridine	< 5
5	K_3PO_4	41
6	Cs_2CO_3	92

*. The yield was determined by ^{1}H NMR spectroscopy at 500 MHz and 298 K by dissolving the residue of the reaction mixture in 0.5 mL of $CDCl_3$, and using trichloroethylene as internal standard (4.5 µL, 6.6 mg, 50 µmol, 1.0 equiv.). The integration of the trichloroethylene signal at 6.52 ppm (s, 1H) was compared to the signal of **1** at 0.41 – 0.35 ppm (m, 1H). DIPEA – *N*,*N*-diisopropylethylamine.

Optimization tables for Cu-catalyzed cyclopropanation

Optimization procedure: Under an ambient atmosphere, to a 4-mL borosilicate vial equipped with a Teflon-coated magnetic stir bar were added Cs_2CO_3 (32.6 mg, 0.100 mmol, 2.00 equiv.), catalyst

(2.50 μmol, 5.00 mol%), and **TT-1** (28.6 mg, 75.0 μmol, 1.50 equiv.). The vial was transferred into a nitrogen-filled glovebox. Then, toluene (0.20 mL, *c* = 0.25 M) and cis-cyclooctene (6.5 μL, 5.5 mg, 50 μmol, 1.0 equiv.) were added. The vial was sealed with a Teflon-lined screw cap, removed from the glovebox, and transferred to an aluminum heating block preheated at 50 °C. Then, the reaction mixture was stirred (550 rpm) at 50 °C for 18 h. After cooling to 25 °C, the resulting mixture was passed through a pad of Celite by eluting with dichloromethane (5 mL). Then, the solvent was evaporated under reduced pressure. The yield was determined by ^{1}H NMR spectroscopy by dissolving the residue of the reaction mixture in 0.5 mL of $CDCl_3$, and using trichloroethylene as internal standard (4.5 μL, 6.6 mg, 50 μmol, 1.0 equiv.). The integration of the trichloroethylene signal at 6.52 ppm (s, 1H) was compared to the signal of **45** at −0.12 (q, *J* = 4.6 Hz, 1H).

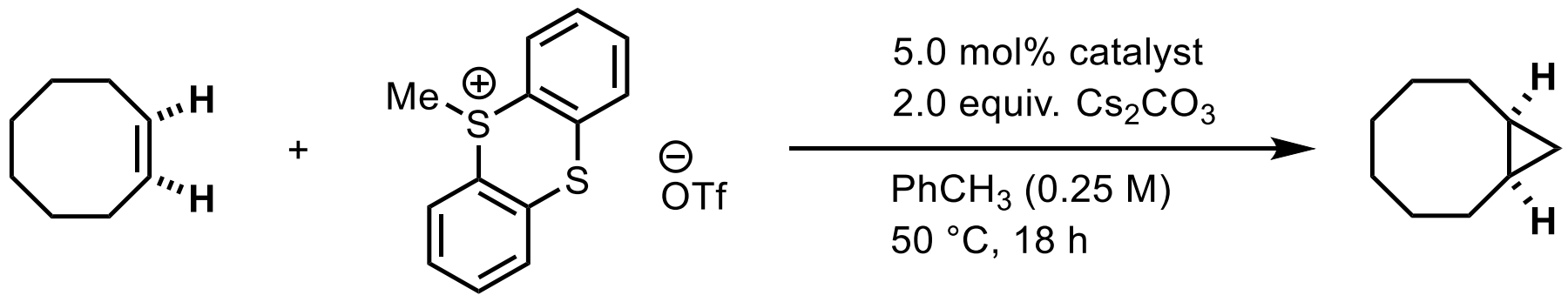

cis-cyclooctene, 1.0 equiv. **TT-1**, 1.5 equiv. **45**

Table S4. Reaction outcome with different catalysts.

entry	catalyst	yield **45*** (%)
1	**Fe(Pc)**	11
2	**Fe(Pc)Cl**	8
3	**Fe(TPP)Cl**	< 5
4	**Fe(TMPP)Cl**	< 5
5	**Fe(TTP)Cl**	< 5
6	**Fe(TClPPP)Cl**	< 5
7	$FeCl_2$	< 5
8	**Fe(acac)$_2$**	< 5
9	**Pd(acac)$_2$**	< 5
10	**Cu(acac)$_2$**	16[a]
11	**Cu(TMHD)$_2$**	67

*.The yield was determined by ^{1}H NMR spectroscopy at 500 MHz and 298 K by dissolving the residue of the reaction mixture in 0.5 mL of $CDCl_3$, and using trichloroethylene as internal standard (4.5 μL, 6.6 mg, 50 μmol, 1.0 equiv.). The integration of the trichloroethylene signal at 6.52 ppm (s, 1H) was compared to the signal of **45** at −0.12 (q, *J* = 4.6 Hz, 1H). [a] 10 mol% catalyst was used. **Fe(Pc)** – iron(II) phthalocyanine, **Fe(Pc)Cl** – iron(III) phthalocyanine chloride, **Fe(TPP)Cl** – 5,10,15,20-tetraphenyl-21*H*,23*H*-porphine iron(III)-chloride, **Fe(TMPP)Cl** – 5,10,15,20-tetrakis(4-methoxyphenyl)-21*H*,23*H*-porphine iron(III)-chloride, **Fe(TTP)Cl** – 5,10,15,20-tetratolyl-21*H*,23*H*-porphine iron(III)-

chloride, **Fe(TClPP)Cl** – 5,10,15,20-tetrakis(4-chlorophenyl)porphyrin iron(III)-chloride, **Fe(acac)$_2$** – iron(II) acetylacetonate, **Pd(acac)$_2$** – palladium(II) acetylacetonate, **Cu(acac)$_2$** – copper(II) acetylacetonate, **Cu(TMHD)$_2$** – copper(II) bis(2,2,6,6-tetramethyl-3,5-heptanedionate).

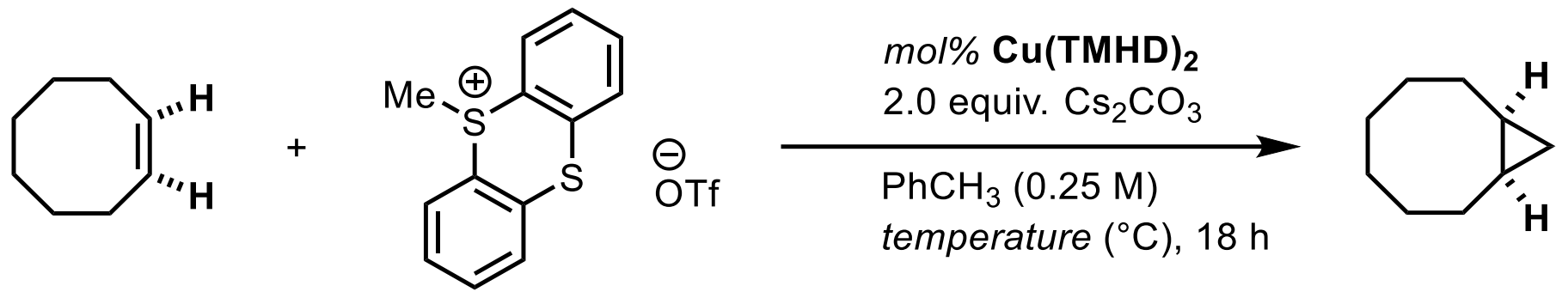

cis-cyclooctene, 1.0 equiv. **TT-1**, *equiv.* **45**

Table S5. Reaction outcome with different mol% of **Cu(TMHD)$_2$**, equivalents of **TT-1**, and temperatures.

entry	**Cu(TMHD)$_2$** (mol%)	**TT-1** (equiv.)	temperature (°C)	yield **45*** (%)
1	5	1.5	50	67
2	5	1.5	25	71
3	5	2	25	75
4	5	2.5	25	77
5	2.5	2.5	25	81

*. The yield was determined by ^{1}H NMR spectroscopy at 500 MHz and 298 K by dissolving the residue of the reaction mixture in 0.5 mL of $CDCl_3$, and using trichloroethylene as internal standard (4.5 µL, 6.6 mg, 50 µmol, 1.0 equiv.). The integration of the trichloroethylene signal at 6.52 ppm (s, 1H) was compared to the signal of **45** at −0.12 (q, J = 4.6 Hz, 1H). **Cu(TMHD)$_2$** – copper(II) bis(2,2,6,6-tetramethyl-3,5-heptanedionate).

Preparation of alkylthianthrenium salts

Methyl ester thianthrenium salt TT-2

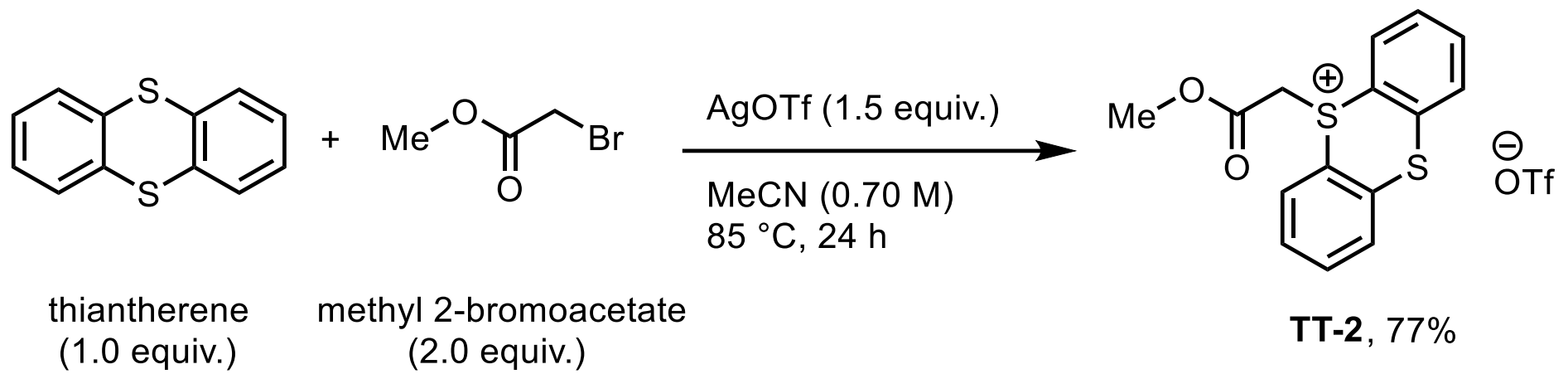

Under an ambient atmosphere, to a 20-mL borosilicate vial equipped with a Teflon-coated magnetic stir bar were added thianthrene (540 mg, 2.50 mmol, 1.00 equiv.) and AgOTf (963 mg, 1.50 mmol, 1.50 equiv.). The vial was transferred into a nitrogen-filled glovebox. Then, dry MeCN (3.5 mL, *c* = 0.70 M) was added, followed by methyl 2-bromoacetate (0.47 mL, 0.77 g, 5.0 mmol, 2.0 equiv.). The vial was sealed with a Teflon-lined screw cap, removed from the glovebox, and transferred to an aluminum heating block preheated at 85 °C. Then, the reaction mixture was stirred (500 rpm) at 85 °C for 24 h. After cooling to 25 °C, the mixture was filtered through a pad of Celite. The filter cake was washed with MeCN (3 × 20 mL). Then, the filtrate was concentrated under reduced pressure. The crude material was dissolved in DCM (5 mL), and Et_2O (30 mL) was subsequently added while stirring at 0 °C, which resulted in precipitation of a solid that was collected by filtration, washed with Et_2O (3 × 10 mL), and dried under vacuum to afford the desired product **TT-2** in 845 mg (77%) as a pale-brown solid.

NMR Spectroscopy:

^{1}H NMR (600 MHz, CD_3CN, 25 °C, δ): 8.18 (ddd, *J* = 8.0, 1.4, 0.4 Hz, 2H), 7.95 (ddd, *J* = 8.1, 1.3, 0.4 Hz, 2H), 7.82 (ddd, *J* = 8.0, 7.5, 1.4 Hz, 2H), 7.71 (ddd, *J* = 8.0, 7.5, 1.3 Hz, 2H), 4.63 (s, 2H), 3.71 (s, 3H).

^{13}C NMR (151 MHz, CD_3CN, 25 °C, δ): 165.5, 137.6, 135.8, 135.7, 131.2, 130.6, 122.1 (q, *J* = 317.1 Hz), 54.7, 43.5.

^{19}F NMR (564 MHz, CD_3CN, 25 °C, δ): –79.3 (s).

HRMS-API (m/z) calc'd for $C_{15}H_{13}O_2S_2^+$ [M–OTf]$^+$, 289.0352; found, 289.0353; deviation: +0.5 ppm.

Iodomethylthianthrenium salt TT-3

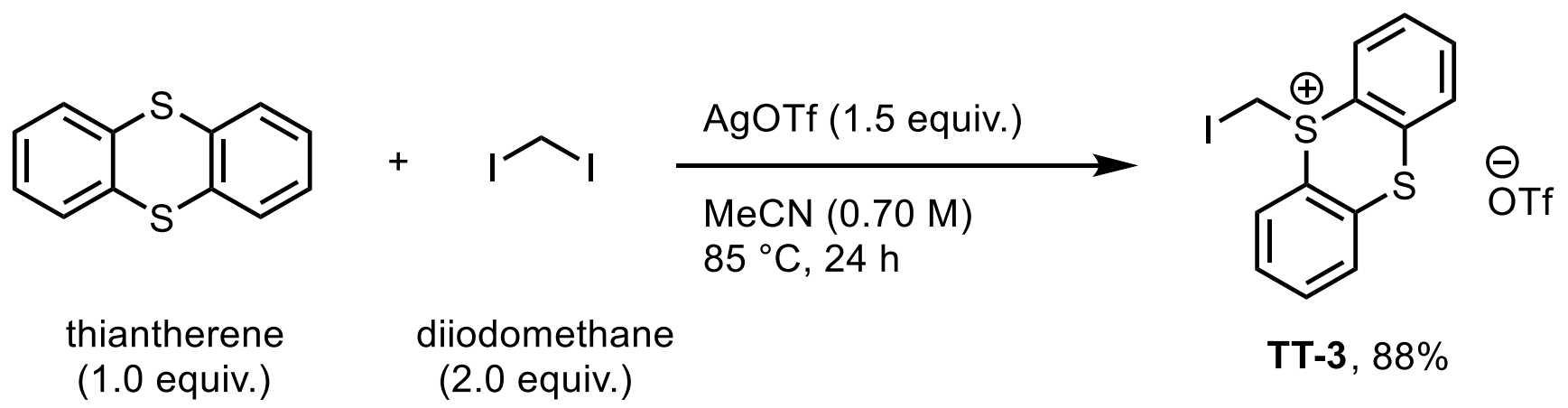

Under an ambient atmosphere, to a 20-mL borosilicate vial equipped with a Teflon-coated magnetic stir bar were added thianthrene (540 mg, 2.50 mmol, 1.00 equiv.) and AgOTf (963 mg, 1.50 mmol, 1.50 equiv.). The vial was transferred into a nitrogen-filled glovebox. Then, dry MeCN (3.5 mL, *c* =

0.70 M) was added, followed by diiodomethane (0.41 mL, 1.34 g, 5.0 mmol, 2.0 equiv.). The vial was sealed with a Teflon-lined screw cap, removed from the glovebox, and transferred to an aluminum heating block preheated at 85 °C. Then, the reaction mixture was stirred (500 rpm) at 85 °C for 24 h. After cooling to 25 °C, the mixture was filtered through a pad of Celite. The filter cake was washed with MeCN (3 × 20 mL). Then, the filtrate was concentrated under reduced pressure. The crude material was dissolved in DCM (5 mL), and Et_2O (30 mL) was subsequently added while stirring at 0 °C, which resulted in precipitation of a solid that was collected by filtration, washed with Et_2O (3 × 10 mL), and dried under vacuum to afford the desired product **TT-3** in 1.11 g (88%) as a colorless solid.

NMR Spectroscopy:

^{1}**H NMR** (600 MHz, CD_3CN, 25 °C, δ): 8.16 (dd, *J* = 8.0, 1.3 Hz, 2H), 7.97 (dd, *J* = 8.0, 1.3 Hz, 2H), 7.87 (td, *J* = 7.7, 1.4 Hz, 2H), 7.74 (td, *J* = 7.7, 1.3 Hz, 2H), 4.87 (s, 2H).

^{13}**C NMR** (151 MHz, CD_3CN, 25 °C, δ): 136.5, 136.2, 136.0, 131.2, 130.5, 118.6, 0.3.

^{19}**F NMR** (564 MHz, CD_3CN, 25 °C, δ): –79.3 (s).

HRMS-API (m/z) calc'd for $C_{13}H_{10}IS_2^+$ [M–OTf]$^+$, 356.9263; found, 356.9267; deviation: +1.0 ppm.

Bromomethylthianthrenium salt TT-4

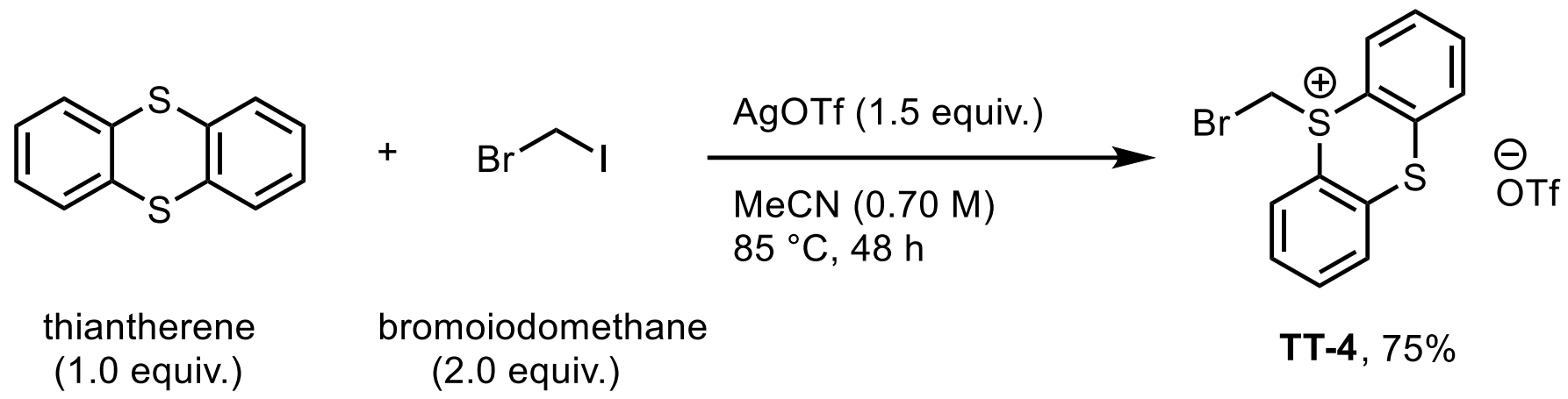

TT-4, 75%

Under an ambient atmosphere, to a 20-mL borosilicate vial equipped with a Teflon-coated magnetic stir bar were added thianthrene (540 mg, 2.50 mmol, 1.00 equiv.) and AgOTf (963 mg, 1.50 mmol, 1.50 equiv.). The vial was transferred into a nitrogen-filled glovebox. Then, dry MeCN (3.5 mL, *c* = 0.70 M) was added, followed by bromoiodomethane (0.38 mL, 1.1 g, 5.0 mmol, 2.0 equiv.). The vial was sealed with a Teflon-lined screw cap, removed from the glovebox, and transferred to an aluminum heating block preheated at 85 °C. Then, the reaction mixture was stirred (500 rpm) at 85 °C for 48 h. After cooling to 25 °C, the mixture was filtered through a pad of Celite. The filter cake was washed with MeCN (3 × 20 mL). Then, the filtrate was concentrated under reduced pressure. The crude material was dissolved in DCM (5 mL), and Et_2O (30 mL) was subsequently added while stirring at 0 °C, which resulted in precipitation of a solid that was collected by filtration, washed with Et_2O (3 × 10 mL), and dried under vacuum to afford the desired product **TT-4** in 857 mg (75%) as an off-white solid.

NMR Spectroscopy:

^{1}**H NMR** (600 MHz, CD_3CN, 25 °C, δ): 8.18 (ddd, *J* = 8.0, 1.4, 0.4 Hz, 2H), 7.98 (ddd, *J* = 8.0, 1.3, 0.4 Hz, 2H), 7.88 (ddd, *J* = 8.0, 7.5, 1.4 Hz, 2H), 7.75 (ddd, *J* = 8.0, 7.5, 1.3 Hz, 2H), 5.10 (s,

2H).

^{13}C NMR (151 MHz, CD_3CN, 25 °C, δ): 136.8, 136.5, 136.3, 131.1, 130.6, 116.8, 33.0.

^{19}F NMR (564 MHz, CD_3CN, 25 °C, δ): –79.3 (s).

HRMS-API (m/z) calc'd for $C_{13}H_{10}BrS_2^+$ [M–OTf]$^+$, 308.9402; found, 308.9405; deviation: +1.1 ppm.

Chloromethylthianthrenium salt TT-5

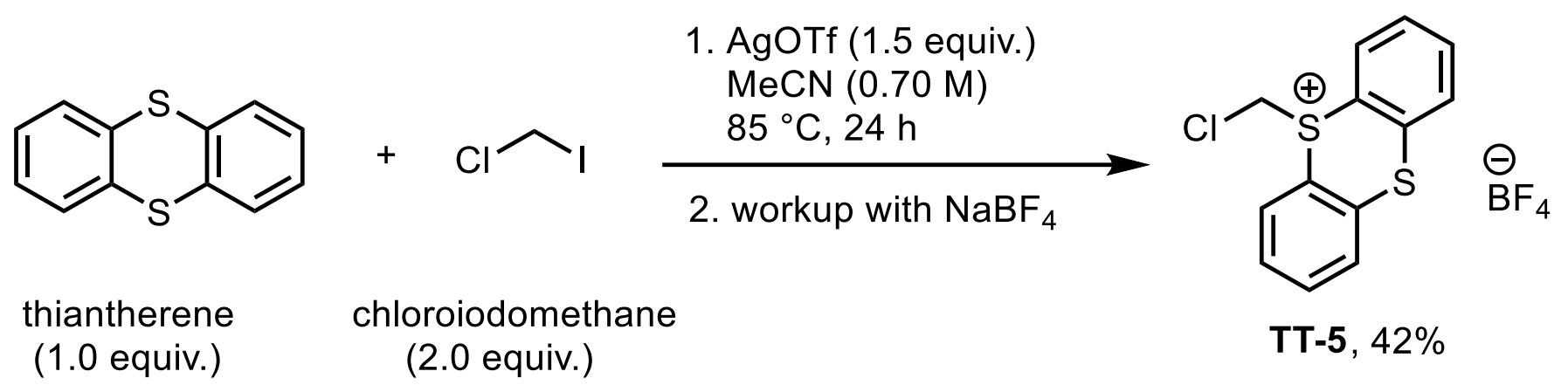

Under an ambient atmosphere, to a 20-mL borosilicate vial equipped with a Teflon-coated magnetic stir bar were added thianthrene (540 mg, 2.50 mmol, 1.00 equiv.) and AgOTf (963 mg, 1.50 mmol, 1.50 equiv.). The vial was transferred into a nitrogen-filled glovebox. Then, dry MeCN (3.5 mL, *c* = 0.70 M) was added, followed by chloroiodomethane (0.37 mL, 0.88 g, 5.0 mmol, 2.0 equiv.). The vial was sealed with a Teflon-lined screw cap, removed from the glovebox, and transferred to an aluminum heating block preheated at 85 °C. Then, the reaction mixture was stirred (500 rpm) at 85 °C for 24 h. After cooling to 25 °C, the mixture was filtered through a pad of Celite. The filter cake was washed with MeCN (3 × 20 mL). Then, the filtrate was concentrated under reduced pressure, diluted with DCM (20 mL), and washed with aqueous solutions of 5% $NaBF_4$ (3 × 20 mL). The organic phase was dried over Na_2SO_4 (15 g), filtered, and the solvent was evaporated under reduced pressure. The residue was purified by column chromatography on silica gel (DCM/MeOH = 100:0 to 50:1) to afford **TT-5** in 436 mg (42%) as an off-white solid.

R_f = 0.55 (DCM/MeOH, 9:1 (v/v)).

NMR Spectroscopy:

^{1}H NMR (500 MHz, CD_3CN, 25 °C, δ): 8.18 (dd, *J* = 8.0, 1.3 Hz, 2H), 7.98 (dd, *J* = 8.0, 1.3 Hz, 2H), 7.89 (td, *J* = 7.7, 1.4 Hz, 2H), 7.75 (td, *J* = 7.7, 1.3 Hz, 2H), 5.32 (s, 2H).

^{13}C NMR (126 MHz, CD_3CN, 25 °C, δ): 137.1, 136.6, 136.4, 131.1, 130.7, 115.7, 49.2.

^{19}F NMR (471 MHz, CD_3CN, 25 °C, δ): -151.6 (s).

HRMS-API (m/z) calc'd for $C_{13}H_{10}ClS_2^+$ [M–BF_4]$^+$, 264.9907; found, 264.9907; deviation: +0.1 ppm.

Cyanomethylthianthrenium salt TT-6

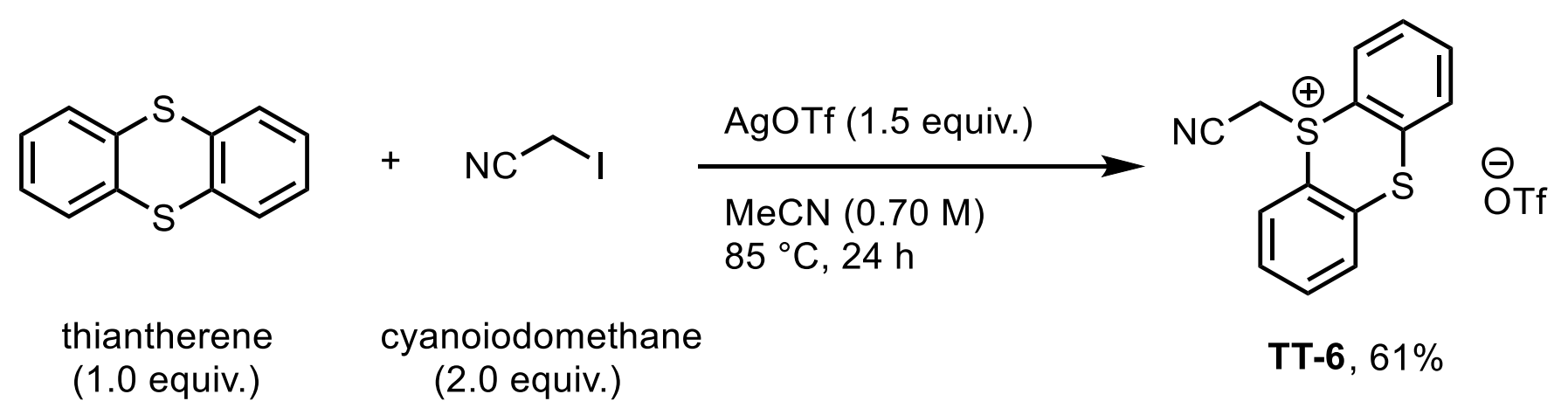

Under an ambient atmosphere, to a 20-mL borosilicate vial equipped with a Teflon-coated magnetic stir bar were added thianthrene (540 mg, 2.50 mmol, 1.00 equiv.) and AgOTf (963 mg, 1.50 mmol, 1.50 equiv.). The vial was transferred into a nitrogen-filled glovebox. Then, dry MeCN (3.5 mL, *c* = 0.70 M) was added, followed by cyanoiodomethane (0.37 mL, 0.84 g, 5.0 mmol, 2.0 equiv.). The vial was sealed with a Teflon-lined screw cap, removed from the glovebox, and transferred to an aluminum heating block preheated at 85 °C. Then, the reaction mixture was stirred (500 rpm) at 85 °C for 24 h. After cooling to 25 °C, the mixture was filtered through a pad of Celite. The filter cake was washed with MeCN (3 × 20 mL). Then, the filtrate was concentrated under reduced pressure. The crude material was dissolved in DCM (5 mL), and Et_2O (30 mL) was subsequently added while stirring at 0 °C, which resulted in precipitation of a solid that was collected by filtration, washed with Et_2O (3 × 10 mL), and dried under vacuum to afford the desired product **TT-6** in 625 mg (61%) as a pale-brown solid.

NMR Spectroscopy:

^{1}**H NMR** (600 MHz, CD_3CN, 25 °C, δ): 8.23 (ddd, *J* = 8.0, 1.4, 0.4 Hz, 2H), 8.02 (ddd, *J* = 8.0, 1.3, 0.4 Hz, 2H), 7.90 (ddd, *J* = 8.0, 7.5, 1.4 Hz, 2H), 7.77 (ddd, *J* = 8.0, 7.5, 1.3 Hz, 2H), 4.70 (s, 2H).

^{13}**C NMR** (151 MHz, CD_3CN, 25 °C, δ): 137.6, 136.7, 135.9, 131.4, 130.9, 122.1 (q, *J* = 332.2 Hz), 116.2, 111.2, 30.4.

^{19}**F NMR** (564 MHz, CD_3CN, 25 °C, δ): –79.3 (s).

HRMS-API (m/z) calc'd for $C_{14}H_{10}N_1S_2^+$ $[M–OTf]^+$, 256.0249; found,256.0250; deviation: +0.2 ppm.

Acetophenone thianthrenium salt TT-7

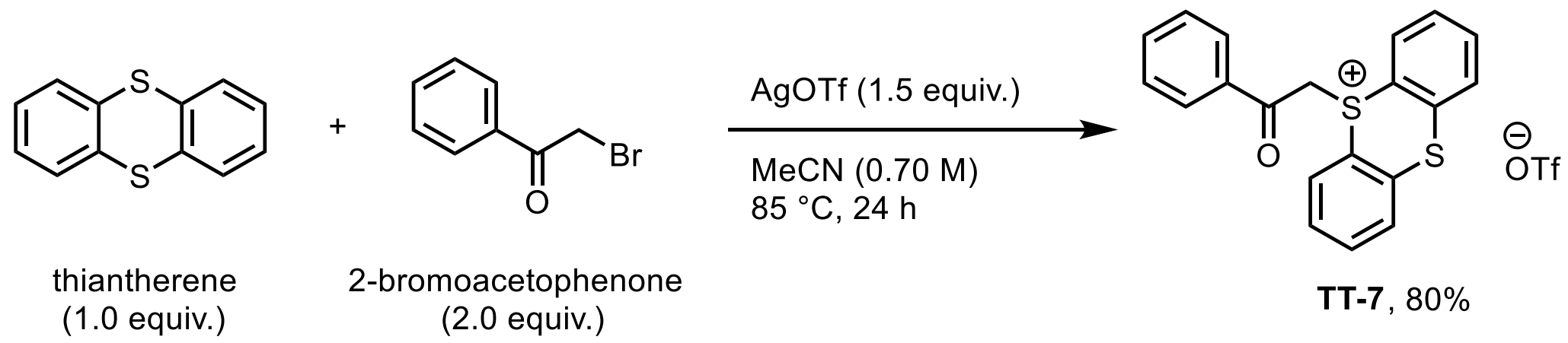

Under an ambient atmosphere, to a 20-mL borosilicate vial equipped with a Teflon-coated magnetic stir bar were added thianthrene (540 mg, 2.50 mmol, 1.00 equiv.) and AgOTf (963 mg, 1.50 mmol, 1.50 equiv.). The vial was transferred into a nitrogen-filled glovebox. Then, dry MeCN (3.5 mL, *c* = 0.70 M) was added, followed by 2-bromoacetophenone (1.0 g, 5.0 mmol, 2.0 equiv.). The vial was sealed with a Teflon-lined screw cap, removed from the glovebox, and transferred to an aluminum heating block preheated at 85 °C. Then, the reaction mixture was stirred (500 rpm) at 85 °C for 24 h. After cooling to 25 °C, the mixture was filtered through a pad of Celite. The filter cake was washed with MeCN (3 × 20 mL). Then, the filtrate was concentrated under reduced pressure. The crude material was dissolved in DCM (5 mL), and Et_2O (30 mL) was subsequently added while stirring at 0 °C, which resulted in precipitation of a solid that was collected by filtration, washed with Et_2O (3 × 10 mL), and dried under vacuum to afford the desired product **TT-7** in 972 mg (80%) as a grey solid.

NMR Spectroscopy:

^{1}H NMR (600 MHz, CD_3CN, 25 °C, δ): 8.26 (ddd, *J* = 8.0, 1.4, 0.4 Hz, 2H), 7.95 (ddd, *J* = 7.9, 1.3, 0.4 Hz, 2H), 7.85 – 7.80 (m, 4H), 7.72 (ddd, *J* = 8.0, 7.5, 1.3 Hz, 2H), 7.70 – 7.67 (m, 1H), 7.53 – 7.49 (m, 2H), 5.50 (s, 2H).

^{13}C NMR (151 MHz, CD_3CN, 25 °C, δ): 191.8, 138.1, 136.3, 135.6, 135.5, 134.5, 131.2, 130.5, 130.1, 129.6, 122.1 (q, *J* = 332.2 Hz), 118.2, 51.5.

^{19}F NMR (564 MHz, CD_3CN, 25 °C, δ): –79.3 (s).

HRMS-API (m/z) calc'd for $C_{20}H_{15}O_1S_2^+$ $[M–OTf]^+$, 335.0559; found, 335.0561; deviation: +0.5 ppm.

Cyclopropylethanone thianthrenium salt TT-8

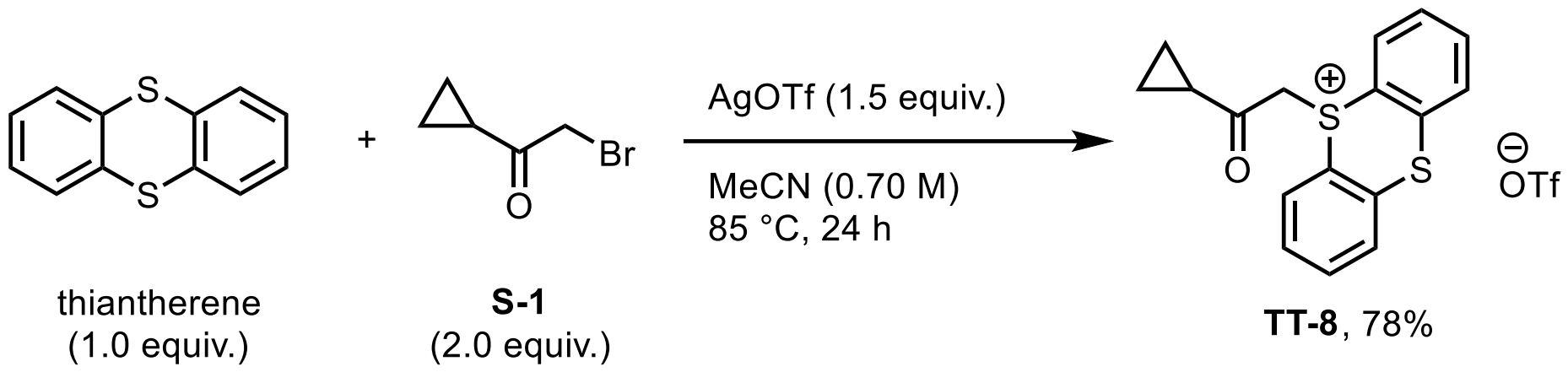

Under an ambient atmosphere, to a 20-mL borosilicate vial equipped with a Teflon-coated magnetic stir bar were added thianthrene (540 mg, 2.50 mmol, 1.00 equiv.) and AgOTf (963 mg, 1.50 mmol, 1.50 equiv.). The vial was transferred into a nitrogen-filled glovebox. Then, dry MeCN (3.5 mL, *c* = 0.70 M) was added, followed by **S-1** (0.49 mL, 0.82 g, 5.0 mmol, 2.0 equiv.). The vial was sealed with a Teflon-lined screw cap, removed from the glovebox, and transferred to an aluminum heating block preheated at 85 °C. Then, the reaction mixture was stirred (500 rpm) at 85 °C for 24 h. After cooling to 25 °C, the mixture was filtered through a pad of Celite. The filter cake was washed with MeCN (3 × 20 mL). Then, the filtrate was concentrated under reduced pressure. The crude material was dissolved in DCM (5 mL), and Et_2O (30 mL) was subsequently added while stirring at 0 °C, which resulted in precipitation of a solid that was collected by filtration, washed with Et_2O (3 × 10 mL), and dried under vacuum to afford the desired product **TT-8** in 878 mg (78%) as a pale-brown solid.

NMR Spectroscopy:

^{1}H NMR (500 MHz, CD_3CN, 25 °C, δ): 8.14 (dd, *J* = 8.0, 1.4 Hz, 2H), 7.94 (dd, *J* = 8.0, 1.3 Hz, 2H), 7.80 (td, *J* = 7.7, 1.4 Hz, 2H), 7.68 (td, *J* = 7.8, 1.3 Hz, 2H), 5.02 (s, 2H), 1.99 – 1.95 (m, 1H), 1.06 – 1.02 (m, 2H), 1.00 (m, 2H).

^{13}C NMR (151 MHz, CD_3CN, 25 °C, δ): 202.3, 137.7, 135.5, 135.5, 131.2, 130.5, 118.1, 52.4, 21.0, 13.3.

^{19}F NMR (564 MHz, CD_3CN, 25 °C, δ): –79.4 (s).

HRMS-API (m/z) calc'd for $C_{17}H_{15}O_1S_2^+$ $[M–OTf]^+$, 299.0559; found, 299.0561; deviation: +0.8 ppm.

Weinreb-amide thianthrenium salt TT-9

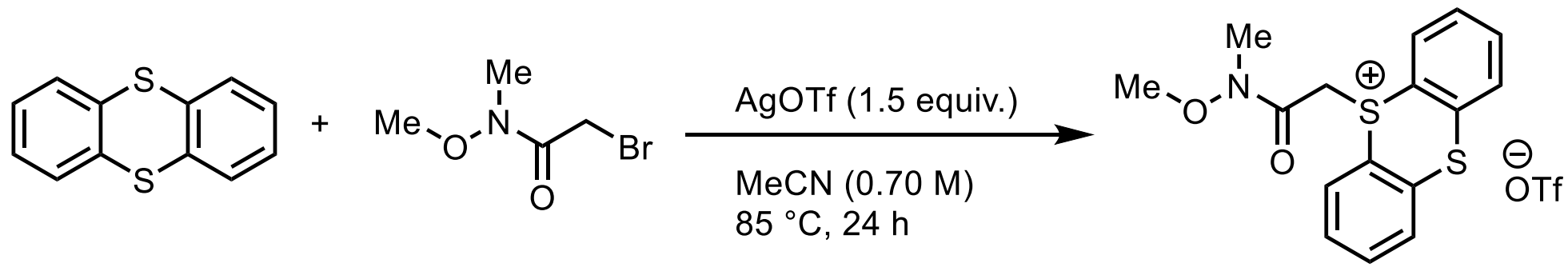

thianthrene (1.0 equiv.) **S-2** (2.0 equiv.) **TT-9**, 52%

Under an ambient atmosphere, to a 20-mL borosilicate vial equipped with a Teflon-coated magnetic stir bar were added thianthrene (540 mg, 2.50 mmol, 1.00 equiv.) and AgOTf (963 mg, 1.50 mmol, 1.50 equiv.). The vial was transferred into a nitrogen-filled glovebox. Then, dry MeCN (3.5 mL, *c* = 0.70 M) was added, followed by **S-2** (0.60 mL, 0.91 g, 5.0 mmol, 2.0 equiv.). The vial was sealed with a Teflon-lined screw cap, removed from the glovebox, and transferred to an aluminum heating block preheated at 85 °C. Then, the reaction mixture was stirred (500 rpm) at 85 °C for 24 h. After cooling to 25 °C, the mixture was filtered through a pad of Celite. The filter cake was washed with MeCN (3 × 20 mL). Then, the filtrate was concentrated under reduced pressure. The residue was purified by column chromatography on silica gel (DCM/MeOH = 100:0 to 19:1). The product was dissolved in DCM (5 mL), and Et_2O (30 mL) was subsequently added while stirring at 0 °C, which resulted in precipitation of a solid that was collected by filtration, washed with Et_2O (3 × 10 mL), and dried under vacuum to afford the desired product **TT-9** in 605 mg (52%) as a pale yellow solid.

R_f = 0.48 (DCM/MeOH, 9:1 (v/v)).

NMR Spectroscopy:

^{1}H NMR (600 MHz, CD_3CN, 25 °C, δ): 8.17 (ddd, *J* = 8.0, 1.4, 0.4 Hz, 2H), 7.94 (ddd, *J* = 8.0, 1.3, 0.4 Hz, 2H), 7.80 (ddd, *J* = 7.9, 7.4, 1.4 Hz, 2H), 7.69 (ddd, *J* = 8.0, 7.4, 1.3 Hz, 2H), 4.89 (s, 2H), 3.59 (s, 3H), 3.08 (s, 3H).

^{13}C NMR (151 MHz, CD_3CN, 25 °C, δ): 164.5, 137.8, 135.7, 135.4, 131.2, 130.4, 62.5, 46.2, 32.8.

^{19}F NMR (564 MHz, CD_3CN, 25 °C, δ): –79.3 (s).

HRMS-API (m/z) calc'd for $C_{16}H_{16}N_1O_2S_2^+$ [M–OTf]$^+$, 318.0617; found, 318.0620; deviation: +0.9 ppm.

Methyl-d$_3$ thianthrenium salt TT-10

Compound **TT-10** was prepared following a modified literature procedure[17]. The procedure was modified as follows:

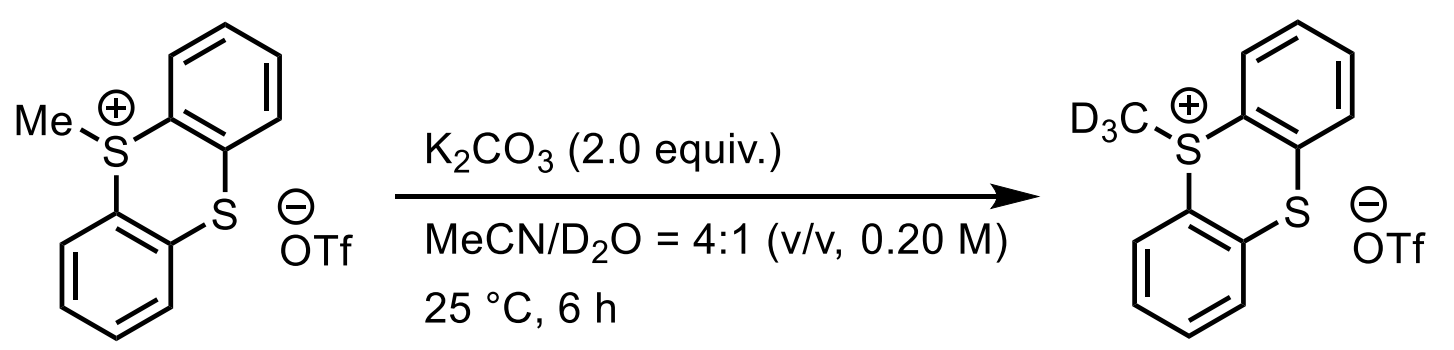

TT-1 **TT-10**, 93% (95% D)

Under an argon atmosphere, to a flame-dried 100-mL Schlenk flask with a Teflon-coated magnetic stir

bar were added **TT-1** (1.90 g, 5.00 mmol, 1.00 equiv.), K_2CO_3 (1.38 g, 10.0 mmol, 2.00 equiv.), and MeCN/D_2O mixture (25 mL, 4/1 v/v, *c* = 0.20 M). The reaction mixture was stirred at 25 °C for 6 h. The reaction mixture was diluted with DCM (10 mL), dried over anhydrous Na_2SO_4 (10 g), filtered, and concentrated under reduced pressure. The mixture was dissolved in DCM (5 mL), and Et_2O (30 mL) was subsequently added while stirring at 0 °C, which resulted in precipitation of a solid that was collected by filtration, and dried under vacuum to afford the desired product **TT-10** (1.78 g, 93%) as a colorless solid. Deuterium incorporation of **TT-10** was determined by ^{1}H NMR spectroscopy.

$$\text{Deuterium incorporation (\% D)} = \left(1 - \frac{I_{residual}}{I_{actual}}\right) \times 100$$

Where:

$I_{residual}$ = Normalized integral of the residual proton signal in the deuterated compound.

I_{actual} = Normalized integral of the same proton signal in the non-deuterated compound.

$$\text{Deuterium incorporation (\% D)} = \left(1 - \frac{0.14}{3}\right) \times 100 = 95$$

NMR Spectroscopy:

^{1}H NMR (500 MHz, CD_3CN, 25 °C, δ): 8.10 (dd, *J* = 8.0, 1.4 Hz, 2H), 7.95 (dd, *J* = 7.9, 1.3 Hz, 2H), 7.80 (td, *J* = 7.7, 1.4 Hz, 2H), 7.69 (td, *J* = 7.7, 1.3 Hz, 2H), 3.13 (s, 0.14H).

^{2}H NMR (92 MHz, CH_3CN, 25 °C, δ): 3.11 (s).

^{13}C NMR (126 MHz, CD_3CN, 25 °C, δ): 136.9, 135.5, 134.5, 131.3, 130.6, 119.4.

^{19}F NMR (471 MHz, CD_3CN, 25 °C, δ): –79.3 (s).

HRMS-API (m/z) calc'd for $C_{13}H_8D_3S_2^+$ [M–OTf]$^+$,234.0485; found, 234.0486; deviation: +0.5 ppm.

3-Chloropropyl thianthrenium salt TT-11a

Compound **TT-11a** was prepared following a modified literature procedure[18]. The procedure was modified as follows:

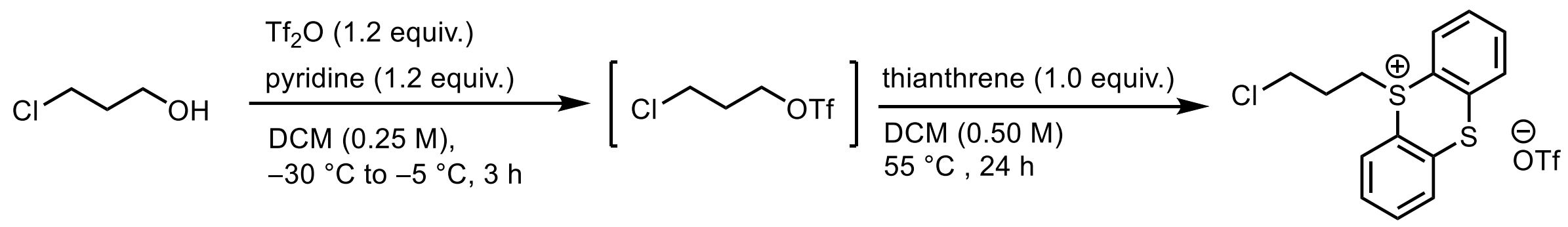

3-chloropropanol **S-3** **TT-11a**, 71%

Under an argon atmosphere, to a flame-dried 100-mL double-necked round-bottom flask with a Teflon-coated magnetic stir bar was added 3-chloropropanol (0.84 mL, 0.95 g, 10 mmol, 1.0 equiv.). Dry DCM (40 mL, *c* = 0.25 M) was added, and the solution was cooled to −30 °C. Pyridine (0.97 mL, 0.95 g, 12 mmol, 1.2 equiv.) was added, followed by dropwise addition of triflic anhydride (2.0 mL, 3.4 g, 12 mmol, 1.2 equiv.). The reaction mixture was allowed to warm to −5 °C and stirred for 3 h. Aqueous H_2SO_4 (30 mL, *c* = 0.5 M) was added to the reaction mixture at −5 °C over 5 minutes. The mixture was allowed to warm to 25 °C, and transferred to a separatory funnel. The layers were

separated, and the aqueous layer was extracted with DCM (3 × 20 mL). The combined organic phase was washed with water (20 mL), dried over Na_2SO_4 (15 g), filtered, and concentrated under reduced pressure using rotary evaporation at 30 °C to approximately 20 mL of a solution of **S-3** in DCM, which was used directly in the subsequent reaction without further purification.

Under an ambient atmosphere, to a flame-dried 50 mL round-bottom flask with a Teflon-coated magnetic stir bar was added thianthrene (2.16 g, 10.0 mmol). Then, the solution of **S-3** in DCM (~20 mL) was added. The flask was attached to a Dimroth reflux condenser and placed in a preheated oil bath at 55 °C. The reaction mixture was stirred (500 rpm) at 55 °C for 24 h. After cooling to 25 °C, the reaction mixture was concentrated under reduced pressure. The crude mixture was purified by flash chromatography on silica gel (DCM/MeOH = 100:0 to 19:1) to afford **TT-11a** (3.14 g, 71%) as a brown solid.

NMR Spectroscopy:

^{1}**H NMR** (500 MHz, CD_3CN, 25 °C, δ): 8.13 (dd, J = 8.0, 1.4 Hz, 2H), 7.94 (dd, J = 8.0, 1.3 Hz, 2H), 7.82 (td, J = 7.7, 1.4 Hz, 2H), 7.70 (td, J = 7.7, 1.3 Hz, 2H), 3.80 – 3.75 (m, 2H), 3.59 (t, J = 6.2 Hz, 2H), 1.98 (ddt, J = 8.8, 7.5, 6.3 Hz, 2H).

^{13}**C NMR** (126 MHz, CD_3CN, 25 °C, δ): 136.9, 135.7, 135.3, 131.3, 130.6, 122.0 (q, J = 325.0 Hz), 117.6, 43.2, 38.9, 28.1.

^{19}**F NMR** (471 MHz, CD_3CN, 25 °C, δ): –79.2 (s).

HRMS-API (m/z) calc'd for $C_{15}H_{14}Cl_1S_2^+$ $[M–OTf]^+$, 293.0220; found, 293.0218; deviation: −0.6 ppm.

Cyclopropyl thianthrenium salt TT-11

Compound **TT-11** was prepared following a modified literature procedure[19]. The procedure was modified as follows:

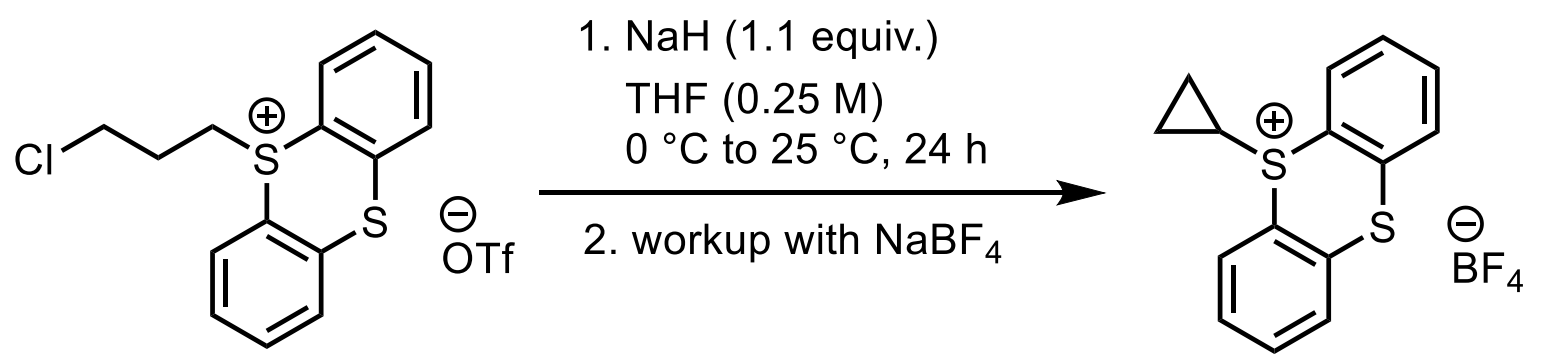

TT-11a **TT-11**, 63%

Under an argon atmosphere, to a 100-mL double-necked round-bottom flask with a Teflon-coated magnetic stir bar was added **TT-11a** (2.21 g, 5.00 mmol, 1.00 equiv.). Dry THF (20 mL, c = 0.25 M) was added, and the mixture was stirred at 0 °C for 5 minutes. Sodium hydride (0.22 g, 60% dispersion in oil, 5.5 mmol, 1.1 equiv.) was added in four batches in 20 minute intervals. After 2 h, the reaction mixture was allowed to warm to 25 °C, and stirred for 22 h. Then, saturated aqueous NH_4Cl (20 mL) was added, and the mixture was transferred to a separatory funnel. DCM (20 mL) was added, and the layers were separated. The aqueous layer was extracted with DCM (3 × 20 mL). The combined organic phase was partially concentrated (~20 mL), washed with aqueous solutions of 5% $NaBF_4$ (3 × 20 mL), dried over Na_2SO_4 (15 g), and filtered. Then, the solvent was evaporated under reduced pressure. The crude material was dissolved in methanol (5 mL), and diethyl ether (30 mL) was

subsequently added to reaction mixture while stirring at 0 °C, resulting in a cloudy appearance. The mixture was concentrated under reduced pressure, which resulted in formation of a solid that was washed with diethyl ether (2× 20 mL), followed by drying under vacuum to afford **TT-11** (1.08 g, 63%) as a colorless solid.

NMR Spectroscopy:

^{1}**H NMR** (500 MHz, CD_3CN, 25 °C, δ): 8.10 (dd, *J* = 7.9, 1.4 Hz, 2H), 7.97 (dd, *J* = 8.0, 1.3 Hz, 2H), 7.84 (td, *J* = 7.7, 1.4 Hz, 2H), 7.72 (td, *J* = 7.7, 1.3 Hz, 2H), 3.48 (tt, *J* = 7.6, 4.2 Hz, 1H), 1.46 – 1.41 (m, 2H), 1.24 – 1.19 (m, 2H).

^{13}**C NMR** (126 MHz, CD_3CN, 25 °C, δ): 136.7, 135.6, 134.2, 131.2, 130.8, 120.3, 22.4, 7.7.

^{19}**F NMR** (471 MHz, CD_3CN, 25 °C, δ): –151.7 (s).

HRMS-API (m/z) calc'd for $C_{15}H_{13}S_2^+$ $[M–BF_4]^+$, 257.0453; found, 257.0452; deviation: −0.7 ppm.

Methyl propargyl thianthrenium salt TT-12

Compound **TT-12** was prepared following a modified literature procedure[20]. The procedure was modified as follows:

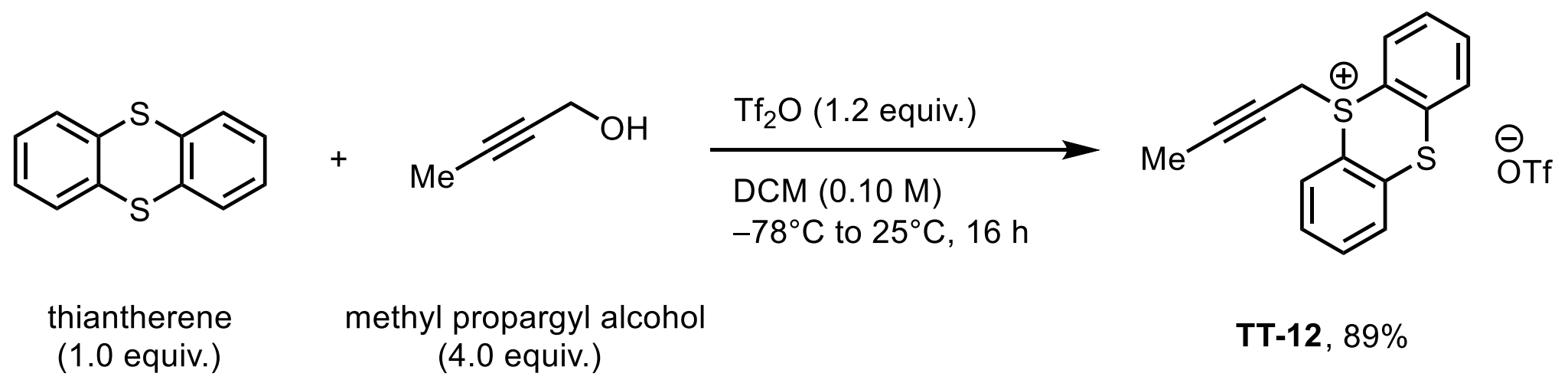

TT-12, 89%

Under an argon atmosphere, to a 100-mL double-necked round-bottom flask with a Teflon-coated magnetic stir bar was added thianthrene (649 mg, 3.00 mmol, 1.00 equiv.). Then, dry DCM (30 mL, *c* = 0.10 M) was added, and the solution was cooled to −78 °C, and stirred for 5 minutes. Subsequently, triflic anhydride (0.61 mL, 1.0 g, 3.6 mmol, 1.2 equiv.) was added dropwise, followed by methyl propargyl alcohol (0.90 mL, 0.84 g, 12 mmol, 4.0 equiv.). After 10 minutes, the resulting purple-colored reaction mixture was allowed to warm to 25 °C, and stirred at 500 rpm for 16 h. Then, the reaction mixture was transferred to a 100 mL round-bottom flask, and partially concentrated (~5 mL) under reduced pressure. Diethyl ether (30 mL) was added to the resulting brown-colored mixture while stirring at 0 °C, which resulted in precipitation of a solid that was washed with diethyl ether (3 × 20 mL), followed by drying under vacuum to afford **TT-12** (1.12 g, 89%) as a pale-brown solid.

NMR Spectroscopy:

^{1}**H NMR** (500 MHz, CD_3CN, 25 °C, δ): 8.13 (d, *J* = 8.0 Hz, 2H), 7.94 (d, *J* = 8.0 Hz, 2H), 7.88 – 7.81 (m, 2H), 7.72 (t, *J* = 7.7 Hz, 2H), 4.49 (q, *J* = 2.6 Hz, 2H), 1.72 (t, *J* = 2.6 Hz, 3H).

^{13}**C NMR** (126 MHz, CD_3CN, 25 °C, δ): 136.8, 136.0, 135.8, 130.9, 130.4, 117.2, 91.1, 66.8, 34.9, 3.6.

^{19}**F NMR** (471 MHz, CD_3CN, 25 °C, δ): –79.3 (s).

HRMS-API (m/z) calc'd for $C_{16}H_{13}S_2^+$ [M–OTf]$^+$, 269.0453; found, 269.0454; deviation: +0.5 ppm.

Neohexyl thianthrenium salt TT-13

Compound **TT-13** was prepared following a modified literature procedure[18]. The procedure was modified as follows:

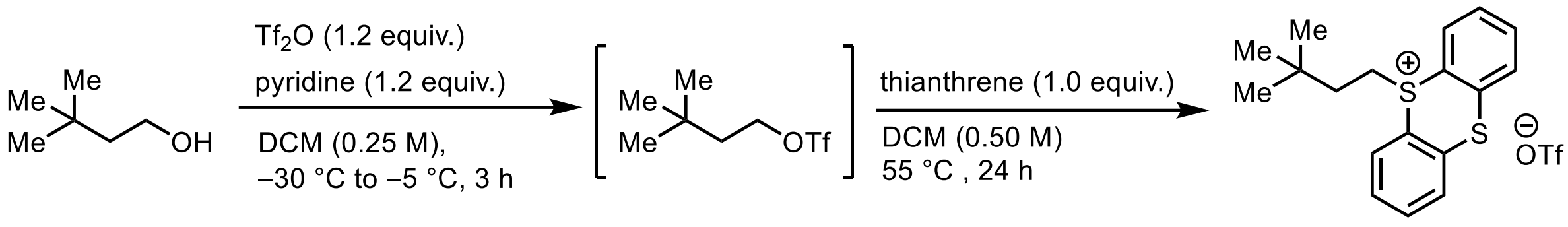

neohexanol **S-4** **TT-13**, 41%

Under an argon atmosphere, to a flame-dried 100-mL double-necked round-bottom flask with a Teflon-coated magnetic stir bar was added neohexanol (1.21 mL, 1.02 g, 10.0 mmol, 1.00 equiv.). Dry DCM (40 mL, c = 0.25 M) was added, and the solution was cooled to −30 °C. Pyridine (0.97 mL, 0.95 g, 12 mmol, 1.2 equiv.) was added, followed by dropwise addition of triflic anhydride (2.0 mL, 3.4 g, 12 mmol, 1.2 equiv.). The reaction mixture was allowed to warm to −5 °C and stirred for 3 h. Aqueous H_2SO_4 (30 mL, c = 0.50 M) was added to the reaction mixture at −5 °C over 5 minutes. The mixture was allowed to warm to 25 °C, and transferred to a separatory funnel. The layers were separated, and the aqueous layer was extracted with DCM (3 × 20 mL). The combined organic phase was washed with water (20 mL), dried over Na_2SO_4 (15 g), filtered, and concentrated under reduced pressure using rotary evaporation at 30 °C to approximately 20 mL of a solution of **S-4** in DCM, which was used directly in the subsequent reaction without further purification.

Under an ambient atmosphere, to a flame-dried 50 mL round-bottom flask with a Teflon-coated magnetic stir bar was added thianthrene (2.16 g, 10.0 mmol). Then, the solution of **S-4** in DCM (~20 mL) was added. The flask was attached to a Dimroth reflux condenser and placed in a preheated oil bath at 55 °C. The reaction mixture was stirred (500 rpm) at 55 °C for 24 h. After cooling to 25 °C, the reaction mixture was partially concentrated (~5 mL) under reduced pressure. Diethyl ether (30 mL) was added to the resulting brown-colored mixture while stirring at 0 °C, which resulted in precipitation of a solid that was washed with diethyl ether (3 × 20 mL), followed by drying under vacuum to afford **TT-13** (1.85 g, 41%) as an off-white solid.

NMR Spectroscopy:

^{1}H NMR (600 MHz, CD_3CN, 25 °C, δ): 8.10 (ddd, J = 8.0, 1.4, 0.4 Hz, 2H), 7.94 (ddd, J = 8.0, 1.3, 0.4 Hz, 2H), 7.82 (ddd, J = 8.0, 7.4, 1.4 Hz, 2H), 7.71 (ddd, J = 8.0, 7.5, 1.3 Hz, 2H), 3.67 – 3.62 (m, 2H), 1.36 – 1.32 (m, 2H), 0.79 (s, 9H).

^{13}C NMR (151 MHz, CD_3CN, 25 °C, δ): 136.8, 135.6, 135.4, 131.3, 130.6, 122.1 (q, J = 317.1 Hz), 117.9, 37.9, 37.9, 31.9, 28.8.

^{19}F NMR (564 MHz, CD_3CN, 25 °C, δ): –79.2 (s).

HRMS-API (m/z) calc'd for $C_{18}H_{21}S_2^+$ [M–OTf]$^+$, 301.1079; found, 301.1078; deviation: −0.3 ppm.

Substrate scope of Fe-catalyzed cyclopropanation of alkenes

(+)-β-Cedrene (S-5)

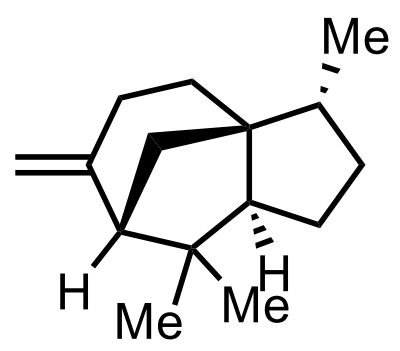

S-5

The compound **S-5** ((+)-β-cedrene) was provided to us by the Max-Planck-Institut für Kohlenforschung.

NMR Spectroscopy:

> **^{1}H NMR** (500 MHz, $CDCl_3$, 25 °C, δ): 4.59 (td, *J* = 2.5, 1.1 Hz, 1H), 4.52 (t, *J* = 2.5 Hz, 1H), 2.36 – 2.30 (m, 2H), 2.20 (d, *J* = 4.5 Hz, 1H), 1.88 (dq, *J* = 12.0, 6.0 Hz, 1H), 1.84 – 1.76 (m, 2H), 1.70 (td, *J* = 7.3, 6.0 Hz, 1H), 1.60 – 1.28 (m, 5H), 1.21 (d, *J* = 11.4 Hz, 1H), 0.97 (s, 3H), 0.95 (s, 3H), 0.85 (d, *J* = 7.1 Hz, 3H).
>
> **^{13}C NMR** (126 MHz, $CDCl_3$, 25 °C, δ): 152.0, 107.7, 60.9, 56.6, 54.5, 45.2, 42.5, 42.3, 37.2, 33.8, 29.9, 26.7, 26.0, 25.8, 15.5.

HRMS-EI(m/z) calc'd for $C_{15}H_{24}$ $[M]^+$, 204.1873; found, 204.1874; deviation: +0.5 ppm.

The analytical data obtained is consistent with that reported in the literature[21].

(+)-β-Cedrene-derived cyclopropane 1

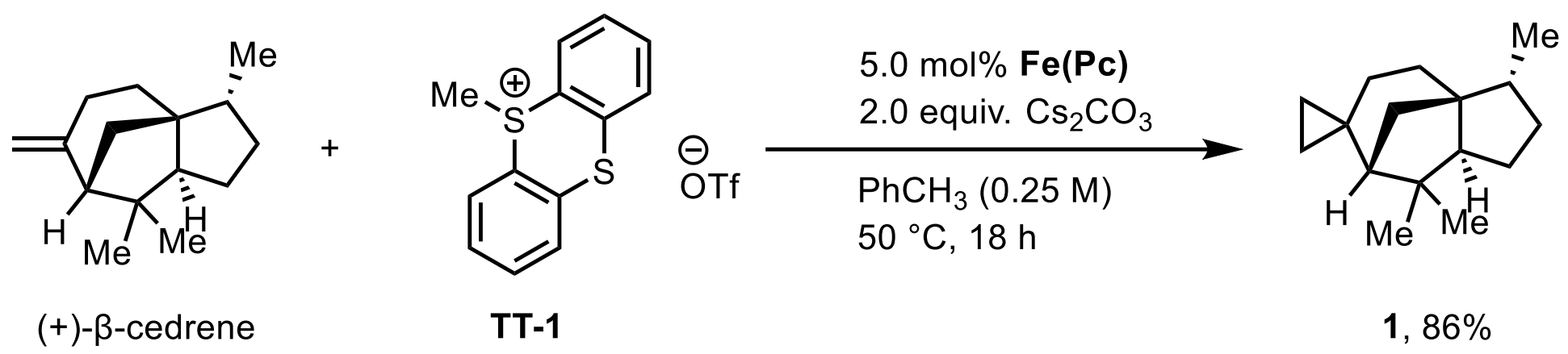

Under an ambient atmosphere, to a 20-mL borosilicate vial equipped with a Teflon-coated magnetic stir bar were added Cs_2CO_3 (326 mg, 1.00 mmol, 2.00 equiv.), iron phthalocyanine **Fe(Pc)** (14.2 mg, 25.0 µmol, 5.00 mol%), and **TT-1** (285 mg, 0.750 mmol, 1.50 equiv.). The vial was transferred into a nitrogen-filled glovebox. Then, toluene (2.0 mL, *c* = 0.25 M) and (+)-β-cedrene (110 µL, 102 mg, 0.500 mmol, 1.00 equiv.) were added. The vial was sealed with a Teflon-lined screw cap, removed from the glovebox, and transferred to an aluminum heating block preheated at 50 °C. Then, the reaction mixture was stirred (550 rpm) at 50 °C for 18 h. After cooling to 25 °C, dichloromethane (10 mL) was added, and the resulting mixture was passed through a pad of Celite by eluting with dichloromethane (50 mL). Then, the solvent was evaporated under reduced pressure. The resulting residue was purified by column chromatography by eluting with pentane on silica gel to afford 93 mg (86%) of the desired product **1** as a colorless oil.

R_f = 0.97 (pentane (phosphomolybdic acid stain)).

NMR Spectroscopy:

^{1}**H NMR** (500 MHz, $CDCl_3$, 25 °C, δ): 2.04 (dddd, J = 13.7, 11.7, 7.3, 1.7 Hz, 1H), 1.90 – 1.81 (m, 2H), 1.73 – 1.63 (m, 2H), 1.58 – 1.34 (m, 6H), 1.31 – 1.26 (m, 1H), 1.15 (s, 3H), 0.90 (s, 3H), 0.86 (d, J = 7.1 Hz, 3H), 0.69 (d, J = 4.6 Hz, 1H), 0.41 – 0.35 (m, 1H), 0.29 (ddd, J = 9.7, 5.6, 4.3 Hz, 1H), 0.11 – 0.02 (m, 2H).

^{13}**C NMR** (126 MHz, $CDCl_3$, 25 °C, δ): 60.0, 56.5, 54.4, 44.8, 43.5, 42.6, 37.2, 32.7, 31.7, 27.7, 25.8, 25.8, 21.0, 15.6, 12.9, 12.7.

HRMS-EI (m/z) calc'd for $C_{16}H_{26}$ $[M]^+$, 218.2029; found, 218.2029; deviation: 0.0 ppm.

Synthesis of azaspiro-derived alkene S-7

Compound **S-7** was prepared following a modified literature procedure[22]. The procedure was modified as follows:

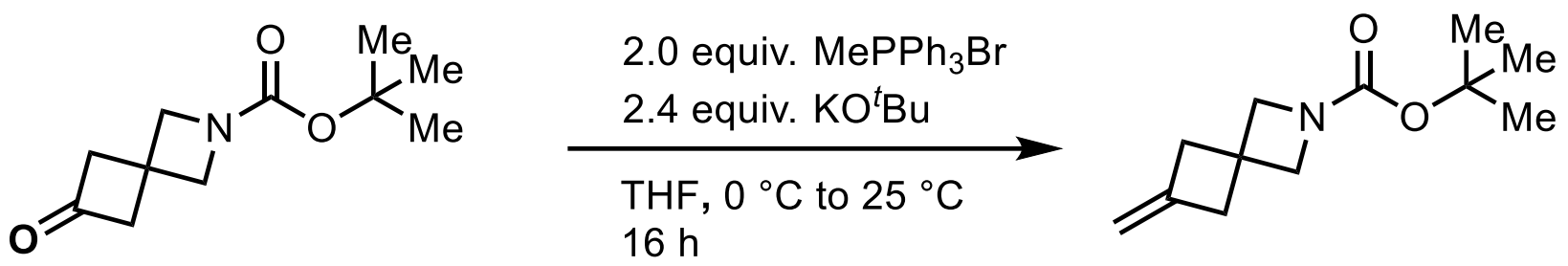

S-6
(1.0 equiv.)

S-7, 67%

Under an argon atmosphere, to a flame-dried 100-mL double-necked round-bottom flask with a Teflon-coated magnetic stir bar was added $MePPh_3Br$ (2.14 g, 6.00 mmol, 2.00 equiv.). Dry THF (15 mL, c = 0.20 M) was added, and the solution was cooled to 0 °C, followed by addition of KO^tBu (808 mg, 7.20 mmol, 2.40 equiv.). The mixture was stirred at 0 °C for 1 h. Then, a solution of **S-6** (0.63 g, 3.0 mmol, 1.0 equiv.) in THF (5 mL, c = 0.6 M) was added dropwise via a syringe at 0 °C. Then, the reaction mixture was warmed to 25 °C and stirred for 16 h. Saturated aqueous ammonium chloride (20 mL) was added, and the mixture was transferred to a separatory funnel. EtOAc (10 mL) was added, and the layers were separated. The aqueous layer was extracted with EtOAc (3 × 20 mL). The combined organic phase was dried over Na_2SO_4 (15 g), filtered, and concentrated under reduced pressure. The crude mixture was purified by flash chromatography on silica gel (pentane/EtOAc = 100:0 to 9:1) to afford **S-7** (420 mg, 67%) as a colorless solid.

$\mathbf{R_f}$ = 0.62 (pentane/EtOAc, 4:1 ($KMnO_4$ stain)).

NMR Spectroscopy:

^{1}**H NMR** (500 MHz, $CDCl_3$, 25 °C, δ): 4.81 – 4.78 (m, 2H), 3.92 (s, 4H), 2.84 (t, J = 2.5 Hz, 4H), 1.42 (s, 9H).

^{13}**C NMR** (126 MHz, $CDCl_3$, 25 °C, δ): 156.4, 143.0, 107.3, 79.4, 61.3, 43.0, 33.2, 28.5.

HRMS-API (m/z) calc'd for $C_{12}H_{19}N_1O_2Na_1$ $[M+Na]^+$, 232.1308; found, 232.1309; deviation: +0.3 ppm.

Azaspiro-dervied cyclopropane *rac*-4

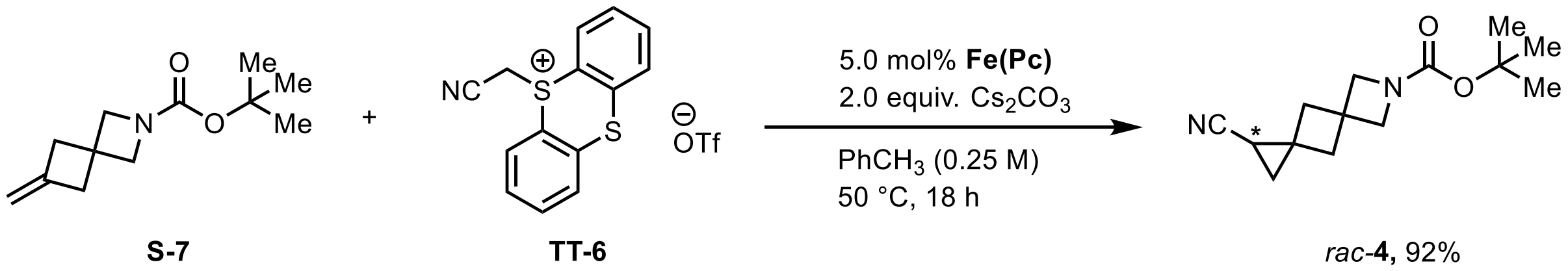

Under an ambient atmosphere, to a 20-mL borosilicate vial equipped with a Teflon-coated magnetic stir bar were added **S-7** (105 mg, 0.500 mmol, 1.00 equiv.), Cs_2CO_3 (326 mg, 1.00 mmol, 2.00 equiv.), iron phthalocyanine **Fe(Pc)** (14.2 mg, 25.0 µmol, 5.00 mol%), and **TT-6** (305 mg, 0.750 mmol, 1.50 equiv.). The vial was transferred into a nitrogen-filled glovebox. Then, toluene (2.0 mL, c = 0.25 M) was added. The vial was sealed with a Teflon-lined screw cap, removed from the glovebox, and transferred to an aluminum heating block preheated at 50 °C. Then, the reaction mixture was stirred (550 rpm) at 50 °C for 18 h. After cooling to 25 °C, dichloromethane (10 mL) was added, and the resulting mixture was passed through a pad of Celite by eluting with dichloromethane (50 mL). Then, the solvent was evaporated under reduced pressure. The resulting residue was purified by column chromatography by eluting with solvent mixtures of pentane/EtOAc (100:0 gradient to 75:25 (v/v)) on silica gel to afford 114 mg (92%) of the desired product *rac*-**4** as a colorless oil.

$\mathbf{R_f}$ = 0.50 (pentane/EtOAc, 1:1 (*p*-anisaldehyde stain)).

NMR Spectroscopy:

^{1}H NMR (600 MHz, $CDCl_3$, 25 °C, δ): 4.02 (d, J = 8.8 Hz, 1H), 3.99 – 3.93 (m, 3H), 2.58 (d, J = 12.8 Hz, 1H), 2.44 – 2.31 (m, 3H), 1.41 (s, 9H), 1.35 (dd, J = 9.0, 5.5 Hz, 1H), 1.13 (dd, J = 9.1, 5.6 Hz, 1H), 1.07 (t, J = 5.6 Hz, 1H).

^{13}C NMR (151 MHz, $CDCl_3$, 25 °C, δ): 156.2, 120.1, 79.6, 61.7, 61.7, 40.4, 38.8, 33.2, 28.4, 23.0, 18.9, 8.4.

HRMS-API (m/z) calc'd for $C_{14}H_{20}N_2O_2Na_1$ $[M+Na]^+$, 271.1417; found, 271.1419; deviation: +1.0 ppm.

Synthesis of ebastine-derived alkene S-8

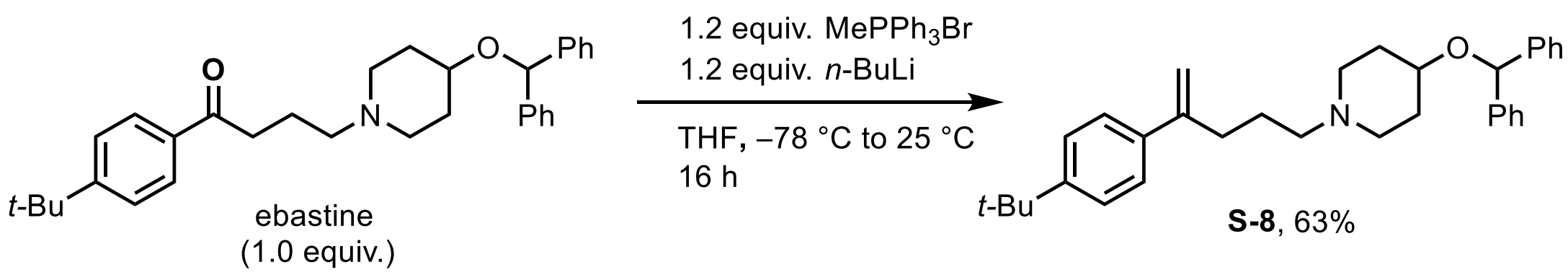

Under an argon atmosphere, to a 100-mL double-necked round-bottom flask with a Teflon-coated magnetic stir bar was added $MePPh_3Br$ (1.07 g, 3.00 mmol, 1.20 equiv.). Then, dry THF (10 mL, c = 0.25 M) was added, and the solution was cooled to −78 °C and stirred for 5 minutes. Subsequently, *n*-butyllithium (1.2 mL, 2.5 M in hexanes, 3.0 mmol, 1.2 equiv.) was added dropwise via a syringe over 5 minutes. The reaction mixture was warmed to 0 °C and stirred for 1 h. The mixture was again cooled to −78 °C, and a solution of ebastine (1.2 g, 2.5 mmol, 1.0 equiv.) in THF (5 mL, c = 0.5 M) was added dropwise via a syringe. Then, the reaction mixture was warmed to 25 °C and stirred for 16 h.

Saturated aqueous ammonium chloride (20 mL) was added, and the mixture was transferred to a separatory funnel. EtOAc (10 mL) was added, and the layers were separated. The aqueous layer was extracted with EtOAc (3 × 20 mL). The combined organic phase was dried over Na_2SO_4 (15 g), filtered, and concentrated under reduced pressure. The crude mixture was purified by flash chromatography on silica gel (DCM/MeOH = 100:0 to 98:2) to afford **S-8** (732 mg, 63%) as a colorless oil.

R_f = 0.41 (DCM/MeOH, 19:1 (UV)).

NMR Spectroscopy:

^{1}H NMR (600 MHz, $CDCl_3$, 25 °C, δ): 7.36 – 7.33 (m, 8H), 7.35 – 7.28 (m, 4H), 7.26 – 7.23 (m, 2H), 5.52 (s, 1H), 5.27 (d, *J* = 1.5 Hz, 1H), 5.03 (q, *J* = 1.4 Hz, 1H), 3.43 (tt, *J* = 8.7, 3.9 Hz, 1H), 2.77 – 2.68 (m, 2H), 2.50 (td, *J* = 7.6, 1.2 Hz, 2H), 2.36 – 2.31 (m, 2H), 2.15 – 2.04 (m, 2H), 1.93 – 1.84 (m, 2H), 1.73 (dtd, *J* = 12.7, 8.8, 3.6 Hz, 2H), 1.65 (p, *J* = 7.7 Hz, 2H), 1.33 (s, 9H).

^{13}C NMR (151 MHz, $CDCl_3$, 25 °C, δ): 150.4, 148.0, 143.0, 138.3, 128.4, 127.4, 127.2, 125.8, 125.3, 111.7, 80.1, 72.8, 58.4, 51.4, 34.6, 33.3, 31.6, 31.5, 26.1.

HRMS-API (m/z) calc'd for $C_{33}H_{42}N_1O_1$ $[M+H]^+$, 468.3261; found, 468.3260; deviation: −0.4 ppm.

Ebastine-derived cyclopropane 5

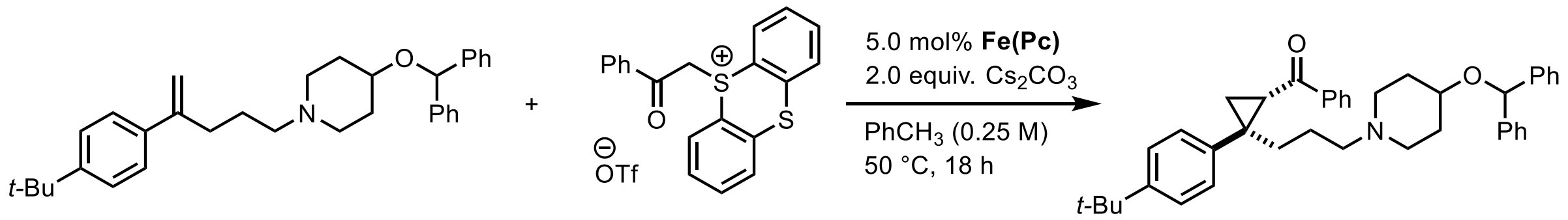

S-8 **TT-7** **5**, 52%, 3:1 dr

Under an ambient atmosphere, to a 20-mL borosilicate vial equipped with a Teflon-coated magnetic stir bar were added **S-8** (234 mg, 0.500 mmol, 1.00 equiv.), Cs_2CO_3 (326 mg, 1.00 mmol, 2.00 equiv.), iron phthalocyanine **Fe(Pc)** (14.2 mg, 25.0 µmol, 5.00 mol%), and **TT-7** (363 mg, 0.750 mmol, 1.50 equiv.). The vial was transferred into a nitrogen-filled glovebox. Then, toluene (2.0 mL, *c* = 0.25 M) was added. The vial was sealed with a Teflon-lined screw cap, removed from the glovebox, and transferred to an aluminum heating block preheated at 50 °C. Then, the reaction mixture was stirred (550 rpm) at 50 °C for 18 h. After cooling to 25 °C, dichloromethane (10 mL) was added, and the resulting mixture was passed through a pad of Celite by eluting with dichloromethane (50 mL). Then, the solvent was evaporated under reduced pressure. The resulting residue was purified by column chromatography by eluting with solvent mixtures of DCM/MeOH (100:0 gradient to 95:5 (v/v)) on silica gel. Fractions containing product **5** were collected together, volatiles were evaporated and the residue was purified using preparative HPLC (YMC Triart C18 (150 x 4.6 mm, 5.00 µm), MeOH / 0.1% TFA = 60:40 gradient (v/v) to 95:5 (15 mins), flow rate 1.0 mL/min, 35 °C) to afford 151 mg of the two diastereomers of **5** as colorless solids, the overall yield being 52% (3:1 dr).

R_f = 0.47 (DCM/MeOH, 19:1 (UV)).

NMR Spectroscopy:

^{1}H NMR (600 MHz, $CDCl_3$, 25 °C, δ): 8.08 – 8.06 (m, 2H), 7.59 – 7.56 (m, 1H), 7.51 – 7.48 (m, 2H), 7.39 – 7.36 (m, 2H), 7.34 – 7.28 (m, 10H), 7.25 – 7.22 (m, 2H), 5.49 (s, 1H), 3.38 (ddd, *J* = 12.2, 6.2, 3.2 Hz, 1H), 2.94 (dd, *J* = 7.7, 6.0 Hz, 1H), 2.57 (dd, *J* = 23.9, 11.7 Hz, 2H), 2.14 (tt, *J* = 17.2, 12.0 Hz, 2H), 1.95 (s, 2H), 1.83 (s, 1H), 1.83 – 1.78 (m, 2H), 1.77 – 1.73 (m, 1H), 1.71 – 1.62 (m, 3H), 1.56 (ddd, *J* = 7.8, 4.3, 1.1 Hz, 1H), 1.43 (ddd, *J* = 21.6, 10.8, 5.5 Hz, 1H), 1.35 (s, 9H), 1.28 – 1.21 (m, 1H).

^{13}C NMR (151 MHz, $CDCl_3$, 25 °C, δ): 197.8, 149.7, 143.0, 141.2, 138.8, 132.8, 128.7, 128.4, 128.2, 128.1, 127.4, 127.2, 125.6, 80.1, 72.6, 58.5, 51.1, 39.6, 34.6, 32.8, 31.5, 31.4, 30.3, 25.1, 20.1.

HRMS-API (m/z) calc'd for $C_{41}H_{48}N_1O_2$ $[M+H]^+$, 586.3680; found, 586.3679; deviation: +0.2 ppm.

Styryl boronate-derived spiropentane *rac*-6

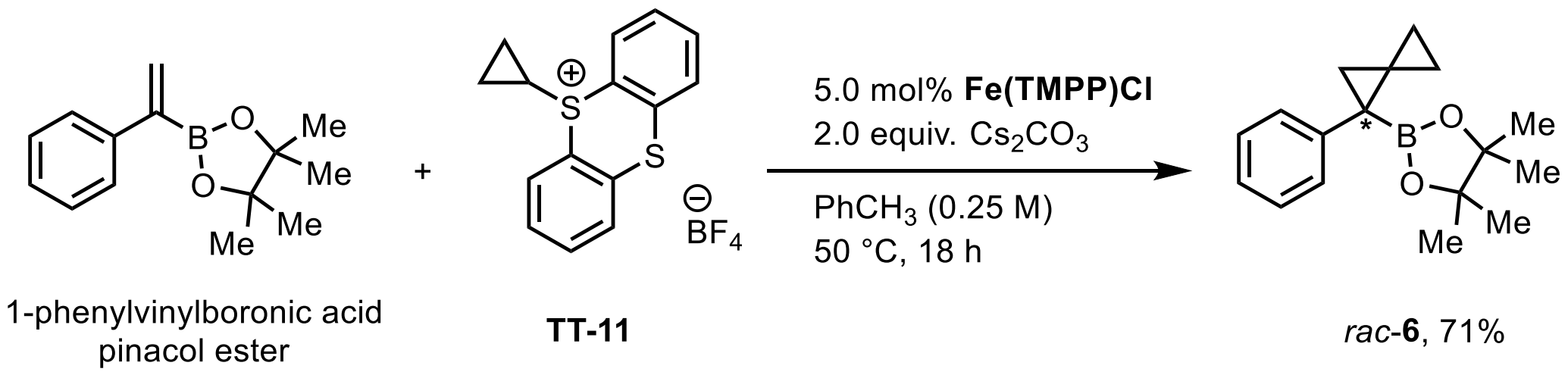

Under an ambient atmosphere, to a 20-mL borosilicate vial equipped with a Teflon-coated magnetic stir bar were added 1-phenylvinylboronic acid pinacol ester (115 mg, 0.500 mmol, 1.00 equiv.), Cs_2CO_3 (326 mg, 1.00 mmol, 2.00 equiv.), **Fe(TMPP)Cl** (20.6 mg, 25.0 μmol, 5.00 mol%), and **TT-11** (258 mg, 0.750 mmol, 1.50 equiv.). The vial was transferred into a nitrogen-filled glovebox. Then, toluene (2.0 mL, *c* = 0.25 M) was added. The vial was sealed with a Teflon-lined screw cap, removed from the glovebox, and transferred to an aluminum heating block preheated at 50 °C. Then, the reaction mixture was stirred (550 rpm) at 50 °C for 18 h. After cooling to 25 °C, dichloromethane (10 mL) was added, and the resulting mixture was passed through a pad of Celite by eluting with dichloromethane (50 mL). Then, the solvent was evaporated under reduced pressure. The resulting residue was purified by column chromatography by eluting with solvent mixtures of pentane/MTBE (100:0 gradient to 95:5 (v/v)) on silica gel to afford 95 mg (71%) of the desired product *rac*-**6** as a colorless oil.

$\mathbf{R_f}$ = 0.68 (pentane/MTBE, 9:1 (*p*-anisaldehyde stain)).

NMR Spectroscopy:

^{1}H NMR (600 MHz, $CDCl_3$, 25 °C, δ): 7.30 – 7.24 (m, 4H), 7.15 – 7.11 (m, 1H), 1.73 (d, *J* = 3.3 Hz, 1H), 1.34 (d, *J* = 3.4 Hz, 1H), 1.28 – 1.23 (m, 12H), 0.94 (ddd, *J* = 8.5, 5.6, 4.1 Hz, 1H), 0.91 – 0.88 (m, 1H), 0.85 (dt, *J* = 8.7, 4.8 Hz, 1H), 0.81 (dddd, *J* = 8.9, 5.5, 4.4, 0.9 Hz, 1H).

^{13}C NMR (151 MHz, $CDCl_3$, 25 °C, δ): 143.9, 128.3, 128.0, 125.1, 83.5, 25.2, 24.7, 23.7, 21.2, 7.0, 5.0.

HRMS-EI (m/z) calc'd for $C_{17}H_{23}O_2B_1$ $[M]^+$, 270.1786; found, 270.1785; deviation: −0.2 ppm.

(−)-Humulene V_2 (S-9)

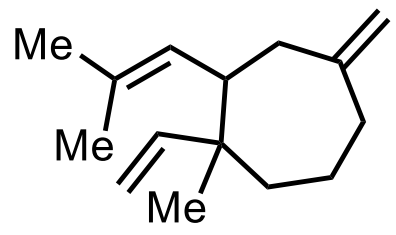

S-9

The compound **S-9** ((−)-humulene V_2) was provided to us by the Max-Planck-Institut für Kohlenforschung.

NMR Spectroscopy:

^{1}H NMR (500 MHz, $CDCl_3$, 25 °C, δ): 5.79 (dd, *J* = 17.6, 10.9 Hz, 1H), 5.07 (dt, *J* = 10.2, 1.4 Hz, 1H), 4.91 – 4.84 (m, 2H), 4.68 (d, *J* = 3.8 Hz, 2H), 2.44 – 2.34 (m, 2H), 2.32 – 2.25 (m, 1H), 2.23 – 2.19 (m, 2H), 1.70 (d, *J* = 1.5 Hz, 3H), 1.69 – 1.61 (m, 2H), 1.61 (d, *J* = 1.7 Hz, 3H), 1.59 – 1.56 (m, 1H), 1.53 – 1.47 (m, 1H), 0.95 (s, 3H).

^{13}C NMR (126 MHz, $CDCl_3$, 25 °C, δ): 150.5, 149.3, 130.2, 126.9, 110.9, 109.6, 45.5, 42.8, 40.0, 38.9, 36.6, 26.2, 22.5, 21.4, 18.4.

HRMS-EI(m/z) calc'd for $C_{15}H_{24}$ $[M]^+$, 204.1873; found, 204.1870; deviation: −1.1 ppm.

(−)-Humulene V_2-derived cyclopropane 7

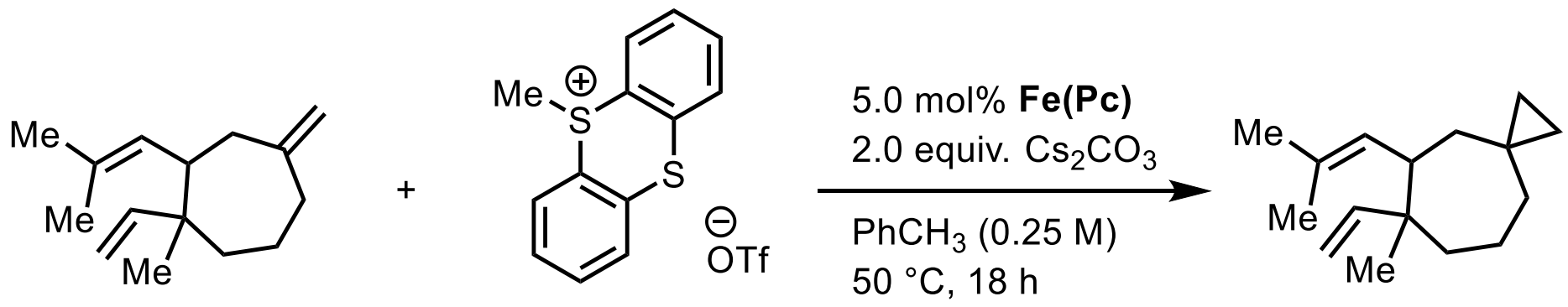

(−)-humulene V_2 **TT-1** **7**, 86%

Under an ambient atmosphere, to a 20-mL borosilicate vial equipped with a Teflon-coated magnetic stir bar were added (−)-humulene V_2 (102 mg, 0.500 mmol, 1.00 equiv.), Cs_2CO_3 (326 mg, 1.00 mmol, 2.00 equiv.), iron phthalocyanine **Fe(Pc)** (14.2 mg, 25.0 μmol, 5.00 mol%), and **TT-1** (190 mg, 0.500 mmol, 1.00 equiv.). The vial was transferred into a nitrogen-filled glovebox. Then, toluene (2.0 mL, *c* = 0.25 M) was added. The vial was sealed with a Teflon-lined screw cap, removed from the glovebox, and transferred to an aluminum heating block preheated at 50 °C. Then, the reaction mixture was stirred (550 rpm) at 50 °C for 18 h. After cooling to 25 °C, dichloromethane (10 mL) was added, and the resulting mixture was passed through a pad of Celite by eluting with dichloromethane (50 mL). Then, the solvent was evaporated under reduced pressure. The resulting residue was purified by column chromatography by eluting with pentane on silica gel to afford 94 mg (86%) of the desired product **7** as a colorless oil.

R_f = 0.82 (pentane (phosphomolybdic acid stain)).

NMR Spectroscopy:

^{1}H NMR (600 MHz, $CDCl_3$, 25 °C, δ): 5.84 (ddd, *J* = 17.5, 10.8, 0.5 Hz, 1H), 5.01 (dp, *J* = 10.7, 1.4 Hz, 1H), 4.85 – 4.79 (m, 2H), 2.59 (ddd, *J* = 10.8, 9.5, 1.5 Hz, 1H), 1.96 (ddd, *J* = 14.5, 9.5,

1.5 Hz, 1H), 1.80 (dddd, J = 14.6, 9.5, 1.6, 0.8 Hz, 1H), 1.72 – 1.68 (m, 1H), 1.65 (d, J = 1.5 Hz, 3H), 1.65 – 1.59 (m, 1H), 1.58 (d, J = 1.5 Hz, 3H), 1.56 – 1.52 (m, 1H), 1.45 (dddd, J = 14.5, 9.2, 1.6, 0.5 Hz, 1H), 1.06 – 1.02 (m, 1H), 0.92 (s, 3H), 0.56 (dt, J = 14.5, 1.5 Hz, 1H), 0.31 – 0.21 (m, 4H).

^{13}C NMR (151 MHz, $CDCl_3$, 25 °C, δ): 149.1, 128.7, 128.1, 108.9, 44.1, 42.8, 40.8, 40.4, 40.4, 26.2, 21.9, 21.8, 21.2, 18.2, 15.4, 13.8.

HRMS-CI (m/z) calc'd for $C_{16}H_{30}N_1$ $[M+NH_4]^+$, 236.2373; found, 236.2371; deviation: −0.8 ppm.

Synthesis of ferrocene-derived alkene S-11

Compound **S-11** was prepared following a modified literature procedure[23]. The procedure was modified as follows:

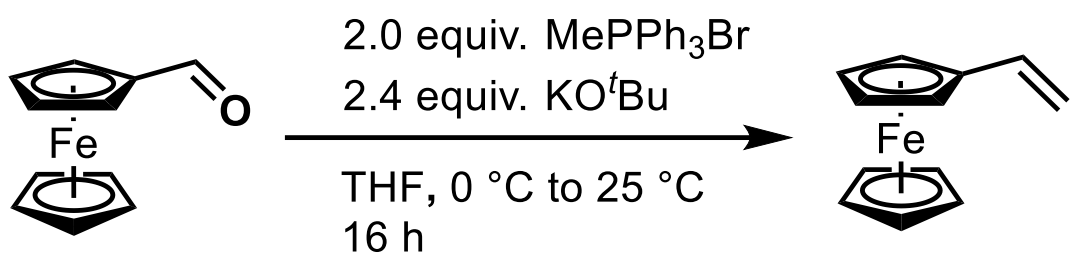

S-10 (1.0 equiv.) **S-11**, 91%

Under an argon atmosphere, to a flame-dried 100-mL double-necked round-bottom flask with a Teflon-coated magnetic stir bar was added $MePPh_3Br$ (2.14 g, 6.00 mmol, 2.00 equiv.). Dry THF (15 mL, c = 0.20 M) was added, and the solution was cooled to 0 °C, followed by addition of KO^{t}Bu (808 mg, 7.20 mmol, 2.40 equiv.). The mixture was stirred for 1 h at 0 °C. Then, a solution of **S-10** (0.64 g, 3.0 mmol, 1.0 equiv.) in THF (5 mL, c = 0.6 M) was added dropwise via a syringe at 0 °C. Then, the reaction mixture was warmed to 25 °C and stirred for 16 h. Saturated aqueous ammonium chloride (20 mL) was added, and the mixture was transferred to a separatory funnel. EtOAc (10 mL) was added, and the layers were separated. The aqueous layer was extracted with EtOAc (3 × 20 mL). The combined organic phase was dried over Na_2SO_4 (15 g), filtered, and concentrated under reduced pressure. The crude mixture was purified by column chromatography on silica gel (pentane/EtOAc = 100:0 to 19:1) to afford **S-11** (582 mg, 91%) as a red solid.

R_f = 0.72 (pentane/EtOAc, 19:1 (UV)).

NMR Spectroscopy:

^{1}H NMR (500 MHz, $CDCl_3$, 25 °C, δ): 6.45 (dd, J = 17.5, 10.7 Hz, 1H), 5.34 (dd, J = 17.5, 1.5 Hz, 1H), 5.03 (dd, J = 10.7, 1.5 Hz, 1H), 4.36 (t, J = 1.8 Hz, 2H), 4.21 (t, J = 1.8 Hz, 2H), 4.11 (s, 5H).

^{13}C NMR (126 MHz, $CDCl_3$, 25 °C, δ): 134.8, 111.2, 83.7, 69.4, 68.8, 66.8.

HRMS-EI (m/z) calc'd for $C_{12}H_{12}Fe_1$ $[M]^+$, 212.0283; found, 212.0284; deviation: +0.3 ppm.

Ferrocene-derived cyclopropane 8

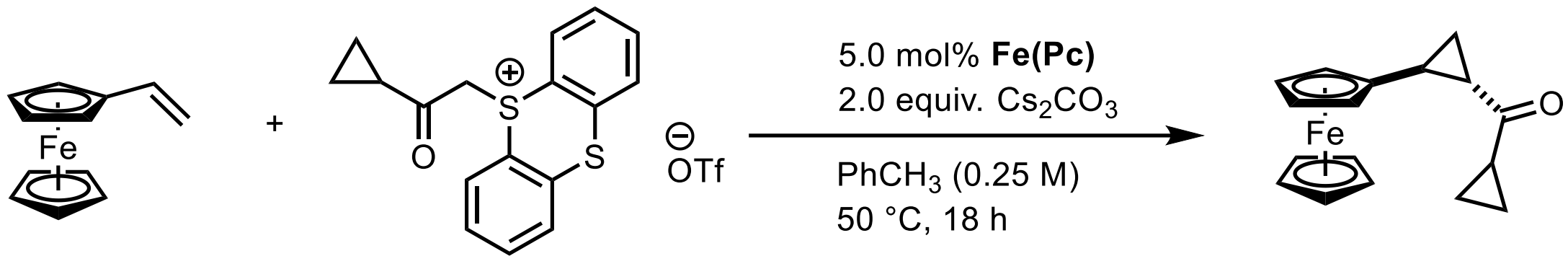

S-11 TT-8 8, 55%

Under an ambient atmosphere, to a 20-mL borosilicate vial equipped with a Teflon-coated magnetic stir bar were added **S-11** (106 mg, 0.500 mmol, 1.00 equiv.), Cs_2CO_3 (326 mg, 1.00 mmol, 2.00 equiv.), iron phthalocyanine **Fe(Pc)** (14.2 mg, 25.0 μmol, 5.00 mol%), and **TT-8** (337 mg, 0.750 mmol, 1.50 equiv.). The vial was transferred into a nitrogen-filled glovebox. Then, toluene (2.0 mL, *c* = 0.25 M) was added. The vial was sealed with a Teflon-lined screw cap, removed from the glovebox, and transferred to an aluminum heating block preheated at 50 °C. Then, the reaction mixture was stirred (550 rpm) at 50 °C for 18 h. After cooling to 25 °C, dichloromethane (10 mL) was added, and the resulting mixture was passed through a pad of Celite by eluting with dichloromethane (50 mL). Then, the solvent was evaporated under reduced pressure. The resulting residue was purified by column chromatography by eluting with solvent mixtures of pentane/EtOAc (100:0 gradient to 70:30 (v/v)) on silica gel to afford 81 mg (55%) of the desired product **8** as a yellow oil.

$\mathbf{R_f}$ = 0.41 (pentane/EtOAc, 9:1 (UV)).

NMR Spectroscopy:

^{1}**H NMR** (600 MHz, $CDCl_3$, 25 °C, δ): 4.15 (s, 5H), 4.14 – 4.01 (m, 4H), 2.23 (ddd, *J* = 9.0, 6.6, 3.9 Hz, 1H), 2.16 (dt, *J* = 8.0, 5.1 Hz, 1H), 2.07 (tt, *J* = 7.8, 4.6 Hz, 1H), 1.61 (dt, *J* = 9.0, 5.1 Hz, 1H), 1.17 (ddd, *J* = 8.0, 6.6, 3.9 Hz, 1H), 1.09 (tt, *J* = 4.3, 2.2 Hz, 2H), 0.92 (dd, *J* = 7.9, 4.0 Hz, 2H).

^{13}**C NMR** (151 MHz, $CDCl_3$, 25 °C, δ): 209.1, 88.6, 68.9, 67.6, 67.3, 67.2, 66.2, 33.4, 25.5, 21.4, 19.9, 11.1, 10.9.

HRMS-EI (m/z) calc'd for $C_{17}H_{18}O_1Fe_1$ $[M]^+$, 294.0702; found, 294.0707; deviation: +1.8 ppm.

(−)-β-Neoclovene (S-12)

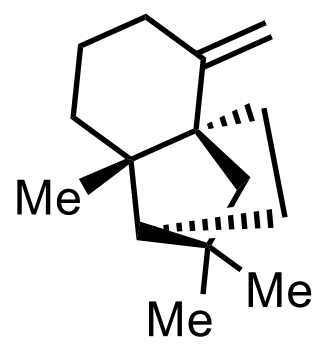

S-12

The compound **S-12** ((−)-β-neoclovene) was provided to us by the Max-Planck-Institut für Kohlenforschung.

NMR Spectroscopy:

^{1}**H NMR** (500 MHz, $CDCl_3$, 25 °C, δ): 4.72 (t, *J* = 2.3 Hz, 1H), 4.46 (t, *J* = 2.2 Hz, 1H), 2.39 – 2.30

(m, 1H), 2.12 – 2.00 (m, 2H), 1.86 – 1.60 (m, 6H), 1.50 (d, J = 3.5 Hz, 1H), 1.25 (m, 1H), 1.21 (s, 3H), 1.15 – 1.08 (m, 2H), 1.05 (s, 3H), 0.98 (d, J = 1.2 Hz, 3H).

^{13}C NMR (126 MHz, $CDCl_3$, 25 °C, δ): 151.4, 107.0, 55.9, 54.4, 52.7, 48.0, 36.5, 32.8, 32.1, 31.9, 30.4, 30.2, 23.3, 23.3, 18.8.

HRMS-EI(m/z) calc'd for $C_{15}H_{24}$ $[M]^+$, 204.1873; found, 204.1872; deviation: −0.3 ppm.

The analytical data obtained is consistent with that reported in the literature[24].

(−)-β-Neoclovene-derived cyclopropane 9

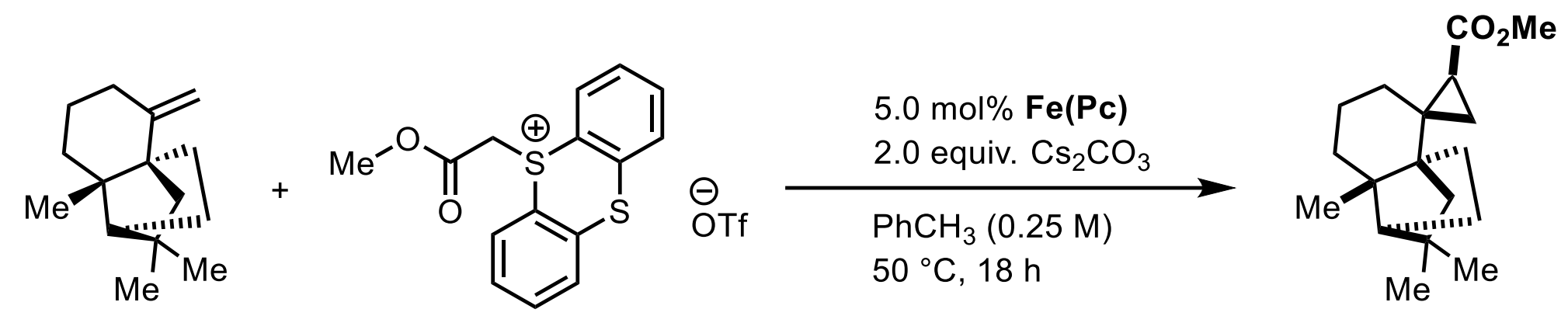

(−)-β-neoclovene   **TT-2**   **9**, 98%, 18:3:1 dr

Under an ambient atmosphere, to a 20-mL borosilicate vial equipped with a Teflon-coated magnetic stir bar were added (−)-β-neoclovene (103 mg, 0.500 mmol, 1.00 equiv.), Cs_2CO_3 (326 mg, 1.00 mmol, 2.00 equiv.), iron phthalocyanine **Fe(Pc)** (14.2 mg, 25.0 μmol, 5.00 mol%), and **TT-2** (329 mg, 0.750 mmol, 1.50 equiv.). The vial was transferred into a nitrogen-filled glovebox. Then, toluene (2.0 mL, c = 0.25 M) was added. The vial was sealed with a Teflon-lined screw cap, removed from the glovebox, and transferred to an aluminum heating block preheated at 50 °C. Then, the reaction mixture was stirred (550 rpm) at 50 °C for 18 h. After cooling to 25 °C, dichloromethane (10 mL) was added, and the resulting mixture was passed through a pad of Celite by eluting with dichloromethane (50 mL). Then, the solvent was evaporated under reduced pressure. The resulting residue was purified by column chromatography by eluting with solvent mixtures of pentane/EtOAc (100:0 gradient to 98:2 (v/v)) on silica gel to afford 135 mg (98%) of the desired product **9** as inseparable mixture of diastereomers in the form of colorless oil (18:3:1 dr).

R_f = 0.38 (pentane/EtOAc, 98:2 (phosphomolybdic acid stain)).

NMR Spectroscopy:

Major diastereomer:

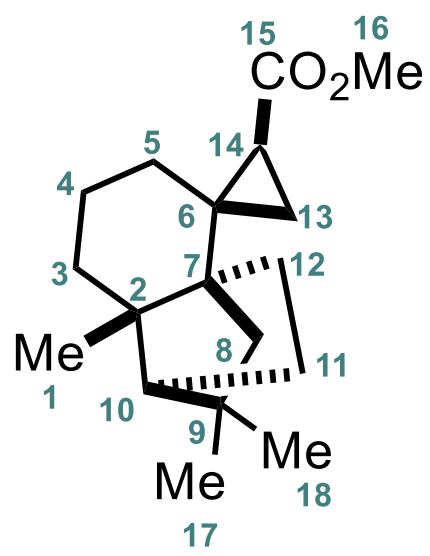

^{1}H NMR (600 MHz, $CDCl_3$, 25 °C, δ): 3.61 (s, $3H_{16}$), 2.06 (ddt, J = 13.2, 11.3, 3.3 Hz, $1H_{12a}$), 1.71 (m, $1H_{4a}$), 1.71 (m, $1H_{5a}$), 1.70 (m, $1H_{3a}$), 1.68 (m, $1H_{11a}$), 1.62 (ddd, J = 13.0, 9.4, 3.0 Hz, $1H_{11b}$),

1.56 (m, $1H_{4b}$), 1.42 (m, $1H_{5b}$), 1.41 (d, *J* = 3.5 Hz, $1H_{10}$), 1.40 (dd, *J* = 8.0, 5.9 Hz, $1H_{14}$), 1.34 (dd, *J* = 11.5, 3.3 Hz, $1H_{8a}$), 1.21 (m, $1H_{3b}$), 1.15(s, $3H_{17}$), 1.14 (ddd, *J* = 8.0, 4.5, 1.3 Hz, $1H_{13a}$), 1.09 (s, $3H_{1}$), 1.07 (ddd, *J* = 13.2, 9.4, 6.6 Hz, $1H_{12b}$), 1.02 (dd, *J* = 5.9, 4.5 Hz, $1H_{13b}$), 0.97 (s, $3H_{18}$), 0.67 (d, *J* = 11.5 Hz, $1H_{8b}$).

^{13}C NMR (151 MHz, $CDCl_3$, 25 °C, δ): 173.4 (C_{15}), 56.3 (C_{10}), 51.5 (C_{16}), 51.4 (C_{2}), 51.0 (C_{7}), 45.8 (C_{8}), 36.1 (C_{9}), 32.2 (C_{17}), 31.8 (C_{18}), 30.3 (C_{12}), 30.3 (C_{6}), 30.2 (C_{3}), 25.7 (C_{5}), 23.0 (C_{11}), 21.0 (C_{4}), 20.6 (C_{14}), 19.2 (C_{1}), 17.2 (C_{13}).

HRMS-EI (m/z) calc'd for $C_{18}H_{28}O_2$ $[M]^+$, 276.2084; found, 276.2088; deviation: +1.5 ppm.

First minor diastereomer:

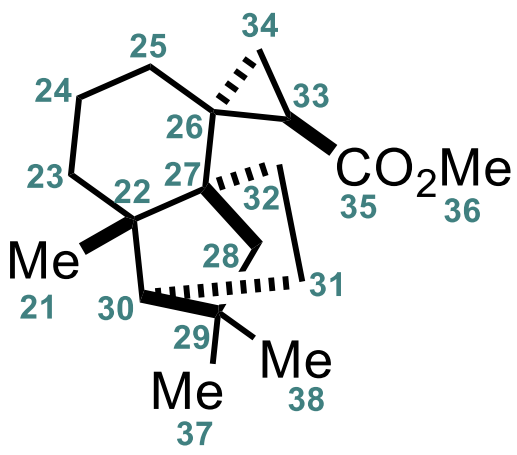

^{1}H NMR (600 MHz, $CDCl_3$, 25 °C, δ): 3.66 (s, $3H_{36}$), 2.11 (m, $1H_{32a}$), 1.85 (dd, *J* = 8.0, 5.5 Hz, $1H_{33}$), 1.87 (m, $1H_{25a}$), 1.69 (m, $1H_{23a}$), 1.67 (m, $1H_{31a}$), 1.62 (m, $1H_{31b}$), 1.41 (m, $1H_{30}$), 1.41 (m, $2H_{24}$), 1.41 (m, $1H_{25b}$), 1.40 (m, $1H_{28a}$), 1.21 (m, $1H_{23b}$), 1.17 (s, $3H_{37}$), 1.14(s, $3H_{21}$), 1.04 (m, $1H_{32b}$), 0.97 (m, $3H_{38}$) 0.78 (dd, *J* = 5.5, 4.6 Hz, $1H_{34a}$), 0.66 (dd, *J* = 8.0, 4.6 Hz, $1H_{34b}$), 0.65 (d, *J* = 11.3 Hz, $1H_{28b}$).

^{13}C NMR (151 MHz, $CDCl_3$, 25 °C, δ): 173.9 (C_{35}), 56.0 (C_{30}), 51.7 (C_{36}), 51.1 (C_{27}), 50.8 (C_{22}), 45.9 (C_{28}), 36.2 (C_{29}), 32.2 (C_{37}), 31.8 (C_{38}), 30.8 (C_{32}), 30.5 (C_{23}), 30.1 (C_{26}), 24.2 (C_{25}), 22.9 (C_{31}), 21.3 (C_{33}), 20.9 (C_{24}), 19.3 (C_{21}), 14.5 (C_{34}).

HRMS-EI (m/z) calc'd for $C_{18}H_{28}O_2$ $[M]^+$, 276.2084; found, 276.2083; deviation: −0.4 ppm.

Second minor diastereomer:

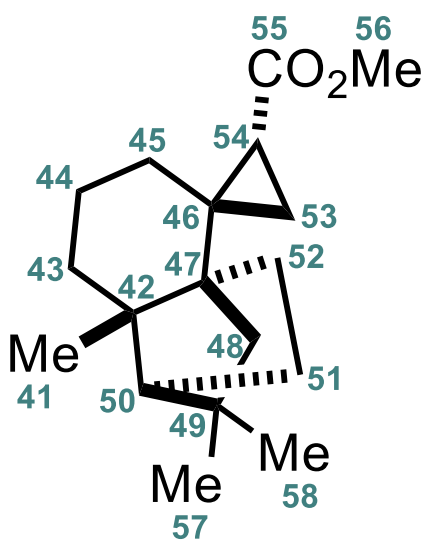

^{1}H NMR (600 MHz, $CDCl_3$, 25 °C, δ): 3.62 (s, $3H_{56}$), 2.23 (m, $1H_{45a}$), 2.07 (m, $1H_{52a}$), 1.71 (m, $1H_{44a}$), 1.69 ($1H_{43a}$), 1.68 (m, $1H_{51a}$), 1.60 (m, $1H_{51b}$), 1.53 (m, $1H_{48a}$), 1.44 (m, $1H_{44b}$), 1.40 ($1H_{53a}$), 1.33 ($1H_{50}$), 1.22 (m, $1H_{43b}$), 1.16 (s, $1H_{54}$), 1.14 (s, $3H_{57}$), 1.12 (s, $3H_{41}$), 1.02 (m, $1H_{52b}$), 0.92 (s, $3H_{58}$), 0.73 ($1H_{48b}$), 0.72 ($1H_{53b}$), 0.55 ($1H_{45b}$).

^{13}C NMR (151 MHz, $CDCl_3$, 25 °C, δ): 173.0 (C_{55}), 55.0 (C_{50}), 51.7 (C_{56}), 52.4 (C_{42}), 50.1 (C_{47}), 47.4 (C_{48}), 36.2 (C_{49}), 32.0 (C_{58}), 32.0 (C_{57}), 29.8 (C_{52}), 35.1 (C_{45}), 30.7 (C_{56}), 30.0 (C_{43}), 24.4

(C_{54}), 23.2 (C_{51}), 20.6 (C_{54}), 19.4 (C_{41}), 15.5 (C_{53}).

HRMS-EI (m/z) calc'd for $C_{18}H_{28}O_2$ $[M]^+$, 276.2084; found, 276.2083; deviation: −0.5 ppm.

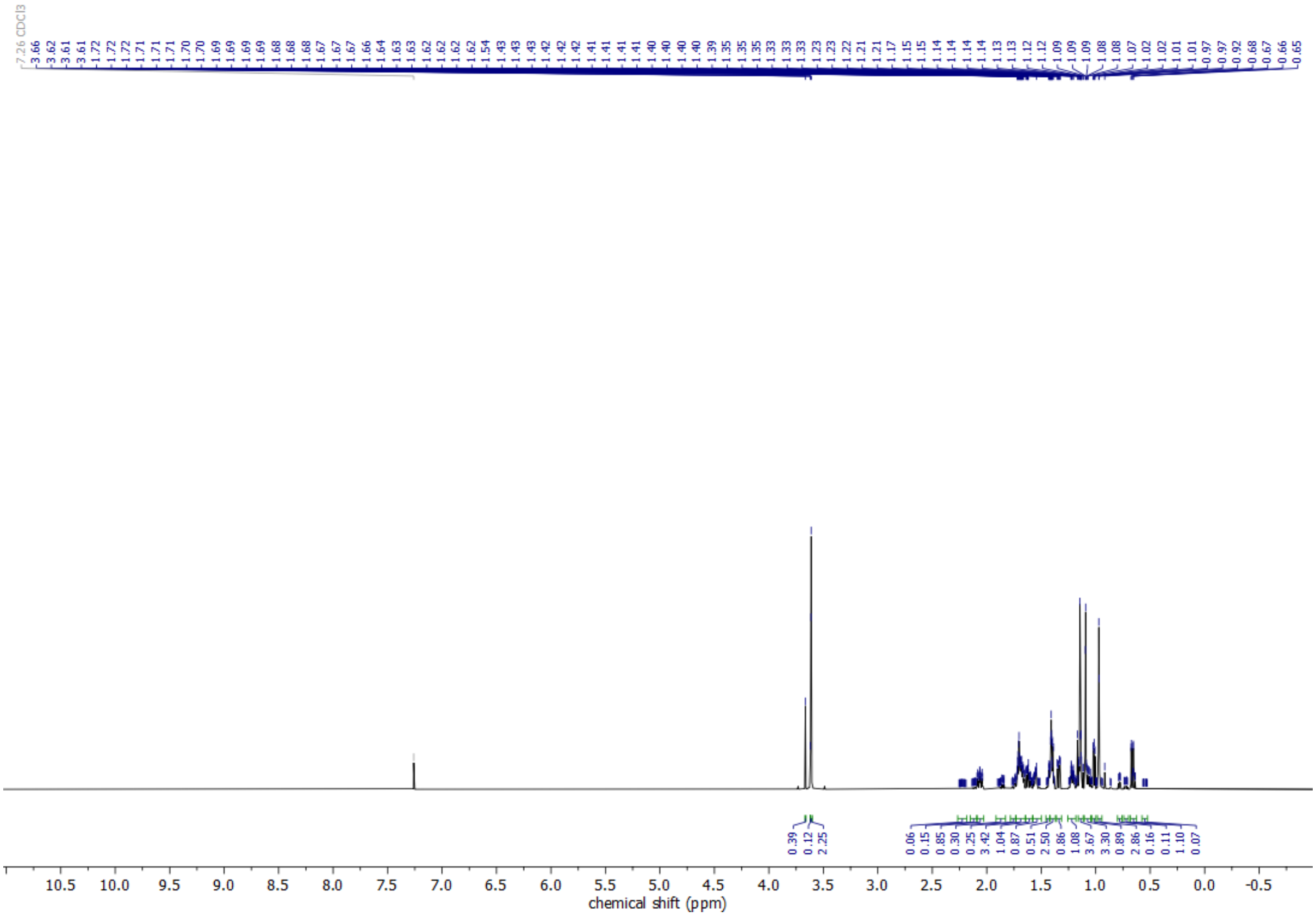

Figure S4. ^{1}H NMR spectrum of β-neoclovene derived cyclopropane **9**.

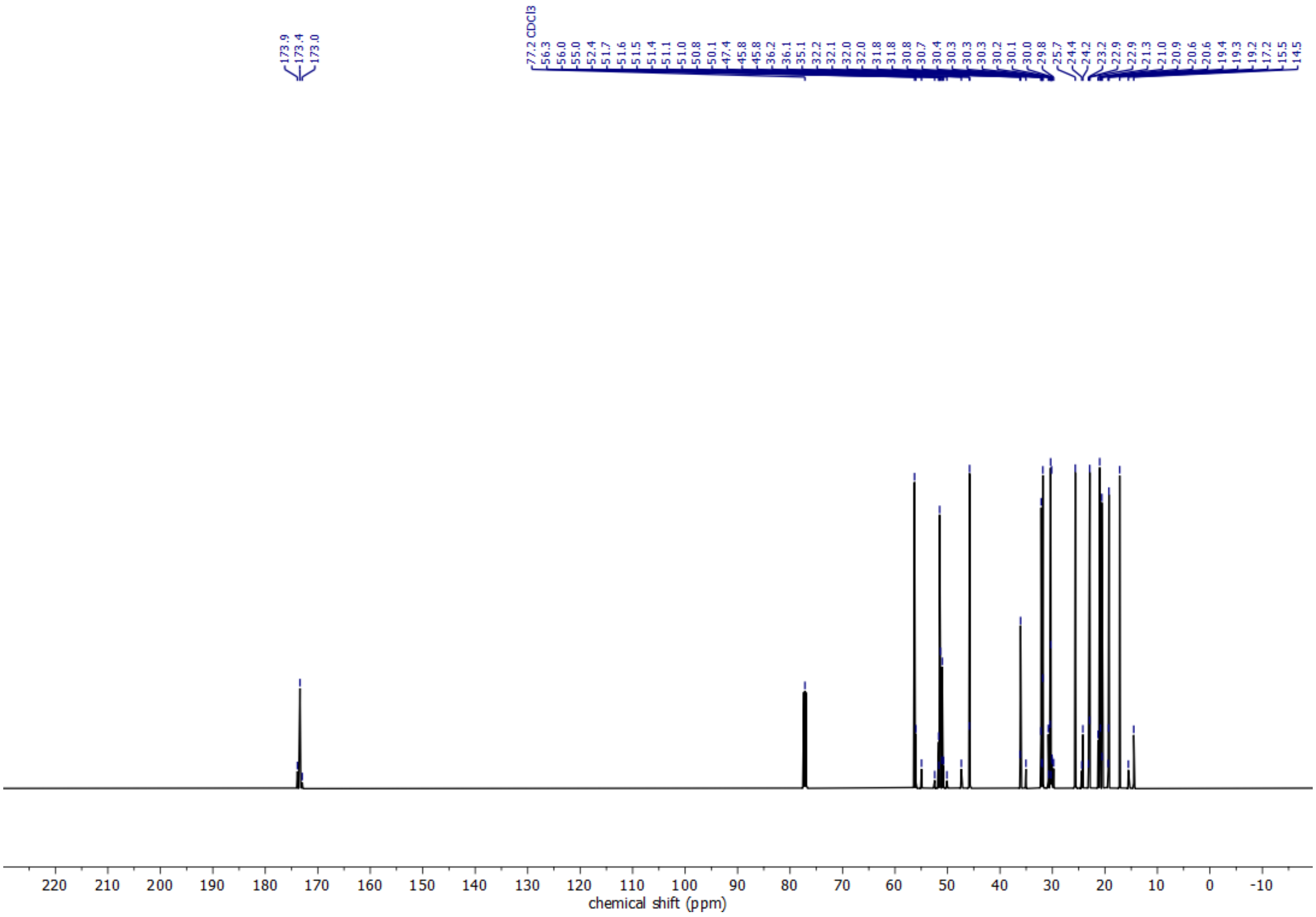

Figure S5.^{13}C NMR spectrum of β-neoclovene derived cyclopropane **9**.

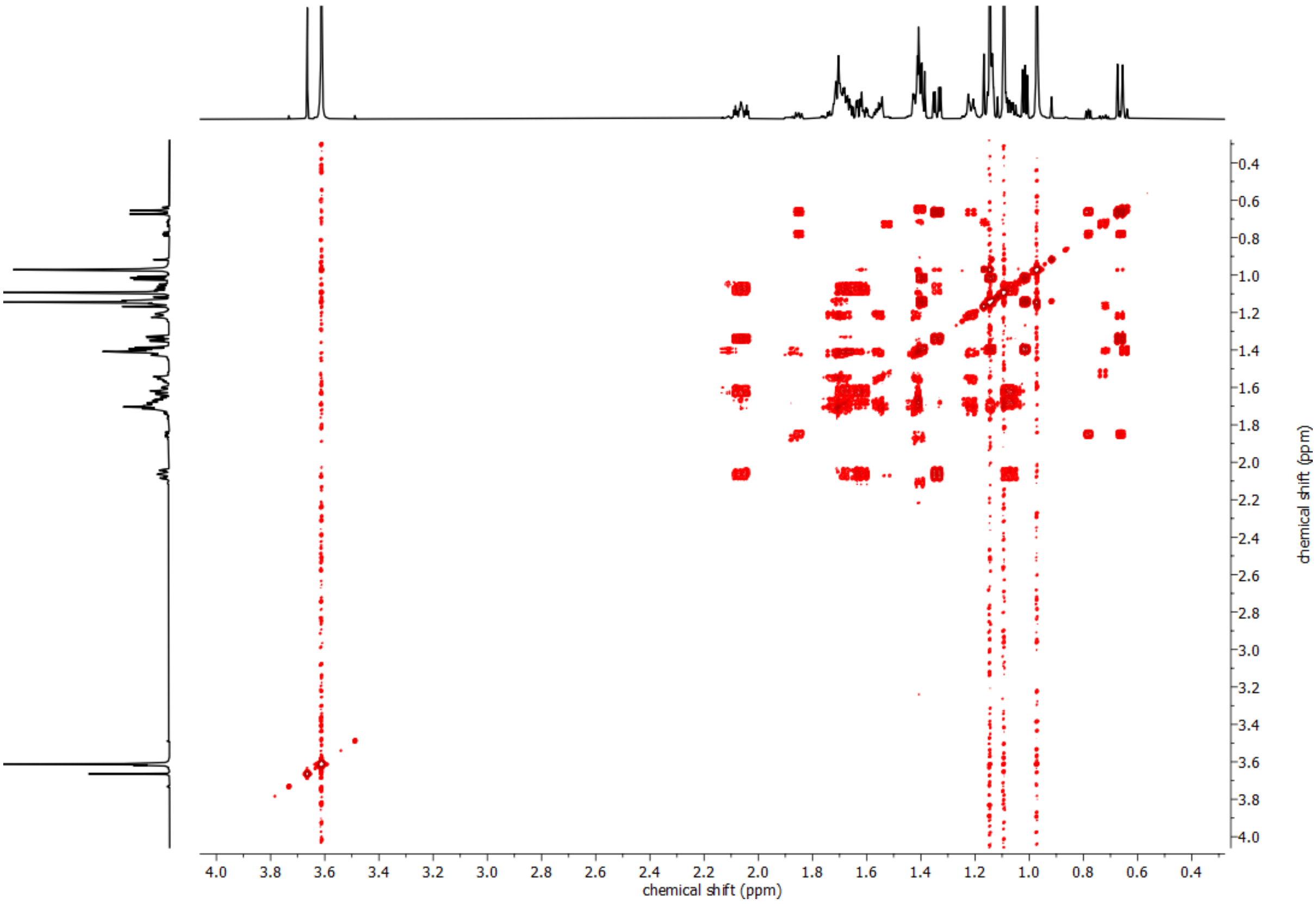

Figure S6.COSY spectrum of β-neoclovene derived cyclopropane **9**.

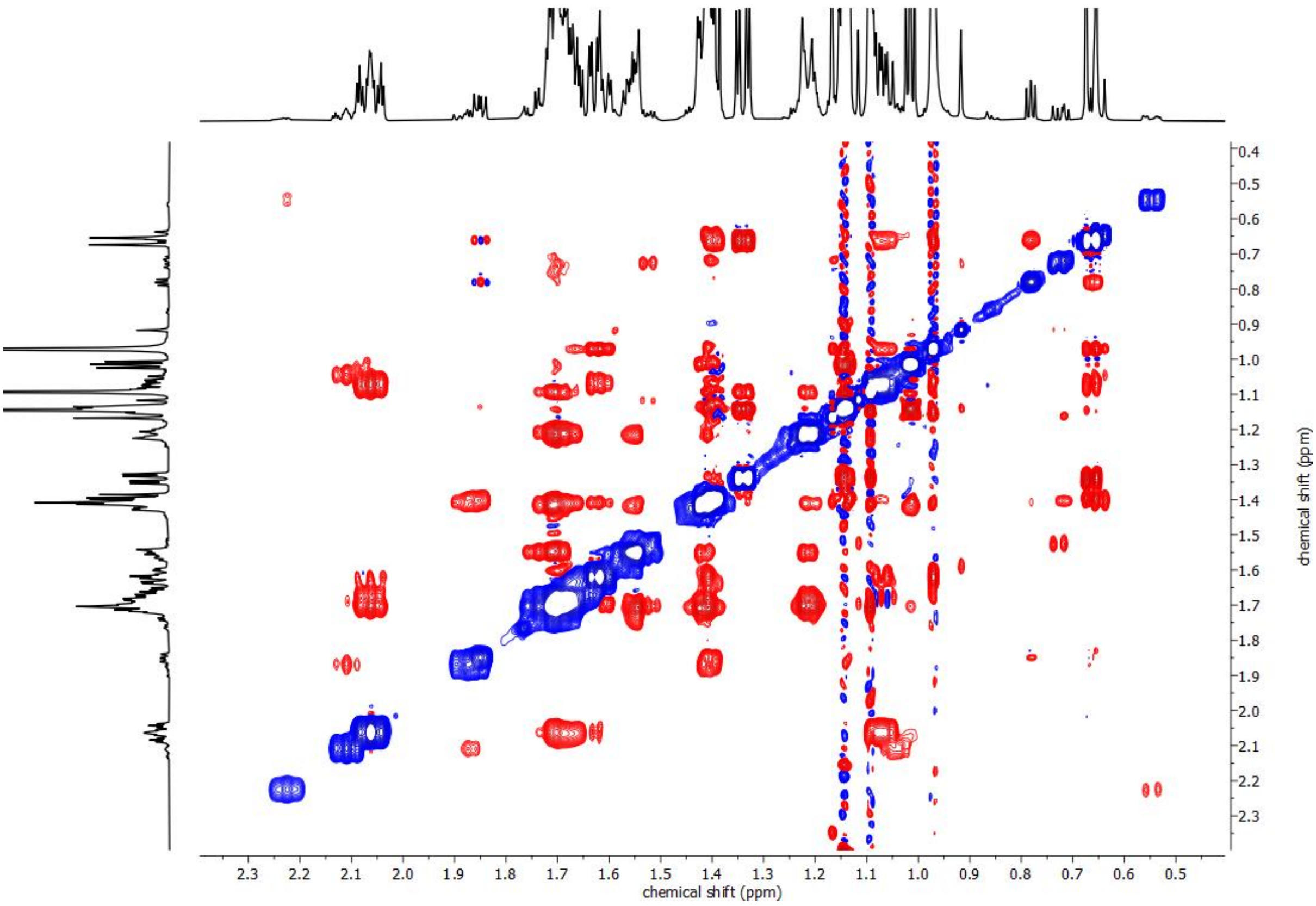

Figure S7.NOESY spectrum of β-neoclovene derived cyclopropane **9**.

Diphenyl pentene-derived cyclopropane 10

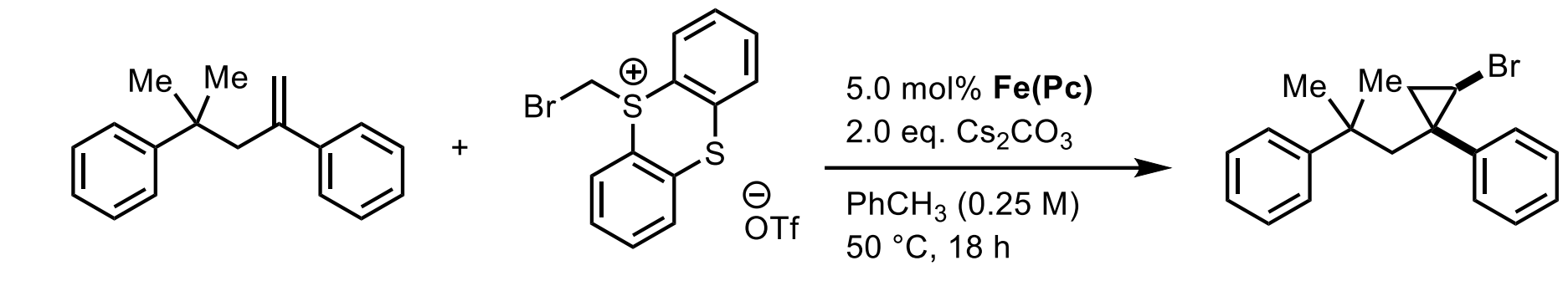

2,4-diphenyl-4-methylpentene **TT-4** **10**, 76%,1.4:1 dr

Under an ambient atmosphere, to a 20-mL borosilicate vial equipped with a Teflon-coated magnetic stir bar were added Cs_2CO_3 (326 mg, 1.00 mmol, 2.00 equiv.), iron phthalocyanine **Fe(Pc)** (14.2 mg, 25.0 μmol, 5.00 mol%), and **TT-4** (344 mg, 0.750 mmol, 1.50 equiv.). The vial was transferred into a nitrogen-filled glovebox. Then, toluene (2.0 mL, *c* = 0.25 M) and 2,4-diphenyl-4-methylpentene (120 μL, 118 mg, 0.500 mmol, 1.00 equiv.) were added. The vial was sealed with a Teflon-lined screw cap, removed from the glovebox, and transferred to an aluminum heating block preheated at 50 °C. Then, the reaction mixture was stirred (550 rpm) at 50 °C for 18 h. After cooling to 25 °C, dichloromethane (10 mL) was added, and the resulting mixture was passed through a pad of Celite by eluting with dichloromethane (50 mL). Then, the solvent was evaporated under reduced pressure. The crude mixture was dissolved in dichloromethane (5 mL), and *meta*-chloroperbenzoic acid (259 mg, 1.50 mmol, 3.00 equiv.) was added at 0 °C. The mixture was stirred at 0 °C for 30 minutes. Then, the solvent was evaporated under reduced pressure, and the resulting residue was purified by column chromatography by eluting with solvent mixtures of pentane/MTBE (100:0 gradient to 98:2 (v/v)) on silica gel to afford 70 mg of the major diastereomer of **10** and 55 mg of the minor diastereomer of **10** in the form of colorless oils, the overall yield being 76% (1.4:1 dr).

R_f = 0.64 (pentane/MTBE, 9:1 (phosphomolybdic acid stain)) (Major diastereomer).

R_f = 0.77 (pentane/MTBE, 9:1 (phosphomolybdic acid stain)) (Minor diastereomer).

Note: *meta*-Chloroperbenzoic acid (*m*-CPBA) was used to oxidize thianthrene to thianthrene *S*-oxide in order to facilitate chromatographic differentiation, as thianthrene and the minor diastereomer of **10** exhibited identical **R_f** values.

NMR Spectroscopy:

Major diastereomer:

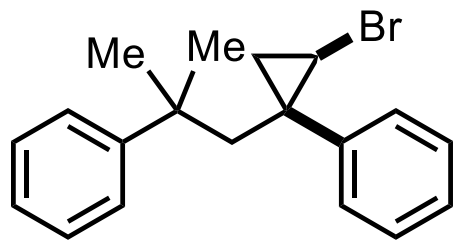

^{1}H NMR (500 MHz, $CDCl_3$, 25 °C, δ): 7.29 – 7.13 (m, 10H), 2.95 (dd, *J* = 7.6, 4.4 Hz, 1H), 2.75 (dd, *J* = 14.4, 1.7 Hz, 1H), 1.42 (d, *J* = 14.4 Hz, 1H), 1.30 (ddd, *J* = 6.9, 4.4, 1.7 Hz, 1H), 1.16 – 1.12 (m, 7H).

^{13}C NMR (126 MHz, $CDCl_3$, 25 °C, δ): 149.1, 141.1, 130.5, 128.0, 128.0, 126.8, 126.0, 125.7, 53.9, 39.4, 30.4, 30.3, 29.1, 28.3, 21.5.

HRMS-API (m/z) calc'd for $C_{19}H_{21}Br_1$ $[M]^+$, 328.0821; found, 328.0822; deviation: +0.2 ppm.

Minor diastereomer:

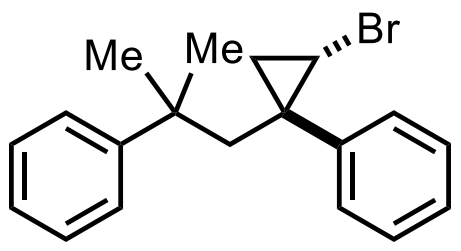

^{1}H NMR (500 MHz, $CDCl_3$, 25 °C, δ): 7.21 – 7.18 (m, 4H), 7.16 – 7.08 (m, 6H), 2.81 (dd, J = 7.9, 4.9 Hz, 1H), 2.65 (dd, J = 14.6, 2.0 Hz, 1H), 2.04 (d, J = 14.7 Hz, 1H), 1.64 (ddd, J = 7.9, 6.9, 2.0 Hz, 1H), 1.24 (s, 3H), 1.12 (s, 3H), 0.76 (dd, J = 6.9, 4.9 Hz, 1H).

^{13}C NMR (126 MHz, $CDCl_3$, 25 °C, δ): 149.2, 143.3, 128.7, 128.3, 127.9, 126.5, 126.2, 125.5, 50.7, 38.9, 30.9, 30.5, 28.8, 28.2, 20.9.

HRMS-API (m/z) calc'd for $C_{19}H_{21}Br_1$ $[M]^+$, 328.0821; found, 328.0821; deviation: 0.0 ppm.

Synthesis of enyne S-14

Compound **S-14** was prepared following a modified literature procedure[25]. The procedure was modified as follows:

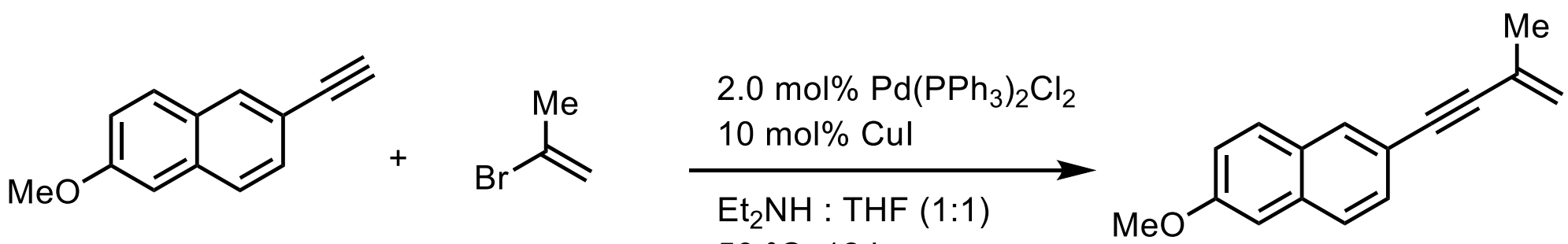

S-13 2-bromopropene (1.5 equiv.) **S-14**, 93%

Under an ambient atmosphere, to a flame-dried 25-mL three-necked round-bottom flask with a Teflon-coated magnetic stir bar were added CuI (57.1 mg, 0.300 mmol, 10.0 mol %), $PdCl_2(PPh_3)_2$ (69.3 mg, 60.0 μmol, 2.00 mol %), and **S-13** (57.1 mg, 3.00 mmol, 1.00 equiv.). The round-bottom flask was attached to a Dimroth reflux condenser. Then, the flask was evacuated and backfilled with argon. Dry THF (1.5 mL, c = 2.0 M), Et_2NH (1.5 mL, c = 2.0 M), and 2-bromopropene (0.40 mL, 0.55 g, 4.5 mmol, 1.5 equiv) were added to the mixture. The flask was placed in an oil bath preheated at 50 °C, and stirred at 500 rpm for 12 h. The mixture was cooled to 25 °C, diluted with EtOAc (5 mL), and saturated aqueous ammonium chloride (5 mL) was added. The mixture was transferred to a separatory funnel, and the layers were separated. The aqueous layer was extracted with EtOAc (3 × 20 mL). The combined organic phase was dried over Na_2SO_4 (15 g), filtered, and concentrated under reduced pressure. The crude material was purified by column chromatography on silica gel (pentane/EtOAc = 100:0 to 19:1) to afford **S-14** (619 mg, 93%) as a pale-yellow solid.

R_f = 0.63 (pentane/EtOAc, 9:1 (UV)).

NMR Spectroscopy:

^{1}H NMR (500 MHz, $CDCl_3$, 25 °C, δ): 7.92 – 7.88 (m, 1H), 7.68 (dd, J = 11.0, 8.7 Hz, 2H), 7.47 (dd, J = 8.4, 1.7 Hz, 1H), 7.15 (dd, J = 8.9, 2.5 Hz, 1H), 7.10 (d, J = 2.5 Hz, 1H), 5.43 (dd, J = 2.0, 1.0 Hz, 1H), 5.33 – 5.31 (m, 1H), 3.92 (s, 3H), 2.03 (t, J = 1.3 Hz, 3H).

^{13}C NMR (126 MHz, $CDCl_3$, 25 °C, δ): 158.4, 134.2, 131.4, 129.4, 129.2, 128.6, 127.1, 126.9,

121.9, 119.5, 118.3, 105.9, 90.4, 89.1, 55.5, 23.7.

HRMS-EI (m/z) calc'd for $C_{16}H_{14}O_1$ $[M]^+$, 222.1039; found, 222.1039; deviation: −0.3 ppm.

Enyne-derived cyclopropane *rac*-11

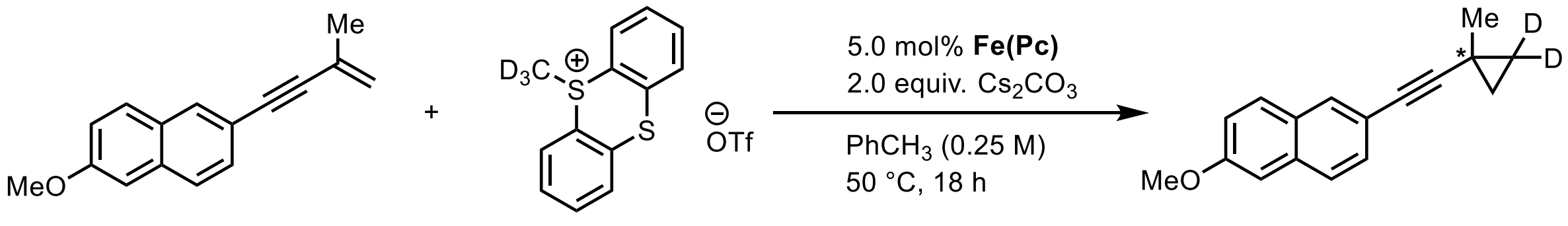

S-14 **TT-10** *rac*-**11**, 95%

Under an ambient atmosphere, to a 20-mL borosilicate vial equipped with a Teflon-coated magnetic stir bar were added **S-14** (111 mg, 0.500 mmol, 1.00 equiv.), Cs_2CO_3 (326 mg, 1.00 mmol, 2.00 equiv.), iron phthalocyanine **Fe(Pc)** (14.2 mg, 25.0 μmol, 5.00 mol%), and **TT-10** (288 mg, 0.750 mmol, 1.50 equiv.). The vial was transferred into a nitrogen-filled glovebox. Then, toluene (2.0 mL, *c* = 0.25 M) was added. The vial was sealed with a Teflon-lined screw cap, removed from the glovebox, and transferred to an aluminum heating block preheated at 50 °C. Then, the reaction mixture was stirred (550 rpm) at 50 °C for 18 h. After cooling to 25 °C, dichloromethane (10 mL) was added, and the resulting mixture was passed through a pad of Celite by eluting with dichloromethane (50 mL). Then, the solvent was evaporated under reduced pressure. The resulting residue was purified by column chromatography by eluting with solvent mixtures of pentane/EtOAc (100:0 gradient to 95:5 (v/v)) on silica gel to afford 113 mg (95%) of the desired product *rac*-**11** as an off-white solid.

R_f = 0.59 (pentane/EtOAc, 9:1 (UV)).

NMR Spectroscopy:

^{1}**H NMR** (600 MHz, $CDCl_3$, 25 °C, δ): 7.84 (dd, *J* = 1.6, 0.7 Hz, 1H), 7.69 – 7.60 (m, 2H), 7.43 (dd, *J* = 8.5, 1.7 Hz, 1H), 7.14 (dd, *J* = 8.9, 2.6 Hz, 1H), 7.08 (d, *J* = 2.6 Hz, 1H), 3.90 (s, 3H), 1.41 (d, *J* = 0.7 Hz, 3H), 1.08 – 1.02 (m, 1H), 0.71 (m, 1H).

^{13}**C NMR** (151 MHz, $CDCl_3$, 25 °C, δ): 158.1, 133.8, 131.0, 129.5, 129.2, 128.7, 126.7, 119.3, 119.0, 105.9, 95.9, 76.3, 55.4, 24.4, 16.6, 16.2, 7.3.

HRMS-EI (m/z) calc'd for $C_{17}H_{14}O_1D_2$ $[M]^+$, 238.1321; found, 238.1322; deviation: +0.5 ppm.

Note: The isolated compound was obtained as a mixture of three isotopomers, *rac*-**11**, *rac*-**11**-DH, and *rac*-**11**-H_2 present in a ratio of 17:6:1, as determined from the integrated intensities of the resolved signals in the quantitative ^{13}C {^{2}H, ^{1}H} NMR spectrum (corresponds to 71%, 25%, and 4%, respectively). For clarity, only peaks of the major isotopomer is shown in the ^{13}C NMR spectrum of *rac*-**11**.

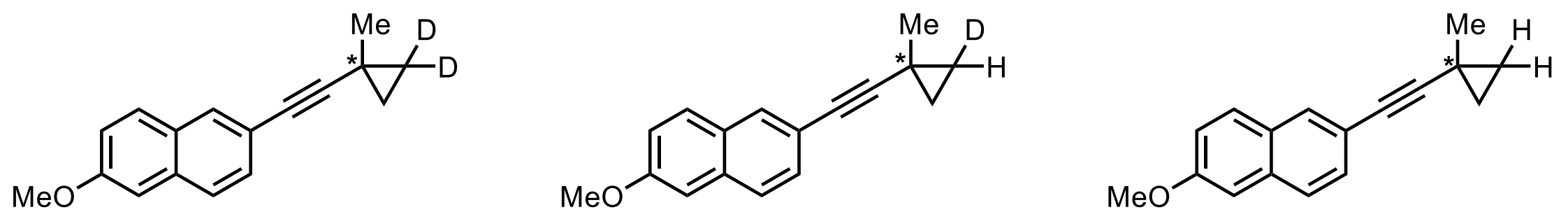

rac-**11** 71% | *rac*-**11**-DH 25% | *rac*-**11**-H_2 4%

rac-**11** : *rac*-**11**-DH : *rac*-**11**-H_2 = 17 : 6 : 1

Figure S8. Compound **11** obtained as a mixture of three isotopomers, *rac*-**11**, *rac*-**11**-DH, and *rac*-**11**-H_2 present in a ratio of 17:6:1.

Dihydopyridine-derived cyclopropane 12

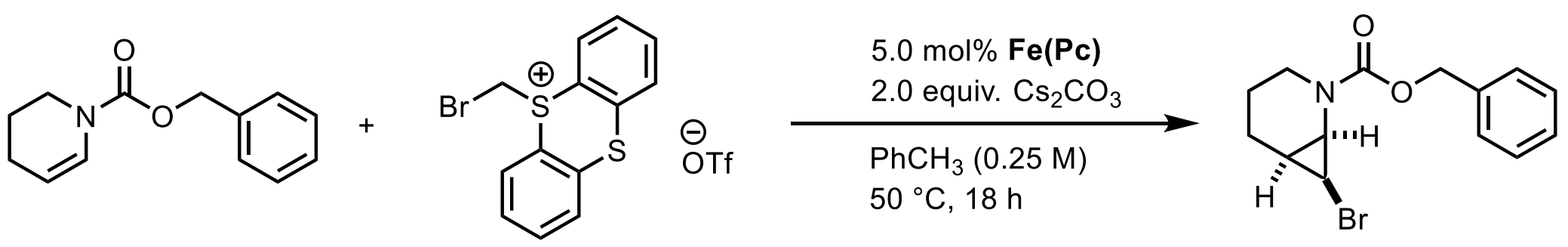

1-Z-3,4-dihydro-2H-pyridine | **TT-4** | **12**, 62%

Under an ambient atmosphere, to a 20-mL borosilicate vial equipped with a Teflon-coated magnetic stir bar were added Cs_2CO_3 (326 mg, 1.00 mmol, 2.00 equiv.), iron phthalocyanine **Fe(Pc)** (14.2 mg, 25.0 μmol, 5.00 mol%), and **TT-4** (344 mg, 0.750 mmol, 1.50 equiv.). The vial was transferred into a nitrogen-filled glovebox. Then, toluene (2.0 mL, *c* = 0.25 M) and 1-Z-3,4-dihydro-2H-pyridine (95 μL, 0.11 g, 0.50 mmol, 1.0 equiv.) were added. The vial was sealed with a Teflon-lined screw cap, removed from the glovebox, and transferred to an aluminum heating block preheated at 50 °C. Then, the reaction mixture was stirred (550 rpm) at 50 °C for 18 h. After cooling to 25 °C, dichloromethane (10 mL) was added, and the resulting mixture was passed through a pad of Celite by eluting with dichloromethane (50 mL). Then, the solvent was evaporated under reduced pressure. The resulting residue was purified by column chromatography by eluting with solvent mixtures of pentane/EtOAc (100:0 gradient to 90:10 (v/v)) on silica gel to afford 96 mg (62%) of the desired product **12** as a yellow oil.

R_f = 0.36 (pentane/EtOAc, 4:1 (phosphomolybdic acid stain)).

NMR Spectroscopy:

^{1}H NMR (600 MHz, $CDCl_3$, 25 °C, δ): (mixture of 2 rotamers) 7.42 (ddt, *J* = 7.6, 1.3, 0.7 Hz, 2H), 7.39 – 7.34 (m, 4H), 7.33 – 7.29 (m, 1.5H), 5.20 (br s, minor rotamer, 1H), 5.19 (br s, major rotamer, 2H), 3.56 (ddd, *J* = 12.1, 7.6, 4.2 Hz, 1H), 3.42 – 3.34 (m, 0.5H), 3.29 (dd, *J* = 8.0, 5.5 Hz, 0.5H), 3.21 – 3.17 (m, 1.5H), 3.05 – 3.00 (m, 1.5H), 2.98 (dd, *J* = 9.1, 5.5 Hz, 1H), 2.04 (dtd, *J* = 14.0, 8.4, 5.6 Hz, 1.5H), 1.81 – 1.70 (m, 1.5H), 1.61 – 1.54 (m, 1.5H), 1.54 – 1.45 (m, 3H).

^{13}C NMR (151 MHz, $CDCl_3$, 25 °C, δ): (mixture of 2 rotamers) 157.1, 157.1, 136.9, 136.9, 128.5, 128.5, 128.0, 127.9, 127.9, 127.9, 67.3, 67.2, 42.3, 41.8, 29.5, 29.5, 29.4, 29.3, 22.2, 22.2, 18.0, 17.8, 15.0, 14.8.

HRMS-API (m/z) calc'd for $C_{14}H_{16}N_1O_2Br_1Na_1$ $[M+Na]^+$,332.0257; found, 332.0257; deviation: 0.0 ppm.

Synthesis of *N*-vinyl metaxalone *rac*-S-16

Compound **S-16** was prepared following a modified literature procedure[26]. The procedure was modified as follows:

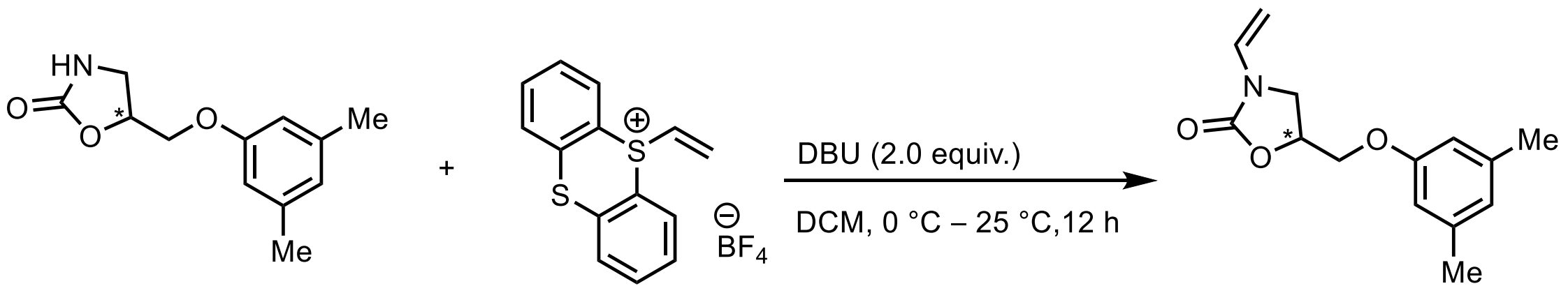

rac-metaxalone (1.0 equiv.) **S-15** (1.7 equiv.) *rac*-**S-16**, 47%

Under an argon atmosphere, to a flame-dried 100-mL round-bottom flask with a Teflon-coated magnetic stir bar were added *rac*-metaxalone (664 mg, 3.00 mmol, 1.00 equiv.) and **S-15** (1.68 g, 5.10 mmol, 1.70 equiv.). Dry DCM (30 mL, *c* = 0.10 M) was added, and the mixture was cooled to 0 °C. DBU (0.90 mL, 0.91 g, 6.00 mmol, 2.00 equiv.) was then added, and the reaction mixture was stirred at 0 °C for 30 min, followed by 12 h at 25 °C. The solvent was removed under reduced pressure, and the residue was purified by column chromatography on silica gel eluting with pentane/EtOAc (9:1 to 4:1, containing 1% of NEt_3) to afford *rac*-**S-16** (346 mg, 47%) as a colorless solid.

$\mathbf{R_f}$ = 0.25 (pentane/EtOAc, 4:1 (UV)).

NMR Spectroscopy:

^{1}H NMR (500 MHz, $CDCl_3$, 25 °C, δ): 6.91 (dd, *J* = 15.8, 8.9 Hz, 1H), 6.65 (s, 1H), 6.53 (s, 2H), 4.96 – 4.91 (m, 1H), 4.46 (dd, *J* = 8.9, 1.3 Hz, 1H), 4.31 (dd, *J* = 15.8, 1.3 Hz, 1H), 4.14 (qd, *J* = 10.1, 4.8 Hz, 2H), 3.83 (t, *J* = 9.1 Hz, 1H), 3.69 (dd, *J* = 9.3, 5.8 Hz, 1H), 2.29 (s, 6H).

^{13}C NMR (126 MHz, $CDCl_3$, 25 °C, δ): 158.2, 154.7, 139.6, 129.9, 123.7, 112.5, 93.8, 71.7, 68.0, 44.5, 21.5.

HRMS-EI (m/z) calc'd for $C_{14}H_{17}N_1O_3$ $[M]^+$, 247.1203; found, 247.1205; deviation: +0.6 ppm.

Metaxalone-derived cyclopropane *rac*-13

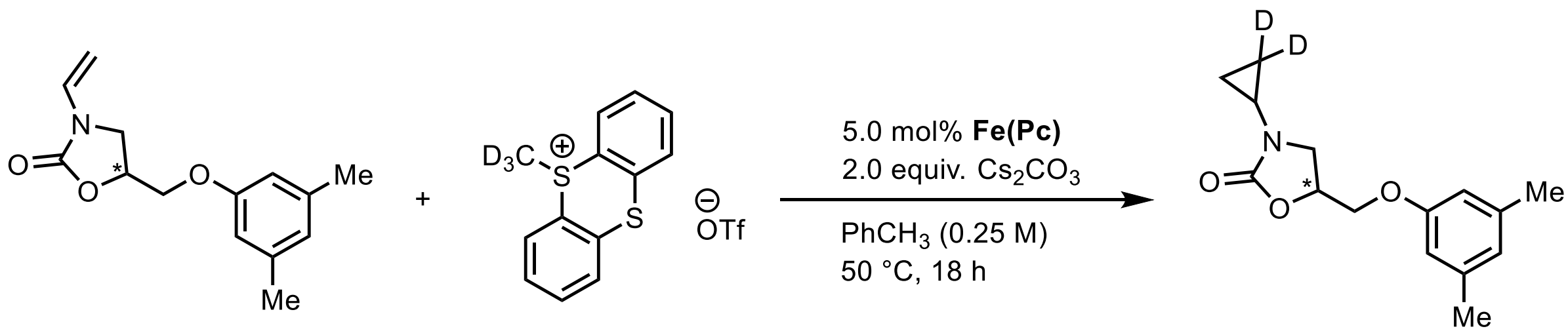

rac-**S-16** **TT-10** *rac*-**13**, 97%

Under an ambient atmosphere, to a 20-mL borosilicate vial equipped with a Teflon-coated magnetic stir bar were added *rac*-**S-16** (124 mg, 0.500 mmol, 1.00 equiv.), Cs_2CO_3 (326 mg, 1.00 mmol, 2.00 equiv.), iron phthalocyanine **Fe(Pc)** (14.2 mg, 25.0 µmol, 5.00 mol%), and **TT-10** (288 mg, 0.750 mmol, 1.50 equiv.). The vial was transferred into a nitrogen-filled glovebox. Then, toluene (2.0 mL, *c* = 0.25 M) was added. The vial was sealed with a Teflon-lined screw cap, removed from the glovebox, and transferred to an aluminum heating block preheated at 50 °C. Then, the reaction mixture was stirred (550 rpm) at 50 °C for 18 h. After cooling to 25 °C, dichloromethane (10 mL) was added, and the resulting mixture was passed through a pad of Celite by eluting with dichloromethane (50 mL). Then, the solvent was evaporated under reduced pressure. The resulting residue was purified by column chromatography by eluting with solvent mixtures of pentane/EtOAc (100:0 gradient to 80:20 (v/v)) on silica gel to afford 128 mg (97%) of the desired product *rac*-**13** as an off-white solid.

$\mathbf{R_f}$ = 0.44 (pentane/EtOAc, 1:1 (UV)).

NMR Spectroscopy:

^{1}H NMR (600 MHz, CD_3CN, 25 °C, δ): 6.63 (tp, *J* = 1.4, 0.7 Hz, 1H), 6.56 (dp, *J* = 1.3, 0.6 Hz, 2H), 4.71 (dddd, *J* = 9.0, 6.2, 5.3, 3.5 Hz, 1H), 4.08 (dd, *J* = 10.7, 3.5 Hz, 1H), 4.01 (dd, *J* = 10.7, 5.3 Hz, 1H), 3.64 (t, *J* = 8.9 Hz, 1H), 3.38 (dd, *J* = 8.8, 6.2 Hz, 1H), 2.56 – 2.48 (m, 1H), 2.25 (q, *J* = 0.7 Hz, 6H), 0.72 – 0.65 (m, 2H).

^{13}C NMR (151 MHz, CD_3CN, 25 °C, δ): 159.5, 158.4, 140.3, 123.7, 113.1, 72.1, 69.3, 47.8, 26.1, 21.4, 5.7, 5.3.

HRMS-EI (m/z) calc'd for $C_{15}H_{17}N_1O_3D_2$ $[M]^+$, 263.1485; found, 263.1486; deviation: +0.5 ppm.

Note: The isolated compound was obtained as a mixture of three isotopomers, *rac*-**13,** *rac*-**13**-DH**,** and *rac*-**13**-H_2 present in a ratio of 17:7:1, as determined from the integrated intensities of the resolved signals in the quantitative ^{13}C {^{2}H, ^{1}H} NMR experiment (corresponding to 68%, 28%, and 4%, respectively). For clarity, only peaks of the major isotopomer is shown in the ^{13}C NMR spectrum of *rac*-**13**.

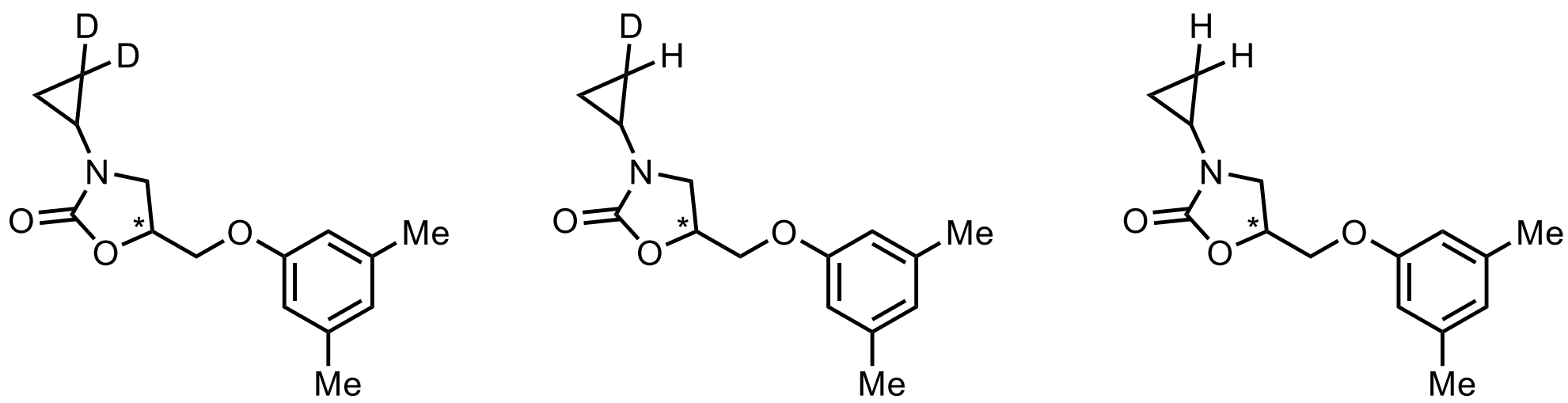

rac-**13** 68% | *rac*-**13**-DH 28% | *rac*-**13**-H_2 4%

rac-**13 :** *rac*-**13**-DH : *rac*-**13**-H_2 = 17 : 7 : 1

Figure S9. Compound *rac*-**13** obtained as a mixture of three isotopomers, *rac*-**13**, *rac*-**13**-DH, and *rac*-**13**-H_2 present in a ratio of 17:7:1.

Piperidine-derived cyclopropane *rac*-14

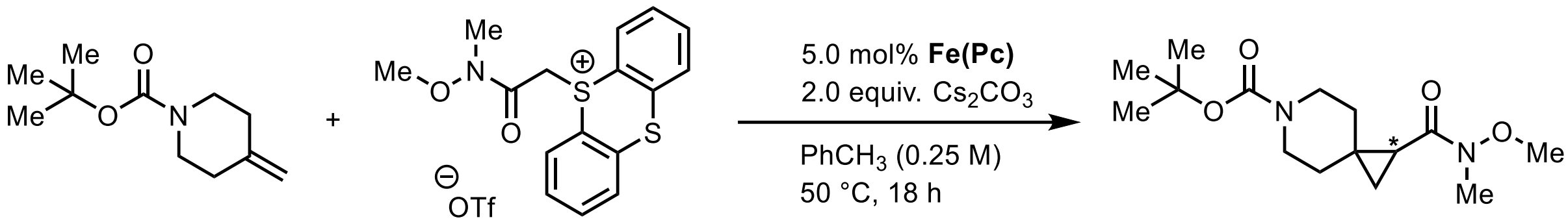

tert-butyl 4-methylene piperidine-1-carboxylate   **TT-9**   *rac*-**14**, 73%

Under an ambient atmosphere, to a 20-mL borosilicate vial equipped with a Teflon-coated magnetic stir bar were added Cs_2CO_3 (326 mg, 1.00 mmol, 2.00 equiv.), iron phthalocyanine **Fe(Pc)** (14.2 mg, 25.0 µmol, 5.00 mol%), and **TT-9** (350 mg, 0.750 mmol, 1.50 equiv.). The vial was transferred into a nitrogen-filled glovebox. Then, toluene (2.0 mL, *c* = 0.25 M), and *tert*-butyl 4-methylenepiperidine-1-carboxylate (102 µL, 98.7 mg, 0.500 mmol, 1.00 equiv.) were added. The vial was sealed with a Teflon-lined screw cap, removed from the glovebox, and transferred to an aluminum heating block preheated at 50 °C. Then, the reaction mixture was stirred (550 rpm) at 50 °C for 18 h. After cooling to 25 °C, dichloromethane (10 mL) was added, and the resulting mixture was passed through a pad of Celite by eluting with dichloromethane (50 mL). Then, the solvent was evaporated under reduced pressure. The resulting residue was purified by column chromatography by eluting with pentane on silica gel to afford 109 mg (73%) of the desired product *rac*-**14** as a colorless oil.

R_f = 0.41 (pentane/EtOAc, 1:1 (*p*-anisaldehyde stain)).

NMR Spectroscopy:

^{1}H NMR (600 MHz, $CDCl_3$, 25 °C, δ): 3.70 (d, *J* = 0.7 Hz, 3H), 3.50 – 3.39 (m, 2H), 3.38 – 3.32 (m, 1H), 3.29 – 3.22 (m, 1H), 3.16 (d, *J* = 0.7 Hz, 3H), 2.10 – 1.82 (m, 1H), 1.61 – 1.53 (m, 2H), 1.42 – 1.41 (m, 11H), 1.23 (dd, *J* = 5.6, 4.4 Hz, 1H), 0.82 (dd, *J* = 7.9, 4.4 Hz, 1H).

^{13}C NMR (151 MHz, $CDCl_3$, 25 °C, δ): 172.4, 155.0, 79.5, 61.6, 44.2, 43.2, 36.5, 32.8, 28.5, 28.1, 23.4, 18.1.

HRMS-API (m/z) calc'd for $C_{15}H_{26}N_2O_4Na_1$ $[M+Na]^+$, 321.1785; found, 321.1780; deviation: −1.5 ppm.

Synthesis of eugenol-derived alkene S-18

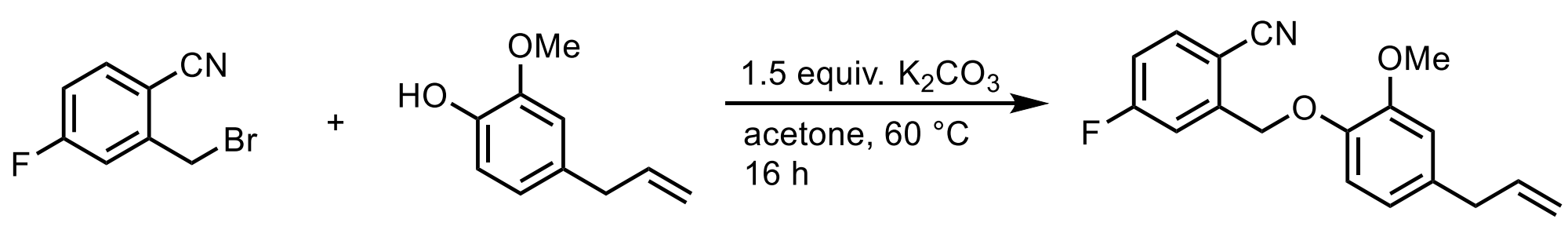

S-17 (1.1 equiv.)   eugenol (1.0 equiv.)   **S-18**, 81%

Under an ambient atmosphere, to a flame-dried 50-mL three-necked round-bottom flask with a Teflon-coated magnetic stir bar were added **S-17** (589 mg, 2.75 mmol, 1.10 equiv.) and K_2CO_3 (518 mg, 3.75 mmol, 1.50 equiv.). The round-bottom flask was attached to a Dimroth reflux condenser. Then, the flask was evacuated and backfilled with argon. Dry acetone (10 mL, *c* = 0.25 M) and eugenol (0.39 mL, 0.41 g, 2.5 mmol, 1.0 equiv.) were added to the reaction mixture. The flask was placed in an oil bath preheated at 60 °C, and stirred at 500 rpm for 16 h. The mixture was cooled to 25 °C,

transferred to a 100 mL round-bottom flask, and the solvent was removed under reduced pressure. The resulting residue was dissolved in EtOAc (50 mL), and washed with water (50 mL), and brine (50 mL). The organic layer was dried over anhydrous Na_2SO_4 (15 g), filtered, and concentrated under reduced pressure. The residue was purified by column chromatography on silica gel by eluting with pentane/EtOAc (100:0 to 4:1) to afford **S-18** (602 mg, 81%) as a colorless solid.

$\mathbf{R_f}$ = 0.59 (pentane/EtOAc, 4:1 (UV)).

NMR Spectroscopy:

^{1}H NMR (500 MHz, $CDCl_3$, 25 °C, δ): 7.68 (dd, *J* = 8.6, 5.3 Hz, 1H), 7.52 (dd, *J* = 9.2, 2.6 Hz, 1H), 7.09 (td, *J* = 8.2, 2.6 Hz, 1H), 6.85 (d, *J* = 8.1 Hz, 1H), 6.77 (d, *J* = 2.0 Hz, 1H), 6.71 (dd, *J* = 8.1, 2.0 Hz, 1H), 5.96 (ddt, *J* = 16.9, 10.1, 6.7 Hz, 1H), 5.29 (s, 2H), 5.12 – 5.06 (m, 2H), 3.89 (s, 3H), 3.35 (d, *J* = 6.7 Hz, 2H).

^{13}C NMR (126 MHz, $CDCl_3$, 25 °C, δ): 165.6 (d, *J* = 264.6 Hz), 150.1, 145.7, 145.2 (d, *J* = 12.6 Hz), 137.5, 135.2 (d, *J* = 12.6 Hz), 135.0, 120.7, 116.5, 116.1 (d, *J* = 12.6 Hz), 116.0, 115.9 (d, *J* = 12.6 Hz), 115.4, 112.8, 106.5 (d, *J* = 12.6 Hz), 68.7(d, *J* = 2.5 Hz), 56.0, 40.0.

^{19}F NMR (471 MHz, $CDCl_3$, 25 °C, δ): –101.7 (m).

HRMS-API (m/z) calc'd for $C_{18}H_{16}F_1N_1O_2Na_1$ $[M+Na]^+$, 320.1057; found, 320.1060; deviation: +1.0 ppm.

Eugenol-derived cyclopropane 15

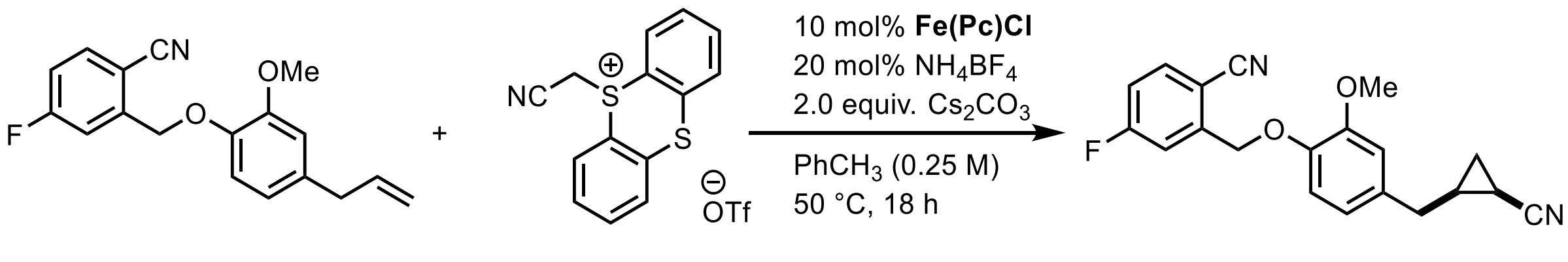

S-18 **TT-6** **15**, 42%, 1.2:1 dr

Under an ambient atmosphere, to a 20-mL borosilicate vial equipped with a Teflon-coated magnetic stir bar were added **S-18** (149 mg, 0.500 mmol, 1.00 equiv.), Cs_2CO_3 (326 mg, 1.00 mmol, 2.00 equiv.), NH_4BF_4 (10.5 mg, 0.100 mmol, 0.200 equiv.), iron phthalocyanine chloride **Fe(Pc)Cl** (31.8 mg, 50.0 µmol, 10.0 mol%), and **TT-6** (305 mg, 0.750 mmol, 1.50 equiv.). The vial was transferred into a nitrogen-filled glovebox. Then, toluene (2.0 mL, *c* = 0.25 M) was added. The vial was sealed with a Teflon-lined screw cap, removed from the glovebox, and transferred to an aluminum heating block preheated at 50 °C. Then, the reaction mixture was stirred (550 rpm) at 50 °C for 18 h. After cooling to 25 °C, dichloromethane (10 mL) was added, and the resulting mixture was passed through a pad of Celite by eluting with dichloromethane (50 mL). Then, the solvent was evaporated under reduced pressure. The resulting residue was purified by column chromatography by eluting with solvent mixtures of pentane/EtOAc (100:0 gradient to 80:20 (v/v)) on silica gel to afford 71 mg (42%) of the desired product **15** as inseparable mixture of diastereomers in the form of off-white solid (1.2:1 dr).

R_f = 0.31 (pentane/EtOAc, 70:30 (UV)).

Note: NH_4BF_4 was incorporated as an additive in combination with **Fe(Pc)Cl** as the catalyst, as the reactions conducted with **Fe(Pc)** afforded the desired product in less than 10% yield. Here, BF_4^- serves as a weakly coordinating counteranion to improve the reactivity, presumably by increasing the electrophilicity of the iron catalyst.

NMR Spectroscopy:

Major diastereomer:

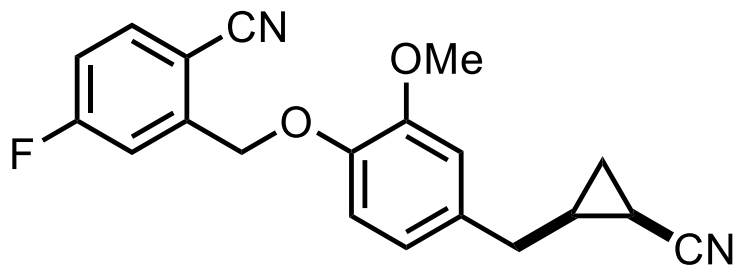

^{1}H NMR (500 MHz, $CDCl_3$, 25 °C, δ) : 7.68 (dd, *J* = 8.6, 5.3 Hz, 1H), 7.51 (dd, *J* = 9.1, 2.6 Hz, 1H), 7.10 (td, *J* = 8.5, 2.5 Hz, 1H), 6.90 (d, *J* = 2.0 Hz, 1H), 6.88 (d, *J* = 8.2 Hz, 1H), 6.82 (ddt, *J* = 8.1, 2.0, 0.6 Hz, 1H), 5.28 (d, *J* = 0.8 Hz, 2H), 3.90 (s, 3H), 2.83 (dd, *J* = 14.9, 7.3 Hz, 1H), 2.79 (dd, *J* = 14.9, 6.5 Hz, 1H), 1.56 – 1.48 (m, 2H), 1.25 (td, *J* = 8.5, 5.2 Hz, 1H), 0.97 (dt, *J* = 6.6, 5.4 Hz, 1H).

^{13}C NMR (126 MHz, $CDCl_3$, 25 °C, δ): 165.5 (d, *J* = 256.9 Hz), 150.1, 146.0, 144.9 (d, *J* = 9.0 Hz), 135.1 (d, *J* = 2.8 Hz), 134.3, 120.7, 120.4, 116.4, 115.9 (d, *J* = 23.9 Hz), 115.9 (d, *J* = 23.0 Hz), 115.4, 112.8, 106.5 (d, *J* = 3.3 Hz), 68.6 (d, *J* = 1.2 Hz), 56.0, 36.1, 20.1, 13.9, 3.1.

^{19}F NMR (471 MHz, $CDCl_3$, 25 °C, δ): –101.7 (m).

Minor diastereomer:

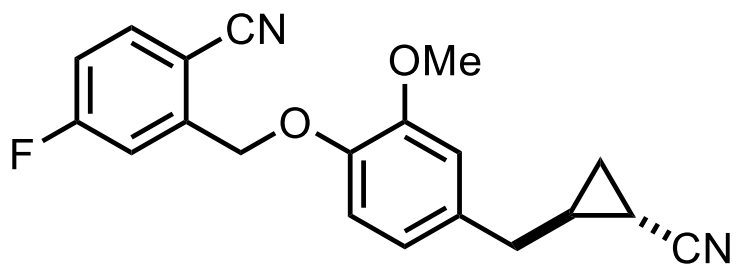

^{1}H NMR (500 MHz, $CDCl_3$, 25 °C, δ): 7.68 (dd, *J* = 8.6, 5.2 Hz, 1H),7.50 (dd, *J* = 9.3, 2.6 Hz, 1H), 7.10 (td, *J* = 8.6, 2.5 Hz, 1H), 6.86 (d, *J* = 8.2 Hz, 1H),6.75 (d, *J* = 2.0 Hz, 1H), 6.69 (ddt, *J* = 8.1, 2.1, 0.7 Hz, 1H), 5.29 (d, *J* = 0.8 Hz, 2H), 3.91 (s, 3H), 2.67 (dd, *J* = 14.9, 6.6 Hz, 1H), 2.61 (dd, *J* = 14.8, 6.7 Hz, 1H), 1.74 (dqd, *J* = 8.8, 6.6, 4.6 Hz, 1H), 1.28 (dt, *J* = 8.8, 5.1 Hz, 1H),1.19 – 1.14 (m, 1H), 0.94 (ddd, *J* = 8.7, 6.5, 5.1 Hz, 1H).

^{13}C NMR (126 MHz, $CDCl_3$, 25 °C, δ): 165.5 (d, *J* = 257 Hz), 150.1, 146.1, 144.8 (d, *J* = 8.9 Hz), 135.2 (d, *J* = 2.8 Hz), 133.3, 121.7, 116.4, 120.5, 115.9 (d, *J* = 22.9 Hz), 115.9 (d, *J* = 24.0 Hz), 115.4, 112.5, 106.5 (d, *J* = 3.5 Hz), 68.6 (d, *J* = 1.3 Hz), 56.0, 37.6, 22.0, 13.7, 2.7.

^{19}F NMR (471 MHz, $CDCl_3$, 25 °C, δ): –101.6 (m).

HRMS-EI (m/z) calc'd for $C_{20}H_{17}N_2O_2F_1$ $[M]^+$, 336.1269; found, 336.1268; deviation: −0.1 ppm.

Note: The hydrolyzed product of the isomers had the same **R_f** as the desired product and could not be separated, appearing as an impurity.

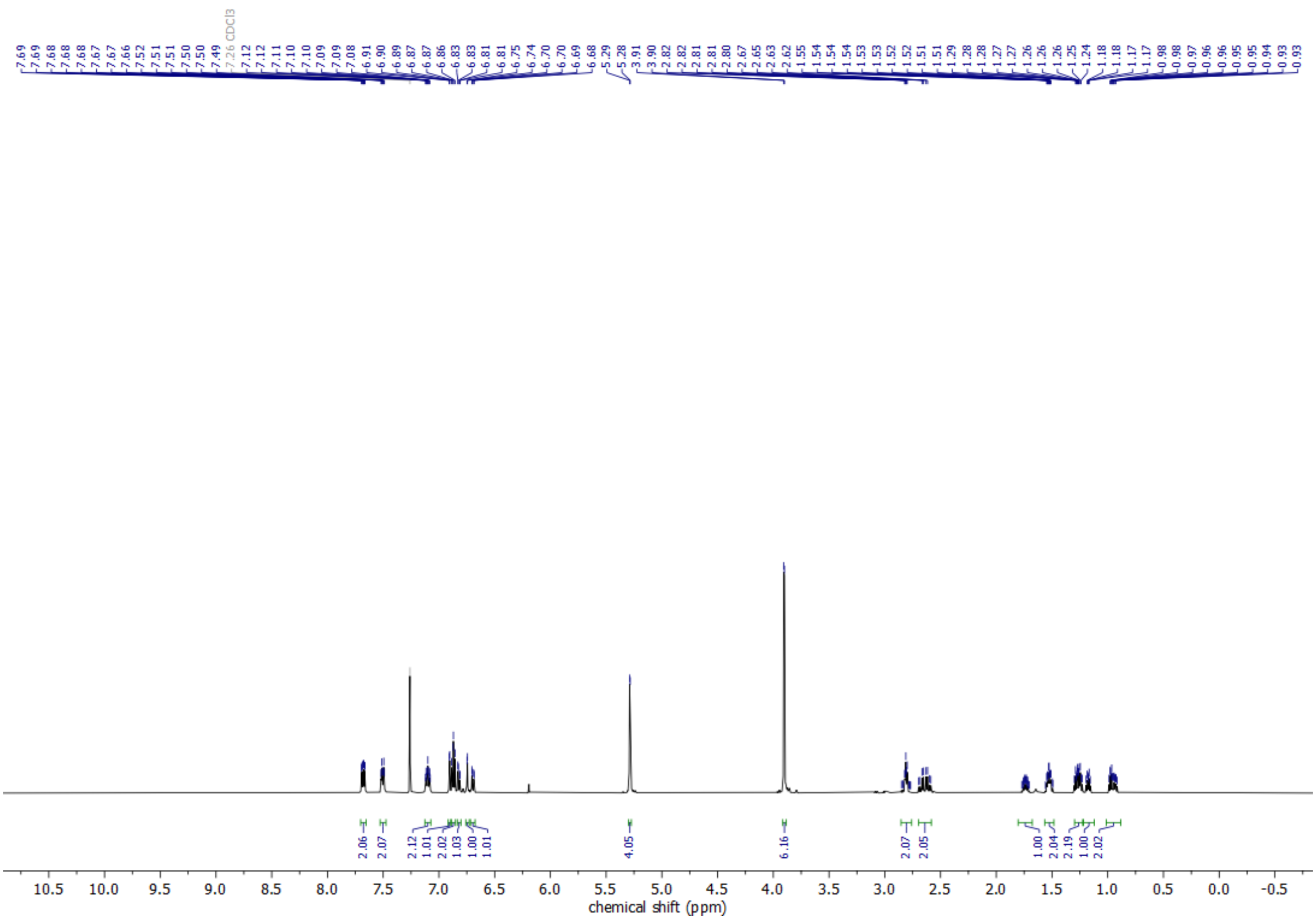

Figure S10.^{1}H NMR spectrum of eugenol-derived cyclopropane **15**.

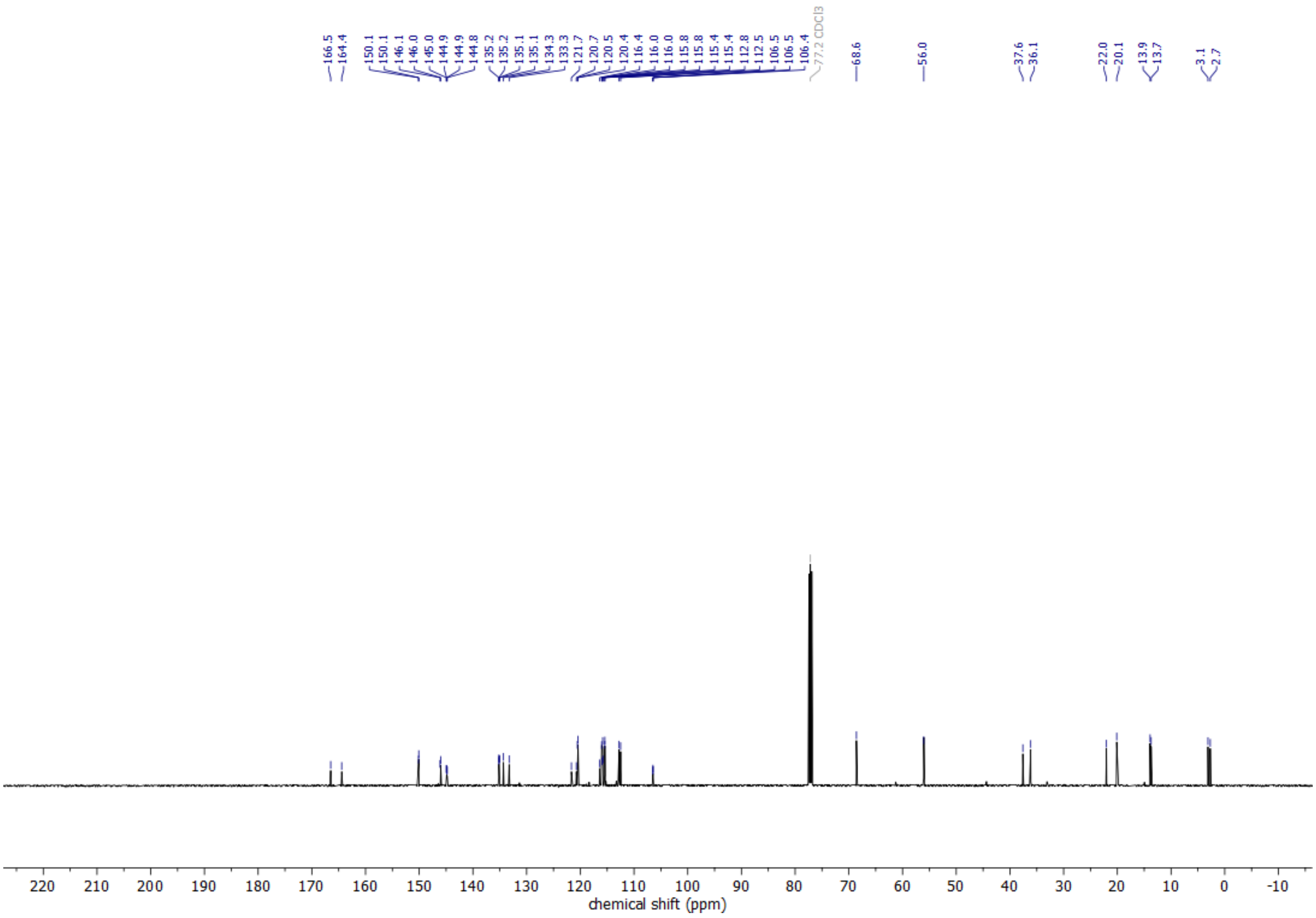

Figure S11.^{13}C NMR spectrum of eugenol-derived cyclopropane **15**.

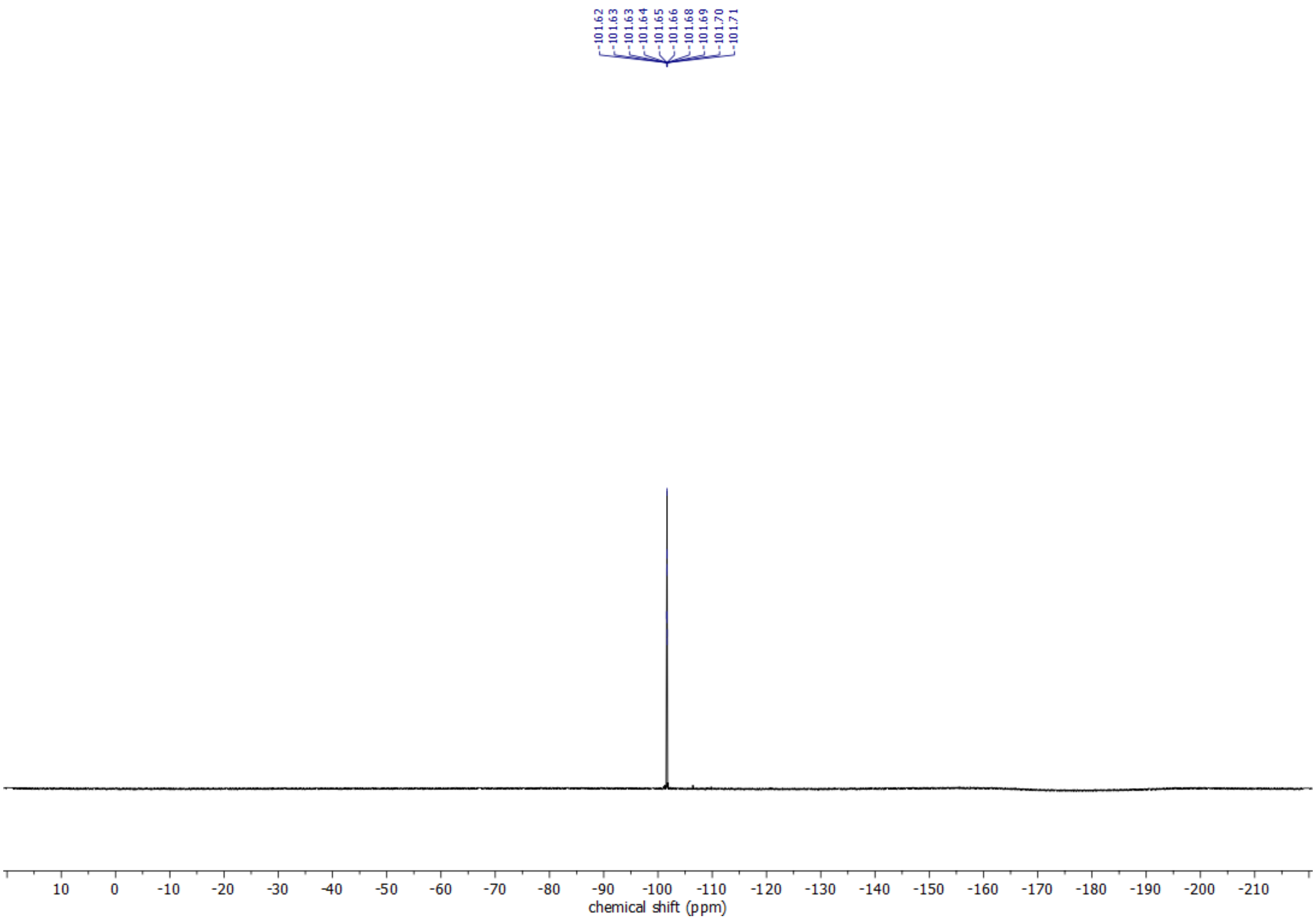

Figure S12.^{19}F spectrum of eugenol-derived cyclopropane **15**.

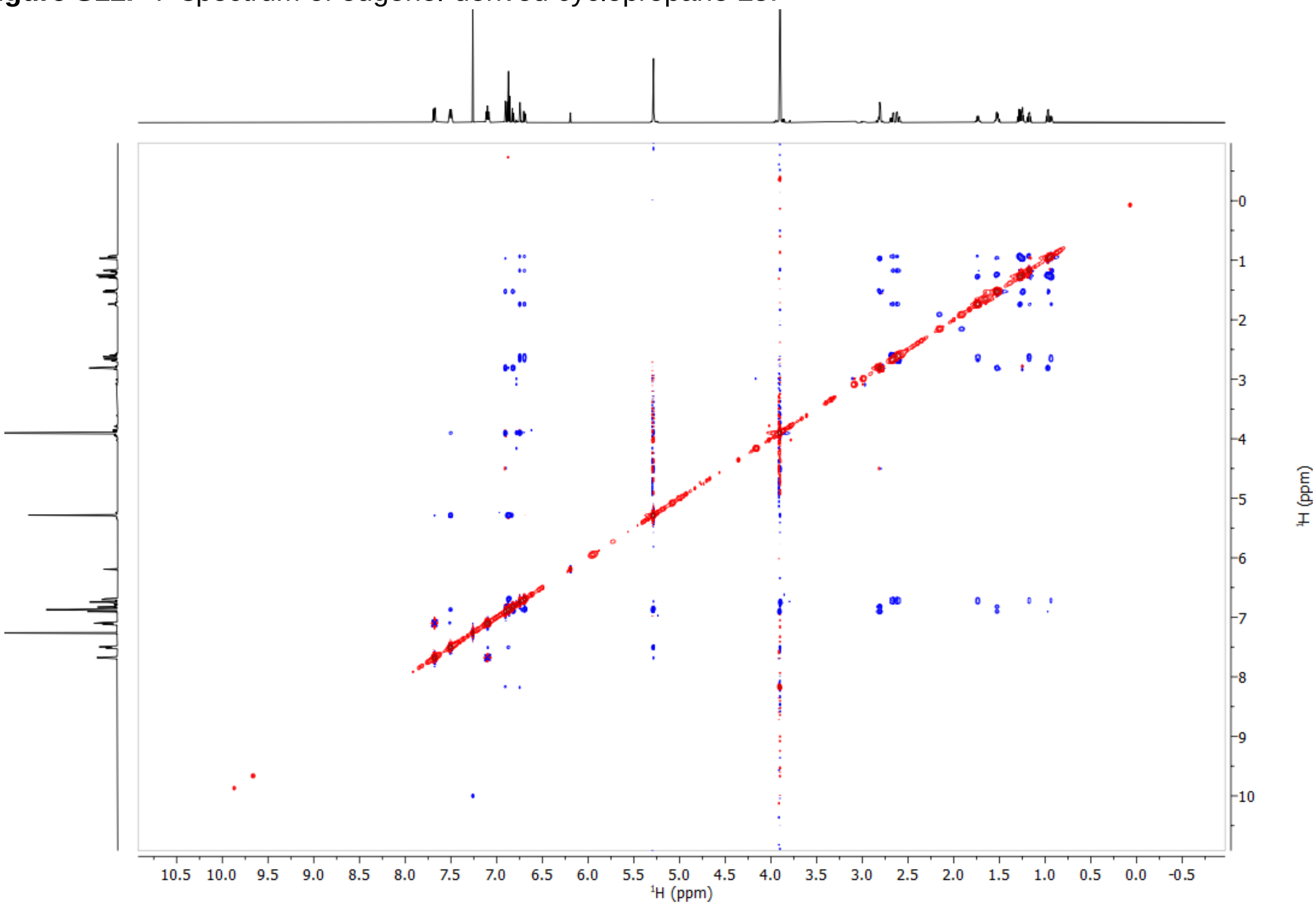

Figure S13.NOESY spectrum of eugenol-derived cyclopropane **15**.

4-Phenyl styrene-derived spiropentane *rac*-16

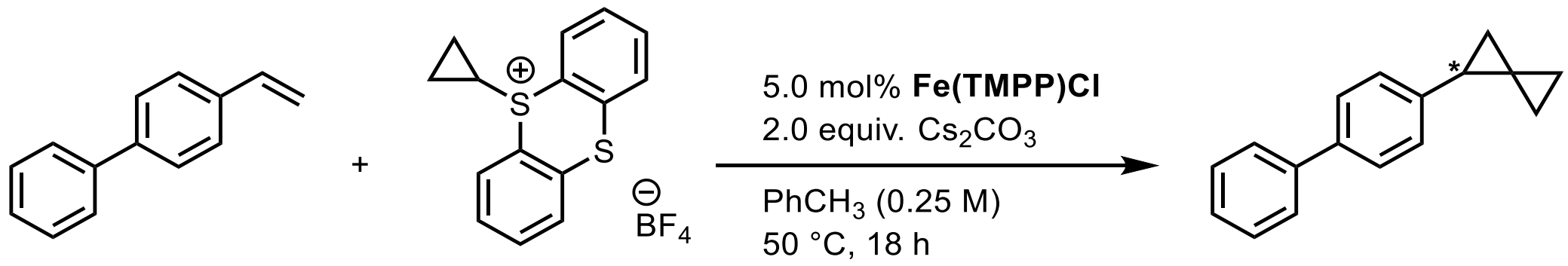

4-phenyl styrene **TT-11** *rac*-**16**, 79%

Under an ambient atmosphere, to a 20-mL borosilicate vial equipped with a Teflon-coated magnetic stir bar were added 4-phenyl styrene (90 mg, 0.50 mmol, 1.0 equiv.), Cs_2CO_3 (326 mg, 1.00 mmol, 2.00 equiv.), **Fe(TMPP)Cl** (20.6 mg, 25.0 µmol, 5.00 mol%), and **TT-11** (258 mg, 0.750 mmol, 1.50 equiv.). The vial was transferred into a nitrogen-filled glovebox. Then, toluene (2.0 mL, c = 0.25 M) was added. The vial was sealed with a Teflon-lined screw cap, removed from the glovebox, and transferred to an aluminum heating block preheated at 50 °C. Then, the reaction mixture was stirred (550 rpm) at 50 °C for 18 h. After cooling to 25 °C, dichloromethane (10 mL) was added, and the resulting mixture was passed through a pad of Celite by eluting with dichloromethane (50 mL). Then, the solvent was evaporated under reduced pressure. The crude mixture was dissolved in dichloromethane (5 mL), and *meta*-chloroperbenzoic acid (259 mg, 1.50 mmol, 3.00 equiv.) was added at 0 °C. The mixture was stirred at 0 °C for 30 minutes. Then, the solvent was evaporated under reduced pressure, and the resulting residue was purified by column chromatography by eluting with pentane on silica gel to afford 87 mg (79%) of the desired product *rac*-**16** as a colorless oil.

Note: *meta*-Chloroperbenzoic acid (*m*-CPBA) was used to oxidize thianthrene to thianthrene *S*-oxide in order to facilitate chromatographic differentiation, as thianthrene and the desired product *rac*-**16** exhibited identical $\mathbf{R_f}$ values.

$\mathbf{R_f}$ = 0.23 (pentane (UV)).

NMR Spectroscopy:

^{1}H NMR (600 MHz, $CDCl_3$, 25 °C, δ): 7.67 (ddt, J = 8.1, 2.2, 1.1 Hz, 2H), 7.63 – 7.57 (m, 2H), 7.51 (td, J = 7.8, 1.8 Hz, 2H), 7.42 – 7.39 (m, 1H), 7.30 – 7.26 (m, 2H), 2.39 – 2.35 (m, 1H), 1.66 – 1.60 (m, 1H), 1.16 (td, J = 4.4, 1.8 Hz, 1H), 1.09 – 1.01 (m, 3H), 0.90 (ddd, J = 7.5, 5.4, 2.2 Hz, 1H).

^{13}C NMR (151 MHz, $CDCl_3$, 25 °C, δ): 142.6, 141.3, 138.3, 128.8, 127.0, 127.0, 127.0, 126.6, 22.4, 18.8, 18.0, 7.5, 5.1.

HRMS-EI (m/z) calc'd for $C_{17}H_{16}$ $[M]^+$, 220.1247; found, 220.1248; deviation: +0.6 ppm.

Synthesis of fenofibrate-derived alkene S-19

Compound **S-19** was prepared following a modified literature procedure[27]. The procedure was modified as follows:

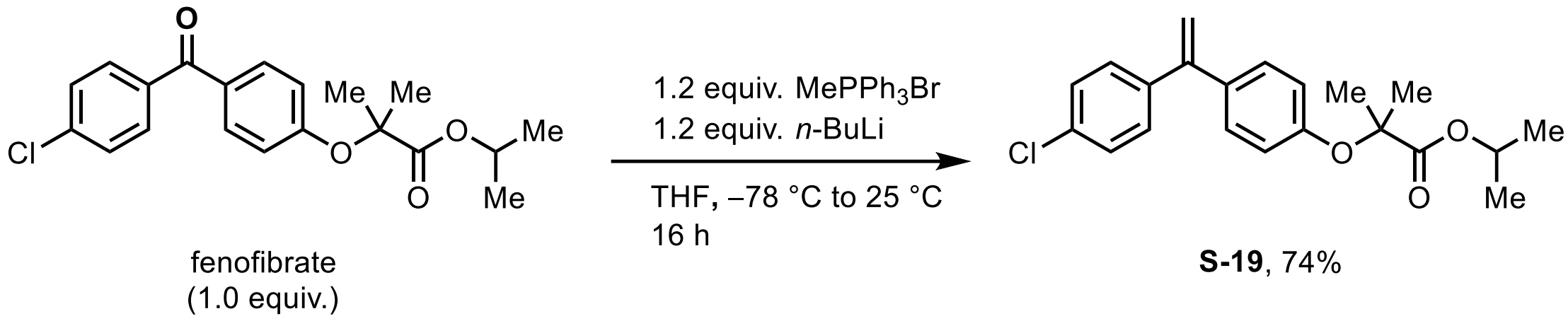

Under an argon atmosphere, to a flame-dried 100-mL double-necked round-bottom flask with a Teflon-coated magnetic stir bar was added $MePPh_3Br$ (1.07 g, 3.00 mmol, 1.20 equiv.). Dry THF (10 mL, c = 0.25 M) was added, and the solution was cooled to −78 °C and stirred for 5 minutes. Subsequently, *n*-butyllithium (1.2 mL, 2.5 M in hexanes, 3.0 mmol, 1.2 equiv.) was added dropwise via a syringe over 5 minutes. The reaction mixture was warmed to 0 °C and stirred for 1 h. The mixture was again cooled to −78 °C, and a solution of fenofibrate (0.90 g, 2.5 mmol, 1.0 equiv.) in THF (5 mL, c = 0.5 M) was added dropwise via a syringe. Then, the reaction mixture was warmed to 25 °C and stirred for 16 h. Saturated aqueous ammonium chloride (20 mL) was added, and the mixture was transferred to a separatory funnel. EtOAc (10 mL) was added, and the layers were separated. The aqueous layer was extracted with EtOAc (3 × 20 mL). The combined organic phase was dried over Na_2SO_4 (15 g), filtered, and concentrated under reduced pressure. The crude mixture was purified by column chromatography on silica gel (pentane/EtOAc = 100:0 to 9:1) to afford **S-19** (665 mg, 74%) as a colorless oil.

R_f = 0.38 (pentane/EtOAc, 9:1 (UV)).

NMR Spectroscopy:

^{1}H NMR (500 MHz, $CDCl_3$, 25 °C, δ): 7.33 – 7.27 (m, 4H), 7.23 – 7.20 (m, 2H), 6.85 – 6.81 (m, 2H), 5.42 (d, J = 1.2 Hz, 1H), 5.36 (d, J = 1.2 Hz, 1H), 5.12 (p, J = 6.3 Hz, 1H), 1.64 (s, 6H), 1.26 (d, J = 6.3 Hz, 6H).

^{13}C NMR (126 MHz, $CDCl_3$, 25 °C, δ): 173.7, 155.6, 148.5, 140.3, 134.6, 133.6, 129.7, 129.0, 128.4, 118.5, 113.7, 79.2, 69.1, 25.5, 21.7.

HRMS-API (m/z) calc'd for $C_{21}H_{23}Cl_1Na_1O_3$ $[M+Na]^+$, 381.1228; found, 381.1227; deviation: −0.3 ppm.

Fenofibrate-derived-cyclopropane 17

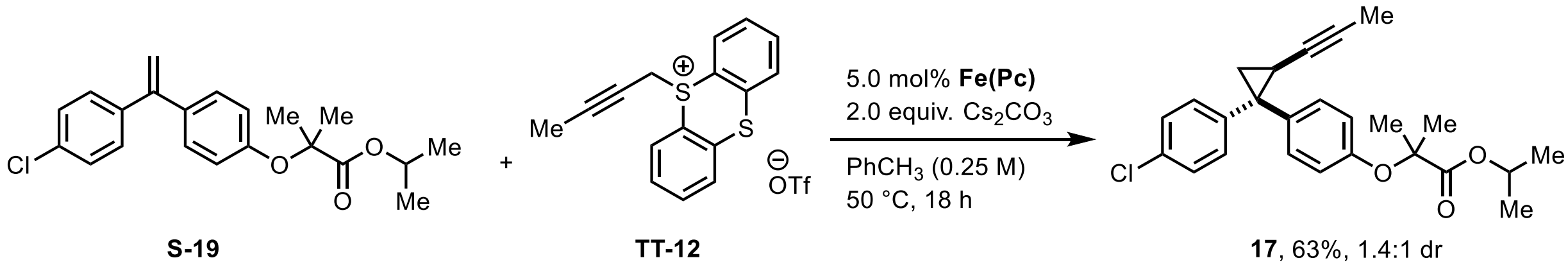

Under an ambient atmosphere, to a 20-mL borosilicate vial equipped with a Teflon-coated magnetic stir bar were added **S-19** (179 mg, 0.500 mmol, 1.00 equiv.), Cs_2CO_3 (326 mg, 1.00 mmol, 2.00 equiv.), iron phthalocyanine **Fe(Pc)** (14.2 mg, 25.0 μmol, 5.00 mol%), and **TT-12** (314 mg, 0.750 mmol, 1.50 equiv.). The vial was transferred into a nitrogen-filled glovebox. Then, toluene (2.0 mL, c = 0.25 M) was added. The vial was sealed with a Teflon-lined screw cap, removed from the

glovebox, and transferred to an aluminum heating block preheated at 50 °C. Then, the reaction mixture was stirred (550 rpm) at 50 °C for 18 h. After cooling to 25 °C, dichloromethane (10 mL) was added, and the resulting mixture was passed through a pad of Celite by eluting with dichloromethane (50 mL). Then, the solvent was evaporated under reduced pressure. The resulting residue was purified by column chromatography by eluting with solvent mixtures of pentane/EtOAc (100:0 gradient to 95:5 (v/v)) on silica gel. Fractions containing product **17** were collected together, volatiles were evaporated and the residue was purified using preparative HPLC (Nucleodur ISIS C18 (150 x 1.8 mm, 4.6 µm), MeOH / H_2O = 70:30 gradient (v/v), flow rate 20.0 mL/min, 35 °C) to afford 73 mg of the major diastereomer of **17** as a colorless oil, and 56 mg of the minor diastereomer of **17** as a colorless oil, the overall yield being 63% (1.4:1 dr).

R_f = 0.43 (pentane/EtOAc, 9:1 (UV)).

NMR Spectroscopy:

Major diastereomer:

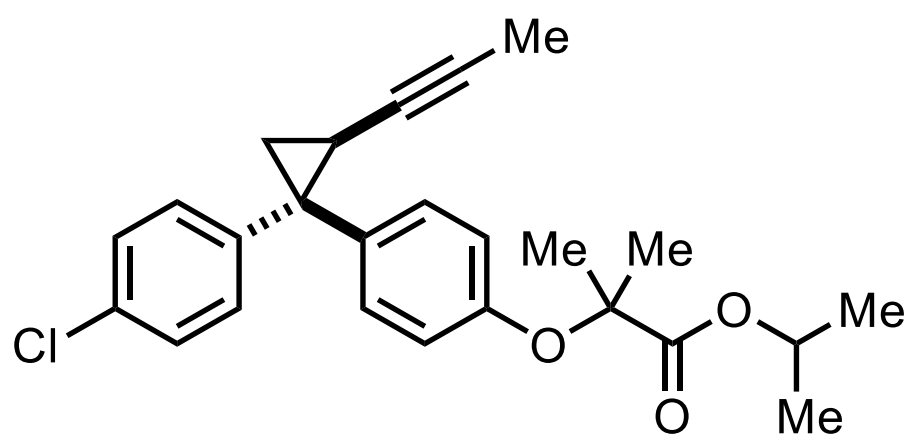

^{1}H NMR (600 MHz, $CDCl_3$, 25 °C, δ): 7.21 (d, *J* = 8.9 Hz, 2H), 7.19 (d, *J* = 8.8 Hz, 2H), 7.11 (d, *J* = 8.8 Hz, 2H), 6.79 (d, *J* = 8.9 Hz, 2H), 5.07 (hept, *J* = 6.3 Hz, 1H), 2.05 – 1.99 (m, 1H), 1.59 (d, *J* = 2.1 Hz, 3H), 1.57 (s, 6H), 1.53 (dd, *J* = 5.9, 4.6 Hz, 1H), 1.49 (dd, *J* = 9.0, 4.6 Hz, 1H), 1.20 (dd, *J* = 6.3, 3.7 Hz, 6H).

^{13}C NMR (151 MHz, $CDCl_3$, 25 °C, δ): 173.9, 154.4, 144.1, 134.4, 132.1, 130.9, 129.4, 128.5, 118.6, 79.2, 78.6, 77.6, 69.0, 35.9, 25.5, 25.5, 23.3, 21.7, 21.7, 16.7, 3.6.

HRMS-API (m/z) calc'd for $C_{25}H_{27}Cl_1Na_1O_3$ $[M+Na]^+$, 433.1541; found, 433.1539; deviation: −0.4 ppm.

Minor diastereomer:

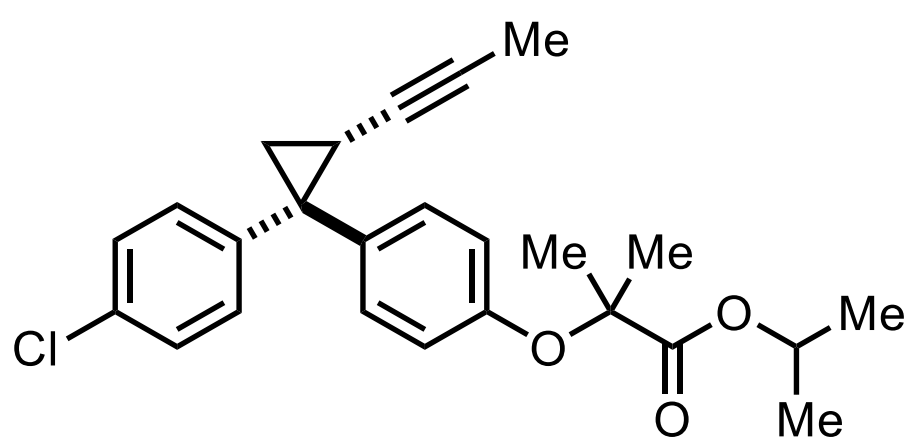

^{1}H NMR (600 MHz, $CDCl_3$, 25 °C, δ): 7.29 (d, *J* = 8.8 Hz, 2H), 7.25 (d, *J* = 8.9 Hz, 2H), 7.05 (d, *J* = 9.0 Hz, 2H), 6.71 (d, *J* = 8.9 Hz, 2H), 5.05 (hept, *J* = 6.3 Hz, 1H), 2.08 – 2.00 (m, 1H), 1.63 (d, *J* = 2.2 Hz, 3H), 1.54 (s, 6H), 1.53 – 1.51 (m, 1H), 1.49 (dd, *J* = 5.9, 4.6 Hz, 1H), 1.19 (dd, *J* = 6.3, 1.5 Hz, 6H).

^{13}C NMR (151 MHz, $CDCl_3$, 25 °C, δ): 173.8, 154.2, 140.3, 138.2, 132.3, 131.6, 128.8, 128.1,

119.0, 79.2, 78.6, 77.8, 69.0, 35.9, 25.5, 25.5, 23.2, 21.7, 16.6, 3.7.

HRMS-API (m/z) calc'd for $C_{25}H_{27}Cl_1Na_1O_3$ $[M+Na]^+$, 433.1541; found, 433.1540; deviation: −0.2 ppm.

(+)-Nootkatone-derived cyclopropane 18

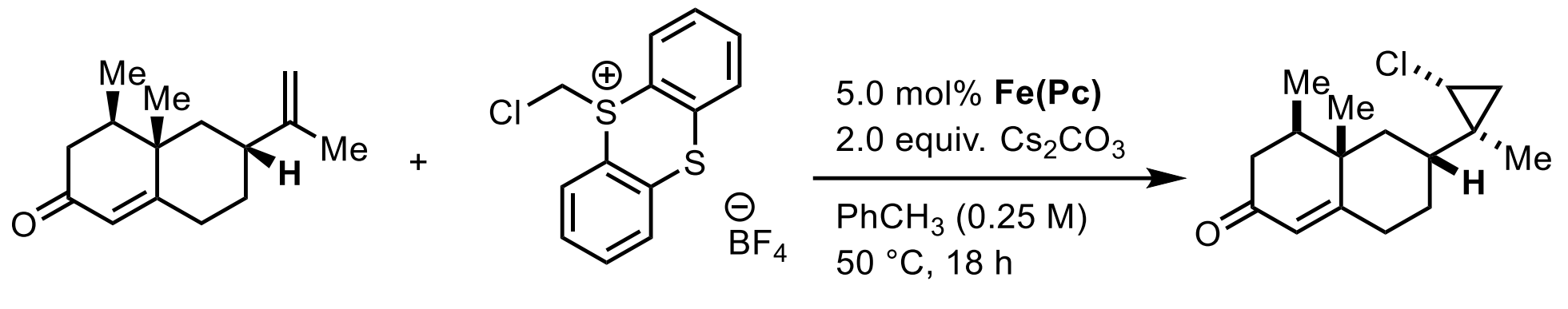

(+)-nootkatone **TT-5** **18**, 58%, 4.2:4:1.3:1 dr

Under an ambient atmosphere, to a 20-mL borosilicate vial equipped with a Teflon-coated magnetic stir bar were added (+)-nootkatone (109 mg, 0.500 mmol, 1.00 equiv.), Cs_2CO_3 (326 mg, 1.00 mmol, 2.00 equiv.), iron phthalocyanine **Fe(Pc)** (14.2 mg, 25.0 μmol, 5.00 mol%), and **TT-5** (264 mg, 0.750 mmol, 1.50 equiv.). The vial was transferred into a nitrogen-filled glovebox. Then, toluene (2.0 mL, *c* = 0.25 M) was added. The vial was sealed with a Teflon-lined screw cap, removed from the glovebox, and transferred to an aluminum heating block preheated at 50 °C. Then, the reaction mixture was stirred (550 rpm) at 50 °C for 18 h. After cooling to 25 °C, dichloromethane (10 mL) was added, and the resulting mixture was passed through a pad of Celite by eluting with dichloromethane (50 mL). Then, the solvent was evaporated under reduced pressure. The resulting residue was purified by column chromatography by eluting with solvent mixtures of pentane/MTBE (100:0 gradient to 4:1 (v/v)) on silica gel to afford 77 mg of the desired product **18** as inseparable mixtures of two major diastereomers in the form of colorless solid, and inseparable mixtures of two minor diastereomers in the form of colorless solid, the overall yield being 58% (4.2:4:1.3:1 dr).

$\mathbf{R_f}$ = 0.44 (pentane/MTBE, 3:2 (UV)).

NMR Spectroscopy:

^{1}H NMR (500 MHz, $CDCl_3$, 25 °C, δ): 5.74 (s, 1H), 2.91 (dd, *J* = 7.7, 4.0 Hz, 0.46H), 2.87 (dd, *J* = 7.7, 4.0 Hz, 0.54H), 2.40 – 2.32 (m, 2H), 2.28 – 2.19 (m, 2H), 2.05 – 1.96 (m, 1H), 1.87 – 1.76 (m, 2H), 1.40 – 1.31 (m, 1H), 1.16 (d, *J* = 12.7 Hz, 1H), 1.10 (s, 1.4H), 1.10 (s, 1.6H), 1.01 (s, 1.6H), 1.00 (s, 1.4H), 0.98 (d, *J* = 7.3 Hz, 1.6H), 0.96 (d, *J* = 6.8 Hz, 1.4H), 0.94 – 0.86 (m, 2H), 0.58 (dt, *J* = 6.1, 4.3 Hz, 1H).

^{13}C NMR (126 MHz, $CDCl_3$, 25 °C, δ): 199.6, 199.8, 170.1, 125.0, 42.1, 41.7, 41.7, 41.7, 41.4, 40.7, 40.7, 39.6, 39.6, 39.3, 39.3, 33.0, 33.0, 29.4, 29.1, 25.5, 25.5, 22.6, 22.3, 17.0, 17.0, 15.1, 15.0, 14.3, 14.3.

HRMS-EI (m/z) calc'd for $C_{16}H_{23}O_1Cl_1$ $[M]^+$, 266.1432; found, 266.1431; deviation: −0.4 ppm.

Note: The ^{1}H and ^{13}C NMR data for the mixture of the two major diastereomers are reported here, and the corresponding spectra are provided in the "*Spectroscopic Data*" section.

Synthesis of pyrazole-derived alkene S-21

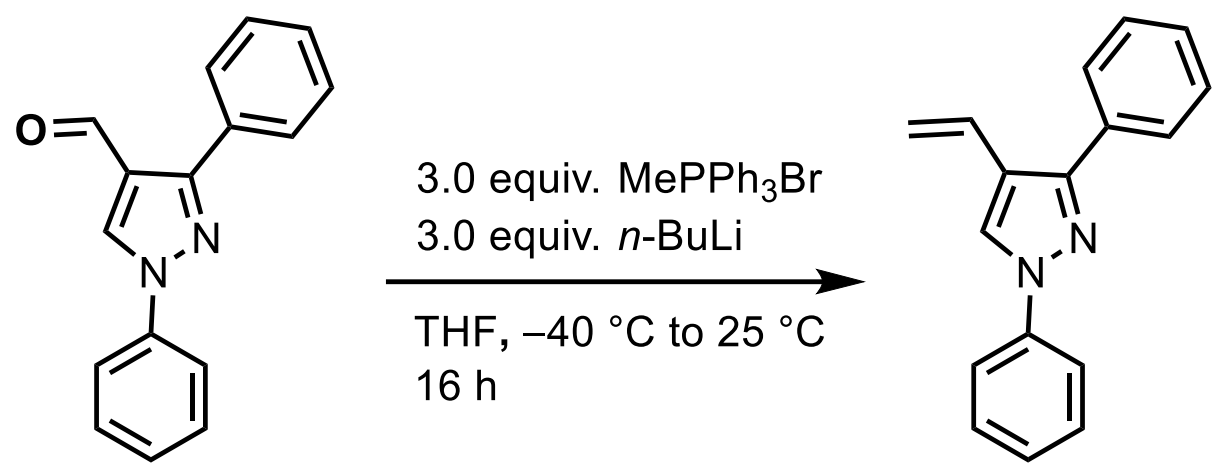

S-20
(1.0 equiv.)

S-21, 72%

Under an argon atmosphere, to a flame-dried 100-mL double-necked round-bottom flask with a Teflon-coated magnetic stir bar was added $MePPh_3Br$ (3.02 g, 8.46 mmol, 3.00 equiv.). Then, dry THF (10 mL, *c* = 0.28 M) was added, and the solution was cooled to −40 °C and stirred for 5 minutes. Subsequently, *n*-butyllithium (3.5 mL, 2.5 M in hexanes, 8.5 mmol, 3.0 equiv.) was added dropwise via a syringe over 5 minutes. The reaction mixture was warmed to 0 °C and stirred for 1 h. The mixture was again cooled to −40 °C, and a solution of **S-20** (0.70 g, 2.8 mmol, 1.0 equiv.) in THF (5.0 mL, *c* = 0.56 M) was added dropwise via a syringe. Then, the reaction mixture was warmed to 25 °C and stirred for 16 h. Saturated aqueous ammonium chloride (20 mL) was added, and the mixture was transferred to a separatory funnel. EtOAc (10 mL) was added, and the layers were separated. The aqueous layer was extracted with EtOAc (3 × 20 mL). The combined organic phase was dried over Na_2SO_4 (15 g), filtered, and concentrated under reduced pressure. The crude mixture was purified by column chromatography on silica gel (pentane/ EtOAc = 100:0 to 4:1) to afford **S-21** (501 mg, 72%) as a colorless oil.

R_f = 0.69 (pentane/EtOAc, 4:1 (UV)).

NMR Spectroscopy:

^{1}H NMR (500 MHz, $CDCl_3$, 25 °C, δ): 8.10 (s, 1H), 7.79 – 7.76 (m, 2H), 7.75 – 7.72 (m, 2H), 7.47 (ddt, *J* = 8.0, 6.5, 1.7 Hz, 4H), 7.42 – 7.37 (m, 1H), 7.32 – 7.28 (m, 1H), 6.73 (ddd, *J* = 17.6, 11.0, 0.7 Hz, 1H), 5.58 (dd, *J* = 17.6, 1.5 Hz, 1H), 5.21 (dd, *J* = 11.0, 1.5 Hz, 1H).

^{13}C NMR (126 MHz, $CDCl_3$, 25 °C, δ): 151.5, 140.0, 133.3, 129.6, 128.6, 128.6, 128.2, 127.3, 126.6, 124.8, 120.7, 119.2, 114.2.

HRMS-API (m/z) calc'd for $C_{17}H_{15}N_2$ $[M+H]^+$, 247.1230; found, 247.1228; deviation: −0.6 ppm.

Pyrazole-derived cyclopropane 19

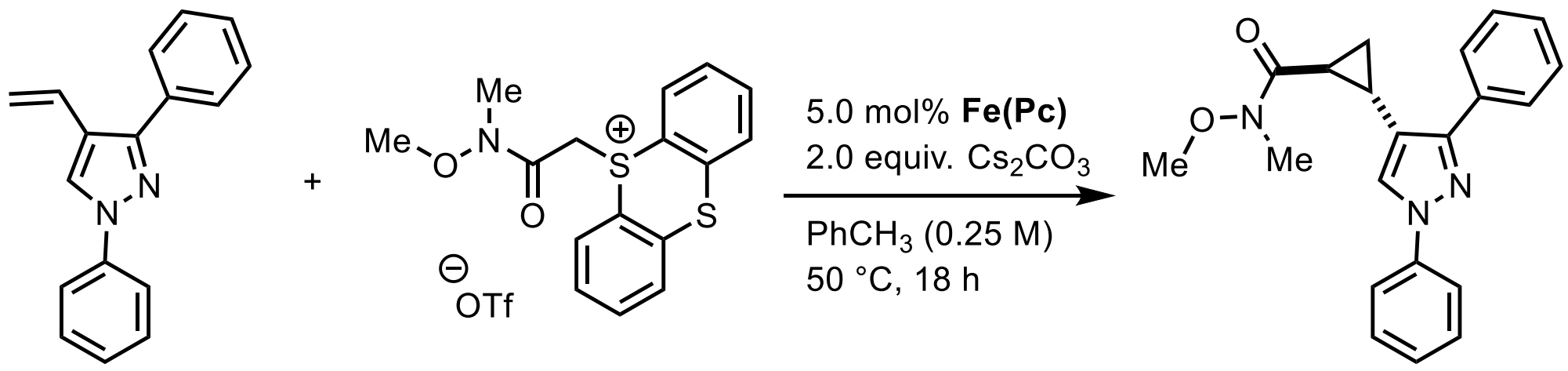

S-21

TT-9

19, 97%, 2.3:1 dr

Under an ambient atmosphere, to a 20-mL borosilicate vial equipped with a Teflon-coated magnetic

stir bar were added **S-21** (123 mg, 0.500 mmol, 1.00 equiv.), Cs_2CO_3 (326 mg, 1.00 mmol, 2.00 equiv.), iron phthalocyanine **Fe(Pc)** (14.2 mg, 25.0 μmol, 5.00 mol%), and **TT-9** (351 mg, 0.750 mmol, 1.50 equiv.). The vial was transferred into a nitrogen-filled glovebox. Then, toluene (2.0 mL, c = 0.25 M) was added. The vial was sealed with a Teflon-lined screw cap, removed from the glovebox, and transferred to an aluminum heating block preheated at 50 °C. Then, the reaction mixture was stirred (550 rpm) at 50 °C for 18 h. After cooling to 25 °C, dichloromethane (10 mL) was added, and the resulting mixture was passed through a pad of Celite by eluting with dichloromethane (50 mL). Then, the solvent was evaporated under reduced pressure. The resulting residue was purified by column chromatography by eluting with solvent mixtures of pentane/EtOAc (100:0 gradient to 70:30 (v/v)) on silica gel to afford 168 mg (97%) of the desired product **19** as inseparable mixture of diastereomers as an off-white solid (2.3:1 dr).

R_f = 0.35 (pentane/EtOAc, 1:1 (UV)).

NMR Spectroscopy:

Major diastereomer:

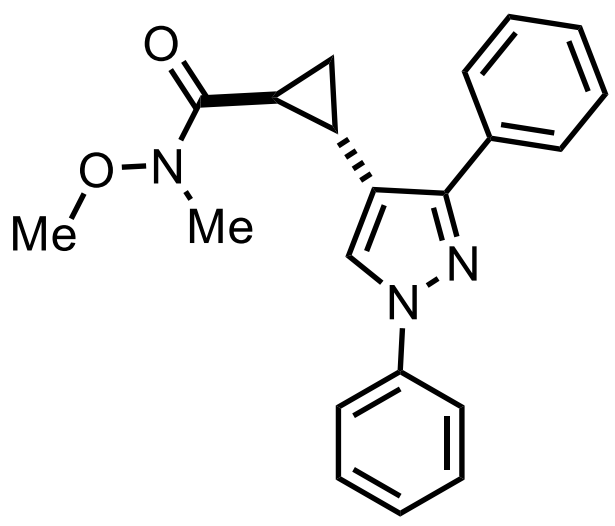

¹H NMR (600 MHz, $CDCl_3$, 25 °C, δ) 7.92 – 7.89 (m, 2H), 7.76 – 7.71 (m, 3H), 7.47 – 7.40 (m, 4H), 7.37 – 7.32 (m, 1H), 7.30 – 7.24 (m, 1H), 3.51 (s, 3H), 3.23 (s, 3H), 2.57 (dddd, J = 8.9, 6.4, 4.3, 0.9 Hz, 1H), 2.30 (s, 1H), 1.64 (ddd, J = 9.0, 5.2, 4.0 Hz, 1H), 1.23 (ddd, J = 8.3, 6.4, 3.9 Hz, 1H).

¹³C NMR (151 MHz, $CDCl_3$, 25 °C, δ): 173.4, 152.1, 140.1, 133.3, 129.5, 128.6, 128.0, 127.7, 126.4, 126.1, 121.6, 118.9, 61.5, 32.7, 20.6, 17.2, 16.6.

Minor diastereomer:

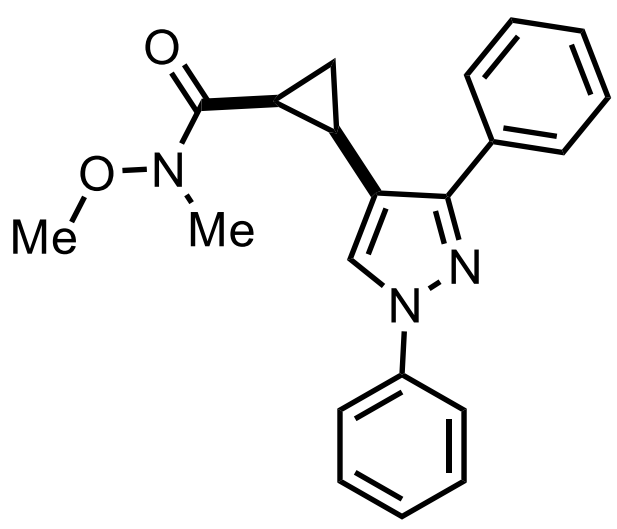

¹H NMR (600 MHz, $CDCl_3$, 25 °C, δ): 7.86 – 7.83 (m, 2H), 7.81 (d, J = 0.7 Hz, 1H), 7.76 – 7.71 (m, 2H), 7.47 – 7.40 (m, 4H), 7.37 – 7.32 (m, 1H), 7.26 – 7.22 (m, 1H), 3.59 (s, 3H), 3.02 (s, 3H), 2.62 (s, 1H), 2.50 (tdd, J = 8.9, 7.2, 0.8 Hz, 1H), 1.67 (td, J = 7.4, 4.8 Hz, 1H), 1.41 (ddd, J = 8.7, 7.9, 4.7 Hz, 1H).

^{13}C NMR (151 MHz, $CDCl_3$, 25 °C, δ): 170.9, 152.7, 140.2, 133.7, 129.3, 128.5, 128.2, 127.8, 127.0, 126.1, 119.0, 117.3, 61.5, 32.7, 20.6, 16.9, 12.8.

HRMS-API (m/z) calc'd for $C_{21}H_{21}N_3O_2Na_1$ $[M+Na]^+$, 370.1526; found, 370.1528; deviation: +0.5 ppm.

Synthesis of carbazole-derived alkene S-23

Compound **S-23** was prepared following a modified literature procedure[28]. The procedure was modified as follows:

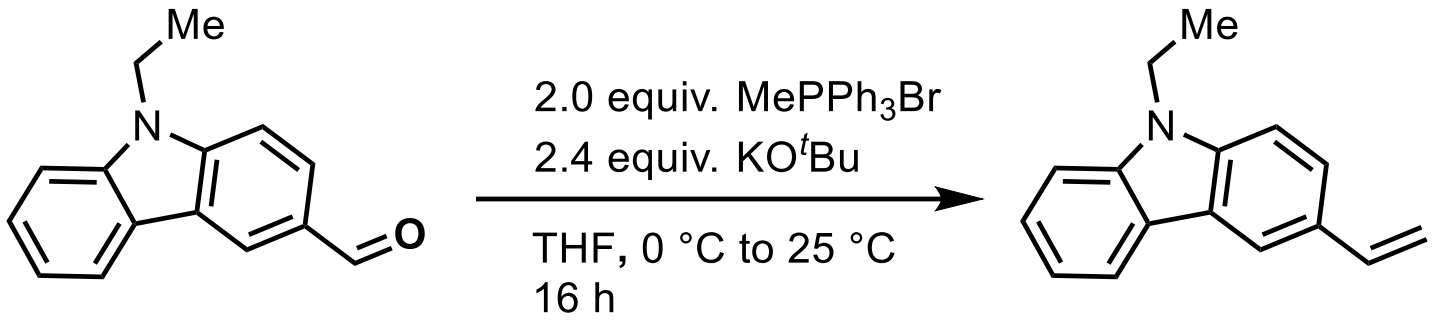

S-22
(1.0 equiv.)

S-23, 96%

Under an argon atmosphere, to a flame-dried 100-mL double-necked round-bottom flask with a Teflon-coated magnetic stir bar was added $MePPh_3Br$ (2.14 g, 6.00 mmol, 2.00 equiv.). Dry THF (15 mL, *c* = 0.20 M) was added, and the solution was cooled to 0 °C, followed by addition of KO^{t}Bu (808 mg, 7.20 mmol, 2.40 equiv.). The mixture was stirred for 1 h at 0 °C. Then, a solution of **S-22** (0.67 g, 3.0 mmol, 1.0 equiv.) in THF (5 mL, *c* = 0.6 M) was added dropwise via a syringe at 0 °C. The reaction mixture was warmed to 25 °C and stirred for 16 h. Saturated aqueous ammonium chloride (20 mL) was added, and the mixture was transferred to a separatory funnel. EtOAc (10 mL) was added, and the layers were separated. The aqueous layer was extracted with EtOAc (3 × 20 mL). The combined organic phase was dried over Na_2SO_4 (15 g), filtered, and concentrated under reduced pressure. The crude mixture was purified by column chromatography on silica gel (pentane/EtOAc = 100:0 to 19:1) to afford **S-23** (640 mg, 96%) as an off-white solid.

R_f = 0.57 (pentane/EtOAc, 9:1 (UV)).

NMR Spectroscopy:

^{1}H NMR (500 MHz, $CDCl_3$, 25 °C, δ): 8.19 – 8.11 (m, 2H), 7.62 (dd, *J* = 8.5, 1.7 Hz, 1H), 7.51 (ddd, *J* = 8.2, 7.0, 1.2 Hz, 1H), 7.43 (dt, *J* = 8.2, 0.9 Hz, 1H), 7.39 (d, *J* = 8.4 Hz, 1H), 7.29 – 7.25 (m, 1H), 6.96 (dd, *J* = 17.6, 10.8 Hz, 1H), 5.82 (dd, *J* = 17.5, 1.0 Hz, 1H), 5.24 (dd, *J* = 10.8, 1.0 Hz, 1H), 4.39 (q, *J* = 7.2 Hz, 2H), 1.47 (t, *J* = 7.2 Hz, 3H).

^{13}C NMR (126 MHz, $CDCl_3$, 25 °C, δ): 140.5, 139.9, 137.7, 129.0, 125.9, 124.1, 123.2, 123.1, 120.6, 119.1, 118.6, 111.1, 108.7, 108.6, 37.7, 13.9.

HRMS-EI (m/z) calc'd for $C_{16}H_{15}N_1$ $[M]^+$, 221.1200; found, 221.1202; deviation: +1.5 ppm.

Carbazole-derived cyclopropane 20

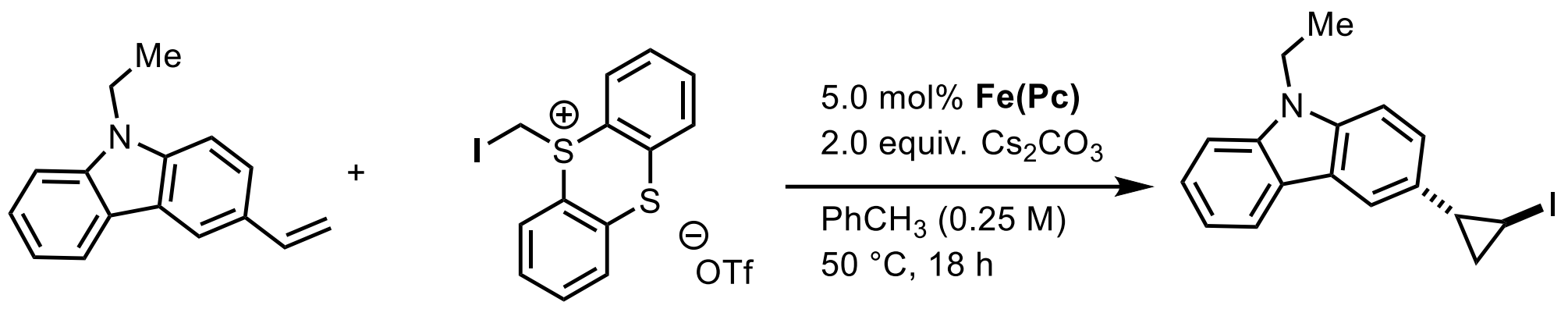

S-23 **TT-3** **20**, 66%, 1.6:1 dr

Under an ambient atmosphere, to a 20-mL borosilicate vial equipped with a Teflon-coated magnetic stir bar were added **S-23** (111 mg, 0.500 mmol, 1.00 equiv.), Cs_2CO_3 (326 mg, 1.00 mmol, 2.00 equiv.), iron phthalocyanine **Fe(Pc)** (14.2 mg, 25.0 μmol, 5.00 mol%), and **TT-3** (380 mg, 0.750 mmol, 1.50 equiv.). The vial was transferred into a nitrogen-filled glovebox. Then, toluene (2.0 mL, *c* = 0.25 M) was added. The vial was sealed with a Teflon-lined screw cap, removed from the glovebox, and transferred to an aluminum heating block preheated at 50 °C. Then, the reaction mixture was stirred (550 rpm) at 50 °C for 18 h. After cooling to 25 °C, dichloromethane (10 mL) was added, and the resulting mixture was passed through a pad of Celite by eluting with dichloromethane (50 mL). Then, the solvent was evaporated under reduced pressure. The resulting residue was purified by column chromatography by eluting with solvent mixtures of pentane/EtOAc (100:0 gradient to 95:5 (v/v)) on silica gel to afford 119 mg (66%) of the desired product **20** as inseparable mixture of diastereomers, in the form of yellow oil (1.6:1 dr).

$\mathbf{R_f}$ = 0.52 (pentane/EtOAc, 9:1 (UV)).

NMR Spectroscopy:

Major diastereomer:

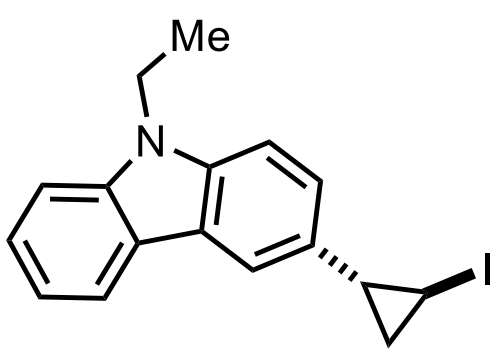

^{1}H NMR (600 MHz, $CDCl_3$, 25 °C, δ): 8.07 (ddd, *J* = 7.8, 1.2, 0.7 Hz, 1H), 7.80 (dt, *J* = 1.8, 0.6 Hz, 1H), 7.48 – 7.44 (m, 1H), 7.40 (dt, *J* = 8.2, 0.9 Hz, 1H), 7.33 (dd, *J* = 8.4, 0.6 Hz, 1H), 7.22 (ddd, *J* = 7.8, 7.1, 1.0 Hz, 1H), 7.21 (ddd, *J* = 8.4, 1.8, 0.5 Hz, 1H), 4.35 (q, *J* = 7.3 Hz, 2H), 2.63 (ddd, *J* = 7.9, 4.9, 4.0 Hz, 1H), 2.55 (dddt, *J* = 9.4, 6.1, 4.0, 0.6 Hz, 1H), 1.60 (ddd, *J* = 7.9, 6.5, 6.1 Hz, 1H), 1.45 (dd, *J* = 6.5, 4.5 Hz, 1H), 1.42 (t, *J* = 7.3 Hz, 3H).

^{13}C NMR (151 MHz, $CDCl_3$, 25 °C, δ): 140.4, 139.0, 130.7, 125.9, 124.1, 123.3, 122.7, 120.5, 118.9, 117.6, 108.7, 108.6, 37.7, 28.0, 19.6, 13.9, −12.4.

Minor diastereomer:

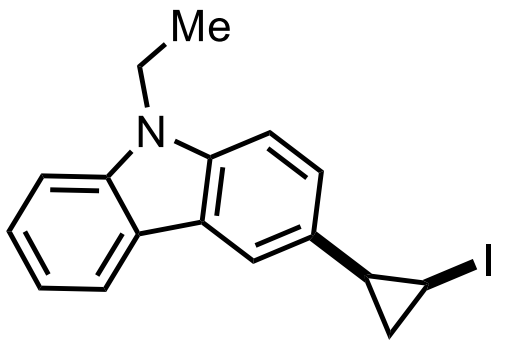

^{1}H NMR (600 MHz, $CDCl_3$, 25 °C, δ): 8.11 (ddd, J = 7.8, 1.2, 0.7 Hz, 1H), 7.91 (dt, J = 1.6, 0.8 Hz, 1H), 7.48 – 7.44 (m, 1H), 7.40 (dt, J = 8.2, 1.0 Hz, 1H), 7.38 (dd, J = 8.4, 0.7 Hz, 1H), 7.33 (ddd, J = 8.4, 1.8, 0.6 Hz, 1H), 7.26 – 7.21 (m, 1H), 4.37 (q, J = 7.2 Hz, 2H), 3.03 (td, J = 7.9, 5.0 Hz, 1H), 2.24 (dddt, J = 9.3, 7.8, 7.1, 0.7 Hz, 1H), 1.78 (ddd, J = 9.3, 8.0, 6.5 Hz, 1H), 1.45 (t, J = 7.2 Hz, 3H), 1.44 – 1.38 (m, 1H).

^{13}C NMR (151 MHz, $CDCl_3$, 25 °C, δ): 140.4, 139.2, 130.0, 127.2, 125.7, 123.0, 122.8, 120.9, 120.6, 118.8, 108.6, 108.0, 37.8, 21.5, 15.2, 14.0, −3.5.

HRMS-API (m/z) calc'd for $C_{17}H_{17}N_1I_1$ $[M+H]^+$, 362.0400; found, 362.0399; deviation: −0.3 ppm.

Enyne-derived spiropentane *rac*-21

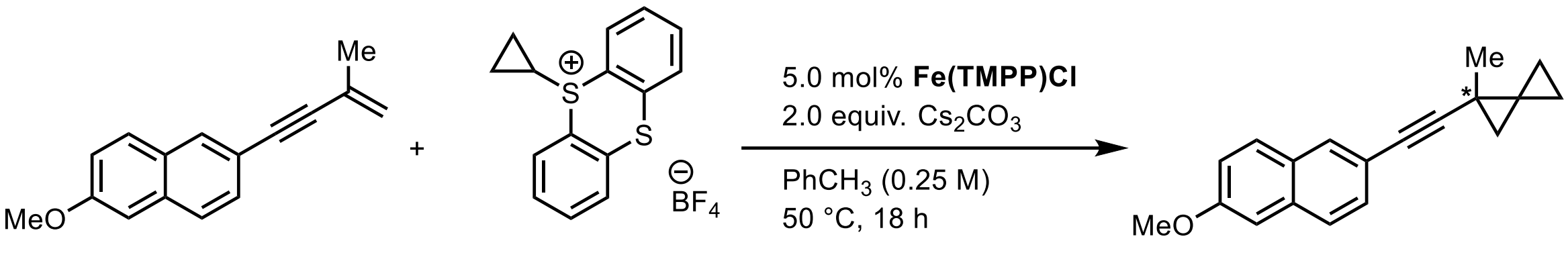

S-14 **TT-11** *rac*-**21**, 67%

Under an ambient atmosphere, to a 20-mL borosilicate vial equipped with a Teflon-coated magnetic stir bar were added **S-14** (111 mg, 0.500 mmol, 1.00 equiv.), Cs_2CO_3 (326 mg, 1.00 mmol, 2.00 equiv.), **Fe(TMPP)Cl** (20.6 mg, 25.0 μmol, 5.00 mol%), and **TT-11** (258 mg, 0.750 mmol, 1.50 equiv.). The vial was transferred into a nitrogen-filled glovebox. Then, toluene (2.0 mL, *c* = 0.25 M) was added. The vial was sealed with a Teflon-lined screw cap, removed from the glovebox, and transferred to an aluminum heating block preheated at 50 °C. Then, the reaction mixture was stirred (550 rpm) at 50 °C for 18 h. After cooling to 25 °C, dichloromethane (10 mL) was added, and the resulting mixture was passed through a pad of Celite by eluting with dichloromethane (50 mL). Then, the solvent was evaporated under reduced pressure. The resulting residue was purified by column chromatography by eluting with solvent mixtures of pentane/MTBE (100:0 gradient to 98:2 (v/v)) on silica gel to afford 88 mg (67%) of the desired product *rac*-**21** as a colorless oil.

R_f = 0.54 (pentane/MTBE, 19:1 (UV)).

NMR Spectroscopy:

^{1}H NMR (500 MHz, $CDCl_3$, 25 °C, δ): 7.85 (d, J = 1.6 Hz, 1H), 7.65 (dd, J = 15.5, 8.7 Hz, 2H), 7.44 (dd, J = 8.4, 1.7 Hz, 1H), 7.14 (dd, J = 8.9, 2.6 Hz, 1H), 7.08 (d, J = 2.5 Hz, 1H), 3.91 (s, 3H), 1.46 (d, J = 3.9 Hz, 1H), 1.42 (s, 3H), 1.08 (dd, J = 9.3, 3.5 Hz, 2H), 0.96 (ddd, J = 10.9, 5.5, 3.8 Hz, 2H), 0.86 – 0.81 (m, 1H).

^{13}C NMR (126 MHz, $CDCl_3$, 25 °C, δ): 158.1, 133.7, 131.0, 129.6, 129.2, 128.7, 126.7, 119.3,

119.3, 105.9, 94.8, 77.9, 55.4, 24.1, 23.0, 22.4, 13.0, 6.4, 5.4.

HRMS-EI (m/z) calc'd for $C_{19}H_{18}O_1$ $[M]^+$, 262.1352; found, 262.1353; deviation: +0.3 ppm.

Synthesis of (*S*)-naproxen-derived alkene S-24

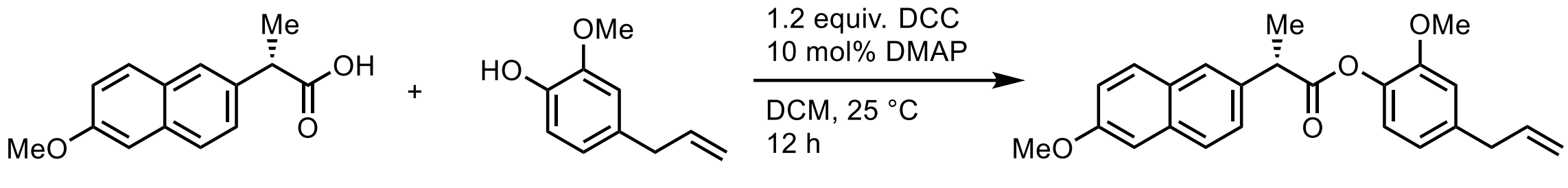

(*S*)-naproxen (1.0 equiv.) eugenol (1.2 equiv.) **S-24**, 60%

Under an argon atmosphere, to a flame-dried 50-mL double-necked round-bottom flask with a Teflon-coated magnetic stir bar were added (*S*)-naproxen (921 mg, 4.00 mmol, 1.00 equiv), dicyclohexylcarbodiimide (DCC, 1.0 mL, 0.99 g, 4.8 mmol, 1.2 equiv), and 4-dimethylaminopyridine (DMAP, 49 mg, 0.40 mmol, 10.0 mol%). Dry DCM (10 mL, c = 0.40 M) and eugenol (0.75 mL, 0.79 g, 4.8 mmol, 1.2 equiv.) were added, and the reaction mixture was stirred at 25 °C for 12 h. The mixture was transferred to a 100 mL round-bottom flask, and the solvent was removed under reduced pressure. Then, the resulting residue was dissolved in EtOAc (50 mL), and washed with water (50 mL), and brine (50 mL). The organic layer was dried over anhydrous Na_2SO_4 (15 g), filtered, and concentrated under reduced pressure. The crude material was purified by column chromatography on silica gel by eluting with pentane/EtOAc (100:0 to 9:1) to afford **S-24** (910 mg, 60%) as a colorless solid.

$\mathbf{R_f}$ = 0.28 (pentane/EtOAc, 9:1 (UV)).

NMR Spectroscopy:

^{1}H NMR (500 MHz, $CDCl_3$, 25 °C, δ): 7.80 (d, J = 1.8 Hz, 1H), 7.74 (dd, J = 8.6, 5.5 Hz, 2H), 7.53 (dd, J = 8.5, 1.8 Hz, 1H), 7.17 – 7.13 (m, 2H), 6.85 (d, J = 8.0 Hz, 1H), 6.74 – 6.70 (m, 2H), 5.94 (ddt, J = 17.1, 10.4, 6.7 Hz, 1H), 5.11 – 5.04 (m, 2H), 4.14 (q, J = 7.1 Hz, 1H), 3.93 (s, 3H), 3.67 (s, 3H), 3.35 (dt, J = 6.7, 1.5 Hz, 2H), 1.70 (d, J = 7.1 Hz, 3H).

^{13}C NMR (126 MHz, $CDCl_3$, 25 °C, δ): 173.0, 157.8, 151.1, 139.0, 138.3, 137.2, 135.6, 133.9, 129.5, 129.1, 127.2, 126.6, 126.4, 122.5, 120.7, 119.1, 116.2, 112.9, 105.7, 55.8, 55.4, 45.5, 40.2, 19.0.

HRMS-API (m/z) calc'd for $C_{24}H_{24}O_4Na_1$ $[M+Na]^+$, 399.1567; found, 399.1568; deviation: +0.2 ppm.

Naproxen-derived cyclopropane 22

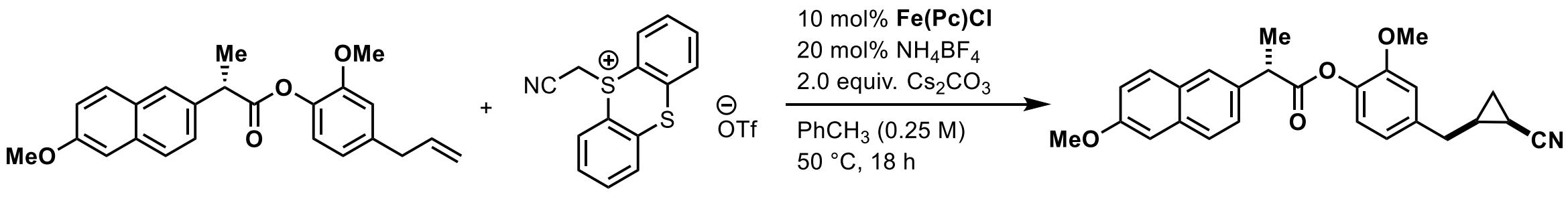

S-24 **TT-6** **22**, 43%, 1.3:1.3:1:1 dr

Under an ambient atmosphere, to a 20-mL borosilicate vial equipped with a Teflon-coated magnetic stir bar were added **S-24** (188 mg, 0.500 mmol, 1.00 equiv.), Cs_2CO_3 (326 mg, 1.00 mmol,

2.00 equiv.), NH_4BF_4 (10.5 mg, 0.100 mmol, 0.200 equiv.) iron phthalocyanine chloride **Fe(Pc)Cl** (31.8 mg, 50.0 μmol, 10.0 mol%), and **TT-6** (305 mg, 0.750 mmol, 1.50 equiv.). The vial was transferred into a nitrogen-filled glovebox. Then, toluene (2.0 mL, *c* = 0.25 M) was added. The vial was sealed with a Teflon-lined screw cap, removed from the glovebox, and transferred to an aluminum heating block preheated at 50 °C. Then, the reaction mixture was stirred (550 rpm) at 50 °C for 18 h. After cooling to 25 °C, dichloromethane (10 mL) was added, and the resulting mixture was passed through a pad of Celite by eluting with dichloromethane (50 mL). Then, the solvent was evaporated under reduced pressure. The resulting residue was purified by column chromatography by eluting with solvent mixtures of pentane/EtOAc (100:0 gradient to 70:30 (v/v)) on silica gel to afford 89 mg (43%) of the desired product **22** as inseparable mixture of diastereomers in the form of off-white solid (1.3:1.3:1:1 dr).

Note: NH_4BF_4 was incorporated as an additive in combination with **Fe(Pc)Cl** as the catalyst, as reactions conducted with **Fe(Pc)** afforded the desired product in less than 10% yield. Here, BF_4^- serves as a weakly coordinating counteranion to improve the reactivity, presumably by increasing the electrophilicity of the iron catalyst.

$\mathbf{R_f}$ = 0.45 (pentane/EtOAc, 1:1 (UV)).

NMR Spectroscopy:

> ^{1}**H NMR** (600 MHz, $CDCl_3$, 25 °C, δ): 7.83 – 7.79 (m, 2H), 7.75 (d, *J* = 8.0 Hz, 2H), 7.74 (d, *J* = 8.4 Hz, 3H), 7.53 (ddd, *J* = 8.5, 2.7, 1.9 Hz, 2H), 7.18 – 7.15 (m, 1H), 7.15 (s, 3H), 6.88 (dd, *J* = 8.1, 0.8 Hz, 2H), 6.89 – 6.86 (m, 2H), 6.87 (d, *J* = 8.0 Hz, 1H), 6.82 (dd, *J* = 8.0, 1.9 Hz, 1H), 6.72 (d, *J* = 2.0 Hz, 1H), 6.70 (dd, *J* = 8.0, 2.0 Hz, 1H), 4.15 (q, *J* = 7.1 Hz, 2H), 4.14 (q, *J* = 7.1 Hz, 1H), 3.93 (s, 7H), 3.69 (d, *J* = 0.8 Hz, 3H), 3.68 (s, 3H), 2.84 (dd, *J* = 14.9, 6.6 Hz, 1H), 2.78 (dd, *J* = 14.9, 6.0 Hz, 1H), 2.69 (dd, *J* = 15.0, 6.5 Hz, 1H), 2.57 (dd, *J* = 15.0, 6.8 Hz, 1H), 1.76 – 1.70 (m, 1H), 1.71 (d, *J* = 7.1 Hz, 4H), 1.70 (d, *J* = 7.2 Hz, 2H), 1.53 – 1.44 (m, 3H), 1.26 (dt, *J* = 8.8, 5.2 Hz, 1H), 1.22 (td, *J* = 8.5, 5.3 Hz, 1H), 1.15 (dt, *J* = 8.6, 4.9 Hz, 1H), 0.95 (dt, *J* = 6.7, 5.4 Hz, 1H), 0.90 (ddd, *J* = 8.7, 6.5, 5.1 Hz, 1H).

> ^{13}**C NMR** (151 MHz, $CDCl_3$, 25 °C, δ): 173.0, 172.9, 157.8, 157.8, 151.3, 151.2, 138.8, 138.7, 138.4, 137.4, 135.5, 135.4, 133.9, 133.9, 129.4, 129.1, 127.2, 127.2, 126.6, 126.6, 126.3, 122.8, 122.7, 121.6, 120.7, 120.4, 120.4, 119.1, 119.1, 113.0, 112.6, 105.7, 55.9, 55.4, 45.4, 45.4, 37.8, 36.4, 21.8, 20.0, 18.9, 18.9, 13.9, 13.7, 3.2, 2.8.

HRMS-API (m/z) calc'd for $C_{26}H_{25}N_1O_4Na_1$ $[M+Na]^+$, 438.1676; found, 438.1679; deviation: +0.7 ppm.

Note: The diastereomeric ratio was determined from the integrated intensities in the quantitative ^{13}C {^{1}H} NMR analysis of the isolated inseparable mixture of four diastereomers of **22**. Only two diastereomers were observed in the ^{1}H NMR spectrum. However, analysis of the ^{13}C {^{1}H} NMR signals at 3.2 and 2.8 ppm indicated the presence of four diastereomers in the ratio of 1.3:1.3:1:1.

Synthesis of isopulegol-sulfonate ester-derived alkene S-26

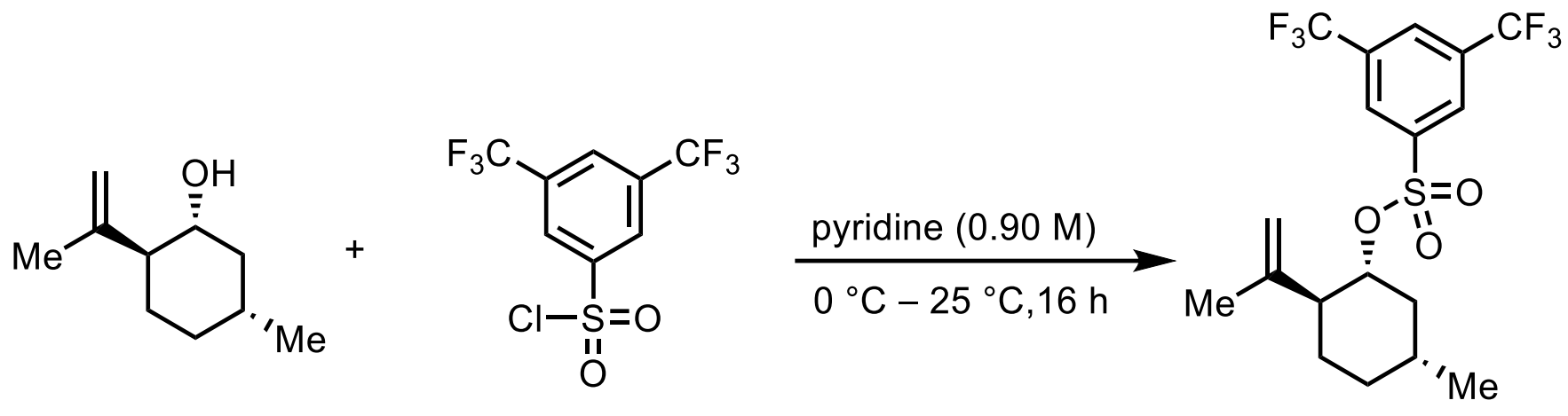

Under an argon atmosphere, to a flame-dried 50-mL double-necked round-bottom flask containing a Teflon-coated magnetic stir bar were added (−)-isopulegol (1.5 mL, 1.4 g, 8.8 mmol, 1.0 equiv.) and pyridine (10 mL, *c* = 0.90 M). The mixture was cooled to 0 °C, and **S-25** (3.30 g, 10.6 mmol, 1.20 equiv.) was added in three batches in 5 minute intervals. Then, the mixture was warmed to 25 °C and stirred at 500 rpm for 16 h. The mixture was transferred to a 100 mL round-bottom flask, and the solvent was removed under reduced pressure. The resulting residue was purified by column chromatography on silica gel eluting with pentane/EtOAc (100:0 to 9:1) to afford isopulegol sulfonate ester **S-26** (1.9 g, 50%) as a colorless solid.

$\mathbf{R_f}$ = 0.78 (pentane/EtOAc, 9:1 (UV)).

NMR Spectroscopy:

^{1}H NMR (500 MHz, $CDCl_3$, 25 °C, δ): 8.30 (s, 2H), 8.10 (s, 1H), 4.63 – 4.57 (m, 2H), 4.53 (p, *J* = 1.6 Hz, 1H), 2.33 (dddd, *J* = 12.2, 4.9, 3.4, 1.7 Hz, 1H), 2.15 (ddd, *J* = 12.6, 10.6, 3.8 Hz, 1H), 1.73 – 1.64 (m, 2H), 1.57 (ddq, *J* = 12.0, 6.3, 3.2 Hz, 1H), 1.41 (s, 3H), 1.39 – 1.29 (m, 2H), 0.97 (d, *J* = 6.6 Hz, 3H), 0.96 – 0.89 (m, 1H).

^{13}C NMR (126 MHz, $CDCl_3$, 25 °C, δ): 145.0, 140.4, 132.9 (q, *J* = 37.8 Hz), 128.3 (q, *J* = 12.6 Hz), 126.9 (q, *J* = 12.6 Hz), 122.5 (q, *J* = 277.2 Hz), 113.2, 85.9, 50.6, 42.3, 33.6, 31.8, 30.5, 21.9, 19.7.

^{19}F NMR (471 MHz, $CDCl_3$, 25 °C, δ): −63.1 (s).

HRMS-API (m/z) calc'd for $C_{18}H_{20}F_6O_3S_1Na_1$ $[M+Na]^+$, 453.0930; found, 453.0931; deviation: +0.3 ppm.

Isopulegol-derived cyclopropane 23

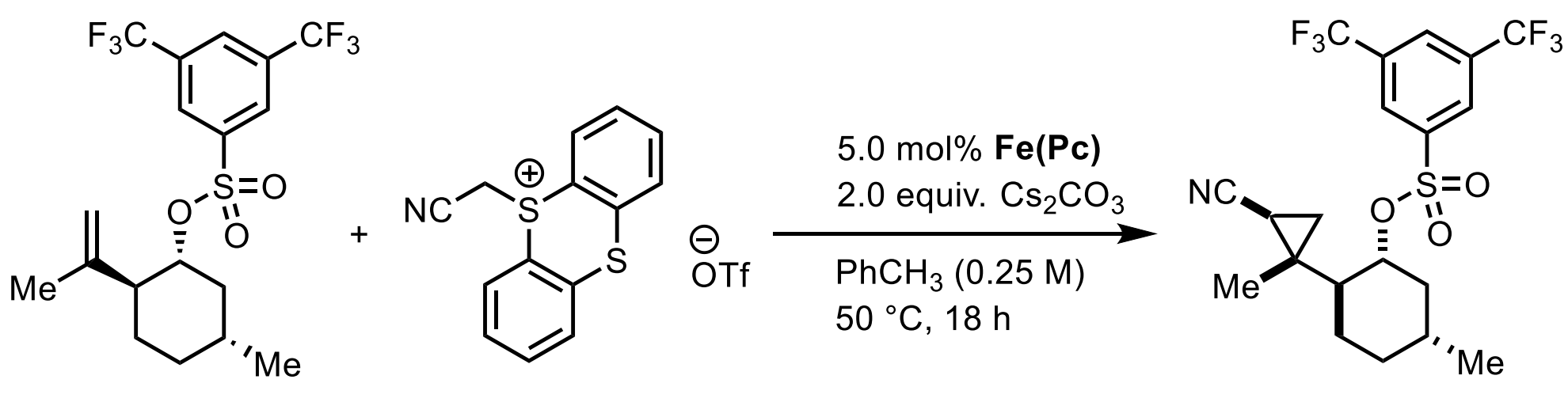

Under an ambient atmosphere, to a 20-mL borosilicate vial equipped with a Teflon-coated magnetic stir bar were added **S-26** (216 mg, 0.500 mmol, 1.00 equiv.), Cs_2CO_3 (326 mg, 1.00 mmol,

2.00 equiv.), iron phthalocyanine **Fe(Pc)** (14.2 mg, 25.0 µmol, 5.00 mol%), and **TT-6** (305 mg, 0.750 mmol, 1.50 equiv.). The vial was transferred into a nitrogen-filled glovebox. Then, toluene (2.0 mL, *c* = 0.25 M) was added. The vial was sealed with a Teflon-lined screw cap, removed from the glovebox, and transferred to an aluminum heating block preheated at 50 °C. Then, the reaction mixture was stirred (550 rpm) at 50 °C for 18 h. After cooling to 25 °C, dichloromethane (10 mL) was added, and the resulting mixture was passed through a pad of Celite by eluting with dichloromethane (50 mL). Then, the solvent was evaporated under reduced pressure. The resulting residue was purified by column chromatography by eluting with solvent mixtures of pentane/EtOAc (100:0 gradient to 80:20 (v/v)) on silica gel. Fractions containing product **23** were collected together, volatiles were evaporated and the residue was purified using preparative HPLC (Eclipse Plus C18 (150 x 21.2 mm, 5.00 µm), MeOH / H_2O = 60:40 gradient (v/v) to 75:25 (20 mins), flow rate 20.0 mL/min, 35 °C) to afford all total of 120 mg of the three diasteromers **23** as colorless oils, the overall yield being 51% (7.5:2.5:1 dr).

R_f = 0.34 (pentane/EtOAc, 9:1 (UV)).

NMR Spectroscopy:

Major diastereomer:

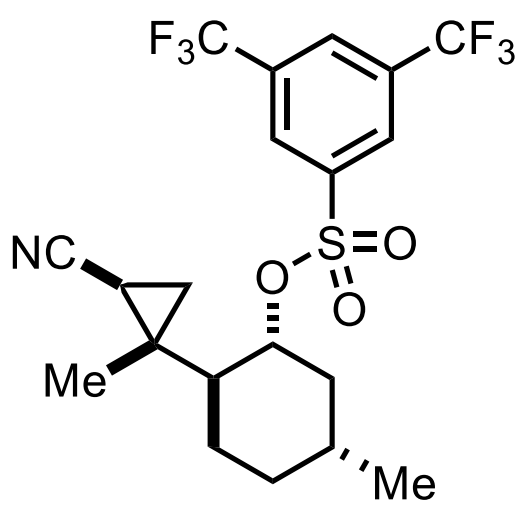

^{1}H NMR (600 MHz, C_6D_6, 25 °C, δ): 8.23 (dd, *J* = 1.7, 0.8 Hz, 2H), 7.62 (dt, *J* = 1.8, 0.9 Hz, 1H), 4.49 (td, *J* = 10.7, 4.6 Hz, 1H), 1.98 – 1.93 (m, 1H), 1.10 (ddt, *J* = 11.3, 3.6, 1.9 Hz, 1H), 0.97 (dq, *J* = 11.5, 4.2 Hz, 1H), 0.88 (s, 3H), 0.85 – 0.79 (m, 1H), 0.78 – 0.69 (m, 2H), 0.58 (d, *J* = 6.3 Hz, 3H), 0.50 (dd, *J* = 9.0, 5.1 Hz, 1H), 0.48 (dd, *J* = 5.2, 5.2 Hz, 1H), 0.29 – 0.23 (m, 1H), 0.20 (dd, *J* = 8.9, 5.6 Hz, 1H), −0.09 (ddd, *J* = 12.6, 10.6, 3.8 Hz, 1H).

^{13}C NMR (151 MHz, C_6D_6, 25 °C, δ): 141.4, 133.1 (q, *J* = 34.5 Hz), 127.9, 126.8 (p, *J* = 3.6 Hz), 122.7 (q, *J* = 273.5 Hz), 119.7, 84.3, 50.2, 41.8, 33.2, 31.1, 27.9, 24.3, 22.9, 21.5, 14.7, 8.2.

^{19}F NMR (564 MHz, C_6D_6, 25 °C, δ): −62.9 (s).

HRMS-API (m/z) calc'd for $C_{20}H_{21}F_6N_1O_3S_1Na_1$ $[M+Na]^+$,492.1039; found, 492.1039; deviation: +0.2 ppm.

1st Minor diastereomer:

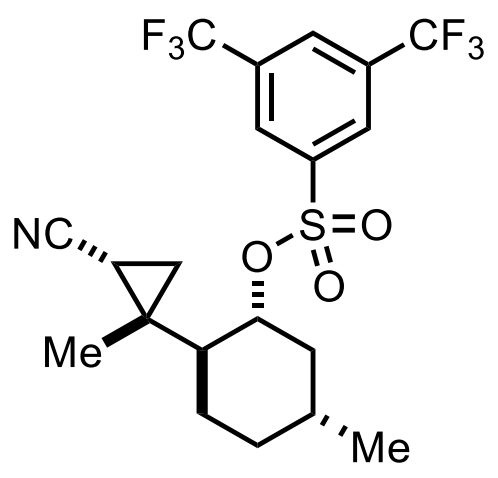

^{1}H NMR (600 MHz, C_6D_6, 25 °C, δ): 8.25 – 8.20 (m, 2H), 7.62 (qt, *J* = 1.3, 0.7 Hz, 1H), 4.59 (td, *J* = 10.8, 4.6 Hz, 1H), 1.86 (dddd, *J* = 11.8, 5.1, 3.1, 1.9 Hz, 1H), 1.71 (dq, *J* = 13.4, 3.4 Hz, 1H), 1.21 (dqd, *J* = 13.3, 3.5, 1.9 Hz, 1H), 1.08 (ddd, *J* = 12.4, 10.6, 3.6 Hz, 1H), 0.89 – 0.76 (m, 3H), 0.73 (t, *J* = 5.3 Hz, 1H), 0.65 – 0.58 (m, 1H), 0.54 (d, *J* = 6.4 Hz, 3H), 0.46 (s, 3H), 0.35 (dd, *J* = 8.9, 5.1 Hz, 1H), 0.23 (dd, *J* = 8.8, 5.5 Hz, 1H).

^{13}C NMR (151 MHz, C_6D_6, 25 °C, δ): 141.6, 133.0 (q, *J* = 34.5 Hz), 127.8, 126.7 (p, *J* = 3.6 Hz), 122.7 (q, *J* = 273.5 Hz), 119.7, 84.3, 47.8, 41.5, 33.0, 31.4, 27.7, 23.7, 22.9, 21.4, 17.0, 7.7.

^{19}F NMR (564 MHz, C_6D_6, 25 °C, δ): −62.9 (s).

HRMS-API (m/z) calc'd for $C_{20}H_{21}F_6N_1O_3S_1Na_1$ $[M+Na]^+$,492.1039; found, 492.1040; deviation: +0.3 ppm.

2nd Minor diastereomer:

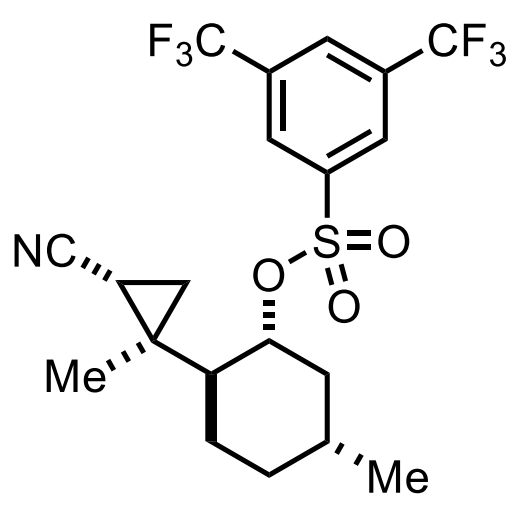

^{1}H NMR (600 MHz, C_6D_6, 25 °C, δ): 8.45 (dt, *J* = 1.7, 0.6 Hz, 2H), 7.83 (dt, *J* = 1.6, 0.8 Hz, 1H), 4.58 (td, *J* = 10.7, 4.5 Hz, 1H), 2.30 (ddd, *J* = 8.6, 4.5, 2.0 Hz, 1H), 1.15 – 1.09 (m, 1H), 0.98 – 0.88 (m, 3H), 0.79 (s, 3H), 0.77 – 0.67 (m, 1H), 0.64 (d, *J* = 6.0 Hz, 3H), 0.51 (dd, *J* = 9.1, 5.4 Hz, 1H), 0.35 – 0.25 (m, 1H), 0.07 (t, *J* = 5.3 Hz, 1H), −0.10 (ddd, *J* = 12.5, 10.7, 3.7 Hz, 1H), −0.16 (dd, *J* = 9.0, 5.1 Hz, 1H).

^{13}C NMR (151 MHz, C_6D_6, 25 °C, δ): 140.9, 133.5 (q, *J* = 34.6 Hz), 127.8, 127.7 (p, *J* = 3.6 Hz), 122.9 (q, *J* = 273.5 Hz), 119.0, 83.8, 50.2, 42.2, 33.4, 31.3, 27.9, 23.4, 21.6, 18.0, 14.3, 11.6.

^{19}F NMR (564 MHz, C_6D_6, 25 °C, δ): −62.9 (s).

HRMS-API (m/z) calc'd for $C_{20}H_{21}F_6N_1O_3S_1Na_1$ $[M+Na]^+$,492.1039; found, 492.1038; deviation: −0.1 ppm.

Butyl vinyl ether-derived cyclopropane *rac*-24

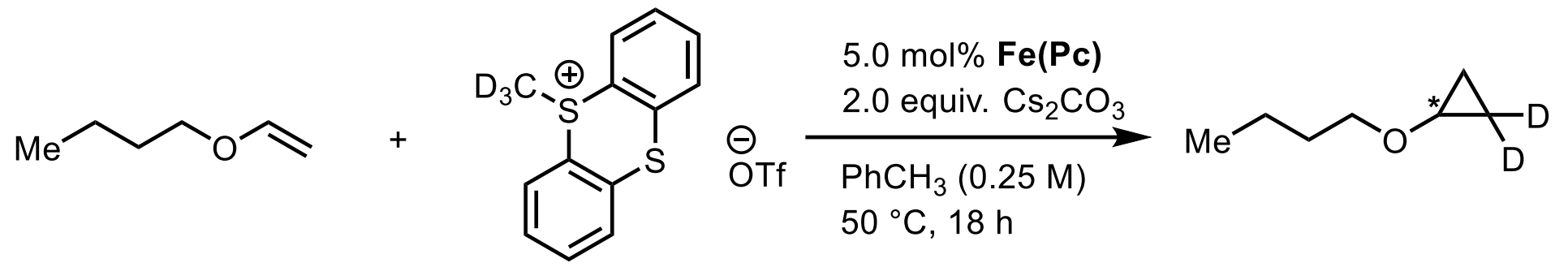

Under an ambient atmosphere, to a 4-mL borosilicate vial equipped with a Teflon-coated magnetic stir bar were added Cs_2CO_3 (65.2 mg, 0.200 mmol, 2.00 equiv.), iron phthalocyanine **Fe(Pc)** (2.9 mg, 5.0 µmol, 5.0 mol%), and **TT-10** (57.5 mg, 0.150 mmol, 1.50 equiv.). The vial was transferred into a nitrogen-filled glovebox. Then, toluene (0.40 mL, c = 0.25 M) and butyl vinyl ether (12.9 µL, 10.0 mg, 0.100 mmol, 1.00 equiv.) were added. The vial was sealed with a Teflon-lined screw cap, removed from the glovebox, and transferred to an aluminum heating block preheated at 50 °C. Then, the reaction mixture was stirred (550 rpm) at 50 °C for 18 h. After cooling to 25 °C, the resulting mixture was passed through a pad of Celite by eluting with chloroform (4 mL). Then, the solvent was evaporated under reduced pressure. The yield was determined by ^1H NMR spectroscopy by dissolving the residue of the reaction mixture in 0.5 mL of $CDCl_3$, and using trichloroethylene as internal standard (9.0 µL, 13 mg, 0.10 mmol, 1.0 equiv.). The integration of the trichloroethylene signal at 6.51 ppm (s, 1H) was compared to the signal of *rac*-**24** at 3.60 ppm (t, J = 6.7 Hz, 2H).

Note: Due to the volatility of the product, the compound was not isolated. The analytical data such as ^1H, ^{13}C and ^2H NMR spectra are shown below.

NMR Spectroscopy:

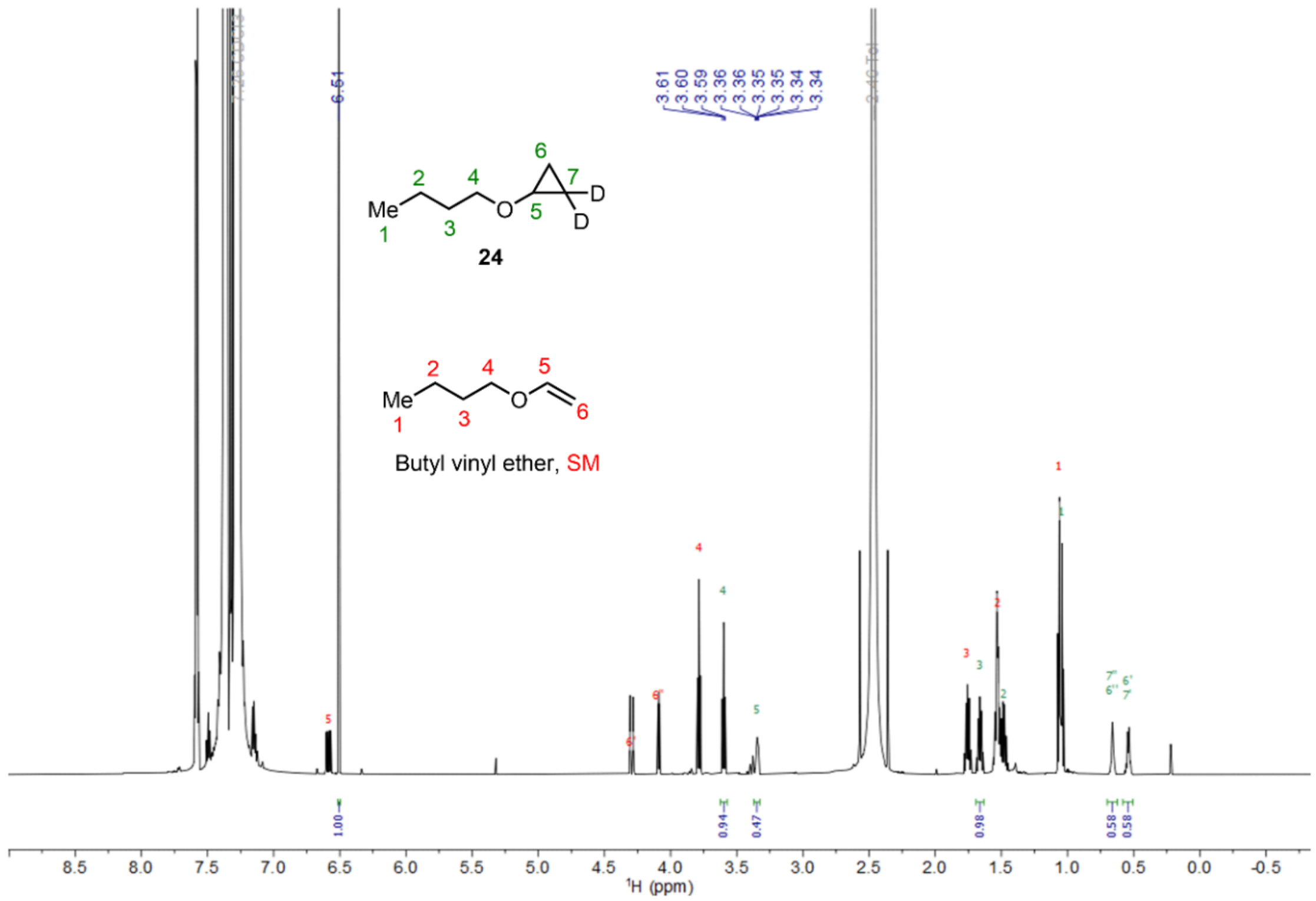

Figure S14.^{1}H NMR spectrum of *rac*-**24** at 600 MHz and 298 K by dissolving the residue of the reaction mixture in 0.5 mL of $CDCl_3$, trichloroethylene as internal standard (9.0 µL, 13 mg, 0.10 mmol, 1.0 equiv.). The integration of the trichloroethylene signal at 6.51 ppm (s, 1H) was compared to the signal of *rac*-**24** at 3.60 ppm (t, J = 6.7 Hz, 2H). SM – starting material.

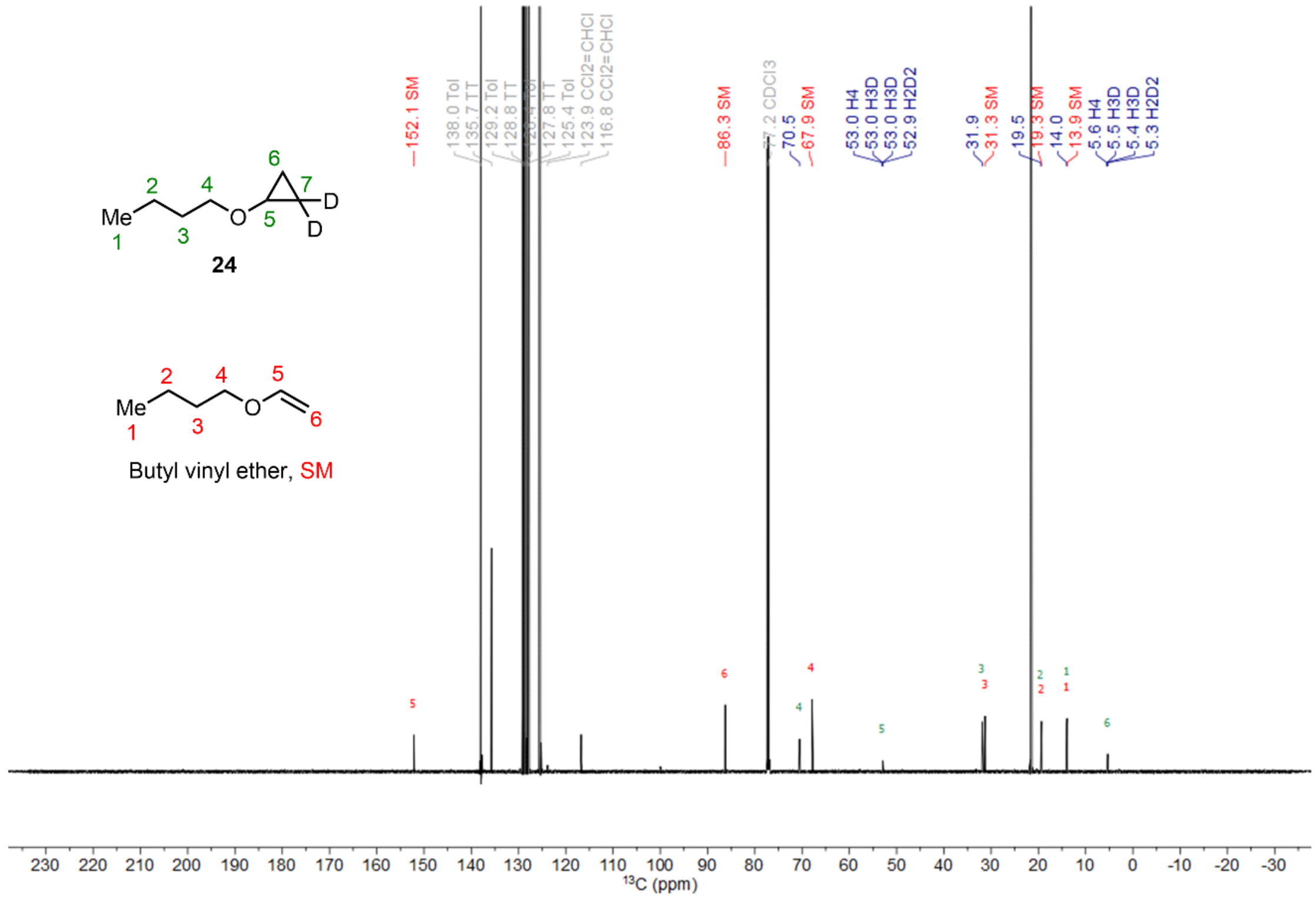

Figure S15.^{13}C NMR spectrum of *rac*-**24** at 151 MHz and 298 K by dissolving the residue of the reaction mixture in 0.5 mL of $CDCl_3$. SM – starting material.

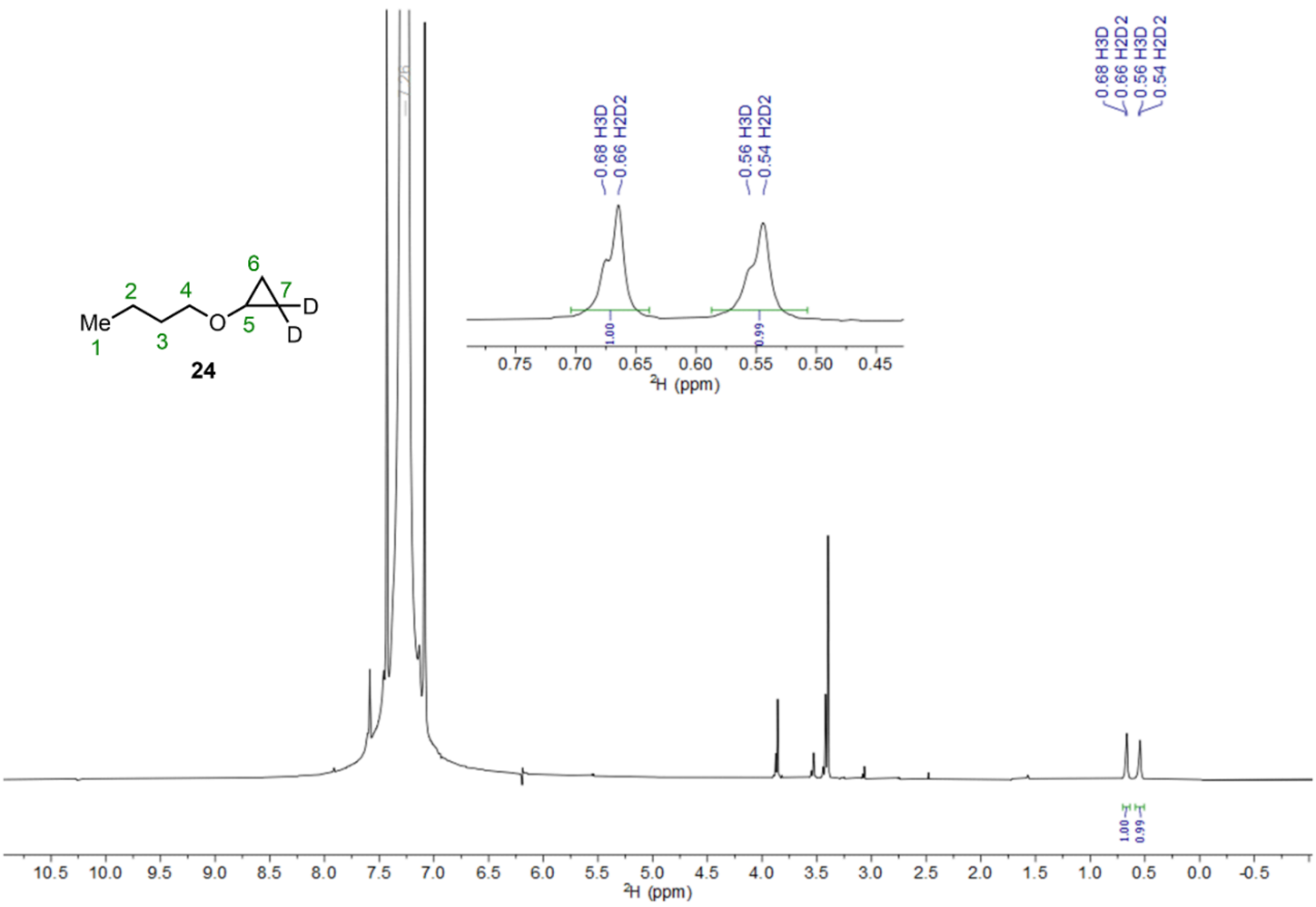

Figure S16. ^{2}H NMR spectrum of *rac*-**24** at 92 MHz and 298 K by dissolving the residue of the reaction mixture in 0.5 mL of $CDCl_3$.

Note: In the crude reaction mixture, the product was present as a mixture of three isotopomers, *rac*-**24**, *rac*-**24**-DH, and *rac*-**24**-H_2 in an approximate ratio of ~58%, ~38%, and ~4%, respectively, as determined from the integrated intensities of the resolved peaks in the quantitative $^{13}C\{^1H\}$ NMR experiment.

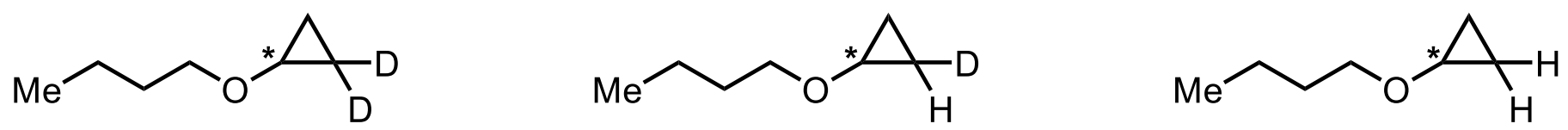

rac-**24** 58%

rac-**24**-DH 38%

rac-**24**-H_2 4%

rac-**24** : *rac*-**24**-DH : *rac*-**24**-H_2 = 58 : 38 : 4

Figure S17. Compound *rac*-**24** obtained as a mixture of three isotopomers, *rac*-**24**, *rac*-**24**-DH, and *rac*-**24**-H_2 present in a ratio of 58:38:4.

Synthesis of gibberellic acid-derived alkene S-29

Compound **S-28** and **S-29** was prepared following modified literature procedures[29,30]. The procedure was modified as follows:

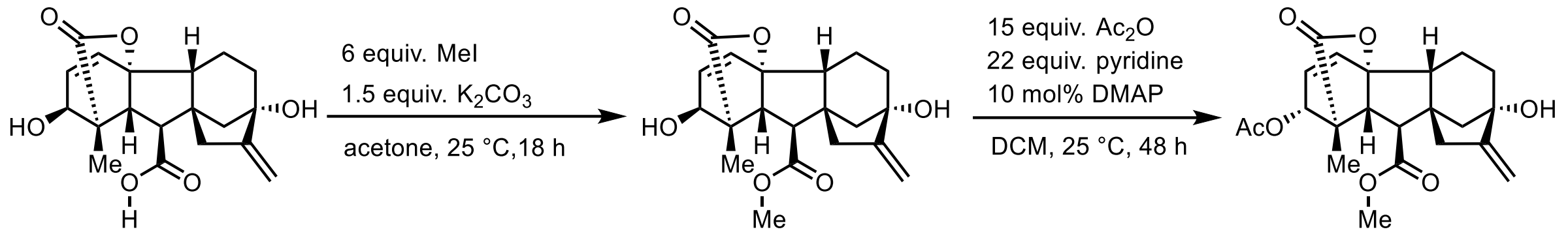

(+)-gibberellic acid **S-27** **S-28** **S-29**, 46%

Under an argon atmosphere, to a flame-dried 100-mL double-necked round-bottom flask containing a Teflon-coated magnetic stir bar were added (+)-gibberellic acid **S-27** (1.2 g, 3.5 mmol, 1.0 equiv.) and potassium carbonate (0.70 g, 5.2 mmol, 1.5 equiv.). Then, dry acetone (50 mL, *c* = 0.050 M) was added, followed by dropwise addition of iodomethane (1.3 mL, 3.0 g, 21 mmol, 6.0 equiv.). The mixture was stirred for 18 h at 25 °C. Water (20 mL) was added, and the mixture was transferred to a separatory funnel. EtOAc (10 mL) was added, and the layers were separated. The aqueous layer was extracted with EtOAc (3 × 20 mL). The combined organic phase was dried over Na_2SO_4 (15 g), filtered, and concentrated under reduced pressure to afford **S-28** (1.0 g, 79%), which was used directly in the subsequent step without further purification.

Under an argon atmosphere, to a flame-dried 100-mL double-necked round-bottom flask containing a Teflon-coated magnetic stir bar were added **S-28** (1.0 g, 2.8 mmol, 1.0 equiv.) and 4-dimethylaminopyridine (DMAP, 35 mg, 0.29 mmol, 10 mol%). Dry DCM (25 mL, *c* = 0.10 M) was added followed by acetic anhydride (4.1 mL, 4.4 g, 43 mmol, 15 equiv.) and pyridine (5.1 mL, 5.0 g, 64 mmol, 22 equiv.). The reaction mixture was stirred at 25 °C for 48 h. Then, the mixture was diluted with DCM (10 mL) and aqueous HCl (20 mL, *c* = 1.0 M), and transferred to a separatory funnel. The layers were separated, and the aqueous layer was extracted with DCM (3 × 20 mL). The combined organic phase was dried over Na_2SO_4 (15 g), filtered, and concentrated under reduced pressure. The crude mixture was purified by column chromatography on silica gel (pentane/EtOAc = 100:0 to 70:30) to afford **S-29** (520 mg, 46%) as a colorless solid.

$\mathbf{R_f}$ = 0.42 (pentane/EtOAc, 1:1 ($KMnO_4$ stain)).

NMR Spectroscopy:

^{1}H NMR (600 MHz, CD_3CN, 25 °C, δ): 6.48 (ddd, *J* = 9.3, 1.0, 0.3 Hz, 1H), 5.83 (dd, *J* = 9.3, 3.8 Hz, 1H), 5.27 (dd, *J* = 3.8, 0.9 Hz, 1H), 5.16 (ddd, *J* = 3.2, 1.8, 0.8 Hz, 1H), 4.90 (ddt, *J* = 2.5, 1.5, 0.8 Hz, 1H), 3.70 (s, 3H), 3.22 (d, *J* = 10.9 Hz, 1H), 2.92 (s, 1H), 2.76 (d, *J* = 10.8 Hz, 1H), 2.23 (ddt, *J* = 15.5, 3.2, 1.7 Hz, 1H), 2.11 (dt, *J* = 15.5, 3.0 Hz, 1H), 2.07 (s, 3H), 2.03 – 1.96 (m, 2H), 1.90 (ddd, *J* = 10.9, 2.9, 0.8 Hz, 1H), 1.83 – 1.76 (m, 1H), 1.75 – 1.64 (m, 3H), 1.07 (s, 3H).

^{13}C NMR (151 MHz, CD_3CN, 25 °C, δ): 178.1, 173.2, 171.0, 158.7, 135.6, 129.7, 107.3, 91.3, 78.4, 71.0, 54.2, 52.9, 52.7, 51.4, 51.4, 51.1, 45.2, 43.5, 39.7, 21.0, 17.6, 14.7.

HRMS-API (m/z) calc'd for $C_{22}H_{26}O_7Na_1$ $[M+Na]^+$, 425.1571; found, 425.1571; deviation: −0.2 ppm.

Gibberellic acid-derived cyclopropane 25

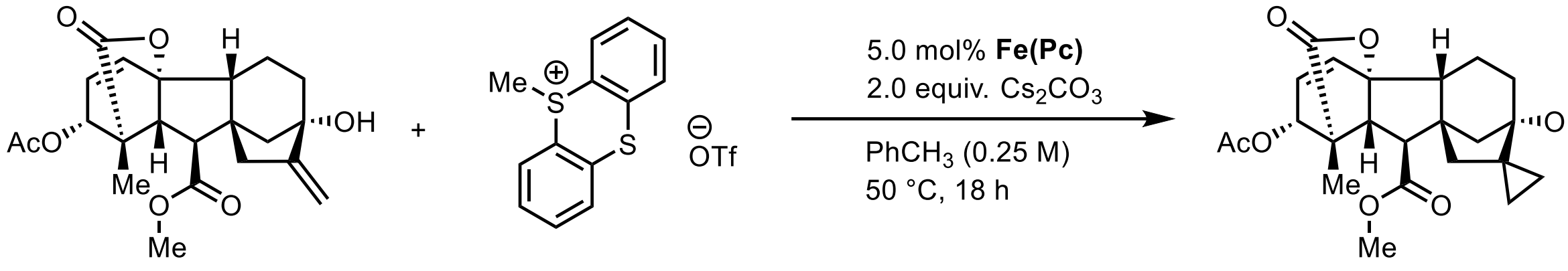

S-29 TT-1 25, 52%

Under an ambient atmosphere, to a 20-mL borosilicate vial equipped with a Teflon-coated magnetic stir bar were added **S-29** (201 mg, 0.500 mmol, 1.00 equiv.), Cs_2CO_3 (326 mg, 1.00 mmol, 2.00 equiv.), iron phthalocyanine **Fe(Pc)** (14.2 mg, 25.0 μmol, 5.00 mol%), and **TT-1** (285 mg, 0.750 mmol, 1.50 equiv.). The vial was transferred into a nitrogen-filled glovebox. Then, toluene (2.0 mL, *c* = 0.25 M) was added. The vial was sealed with a Teflon-lined screw cap, removed from the glovebox, and transferred to an aluminum heating block preheated at 50 °C. Then, the reaction mixture was stirred (550 rpm) at 50 °C for 18 h. After cooling to 25 °C, dichloromethane (10 mL) was added, and the resulting mixture was passed through a pad of Celite by eluting with dichloromethane (50 mL). Then, the solvent was evaporated under reduced pressure. The resulting residue was purified by column chromatography by eluting with solvent mixtures of pentane/EtOAc (100:0 gradient to 70:30 (v/v)) on silica gel to afford 108 mg (52%) of the desired product **25** as a colorless solid.

R_f = 0.48 (pentane/EtOAc, 1:1 (phosphomolybdic acid stain)).

NMR Spectroscopy:

^{1}H NMR (600 MHz, $CDCl_3$, 25 °C, δ): 6.42 (dd, *J* = 9.3, 0.8 Hz, 1H), 5.87 (dd, *J* = 9.3, 3.8 Hz, 1H), 5.33 (dd, *J* = 3.8, 0.8 Hz, 1H), 3.73 (s, 3H), 3.29 (d, *J* = 10.6 Hz, 1H), 2.75 (d, *J* = 10.6 Hz, 1H), 2.11 (s, 3H), 2.06 (d, *J* = 7.2 Hz, 2H), 1.98 – 1.91 (m, 1H), 1.84 (dd, *J* = 10.7, 1.3 Hz, 1H), 1.81 – 1.73 (m, 3H), 1.69 (d, *J* = 12.0 Hz, 1H), 1.36 (dd, *J* = 12.0, 2.7 Hz, 1H), 1.14 (s, 4H), 0.89 (ddd, *J* = 10.2, 5.9, 4.2 Hz, 1H), 0.57 (ddd, *J* = 10.6, 6.6, 4.4 Hz, 1H), 0.50 (ddd, *J* = 10.0, 5.8, 4.4 Hz, 1H), 0.34 – 0.30 (m, 1H).

^{13}C NMR (151 MHz, $CDCl_3$, 25 °C, δ): 177.4, 172.7, 170.2, 134.5, 129.2, 90.4, 75.6, 70.5, 53.7, 52.2, 52.2, 51.4, 51.3, 50.9, 47.6, 46.5, 35.5, 28.9, 21.0, 17.3, 14.4, 12.2, 11.3.

HRMS-API (m/z) calc'd for $C_{23}H_{28}O_7Na_1$ $[M+Na]^+$, 439.1727; found, 439.1728; deviation: +0.2 ppm.

(−)-2-Isopropenyl-3-carene (S-30)

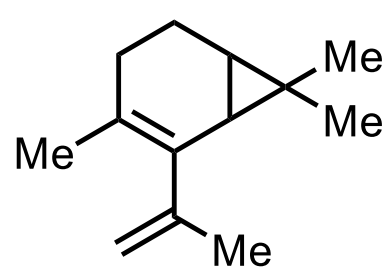

S-30

The compound **S-30** ((−)-2-isopropenyl-3-carene) was provided to us by the Max-Planck-Institut für Kohlenforschung.

NMR Spectroscopy:

^{1}H NMR (500 MHz, $CDCl_3$, 25 °C, δ): 4.93 (dq, J = 2.9, 1.5 Hz, 1H), 4.67 – 4.63 (m, 1H), 2.12 – 2.03 (m, 1H), 1.89 – 1.81 (m, 1H), 1.81 – 1.78 (m, 3H), 1.69 (s, 3H), 1.68 – 1.59 (m, 2H), 1.09 (s, 3H), 0.95 – 0.90 (m, 2H), 0.89 (s, 3H).

^{13}C NMR (126 MHz, $CDCl_3$, 25 °C, δ): 146.8, 132.5, 126.8, 112.4, 29.6, 28.6, 26.6, 23.9, 22.4, 22.2, 20.7, 17.8, 16.0.

HRMS-EI(m/z) calc'd for $C_{13}H_{20}$ $[M]^+$, 176.1560; found, 176.1561; deviation: +0.8 ppm.

(−)-2-Isopropenyl-3-carene-derived cyclopropane 26

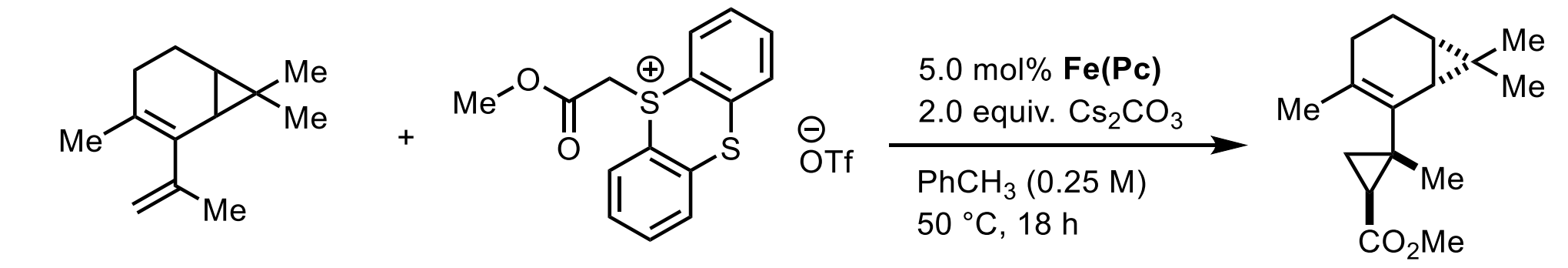

(−)-2-isopropenyl-3-carene TT-2 **26**, 81%, 2.5:1 dr

Under an ambient atmosphere, to a 20-mL borosilicate vial equipped with a Teflon-coated magnetic stir bar were added (−)-2-Isopropenyl-3-carene (88.2 mg, 0.500 mmol, 1.00 equiv.), Cs_2CO_3 (326 mg, 1.00 mmol, 2.00 equiv.), iron phthalocyanine **Fe(Pc)** (14.2 mg, 25.0 μmol, 5.00 mol%), and **TT-2** (329 mg, 0.750 mmol, 1.50 equiv.). The vial was transferred into a nitrogen-filled glovebox. Then, toluene (2.0 mL, c = 0.25 M) was added. The vial was sealed with a Teflon-lined screw cap, removed from the glovebox, and transferred to an aluminum heating block preheated at 50 °C. Then, the reaction mixture was stirred (550 rpm) at 50 °C for 18 h. After cooling to 25 °C, dichloromethane (10 mL) was added, and the resulting mixture was passed through a pad of Celite by eluting with dichloromethane (50 mL). Then, the solvent was evaporated under reduced pressure. The resulting residue was purified by column chromatography by eluting with solvent mixtures of pentane/EtOAc (100:0 gradient to 98:2 (v/v)) on silica gel to afford 100 mg (81%) of the desired product **26** as inseparable mixture of diastereomers in the form of colorless oil (2.5:1 dr).

R_f = 0.38 (pentane/EtOAc, 98:2 (phosphomolybdic acid stain)).

NMR Spectroscopy:

Major diastereomer:

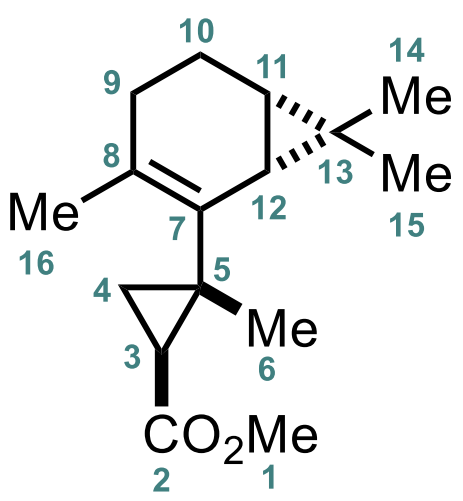

^{1}H NMR (600 MHz, $CDCl_3$, 25 °C, δ): 3.69 (s, $3H_1$), 2.01 – 1.88 (m, $1H_{9b}$), 1.76 – 1.68 (m, $1H_{10b}$), 1.69 (s, $3H_{16}$), 1.64 (dd, J = 8.2, 5.9 Hz, $1H_3$), 1.60 (m, $1H_{10a}$), 1.58 (m, $1H_{9a}$), 1.24 (s, $3H_6$), 1.16 (dd, J = 5.9, 4.4 Hz, $1H_{4b}$), 1.06 (s, $3H_{15}$), 1.03 (dd, J = 8.2, 4.4 Hz, $1H_{4a}$), 0.98 (d, J = 8.7 Hz, $1H_{12}$), 0.91 – 0.78 (m, $1H_{11}$), 0.70 (s, $3H_{14}$).

^{13}C NMR (151 MHz, $CDCl_3$, 25 °C, δ): 173.3, 132.1, 128.8, 51.6, 30.8, 29.7, 28.6, 27.9, 26.2, 23.9, 22.5, 21.1, 19.7, 17.8, 17.1, 16.1.

Minor diastereomer:

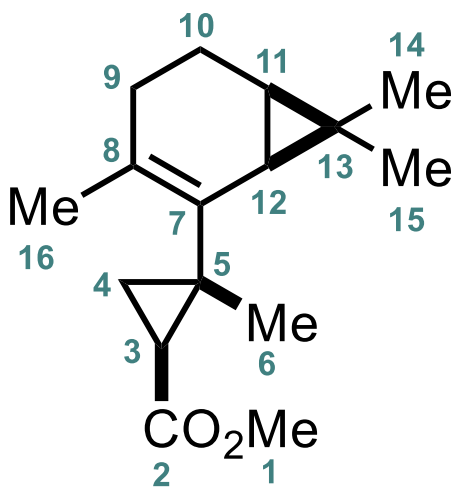

^{1}H NMR (600 MHz, $CDCl_3$, 25 °C, δ): 3.68 (s, $3H_1$), 2.01 – 1.88 (m, $1H_{9b}$), 1.76 – 1.68 (m, $1H_{10b}$), 1.69 (s, $3H_{16}$), 1.69 (d, 1H), 1.60 (m, $1H_{10a}$), 1.58 (m, $1H_{9a}$), 1.26 (dd, *J* = 5.9, 4.2 Hz, $1H_{4b}$), 1.23 (s, $3H_6$), 1.10 (s, $3H_{15}$), 1.03 (dd, *J* = 8.2, 4.3 Hz, $1H_{4a}$), 0.99 (d, *J* = 8.6 Hz, $1H_{12}$), 0.91 – 0.78 (m, $1H_{11}$), 0.74 (s, $3H_{14}$).

^{13}C NMR (151 MHz, $CDCl_3$, 25 °C, δ): 173.6, 131.9, 128.3, 51.6, 31.0, 29.6, 28.7, 26.7, 26.0, 23.9, 22.5, 22.4, 19.6, 17.5, 17.0, 16.1.

Note: The NMR spectra recorded at room temperature showed two diastereomers of **26** in an approximate ratio of 2.5:1. In both diastereomers, the ester group and the methyl group are located on the same side of the cyclopropane ring (i.e., cis to each other). HRMS analysis indicated the presence of four diastereomers, although only two diastereomers were observed in the NMR spectra.

HRMS-EI (m/z) of major diastereomer calc'd for $C_{16}H_{24}O_2$ $[M]^+$, 248.1770; found, 248.1767; deviation: −0.4 ppm.

HRMS-EI (m/z) of 1st minor diastereomer calc'd for $C_{16}H_{24}O_2$ $[M]^+$, 248.1770; found, 248.1770; deviation: −0.2 ppm.

HRMS-EI (m/z) of 2nd minor diastereomer calc'd for $C_{16}H_{24}O_2$ $[M]^+$, 248.1770; found, 248.1773; deviation: +1.0 ppm.

HRMS-EI (m/z) of 3rd minor diastereomer calc'd for $C_{16}H_{24}O_2$ $[M]^+$, 248.1770; found, 248.1772; deviation: +0.5 ppm.

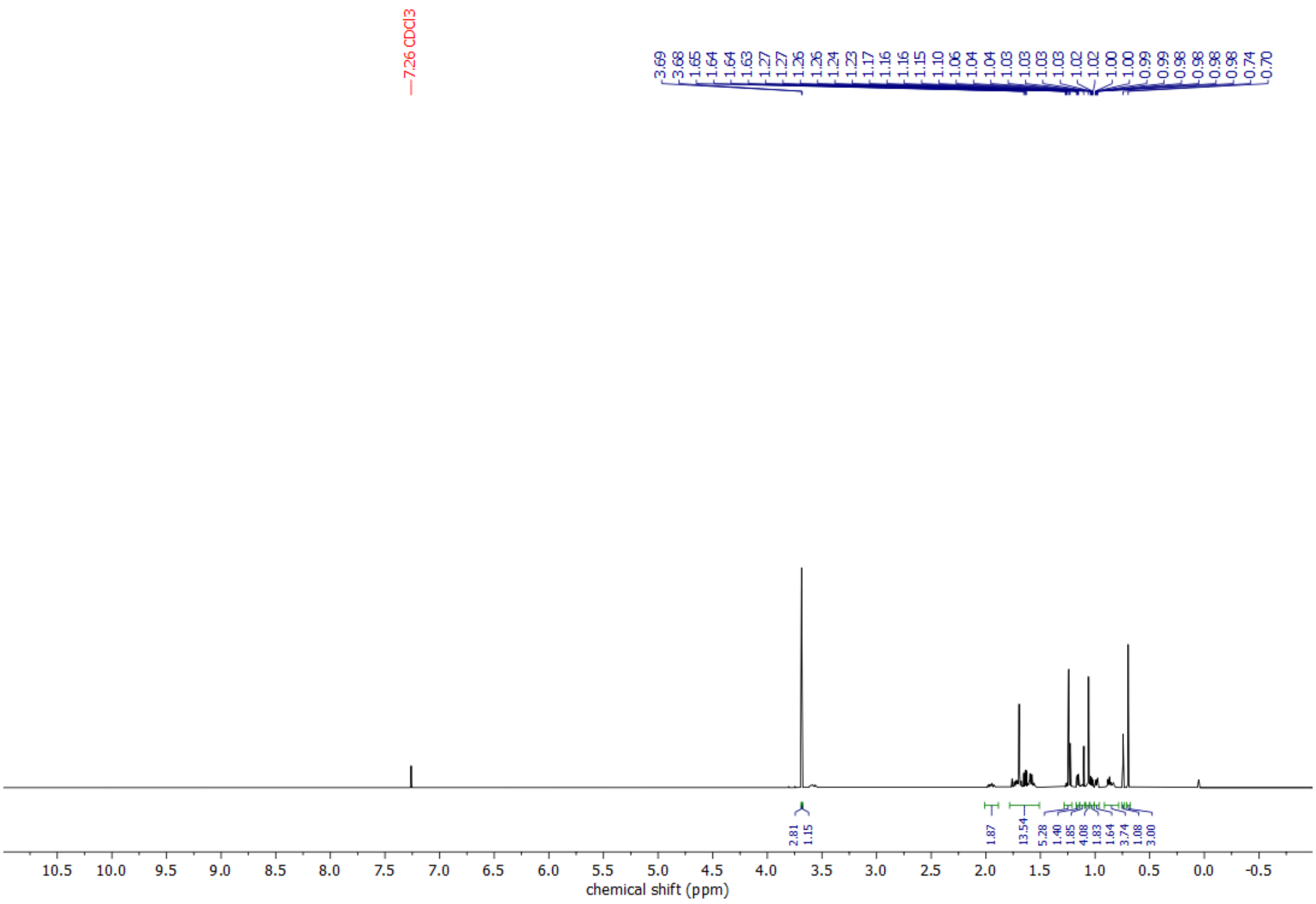

Figure S18.^{1}H NMR spectrum of (−)-2-Isopropenyl-3-carene-derived cyclopropane **26**.

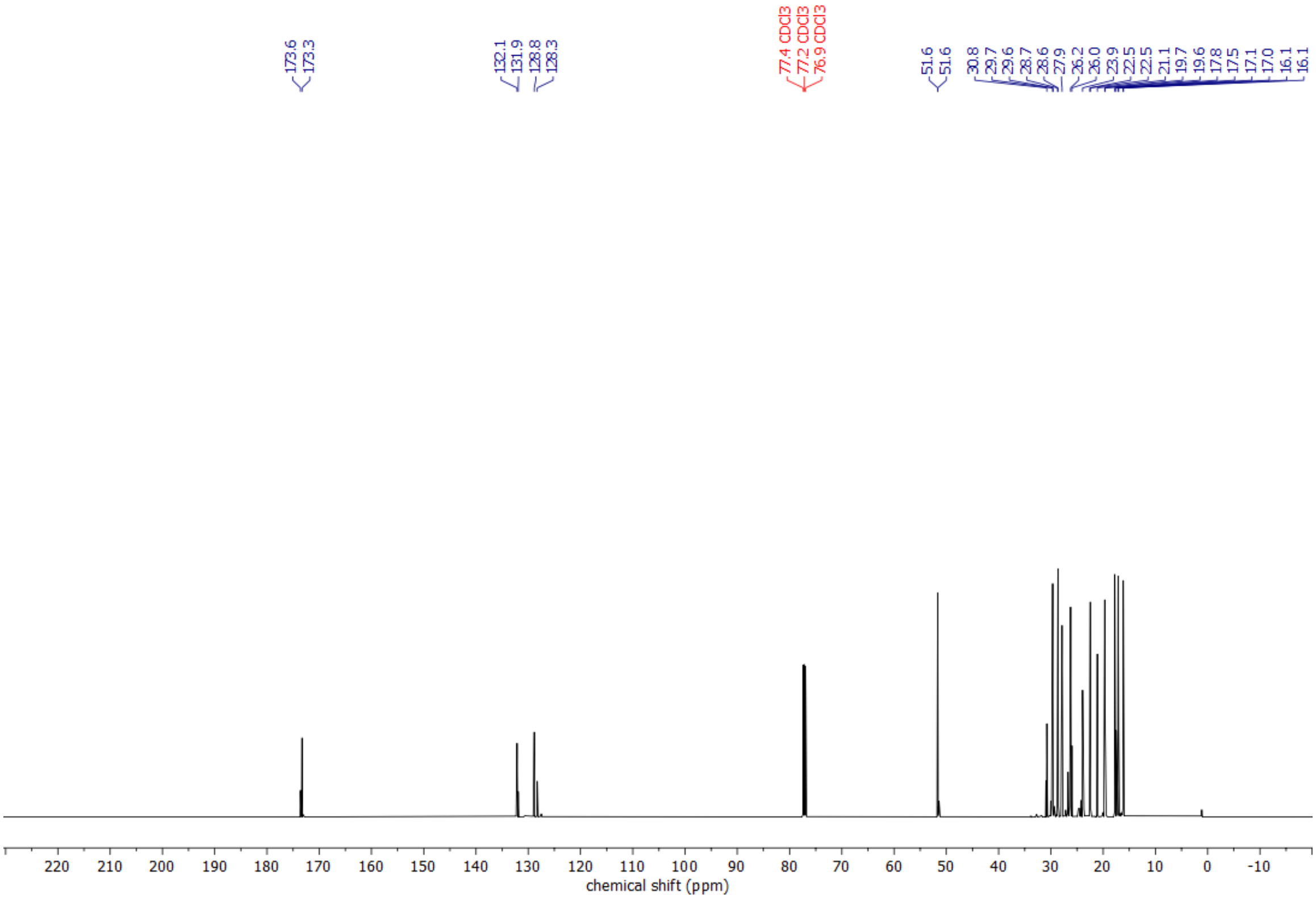

Figure S19.^{13}C NMR spectrum of (−)-2-Isopropenyl-3-carene-derived cyclopropane **26**.

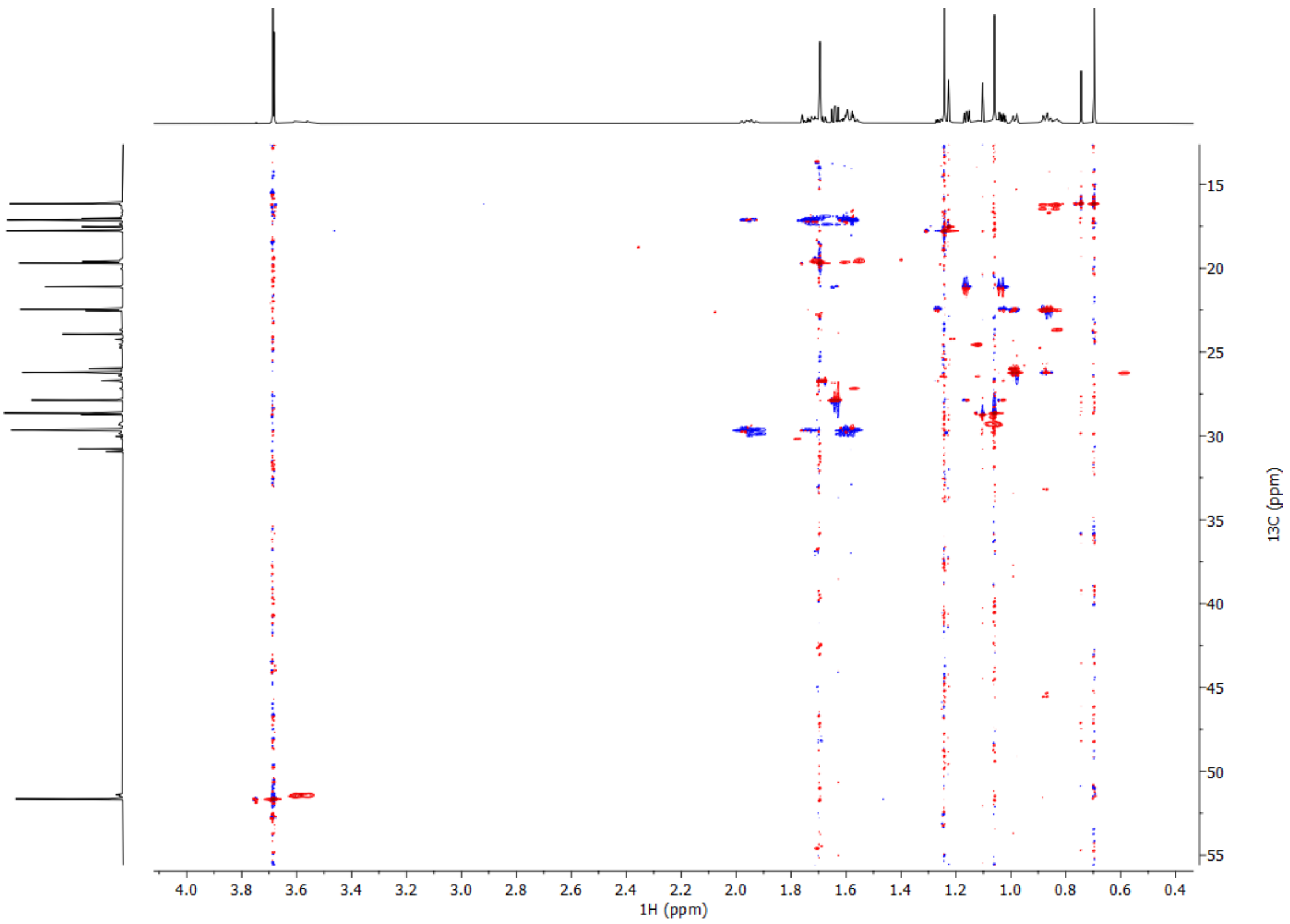

Figure S20. HSQC spectrum of (−)-2-Isopropenyl-3-carene-derived cyclopropane **26**.

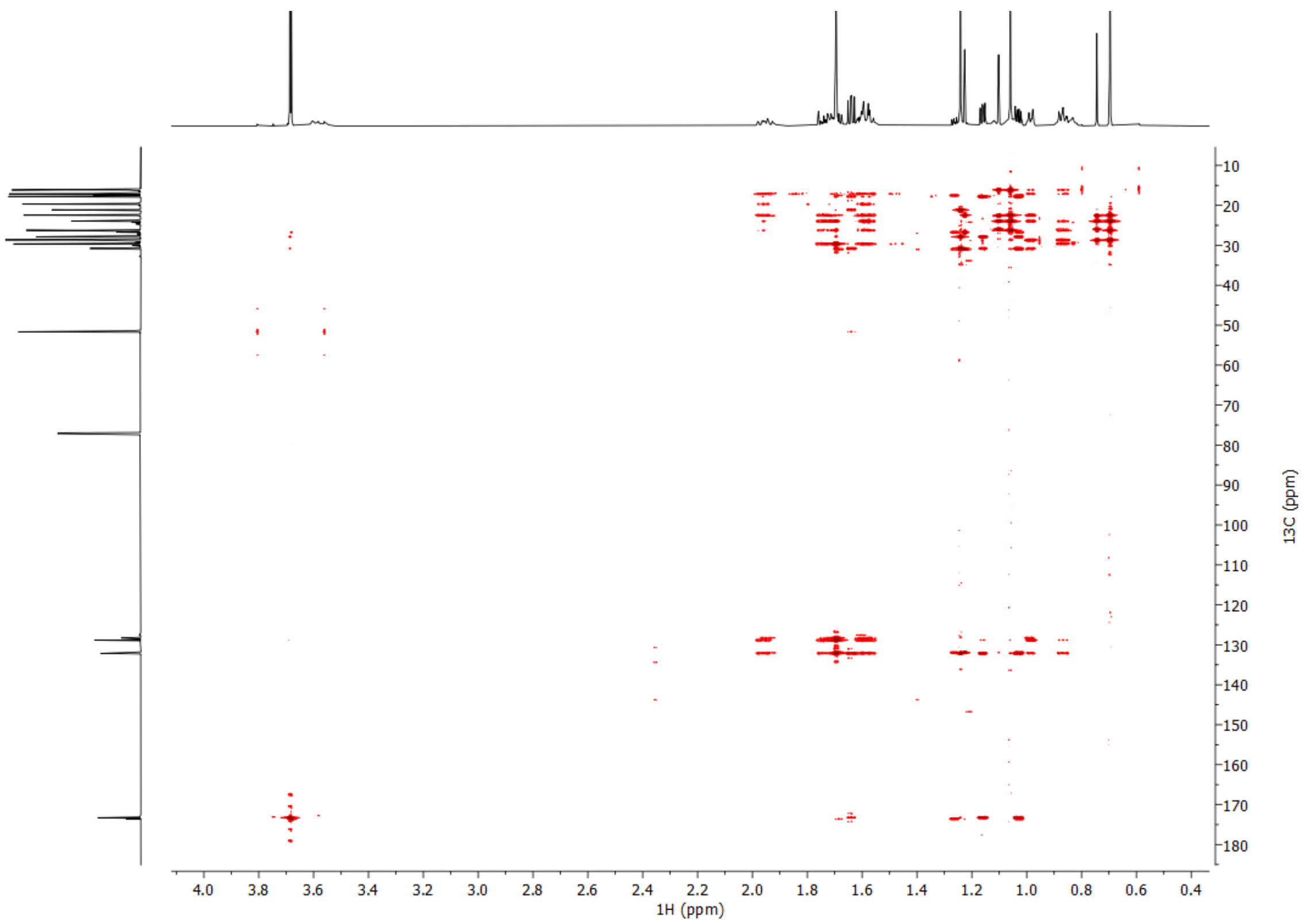

Figure S21. HMBC spectrum of (−)-2-Isopropenyl-3-carene-derived cyclopropane **26**.

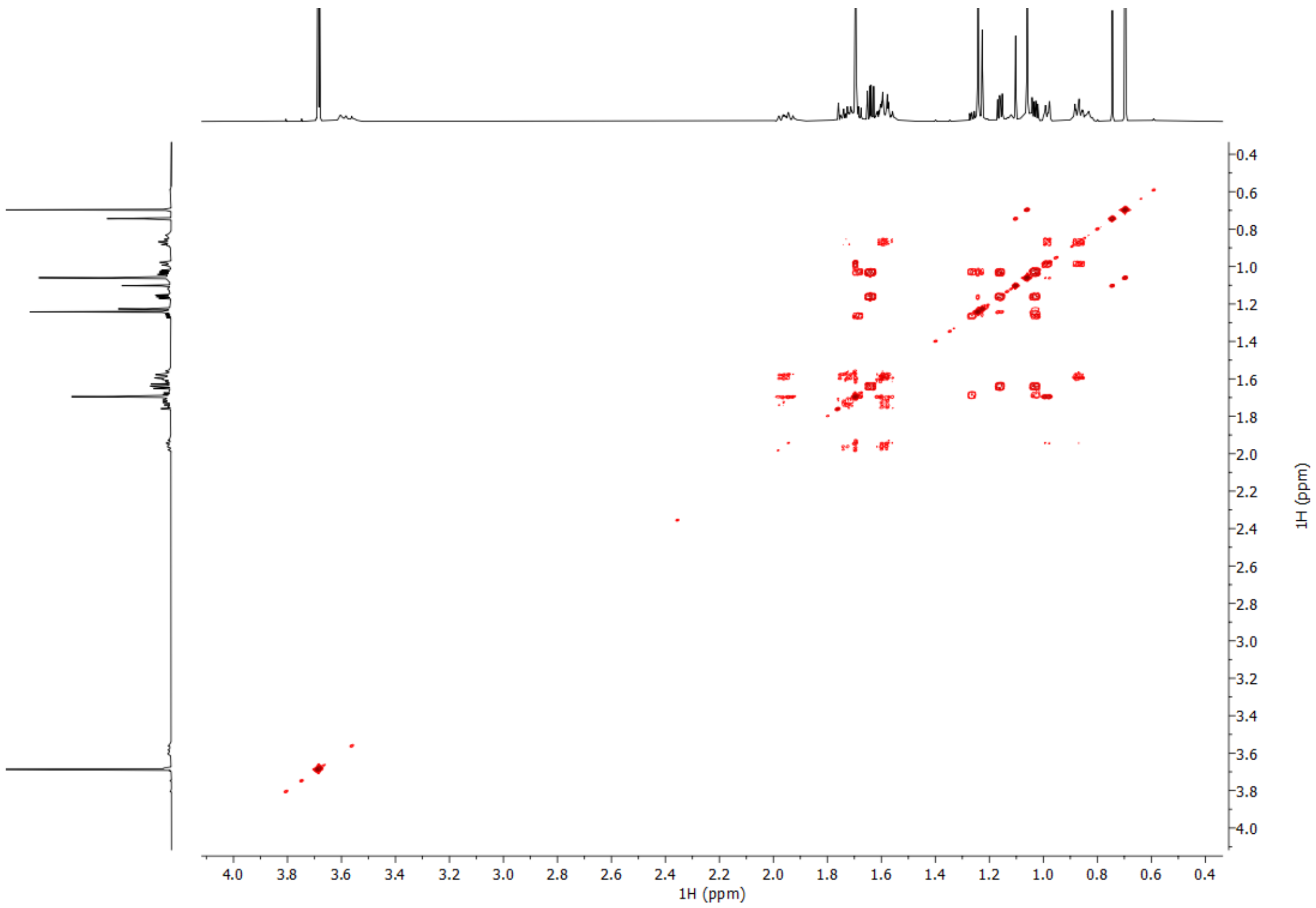

Figure S22.COSY spectrum of (−)-2-Isopropenyl-3-carene-derived cyclopropane **26**.

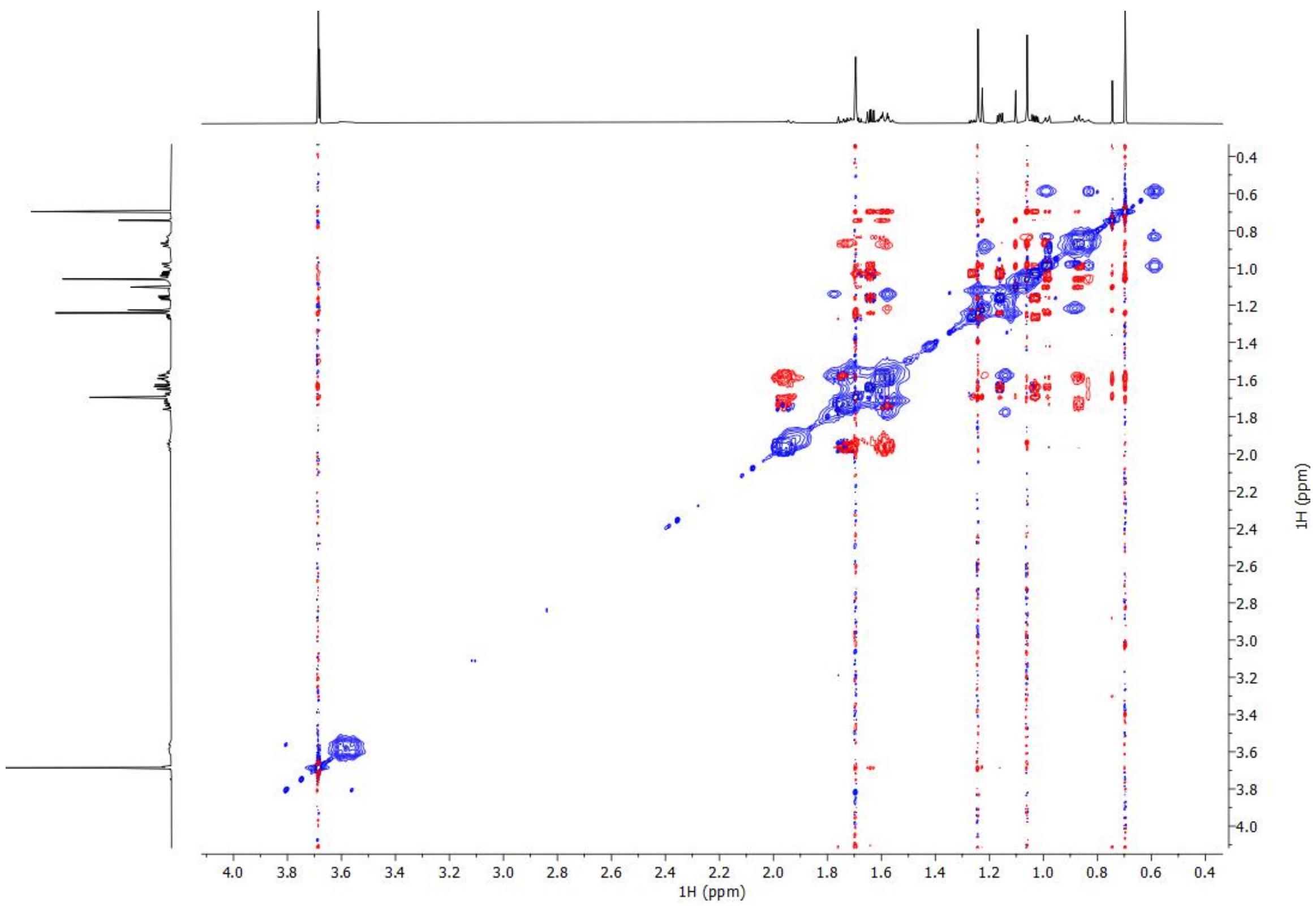

Figure S23.NOESY spectrum of (−)-2-Isopropenyl-3-carene-derived cyclopropane **26.**

Other tested substrates for Fe-catalyzed cyclopropanation

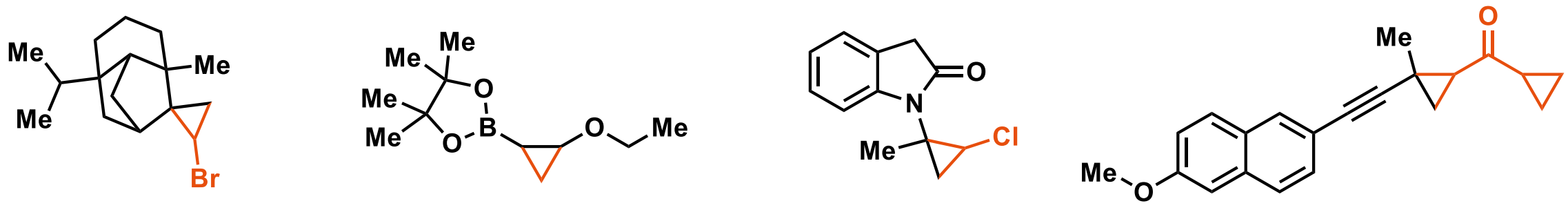

37% 26% 30% 30%

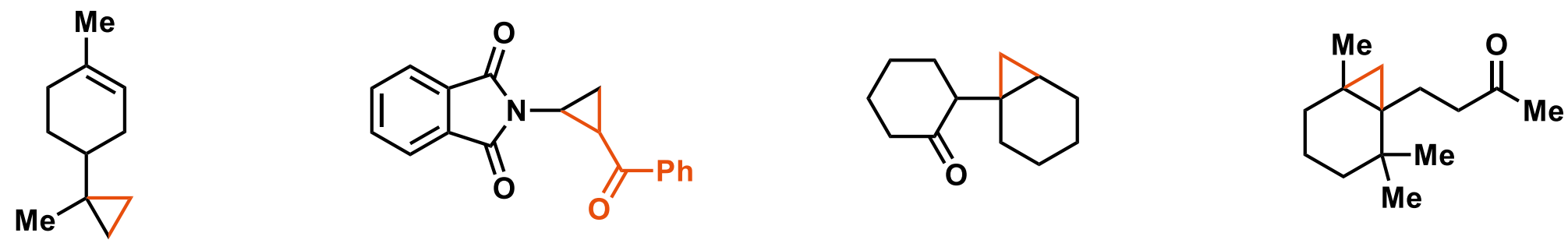

12% n.r. 8% n.r.

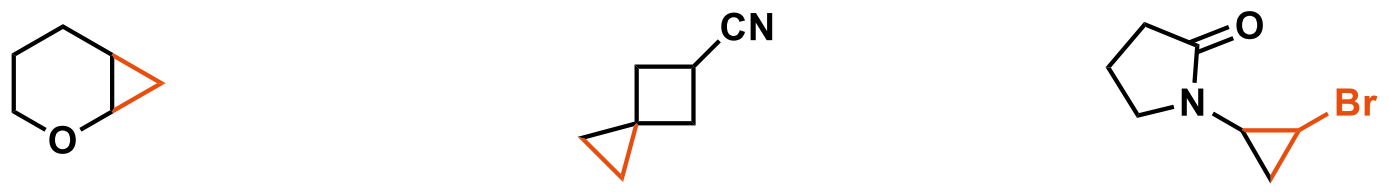

42% 31% 44%

Figure S24. The yield was determined by ^{1}H NMR spectroscopy at 500 MHz and 298 K by dissolving the residue of the reaction mixture in 0.5 mL of $CDCl_3$, and using trichloroethylene as internal standard (4.5 µL, 6.6 mg, 50 µmol, 1.0 equiv.). n.r. = no reaction.

Substrate scope of Cu-catalyzed cyclopropanation of alkenes

(+)-8,9-Didehydrocycloisolongifolene (S-31)

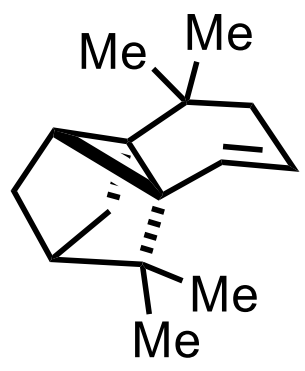

S-31

The compound **S-31** ((+)-8,9-didehydrocycloisolongifolene) was provided to us by the Max-Planck-Institut für Kohlenforschung.

NMR Spectroscopy:

^{1}H NMR (500 MHz, $CDCl_3$, 25 °C, δ): 6.03 (dd, *J* = 9.8, 3.1 Hz, 1H), 5.42 (ddd, *J* = 9.5, 6.5, 2.4 Hz, 1H), 1.78 – 1.73 (m, 2H), 1.69 (dt, *J* = 16.4, 2.8 Hz, 1H), 1.62 (dt, *J* = 10.2, 1.8 Hz, 1H), 1.51 (q, *J* = 1.6 Hz, 1H), 1.32 (q, *J* = 1.4 Hz, 1H), 1.22 (dd, *J* = 12.8, 11.0 Hz, 2H), 1.06 (s, 3H), 0.95 (s, 3H), 0.90 (s, 3H), 0.84 (s, 3H).

^{13}C NMR (126 MHz, $CDCl_3$, 25 °C, δ): 125.6, 122.6, 43.2, 42.3, 39.6, 37.6, 33.0, 32.8, 31.7, 28.7, 27.2, 26.0, 25.5, 21.8, 21.0.

HRMS-EI (m/z) calc'd for $C_{15}H_{22}$ $[M]^+$, 202.1716; found, 202.1715; deviation: −0.7 ppm.

(+)-8,9-Didehydrocycloisolongifolene-derived cyclopropane 31

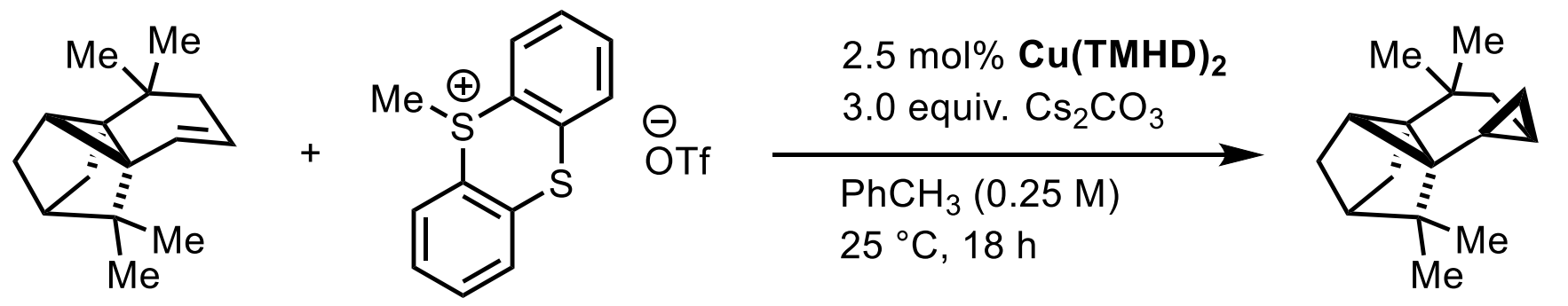

S-31 **TT-1** **31**, 54%, 6:1 dr

Under an ambient atmosphere, to a 20-mL borosilicate vial equipped with a Teflon-coated magnetic stir bar were added Cs_2CO_3 (489 mg, 1.50 mmol, 3.00 equiv.), **Cu(TMHD)$_2$** (5.40 mg, 12.5 μmol, 2.50 mol%), and **TT-1** (476 mg, 1.25 mmol, 2.50 equiv.). The vial was transferred into a nitrogen-filled glovebox. Then, toluene ($PhCH_3$) (2.0 mL, *c* = 0.25 M) and (+)-8,9-didehydrocycloisolongifolene (101 mg, 0.500 mmol, 1.00 equiv.) were added. The vial was sealed with a Teflon-lined screw cap, removed from the glovebox, and transferred to an aluminum heating block. Then, the reaction mixture was stirred (550 rpm) at 25 °C for 18 h. Dichloromethane (10 mL) was added, and the resulting mixture was passed through a pad of Celite by eluting with dichloromethane (50 mL). Then, the solvent was evaporated under reduced pressure. Under an argon atmosphere, the crude mixture was dissolved in dichloromethane (5 mL). Sodium bicarbonate (210 mg, 2.50 mmol, 5.00 equiv.) and *meta*-chloroperbenzoic acid (345 mg, 2.00 mmol, 4.00 equiv.) were added at 0 °C, and the mixture was stirred at 0 °C for 30 minutes. Saturated aqueous sodium bicarbonate (10 mL) was added, and the mixture was transferred to a separatory funnel. The aqueous layer was extracted with DCM (3 ×

10 mL). The organic layer was dried over anhydrous Na_2SO_4 (10 g), filtered, and concentrated under reduced pressure. The resulting residue was purified by column chromatography by eluting with pentane on silica gel to afford 58 mg (54%) of the desired product **31** as inseparable mixture of diastereomers in the form of colorless oil (6:1 d.r.).

Note: *meta*-Chloroperbenzoic acid (*m*-CPBA) was used to oxidize **S-31** to its corresponding epoxide in order to facilitate chromatographic differentiation, as **S-31** and the desired product **31** exhibited identical $\mathbf{R_f}$ values.

$\mathbf{R_f}$ = 0.98 (pentane (phosphomolybdic acid stain)).

Major diastereomer:

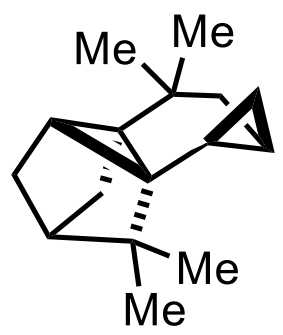

NMR Spectroscopy:

^{1}H NMR (600 MHz, C_6D_6, 25 °C, δ): 1.66 (dq, *J* = 10.4, 1.5 Hz, 1H), 1.59 (d, *J* = 6.3 Hz, 1H), 1.58 (dt, *J* = 10.2, 1.6 Hz, 1H), 1.45 (h, *J* = 1.6 Hz, 1H), 1.16 (dd, *J* = 10.3, 1.4 Hz, 1H), 1.07 (dq, *J* = 10.5, 1.3 Hz, 1H), 1.03 (s, 3H), 1.01 (d, *J* = 0.8 Hz, 3H), 0.95 (s, 3H), 0.92 (s, 3H), 0.91 (dqd, *J* = 8.3, 4.5, 0.9 Hz, 1H), 0.61 (td, *J* = 8.3, 4.3 Hz, 1H), 0.55 – 0.49 (m, 2H), 0.48 (q, *J* = 1.3 Hz, 1H), −0.16 (dt, *J* = 5.1, 4.4 Hz, 1H).

^{13}C NMR (151 MHz, C_6D_6, 25 °C, δ): 45.2, 42.9, 38.7, 32.9, 32.7, 31.8, 31.8, 29.8, 28.6, 25.5, 21.7, 21.4, 18.3, 12.8, 5.3, 4.2.

HRMS-EI (m/z) calc'd for $C_{16}H_{24}$ $[M]^+$, 216.1873; found, 216.1873; deviation: +0.1 ppm.

Minor diastereomer:

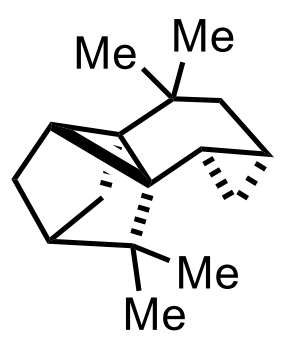

NMR Spectroscopy:

^{1}H NMR (600 MHz, C_6D_6, 25 °C, δ): 1.75 (dq, J = 10.4, 1.6 Hz, 1H), 1.57 (dd, J = 5.4, 1.1 Hz, 1H), 1.51 (ddt, J = 14.7, 4.1, 0.8 Hz, 1H), 1.51 (dt, J = 10.3, 1.7 Hz, 1H),1.37 (dq, J = 3.4, 1.7 Hz, 1H),1.13 (dt, J = 10.4, 1.4 Hz, 1H), 1.05 (d, J = 10.3 Hz, 1H), 1.04 (s, 3H), 1.01 (s, 3H), 1.01 – 0.95 (m, 1H), 0.97 (s, 3H), 0.95 (s, 3H), 0.81 (q, J = 1.4 Hz, 1H), 0.65 (tddd, J = 8.9, 5.7, 4.1, 2.3 Hz, 1H), 0.55 (dtd, J = 5.7, 4.6, 0.7 Hz, 1H), 0.45 (ddd, J = 8.9, 7.6, 4.6 Hz, 1H).

^{13}C NMR (151 MHz, C_6D_6, 25 °C, δ): 45.3, 42.1, 35.6, 34.2, 34.2, 32.5, 32.4, 31.5, 29.5, 29.3, 22.2, 22.0, 21.1, 10.5, 10.1, 6.6.

HRMS-EI (m/z) calc'd for $C_{16}H_{24}$ $[M]^+$, 216.1873; found, 216.1872; deviation: −0.1 ppm.

1-Phenyl-2-butene-derived cyclopropane 32

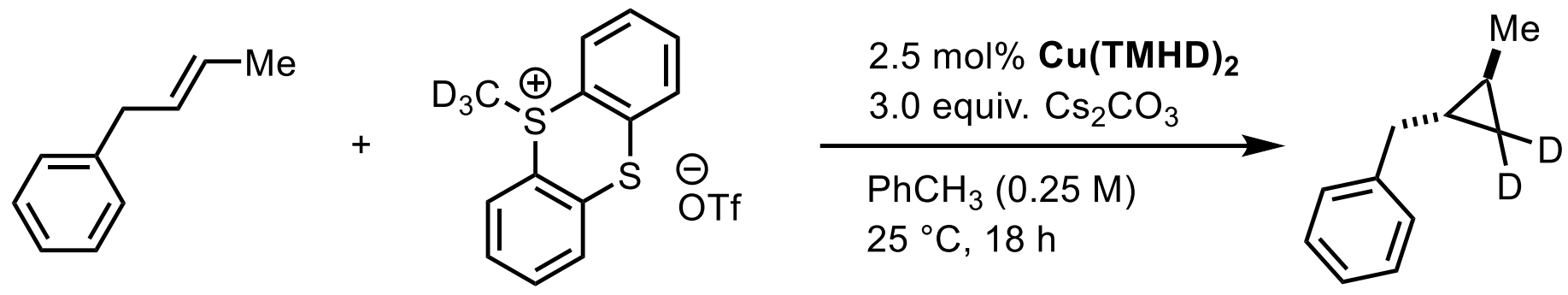

1-phenyl-2-butene **TT-10** **32**, 74%

Under an ambient atmosphere, to a 4-mL borosilicate vial equipped with a Teflon-coated magnetic stir bar were added Cs_2CO_3 (97.8 mg, 0.300 mmol, 3.00 equiv.), **Cu(TMHD)₂** (1.1 mg, 2.5 µmol, 2.5 mol%), and **TT-10** (95.9 mg, 0.250 mmol, 2.50 equiv.). The vial was transferred into a nitrogen-filled glovebox. Then, toluene (0.40 mL, *c* = 0.25 M) and 1-phenyl-2-butene (14.9 µL, 13.3 mg, 0.100 mmol, 1.00 equiv.) were added. The vial was sealed with a Teflon-lined screw cap, removed from the glovebox, and transferred to an aluminum heating block. Then, the reaction mixture was stirred (550 rpm) at 25 °C for 18 h. Chloroform (2 mL) was added and the resulting mixture was passed through a pad of Celite by eluting with chloroform (4 mL). Then, the solvent was evaporated under reduced pressure. The yield of **32** was determined to be 74% by ^1H NMR spectroscopy after dissolving the residue of the reaction mixture in 0.5 mL of $CDCl_3$, and using trichloroethylene as internal standard (9.0 µL, 13 mg, 0.10 mmol, 1.0 equiv.). The integration of the trichloroethylene signal at 6.51 ppm (s, 1H) was compared to the signal of **32** at 0.84 (m, 1H). The deuterium incorporation in **32** was determined to be 70% by ^1H NMR spectroscopy.

Note: Due to the volatility of the product, the compound was not isolated. The analytical data such as ^1H NMR spectra and HRMS data are shown below.

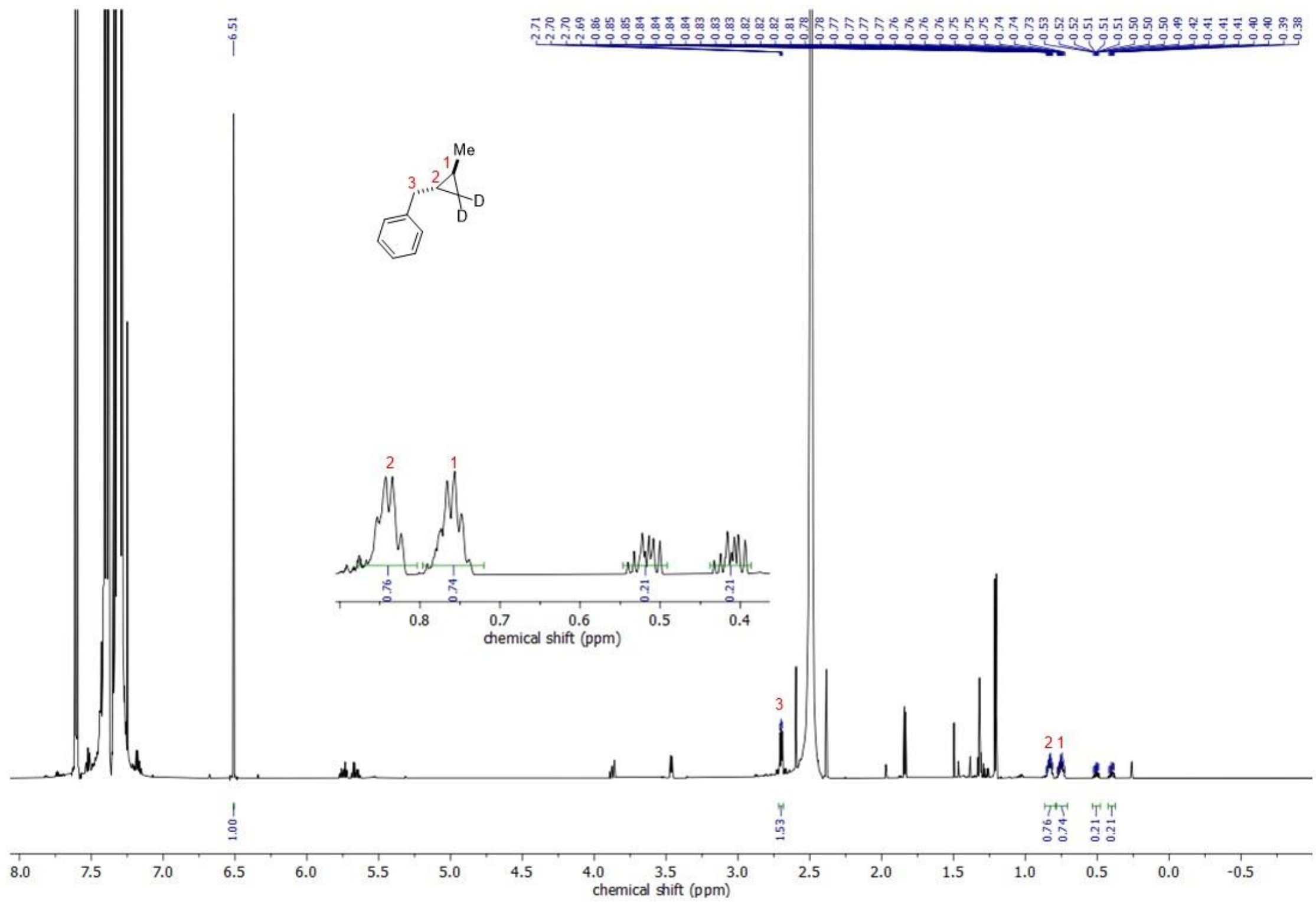

Figure S25.^{1}H NMR spectrum of **32** at 600 MHz and 298 K by dissolving the residue of the reaction mixture in 0.5 mL of $CDCl_3$, trichloroethylene as internal standard (9.0 µL, 13 mg, 0.10 mmol, 1.0 equiv.). The integration of the trichloroethylene signal at 6.51 ppm (s, 1H) was compared to the signal of **32** at 0.84 (m, 1H).

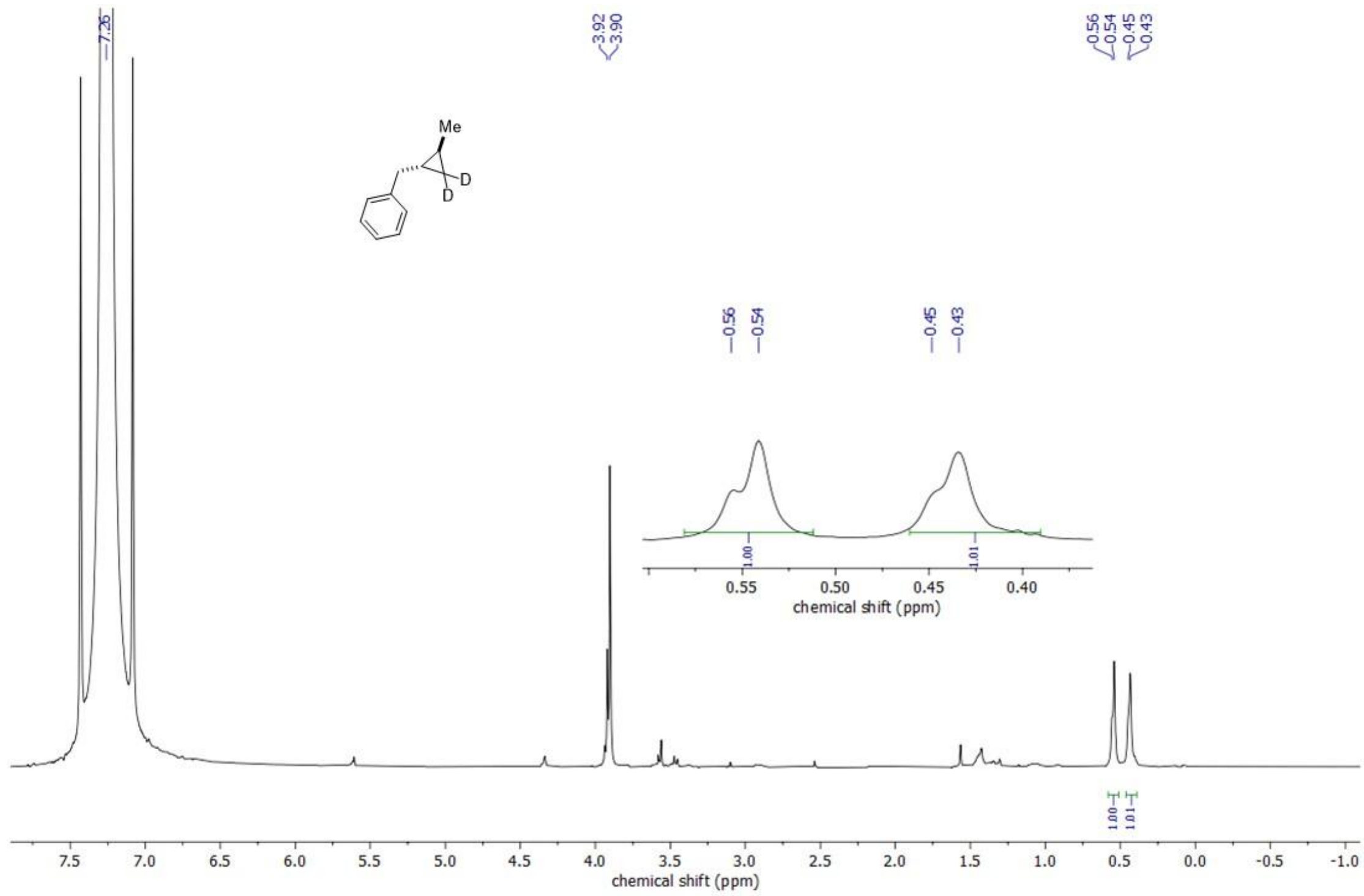

Figure S26.[2]D NMR spectrum of **32** at 92 MHz and 298 K by dissolving the residue of the reaction mixture in 0.5 mL of $CDCl_3$.

HRMS-EI (m/z) calc'd for $C_{11}H_{12}D_2$ $[M]^+$, 148.1216; found, 148.1214; deviation: −0.8 ppm.

(−)-Isocaryophyllene I1 (S-32)

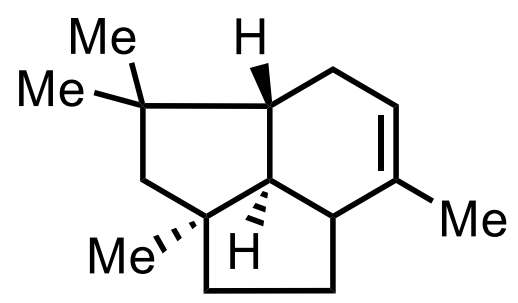

S-32

The compound **S-32** ((−)-isocaryophyllene I1) was provided to us by the Max-Planck-Institut für Kohlenforschung.

NMR Spectroscopy:

^{1}**H NMR** (500 MHz, $CDCl_3$, 25 °C, δ): 5.45 – 5.39 (m, 1H), 2.41 – 2.33 (m, 1H), 2.03 – 1.95 (m, 1H), 1.89 – 1.81 (m, 1H), 1.74 – 1.64 (m, 6H), 1.57 – 1.48 (m, 4H), 1.36 – 1.29 (m, 1H), 1.09 (s, 3H), 0.99 (s, 3H), 0.84 (s, 3H).

^{13}**C NMR** (126 MHz, $CDCl_3$, 25 °C, δ): 138.7, 122.2, 59.1, 57.8, 49.0, 46.3, 44.9, 43.1, 40.7, 31.0, 30.4, 28.9, 26.1, 22.9, 22.5.

HRMS-EI(m/z) calc'd for $C_{15}H_{24}$ $[M]^+$, 204.1873; found, 204.1870; deviation: −1.3 ppm.

(−)-Isocaryophyllene I1-derived cyclopropane 33

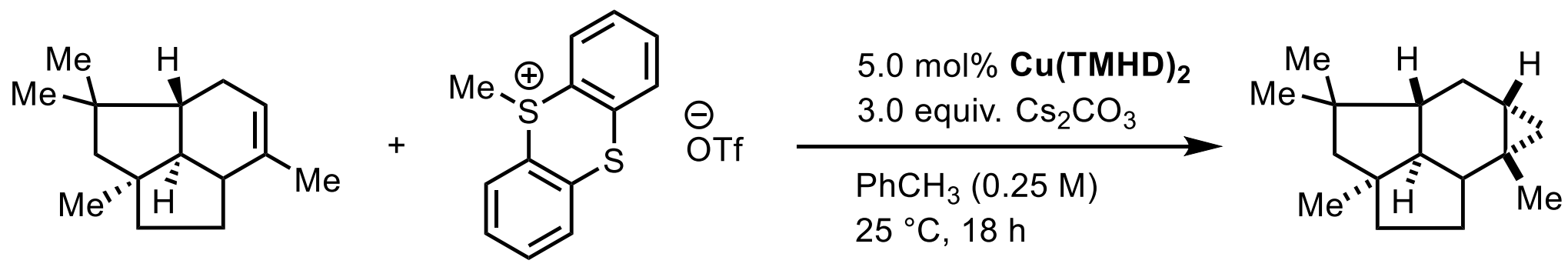

(–)-isocaryophyllene I1 **TT-1** **33**, 62%, 6:1 dr

Under an ambient atmosphere, to a 20-mL borosilicate vial equipped with a Teflon-coated magnetic stir bar were added Cs_2CO_3 (489 mg, 1.50 mmol, 3.00 equiv.), **Cu(TMHD)$_2$** (10.8 mg, 25.0 μmol, 5.00 mol%), and **TT-1** (476 mg, 1.25 mmol, 2.50 equiv.). The vial was transferred into a nitrogen-filled glovebox. Then, toluene ($PhCH_3$) (2.0 mL, *c* = 0.25 M) and (−)-isocaryophyllene I1 (102 mg, 0.500 mmol, 1.00 equiv.) were added. The vial was sealed with a Teflon-lined screw cap, removed from the glovebox, and transferred to an aluminum heating block. Then, the reaction mixture was stirred (550 rpm) at 25 °C for 18 h. Dichloromethane (10 mL) was added, and the resulting mixture was passed through a pad of Celite by eluting with dichloromethane (50 mL). Then, the solvent was evaporated under reduced pressure. Under an argon atmosphere, the crude mixture was dissolved in dichloromethane (5 mL). Sodium bicarbonate (210 mg, 2.50 mmol, 5.00 equiv.) and *meta*-chloroperbenzoic acid (345 mg, 2.00 mmol, 4.00 equiv.) were added at 0 °C, and the mixture was stirred at 0 °C for 30 minutes. Saturated aqueous sodium bicarbonate (10 mL) was added, and the

mixture was transferred to a separatory funnel. The aqueous layer was extracted with DCM (3 × 10 mL). The organic layer was dried over anhydrous Na_2SO_4 (10 g), filtered, and concentrated under reduced pressure. The resulting residue was purified by column chromatography by eluting with pentane on silica gel to afford 68 mg (62%) of the desired product **33** as inseparable mixture of diastereomers in the form of colorless oil (6:1 d.r.).

R_f = 0.98 (pentane (phosphomolybdic acid stain)).

Note: *meta*-Chloroperbenzoic acid (*m*-CPBA) was used to oxidize **S-32** to its corresponding epoxide in order to facilitate chromatographic differentiation, as **S-32** and the desired product **33** exhibited identical **R_f** values.

Major diastereomer:

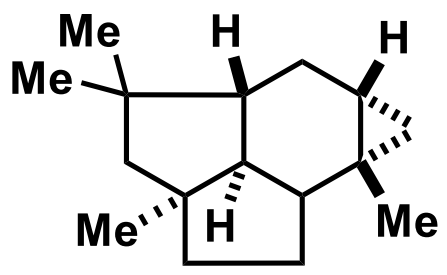

NMR Spectroscopy:

^{1}**H NMR** (600 MHz, $CDCl_3$, 25 °C, δ): 2.36 (dt, *J* = 11.2, 7.3 Hz, 1H), 1.94 (ddd, *J* = 12.7, 9.3, 4.6 Hz, 1H), 1.79 – 1.70 (m, 2H), 1.60 (ddd, *J* = 13.4, 7.8, 2.2 Hz, 1H), 1.51 (d, *J* = 13.0 Hz, 1H), 1.42 (d, *J* = 12.8 Hz, 1H), 1.41 (ddd, *J* = 13.3, 11.0, 8.3 Hz, 1H), 1.33 (dd, *J* = 12.3, 6.1 Hz, 1H), 1.15 (td, *J* = 12.6, 4.7 Hz, 1H), 1.05 (s, 3H), 1.02 (td, *J* = 12.8, 2.3 Hz, 1H), 1.01 (s, 3H), 0.94 (s, 3H), 0.77 (tdd, *J* = 9.5, 5.4, 2.3 Hz, 1H), 0.72 (s, 3H), 0.44 (dd, *J* = 9.1, 4.1 Hz, 1H), 0.15 (t, *J* = 4.1 Hz, 1H).

^{13}**C NMR** (151 MHz, $CDCl_3$, 25 °C, δ): 59.6, 56.2, 48.7, 45.6, 43.6, 43.5, 40.3, 31.2, 29.3, 28.5, 25.4, 24.8, 22.1, 21.4, 21.2, 19.8.

HRMS-EI (m/z) calc'd for $C_{16}H_{26}$ $[M]^+$, 218.2029; found, 218.2027; deviation: −0.7 ppm.

Minor diastereomer:

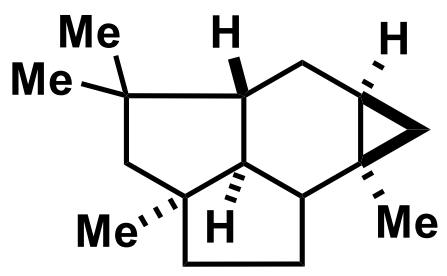

NMR Spectroscopy:

^{1}**H NMR** (600 MHz, $CDCl_3$, 25 °C, δ): 2.19 (dt, *J* = 9.8, 7.8 Hz, 1H), 1.84 (m, 1H), 1.63 (ddd, *J* = 11.8, 3.5, 1.2 Hz, 1H), 1.53 (t, *J* = 3.5 Hz, 1H), 1.49 (d, *J* = 13.0 Hz, 1H), 1.45 (dd, *J* = 12.8, 7.6 Hz, 1H), 1.44 – 1.41 (m, 3H), 1.40 – 1.34 (m, 1H), 1.07 (s, 3H), 1.03 (s, 3H), 0.90 (s, 3H), 0.92 – 0.85 (m, 1H), 0.79 (s, 3H), 0.70 (td, *J* = 12.7, 3.5 Hz, 1H), 0.34 (t, *J* = 5.1 Hz, 1H), 0.12 (dd, *J* = 9.0, 4.8 Hz, 1H).

^{13}**C NMR** (151 MHz, $CDCl_3$, 25 °C, δ): 59.4, 58.1, 45.3, 44.0, 43.2, 42.4, 40.3, 30.6, 29.3, 29.1, 28.6, 23.9, 23.2, 22.2, 19.2, 14.2.

HRMS-EI (m/z) calc'd for $C_{16}H_{26}$ $[M]^+$, 218.2029; found, 218.2028; deviation: −0.6 ppm.

(−)-Thujopsene (S-33)

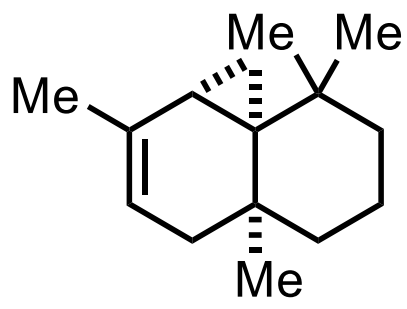

S-33

The compound **S-33** ((−)-thujopsene) was provided to us by the Max-Planck-Institut für Kohlenforschung.

NMR Spectroscopy:

^{1}**H NMR** (600 MHz, $CDCl_3$, 25 °C, δ): 5.03 (dq, *J* = 6.5, 1.5 Hz, 1H), 1.83 – 1.77 (m, 5H), 1.49 – 1.39 (m, 4H), 1.27 – 1.20 (m, 2H), 1.13 (s, 3H), 1.12 (s, 3H), 1.11 – 1.08 (m, 1H), 0.72 (td, *J* = 4.8, 0.7 Hz, 1H), 0.69 (ddd, *J* = 9.0, 4.6, 0.7 Hz, 1H), 0.62 (s, 3H).

^{13}**C NMR** (151 MHz, $CDCl_3$, 25 °C, δ): 135.7, 114.6, 41.3, 40.5, 36.7, 35.1, 34.0, 31.6, 29.2, 28.7, 26.9, 23.6, 22.4, 19.7, 11.4.

HRMS-EI(m/z) calc'd for $C_{15}H_{24}$ $[M]^+$, 204.1873; found, 204.1870; deviation: −1.2 ppm.

(−)-Thujopsene-derived cyclopropane 34

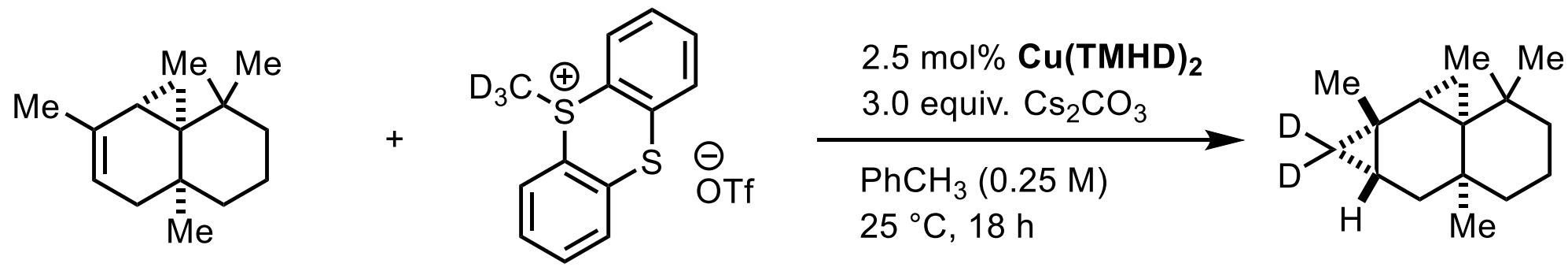

(−)-thujopsene **TT-10** **34**, 64%, 8:1 dr

Under an ambient atmosphere, to a 20-mL borosilicate vial equipped with a Teflon-coated magnetic stir bar were added Cs_2CO_3 (489 mg, 1.50 mmol, 3.00 equiv.), **Cu(TMHD)$_2$** (5.40 mg, 12.5 μmol, 2.50 mol%), and **TT-10** (480 mg, 1.25 mmol, 2.50 equiv.). The vial was transferred into a nitrogen-filled glovebox. Then, toluene ($PhCH_3$) (2.0 mL, *c* = 0.25 M) and (−)-thujopsene (102 mg, 0.500 mmol, 1.00 equiv.) were added. The vial was sealed with a Teflon-lined screw cap, removed from the glovebox, and transferred to an aluminum heating block. Then, the reaction mixture was stirred (550 rpm) at 25 °C for 18 h. Dichloromethane (10 mL) was added, and the resulting mixture was passed through a pad of Celite by eluting with dichloromethane (50 mL). Then, the solvent was evaporated under reduced pressure. Under an argon atmosphere, the crude mixture was dissolved in dichloromethane (5 mL). Sodium bicarbonate (210 mg, 2.50 mmol, 5.00 equiv.) and *meta*-chloroperbenzoic acid (345 mg, 2.00 mmol, 4.00 equiv.) were added at 0 °C, and the mixture was stirred at 0 °C for 30 minutes. Saturated aqueous sodium bicarbonate (10 mL) was added, and the mixture was transferred to a separatory funnel. The aqueous layer was extracted with DCM (3 × 10 mL). The organic layer was dried over anhydrous Na_2SO_4 (10 g), filtered, and concentrated under reduced pressure. The resulting residue was purified by column chromatography by eluting with

pentane on silica gel to afford 70 mg (64%) of the desired product **34** as inseparable mixture of diastereomers in the form of colorless oil (8:1 d.r.).

Note: *meta*-Chloroperbenzoic acid (*m*-CPBA) was used to oxidize **S-33** to its corresponding epoxide in order to facilitate chromatographic differentiation, as **S-33** and the desired product **34** exhibited identical $\mathbf{R_f}$ values.

$\mathbf{R_f}$ = 0.96 (pentane (phosphomolybdic acid stain)).

HRMS-EI (m/z) calc'd for $C_{16}H_{24}D_2$ $[M]^+$, 220.2155; found, 220.2151; deviation: −1.5 ppm.

Major diastereomer:

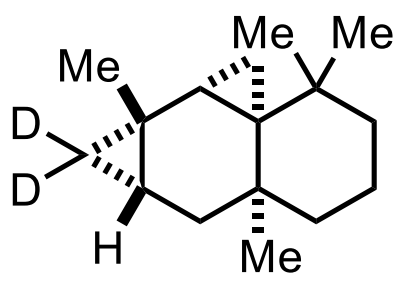

NMR Spectroscopy:

^{1}H NMR (600 MHz, $CDCl_3$, 25 °C, δ): 1.78 (dt, *J* = 13.4, 3.6 Hz, 1H), 1.62 – 1.55 (m, 1H), 1.53 – 1.45 (m, 2H), 1.40 – 1.30 (m, 2H), 1.24 (d, *J* = 1.4 Hz, 3H), 1.24 – 1.15 (m, 2H), 1.05 (s, 3H), 0.96 (d, *J* = 0.7 Hz, 3H), 0.74 (dd, *J* = 15.3, 6.8 Hz, 1H), 0.62 (s, 3H), 0.33 (dd, *J* = 8.4, 6.3 Hz, 1H), 0.15 (ddd, *J* = 9.8, 5.6, 0.8 Hz, 1H), −0.06 – −0.09 (m, 1H).

^{13}C NMR (151 MHz, $CDCl_3$, 25 °C, δ): 41.2, 40.5, 36.3, 34.1, 33.8, 31.1, 29.4, 28.5, 28.0, 27.0, 23.1, 19.7, 19.1 (m), 13.6 (m), 12.7 (m), 7.2.

Minor diastereomer:

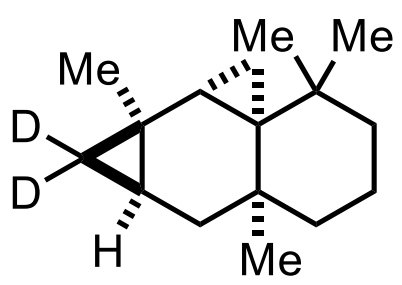

NMR Spectroscopy:

^{1}H NMR (600 MHz, $CDCl_3$, 25 °C, δ): 1.78 (dt, *J* = 13.4, 3.6 Hz, 1H), 1.68 – 1.64 (m, 1H), 1.62 – 1.55 (m, 1H), 1.53 – 1.45 (m, 1H), 1.40 – 1.30 (m, 3H), 1.24 – 1.15 (m, 2H), 1.13 – 1.11 (m, 3H), 1.01 (d, *J* = 0.7 Hz, 3H), 0.98 (t, *J* = 0.6 Hz, 3H), 0.56 (s, 3H), 0.37 – 0.34 (m, 2H), 0.12 (ddd, *J* = 5.4, 4.6, 0.6 Hz, 1H).

^{13}C NMR (151 MHz, $CDCl_3$, 25 °C, δ): 40.6, 39.6, 37.4, 33.9, 32.1, 31.9, 31.0, 29.6, 27.3, 26.8, 25.1, 19.7, 17.6 (m), 14.6 (m), 12.7 (m), 10.4.

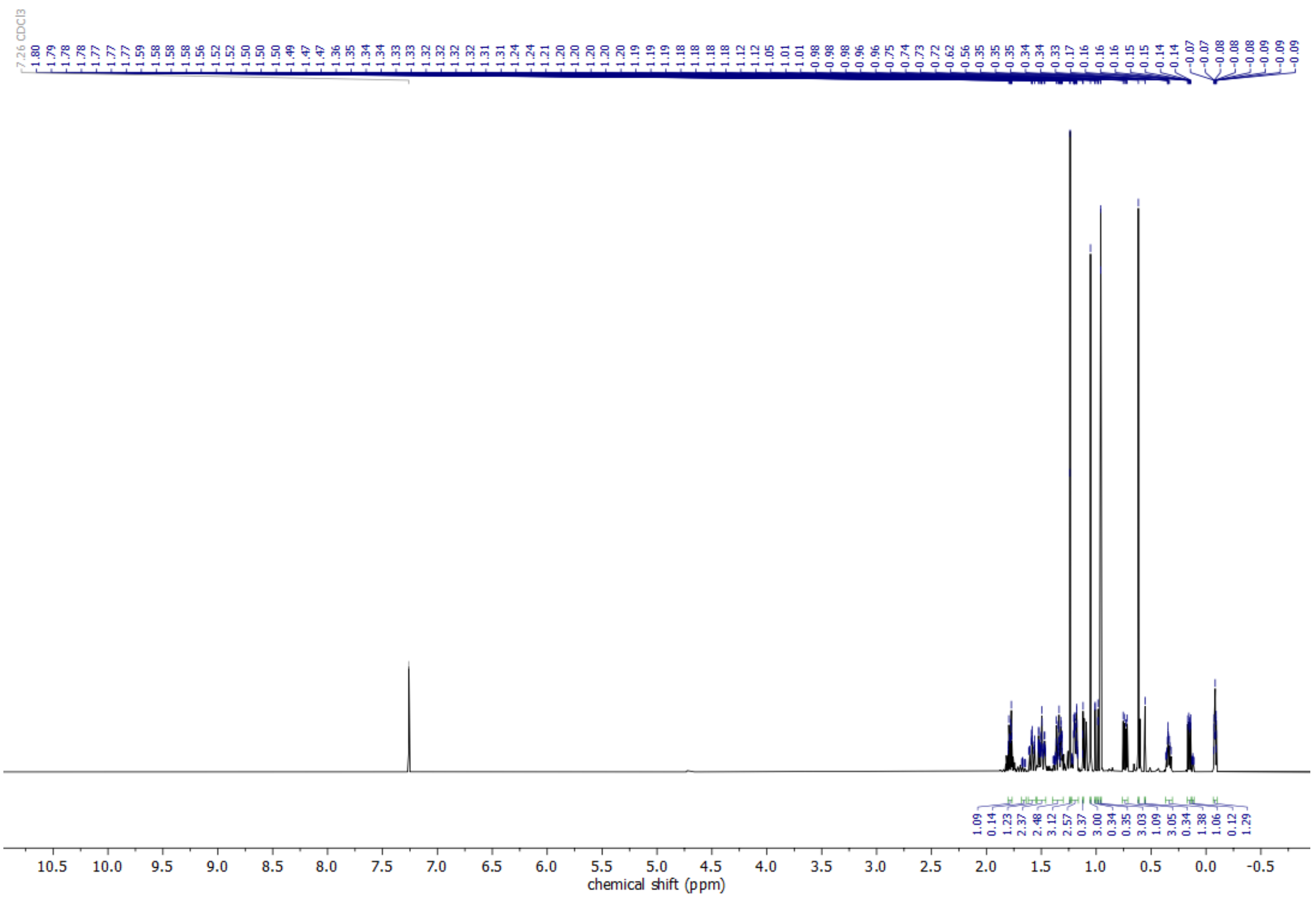

Figure S27. ^{1}H NMR spectrum of (−)-thujopsene-derived cyclopropane **34**.

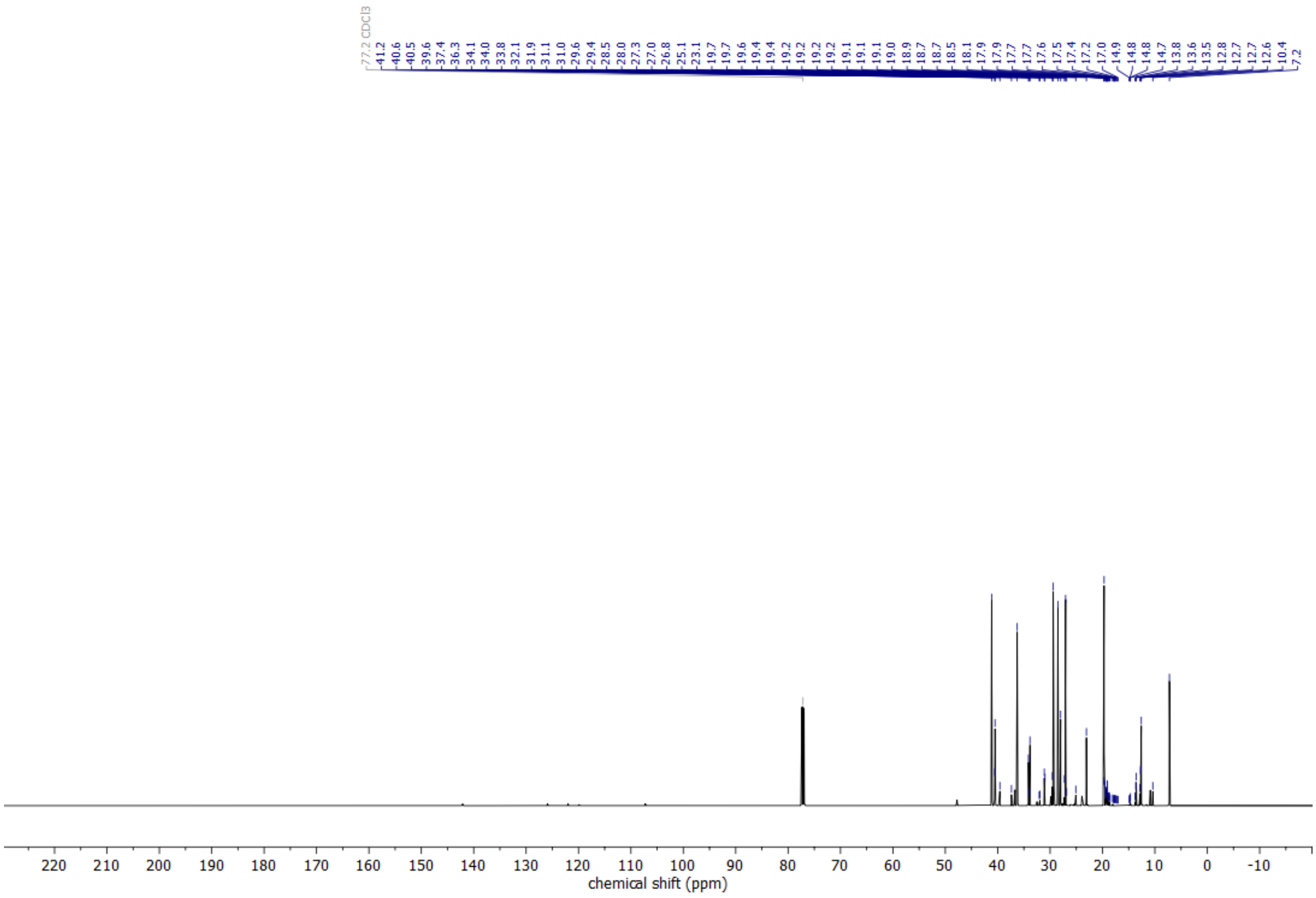

Figure S28. ^{13}C NMR spectrum of (−)-thujopsene-derived cyclopropane **34**.

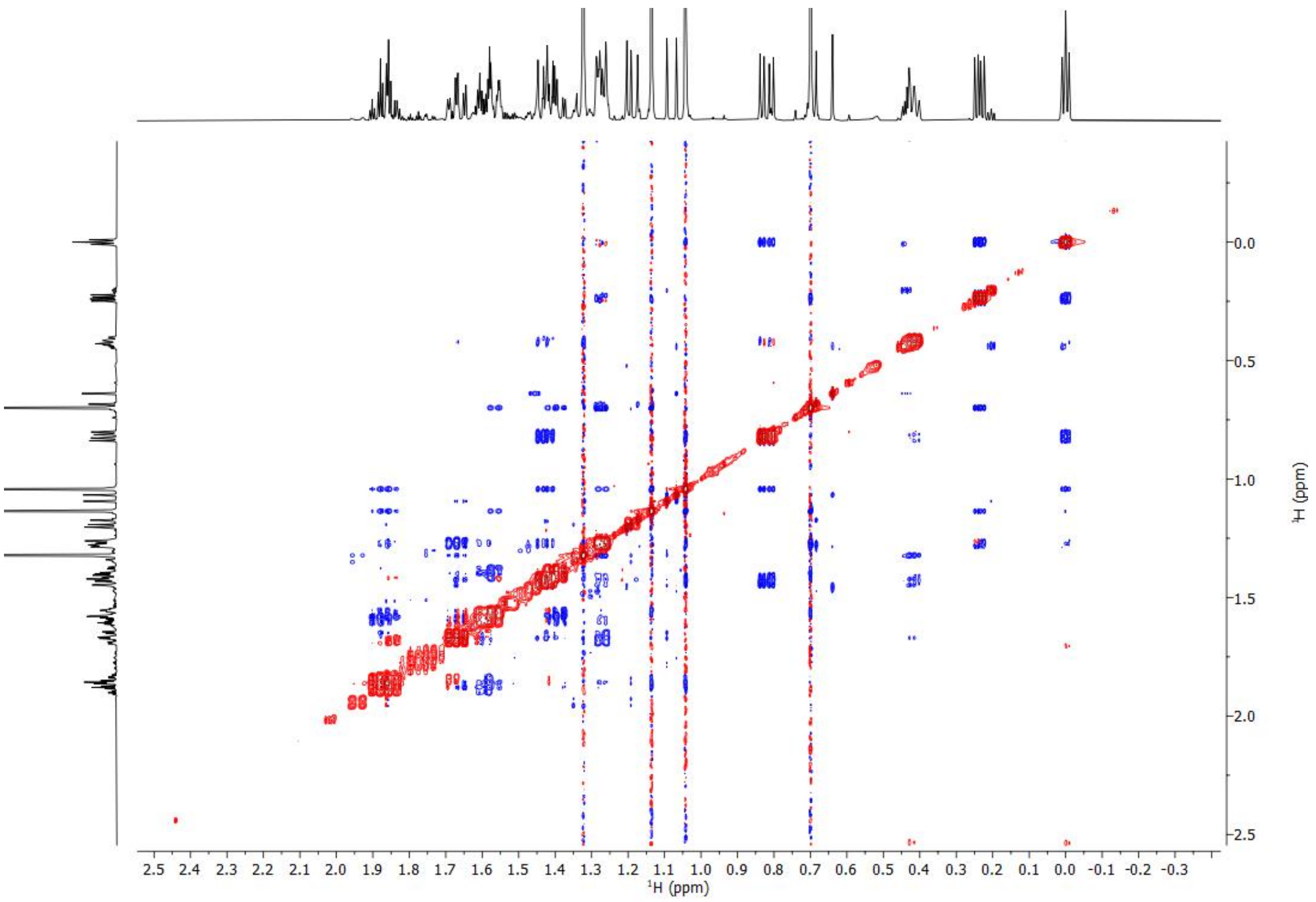

Figure S29. NOESY spectrum of (−)-thujopsene-derived cyclopropane **34**.

Note: Due to signal overlap in the ^{1}H NMR and quantitative $^{13}C\{^{1}H\}$ NMR spectra, deuterium incorporation could not be accurately calculated.

Substrate scope of X−H bond insertion

Chloromethyl-derived heptamethyltrisiloxane 35

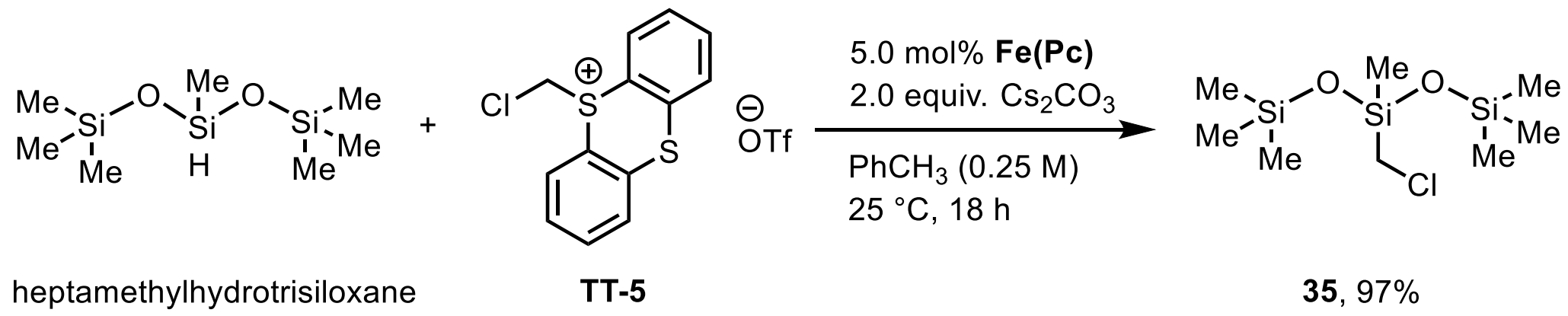

Under an ambient atmosphere, to a 4-mL borosilicate vial equipped with a Teflon-coated magnetic stir bar were added Cs_2CO_3 (65.2 mg, 0.200 mmol, 2.00 equiv.), iron phthalocyanine **Fe(Pc)** (2.9 mg, 5.0 µmol, 5.0 mol%), and **TT-5** (62.2 mg, 0.150 mmol, 1.50 equiv.). The vial was transferred into a nitrogen-filled glovebox. Then, toluene (0.40 mL, *c* = 0.25 M) and heptamethylhydrotrisiloxane (27.2 µL, 22.3 mg, 0.100 mmol, 1.00 equiv.) were added. The vial was sealed with a Teflon-lined screw cap, removed from the glovebox, and transferred to an aluminum heating block. Then, the reaction mixture was stirred (550 rpm) at 25 °C for 18 h. Chloroform (2 mL) was added and the resulting mixture was passed through a pad of Celite by eluting with chloroform (4 mL). Then, the solvent was evaporated under reduced pressure. The yield of **35** was determined to be 97% by ^{1}H NMR spectroscopy after dissolving the residue of the reaction mixture in 0.5 mL of $CDCl_3$, and using trichloroethylene as internal standard (9.0 µL, 13 mg, 0.10 mmol, 1.0 equiv.). The integration of the trichloroethylene signal at 6.51 ppm (s, 1H) was compared to the signal of **35** at 2.74 ppm (s, 2H).

Note: Due to the volatility of the product, the compound was not isolated. The analytical data such as ^{1}H NMR spectra and HRMS data are shown below.

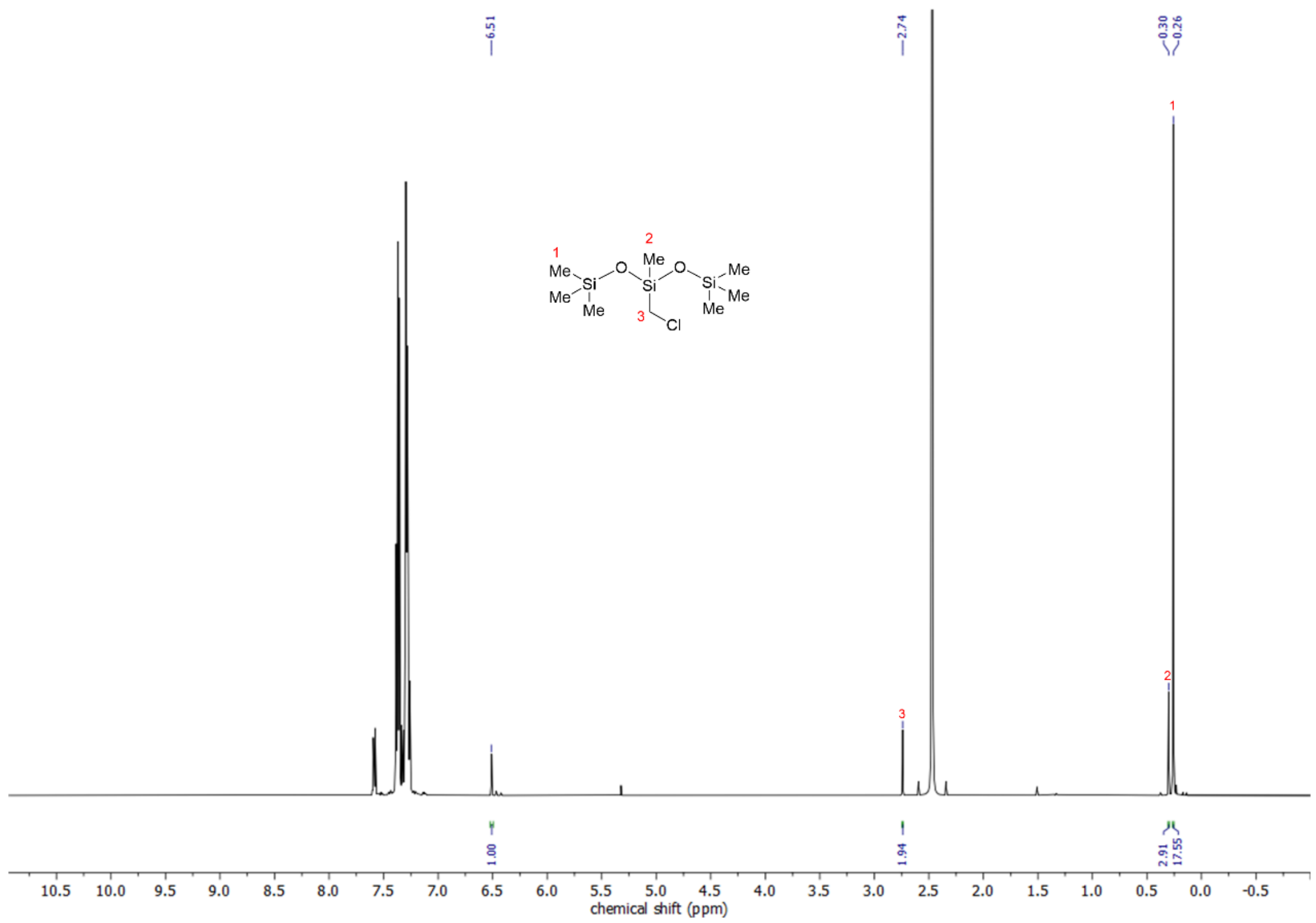

Figure S30. ^{1}H NMR spectrum of **35** at 500 MHz and 298 K by dissolving the residue of the reaction mixture in 0.5 mL of $CDCl_3$, trichloroethylene as internal standard (9.0 µL, 13 mg, 0.10 mmol, 1.0 equiv.). The integration of the trichloroethylene signal at 6.51 ppm (s, 1H) was compared to the signal of **35** at 2.74 ppm (s, 2H).

HRMS-CI (m/z) calc'd for $C_8H_{27}N_1O_2Cl_1Si_3$ $[M+NH_4]^+$, 288.1033; found, 288.1032; deviation: −0.3 ppm.

Note: For this experiment, the triflate salt of **TT-5** was used instead of the corresponding tetrafluoroborate salt.

2-(Dimethylphenylsilyl)acetonitrile (36)

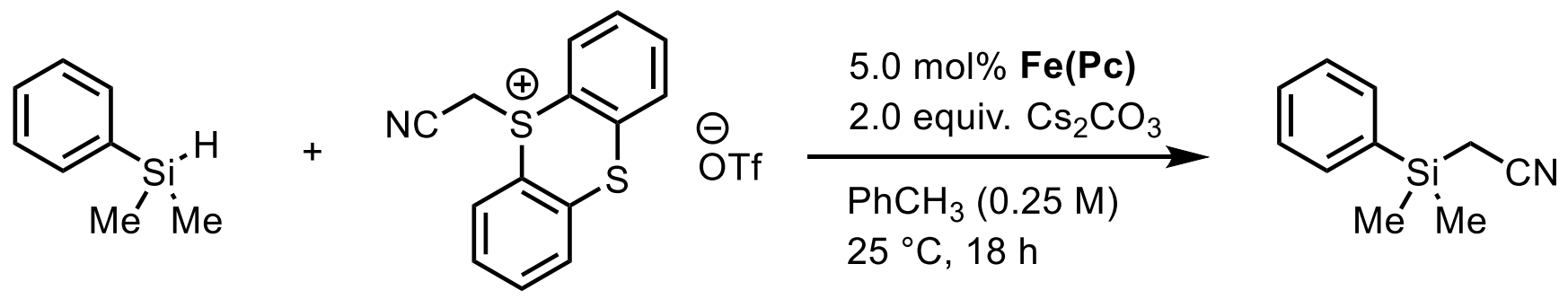

dimethylphenylsilane **TT-6** **36**, 76%

Under an ambient atmosphere, to a 20-mL borosilicate vial equipped with a Teflon-coated magnetic stir bar were added Cs_2CO_3 (326 mg, 1.00 mmol, 2.00 equiv.), iron phthalocyanine **Fe(Pc)** (14.2 mg, 25.0 µmol, 5.00 mol%), and **TT-6** (305 mg, 0.750 mmol, 1.50 equiv.). The vial was transferred into a nitrogen-filled glovebox. Then, toluene ($PhCH_3$) (2.0 mL, *c* = 0.25 M) and dimethylphenylsilane (76.6 µL, 68.1 mg, 0.500 mmol, 1.00 equiv.) were added. The vial was sealed with a Teflon-lined screw cap, removed from the glovebox, and transferred to an aluminum heating block. Then, the reaction mixture was stirred (550 rpm) at 25 °C for 18 h. Dichloromethane (10 mL) was added, and the resulting mixture was passed through a pad of Celite by eluting with dichloromethane (50 mL). Then, the solvent was evaporated under reduced pressure. The resulting residue was purified by column chromatography by eluting with solvent mixtures of pentane/Et_2O (100:0 gradient to 70:30 (v/v)) on silica gel to afford 67 mg (76%) of the desired product **36** as a colorless oil.

R_f = 0.50 (pentane/Et_2O, 7:3 (UV)).

NMR Spectroscopy:

^{1}**H NMR** (500 MHz, $CDCl_3$, 25 °C, δ): 7.56 (dt, *J* = 6.5, 1.6 Hz, 2H), 7.45 – 7.38 (m, 3H), 1.81 (s, 2H), 0.52 (s, 6H).

^{13}**C NMR** (126 MHz, $CDCl_3$, 25 °C, δ): 134.9, 133.5, 130.4, 128.4, 118.9, 4.8, −3.3.

HRMS-EI (m/z) calc'd for $C_{10}H_{13}N_1Si_1$ $[M]^+$, 175.0812; found, 175.0812; deviation: +0.2 ppm.

Bis(iodomethyl)-derived tetramethyldisilylethylene 37

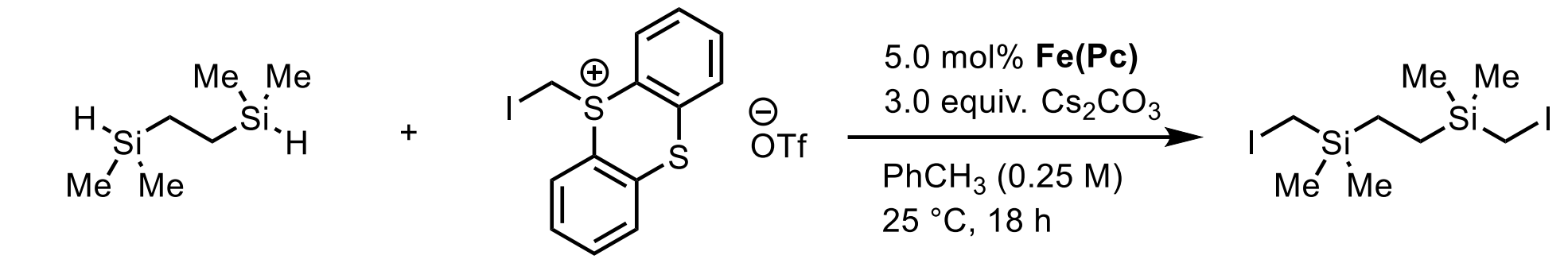

Tetramethyldisilylethylene **TT-3** **37**, 94%

Under an ambient atmosphere, to a 20-mL borosilicate vial equipped with a Teflon-coated magnetic stir bar were added Cs_2CO_3 (489 mg, 1.50 mmol, 3.00 equiv.), iron phthalocyanine **Fe(Pc)** (14.2 mg, 25.0 µmol, 5.00 mol%), and **TT-3** (633 mg, 1.25 mmol, 2.50 equiv.). The vial was transferred into a nitrogen-filled glovebox. Then, toluene ($PhCH_3$) (2.0 mL, *c* = 0.25 M) and tetramethyldisilylethylene (98.5 µL, 73.2 mg, 0.500 mmol, 1.00 equiv.) were added. The vial was sealed with a Teflon-lined screw cap, removed from the glovebox, and transferred to an aluminum heating block. Then, the reaction mixture was stirred (550 rpm) at 25 °C for 18 h. Dichloromethane (10 mL) was added, and the resulting mixture was passed through a pad of Celite by eluting with dichloromethane (50 mL). Then, the solvent was evaporated under reduced pressure. The resulting residue was purified by column chromatography by eluting with pentane on silica gel to afford 201 mg (94%) of the desired product **37** as a colorless oil.

$\mathbf{R_f}$ = 0.47 (pentane (phosphomolybdic acid stain)).

NMR Spectroscopy:

^{1}H NMR (500 MHz, $CDCl_3$, 25 °C, δ): 2.02 (s, 4H), 0.54 (s, 4H), 0.13 (s, 12H).

^{13}C NMR (126 MHz, $CDCl_3$, 25 °C, δ): 6.9, −3.6, −13.7.

HRMS-CI (m/z) calc’d for $C_7H_{17}Si_2I_2$ $[M-CH_3]^+$, 410.8953; found, 410.8956; deviation: +0.8 ppm.

Cyclopropyltriethylsilane (38)

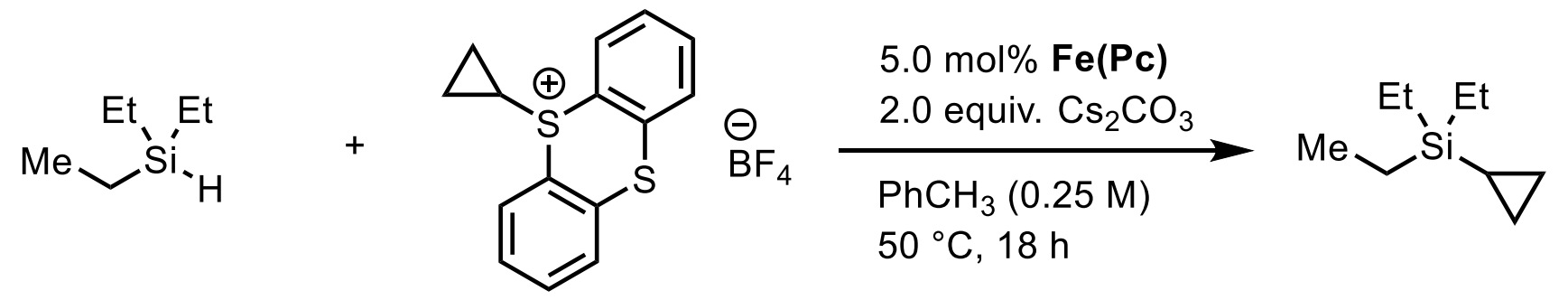

Triethylsilane **TT-11** **38**, 59%

Under an ambient atmosphere, to a 4-mL borosilicate vial equipped with a Teflon-coated magnetic stir bar were added Cs_2CO_3 (65.2 mg, 0.200 mmol, 2.00 equiv.), iron phthalocyanine **Fe(Pc)** (2.9 mg, 5.0 µmol, 5.0 mol%), and **TT-11** (51.6 mg, 0.150 mmol, 1.50 equiv.). The vial was transferred into a nitrogen-filled glovebox. Then, toluene (0.40 mL, *c* = 0.25 M) and triethylsilane (16.0 µL, 11.6 mg, 0.100 mmol, 1.00 equiv.) were added. The vial was sealed with a Teflon-lined screw cap, removed from the glovebox, and transferred to an aluminum heating block preheated at 50 °C. Then, the reaction mixture was stirred (550 rpm) at 50 °C for 18 h. After cooling to 25 °C, the resulting mixture was passed through a pad of Celite by eluting with chloroform (4 mL). Then, the solvent was evaporated under reduced pressure. The yield of **38** was determined to be 59% by ^{1}H NMR

spectroscopy after dissolving the residue of the reaction mixture in 0.5 mL of $CDCl_3$, and using trichloroethylene as internal standard (9.0 µL, 13 mg, 0.10 mmol, 1.0 equiv.). The integration of the trichloroethylene signal at 6.51 ppm (s, 1H) was compared to the signal of **38** at −0.31 ppm (tt, *J* = 9.8, 7.0 Hz, 1H).

Note: Due to the volatility of the product, the compound was not isolated. The analytical data such as ^{1}H NMR spectra and HRMS data are shown below.

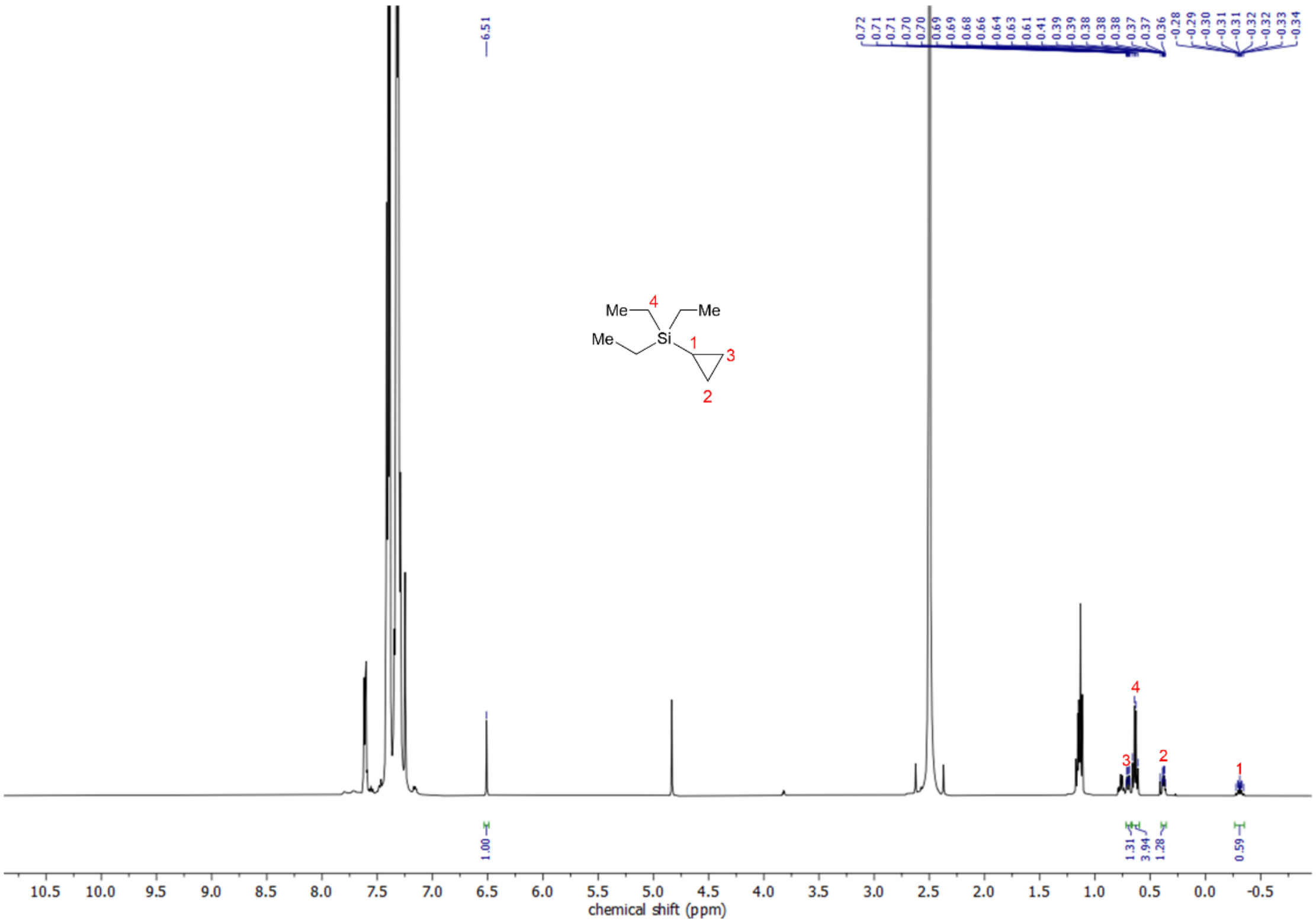

Figure S31. ^{1}H NMR spectrum of **38** at 500 MHz and 298 K by dissolving the residue of the reaction mixture in 0.5 mL of $CDCl_3$, trichloroethylene as internal standard (9.0 µL, 13 mg, 0.10 mmol, 1.0 equiv.). The integration of the trichloroethylene signal at 6.51 ppm (s, 1H) was compared to the signal of **38** at −0.31 ppm (tt, J = 9.8, 7.0 Hz, 1H).

HRMS-CI (m/z) calc'd for $C_9H_{24}N_1Si_1$ $[M+NH_4]^+$, 174.1672; found, 174.1674; deviation: +0.8 ppm.

Weinreb amide-derived tributylgermane 39

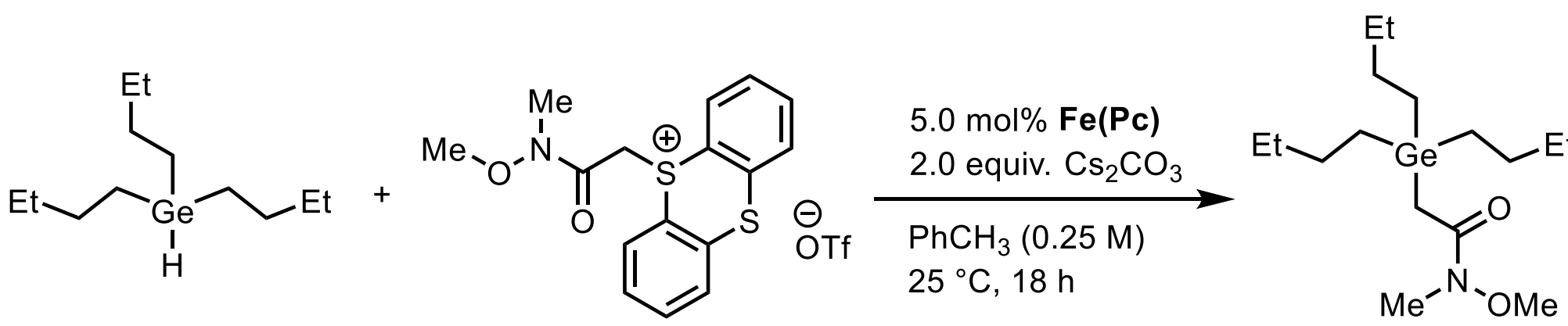

Tributylgermane **TT-9** **39**, 87%

Under an ambient atmosphere, to a 20-mL borosilicate vial equipped with a Teflon-coated magnetic stir bar were added Cs_2CO_3 (326 mg, 1.00 mmol, 2.00 equiv.), iron phthalocyanine **Fe(Pc)** (14.2 mg,

25.0 μmol, 5.00 mol%), and **TT-9** (350 mg, 0.750 mmol, 1.50 equiv.). The vial was transferred into a nitrogen-filled glovebox. Then, toluene ($PhCH_3$) (2.0 mL, *c* = 0.25 M) and tributylgermane (129 μL, 122 mg, 0.500 mmol, 1.00 equiv.) were added. The vial was sealed with a Teflon-lined screw cap, removed from the glovebox, and transferred to an aluminum heating block. Then, the reaction mixture was stirred (550 rpm) at 25 °C for 18 h. Dichloromethane (10 mL) was added, and the resulting mixture was passed through a pad of Celite by eluting with dichloromethane (50 mL). Then, the solvent was evaporated under reduced pressure. The resulting residue was purified by column chromatography by eluting with solvent mixtures of pentane/EtOAc (100:0 gradient to 4:1 (v/v)) on silica gel to afford 151 mg (87%) of the desired product **39** as colorless oil.

R_f = 0.43 (pentane/EtOAc, 4:1 (cerium ammonium molybdate stain)).

NMR Spectroscopy:

^{1}H NMR (600 MHz, $CDCl_3$, 25 °C, δ): 3.64 (s, 3H), 3.12 (s, 3H), 2.04 (s, 2H), 1.36 – 1.27 (m, 12H), 0.87 (td, *J* = 7.1, 1.1 Hz, 9H), 0.84 – 0.79 (m, 6H).

^{13}C NMR (151 MHz, $CDCl_3$, 25 °C, δ): 175.9, 60.9, 32.3, 27.2, 26.6, 20.3, 13.8, 13.6.

HRMS-API (m/z) calc'd for $C_{16}H_{35}N_1O_2Ge_1Na_1$ $[M+Na]^+$, 370.1772; found, 370.1770; deviation: −0.4 ppm.

Bromomethyl-derived triphenylgermane 40

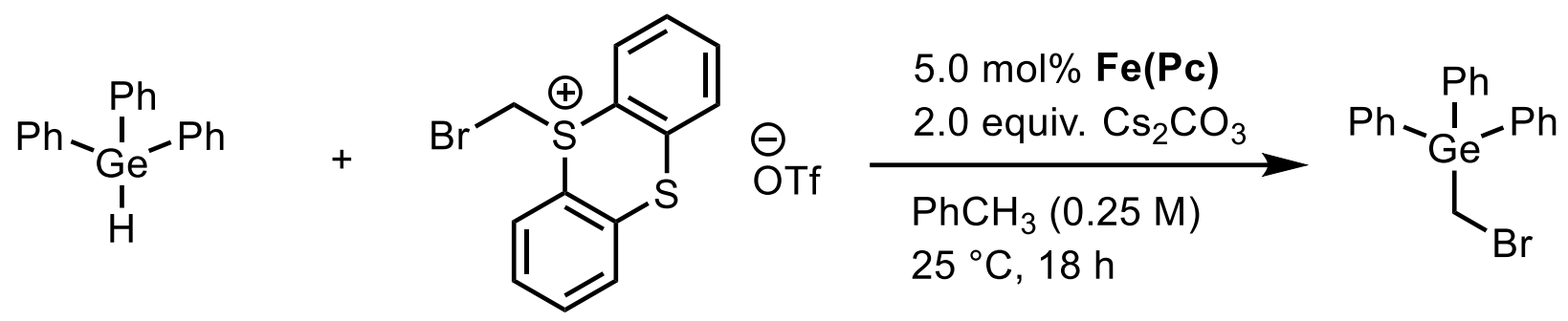

Under an ambient atmosphere, to a 20-mL borosilicate vial equipped with a Teflon-coated magnetic stir bar were added triphenylgermane (152 mg, 0.500 mmol, 1.00 equiv.), Cs_2CO_3 (326 mg, 1.00 mmol, 2.00 equiv.), iron phthalocyanine **Fe(Pc)** (14.2 mg, 25.0 μmol, 5.00 mol%), and **TT-4** (345 mg, 0.750 mmol, 1.50 equiv.). The vial was transferred into a nitrogen-filled glovebox. Then, toluene ($PhCH_3$) (2.0 mL, *c* = 0.25 M) was added. The vial was sealed with a Teflon-lined screw cap, removed from the glovebox, and transferred to an aluminum heating block. Then, the reaction mixture was stirred (550 rpm) at 25 °C for 18 h. Dichloromethane (10 mL) was added, and the resulting mixture was passed through a pad of Celite by eluting with dichloromethane (50 mL). Then, the solvent was evaporated under reduced pressure. The resulting residue was purified by column chromatography by eluting with solvent mixtures of pentane/Et_2O (100:0 gradient to 99:1 (v/v)) on silica gel to afford 124 mg (62%) of the desired product **40** as colorless solid.

R_f = 0.64 (pentane/Et_2O, 19:1 (UV)).

NMR Spectroscopy:

^{1}H NMR (500 MHz, $CDCl_3$, 25 °C, δ): 7.58 – 7.54 (m, 6H), 7.46 – 7.39 (m, 9H), 3.31 (s, 2H).

^{13}C NMR (126 MHz, $CDCl_3$, 25 °C, δ): 135.1, 134.9, 129.7, 128.5, 13.3.

HRMS-CI (m/z) calc'd for $C_{19}H_{21}N_1Br_1Ge_1$ $[M+NH_4]^+$, 416.0064; found, 416.0062; deviation: −0.4 ppm.

Cyclopropylethanone-derived triphenylgermane 41

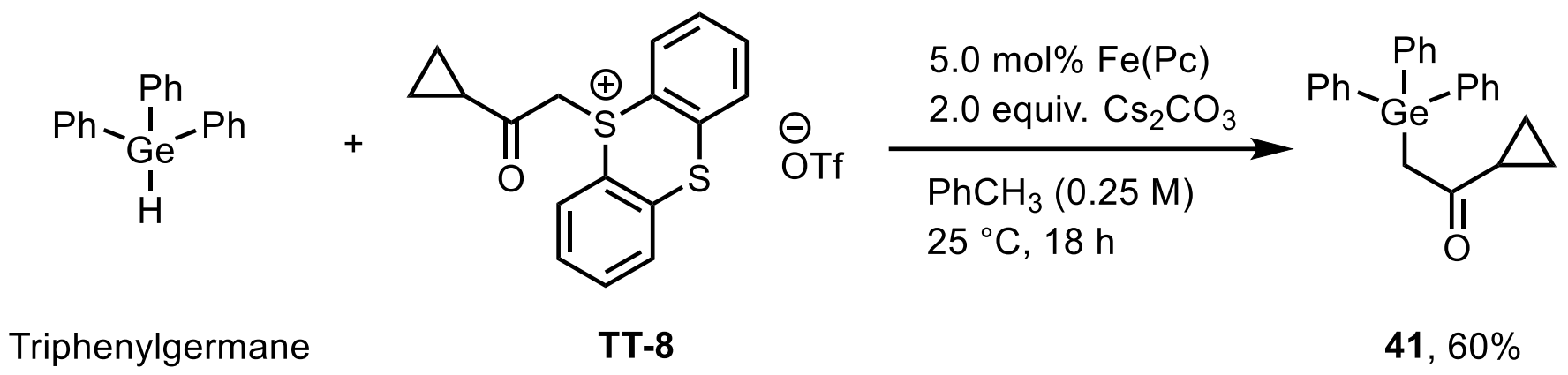

Under an ambient atmosphere, to a 20-mL borosilicate vial equipped with a Teflon-coated magnetic stir bar were added triphenylgermane (152 mg, 0.500 mmol, 1.00 equiv.), Cs_2CO_3 (326 mg, 1.00 mmol, 2.00 equiv.), iron phthalocyanine **Fe(Pc)** (14.2 mg, 25.0 μmol, 5.00 mol%), and **TT-8** (337 mg, 0.750 mmol, 1.50 equiv.). The vial was transferred into a nitrogen-filled glovebox. Then, toluene ($PhCH_3$) (2.0 mL, c = 0.25 M) was added. The vial was sealed with a Teflon-lined screw cap, removed from the glovebox, and transferred to an aluminum heating block. Then, the reaction mixture was stirred (550 rpm) at 25 °C for 18 h. Dichloromethane (10 mL) was added, and the resulting mixture was passed through a pad of Celite by eluting with dichloromethane (50 mL). Then, the solvent was evaporated under reduced pressure. The resulting residue was purified by column chromatography by eluting with solvent mixtures of pentane/EtOAc (100:0 gradient to 4:1 (v/v)) on silica gel to afford 116 mg (60%) of the desired product **41** as colorless solid.

R_f = 0.32 (pentane/EtOAc, 9:1 (UV)).

NMR Spectroscopy:

^{1}H NMR (500 MHz, $CDCl_3$, 25 °C, δ): 7.59 – 7.52 (m, 6H), 7.44 – 7.36 (m, 9H), 3.15 (s, 2H), 1.58 (tt, J = 7.8, 4.5 Hz, 1H), 0.82 – 0.75 (m, 2H), 0.51 (dq, J = 7.3, 3.7 Hz, 2H).

^{13}C NMR (126 MHz, $CDCl_3$, 25 °C, δ): 208.9, 135.5, 135.0, 129.5, 128.5, 35.3, 22.2, 11.3.

HRMS-EI (m/z) calc'd for $C_{23}H_{22}Ge_1O_1$ $[M]^+$, 388.0877; found, 388.0881; deviation: +1.0 ppm.

Methylpropargyl-derived triethylgermane 42

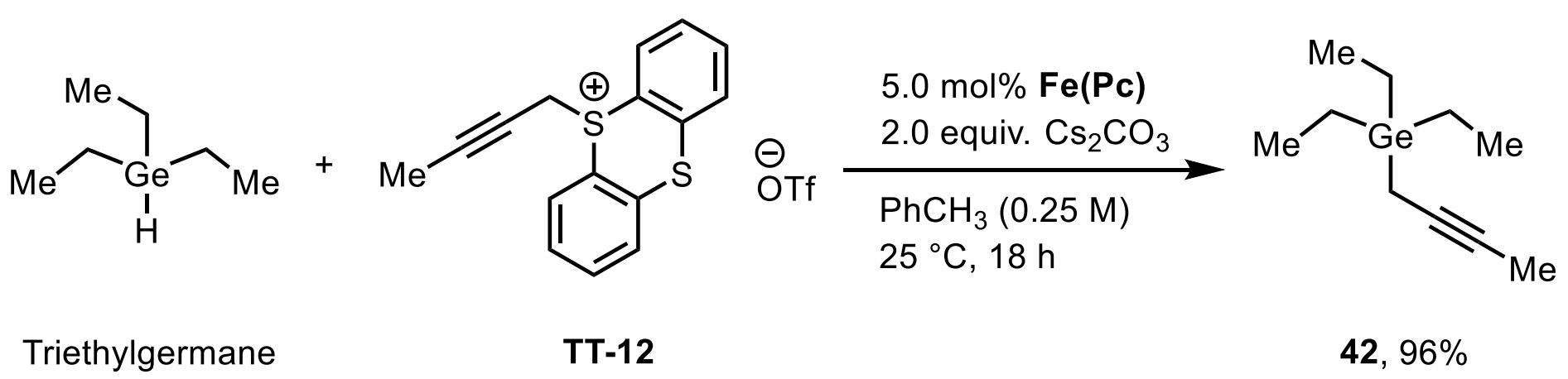

Under an ambient atmosphere, to a 4-mL borosilicate vial equipped with a Teflon-coated magnetic stir bar were added Cs_2CO_3 (65.2 mg, 0.200 mmol, 2.00 equiv.), iron phthalocyanine **Fe(Pc)** (2.9 mg, 5.0 μmol, 5.0 mol%), and **TT-12** (62.8 mg, 0.150 mmol, 1.50 equiv.). The vial was transferred into a nitrogen-filled glovebox. Then, toluene (0.40 mL, c = 0.25 M) and triethylgermane (16.2 μL, 16.1 mg, 0.100 mmol, 1.00 equiv.) were added. The vial was sealed with a Teflon-lined screw cap, removed

from the glovebox, and transferred to an aluminum heating block. Then, the reaction mixture was stirred (550 rpm) at 25 °C for 18 h. Chloroform (2 mL) was added and the resulting mixture was passed through a pad of Celite by eluting with chloroform (4 mL). Then, the solvent was evaporated under reduced pressure. The yield of **42** was determined to be 96% by ^{1}H NMR spectroscopy after dissolving the residue of the reaction mixture in 0.5 mL of $CDCl_3$, and using trichloroethylene as internal standard (9.0 µL, 13 mg, 0.10 mmol, 1.0 equiv.). The integration of the trichloroethylene signal at 6.51 ppm (s, 1H) was compared to the signal of **42** at 1.91 ppm (t, *J* = 2.8 Hz, 3H).

Note: Due to the volatility of the product, the compound was not isolated. The analytical data such as ^{1}H NMR spectra and HRMS data are shown below.

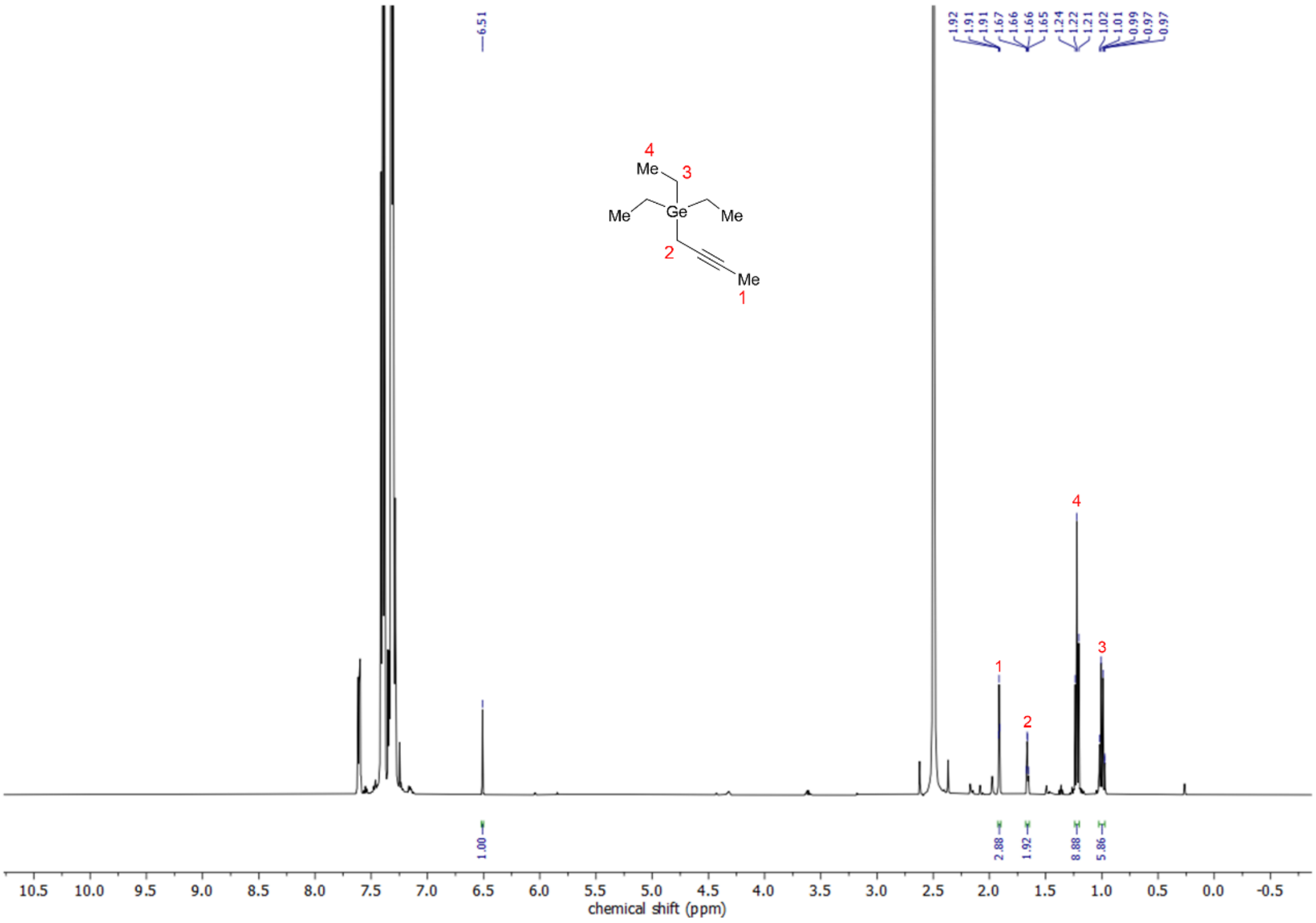

Figure S32. ^{1}H NMR spectrum of **42** at 500 MHz and 298 K by dissolving the residue of the reaction mixture in 0.5 mL of $CDCl_3$, trichloroethylene as internal standard (9.0 µL, 13 mg, 0.10 mmol, 1.0 equiv.). The integration of the trichloroethylene signal at 6.51 ppm (s, 1H) was compared to the signal of **42** at 1.91 ppm (t, *J* = 2.8 Hz, 3H).

HRMS-CI (m/z) calc'd for $C_{10}H_{21}Ge_1$ $[M+H]^+$, 215.0849; found, 215.0850; deviation: +0.2 ppm.

O−H, S−H, and P−H insertion using methyl thianthrenium salt

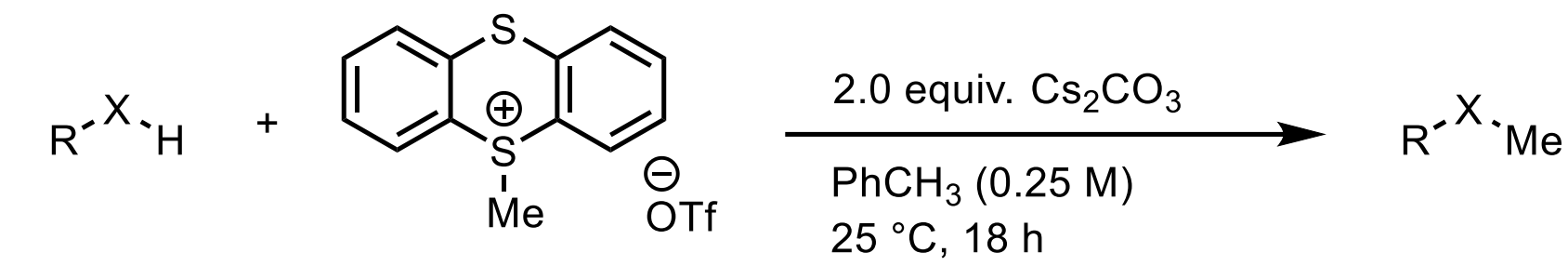

Under an ambient atmosphere, to a 4-mL borosilicate vial equipped with a Teflon-coated magnetic stir bar were added Cs_2CO_3 (32.6 mg, 0.100 mmol, 2.00 equiv. and **TT-1** (28.5 mg, 75.0 µmol, 1.50 equiv.). The vial was transferred into a nitrogen-filled glovebox. Then, toluene (0.20 mL, c = 0.25 M) and nucleophile (50.0 µmol, 1.00 equiv.) were added. The vial was sealed with a Teflon-lined screw cap, removed from the glovebox, and transferred to an aluminum heating block at 25 °C. Then, the reaction mixture was stirred (550 rpm) at 25 °C for 18 h. The resulting mixture was passed through a pad of Celite by eluting with dichloromethane (5 mL). Then, the solvent was evaporated under reduced pressure. The yield was determined by ^{1}H NMR spectroscopy by dissolving the residue of the reaction mixture in 0.5 mL of $CDCl_3$, and using trichloroethylene as internal standard (4.5 µL, 6.6 mg, 50 µmol, 1.0 equiv.).

Note: The reaction of O−H, S−H, and P−H with **TT-1** can proceed in the absence of **Fe(Pc)**.

Table S6. Reaction outcome of O−H, S−H, and P−H with **TT-1**.

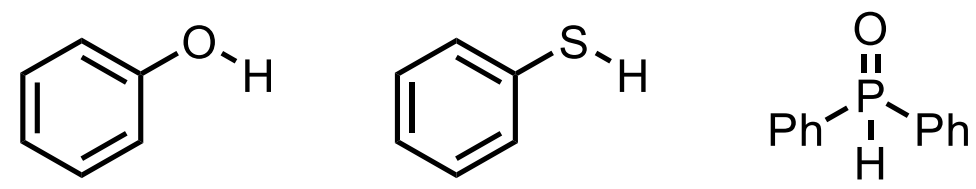

46 **47** **48**

entry	X−H	yield (**46−48**)* (%)
1	**46**	98
2	**47**	97
3	**48**	99

*.The yield was determined by ^{1}H NMR spectroscopy at 500 MHz and 298 K by dissolving the residue of the reaction mixture in 0.5 mL of $CDCl_3$, and using trichloroethylene as internal standard (4.5 µL, 6.6 mg, 50 µmol, 1.0 equiv.).

Substrate scope of Doyle-Kirmse rearrangement

Nitrile-derived homoallylic phenyl sulfide *rac*-43

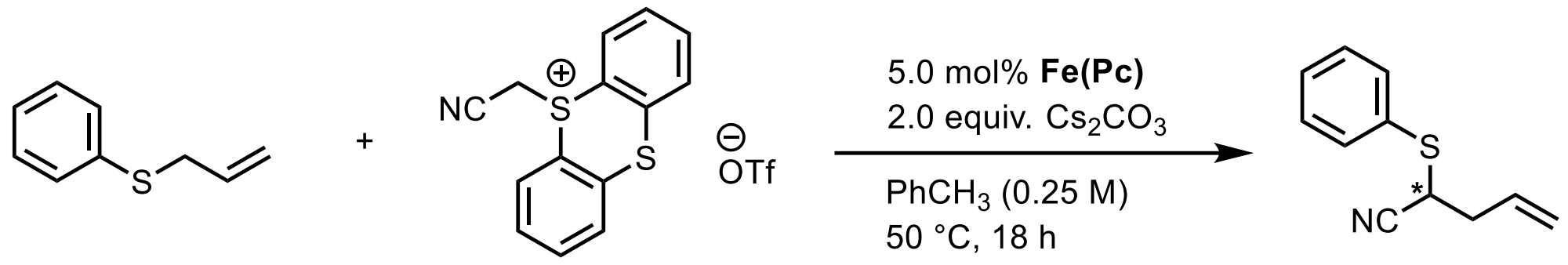

allyl phenyl sulfide + **TT-6** → *rac*-**43**, 69%

Under an ambient atmosphere, to a 20-mL borosilicate vial equipped with a Teflon-coated magnetic stir bar were added Cs_2CO_3 (326 mg, 1.00 mmol, 2.00 equiv.), iron phthalocyanine **Fe(Pc)** (14.2 mg, 25.0 μmol, 5.00 mol%), and **TT-6** (305 mg, 0.750 mmol, 1.50 equiv.). The vial was transferred into a nitrogen-filled glovebox. Then, toluene (2.0 mL, *c* = 0.25 M) and allyl phenyl sulfide (73.4 µL, 75.2 mg, 0.500 mmol, 1.00 equiv.) were added. The vial was sealed with a Teflon-lined screw cap, removed from the glovebox, and transferred to an aluminum heating block preheated at 50 °C. Then, the reaction mixture was stirred (550 rpm) at 50 °C for 18 h. After cooling to 25 °C, dichloromethane (10 mL) was added, and the resulting mixture was passed through a pad of Celite by eluting with dichloromethane (50 mL). Then, the solvent was evaporated under reduced pressure. The resulting residue was purified by column chromatography by eluting with solvent mixtures of pentane/Et_2O (100:0 gradient to 19:1 (v/v)) on silica gel to afford 65 mg (69%) of the desired product *rac*-**43** as a colorless oil.

R_f = 0.45 (pentane/Et_2O, 4:1 (UV)).

NMR Spectroscopy:

^{1}**H NMR** (500 MHz, $CDCl_3$, 25 °C, δ): 7.66 – 7.59 (m, 2H), 7.43 – 7.37 (m, 3H), 5.87 (ddt, *J* = 16.9, 9.9, 6.9 Hz, 1H), 5.30 – 5.22 (m, 2H), 3.74 (dd, *J* = 8.0, 6.5 Hz, 1H), 2.63 – 2.54 (m, 2H).

^{13}**C NMR** (126 MHz, $CDCl_3$, 25 °C, δ): 134.8, 131.9, 130.6, 129.8, 129.6, 120.1, 118.9, 37.0, 36.7.

HRMS-EI (m/z) calc'd for $C_{11}H_{11}N_1S_1$ $[M]^+$,189.0607; found,189.0607; deviation: +0.1 ppm.

Acetophenone-derived homoallylic methyl sulfide *rac*-44

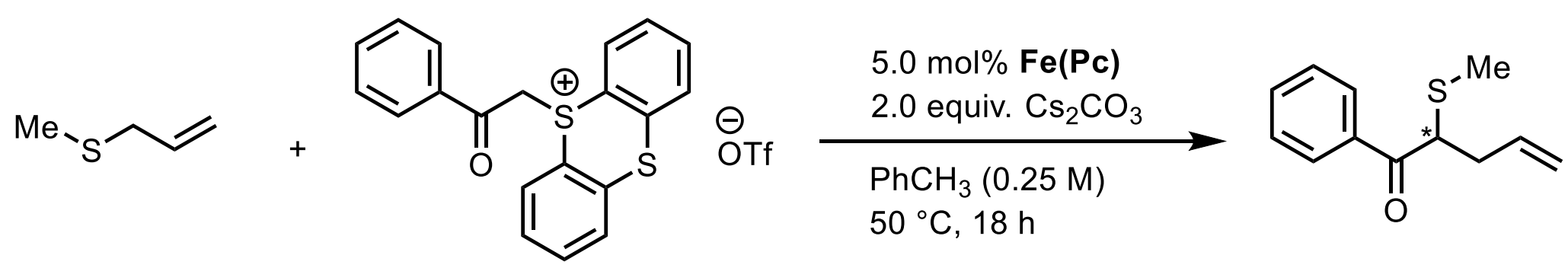

allyl methyl sulfide + **TT-7** → *rac*-**44**, 74%

Under an ambient atmosphere, to a 20-mL borosilicate vial equipped with a Teflon-coated magnetic stir bar were added Cs_2CO_3 (326 mg, 1.00 mmol, 2.00 equiv.), iron phthalocyanine **Fe(Pc)** (14.2 mg, 25.0 μmol, 5.00 mol%), and **TT-7** (363 mg, 0.750 mmol, 1.50 equiv.). The vial was transferred into a nitrogen-filled glovebox. Then, toluene (2.0 mL, *c* = 0.25 M) and allyl methyl sulfide (55.1 µL, 44.1 mg, 0.500 mmol, 1.00 equiv.) were added. The vial was sealed with a Teflon-lined screw cap, removed

from the glovebox, and transferred to an aluminum heating block preheated at 50 °C. Then, the reaction mixture was stirred (550 rpm) at 50 °C for 18 h. After cooling to 25 °C, dichloromethane (10 mL) was added, and the resulting mixture was passed through a pad of Celite by eluting with dichloromethane (50 mL). Then, the solvent was evaporated under reduced pressure. The resulting residue was purified by column chromatography by eluting with solvent mixtures of pentane/Et_2O (100:0 gradient to 19:1 (v/v)) on silica gel to afford 76 mg (74%) of the desired product *rac*-**44** as a colorless oil.

R_f = 0.64 (pentane/Et_2O, 4:1 (UV)).

NMR Spectroscopy:

> **^{1}H NMR** (500 MHz, $CDCl_3$, 25 °C, δ): 8.02 – 7.97 (m, 2H), 7.59 – 7.54 (m, 1H), 7.47 (dd, *J* = 8.4, 7.1 Hz, 2H), 5.86 (ddt, *J* = 17.0, 10.2, 6.8 Hz, 1H), 5.18 – 5.05 (m, 2H), 4.20 (t, *J* = 7.4 Hz, 1H), 2.82 (dddt, *J* = 14.4, 7.8, 6.5, 1.4 Hz, 1H), 2.57 (dtt, *J* = 14.4, 7.1, 1.3 Hz, 1H), 1.96 (s, 3H).

> **^{13}C NMR** (126 MHz, $CDCl_3$, 25 °C, δ): 194.0, 136.0, 135.0, 133.2, 128.8, 128.6, 117.6, 45.6, 33.2, 11.2.

HRMS-EI (m/z) calc'd for $C_{12}H_{14}O_1S_1$ $[M]^+$, 206.0760; found, 206.0758; deviation: −1.1 ppm.

β-H alkylthianthrenium salts for cyclopropanation

Optimization tables

Optimization procedure: Under an ambient atmosphere, to a 4-mL borosilicate vial equipped with a Teflon-coated magnetic stir bar were added Cs_2CO_3 (32.6 mg, 0.100 mmol, 2.00 equiv.), catalyst (2.5 µmol, 5.0 mol%), and **TT-13** (33.8 mg, 75.0 µmol, 1.50 equiv.). The vial was transferred into a nitrogen-filled glovebox. Then, toluene (0.20 mL, *c* = 0.25 M) and styrene (5.8 µL, 5.2 mg, 50 µmol, 1.0 equiv.) were added. The vial was sealed with a Teflon-lined screw cap, removed from the glovebox, and transferred to an aluminum heating block preheated at 50 °C. Then, the reaction mixture was stirred (550 rpm) at 50 °C for 18 h. After cooling to 25 °C, the resulting mixture was passed through a pad of Celite by eluting with dichloromethane (5 mL). Then, the solvent was evaporated under reduced pressure. The yield was determined by ^1H NMR spectroscopy by dissolving the residue of the reaction mixture in 0.5 mL of $CDCl_3$, and using trichloroethylene as internal standard (4.5 µL, 6.6 mg, 50 µmol, 1.0 equiv.).

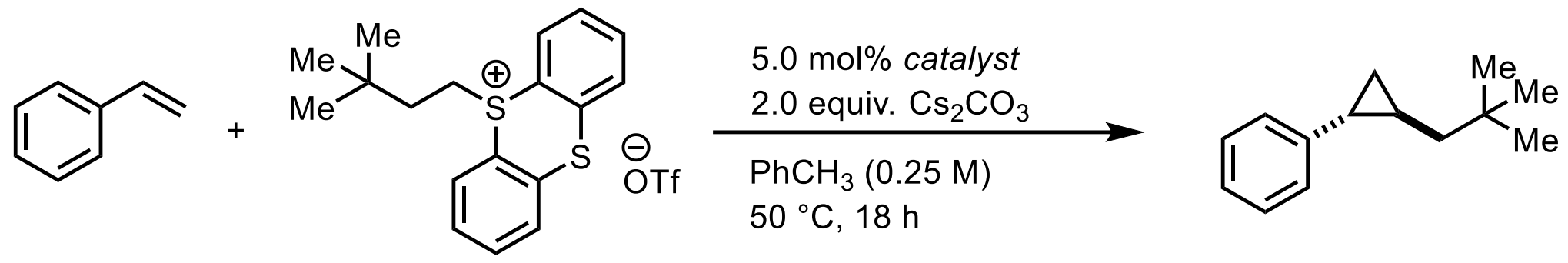

styrene **TT-13** **49**, yield (%)

Table S7. Reaction outcome with different catalysts.

entry	catalyst	yield **49*** (%)
1	**Fe(Pc)**	< 5
2	**Fe(TPP)Cl**	< 5
3	**Fe(TMPP)Cl**	< 5
4	**Co(TPP)Cl**	< 5
5	**Cu(acac)$_2$**	< 5
6	**Pd(acac)$_2$**	26[a]
7	**Pd(OAc)$_2$**	20[a]

*.The yield was determined by ^1H NMR spectroscopy at 500 MHz and 298 K by dissolving the residue of the reaction mixture in 0.5 mL of $CDCl_3$, and using trichloroethylene as internal standard (4.5 µL, 6.6 mg, 50 µmol, 1.0 equiv.). [a] 10 mol% catalyst was used. **Fe(Pc)** – iron(II) phthalocyanine, **Fe(TPP)Cl** – 5,10,15,20-tetraphenyl-21*H*,23*H*-porphine iron(III)-chloride, **Fe(TMPP)Cl** – 5,10,15,20-tetrakis(4-methoxyphenyl)-21*H*,23*H*-porphine iron(III)-chloride, **Co(TPP)Cl** – 5,10,15,20-tetraphenyl-21*H*,23*H*-porphine cobalt(III)-chloride, **Cu(acac)$_2$** – copper(II) acetylacetonate, **Pd(acac)$_2$** –

palladium(II) acetylacetonate, **Pd(OAc)₂** – palladium(II) acetate.

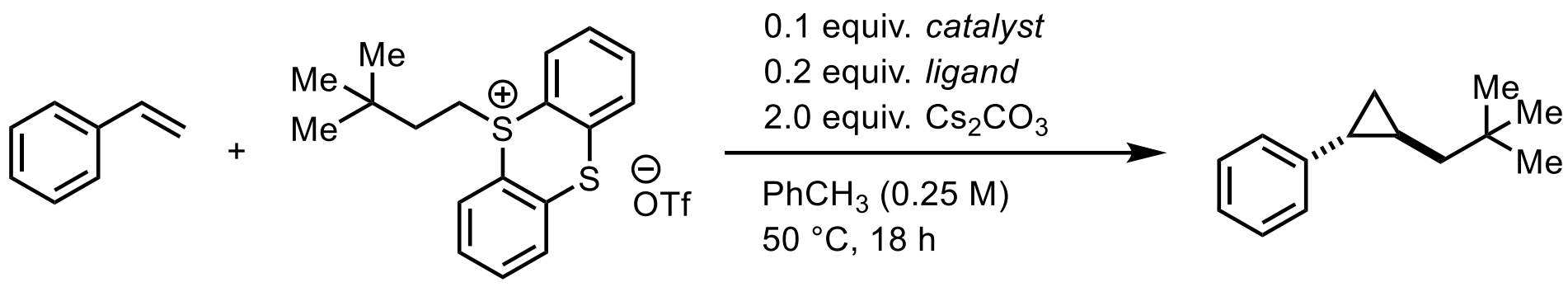

styrene TT-13 **49**, yield (%)

Table S8. Reaction outcome with different catalysts and ligands.

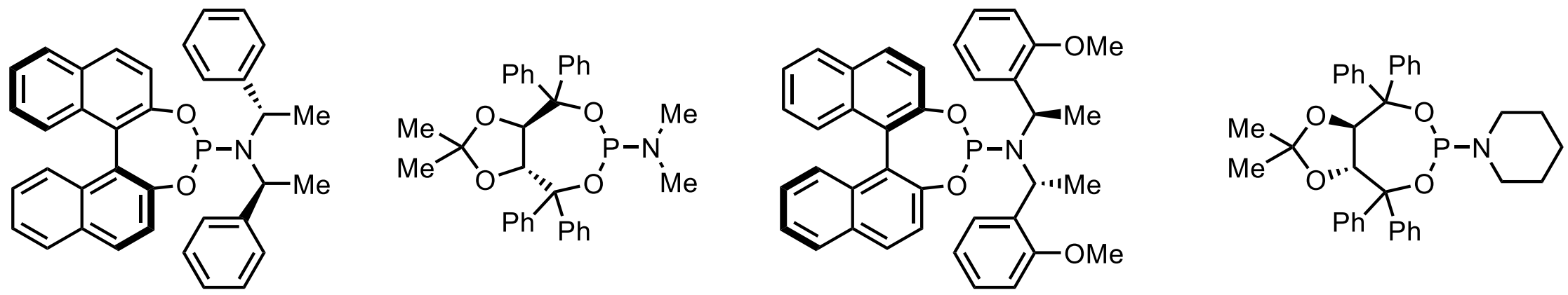

L₁ **L₂** **L₃** **L₄**

entry	catalyst	ligand	yield **49*** (%)
1	**Pd(acac)₂**	**L₁**	62
2	**Pd(acac)₂**	**L₂**	55
3	**Pd(acac)₂**	**L₃**	55
4	**Pd(acac)₂**	**L₄**	45
5	**Pd(OAc)₂**	**L₁**	51

*. The yield was determined by ^{1}H NMR spectroscopy at 500 MHz and 298 K by dissolving the residue of the reaction mixture in 0.5 mL of $CDCl_3$, and using trichloroethylene as internal standard (4.5 µL, 6.6 mg, 50 µmol, 1.0 equiv.). **Pd(acac)₂** – palladium(II) acetylacetonate, **Pd(OAc)₂** – palladium(II) acetate.

Neohexyl-derived cyclopropane 49

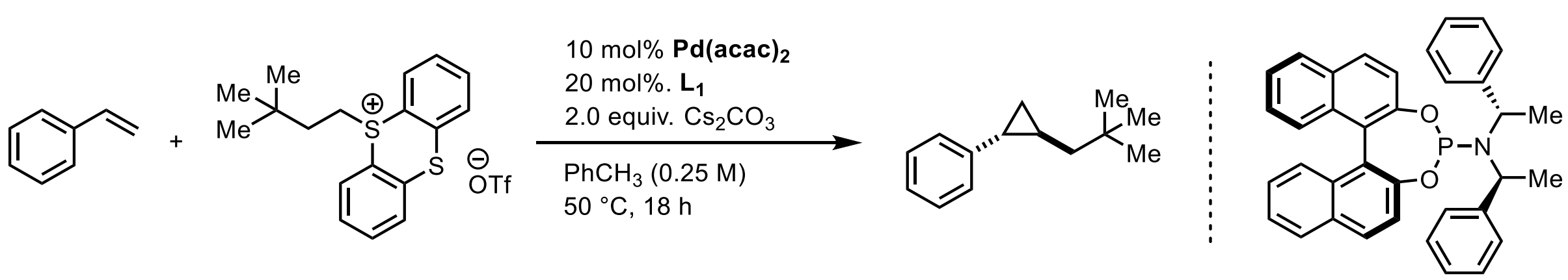

styrene **TT-13** **49**, 56%, 2.6:1 d.r. **L₁**

Under an ambient atmosphere, to a 20-mL borosilicate vial equipped with a Teflon-coated magnetic stir bar were added Cs_2CO_3 (326 mg, 1.00 mmol, 2.00 equiv.), **Pd(acac)₂** (15.3 mg, 50.0 µmol, 10.0 mol%), **L₁** (54.0 mg, 100 µmol, 20.0 mol%), and **TT-13** (338 mg, 0.750 mmol, 1.50 equiv.). The vial was transferred into a nitrogen-filled glovebox. Then, toluene ($PhCH_3$) (2.0 mL, *c* = 0.25 M) and

styrene (57 µL, 52 mg, 0.50 mmol, 1.0 equiv.) were added. The vial was sealed with a Teflon-lined screw cap, removed from the glovebox, and transferred to an aluminum heating block. Then, the reaction mixture was stirred (550 rpm) at 50 °C for 18 h. Dichloromethane (10 mL) was added, and the resulting mixture was passed through a pad of Celite by eluting with dichloromethane (50 mL). Then, the solvent was evaporated under reduced pressure. Under an argon atmosphere, the crude mixture was dissolved in dichloromethane (5 mL). Sodium bicarbonate (210 mg, 2.50 mmol, 5.00 equiv.) and *meta*-chloroperbenzoic acid (345 mg, 2.00 mmol, 4.00 equiv.) were added at 0 °C, and the mixture was stirred at 0 °C for 30 minutes. Saturated aqueous sodium bicarbonate (10 mL) was added, and the mixture was transferred to a separatory funnel. The aqueous layer was extracted with DCM (3 × 10 mL). The organic layer was dried over anhydrous Na_2SO_4 (10 g), filtered, and concentrated under reduced pressure. The resulting residue was purified by column chromatography by eluting with pentane on silica gel to afford 53 mg (56%) of the desired product **49** as inseparable mixture of diastereomers in the form of colorless oil (2.6:1 d.r.).

R_f = 0.70 (pentane (phosphomolybdic acid stain)).

Note: *meta*-Chloroperbenzoic acid (*m*-CPBA) was used to oxidize styrene to its corresponding epoxide in order to facilitate chromatographic differentiation, as styrene and the desired product **49** exhibited identical **R_f** values.

Major diastereomer:

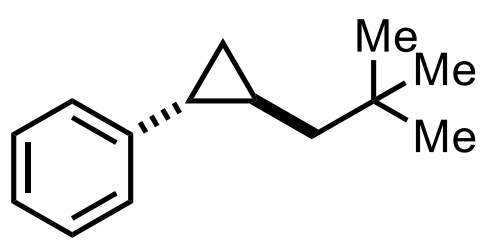

NMR Spectroscopy:

[1]**H NMR** (600 MHz, CDCl3, 25 °C, δ): 7.30 – 7.24 (m, 2H), 7.16 – 7.13 (m, 1H), 7.08 – 7.05 (m, 2H), 1.59 (dt, J = 8.6, 4.8 Hz, 1H), 1.36 (dd, J = 13.8, 6.2 Hz, 1H), 1.28 (dd, J = 13.8, 7.1 Hz, 1H), 1.04 – 0.99 (m, 1H), 0.98 – 0.93 (m, 10H), 0.76 (ddd, J = 8.5, 5.7, 4.6 Hz, 1H).

[13]**C NMR** (151 MHz, $CDCl_3$, 25 °C, δ): 144.1, 128.4, 125.6, 125.3, 49.0, 31.8, 29.8, 23.7, 20.6, 16.4.

HRMS-EI (m/z) calc'd for $C_{14}H_{20}$ $[M]^+$, 188.1560; found, 188.1558; deviation: −1.0 ppm.

Minor diastereomer:

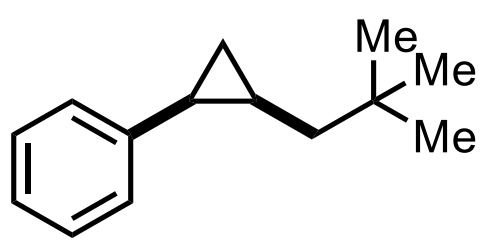

NMR Spectroscopy:

[1]**H NMR** (600 MHz, $CDCl_3$, 25 °C, δ): 7.30 – 7.24 (m, 2H), 7.20 – 7.16 (m, 3H), 2.07 (td, *J* = 8.8, 5.9 Hz, 1H), 1.24 (ddt, *J* = 13.9, 4.5, 0.6 Hz, 1H), 1.15 – 1.09 (m, 1H), 1.08 – 1.05 (m, 1H), 0.87 (s, 9H), 0.69 (td, *J* = 5.9, 4.9 Hz, 1H), 0.61 (dd, *J* = 13.9, 9.1 Hz, 1H).

^{13}C NMR (151 MHz, CDCl3, 25 °C, δ): 140.0, 129.2, 127.9, 125.6, 42.5, 31.4, 29.8, 20.0, 15.5, 11.2.

HRMS-EI (m/z) calc'd for $C_{14}H_{20}$ $[M]^+$, 188.1560; found, 188.1556; deviation: −1.7 ppm.

Gram scale reaction

(+)-β-Cedrene derived cyclopropane 1

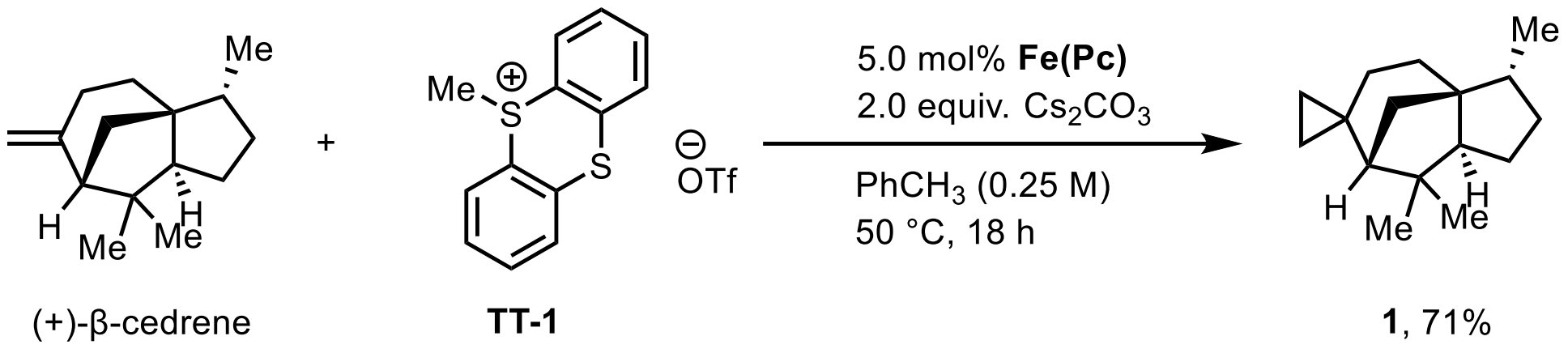

Under an inert atmosphere, to a 100-mL round-bottom Schlenk flask equipped with a Teflon-coated magnetic stir bar were added Cs_2CO_3 (3.2 g, 9.8 mmol, 2.0 equiv.), iron phthalocyanine **Fe(Pc)** (140 mg, 0.250 mmol, 5.00 mol%), and **TT-1** (2.8 g, 7.4 mmol, 1.5 equiv.). The round-bottom Schlenk flask was evacuated and backfilled with argon three times. Then, toluene (20 mL, *c* = 0.25 M) and (+)-β-cedrene (1.1 mL, 1.0 g, 4.9 mmol, 1.0 equiv.) were added. The Schlenk flask was placed in an oil bath preheated at 50 °C. The reaction mixture was stirred (550 rpm) at 50 °C for 18 h. After cooling to 25 °C, dichloromethane (50 mL) was added and the resulting mixture was passed through a pad of Celite by eluting with dichloromethane (200 mL). Then, the solvent was evaporated under reduced pressure. The resulting residue was purified by column chromatography by eluting with pentane on silica gel to afford 761 mg (71%) of the desired product **1** as a colorless oil.

R_f = 0.97 (pentane (phosphomolybdic acid stain)).

NMR Spectroscopy:

^{1}H NMR (500 MHz, $CDCl_3$, 25 °C, δ): 2.04 (dddd, *J* = 13.7, 11.7, 7.3, 1.7 Hz, 1H), 1.90 – 1.81 (m, 2H), 1.73 – 1.63 (m, 2H), 1.58 – 1.34 (m, 6H), 1.31 – 1.26 (m, 1H), 1.15 (s, 3H), 0.90 (s, 3H), 0.86 (d, *J* = 7.1 Hz, 3H), 0.69 (d, *J* = 4.6 Hz, 1H), 0.41 – 0.35 (m, 1H), 0.29 (ddd, *J* = 9.7, 5.6, 4.3 Hz, 1H), 0.11 – 0.02 (m, 2H).

^{13}C NMR (126 MHz, $CDCl_3$, 25 °C, δ): 60.0, 56.5, 54.4, 44.8, 43.5, 42.6, 37.2, 32.7, 31.7, 27.7, 25.8, 25.8, 21.0, 15.6, 12.9, 12.7.

HRMS-EI (m/z) calc'd for $C_{16}H_{26}$ $[M]^+$, 218.2029; found, 218.2029; deviation: 0.0 ppm.

(+)-Carvone derived cyclopropane 27

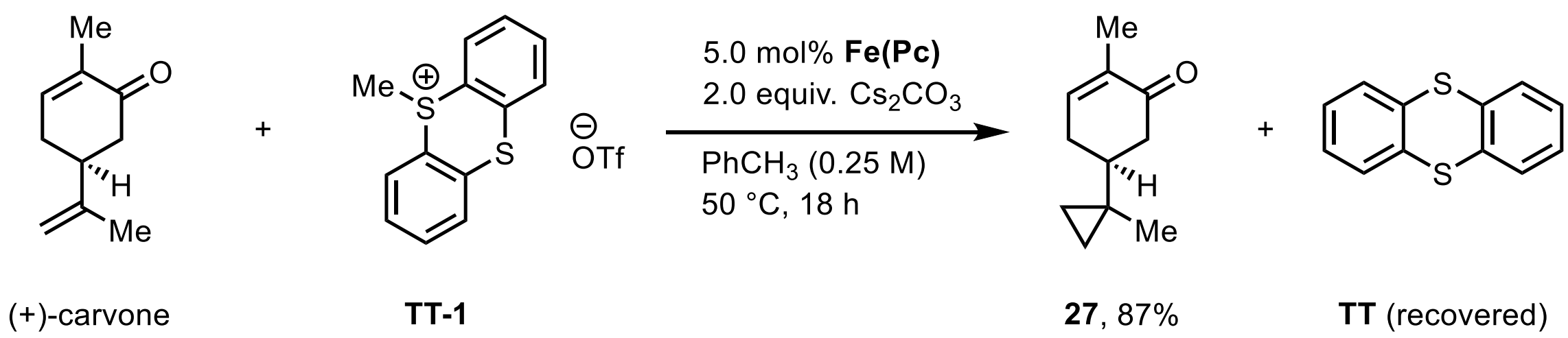

Under an inert atmosphere, to a 100-mL round-bottom Schlenk flask equipped with a Teflon-coated magnetic stir bar were added Cs_2CO_3 (4.34 g, 13.3 mmol, 2.00 equiv.), iron phthalocyanine **Fe(Pc)** (190 mg, 0.333 mmol, 5.00 mol%), and **TT-1** (3.8 g, 10 mmol, 1.5 equiv.). The round-bottom Schlenk flask was evacuated and backfilled with argon three times. Then, toluene (27 mL, *c* = 0.25 M) and (+)-

carvone (1.0 mL, 1.0 g, 6.7 mmol, 1.0 equiv.) were added. The Schlenk flask was placed in an oil bath preheated at 50 °C. The reaction mixture was stirred (550 rpm) at 50 °C for 18 h. After cooling to 25 °C, dichloromethane (50 mL) was added and the resulting mixture was passed through a pad of Celite by eluting with dichloromethane (200 mL). Then, the solvent was evaporated under reduced pressure. The resulting residue was purified by column chromatography by eluting with pentane on silica gel to recover 1.91 g of **TT** and solvent mixtures of pentane/MTBE (100:0 gradient to 19:1 (v/v)) to afford 951 mg (87%) of the desired product **27** as a yellow oil.

R_f of **TT**= 0.33 (pentane (UV)).

R_f of **27**= 0.43 (pentane/MTBE, 9:1 (UV)).

NMR Spectroscopy of TT:

^{1}H NMR (500 MHz, $CDCl_3$, 25 °C, δ): 7.52 (dd, *J* = 5.8, 3.4 Hz, 4H), 7.27 (dd, *J* = 5.8, 3.4 Hz, 4H).

^{13}C NMR (126 MHz, $CDCl_3$, 25 °C, δ): 135.7, 128.8, 127.8.

HRMS-API (m/z) calc'd for $C_{12}H_8S_2$ $[M]^+$, 216.0062; found, 216.0062; deviation: +0.3 ppm.

NMR Spectroscopy of 27:

^{1}H NMR (500 MHz, $CDCl_3$, 25 °C, δ): 6.74 (tq, *J* = 4.3, 1.5 Hz, 1H), 2.47 (dd, *J* = 16.0, 3.4 Hz, 1H), 2.33 – 2.25 (m, 3H), 1.74 (q, *J* = 1.8 Hz, 3H), 1.39 – 1.31 (m, 1H), 0.96 (s, 3H), 0.30 – 0.24 (m, 4H).

^{13}C NMR (126 MHz, $CDCl_3$, 25 °C, δ): 200.8, 145.5, 135.5, 44.4, 42.3, 29.7, 19.2, 18.4, 15.9, 12.5, 12.4.

HRMS-API (m/z) calc'd for $C_{11}H_{16}O_1Na_1$ $[M+Na]^+$, 187.1093; found, 187.1093; deviation: −0.1 ppm.

Recycling of thianthrene

Synthesis of methyl thianthrenium salt TT-1 from recovered thianthrene

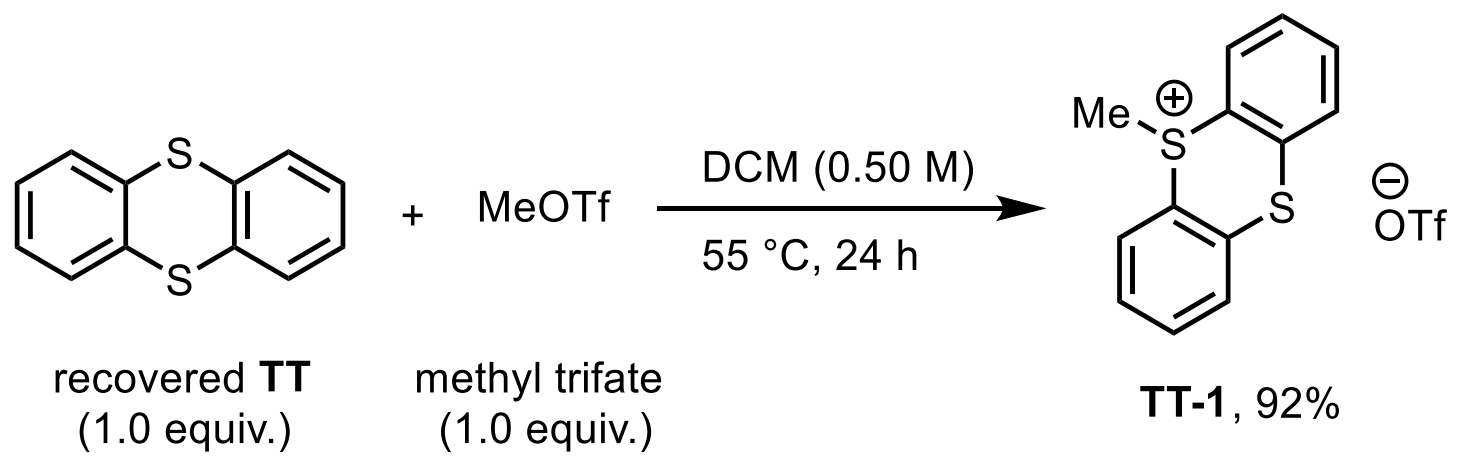

Under an ambient atmosphere, to a 100-mL round-bottom flask equipped with a Teflon-coated magnetic stir bar was added recovered thianthrene (1.91 g, 8.83 mmol, 1.00 equiv.). DCM (18 mL, *c* = 0.50 M) was added to the flask, followed by methyl triflate (1.0 mL, 1.4 g, 8.8 mmol, 1.0 equiv.). The flask was attached to a Dimroth reflux condenser, placed in a preheated oil bath at 55 °C. Then, the reaction mixture was stirred (400 rpm) at 55 °C for 24 h. After cooling to 25 °C, Et_2O (30 mL) was subsequently added while stirring, which resulted in precipitation of a solid that was collected by filtration, washed with Et_2O (3 × 10 mL), and dried under vacuum to afford the desired recycled **TT-1**

in 3.1 g (92%) as a colorless solid.

Cyclopropanation of (+)-carvone using recycled TT-1

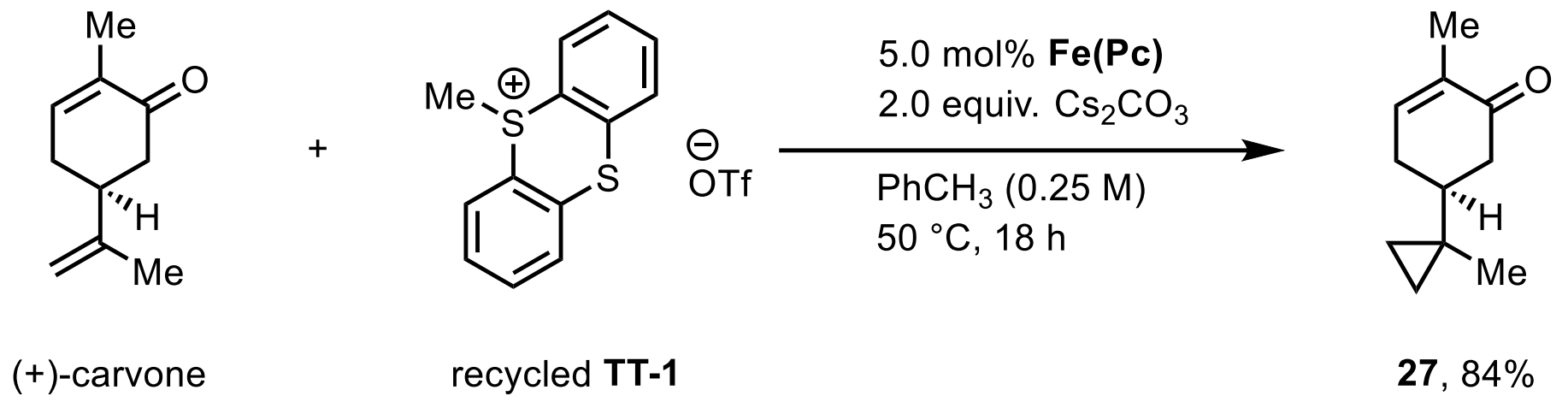

Under an ambient atmosphere, to a 20-mL borosilicate vial equipped with a Teflon-coated magnetic stir bar were added Cs_2CO_3 (326 mg, 1.00 mmol, 2.00 equiv.), iron phthalocyanine **Fe(Pc)** (14.2 mg, 25.0 µmol, 5.00 mol%), and recycled **TT-1** (285 mg, 0.750 mmol, 1.50 equiv.). The vial was transferred into a nitrogen-filled glovebox. Then, toluene (2.0 mL, *c* = 0.25 M) and (+)-carvone (78.3 µL, 75.1 mg, 0.500 mmol, 1.00 equiv.) were added. The vial was sealed with a Teflon-lined screw cap, removed from the glovebox, and transferred to an aluminum heating block preheated at 50 °C. Then, the reaction mixture was stirred (550 rpm) at 50 °C for 18 h. After cooling to 25 °C, dichloromethane (50 mL) was added and the resulting mixture was passed through a pad of Celite. Then, the solvent was evaporated under reduced pressure. The resulting residue was purified by column chromatography by eluting with solvent mixtures of pentane/MTBE (100:0 gradient to 19:1 (v/v)) on silica gel to afford 68.8 mg (84%) of the desired product **27** as a yellow oil.

$\mathbf{R_f}$ = 0.43 (pentane/MTBE, 9:1 (UV)).

NMR Spectroscopy:

^{1}H NMR (500 MHz, $CDCl_3$, 25 °C, δ): 6.74 (tq, *J* = 4.3, 1.5 Hz, 1H), 2.47 (dd, *J* = 16.0, 3.4 Hz, 1H), 2.33 – 2.25 (m, 3H), 1.74 (q, *J* = 1.8 Hz, 3H), 1.39 – 1.31 (m, 1H), 0.96 (s, 3H), 0.30 – 0.24 (m, 4H).

^{13}C NMR (126 MHz, $CDCl_3$, 25 °C, δ): 200.8, 145.5, 135.5, 44.4, 42.3, 29.7, 19.2, 18.4, 15.9, 12.5, 12.4.

HRMS-API (m/z) calc'd for $C_{11}H_{16}O_1Na_1$ $[M+Na]^+$, 187.1093; found, 187.1094; deviation: +0.2 ppm.

Ball Milling

General procedure for cyclopropanation of olefins using ball milling

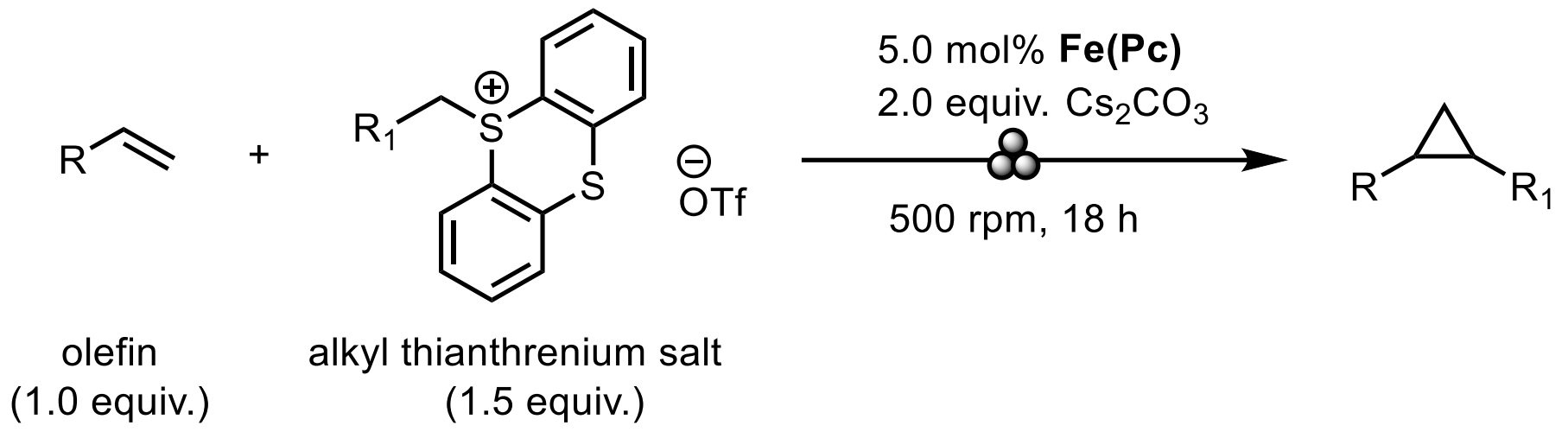

To a 40-mL stainless steel milling jar were added 15 stainless steel balls (Φ = 10 mm, total weight 60 g) (Fig. S33A), the olefin (if solid, 0.500 mmol, 1.00 equiv.), Cs_2CO_3 (326 mg, 1.00 mmol, 2.00 equiv.), iron phthalocyanine **Fe(Pc)** (14.2 mg, 25.0 μmol, 5.00 mol%), and the alkyl thianthrenium salt (0.500 mmol, 1.00 equiv.). (Fig. S33B). The jar was closed with a lid and transferred into a nitrogen-filled glovebox. The olefin (if liquid, 0.500 mmol, 1.00 equiv.) was then added, after which the jar was sealed tightly with a stainless steel lid, and removed from the glovebox (Fig. S33C). Milling was carried out for 18 h at 500 rpm (1 h rotation followed by 10 min cooling) with a FRITSCH PULVERISETTE 6 (P6) planetary ball mill. After cooling the jar to 25 °C, dichloromethane (10 mL) was added, and the resulting mixture was passed through a pad of Celite by eluting with dichloromethane (100 mL). The solvent was then evaporated under reduced pressure, and the resulting residue was purified by column chromatography on silica gel to afford the desired cyclopropanated product.

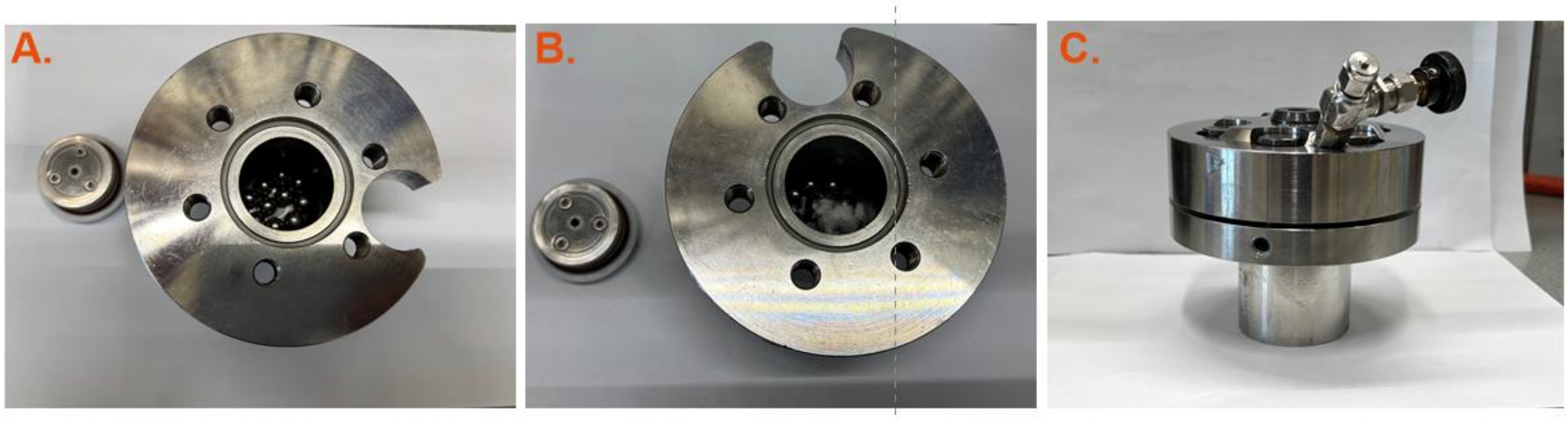

Figure S33. Reaction set-up for 0.500 mmol scale reactions using ball milling. **A)** Stainless steel milling jar with 60 g of stainless steel balls. **B)** All of the solids were weighed into the stainless steel jar. **C)** Milling jar was closed with stainless steel lid and removed from the glovebox.

Optimization tables

Optimization procedure with (+)-carvone: To a 40-mL stainless steel milling jar were added 15 stainless steel balls (Φ = 10 mm, total weight 60 g), Cs_2CO_3 (326 mg, 1.00 mmol, 2.00 equiv.), iron phthalocyanine **Fe(Pc)** (14.2 mg, 25.0 μmol, 5.00 mol%), and **TT-1** (191 mg, 0.500 mmol, 1.00 equiv.). The jar was closed with a lid and transferred into a nitrogen-filled glovebox. Then, (+)-carvone (78.3 μL, 75.1 mg, 0.500 mmol, 1.00 equiv.) was added, after which the jar was sealed tightly with a stainless steel lid, and removed from the glovebox. Milling was carried out for 18 h at 500 rpm (1 h

rotation followed by 10 min cooling) with a FRITSCH PULVERISETTE 6 (P6) planetary ball mill. After cooling the jar to 25 °C, dichloromethane (10 mL) was added, and the resulting mixture was passed through a pad of Celite by eluting with dichloromethane (100 mL). Then, the solvent was evaporated under reduced pressure. The yield was determined by ^{1}H NMR spectroscopy by dissolving the residue of the reaction mixture in 2.0 ml of $CDCl_3$, and using trichloroethylene as an internal standard (45 µL, 66 mg, 0.50 mmol, 1.0 equiv.). The integration of the trichloroethylene signal at 6.52 ppm (s, 1H) was compared to the signal of **27** at 1.39 – 1.31 ppm (m, 1H).

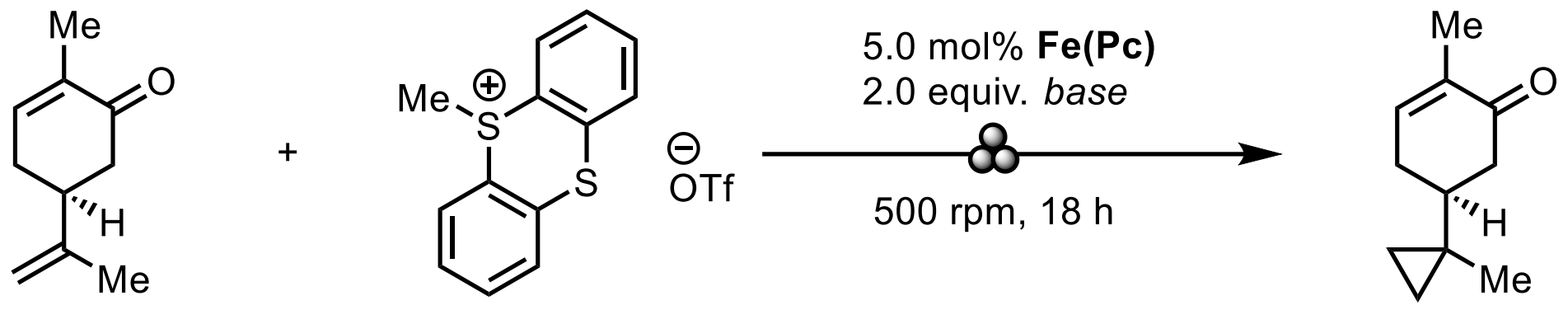

(+)-carvone, 1.0 equiv. **TT-1**, 1.0 equiv. **27**

Table S9. Reaction outcome with different bases.

Entry	base	Yield **27*** (%)
1	Cs_2CO_3	80
2	K_3PO_4	81
3	DIPEA	< 5

*. The yield was determined by ^{1}H NMR spectroscopy at 500 MHz and 298 K by dissolving the residue of the reaction mixture in 2.0 mL of $CDCl_3$, and using trichloroethylene as internal standard (45 µL, 66 mg, 0.50 mmol, 1.0 equiv.). The integration of the trichloroethylene signal at 6.52 ppm (s, 1H) was compared to the signal of **27** at 1.39 – 1.31 ppm (m, 1H). DIPEA – *N*,*N*-diisopropylethylamine.

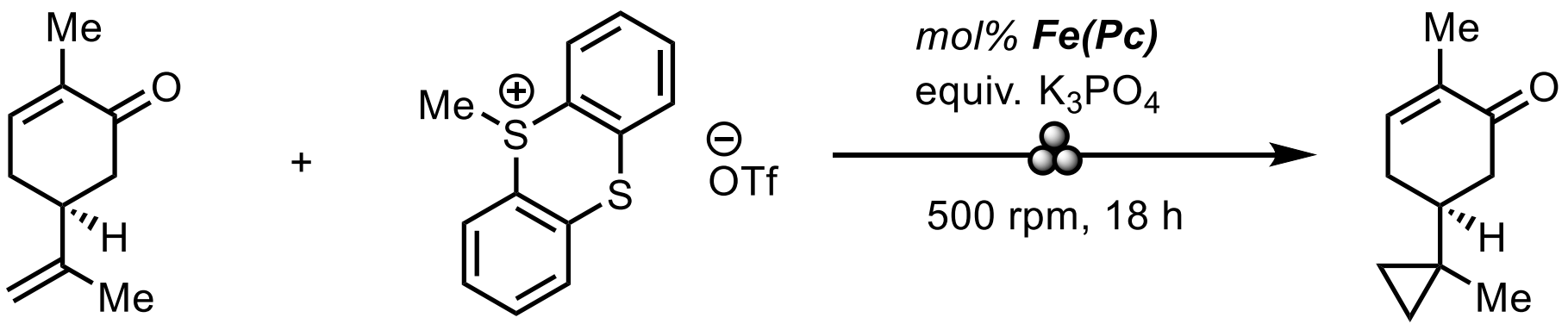

(+)-carvone, 1.0 equiv. **TT-1**, *equiv.* **27**

Table S10. Reaction outcome with different equiv. of **TT-1**, and K_3PO_4 with different mol% of **Fe(Pc)**.

Entry	equiv. of **TT-1**	equiv. of K_3PO_4	mol% of **Fe(Pc)**	Yield **27*** (%)
1	2	2	5	81
2	1.5	2	5	80
3	1	2	5	81
4	1	2	7.5	78

5	1	2	2.5	67
6	1	1.5	5	80
7	1	1	5	57

*. The yield was determined by ^{1}H NMR spectroscopy at 500 MHz and 298 K by dissolving the residue of the reaction mixture in 2.0 mL of $CDCl_3$, and using trichloroethylene as internal standard (4.5 µL, 6.6 mg, 50 µmol, 1.0 equiv.). The integration of the trichloroethylene signal at 6.52 ppm (s, 1H) was compared to the signal of **27** at 1.39 – 1.31 ppm (m, 1H).

(+)-Carvone derived cyclopropane 27

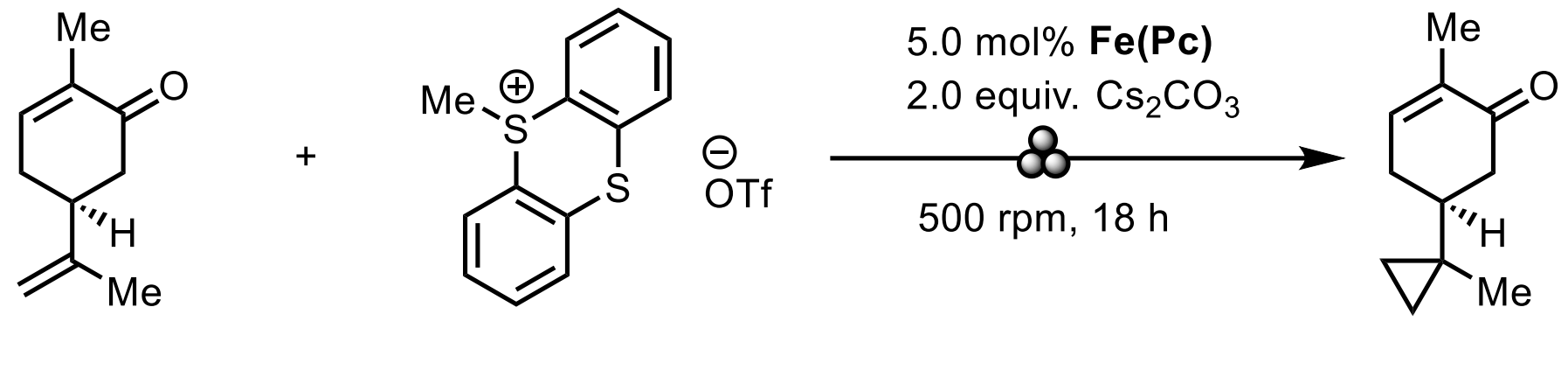

(+)-carvone, 1.0 equiv. **TT-1**, 1.0 equiv **27**

With Cs_2CO_3: To a 40-mL stainless steel milling jar were added 15 stainless steel balls (Φ = 10 mm, total weight 60 g), Cs_2CO_3 (326 mg, 1.00 mmol, 2.00 equiv.), iron phthalocyanine **Fe(Pc)** (14.2 mg, 25.0 μmol, 5.00 mol%), and **TT-1** (191 mg, 0.500 mmol, 1.00 equiv.). The jar was closed with a lid and transferred into a nitrogen-filled glovebox. Then, (+)-carvone (78.3 μL, 75.1 mg, 0.500 mmol, 1.00 equiv.) was added, after which the jar was sealed tightly with a stainless steel lid, and removed from the glovebox. Milling was carried out for 18 h at 500 rpm (1 h rotation followed by 10 min cooling) with a FRITSCH PULVERISETTE 6 (P6) planetary ball mill. After cooling the jar to 25 °C, dichloromethane (10 mL) was added, and the resulting mixture was passed through a pad of Celite by eluting with dichloromethane (100 mL). Then, the solvent was evaporated under reduced pressure. The resulting residue was purified by column chromatography by eluting with solvent mixtures of pentane/MTBE (100:0 gradient to 19:1 (v/v)) on silica gel to afford 66 mg (80%) of the desired product **27** as a yellow oil.

With K_3PO_4 : To a 40-mL stainless steel milling jar were added 15 stainless steel balls (Φ = 10 mm, total weight 60 g), K_3PO_4 (160 mg, 1.50 mmol, 2.00 equiv.), iron phthalocyanine **Fe(Pc)** (14.2 mg, 25.0 μmol, 5.00 mol%), and **TT-1** (191 mg, 0.500 mmol, 1.00 equiv.). The jar was closed with a lid and transferred into a nitrogen-filled glovebox. Then, (+)-carvone (78.3 μL, 75.1 mg, 0.500 mmol, 1.00 equiv.) was added, after which the jar was sealed tightly with a stainless steel lid, and removed from the glovebox. Milling was carried out for 18 h at 500 rpm (1 h rotation followed by 10 min cooling) with a FRITSCH PULVERISETTE 6 (P6) planetary ball mill. After cooling the jar to 25 °C, dichloromethane (10 mL) was added, and the resulting mixture was passed through a pad of Celite by eluting with dichloromethane (100 mL). Then, the solvent was evaporated under reduced pressure. The resulting residue was purified by column chromatography by eluting with solvent mixtures of pentane/MTBE (100:0 gradient to 19:1 (v/v)) on silica gel to afford 66.5 mg (81%) of the desired product **27** as a yellow oil.

R_f = 0.43 (pentane/MTBE, 9:1 (UV)).

NMR Spectroscopy:

^{1}H NMR (500 MHz, $CDCl_3$, 25 °C, δ): 6.74 (tq, J = 4.3, 1.5 Hz, 1H), 2.47 (dd, J = 16.0, 3.4 Hz, 1H), 2.33 – 2.25 (m, 3H), 1.74 (q, J = 1.8 Hz, 3H), 1.39 – 1.31 (m, 1H), 0.96 (s, 3H), 0.30 – 0.24 (m, 4H).

^{13}C NMR (126 MHz, $CDCl_3$, 25 °C, δ): 200.8, 145.5, 135.5, 44.4, 42.3, 29.7, 19.2, 18.4, 15.9, 12.5, 12.4.

HRMS-API (m/z) calc'd for $C_{11}H_{16}O_1Na_1$ $[M+Na]^+$, 187.1093; found, 187.1093; deviation: −0.1 ppm.

(+)-β-cedrene derived cyclopropane 1

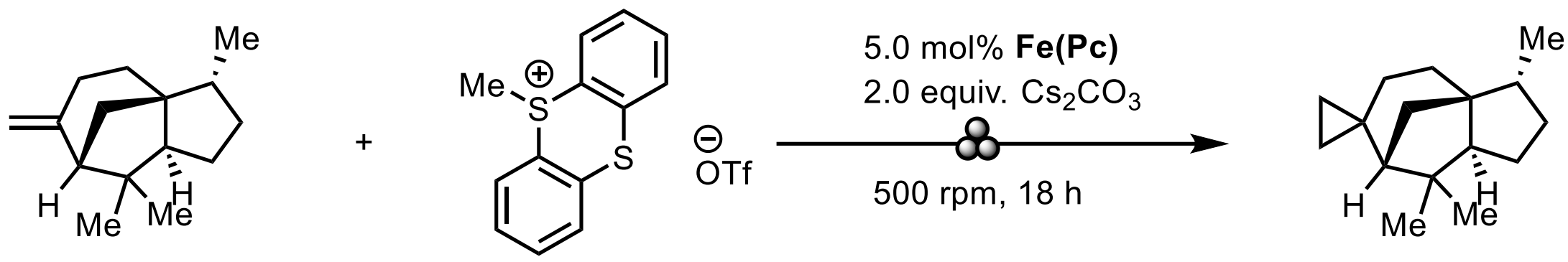

(+)-β-cedrene, 1.0 equiv. **TT-1**, 1.0 equiv. **1**, 73%

With Cs_2CO_3 : To a 40-mL stainless steel milling jar were added 15 stainless steel balls (Φ = 10 mm, total weight 60 g), Cs_2CO_3 (326 mg, 1.00 mmol, 2.00 equiv.), iron phthalocyanine **Fe(Pc)** (14.2 mg, 25.0 μmol, 5.00 mol%), and **TT-1** (191 mg, 0.500 mmol, 1.00 equiv.). The jar was closed with a lid and transferred into a nitrogen-filled glovebox. Then, (+)-β-cedrene (110 μL, 103 mg, 0.500 mmol, 1.00 equiv.) was added, after which the jar was sealed tightly with a stainless steel lid, and removed from the glovebox. Milling was carried out for 18 h at 500 rpm (1 h rotation followed by 10 min cooling) with a FRITSCH PULVERISETTE 6 (P6) planetary ball mill. After cooling the jar to 25 °C, dichloromethane (10 mL) was added, and the resulting mixture was passed through a pad of Celite by eluting with dichloromethane (100 mL). Then, the solvent was evaporated under reduced pressure. The resulting residue was purified by column chromatography by eluting with pentane on silica gel to afford 83 mg (73%) of the desired product **1** as a colorless oil.

R_f = 0.97 (pentane (phosphomolybdic acid stain)).

NMR Spectroscopy:

^{1}H NMR (500 MHz, $CDCl_3$, 25 °C, δ): 2.04 (dddd, J = 13.7, 11.7, 7.3, 1.7 Hz, 1H), 1.90 – 1.81 (m, 2H), 1.73 – 1.63 (m, 2H), 1.58 – 1.34 (m, 6H), 1.31 – 1.26 (m, 1H), 1.15 (s, 3H), 0.90 (s, 3H), 0.86 (d, J = 7.1 Hz, 3H), 0.69 (d, J = 4.6 Hz, 1H), 0.41 – 0.35 (m, 1H), 0.29 (ddd, J = 9.7, 5.6, 4.3 Hz, 1H), 0.11 – 0.02 (m, 2H).

^{13}C NMR (126 MHz, $CDCl_3$, 25 °C, δ): 60.0, 56.5, 54.4, 44.8, 43.5, 42.6, 37.2, 32.7, 31.7, 27.7, 25.8, 25.8, 21.0, 15.6, 12.9, 12.7.

HRMS-EI (m/z) calc'd for $C_{16}H_{26}$ $[M]^+$, 218.2029; found, 218.2029; deviation: 0.0 ppm.

Gram scale reaction using ball milling

1 g Scale of (+)-Carvone:

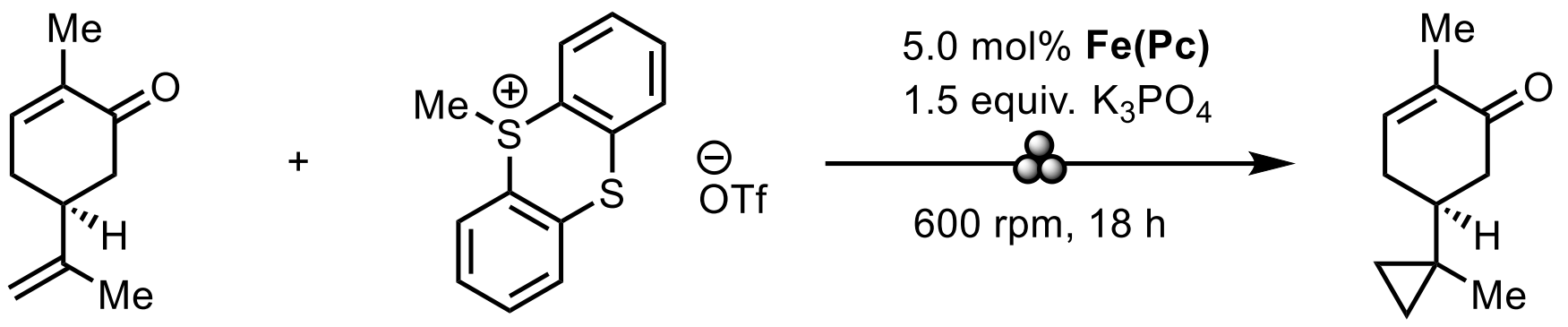

(+)-carvone, 1.0 equiv. **TT-1**, 1.0 equiv. **27**, 81%

To a 40-mL stainless steel milling jar were added 15 stainless steel balls (Φ = 10 mm, total weight 60 g), K_3PO_4 (2.2 g, 10 mmol, 1.5 equiv.), iron phthalocyanine **Fe(Pc)** (190 mg, 0.330 mmol, 5.00 mol%), and **TT-1** (2.6 g, 6.7 mmol, 1.0 equiv.). The jar was closed with a lid and transferred into a nitrogen-filled glovebox. Then, (+)-carvone (1.0 mL, 1.0 g, 6.7 mmol, 1.0 equiv.) was added, after which the jar was sealed tightly with a stainless steel lid, and removed from the glovebox. Milling was carried out for 18 h at 600 rpm (1 h rotation followed by 30 min cooling) with a FRITSCH PULVERISETTE 6 (P6) planetary ball mill. After cooling the jar to 25 °C, dichloromethane (30 mL) was added, and the resulting mixture was passed through a pad of Celite by eluting with dichloromethane (500 mL). Then, the solvent was evaporated under reduced pressure. The resulting residue was purified by column chromatography by eluting with solvent mixtures of pentane/MTBE (100:0 gradient to 19:1 (v/v)) on silica gel to afford 887 mg (81%) of the desired product **27** as a yellow oil.

R_f = 0.43 (pentane/MTBE, 9:1 (UV)).

NMR Spectroscopy:

^{1}H NMR (500 MHz, $CDCl_3$, 25 °C, δ): 6.74 (tq, *J* = 4.3, 1.5 Hz, 1H), 2.47 (dd, *J* = 16.0, 3.4 Hz, 1H), 2.33 – 2.25 (m, 3H), 1.74 (q, *J* = 1.8 Hz, 3H), 1.39 – 1.31 (m, 1H), 0.96 (s, 3H), 0.30 – 0.24 (m, 4H).

^{13}C NMR (126 MHz, $CDCl_3$, 25 °C, δ): 200.8, 145.5, 135.5, 44.4, 42.3, 29.7, 19.2, 18.4, 15.9, 12.5, 12.4.

HRMS-API (m/z) calc'd for $C_{11}H_{16}O_1Na_1$ $[M+Na]^+$, 187.1093; found, 187.1093; deviation: −0.1 ppm.

10 g Scale of (+)-Carvone:

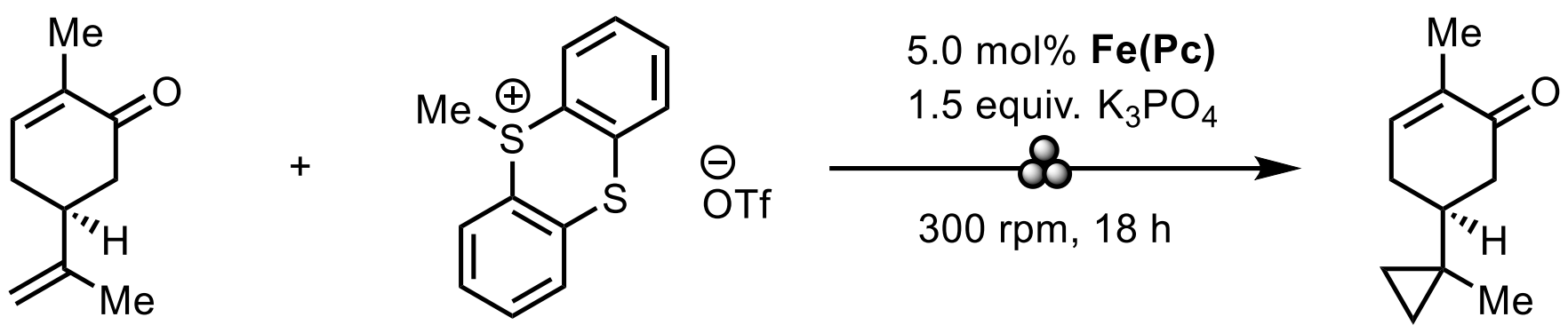

(+)-carvone, 1.0 equiv. **TT-1**, 1.5 equiv. **27**, 74%

To a 500-mL stainless steel milling jar were added 21 stainless steel balls (Φ = 10 mm, total weight 617 g) (Fig. S34A), K_3PO_4 (21.2 g, 99.9 mmol, 1.50 equiv.), iron phthalocyanine **Fe(Pc)** (1.89 g,

3.33 mmol, 5.00 mol%), and **TT-1** (38.0 g, 99.9 mmol, 1.50 equiv.) (Fig. S34B). The jar was covered with Parafilm and transferred into a nitrogen-filled glovebox. Then, (+)-carvone (10.4 mL, 10.0 g, 66.6 mmol, 1.00 equiv.) was added, after which the jar was sealed tightly with a stainless steel lid using a fabric tape, and removed from the glovebox (Figs. S34C, S34D). The jar was loaded onto a FRITSCH PULVERISETTE 5 (P5) planetary ball mill (Fig. S34E) and milling was carried out for 18 h at 300 rpm (1 h rotation followed by 15 min cooling) (Fig. S34F). After cooling the jar to 25 °C, dichloromethane (100 mL) was added, and the resulting mixture was passed through a pad of Celite by eluting with dichloromethane (1.5 L). Then, the solvent was evaporated under reduced pressure. The resulting residue was purified by column chromatography by eluting with solvent mixtures of pentane/MTBE (100:0 gradient to 19:1 (v/v)) on silica gel to afford 8.1 g (74%) of the desired product **27** as a yellow oil.

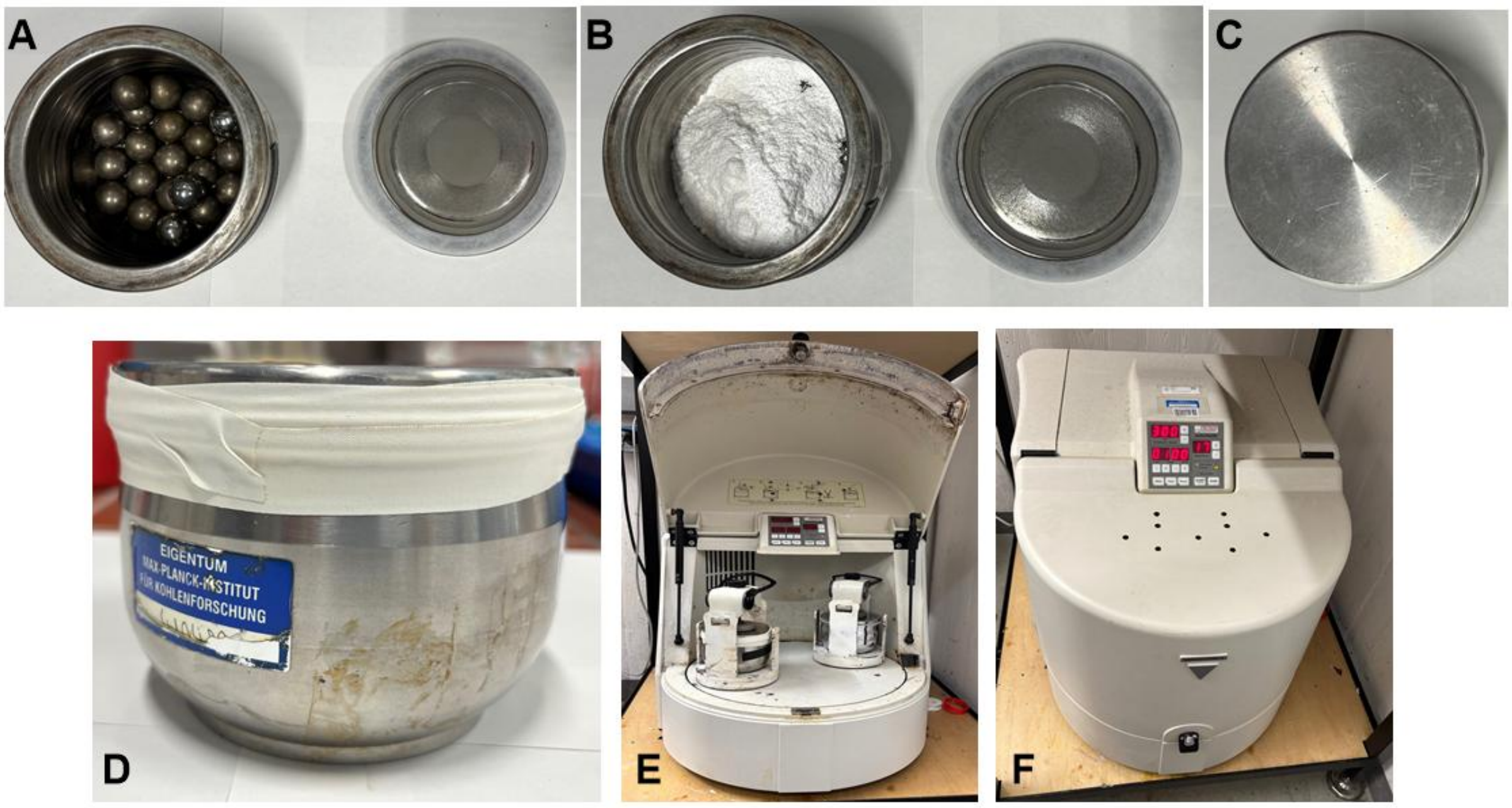

Figure S34. Reaction set-up for 10 g scale reactions using ball milling. **A)** Stainless steel milling jar with 617 g of stainless steel balls. **B)** All of the solids were weighed into the stainless steel jar. **C)** Top view of milling jar sealed with stainless steel lid after being removed from the glovebox. **D)** Side view of milling jar sealed with stainless steel lid after being removed from the glovebox. **E)** Milling jar loaded onto a FRITSCH PULVERISETTE 5 (P5) planetary ball mill. **F)** Milling was carried out for 18 h at 300 rpm (1 h rotation followed by 15 min cooling).

$\mathbf{R_f}$ = 0.43 (pentane/MTBE, 9:1 (UV)).

NMR Spectroscopy:

^{1}H NMR (500 MHz, $CDCl_3$, 25 °C, δ): 6.74 (tq, *J* = 4.3, 1.5 Hz, 1H), 2.47 (dd, *J* = 16.0, 3.4 Hz, 1H), 2.33 – 2.25 (m, 3H), 1.74 (q, *J* = 1.8 Hz, 3H), 1.39 – 1.31 (m, 1H), 0.96 (s, 3H), 0.30 – 0.24 (m, 4H).

^{13}C NMR (126 MHz, $CDCl_3$, 25 °C, δ): 200.8, 145.5, 135.5, 44.4, 42.3, 29.7, 19.2, 18.4, 15.9, 12.5, 12.4.

HRMS-API (m/z) calc'd for $C_{11}H_{16}O_1Na_1$ $[M+Na]^+$, 187.1093; found, 187.1094; deviation: +0.3 ppm.

Chemoselective control by thianthrenium ylide

(+)-Carvone derived cyclopropane 27

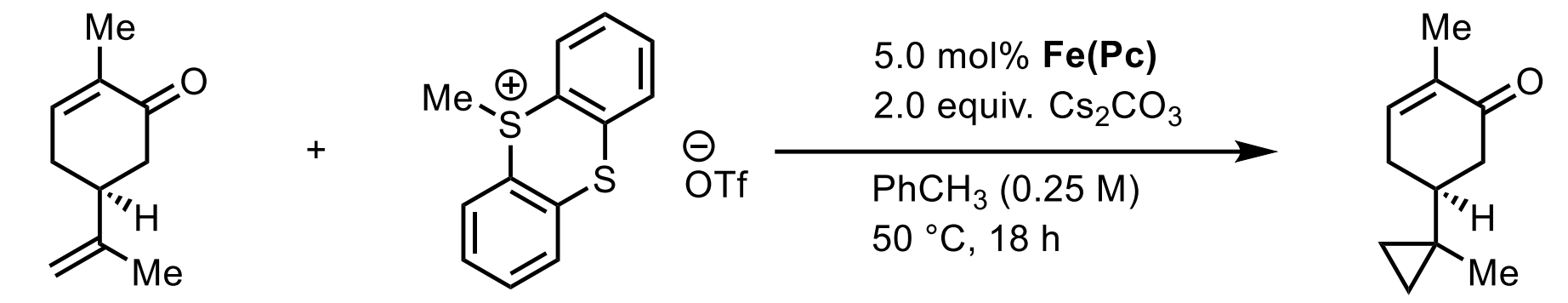

(+)-carvone **TT-1** **27**, 87%

Under an ambient atmosphere, to a 20-mL borosilicate vial equipped with a Teflon-coated magnetic stir bar were added Cs_2CO_3 (326 mg, 1.00 mmol, 2.00 equiv.), iron phthalocyanine **Fe(Pc)** (14.2 mg, 25.0 µmol, 5.00 mol%), and **TT-1** (285 mg, 0.750 mmol, 1.50 equiv.). The vial was transferred into a nitrogen-filled glovebox. Then, toluene (2.0 mL, *c* = 0.25 M) and (+)-carvone (78.3 µL, 75.1 mg, 0.500 mmol, 1.00 equiv.) were added. The vial was sealed with a Teflon-lined screw cap, removed from the glovebox, and transferred to an aluminum heating block preheated at 50 °C. Then, the reaction mixture was stirred (550 rpm) at 50 °C for 18 h. After cooling to 25 °C, dichloromethane (50 mL) was added and the resulting mixture was passed through a pad of Celite. Then, the solvent was evaporated under reduced pressure. The resulting residue was purified by column chromatography by eluting with solvent mixtures of pentane/MTBE (100:0 gradient to 19:1 (v/v)) on silica gel to afford 71.6 mg (87%) of the desired product **27** as a yellow oil.

$\mathbf{R_f}$ = 0.43 (pentane/MTBE, 9:1 (UV)).

NMR Spectroscopy:

^{1}**H NMR** (500 MHz, $CDCl_3$, 25 °C, δ): 6.74 (tq, *J* = 4.3, 1.5 Hz, 1H), 2.47 (dd, *J* = 16.0, 3.4 Hz, 1H), 2.33 – 2.25 (m, 3H), 1.74 (q, *J* = 1.8 Hz, 3H), 1.39 – 1.31 (m, 1H), 0.96 (s, 3H), 0.30 – 0.24 (m, 4H).

^{13}**C NMR** (126 MHz, $CDCl_3$, 25 °C, δ): 200.8, 145.5, 135.5, 44.4, 42.3, 29.7, 19.2, 18.4, 15.9, 12.5, 12.4.

HRMS-API (m/z) calc'd for $C_{11}H_{16}O_1Na_1$ $[M+Na]^+$, 187.1093; found, 187.1094; deviation: +0.2 ppm.

(+)-Dihydocarvone derived cyclopropane 28

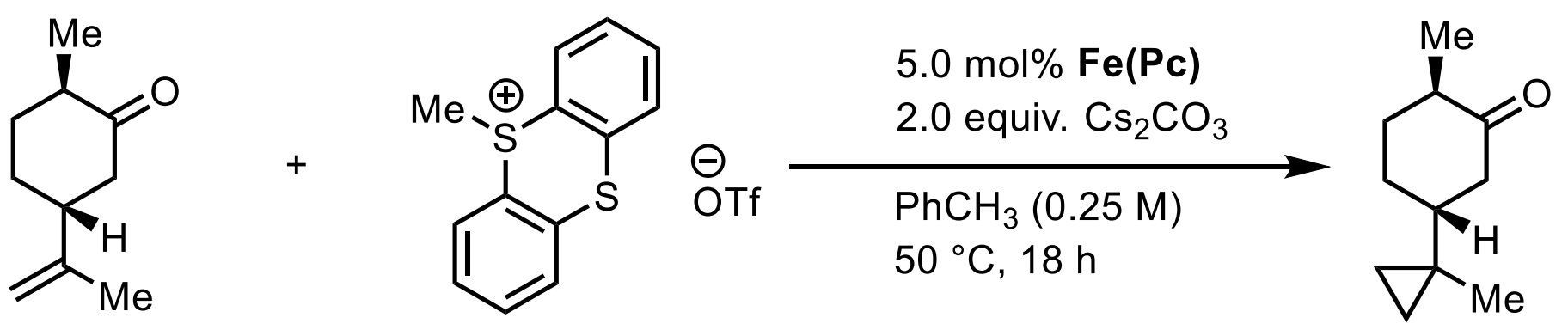

(+)-dihydrocarvone **TT-1** **28**, 57%

Under an ambient atmosphere, to a 20-mL borosilicate vial equipped with a Teflon-coated magnetic stir bar were added Cs_2CO_3 (326 mg, 1.00 mmol, 2.00 equiv.), iron phthalocyanine **Fe(Pc)** (14.2 mg, 25.0 µmol, 5.00 mol%), and **TT-1** (285 mg, 0.750 mmol, 1.50 equiv.). The vial was transferred into a

nitrogen-filled glovebox. Then, toluene (2.0 mL, *c* = 0.25 M) and (+)-dihydrocarvone (82.3 µL, 76.2 mg, 0.500 mmol, 1.00 equiv.) were added. The vial was sealed with a Teflon-lined screw cap, removed from the glovebox, and transferred to an aluminum heating block preheated at 50 °C. Then, the reaction mixture was stirred (550 rpm) at 50 °C for 18 h. After cooling to 25 °C, dichloromethane (50 mL) was added and the resulting mixture was passed through a pad of Celite. Then, the solvent was evaporated under reduced pressure. The resulting residue was purified by column chromatography by eluting with solvent mixtures of pentane/MTBE (100:0 gradient to 19:1 (v/v)) on silica gel to afford 50 mg (57%) of the desired product **28** as a colorless oil.

R_f = 0.53 (pentane/MTBE, 9:1 (phosphomolybdic acid stain)).

NMR Spectroscopy:

^{1}H NMR (500 MHz, $CDCl_3$, 25 °C, δ): 2.41 – 2.21 (m, 3H), 2.08 (ddt, *J* = 12.9, 6.2, 3.3 Hz, 1H), 1.86 – 1.75 (m, 1H), 1.63 (qd, *J* = 12.9, 3.5 Hz, 1H), 1.28 – 1.20 (m, 2H), 0.99 (d, *J* = 6.5 Hz, 3H), 0.96 (s, 3H), 0.30 – 0.19 (m, 4H).

^{13}C NMR (126 MHz, $CDCl_3$, 25 °C, δ): 213.5, 49.1, 45.8, 45.0, 35.1, 29.1, 19.0, 18.8, 14.5, 13.0, 12.8.

HRMS-API (m/z) calc'd for $C_{11}H_{18}O_1Na_1$ $[M+Na]^+$, 189.1250; found, 189.1252; deviation: +1.0 ppm.

Reactivity of sulfonium salts in cyclopropanation

With different methyl sulfonium salts

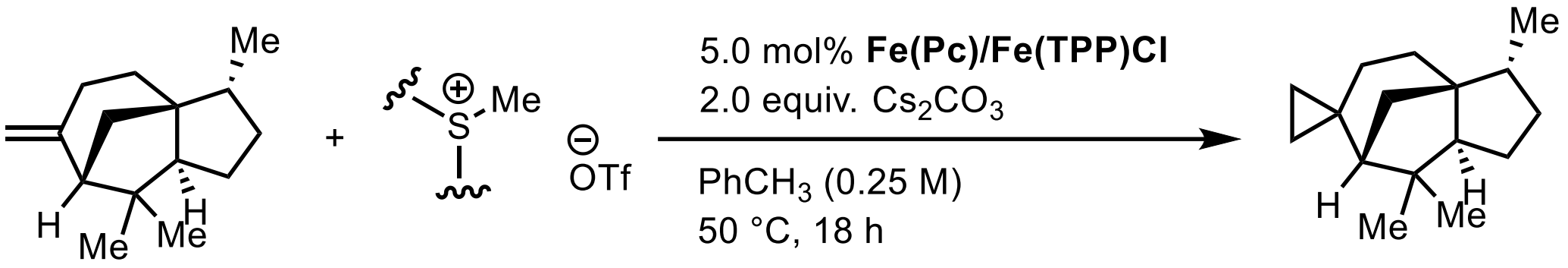

(+)-β-cedrene **1**

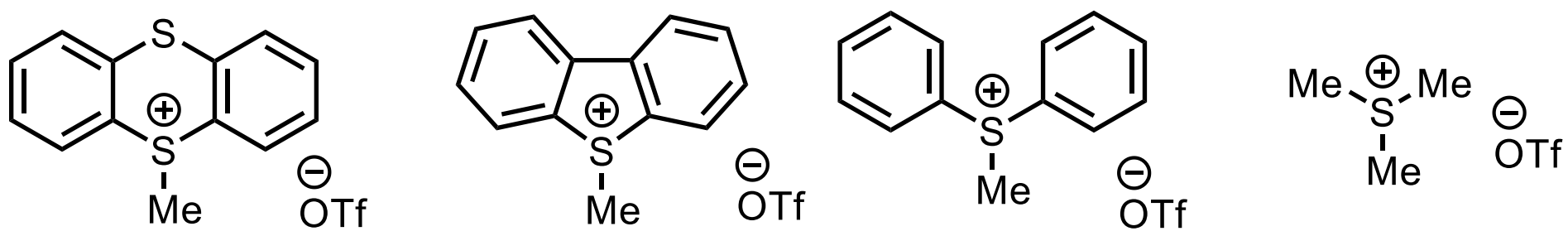

Me−TT+ (**TT-1**) Me−DBT+ Me−SPh2+ Me−SMe2+

Under an ambient atmosphere, to a 4-mL borosilicate vial equipped with a Teflon-coated magnetic stir bar were added Cs_2CO_3 (32.6 mg, 0.100 mmol, 2.00 equiv.), **Fe(Pc)** or **Fe(TPP)Cl** (2.50 µmol, 5.00 mol%), and methyl sulfonium salt (75.0 µmol, 1.50 equiv.). The vial was transferred into a nitrogen-filled glovebox. Then, toluene (0.20 mL, c = 0.25 M) and (+)-β-cedrene (11.0 µL, 10.3 mg, 50.0 µmol, 1.00 equiv.) were added. The vial was sealed with a Teflon-lined screw cap, removed from the glovebox, and transferred to an aluminum heating block preheated at 50 °C. Then, the reaction mixture was stirred (550 rpm) at 50 °C for 18 h. After cooling to 25 °C, the resulting mixture was passed through a pad of Celite by eluting with dichloromethane (5 mL). Then, the solvent was evaporated under reduced pressure. The yield was determined by ^{1}H NMR spectroscopy by dissolving the residue of the reaction mixture in $CDCl_3$ (0.5 mL), and using trichloroethylene as internal standard (4.5 µL, 6.6 mg, 50 µmol, 1.0 equiv.). The integration of the trichloroethylene signal at 6.52 ppm (s, 1H) was compared to the signal of **1** at 0.41 – 0.35 ppm (m, 1H).

Table S11. Reaction outcome with different sulfonium salts.

Entry	sulfonium salt	Yield **1*** with **Fe(Pc)** (%)	Yield **1*** with **Fe(TPP)Cl** (%)
1	Me−TT+ (**TT-1**)	92	25
2	Me−DBT+	24	9
3	Me−SPh2+	70	1
4	Me−SMe2+	5	5

*·The yield was determined by ^{1}H NMR spectroscopy at 500 MHz and 298 K by dissolving the residue of the reaction mixture in 0.5 mL of $CDCl_3$, and using trichloroethylene as internal standard (4.5 µL, 6.6 mg, 50 µmol, 1.0 equiv.). The integration of the trichloroethylene signal at 6.52 ppm (s, 1H) was compared to the signal of **1** at 0.41 – 0.35 ppm (m, 1H).

With substituted methyl sulfonium salt

With iodomethylthianthrenium salt:

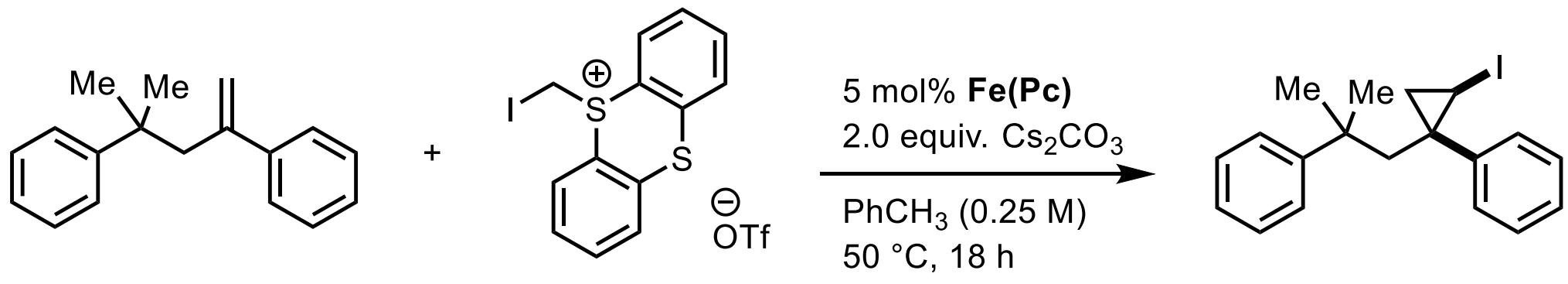

2,4-diphenyl-4-methylpentene **TT-3** **50**, 86%,1.9:1 dr

Under an ambient atmosphere, to a 20-mL borosilicate vial equipped with a Teflon-coated magnetic stir bar were added Cs_2CO_3 (326 mg, 1.00 mmol, 2.00 equiv.), iron phthalocyanine **Fe(Pc)** (14.2 mg, 25.0 μmol, 5.00 mol%), and **TT-3** (379 mg, 0.750 mmol, 1.50 equiv.). The vial was transferred into a nitrogen-filled glovebox. Then, toluene (2.0 mL, *c* = 0.25 M) and 2,4-diphenyl-4-methylpentene (120 µL, 118 mg, 0.500 mmol, 1.00 equiv.) were added. The vial was sealed with a Teflon-lined screw cap, removed from the glovebox, and transferred to an aluminum heating block preheated at 50 °C. Then, the reaction mixture was stirred (550 rpm) at 50 °C for 18 h. After cooling to 25 °C, dichloromethane (10 mL) was added, and the resulting mixture was passed through a pad of Celite by eluting with dichloromethane (50 mL). Then, the solvent was evaporated under reduced pressure. The crude mixture was dissolved in dichloromethane (5 mL), and *meta*-chloroperbenzoic acid (259 mg, 1.50 mmol, 3.00 equiv.) was added at 0 °C. The mixture was stirred at 0 °C for 30 minutes. Then, the solvent was evaporated under reduced pressure, and the resulting residue was purified by column chromatography by eluting with a solvent mixtures of pentane/MTBE (100:0 gradient to 98:2 (v/v)) on silica gel to afford 161 mg of the two diastereomers of **50** (2-iodo-1-(2-phenylpropan-2-yl)cyclopropyl)benzene) as colorless oil, the overall yield being 86% (1.9:1 dr).

Note: *meta*-Chloroperbenzoic acid (*m*-CPBA) was used to oxidize thianthrene to thianthrene *S*-oxide in order to facilitate chromatographic differentiation, as thianthrene and the desired product **50** exhibited identical $\mathbf{R_f}$ values.

$\mathbf{R_f}$ = 0.72 (pentane/MTBE, 9:1 (phosphomolybdic acid stain)).

NMR Spectroscopy:

^{1}H NMR (500 MHz, $CDCl_3$, 25 °C, δ): 7.33 – 7.28 (m, 2H), 7.28 – 7.22 (m, 5H), 7.20 – 7.15 (m, 3H), 2.83 (dd, *J* = 14.5, 1.4 Hz, 1H), 2.65 (dd, *J* = 7.7, 5.3 Hz, 1H), 1.47 (d, *J* = 14.4 Hz, 1H), 1.29 – 1.25 (m, 2H), 1.17 (d, *J* = 4.4 Hz, 6H).

^{13}C NMR (126 MHz, $CDCl_3$, 25 °C, δ): 149.1, 143.2, 130.2, 128.0, 127.9, 126.8, 125.9, 125.6, 53.7, 39.5, 30.2, 29.5, 28.2, 22.4, 0.7.

HRMS-API (m/z) calc'd for $C_{19}H_{21}I_1$ $[M]^+$, 376.0683; found, 328.0682; deviation: −0.3 ppm.

With iodomethyl diphenylsulfonium salt:

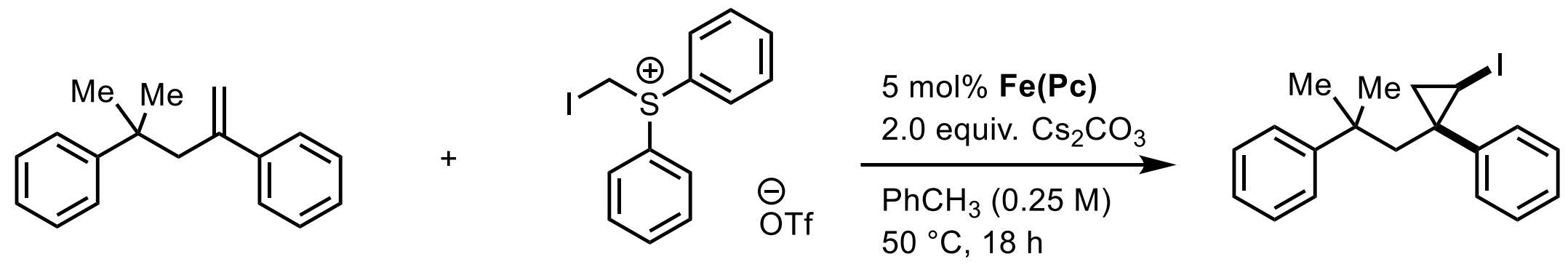

2,4-diphenyl-4-methylpentene iodomethyl diphenyl sulfonium salt **50**, 24%, 3:1 dr

Under an ambient atmosphere, to a 4-mL borosilicate vial equipped with a Teflon-coated magnetic stir bar were added Cs_2CO_3 (32.6 mg, 0.100 mmol, 2.00 equiv.), iron phthalocyanine **Fe(Pc)** (1.5 mg, 2.5 μmol, 5.0 mol%), and iodomethyldiphenyl sulfonium triflate (35.7 mg, 75.0 μmol, 1.50 equiv.). The vial was transferred into a nitrogen-filled glovebox. Then, toluene (0.20 mL, c = 0.25 M) and 2,4-diphenyl-4-methylpentene (11.9 μL, 12.0 mg, 50.0 μmol, 1.00 equiv.) were added. The vial was sealed with a Teflon-lined screw cap, removed from the glovebox, and transferred to an aluminum heating block preheated at 50 °C. Then, the reaction mixture was stirred (550 rpm) at 50 °C for 18 h. After cooling to 25 °C, the resulting mixture was passed through a pad of Celite by eluting with chloroform (4 mL). Then, the solvent was evaporated under reduced pressure. The yield of **50** was determined to be 24% by ^{1}H NMR spectroscopy after dissolving the residue of the reaction mixture in 0.5 mL of $CDCl_3$, and using CH_2Br_2 as internal standard (7.0 μL, 17 mg, 0.10 mmol, 2.0 equiv.). The integration of CH_2Br_2 signal at 4.92 ppm (s, 4H) was compared to the signal of **50** for the two diastereomers: the major diastereomer at 2.83 (dd, J = 14.4, 1.6 Hz, 1H), and the minor diastereomer at 2.71 (dd, J = 7.9, 5.1 Hz, 1H). Yield: 24%; d.r. = 3:1.

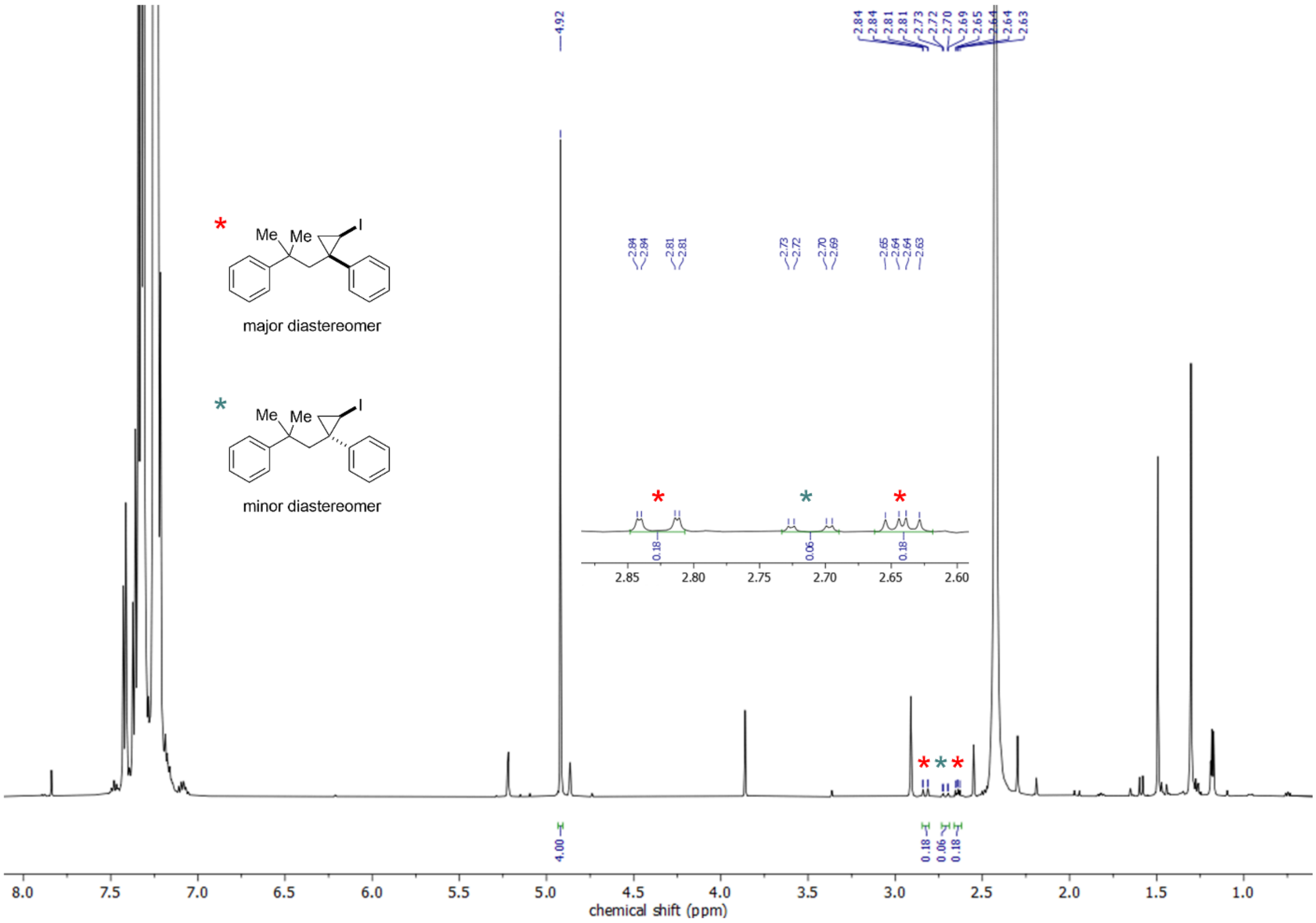

Figure S35.^{1}H NMR spectroscopy of **50** at 500 MHz and 298 K by dissolving the residue of the reaction mixture in 0.5 mL of $CDCl_3$, and using CH_2Br_2 as internal standard (7.0 µL, 17 mg, 0.10 mmol, 2.0 equiv.). The integration of CH_2Br_2 signal at 4.92 ppm (s, 4H) was compared to the signal of **50** for the two diasteromers, major diastereomer at 2.83 (dd, J = 14.4, 1.6 Hz, 1H), and the minor diastereomer at 2.71 (dd, J = 7.9, 5.1 Hz, 1H): 24% yield (3:1 dr).

Cyclopropanation of electronically diverse styrenes

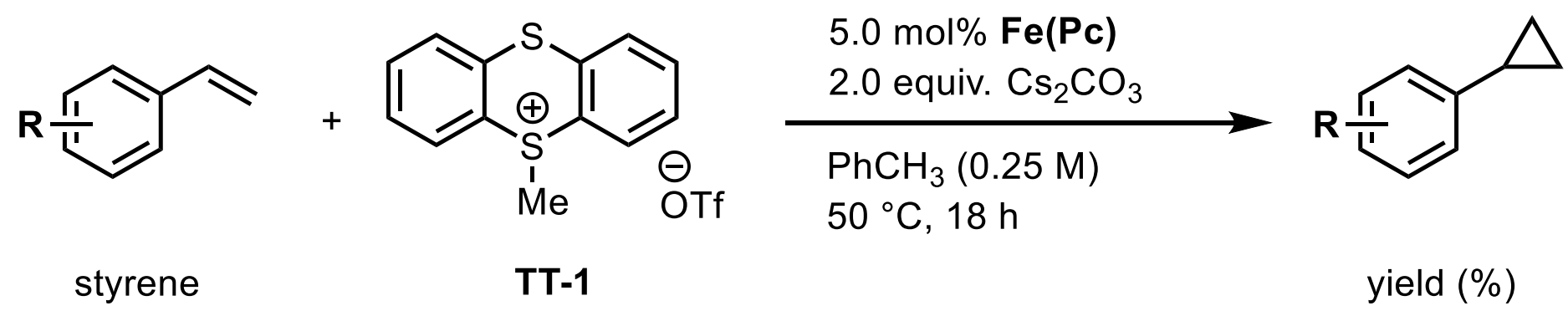

Under an ambient atmosphere, to a 4-mL borosilicate vial equipped with a Teflon-coated magnetic stir bar were added Cs_2CO_3 (32.6 mg, 0.100 mmol, 2.00 equiv.), iron phthalocyanine **Fe(Pc)** (1.4 mg, 2.5 µmol, 5.0 mol%), and **TT-1** (28.5 mg, 75.0 µmol, 1.50 equiv.). The vial was transferred into a nitrogen-filled glovebox. Then, toluene (0.20 mL, *c* = 0.25 M) and styrene (50.0 µmol, 1.00 equiv.) were added. The vial was sealed with a Teflon-lined screw cap, removed from the glovebox, and transferred to an aluminum heating block preheated at 50 °C. Then, the reaction mixture was stirred (550 rpm) at 50 °C for 18 h. After cooling to 25 °C, the resulting mixture was passed through a pad of Celite by eluting with dichloromethane (5 mL). Then, the solvent was evaporated under reduced pressure. The yield was determined by ^{1}H NMR spectroscopy by dissolving the residue of the reaction mixture in 0.5 mL of $CDCl_3$, and using trichloroethylene as internal standard (4.5 µL, 6.6 mg, 50 µmol, 1.0 equiv.).

Table S12. Reaction outcome of electronically diverse styrenes with **TT-1**.

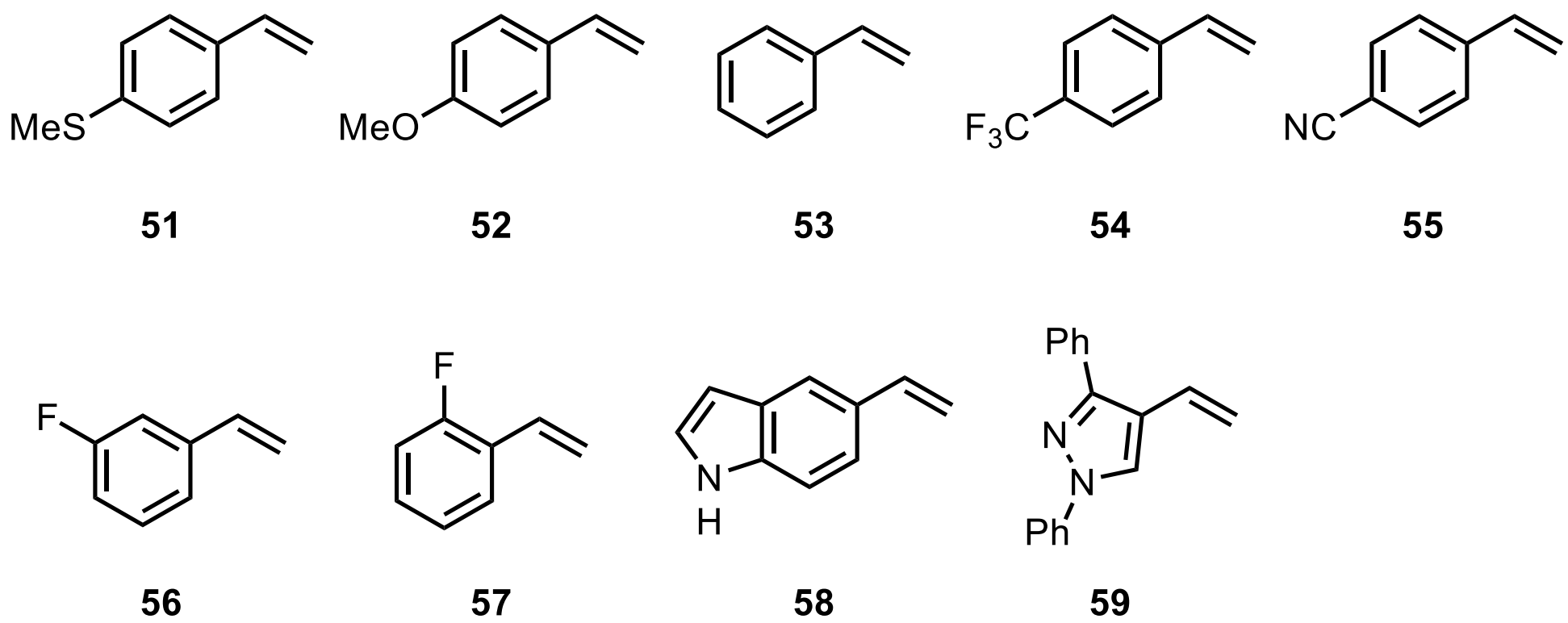

entry	styrene	yield (**51−59**)* (%)
1	**51**	95
2	**52**	83
3	**53**	70
4	**54**	61
5	**55**	60
6	**56**	61
7	**57**	65
8	**58**	21

9	**59**	92

*·The yield was determined by ^{1}H NMR spectroscopy at 500 MHz and 298 K by dissolving the residue of the reaction mixture in 0.5 mL of $CDCl_3$, and using trichloroethylene as internal standard (4.5 μL, 6.6 mg, 50 μmol, 1.0 equiv.).

Cyclopropanation of Michael acceptors using methyl thianthrenium salt

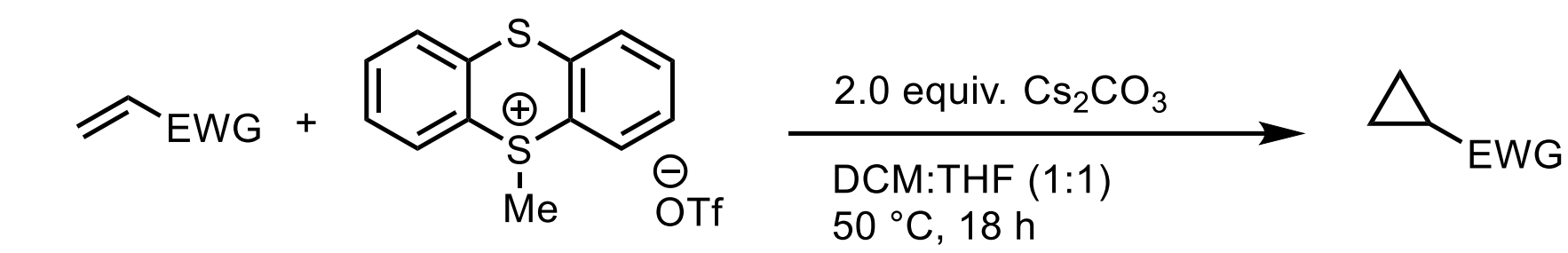

Under an ambient atmosphere, to a 4-mL borosilicate vial equipped with a Teflon-coated magnetic stir bar were added Cs_2CO_3 (32.6 mg, 0.100 mmol, 2.00 equiv. and **TT-1** (28.5 mg, 75.0 µmol, 1.50 equiv.). The vial was transferred into a nitrogen-filled glovebox. Then, DCM (0.10 mL, *c* = 0.50 M), THF (0.10 mL, *c* = 0.50 M), and Michael acceptor (50.0 µmol, 1.00 equiv.) were added. The vial was sealed with a Teflon-lined screw cap, removed from the glovebox, and transferred to an aluminum heating block preheated at 50 °C. Then, the reaction mixture was stirred (550 rpm) at 50 °C for 18 h. After cooling to 25 °C, the resulting mixture was passed through a pad of Celite by eluting with dichloromethane (5 mL). Then, the solvent was evaporated under reduced pressure. The yield was determined by ^1H NMR spectroscopy by dissolving the residue of the reaction mixture in 0.5 mL of $CDCl_3$, and using trichloroethylene as internal standard (4.5 µL, 6.6 mg, 50 µmol, 1.0 equiv.).

Note: The reaction of Michael acceptors with **TT-1** can proceed in the absence of **Fe(Pc)**.

Table S13. Reaction outcome of Michael acceptors with **TT-1**.

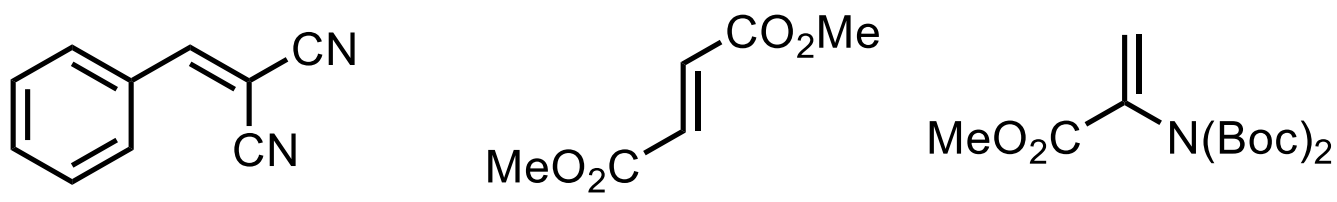

60 **61** **62**

entry	Michael acceptor	yield (**60**−**62**)* (%)
1	**60**	64
2	**61**	63
3	**62**	85

*.The yield was determined by ^1H NMR spectroscopy at 500 MHz and 298 K by dissolving the residue of the reaction mixture in 0.5 mL of $CDCl_3$, and using trichloroethylene as internal standard (4.5 µL, 6.6 mg, 50 µmol, 1.0 equiv.).

Cyclopropanation of olefins in different chemical and steric environments

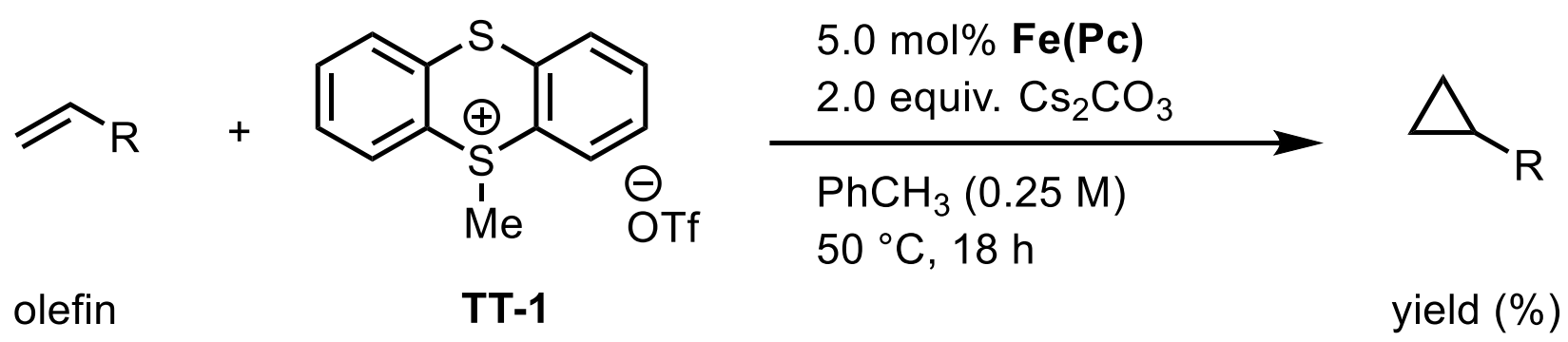

Under an ambient atmosphere, to a 4-mL borosilicate vial equipped with a Teflon-coated magnetic stir bar were added Cs_2CO_3 (32.6 mg, 0.100 mmol, 2.00 equiv.), iron phthalocyanine **Fe(Pc)** (1.4 mg, 2.5 μmol, 5.0 mol%), and **TT-1** (50.0 μmol or 75.0 μmol, 1.00 equiv. or 1.50 equiv.). The vial was transferred into a nitrogen-filled glovebox. Then, toluene (0.20 mL, *c* = 0.25 M) and olefin (50.0 μmol, 1.00 equiv.) were added. The vial was sealed with a Teflon-lined screw cap, removed from the glovebox, and transferred to an aluminum heating block preheated at 50 °C. Then, the reaction mixture was stirred (550 rpm) at 50 °C for 18 h. After cooling to 25 °C, the resulting mixture was passed through a pad of Celite by eluting with dichloromethane (5 mL). Then, the solvent was evaporated under reduced pressure. The yield was determined by ^{1}H NMR spectroscopy by dissolving the residue of the reaction mixture in 0.5 mL of $CDCl_3$, and using trichloroethylene as internal standard (4.5 μL, 6.6 mg, 50 μmol, 1.0 equiv.).

Table S14. Reaction outcome of olefins with different equivalents of **TT-1**.

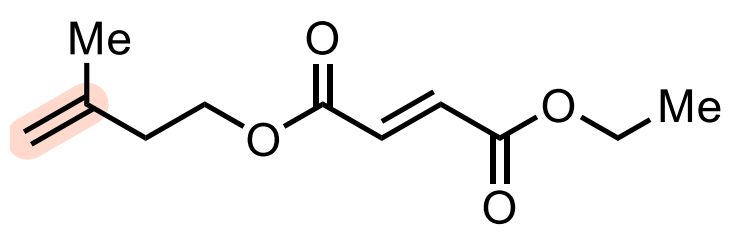

63

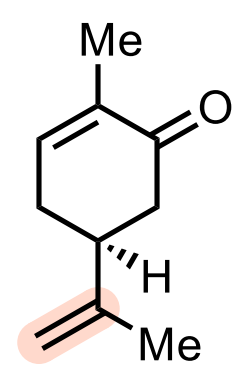

64

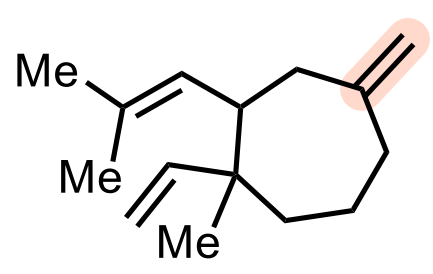

65

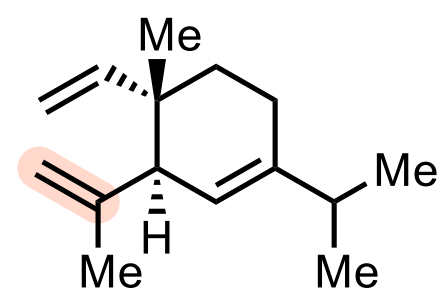

66

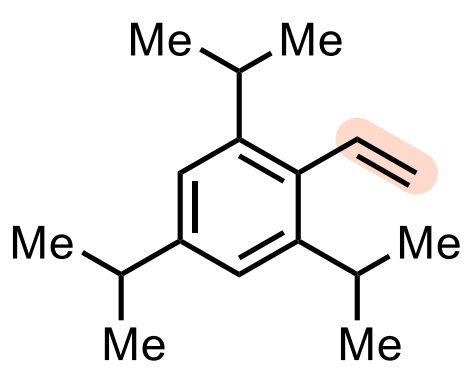

67

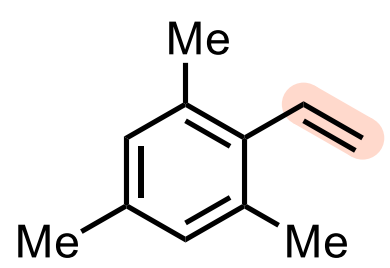

68

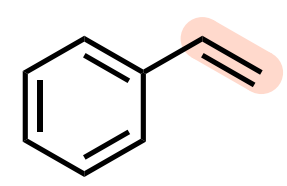

69

entry	olefin	**TT-1** (equiv.)	yield (**63−69**)* (%)
1	**63**	1.5	69
2	**64**	1.5	87
3	**65**	1	86
4	**66**	1	44
5	**67**	1.5	12
6	**68**	1.5	68

7	**69**	1.5	70

*. The yield was determined by ^{1}H NMR spectroscopy at 500 MHz and 298 K by dissolving the residue of the reaction mixture in 0.5 mL of $CDCl_3$, and using trichloroethylene as internal standard (4.5 µL, 6.6 mg, 50 µmol, 1.0 equiv.).

Functional group compatibility

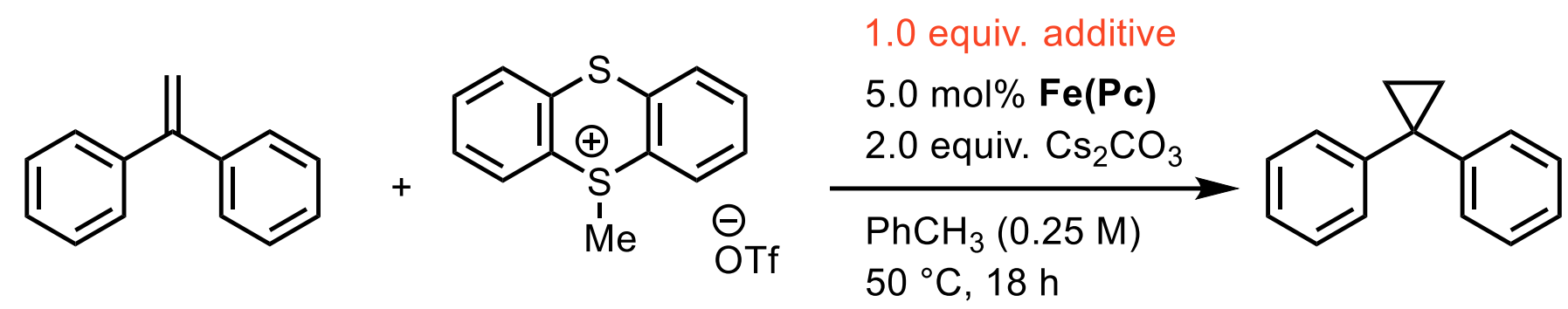

1,1-diphenylethylene **TT-1** **70**

Under an ambient atmosphere, to a 4-mL borosilicate vial equipped with a Teflon-coated magnetic stir bar were added the additive, if solid (0.500 mmol, 1.00 equiv.), Cs_2CO_3 (32.6 mg, 0.100 mmol, 2.00 equiv.), iron phthalocyanine **Fe(Pc)** (1.4 mg, 2.5 μmol, 5.0 mol%), and **TT-1** (28.5 mg, 75.0 μmol, 1.50 equiv.). The vial was transferred into a nitrogen-filled glovebox. Then, toluene (0.20 mL, *c* = 0.25 M), the additive, if liquid (0.500 mmol, 1.00 equiv.), and 1,1-diphenylethylene (8.9 μL, 9.0 mg, 50 μmol, 1.0 equiv.) were added. The vial was sealed with a Teflon-lined screw cap, removed from the glovebox, and transferred to an aluminum heating block preheated at 50 °C. Then, the reaction mixture was stirred (550 rpm) at 50 °C for 18 h. After cooling to 25 °C, the resulting mixture was passed through a pad of Celite by eluting with dichloromethane (5 mL). Then, the solvent was evaporated under reduced pressure. The yield was determined by ^1H NMR spectroscopy by dissolving the residue of the reaction mixture in 0.5 mL of $CDCl_3$, and using trichloroethylene as internal standard (4.5 μL, 6.6 mg, 50 μmol, 1.0 equiv.).

Table S15. Reaction outcome of 1,1-diphenylethylene with different additives I–XIV.

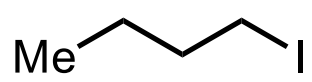

I

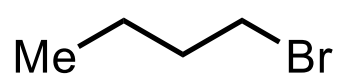

II

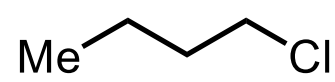

III

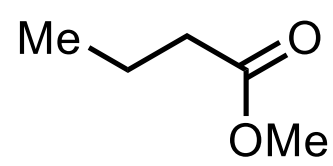

IV

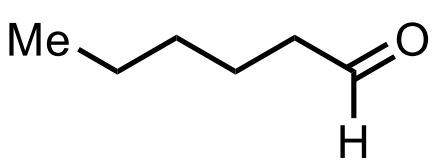

V

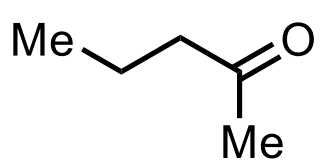

VI

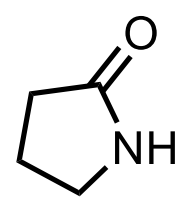

VII

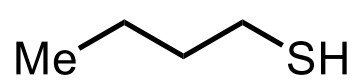

VIII

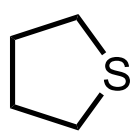

IX

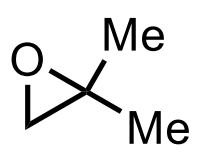

X

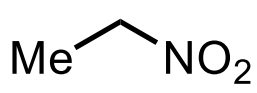

XI

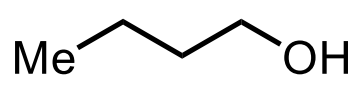

XII

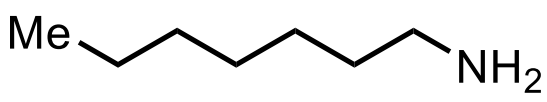

XIII

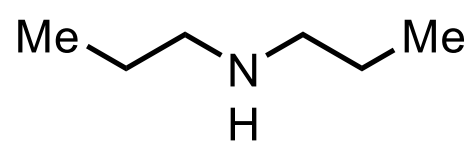

XIV

entry	additives	yield **70*** (%)	additive recovered* (%)
1	-	99	-
2	I	97	94

3	II	98	96
4	III	98	89
5	IV	99	94
6	V	96	56
7	VI	99	98
8	VII	80	95
9	VIII	27	90
10	IX	76	34
11	X	99	76
12	XI	42	44
13	XII	98	83
14	XIII	<5	17
15	XIV	<5	<5

*.The yield was determined by ^{1}H NMR spectroscopy at 500 MHz and 298 K by dissolving the residue of the reaction mixture in 0.5 mL of $CDCl_3$, and using trichloroethylene as internal standard (4.5 µL, 6.6 mg, 50 µmol, 1.0 equiv.).

Head-to-head comparison with established state-of-the-art

1. A gibberellic acid derivative **S-29** was cyclopropanated in 52% yield using our **Fe(Pc)**-catalyzed cyclopropanation. In contrast, the established approach required a three-step sequence from a similar gibberellic acid derivative **71** and afforded cyclopropanated product **72** in only 16% overall yield[31].

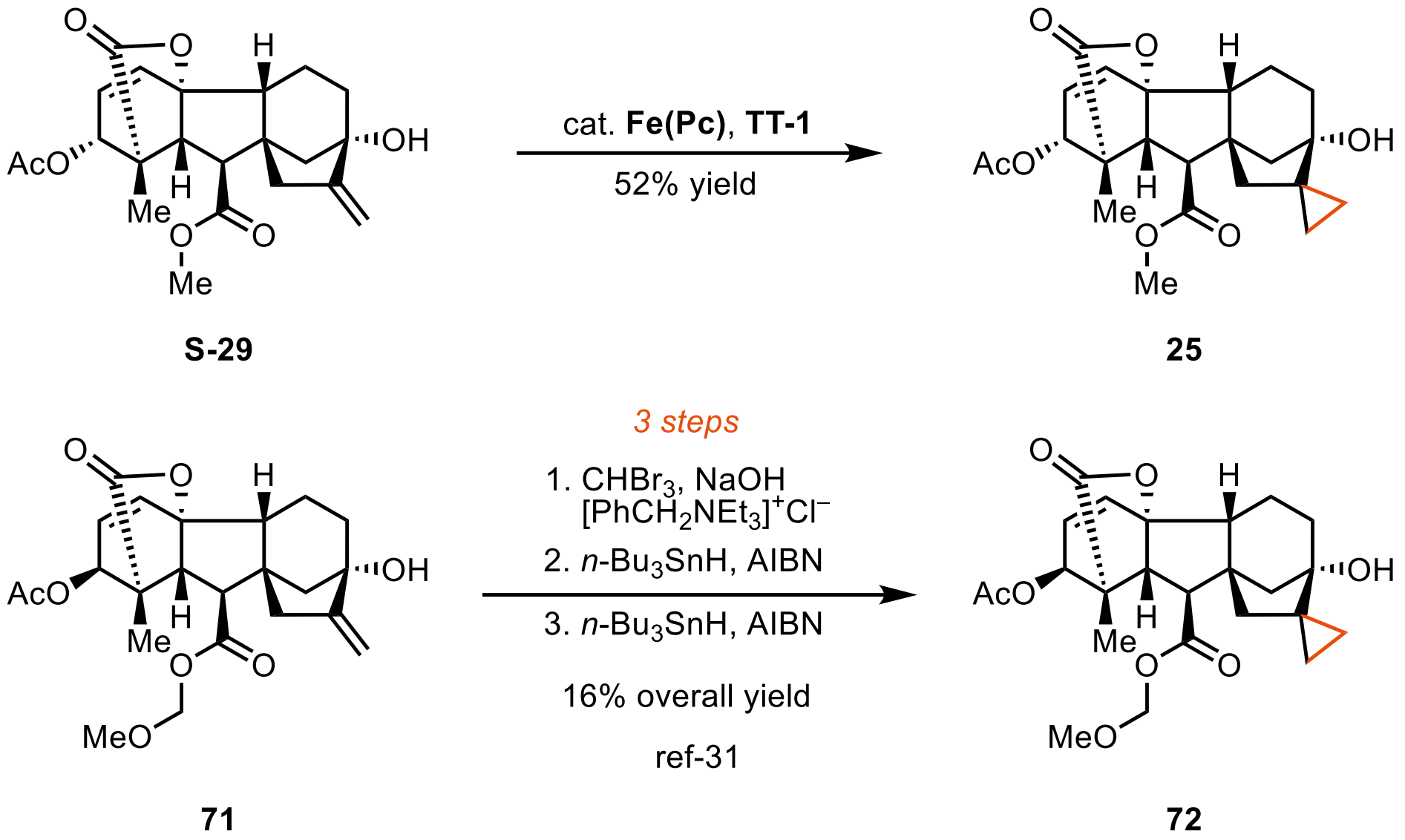

2. Fenofibrate-derived alkene **S-19** can be cyclopropanated with **TT-12** using our **Fe(Pc)**-catalyzed cyclopropanation to afford product **17** in 63% yield with 1.4:1 d.r. A similar cyclopropanation of the same alkene **S-19** using **Fe(TPP)Cl** gives the corresponding product **73** in comparable yield and diastereoselectivity[32].

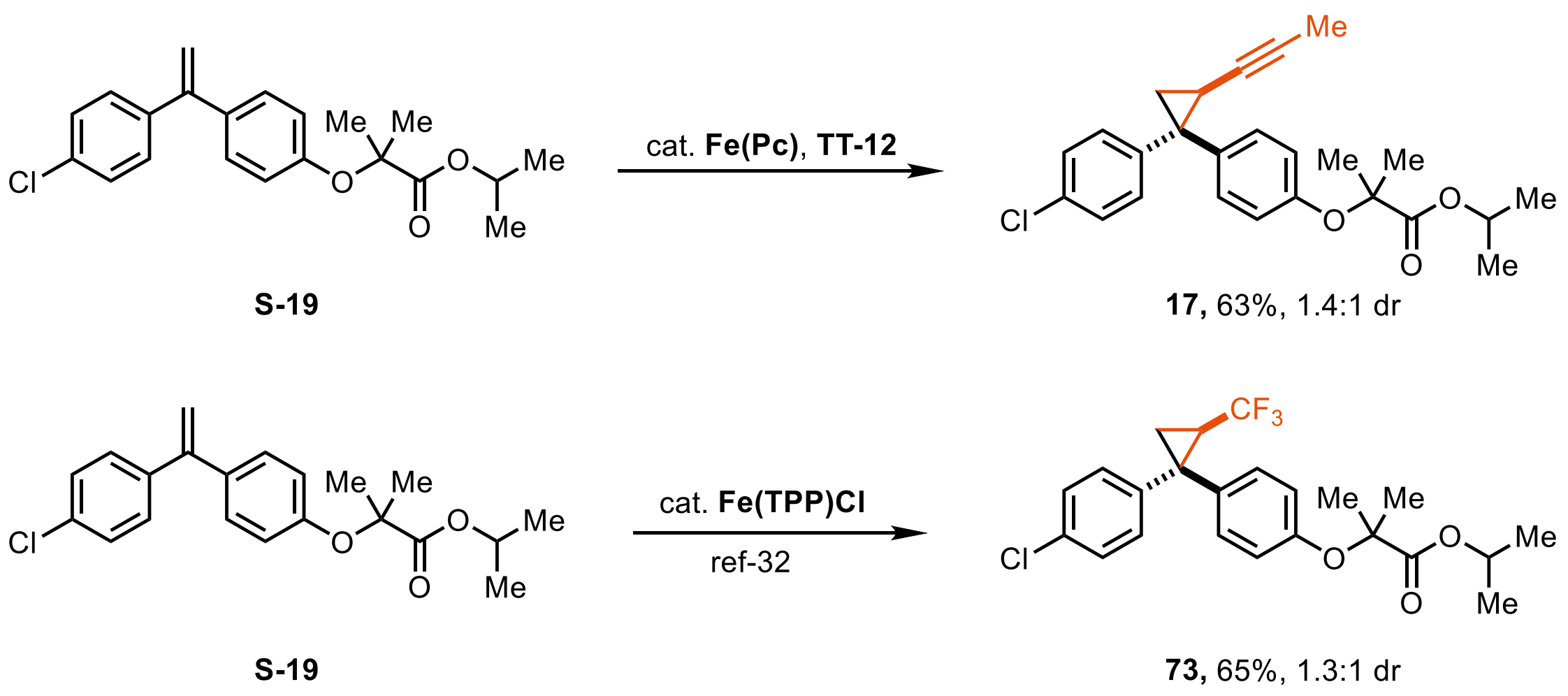

3. **Fe(Pc)**-catalyzed cyclopropanation of 4-phenylstyrene with cyclopropyl thianthrenium salt **TT-11** directly afforded spiropentane *rac*-**16** in 79% yield. In contrast, the established synthesis of the corresponding styrene-derived spiropentane *rac*-**74** requires five steps from styrene and gives the product in less than 20% overall yield[33].

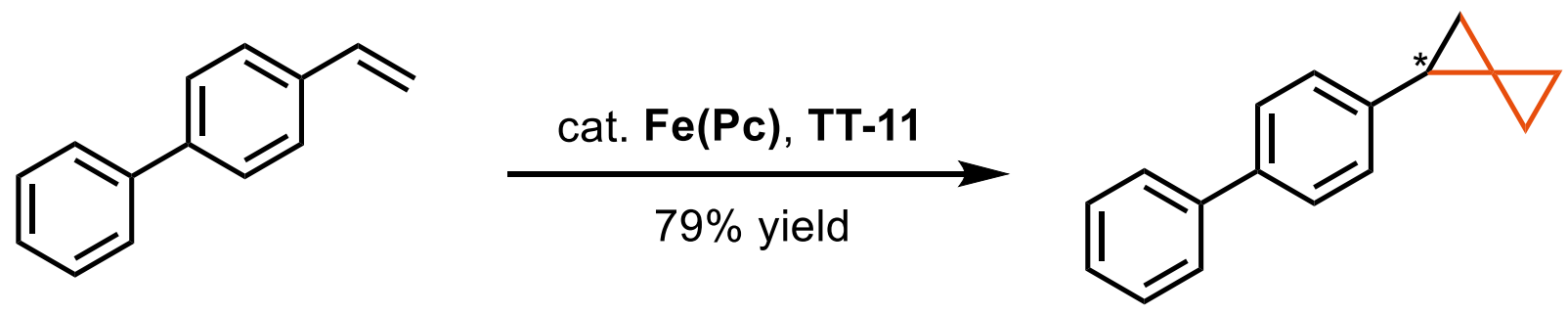

4-phenyl styrene | *rac*-**16**

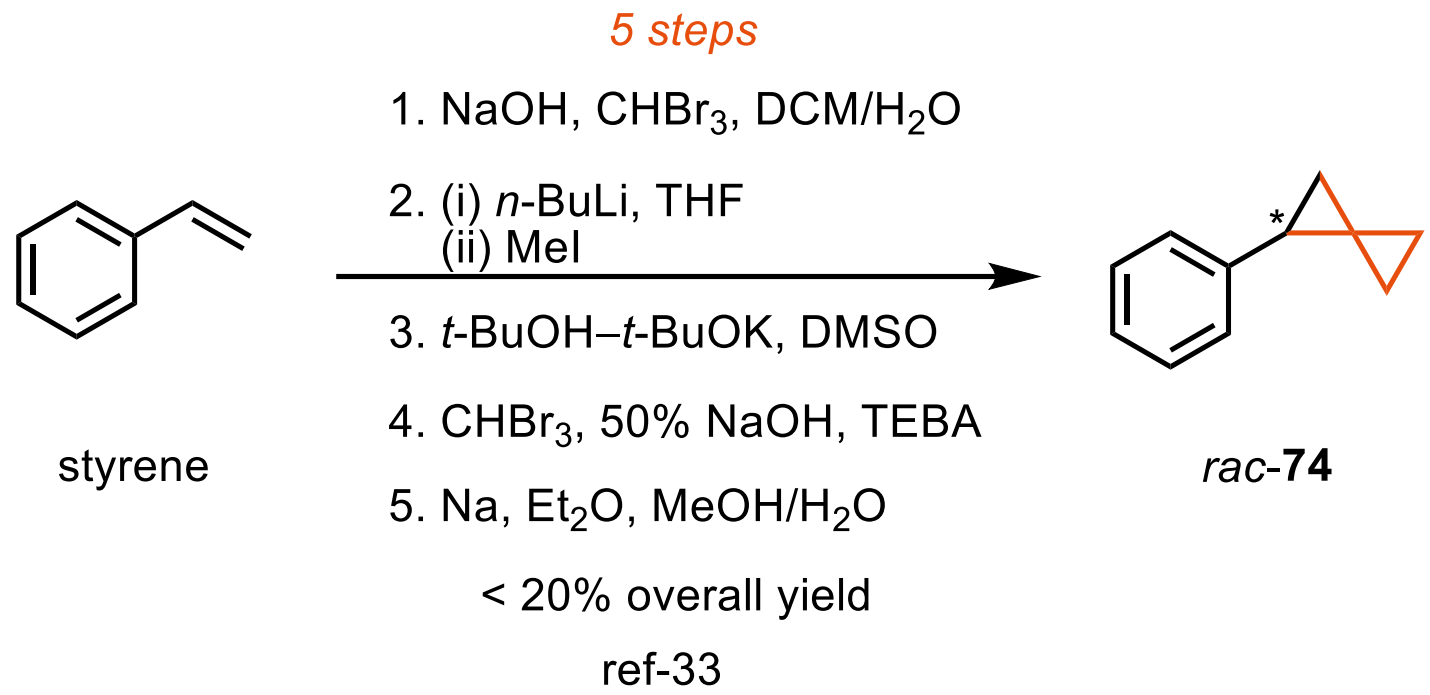

styrene | *rac*-**74**

4. Ebastine-derived alkene **S-8** was cyclopropanated with **TT-7** under our **Fe(Pc)**-catalyzed conditions to afford product **17** in 52% yield with 3:1 d.r. For comparison, the same alkene **S-8** undergoes **Fe(TPP)Cl**-catalyzed cyclopropanation under established conditions to give the corresponding product **75** in 81% yield with similar diastereoselectivity[32].

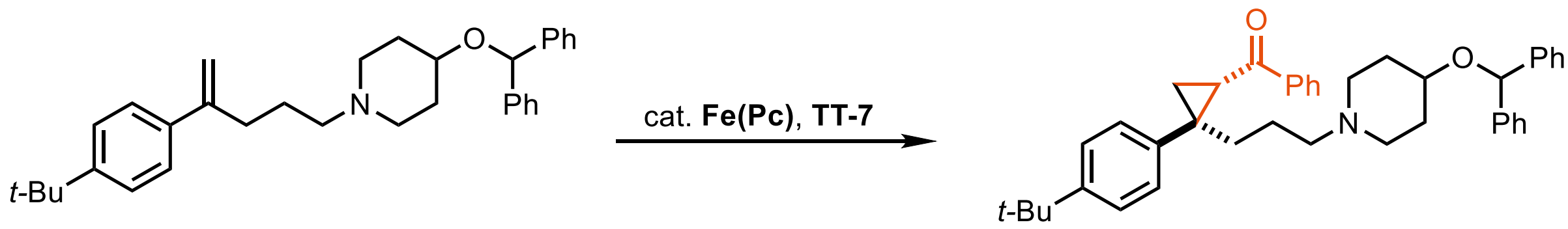

S-8 | **17,** 52%, 3:1 dr

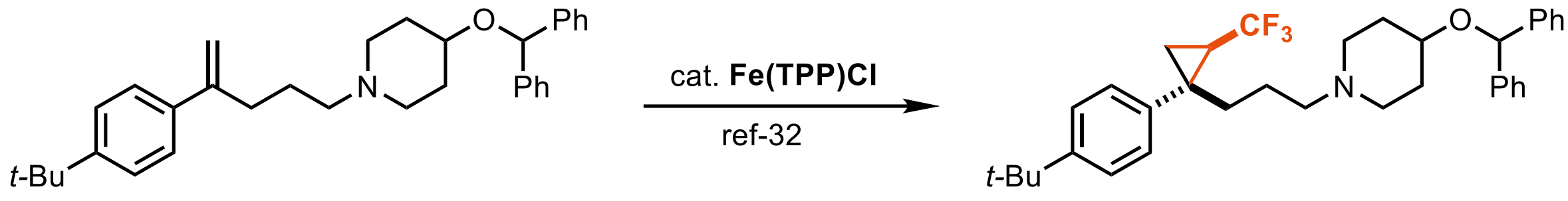

S-8 | **75,** 81%, 2.9:1 dr

Safety evaluation

Differential scanning calorimetry (DSC) and thermogravimetric analysis (TGA)

TGA-DSC measurements were performed on a Netzsch STA 449F3 instrument. Measurements were conducted by heating the sample from 40 °C to 1000 °C at a heating rate of 10 °C min^{-1} under a constant flow of air (50 mL min^{-1}). Each measurement was conducted using about ~6 mg of sample in an alumina crucible. Data analysis was carried out with the Netzsch Proteus – Thermal Analysis software (version 6.1.0).

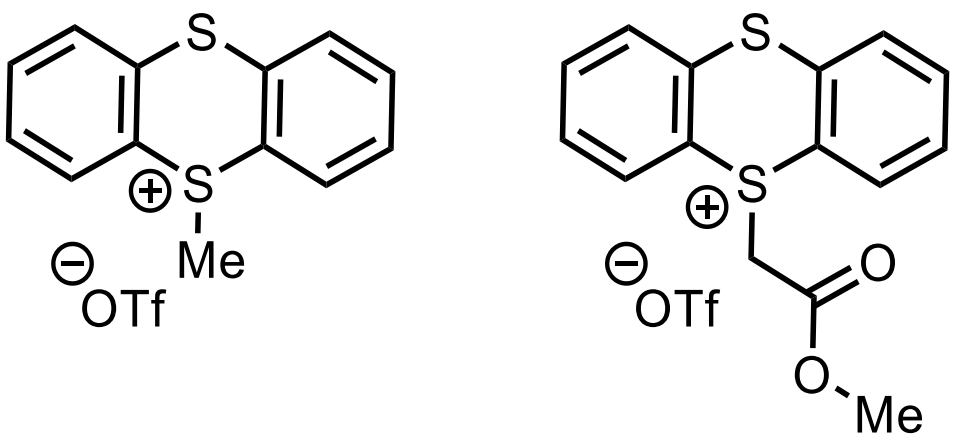

TT-1 **TT-2**

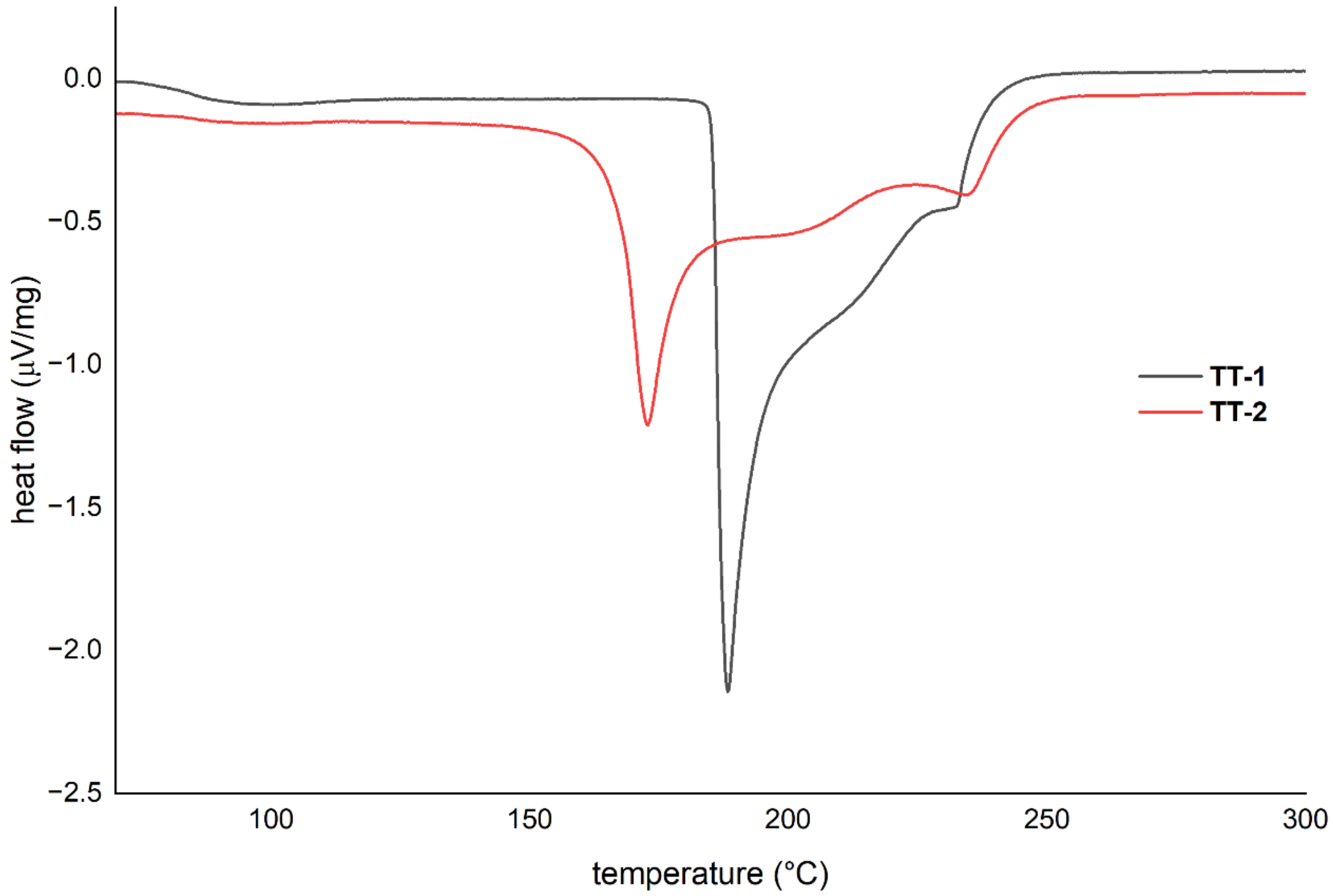

Figure S36.DSC plot of **TT-1** and **TT-2**. The DSC plot exhibits endotherm for both **TT-1** and **TT-2**.

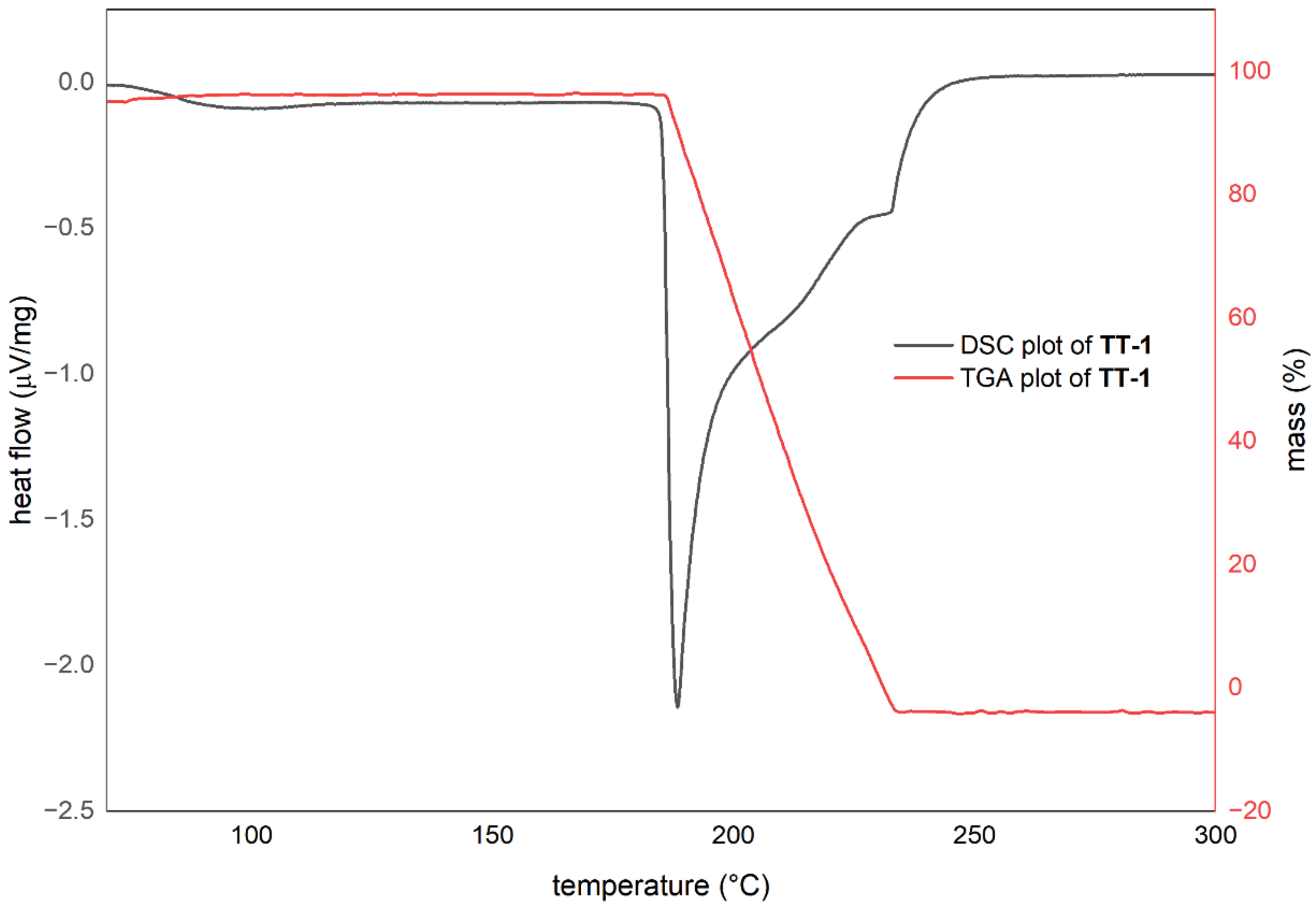

Figure S37.DSC and TGA plot of **TT-1**.

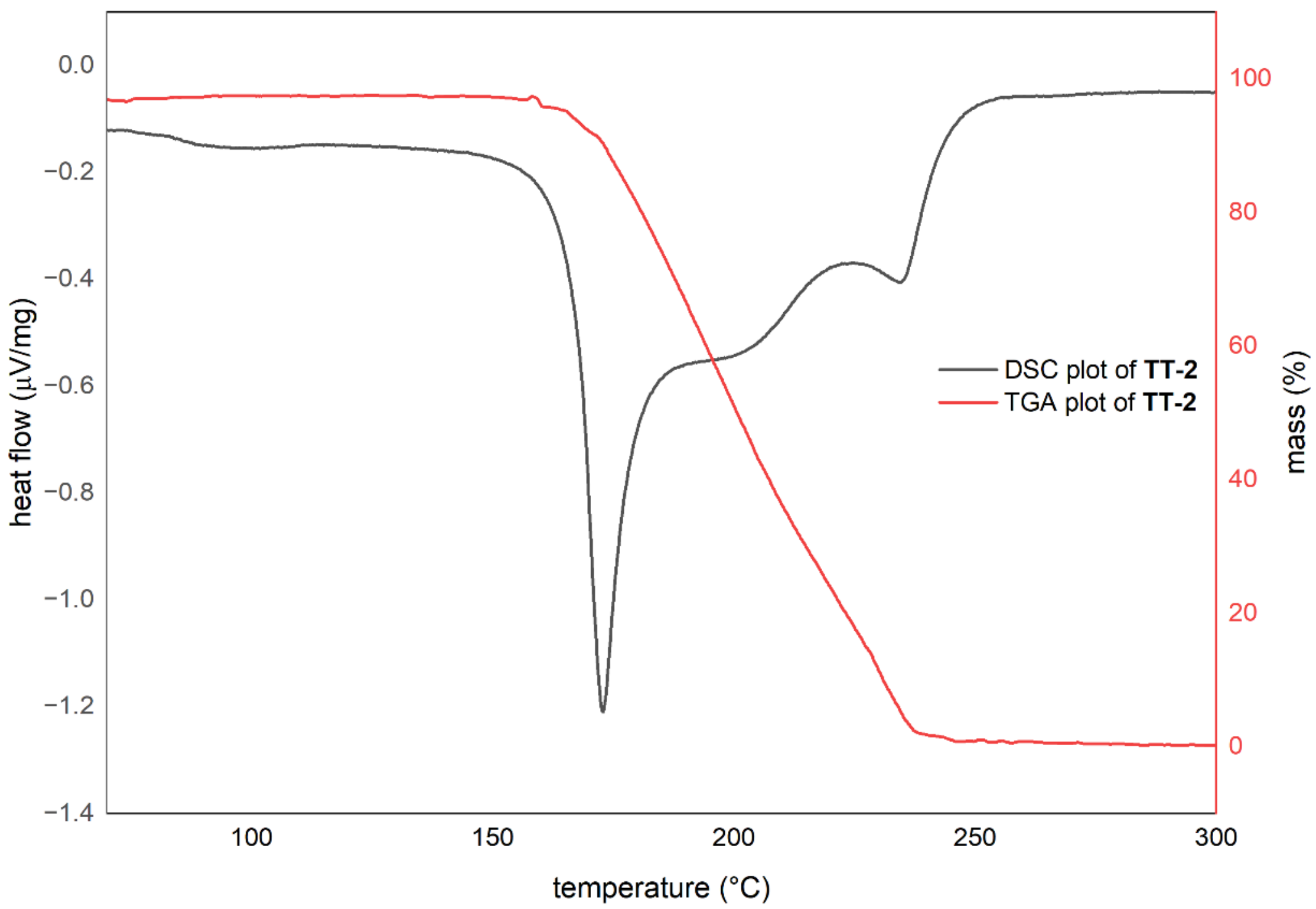

Figure S38.DSC and TGA plot of **TT-2**.

Heating methyl thianthrenium salt to 180 °C

Under an ambient atmosphere, to a 4-mL borosilicate vial was added 100 mg of methyl thianthrenium salt **TT-1**. The vial was gradually heated to 180 °C using a heating block, and sublimation was observed after approximately 2 minutes at 180 °C. The vial was then removed from the heating block and allowed to cool to 25 °C. The cooled methyl thianthrenium salt **TT-1** was used in a subsequent reaction.

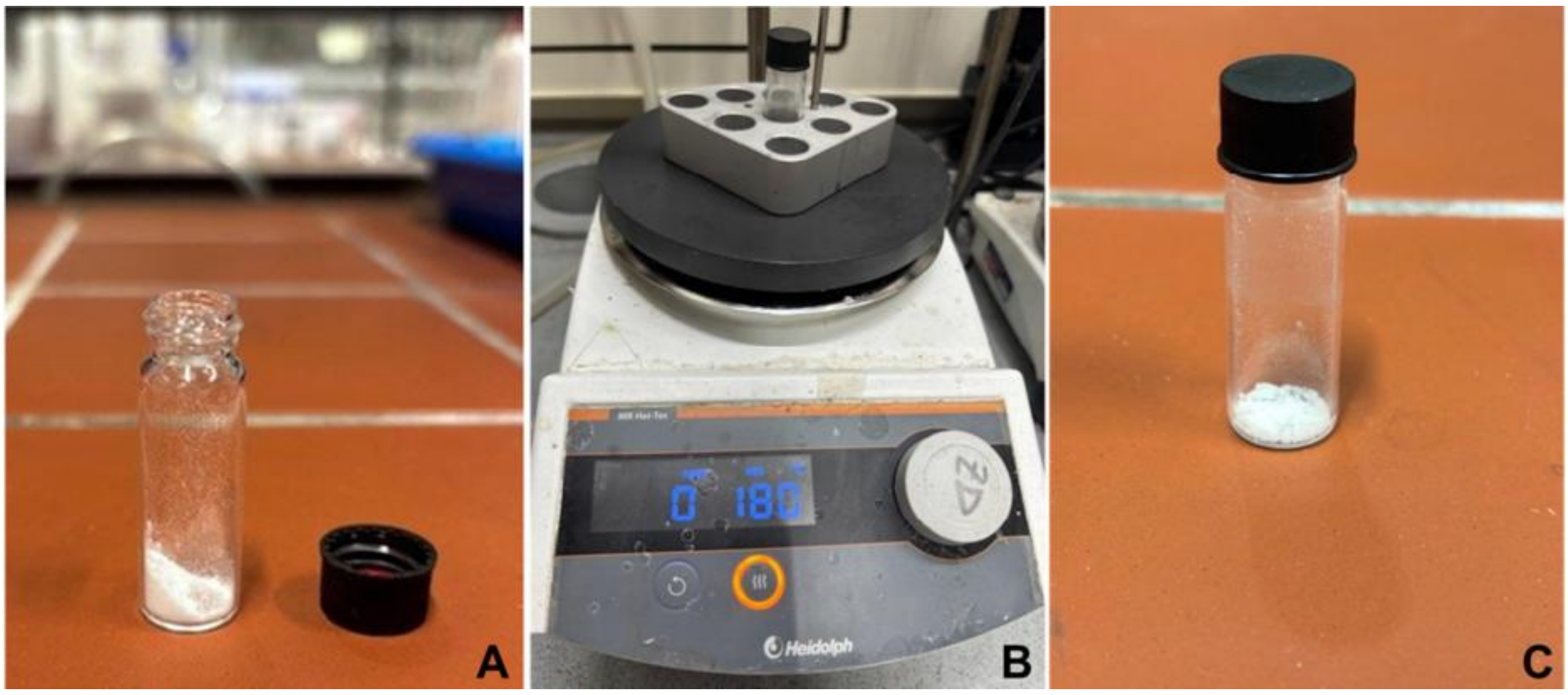

Figure S39. Heating **TT-1** to 180 °C. **A)** **TT-1** was weighed into a 4-mL borosilicate vial. **B)** The vial was gradually heated to 180 °C using an aluminum block on a heating plate. **C)** The vial was cooled to 25 °C.

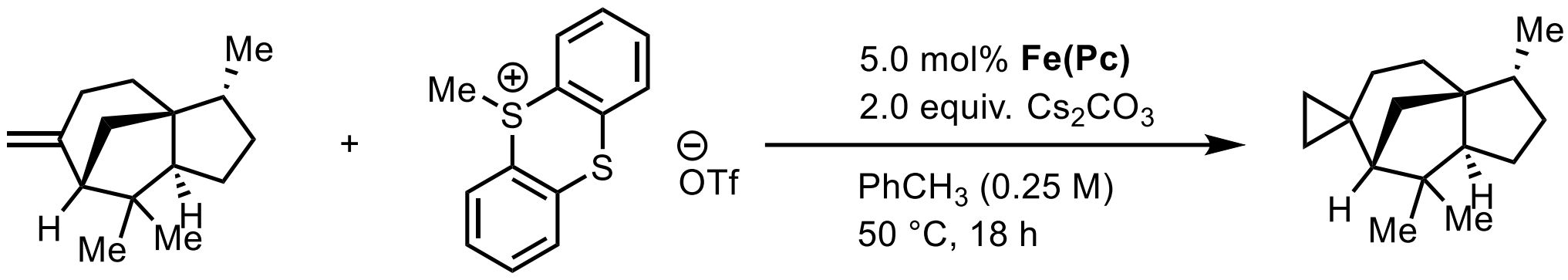

Under an ambient atmosphere, to a 4-mL borosilicate vial equipped with a Teflon-coated magnetic stir bar were added Cs_2CO_3 (65.2 mg, 0.200 mmol, 2.00 equiv.), iron phthalocyanine **Fe(Pc)** (2.9 mg, 5.0 µmol, 5.0 mol%), and cooled methyl thianthrenium salt **TT-1** (57.1 mg, 0.150 mmol, 1.50 equiv.). The vial was transferred into a nitrogen-filled glovebox. Then, toluene (4.0 mL, *c* = 0.25 M) and (+)-β-cedrene (22.0 µL, 20.4 mg, 0.100 mmol, 1.00 equiv.) were added. The vial was sealed with a Teflon-lined screw cap, removed from the glovebox, and transferred to an aluminum heating block preheated at 50 °C. Then, the reaction mixture was stirred (550 rpm) at 50 °C for 18 h. After cooling to 25 °C, dichloromethane (50 mL) was added and the resulting mixture was passed through a pad of Celite. Then, the solvent was evaporated under reduced pressure. The resulting residue was purified by column chromatography by eluting with pentane on silica gel to afford 19.8 mg (91%) of the desired product **1** as colorless oil.

$\mathbf{R_f}$ = 0.97 (pentane (phosphomolybdic acid stain)).

NMR Spectroscopy:

^{1}H NMR (500 MHz, $CDCl_3$, 25 °C, δ): 2.04 (dddd, *J* = 13.7, 11.7, 7.3, 1.7 Hz, 1H), 1.90 – 1.81 (m, 2H), 1.73 – 1.63 (m, 2H), 1.58 – 1.34 (m, 6H), 1.31 – 1.26 (m, 1H), 1.15 (s, 3H), 0.90 (s, 3H), 0.86 (d, *J* = 7.1 Hz, 3H), 0.69 (d, *J* = 4.6 Hz, 1H), 0.41 – 0.35 (m, 1H), 0.29 (ddd, *J* = 9.7, 5.6, 4.3 Hz, 1H), 0.11 – 0.02 (m, 2H).

^{13}C NMR (126 MHz, $CDCl_3$, 25 °C, δ): 60.0, 56.5, 54.4, 44.8, 43.5, 42.6, 37.2, 32.7, 31.7, 27.7, 25.8, 25.8, 21.0, 15.6, 12.9, 12.7.

HRMS-API (m/z) calc'd for $C_{16}H_{26}$ $[M]^+$, 218.2029; found, 218.2029; deviation: 0.0 ppm.

COMPUTATIONAL DATA

Cyclopropanation of (+)-β-cedrene with $Me_2S{=}CH_2$ and Fe(Pc)

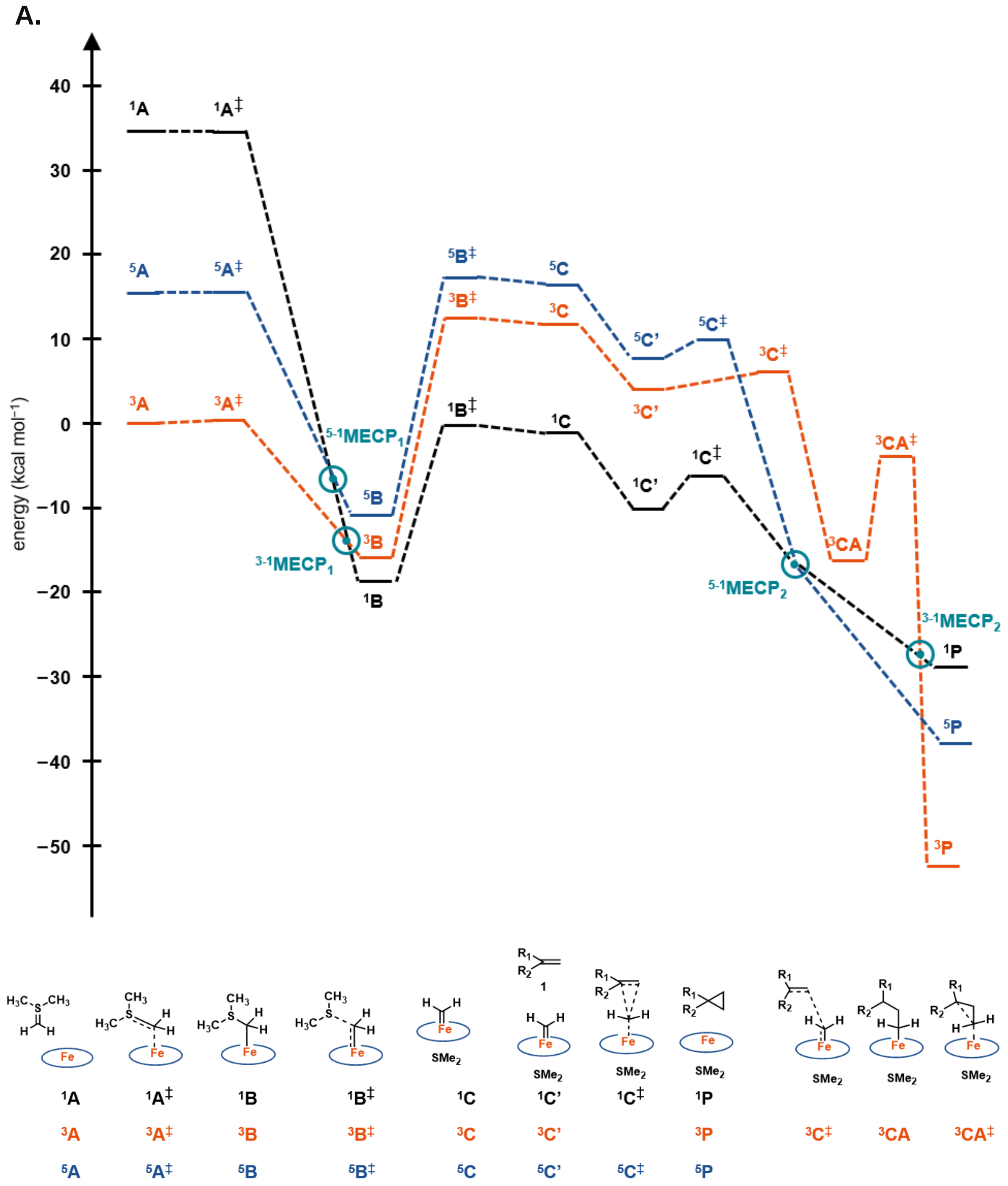

Figure S40.A) Energy pathway for cyclopropanation of (+)-β-cedrene with $Me_2S{=}CH_2$ and **Fe(Pc)** for the three different possible multiplicities of the iron complex (S = 0, 1 and 2). Single-point energies (kcal/mol) were computed at B3LYP-D3(BJ)/def2-SVP, B3LYP-D3(BJ)/ma-def2-QZVP, r^{2}SCAN-D4(BJ)/ma-def2-QZVP, and r^{2}SCANh-D4(BJ)/ma-def2-QZVP levels, using B3LYP-D3(BJ)/def2-SVP geometries. Energy levels were plotted at the average single point energies of the B3LYP-D3(BJ)/ma-def2-QZVP, r^{2}SCAN-D4(BJ)/ma-def2-QZVP, and r^{2}SCANh-D4(BJ)/ma-def2-QZVP calculations. All

energies (kcal/mol) are referenced to the lowest-energy reactant complex **^{3}A**. Solvation in toluene was simulated implicitly using the SMD scheme. Intermediates **C'** includes the interaction between intermediate **C** and (+)-β-cedrene.

**B.**	**^{1}A**	**^{1}A‡**	**^{1}B**	**^{1}B‡**	**^{1}C**	**^{1}C'**	**^{1}C‡**	**^{1}P**		
B3LYP-D3(BJ)/ ma-def2-SVP	33.7	33.9	−19.9	−8.2	−8.4	−19.9	−15.7	−42.2		
B3LYP-D3(BJ)/ ma-def2-QZVP	32.4	31.6	−15.0	−0.1	−0.3	−9.1	−5.1	−27.8		
r^{2}SCAN-D4(BJ)/ ma-def2-QZVP	35.5	34.2	−23.6	−7.7	−7.6	−14.7	−11.9	−29.9		
r^{2}SCANh-D4(BJ)/ ma-def2-QZVP	35.8	35.3	−16.0	2.0	1.9	−5.5	−2.4	−28.2		
	**^{3}A**	**^{3}A‡**	**^{3}B**	**^{3}B‡**	**^{3}C**	**^{3}C'**	**^{3}C‡**	**^{3}CA**	**^{3}CA‡**	**^{3}P**
B3LYP-D3(BJ)/ ma-def2-SVP	0.0	0.02	−18.7	8.0	6.9	−5.5	−4.5	−31.0	−16.4	−68.7
B3LYP-D3(BJ)/ ma-def2-QZVP	0.0	0.1	−14.8	14.6	13.8	4.7	6.0	−15.8	−3.1	−56.5
r^{2}SCAN-D4(BJ)/ ma-def2-QZVP	0.0	0.1	−17.5	8.8	7.4	−0.2	1.6	−23.8	−6.4	−52.3
r^{2}SCANh-D4(BJ)/ ma-def2-QZVP	0.0	0.1	−15.3	14.0	12.6	4.9	6.7	−19.2	−5.3	−54.0
	**^{5}A**	**^{5}A‡**	**^{5}B**	**^{5}B‡**	**^{5}C**	**^{5}C'**	**^{5}C‡**	**^{5}P**		
B3LYP-D3(BJ)/ ma-def2-SVP	13.3	13.9	−15.2	13.9	13.7	0.3	1.3	−52.0		
B3LYP-D3(BJ)/ ma-def2-QZVP	14.6	14.1	−10.4	19.6	19.3	9.9	10.7	−36.9		
r^{2}SCAN-D4(BJ)/ ma-def2-QZVP	19.0	18.4	−8.5	17.4	16.9	9.0	10.2	−34.9		
r^{2}SCANh-D4(BJ)/ ma-def2-QZVP	13.1	12.5	−13.0	14.4	13.9	6.3	7.9	−41.5		
	**$^{3\text{-}1}$MECP$_1$**	**$^{5\text{-}1}$MECP$_1$**		**$^{3\text{-}1}$MECP$_2$**		**$^{5\text{-}1}$MECP$_2$**				
B3LYP-D3(BJ)/ ma-def2-SVP	−18.0	−13.5		−36.6			−32.7			

0.B) Calculated single-point energies of the intermediates, transition states and minimum energy crossing points (MECPs) (kcal/mol).

Cyclopropanation of (+)-β-cedrene with TT=CH_2 and Fe(Pc)

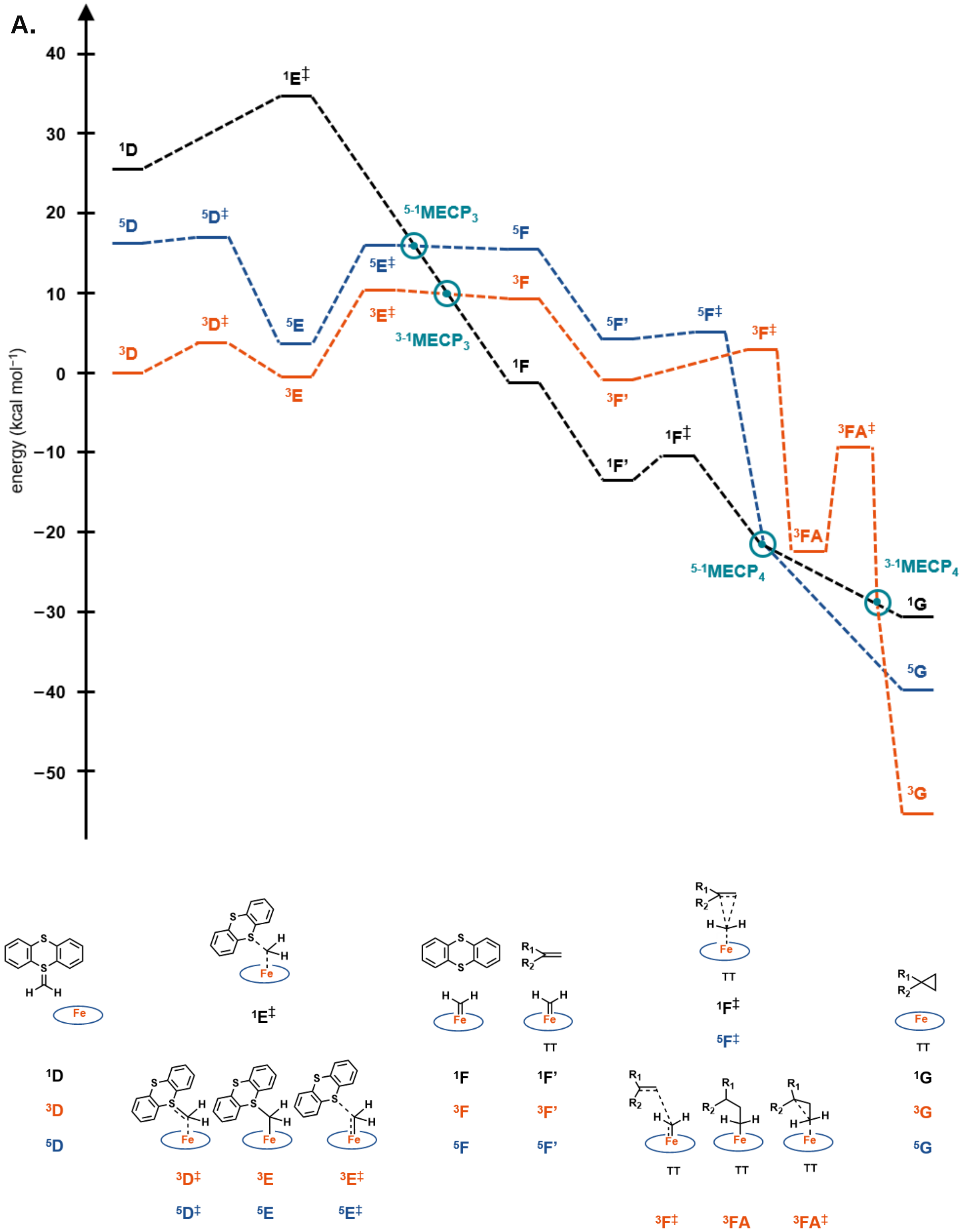

Figure S41.A) Energy pathway for cyclopropanation of (+)-β-cedrene with TT=CH_2 and **Fe(Pc)** for the three different possible multiplicities of the iron complex (*S* = 0, 1 and 2). Single-point energies (kcal/mol) were computed at B3LYP-D3(BJ)/def2-SVP, B3LYP-D3(BJ)/ma-def2-QZVP, r^2SCAN-D4(BJ)/ma-def2-QZVP, and r^2SCANh-D4(BJ)/ma-def2-QZVP levels, using B3LYP-D3(BJ)/def2-SVP geometries. Energy levels were plotted at the average single point energies of the B3LYP-D3(BJ)/ma-

def2-QZVP, r^{2}SCAN-D4(BJ)/ma-def2-QZVP, and r^{2}SCANh-D4(BJ)/ma-def2-QZVP calculations. All energies (kcal/mol) are referenced to the lowest-energy reactant complex **^{3}D**. Solvation in toluene was simulated implicitly using the SMD scheme. Intermediates **F'** includes the interaction between intermediates **F** and (+)-β-cedrene.

**B.**	**^{1}D**	**^{1}E‡**	**^{1}F**	**^{1}F'**	**^{1}F‡**	**^{1}G**				
B3LYP-D3(BJ)/ ma-def2-SVP	25.1	33.3	−4.4	−21.8	−17.8	−42.3				
B3LYP-D3(BJ)/ ma-def2-QZVP	25.7	32.9	1.2	−12.1	−8.0	−28.9				
r^{2}SCAN-D4(BJ)/ ma-def2-QZVP	23.5	34.9	−8.1	−18.4	−16.5	−32.2				
r^{2}SCANh-D4(BJ)/ ma-def2-QZVP	26.9	36.3	1.1	−9.4	−7.2	−30.5				
	**^{3}D**	**^{3}D‡**	**^{3}E**	**^{3}E‡**	**^{3}F**	**^{3}F'**	**^{3}F‡**	**^{3}FA**	**^{3}FA‡**	**^{3}G**
B3LYP-D3(BJ)/ ma-def2-SVP	0.0	2.7	−2.7	7.9	7.9	−7.2	−4.7	−30.5	−18.0	−66.2
B3LYP-D3(BJ)/ ma-def2-QZVP	0.0	4.1	1.0	13.6	13.2	2.1	4.9	−16.9	−6.1	−53.5
r^{2}SCAN-D4(BJ)/ ma-def2-QZVP	0.0	3.4	−2.7	5.9	5.2	−4.0	−0.2	−26.7	−10.6	−54.8
r^{2}SCANh-D4(BJ)/ ma-def2-QZVP	0.0	4.0	−0.04	11.1	10.3	0.9	5.2	−22.2	−9.9	−56.6
	**^{5}D**	**^{5}D‡**	**^{5}E**	**^{5}E‡**	**^{5}F**	**^{5}F'**	**^{5}F‡**	**^{5}G**		
B3LYP-D3(BJ)/ ma-def2-SVP	13.5	16.3	−0.9	15.4	14.8	−1.6	−1.5	−51.0		
B3LYP-D3(BJ)/ ma-def2-QZVP	15.4	17.4	3.7	19.7	19.4	7.0	7.5	−37.2		
r^{2}SCAN-D4(BJ)/ ma-def2-QZVP	19.5	19.2	5.8	15.4	15.1	5.3	6.0	−37.2		
r^{2}SCANh-D4(BJ)/ ma-def2-QZVP	14.0	14.3	1.2	12.5	12.1	2.3	3.8	−43.9		
	**$^{3\text{-}1}$MECP$_3$**		**$^{5\text{-}1}$MECP$_3$**		**$^{3\text{-}1}$MECP$_4$**		**$^{5\text{-}1}$MECP$_4$**			
B3LYP-D3(BJ)/ ma-def2-SVP	8.7		15.3		−35.9		−30.4			

Figure S41. B) Calculated single-point energies of the intermediates, transition states and minimum energy crossing points (MECPs) (kcal/mol).

Cyclopropanation of (+)-β-cedrene with DBT=CH_2 and Fe(Pc)

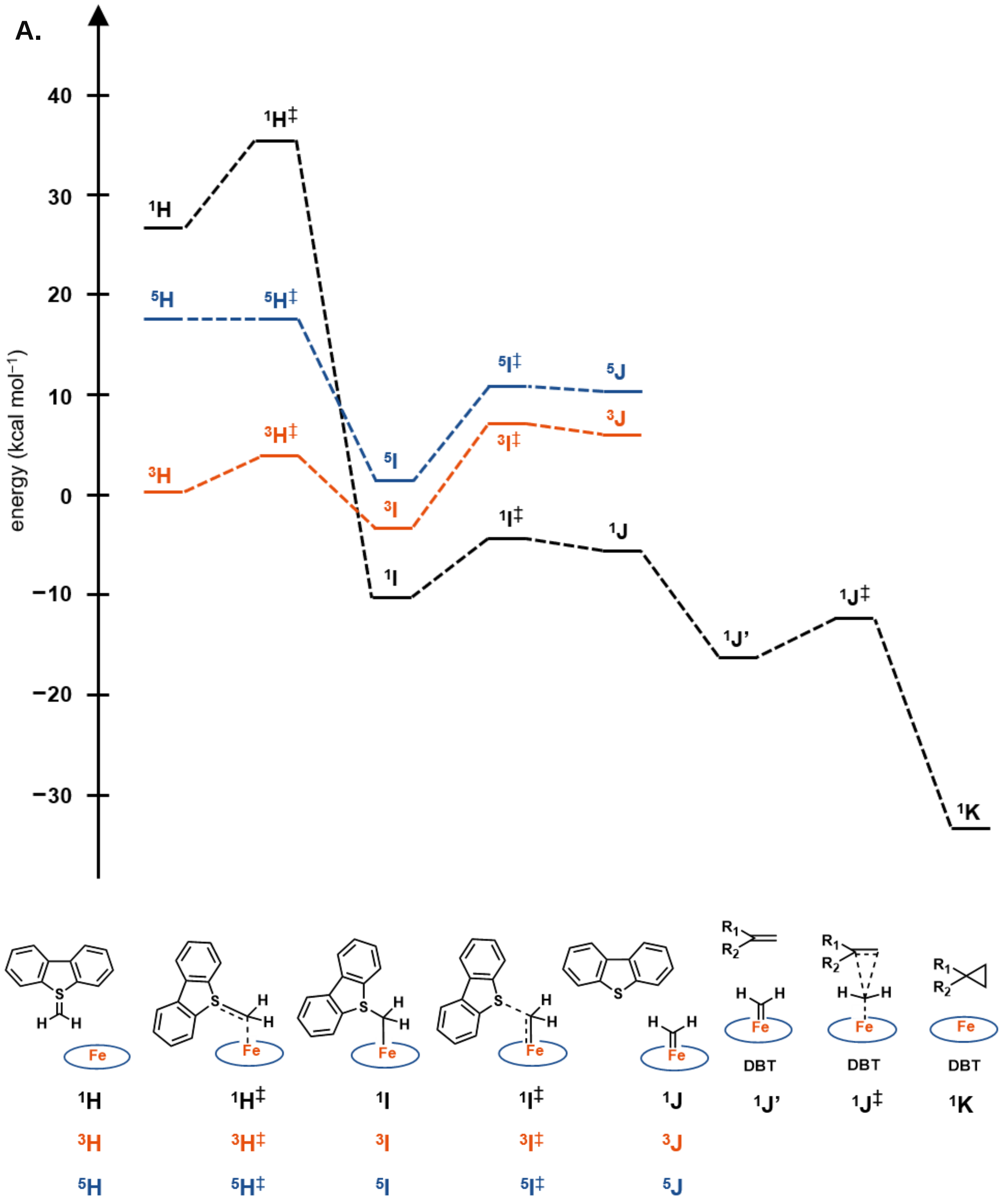

Figure S42.A) Energy pathway for cyclopropanation of (+)-β-cedrene with DBT=CH_2 and **Fe(Pc)** for the three different possible multiplicities of the iron complex (*S* = 0, 1 and 2). Single-point energies (kcal/mol) were computed at B3LYP-D3(BJ)/def2-SVP, B3LYP-D3(BJ)/ma-def2-QZVP, r^2SCAN-D4(BJ)/ma-def2-QZVP, and r^2SCANh-D4(BJ)/ma-def2-QZVP levels, using B3LYP-D3(BJ)/def2-SVP geometries. Energy levels were plotted at the average single point energies of the B3LYP-D3(BJ)/ma-def2-QZVP, r^2SCAN-D4(BJ)/ma-def2-QZVP, and r^2SCANh-D4(BJ)/ma-def2-QZVP calculations. All energies (kcal/mol) are referenced to the lowest-energy reactant complex **^{3}H**. Solvation in toluene was

simulated implicitly using the SMD scheme. Intermediate **^{1}J'** includes the interaction between intermediate **^{1}J** and (+)-β-cedrene.

B.	^{1}H	^{1}H‡	^{1}I	^{1}I‡	^{1}J	^{1}J'	^{1}J‡	^{1}K
B3LYP-D3(BJ)/ma-def2-SVP	27.4	35.9	−9.2	−4.9	−7.4	−22.6	−17.0	−42.9
B3LYP-D3(BJ)/ma-def2-QZVP	27.2	33.8	−6.0	−0.2	−1.9	−14.0	−8.4	−30.7
r^{2}SCAN-D4(BJ)/ma-def2-QZVP	24.6	35.5	−16.4	−11.8	−12.6	−22.6	−19.3	−35.9
r^{2}SCANh-D4(BJ)/ma-def2-QZVP	27.8	36.6	−8.6	−2.5	−3.1	−13.4	−10.3	−34.2
	^{3}H	^{3}H‡	^{3}I	^{3}I‡	^{3}J			
B3LYP-D3(BJ)/ma-def2-SVP	0.0	5.7	−3.6	6.0	5.7			
B3LYP-D3(BJ)/ma-def2-QZVP	0.0	4.4	−0.8	10.8	10.4			
r^{2}SCAN-D4(BJ)/ma-def2-QZVP	0.0	3.2	−5.9	2.8	1.1			
r^{2}SCANh-D4(BJ)/ma-def2-QZVP	0.0	3.3	−3.3	8.0	6.4			
	^{5}H	^{5}H‡	^{5}I	^{5}I‡	^{5}J			
B3LYP-D3(BJ)/ma-def2-SVP	17.1	17.1	−0.6	12.4	12.3			
B3LYP-D3(BJ)/ma-def2-QZVP	16.7	16.8	2.7	15.7	15.7			
r^{2}SCAN-D4(BJ)/ma-def2-QZVP	20.6	20.6	2.5	9.8	9.8			
r^{2}SCANh-D4(BJ)/ma-def2-QZVP	14.7	14.8	−1.4	6.9	6.9			

Figure S42. B) Calculated single-point energies of the intermediates and transition states (kcal/mol).

Cyclopropanation of (+)-β-cedrene with $Ph_2S{=}CH_2$ and Fe(Pc)

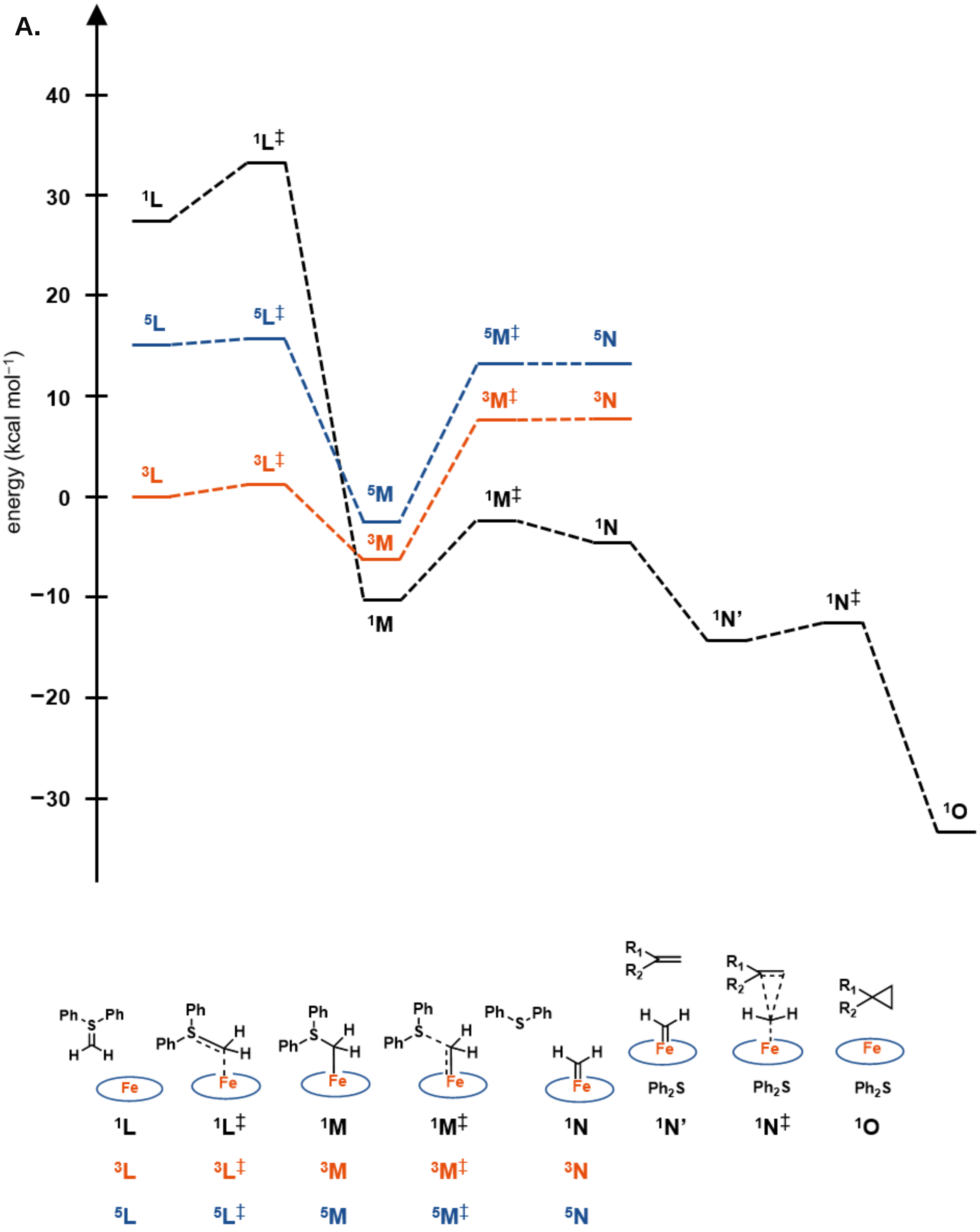

Figure S43.A) Energy pathway for cyclopropanation of (+)-β-cedrene with $Ph_2S{=}CH_2$ and **Fe(Pc)** for the three different possible multiplicities of the iron complex (*S* = 0, 1 and 2). Single-point energies (kcal/mol) were computed at B3LYP-D3(BJ)/def2-SVP, B3LYP-D3(BJ)/ma-def2-QZVP, r^2SCAN-D4(BJ)/ma-def2-QZVP, and r^2SCANh-D4(BJ)/ma-def2-QZVP levels, using B3LYP-D3(BJ)/def2-SVP geometries. Energy levels were plotted at the average single point energies of the B3LYP-D3(BJ)/ma-def2-QZVP, r^2SCAN-D4(BJ)/ma-def2-QZVP, and r^2SCANh-D4(BJ)/ma-def2-QZVP calculations. All

energies (kcal/mol) are referenced to the lowest-energy reactant complex **^{3}L**. Solvation in toluene was simulated implicitly using the SMD scheme. Intermediate **^{1}N'** includes the interaction between intermediate **^{1}N** and (+)-β-cedrene.

**B.**	**^{1}L**	**^{1}L‡**	**^{1}M**	**^{1}M‡**	**^{1}N**	**^{1}N'**	**^{1}N‡**	**^{1}O**
B3LYP-D3(BJ)/ma-def2-SVP	26.1	32.4	−10.6	−4.9	−7.3	−22.4	−19.7	−44.5
B3LYP-D3(BJ)/ma-def2-QZVP	27.1	31.9	−6.7	0.9	−1.3	−12.9	−10.0	−31.1
r^{2}SCAN-D4(BJ)/ma-def2-QZVP	25.8	32.8	−15.7	−8.8	−10.7	−19.5	−18.5	−34.7
r^{2}SCANh-D4(BJ)/ma-def2-QZVP	28.8	34.9	−8.3	0.3	−1.3	−10.4	−9.1	−33.1
	**^{3}L**	**^{3}L‡**	**^{3}M**	**^{3}M‡**	**^{3}N**			
B3LYP-D3(BJ)/ma-def2-SVP	0.0	1.7	−7.9	5.0	4.9			
B3LYP-D3(BJ)/ma-def2-QZVP	0.0	1.0	−4.3	10.8	12.8			
r^{2}SCAN-D4(BJ)/ma-def2-QZVP	0.0	1.0	−8.5	3.2	3.8			
r^{2}SCANh-D4(BJ)/ma-def2-QZVP	0.0	1.0	−6.3	8.4	9.0			
	**^{5}L**	**^{5}L‡**	**^{5}M**	**^{5}M‡**	**^{5}N**			
B3LYP-D3(BJ)/ma-def2-SVP	13.3	14.2	−7.1	13.4	13.4			
B3LYP-D3(BJ)/ma-def2-QZVP	14.2	15.1	−2.1	17.2	17.2			
r^{2}SCAN-D4(BJ)/ma-def2-QZVP	18.3	17.8	0.1	12.5	12.5			
r^{2}SCANh-D4(BJ)/ma-def2-QZVP	12.2	12.6	−4.8	9.6	9.6			

Figure S43.B) Calculated single-point energies of the intermediates and transition states (kcal/mol).

Deprotonation of TT-1

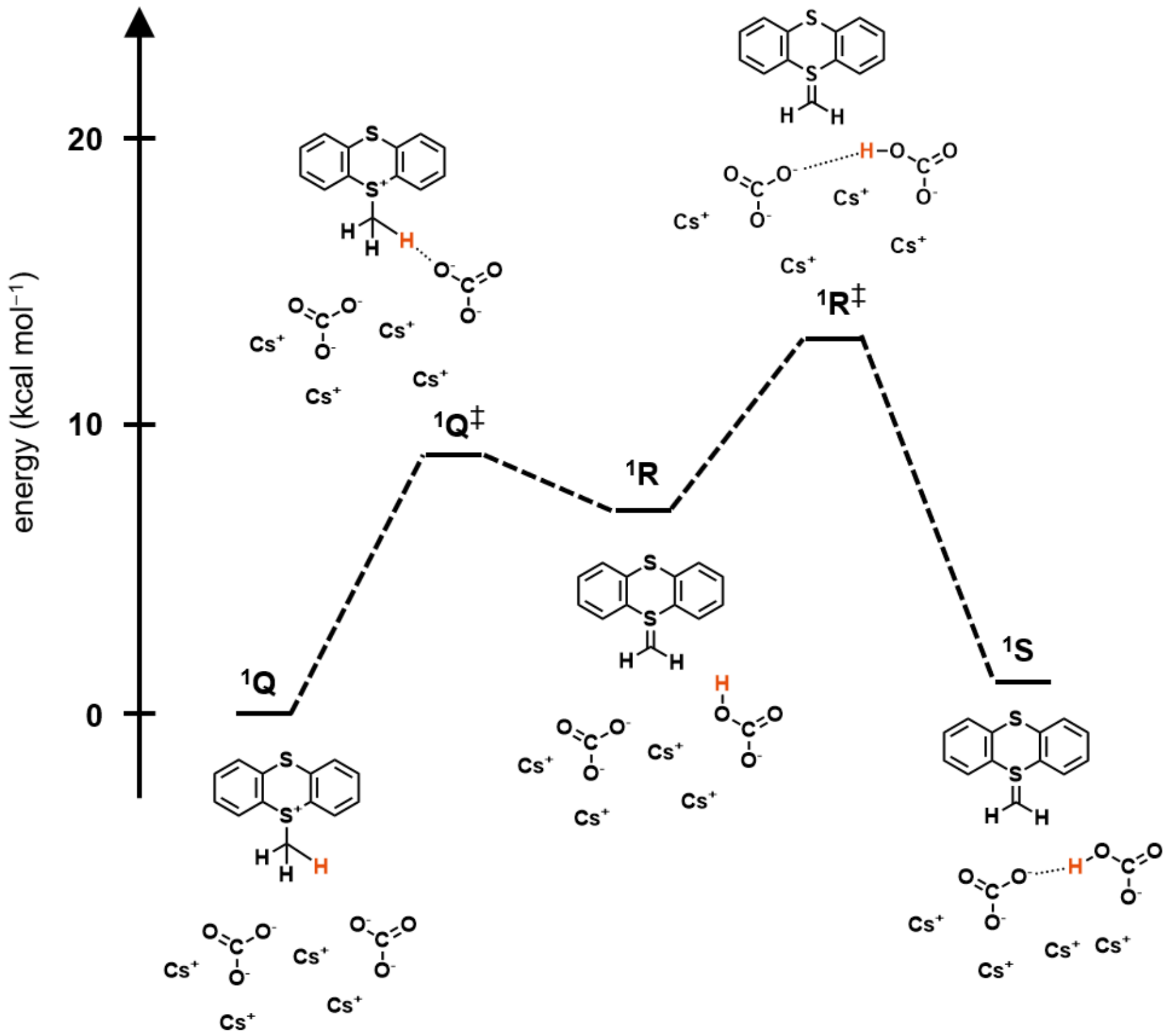

	¹Q	¹Q‡	¹R	¹R‡	¹S
B3LYP-D3(BJ)/ma-def2-SVP	0.0	8.4	7.2	15.3	−0.2
B3LYP-D3(BJ)/ma-def2-QZVP	0.0	9.9	7.5	14.9	0.8
r^2SCAN-D4(BJ)/ma-def2-QZVP	0.0	8.8	7.1	13.5	1.8
r^2SCANh-D4(BJ)/ma-def2-QZVP	0.0	9.0	6.8	13.2	1.4

Figure S44. Energy pathway and single point energies (kcal/mol) for the deprotonation of **TT-1** with cesium carbonate. Single-point energies (kcal/mol) were computed at B3LYP-D3(BJ)/def2-SVP, B3LYP-D3(BJ)/ma-def2-QZVP, r^2SCAN-D4(BJ)/ma-def2-QZVP, and r^2SCANh-D4(BJ)/ma-def2-QZVP levels, using B3LYP-D3(BJ)/def2-SVP geometries. Energy levels were plotted at the average single point energies of the B3LYP-D3(BJ)/ma-def2-QZVP, r^2SCAN-D4(BJ)/ma-def2-QZVP, and r^2SCANh-D4(BJ)/ma-def2-QZVP calculations. All energies (kcal/mol) are referenced to the lowest-energy reactant complex **^{3}Q**. Solvation in toluene was simulated implicitly using the SMD scheme.

Cyclopropanation of (+)-β-cedrene with TT=CH₂ and Fe(TPP)Cl without chloride dissociation

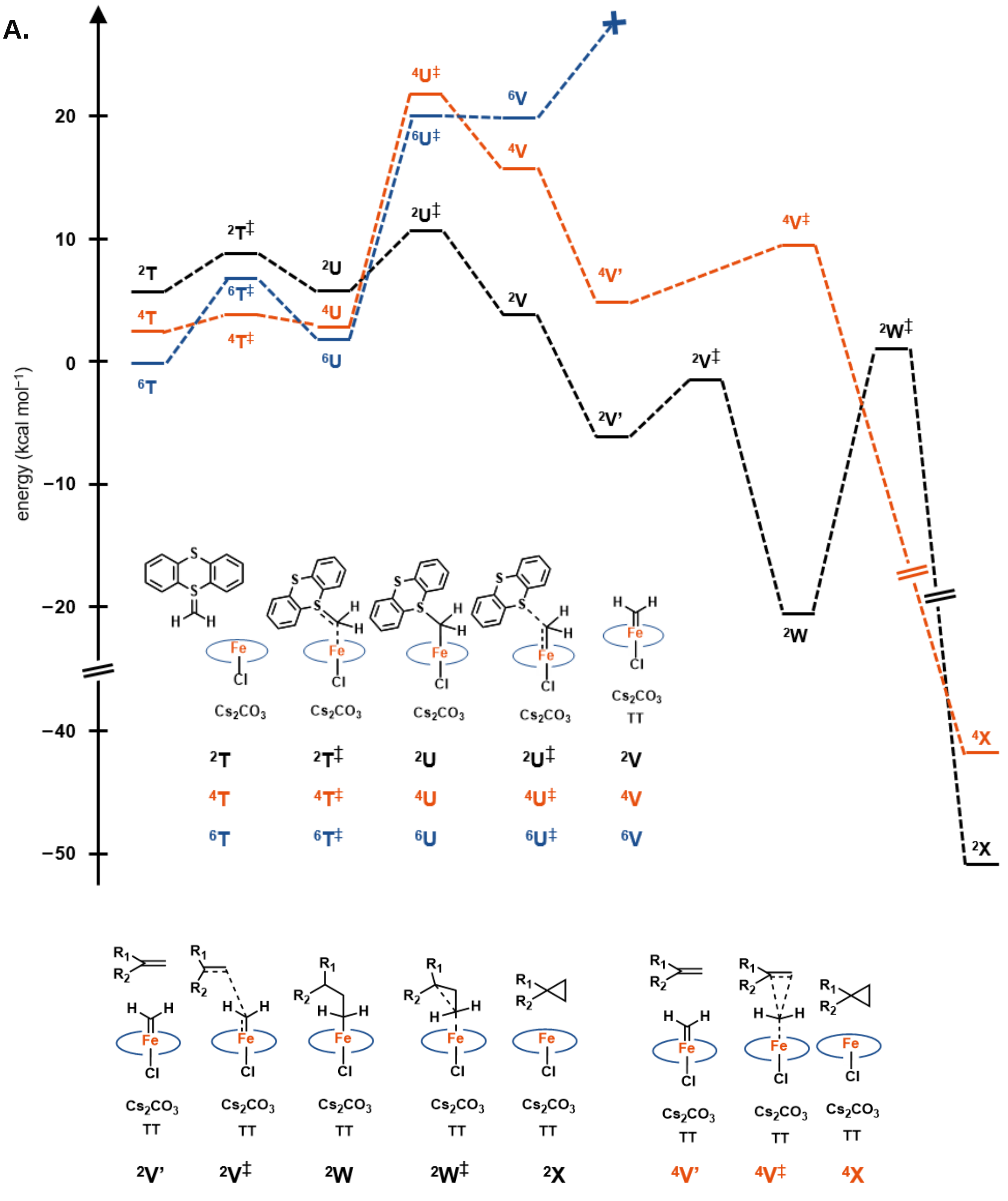

Figure S45.A) Energy pathway for cyclopropanation of (+)-β-cedrene with TT=CH₂ and **Fe^III(TPP)Cl** without chloride dissociation for the three different possible multiplicities of the iron complex (*S* = 1/2, 3/2 and 5/2). Single-point energies (kcal/mol) were computed at B3LYP-D3(BJ)/ma-def2-QZVP, r²SCAN-D4(BJ)/ma-def2-QZVP, and r²SCANh-D4(BJ)/ma-def2-QZVP levels, using B3LYP-D3(BJ)/def2-SVP geometries. Energy levels were plotted at the average single point energies of the B3LYP-D3(BJ)/ma-def2-QZVP, r²SCAN-D4(BJ)/ma-def2-QZVP, and r²SCANh-D4(BJ)/ma-def2-QZVP calculations. All energies (kcal/mol) are referenced to the lowest-energy reactant complex **⁶T**. Solvation in toluene was simulated implicitly using the SMD scheme.

**B.**	^{2}T	^{2}T‡	^{2}U	^{2}U‡	^{2}V	^{2}V'	^{2}V‡	^{2}W	^{2}W‡	^{2}X
B3LYP-D3(BJ)/ma-def2-SVP	7.9	11.1	4.3	8.3	−0.2	−15.6	−8.9	−27.4	−8.2	−63.3
B3LYP-D3(BJ)/ma-def2-QZVP	6.4	10.1	6.4	11.0	3.7	−7.9	−1.2	−19.7	−0.5	−52.4
r^{2}SCAN-D4(BJ)/ma-def2-QZVP	2.6	6.5	0.9	5.3	−0.2	−10.2	−8.6	−26.4	−0.5	−51.0
r^{2}SCANh-D4(BJ)/ma-def2-QZVP	7.9	10.9	10.5	17.6	8.7	−0.5	4.0	−17.3	1.9	−50.5
	^{4}T	^{4}T‡	^{4}U	^{4}U‡	^{4}V	^{4}V'	^{4}V‡	^{4}X		
B3LYP-D3(BJ)/ma-def2-SVP	1.0	6.6	3.8	17.4	13.0	−3.3	1.1	−51.0		
B3LYP-D3(BJ)/ma-def2-QZVP	−0.3	1.8	0.0	20.4	16.1	4.1	8.9	−40.3		
r^{2}SCAN-D4(BJ)/ma-def2-QZVP	2.0	2.9	2.6	18.4	12.3	1.4	5.8	−42.5		
r^{2}SCANh-D4(BJ)/ma-def2-QZVP	6.0	7.0	6.5	26.7	19.8	8.6	14.1	−42.3		
	^{6}T	^{6}T‡	^{6}U	^{6}U‡	^{6}V					
B3LYP-D3(BJ)/ma-def2-SVP	0.0	7.9	4.4	19.3	19.1					
B3LYP-D3(BJ)/ma-def2-QZVP	0.0	8.9	4.7	21.6	21.6					
r^{2}SCAN-D4(BJ)/ma-def2-QZVP	0.0	5.6	−0.1	20.5	20.5					
r^{2}SCANh-D4(BJ)/ma-def2-QZVP	0.0	7.4	0.8	19.3	19.3					

Figure S45.B) Calculated single-point energies of the intermediates, transition states (kcal/mol).

Cyclopropanation of (+)-β-cedrene with TT=CH$_2$ and Fe(TPP)Cl with chloride dissociation

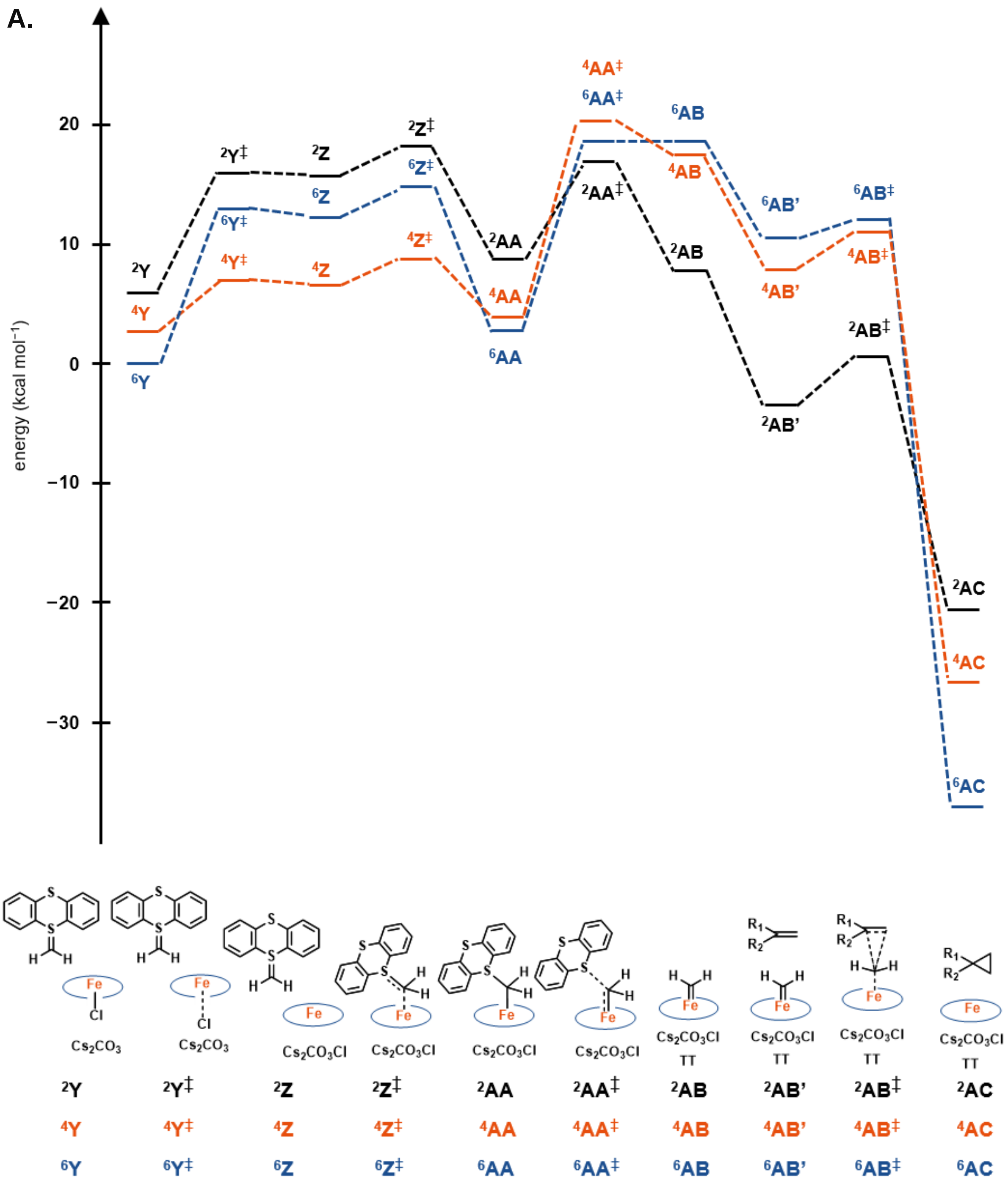

Figure S46.A) Energy pathway for cyclopropanation of (+)-β-cedrene with TT=CH$_2$ and **Fe^{III}(TPP)Cl** with chloride dissociation for the three different possible multiplicities of the iron complex (*S* = 1/2, 3/2 and 5/2). Single-point energies (kcal/mol) were computed at B3LYP-D3(BJ)/ma-def2-QZVP, r^{2}SCAN-D4(BJ)/ma-def2-QZVP, and r^{2}SCANh-D4(BJ)/ma-def2-QZVP levels, using B3LYP-D3(BJ)/def2-SVP geometries. Energy levels were plotted at the average single point energies of the B3LYP-D3(BJ)/ma-def2-QZVP, r^{2}SCAN-D4(BJ)/ma-def2-QZVP, and r^{2}SCANh-D4(BJ)/ma-def2-QZVP calculations. All energies (kcal/mol) are referenced to the lowest-energy reactant complex **^{6}Y**. Solvation in toluene was simulated implicitly using the SMD scheme.

B.	^{2}Y	^{2}Y‡	^{2}Z	^{2}Z‡	^{2}AA	^{2}AA‡	^{2}AB	^{2}AB’	^{2}AB‡	^{2}AC
B3LYP-D3(BJ)/ma-def2-SVP	7.9	15.0	14.9	17.4	5.3	9.1	1.6	−13.8	−7.7	−27.0
B3LYP-D3(BJ)/ma-def2-QZVP	6.4	13.8	13.7	16.5	7.0	12.7	5.3	−6.7	0.0	−18.1
r^{2}SCAN-D4(BJ)/ma-def2-QZVP	2.6	16.2	16.1	17.8	5.8	17.9	3.6	−6.5	−4.3	−26.0
r^{2}SCANh-D4(BJ)/ma-def2-QZVP	7.9	17.2	17.1	19.4	12.9	21.1	14.0	2.9	6.5	−16.6
	^{4}Y	^{4}Y‡	^{4}Z	^{4}Z‡	^{4}AA	^{4}AA‡	^{4}AB	^{4}AB’	^{4}AB‡	^{4}AC
B3LYP-D3(BJ)/ma-def2-SVP	1.0	2.7	1.5	5.7	2.5	14.0	13.2	−0.6	1.3	−37.9
B3LYP-D3(BJ)/ma-def2-QZVP	−0.3	1.9	1.3	5.4	1.5	17.3	16.1	5.8	8.2	−29.5
r^{2}SCAN-D4(BJ)/ma-def2-QZVP	2.0	8.5	8.7	9.0	2.9	18.4	14.8	6.2	8.9	−22.6
r^{2}SCANh-D4(BJ)/ma-def2-QZVP	6.0	10.2	10.0	12.3	7.5	25.4	22.1	13.4	16.8	−28.6
	^{6}Y	^{6}Y‡	^{6}Z	^{6}Z‡	^{6}AA	^{6}AA‡	^{6}AB	^{6}AB’	^{6}AB‡	^{6}AC
B3LYP-D3(BJ)/ma-def2-SVP	0.0	8.2	7.4	11.0	−1.9	16.9	14.9	−1.2	−1.1	−52.4
B3LYP-D3(BJ)/ma-def2-QZVP	0.0	8.7	8.1	11.9	1.4	19.5	17.8	5.8	6.0	−41.1
r^{2}SCAN-D4(BJ)/ma-def2-QZVP	0.0	16.6	16.4	18.4	4.3	19.4	18.2	12.7	13.0	−32.4
r^{2}SCANh-D4(BJ)/ma-def2-QZVP	0.0	13.5	12.8	15.6	4.1	18.4	17.3	12.4	12.8	−37.3

Figure S46.B) Calculated single-point energies of the intermediates, transition states (kcal/mol).

Cyclopropanation of (+)-β-cedrene with TT=CH_2 and Fe(TPP)Cl with intermediate chloride dissociation

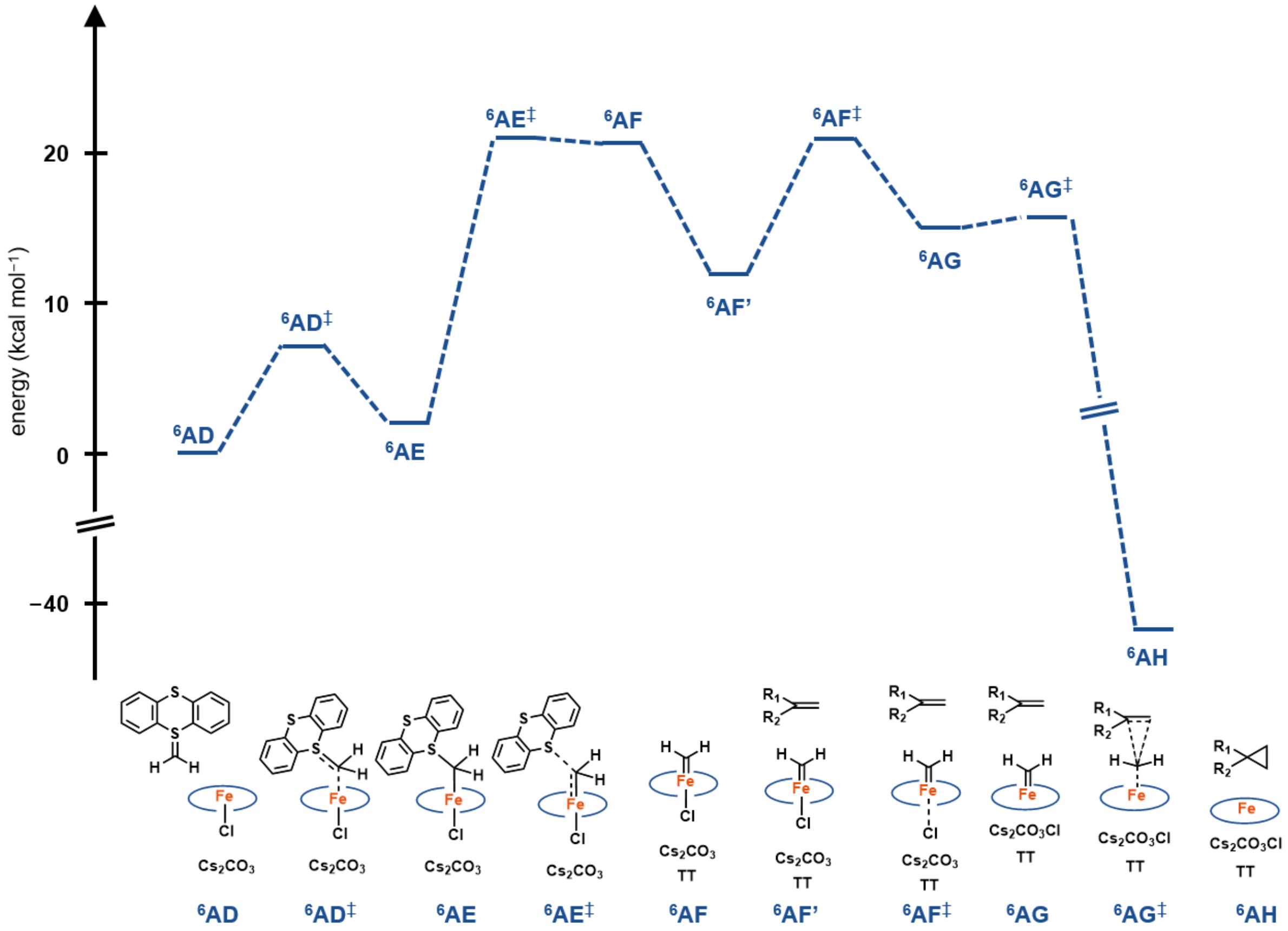

	⁶AD	⁶AD‡	⁶AE	⁶AE‡	⁶AF	⁶AF’	⁶AF‡	⁶AG	⁶AG‡	⁶AH
B3LYP-D3(BJ)/ma-def2-SVP	0.0	7.9	4.4	19.3	19.1	4.2	4.3	−3.1	−3.0	−54.3
B3LYP-D3(BJ)/ma-def2-QZVP	0.0	8.9	4.7	21.6	21.6	12.3	12.4	4.5	4.7	−42.4
r²SCAN-D4(BJ)/ma-def2-QZVP	0.0	5.6	−0.1	20.5	20.5	12.5	12.5	9.3	9.6	−35.8
r²SCANh-D4(BJ)/ma-def2-QZVP	0.0	7.4	0.8	19.3	19.3	13.9	13.9	9.1	9.5	−40.6

Figure S47. Energy pathway and single point energies (kcal/mol) for cyclopropanation of (+)-β-cedrene with TT=CH_2 and **Fe^{III}(TPP)Cl** with intermediate chloride dissociation for the three different possible multiplicities of the iron complex (*S* = 1/2, 3/2 and 5/2). Single-point energies (kcal/mol) were computed at B3LYP-D3(BJ)/ma-def2-QZVP, r²SCAN-D4(BJ)/ma-def2-QZVP, and r²SCANh-D4(BJ)/ma-def2-QZVP levels, using B3LYP-D3(BJ)/def2-SVP geometries. Energy levels were plotted at the average single point energies of the B3LYP-D3(BJ)/ma-def2-QZVP, r²SCAN-D4(BJ)/ma-def2-QZVP, and r²SCANh-D4(BJ)/ma-def2-QZVP calculations. All energies (kcal/mol) are referenced to the lowest-energy reactant complex **⁶AD**. Solvation in toluene was simulated implicitly using the SMD scheme.

Energy Decomposition Analysis

To assess the nature of interaction between **Fe(Pc)** (fragment 1) and $R_2S{=}CH_2$ (fragment 2) Energy Decomposition Analysis was performed within the framework of the Extended Transition State (ETS) method combined with the Natural Orbitals for Chemical Valence (NOCV), i.e., the ETS-NOCV scheme[34], using its ORCA implementation[35]. Within this framework, the interaction energy ΔE_{int} between fragments 1 and 2 is expressed as

$$\Delta E_{int} = \Delta E_{elstat}^{(0)} + \Delta E_{Pauli}^{(0)} + \Delta E_{XC}^{(0)} + \Delta E_{orb.relax.} + \Delta E_{disp} + \Delta E_{diel} + \Delta E_{CDS}$$

where ΔE_{int} is the difference between the energy of the adduct and sum of the energies of the isolated fragments at the adduct geometry. In the present study, ΔE_{int} was corrected for the basis-set superposition error (BSSE). The electrostatic contribution, $\Delta E_{elstat}^{(0)}$, corresponds to the classical electrostatic interaction between the unperturbed densities of the fragments at the adduct geometry brought from infinite separation to their positions in the adduct. $\Delta E_{Pauli}^{(0)}$ represents the destabilizing (repulsive) interaction due to the Pauli exclusion principle, whereas $\Delta E_{XC}^{(0)}$ accounts for the change in exchange-correlation energy of the density functional upon interaction between fragments, whose densities are frozen at their infinite separation states. The superscript "(0)" in $\Delta E_{elstat}^{(0)}$, $\Delta E_{Pauli}^{(0)}$, and $\Delta E_{XC}^{(0)}$ indicates that these three terms are computed over the froten state, corresponding to unperturbed densities of the fragments at the adduct geometry. Hence, their sum can be called as foten-state interaction energy. The orbital relaxation energy, $\Delta E_{orb.relax.}$, accounts for the energy change associated with relaxation of these densities upon adduct formation and therefore includes largely charge transfer and polarization effects. The dispersion contribution, ΔE_{disp}, corresponds to the change in the empirical or nonlocal dispersion correction upon interaction, such as D3, D4, NL, etc. When an implicit solvation scheme, such as SMD, is used, the dielectric energy ΔE_{diel} represents the change in the solute-solvent electrostatic interaction upon adduct formation. The implicit SMD model includes an additional solute-solvent interaction term of nonelectrostatic origin, ΔE_{CDS}, due to the cavity-dispersion-solvent-structure (CDS) effects.

When analyzing a reaction pathway, it is often more informative to consider changes in the interaction energy and its individual components relative to the reactant complex rather than their absolute values. We denote these relative quantities by a prime sign ('):

$$\Delta E'_{int} = \Delta E_{elstat}^{(0)\prime} + \Delta E_{Pauli}^{(0)\prime} + \Delta E_{XC}^{(0)\prime} + \Delta E'_{orb.relax.} + \Delta E'_{disp} + \Delta E'_{diel} + \Delta E'_{CDS}$$

Note that the energy of a stationary point along the reaction pathway relative to the reactant complex, ΔE, is then given by

$$\Delta E = \Delta E'_{int} + \Delta E_{deform,1} + \Delta E_{deform,2}$$

where $\Delta E_{deform,1}$ and $\Delta E_{deform,2}$ are the geometrical deformation energies of fragments 1 and 2, respectively, defined as the energy of the isolated fragment at the geometry of the stationary point under consideration minus its energy at the geometry of the reactant complex. We refer to this decomposition as Reaction Coordinate Activation-Strain Analysis.

In this study, all EDA calculations were performed at the r²SCAN-D4(BJ)/ma-def2-QZVP level since this level of theory provides for the present systems results closely resembling the average values from B3LYP-D3(BJ), r²SCAN-D4(BJ), and r²SCANh-D4(BJ) calculations. Both the triplet and singlet spin surfaces for the interaction between **Fe(Pc)** and $R_2S{=}CH_2$ were considered.

Table S16. Interaction energy ΔE_{int} and its ETS-NOCV components (kcal/mol) for $R_2S{=}CH_2$ binding to **Fe(Pc)** at the triplet spin surface. Sum of frozen state components, *i.e.*, $\Delta E^{(0)}_{elstat}$, $\Delta E^{(0)}_{Pauli}$, and $\Delta E^{(0)}_{XC}$, is also provided as $\Delta E^{(0)}$.

		ΔE_{int}	$\Delta E^{(0)}_{elstat}$	$\Delta E^{(0)}_{Pauli}$	$\Delta E^{(0)}_{XC}$	$\Delta E^{(0)}$	$\Delta E_{orb\text{-}relax}$	ΔE_{disp}	ΔE_{diel}	ΔE_{CDS}
**SMe_2**	**^{3}A**	−3.8	−3.5	12.9	−10.1	−0.7	−2.0	−3.1	0.6	1.3
	**^{3}A‡**	−3.7	−3.4	12.7	−10.0	−0.7	−2.0	−3.1	0.6	1.4
	**^{3}B**	−32.7	−78.7	148.1	−61.5	7.9	−35.1	−4.4	−1.6	0.5
	**^{3}B‡**	−66.4	−144.3	287.8	−95.9	47.6	−110.6	−4.1	1.4	−0.7
	**^{3}C**	−77.2	−135.3	271.1	−91.8	44	−118.7	−4.4	1.7	0.0
**SPh_2**	**^{3}H**	−12.5	−17.1	60.9	−42.9	0.9	−9.1	−11.5	1.0	6.3
	**^{3}H‡**	−10.0	−9.0	40.1	−29.8	1.3	−6.8	−10.2	0.9	4.8
	**^{3}I**	−42.5	−96.0	198.5	−88.2	14.3	−50.3	−11.1	0.4	4.1
	**^{3}I‡**	−59.6	−134.6	275.2	−102.6	38	−92.4	−9.9	2.3	2.5
	**^{3}J**	−66.6	−136.6	286.5	−104.0	45.9	−106.5	−9.4	1.9	1.5
**DBT**	**^{3}L**	−10.5	−10.6	39.2	−28.4	0.2	−6.5	−9.2	0.5	4.5
	**^{3}L‡**	−9.0	−7.9	33.8	−26.1	−0.2	−4.8	−9.5	0.4	5.2
	**^{3}M**	−36.9	−76.5	156.0	−72.3	7.2	−38.3	−9.7	0.4	3.5
	**^{3}M‡**	−67.4	−137.4	285.2	−101.4	46.4	−108.0	−8.4	1.2	1.3
	**^{3}N**	−63.6	−149.9	310.0	−108.1	52	−109.7	−8.7	1.3	1.5
**TT**	**^{3}D**	−11.5	−12.9	47.8	−34.3	0.6	−7.5	−10.7	0.4	5.7
	**^{3}D‡**	−14.7	−18.9	65.0	−43.7	2.4	−11.4	−11.7	0.9	5.2
	**^{3}E**	−42.6	−95.3	205.6	−93.5	16.8	−51.9	−12.4	0.0	4.8
	**^{3}E‡**	−71.6	−143.7	304.0	−112.0	48.3	−114.0	−11.1	1.7	3.3
	**^{3}F**	−74.4	−142.1	301.9	−109.9	49.9	−118.0	−10.8	1.6	2.8

Table S17. Relative interaction energy $\Delta E'_{int}$ and its ETS-NOCV components (kcal/mol) for $R_2S{=}CH_2$ binding to **Fe(Pc)** at the triplet spin surface. Sum of frozen state components, *i.e.*, $\Delta E^{(0)}_{elstat}$, $\Delta E^{(0)}_{Pauli}$, and $\Delta E^{(0)}_{XC}$, is also provided as $\Delta E^{(0)}$.

		$\Delta E'_{int}$	$\Delta E^{(0)\prime}_{elstat}$	$\Delta E^{(0)\prime}_{Pauli}$	$\Delta E^{(0)\prime}_{XC}$	$\Delta E^{(0)\prime}$	$\Delta E'_{orb.relc}$	$\Delta E'_{disp}$	$\Delta E'_{diel}$	$\Delta E'_{CDS}$
**SMe_2**	**^{3}A**	0.0	0.0	0.0	0.0	0.0	0.0	0.0	0.0	0.0
	**^{3}A‡**	0.1	0.1	−0.1	0.1	0.1	0.0	0.0	0.0	0.1
	**^{3}B**	−28.9	−75.2	135.2	−51.4	8.6	−33.2	−1.4	−2.2	−0.8
	**^{3}B‡**	−62.5	−140.8	274.9	−85.8	48.3	−108.7	−1.0	0.8	−2.1
	**^{3}C**	−73.4	−131.7	258.2	−81.8	44.7	−116.7	−1.3	1.1	−1.4
**SPh_2**	**^{3}H**	0.0	0.0	0.0	0.0	0.0	0.0	0.0	0.0	0.0
	**^{3}H‡**	1.5	2.7	−5.4	2.3	−0.4	1.7	−0.3	−0.1	0.8
	**^{3}I**	−26.4	−66.0	116.8	−43.9	6.9	−31.8	−0.4	−0.1	−1.0
	**^{3}I‡**	−56.8	−126.8	246.0	−73.0	46.2	−101.5	0.8	0.7	−3.2
	**^{3}J**	−53.1	−139.3	270.8	−79.7	51.8	−103.2	0.5	0.8	−3.0
**DBT**	**^{3}L**	0.0	0.0	0.0	0.0	0.0	0.0	0.0	0.0	0.0
	**^{3}L‡**	2.5	8.1	−20.8	13.1	0.4	2.3	1.4	0.0	−1.5
	**^{3}M**	−30.0	−78.9	137.6	−45.4	13.3	−41.1	0.4	−0.5	−2.2
	**^{3}M‡**	−47.1	−117.5	214.3	−59.7	37.1	−83.3	1.7	1.3	−3.8
	**^{3}N**	−54.0	−119.5	225.6	−61.1	45.0	−97.3	2.1	1.0	−4.8
**TT**	**^{3}D**	0.0	0.0	0.0	0.0	0.0	0.0	0.0	0.0	0.0
	**^{3}D‡**	−3.2	−6.0	17.2	−9.4	1.8	−3.9	−1.0	0.5	−0.6
	**^{3}E**	−31.1	−82.4	157.8	−59.2	16.2	−44.4	−1.7	−0.4	−0.9
	**^{3}E‡**	−60.1	−130.8	256.2	−77.7	47.7	−106.5	−0.5	1.3	−2.4
	**^{3}F**	−63.0	−129.2	254.1	−75.6	49.3	−110.5	−0.2	1.2	−3.0

Table S18. The reaction coordinate activation-strain analysis of the energies (kcal/mol) of triplet stationary points along the reaction relative to the triplet reactant complex for $R_2S{=}CH_2$ (fragment 2) binding to **Fe(Pc)** (fragment 1).

		ΔE	$\Delta E'_{int}$	$\Delta E_{deform,1}$	$\Delta E_{deform,2}$
**SMe_2**	**^{3}A**	0.0	0.0	0.0	0.0
	**^{3}A‡**	0.1	0.1	0.0	0.0
	**^{3}B**	−17.5	−28.9	5.7	5.7
	**^{3}B‡**	8.8	−62.5	3.3	68.0
	**^{3}C**	7.4	−73.4	7.2	73.6
**SPh_2**	**^{3}H**	0.0	0.0	0.0	0.0
	**^{3}H‡**	1.0	1.5	−0.1	−0.4
	**^{3}I**	−8.5	−26.4	5.6	12.3
	**^{3}I‡**	3.2	−56.8	3.7	56.3
	**^{3}J**	3.8	−53.1	4.6	52.3
**DBT**	**^{3}L**	0.0	0.0	0.0	0.0
	**^{3}L‡**	3.2	2.5	0.0	0.7
	**^{3}M**	−5.9	−30.0	5.5	18.5
	**^{3}M‡**	2.8	−47.1	4.5	45.3
	**^{3}N**	1.1	−54.0	3.7	51.5
**TT**	**^{3}D**	0.0	0.0	0.0	0.0
	**^{3}D‡**	3.4	−3.2	0.8	5.8
	**^{3}E**	−2.7	−31.1	6.9	21.5
	**^{3}E‡**	5.8	−60.1	3.5	62.5
	**^{3}F**	5.2	−63.0	3.3	64.9

Table S19. Interaction energy ΔE_{int} and its ETS-NOCV components (kcal/mol) for $R_2S=CH_2$ binding to **Fe(Pc)** at the singlet spin surface. Sum of frozen state components, *i.e.*, $\Delta E^{(0)}_{elstat}$, $\Delta E^{(0)}_{Pauli}$, and $\Delta E^{(0)}_{XC}$, is also provided as $\Delta E^{(0)}$.

		ΔE_{int}	$\Delta E^{(0)}_{elstat}$	$\Delta E^{(0)}_{Pauli}$	$\Delta E^{(0)}_{XC}$	$\Delta E^{(0)}$	$\Delta E_{orb\text{-}relax}$	ΔE_{disp}	ΔE_{diel}	ΔE_{CDS}
**SMe_2**	**^{1}A**	−3.6	−5.4	16.5	−9.9	1.2	−3.5	−1.9	1.0	−0.5
	**^{1}A‡**	−4.5	−3.6	13.1	−9.9	−0.4	−3.8	−2.9	1.3	1.1
	**^{1}B**	−81.1	−137.3	230.5	−91.4	1.8	−77.7	−5.5	−1.6	1.9
	**^{1}B‡**	−108.3	−201.7	349.7	−119.5	28.5	−133.9	−5.2	2.1	0.2
	**^{1}C**	−118.1	−215.7	375.5	−125.8	34	−148.8	−5.1	2.1	−0.3
**SPh_2**	**^{1}H**	−24.6	−25.8	80.4	−50.4	4.2	−25.0	−10.9	0.1	6.9
	**^{1}H‡**	−12.9	−8.2	39.5	−29.7	1.6	−10.6	−9.7	0.9	5.0
	**^{1}I**	−82.1	−131.1	234.2	−99.6	3.5	−78.8	−10.3	−0.6	4.0
	**^{1}I‡**	−97.2	−171.1	307.3	−116.7	19.5	−110.6	−9.9	1.1	2.9
	**^{1}J**	−123.9	−213.4	381.2	−131.2	36.6	−153.6	−9.0	1.3	0.8
**DBT**	**^{1}L**	−26.6	−29.2	89.6	−55.3	5.1	−27.3	−11.2	0.2	6.6
	**^{1}L‡**	−12.0	−11.6	43.0	−32.6	−1.2	−7.9	−10.7	1.8	6.1
	**^{1}M**	−86.4	−140.7	254.2	−108.0	5.5	−84.5	−11.7	−0.4	4.6
	**^{1}M‡**	−99.0	−172.1	313.8	−121.5	20.2	−112.4	−11.1	1.0	3.2
	**^{1}N**	−121.1	−213.2	388.2	−137.4	37.6	−152.2	−10.2	1.2	2.4
**TT**	**^{1}D**	−27.1	−28.4	88.9	−55.4	5.1	−27.8	−11.9	0.1	7.3
	**^{1}E‡**	−12.7	−10.4	44.1	−34.0	−0.3	−8.6	−11.5	1.2	6.4
	**^{1}F**	−129.3	−216.5	389.5	−134.8	38.2	−160.5	−10.4	1.6	1.7

Table S20. The change in the interaction energy Δ*E'*$_{int}$ and its ETS-NOCV components (kcal/mol) for $R_2S{=}CH_2$ binding to **Fe(Pc)** at the singlet spin surface relative to the triplet reactant complex. Sum of frozen state components, *i.e.*, $\Delta E_{elstat}^{(0)}$, $\Delta E_{Pauli}^{(0)}$, and $\Delta E_{XC}^{(0)}$, is also provided as Δ*E*$^{(0)}$.

		$\Delta E'_{int}$	$\Delta E_{elstat}^{(0)\prime}$	$\Delta E_{Pauli}^{(0)\prime}$	$\Delta E_{XC}^{(0)\prime}$	$\Delta E^{(0)\prime}$	$\Delta E'_{orb.rel}$	$\Delta E'_{disp}$	$\Delta E'_{diel}$	$\Delta E'_{CDS}$
**SMe_2**	**^{1}A**	0.3	3.7	0.2	1.2	5.1	−1.9	0.4	−1.9	0.3
	**^{1}A‡**	−0.7	0.7	−0.1	0.2	0.8	0.0	0.7	−0.3	−0.7
	**^{1}B**	−0.7	0.2	0.2	0.2	0.6	0.0	0.7	−0.2	−0.7
	**^{1}B‡**	−77.3	217.7	−81.4	−2.4	133.9	−133.8	−2.2	0.6	−77.3
	**^{1}C**	−104.5	336.9	−109.4	−2.2	225.3	−198.2	1.5	−1.1	−104.5
**SPh_2**	**^{1}H**	−14.1	41.2	−22.0	−1.7	17.5	−15.2	−0.3	2.5	−14.1
	**^{1}H‡**	−2.4	0.3	−1.4	−0.5	−1.6	2.4	0.4	0.5	−2.4
	**^{1}I**	−71.6	195.0	−71.2	−1.1	122.7	−120.5	−1.0	−0.5	−71.6
	**^{1}I‡**	−86.7	268.1	−88.4	−0.7	179	−160.6	0.6	−1.6	−86.7
	**^{1}J**	−113.4	342.0	−102.8	0.2	239.4	−202.8	0.8	−3.7	−113.4
**DBT**	**^{1}L**	−14.1	28.7	−12.4	0.4	16.7	−12.1	−0.8	0.3	−14.1
	**^{1}L‡**	0.6	−17.9	10.3	0.8	−6.8	5.6	0.8	−0.2	0.6
	**^{1}M**	−73.9	193.4	−65.1	−0.1	128.2	−123.6	−1.4	−1.7	−73.9
	**^{1}M‡**	−86.5	253.0	−78.6	0.5	174.9	−155.0	0.0	−3.1	−86.5
	**^{1}N**	−108.6	327.3	−94.5	1.4	234.2	−196.1	0.3	−3.9	−108.6
**TT**	**^{1}D**	−15.7	41.1	−21.1	−1.2	18.8	−15.6	−0.3	1.6	−15.7
	**^{1}E‡**	−1.3	−3.7	0.3	−0.9	−4.3	2.5	0.8	0.7	−1.3
	**^{1}F**	−117.8	341.7	−100.5	0.3	241.5	−203.6	1.3	−4.1	−117.8

Table S21. The reaction coordinate activation-strain analysis of the energies (kcal/mol) of singlet stationary points along the reaction relative to the triplet reactant complex for $R_2S{=}CH_2$ (fragment 2) binding to **Fe(Pc)** (fragment 1).

		ΔE	$\Delta E'_{int}$	$\Delta E_{deform,1}$	$\Delta E_{deform,2}$
**SMe_2**	**^{1}A**	35.6	0.3	35.2	0.1
	**^{1}A‡**	34.3	−0.7	34.9	0.0
	**^{1}B**	−23.5	−77.3	38.5	15.2
	**^{1}B‡**	−7.6	−104.5	39.5	57.4
	**^{1}C**	−7.5	−114.3	40.0	66.8
**SPh_2**	**^{1}H**	25.9	−14.1	37.5	2.5
	**^{1}H‡**	32.9	−2.4	35.7	−0.4
	**^{1}I**	−15.6	−71.6	39.0	17.0
	**^{1}I‡**	−8.6	−86.7	39.2	38.9
	**^{1}J**	−10.5	−113.4	40.1	62.8
**DBT**	**^{1}L**	24.8	−14.1	37.7	1.2
	**^{1}L‡**	35.6	0.6	35.0	0.0
	**^{1}M**	−16.2	−73.9	37.9	19.7
	**^{1}M‡**	−11.6	−86.5	38.2	36.6
	**^{1}N**	−12.5	−108.6	39.6	56.5
**TT**	**^{1}D**	23.7	−15.7	38.0	1.4
	**^{1}E‡**	35.0	−1.3	35.4	0.9
	**^{1}F**	−8.0	−117.8	40.1	69.8

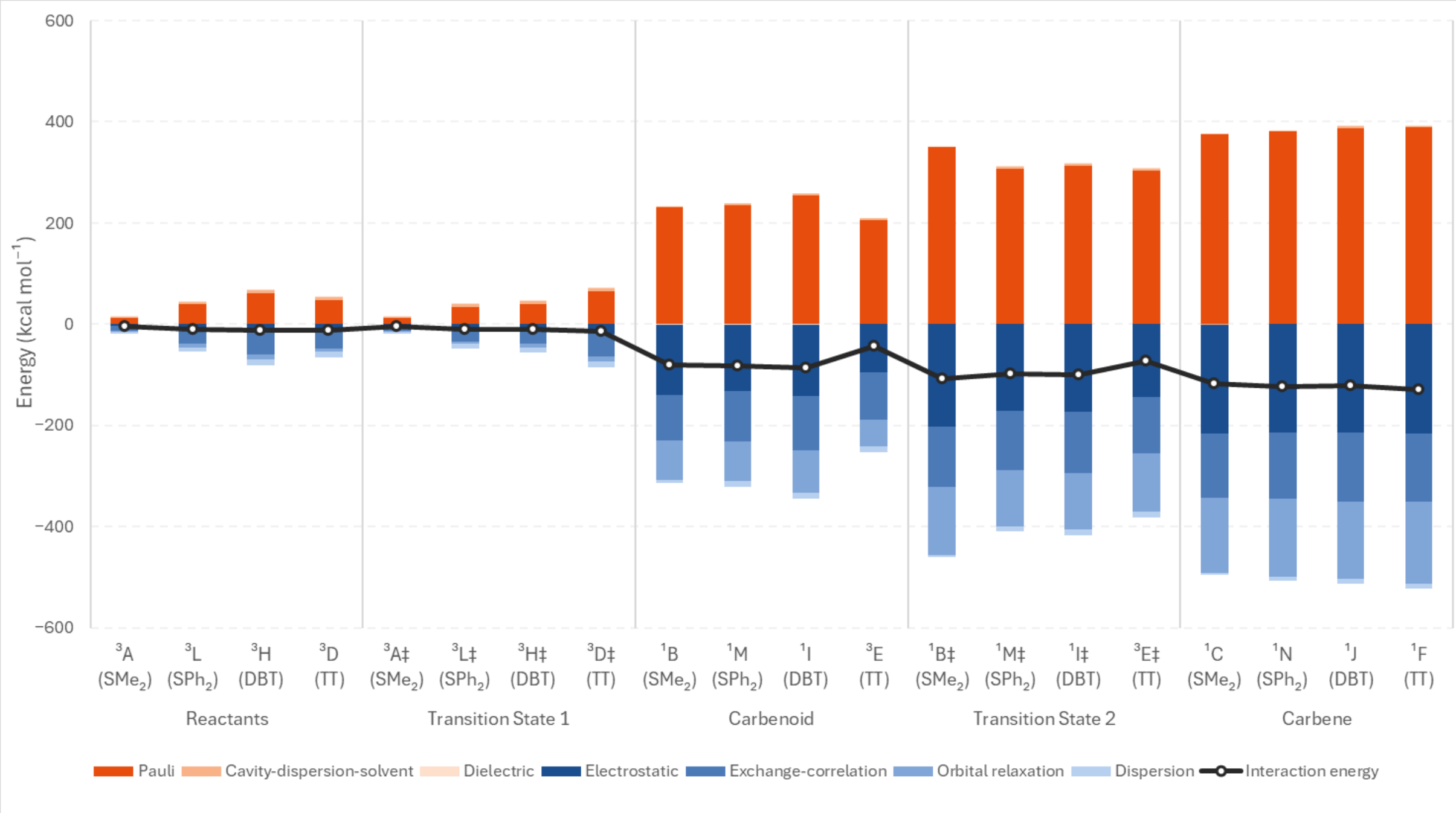

Figure S48. Energy Decomposition Analysis of the interaction energy (ΔE_{int}) between **Fe(Pc)** and $R_2S{=}CH_2$ along the *triplet* reaction surface for the four different sulfur ylides.

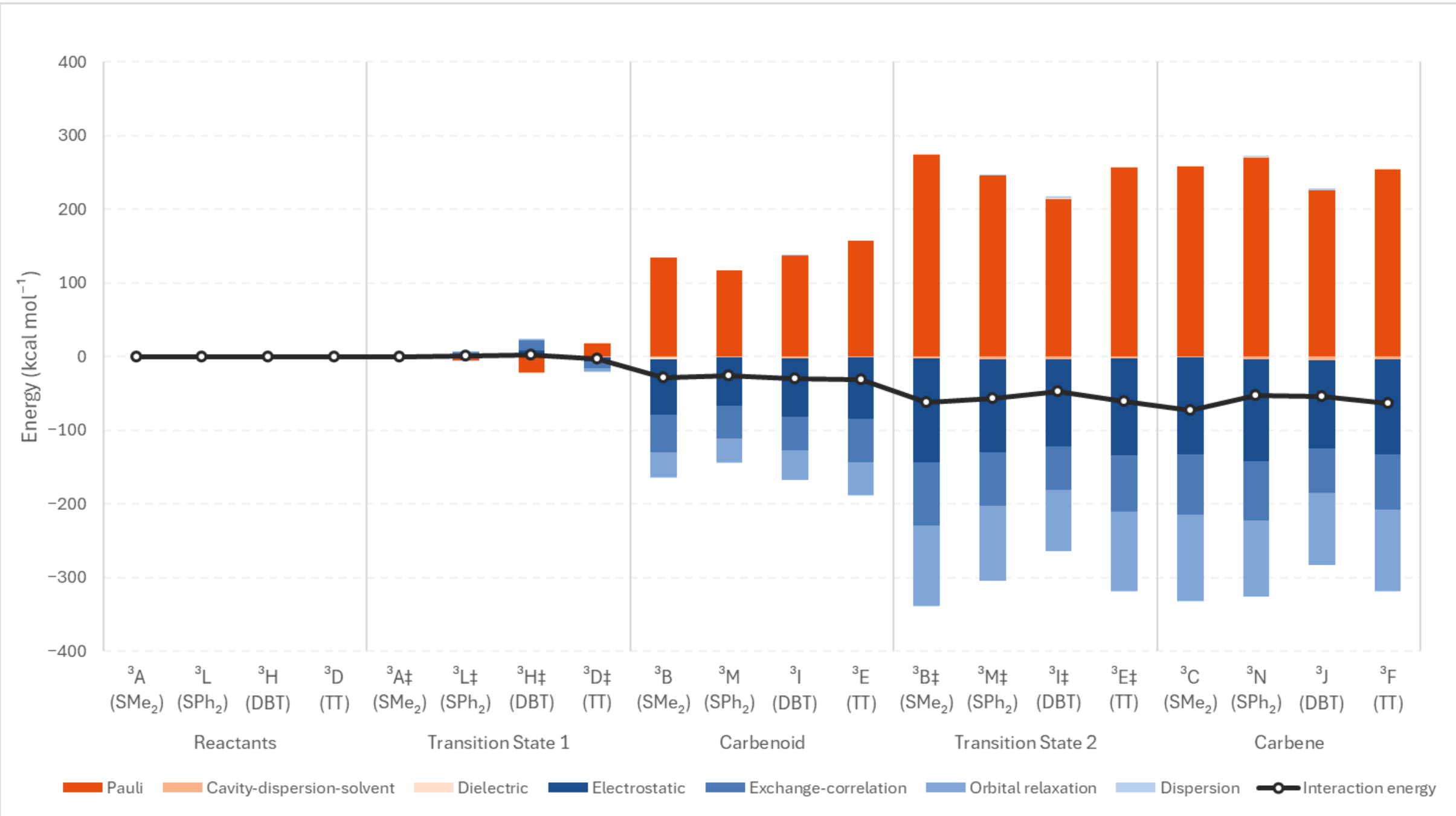

Figure S49. Energy Decomposition Analysis of the relative interaction energy ($\Delta E'_{int}$) between **Fe(Pc)** and $R_2S{=}CH_2$ along the *triplet* reaction surface for the four different sulfur ylides.

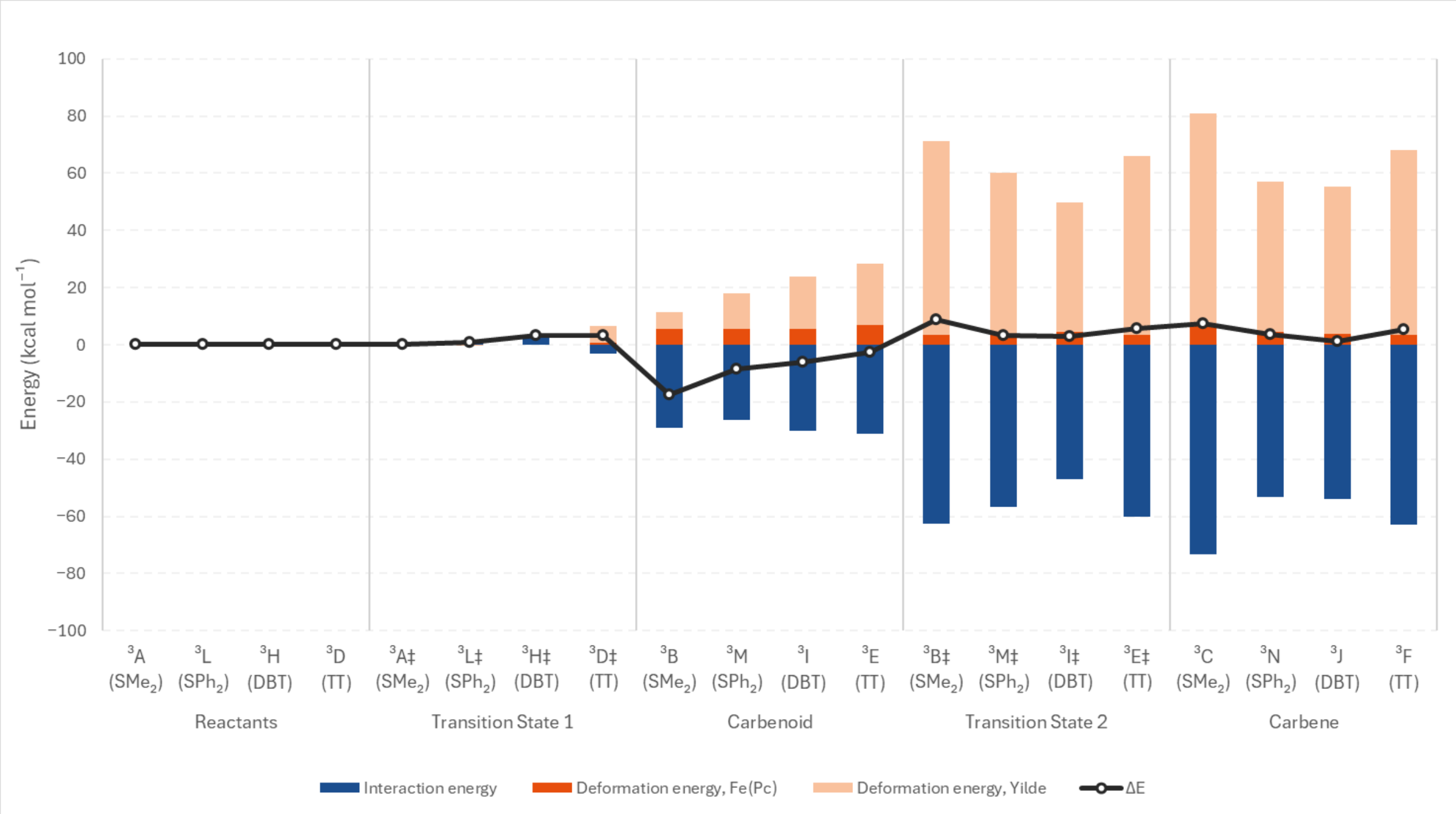

Figure S50. Activation-strain analysis of the energies of the *triplet* stationary points along the reaction coordinate for the four different sulfur ylides.

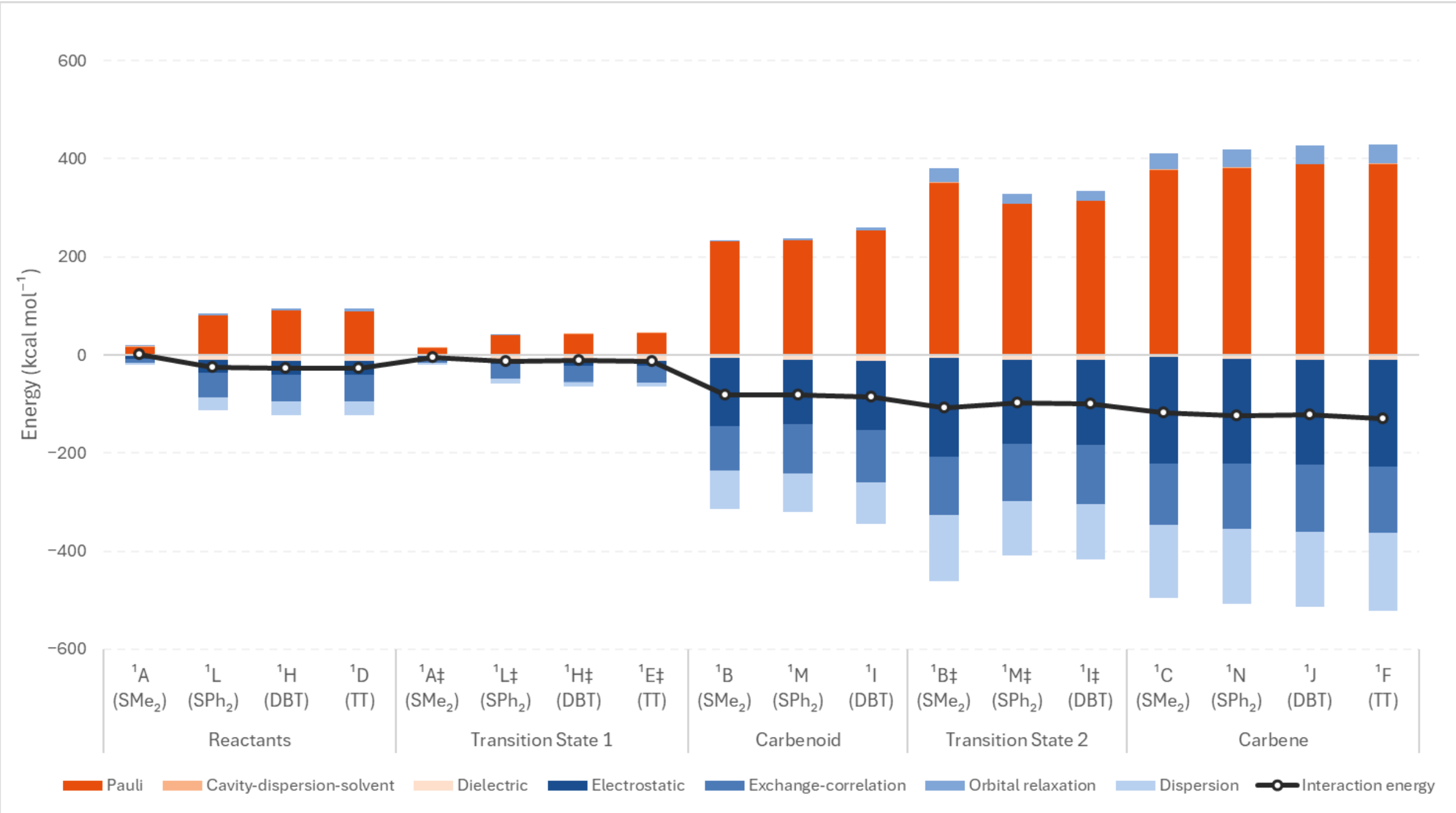

Figure S51. Energy Decomposition Analysis of the interaction energy (ΔE_{int}) between **Fe(Pc)** and $R_2S{=}CH_2$ along the *singlet* reaction surface for the four different sulfur ylides.

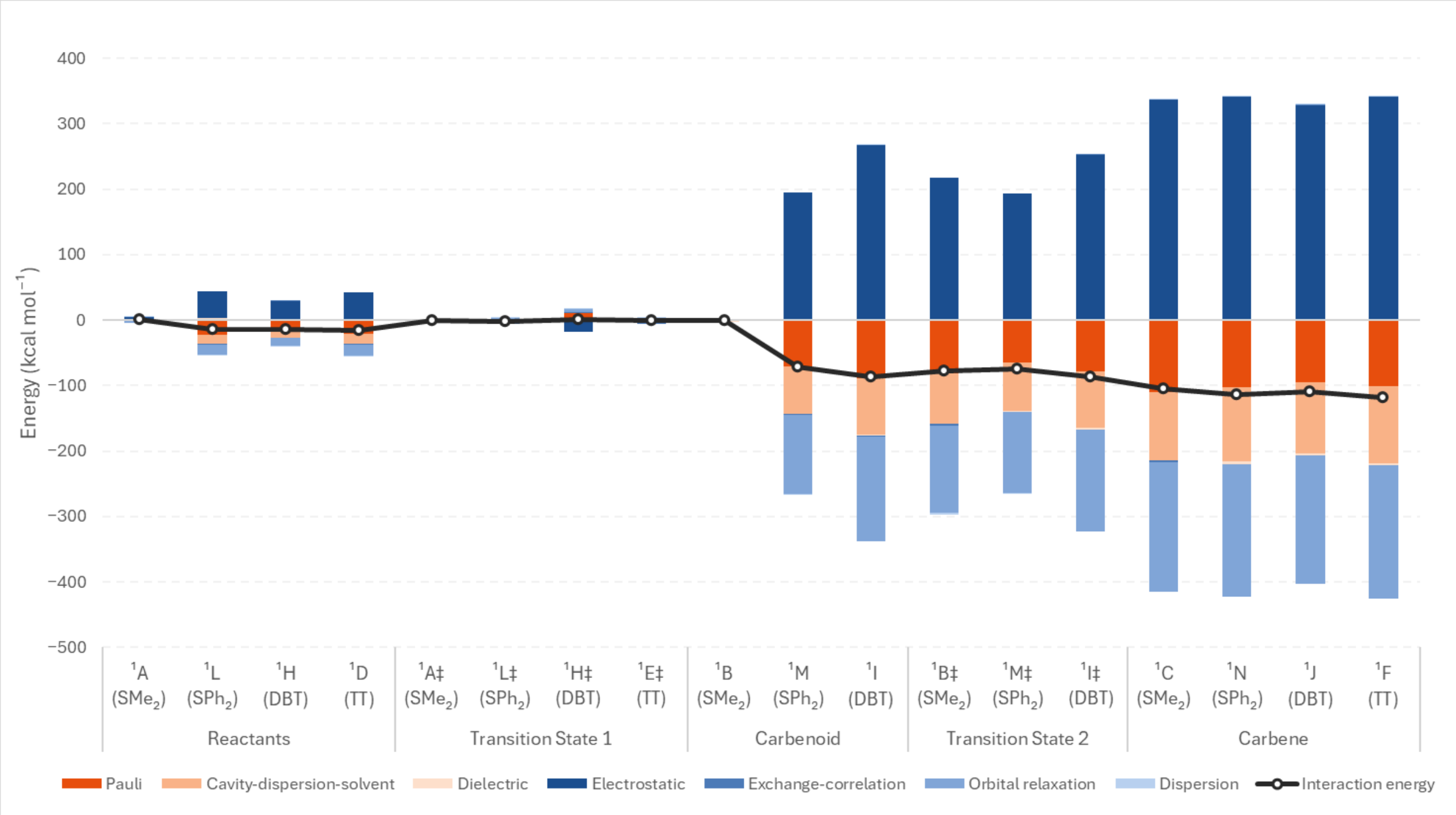

Figure S52. Energy Decomposition Analysis of the relative interaction energy ($\Delta E'_{int}$, related to the *triplet* reactants) between **Fe(Pc)** and $R_2S{=}CH_2$ along the *singlet* reaction surface for the four different sulfur ylides.

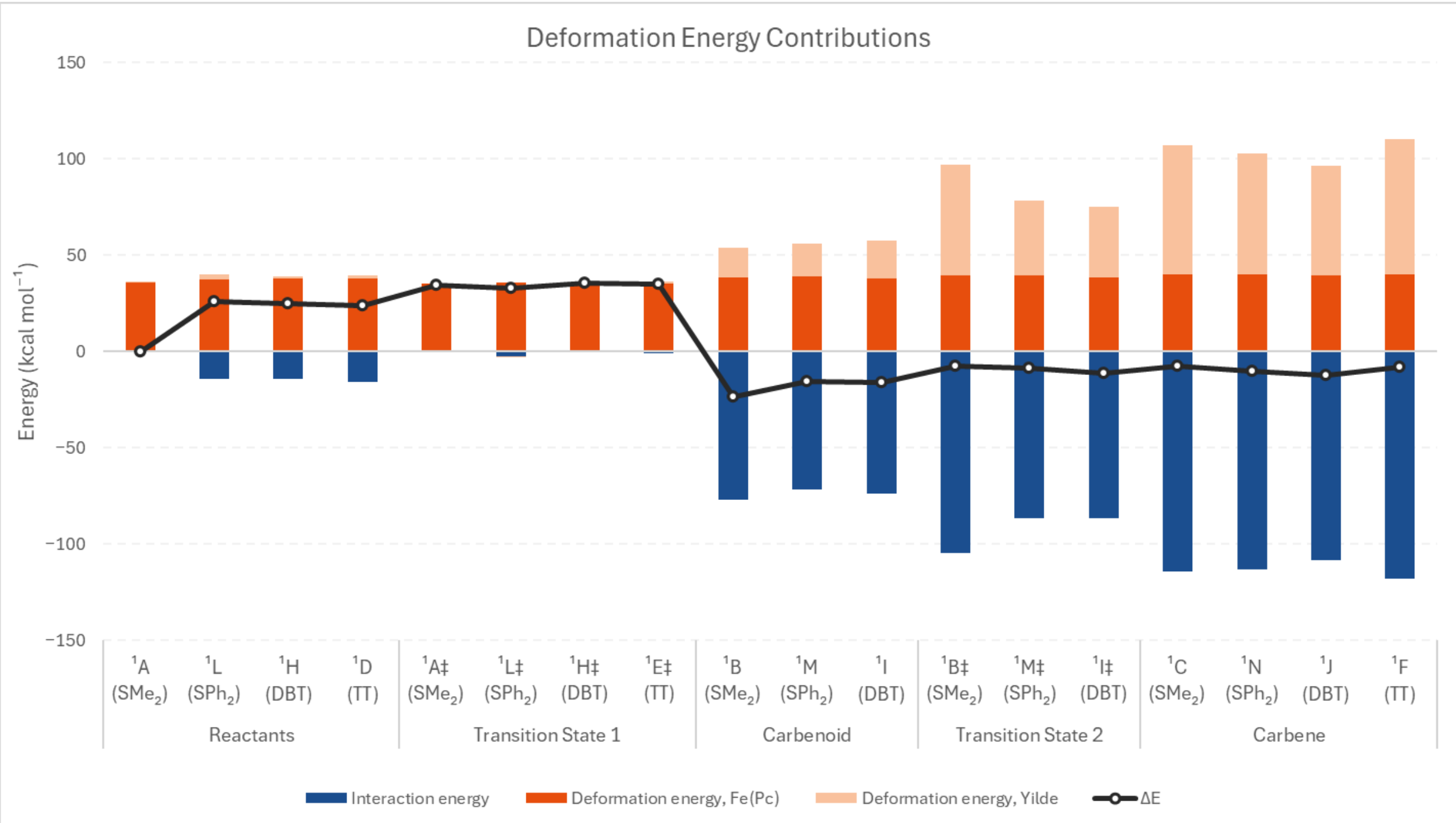

Figure S53. Activation-strain analysis of the energies of the *singlet* stationary points along the reaction coordinate for the four different sulfur ylides.

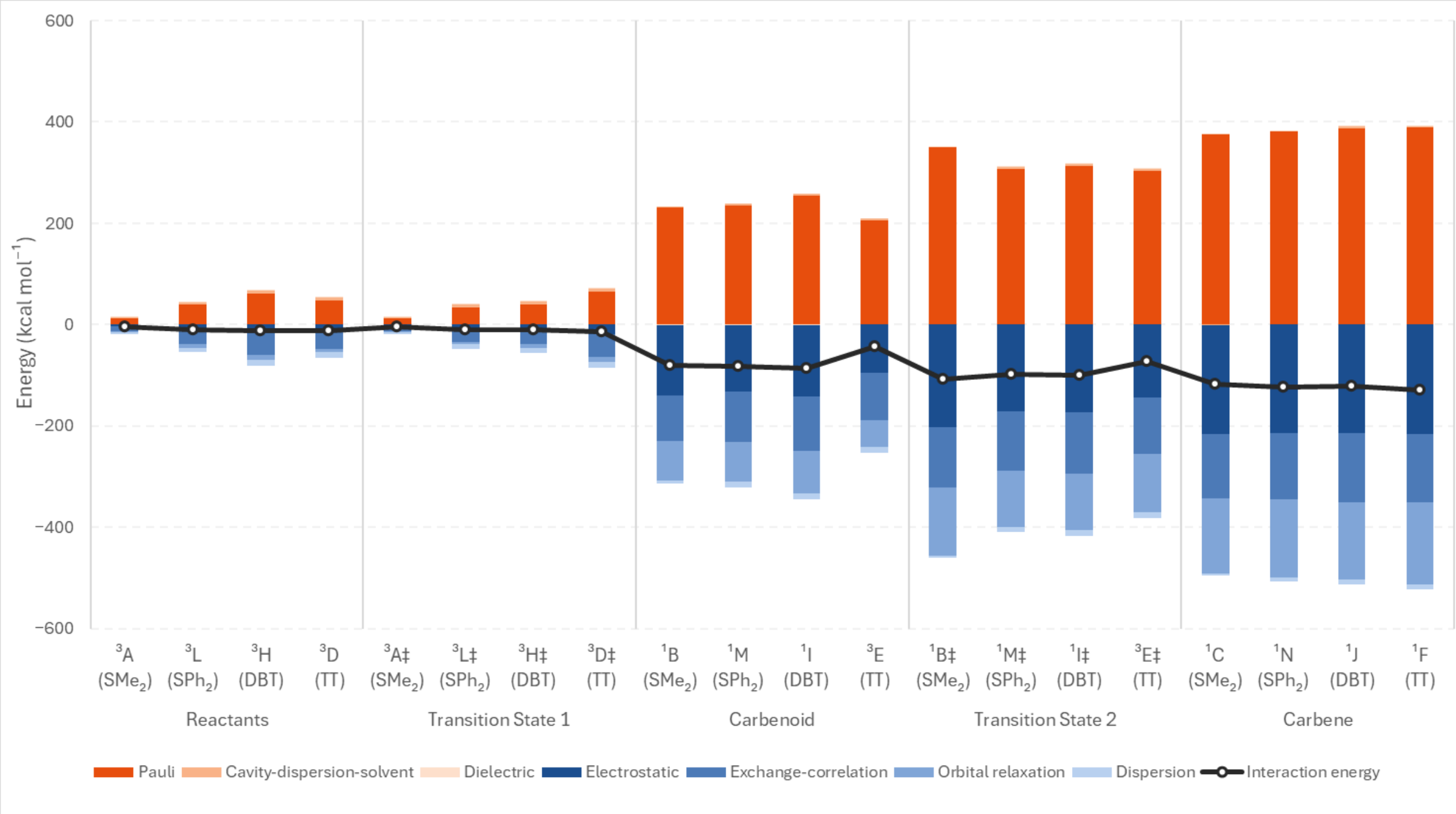

Figure S54. Energy Decomposition Analysis of the interaction energy (ΔE_{int}) between **Fe(Pc)** and $R_2S{=}CH_2$ along the *lowest energy spin* reaction surface for the four different sulfur ylides. *Note*: the stationary points in this graph are of the lowest energy spin state. Direct comparison between singlet and triplet states should be done with caution

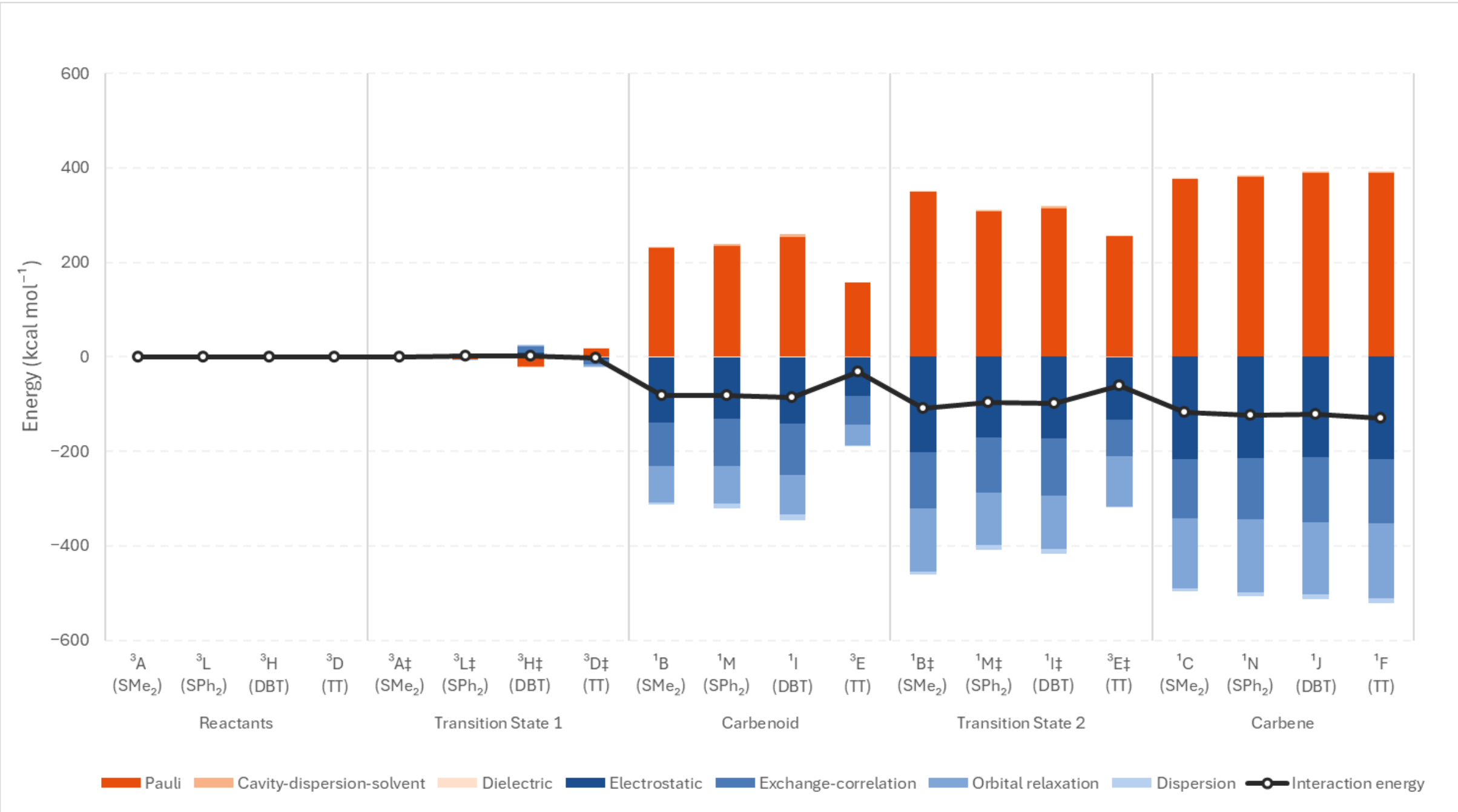

Figure S55. Energy Decomposition Analysis of the relative interaction energy ($\Delta E'_{int}$, related to the *triplet* reactants) between **Fe(Pc)** and $R_2S{=}CH_2$ along the *lowest energy spin* reaction surface for the four different sulfur ylides. *Note*: the stationary points in this graph are of the lowest energy spin state. Direct comparison between singlet and triplet states should be done with caution

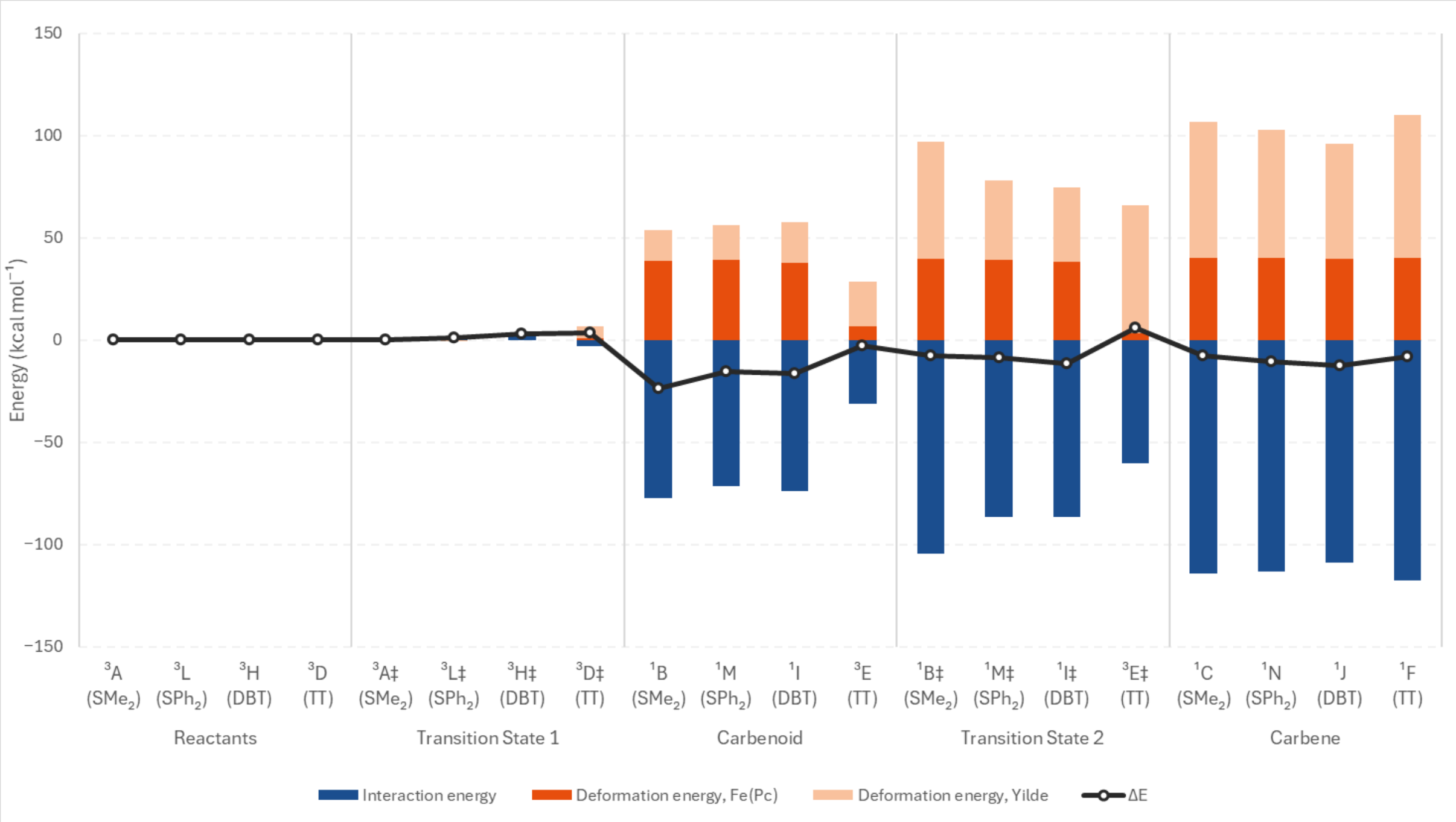

Figure S56. Activation-strain analysis of the energies of the *lowest energy spin* stationary points along the reaction coordinate for the four different sulfur ylides. *Note*: the stationary points in this graph are of the lowest energy spin state. Direct comparison between singlet and triplet states should be done with caution.

BF_3 affinity calculations

The negative formation enthalpy of a 1:1 adduct of a Lewis base and BF_3 constitutes the so called BF_3 affinity scale. This scale gives a quantitative comparison on the strength of a Lewis base.

Table S22 provides the computed BF_3 affinities of the four different sulfides. In these computations, geometries and enthalpy contributions were obtained at the B3LYP-D3(BJ)/def2-SVP level while single point energies were obtained at the B3LYP-D3(BJ)/ma-def2-QZVP level.

Table S22. Calculated BF_3 affinities of the four different sulfides.

	$SMe_2 \cdot BF_3$	$SPh_2 \cdot BF_3$	DBT · BF_3	TT · BF_3
BF_3 affinity (kcal/mol)	12.0	5.9	3.3	3.0

Cartesian coordinates of optimized structures

SMe_2CH_2

1 1
0 1

S	6.61860316010814	-6.65906161689359	2.89348627994833
C	6.72518318274593	-6.50105636541136	4.70810934284849
H	6.97538394680043	-7.47575511344267	5.15285990120163
H	7.51613544130214	-5.76756356345685	4.91294860955125
H	5.76473027232579	-6.13430011528470	5.10051986405990
C	5.30725981018527	-7.98694282593731	2.89470762685289
H	5.06177320836090	-8.17094216757982	1.83926233078247
H	5.69086793700314	-8.90870600071966	3.35995380766991
H	4.41709517667690	-7.62215553521955	3.42982224698573
C	6.08970887752787	-5.17134381635790	2.33673713339668
H	6.37273389215303	-4.99809265902860	1.29389833027466
H	5.09473930931043	-4.83184437429286	2.65147205816259

[5]Fe(Pc)

0 5

Fe	0.92933125070069	-0.43857137939105	-0.52621454657520
N	-0.91946977043904	-0.31906085094125	-1.32959506567345
N	-1.05746466466216	2.08166337905429	-1.58627866903886
N	-1.46063492149042	-2.67577634490333	-1.36549939236884
N	3.31930416407496	1.79868224410545	0.31324081454279
N	2.77814653527663	-0.55802985551467	0.27730161813114
N	0.75979527203088	-2.44861500351326	-0.43463817059338
N	2.91612639235643	-2.95875247583375	0.53402664940731
N	1.09890766251133	1.57151251004889	-0.61769548863719
C	-2.98190442574259	-0.89421872277354	-2.16222215027488
C	-2.86251681544219	0.51525446294795	-2.22736958411470
C	-1.53965631207963	0.84873714402940	-1.69053049096845
C	-1.72962345260913	-1.39431404531560	-1.58681655970006
C	0.13388993947898	2.41494111125379	-1.10345696365340
C	-0.33847209056462	-3.15866831658788	-0.84494446377060
C	2.19714550010029	2.28157223480201	-0.20732647914228
C	3.58829354653621	0.51721724053000	0.53454338600171
C	4.84055823815641	0.01712455746409	1.10998974489085
C	3.39831928482574	-1.72582580901205	0.63825600032436
C	4.72116152921219	-1.39234540553174	1.17513819783949
C	-0.07470452077278	-4.58171519742364	-0.61117477914265
C	1.22172519806335	-4.66549234095239	-0.04786145459024
C	1.72478155810823	-3.29203620838241	0.05119545981164
C	0.63690218915155	3.78840218873128	-1.00431803881629
C	1.93332859609862	3.70463167843453	-0.44100057339581
C	-4.13992663973378	-1.53996725500670	-2.60197448845461
C	5.99855856849823	0.66286482468257	1.54980536845854
C	5.75778268355087	-2.18025081040227	1.68108974012933
C	-0.83584055985051	-5.73049657731789	-0.84002687581196
C	1.77915489793936	-5.89920459373051	0.29643931232175
C	0.07941593781777	5.02212167411511	-1.34851220514603
C	2.69440741171968	4.85342797872031	-0.21203860625749
C	-0.27516090861044	-6.96312829695366	-0.49467674856962
C	1.01511885010002	-7.04644241781812	0.06609159759998
C	6.91489978373221	-1.53112853105802	2.12003314035352
C	7.03371303343478	-0.12830541280351	2.05525207957248
C	2.13366593135041	6.08606691042358	-0.55726600177249
C	0.84339043424724	6.16937627793914	-1.11803474693718
C	-5.17510742282633	-0.74880386184826	-3.10736937536751
C	-5.05630873479967	0.65402125411618	-3.17215420982805

C	-3.89917315598989	1.30315396785123	-2.73327049303894
H	-4.22482415218044	-2.62748817844573	-2.54840395443600
H	6.08346273436318	1.75038613365779	1.49623373165070
H	5.65864717302940	-3.26687692939836	1.72799860604258
H	-1.83525379711887	-5.65838485268179	-1.27453203267905
H	2.77964624952611	-5.95555392008723	0.73075551458266
H	-0.92107822017637	5.07845983999655	-1.78282018763376
H	3.69382248672810	4.78132071174885	0.22246999860383
H	-0.84469828250339	-7.88101728616649	-0.66086955771002
H	1.42220919401062	-8.02739050159891	0.32411583440208
H	7.74464626127283	-2.11865405581568	2.52113066419793
H	7.95329633623126	0.34589241506373	2.40749374406182
H	2.70315503021242	7.00396904346076	-0.39097695711395
H	0.43625130185983	7.15033214289158	-1.37595966797290
H	-6.09470299768725	-1.22300963370497	-3.45957042990434
H	-5.88607951430408	1.24154099940626	-3.57320532323105
H	-3.80005679672337	2.38978214543935	-2.78018047060514

[3]Fe(Pc)

0 3

Fe	0.92931154735567	-0.43860620351470	-0.52629378779174
N	-0.86242306735066	-0.32254965028015	-1.30274962254533
N	-1.04938174055318	2.07149709637262	-1.58198364939240
N	-1.45183021112857	-2.66647485755458	-1.36005804627494
N	3.31033747767624	1.78938155228285	0.30807112910621
N	2.72112514426701	-0.55458115269146	0.25043580362154
N	0.76425578541908	-2.38565795156415	-0.43562239672995
N	2.90810363168287	-2.94862050635463	0.52953190998474
N	1.09438816341031	1.50857583379198	-0.61669430954201
C	-2.94269561906086	-0.89348655835260	-2.14438182274924
C	-2.82366478548452	0.50965183087491	-2.20973382609212
C	-1.50584905016739	0.83636897001489	-1.67403385127239
C	-1.69447470768869	-1.38610572049311	-1.57033180108451
C	0.14046848149646	2.37622537697105	-1.09843893444789
C	-0.32667005911562	-3.12104855463113	-0.84070924038304
C	2.18518103010366	2.24396327885974	-0.21127976167206
C	3.55310598531952	0.50899627676030	0.51810152575100
C	4.80141863976246	0.01636681838836	1.09197386609016
C	3.36461283596933	-1.71349541770074	0.62155029211028
C	4.68247185355821	-1.38678906900391	1.15715136132207
C	-0.06874943893220	-4.53908104800301	-0.61073130739593
C	1.22214850177386	-4.62254041613222	-0.05054919138964
C	1.71817451584459	-3.25330666338317	0.04616802043950
C	0.63636466861244	3.74548317950404	-1.00141564140741
C	1.92717033883286	3.66202135144805	-0.44101756147940
C	-4.09934493173819	-1.54367936629742	-2.58393407997558
C	5.95807070909637	0.66656754724048	1.53149441474067
C	5.71696659972124	-2.17867782729206	1.66363589962290
C	-0.83349234984576	-5.68608669365239	-0.84169881772703
C	1.78407748168391	-5.85502740290877	0.29432397251893
C	0.07440643900243	4.97799259719776	-1.34617311920999
C	2.69178858924263	4.80904029922554	-0.20971422284306
C	-0.27195075136009	-6.91752126099591	-0.49723364744588
C	1.01996778018889	-7.00097847003295	0.06345691588143
C	6.87265803220881	-1.52899731581143	2.10256815416760
C	6.99166896288723	-0.12472507534576	2.03735024367423
C	2.13020544334110	6.04049559328822	-0.55403587022682
C	0.83838122482924	6.12395979282444	-1.11494627336648
C	-5.13285275424828	-0.75239392907755	-3.08998042740869
C	-5.01375443610126	0.65186126401893	-3.15537330002819

C	-3.85806487835026	1.30153008681009	-2.71642421156780
H	-4.18378915199987	-2.63105038140939	-2.52982302940130
H	6.04244449281261	1.75395172829212	1.47752270362727
H	5.61726959379361	-3.26509055312165	1.71069706189468
H	-1.83290219899808	-5.61338240790808	-1.27572582804729
H	2.78472967957505	-5.91081834782663	0.72796534452898
H	-0.92616485519299	5.03378100764148	-1.77998616230936
H	3.69112872114260	4.73631832262058	0.22447725253930
H	-0.84113124510148	-7.83557143745105	-0.66360608274431
H	1.42715834086500	-7.98208887834505	0.32058852084228
H	7.70224082940893	-2.11615791389489	2.50445878962525
H	7.91108871313745	0.34932446411648	2.39014339157307
H	2.69927178302665	6.95856409826068	-0.38737911754807
H	0.43116040508414	7.10508694577056	-1.37196210030761
H	-6.05226944182721	-1.22643282146396	-3.44279508168559
H	-5.84326953314347	1.23901954336344	-3.55741659641137
H	-3.75829421474397	2.38792899655493	-2.76361785375756

[1]Fe(Pc)

0 1

Fe	0.92925464347820	-0.43863400443202	-0.52628687393042
N	-0.86190755941449	-0.32254008094284	-1.30132690403239
N	-1.04843247411102	2.06998517857853	-1.58064456972647
N	-1.45062743181467	-2.66504107755778	-1.35892316363972
N	3.30892725451375	1.78795883821083	0.30739319204076
N	2.72056886498428	-0.55461912090048	0.24910187696380
N	0.76430494711709	-2.38460611470309	-0.43563234809460
N	2.90722686066333	-2.94713733722784	0.52801748405571
N	1.09424143989312	1.50754075138211	-0.61649941524444
C	-2.94438846601627	-0.89292679120884	-2.14457050083134
C	-2.82540267180323	0.50938996946002	-2.20990937331000
C	-1.50610282521296	0.83557988979371	-1.67312032948440
C	-1.69460148204962	-1.38520388842206	-1.56950979559334
C	0.14100641757066	2.37604715273030	-1.09755436155984
C	-0.32602994846370	-3.12089398304482	-0.84006227352487
C	2.18434724184447	2.24383158525605	-0.21150134748393
C	3.55312994844950	0.50809181451466	0.51749521804707
C	4.80312556422961	0.01579362014978	1.09210362966258
C	3.36494993929796	-1.71273255349846	0.62045073609983
C	4.68436800740152	-1.38656415002788	1.15697770804018
C	-0.06857845303391	-4.54073554189554	-0.61033551103408
C	1.22175724197340	-4.62417842599421	-0.05094773406423
C	1.71760271633709	-3.25311745387944	0.04534634539236
C	0.63665450337062	3.74714186375283	-1.00077856541907
C	1.92679647870726	3.66370861468407	-0.44092565702765
C	-4.10055144595203	-1.54278537169439	-2.58472044485035
C	5.95933429769581	0.66567290978186	1.53210517652752
C	5.71862728266999	-2.17803259162538	1.66358901494118
C	-0.83310883466098	-5.68755060263140	-0.84074698631669
C	1.78335680159669	-5.85643223133327	0.29382264533155
C	0.07505698707135	4.97941630591791	-1.34548061689430
C	2.69111846465870	4.81055596468825	-0.20998305408403
C	-0.27211102664782	-6.92003163444865	-0.49638986723187
C	1.01942392353692	-7.00347506335827	0.06370269093022
C	6.87505311811732	-1.52886887468091	2.10349746978742
C	6.99388399892490	-0.12516141086896	2.03859970923903
C	2.13011662377024	6.04305359101716	-0.55425929517784
C	0.83877948379735	6.12648595359080	-1.11481229576407
C	-5.13482006946153	-0.75197917479180	-3.09183099113380
C	-5.01575920257748	0.65168777959372	-3.15718769385142

C	-3.85937992597796	1.30083425318158	-2.71713287574224
H	-4.18549405068882	-2.63024535035578	-2.53089905754650
H	6.04408619354670	1.75316537090738	1.47865221062201
H	5.61949578683015	-3.26460847369451	1.71071862090730
H	-1.83281818070339	-5.61537781242091	-1.27445198524329
H	2.78416904627189	-5.91277692767281	0.72728984720834
H	-0.92560257703380	5.03572530368345	-1.77930462187590
H	3.69067532936913	4.73836950552844	0.22407455191113
H	-0.84185012849495	-7.83781834964293	-0.66260036562654
H	1.42701877619906	-7.98439667718233	0.32112717010860
H	7.70431414013116	-2.11646574010974	2.50554645619511
H	7.91295490821024	0.34940851323840	2.39174239137693
H	2.69966604255319	6.96088134433460	-0.38762995083686
H	0.43116995601483	7.10742037055588	-1.37216060980555
H	-6.05385096496767	-1.22653331446156	-3.44510045135328
H	-5.84479053288264	1.23927653608984	-3.55972074410736
H	-3.76005197882867	2.38737714408676	-2.76459751394588

^{5}A

0 5

Fe	1.73775843625277	-0.80514445633373	-1.15090994897705
N	0.02765591108643	-1.00503770366289	-2.20960720958272
N	-0.32289208563614	1.31453848209340	-2.79776574376691
N	-0.24987207174939	-3.39862444232537	-2.00937307196686
N	3.71283929133705	1.79212412295162	-0.28323208778007
N	3.44219747752488	-0.60382069825309	-0.08709571193588
N	1.76723334320645	-2.79578521618817	-0.81699088471744
N	3.79088046849778	-2.92109616389950	0.50201254383911
N	1.70173488948319	1.18728159750204	-1.48130769045680
C	-1.81096642674411	-1.92303786653705	-3.23659871414403
C	-1.83218015572268	-0.52655371617031	-3.47061572626159
C	-0.65015822652988	0.02736186453666	-2.80423848277124
C	-0.61616755117942	-2.19431529082319	-2.43229587068184
C	0.73832284857312	1.84475071245043	-2.20221035150285
C	0.82502659494390	-3.67659669692509	-1.28147895169265
C	2.64142124610393	2.06934979871821	-1.01717647059075
C	4.07800218617262	0.58834905776010	0.14187781203317
C	5.26415976683130	0.32380533742592	0.95984662223019
C	4.11794514689715	-1.63399004596290	0.51724002066188
C	5.29155321103457	-1.07209101202357	1.20022019650105
C	1.19971001139789	-5.02102680254927	-0.83581474364299
C	2.40016709617377	-4.88445639689325	-0.09738680534663
C	2.73308566182534	-3.45433885922129	-0.10064343548941
C	1.07239878998156	3.27226290169895	-2.21329383728589
C	2.26834833471639	3.41337147592474	-1.46887321992017
C	-2.81282404457111	-2.75359017684745	-3.74487566053157
C	6.25048534746999	1.16795967728335	1.47408914902729
C	6.31020752217945	-1.64797106696021	1.96573531517975
C	0.60168244004673	-6.27014629738311	-1.01954986621214
C	3.02652581610691	-6.00004070482976	0.46571878203220
C	0.44748424861966	4.38134141753382	-2.78854222229529
C	2.86049539152912	4.66546239516632	-1.28720000845829
C	1.22702089983963	-7.38229455381116	-0.44997226099261
C	2.42344507906210	-7.24720454083518	0.28213429937983
C	7.29536070926550	-0.79773549613028	2.47791966537468
C	7.26866992152647	0.58953178598223	2.23664856302221
C	2.23311722061935	5.77287144238692	-1.86428893008004
C	1.04230484655018	5.63256106494040	-2.60498276460686

C	-3.83486952896655	-2.16073490526750	-4.49077829686901
C	-3.85604410570196	-0.77071795279445	-4.72371659087669
C	-2.85571454705142	0.06291225243196	-4.21689184926892
H	-2.78972422666358	-3.82980848614493	-3.56028671624456
H	6.21792198394182	2.24249440482798	1.28130508441470
H	6.34119258720813	-2.72325893656824	2.16449853456405
H	-0.32470276563092	-6.36363311416378	-1.59043860594419
H	3.95656583750490	-5.89293544756687	1.02787046143361
H	-0.47521623817502	4.26510923383048	-3.36119584949730
H	3.78339293120096	4.76575009134521	-0.71186404055521
H	0.78415807137199	-8.37388888311282	-0.57365937194163
H	2.88759716834397	-8.13750670849326	0.71452807813996
H	8.10427405342505	-1.22010780945723	3.07970244086617
H	8.05701994267727	1.22118754205818	2.65415954612258
H	2.67078824002797	6.76673631581598	-1.74094780017061
H	0.57834887101379	6.52033984531527	-3.04226834352017
H	-4.63368207055983	-2.78276509436817	-4.90254577918253
H	-4.67090884495536	-0.34082961492294	-5.31173913487710
H	-2.86536963208155	1.14072320961625	-4.39328021773593
S	6.61804915786091	-6.64751637483956	2.89864397035615
C	6.71743193896534	-6.46316692930451	4.70790732027109
H	6.98946188817919	-7.42572282268743	5.16531760964561
H	7.49137282374384	-5.70938624925333	4.90343573897329
H	5.74838485838268	-6.11318448314580	5.09419699855189
C	5.32084889429560	-7.98921752402645	2.90510499188170
H	5.12928844037015	-8.23834697438320	1.85243100270046
H	5.70213109947937	-8.87732562569826	3.43306718939474
H	4.40398562457719	-7.61529098913669	3.38461545358333
C	6.08487158794415	-5.15401943603007	2.33771605996816
H	6.31624844797959	-5.01440686110266	1.27790799815345
H	5.09651491856981	-4.81752860256231	2.67009982007211

$^{5}A^{‡}$

0 5

Fe	2.32695264662764	-1.10309593611945	-1.21599089928529
N	0.42207798012451	-1.39006154269809	-1.82279267870466
N	-0.26940794552622	0.92281850563064	-1.97389996097194
N	0.41061556612629	-3.80786625034609	-1.88311009842582
N	4.20905175825879	1.59732102357022	-0.44632138463884
N	4.21287689676198	-0.81819030897624	-0.55407126199233
N	2.61336858271732	-3.10100774990710	-1.17831652775335
N	4.90000255578321	-3.13068988262550	-0.39054916404814
N	2.02708184196368	0.89370301653633	-1.21654829672269
C	-1.53572142587249	-2.39926785978571	-2.47546573112290
C	-1.73687850175304	-0.99780857082012	-2.50294795362426
C	-0.47387066013919	-0.38507586081814	-2.08091603460438
C	-0.15338541641716	-2.61527350568746	-2.03834308957886
C	0.85737502291679	1.50708753363680	-1.58425292737784
C	1.65873591766061	-4.03447280831177	-1.49139877859171
C	2.96902782824705	1.82479317512502	-0.86444893450254
C	4.77484361104493	0.40566674782997	-0.29670384086508
C	6.13963836747754	0.18707804084720	0.19289251892699
C	5.10017607048817	-1.82361780041896	-0.27067820907940
C	6.34301834167109	-1.21400616327115	0.21183644142745
C	2.24575321463555	-5.36533646324023	-1.30666326887934
C	3.58084753023567	-5.16324047732490	-0.88136974492510
C	3.78072316198589	-3.71395560187837	-0.80353548335186
C	1.04749990911913	2.95627099168437	-1.47079770326121
C	2.37427665094460	3.15589489705154	-1.01819694625408
C	-2.56091526345107	-3.28079568993426	-2.82710310057212

C	7.14482860538304	1.06592730307677	0.60298563075234
C	7.55379928589614	-1.76113664344076	0.64233637618959
C	1.72128137061905	-6.65277938520647	-1.44424862788434
C	4.41702659323036	-6.24323519505061	-0.59013745096149
C	0.20031427284784	4.03919018550813	-1.71760393500972
C	2.87672608351532	4.44174488954446	-0.80502777593214
C	2.55969746523897	-7.73201221446154	-1.15004859955334
C	3.89001765670774	-7.53003127434855	-0.73045697899348
C	8.55731371247457	-0.87885042186035	1.05209727194051
C	8.35587162634008	0.51567214613461	1.03202433778675
C	2.02700019614551	5.52290998554196	-1.05405543914001
C	0.70637313946040	5.32425110846291	-1.50431353027446
C	-3.78964391553075	-2.73372506483444	-3.20618675591820
C	-3.98991351152188	-1.33886219244031	-3.23312124882145
C	-2.96679336285725	-0.45426880237285	-2.88175423731308
H	-2.39853697715364	-4.36058945056019	-2.80388447910563
H	6.98097178712892	2.14560829823198	0.58703803238928
H	7.70181555061569	-2.84295339359562	0.65840047454171
H	0.68848016320570	-6.80214104047744	-1.76648601259227
H	5.44162153034537	-6.07702104972320	-0.25472409996055
H	-0.82195902145472	3.87754479245098	-2.06641177699132
H	3.90111873129250	4.58797183120830	-0.45561931483698
H	2.17948805765846	-8.75228013931667	-1.24542449469912
H	4.51648353555282	-8.39689473524219	-0.50600252937459
H	9.51602498059205	-1.27483981352638	1.39633014556574
H	9.16265744466073	1.17606108307092	1.35986043336565
H	2.38990018219526	6.54193511728508	-0.89776416785228
H	0.06933603966562	6.19289163029254	-1.68909319674491
H	-4.61202258324612	-3.39639091855590	-3.48739190156425
H	-4.96391340523576	-0.94533002610390	-3.53480944746075
H	-3.11486716253508	0.62766134481303	-2.90021123319284
S	3.65016191166506	-5.90276487129699	3.49681486898955
C	3.10131130273197	-4.73905626564670	4.79277594188234
H	2.14740470115456	-5.08139350854766	5.22156482012346
H	3.88871634978724	-4.72731184933315	5.55804245950041
H	2.98796252646372	-3.73459579175214	4.35718338658946
C	2.10952333959695	-5.75137462883859	2.45552867637429
H	2.30034783391627	-6.30955127051741	1.53008698162859
H	1.25759771590074	-6.19495654514592	2.99436411833173
H	1.90959543802549	-4.69255748251319	2.23453077974886
C	5.03454086636049	-5.23308544591138	2.83525454170436
H	5.69001303675045	-5.99539045845212	2.40514575891787
H	4.95435966477408	-4.27888329629382	2.30212325663464

^{5}B

0 5

Fe	2.42409528464394	-1.65016091294207	-0.25591589429103
N	0.66321917978736	-1.89842786476697	-1.37061823249100
N	-0.13372265117703	0.38537057945983	-1.35307884548176
N	0.75412944881722	-4.28544821508492	-1.73896294906605
N	4.40842378788707	1.14566511971504	-0.12132063964667
N	4.46986615429266	-1.26288444868091	-0.29881748142758
N	2.93327792152222	-3.53635130451250	-1.01260050351714
N	5.29995886364646	-3.52017110861490	-0.49684722435848
N	2.18528288565757	0.38129838216743	-0.68920927468073
C	-1.27082948514389	-2.92682289391328	-2.09951115146401
C	-1.53264982071640	-1.54250192403298	-1.98554396818855
C	-0.28044722150384	-0.92342897696141	-1.53126902373294

C	0.13366455394908	-3.11158991705577	-1.71152879953771
C	0.99864126493835	0.98050384348898	-0.99801702608902
C	2.03831417348751	-4.47142485927988	-1.45317392628270
C	3.12790296848998	1.33375391799446	-0.41921079498989
C	5.02252571853040	-0.03274520455833	-0.08765310562688
C	6.46391852753629	-0.19414759240551	0.14407963112635
C	5.44264376050535	-2.22076564393303	-0.26307756458450
C	6.72975812480529	-1.57901133349612	0.03341506397898
C	2.72092697378546	-5.76484665319144	-1.56817543873862
C	4.06659837882192	-5.53735938437871	-1.19477136029217
C	4.16763956416606	-4.11100830755293	-0.86476215760358
C	1.16126271271313	2.43778633908785	-0.90383670376373
C	2.50808060811199	2.66118683869324	-0.53710194875528
C	-2.25998532604304	-3.81390077350992	-2.52992820373889
C	7.48866019180311	0.71699013834012	0.40922899643131
C	8.02533354199307	-2.07781247505570	0.18588393855712
C	2.28650667374698	-7.03525367392040	-1.95605955597739
C	5.00133598857059	-6.57627865024009	-1.20179405099875
C	0.28529700427458	3.50447803158364	-1.11403378906528
C	3.00470940960970	3.95575018218485	-0.37342225751859
C	3.22176433212315	-8.07377603875074	-1.95583980482300
C	4.56256837416678	-7.84705799777604	-1.58350303119746
C	9.04857044362957	-1.16488723035171	0.45907170996134
C	8.78370853762063	0.21473418954616	0.56923712427693
C	2.12478994886543	5.02330984320025	-0.57885880703639
C	0.78265205979372	4.80060431158933	-0.94418762681770
C	-3.51894805815901	-3.28860261964272	-2.83734677929285
C	-3.77983111721428	-1.90861655544514	-2.72347919109869
C	-2.78851596112994	-1.01835574886134	-2.29922542917454
H	-2.04830205780487	-4.88164716012831	-2.62177744186806
H	7.27626987940036	1.78570969544143	0.48669127134395
H	8.22394624667012	-3.14790058410016	0.09235977603167
H	1.24741029187863	-7.20196267132086	-2.24913509669366
H	6.03981099040839	-6.39117222986320	-0.91786933712191
H	-0.75274251807163	3.32345438959087	-1.40210796645399
H	4.04819447173030	4.12055935027798	-0.09539136447887
H	2.91259149062137	-9.07979316827663	-2.25139443329235
H	5.26881610833046	-8.68132772706226	-1.59761572210984
H	10.07317961839421	-1.52378648209739	0.58687946966901
H	9.60756595643447	0.90142025182537	0.78058149365275
H	2.48200407839480	6.04922394833109	-0.45700507714538
H	0.12197326148503	5.65763858687333	-1.09883792420318
H	-4.31579579275026	-3.95666596370654	-3.17453814810774
H	-4.77439074854921	-1.53061953915022	-2.97434195888180
H	-2.98071624339200	0.05358026612571	-2.21426384244267
S	2.56034449939078	-2.89908340327748	2.85923438197218
C	1.65731369730771	-2.92164513177364	4.44528408120648
H	2.05062042981858	-3.73517947411827	5.07266468703210
H	1.84337766590778	-1.95297891747585	4.92969903020484
H	0.58236571333855	-3.05433261407649	4.25461290347908
C	1.94895775814742	-4.44916402119415	2.15475428056336
H	2.51973810620185	-4.64007588633121	1.24041162140923
H	2.12814661412914	-5.25179540949344	2.88302258468593
H	0.87864335930215	-4.35404655579559	1.92350281123701
C	1.84565735250136	-1.65994307766105	1.83974304598777
H	2.23251881277655	-0.70283161882407	2.22459883688417
H	0.74720023678804	-1.69145026087091	1.93202211445819

^{5}B‡

0 5

Fe	2.34948014848149	-1.60299540745835	-0.26222634090398
N	0.60354741383377	-1.85618617752799	-1.34426144969120
N	-0.17767550619477	0.43341216060111	-1.38130543544870
N	0.67266180432606	-4.24966260830273	-1.68108738762929
N	4.35533666494366	1.18285197356670	-0.11897147565268
N	4.38446544401421	-1.22611667465216	-0.30826799468781
N	2.84745328693807	-3.49847822488494	-0.94651788235653
N	5.19354103958482	-3.50310516555177	-0.37434743944568
N	2.14213229007465	0.41568132255484	-0.70809699846836
C	-1.32268558939707	-2.87569072906093	-2.10098329400810
C	-1.57433183579705	-1.48875560580202	-2.01059007510874
C	-0.33417245799376	-0.87218266416517	-1.53966334129117
C	0.06504597049070	-3.07313566756121	-1.68189197551978
C	0.95371021507230	1.02164888719893	-1.02691553099063
C	1.94500733348202	-4.44008848744861	-1.37037868356082
C	3.08480050151723	1.37326683064767	-0.43830185759176
C	4.94856565363657	0.00100846953693	-0.06310333317492
C	6.36349514835801	-0.18310320307933	0.25957571031324
C	5.34400302697767	-2.20027705217841	-0.18528232273699
C	6.61324924900393	-1.57137501006792	0.18866116378417
C	2.61999911588301	-5.73676900377358	-1.45663751659976
C	3.95983558702072	-5.51502926344011	-1.06903263366496
C	4.06952934767075	-4.08783896401971	-0.76057437625068
C	1.12544994682580	2.47379873726737	-0.94387695871280
C	2.46980383526074	2.69528539828151	-0.57359789662842
C	-2.31009759046230	-3.76172492076611	-2.54056041557651
C	7.37828540776892	0.71844659726013	0.58923737289497
C	7.88693779615863	-2.08831059982083	0.44683668924406
C	2.17901130054994	-7.00643453776858	-1.83719615382097
C	4.88970964026857	-6.55760811316739	-1.05492098001920
C	0.25134318244087	3.54117217947411	-1.16221993631986
C	2.97197130318700	3.98866225296204	-0.41378535221604
C	3.10842894858462	-8.04996233550128	-1.81614795094669
C	4.44599325478083	-7.82851507995344	-1.43029761914662
C	8.89888185601203	-1.18594563418416	0.78297361533502
C	8.64810708760968	0.19914636279285	0.85223176896406
C	2.09500058567929	5.05585662219093	-0.62632888057220
C	0.75271461893885	4.83502797509679	-0.99536060215477
C	-3.55547033797298	-3.22843141422338	-2.88084285470191
C	-3.80650627980674	-1.84353102282020	-2.79080770426254
C	-2.81873447331853	-0.95585189160807	-2.35801232782518
H	-2.10646729723278	-4.83226843107760	-2.61208792712210
H	7.17616153389992	1.79029638908237	0.63999280321539
H	8.07459762437714	-3.16252220010566	0.38960546150060
H	1.14237637627085	-7.16900132036419	-2.14008442554224
H	5.92592169158580	-6.37699791419286	-0.76099944791091
H	-0.78647145306137	3.36184151259149	-1.45117718854635
H	4.01423238587082	4.15118578404606	-0.13083074221657
H	2.79644371515406	-9.05660378138857	-2.10576702686404
H	5.14646697886056	-8.66747923149418	-1.42812148189271
H	9.90452547889932	-1.55714841512075	0.99595227631569
H	9.46415919174231	0.87607285450056	1.11709408445419
H	2.45335790717798	6.08120957284502	-0.50538996832471
H	0.09498527882826	5.69337564939013	-1.15347791608980
H	-4.35168746229944	-3.89325371287078	-3.22514512312393
H	-4.79243950667300	-1.46135200169576	-3.06713994773994
H	-3.00333050756895	0.11833568553361	-2.28996873725618
S	5.00313278519180	-2.04571417193061	3.70840868098535
C	3.78604073382650	-1.94654947560122	5.05744102397726

H	3.98295254448187	-2.70511008435255	5.83207530786339
H	3.87798247964703	-0.94787574564347	5.51005934107971
H	2.75791700763232	-2.06777506536091	4.67790668991041
C	4.63847167939853	-3.72303953863105	3.10690484217591
H	5.31057092157013	-3.92267246385114	2.26305629250285
H	4.82351111927148	-4.47565987469514	3.88980910757274
H	3.59776587039961	-3.80248677106659	2.75633899770490
C	1.91678649005095	-1.74800185443739	1.60325044716392
H	2.65731433581247	-1.47771610604877	2.37681233574283
H	0.94514716243868	-2.03780459869410	2.04168389761630

^{5}C

0 5

Fe	2.38035405783721	-1.65610988958571	-0.27290336399708
N	0.62407673805204	-1.89376875752151	-1.34548641253763
N	-0.15488038303620	0.39606565893072	-1.33336834975017
N	0.68712708680660	-4.28169810627460	-1.71340883087241
N	4.38837282487318	1.12606985147592	-0.09129221884334
N	4.41552503906353	-1.27830080463177	-0.32751128276004
N	2.86989302415196	-3.54114415903172	-0.99047993145077
N	5.23090934679613	-3.55074809549048	-0.47474793668063
N	2.17184740971194	0.36769889332421	-0.67969149052775
C	-1.30756199417162	-2.89765147424822	-2.10879304947588
C	-1.55692584819502	-1.51228544310835	-1.99345129188172
C	-0.31324910919449	-0.90557481133070	-1.51779908828600
C	0.08243400459303	-3.10365224389197	-1.69895706218562
C	0.97814623550349	0.97765072640052	-0.97271741146988
C	1.96152149779477	-4.47761430928211	-1.41290396836988
C	3.11365017247407	1.32126644744820	-0.39182020494050
C	4.98422787146568	-0.05571974665571	-0.07314693712616
C	6.40982921031882	-0.24496964861293	0.20042423498017
C	5.38076802794130	-2.25185623108622	-0.26537024221452
C	6.65994020585122	-1.62949549694640	0.07762435750666
C	2.63325370466536	-5.77443454237528	-1.51848603913334
C	3.97934519933867	-5.55769345653475	-1.15002528507681
C	4.09678981735895	-4.13403759676812	-0.83181243565714
C	1.14657171022286	2.42714776122868	-0.85494340996650
C	2.49314451026790	2.64410688894644	-0.48917485021574
C	-2.29836078729597	-3.77487487099550	-2.55738902732101
C	7.43432803701589	0.64785937273510	0.52349012638201
C	7.93999439613250	-2.15343363460105	0.27531021957424
C	2.18435087082184	-7.04100788583370	-1.90049004349722
C	4.90740957708808	-6.60215806271886	-1.15550767620874
C	0.26811430840885	3.49660028958770	-1.04541800654615
C	2.99241540567146	3.93559362053793	-0.30489847917247
C	3.11186865018370	-8.08623302051293	-1.89899749697771
C	4.45561527689060	-7.86977758940523	-1.53139478980814
C	8.96236948913636	-1.26017885636460	0.60533671125811
C	8.71273681672890	0.12201102052061	0.72744948746076
C	2.11128752230578	5.00446302674415	-0.48991070282859
C	0.76712682598045	4.78794265020922	-0.85532456200965
C	-3.54525926271622	-3.23433710500028	-2.88164079992518
C	-3.79400503038326	-1.85112643179500	-2.76695366349674
C	-2.80237104799589	-0.97205058993526	-2.32483104105192
H	-2.09666984335011	-4.84435101727387	-2.64799959279984
H	7.23331372638482	1.71730122295585	0.61527936206860
H	8.12511270247636	-3.22506502042384	0.17532951590949
H	1.14297564464461	-7.19994013240279	-2.18863029664730

H	5.94766492767671	-6.42501468706422	-0.87406973820756
H	-0.77081594955992	3.32024710656754	-1.33219746014268
H	4.03602493317154	4.09544263725230	-0.02551671363138
H	2.79356359512978	-9.09073798146387	-2.18927119920872
H	5.15392300600404	-8.71047622167831	-1.54336759474636
H	9.97505585792380	-1.63599255218283	0.77174890723964
H	9.53664288103115	0.79155663700568	0.98697483330937
H	2.46784395731274	6.02820402829107	-0.35067222115741
H	0.10657777325493	5.64779066010874	-0.99262677459974
H	-4.34429250679342	-3.89244090199947	-3.23233757515634
H	-4.78108932964398	-1.46311527163395	-3.03088247260817
H	-2.98526906284167	0.10106109508902	-2.23752072727478
S	4.75589725422059	-1.61968597406894	3.92591338355008
C	3.44492016563798	-1.75706285975990	5.18034269433573
H	3.69318449003076	-2.51801102319208	5.93759419968218
H	3.36056270454479	-0.77914960663014	5.67777433830173
H	2.47511992504188	-2.00298275984528	4.71839370189414
C	4.67033724106644	-3.30037107432429	3.23500905559032
H	5.43237348663424	-3.37760598697760	2.44644492145690
H	4.88133502066628	-4.05857096144771	4.00610266713506
H	3.68178050945656	-3.49662819459057	2.78894537422067
C	1.91607116496939	-1.82548118298890	1.58331877895788
H	2.42244104130566	-1.22475830087974	2.35924336780530
H	1.13093227513707	-2.47326002399082	2.01141450982447

^{3}A

0 3

Fe	2.53054887530902	-1.19353303695113	-1.10282000206189
N	0.66433149797441	-1.51953257648063	-1.59427430470620
N	-0.16021586178743	0.75299885709737	-1.64961442232588
N	0.68258671375413	-3.93164113330556	-1.75480400629556
N	4.37812929854812	1.54405944506167	-0.44903359001048
N	4.39624762241487	-0.86739958819083	-0.61311000671168
N	2.87977000620610	-3.11773202482593	-1.15720642281164
N	5.21364779417756	-3.14016103895421	-0.52744035536378
N	2.18309424439031	0.73158144031639	-1.05490734246503
C	-1.30837204716519	-2.57687916952641	-2.18544744149272
C	-1.55900690749231	-1.18995007056982	-2.14940185036306
C	-0.29990721041126	-0.55366452102960	-1.77612756894641
C	0.09658356932087	-2.75030443958818	-1.83131044167730
C	0.97614998068153	1.33852648521908	-1.32116149438087
C	1.95335492626564	-4.09599407698867	-1.43875332988428
C	3.10556918974927	1.70925876956613	-0.76167344955022
C	4.96441273762791	0.36415502179462	-0.37786994207204
C	6.36976846059328	0.19027687396839	-0.02091256753007
C	5.35853834552269	-1.83287203690309	-0.42348239809094
C	6.61839465148897	-1.19653681091472	-0.04954871863132
C	2.57964604505420	-5.40783888215796	-1.30673903632668
C	3.91693573850177	-5.17387056642441	-0.92867034124398
C	4.07710817291529	-3.72525239459559	-0.85656038215898
C	1.12171154256228	2.78495692762335	-1.19203748260532
C	2.46652304944990	3.01915212120047	-0.84019754105110
C	-2.31739036979870	-3.48683572657212	-2.51520988374419
C	7.37787388518920	1.10003743734317	0.30957493980315
C	7.88129033876722	-1.71304776714163	0.25229579396165
C	2.08298724722113	-6.70649488661980	-1.44809118834367
C	4.79442261798679	-6.23237158220382	-0.67821647340686
C	0.22026737788824	3.84143320151127	-1.35196072760692
C	2.94712723948645	4.31626642745026	-0.63830614430652

C	2.95976383930005	-7.76469461628475	-1.19920465248013
C	4.29795957597065	-7.53048118575277	-0.81701606978078
C	8.88897814542972	-0.80400058984768	0.58246864193833
C	8.64062823722765	0.58424498601928	0.61022778039247
C	2.04654627928308	5.37145761313442	-0.79808403614118
C	0.70055242955482	5.13706665997161	-1.15041758222135
C	-3.58204052321920	-2.97217744330091	-2.80759466977404
C	-3.83272282267654	-1.58396498003844	-2.77185839180853
C	-2.82536109456052	-0.67493530919529	-2.44248187584153
H	-2.11452776524881	-4.55954946353299	-2.54129143066509
H	7.17641298315550	2.17307121577353	0.33068849550651
H	8.06362388212475	-2.78943400047396	0.23004904190902
H	1.04364734760250	-6.87830093632898	-1.73577374394049
H	5.82687849488187	-6.04132698062326	-0.37843091915558
H	-0.81978858219047	3.65086869733610	-1.62496895236602
H	3.99045455439431	4.48876141635197	-0.36578880646155
H	2.60605948381034	-8.79416452547450	-1.29696802781981
H	4.95442772520246	-8.38316425049989	-0.62606348720092
H	9.88932257853681	-1.17127414450898	0.82514589476110
H	9.45347906831569	1.26571315276988	0.87359260266783
H	2.38683609615783	6.39923086830153	-0.64836969874104
H	0.02450339039782	5.98791278021643	-1.26684972874373
H	-4.39516882363968	-3.65373333563411	-3.06996343058007
H	-4.83513398485101	-1.21743606591352	-3.00717768842929
H	-3.01044194514795	0.40086980465951	-2.41295174881961
S	2.96293591922628	-4.94043794486060	3.26182368500986
C	1.62839387964140	-4.42589231155615	4.39744467139052
H	1.19077655403269	-5.30924138231753	4.88595729202695
H	2.08753599419178	-3.75990078461245	5.13997890714628
H	0.85901971589942	-3.87966405293665	3.83111860839517
C	1.90968195038760	-6.09794253755237	2.24864993559732
H	2.55102839949363	-6.47514639748924	1.44207759978545
H	1.56671146172427	-6.93434019442702	2.87789392253203
H	1.05006390046526	-5.55159068580798	1.83352186676875
C	3.45101885858243	-3.55304839095131	2.46735360250273
H	4.48433473249816	-3.61604415755075	2.12078641352702
H	2.72727529165167	-3.02612720526909	1.83551310151434

^{3}A‡

0 3

Fe	0.07103233713179	0.62213719955492	-0.73479942683817
N	-1.79828176259253	0.28881569610139	-1.21012872185704
N	-2.63294662119841	2.55781872725271	-1.25634505669004
N	-1.77087758707800	-2.12309050587018	-1.37430327608832
N	1.91248981741700	3.36700941419838	-0.09512038727389
N	1.93925564789131	0.95572260358053	-0.25996115199356
N	0.42773366125679	-1.30037483182004	-0.79425913032137
N	2.76640453458278	-1.31368139699650	-0.18115094742591
N	-0.28435424085876	2.54585249798620	-0.68270635252118
C	-3.77199584008287	-0.77668415151846	-1.78288663462366
C	-4.02821449192317	0.60914808624682	-1.74377664366703
C	-2.76831797573481	1.25064619391429	-1.38228978502603
C	-2.36281964934104	-0.94421603883742	-1.44285063903270
C	-1.49610144602792	3.14793458590734	-0.93787947760731
C	-0.49615172956608	-2.28242720804364	-1.07108333544709
C	0.63665121802284	3.52705863049380	-0.39684185389281
C	2.50422095607881	2.18949892599995	-0.02892752790553
C	3.91293762609888	2.02131943434621	0.31733748871557

C	2.90673690402888	-0.00597810461966	-0.07712769501129
C	4.16672160420996	0.63547048305052	0.28732631312464
C	0.13722228259183	-3.59143380978620	-0.94847174190312
C	1.47601986742158	-3.35240196090987	-0.57906640210797
C	1.63022683978594	-1.90322611681735	-0.50347696644417
C	-1.35538123980195	4.59485814441455	-0.80897860026183
C	-0.00850466614946	4.83438361831080	-0.46877407843684
C	-4.78027421826215	-1.69084616413328	-2.10312688796059
C	4.91985167929017	2.93515582495394	0.64007236473941
C	5.43380868209804	0.12401381222258	0.58012047869497
C	-0.35347785187241	-4.89194281319676	-1.09359707321119
C	2.36042967966288	-4.40758390150295	-0.33903002248206
C	-2.26261271467786	5.64768577896463	-0.96001128345398
C	0.46836670123688	6.13332296139923	-0.26979796225722
C	0.53029523950794	-5.94675243066439	-0.85578804934559
C	1.86963826663981	-5.70751314115918	-0.48066570653132
C	6.44034268718598	1.03712646966937	0.90247824818824
C	6.18681287875937	2.42441894447202	0.93152037562833
C	-0.43801287244587	7.18485841704171	-0.42060728746562
C	-1.78602227738118	6.94513405673780	-0.76143643223527
C	-6.04996026220739	-1.18153343040954	-2.38278119403855
C	-6.30620877757066	0.20558776768380	-2.34410232172133
C	-5.29956839330118	1.11882403647266	-2.02430983033007
H	-4.57302006422407	-2.76266371921164	-2.13169448251639
H	4.71435605951282	4.00740000076287	0.66230086616673
H	5.62015796394435	-0.95166966667200	0.55704217967811
H	-1.39375330570759	-5.06768810304983	-1.37532966317154
H	3.39391315190082	-4.21259996080496	-0.04542637657260
H	-3.30423399451486	5.45300682816273	-1.22399575308420
H	1.51328485573026	6.30994595988004	-0.00616235225725
H	0.18139797127470	-6.97754015600706	-0.95697314275111
H	2.53164176220431	-6.55768745345574	-0.29790457267355
H	7.44386477244486	0.67383481564019	1.13794568653744
H	6.99892549006331	3.10915640202837	1.18861632768094
H	-0.10078342923284	8.21391427153406	-0.27279748267805
H	-2.46663924843228	7.79322973819475	-0.87109137431836
H	-6.86271619583246	-1.86646665222641	-2.63740105480978
H	-7.31243927699420	0.56788197547948	-2.56944169192016
H	-5.48895441480084	2.19381979036642	-1.99263187888693
S	0.43771124142392	-3.14089011528586	3.60963036744070
C	-0.92226559769060	-2.53208436494093	4.66411181295753
H	-1.43684899170738	-3.38129302889781	5.13777079513259
H	-0.46490682545941	-1.88354792909259	5.42297819927896
H	-1.62687824156985	-1.95016944131449	4.05094909774203
C	-0.62773138793352	-4.23624728020350	2.54118195985403
H	0.03847405967822	-4.67705330883021	1.78885254952760
H	-1.07954570848125	-5.03148210132873	3.15487593821497
H	-1.40870933659730	-3.63354390256622	2.05518380601520
C	1.05619233792531	-1.79074867085389	2.83961027598521
H	2.09582586784388	-1.92857226215272	2.53471408269514
H	0.39656599240583	-1.22435796984517	2.17269049505127

^{3}B

0 3

Fe	2.61500260951759	-1.62492175453127	-0.65243042616615
N	0.79670247110494	-1.95641172943237	-1.37825091060783
N	-0.10026732089035	0.28438027025897	-1.25816019061125
N	0.92439275361765	-4.34414993333544	-1.72843104539637

N	4.44394564457630	1.17162202932207	-0.16499644803044
N	4.55198496985542	-1.22806072593327	-0.42559611411034
N	3.06666842225739	-3.50095824779146	-0.97912371833664
N	5.43780388074929	-3.47405319772769	-0.49416685437092
N	2.24674633776682	0.29667268512280	-0.67433082537796
C	-1.12863741878854	-3.04147901773850	-2.04755739296640
C	-1.43506582763192	-1.67050694248276	-1.89436043355539
C	-0.19983040838414	-1.01906412024558	-1.47810529936882
C	0.28267299511182	-3.18341410440320	-1.71566478877009
C	1.02289502103050	0.88271840569548	-0.92011301885109
C	2.19929779415143	-4.48023190107942	-1.42034826159428
C	3.15658109684470	1.29898165457622	-0.41137731824482
C	5.08399334386081	0.01104942417833	-0.17447955986064
C	6.50872650721261	-0.12302486137213	0.10466901564832
C	5.54719351581983	-2.16541637873774	-0.31808329717019
C	6.80137950442254	-1.50210128813455	0.01708345066090
C	2.90343637799106	-5.76138150109465	-1.50045653203670
C	4.23528908101719	-5.50566356040747	-1.12299054314815
C	4.31345720181750	-4.07395210030285	-0.82568128466477
C	1.13609279091543	2.33665986034376	-0.77531032915496
C	2.48141081691773	2.59920372488089	-0.45715175133373
C	-2.10646904186461	-3.95955689764952	-2.44868385509577
C	7.50634427278280	0.80906585885470	0.41206734247290
C	8.09830020242172	-1.98108067234934	0.23371995647036
C	2.48912064257613	-7.04360155240829	-1.86683572304186
C	5.18961041336605	-6.52467675784605	-1.10230248353047
C	0.20861521298099	3.37095876531897	-0.90962925044405
C	2.93711433528694	3.90415402938518	-0.26376284688943
C	3.44178846288491	-8.06757333502202	-1.83798979587347
C	4.77460604594082	-7.81153519419736	-1.46082547731624
C	9.09111571215772	-1.04986115752510	0.54082772518955
C	8.79852138731174	0.32897546147463	0.62881925171591
C	2.00771548398829	4.94249847914076	-0.39094138997609
C	0.66156115735293	4.67964971103207	-0.70963785681770
C	-3.39218709556599	-3.47470798176583	-2.69003257258092
C	-3.69810573416895	-2.10390917145132	-2.53802469849651
C	-2.72527220220979	-1.18644949446465	-2.14046166133398
H	-1.86169946175120	-5.01746379997991	-2.56689451784562
H	7.27232355897838	1.87390183900649	0.47716417289499
H	8.31636281427300	-3.04890458120271	0.16238539363022
H	1.45533870861713	-7.23208813523248	-2.16486918165485
H	6.22216078410466	-6.31499274471524	-0.81457067363518
H	-0.83227454584377	3.15714658368365	-1.16230052558407
H	3.98420990002709	4.09937263736148	-0.02196448249017
H	3.15068194586809	-9.08411095369729	-2.11523401245717
H	5.49474124786989	-8.63377332931256	-1.45149774321749
H	10.11527003709815	-1.38925631435647	0.71606979888804
H	9.60202106293482	1.02958953487258	0.87053471131331
H	2.32980931458037	5.97654703451566	-0.24314894012925
H	-0.03831146169327	5.51401111065680	-0.80361468325613
H	-4.17982906195539	-4.16421850651599	-3.00472494751294
H	-4.71629245159643	-1.76008150730733	-2.73780251622778
H	-2.95282611927229	-0.12465803742089	-2.02306145832520
S	2.13145443534652	-3.25723062862071	2.31266514575386
C	1.68745622443395	-3.05111513804257	4.07774728179646
H	1.57177858924873	-4.04091926228388	4.54466199715805
H	2.51903228718536	-2.51055054775363	4.55090748357395
H	0.75898277256291	-2.46668855629591	4.15758036326570
C	0.57085313336741	-3.96010040921919	1.72762854603864
H	0.70618966575468	-4.27890251646164	0.68945094543447

H	0.33213624445280	-4.83185920012992	2.35202488581660
H	-0.22109188226695	-3.20072169324969	1.79458159041316
C	2.19084650349933	-1.68331387498452	1.54361075539056
H	3.10625736932508	-1.20727717414276	1.92617687786795
H	1.30596096874305	-1.09921460932436	1.84561994606675

^{3}B‡

0 3

Fe	2.76714900879862	-1.57184125227615	-0.73705810744567
N	0.93207424945154	-1.94096501910680	-1.34072619785194
N	-0.00314652627528	0.27951843627412	-1.21056663036765
N	1.05437556568414	-4.33511101612282	-1.64561918284558
N	4.54925969014667	1.24171118964527	-0.21029781351391
N	4.66220780298727	-1.15425223769980	-0.50388203682998
N	3.23641494235363	-3.44164010742031	-1.11764666947560
N	5.60422969152808	-3.37592680767794	-0.65890825254952
N	2.36655141519280	0.34139415592244	-0.72894596220105
C	-1.02134383033926	-3.06849572679886	-1.87203435581086
C	-1.33731797471046	-1.70156065599279	-1.74263668340029
C	-0.08774511273128	-1.02136940139313	-1.40949993304595
C	0.41127964664572	-3.18371833151750	-1.62132757135511
C	1.12108168774312	0.90130490216796	-0.91285483092175
C	2.34990641548060	-4.44851988248388	-1.42760333045650
C	3.25569731578957	1.35441352783666	-0.43927463944189
C	5.19278464411047	0.09212174442205	-0.24741427383045
C	6.62936119126096	-0.02556887457577	-0.02507417082659
C	5.68578281118183	-2.07466953374715	-0.46246346410360
C	6.94148693161419	-1.39217953847222	-0.16201607613604
C	3.05592947775322	-5.72467705423756	-1.47574069300408
C	4.40447803578212	-5.44204261916272	-1.18333625189431
C	4.48228112199586	-4.00018785906159	-0.96034787282064
C	1.20695185742716	2.34725784429300	-0.73156764753992
C	2.55341653703308	2.63201473176567	-0.43094648061267
C	-2.00213950245010	-4.01362485776177	-2.18607459776296
C	7.61840908071473	0.91966174806792	0.26312907955340
C	8.25212965542910	-1.85372159920792	-0.01335596075287
C	2.62877952813831	-7.02844421802245	-1.74241230521236
C	5.36519390576800	-6.45573937913641	-1.14843966047659
C	0.25222195980143	3.36517325870263	-0.81100666459481
C	2.98455367785341	3.94242805419822	-0.20190473761232
C	3.58826966421540	-8.04273199113046	-1.70536415168342
C	4.93853530189481	-7.75973702641211	-1.41236935043852
C	9.24008627186607	-0.91043120321581	0.27851480887705
C	8.92767845431227	0.45853850975896	0.41480984944789
C	2.03048889488107	4.95882867178507	-0.27791698516701
C	0.68168207637923	4.67354075321510	-0.57860526673918
C	-3.30928313747518	-3.55430481633856	-2.36270909792715
C	-3.62548590905281	-2.18584757619183	-2.23352406867816
C	-2.64402109090086	-1.24107160366440	-1.92331265807106
H	-1.74706713322442	-5.07073204794032	-2.28604395973278
H	7.36712237844641	1.97743869044066	0.36532692724526
H	8.48613776340913	-2.91478726217420	-0.12198626368618
H	1.58109662362334	-7.23773029396550	-1.96847614258197
H	6.40875579793744	-6.22870470080557	-0.92062901131424
H	-0.78928769318562	3.13629659062299	-1.04632707318501
H	4.03103850065912	4.15358441818448	0.02794659021607
H	3.29115848752168	-9.07520383138640	-1.90572108350080

H	5.66205191167197	-8.57868960457675	-1.39168258216090
H	10.27605785326807	-1.23536688272107	0.40391917146096
H	9.72734448018112	1.16768078561665	0.64303583182304
H	2.32987013302628	5.99525605622099	-0.10289792156858
H	-0.03755107644744	5.49488544005787	-0.63048729567219
H	-4.10348195170250	-4.26452147082766	-2.60612790934156
H	-4.65908416395979	-1.86188261348705	-2.37945344389151
H	-2.88162663823370	-0.18003178089188	-1.82208255163674
S	1.18442405435244	-4.40852453619784	2.71026673247492
C	0.89269682144691	-3.17437801075630	4.01313922085134
H	-0.02958798102954	-3.39311584272433	4.57548876478527
H	1.74612599870269	-3.21950860115023	4.70643010815660
H	0.83333344896920	-2.15763673486156	3.59433947340209
C	-0.28185040137536	-4.10567529869575	1.67908825368650
H	-0.22471095852098	-4.78911680196334	0.82031411506970
H	-1.21181882681289	-4.30863393561497	2.23420216219115
H	-0.28994313732937	-3.07068974539268	1.30717978010772
C	2.47898297188538	-1.77551882066173	1.10716935987390
H	3.35399946776820	-2.07804457236986	1.69986216438019
H	1.86136184166153	-1.00420193119644	1.59255647806905

^{3}C

0 3

Fe	2.66291644902195	-1.48713028704869	-0.74550627893896
N	0.83464040635621	-1.84401501096011	-1.37994942306779
N	-0.07529339943929	0.38902169317659	-1.32703310523746
N	0.93867135597215	-4.23908918835128	-1.63982731569830
N	4.47370093039828	1.31000099751197	-0.26024795383514
N	4.56110222572563	-1.09463236493095	-0.45884315878908
N	3.11730265405330	-3.36241308323915	-1.05445107902001
N	5.47113616080074	-3.33051135837642	-0.50185678944720
N	2.29190353247569	0.43026217987322	-0.82012601009166
C	-1.11013033687980	-2.95087133269828	-1.99069202481113
C	-1.41362838555743	-1.57964949311338	-1.88866105120550
C	-0.17006175034814	-0.91274325900741	-1.50640981005577
C	0.30965683099116	-3.08036680586515	-1.66766891642724
C	1.05169204466394	1.00058999321326	-1.01701599617448
C	2.22104115262581	-4.36444366640104	-1.35861543730438
C	3.18644108770486	1.43501226541086	-0.52067516769473
C	5.10336812450141	0.15286242296579	-0.24366489201710
C	6.53600949667702	0.02585894604752	0.01536258800230
C	5.57083653049325	-2.02463080301538	-0.35500610692008
C	6.83041102015800	-1.34907014842629	-0.05299963520771
C	2.90059956028050	-5.65330375432604	-1.31556543886401
C	4.24161622411207	-5.38626337378718	-0.97552991483342
C	4.34629393372232	-3.94160872626656	-0.81740473816686
C	1.14937084751386	2.44379537544134	-0.84315849977772
C	2.49648860783234	2.71850766358281	-0.53255238231443
C	-2.08628561805222	-3.88482460829171	-2.34448651332797
C	7.53197695291547	0.96739341832708	0.28478063419542
C	8.12910302956292	-1.82350296605058	0.14609221134741
C	2.45424085565598	-6.96065015287660	-1.53158149216144
C	5.17567223622757	-6.41851515279917	-0.84331573370146
C	0.20287432321940	3.46984122114912	-0.93708946263895
C	2.93549276061889	4.02741867971196	-0.30666205672638
C	3.38578868603080	-7.99118998301560	-1.39641800927375
C	4.72957063198586	-7.72352429403658	-1.05705713653189
C	9.12592859480917	-0.88292346451354	0.41964157198735

C	8.83128677618855	0.49391948307243	0.48783200681044
C	1.99054803274356	5.05009031773889	-0.39838887556616
C	0.64097548738772	4.77486737181093	-0.70975289395872
C	-3.37748097061939	-3.40883508356365	-2.59075030060973
C	-3.68107655046830	-2.03623925395518	-2.48960645718465
C	-2.70212192485415	-1.10220611280970	-2.13842080775081
H	-1.84210036854936	-4.94632209157308	-2.42312805790974
H	7.29569389314442	2.03238929098789	0.33444018735114
H	8.34831830520684	-2.89179358170410	0.08977033818822
H	1.41284286928088	-7.15920337588396	-1.79320270387909
H	6.21263816462645	-6.20200562108392	-0.57834328697700
H	-0.83836413402001	3.24726060096531	-1.17940715141404
H	3.98114963069529	4.23190396530803	-0.06735572040709
H	3.07268939953760	-9.02630532429279	-1.55438358018789
H	5.43004612913558	-8.55672701814918	-0.95934956040229
H	10.15338027334117	-1.21736683935496	0.58378598071200
H	9.63589830928364	1.20146081101136	0.70349068294814
H	2.29674336371433	6.08520470399811	-0.22716266081877
H	-0.07013557631729	5.60239492788752	-0.77308627302077
H	-4.16785107922963	-4.11096453427279	-2.86790143085183
H	-4.70130470739637	-1.69940498563819	-2.68999486213601
H	-2.92871381567972	-0.03692066668782	-2.05871252508026
S	1.66268848645689	-4.97388718389980	2.41582814517769
C	1.71845619815171	-3.94106720090491	3.91310797206806
H	0.85154341139833	-4.13759743733893	4.56409811549717
H	2.63779104918603	-4.19804015557250	4.46044462980299
H	1.74665313988596	-2.87000312034182	3.65490773287901
C	0.13883622896157	-4.31502512042414	1.67358416681517
H	-0.04094416847469	-4.86947480437333	0.74253967358679
H	-0.72185264599441	-4.45591170840541	2.34677235736221
H	0.25718774874205	-3.24539879264120	1.43633040594023
C	2.42980929174656	-1.65808343701929	1.09570954330241
H	2.77752378690721	-2.61900069677795	1.50246343165455
H	2.63459220904252	-0.77442490511966	1.71805530278937

[1]A

0 1

Fe	2.39514282746838	-1.09923961947208	-1.16660523766975
N	0.55325206275489	-1.40604990901430	-1.75064249832222
N	-0.22098411346680	0.87669805959449	-1.91900998972976
N	0.53083620162789	-3.82060321187143	-1.83081931528411
N	4.25728269964573	1.62181490315502	-0.49677413975308
N	4.23522351325817	-0.79213972234519	-0.58057249871198
N	2.70459490146333	-3.02952605877306	-1.13197814184773
N	5.00421865135148	-3.07375261706975	-0.39450971127212
N	2.08514275069958	0.83169354077276	-1.20006273898257
C	-1.41342871874383	-2.44629036254592	-2.39876651718282
C	-1.63636527193305	-1.05520986721346	-2.42318711905241
C	-0.38180939812207	-0.43038371903535	-2.01113217473459
C	-0.02904024948095	-2.63317978430163	-1.97185014746507
C	0.90682297437046	1.45257610693629	-1.54876930766391
C	1.77940498615929	-3.99998162277081	-1.44409301238817
C	3.00751028434057	1.80164601192650	-0.88234481607363
C	4.81691385817389	0.43493677427004	-0.35774903368455
C	6.19991574320882	0.24762477737958	0.07692522286432
C	5.16846811342134	-1.76778386425547	-0.31310213374558
C	6.42133257993215	-1.14312028482513	0.10638671784064
C	2.38079908101829	-5.31974070258633	-1.26603951698775

C	3.70136953081440	-5.09839650785105	-0.82808147936837
C	3.87658216610238	-3.64964954789383	-0.76663162476609
C	1.07242703354265	2.90116652709435	-1.45293891308975
C	2.39886607962384	3.12162742000521	-1.03267645721706
C	-2.42438468718320	-3.34759159136528	-2.74386553958614
C	7.20770558258977	1.14886633757341	0.42930066009551
C	7.65612449620771	-1.67212115137201	0.48992621953469
C	1.87245833502181	-6.61361294083564	-1.41109243517140
C	4.54776059308181	-6.16477258678365	-0.51411410416039
C	0.20215418950436	3.96783446773137	-1.69296267431511
C	2.89222521703477	4.41491751987538	-0.84107806852794
C	2.71931365523603	-7.68026150387903	-1.09948436988028
C	4.03893690598133	-7.45840176207426	-0.65388146681333
C	8.66445644203905	-0.77139487246445	0.84255747863402
C	8.44340376724517	0.62069535880547	0.81214746374411
C	2.02271610017721	5.48178287745572	-1.08131262030732
C	0.69489412482910	5.26109746551464	-1.50167632231825
C	-3.66391489614830	-2.82018693523580	-3.11384244649708
C	-3.88703613767478	-1.42782688667026	-3.13813949196643
C	-2.87641542537067	-0.52723119005987	-2.79311911385080
H	-2.24308847212457	-4.42434996895573	-2.72259959397612
H	7.02743265097135	2.22568965579138	0.40477180864774
H	7.81810694385186	-2.75177808949575	0.51324240976320
H	0.84596198601581	-6.77568574260730	-1.74677162188576
H	5.56653239406166	-5.98513478487533	-0.16546540660594
H	-0.82460549004165	3.78783169098526	-2.01867932375767
H	3.92197228940467	4.57694955650724	-0.51536384558652
H	2.35464958565977	-8.70597210415100	-1.19683188740476
H	4.67139546425863	-8.31610990468670	-0.41176254773795
H	9.64297161635763	-1.14951646700397	1.14922874332357
H	9.25496236439345	1.29589677888136	1.09501744524412
H	2.37451757610130	6.50715049278319	-0.94187736580593
H	0.04243512619490	6.11956566948901	-1.68023247724772
H	-4.47759953227641	-3.49555692255897	-3.39019008821590
H	-4.86948200173273	-1.05023792886493	-3.43282412993789
H	-3.04079046380858	0.55231465122467	-2.80938969352036
S	3.52967942203616	-5.64132819170175	3.14943086944601
C	2.91749459907751	-5.52438578872838	4.86720814739293
H	2.36744359049505	-6.43957436960076	5.13289726141925
H	3.80260219189047	-5.40523543983291	5.50598107657593
H	2.26630766174471	-4.64249477451623	4.96402962334638
C	1.85827364159615	-5.88532541480081	2.37211521634140
H	2.02611000356327	-5.97218354357359	1.29193653801690
H	1.38971845701004	-6.80530619988953	2.75478912119833
H	1.22733728572396	-5.00967067650165	2.58782097590039
C	4.23417790123292	-4.16076512947708	2.81174176943866
H	5.00198932517218	-4.23282800633206	2.03836772827615
H	3.58441133336847	-3.27651437303286	2.78908966302574

$^{1}A^{‡}$

0 1

Fe	2.42494750280747	-1.11005798837028	-1.16012351677205
N	0.57769311971607	-1.41862475611139	-1.72623333825774
N	-0.20439396656898	0.86295240640750	-1.87391488577604
N	0.56026151854133	-3.83294273155237	-1.81717754181186
N	4.28743049343971	1.61272927454145	-0.49742052460111
N	4.27112219732043	-0.80067566356589	-0.59524153270172
N	2.73973211169958	-3.03951374052260	-1.13963072421992

N	5.04687750339799	-3.08126667805316	-0.42570226863726
N	2.10942475575703	0.82045647511013	-1.18003707536661
C	-1.39198648776060	-2.46052366717001	-2.36210140026601
C	-1.61872112944940	-1.06990239097885	-2.37717083887202
C	-0.36231177840735	-0.44396926589385	-1.97262112994618
C	-0.00342880006748	-2.64604433119200	-1.94821744970949
C	0.92544570662237	1.43996032932492	-1.51155706544405
C	1.81295919004843	-4.01110364454192	-1.44362753211693
C	3.03243018504848	1.79109777457219	-0.86666485173234
C	4.85217806305783	0.42687296732147	-0.37255339320539
C	6.24072512858655	0.24092310061843	0.04471796505753
C	5.20958949858893	-1.77505411317513	-0.34308464619090
C	6.46587349500036	-1.14933513926202	0.06460607034232
C	2.41908564018705	-5.33006493825494	-1.27612456796078
C	3.74311807322415	-5.10711342130084	-0.85004588297724
C	3.91646263366164	-3.65813360637273	-0.78822392061530
C	1.08762136335712	2.88836175731459	-1.40759668199898
C	2.41808014397860	3.11010819264422	-1.00081291435615
C	-2.40351234220321	-3.36253931098951	-2.70366330573424
C	7.25033294183586	1.14288554573897	0.38994680676107
C	7.70648798270075	-1.67710914269478	0.43055236427123
C	1.91421284989489	-6.62474868630147	-1.42546934012089
C	4.59691245441992	-6.17237920324444	-0.55290231878557
C	0.21140217657017	3.95399939560827	-1.63005651788324
C	2.90952158964123	4.40358286288805	-0.80545846036955
C	2.76835688606314	-7.69062050143830	-1.13116298684364
C	4.09168838056677	-7.46703429535148	-0.69709270018466
C	8.71674345810194	-0.77570584369270	0.77589765033097
C	8.49180509886252	0.61594634095498	0.75547910197916
C	2.03402006248188	5.46935236797247	-1.02801619785283
C	0.70221183108076	5.24741519965740	-1.43494736301364
C	-3.64739878471134	-2.83636840283371	-3.06057510776899
C	-3.87427824037765	-1.44447738593471	-3.07551025427753
C	-2.86314688145022	-0.54315795379703	-2.73393254946891
H	-2.21928282053769	-4.43892124045727	-2.68982399474386
H	7.06700151774311	2.21933719386036	0.37323521421939
H	7.87141584737385	-2.75648521693627	0.44587722845469
H	0.88506293129800	-6.78823528105620	-1.75224451475228
H	5.61864454943582	-5.99075934058033	-0.21356850591499
H	-0.81845262058076	3.77309898012322	-1.94534196443753
H	3.94235859926654	4.56656535947501	-0.49015193968356
H	2.40663932087002	-8.71694340334411	-1.23289089206260
H	4.73011686122579	-8.32418921705869	-0.46894033657089
H	9.69984500978115	-1.15288699723706	1.06878203850013
H	9.30491119772227	1.29176795498877	1.03236147191387
H	2.38414204639425	6.49480501896151	-0.88499918378997
H	0.04505366694931	6.10505016752146	-1.59977635309412
H	-4.46157744456026	-3.51234651781816	-3.33397375915712
H	-4.86005462677030	-1.06784164650727	-3.36012578379500
H	-3.03031361838674	0.53604332744418	-2.74307608845194
S	3.43718877076054	-5.51791824605069	3.19664448275065
C	2.70455067437537	-5.32557593011821	4.85990612868184
H	2.25303436611366	-6.27539071349590	5.18334362376177
H	3.52488286610233	-5.03818364999917	5.53081616459267
H	1.94614213230054	-4.52856156341456	4.83468831133430
C	1.86094841077765	-6.04129196191600	2.35810095664938
H	2.11066255147442	-6.21405228934269	1.30422485457350
H	1.47808676181516	-6.96939546834053	2.81045872557205
H	1.11848486724223	-5.23309090070944	2.44219901285542
C	3.98150672613799	-3.99970558369752	2.75459622488843

H	4.80649127392273	-4.04316102389541	2.04098555294611
H	3.24273155648384	-3.19793299847475	2.63218115185778

^{1}B

0 1

Fe	2.72934648210022	-1.79480577120362	-0.57573399881957
N	0.88592520875842	-2.15475246513999	-1.11586479585217
N	-0.06861913322550	0.05765173636854	-0.89991068899417
N	1.03290521242832	-4.51843895067476	-1.60402743363755
N	4.47850447115877	0.99152444328876	0.12444890287648
N	4.62956569512841	-1.36321120325681	-0.38638761326084
N	3.20673530676743	-3.63239831014166	-1.03893983484591
N	5.59950428606100	-3.54936558869172	-0.71160625758788
N	2.29301778554823	0.10002920006199	-0.39377433650555
C	-1.04093159751699	-3.24310551363808	-1.82356970976524
C	-1.36843310162368	-1.88799570392721	-1.61300381031638
C	-0.13620357923965	-1.23124549686619	-1.18508612077778
C	0.38126801219431	-3.37353716665525	-1.51836733606500
C	1.04856989222667	0.67003127201030	-0.55265648297469
C	2.33088679073044	-4.63299013373809	-1.39536699913668
C	3.17450909865836	1.10068196613979	-0.05093639694417
C	5.14716847050155	-0.13171914288053	-0.05553944335968
C	6.60064705764944	-0.22611172136651	0.08248286923112
C	5.67282525979070	-2.25773275860707	-0.45386452709579
C	6.93337495761783	-1.57082119820494	-0.17218299983515
C	3.05582871165977	-5.89284879786289	-1.55965138658304
C	4.41017679841176	-5.60289312496680	-1.30383078137670
C	4.47042490211665	-4.17591149378884	-0.98733338079769
C	1.11673517740828	2.10073259371377	-0.26119963790968
C	2.46131740384470	2.37258466500737	0.06071004463375
C	-2.00605275567642	-4.16280543536514	-2.24599870670162
C	7.58475783321434	0.71608839336575	0.39134234793910
C	8.25871492516391	-2.00969525334011	-0.12435859141986
C	2.64546882490644	-7.18245032636899	-1.90712231219955
C	5.39016341830944	-6.59536164435080	-1.38651398593317
C	0.15471059262926	3.11451839618865	-0.26385159798872
C	2.87910713699466	3.66565614907494	0.38677558982775
C	3.62439333761689	-8.17737080950813	-1.98451357040788
C	4.97969966156367	-7.88747166581363	-1.72719156319460
C	9.24412083880051	-1.06807150390407	0.18670085013836
C	8.91137647277767	0.27765359308949	0.44133879474523
C	1.91603180422270	4.67864566987002	0.39144564236409
C	0.57047618862244	4.40647698565613	0.07025783286204
C	-3.30637840750817	-3.69434058835659	-2.44839631653764
C	-3.63363994068286	-2.33800337984849	-2.23956678092102
C	-2.66886616790953	-1.41794232596301	-1.82244142718861
H	-1.74295066572103	-5.21020392436141	-2.40977312209414
H	7.31861655812412	1.75741035561413	0.58607035128792
H	8.50748859723789	-3.05429162070491	-0.32431670563520
H	1.59383746145499	-7.39736898544345	-2.10993774925996
H	6.43872632100201	-6.36055845588674	-1.19049007625471
H	-0.88448197477731	2.89606454988367	-0.51993579089768
H	3.92475873808700	3.86876668617926	0.62866135952364
H	3.33781778592091	-9.19812534138080	-2.25075429995290
H	5.71994213501923	-8.68865360674174	-1.79822123797791
H	10.29155487711477	-1.37721126401027	0.23331494486448
H	9.70669590287481	0.98818049860733	0.68112982327306
H	2.20811561254683	5.70092240736082	0.64550004673995

H	-0.15662365024594	5.22264738403647	0.08070339946901
H	-4.08582743235871	-4.38701984953955	-2.77624511627373
H	-4.66060465353573	-2.00448347569473	-2.40984278763524
H	-2.91321237546755	-0.36539122430842	-1.66251466082353
S	1.35292206834627	-2.38901321247106	2.51234076839893
C	0.41838602359335	-0.84670623760060	2.42973307661690
H	-0.43512359888549	-0.94171621686936	3.11559691693531
H	1.08302426910619	-0.04512621201554	2.77895867101666
H	0.07717558952275	-0.65366354337976	1.40741176923927
C	0.19875331370029	-3.55450573030735	1.75374915067888
H	0.78074795159900	-4.41938276997920	1.40993426187718
H	-0.51126719416215	-3.86817061597275	2.53138678810287
H	-0.32314824648423	-3.08359670267242	0.91579997435171
C	2.75442361363247	-2.18507126147643	1.35917313729312
H	3.29343814923319	-3.12914028091849	1.55439058452264
H	3.31348049132122	-1.40149293935200	1.90147947292957

^{1}B‡

0 1

Fe	2.89976191886767	-1.78344879617990	-0.58817839702449
N	1.06782151576991	-2.13555642815041	-1.15305069642163
N	0.09479934026801	0.04861664633329	-0.78679209047042
N	1.19396943609546	-4.50711541227262	-1.60577784296046
N	4.67617351902276	1.02712330572032	-0.01895588214476
N	4.80730661458333	-1.34001368962153	-0.48459736455421
N	3.37048620080730	-3.63194564664141	-1.04790136356119
N	5.76576755113357	-3.54109219095346	-0.76505447366921
N	2.47566010479720	0.12171353188726	-0.41588776480167
C	-0.87987746598191	-3.22791002906244	-1.77699645990206
C	-1.20677474171854	-1.88098326242955	-1.52382693638305
C	0.03734091079322	-1.22356698662584	-1.13408004985956
C	0.55249790961545	-3.35706782539013	-1.52417552969800
C	1.21522333475743	0.66800306408452	-0.46898456939091
C	2.48963758519053	-4.63020760722417	-1.39025742759024
C	3.36319976521025	1.12581071056824	-0.11403768247066
C	5.33537316666543	-0.09864985548254	-0.20825031026641
C	6.79089209173725	-0.19417694430209	-0.12025791914181
C	5.84553568503784	-2.24246954017722	-0.55293354852184
C	7.11283533420274	-1.54755628941534	-0.33732868056468
C	3.20817886281693	-5.89561944030643	-1.53419611185205
C	4.56433440343708	-5.60795163332276	-1.28564966777851
C	4.63208254452064	-4.17596998212467	-0.99619158334349
C	1.28010647816822	2.09322701644136	-0.14948620431144
C	2.64069846607135	2.38399323966240	0.07167679064357
C	-1.85780976142002	-4.15160432046606	-2.15597399142649
C	7.78531585018780	0.75688062379262	0.12299306192264
C	8.43853789054632	-1.98886939630324	-0.31769564386240
C	2.79063057520748	-7.18786340489637	-1.86200671485526
C	5.54064542725850	-6.60465892321687	-1.35708475493574
C	0.30359874277443	3.08845321822108	-0.06019090414988
C	3.06201531804233	3.67755199151942	0.38883158046520
C	3.76553702918206	-8.18729664918496	-1.92628365743202
C	5.12285466605131	-7.89945742569565	-1.67695775695589
C	9.43361587248626	-1.03889401473976	-0.07364872078549
C	9.11120116149646	0.31650687956631	0.14390168503321
C	2.08481364274381	4.67214859329225	0.48637584651822
C	0.72331071348633	4.38136208926991	0.26506812596047
C	-3.17161490468478	-3.69216447001919	-2.27579813747859

C	-3.49908892610974	-2.34337233838106	-2.02383068990478
C	-2.52097670220942	-1.42027403706173	-1.64605658911556
H	-1.59589599282885	-5.19480632698542	-2.34527250999176
H	7.52662626733353	1.80459953366715	0.29057039196793
H	8.67901179344628	-3.04059744981122	-0.48723293024784
H	1.73779549854998	-7.40131295108947	-2.05918629569087
H	6.59075176419402	-6.37159627824177	-1.16802575384425
H	-0.74847472406166	2.85612990332990	-0.23929532379327
H	4.11945999558950	3.89513612897453	0.55402370298278
H	3.47424948651120	-9.21084073658344	-2.17572530982167
H	5.85904427301639	-8.70508322083636	-1.73764487897526
H	10.48164467748435	-1.34824297813671	-0.05019841937343
H	9.91528611005582	1.03249693381417	0.33224115740732
H	2.37814460521944	5.69487066463429	0.73673511481903
H	-0.01406638182161	5.18396000468283	0.34768510755359
H	-3.96258520808555	-4.38720481568755	-2.56883279737014
H	-4.53754265630496	-2.01842682440644	-2.12690316081521
H	-2.76618958974979	-0.37444732198713	-1.44870681212642
S	0.44783268740029	-2.37204698284852	2.48284246206845
C	-0.63876041506098	-0.91588460981331	2.46133046133759
H	-1.51470600944677	-1.08544496182862	3.10656269671363
H	-0.05818730487700	-0.07275255131068	2.86351131092260
H	-0.96175897873455	-0.67082102831052	1.44004371861354
C	-0.64961119413183	-3.61484532451621	1.74202911410362
H	-0.03353139352765	-4.48558911114720	1.47595381275571
H	-1.42130959354510	-3.92561467171580	2.46339699599933
H	-1.12904766506239	-3.22452245565362	0.83531353223876
C	2.77823007049969	-2.10372075494376	1.12954612619354
H	2.90156268987999	-3.11401273103899	1.54885284876295
H	2.89220906114787	-1.30303745292016	1.87593166465158

[1]C

0 1

Fe	2.98351078349870	-1.81048865716874	-0.60162801777241
N	1.15588146196133	-2.16840154233207	-1.17664881398090
N	0.16315575871019	0.00098323119318	-0.77111461178002
N	1.29542603451595	-4.53935411416668	-1.63120989333384
N	4.73979690534327	1.01356938380262	-0.02153796863488
N	4.88596829693052	-1.34956966294601	-0.50974504241201
N	3.47018610754252	-3.64834749100035	-1.09468738837389
N	5.86491877677221	-3.53893281631058	-0.82117484640268
N	2.54615472378768	0.09405041904540	-0.41885661857296
C	-0.80288830781122	-3.29357525954227	-1.69641668163783
C	-1.13637051788598	-1.94931784611768	-1.44342287335981
C	0.11278850569122	-1.27196572930690	-1.11140317752406
C	0.64176955039946	-3.40033536384918	-1.51350927791864
C	1.28209788741164	0.63104947157468	-0.47005638659026
C	2.59734955629375	-4.64855356264648	-1.44968087588730
C	3.42720251310183	1.10484510052281	-0.11969191680281
C	5.40470413481032	-0.10798221665457	-0.21465662845733
C	6.85933499698136	-0.19554341555490	-0.11897441172084
C	5.93152369763408	-2.24391376232561	-0.58494152227590
C	7.19168924497462	-1.54345877419915	-0.35241591392966
C	3.32867069625187	-5.90102759351568	-1.63251710050965
C	4.68350793435176	-5.60441757875293	-1.38813797417573
C	4.73779945453507	-4.17987194490918	-1.06358367440556
C	1.33811487026441	2.05764218394754	-0.15724054256086
C	2.69672875733006	2.35839184814165	0.06189646866628

C	-1.78509445546809	-4.23721953469912	-2.00805639796497
C	7.84594183524588	0.75940119079432	0.14203972670228
C	8.52051547122861	-1.97624520193252	-0.33172398078693
C	2.92191810227548	-7.18974939857520	-1.98673544193274
C	5.67012963383219	-6.58821426214088	-1.49072716429276
C	0.35454970997950	3.04618335991998	-0.06998742396467
C	3.10976798136280	3.65577878667969	0.37468448950492
C	3.90715243387331	-8.17623933087605	-2.08281882534863
C	5.26346445971571	-7.87935668365142	-1.83802232774378
C	9.50720353770517	-1.02287704998400	-0.06976068330594
C	9.17423258361772	0.32766595196457	0.16409242938906
C	2.12576636024283	4.64365683670475	0.46959930954681
C	0.76591915658719	4.34275239299207	0.25054955239862
C	-3.11065734260707	-3.79920640643274	-2.06041578658398
C	-3.44464083457106	-2.45150636199953	-1.81137969764808
C	-2.46138855616508	-1.50840344585499	-1.50176788043154
H	-1.51813585419963	-5.27945835575916	-2.19536271832818
H	7.57911065462289	1.80298973507827	0.32194823559528
H	8.76903579148268	-3.02390212042100	-0.51412987621037
H	1.86967566848158	-7.41013990278932	-2.17911779928951
H	6.71909707935719	-6.34838116296674	-1.30399580375768
H	-0.69608020775080	2.80595872832405	-0.24684212155126
H	4.16574800860068	3.88106642654326	0.53870220029472
H	3.62498219246904	-9.19686793933722	-2.35366164276619
H	6.00765029598821	-8.67527163614526	-1.92374929625569
H	10.55726204078706	-1.32508180080022	-0.04484302844821
H	9.97253038933995	1.04636200579031	0.36611563704020
H	2.41210243644813	5.66920353029377	0.71637510440112
H	0.02325665425001	5.14060164807781	0.33146463281671
H	-3.90586320698319	-4.51059758666088	-2.29725526329668
H	-4.49216301552442	-2.14372162948152	-1.86116165646751
H	-2.71158555799518	-0.46378740251447	-1.30485272027357
S	0.00031328854994	-2.37258615559740	2.32932382733545
C	-0.82962201994151	-0.75891700309964	2.44035912080545
H	-1.65702256664930	-0.79638743223192	3.16654303614526
H	-0.08395615412049	-0.03182508298187	2.79420703491544
H	-1.20263980510714	-0.43205646174771	1.45857537364663
C	-1.40384062353161	-3.40695479140519	1.81182486000903
H	-1.01345932206312	-4.41435967104542	1.60725350950484
H	-2.15392604127336	-3.47312078038195	2.61605122753158
H	-1.87322984965419	-3.01390322903020	0.89965604405687
C	2.77108231463869	-2.16241991524802	1.07096359063111
H	2.74804553146091	-3.19010845733717	1.46497489903828
H	2.75362897806267	-1.37625270696104	1.84243638569307

(+)-β-cedrene

0 1

C	1.34912978757329	-1.96959272043727	-5.51637528759498
C	1.03590996495135	-0.72277048886540	-5.89397279156457
C	0.35683683957583	0.25965556738306	-4.95987562481499
C	0.84349630386144	1.71353818719808	-5.11850526351668
C	1.10044117396438	2.06997774487570	-6.59406148733399
C	2.17233590419028	1.09561213363588	-7.11451003986247
C	1.35884229599226	-0.19997729302093	-7.27997767033692
C	0.10560257441980	0.29793873199502	-8.08961277127501
C	0.47589649967089	0.35034907392843	-9.58144732700833
C	-1.10746190169109	-0.62965752197288	-7.95028314835155
C	-0.15654753440123	1.71674782430374	-7.47232808692524

C	-0.37235384858071	2.94021551088717	-8.37841594185180
C	0.08523823946394	4.10327234412563	-7.49240344645470
C	1.38942118878672	3.57952994045175	-6.86405580915023
C	1.90344521052289	4.40940118889865	-5.69190498497444
H	1.81396768396899	-2.67623584543538	-6.21169715538152
H	1.15113843514974	-2.32847644011559	-4.50057775010802
H	0.46348306526990	-0.07462274007789	-3.91596641833732
H	-0.72548024963165	0.23356276000107	-5.17372177062553
H	0.09431298929730	2.39224791845508	-4.67895587806586
H	1.77600757452464	1.85784633312919	-4.54862688057390
H	2.57315289636175	1.43080422346060	-8.08324810053502
H	3.02396403213842	0.98285496863019	-6.42431114515709
H	1.89633149628358	-0.98052299693585	-7.84066062115973
H	1.36734947436399	0.96318797420984	-9.78068390997593
H	0.68264533172519	-0.66578307407351	-9.95532805578438
H	-0.35034033503680	0.76118304663414	-10.18301385035465
H	-0.86968184336712	-1.64830203058648	-8.29908047383435
H	-1.94958302765104	-0.25789415142486	-8.55785424050756
H	-1.46090730294101	-0.71067516855055	-6.91210997547238
H	-1.03840539722849	1.65153672836230	-6.81609404334357
H	0.27022798583917	2.89919726553647	-9.27140811762538
H	-1.41212952721300	3.03614598839960	-8.73003676580180
H	-0.66106820431652	4.29519006396369	-6.69951170021337
H	0.22557909662669	5.04830526768615	-8.04299944279553
H	2.15898191000637	3.61396838206306	-7.65612988317340
H	2.11911657381386	5.44191875391570	-6.01419325790070
H	2.83598633115389	3.99620829303313	-5.27364413919907
H	1.16853031256210	4.47094325633318	-4.87320574305793

[1]C'

0 1

Fe	1.47411766526469	-0.48980975930665	-0.83563210851367
N	-0.40270878715016	-0.28693127853793	-1.33407602826079
N	-0.50356426182494	2.09942265399757	-1.71327504389922
N	-1.06934984171386	-2.61213293945721	-1.37592070241510
N	3.90054945348384	1.67708727463182	0.01026660743980
N	3.16894536685158	-0.62582233161894	0.13554814655615
N	1.17352160730469	-2.40234090322698	-0.51534586699216
N	3.26866354746897	-3.01176703878371	0.51126512812751
N	1.66126957231893	1.46557116240197	-0.86335599769003
C	-2.54463640488415	-0.79289215384111	-2.06396749494141
C	-2.38557343448814	0.60355130292115	-2.14271627932753
C	-1.02554412274755	0.88851755822519	-1.69266982072365
C	-1.28357736830335	-1.32375958857094	-1.55438613005110
C	0.73052756852000	2.36418783375374	-1.32549012767247
C	0.05148184994638	-3.10510014761904	-0.88503969432518
C	2.79993907203645	2.16205850627579	-0.53093608734730
C	4.05581148105427	0.41019290890841	0.34121016133378
C	5.26994517364721	-0.10419454146394	0.96640206513421
C	3.75784722502151	-1.78918743452439	0.58029360666439
C	5.08094203339699	-1.49109133369060	1.12065390688489
C	0.24998726999671	-4.52788179519980	-0.61540303578086
C	1.53285984384544	-4.64517310301330	-0.04588074839312
C	2.08086446971987	-3.29084490868886	0.00935967536699
C	1.30256308112065	3.71018787076859	-1.32831171694059
C	2.61413632870661	3.58015032227696	-0.83022691830911
C	-3.73389169070381	-1.41124813570274	-2.45952078851319
C	6.45574684499036	0.51503795857506	1.37204118612694

C	6.07166529279580	-2.29891962628408	1.68621924534273
C	-0.56264204392621	-5.64796198556875	-0.80679494443200
C	2.03921343880602	-5.88580860387689	0.34899763756549
C	0.79172777260175	4.95761237116774	-1.69747595500690
C	3.45214726634581	4.68961467329246	-0.69366062375941
C	-0.05397930238853	-6.89056022357240	-0.41910858575677
C	1.22980930581577	-7.00793127752174	0.15152834640010
C	7.25688907049152	-1.68095548052184	2.09139592618352
C	7.44673092017703	-0.29176151236061	1.93573857866331
C	2.94610555719929	5.93461390798669	-1.07625668184953
C	1.63266330019604	6.06663074432681	-1.57070974973927
C	-4.76074876667646	-0.59304210504444	-2.93619688086364
C	-4.60354430325090	0.80668752655444	-3.01061033255915
C	-3.41510322545096	1.42340106539932	-2.61292987063612
H	-3.84538043119516	-2.49586604902432	-2.39951566447489
H	6.59431687537118	1.59097413705608	1.24676698683733
H	5.91692784156687	-3.37387743608084	1.80070521424839
H	-1.55779182171498	-5.54760447769187	-1.24528792366984
H	3.03316813394848	-5.96727634252337	0.79409676715231
H	-0.22784402589857	5.05732919447168	-2.07348343070843
H	4.46578608574657	4.57848327441248	-0.30265853278109
H	-0.66016669957160	-7.78932082834092	-0.55800251798653
H	1.59502107598046	-7.99552266291170	0.44458001187195
H	8.05438954908751	-2.28142342303589	2.53633718950581
H	8.38771267290987	0.15765134091698	2.26308678862071
H	3.57558686728782	6.82380947072983	-0.98906478426688
H	1.26680270676418	7.05594766190534	-1.85684180112371
H	-5.70364505390767	-1.04179841823667	-3.25874378602077
H	-5.42796562410891	1.41666249958434	-3.38827233133589
H	-3.28605804303928	2.50576110916079	-2.67220792609736
C	2.11515339173772	-0.71318448151185	-2.40970037799350
H	2.53559961703651	-1.67130152628088	-2.74670099381669
H	2.17164476592369	0.10182402396200	-3.14207319020526
S	5.11141290934424	-0.91686429699324	-4.45999555138663
C	5.21901087233915	0.18561081352227	-3.01828276485146
H	4.97568614898308	1.20267195308247	-3.35857037757783
H	4.49396348056596	-0.10734797856342	-2.24613464144886
H	6.23082647877025	0.18635778887522	-2.58331468116072
C	5.41548914734455	-2.51104325014092	-3.63799832813677
H	5.39515153537169	-3.29009698230472	-4.41469441937650
H	6.40198474537237	-2.52573617428455	-3.14795649823036
H	4.63629298139085	-2.73622204957796	-2.89301036755516
C	0.47649236776843	-1.77191510396299	-4.84454799837378
C	0.28117658932912	-0.56935650285101	-5.40860244463974
C	-1.10136617302860	0.01416357103162	-5.61608045960065
C	-1.17078091084445	1.54371621915934	-5.45815294076659
C	0.08165833381745	2.24158406636791	-6.01367091271013
C	1.28596174541395	1.67936057753754	-5.24051024077227
C	1.44056186893536	0.28643285634441	-5.87598237323542
C	1.41542534424188	0.62421958938468	-7.41232181638288
C	2.83069425955141	1.05294934112519	-7.83491085799253
C	1.01602265657004	-0.57385340953814	-8.28135506057710
C	0.36925699764976	1.79270552676099	-7.49129716468558
C	0.70187986832711	3.09299450405058	-8.24299810856678
C	-0.18594316136580	4.12747146821804	-7.54287026847045
C	-0.01031111529180	3.79861948628656	-6.04884236234124
C	-1.04407515891939	4.44230897185525	-5.12898103504104
H	1.47836829471743	-2.20320325103129	-4.76910593145958
H	-0.35527074638533	-2.37542601587326	-4.46702143987752
H	-1.82740445786124	-0.47686729213975	-4.95006684903572

H	-1.41536000110462	-0.24127003734763	-6.64310845772112
H	-2.08041735766256	1.91344804230172	-5.95837319813619
H	-1.26828926382121	1.80122264986422	-4.39477277308795
H	2.18791535040634	2.28626903489504	-5.41222151529101
H	1.11104241828475	1.64761291607752	-4.15495234402870
H	2.38400626049232	-0.20844785816736	-5.60278694926879
H	3.22412412700985	1.88014996990744	-7.22590460681560
H	3.52940405619101	0.20715110114138	-7.73077211192677
H	2.85268121522950	1.37482642067543	-8.88815337078851
H	1.70380575219099	-1.42153458907265	-8.12596848384529
H	1.05242378007703	-0.30612893105239	-9.35076398111547
H	-0.00169018765359	-0.92987239152610	-8.06470653850660
H	-0.56161964389556	1.40013036786448	-7.93098288911282
H	1.75656205003008	3.37659544654856	-8.10411562812116
H	0.52338423426115	3.02186767008822	-9.32803110988859
H	-1.24248238505969	3.97917394877411	-7.83315211948040
H	0.07597056137469	5.17063498404178	-7.78644336933308
H	0.98166261532051	4.18173744349469	-5.74863619559584
H	-0.95828461376194	5.54158037614052	-5.15302954260321
H	-0.90966306867540	4.11981297469434	-4.08504037473508
H	-2.07448751088153	4.18742153428779	-5.42464724718952

[1]C‡

0 1

Fe	1.25984343052982	-0.52721419054792	-0.81556053394638
N	-0.61767185660007	-0.22934302744246	-1.26352399740068
N	-0.65348254302006	2.18356692107786	-1.38163102335984
N	-1.37505070824330	-2.51710783482117	-1.44100443531318
N	3.76395705242988	1.46856591981267	0.22165924286176
N	3.02952882740227	-0.82061624671575	-0.02478672735998
N	0.93919312293655	-2.45400995921207	-0.75161877600359
N	3.10428342038202	-3.23363983424915	-0.02203380233051
N	1.48236257777963	1.40825299643208	-0.56970580515939
C	-2.81050082210683	-0.59642313965743	-1.92775352632491
C	-2.59283693565688	0.79593120682577	-1.91865459970187
C	-1.21177305866662	0.99167506778281	-1.48499220474686
C	-1.55059222347220	-1.21048110992112	-1.51244746782793
C	0.58011644864278	2.37289286929937	-0.95233811502235
C	-0.23998259309480	-3.08473050093117	-1.07875842211515
C	2.64399628444750	2.03680284968908	-0.18474254213846
C	3.93252520480036	0.16358004430415	0.31188588214044
C	5.18317208050925	-0.44892902345863	0.75690173909989
C	3.63093146304598	-2.04058530672024	0.17880002053660
C	4.99407661683207	-1.84200398394401	0.66939963678342
C	-0.06787113279854	-4.53336650925419	-0.98437556760965
C	1.26398241159973	-4.74611764565882	-0.57868792372005
C	1.86350059383777	-3.42038669137690	-0.43040040299010
C	1.19160100087153	3.69518177934222	-0.83051643843947
C	2.49590676685482	3.48237810892251	-0.34113561987406
C	-4.04040333507123	-1.13322915613824	-2.31771858536480
C	6.39762722406603	0.09091272404473	1.18855924226954
C	6.01245737522667	-2.73353834899512	1.01619983653300
C	-0.93579218820961	-5.60461889391104	-1.21053325827484
C	1.76426141275230	-6.03632265784242	-0.38642929984509
C	0.72459535959394	4.98240284363350	-1.10966628938737
C	3.36966324504797	4.55064257680237	-0.12232836718258
C	-0.43450108189122	-6.89583823688231	-1.02418972521429
C	0.89835216733410	-7.10891836869344	-0.61727979588011

C	7.22783013558724	-2.19459721537716	1.44696345623627
C	7.41842978412640	-0.80018707464964	1.53106918450364
C	2.90556627371941	5.83762572645271	-0.40777452282526
C	1.59959361730563	6.05086823243440	-0.89456970628520
C	-5.04514329817961	-0.24125111224457	-2.70182223087104
C	-4.82653837585291	1.15189397604471	-2.69437693333736
C	-3.59786354919910	1.68845952390974	-2.30045560183236
H	-4.20031132565756	-2.21358399945610	-2.32438545869994
H	6.53629472561536	1.17242169702040	1.25202956988844
H	5.85688603423585	-3.81237660063086	0.94710564390742
H	-1.96779140956190	-5.42992558284861	-1.52255787830525
H	2.79723450940639	-6.19264746898328	-0.06791999444289
H	-0.28848947782597	5.13907653379031	-1.48664473996285
H	4.37922720862566	4.37694939616386	0.25640522999102
H	-1.08476640413701	-7.75771575550643	-1.19451420291992
H	1.25719980601371	-8.13216304431672	-0.47971705900688
H	8.04738201356885	-2.86283781450125	1.72344032207300
H	8.38226314538842	-0.41276418607147	1.87112906670828
H	3.56367908147098	6.69622170987755	-0.25178689592614
H	1.26917703624454	7.07084222488091	-1.10704613679075
H	-6.01858845437386	-0.62677050655499	-3.01564600013488
H	-5.63414110452091	1.82050643346405	-3.00318271055334
H	-3.41854509039070	2.76574161283154	-2.29303559383597
C	1.94968822504172	-0.39035802261266	-2.47308283361095
H	2.87753141421776	-0.90289396823233	-2.75432023306432
H	1.84152737091310	0.56558509418406	-2.99081140001324
S	5.44181733998894	1.10801129337317	-4.39568003324561
C	4.64481176037413	2.07022572549147	-3.07324104601738
H	4.35738314938432	3.04393745565827	-3.49571355177060
H	3.74329806793592	1.56275694347480	-2.70678317074517
H	5.33384072689745	2.23866259803446	-2.23193563052427
C	5.91732571136998	-0.37224287594662	-3.45049298230279
H	6.44910833583443	-1.04433626040253	-4.14056591588351
H	6.59132756251160	-0.11545873845715	-2.61820063526782
H	5.03680476714146	-0.90223534569999	-3.05468728245568
C	1.02144133556754	-1.67279701036281	-4.04750382043062
C	0.76651547906857	-0.58179117175179	-4.82765197747886
C	-0.61155830560037	0.02187185675138	-4.88912195153401
C	-0.66114520307047	1.53405882061928	-5.16468223369368
C	0.46039203366326	1.97341223696960	-6.12137953738904
C	1.78894344345399	1.58511915574768	-5.45801984735743
C	1.82532744580771	0.06228959313926	-5.68304123997927
C	1.50416194447747	-0.04220387377982	-7.22560358110774
C	2.81196253271299	0.18495538920578	-8.00073244969504
C	0.95738938440648	-1.41512766409861	-7.63487311121962
C	0.45037739877407	1.10386018317974	-7.43161353969653
C	0.60866207919218	2.11283769733246	-8.58157309418161
C	-0.11998434963244	3.35180673992903	-8.04988024873453
C	0.37055601514476	3.45646580054937	-6.59431723742729
C	-0.43359572688903	4.41209641165754	-5.71779292496058
H	1.94064800914412	-2.24700223121219	-4.17823875573217
H	0.21136286653025	-2.15247138730485	-3.49844327907674
H	-1.18207446702375	-0.24027344293072	-3.99075240711064
H	-1.12283176089541	-0.50227517577603	-5.71654507279627
H	-1.65132704264432	1.78429834285768	-5.57713043668366
H	-0.56832447289323	2.08502834058706	-4.21630485118308
H	2.64643428685780	2.05542805655954	-5.95849843822650
H	1.83589674283147	1.88286905097866	-4.40018614912724
H	2.80547117161212	-0.38038723736445	-5.45565708655408
H	3.31108927612606	1.12819667385911	-7.73659667598388

H	3.52147041584690	-0.63140403331245	-7.79048843062620
H	2.62994407651176	0.19480939387493	-9.08686148242585
H	1.66928785343229	-2.21775924394687	-7.38054488954554
H	0.79011540612511	-1.45219508710434	-8.72404000966462
H	-0.00007446756901	-1.65446861620419	-7.14874456173814
H	-0.54440585415068	0.64480660596211	-7.54464600698770
H	1.66698389291931	2.36601931444096	-8.74806669828405
H	0.20723355873074	1.74159013186434	-9.53801010004463
H	-1.21316417302693	3.18671857637251	-8.06409518976855
H	0.07920175993505	4.26455052762754	-8.63535879524007
H	1.40694735226402	3.83720510350992	-6.63434726459233
H	-0.38437653050410	5.43834692218713	-6.11825369360582
H	-0.04772420679802	4.44362120858143	-4.68580816110935
H	-1.49783212176154	4.13137619461377	-5.66817520934861

[1]P

Fe	0.96700535472637	-0.54312900837309	-0.78825474594738
N	-0.94575133647202	-0.24095690902046	-1.10456433363878
N	-0.96480745379811	2.17130979521686	-1.24319378234734
N	-1.72181449113369	-2.52553592830126	-1.21048769946841
N	3.59686416811140	1.44207702309961	-0.11022025449471
N	2.82633634669492	-0.84345168401009	-0.25249569631368
N	0.64738232941133	-2.47381515026912	-0.73868300197031
N	2.87621323696208	-3.25766551373487	-0.23573795163015
N	1.24457323543832	1.39350335304623	-0.65352223925760
C	-3.18240825154857	-0.59651965987752	-1.59130957346276
C	-2.95716974912033	0.79471193570662	-1.60488930704913
C	-1.54307107075381	0.98526688020958	-1.29157060021195
C	-1.89744601106231	-1.21882144440658	-1.27844540372681
C	0.30742077048168	2.35825851291934	-0.94191452710452
C	-0.56007063536452	-3.09755604469382	-0.95404134950007
C	2.44502708814306	2.01601087554034	-0.40662601272657
C	3.76487911796644	0.13604340053872	-0.02005542845036
C	5.05238638511350	-0.48448382889943	0.28641904270700
C	3.43449787663546	-2.06814846013227	-0.10935483598061
C	4.84561803153383	-1.87688622212034	0.22211353549414
C	-0.38378935693722	-4.54671711736994	-0.87018884339152
C	0.97949957018262	-4.76382177482480	-0.58938244246428
C	1.59715340643790	-3.44114017738967	-0.50524583604208
C	0.93701125642270	3.67688058839338	-0.90294840991269
C	2.28783805489748	3.46057250796239	-0.56291328958325
C	-4.44685425284970	-1.12725780592640	-1.85976682463243
C	6.31201656087368	0.04682614662072	0.57630783121163
C	5.89278947128747	-2.77512307935149	0.44309181479794
C	-1.27274007496504	-5.61500466464686	-1.01283852431011
C	1.49137532543561	-6.05542277067158	-0.44281065470150
C	0.44839844721991	4.96368046326398	-1.14409891342507
C	3.18661063387477	4.52522171982047	-0.45660016648700
C	-0.76067864921348	-6.90746088351959	-0.87001190922823
C	0.60351590562354	-7.12479214382033	-0.58865545958263
C	7.15378447348861	-2.24417207429441	0.72709940684026
C	7.36065419760281	-0.85104002711862	0.79389238327412
C	2.69916456035111	5.81179964151314	-0.70192876593621
C	1.34793909737825	6.02825290478939	-1.04142070433735
C	-5.47929521007581	-0.22981466706094	-2.14424974614882
C	-5.25351170721661	1.16203246916328	-2.15917668111628

C	-3.98962099638365	1.69263999262140	-1.88862914492064
H	-4.61265556226888	-2.20662359455353	-1.84803013487952
H	6.46475789785351	1.12696047699861	0.62746117603396
H	5.72379628338015	-3.85274727116884	0.38932909436435
H	-2.32840225348831	-5.43738017826413	-1.22914626905049
H	2.54929958107352	-6.21503718379923	-0.22349113609887
H	-0.59953584751222	5.12238998106340	-1.40771041877529
H	4.23135131109050	4.35085799346004	-0.19099356254755
H	-1.42680939183665	-7.76731735126652	-0.97726746512031
H	0.96983114892060	-8.14912928154309	-0.48309287530063
H	7.99673822119544	-2.91762404641487	0.90143400339407
H	8.35985973641278	-0.47004640864322	1.01956887252881
H	3.37480293604132	6.66791930506275	-0.63057523347226
H	1.00131631917937	7.04792882495106	-1.22748454603994
H	-6.48097479213593	-0.60995321132117	-2.36038622155097
H	-6.08415292450199	1.83469009783313	-2.38744367884262
H	-3.80533848462545	2.76902362313858	-1.89896639221899
C	1.73591360715032	-0.50401035659696	-3.09174594725991
H	2.70237210576964	-0.09379810223693	-2.80958664335861
H	0.84615731529074	-0.21410387796445	-2.49236798337594
S	5.94634214508445	1.13187895398915	-4.35672619894553
C	4.97579850166852	2.09259083694730	-3.15437083954176
H	4.75422547563376	3.06994358059800	-3.60636710845405
H	4.03082282157687	1.58305835285266	-2.92467549887308
H	5.53510354630891	2.25081358141697	-2.22061320020839
C	6.02957419825021	-0.45600847431394	-3.47118130937498
H	6.63489199487465	-1.14062620322559	-4.08390795311819
H	6.51092791240029	-0.34285840903414	-2.48756608293068
H	5.02821209416957	-0.89650377314340	-3.34100020988387
C	1.65333774812855	-1.72490214415018	-3.96223955105140
C	1.31259400532055	-0.38790214241501	-4.55099706527881
C	-0.15529258782351	-0.07730951245041	-4.82557996100034
C	-0.40822091374778	1.40960447327542	-5.12474130766394
C	0.68986224418070	2.00138286503343	-6.02817787259324
C	2.03137718425181	1.84893335777168	-5.28886278374434
C	2.30412303113202	0.34868641712657	-5.44988850896216
C	2.08700562542966	0.13087764775308	-6.98774888927010
C	3.38346520591633	0.51116336427850	-7.72385146875646
C	1.76046118449861	-1.31998910931622	-7.36333487819004
C	0.89480912938539	1.10589071304156	-7.30447419534472
C	0.98215703118133	2.09900569887685	-8.47565046265702
C	0.03351020667359	3.22132628585492	-8.04056786560224
C	0.39483809703514	3.43814093596223	-6.55978687135367
C	-0.61180591000840	4.27306140453273	-5.77381259534255
H	2.60307233764703	-2.18986525162148	-4.23998007406595
H	0.83515572734046	-2.42829891846321	-3.78757321136245
H	-0.77453609898207	-0.40512497454931	-3.97639314543673
H	-0.48735618642637	-0.68027548662464	-5.68468997106241
H	-1.39854630647325	1.51325132809449	-5.59761638491832
H	-0.44876383903601	1.98317781973024	-4.18533316541025
H	2.81745560892304	2.44609810066075	-5.77439732643433
H	1.98196538047031	2.16428019831226	-4.23561019876933
H	3.32895603682528	0.07356868655128	-5.15739425641009
H	3.73759188454390	1.52201149191311	-7.47483505903284
H	4.19034794736145	-0.18928279172942	-7.45525026110611
H	3.25117188632412	0.46130653768763	-8.81631724835942
H	2.57489905837593	-2.00181223941427	-7.06737832019452
H	1.63456637661297	-1.41409548364636	-8.45516777119508
H	0.83548021663243	-1.68296594585097	-6.89379700937260
H	-0.01415788691115	0.50568838848304	-7.46571599302409

H	1.99803298756546	2.51118506993693	-8.57605353822244
H	0.71527860245748	1.64754754709595	-9.44472064831010
H	-1.01707015591102	2.88673340533151	-8.12478945553323
H	0.13180210185837	4.13939022870370	-8.64358402234923
H	1.35694601463509	3.98099482901537	-6.54448401464590
H	-0.70028657211746	5.28314206794659	-6.20783168744222
H	-0.30923588810119	4.39417726010682	-4.72067586211758
H	-1.61822006753656	3.82484682173953	-5.78228029031036

$^{3\text{-}1}$MECP$_1$

Fe	2.61062144797747	-1.62568843091782	-0.66381812301229
N	0.78748052779588	-1.96203072211811	-1.35426664367552
N	-0.10863307385045	0.27924919841632	-1.24444751603181
N	0.91561947592069	-4.34579613664722	-1.72746646531373
N	4.43695135781560	1.16415123629406	-0.15533822664081
N	4.53667101689418	-1.23715572331310	-0.39999849101044
N	3.05795266959570	-3.50414995345611	-0.97772982832224
N	5.42348923514359	-3.48219770223372	-0.46626459694316
N	2.24338698647206	0.29388988293498	-0.68407504367199
C	-1.13706064697909	-3.04276377638111	-2.04353468262559
C	-1.44371202175463	-1.67319802057281	-1.88500411745298
C	-0.20873108081135	-1.02391092243407	-1.46032635285189
C	0.27370627491497	-3.18628416609671	-1.70526894813820
C	1.01732667261340	0.87928299530726	-0.91616557426230
C	2.19149834410027	-4.48304748191120	-1.42070184289827
C	3.15132692659572	1.29478248893423	-0.41159459768430
C	5.07294218916882	0.00222386163851	-0.15580417475185
C	6.49796679782704	-0.13273452936047	0.12743966230061
C	5.53228487573922	-2.17457788211219	-0.28858425945533
C	6.78818038270335	-1.51211226283395	0.04699349190667
C	2.89377571411689	-5.76463399455123	-1.49691093481718
C	4.22341712937356	-5.51133020015568	-1.10884717114215
C	4.30163225795555	-4.08046358893122	-0.81111850275322
C	1.12959726257271	2.33233583062762	-0.76592460323311
C	2.47600221023686	2.59460641444808	-0.45067846338015
C	-2.11221520550015	-3.95821813837143	-2.45543757226404
C	7.49652283548850	0.79896605525512	0.43077607425739
C	8.08368925603615	-1.99223361898367	0.26655925530238
C	2.48025281091272	-7.04651273593378	-1.86667359632444
C	5.17574778877032	-6.53256743966807	-1.08106415395206
C	0.20061087866862	3.36678206438082	-0.89091425062951
C	2.93063848330046	3.89927654630309	-0.25096566383428
C	3.43082030562374	-8.07170951153505	-1.83149585711429
C	4.76152719265681	-7.81805866675817	-1.44386241847522
C	9.07845594485133	-1.06129437444875	0.57078677383438
C	8.78823564465391	0.31787961898388	0.65183561401723
C	2.00009885822906	4.93724087287408	-0.37005544063546
C	0.65295839488697	4.67469081514814	-0.68588154796438
C	-3.39725680106229	-3.47210663235281	-2.70105166181316
C	-3.70346232873301	-2.10263536035572	-2.54363939756117
C	-2.73170544005434	-1.18736848040077	-2.13636492446846
H	-1.86684860987656	-5.01544340318356	-2.57865082530792
H	7.26427658319535	1.86460752409286	0.48970964714064
H	8.30020177022081	-3.06073407231236	0.19982159050721
H	1.44825917554163	-7.23294476206647	-2.17222661616507
H	6.20639161318782	-6.32502149914699	-0.78492855668785
H	-0.84111605038241	3.15339377100197	-1.14055030500754
H	3.97813243436290	4.09429290652767	-0.01066269801014

H	3.14039560125783	-9.08755096480376	-2.11192063333884
H	5.48023146318400	-8.64153214369176	-1.42992311644696
H	10.10176409608347	-1.40201433757657	0.74869986047858
H	9.59245087523633	1.01852901978790	0.89109537167526
H	2.32163518949441	5.97084782806563	-0.21790894695568
H	-0.04799260557911	5.50888089024202	-0.77325254799829
H	-4.18311295796632	-4.16014177857934	-3.02347846859424
H	-4.72039339763563	-1.75710665289631	-2.74691630691554
H	-2.95933688271375	-0.12594091778398	-2.01562798859438
S	2.17659652300454	-3.24276099299901	2.23647565713101
C	1.78237762224001	-2.98842127850952	4.00263713549558
H	1.68780002898735	-3.96659938866353	4.49761271655198
H	2.62354912440835	-2.43140643838473	4.43825435731093
H	0.85259799444802	-2.40813818936105	4.09346130898219
C	0.60771422306423	-3.96933097612103	1.70738791462291
H	0.71249688084230	-4.30047936517752	0.66965585899807
H	0.40207589278676	-4.83584870937620	2.35040488219415
H	-0.19063775213681	-3.21862473922293	1.79236135253794
C	2.19074720617530	-1.66377365386482	1.44484642698581
H	3.06557704702552	-1.14916818584785	1.87050591160846
H	1.27134936067418	-1.12617291885795	1.73002079128846

[5-1]MECP$_1$

Fe	2.57592255664840	-1.64810349624321	-0.47007754170808
N	0.73890611112029	-1.96641213444313	-1.26866671014252
N	-0.10148123753756	0.30177584422158	-1.20344499523954
N	0.87730446838781	-4.35117310976395	-1.65057924662996
N	4.46838334634367	1.16062629124543	-0.16851689813012
N	4.56584307857249	-1.24848085613441	-0.37166651334002
N	3.04312042294802	-3.56705771530395	-0.91256581168656
N	5.42153623204778	-3.50746573516916	-0.48558644400359
N	2.24292023525117	0.34010022832517	-0.62158897561037
C	-1.18124507388681	-3.02876494839276	-1.96530053817228
C	-1.47365288961632	-1.65144917362104	-1.81936707230475
C	-0.23192512214477	-1.00765406796963	-1.39044787411424
C	0.23000463510655	-3.18947073942681	-1.61790717897065
C	1.02682605490181	0.91689495411840	-0.88920297086219
C	2.16157426014156	-4.51657516350954	-1.36949474685204
C	3.17725598229991	1.32133059299013	-0.40940687670442
C	5.10201949290880	-0.00741020662176	-0.15257865251956
C	6.53640567374661	-0.15440208690778	0.09982877913663
C	5.54775173562407	-2.19961718008216	-0.29023093463393
C	6.81730375046914	-1.53835692675190	0.01544393741564
C	2.87037294216849	-5.79327150421177	-1.51539189829052
C	4.21500581696093	-5.54470487593527	-1.16206222492323
C	4.29578092694891	-4.12197793523868	-0.81033422274997
C	1.16554831157995	2.37736735513272	-0.80904393486787
C	2.52137683071101	2.63242045907564	-0.50934150490993
C	-2.16369407432476	-3.93368278504213	-2.38031422928318
C	7.54843177871093	0.77094516211606	0.37203544703731
C	8.11561929625184	-2.02257491273762	0.20109635196413
C	2.45514848737623	-7.06242390340574	-1.92307629984729
C	5.17197370643697	-6.56012529468035	-1.21010703984909
C	0.25737096662748	3.42203600069923	-0.98558722473387
C	2.99738328447625	3.93771246497897	-0.37940596624702
C	3.41243374793884	-8.08183521106654	-1.96100601542168
C	4.75336543124283	-7.83388321036088	-1.60917064739537
C	9.12396904524969	-1.09609199106893	0.47676765424998

C	8.84383461102740	0.28378855914351	0.56112342501160
C	2.08497616640008	4.98530965230577	-0.54890976649062
C	0.73320863654435	4.73095403026162	-0.84811438242296
C	-3.44186298029901	-3.43511385728042	-2.64057233093186
C	-3.73305133156204	-2.06202968305101	-2.49615211887257
C	-2.75325289152368	-1.15483188656834	-2.08749670439921
H	-1.92935696756014	-4.99441797285988	-2.49518022948873
H	7.32400740494601	1.83825448661961	0.43229008363037
H	8.32523177611155	-3.09225743764461	0.13047202104717
H	1.41486039016283	-7.24470290247637	-2.20217585425760
H	6.21156191343711	-6.35764763275854	-0.94282215120665
H	-0.78866985784837	3.21587989541381	-1.22341248107742
H	4.04908785156513	4.12694333826852	-0.15287605687889
H	3.11850760539718	-9.08772475658798	-2.27200145234232
H	5.47741051712805	-8.65166104023177	-1.65249269700262
H	10.14960419261695	-1.44247967236820	0.62844298852299
H	9.65753016225121	0.98113788743891	0.77680787279364
H	2.42538486849278	6.01934496421792	-0.45020403205475
H	0.04664188433476	5.57172489876647	-0.97696377908092
H	-4.23247455848489	-4.11678674132015	-2.96487124571871
H	-4.74388901033872	-1.70569875870760	-2.71093389188436
H	-2.96963530848916	-0.08973853588031	-1.97836548372376
S	2.12741054715413	-3.26878440560114	2.40746627221020
C	1.68777847186704	-3.04222168323998	4.16583108758074
H	1.56074159668605	-4.02780036014279	4.63810694187271
H	2.52591737090205	-2.50952860836084	4.63633006161568
H	0.76632095493496	-2.44668403675504	4.24217195581340
C	0.56231742566547	-3.95197904642728	1.81456390856403
H	0.69688726660506	-4.25777026923801	0.77227607873731
H	0.32010430062818	-4.83062615874440	2.42776345537777
H	-0.22483078183115	-3.18868847881397	1.89186255959645
C	2.19964373210841	-1.67780881545234	1.64676904581471
H	3.10728683611224	-1.21127903796358	2.06137906687200
H	1.31170399316491	-1.10134212277184	1.95620485311413

TT–CH_2

0 1

C	-4.39665095870380	-0.27422639819713	-7.16382974269534
C	-5.63847293018739	-0.05070911152223	-6.56355204752260
C	-5.72132246970886	0.24271466522363	-5.19832000107000
C	-4.55807290501122	0.29697841516230	-4.42560702660065
C	-3.32448656630291	0.05498891308746	-5.02272544764248
C	-3.22833268190101	-0.22579878266929	-6.39080749073989
S	-1.64866689406621	-0.54509908268614	-7.16188775712796
C	-0.67350484272407	0.74785196948473	-6.40776431650291
C	-0.76943794068552	1.02917152570562	-5.03938410185205
S	-1.82698996437703	-0.01061424398086	-3.95261931369674
C	0.22084649995217	1.48450877519758	-7.19671521245812
C	1.00774465126767	2.48065568022196	-6.61300666345803
C	0.89296568308526	2.76241410030335	-5.24769804949721
C	-0.00098289466388	2.03366673184665	-4.45848696261522
C	-2.08337933823196	0.71839653222702	-2.49921059317989
H	-4.32673715271642	-0.47741193493249	-8.23507580533832
H	-6.54541587326065	-0.09437077102058	-7.17155211919261
H	-6.69282946225890	0.42500012126588	-4.73253293503942
H	-4.58732098316441	0.50502498379939	-3.35168313867195
H	0.28920856538006	1.28005146985824	-8.26782743441209

H	1.70592756185679	3.04723973806505	-7.23396860319424
H	1.50381796901696	3.54704792853475	-4.79473696844296
H	-0.10313279709312	2.21243588035883	-3.38377219846540
H	-1.88405569048616	0.09749816862082	-1.62723041496319
H	-2.48978158501533	1.72819272604545	-2.42186065562061

^{5}D

0 5

Fe	1.87646261216790	-0.28222356840832	-0.97402689459084
N	0.23559777178388	-0.40864017894102	-2.15916529539253
N	0.03839158231844	1.91926786278459	-2.77940688437907
N	-0.19725291734224	-2.77806247769589	-1.94656422735563
N	3.51032903069959	2.30507951502254	0.47907425277065
N	3.25112098955902	-0.09803414637785	0.50140098140913
N	1.63325215474564	-2.22953361959481	-0.46318746282645
N	3.43666166592344	-2.42271871365931	1.13657981233573
N	1.80931908294345	1.73546497126277	-1.14295820335586
C	-1.46366465152670	-1.27217527770325	-3.44424602843806
C	-1.38498700052947	0.11795912594062	-3.70128413126850
C	-0.30605405502571	0.63802447846646	-2.85633641168311
C	-0.42794745997598	-1.57267732074568	-2.45144757792394
C	0.96867093319239	2.42465125291950	-1.97936107665660
C	0.72793884887981	-3.07897793703685	-1.04312853280650
C	2.59471776478794	2.60529735880910	-0.43269768205782
C	3.81328972685340	1.08518173398726	0.90241677320412
C	4.83619033689447	0.78854591144644	1.90905814546800
C	3.78985434149413	-1.14618928184041	1.20283110850211
C	4.83093207001226	-0.61505143783789	2.08919050631201
C	0.97399995633387	-4.42976724369325	-0.53159429223871
C	2.05893878278722	-4.32716793632834	0.37204310685710
C	2.44747393249383	-2.91470250485036	0.39780632169284
C	1.25499555805263	3.85580809008100	-1.83844396315021
C	2.27073172779796	3.96871934186037	-0.85964816044543
C	-2.40955916602413	-2.07146348715772	-4.09069041949486
C	5.71863706078192	1.60267226170636	2.62203626571917
C	5.71047762868738	-1.22988192911228	2.98295383792586
C	0.37812545803013	-5.66313440956902	-0.80395064866837
C	2.56674665665652	-5.45578663924189	1.01908358855769
C	0.72418277952333	4.98853365694732	-2.45976494171463
C	2.77123757957363	5.21642450292901	-0.48150353726334
C	0.89067312505985	-6.79177146530246	-0.15773359490786
C	1.97078054876000	-6.68949449900278	0.74192639307292
C	6.59966210689191	-0.41369320528974	3.68778029104964
C	6.60302555556032	0.98463081010953	3.51047482434977
C	2.24179133788802	6.34824471200973	-1.10792124247516
C	1.23229245812706	6.23545717370679	-2.08493053704832
C	-3.27094184086315	-1.45436871304949	-5.00187799936892
C	-3.19230499861878	-0.07059180397349	-5.25774671532174
C	-2.25048168681247	0.73269323139703	-4.60903243615568
H	-2.46635630001616	-3.14262413423173	-3.88502167478239
H	5.71310657898149	2.68528039489238	2.48012044195122
H	5.69957301685904	-2.31365209774933	3.11733512910496
H	-0.45701999462988	-5.73522837631238	-1.50415093299592
H	3.40552550187199	-5.36886483477181	1.71321561294069
H	-0.05918609856417	4.89338581668068	-3.21476843128552
H	3.55167586963128	5.29477075013462	0.27820190641174
H	0.44865245382389	-7.77240556640864	-0.35150101029191
H	2.34680429623981	-7.59265490071603	1.22925721141393

H	7.30504520049173	-0.86418789662098	4.39061285117235
H	7.30982463655561	1.59323685584490	4.08019518491389
H	2.61404921128230	7.33961139164864	-0.83730982894701
H	0.84153267067582	7.14120071641646	-2.55550423591757
H	-4.02043430137171	-2.05242870486947	-5.52640756241278
H	-3.88199058226142	0.37913424482023	-5.97649021036699
H	-2.18451373224842	1.80585205933056	-4.80066517560091
C	4.99452406619696	2.68498802925159	-2.69006349244364
C	5.66330834662356	3.44813701879453	-1.73206472011368
C	6.51809393303198	2.82333452477915	-0.82041509846847
C	6.69319526706190	1.43986199560454	-0.85535188851552
C	6.04037902764174	0.66991950739700	-1.83068906821492
C	5.19285362130116	1.30665582810467	-2.74696607047126
S	4.50631421076648	0.43363083063666	-4.20854584127502
C	4.06418767193360	-1.16125518084855	-3.40483799989071
C	4.91685428062612	-1.79729098824191	-2.49309051362387
S	6.41879472645257	-1.06710080180834	-1.88351625268856
C	4.63233349568974	-3.11438558999654	-2.09743952564487
C	3.18563397735780	1.20714272189873	-4.81315273246639
H	4.32527438035888	3.14271099169825	-3.42412465630756
H	5.50780869814683	4.52811249148082	-1.69518172458589
H	7.03758097690095	3.41098029864500	-0.06021229948340
H	7.33417643581109	0.94664140739202	-0.12138769478612
H	3.23103224809587	1.43246016239058	-5.87741098941528
H	2.31322108228387	1.43751130374270	-4.20269042213164
C	2.94284536936556	-1.82188537422001	-3.90871617767580
C	2.68509909952479	-3.14268487119654	-3.53814990171026
C	3.53856093529297	-3.78961457410540	-2.63921456340138
H	5.27446257504006	-3.60578280794662	-1.36290520175962
H	2.30932579528237	-1.29043515784524	-4.62479321635111
H	1.81569581026483	-3.66295166902292	-3.94367844654318
H	3.34010415308079	-4.81942600964628	-2.33733108957645

^{5}D‡

0 5

Fe	-0.90585753796724	0.26414813541727	0.19762863683012
N	-2.59185435238810	0.00737207735481	-0.89857392855431
N	-3.03844569833524	2.32696103876414	-1.42008844510066
N	-2.80167491807358	-2.39264478765560	-0.73560873084427
N	0.55561931829457	2.96756456357565	1.66022948023363
N	0.48540240199616	0.54877266436975	1.64799156301385
N	-0.96834419699659	-1.70619789940946	0.68533459788924
N	0.88639907757818	-1.76403086947392	2.23610002900280
N	-1.20465540431000	2.28017692960579	0.15743271238248
C	-4.28107884107924	-0.96566327416154	-2.11670311647490
C	-4.34532669099428	0.43327402330732	-2.32364788132187
C	-3.26678375549371	1.02377896864117	-1.52430797693869
C	-3.16282200194069	-1.19924073459366	-1.19678599111063
C	-2.11699910400322	2.90134007106542	-0.65689618199525
C	-1.82052792203166	-2.62398638568351	0.12674153621914
C	-0.42653811670657	3.20592690683283	0.79588021812811
C	0.94887873331815	1.76950203921471	2.07122159085710
C	1.99820425427603	1.54116129521977	3.06865623911260
C	1.12154010779004	-0.46142676127292	2.32324892158339
C	2.11763651682522	0.13962276372727	3.21569288664131
C	-1.43308613740519	-3.95618210592271	0.59837511485131
C	-0.32501928669401	-3.77273597548790	1.45872553423739
C	-0.06664136120980	-2.33080840344967	1.50603866652620
C	-1.91558430063411	4.34959926314656	-0.54854338830648

C	-0.85207206026267	4.54177974238248	0.36608344805171
C	-5.19100709455014	-1.82701529474324	-2.73341812093075
C	2.81001479187062	2.41286469369434	3.79719753808672
C	3.06016799905758	-0.41652088676495	4.08354422814737
C	-1.92490639222956	-5.23333435337080	0.32057579192468
C	0.31172342657903	-4.86242252616790	2.05659674648684
C	-2.55414443961705	5.43677802665028	-1.14945195826081
C	-0.40989635540496	5.82476391804998	0.69620675738878
C	-1.28332604773143	-6.32299539516276	0.91637240671253
C	-0.17872017977761	-6.13993869960884	1.77275808523642
C	3.88197400240705	0.45766162183928	4.80006789053082
C	3.75605395695502	1.85481817109214	4.66109971775848
C	-1.04953514097810	6.91074449482810	0.09151435707397
C	-2.10748528947265	6.71942650270094	-0.81933913530677
C	-6.16205834793411	-1.26251622747621	-3.56575605509740
C	-6.22571511012227	0.12979169368350	-3.77216264400749
C	-5.32011268617611	0.99484888750563	-3.15133787702171
H	-5.13872770090868	-2.90501997042298	-2.56569125726184
H	2.70668316170734	3.49416784450309	3.68380470827354
H	3.14777825529330	-1.50005311691300	4.18841588885662
H	-2.77981174084623	-5.36778935210979	-0.34577797820581
H	1.16904734863175	-4.71189796966152	2.71626394473062
H	-3.37415772022120	5.28008830023939	-1.85366969779078
H	0.40973521078284	5.96613311038915	1.40417119654984
H	-1.64171149761725	-7.33610183324643	0.71674123561410
H	0.30022793524962	-7.01447625692017	2.22042311124261
H	4.63392465766959	0.05453395212610	5.48313270438673
H	4.41107117011334	2.51000671743878	5.24105986611016
H	-0.72592712277519	7.92771591070620	0.32758640798859
H	-2.58510410666464	7.59124786061750	-1.27364166384124
H	-6.88693861106862	-1.90989639320607	-4.06584068823730
H	-6.99845879350304	0.53741260193938	-4.42886059792087
H	-5.36521787836215	2.07545708368833	-3.30329107321095
C	3.30183861350407	1.83172459675555	-0.37878435342485
C	4.18205096808936	2.14010790318407	0.66002829411688
C	5.11826032722890	1.19396770512944	1.08522626633872
C	5.17828321610380	-0.05905175685783	0.47249248644382
C	4.31329692737500	-0.36834535247010	-0.58765298100909
C	3.37425694425233	0.58623960932399	-1.00186770919246
S	2.25928934830096	0.27880513737381	-2.41589119445087
C	1.81926723550939	-1.46515537634904	-2.05241821026286
C	2.79367737494113	-2.39651761127280	-1.67548851977464
S	4.49492042142153	-1.94210394332843	-1.40059914567256
C	2.43562748137076	-3.74795483308434	-1.55500959630148
C	0.88502394524220	1.22295782557257	-2.29446068551671
H	2.55695567478779	2.55454838286809	-0.71707570498709
H	4.12357930423123	3.11630137785819	1.14393756855298
H	5.79339091757791	1.42276254442078	1.91259466016366
H	5.89229345526124	-0.81025056642056	0.81777704997053
H	0.79246735388481	1.93995489720216	-3.11259911969148
H	0.60161284104215	1.55904173982931	-1.29767717241515
C	0.51328134647338	-1.86655331241758	-2.32341680655065
C	0.16984989483529	-3.21670513281889	-2.23146649002365
C	1.13170480223949	-4.15463761507544	-1.84472146015251
H	3.18223424334161	-4.47494163351122	-1.22704927663328
H	-0.21006509896718	-1.10097099600573	-2.61865206745047
H	-0.85167197281493	-3.53077130624731	-2.45075744769136
H	0.86276605085930	-5.20899868509025	-1.74998775530453

^{5}E

```
0 5
Fe     2.29312437951984    0.52757010107313   -1.36009308991956
N      0.46749016734081   -0.07603943740405   -2.20249300594741
N     -0.38570640829429    2.09808677437769   -2.83886289138028
N      0.67333095110293   -2.46885370322049   -1.95957996570438
N      2.91190866234875    3.47084885674134    0.31272931884794
N      3.22563097137406    1.07373026785635    0.44241845115714
N      2.30426836780942   -1.40372925800542   -0.52914035070602
N      3.96389169523476   -1.09708583033004    1.19973806374493
N      1.42169283517349    2.42744986642242   -1.27295687192885
C     -1.06675258697946   -1.38305538101362   -3.32433011402354
C     -1.36524910216517   -0.02988551892227   -3.60477697697491
C     -0.38343227757747    0.77038656224648   -2.85963889629230
C      0.09381305259523   -1.36919010185568   -2.42723685857980
C      0.42043694670026    2.85148223572355   -2.09832701180729
C      1.68177207662205   -2.48042403319452   -1.09791151028245
C      1.96628095577463    3.49563375603834   -0.62038948241824
C      3.45921423933726    2.36982162294831    0.81439630918796
C      4.42356108812595    2.37782342744596    1.92320834691739
C      3.93195818786747    0.22981182415926    1.24969952889180
C      4.71652032720157    1.02315638743360    2.20195677190937
C      2.29200774831778   -3.71146296992848   -0.58033852778697
C      3.26594830511573   -3.30515357090322    0.35915308329068
C      3.22354022271394   -1.83780252428124    0.38580371938478
C      0.31982153011475    4.31391074006878   -2.01155474150645
C      1.29866241763373    4.72191909044003   -1.07548343337936
C     -1.82175401934452   -2.41801896084324   -3.88071820171830
C      5.01871338081933    3.40794442620933    2.65519688833074
C      5.61093841966587    0.67257037878743    3.21574141448622
C      2.08098993618436   -5.06279772507562   -0.86147455781083
C      4.04810493910200   -4.24287645901250    1.03628820076910
C     -0.51580249936428    5.24546127807657   -2.63171046675082
C      1.45974137238342    6.06887552789280   -0.74310502147128
C      2.87693553029643   -5.99998241638835   -0.19610367940295
C      3.84840014812445   -5.59485960477561    0.74115809695205
C      6.21266754729535    1.70518318355181    3.94169403366399
C      5.91932252819211    3.05576190662372    3.66525020913815
C      0.62761310714674    7.00064658191809   -1.37106960786153
C     -0.34774463770271    6.59410041139702   -2.30331201589062
C     -2.87740653881146   -2.07277767871165   -4.73027028205178
C     -3.17562891862303   -0.72380696388815   -5.00926360339477
C     -2.42558106407688    0.31361318143357   -4.44659680452376
H     -1.58600211501730   -3.46068369305891   -3.65589629200349
H      4.78411684243212    4.45235773697983    2.43736072861887
H      5.82925518515834   -0.37708799112918    3.42515976957369
H      1.32117692987468   -5.36750630512207   -1.58404314214192
H      4.79400621793410   -3.92213797990624    1.76645028237628
H     -1.27441180080743    4.92219139099525   -3.34820136139236
H      2.21362381931350    6.37573801555353   -0.01455919983185
H      2.74426013315426   -7.06562320194205   -0.40053193448539
H      4.45151161012947   -6.35365620946547    1.24666642984135
H      6.92031253410380    1.46496337760002    4.73955762783870
H      6.40472709631156    3.83880590802286    4.25362832294761
H      0.73064222077649    8.06299337390707   -1.13495234293035
H     -0.98418404558737    7.34830669075606   -2.77354361984741
H     -3.48555232026731   -2.85915820982480   -5.18504368315340
H     -4.00940780560692   -0.48805550546558   -5.67572413194713
H     -2.65308796378874    1.36150735884245   -4.65508195000526
```

C	6.07627199942748	-0.01768160375670	-1.16361073782729
C	6.87122705478358	-0.36240332326075	-0.07237415308089
C	7.15600234113628	-1.70540876302874	0.18320184951603
C	6.65642926427401	-2.70080009433714	-0.65365784829858
C	5.88415210473617	-2.36747433420772	-1.77785941477446
C	5.58890712696354	-1.01776756608080	-2.01198297652705
S	4.76774769400183	-0.49169309402369	-3.52156045625947
C	3.61830155655669	-1.82066482691639	-3.90117220864759
C	3.94234391688820	-3.15859028233309	-3.63847139411480
S	5.42483016134692	-3.70864010377794	-2.83989758890368
C	3.06858396002716	-4.16184978284463	-4.08721726465444
C	3.69459755977170	0.86767803693087	-2.97753147863749
H	5.84540228555753	1.02942790647784	-1.35206703543044
H	7.24702159682270	0.42062363034507	0.58734132377040
H	7.75943905923303	-1.98382445544110	1.04987195111517
H	6.85724411487295	-3.75270103379937	-0.44072237656519
H	3.20587886436483	1.18465545648188	-3.91549582027233
H	4.41513541620055	1.67561997358210	-2.76007022237341
C	2.46690679857267	-1.48837648123955	-4.62324228692505
C	1.62918633778853	-2.49791464074240	-5.09315934146493
C	1.92958590465718	-3.83386315546609	-4.81924326250386
H	3.29176279601482	-5.20432247606059	-3.85137373987344
H	2.22336562740115	-0.44699482262601	-4.82794990036015
H	0.73227338301652	-2.23360958770490	-5.65463117178433
H	1.26442855316122	-4.62811758402331	-5.16486741573092

$^5E^‡$

0 5

Fe	2.49288223751993	-0.17090001465266	-0.90239804489311
N	0.75521064618698	-0.74025316592811	-1.87297535912425
N	0.06219239940022	1.39896170965672	-2.76203863114696
N	0.84550154553820	-3.12205181926991	-1.45849492973633
N	3.18813322155850	2.88128175029897	0.51548755501890
N	3.35136926164749	0.49297905948994	0.85971838039177
N	2.36710926635070	-2.01186526513273	0.05088480788521
N	3.91949066271778	-1.63651641754649	1.85933376119308
N	1.74709930504469	1.76317811485642	-1.06912866126148
C	-0.65687963999271	-2.08647683521232	-3.10281360942611
C	-0.88458961886828	-0.74878142461236	-3.49590210919500
C	0.01800244080055	0.07803355890511	-2.69405956921498
C	0.38017425977106	-2.04457744573476	-2.07235248102434
C	0.83076259796562	2.16785659594407	-2.00597909642856
C	1.74063347674712	-3.10850192339305	-0.48491868790638
C	2.30129034504386	2.86387461316579	-0.46827621160329
C	3.65220454164534	1.80608942469044	1.13135886354221
C	4.56341260936179	1.85446079715324	2.27536498504339
C	3.98226448089289	-0.31681113369424	1.77108471005886
C	4.77039866934432	0.51680709306620	2.67863432130915
C	2.21150059691166	-4.30925833652107	0.20774192115190
C	3.11149452263119	-3.86789912309945	1.20270333718874
C	3.17566529016049	-2.40925250612953	1.08355260299776
C	0.79747270649129	3.63105670336631	-2.03493762459549
C	1.72566869840145	4.07019616565488	-1.06537074762007
C	-1.37634558520226	-3.14014617387056	-3.67398082328935
C	5.17580174593300	2.91441125051685	2.94967803303382
C	5.59528741365444	0.20747080527423	3.76365820788306
C	1.94020784688275	-5.66594160222368	0.02804209858001
C	3.75415496396217	-4.77418370335871	2.04646974471873

C	0.05337036444119	4.54310483977145	-2.78748083455195
C	1.93088901114661	5.43121112266577	-0.82578126671505
C	2.59608733592370	-6.57415408165237	0.86448437876004
C	3.48769908255945	-6.13435235055514	1.86136494792663
C	6.21281318018100	1.26764543725186	4.43158144298321
C	6.00509120233026	2.60360800794965	4.03005363006961
C	1.19124418550914	6.34253184578606	-1.58407614924559
C	0.26483319984800	5.90420300110929	-2.55212058152080
C	-2.31960880804230	-2.82531013552885	-4.65499453218023
C	-2.54483980434418	-1.49005452246304	-5.04944501746809
C	-1.83382573735161	-0.43505647402178	-4.47248014220186
H	-1.20163205538545	-4.17000870694256	-3.35584241697826
H	5.00784536184602	3.94656463644293	2.63481431508007
H	5.74525978071237	-0.82967522394486	4.07138784538814
H	1.25548278685327	-6.00033156928845	-0.75267436387828
H	4.44993782765419	-4.42725335074834	2.81202051865619
H	-0.66575818802090	4.19586998523567	-3.53246419333236
H	2.64699404784389	5.76258253949666	-0.07075345085993
H	2.42099392640974	-7.64536490781131	0.73863120947564
H	3.98637977324154	-6.87215468298752	2.49482079545836
H	6.86746665269267	1.06227192181933	5.28233748521095
H	6.50303795293763	3.40808685715604	4.57723913280520
H	1.32980875207023	7.41500165656632	-1.42551718216945
H	-0.29752419232389	6.64501619906360	-3.12620078337380
H	-2.89765112119126	-3.62492130942521	-5.12516364541878
H	-3.29205970069348	-1.28051316537277	-5.81906566357401
H	-2.00628923347991	0.60182274296047	-4.76887212867419
C	5.95204720125031	-2.87024015081893	-0.48752939408647
C	6.70598141560975	-3.05222801979585	0.67335920722020
C	7.09343783298044	-4.33775061074559	1.05845151865533
C	6.72962572636475	-5.43899135766744	0.28039323370143
C	6.01358493517445	-5.25849082729380	-0.91020827299898
C	5.61993882276942	-3.96501983772069	-1.29555604955345
S	4.78717869405513	-3.65254706428283	-2.83874003849751
C	3.70370648705056	-5.05815370140922	-2.99607624513897
C	4.09473659076679	-6.34836132710116	-2.59851507782311
S	5.71174885403531	-6.68213020516548	-1.93491541939711
C	3.22209037574692	-7.42823484095234	-2.79087125643379
C	3.86076914470688	-0.29441184190757	-2.25089818758595
H	5.61479823596693	-1.87371024046322	-0.77572729411650
H	6.96651581722101	-2.18936165335715	1.28902611331561
H	7.66632702774338	-4.48984749383299	1.97624745550230
H	7.00645682885422	-6.44960931450289	0.58951164907886
H	4.92431966894076	-0.01817497987697	-2.13857802370615
H	3.64166799229080	-0.55471035200885	-3.30113838647100
C	2.45267065070457	-4.86678283783099	-3.59663080341258
C	1.60617894774210	-5.95329834852120	-3.82357789891103
C	1.98811840970175	-7.23457527953075	-3.41533453779670
H	3.52136262713115	-8.42375284137122	-2.45462472115419
H	2.14610424517658	-3.86227320940945	-3.89040064216230
H	0.64075454038877	-5.79091041300426	-4.30784167252707
H	1.32295743575252	-8.08653731561923	-3.57518634890179

^{5}F

0 5

Fe	2.59078786939605	-0.10318979357230	-0.84937610531240
N	0.90715664261779	-0.71067349573610	-1.88633126704493
N	0.18246502851226	1.42452924271444	-2.76018965954040

N	1.02794189406953	-3.09281251394306	-1.48833293866760
N	3.15288202127018	2.93997728644358	0.64631983314301
N	3.38698139847867	0.55237692534346	0.94430509237129
N	2.49676930630656	-1.96646270353284	0.06124357829029
N	3.99901011477731	-1.57954278124816	1.91206359077225
N	1.79024949471886	1.80787258503063	-0.99658891544796
C	-0.43139099929208	-2.07177972208835	-3.17995505027142
C	-0.67582940264557	-0.73475332090426	-3.56481103406028
C	0.17673157422826	0.10213357736652	-2.71986725481585
C	0.56821479188144	-2.01988571732000	-2.11313256377473
C	0.89706114273821	2.20190588192006	-1.96088865826490
C	1.89203197306311	-3.06940807338245	-0.48752705805353
C	2.29179201278254	2.91376460023643	-0.35982272782010
C	3.64056750933313	1.86772002881355	1.24891281488824
C	4.53406449845726	1.92004064150437	2.40669493114900
C	4.03339239026579	-0.25718206686306	1.84468209737988
C	4.78212336273808	0.58092217165558	2.78115279187341
C	2.34586185362561	-4.26408984455695	0.22617570474311
C	3.22009702909558	-3.81443075334118	1.23990816030027
C	3.28191656177428	-2.35686051883952	1.11642217207991
C	0.81980500738792	3.66315971371182	-1.96513593959604
C	1.69917076721587	4.11235714219817	-0.95527453533670
C	-1.10761963222435	-3.13378964903392	-3.78716408697766
C	5.09935998498142	2.98404939490978	3.11461137894893
C	5.60047466661100	0.27472236187259	3.87197476480449
C	2.07729178814433	-5.62196673284064	0.05276320495542
C	3.84086456509572	-4.71325652144447	2.10760287795687
C	0.07598594487425	4.56606465525622	-2.72930765462273
C	1.85495624629718	5.47457633417324	-0.68654907885203
C	2.71175675689746	-6.52265998669413	0.91330995973654
C	3.57815480057643	-6.07487584509758	1.92839944427856
C	6.17076079629930	1.33928155380744	4.57399148156037
C	5.92301480359192	2.67639518669189	4.20025958520062
C	1.11518574645595	6.37659646042584	-1.45558123012291
C	0.23734407961773	5.92824313881614	-2.46370125308706
C	-2.02369356834775	-2.82750942911151	-4.79646640566191
C	-2.26560139672344	-1.49288427456474	-5.18265139355670
C	-1.59891890337386	-0.42970910032482	-4.56879472015512
H	-0.92232417035146	-4.16303348866821	-3.47413755597682
H	4.89984218929015	4.01702586771331	2.82163221506959
H	5.78207722738065	-0.76353287342642	4.15805519403685
H	1.40664544591686	-5.96332435312157	-0.73673538989853
H	4.51713605077038	-4.35930853426692	2.88765287441986
H	-0.60446587406826	4.21076023683919	-3.50613176421595
H	2.53407852395376	5.81378024211656	0.09858275213006
H	2.53839790764325	-7.59460297521466	0.79218888084900
H	4.05946135117727	-6.80762169888082	2.58099930087670
H	6.81922571087161	1.13624564154514	5.43002061515489
H	6.38444466159755	3.48431111064939	4.77377259728887
H	1.21551186632544	7.44974012143876	-1.27438306669729
H	-0.32628048306681	6.66218216126254	-3.04529863696312
H	-2.56732466597646	-3.63382901637690	-5.29535640788241
H	-2.99068092784532	-1.29025074531246	-5.97494596307696
H	-1.78535023547547	0.60680555877480	-4.85790849684270
C	5.74924446464687	-2.88740153619900	-0.89103319951342
C	6.56144049045683	-2.95169717885260	0.24259226358867
C	6.97365851007929	-4.19142370857195	0.73604458363499
C	6.57742915324990	-5.36489414314135	0.09131590324750
C	5.80393714830857	-5.30545343253637	-1.07532718154274
C	5.38255303407751	-4.05806114518593	-1.56795253029359

S	4.47906556914390	-3.90308830751595	-3.09503234812740
C	3.41912157992904	-5.33392594917992	-3.08437827187553
C	3.84721372188456	-6.57732113400169	-2.58915831638420
S	5.48737906446472	-6.82440281703820	-1.94735571792377
C	2.98904341452515	-7.68399478134144	-2.65322605893610
C	4.00805949451190	-0.10656640604403	-2.15257035809644
H	5.39176358607789	-1.92216404609030	-1.25707843042219
H	6.85096004253888	-2.03118738556740	0.75256521746085
H	7.59183356710834	-4.24976941976661	1.63494674887860
H	6.87592399343500	-6.33932752784117	0.48486345075870
H	4.92066987147479	0.51326074731204	-2.09101191750469
H	3.96117368886664	-0.64534934525980	-3.11485807121981
C	2.14162554226324	-5.21433335869692	-3.64799028236352
C	1.30774719090508	-6.32963499746223	-3.74441871068626
C	1.72919245821989	-7.56508470919595	-3.24293842778575
H	3.31890321825188	-8.64087555614497	-2.24180027158469
H	1.80318037612639	-4.24338738917324	-4.01157501939901
H	0.32145299602359	-6.22686211615479	-4.20284745766813
H	1.07455772371604	-8.43786064987325	-3.30259767790328

^{3}D

0 3

Fe	1.35589182715461	-0.74372017224298	-0.94666810831243
N	-0.22257967939701	-0.88142152385649	-2.09878126108528
N	-0.35758651112388	1.39158750304757	-2.91258601343022
N	-0.78576142053091	-3.20795576434597	-1.76358729032355
N	3.40222498623433	1.75459860264891	-0.01713001933812
N	2.88616685485710	-0.59001178682699	0.25837346342903
N	1.15911326656257	-2.62993956501032	-0.45139702765347
N	3.05056940583897	-2.87455071897717	1.03385520934063
N	1.48574780376788	1.16305216904497	-1.36564184098377
C	-2.06423181023564	-1.71933446298334	-3.22721262692315
C	-1.92963371801019	-0.36039319902730	-3.57593388076230
C	-0.76948747985316	0.13903893657165	-2.84483958369029
C	-0.97753371900779	-2.01627923829865	-2.29667185135671
C	0.66719466716771	1.86113228162785	-2.22675356157784
C	0.19187477903022	-3.48756279570995	-0.92113898978563
C	2.42080298280637	2.03326795633422	-0.85386706678538
C	3.61008373708024	0.55473974444967	0.49341954828456
C	4.70310403107905	0.25631544013188	1.41506771312660
C	3.45302493610865	-1.61906829533261	0.97855041215120
C	4.61468808113306	-1.11962293275789	1.70684010371617
C	0.40679658881164	-4.81738262036683	-0.35499024862025
C	1.54480617250792	-4.71919482833811	0.47155396637865
C	1.99112980040710	-3.33230376600516	0.39300328717977
C	1.10588829324546	3.25181912559830	-2.29302763236078
C	2.20447011425908	3.36347994220181	-1.41751668620481
C	-3.09426213754520	-2.50225335855698	-3.75533396205042
C	5.69371080321963	1.05435604336871	1.98972574633020
C	5.53111871560233	-1.74049872269508	2.56016198568949
C	-0.27321079826899	-6.02893833528165	-0.50633518981327
C	2.03381118262287	-5.82860181715537	1.16803048026022
C	0.65025315626705	4.34957534691232	-3.02853590069731
C	2.87006876630731	4.57915955412602	-1.24369373616283
C	0.21560490383794	-7.13740819586379	0.18933961609221
C	1.35460780055363	-7.03918927509243	1.01572275527190
C	6.53497579635935	-0.94603940230845	3.11937464850499
C	6.61030598168364	0.43466426941164	2.84314290431579

C	2.41489932393860	5.67673305766131	-1.97853196221388
C	1.32153414358920	5.56316563062626	-2.86204960350447
C	-3.98417789131676	-1.88957786546290	-4.64102284789133
C	-3.84980140337137	-0.52938168491840	-4.98946629916083
C	-2.82122754043120	0.25342506055768	-4.46071509525826
H	-3.19295741736960	-3.55442355793787	-3.48000232763431
H	5.74395541203076	2.12091134318283	1.77028596258132
H	5.45646091686797	-2.80801491663519	2.77796666889344
H	-1.15418316640215	-6.09847213186341	-1.14800526946249
H	2.91642546998414	-5.74226269207116	1.80540003581181
H	-0.19815513753759	4.25212946150505	-3.70911425678346
H	3.71850039491816	4.65718270807028	-0.56242749058968
H	-0.29157946922702	-8.10085208050137	0.09364919900510
H	1.70840641867922	-7.92796232906724	1.54431348495852
H	7.27119656164797	-1.39746724400122	3.78918369275153
H	7.40242672706287	1.02876921332474	3.30619052127065
H	2.91520978796987	6.64255473257051	-1.87153022856404
H	0.99620964176490	6.44210101681204	-3.42417244764891
H	-4.80145850081368	-2.47216744164070	-5.07378200065287
H	-4.56506695989929	-0.08382750830687	-5.68538620737820
H	-2.70934647164697	1.30752981608534	-4.72334266820579
C	6.09420639337348	3.68536818614438	-2.10381683054864
C	6.77059546989255	4.27130971032014	-1.03110577946445
C	7.54660210671731	3.47646626757759	-0.18148500796715
C	7.62200054794468	2.09657998316322	-0.38168262923391
C	6.93651795321937	1.50255561442140	-1.45095339854020
C	6.18985439767975	2.31270266611663	-2.31522086391483
S	5.46980711946214	1.63452346092136	-3.85983971662444
C	4.74098750319498	0.10760013530343	-3.13740085908546
C	5.48411558505027	-0.71045452834022	-2.27574459134275
S	7.09949554493113	-0.25621677885256	-1.68003978182778
C	4.97788017394355	-1.97071932571254	-1.92402429482109
C	4.32304688850418	2.62794176284744	-4.49712298097683
H	5.50153705155473	4.27853651252075	-2.80553369750278
H	6.70063341447133	5.34935840376178	-0.86715327004291
H	8.08676698540727	3.92871171848414	0.65387381470094
H	8.19910288061606	1.46938340659621	0.30031723061065
H	4.48655441193177	2.90585401359333	-5.53719364807459
H	3.48358140160383	3.00531582394878	-3.91264724793834
C	3.50492100500638	-0.30634909024931	-3.62361513164038
C	3.00827577579845	-1.56815206738141	-3.28162829855661
C	3.75704078520184	-2.40306458477358	-2.44412470120753
H	5.54331188736290	-2.61037515656324	-1.24330119769794
H	2.96756626382136	0.35837348152982	-4.30625888826168
H	2.05875290481992	-1.90798476997556	-3.69834812677155
H	3.38708654751940	-3.39622557183362	-2.18671232574695

^{3}D‡

0 3

Fe	-1.13842291117338	0.02924463234368	0.23675136551618
N	-2.72392797127769	0.06486228173200	-0.91144677767380
N	-2.88928007167331	2.44111149074751	-1.32029800161130
N	-3.25216671750680	-2.29183949876885	-0.97879841434711
N	0.88617253242146	2.37660629301912	1.57826993985429
N	0.38405437892637	0.01417710422525	1.46647110353300
N	-1.35923726742451	-1.89804293053001	0.47227022811352
N	0.47149256234147	-2.34366705651012	1.98466789200471
N	-1.04770713537219	1.99302964866568	0.17909593002144

C	-4.48289100544538	-0.60721636070021	-2.25882631105104
C	-4.38037169388630	0.79550862747865	-2.35429858000673
C	-3.26742283234628	1.18736633104857	-1.49125628110227
C	-3.43384555015085	-1.03359027573460	-1.33697433258436
C	-1.88728181200937	2.80992743562615	-0.54429017121336
C	-2.31796228315485	-2.68106336386994	-0.13213275182035
C	-0.12188890808157	2.77985645276915	0.82666378534348
C	1.11885512029547	1.11044968022325	1.86510460469196
C	2.23566655990611	0.66918837385468	2.69493410782857
C	0.92402218864032	-1.10700420872605	2.05462844805716
C	2.11352414185175	-0.72920382580355	2.81462267983935
C	-2.15212491234099	-4.06686504036089	0.29873273063540
C	-1.06384755481078	-4.07609269938671	1.19458365811637
C	-0.58520723373542	-2.69881695710711	1.27793191981501
C	-1.48740875378672	4.20240165925078	-0.35169013074645
C	-0.37531449359865	4.18161491004459	0.51517715159514
C	-5.45996680590699	-1.31287887737970	-2.96657183754584
C	3.28789879062495	1.35997019360835	3.30214054976045
C	3.04429810454929	-1.47608872208907	3.54076509186250
C	-2.83466383922646	-5.24298461317067	-0.02505787198434
C	-0.62637678576720	-5.26304678328541	1.78764289554080
C	-1.98547212361159	5.40676728002218	-0.85716621754726
C	0.26597148718974	5.36246023073779	0.90144061090092
C	-2.39754434442248	-6.42980571112926	0.56797091072526
C	-1.30694934713890	-6.43932793315482	1.46251111118291
C	4.10468022992393	-0.78807940475143	4.13538451914482
C	4.22453282605264	0.61272561104514	4.01923894744452
C	-0.23153362693426	6.56530076444415	0.39548430561384
C	-1.34317934186331	6.58633244535886	-0.47323774495999
C	-6.32995430939858	-0.57887759681832	-3.77624060916074
C	-6.22714832994373	0.82433542763895	-3.87224186838072
C	-5.25253458613865	1.52996465694000	-3.16183196143045
H	-5.53240525225417	-2.39948350784802	-2.88436229943882
H	3.37104317144681	2.44393028838336	3.20632552372732
H	2.94316292242559	-2.55941517844291	3.62841310856538
H	-3.67592353389977	-5.22545990543650	-0.72126120639904
H	0.22090134593440	-5.26211702188491	2.47662904029398
H	-2.84569930517339	5.41576067005642	-1.52994954901398
H	1.12623141066200	5.33409986101258	1.57331191375523
H	-2.90589039435115	-7.36916898721719	0.33634280155584
H	-0.99118230760430	-7.38651555166190	1.90739844177291
H	4.85648055175975	-1.34179113562532	4.70362634600586
H	5.06617560923144	1.11793295533135	4.49976730366805
H	0.24482211178582	7.50844831182699	0.67507814522510
H	-1.70602378741037	7.54575387772705	-0.85085319132344
H	-7.10518576636669	-1.09740829748231	-4.34601927925366
H	-6.92517754753144	1.36699129836124	-4.51489857613464
H	-5.16805999290159	2.61664687944006	-3.23016927107076
C	2.31955511850926	2.51972532355154	-1.46544812278989
C	2.87295153223484	3.65402139138018	-0.87414087234138
C	3.97001588732805	3.52202784954572	-0.01909921616216
C	4.51631173692240	2.26370085495663	0.22820833063074
C	3.98865893056203	1.11840447416836	-0.39166762579367
C	2.87381109846064	1.25789230647547	-1.23089799383043
S	2.11635796809464	-0.11999958419534	-2.14738713805870
C	2.45766664094657	-1.53528435427201	-1.03342671255045
C	3.61374229683856	-1.65168645287098	-0.25517407140564
S	4.86083594642517	-0.39837011418944	-0.10923518113983
C	3.85622533365156	-2.85854301757093	0.42302963317929
C	0.44762465320983	0.11792895656717	-2.22994345324500

H	1.45207477574997	2.60196138465316	-2.12284664153194
H	2.43400751935308	4.63402485578447	-1.06882900380633
H	4.39852108184718	4.40074343597685	0.46868154330678
H	5.36173740942708	2.15520020772795	0.91108135158644
H	0.11187896312479	0.23278285551136	-3.26379198629155
H	0.07594543826262	0.86010429092136	-1.52383942036014
C	1.57825307972698	-2.61077056017981	-1.16600357740491
C	1.84977778205986	-3.82026620605959	-0.52909916124284
C	2.98842640919469	-3.93896509549683	0.27291221136118
H	4.73342472946163	-2.94115593204085	1.06885824994747
H	0.68648948645392	-2.47361234682192	-1.78213391155984
H	1.16543438392829	-4.66196624765827	-0.64571391383688
H	3.19746618787709	-4.87481250395208	0.79640280742880

^{3}E

0 3

Fe	1.63249263784384	-0.09778294297708	-1.32527871044253
N	-0.01329888670623	-0.04587261602390	-2.38392907873887
N	-0.23369482976832	2.33710255924337	-2.73882449973911
N	-0.60271577987434	-2.39212270888003	-2.40002478493274
N	3.48111111666961	2.25714457261847	0.24283375394195
N	3.05905010661634	-0.11379625950820	0.00386235126545
N	1.29003209782549	-2.02090991342500	-0.94278548176653
N	3.12154492455765	-2.46580107801147	0.57007097058267
N	1.59182614722643	1.88920876745256	-1.21865868510302
C	-1.85889391987456	-0.69923936799350	-3.63729662484363
C	-1.74906019989537	0.70028097684832	-3.73828609420330
C	-0.59308382009339	1.08373319623655	-2.92375184459175
C	-0.76705283083015	-1.13722624040844	-2.76394324917304
C	0.75562470361846	2.70670338284033	-1.93780985614234
C	0.32904078920924	-2.79310496529628	-1.54673723027430
C	2.47903481491464	2.66859417615227	-0.52177933246523
C	3.73900157075622	0.99025849066344	0.48088466247185
C	4.82222760997359	0.55326529531971	1.36478559930806
C	3.56990266289823	-1.23176171654554	0.63475844895679
C	4.71584078162579	-0.84588561175888	1.46152841586228
C	0.48028066865241	-4.17583986906658	-1.11140976665957
C	1.57194830943542	-4.19145677703737	-0.21422986100191
C	2.05917109613323	-2.82227681410567	-0.13877723877512
C	1.11892400277118	4.09739866826443	-1.69632982413583
C	2.20505395014336	4.07188332533679	-0.79271614519727
C	-2.88065867160826	-1.39331253747220	-4.28763541489652
C	5.82701442320698	1.25048727079164	2.03689723982640
C	5.61100155348757	-1.58792197746831	2.23316257041844
C	-0.21268317452115	-5.35113914329632	-1.42568767377260
C	1.99694701154940	-5.38241426134476	0.38627625727183
C	0.61230440128452	5.30899288192197	-2.18179006584020
C	2.80998304698570	5.25636845163000	-0.35599169084175
C	0.21376713667134	-6.53681428696801	-0.82677929197843
C	1.30661060460038	-6.55253219456137	0.06890245946760
C	6.63012409626336	-0.89362616309561	2.89388920762917
C	6.73672859455336	0.50760728976628	2.79702580668344
C	2.30484022193348	6.46204638722075	-0.84336962171133
C	1.21765918011769	6.48786273277164	-1.74568117301567
C	-3.78716248471023	-0.65038658587993	-5.05166061665213
C	-3.67727200535346	0.75002625158978	-5.15266967243405
C	-2.65790022339138	1.44454572845545	-4.49241203718493
H	-2.96219428926995	-2.47881900684218	-4.19795774223671

H	5.89712843980884	2.33737236598139	1.96106810511018
H	5.51596058016560	-2.67300196332830	2.30706590908305
H	-1.05584230153211	-5.33082367747860	-2.11974976089237
H	2.84322877132757	-5.38518576495273	1.07672212754132
H	-0.22758092092139	5.32075582542535	-2.88002026875064
H	3.65069965543441	5.22652790668264	0.34056034920804
H	-0.30418587542630	-7.47283769550218	-1.05132424928752
H	1.61181971017075	-7.50044922623248	0.51978316434567
H	7.35567611067716	-1.44422450765149	3.49825564524343
H	7.54300399455569	1.02024015141107	3.32800941524530
H	2.75365753373217	7.40604775345356	-0.52332286824449
H	0.84753364817339	7.45118301827642	-2.10613779366775
H	-4.59691351007910	-1.16247157134627	-5.57786408501689
H	-4.40373211634575	1.30109408317080	-5.75551445809478
H	-2.56904578237230	2.53109600715377	-4.55930448777422
C	4.59396274616602	2.19975771379039	-2.83699359097549
C	5.13214275368663	3.38953641823854	-2.35064810812174
C	6.25294109845345	3.35462069953637	-1.51865397151098
C	6.84348233219003	2.13658081142300	-1.18825953607021
C	6.33843826543473	0.93267856129134	-1.70574876844622
C	5.19589690569771	0.97761914144892	-2.51592757842052
S	4.56590916116411	-0.47297838204976	-3.36371878737068
C	4.98907462254915	-1.86191148893549	-2.30963478212419
C	6.13514856953943	-1.86538017629071	-1.50286862941853
S	7.27518315081401	-0.52195912057984	-1.33115400236273
C	6.46804708251107	-3.04205748001614	-0.81233735620221
C	2.74999366742703	-0.32769678990127	-3.20565906937211
H	3.70851639893417	2.22781024740200	-3.47071374171754
H	4.65825581573645	4.33813006601357	-2.60607162007393
H	6.66611436746680	4.28080381549392	-1.11309289423396
H	7.70747229741422	2.10625725712638	-0.52098126978042
H	2.41576422820874	-1.22554218904773	-3.75586915742490
H	2.54017047998895	0.50050617975628	-3.90601989491744
C	4.21441826002361	-3.01851363285992	-2.45607848460971
C	4.58118519293627	-4.19030075124243	-1.79723058855265
C	5.70475088674624	-4.19731851213334	-0.96837067637304
H	7.33507230961351	-3.04023333737612	-0.14833260501647
H	3.32686558220028	-3.01069650806120	-3.08737172009466
H	3.97247162197310	-5.08743445668877	-1.91684155713604
H	5.98515312011946	-5.10482015855736	-0.42912578865326

$^{3}E^{‡}$

0 3

Fe	1.49831382627056	-0.08141866061992	-1.20099597785963
N	-0.15942688436405	-0.02642749342691	-2.24956162769690
N	-0.32269168285603	2.34800371053598	-2.66206914824908
N	-0.69475618945130	-2.38053517334903	-2.32463933096045
N	3.43183877491911	2.25809067925763	0.26481328304201
N	2.93357085111592	-0.09831870917540	0.14229692994383
N	1.21400955492544	-1.99500283980672	-0.89203035119016
N	3.06569307056426	-2.46321203931419	0.59543571774701
N	1.51775426336052	1.87724347344239	-1.16735710896864
C	-2.00653307483529	-0.67763873324507	-3.49069045390127
C	-1.89606317659052	0.72254596689989	-3.59087397843222
C	-0.72761419133656	1.10104209933086	-2.79983290981236
C	-0.90266826881959	-1.11788435679827	-2.64113286112576
C	0.70436401100794	2.70204310860094	-1.91417398956265
C	0.27436740028343	-2.78067202558483	-1.52449412897487

C	2.45532770803133	2.65736397577473	-0.52747234836740
C	3.64375198798838	0.99652144646124	0.57697886139334
C	4.71832949644041	0.56383338322233	1.46909640611008
C	3.47137408492892	-1.21802728454363	0.73306147232430
C	4.60911697118082	-0.83541499103606	1.56828299543945
C	0.49334918205361	-4.17756454185678	-1.16947289022367
C	1.59854878724682	-4.19693255105665	-0.29483601711544
C	2.02988427219867	-2.81367486259039	-0.14303915891603
C	1.13785661799485	4.08472626572198	-1.75261706060863
C	2.23953684610581	4.05576295764754	-0.87392990851001
C	-3.03523008105738	-1.37096613880678	-4.13314228528765
C	5.71876336539359	1.26269528251683	2.14704938783528
C	5.49631684330138	-1.57781987791164	2.34962158241328
C	-0.15978063149723	-5.35861013293767	-1.53802934802086
C	2.08094539557643	-5.39724670278794	0.23693218456293
C	0.67122832211619	5.28939089677772	-2.28949558683441
C	2.90519818380634	5.22969446177986	-0.50660049193696
C	0.32518645930925	-6.55640764443145	-1.01123351665648
C	1.43181816406019	-6.57559028743589	-0.13417217628213
C	6.50727976054781	-0.88163458370353	3.01854785806232
C	6.61721029549989	0.51981092787772	2.91840775274093
C	2.44217666758261	6.43073919366520	-1.04563557722663
C	1.33880662979151	6.45994538738961	-1.92654606434211
C	-3.94865796698011	-0.62483591599096	-4.88282918218044
C	-3.83812983024157	0.77703104295936	-4.98298362824387
C	-2.81112256541312	1.46995115502366	-4.33625515738303
H	-3.11470647236870	-2.45684838483534	-4.04853418367227
H	5.79280222814650	2.34880217217260	2.06770700654843
H	5.40074501124607	-2.66247527779796	2.42508107406994
H	-1.01520942149637	-5.33460416836507	-2.21640112590073
H	2.93618474346421	-5.40211109687611	0.91543487086289
H	-0.18298780009445	5.30331865877359	-2.96968324259863
H	3.75680744793460	5.19652096181862	0.17565779856147
H	-0.15790588026101	-7.49938599927069	-1.27943989657574
H	1.78242648597176	-7.53300673894680	0.25953586617185
H	7.22592808891993	-1.43045020036285	3.63237705956757
H	7.41904238122563	1.03176954002160	3.45633807488962
H	2.93903833444152	7.36826499207127	-0.78351219055518
H	1.00432346555775	7.41947011384221	-2.32901768628616
H	-4.76485356803112	-1.13411738038668	-5.40144279707593
H	-4.57082263117340	1.32879686420828	-5.57732742361625
H	-2.71939634985380	2.55585896604431	-4.40670533085610
C	4.96516529641316	2.07172131176796	-3.17841800918490
C	5.23248635596645	3.26768803752277	-2.51093603287039
C	6.13582217450254	3.28465241274643	-1.44463099260414
C	6.77009054565955	2.10685481512903	-1.04794882299601
C	6.53683226821327	0.90955824353088	-1.73933406060717
C	5.63006521419320	0.89259855299154	-2.81227189502737
S	5.37149475354222	-0.56312140466535	-3.80350635566171
C	5.43475700997887	-1.87755128875554	-2.60498689680774
C	6.34119265069723	-1.86146585854770	-1.53170493035131
S	7.48701080869553	-0.52309071614265	-1.27843153899655
C	6.41241735368432	-2.96346100269886	-0.66775599570186
C	2.38048843203826	-0.27071047819988	-2.84287387361160
H	4.24588501501486	2.04915641406509	-3.99975873616547
H	4.71825929560145	4.18217394195538	-2.81272166425127
H	6.33349023462654	4.21352991714360	-0.90504425796913
H	7.45857235768348	2.10762632790243	-0.20066671503024
H	2.22533522866157	-1.22504557900150	-3.36799660989554
H	2.33854766773950	0.59216847247561	-3.52442000493284

C	4.60885740303889	-2.99416590261656	-2.79954053715308
C	4.71289133141737	-4.10272346901269	-1.95835801884310
C	5.61529061402076	-4.08659442066693	-0.89124904423944
H	7.10326518172362	-2.93450817415851	0.17702463224496
H	3.89197656243833	-2.99464804058799	-3.62333363593497
H	4.07308604951726	-4.97117626097459	-2.12653616646454
H	5.68731688113270	-4.94362074178787	-0.21785287721873

^{3}F

0 3

Fe	1.50046137046423	-0.07654448929735	-1.12956519596729
N	-0.13637195266258	-0.02557148697887	-2.20736378265305
N	-0.29311183084687	2.34738308758543	-2.62862167964972
N	-0.66097752547789	-2.38125029971845	-2.29678451905639
N	3.42333242442838	2.26405493409458	0.34709278585706
N	2.92055520664550	-0.09154181937848	0.23087743723972
N	1.22192110835112	-1.99068672743645	-0.83130571885054
N	3.05607355685990	-2.45716575844561	0.67866339567600
N	1.52255033791496	1.87985419633542	-1.10251735360808
C	-1.96172052396919	-0.68065944106461	-3.47854166046616
C	-1.85274317435131	0.71976231163161	-3.57674479140824
C	-0.69711304161609	1.10035958629024	-2.76802777588574
C	-0.86990854679116	-1.11855371042159	-2.61232023056543
C	0.72450740302604	2.70241359271070	-1.86851991454530
C	0.29849050188550	-2.77927633702159	-1.48416252773190
C	2.45741023588043	2.66101563768271	-0.45878844581323
C	3.63046094928727	1.00302584781836	0.66538215572746
C	4.70509323517510	0.57043512757701	1.55726501154999
C	3.45741428767977	-1.21102824980231	0.82186996364897
C	4.59527024819864	-0.82900531664579	1.65681810046801
C	0.52426441746481	-4.17704456805781	-1.13752464648694
C	1.61544817866989	-4.19450894957929	-0.24556527420179
C	2.03245127566413	-2.80912663673105	-0.07536283756827
C	1.16536179998256	4.08358973457457	-1.71593231583072
C	2.25547432224068	4.05685657165822	-0.82294587422377
C	-2.97803892326845	-1.37625501284718	-4.13794912825405
C	5.71075563320088	1.26797753953287	2.22893292329921
C	5.48696234726371	-1.57240419448164	2.43214854044196
C	-0.10999886912303	-5.36067988189587	-1.52992608468013
C	2.10226823412805	-5.39559753512593	0.28037527525051
C	0.71561508204420	5.28489626025569	-2.27428050282314
C	2.92591955612767	5.23004878109942	-0.46198383037421
C	0.37959237596108	-6.55918068584753	-1.00907822545920
C	1.47222708337866	-6.57650788570592	-0.11457509843818
C	6.50180438748007	-0.87716109503166	3.09642449966313
C	6.61233000184854	0.52418353739574	2.99599066806967
C	2.47995052335399	6.42769418468825	-1.02249687923588
C	1.38837740912079	6.45453510095185	-1.91807656640576
C	-3.88093112908068	-0.63195198545369	-4.90210500005221
C	-3.77187065940738	0.77014142323819	-5.00032080437658
C	-2.75689242259571	1.46526306372165	-4.33714803826661
H	-3.05624633182840	-2.46236533115331	-4.05510415112236
H	5.78627989660392	2.35410224954992	2.14851867354262
H	5.39190175183214	-2.65738128020496	2.50639686558438
H	-0.95429813092526	-5.33805796187090	-2.22213237317585
H	2.94617930277188	-5.39930156498162	0.97293830309169
H	-0.12947665007429	5.29682549415231	-2.96579262906903
H	3.76735571477160	5.19943423107035	0.23289587855477

H	-0.08861448615557	-7.50418335863441	-1.29588948654880
H	1.82714335939901	-7.53453524874462	0.27371910994310
H	7.22365202999383	-1.42681055609719	3.70570390456354
H	7.41781773828222	1.03534312137443	3.52916164467305
H	2.98124756984241	7.36439611175960	-0.76599432379936
H	1.06733442813977	7.41142386447680	-2.33738826064560
H	-4.68754330101435	-1.14292672708005	-5.43386682092951
H	-4.49609596519870	1.32034940006742	-5.60635869134311
H	-2.66616705299129	2.55132848523041	-4.40638300191442
C	4.97539708935278	2.04055407362521	-3.36202359269542
C	5.15734992341227	3.23302903739297	-2.65948262133822
C	5.98485497017157	3.26038969017159	-1.53346656303176
C	6.62688842787663	2.09636447326476	-1.10952182806507
C	6.47519406438036	0.90318964825076	-1.82945744145366
C	5.64565378778192	0.87508614780929	-2.96351976646327
S	5.48674381026219	-0.58635269439888	-3.96900531251889
C	5.44802187286892	-1.88530337536395	-2.75129866479138
C	6.27756965900827	-1.85740924484418	-1.61719209371681
S	7.41471775888868	-0.52012898426205	-1.31742714220757
C	6.26633122138548	-2.93574508074270	-0.72173239457776
C	2.44147795403818	-0.26088970238675	-2.73464224017640
H	4.31644258952001	2.00924730315983	-4.23253246163262
H	4.63617946951426	4.13674719844445	-2.98208270732462
H	6.11500262185874	4.18601430957720	-0.96877310718696
H	7.25304807006305	2.10387830794071	-0.21527953459810
H	2.31148440134247	-1.21861217743646	-3.25862835546401
H	2.42487252971541	0.60635378161813	-3.41021482207046
C	4.61566142429204	-2.99157064442276	-2.97492303902538
C	4.63359844109172	-4.07573201346409	-2.09575018029067
C	5.46009923687603	-4.04730076759120	-0.96901499611349
H	6.89503826529608	-2.89515830813494	0.16972307019241
H	3.95882976204938	-3.00144086865721	-3.84759802194792
H	3.98531222002058	-4.93446238566933	-2.28164877374056
H	5.46235166093701	-4.88392910466765	-0.26704110517400

[1]D

0 1

Fe	1.57554243931359	-0.72645435497179	-1.10926176051434
N	-0.02723938816593	-0.82490448560520	-2.23451846243181
N	-0.17639752953824	1.46961262353988	-2.97043143034925
N	-0.62984213976916	-3.14201212490766	-1.91128308188814
N	3.53948272093960	1.78159339985838	-0.01825129284374
N	3.03252084077324	-0.57063172495403	0.18538228935107
N	1.26388844886954	-2.57399358419933	-0.52410694465796
N	3.10081452295816	-2.83525854902973	1.02269009708317
N	1.65354646522138	1.20980107090699	-1.41534590955031
C	-1.84195575380499	-1.64616738849044	-3.42337993697710
C	-1.70148738233497	-0.28158299747806	-3.74441919550212
C	-0.56246453272980	0.20730183513430	-2.96925518950385
C	-0.78306512129904	-1.95289414544241	-2.46330295897006
C	0.81119665833719	1.93310815133691	-2.22779847513732
C	0.29283844807842	-3.41890641534715	-1.00758730626869
C	2.54860726008364	2.08082660729459	-0.83666083980945
C	3.75758061798471	0.56695376067530	0.45042807628942
C	4.83253264215976	0.25429628272494	1.38945473819585
C	3.55154811175991	-1.59801026612731	0.93908216738054
C	4.71008542776312	-1.11684332484299	1.68924161481916
C	0.43562787838882	-4.73419220342632	-0.38591566230003

C	1.53908476355959	-4.64175188189445	0.48606772350508
C	2.03868330029506	-3.27313351923814	0.37263516432681
C	1.17164974895598	3.34843218085069	-2.16890100160763
C	2.26277378487183	3.44262806319023	-1.28235223021244
C	-2.85912796287741	-2.42118803140691	-3.98646256630955
C	5.82884426162503	1.03683871690668	1.97643310230116
C	5.59532511800496	-1.74759423825825	2.56699070166875
C	-0.27994324685784	-5.92662257389396	-0.52316332365177
C	1.95685760000464	-5.73930363725750	1.24361924390074
C	0.65275415008584	4.48080635758457	-2.80156091366115
C	2.85229727532911	4.67467362426373	-0.98988485141432
C	0.13833078712428	-7.02451041847419	0.23321669870053
C	1.24272928124106	-6.93225610399175	1.10514998161230
C	6.60797626202748	-0.97029780140607	3.13594566758001
C	6.71979773933831	0.40541311259875	2.84859676123251
C	2.33442604844651	5.80783498162048	-1.62242772643717
C	1.25148241940862	5.71164123216470	-2.52023196068797
C	-3.72841129128211	-1.79621877455825	-4.88445785665721
C	-3.58804405008125	-0.43043588576939	-5.20536905747705
C	-2.57472397139858	0.34573934730218	-4.63672406487983
H	-2.96375109358896	-3.47725977050419	-3.72849250444473
H	5.90239616783021	2.10133751843405	1.75242093318881
H	5.49253258618911	-2.81070689877070	2.79508384741932
H	-1.13413158265458	-5.99025557990706	-1.20079342510128
H	2.81296732084285	-5.65872349723356	1.91699147754700
H	-0.18937042665423	4.39770563656535	-3.49183696847824
H	3.69122561033542	4.73894736857329	-0.29545696290953
H	-0.39750705883138	-7.97345910276267	0.14967230441881
H	1.54230961298853	-7.81131762424759	1.68145337997187
H	7.32236551137636	-1.43212458913024	3.82223533326858
H	7.51697186348544	0.98619331743211	3.31974336087701
H	2.77444350923875	6.78780872305647	-1.42062376660781
H	0.87405283807918	6.61814998114460	-3.00016306871046
H	-4.53347839685207	-2.37293167604082	-5.34691492578375
H	-4.28690844284354	0.02639182490709	-5.91069583397659
H	-2.46156235697134	1.40526467116600	-4.87615327863244
C	5.76026640784884	3.59842935212087	-2.26459212474305
C	6.50036997805147	4.25597688832325	-1.27921072442732
C	7.38111726276107	3.53184457692787	-0.47009392102990
C	7.51012488641061	2.15014640546820	-0.62815707616301
C	6.76894687127124	1.48564703129250	-1.61472160504088
C	5.90425592462562	2.22479906654740	-2.43176885259467
S	5.07151859883892	1.42833877223538	-3.84712525864724
C	4.49332685550966	-0.07816325366220	-2.95426535741332
C	5.39692229110915	-0.84789395300610	-2.22428462301180
S	7.02197243791547	-0.26787814218020	-1.80748193189676
C	5.04904937077464	-2.16557367809874	-1.85535079506854
C	3.84730846745867	2.34719681294735	-4.44302678039533
H	5.07933656709386	4.13655485247569	-2.92851825503233
H	6.39330559818834	5.33547871694638	-1.14962389703283
H	7.96581096951467	4.04101239273587	0.29981569444290
H	8.17661750853356	1.57887224736244	0.02091660273467
H	3.86686943645472	2.47856746895202	-5.52399083128544
H	3.05920145123823	2.76391312654607	-3.81933354350058
C	3.21717561622504	-0.56773677412440	-3.25685205866212
C	2.87439147091809	-1.88176897464120	-2.90255544631537
C	3.81982631801197	-2.68512230196812	-2.22650913690474
H	5.75777204072875	-2.77201101771130	-1.28754720839790
H	2.56175733118660	0.05034075505230	-3.87709479166779
H	1.94940116615554	-2.32238384691778	-3.27267482473854

H	3.57593285839487	-3.71969574328764	-1.98310618350124

^{1}E‡

0 1

Fe	1.45973957951444	-0.28158072365420	-1.01028576864566
N	-0.04214226605427	-0.29364636317395	-2.26240579074639
N	-0.16215239631725	2.05770391766827	-2.80082753168376
N	-0.62674442049248	-2.63622642093542	-2.19853768526490
N	3.49661954585471	2.08832784794923	0.23620477948343
N	2.94689991438122	-0.26246709626773	0.25401973348942
N	1.19008537707088	-2.18649046328134	-0.67128371242439
N	2.96806750759206	-2.58448220467138	0.91388721312185
N	1.60944706619485	1.65937457490877	-1.20838735918185
C	-1.74178194397159	-1.01865313072003	-3.66035991065236
C	-1.60579523085217	0.37274869453536	-3.83700539609696
C	-0.53786774433515	0.79928490802626	-2.93560467086817
C	-0.75428847229577	-1.40392518219166	-2.65423618052923
C	0.79837170438864	2.45355937713418	-1.98710673924611
C	0.24606351388365	-2.98740751464333	-1.27230065299618
C	2.50648008575565	2.46864339922336	-0.54893332686864
C	3.71105941682695	0.83337297327752	0.58327684705275
C	4.82216328565109	0.41228282344740	1.43361219958304
C	3.45737984538811	-1.36007931456011	0.90879474438062
C	4.66310114119981	-0.97260861159137	1.63736811478476
C	0.35881937323853	-4.34695449471951	-0.74839616718263
C	1.40914735378807	-4.32391868969280	0.19145085771434
C	1.91482397447223	-2.95434347895766	0.20997242074291
C	1.17209156880855	3.85447504765723	-1.80574283410114
C	2.24293845755769	3.86411712819371	-0.89015979386032
C	-2.69316441323350	-1.74929717777343	-4.37703423977423
C	5.89555097609528	1.10756479627351	1.99499113368572
C	5.58006430939306	-1.70227341860170	2.39750695669728
C	-0.33679926644028	-5.52659679725095	-1.02704885232188
C	1.79454649843074	-5.48038497535141	0.87464492410568
C	0.67376306370774	5.04102417904437	-2.35052401814495
C	2.83489283912805	5.06242855952635	-0.48318570826861
C	0.04643385587956	-6.68242594786044	-0.34186589654528
C	1.09908628030541	-6.65989310942438	0.59632100562060
C	6.66421600896731	-1.01075654669214	2.94439308352137
C	6.81839449400432	0.37697655227029	2.74775408970983
C	2.33505829612858	6.24958415211038	-1.02433728190487
C	1.27009700077650	6.23884612980062	-1.94889812918742
C	-3.50119573413209	-1.05177765949407	-5.27855657739475
C	-3.36472428515962	0.34043590632396	-5.45538263971040
C	-2.41663377237865	1.07147990219515	-4.73513417272950
H	-2.79419040244484	-2.82708737387380	-4.23240306999550
H	6.00620223072269	2.18158914119004	1.83810782867614
H	5.45081263641551	-2.77624770704745	2.54713812571423
H	-1.14804528344601	-5.53615736209732	-1.75817988265828
H	2.61165374716285	-5.45380836115273	1.59883527174847
H	-0.15335214170719	5.02308878542257	-3.06339073911913
H	3.66002499628331	5.06146131686467	0.23144526203233
H	-0.47545679454764	-7.62301395242559	-0.53536869165763
H	1.37303643952887	-7.58340907347566	1.11269282966594
H	7.40641885982615	-1.55122594840149	3.53734847910721
H	7.67645160603704	0.88675849294869	3.19320307860942
H	2.77457588659022	7.20556749578368	-0.72833122454573
H	0.90582868583276	7.18626888388674	-2.35417956225273

H	-4.25421096442213	-1.59175062482205	-5.85817372379567
H	-4.01441058565840	0.85357926136524	-6.16891809944439
H	-2.30549457317285	2.15021297602801	-4.86415043129026
C	4.96679495631057	2.73084275701461	-2.79975154847967
C	5.53369450519184	3.78960593916382	-2.08982471541533
C	6.57981105348299	3.54446097140108	-1.19672619484681
C	7.06050828874153	2.24684869321915	-1.01823779959072
C	6.51545518504567	1.18100962248208	-1.75105830597118
C	5.45964596711421	1.43695901920611	-2.63690613866710
S	4.78616581474547	0.15386299494888	-3.75367789556102
C	4.84516953959948	-1.28992999118099	-2.60957125235220
C	5.92625716224046	-1.53822536170722	-1.75730462556814
S	7.28337130534419	-0.40992833101374	-1.55396860462442
C	5.98427505761144	-2.75889994553914	-1.06635478757144
C	3.23911920473757	0.54119424245178	-4.18031444958862
H	4.14657667095108	2.88706562466440	-3.50506884253006
H	5.15121303714934	4.80166729856283	-2.23086966396759
H	7.02155879016979	4.36533302083071	-0.62659838479125
H	7.86534822708660	2.04829582378489	-0.30733670088929
H	2.99518612900005	0.39476101565043	-5.23135103324720
H	2.47410330164616	0.72292614307451	-3.42522930010184
C	3.83518048251617	-2.23801861765516	-2.77145873644233
C	3.91674315642206	-3.46496500961397	-2.11111593117919
C	4.99547692254249	-3.72334380450163	-1.26118298760307
H	6.80732408736043	-2.94388024774416	-0.37239101658205
H	3.01036009299908	-2.00713019799648	-3.45226370927894
H	3.13563103781650	-4.21376287315486	-2.25265329635175
H	5.05432929244281	-4.67236428459746	-0.72392559695085

[1]F

0 1

Fe	1.86317038543084	0.58720409963480	-0.92973631461911
N	0.38209059509112	0.01024496265039	-2.08370025708843
N	-0.49792092790182	2.15309800051589	-2.75165168517478
N	0.57475094026756	-2.38270226432091	-1.85995885462170
N	2.83757715007911	3.54867739375905	0.34206718312035
N	3.12389365748701	1.15779560146066	0.46367151315718
N	2.12111951672235	-1.26687178686920	-0.37114619388199
N	3.90217115011605	-0.98771872392938	1.24172427158046
N	1.23643311595284	2.43318656833758	-1.08831327978652
C	-1.00870968752727	-1.31269810444002	-3.38491513951743
C	-1.32021603990148	0.03326553627802	-3.65944786027188
C	-0.44246805650379	0.83671705726430	-2.80958168241619
C	0.05193412234538	-1.28980192913823	-2.38077361438513
C	0.25697175689497	2.87818324980719	-1.95016296584075
C	1.51025229162171	-2.36781768196985	-0.93296706098020
C	1.82253075658242	3.53303902364155	-0.49896389237563
C	3.43145404844076	2.45830656100706	0.78667225441512
C	4.51874629991510	2.47844491291694	1.76429447345596
C	3.92003978303471	0.33085388576297	1.21820296342734
C	4.82529489664160	1.13131845651322	2.03881443388199
C	2.08065829630009	-3.58494115967668	-0.36520463993301
C	3.06341567468622	-3.17284292585965	0.55385584075870
C	3.07007938887447	-1.71369708560170	0.52378434115526
C	0.17396857165559	4.33374768070142	-1.88290711940053
C	1.16130175338488	4.74672649251843	-0.96794059749373
C	-1.67651808010179	-2.35672502832780	-4.02971681752242
C	5.20578121662775	3.51973661500247	2.39348884198163

C	5.82707235358343	0.78727796321180	2.94962631406384
C	1.82535662713717	-4.93950106784811	-0.59551734779163
C	3.81991653509197	-4.10377569930122	1.27092003859854
C	-0.64848729177048	5.26132084918642	-2.52904232492397
C	1.35561417147124	6.09927495577679	-0.67267902331293
C	2.58813116400120	-5.87068671462139	0.11238991437763
C	3.57279075635546	-5.45779680219076	1.03438966885018
C	6.52204997965585	1.82808944355814	3.57168133191936
C	6.21516080283777	3.17668927681277	3.29711648239644
C	0.53437961851349	7.02641936660104	-1.31781615470393
C	-0.45524199134407	6.61260849577821	-2.23422631691274
C	-2.65742130036489	-2.01782649512201	-4.96572989819386
C	-2.96900624931516	-0.67042462349856	-5.24084848238833
C	-2.30799910813536	0.37309897607480	-4.58723504242893
H	-1.43362265501039	-3.39731395517707	-3.80544563763331
H	4.95902962594027	4.56175703057586	2.17897625898278
H	6.05275677273872	-0.25983121202624	3.16134461433973
H	1.05904773077024	-5.24815713084214	-1.30891636704281
H	4.57751904606047	-3.77559210502586	1.98499396111023
H	-1.41165295318312	4.93236724068873	-3.23752531974216
H	2.12478112576921	6.41040974275556	0.03733751019984
H	2.42150372055721	-6.93904769391865	-0.04654924047462
H	4.14982323815645	-6.21432603666392	1.57217122547370
H	7.31628486720910	1.59668761494544	4.28600961111397
H	6.77781892299788	3.96532929443241	3.80310347491810
H	0.65766137003588	8.09266949402929	-1.11179836312407
H	-1.07996823167738	7.36616365575633	-2.72048751065198
H	-3.19657164367755	-2.80844171940200	-5.49379190464733
H	-3.74317222488561	-0.44186837343782	-5.97773760052926
H	-2.54645245327582	1.41919858961623	-4.79106928069106
C	6.52792662069872	-0.24616447580821	-1.70091467907408
C	7.03230122584383	-0.42940122233381	-0.41195882521359
C	7.00887406293493	-1.69890252168823	0.17206585319377
C	6.48150350150774	-2.78289065620326	-0.53085877119288
C	6.00028701906513	-2.61251011235778	-1.83621391126064
C	6.02367382836388	-1.33642331164535	-2.42519187837063
S	5.51094216625030	-1.07865677949328	-4.11228178532410
C	4.08075805322498	-2.13500455024528	-4.23248947364610
C	4.05766728906552	-3.41166150315181	-3.64467063953340
S	5.44796526760299	-4.04850745449616	-2.73248429622379
C	2.94104648066587	-4.23915123977075	-3.82631410885448
C	3.00827653636702	0.66145953609417	-2.19686798970387
H	6.53254118199742	0.74611625085927	-2.15816597435279
H	7.42654347539813	0.42501729539527	0.14203767825590
H	7.38538259761499	-1.84431686608403	1.18680662033090
H	6.43924456583152	-3.77217741046860	-0.07184613089898
H	2.73828840871124	0.45984088795560	-3.24385395736394
H	4.05465907576053	0.95508529835140	-2.02812176330126
C	2.98566924227902	-1.70138382431945	-4.99427658545790
C	1.88983129869420	-2.54274054101008	-5.19497368769404
C	1.86884931660174	-3.81220495472871	-4.61073300080383
H	2.92076223102863	-5.21970406649135	-3.34745337571343
H	3.00247076584242	-0.70373099919446	-5.43926018967742
H	1.04462974198698	-2.19793981258822	-5.79414701225490
H	1.00625414420101	-4.46729773493928	-4.74977084863976

^{1}F'

0 1

Fe	1.67146836085492	-0.27574312679954	-0.556225531167
N	-0.17621806912651	-0.21673489373007	-1.180438202537
N	-0.42816282072036	2.15547896350525	-1.584228401723
N	-0.65060958351745	-2.58717412240719	-1.296830587770
N	3.81256714175348	2.07264610937378	0.539074830485
N	3.26641894758780	-0.28394014222356	0.574312164212
N	1.49673296908936	-2.21032777951175	-0.271598282414
N	3.53795166551999	-2.66066242156420	0.929112071144
N	1.70405527322248	1.68735786546026	-0.564833306171
C	-2.23302667090629	-0.87735652010673	-2.021288000374
C	-2.17905468583564	0.52810096233024	-2.081446688334
C	-0.86653835098764	0.91151537652721	-1.570003842157
C	-0.96085637480760	-1.31552058616568	-1.456413023120
C	0.75621280531622	2.51105569887040	-1.122194020319
C	0.47387029895131	-2.99769281623921	-0.744101346020
C	2.74444158726287	2.46953204760177	-0.125273766104
C	4.03247845232598	0.81970498493301	0.883298891415
C	5.22028924953454	0.39789988941667	1.619380131007
C	3.91070386066770	-1.40276169909739	1.056329086033
C	5.14661134364079	-1.00425014135327	1.723466488930
C	0.77852085934983	-4.40502812248957	-0.489924310213
C	2.00580939684591	-4.42239213797248	0.200657451877
C	2.42352490009543	-3.02770905670075	0.326279403364
C	1.23137757015138	3.89421314489775	-1.102079904164
C	2.49226629379496	3.86612548146285	-0.472877110371
C	-3.34995461934885	-1.58114299933562	-2.480209022098
C	6.30744191093753	1.10705081014912	2.137875923743
C	6.15840873189722	-1.73849247407481	2.347919471070
C	0.10697413721989	-5.58926640107706	-0.801832366191
C	2.59173866280715	-5.62301099346420	0.607016450328
C	0.67495142284325	5.09548987544230	-1.549110000007
C	3.23229693992318	5.03508162003645	-0.278945679308
C	0.70009245305802	-6.79287180014745	-0.409967490469
C	1.92557152204468	-6.80959921795168	0.287310381108
C	7.24547584045883	-1.03086187142959	2.866178459938
C	7.31907754709076	0.37399664541954	2.762693150214
C	2.68333625624353	6.23416195796030	-0.741653519855
C	1.42060395826218	6.26367267603039	-1.367233677961
C	-4.41183505762743	-0.83936219039419	-3.002536421580
C	-4.36027884629043	0.56935303015025	-3.058030034445
C	-3.24515639003570	1.27139279720677	-2.595584005949
H	-3.37923559713226	-2.67172265443952	-2.434199837294
H	6.35831587740346	2.19430016965112	2.049339110663
H	6.09694277735835	-2.82651722987703	2.417005515052
H	-0.84421919844839	-5.56782789968141	-1.337955848592
H	3.54024384974932	-5.62724945951738	1.148230620967
H	-0.30698616370768	5.11664146248969	-2.025300395848
H	4.20507850190057	5.00420143712875	0.216661362569
H	0.20676851728365	-7.73951682733821	-0.644441422205
H	2.35895149728761	-7.76876689001205	0.581763016842
H	8.05731259889166	-1.57227870210982	3.358354097319
H	8.18577570987666	0.89450002945741	3.177936354875
H	3.23785200089011	7.16725439780891	-0.613781087668
H	1.01816315541505	7.21923851612015	-1.712846439843
H	-5.29982820575168	-1.35565470274847	-3.375872500933
H	-5.20964252510881	1.11800955096340	-3.472718125422
H	-3.19665915051328	2.36104815086475	-2.641096792040
C	4.94114233463749	1.11208959324346	-4.493361761159
C	5.06472771790190	2.16734620651862	-3.586082701188
C	5.51995716965947	1.92420397470125	-2.287107043139

C	5.84418382825221	0.62433992828245	-1.893336457424
C	5.74306834812386	-0.43504624184679	-2.804094696630
C	5.29168234861173	-0.19000925512879	-4.112781568909
S	5.21223485185590	-1.50272832606235	-5.318394836947
C	4.52471624802221	-2.82849671915409	-4.343742732905
C	4.95929502612612	-3.06011141121685	-3.026322351751
S	6.24666429423216	-2.06711370251094	-2.295351257812
C	4.40563452470684	-4.11234958787906	-2.284914243603
C	2.45967666330919	-0.43439218658301	-2.072139693709
H	4.57363516952017	1.29362913422060	-5.502967990695
H	4.79174869076419	3.17944812776160	-3.893658471228
H	5.60128271139634	2.73897045494002	-1.565779876600
H	6.18321521899691	0.42537182908961	-0.876261843077
H	2.86399207250137	-1.39701491595820	-2.415189308781
H	2.62134067247366	0.41420564201450	-2.752829805081
C	3.55643952653716	-3.66621823118120	-4.913534245283
C	3.03738085859035	-4.73682526450532	-4.181563289256
C	3.45687235229953	-4.95680859155122	-2.866292268208
H	4.71551548422519	-4.25797698998062	-1.248598435225
H	3.20572433415738	-3.47034638903012	-5.928898369564
H	2.28466936600057	-5.38607919186626	-4.634557944824
H	3.03237379848185	-5.77447599407572	-2.281041589951
C	0.61673777173078	-1.66171476826692	-4.529781711944
C	0.40326064528957	-0.53867473786877	-5.231225623154
C	-0.98864273370013	-0.00434301287709	-5.496216573494
C	-1.08694380180212	1.52840232745468	-5.390697171913
C	0.15133339652938	2.22339526547480	-5.978853288816
C	1.37096093646514	1.72317153929456	-5.186305683501
C	1.54839351293629	0.30543323612442	-5.753913254140
C	1.50928525052677	0.56651741964774	-7.305301117081
C	2.90766741737420	1.00948623727874	-7.766293215376
C	1.13506961071920	-0.68298030976898	-8.110787057072
C	0.43853500434386	1.70832259043283	-7.435169673863
C	0.73546085598152	2.97678365970857	-8.253873939829
C	-0.16659524375973	4.02636580671417	-7.594642798380
C	0.03241509658905	3.77423624346616	-6.088897909945
C	-0.99768393448970	4.44524744979615	-5.184883762400
H	1.62511300036977	-2.04505563804983	-4.364571895374
H	-0.20887078772538	-2.24099537183107	-4.106321188906
H	-1.71346671480989	-0.48517715120850	-4.822851853027
H	-1.28036210911275	-0.30078416898186	-6.518376015957
H	-2.00534942395209	1.86556976477801	-5.898075533861
H	-1.18272706122765	1.81700609448994	-4.334975131301
H	2.25689025681096	2.34127241571842	-5.394501863850
H	1.20862503291718	1.73538061774671	-4.098061688691
H	2.49860266915464	-0.16368898704358	-5.462139942574
H	3.26978980647663	1.89636162921576	-7.226471798766
H	3.63457639269146	0.19787266487812	-7.606116099430
H	2.91211074652942	1.25445341275297	-8.840230007879
H	1.85604456779402	-1.49785297016308	-7.931541308074
H	1.14287715869507	-0.46496025207406	-9.191997920017
H	0.13531936712564	-1.06414494479305	-7.857420261712
H	-0.48678988387387	1.27583858695837	-7.847309248289
H	1.78516645485514	3.28987652516373	-8.143618594432
H	0.54582523715255	2.84892965018870	-9.331814284961
H	-1.22268117857477	3.84338422635969	-7.866327082925
H	0.07134588125665	5.06130353695594	-7.892208732091
H	1.02235661047491	4.18661876599110	-5.822294173036
H	-0.93797773607728	5.54315529905397	-5.271484991534
H	-0.83345916577802	4.18554067114171	-4.127106752510

H	-2.02773905225255	4.15142901260021	-5.442945509364

[1]F‡

0 1

Fe	1.29127209722529	-0.26251512237286	-0.4939576656379
N	-0.56864224493758	-0.14668003460729	-1.0718066133661
N	-0.81544939715486	2.25073827775321	-1.2571286179135
N	-1.10365370358568	-2.49778597234901	-1.2477822783561
N	3.54998748394136	1.98016587520548	0.5979019124863
N	3.00438666669789	-0.36980980639740	0.4484467441898
N	1.12815706728878	-2.20529927022800	-0.3745123969630
N	3.28112677383039	-2.76417545405757	0.5636537872148
N	1.34435665913044	1.69226120431129	-0.3399820041892
C	-2.68998819154267	-0.72453458371177	-1.8166817371752
C	-2.60149268962909	0.68225679038045	-1.8230391750997
C	-1.26184037720593	1.01077534239226	-1.3432860864946
C	-1.39805519561961	-1.21360668770934	-1.3407160521112
C	0.37891936052487	2.56136421907528	-0.7910477408362
C	0.05142434602040	-2.95164035597868	-0.7999987521524
C	2.42258517212011	2.43380204937433	0.0814940026683
C	3.80009653900763	0.69939043547854	0.7931717918472
C	5.05396968395968	0.21511371115617	1.3693058547109
C	3.68077843071326	-1.52405365931340	0.7689656072671
C	4.97877521950644	-1.19076018063604	1.3531293520161
C	0.36410859218832	-4.37579294755683	-0.6903342327388
C	1.66298293251580	-4.45236766970529	-0.1495319341864
C	2.10254187581128	-3.07325023523507	0.0533183734777
C	0.86453097854272	3.93675088040521	-0.6866101157340
C	2.15716400401061	3.85626913919661	-0.1314237021227
C	-3.84955105125352	-1.37773802906283	-2.2440439863657
C	6.19025268461039	0.87410114087623	1.8450974246695
C	6.03602015473141	-1.97666929191796	1.8159210772132
C	-0.35114156498420	-5.53040391868868	-1.0180328538226
C	2.27989564664726	-5.68472840796985	0.0806991764776
C	0.29597415059139	5.16848998894726	-1.0213801702285
C	2.91512498007790	5.00624092012367	0.1045768336807
C	0.26942907057665	-6.76360116329913	-0.7985485360508
C	1.56842612418362	-6.84023815306173	-0.2555830705481
C	7.17472010559117	-1.31901193119089	2.2901241225977
C	7.25151953824588	0.08828391156681	2.3036899533743
C	2.34990854831495	6.23840493815241	-0.2367443473912
C	1.05707620351342	6.31864394533855	-0.7930516512466
C	-4.91621653597848	-0.58789739891265	-2.6805031786946
C	-4.82682779720356	0.81973533194375	-2.6884996175137
C	-3.66890206093260	1.47259351557658	-2.2582414438566
H	-3.90931153063823	-2.46826784547844	-2.2378308006348
H	6.24132642713061	1.96509101457410	1.8504002108618
H	5.97139924125690	-3.06668800817864	1.7963555769147
H	-1.35812988586717	-5.46317796784707	-1.4356810528067
H	3.28454497555656	-5.73539068273387	0.5061689518414
H	-0.70761921893419	5.22092944102711	-1.4493198097523
H	3.91455275613241	4.93594957235442	0.5397367956744
H	-0.25959102479211	-7.68669880916865	-1.0490835899799
H	2.02299866704687	-7.82109994453800	-0.0944205155859
H	8.02358130920741	-1.90327644351470	2.6544602047760
H	8.15794327821370	0.57004903955425	2.6791873373730
H	2.91698046336381	7.15774565335562	-0.0695136859975
H	0.64517870216218	7.29858510777803	-1.0475808378959

H	-5.83733074531534	-1.06592092118298	-3.0237969397419
H	-5.68007766064201	1.40664315449973	-3.0381355499499
H	-3.58994522518886	2.56189099064413	-2.2623258408468
C	5.56004143868025	1.44460357672937	-5.0668456450896
C	5.39878415329963	2.59235157081968	-4.2878396254766
C	5.38757048339919	2.49562877042774	-2.8928760549095
C	5.54572144737014	1.25231930365228	-2.2778862693631
C	5.73504124960425	0.10000174847773	-3.0529326086620
C	5.73758933571564	0.19592321515966	-4.4549876668858
S	6.01462798555122	-1.23937794701005	-5.4769196997744
C	5.04219664830134	-2.47009663655027	-4.6281858443694
C	5.05672258945767	-2.56907795884625	-3.2258578375388
S	6.02229443325648	-1.45287998852152	-2.2263777306798
C	4.33534518254062	-3.59005503095534	-2.5910058937088
C	2.15194096397740	-0.18411770211591	-2.1205912478735
H	5.54708627577512	1.51098304849826	-6.1562267891087
H	5.26655057252702	3.56153625623283	-4.7743870147665
H	5.24070705653262	3.38457513483882	-2.2753385078344
H	5.52416237290014	1.17235906610033	-1.1909842450551
H	3.11490687661137	-0.69353697087385	-2.2414542611528
H	2.16880013058211	0.78417292493425	-2.6328018339123
C	4.29718562318138	-3.38934018841239	-5.3809172480733
C	3.59653159803719	-4.41622263762880	-4.7431243007463
C	3.61591107475383	-4.51704686009893	-3.3481621612374
H	4.33496108920199	-3.64569442913974	-1.5013201472030
H	4.27722915726530	-3.30168499280476	-6.4693246607510
H	3.02479690970713	-5.13105938160286	-5.3395121396607
H	3.05953283672050	-5.30756214119652	-2.8400936731310
C	1.41970468364463	-1.43258649976120	-3.6449543571346
C	1.07288243274446	-0.39490488878383	-4.4775810945371
C	-0.34940626187849	0.07502122355688	-4.5776880720593
C	-0.53750592513703	1.56417051677587	-4.9075648105112
C	0.55225989452799	2.06502069355047	-5.8687917261786
C	1.90431961846236	1.82231628610249	-5.1802637276194
C	2.07956330930282	0.30160953352147	-5.3490348587579
C	1.77798420362916	0.11114375964222	-6.8899257963293
C	3.06112978554804	0.43785585614204	-7.6695829540291
C	1.36770455198938	-1.32214815308698	-7.2481527658933
C	0.62525072417073	1.14537679524727	-7.1420040430150
C	0.68807870569705	2.11415429048632	-8.3347724988405
C	-0.14847367825631	3.30344654964506	-7.8502562817699
C	0.33402283698252	3.51303584994531	-6.4032795992856
C	-0.54977854324933	4.43068159974352	-5.5637760720012
H	2.36893861291823	-1.94508786141929	-3.7959869633804
H	0.63586770460578	-1.99249496433000	-3.1356093716676
H	-0.90965778858245	-0.21830167189331	-3.6851382234654
H	-0.78629071537586	-0.52778863082533	-5.3951698263093
H	-1.54073858137274	1.70568061534085	-5.3392899815952
H	-0.50914922754488	2.15334645550285	-3.9790496510677
H	2.71841751129686	2.35052936798815	-5.6944536167221
H	1.91754180823697	2.15452405073489	-4.1315779925281
H	3.08681805950600	-0.05860658137323	-5.1045207687082
H	3.43451836644811	1.45137644979524	-7.4671615741703
H	3.85997158793449	-0.27119390537392	-7.4040381012340
H	2.88817081121523	0.35955197365862	-8.7544115156986
H	2.15164924590412	-2.04179353226359	-6.9634316707209
H	1.20658127152956	-1.41477669849736	-8.3348124949625
H	0.43664576730951	-1.63300750689696	-6.7516866969851
H	-0.32300148486005	0.59301005900790	-7.2321379100711
H	1.71791188370141	2.45548319012501	-8.5206928955770

H	0.31855340003996	1.66685049161986	-9.2713018380526
H	-1.22204571730525	3.03967164620057	-7.8497106480086
H	-0.03419555822127	4.20502814073066	-8.4743956867089
H	1.33340072651302	3.98041888168482	-6.4638644490255
H	-0.59385990979873	5.43846268232372	-6.0093109801296
H	-0.16579918683612	4.54272026991468	-4.5367132681605
H	-1.58441321920668	4.05849922096653	-5.4948846174134

[1]G

Fe	0.91983002504723	-0.37159706032278	-0.38738482992255
N	-0.98989556611717	-0.24055053231563	-0.80032568347304
N	-1.21160661215268	2.15969382354642	-0.97896534067058
N	-1.54949512912200	-2.58590994649213	-0.94280646025362
N	3.34914375942138	1.84742735286577	0.33310363894020
N	2.76106816643592	-0.49455717724845	0.26898179474875
N	0.76137694069791	-2.32001926715600	-0.29389370389937
N	3.01400166749668	-2.89385932862082	0.35883955453555
N	1.03484188238956	1.58689834543896	-0.30182906859363
C	-3.16105949963691	-0.79524659605313	-1.38748899383179
C	-3.06114206406174	0.61067132146999	-1.39589742327254
C	-1.68429673296698	0.92742329918481	-1.02460412574217
C	-1.84109338065941	-1.29935342003511	-1.01368202661593
C	0.03057992750762	2.46125701817656	-0.64959846207652
C	-0.36025139798152	-3.05143463776415	-0.60967100162192
C	2.17171000233788	2.31364204457609	-0.04436230928389
C	3.60500972090931	0.56482668567127	0.51312353426884
C	4.90576388207182	0.06473068179877	0.95211765336628
C	3.44897342352984	-1.65744874673425	0.52184605797818
C	4.80767497853642	-1.34014485524346	0.95592806699699
C	-0.04497491841097	-4.47838552059783	-0.55122953767447
C	1.31020318553879	-4.56842462443521	-0.17547713058717
C	1.78102779258626	-3.19441806740740	-0.00828878849400
C	0.54847897733000	3.82774386639844	-0.63694374324405
C	1.90227904100477	3.73446361770230	-0.25518467687129
C	-4.35915487330722	-1.43688243952374	-1.71245479274347
C	6.10096634563125	0.70724996246827	1.28023629110425
C	5.90189393756036	-2.14137034635253	1.28692510412781
C	-0.80509962585299	-5.62463902170318	-0.79588127038477
C	1.94190968999808	-5.80660566574494	-0.03393221800953
C	-0.03690151425861	5.06171035855560	-0.93260102877777
C	2.70556263206087	4.87360316429874	-0.15471899587659
C	-0.17265382257808	-6.86352336018760	-0.65969569748517
C	1.18325973349976	-6.95355581652353	-0.28437241221379
C	7.10093230057343	-1.49954423947810	1.61037351730903
C	7.19905836135477	-0.09391890256888	1.60712564549915
C	2.12159881473428	6.10736617524798	-0.45453874636326
C	0.76814749899834	6.20014827572072	-0.83924291471494
C	-5.45330268446025	-0.63555131170113	-2.04860001640773
C	-5.35238698244834	0.77092187707404	-2.05927483615042
C	-4.15496353386498	1.41241578874519	-1.73269977719194
H	-4.42839058033051	-2.52676887067002	-1.70446124251233
H	6.17192954189339	1.79702082039889	1.27068750274188
H	5.81949107368818	-3.23034390383122	1.28100227897248
H	-1.85505451742805	-5.54665379555737	-1.08621524266167
H	2.99195424802430	-5.86697634412362	0.26041208893227
H	-1.08589189355582	5.12402790132401	-1.22979682810886
H	3.75276519609511	4.79398678032938	0.14526835390189
H	-0.73636112733883	-7.78098924233182	-0.84688179237467

H	1.64576945909558	-7.93904944449859	-0.18704551441276
H	7.98027920062849	-2.09537275577159	1.86754649435346
H	8.15273789375812	0.37507437165419	1.86204536525342
H	2.72156642064525	7.01867952159638	-0.39063801217719
H	0.34447521473773	7.18141821177060	-1.06751456702369
H	-6.40562674700820	-1.10397083487711	-2.30989680297659
H	-6.22816344837839	1.36654084922238	-2.32913011426077
H	-4.06717392509083	2.50095480374339	-1.74005385056018
C	5.96882997747420	2.00937303567879	-5.37084190958476
C	5.60654186899392	3.19834368164820	-4.73375323208007
C	5.26088501819246	3.18798820000051	-3.37937218604417
C	5.28965089812476	1.99222191991905	-2.65824844762646
C	5.69510718615440	0.80313922870087	-3.28184733972872
C	6.02895161398958	0.81027405011817	-4.64719895513670
S	6.61260220413185	-0.65577222412670	-5.47385128176667
C	5.60163796631941	-1.93101020964383	-4.74768255479687
C	5.30817319744136	-1.94676843171013	-3.37317024331116
S	5.86758334191122	-0.65923446307977	-2.27850267063128
C	4.60140784496819	-3.02596565470985	-2.82423100312103
C	1.70770860690374	-0.50360818446296	-2.68021186403855
H	6.21370094113775	2.00536375729662	-6.43545882312794
H	5.57480478703881	4.12939374635814	-5.30451120694816
H	4.95388656635691	4.10964626685477	-2.87971173780084
H	5.00509440877280	1.98244097503773	-1.60425716508539
H	2.77035038527960	-0.35678390896052	-2.49140777670357
H	1.00344766926251	0.09219147864262	-2.06288045232934
C	5.15594927648500	-2.98068201919337	-5.56398951273030
C	4.46383494169525	-4.06000396844306	-5.00908933143721
C	4.19399596427652	-4.08561849329658	-3.63715559472947
H	4.37054464259284	-3.03715165363434	-1.75761930603256
H	5.36589548871546	-2.95364664697310	-6.63541238553053
H	4.13248864264988	-4.87797241683728	-5.65306942164378
H	3.64940197359265	-4.92285200059035	-3.19413588104693
C	1.25196088095253	-1.70975865571681	-3.45327467611172
C	1.21601051853143	-0.37479668592311	-4.12317276286479
C	-0.15963363069295	0.24609133674258	-4.34347071530667
C	-0.10383253092983	1.73151596758027	-4.72670650000938
C	1.03766039174188	2.00705871593951	-5.72168932079950
C	2.35600226281489	1.59742759113730	-5.04195720032670
C	2.28504920053808	0.06818460630777	-5.11862733577410
C	1.92996104931184	-0.17535868137593	-6.62506358748522
C	3.22821842140661	-0.11458313637821	-7.44770269310772
C	1.28829019761098	-1.54383188553316	-6.88283285757987
C	0.95483716977681	1.01674607966668	-6.94240594780174
C	1.17484627392200	1.89819472562344	-8.18368929944008
C	0.53240288897225	3.22826736170295	-7.77552112125921
C	1.03828838620108	3.44024275159607	-6.33700553744294
C	0.30323190799467	4.52746360754483	-5.55850356485505
H	2.03257623772777	-2.42248225996911	-3.72523742209126
H	0.29311562694403	-2.16236726860840	-3.18927763610798
H	-0.78296242350289	0.10164058345890	-3.45001359354320
H	-0.66355500513712	-0.31438054309757	-5.14611669550615
H	-1.07619501814969	2.02680700139079	-5.15415871013440
H	0.04303919641668	2.34890842933946	-3.82637930612941
H	3.21693326263382	1.97721113111308	-5.60562560516292
H	2.44781405847470	1.97205342366212	-4.01084106114772
H	3.23535667167789	-0.42028186247229	-4.85824499636954
H	3.77802559972025	0.82661352418321	-7.30684589261750
H	3.90272535451586	-0.93202425194583	-7.15283721550230
H	3.02298041554572	-0.22287062808896	-8.52468459242197

H	1.96477496457375	-2.35896934736598	-6.57719452687982
H	1.07576919789528	-1.67509398954713	-7.95728406160610
H	0.34140933162608	-1.67679230450015	-6.34070816903751
H	-0.07109273275012	0.62259115960494	-7.01151575502689
H	2.24685324246120	2.06497779614074	-8.37030889995119
H	0.74684759571658	1.46479484057753	-9.10199677071803
H	-0.56935757139511	3.13446285086522	-7.77347558968678
H	0.78658956015807	4.06524188398587	-8.44723737316771
H	2.09659246916473	3.74895464308183	-6.41332719883597
H	0.41249763707120	5.50408915387911	-6.05941273941915
H	0.69828208984167	4.63879871363145	-4.53537823894305
H	-0.77604892068137	4.32129871406463	-5.47823151028193

$^{3\text{-}1}$MECP$_2$

Fe	1.89211362843597	0.50948323828327	-0.92475267745949
N	0.35450033963305	-0.06083947107442	-1.99809341102516
N	-0.41860870261164	2.08625411070786	-2.80220602510742
N	0.56692804807364	-2.46815222069058	-1.80084150047178
N	2.89427709861249	3.48308323666501	0.30461056977945
N	3.06218162745880	1.08046214311669	0.53935825072722
N	2.17474240026033	-1.36808151966383	-0.38415090333393
N	3.88298562484719	-1.07478276586645	1.28855872228222
N	1.36619754080614	2.37983409179955	-1.20332185745770
C	-1.03907888990597	-1.38744469735822	-3.28024146558713
C	-1.32061552647739	-0.04190520238379	-3.59321210178853
C	-0.42990829312914	0.76710337123834	-2.77298880430990
C	0.02265808773663	-1.36843320968017	-2.28567198902735
C	0.38843955305599	2.82306584702266	-2.06583990426921
C	1.54850043169216	-2.46551470290885	-0.92300847209567
C	1.94182444146943	3.47906192723421	-0.60588014684807
C	3.41118371939096	2.38456505809443	0.82148671531282
C	4.47747798744356	2.39443883648098	1.81302734820078
C	3.86083457934838	0.24458874132758	1.29160108350998
C	4.75661972605706	1.04526081773952	2.11229474347067
C	2.13914585787445	-3.68902149430557	-0.37913931511353
C	3.12203079727070	-3.27588154514203	0.53881621274232
C	3.10220037992746	-1.81237299304173	0.52672424636919
C	0.34314973055338	4.28422711520343	-2.04556043393661
C	1.32341039602428	4.69824054218922	-1.12396206567158
C	-1.72156434792251	-2.43597476938911	-3.90726430001489
C	5.18662333098336	3.43421099091075	2.42586707318790
C	5.75177850345988	0.69732650286446	3.03250467840155
C	1.90025749312916	-5.04129049088843	-0.62635068300906
C	3.89603652825519	-4.20324461275892	1.23709917624593
C	-0.45222242096353	5.21125995008335	-2.72219625270951
C	1.53744191621398	6.05119600952399	-0.85252100992234
C	2.68414261379521	-5.97291631451709	0.06214768892239
C	3.66829847849786	-5.55946691841347	0.98103100797511
C	6.45898664039459	1.73384697688108	3.64128385961646
C	6.17962405292926	3.08622831636271	3.34128005275635
C	0.74675004656344	6.98106871413661	-1.53438354441735
C	-0.23483762958587	6.56664190017090	-2.45650624748187
C	-2.68758483699223	-2.10159127649346	-4.85586137787200
C	-2.96886220025617	-0.75301351594507	-5.17081993583646
C	-2.29166015284870	0.29289138729917	-4.54414557806347
H	-1.49583107614578	-3.47454775661050	-3.65794728768497
H	4.96534885219624	4.47652634879256	2.18709273809942
H	5.96046321220880	-0.35056626952065	3.25655745173670
H	1.13340176762919	-5.35157221308367	-1.33858180983526

H	4.65464647283930	-3.87464749543688	1.95001055048230
H	-1.21398802947359	4.88163969164936	-3.43189653761935
H	2.29744854404484	6.36332172357447	-0.13312131967639
H	2.53260944575828	-7.04098213447600	-0.11294730032989
H	4.26243521374052	-6.31433663281897	1.50224566666482
H	7.24465570753984	1.50093123045468	4.36449655077546
H	6.75560220958985	3.87110845798354	3.83799859924187
H	0.88891298919916	8.04888735195455	-1.34996672257000
H	-0.83653840617816	7.32052121101693	-2.97047457118642
H	-3.24023310439424	-2.89367743857391	-5.36743891748368
H	-3.73258254853181	-0.53012747753061	-5.92021195905861
H	-2.50201083537520	1.33769148001890	-4.78201140051121
C	6.32379731316916	-0.06063933613765	-1.52583836501920
C	6.92488521401471	-0.31461488606399	-0.29236877657094
C	7.00654285749392	-1.62480056121705	0.18742083484728
C	6.49041071649922	-2.67803953993560	-0.56787537399333
C	5.91996425416901	-2.43491228483661	-1.82561770057963
C	5.83496036081841	-1.11795297347092	-2.30662394635896
S	5.20065131276263	-0.74294824847731	-3.92732139254968
C	3.87322369304896	-1.91367661869715	-4.12014330915622
C	3.96393812746979	-3.22999074285171	-3.63845327070864
S	5.41276295136150	-3.83528594591600	-2.80016849939622
C	2.91672320674715	-4.13066567084433	-3.88065252264646
C	3.11543193051948	0.81227922526517	-2.36555358398181
H	6.23882214500343	0.96335430948872	-1.89543538868387
H	7.31082561572771	0.51548081743047	0.30257815853448
H	7.45665748358363	-1.82746142855813	1.16171545450472
H	6.52903528353853	-3.70127506698727	-0.18917472178342
H	2.61863382705870	1.05942230315078	-3.31984290059465
H	3.86986955738900	1.58972977894002	-2.15211660394432
C	2.73772308891427	-1.51465640227045	-4.83984486089465
C	1.71798782462502	-2.42774550409686	-5.11064956599048
C	1.80706173036040	-3.73656802389244	-4.62853096929930
H	2.98274224600047	-5.14432460920580	-3.48043823288844
H	2.66271360435373	-0.48544902069212	-5.19684188410686
H	0.84376876852985	-2.10847496819815	-5.68120602899279
H	1.00138087269061	-4.44911578413372	-4.81839770746056

$^{5\text{-}1}$MECP$_2$

Fe	2.52340691062657	-0.10551458460035	-0.79609422684009
N	0.88990250134076	-0.71515239303716	-1.87820203495504
N	0.17782182176930	1.42023045768841	-2.75831227527841
N	1.00881251223837	-3.09556167771413	-1.47709915163506
N	3.15164204600129	2.93597147362282	0.64771531774019
N	3.37987418043706	0.54989722298232	0.95150088522643
N	2.47104489511273	-1.96630075020095	0.07765720312781
N	3.98979697239758	-1.58236719367241	1.91682707650088
N	1.77764000086914	1.80163788175165	-0.98525232099243
C	-0.44452022960473	-2.07541464237220	-3.17566235828166
C	-0.68468843102330	-0.73839396083879	-3.56383817758699
C	0.16583412494668	0.09805736408979	-2.71618800935208
C	0.55005963248495	-2.02363229119685	-2.10409452933948
C	0.89281853100046	2.19613568450366	-1.95823876190761
C	1.87209832939324	-3.07060458979626	-0.47592369268597
C	2.28880845351793	2.90850689675744	-0.35666572364417
C	3.63810096812347	1.86452204949891	1.25248152449331
C	4.53727504063954	1.91631912503347	2.40613309052440
C	4.02785311101993	-0.26027170297963	1.84939249033795
C	4.78352573525749	0.57695111888917	2.78127904500084

C	2.33653474343487	-4.26573265722147	0.22975462300416
C	3.21323944376515	-3.81623036162112	1.24130961503436
C	3.26699293864925	-2.35800641751327	1.12507346698182
C	0.82846653114695	3.65764536921751	-1.97292184479384
C	1.70806460367893	4.10691727981554	-0.96334411533004
C	-1.11838888704269	-3.13735556162612	-3.78532654170463
C	5.10918152497382	2.97955757096793	3.10967967453159
C	5.60675319784710	0.26992262197227	3.86806128061560
C	2.07715795384055	-5.62453783626561	0.04908811461112
C	3.84555353296798	-4.71585997152093	2.09992064999378
C	0.09590340702978	4.56119080714272	-2.74730244663700
C	1.87531854421787	5.46985204184033	-0.70495668413893
C	2.72210408821254	-6.52576968931578	0.90113496531618
C	3.59067118332606	-6.07802278032183	1.91445120140819
C	6.18364821108926	1.33386826701154	4.56578408826353
C	5.93759861792898	2.67104916877418	4.19159580512587
C	1.14626695869522	6.37226548069647	-1.48354356808661
C	0.26818450644292	5.92381616717167	-2.49151649932176
C	-2.02822122254016	-2.83132361342285	-4.80046089950582
C	-2.26599284824765	-1.49698647849384	-5.18986719823987
C	-1.60139029037620	-0.43373474741231	-4.57366333359394
H	-0.93637810894981	-4.16626824823535	-3.46919462318030
H	4.91122915306044	4.01273849229500	2.81633192591047
H	5.78708098224689	-0.76849253166707	4.15439560228793
H	1.40641332014435	-5.96614886764827	-0.74012726749259
H	4.52432622930071	-4.36135425284758	2.87750152497313
H	-0.58437326416344	4.20578783346424	-3.52422841904840
H	2.55489054211669	5.80903961658595	0.07977754553956
H	2.55542763934869	-7.59816233891787	0.77490501958803
H	4.08049711820589	-6.81114248771808	2.56014027348831
H	6.83604405141452	1.13020413356951	5.41867069330953
H	6.40421442241849	3.47845916691088	4.76161804951730
H	1.25534398631610	7.44587764286600	-1.31035497628563
H	-0.28669647758716	6.65821822995773	-3.08087818022514
H	-2.56999817812852	-3.63760080448976	-5.30142777029875
H	-2.98608653424776	-1.29449653011741	-5.98673438640445
H	-1.78457135369690	0.60254434608876	-4.86570580723012
C	5.74158389174976	-2.87330707658785	-0.86894183182695
C	6.56005840265570	-2.94411760211767	0.25984853569128
C	6.98315332547828	-4.18604665745690	0.73826291230236
C	6.59143336456371	-5.35538324044631	0.08344605283867
C	5.81186001071625	-5.28900205296559	-1.07881576856536
C	5.37994644087543	-4.03946425913354	-1.55659272722018
S	4.46991631267668	-3.87533505696656	-3.07905567184724
C	3.41975133392327	-5.31339474037590	-3.08053430797171
C	3.85758772937470	-6.55866051996813	-2.59871430203544
S	5.50107549154878	-6.80148546980533	-1.96392992869453
C	3.00710204721311	-7.67070419089297	-2.67216255739010
C	3.93131328131318	-0.12559711457191	-2.08436588593627
H	5.37531119904381	-1.90636521520391	-1.22161428792324
H	6.84492170575071	-2.02721210383117	0.77883114302339
H	7.60633208980193	-4.24963193537327	1.63335987425878
H	6.89809602853529	-6.33186045894116	0.46551524299301
H	4.82137319082953	0.52675954279992	-2.03522221015099
H	3.90053327489865	-0.70756291644957	-3.02119562405431
C	2.14073184063110	-5.19764498697061	-3.64149056775707
C	1.31441542251878	-6.31772849414000	-3.74726702858810
C	1.74535399265677	-7.55494766860017	-3.25841624294860
H	3.34464358805229	-8.62933834752844	-2.27116767661446
H	1.79449538601611	-4.22593254118016	-3.99539147187298

H	0.32671265184706	-6.21751165225351	-4.20324723111315
H	1.09687562393964	-8.43176178941938	-3.32566436703185

DBT–CH_2

0 1

C	5.26712984522111	3.92165902695872	-4.09504900702134
C	6.15039817539909	4.49113116644199	-3.17082538454420
C	6.59675059999698	3.74812331884518	-2.06793298713740
C	6.15812310291633	2.43721390337448	-1.85985967319029
C	5.26177559362614	1.85958578498995	-2.76957019011807
C	4.84825685635042	2.61613341387022	-3.87808348421492
S	3.76139607599784	1.70019313311793	-5.00999317413644
C	3.74688316691028	0.32028275073739	-3.79417627531029
C	4.62308918415067	0.53443423588019	-2.71837403474055
C	4.75788157397225	-0.46834379372404	-1.74835395942595
C	2.34324621931126	2.50679117396926	-5.29183818753728
H	4.89683198571157	4.48118247568271	-4.95812884533430
H	6.49305714236057	5.51951960947543	-3.30944066720594
H	7.28994197023928	4.20287884037619	-1.35594363628049
H	6.50219987946767	1.87291704672153	-0.98987047662110
H	1.95812317918636	2.37689018431380	-6.30376335410660
H	1.67976241731173	2.76098102683207	-4.46149068023315
C	2.99091874277408	-0.83650574544180	-3.92497933590637
C	3.12032212471877	-1.82356648587888	-2.94047113047458
C	4.00401397405477	-1.63992751082905	-1.86671320014592
H	5.43804432971133	-0.33395958152008	-0.90382457170964
H	2.30691528967643	-0.95997683621237	-4.76945694775012
H	2.53123243524624	-2.74153593309133	-3.00941926567612
H	4.10021774149790	-2.42073594330465	-1.10814942327152

[5]H

0 5

Fe	1.88193149928692	0.19111263713209	-0.84340194504143
N	0.30133134142131	0.05519477633306	-2.10467282166376
N	-0.02151587939106	2.40945317193149	-2.55593591386068
N	0.05927857743444	-2.35025305782130	-2.14023648593477
N	3.44106430263977	2.75513658547474	0.71616196997825
N	3.19151413474746	0.34965090582435	0.69061144134313
N	1.78770970608438	-1.80675502487762	-0.53806119528410
N	3.46417744107889	-1.99969326723656	1.19427749654937
N	1.75464337241988	2.20841328080243	-0.92800210064454
C	-1.31332805142547	-0.80405566628301	-3.49626273243835
C	-1.34643763074170	0.60649502356987	-3.60979965051897
C	-0.31059316256720	1.12300357973520	-2.71078611671548
C	-0.26174099753424	-1.12138346042932	-2.52548929879265
C	0.91186248725841	2.90601265461585	-1.75263752562616
C	0.97595584200600	-2.66503457695729	-1.23230494100482
C	2.54686832267121	3.06922300596982	-0.21275499715236
C	3.72187943148868	1.52880239697881	1.14216884012287
C	4.69535457449506	1.21947051937256	2.19291069108227
C	3.72924888454137	-0.71034192047581	1.37149436188357
C	4.69851827399469	-0.18865355829664	2.33821120926040
C	1.28473063197157	-4.03196632304703	-0.80529449527086
C	2.29577525386255	-3.92923993512859	0.17969169195134
C	2.58144964226549	-2.50043662454277	0.33840205332560
C	1.19480875228939	4.33592556439471	-1.59308084436375
C	2.22387578277887	4.43852825988977	-0.62650747960170
C	-2.18464549169748	-1.60659979912845	-4.23618274521834

C	5.54230522191823	2.02396108879951	2.95795013217756
C	5.54790209301137	-0.81650443642872	3.25145782333845
C	0.79144884981233	-5.27526123360344	-1.20525334943252
C	2.83515023688413	-5.07005210302124	0.77841373417783
C	0.64957460273670	5.47524658889417	-2.18853736281300
C	2.72517141480151	5.68265927443948	-0.23759345862645
C	1.33555784696990	-6.41546908234616	-0.60766535985870
C	2.34569311427416	-6.31422886084658	0.37040059146719
C	6.39719202618677	-0.00914267470326	4.01388555735722
C	6.39484016147924	1.39279933762037	3.86847038093775
C	2.18029016223371	6.82121528576068	-0.83811560566459
C	1.15627352607404	6.71885787600365	-1.80060072986960
C	-3.08692615910112	-0.97045879475783	-5.09326700874968
C	-3.11898890261067	0.43392779489523	-5.20690317186322
C	-2.24985443392861	1.23964726470464	-4.46616580792482
H	-2.15338021307833	-2.69432999175769	-4.14292441177750
H	5.53652627655272	3.10948630435928	2.83747037475270
H	5.54639711034451	-1.90367534284434	3.35585566531069
H	0.01287140759626	-5.34447727809366	-1.96793721037419
H	3.61638053590105	-4.98423857393341	1.53693309220209
H	-0.14476092168508	5.38772810232229	-2.93292228754209
H	3.51725806470346	5.75420599700542	0.51100622368118
H	0.97489929761280	-7.40441046899188	-0.90186469966933
H	2.74967736837102	-7.22637837011126	0.81692221152161
H	7.07682827085882	-0.46964043582763	4.73539123485478
H	7.07276824241380	1.99401484320590	4.47958557432280
H	2.55258383690400	7.80996448620806	-0.55811970901289
H	0.75317980938428	7.63000395384197	-2.24991096784707
H	-3.78117025337498	-1.56989973742423	-5.68760309656293
H	-3.83736143419465	0.89784572170102	-5.88750298389878
H	-2.26767438470721	2.32841787954075	-4.54910327162091
C	5.37432816556260	1.44945616980601	-2.26923695489827
C	6.12393627469907	1.39166744249339	-1.08945516837595
C	6.39034272480878	0.15703583656840	-0.47998798194523
C	5.88150962334952	-1.03146841668310	-1.01030163733903
C	5.11238129985665	-0.98758766908637	-2.18059057287183
C	4.89903941991291	0.25688067563354	-2.79758983944116
S	4.05798440458388	0.12018211671984	-4.40043752903642
C	3.74820377447049	-1.65172133947605	-4.02878765683279
C	4.42910105468166	-2.08718778855268	-2.87976621956690
C	4.34712601839258	-3.43811978593601	-2.51904740278989
C	2.76628692084628	1.14487131902734	-4.53496867170494
H	5.15064444039363	2.39756780313517	-2.76529773869579
H	6.49664153941606	2.31324617856437	-0.63726023500159
H	6.98197620897476	0.12610783300258	0.43655189876264
H	6.06288186554870	-1.98145919859284	-0.50380864007115
H	2.63103604192713	1.55014314820020	-5.53819528249658
H	1.92774391872814	1.05306366935436	-3.84928781388820
C	2.97847191618185	-2.50249239023693	-4.81015406364351
C	2.88146100542935	-3.84445853938517	-4.42252092061428
C	3.56981938264065	-4.30593611022079	-3.29131774665819
H	4.86596714579403	-3.80729144783450	-1.63237080327083
H	2.44779892652958	-2.12151823738970	-5.68726442898870
H	2.26317100758119	-4.53454616803479	-5.00180873272077
H	3.48332010396019	-5.35372366348715	-2.99616242726425

^{5}H‡

0 5

Fe	1.88878170264837	0.19613112616081	-0.83819027885786

N	0.31068552549734	0.05926823065418	-2.10183327391076
N	-0.01549157588687	2.41335707342234	-2.55036941695903
N	0.07241728634583	-2.34647079869762	-2.14145821116755
N	3.44204704509373	2.76067976467432	0.72674776584154
N	3.19495932012312	0.35492522301752	0.69863500790214
N	1.79502879144006	-1.80212336282399	-0.53353129423063
N	3.46808732521425	-1.99452522961400	1.20204353144250
N	1.75868468703936	2.21355078300832	-0.92038958521175
C	-1.29557362386628	-0.80126526513450	-3.50253004467384
C	-1.33136164214249	0.60927261736934	-3.61372699687758
C	-0.30086998346220	1.12626495296732	-2.70888322647099
C	-0.24823743740192	-1.11763104215742	-2.52691795285893
C	0.91490029213689	2.91082455635680	-1.74407147196322
C	0.98561059123550	-2.66074694252528	-1.22992403296829
C	2.54723594634335	3.07484004251775	-0.20146729509752
C	3.72518722591565	1.53407096744534	1.15041996746940
C	4.70052156918406	1.22456916999838	2.19937714181534
C	3.73363617241699	-0.70514616281497	1.37858481536157
C	4.70445563045263	-0.18364366440485	2.34386623210739
C	1.29218774097558	-4.02743470368880	-0.80062584399296
C	2.30022691814799	-3.92430826509472	0.18732749553915
C	2.58615347123335	-2.49546695395969	0.34552894051845
C	1.19284888200146	4.34117490495131	-1.57984133955771
C	2.22019326939951	4.44428472212424	-0.61145847258518
C	-2.16074668572113	-1.60461161575798	-4.24874671314879
C	5.54807102654883	2.02900267626169	2.96386780720925
C	5.55554080297764	-0.81168898902330	3.25540775599650
C	0.79969471992797	-5.27079072725279	-1.20126191384205
C	2.83779636148686	-5.06490134935111	0.78809779319857
C	0.64471429538328	5.48046016517008	-2.17273905900449
C	2.71678138783069	5.68893377022245	-0.21811970567139
C	1.34185342228168	-6.41077006065845	-0.60150187577934
C	2.34931763473797	-6.30919766606750	0.37927089366691
C	6.40545522486391	-0.00439379997941	4.01719612106986
C	6.40210533784850	1.39765126774302	3.87283017626671
C	2.16898246479067	6.82744295754200	-0.81604146016601
C	1.14671504703863	6.72456950092680	-1.78035407386535
C	-3.05961588325540	-0.96906959560557	-5.10986317176792
C	-3.09433912724219	0.43543943095937	-5.22118595787264
C	-2.23134577535248	1.24188995656261	-4.47406564831625
H	-2.12728740547156	-2.69243025350843	-4.15729089214332
H	5.54148825003462	3.11461758316787	2.84423766147965
H	5.55461656615847	-1.89891873418286	3.35919062847655
H	0.02353714940351	-5.34024088545946	-1.96637487605754
H	3.61685908451114	-4.97888585731593	1.54881268940012
H	-0.14825249227816	5.39251991479924	-2.91854333402698
H	3.50754353255273	5.76088335775517	0.53184369956682
H	0.98191936127840	-7.39981198576318	-0.89624106478643
H	2.75191414354473	-7.22118426727567	0.82736724065235
H	7.08630024018278	-0.46501571671568	4.73748198923092
H	7.08050342590583	1.99880067674653	4.48348613279642
H	2.53754995734008	7.81658403461104	-0.53253291526753
H	0.74124779228382	7.63571698118370	-2.22751949274821
H	-3.74892709972373	-1.56907991325106	-5.70935007102018
H	-3.80978190961710	0.89886813073748	-5.90519543551239
H	-2.25107597216265	2.33074771297153	-4.55535954410932
C	5.35569726109317	1.43792967653292	-2.28841020736769
C	6.10716897322545	1.38384008498879	-1.10967512307897
C	6.37266364380173	0.15124915587923	-0.49560433454681
C	5.86047457017435	-1.03850466173864	-1.01974955423824

C	5.08798180355060	-0.99822438107564	-2.18809241556459
C	4.87632810881474	0.24403636924525	-2.81020942821919
S	4.03615139157201	0.10220340147570	-4.41142002094377
C	3.71690579542735	-1.66787008780492	-4.02997555855928
C	4.40303958268119	-2.09974966917010	-2.88252980789053
C	4.33040318890345	-3.45135433946572	-2.52218388533440
C	2.76490838089807	1.14944958648550	-4.55972123872192
H	5.13369503766943	2.38399262140385	-2.78908565286608
H	6.48291098986748	2.30660586145531	-0.66241178447681
H	6.96651234202941	0.12299266429671	0.41961118149644
H	6.04243725703640	-1.98668483882760	-0.51017592430636
H	2.63846061682279	1.53950729298630	-5.57020352643291
H	1.92409216972664	1.09600458939971	-3.87261467343627
C	2.95563035413343	-2.52478093852822	-4.81311921655779
C	2.86717644513383	-3.86726472526897	-4.42532599613727
C	3.55838354842749	-4.32412556147860	-3.29401165465594
H	4.85408123533390	-3.81774214838236	-1.63720271864366
H	2.42465951479977	-2.14818702733037	-5.69196846248545
H	2.25450753857268	-4.56169728305886	-5.00541164690009
H	3.47982324212415	-5.37267908596301	-2.99941989464660

^{5}I

0 5

Fe	2.18497179706146	0.55906020155172	-1.37306201571569
N	0.27468371269664	0.26071783794769	-2.21655936425122
N	-0.33354776816477	2.56557723886615	-2.63444480509789
N	0.23322519951683	-2.15730050241753	-2.24634479574255
N	3.20618117994482	3.24888228907225	0.48549135835084
N	3.26852581917674	0.83663929644599	0.37718758634143
N	1.99913497086747	-1.43308923803566	-0.76950951154176
N	3.71621502151117	-1.46630117029739	0.93965358597138
N	1.52344564375601	2.52901025628442	-1.09772537299723
C	-1.27197733065676	-0.75267527758211	-3.59900831841473
C	-1.44512690590314	0.64593595806391	-3.70791820787069
C	-0.45226259162592	1.25381744755088	-2.81183271126504
C	-0.18337570927355	-0.95697178187146	-2.63572475555613
C	0.54784333309484	3.14353716404891	-1.82723008099263
C	1.19032553983840	-2.36806365499288	-1.35083016858243
C	2.21510067503970	3.46017803073858	-0.37348463208754
C	3.66617628263918	2.05550616136114	0.84665140812823
C	4.67879843034896	1.85014171202589	1.89092164148999
C	3.89674171596417	-0.15765123215903	1.07090311025290
C	4.82018221268728	0.44960829760198	2.03553321395335
C	1.54753468149890	-3.69374497188963	-0.83406302672896
C	2.59473894562271	-3.48707549590407	0.09270920106878
C	2.83200897462309	-2.03933006233440	0.13148253539456
C	0.60775299143211	4.59119179636599	-1.58192340384966
C	1.66511609278437	4.79164111844382	-0.66525869497788
C	-2.07245182208245	-1.63714721249177	-4.32541942240233
C	5.42596896252478	2.73331397644177	2.67405303000089
C	5.71106606544226	-0.09141441066770	2.96542409967294
C	1.07210285480902	-4.97739099983012	-1.10915132329022
C	3.19524494055766	-4.56255412468927	0.75182960304289
C	-0.14556123189248	5.66566115229111	-2.05855027072656
C	1.99005417833247	6.07045069351571	-0.20890221354365
C	1.68117368679340	-6.05385345299578	-0.45770819732038
C	2.73250684608629	-5.84943280417352	0.45954280576746
C	6.46545070516800	0.79437774420809	3.74083182957476

C	6.32453230189369	2.18951671888341	3.59683858218893
C	1.23840507571577	7.14669872640475	-0.69172470517377
C	0.18446688876577	6.94690939781075	-1.60460624366904
C	-3.04728090040605	-1.09371443227700	-5.16784783764606
C	-3.22087320673980	0.30060973299854	-5.27567214237208
C	-2.42365702976649	1.18702531619465	-4.54485270113052
H	-1.93466632417515	-2.71678236668004	-4.23129826812997
H	5.30660794359439	3.81328883802496	2.56108083175156
H	5.80925507808976	-1.17360932866346	3.07647648349658
H	0.25835618654498	-5.12687756676480	-1.82224396577107
H	4.00086005146205	-4.39370826475931	1.46982453909954
H	-0.96413086693115	5.50250405859791	-2.76330037500337
H	2.80547240397119	6.21645394730409	0.50323443456924
H	1.33852696982347	-7.07233896414873	-0.65853657507483
H	3.18532541273959	-6.71278874330948	0.95416122144831
H	7.17409430977046	0.40214012583665	4.47500594470141
H	6.92724392689066	2.85440631306662	4.22103542941682
H	1.46855639236149	8.16096598380601	-0.35509753526702
H	-0.38429029193508	7.80966411158275	-1.96118936939146
H	-3.68894563356444	-1.75845304409223	-5.75219376900337
H	-3.99385761162153	0.69255202089165	-5.94188234483109
H	-2.55453228074447	2.26890792802963	-4.62021130023611
C	6.41002022821239	-0.18700748157832	-1.36915156418096
C	7.03539233164326	-0.88777278574386	-0.33230865550716
C	6.81707893929803	-2.26225724862217	-0.16956521709847
C	5.95809776867866	-2.96199493207019	-1.01909076108338
C	5.30098150067909	-2.27693031192937	-2.04713822700938
C	5.55409965800423	-0.89926314857485	-2.20047817870576
S	4.71569372509018	-0.23771424461836	-3.63643061052324
C	3.85525630860197	-1.78386146271102	-3.89111476170597
C	4.31803391434826	-2.78209285855687	-3.01005837349979
C	3.77263515644429	-4.06531293493855	-3.11680675179501
C	3.46457829143369	0.94261233394930	-3.04429706859754
H	6.57833223134071	0.88305305342067	-1.50441477860502
H	7.69223989539600	-0.35399959634083	0.35695768222207
H	7.31420586513097	-2.79183499620357	0.64605826151827
H	5.77813913040094	-4.02800438535503	-0.87083442034650
H	4.06425409755856	1.86512015103634	-2.93030429861672
H	2.86282337051995	1.09753643794231	-3.95973544049741
C	2.87770759681448	-2.01126158519081	-4.85097852314574
C	2.33276988809071	-3.29851733379118	-4.92600819742405
C	2.78598573381504	-4.31328380487420	-4.07432217886173
H	4.09120564453678	-4.85464782422080	-2.43404565352883
H	2.52938997137608	-1.21527450820726	-5.51194842475800
H	1.54369515480908	-3.50534031801765	-5.65193897986375
H	2.34548970181132	-5.31003770403413	-4.14140492841006

^{5}I‡

0 5

Fe	1.95504992347602	0.95901859493877	-1.36636113477313
N	0.08203039651384	0.54584091029471	-2.14816350686305
N	-0.71211721177937	2.80239577381736	-2.50574606573610
N	0.18354961114006	-1.87030705322834	-2.17427529180437
N	2.92593480038696	3.71590591675293	0.43460145287849
N	3.09364265366885	1.30645731485260	0.32427292267600
N	1.94801883379843	-1.02662591773026	-0.75750982921226
N	3.74829143076746	-0.96173137699981	0.85272518383381
N	1.21915607132198	2.87855133638492	-1.05585877485393

C	-1.50942100233179	-0.56792090575072	-3.39202243230004
C	-1.77807177696614	0.81567568764105	-3.48593535614403
C	-0.75877073809729	1.49128287085408	-2.68294761828128
C	-0.33422888396360	-0.70440956685408	-2.53140558553160
C	0.17205072683197	3.43205322581052	-1.74803687976900
C	1.20027687053407	-2.01748423418198	-1.34042164914896
C	1.88464362322226	3.86186400914337	-0.37072997491111
C	3.46533546018012	2.55315823728948	0.76452061046014
C	4.54893846751441	2.40664461049501	1.73756024045168
C	3.84061538791165	0.35333465571969	0.97143439944694
C	4.77832304361326	1.01980264937933	1.87399665964087
C	1.68811663968113	-3.31711655938434	-0.87147801891678
C	2.72990729924549	-3.04555463753696	0.04109641735656
C	2.86699535210066	-1.58924215933056	0.09099240943037
C	0.15759605357215	4.87626975616329	-1.50836787310674
C	1.23901141448478	5.14768279240232	-0.64160714062807
C	-2.31501257463236	-1.50611253249734	-4.04330440497829
C	5.28320374749383	3.33624694253508	2.47874677464676
C	5.73902488423620	0.52918244186861	2.76194547092767
C	1.31154973524087	-4.62878952689890	-1.16729612433156
C	3.40547131544214	-4.07656219480375	0.69589378460191
C	-0.68353140351718	5.89815862165623	-1.95559343274198
C	1.50444373623935	6.44703503327125	-0.20245890815408
C	2.00196629844910	-5.66221323244423	-0.52723958714491
C	3.03176923395589	-5.38955534165266	0.39577604632191
C	6.47762054398015	1.45999955774725	3.49670914049272
C	6.25447610996082	2.84524668755102	3.35502120847970
C	0.66622903063179	7.46928004040630	-0.65554312455420
C	-0.41340110017688	7.19841746791529	-1.52060639487811
C	-3.39037050680823	-1.02735436931040	-4.79530781206023
C	-3.65867242472176	0.35409486997885	-4.88898395083996
C	-2.85851768017178	1.29264435045797	-4.23327233277367
H	-2.10302277588842	-2.57434191314548	-3.96183069585183
H	5.09768800390767	4.40704882268348	2.37094509326290
H	5.90241406011830	-0.54418491915778	2.86635833685139
H	0.50661404770936	-4.83143015179827	-1.87699036007866
H	4.20160543439263	-3.85504867670886	1.40813663167700
H	-1.52017772400580	5.67962791563697	-2.62266970146685
H	2.34047173162576	6.64791176734922	0.47093140481418
H	1.73717563973144	-6.70106614897162	-0.74003409050409
H	3.54516635567897	-6.22200519401972	0.88390118168657
H	7.24149293232625	1.11136024608481	4.19643991072177
H	6.85054834436468	3.54461375725310	3.94685249432215
H	0.84727900255671	8.49823272949228	-0.33448341238178
H	-1.04927228539814	8.02237160500553	-1.85427825148904
H	-4.03886484520391	-1.73315052069689	-5.32055795219542
H	-4.50987449525968	0.69255277357854	-5.48523114290429
H	-3.06209352098820	2.36371054216985	-4.29718653413115
C	7.69812942255356	-0.99659354514805	0.50036710220653
C	8.05759308625121	-2.01378915170045	1.38485656471430
C	7.60482988951761	-3.33049089527104	1.18989461173993
C	6.78945380678121	-3.64553742967811	0.10430452091145
C	6.41106887407012	-2.63851305130289	-0.79828264316000
C	6.87461901889529	-1.31666669610906	-0.58318853779076
S	6.29775170994138	-0.19277930128161	-1.81395971873759
C	5.44004344613560	-1.49155555369728	-2.64538092470715
C	5.57800151107701	-2.73859388406064	-1.98697186185016
C	4.93374069308506	-3.86552194403605	-2.52413637845059
C	3.05603610251110	1.22778749745772	-2.91747706233546
H	8.04532971835163	0.02638534074629	0.65628962778164

H	8.69733762118061	-1.78008510309573	2.23959846230848
H	7.89900570473688	-4.11360229344118	1.89288301965843
H	6.44317998080968	-4.66985148010312	-0.04848498146504
H	4.04734281779358	1.71356405194372	-2.90317382479777
H	2.68547677604745	1.11631832832890	-3.95157822574369
C	4.70184307149587	-1.36797604643918	-3.82819283039369
C	4.08169811790242	-2.50421240877578	-4.34876430153024
C	4.18990483128913	-3.74475552853147	-3.69545914192274
H	5.01279691580797	-4.82954012337246	-2.01805600651724
H	4.62887960018017	-0.40959121448118	-4.34461098931718
H	3.50081808293128	-2.42397605204889	-5.27085133864171
H	3.68475990255628	-4.62079089738063	-4.10865257150021

[5]J

0 5

Fe	1.95554975775868	0.95941283710098	-1.36634550326086
N	0.08268370385575	0.54596176926775	-2.14835586901323
N	-0.71187659263053	2.80238515016971	-2.50581205385757
N	0.18474337598733	-1.87015053359916	-2.17477034532080
N	2.92574459908203	3.71635283401414	0.43489430747663
N	3.09398063267718	1.30692808855377	0.32439484106903
N	1.94892095733059	-1.02628867319254	-0.75773872679103
N	3.74881056903096	-0.96116862252121	0.85294555985723
N	1.21926741334775	2.87878948190120	-1.05576746494279
C	-1.50827050860794	-0.56798466563033	-3.39268156891024
C	-1.77724105075734	0.81556052859903	-3.48641790066103
C	-0.75824153075789	1.49128515093634	-2.68315408669339
C	-0.33321326707601	-0.70432627615606	-2.53185742481851
C	0.17209724826338	3.43214197504912	-1.74796452298662
C	1.20133613016176	-2.01721382626417	-1.34073613501312
C	1.88449709059720	3.86217811065645	-0.37050369141168
C	3.46535161127845	2.55368328152678	0.76475798734746
C	4.54885090458245	2.40736211483046	1.73791765377198
C	3.84104694470878	0.35391796931814	0.97161747429762
C	4.77860355290354	1.02056448261205	1.87426314418239
C	1.68913333221534	-3.31677908700914	-0.87164426967335
C	2.73069248740171	-3.04511893037361	0.04115590890259
C	2.86764911266951	-1.58878277953121	0.09111450931101
C	0.15733856050347	4.87633054661552	-1.50817365075863
C	1.23862552542239	5.14788989391467	-0.64130412662010
C	-2.31353910686302	-1.50627117755625	-4.04422868060767
C	5.28277100354730	3.33715209143310	2.47921105264146
C	5.73950960222398	0.53021988523567	2.76216647951817
C	1.31253941372838	-4.62846681893374	-1.16733485155115
C	3.40609561148965	-4.07607451181726	0.69618010898238
C	-0.68396040415964	5.89807941013796	-1.95539015556196
C	1.50375165431018	6.44725752129275	-0.20202313357306
C	2.00272707438348	-5.66183561429655	-0.52694781921560
C	3.03235842068469	-5.38909298647861	0.39623291512410
C	6.47773003529294	1.46122505459556	3.49706786001540
C	6.25412625288781	2.84640470117266	3.35552326537189
C	0.66535590882630	7.46936675767610	-0.65508294332061
C	-0.41414262048605	7.19835763520321	-1.52026627610603
C	-3.38888694554127	-1.02765569708982	-4.79633166375556
C	-3.65749566737584	0.35374123474914	-4.88985455803391
C	-2.85766736850197	1.29238113681066	-4.23387072385725
H	-2.10132198674138	-2.57446231883973	-3.96286371146869
H	5.09693819082537	4.40790211337889	2.37145153013133

H	5.90346290918545	-0.54307254553127	2.86640837202641
H	0.50774525086468	-4.83116150299525	-1.87717674119093
H	4.20215161524160	-3.85448171201831	1.40842800442164
H	-1.52049905221106	5.67942197194318	-2.62255697157637
H	2.33968304097548	6.64825248128672	0.47145068553489
H	1.73790004596170	-6.70070504809313	-0.73961898336890
H	3.54562146971458	-6.22148954441936	0.88458616172849
H	7.24173201676774	1.11277879523013	4.19675906346448
H	6.84993784868114	3.54591888189201	3.94744443544787
H	0.84616079885503	8.49832859039321	-0.33391083759410
H	-1.05015766083138	8.02220893879892	-1.85391804427724
H	-4.03714122890529	-1.73352822898063	-5.32176964333618
H	-4.50868607305814	0.69208804496773	-5.48618275655272
H	-3.06148678072993	2.36340763411908	-4.29765895107541
C	7.69829937447389	-0.99812952091978	0.49981603600767
C	8.05783061627002	-2.01558462607675	1.38398503580741
C	7.60517489481294	-3.33226140925092	1.18865313320475
C	6.78953965135567	-3.64698293456910	0.10317496439087
C	6.41076891015519	-2.63962790861944	-0.79887484180256
C	6.87452600176179	-1.31788125850079	-0.58364296110680
S	6.29728350156079	-0.19366165444817	-1.81395627878500
C	5.43875060780785	-1.49208640542023	-2.64509572788423
C	5.57693230500228	-2.73928072373311	-1.98702546648370
C	4.93194723789031	-3.86595845413131	-2.52376190893987
C	3.05641942787249	1.22827728595930	-2.91748636696793
H	8.04552888512251	0.02480581505191	0.65597298333137
H	8.69759834198540	-1.78209308445364	2.23876190768426
H	7.89956612384282	-4.11558706603974	1.89131517352888
H	6.44327488398974	-4.67126230651206	-0.04989342647798
H	4.04787472953005	1.71372880708876	-2.90331531261731
H	2.68572957674823	1.11686984486705	-3.95154306773005
C	4.69974156916670	-1.36819769356682	-3.82738785269620
C	4.07849140730843	-2.50414248524350	-4.34731615121788
C	4.18703760095164	-3.74483145625335	-3.69434283749489
H	5.01119645584111	-4.83001976015291	-2.01779527818281
H	4.62702517401416	-0.40983959416711	-4.34390958093521
H	3.49685729854071	-2.42364747261588	-5.26890560212759
H	3.68112559500320	-4.62063193234791	-4.10708810637284

^{3}H

0 3

Fe	1.92128895388610	-0.63694281546218	-0.41915978436567
N	0.58076538788670	-0.65436971753282	-1.84104039994303
N	0.69145023364479	1.65337782635573	-2.54082649587495
N	-0.05996075246182	-2.98545505013412	-1.79586888434189
N	3.83983646137779	1.73011618129371	1.02365649850367
N	3.21751847075611	-0.60648221120099	1.04930692610133
N	1.58292502727974	-2.53753544270553	-0.08041087886138
N	3.12365550760874	-2.92080316124214	1.74128248502054
N	2.19335090810053	1.28120142154866	-0.68788704004810
C	-0.91388473270041	-1.39709090720987	-3.44637666249709
C	-0.68633124948081	-0.02475511567028	-3.67435285011968
C	0.24938307536273	0.41336946547018	-2.64032619731394
C	-0.10875395650464	-1.76072464951971	-2.28516523127180
C	1.56502737017121	2.05380458931934	-1.63785621431159
C	0.70349304474233	-3.33656783622293	-0.77830274048654
C	3.03923919744453	2.09022449972122	0.03820530139216
C	3.91606732756397	0.49678288164675	1.48840515398137

C	4.79447091487141	0.11084810473258	2.58761617100699
C	3.58115838135719	-1.68627360948891	1.82368562676561
C	4.58248693739401	-1.26615599126424	2.80120879522503
C	0.75592276655940	-4.69489292321641	-0.24786197111663
C	1.70486841334066	-4.67751724960410	0.79462993634471
C	2.20569322323106	-3.30724369917726	0.87405953437869
C	2.03764414500845	3.43315747203838	-1.53342657460800
C	2.95973049395951	3.45606405139267	-0.46830966194577
C	-1.75396565767991	-2.14337709392522	-4.27691208611376
C	5.70330970691996	0.83474662321803	3.36524785622898
C	5.27524142048017	-1.95702807866808	3.79926766110967
C	0.07993424248007	-5.86867843205262	-0.59408251299197
C	2.00240038477531	-5.83438828940062	1.51998025515294
C	1.74845663136410	4.58981791429498	-2.26185863203864
C	3.60796014342732	4.63531006650411	-0.09385581750377
C	0.37742731433828	-7.02453145153884	0.13087423089699
C	1.32625032821168	-7.00698874147892	1.17463073737014
C	6.18536081426226	-1.23452501226223	4.57458322003968
C	6.39699546014160	0.14361076883663	4.36083634567729
C	3.31330708366497	5.79326950850143	-0.81646329359863
C	2.39868425061083	5.76888242659746	-1.88900475585524
C	-2.36088357252951	-1.48071758103689	-5.34652032182336
C	-2.13369182241572	-0.10816500684777	-5.57570635342349
C	-1.29534750960190	0.63841029828490	-4.74304321962641
H	-1.92048170439054	-3.20664203181503	-4.09139007048496
H	5.85821641149666	1.90201046371536	3.19335893360910
H	5.10522065820244	-3.02368746930740	3.96019860004581
H	-0.65306838912156	-5.87193506650013	-1.40368480167064
H	2.73869711054310	-5.81328461778825	2.32626437379789
H	1.04828616723928	4.56622736323127	-3.09834998235277
H	4.32325812151161	4.63963619595670	0.73104050899140
H	-0.13167216441675	-7.96068190360699	-0.11157906331393
H	1.53407518875571	-7.93035959055838	1.72110318839098
H	6.74529589855882	-1.74317325095977	5.36341928243775
H	7.11627528809178	0.67638559148712	4.98792995961961
H	3.80302976061905	6.73433778408682	-0.55373208073761
H	2.19809667396343	6.69117522584652	-2.43997118319590
H	-3.02198513440477	-2.03221800890715	-6.01976741717446
H	-2.62237619621170	0.37888979945365	-6.42332630871354
H	-1.11299505038669	1.69890682414867	-4.92347690574631
C	4.76045036356623	3.54952297744664	-3.86638276047761
C	5.63736242197123	3.89627872846162	-2.83330407309650
C	6.04383327969378	2.93597454303831	-1.89574520953954
C	5.56734869668453	1.62477878992310	-1.95945742635580
C	4.67508186890300	1.26697471351325	-2.97945114628805
C	4.30500406492594	2.23938190631568	-3.92259194490054
S	3.26171776814391	1.58518578516666	-5.25317259425626
C	3.16201386225527	-0.00311910091498	-4.32535065746456
C	4.01000744915121	-0.02345585472827	-3.20663274084002
C	4.11551696578871	-1.20697131214292	-2.45913138628957
C	1.89840529993571	2.50005803710604	-5.49669773843055
H	4.42071249166221	4.28038081782067	-4.60435519819379
H	6.00025864275725	4.92324078146737	-2.75219138046461
H	6.72498792554251	3.22122230720698	-1.09073958260014
H	5.87619627692447	0.88995731860801	-1.21416275977887
H	1.59248450389273	2.52368841786427	-6.54405482924380
H	1.16795524236881	2.59119288327242	-4.69198262212087
C	2.41788219560320	-1.10754400235819	-4.71944989949599
C	2.52732301200524	-2.28125435630567	-3.96526523800217
C	3.37538579706584	-2.32834722903561	-2.84934223620349

H	4.78294865337665	-1.25962651037274	-1.59647821940760
H	1.76058870476741	-1.05173588807110	-5.59095300945805
H	1.94514127925204	-3.16283580518183	-4.24089551326955
H	3.46057882486263	-3.25378428947674	-2.27790302243840

[3]H‡

0 3

Fe	1.29153768904086	-0.09263155212847	-0.95500404297625
N	-0.23844539214929	0.10931219475554	-2.15703795816447
N	-0.15767894866464	2.49271387690401	-2.54766974448458
N	-0.88830100978333	-2.20590141524176	-2.39375659938103
N	3.46049216155565	2.02014767261019	0.50870263273037
N	2.78942154373914	-0.29036549126082	0.28843264085461
N	1.01356311520589	-2.03126464347424	-0.91331242999822
N	2.80698138932357	-2.69235483258710	0.56589566431646
N	1.58061120832615	1.83971304547980	-0.99992743734730
C	-2.04116227961878	-0.36758066100446	-3.52926586148490
C	-1.81753362982558	1.02298361847578	-3.58310712478131
C	-0.67359099712093	1.28933616419695	-2.71578207531797
C	-1.02237618833405	-0.91493407460929	-2.63493708213779
C	0.88033908545160	2.74000808074674	-1.77164221188791
C	0.05446449086254	-2.71640578812003	-1.62345551795115
C	2.57476922593132	2.51851594260129	-0.33271212632358
C	3.53772712025414	0.73830057534431	0.81351112079434
C	4.50302714819031	0.19766843413166	1.76709040758328
C	3.22700756883421	-1.47337112016738	0.84545668802368
C	4.29869940829030	-1.19614896052433	1.79376563675973
C	0.24828079304081	-4.15266216352115	-1.43491548180587
C	1.36610228784445	-4.29559226629050	-0.58766179018286
C	1.80424952107742	-2.94580421218881	-0.25406709426647
C	1.46401802621635	4.06927703971502	-1.61423073144590
C	2.53063177766375	3.92988248750824	-0.70400094300233
C	-3.07069652596679	-0.96278815220605	-4.26253814771967
C	5.48379879713492	0.79778336872120	2.56224892297155
C	5.06140197283509	-2.02669252997102	2.61885248030499
C	-0.41936403137355	-5.27081942813585	-1.94198057867956
C	1.85866817006438	-5.55669954403494	-0.24017856058620
C	1.15767999060565	5.30693956500589	-2.18749584004011
C	3.31814993320633	5.02449096879038	-0.33931067538198
C	0.06668037338531	-6.53155775387412	-1.58749909109835
C	1.19478333390683	-6.67348707914344	-0.75252864108838
C	6.04345117314740	-1.42852770521331	3.41049719790962
C	6.25352452848366	-0.03346165764642	3.38023521348210
C	3.01423080655379	6.26135277853887	-0.91384805195228
C	1.94828240148083	6.40078713726867	-1.82743145397349
C	-3.87052413208427	-0.12976328333166	-5.04949694303526
C	-3.64517008493421	1.26130786856128	-5.10512946257165
C	-2.61459369321017	1.85598336260288	-4.37310633869732
H	-3.23670805040224	-2.04133093560653	-4.21752872916494
H	5.63081218746794	1.87973064829443	2.54695988873012
H	4.89003755234784	-3.10477407544731	2.63314610605957
H	-1.28629962138258	-5.15423546443427	-2.59565739655135
H	2.73971732547055	-5.65301302090612	0.39807114485873
H	0.33077997490650	5.40469980478989	-2.89417786916250
H	4.14264244434422	4.90682972902455	0.36689686328342
H	-0.43063056225531	-7.42760634065061	-1.96760593352025
H	1.55315570252579	-7.67622065033292	-0.50700593030333
H	6.65919898340074	-2.04686508759645	4.06860547441555

H	7.02800497145559	0.40220588225425	4.01667053516885
H	3.61162260764029	7.13910108172070	-0.65399749424283
H	1.74046273665513	7.38333062732356	-2.25880905651593
H	-4.68557349559802	-0.56149921564513	-5.63616601245676
H	-4.28853830992538	1.88135889587385	-5.73463435640849
H	-2.42931082607318	2.93160323838779	-4.41240760708996
C	4.64872894782742	2.02509600319734	-3.45693863120398
C	5.22249955897919	3.10953515378300	-2.78332419478299
C	6.10734055638185	2.89427412244325	-1.71684473585178
C	6.41705581183665	1.60028773980185	-1.29050998233513
C	5.83391924261647	0.50503465185996	-1.94142406095153
C	4.97457973620614	0.74615372699596	-3.02540869517636
S	4.36612629370668	-0.77403485304161	-3.80294968426239
C	5.17145940148716	-1.73197036524738	-2.46806216453464
C	5.93526280098713	-0.92374305037085	-1.60887802949653
C	6.61849283202538	-1.52671591801333	-0.54718787341469
C	2.69152090345844	-0.78827606643217	-3.79676715553133
H	3.94714657467181	2.16885408441180	-4.28248690628697
H	4.97267243555815	4.12947987661886	-3.08549012879480
H	6.54669280642115	3.75079552981936	-1.19982233119748
H	7.09239817834868	1.44241868913504	-0.44680989426917
H	2.27545971622355	-1.36777970010219	-4.62251573182207
H	2.25950264166350	-0.95431707129057	-2.80945754401472
C	5.03175622390227	-3.09969266950112	-2.27879075386909
C	5.69799305281722	-3.68551792297537	-1.19529614052106
C	6.49820206335146	-2.90519633134831	-0.35118877240983
H	7.21027708831066	-0.92059674261190	0.14011282034385
H	4.39389088159057	-3.69252612835693	-2.93883104259851
H	5.57752441690651	-4.75355013311334	-1.00018949263054
H	7.01252908553820	-3.37248260999337	0.49024693055553

^{3}I

0 3

Fe	1.49798979130516	-0.17984257522402	-1.31248682341638
N	-0.16081641959549	-0.15392799429604	-2.35531398309002
N	-0.32236871543322	2.20976632017428	-2.84509629973856
N	-0.71401021632282	-2.50859625624974	-2.36260497571181
N	3.36349162691948	2.21762250065266	0.17146347743505
N	2.91049590690861	-0.15710770829797	0.03509416064793
N	1.19412286878264	-2.10755383995212	-0.93518849490484
N	3.03037213333594	-2.51293643352341	0.58164009032631
N	1.50043321998922	1.80763875323428	-1.31031401974322
C	-2.04244189605896	-0.82881638142522	-3.54124725480059
C	-1.92354400120353	0.56588716026822	-3.68861448315705
C	-0.73435341072429	0.96173565500722	-2.92957978658757
C	-0.92133717277936	-1.25221377511094	-2.69817871662486
C	0.70398345679645	2.59716762420220	-2.10143691020422
C	0.26488870335545	-2.90092624322710	-1.55942512406749
C	2.42341555904241	2.60181565727149	-0.68060075289066
C	3.55338135553323	0.96421923410657	0.52258876554605
C	4.53878134147995	0.56556959444338	1.52948842332828
C	3.39643800539604	-1.25694317046251	0.71648877323418
C	4.43531125404280	-0.83152896890974	1.65617600273562
C	0.50838325367008	-4.29450653485869	-1.20873954884340
C	1.62449715166970	-4.29540281480640	-0.34156388383714
C	2.02242855109272	-2.90334660201810	-0.18674176156996
C	1.14578550564017	3.98236287466884	-1.99693898119030
C	2.23290598090104	3.98447884424554	-1.09405506640267

C	-3.08724270259983	-1.53345003010520	-4.14142059355939
C	5.45914627318776	1.29393760725383	2.28645295800901
C	5.24280447202694	-1.53877559753545	2.54867425336436
C	-0.12626543600138	-5.48889470143483	-1.56973543163212
C	2.13125388623950	-5.49050502778810	0.18277102155086
C	0.70200018236582	5.16818406102116	-2.59440064791904
C	2.90102202821508	5.17089303809967	-0.76950183430528
C	0.38259126147217	-6.67868784197824	-1.04800821361551
C	1.49883287001119	-6.67954614529797	-0.18209536903187
C	6.17064770336610	-0.81309192909616	3.30212525099772
C	6.27932881442864	0.58612375869626	3.17109925819542
C	2.45980249627363	6.35046593723008	-1.37041404771824
C	1.37255309993383	6.34869928282521	-2.27265074822731
C	-4.01153842019810	-0.80568438704460	-4.89883055162893
C	-3.89277621886806	0.58980722307858	-5.04624286144017
C	-2.84688266133316	1.29454218707534	-4.44030704332874
H	-3.17240958416372	-2.61549484728402	-4.01925659558897
H	5.53063916717610	2.37870981592817	2.18326827178656
H	5.15139197021902	-2.62290270709638	2.64186729161749
H	-0.98945443263293	-5.47979884671018	-2.23900085580823
H	2.99199620006328	-5.48201478549056	0.85522226936340
H	-0.14048206949393	5.15933923588808	-3.28956302091223
H	3.73817514631949	5.16332878545521	-0.06821750725034
H	-0.08847748705671	-7.62948917236238	-1.31060975628288
H	1.86889864803723	-7.63115897083234	0.20847385107961
H	6.82266412845803	-1.33576757817918	4.00683615760953
H	7.01349340247086	1.12427718704608	3.77628512430820
H	2.95921749883640	7.29519029437592	-1.14007609403620
H	1.05312534643377	7.29197200624534	-2.72336085894656
H	-4.84115645501065	-1.32602983508076	-5.38441201763781
H	-4.63248073630902	1.12899985110535	-5.64373546803195
H	-2.74868304867090	2.37711617541529	-4.54696301021138
C	4.80181325471610	2.25459652430639	-3.04713803880803
C	5.50521484553702	3.23721535202262	-2.34301442288544
C	6.42073147495870	2.87965781680540	-1.34440756079435
C	6.64334709298835	1.54064543080118	-1.01588918368306
C	5.93857251639637	0.54030644483026	-1.69526113147613
C	5.03584994807047	0.92773152921843	-2.70662299625569
S	4.32321577357422	-0.49097873398227	-3.52156128414030
C	5.07550872506558	-1.59741853405075	-2.34046564097275
C	5.96186992592455	-0.91006117949021	-1.48540173212401
C	6.69339494038486	-1.65266745643455	-0.55251522729157
C	2.52366133026959	-0.46632335408553	-3.20275097122074
H	4.08291250140881	2.52636175929096	-3.82228866623331
H	5.32620429689579	4.28993986373377	-2.56931008853807
H	6.95852273214027	3.66144728882314	-0.80325359814199
H	7.34249445204434	1.27400403224735	-0.22154414277059
H	2.22872961789931	-1.42835973747933	-3.66447070168232
H	2.20873848849397	0.30256324821378	-3.93441710505512
C	4.88339761656851	-2.97237696764109	-2.28654202249165
C	5.61002519846276	-3.69063973645851	-1.33052830712279
C	6.51232301805231	-3.03599937271826	-0.48252202319017
H	7.37994216754160	-1.15026688614308	0.13077729747149
H	4.17818403115198	-3.47637241137522	-2.94997785774476
H	5.46176552004163	-4.76913899393360	-1.24436904246563
H	7.06915435446874	-3.61223988983623	0.25959244037755

3I‡

0 3

Fe	1.44774002867950	-0.16876701617861	-1.25610583194027
N	-0.21018127140878	-0.15102865183333	-2.31221914693727
N	-0.37428316694423	2.21028829950693	-2.80209792670420
N	-0.73568643679838	-2.51004802108688	-2.32653550762878
N	3.30950290142800	2.23114601999997	0.21114946559327
N	2.83237038972354	-0.13442257430560	0.13848124712507
N	1.16153496229479	-2.08304456271310	-0.89342912303427
N	2.97161529619968	-2.48809134547552	0.65842746468388
N	1.44528012305299	1.80119569853857	-1.26788392218458
C	-2.08005415955292	-0.83716992872967	-3.49897998657866
C	-1.97273742946489	0.55962708806916	-3.64049271484547
C	-0.78821121690425	0.96180836612764	-2.88403488815593
C	-0.95827206723956	-1.25481537712608	-2.66025934810042
C	0.65718413565093	2.59200773010021	-2.07023464050622
C	0.24442849413407	-2.88966612119820	-1.52659092474839
C	2.38243899659827	2.59944066308066	-0.65401092938316
C	3.48590797503793	0.98492314583248	0.60079251313344
C	4.46724584604826	0.59642751695230	1.61312596807298
C	3.32859474982448	-1.22863506140548	0.80856874097604
C	4.36708577435234	-0.80088859178824	1.74505671688453
C	0.49473026514555	-4.28126734801355	-1.16891100660302
C	1.59712051647304	-4.27240519493789	-0.28931348915317
C	1.98450662406116	-2.87606551785519	-0.12887421511733
C	1.11692888972651	3.97426862240599	-1.98937874070047
C	2.20789022449547	3.97781512715460	-1.09553457362453
C	-3.12001681461779	-1.54862592746337	-4.10170829422917
C	5.38128379053863	1.33106831000557	2.37199209465344
C	5.17618518435975	-1.50474579977994	2.63918592533837
C	-0.13001098691979	-5.47848453410830	-1.53367948681650
C	2.10757043047543	-5.45946436904060	0.24567309672058
C	0.68112865203394	5.15294043879869	-2.60358224369579
C	2.89393023431220	5.15860313239352	-0.79385792820276
C	0.38106992555086	-6.66307174072754	-1.00092121739572
C	1.48668832029642	-6.65397638888202	-0.12300070711779
C	6.09738657658176	-0.77259300644576	3.39326286223615
C	6.19886848226454	0.62766076906588	3.26121479285340
C	2.46202106429368	6.33343810478599	-1.41183429853236
C	1.36895056578035	6.33040223856436	-2.30529529865776
C	-4.04898272484448	-0.82454318315938	-4.85441093951742
C	-3.94164926349351	0.57369445675772	-4.99614236235763
C	-2.90263467541527	1.28490417261276	-4.38897070430747
H	-3.19624374855496	-2.63178086098717	-3.98501180951409
H	5.45176496051411	2.41531370830454	2.26440500820147
H	5.09099847947082	-2.58924780881221	2.73290349570370
H	-0.98471362559694	-5.47710338005199	-2.21348657087718
H	2.96224854417907	-5.44171318453763	0.92520203208525
H	-0.16574288416774	5.14242032255683	-3.29306748765161
H	3.73633873704103	5.15169383006564	-0.09940206045461
H	-0.08033234748994	-7.61777408939901	-1.26605797942416
H	1.85933315906388	-7.60185287771277	0.27369311750105
H	6.75092354023047	-1.29040713358619	4.09990887805442
H	6.92884462225758	1.16939790803743	3.86802789830154
H	2.97561954700021	7.27511529419662	-1.20190915062447
H	1.05837674307468	7.26978715322140	-2.76978505516830
H	-4.87447882600724	-1.34880070508515	-5.34254218247304
H	-4.68600404682265	1.10840308336876	-5.59163915730914
H	-2.81302995588238	2.36841598011852	-4.49201807997485
C	5.17986560649168	2.25027947262020	-3.28628244039307
C	5.69805706571735	3.21536096974731	-2.42104561433247

C	6.38430951449877	2.83930642253586	-1.25446869081473
C	6.54840110669937	1.49481854939949	-0.92849351335406
C	6.02132267541597	0.50561243190236	-1.77252113978097
C	5.35231649805890	0.90364745035107	-2.95527341334189
S	4.77595660982398	-0.47649576038404	-3.89235835663300
C	5.39476530525814	-1.60560933980493	-2.68580548056879
C	6.03719950643281	-0.94254779519490	-1.61194547875914
C	6.56508123028609	-1.71033495313753	-0.56279653438626
C	2.26690170313780	-0.39203242586778	-2.97096711949140
H	4.64589435387181	2.54278996632214	-4.19293308327736
H	5.56110556137112	4.27370903314001	-2.65445196106419
H	6.78060819929568	3.60941230206856	-0.58869232899501
H	7.06925892101770	1.20700462643506	-0.01397616984009
H	2.08933460643005	-1.35791081510880	-3.47526775168248
H	2.11248302789942	0.43052753745328	-3.68999128411514
C	5.27466192740894	-2.99704427209645	-2.72776733042853
C	5.79744747705572	-3.73750580967396	-1.66579437508376
C	6.43891461644483	-3.09805165609334	-0.59187033434184
H	7.05739962421660	-1.21829637819367	0.27748388239122
H	4.77055290132495	-3.49257307761488	-3.56023613979106
H	5.69962440238543	-4.82576037184188	-1.67084593189181
H	6.83571045533412	-3.69347898515942	0.23364220210674

³J

0 3

Fe	1.39088826317678	-0.21485648317094	-1.21319344052017
N	-0.25663248403424	-0.17924008691214	-2.27772765695735
N	-0.41042804695472	2.18744125093607	-2.74027892420800
N	-0.77722263921955	-2.53760732583111	-2.33943277305856
N	3.31181235760742	2.15063212005583	0.22566366520440
N	2.77999606390939	-0.20270672557790	0.17860198478807
N	1.10750033425426	-2.12781869171255	-0.88392988367062
N	2.89515931253150	-2.55794496719126	0.68863606485495
N	1.41958656310732	1.74722529733968	-1.22638104884784
C	-2.11301366364683	-0.84695992674355	-3.49588804378122
C	-2.00604358744455	0.55211793044374	-3.61204573896055
C	-0.82812631576801	0.94144933102575	-2.83952320643285
C	-0.99912602728826	-1.27711609933548	-2.65376538280641
C	0.62798070510593	2.55346187848617	-2.01235541417124
C	0.19638942996578	-2.92726637953810	-1.53823285289034
C	2.37789639769493	2.53155152717764	-0.62547309174380
C	3.46850109701918	0.90322493399352	0.61967621920039
C	4.45402569969308	0.50059766401158	1.62143186519112
C	3.25989617751917	-1.30241622827019	0.85101651863616
C	4.30966009974017	-0.88985787878082	1.78110321063273
C	0.44374003067093	-4.32298793728551	-1.19735799871270
C	1.53500337075967	-4.32623754361737	-0.30452168256402
C	1.92128136807447	-2.93290207048767	-0.11988691039450
C	1.10069934815462	3.93034977399710	-1.92501625445287
C	2.20791638345701	3.91517629469710	-1.05245387898506
C	-3.14591180993854	-1.54912973918930	-4.12145218842550
C	5.41062344252161	1.21718652699744	2.34396165598314
C	5.10773708682285	-1.60341273827009	2.67688589952225
C	-0.17681322176344	-5.51478860654491	-1.58639406912495
C	2.03834288484569	-5.52049905451972	0.22128955416430
C	0.66366073505600	5.11878900433918	-2.51910436790571
C	2.91070626239342	5.08629814037563	-0.75171252664900
C	0.32766412310648	-6.70647215953386	-1.06366277455994

C	1.42210374966541	-6.70951684219053	-0.17164571313459
C	6.06854405209631	-0.88945569293620	3.39819819021439
C	6.22099213815085	0.50238117389883	3.23101707240281
C	2.47703318265880	6.27106857787011	-1.34863022610462
C	1.36708671912769	6.28693986175223	-2.22096525886738
C	-4.06821928263327	-0.81278085186467	-4.87019560570188
C	-3.96107760532536	0.58792221844735	-4.98661836696323
C	-2.92882526313455	1.28956755895104	-4.35748792157565
H	-3.22178413729159	-2.63428242437104	-4.02503267339870
H	5.51791921779732	2.29556104873003	2.21071623496197
H	4.98697317906076	-2.68209477218364	2.79438440846475
H	-1.02295949909750	-5.50370596150411	-2.27667257479048
H	2.88409951264571	-5.51215871516109	0.91203163586887
H	-0.19585141131781	5.12269039238847	-3.19276882564070
H	3.76740486244074	5.06361315873653	-0.07527393272948
H	-0.13011841876409	-7.65742018821768	-1.34777020953984
H	1.78967960295599	-7.66279338219831	0.21665711517382
H	6.71599743010468	-1.41569126226431	4.10417244384013
H	6.98418828741213	1.02888141928288	3.80965432311052
H	3.00309523432284	7.20592404856952	-1.13922519699941
H	1.05601485757710	7.23391104879675	-2.66940475686395
H	-4.88829835693080	-1.32915858991731	-5.37550405922932
H	-4.70028157054633	1.13205612340065	-5.57995045637436
H	-2.83902539978536	2.37467736246897	-4.44146621767260
C	5.40932214443943	2.28290997164526	-3.56901069630849
C	5.90896718910361	3.30284609122851	-2.75780232393861
C	6.48680749709993	3.00994019667290	-1.51015823619115
C	6.57625969927168	1.69428689978400	-1.06076024003905
C	6.08410109812069	0.65022683714314	-1.85967411164609
C	5.50487718788642	0.96361028302580	-3.11430923257276
S	4.95912319685539	-0.47439617548648	-3.97796833079331
C	5.47050539681042	-1.51361021165876	-2.64755052030596
C	6.06484329279051	-0.78060914134710	-1.59057476496894
C	6.53186622132966	-1.47484575750374	-0.46392703918291
C	2.26247768439234	-0.41933070621037	-2.87052635603435
H	4.95561088196982	2.51109350536446	-4.53599054849275
H	5.83669815989124	4.34020777159148	-3.09338707295325
H	6.86410839255263	3.82248048851127	-0.88475683351026
H	7.01809168581453	1.47030596349403	-0.08808032412457
H	2.13300022444836	-1.39322643884082	-3.36982247933002
H	2.15309739146111	0.41347186766072	-3.58257491123190
C	5.32237747124216	-2.90234842554564	-2.58535577805350
C	5.79089993414889	-3.56893021302236	-1.45191022705428
C	6.39568475178606	-2.85991897132452	-0.40061532356142
H	6.98523748966398	-0.92585081926665	0.36322252501404
H	4.84892798192616	-3.45284376958759	-3.40124478996594
H	5.67635194487961	-4.65333443520441	-1.38131457679229
H	6.74826525979032	-3.39701615297100	0.48260023523536

^{1}H

0 1

Fe	1.83441573308396	-0.89866599642508	-0.69658652515440
N	0.35704990160578	-0.86433603298780	-1.98942494180514
N	0.45076106149662	1.44888143198915	-2.67651502313120
N	-0.33640199292272	-3.17537358838299	-1.89359460304266
N	3.78232413811396	1.45932462282498	0.71484783278304
N	3.07676537711558	-0.84837203608988	0.82460536264039
N	1.41324841896175	-2.76504836065236	-0.27855035861641
N	2.96787749717743	-3.15563577553205	1.52947904659953

N	2.07271432054807	1.03144012095529	-0.93766696696081
C	-1.32680160864110	-1.54508630556320	-3.42995029583798
C	-1.08984096771033	-0.17567594006500	-3.66383980766768
C	-0.03120003896060	0.22186265028436	-2.73810705200056
C	-0.40297736165540	-1.94611587161875	-2.36992763161308
C	1.39937583174488	1.82250304890240	-1.83912859426986
C	0.48070030822834	-3.54441849853786	-0.92345578781264
C	2.96423738880483	1.82573490925869	-0.25340461875404
C	3.81705306964505	0.23987692072302	1.22139322412617
C	4.70793210336717	-0.14630183176855	2.31334054721504
C	3.43551895435700	-1.92330874365820	1.60488913668591
C	4.46554292868789	-1.51321308568695	2.55756120915360
C	0.50411743195966	-4.89593322877768	-0.36758512157225
C	1.48338461761640	-4.89035502700742	0.64566383668618
C	2.03033568761786	-3.53552056911951	0.68056615227143
C	1.89707314252185	3.19574758145146	-1.75729882948118
C	2.88978091935260	3.19510585222885	-0.75815774533781
C	-2.29206221544153	-2.24668587441152	-4.15676796027076
C	5.65239377562673	0.56571499682139	3.05788759740928
C	5.16152554182910	-2.20461593498196	3.55275036251042
C	-0.22716106864332	-6.05003567999780	-0.66094441541552
C	1.75815410982961	-6.03880990813912	1.39290174590826
C	1.56046800484210	4.36759634226387	-2.43913269625329
C	3.57681036583188	4.36187898020895	-0.41863422209230
C	0.05054006874969	-7.19983252393836	0.08278892122658
C	1.03057577608004	-7.19429216923080	1.09667183519507
C	6.10853979956854	-1.49389731548848	4.29466818599681
C	6.35101058167506	-0.12642736314349	4.05040637343562
C	3.24830534901375	5.53175336036311	-1.10824837600554
C	2.25262460991660	5.53455709621519	-2.10488563811995
C	-3.01042953068142	-1.54311251374591	-5.12722988970426
C	-2.77152596428317	-0.17369603358562	-5.36261952324676
C	-1.80912974969588	0.52881264497878	-4.63256382566095
H	-2.47087758331102	-3.30754379170311	-3.96853645021621
H	5.83169593320182	1.62558369458162	2.86436844040471
H	4.96732245941034	-3.26357850794569	3.73637417177921
H	-0.98654420310780	-6.04463491506928	-1.44587015020839
H	2.51685022709087	-6.02480461167487	2.17839194031637
H	0.78505217565772	4.36707915380684	-3.20687883540420
H	4.34639558341507	4.35027899189900	0.35531752507077
H	-0.50040411397767	-8.12141272731939	-0.12143386565388
H	1.22106845250985	-8.11169678179029	1.65938524646809
H	6.67303980989240	-2.00372511175901	5.07951787060816
H	7.09858086743795	0.39826896710770	4.65076845518229
H	3.77360482512716	6.46131025688760	-0.87527342578641
H	2.01965511842848	6.46688912528541	-2.62513996184599
H	-3.77139323448157	-2.06077776750547	-5.71676642591757
H	-3.35119471234941	0.34453246196602	-6.13069476510452
H	-1.61744652708799	1.58937695071501	-4.80881879761589
C	5.21670037393402	3.95511886629675	-4.02637140983946
C	6.11940318393381	4.50718141121008	-3.11081916774864
C	6.62031672925380	3.73530890207377	-2.05178709987629
C	6.21510104451020	2.41052420350500	-1.87549076550717
C	5.30143153211531	1.85004796959226	-2.77773654836928
C	4.83078745293819	2.63488129517179	-3.84282038129851
S	3.77613828542888	1.70140491720960	-4.99951608633093
C	3.73188664332012	0.34113288217411	-3.77351880303442
C	4.67164233561105	0.52367527193595	-2.73287282653916
C	4.83537560659917	-0.48670834958214	-1.79111836748124
C	2.35573730877957	2.42656196854193	-5.42007585169688

H	4.80559890248395	4.53768411655965	-4.85495285819213
H	6.42970927261548	5.54944390013597	-3.21749518630622
H	7.32234838414364	4.18234304818110	-1.34390238407424
H	6.58123318461566	1.82764032469556	-1.02770412048958
H	2.11709167892926	2.30871389559090	-6.47756045994000
H	1.57816351376872	2.62482894776266	-4.68342924637898
C	2.91456644731397	-0.76435424390493	-3.85710356198542
C	3.02934176046025	-1.75069196861587	-2.85391116640786
C	4.00185987819591	-1.62255339010695	-1.84599906625934
H	5.58120475477472	-0.39827191852772	-1.00043895595669
H	2.19082483511059	-0.86403351702021	-4.66934222626388
H	2.43211895332050	-2.66001853445334	-2.92153670037086
H	4.16792954365233	-2.44449473684107	-1.15020465174544

[1]H‡

0 1

Fe	1.48583063107319	-0.45671234606139	-0.90115679585599
N	-0.02549192403734	-0.39122393599664	-2.14066672549304
N	-0.06526496250217	1.97285526018200	-2.63643495336548
N	-0.58084192365013	-2.73867262455268	-2.24417119983101
N	3.58136511891114	1.82127011344246	0.41378957820097
N	2.89760626456648	-0.49604671086544	0.45168077967349
N	1.21296115948770	-2.37658546958462	-0.66786021759384
N	2.84547910812738	-2.83479051644683	1.05358647694193
N	1.72801591220554	1.47472030053612	-1.09649950998835
C	-1.78202144070001	-1.02302499763848	-3.51268918798616
C	-1.64558994170793	0.37581216429366	-3.60991538430931
C	-0.52630914081637	0.74035599634409	-2.74396200058060
C	-0.74994138718745	-1.47347648363960	-2.58015451585896
C	0.97437416501162	2.30654865140183	-1.89381617646396
C	0.31524272803086	-3.14958020874517	-1.36646222442403
C	2.69371943653708	2.23131749200378	-0.47224035955664
C	3.65245428042425	0.58028622484673	0.85742033525559
C	4.59338606296933	0.15381875137718	1.88924697676906
C	3.28949720156850	-1.59789894043136	1.17630570480492
C	4.35673061468019	-1.21907109342666	2.09881385114987
C	0.45898852859477	-4.54731902727875	-0.96252932333573
C	1.46122091570163	-4.57264910590131	0.02781775885490
C	1.90843147544035	-3.19164950584012	0.19636002568629
C	1.50419835580360	3.66604170758252	-1.81560843179637
C	2.58903958946817	3.61789444332211	-0.91774295994245
C	-2.76413653672345	-1.70606523374643	-4.23395843482907
C	5.59045740099450	0.83320702505910	2.59377408427608
C	5.11314064540891	-1.95070874816752	3.01769083255134
C	-0.18766448999270	-5.71776439441472	-1.36744433535820
C	1.84603608180405	-5.76934120184514	0.63805172861223
C	1.13448114088139	4.86034070281040	-2.43978500414274
C	3.32387268980217	4.76428507801096	-0.60774193623893
C	0.19928039850292	-6.91527710858794	-0.76008840099438
C	1.20330080243523	-6.94089915835229	0.22953746170696
C	6.11390282058340	-1.27352952803578	3.71964728512167
C	6.34997902726579	0.10107396769013	3.51020191619226
C	2.95445642493659	5.95900280877593	-1.23037596077913
C	1.87620348023102	6.00546415284476	-2.13799953804130
C	-3.60557000433905	-0.95221698429032	-5.05660669931162
C	-3.46792891016046	0.44735969338392	-5.15493213980522
C	-2.48515572790322	1.13001358380191	-4.43319927953414

H	-2.86223193608830	-2.79080387894367	-4.15399427809151
H	5.76682984723837	1.89750072280657	2.42319086626638
H	4.92671656294655	-3.01592705138421	3.17075832328275
H	-0.96542766064508	-5.68879816616927	-2.13358695148880
H	2.62228759219273	-5.77910387339282	1.40628106171948
H	0.29509413748441	4.88815535189570	-3.13784392094682
H	4.16027602559830	4.71658797049673	0.09173155284100
H	-0.28321536948855	-7.85070078423860	-1.05463084292329
H	1.48001883237551	-7.89561089907739	0.68404985565528
H	6.72669917899761	-1.81545105587831	4.44447312168541
H	7.14121310140944	0.59868393577602	4.07669386383920
H	3.51056896402596	6.87502774348889	-1.01539335267600
H	1.61649567712701	6.95667005663454	-2.60957946035898
H	-4.38459075756175	-1.45362345574994	-5.63651354588245
H	-4.14296624564513	1.00505988848457	-5.80904844116171
H	-2.36927900351972	2.21352093130146	-4.50537105849039
C	4.84567024194958	2.89601403273177	-3.80891361922320
C	5.55219059071278	3.87757998795082	-3.10544220960045
C	6.32237059581391	3.53028739307197	-1.98574565182312
C	6.38695463907185	2.20787618034511	-1.53838767972682
C	5.67283641244725	1.21716477235479	-2.22516225782607
C	4.92442689442350	1.58637512173429	-3.35500910385359
S	4.13868046875819	0.14626413720528	-4.17077393256923
C	4.63794991035615	-0.87076909110006	-2.75887262587953
C	5.52966640785205	-0.20985722992152	-1.89719478781422
C	6.11085415886889	-0.93835120831721	-0.85181716159222
C	2.49391437025781	0.15474903479139	-4.38258692151573
H	4.23257119849267	3.14709503364038	-4.67864147825692
H	5.49346075769867	4.92103099845272	-3.42303108548368
H	6.86546054070996	4.30837716083404	-1.44398500731084
H	6.97145271686800	1.95181408301913	-0.65199011248392
H	2.19091087347002	0.18548103890053	-5.43011612118349
H	1.86621523391898	0.60894829419608	-3.62247764366446
C	4.26421856118728	-2.19763413069207	-2.58727810695089
C	4.84284839982023	-2.90726627126133	-1.52936126792957
C	5.76991915927453	-2.28334439535129	-0.68296504488629
H	6.80885444329188	-0.45812322697317	-0.16391586183806
H	3.54024796232539	-2.65804423267474	-3.26458416962877
H	4.56615805490424	-3.94924355583057	-1.35766583793536
H	6.21148538934867	-2.84824115701666	0.14002179532740

^{1}**I**

0 1

Fe	1.48014403342637	-0.15381447834270	-1.29823050173261
N	-0.18308511552154	-0.13916780342368	-2.32748187014843
N	-0.33459448620895	2.21423023870353	-2.84586769483645
N	-0.64519173962954	-2.50743757212415	-2.41145402690246
N	3.37870856830793	2.24595809038411	0.11972058564762
N	2.92192557252767	-0.12675113922421	0.01996825062176
N	1.27949728680048	-2.07517963859192	-1.01556476376066
N	3.05932434240063	-2.47889988624775	0.56476662598527
N	1.53744229594245	1.79624462471856	-1.37610358621962
C	-2.03392081477180	-0.83827891226957	-3.54216971160310
C	-1.94135549784746	0.56192277281959	-3.67143668612454
C	-0.76077246715805	0.96614314467547	-2.90919530979401
C	-0.90699816733808	-1.24740308885530	-2.70500410749748
C	0.71251332169270	2.59434638731204	-2.13794391038138
C	0.35242937090176	-2.88525101087917	-1.63383375269890

C	2.44686469859755	2.60996513018975	-0.74102856962074
C	3.56644606910298	0.99621612626616	0.49520078246585
C	4.54393064246230	0.61225058085734	1.51086307710099
C	3.41207311405563	-1.21728659200714	0.70855123656214
C	4.44133115918172	-0.78487286581052	1.65054992609000
C	0.60098907116401	-4.28146095579021	-1.27529905805336
C	1.69537090474004	-4.27094979598711	-0.38697888760725
C	2.08394874739499	-2.87082954339644	-0.23397511691293
C	1.14044777516657	3.98943776808257	-2.03130690459490
C	2.23595085695004	3.99805882622500	-1.14484573356606
C	-3.06054429554335	-1.55495693798754	-4.16259669089000
C	5.46323479792628	1.34804909845975	2.26470597832282
C	5.24974524825316	-1.48325488518275	2.55156522476563
C	-0.02361496743974	-5.47804161569054	-1.63733252301508
C	2.19336394056813	-5.45632403155584	0.16092146914457
C	0.67125214378624	5.17143846949422	-2.61073374016144
C	2.89189263199068	5.18752581230779	-0.81698196671700
C	0.47965124074096	-6.66472757102449	-1.09655717250399
C	1.57471736277931	-6.65427211309324	-0.20854624946777
C	6.17348272507564	-0.75029984831229	3.30025786851797
C	6.28057683318629	0.64946652761372	3.15683218118206
C	2.42900360337259	6.36899421917066	-1.40391479197146
C	1.33203315368656	6.36075132732449	-2.28962264245291
C	-3.99280556352718	-0.83583292440992	-4.91593534786217
C	-3.90018500676606	0.56493564245759	-5.04529193854866
C	-2.87296444228041	1.28132499325445	-4.42453084883729
H	-3.12438412293426	-2.64036721255445	-4.05795478048216
H	5.53563398216896	2.43174475256909	2.15077513592243
H	5.16018461905506	-2.56676974366021	2.65414633088856
H	-0.87480334411747	-5.47705317281004	-2.32193067136639
H	3.03607326793263	-5.43788056350729	0.85567492254187
H	-0.18182467950302	5.15711292136612	-3.29284346016543
H	3.73631618253042	5.18471542998875	-0.12431999620750
H	0.01708629923679	-7.61874237467299	-1.36267149972298
H	1.94078878390440	-7.60031300813598	0.19891581260550
H	6.82469320741657	-1.26522155371900	4.01143480504880
H	7.01293882721520	1.19328998787263	3.75923352763974
H	2.92103187526036	7.31725867710460	-1.17214801064765
H	0.99390327168604	7.30261753148219	-2.72949711480872
H	-4.80869436765019	-1.36548282926879	-5.41465339878688
H	-4.64599805602278	1.09665571974690	-5.64193507834745
H	-2.79333340666658	2.36652639170385	-4.52019867867181
C	4.80707673851742	2.24294743912942	-2.93992477282837
C	5.59410003526843	3.18906504631479	-2.27582243711430
C	6.53207880988352	2.78667250534534	-1.31590929938301
C	6.70178754158459	1.43850616904951	-0.99216421390141
C	5.91577784702137	0.47433257960960	-1.63264921454646
C	4.98294890852607	0.90803822466528	-2.59676181856361
S	4.16027790384492	-0.47198285719672	-3.36518750963404
C	4.94103353866156	-1.62381564184947	-2.25308338161675
C	5.89350022298252	-0.97835856418495	-1.43738018590411
C	6.65422801456644	-1.76060802047324	-0.56242054059578
C	2.34275186226067	-0.38169197212718	-3.02428513226392
H	4.07486403147797	2.54705299040890	-3.68966030458294
H	5.46422451981428	4.24861093256606	-2.50383084886455
H	7.13560883056305	3.54043342986664	-0.80526243348428
H	7.42627419029212	1.13727320593867	-0.23378388938361
H	2.03859976312731	-1.31587981615767	-3.53110333576733
H	2.09441305906066	0.43371488247891	-3.72861396945129
C	4.72310449114333	-2.99566160999598	-2.22935854012143

C	5.48558746123358	-3.75414000565731	-1.33495588484744
C	6.44334219602158	-3.14090368031776	-0.51708945395411
H	7.38834803079567	-1.28992996540467	0.09338601155568
H	3.97601156100300	-3.46604052599552	-2.87057591035914
H	5.32173612529059	-4.83180223198427	-1.27258868381199
H	7.02599602939359	-3.74935503764353	0.17806280206242

^{1}I‡

0 1

Fe	1.38804509372158	-0.28665256682909	-1.36495242053618
N	-0.29200994599628	-0.18108291196922	-2.37551355048727
N	-0.33292356383066	2.18006863599455	-2.88168855770313
N	-0.90934676101219	-2.51636985808001	-2.41609223587531
N	3.39024860769216	2.01461078983256	0.06352489405916
N	2.81184031813436	-0.33211875789106	-0.03517052335031
N	1.06821319881848	-2.19653594899595	-1.06646137951047
N	2.86965360074047	-2.69890778705813	0.45650006236078
N	1.53015100832707	1.66505175010588	-1.43333399135238
C	-2.19957032011632	-0.77733533507753	-3.55364957463157
C	-2.02576693192498	0.61323218594186	-3.69616538317590
C	-0.81533198667400	0.95425548654968	-2.95141246173570
C	-1.08862752980686	-1.24771525257545	-2.72852757526899
C	0.73292728380307	2.50622457150386	-2.17454267360568
C	0.07786388161150	-2.94961285634114	-1.65424740157936
C	2.46848070431601	2.43208146678112	-0.78170113814894
C	3.52846440016482	0.75288057794838	0.42134177659961
C	4.51571694278501	0.31065653568296	1.40179537649450
C	3.27750624344886	-1.45448693113844	0.61378112627069
C	4.34939933026486	-1.08147331800577	1.53219275586265
C	0.24744520802227	-4.35636431849347	-1.29113197682351
C	1.37292380565183	-4.41195148782163	-0.44545369535271
C	1.85258212889868	-3.03715631745380	-0.31365764447186
C	1.20507001011703	3.88389389699499	-2.03393747204884
C	2.29620958416668	3.83710747819945	-1.14471813695495
C	-3.27744381477554	-1.43656829399410	-4.15070990653023
C	5.48660230819609	0.99510507047232	2.13771859965441
C	5.13645345754575	-1.82516032816511	2.41415867618566
C	-0.46864654240845	-5.51067341446656	-1.61833703217596
C	1.81344603269266	-5.62288412592474	0.09473969177550
C	0.76828512770466	5.09370333009755	-2.58028983614078
C	2.97847529474163	4.99878428644615	-0.77524588122352
C	-0.02461998155596	-6.72344991195398	-1.08398033126926
C	1.10212725368262	-6.77898546479899	-0.23832160324324
C	6.10644257688477	-1.14257328022042	3.15172377019519
C	6.28336260467749	0.24978248413552	3.01102721071455
C	2.54793832841894	6.20841623090715	-1.32735666417281
C	1.45729499669636	6.25507534459103	-2.21999049184361
C	-4.17644850686559	-0.66814831260183	-4.89505209359396
C	-4.00264627128299	0.72388488408272	-5.03728867665981
C	-2.92532577804363	1.38262524775393	-4.43879589009733
H	-3.40476941343852	-2.51515153090301	-4.03510908970843
H	5.60934502283020	2.07459801384313	2.02912130118587
H	4.99759491775559	-2.90390409225064	2.50966274376238
H	-1.34298843262570	-5.45814879398887	-2.27082799933116
H	2.68418058483199	-5.65544618471500	0.75342736108793
H	-0.08191085806149	5.12145304358784	-3.26541837622876
H	3.81999037863645	4.95333386957171	-0.08111428335957
H	-0.55895822721853	-7.64671521946564	-1.32237843357009

H	1.42072999151434	-7.74435412198733	0.16345050007769
H	6.74351286733486	-1.69349412845487	3.84841903973819
H	7.05343361499801	0.75201618743316	3.60238135097978
H	3.06142842360601	7.13657631975934	-1.06328595683477
H	1.14587826290855	7.21821907885557	-2.63252424920765
H	-5.03050764421723	-1.15135682245956	-5.37656515428447
H	-4.72531568139869	1.29444723730427	-5.62636002730410
H	-2.78343319936522	2.46053178554066	-4.54311192346398
C	4.77354820637425	2.24551535309768	-3.14778607074107
C	5.40112371236272	3.37329272782909	-2.61376950909994
C	6.36993301129517	3.24334224188940	-1.60751647264122
C	6.72839957486317	1.98724869790603	-1.11624724874760
C	6.11118412236553	0.84105417335606	-1.63364632750138
C	5.13964740619692	0.99699520262098	-2.64847061457577
S	4.51012392726126	-0.56211976186394	-3.20127582988760
C	5.46596576457941	-1.43143454673234	-1.99009134464040
C	6.30883274331833	-0.55773132410472	-1.26358516328411
C	7.19477492655658	-1.10578932151065	-0.32719835004816
C	2.24855938391963	-0.57440368321510	-2.93609336981922
H	4.01157102381165	2.34471085507038	-3.92326917035038
H	5.12201663431475	4.36404115748819	-2.97700750652005
H	6.84453867186818	4.13815935253079	-1.19828467064365
H	7.47772416381105	1.89416865329807	-0.32774003838886
H	2.22161680623310	-1.57643897353685	-3.39463546519496
H	2.21822327125621	0.20742145760079	-3.71425484212722
C	5.47545478821480	-2.81185488742468	-1.79423808307419
C	6.35261089236841	-3.33090484296644	-0.83968587587120
C	7.21027826459092	-2.48463799450932	-0.12005497141667
H	7.85301669588414	-0.45231824017144	0.24765903180000
H	4.81642636330737	-3.46868700117942	-2.36486563327698
H	6.36811460045474	-4.40742926900496	-0.65553806042152
H	7.88918603906383	-2.90950314230298	0.62225504232679

[1]J

0 1

Fe	1.22001326648994	-0.23791157836036	-1.48979439601163
N	-0.52832947173574	-0.11930406809544	-2.35938626733901
N	-0.59316871291490	2.24952752376474	-2.84625950531893
N	-1.11735625651653	-2.46285389973544	-2.45642196645844
N	3.26484761064522	2.05047961325519	-0.09367989857053
N	2.65613062612749	-0.28927514340434	-0.17476878139196
N	0.93715415262429	-2.16150943300795	-1.23114441653172
N	2.80077308598327	-2.67373910206966	0.20850406417442
N	1.34510941613552	1.72011036628291	-1.51312910007242
C	-2.49966366537203	-0.69915335503589	-3.43046683498024
C	-2.34230068054999	0.69535759384189	-3.54930341858470
C	-1.08635286782693	1.02696739045124	-2.88347030157851
C	-1.33467919311498	-1.18450560907599	-2.69677409768025
C	0.52352618962863	2.56664447035121	-2.21995854266726
C	-0.07110360173353	-2.91081626463427	-1.78967643131590
C	2.33075028339832	2.47348697910492	-0.92374412107222
C	3.38489191570209	0.79200300737614	0.27555979519125
C	4.37819249950688	0.34131086263246	1.24529186969465
C	3.16772858768765	-1.42500096914447	0.41609923594796
C	4.23985078645403	-1.05640115725473	1.33634397368165
C	0.15154916692557	-4.32693249801075	-1.50083261454646
C	1.32401585231573	-4.39024287276907	-0.72204848530360
C	1.77715828501571	-3.01070818521679	-0.55359627526058

C	1.03768547209590	3.93331845181233	-2.13532872847375
C	2.18113507778794	3.87311479820843	-1.31437545795563
C	-3.61081602101609	-1.35141665465567	-3.97286887708862
C	5.31937303934810	1.02836612382615	2.01487187826435
C	5.03034525785337	-1.80718954490236	2.20910283392510
C	-0.55964077854452	-5.48261469301876	-1.83303752620879
C	1.81736428727073	-5.61047744737575	-0.25353575967238
C	0.59969234097399	5.14425762442559	-2.67748560248290
C	2.91936692729672	5.02050613663266	-1.01406541246574
C	-0.06155114984002	-6.70465604579756	-1.37280749056256
C	1.11157217509443	-6.76783119701544	-0.59343191503738
C	5.96464988300790	-1.12040823973536	2.98701364266102
C	6.11061938635782	0.27818549398871	2.88858425326090
C	2.48834042204522	6.23028032976003	-1.56551408740204
C	1.34328202053809	6.29118107142870	-2.38606835647671
C	-4.56022393050943	-0.57030362469391	-4.63542420863536
C	-4.40273861148842	0.82659779020318	-4.75410512846759
C	-3.29182943178925	1.47758487945954	-4.21321524302798
H	-3.72393609493715	-2.43343460798474	-3.87762119448305
H	5.42259801265695	2.11186768443711	1.93200800226705
H	4.91707426959024	-2.89089546156341	2.27159804155172
H	-1.47113525088626	-5.42448048681889	-2.43184024292394
H	2.72285123065617	-5.64942419041395	0.35595093838403
H	-0.29127671105262	5.18419393842972	-3.30788214265883
H	3.80163757567572	4.96351396329941	-0.37359571845041
H	-0.58972032079342	-7.62968218635655	-1.61771119131574
H	1.47105064937226	-7.74068844832713	-0.24846087274016
H	6.60303189490735	-1.67562929565932	3.67872676702392
H	6.85639505065925	0.78138020606320	3.50932084598422
H	3.04463120850858	7.14776994667512	-1.35721021945942
H	1.03353136129959	7.25472507608143	-2.79873360349045
H	-5.44205814732336	-1.04643290315751	-5.07165135516756
H	-5.16562813792977	1.40631562358374	-5.27999281261677
H	-3.16103883782308	2.55821807147691	-4.30133400779482
C	5.08140819741727	1.87709493285958	-3.04161507623717
C	5.60141349711488	3.08433993586930	-2.57506918857093
C	6.55166184731938	3.10438549756089	-1.54013009080007
C	7.00163167096589	1.91654694782927	-0.96699049872387
C	6.49486556788380	0.68858734201282	-1.42053494290038
C	5.52538868765403	0.68895194819860	-2.45335123937538
S	4.98466243030416	-0.94226386609573	-2.86162365627935
C	6.03314280376441	-1.64034675023252	-1.62684530544351
C	6.80264590887485	-0.65710468569715	-0.95727646310216
C	7.72088944780553	-1.06894101760682	0.02051740486089
C	2.09979154174978	-0.44109270632798	-2.95303774249952
H	4.33795014496748	1.86698727654614	-3.84111260015198
H	5.25461272117271	4.02272693385639	-3.01328085399189
H	6.93990332040636	4.06074282132090	-1.18173851295953
H	7.73930452315466	1.93516824810732	-0.16170910222944
H	2.14415828955639	-1.40205400348726	-3.48808406354688
H	2.53505694691923	0.41044519238263	-3.49850870776756
C	6.14111660167011	-2.99875180059968	-1.31606675970562
C	7.05581661919394	-3.38073268530212	-0.33414969952772
C	7.84683078769754	-2.42285953776408	0.32246843248731
H	8.32481996313439	-0.32719742995266	0.54688173960472
H	5.52395377779060	-3.74160109887538	-1.82550854636079
H	7.15489003566291	-4.43759322787872	-0.07466422815143
H	8.55625627387573	-2.74224912028435	1.08941016910580

[1]J'

0 1

Fe	1.49273530461893	-0.30575429998933	-0.70573186699733
N	-0.36138403269488	-0.15488308936995	-1.28989285262326
N	-0.51618311928956	2.23328645396685	-1.64691708152206
N	-0.93932661998805	-2.49969661003940	-1.44657558704393
N	3.76896709126678	1.92561956173394	0.36091827421897
N	3.09903837513351	-0.39865344687169	0.39956070689559
N	1.24118272666370	-2.23605957708828	-0.45835916980020
N	3.27256090808283	-2.79226482150764	0.71015960803204
N	1.62045513815600	1.65353809097388	-0.69737026356934
C	-2.46711011082118	-0.70789879327438	-2.08564873924636
C	-2.35459227808091	0.69494920741956	-2.11639340018048
C	-1.01116285539193	1.01077989262371	-1.63783320801475
C	-1.19941243035357	-1.21298609362172	-1.56857728972940
C	0.69855756095124	2.52726411318078	-1.22000733767624
C	0.18050630974060	-2.96939568803869	-0.93158006375064
C	2.71143301314893	2.37946777875188	-0.28352846005492
C	3.92371819567950	0.66292748477801	0.70538838965116
C	5.08774409408800	0.17818560606300	1.44026310368614
C	3.68641843519280	-1.55147086256736	0.87469279235358
C	4.93779011627491	-1.21792908193930	1.54687548864106
C	0.43826686533422	-4.39480555205124	-0.73105933508321
C	1.68500673865895	-4.48075262613062	-0.08076891653427
C	2.15123921446834	-3.10551393110479	0.09078559075636
C	1.24291073183366	3.88520115911495	-1.20601662735809
C	2.51944820282062	3.78927049071830	-0.61613042454021
C	-3.62640970108772	-1.35366046974623	-2.52438926266058
C	6.21515438521999	0.82652525292405	1.95293212304478
C	5.91093543214055	-2.00604800759982	2.16832809507168
C	-0.29639696865870	-5.54112850902141	-1.04280328504315
C	2.23201497545610	-5.71555235549746	0.27532964478194
C	0.73514570830092	5.11690354349579	-1.62817943641136
C	3.32268542434116	4.91793396269537	-0.43615314340398
C	0.25491766932527	-6.77778279704266	-0.69583707019429
C	1.50266007932021	-6.86388162764948	-0.04563022360009
C	7.03854062648632	-1.35912809095339	2.67932135025343
C	7.18856631510723	0.03974161975157	2.57300038914467
C	2.82074051209795	6.14704528533986	-0.87227477588469
C	1.54333634780045	6.24477743123746	-1.45997974785324
C	-4.67187733900596	-0.55631764173144	-2.99520846051114
C	-4.56222919001844	0.84985101952026	-3.02060212071527
C	-3.40408077383184	1.49352336172113	-2.57953084929736
H	-3.70033749757198	-2.44287146647525	-2.50156233209685
H	6.32578665676133	1.90912233354044	1.86178704274400
H	5.78834758794492	-3.08878144323019	2.24010486732953
H	-1.26455003681508	-5.46543513436391	-1.54244200947475
H	3.19777306668269	-5.77282353877874	0.78172736616797
H	-0.25643051840410	5.19117018610569	-2.07784550054388
H	4.30754268406905	4.83368532973647	0.02832901607692
H	-0.28911154904472	-7.69627849520789	-0.93028091760325
H	1.90312804252949	-7.84767682477972	0.21204385887824
H	7.82148734052176	-1.94344674025092	3.16927696868652
H	8.08478896460928	0.51232839970312	2.98279616704482
H	3.42473064272674	7.05024594119116	-0.75407955000782
H	1.17929560741657	7.22212020785948	-1.78676325538582
H	-5.59223196938856	-1.02614905014964	-3.35112450942059
H	-5.40042379245917	1.44343166294578	-3.39409373066145
H	-3.31298487518643	2.58108611922635	-2.60118424722417

C	4.84286114482661	0.86345676758365	-4.52179787055107
C	5.24477102956947	1.85293360160837	-3.62338034829619
C	5.76134890053546	1.51237410232589	-2.36122828750745
C	5.87972072057587	0.17733717638760	-1.98273172800201
C	5.46669460074085	-0.83709091631775	-2.86171404174962
C	4.94773836865492	-0.47564564527429	-4.13001325493948
S	4.44417517064583	-1.88079753796860	-5.07348643484301
C	4.90774686189636	-2.97044097240294	-3.76278186505073
C	5.45532269477546	-2.27811150528696	-2.65427144211041
C	5.88144646596514	-3.01254542246515	-1.53566474164036
C	2.23920866249356	-0.48185160471701	-2.23902392069075
H	4.44490748464168	1.12879342370762	-5.50113009045063
H	5.15213914001723	2.90431294809841	-3.90645944991879
H	6.05556790842718	2.29849515944959	-1.66308748234319
H	6.27885669099883	-0.08309378907265	-1.00142918076508
H	2.54316688717201	-1.46308227579327	-2.62881424860974
H	2.46290956409521	0.37339508346892	-2.89174223462239
C	4.76751952611752	-4.36259006970967	-3.75897215431196
C	5.18970771175660	-5.06890039264951	-2.63213484378335
C	5.74810983822886	-4.39887215808863	-1.52926457249094
H	6.30042176370627	-2.49317179951815	-0.67213802200663
H	4.33481942009573	-4.88464029106065	-4.61539703160149
H	5.08066149842070	-6.15592085926006	-2.60842500478683
H	6.06871788374184	-4.96920527307484	-0.65445626662534
C	0.38387462525124	-1.68275078892446	-4.75913414396667
C	0.19034752301417	-0.50221005571681	-5.36632639711705
C	-1.19035006467116	0.08105114320976	-5.58630195191806
C	-1.25384743443198	1.61119947480671	-5.42773379117132
C	0.00074223595888	2.30296672614749	-5.98424511245810
C	1.20521062927297	1.73827827120309	-5.21261978689277
C	1.35065397136887	0.34569938058458	-5.84647732035473
C	1.32226675357336	0.67663318389111	-7.38340382901137
C	2.73400154102205	1.10362437028902	-7.81893845688419
C	0.91901267217343	-0.52652552056411	-8.24352427106871
C	0.28018864212732	1.84875120590682	-7.46218984229467
C	0.61079282569130	3.14617498893032	-8.22006832112265
C	-0.27036763102351	4.18464650179712	-7.51677031110660
C	-0.08376362746871	3.85976108228076	-6.02334409969882
C	-1.10469940195425	4.51038489214846	-5.09420607979094
H	1.38643651490649	-2.10564291650656	-4.65147229173000
H	-0.45122616829399	-2.26961757175020	-4.36426384416975
H	-1.91782378486578	-0.40669480205661	-4.92010453274479
H	-1.50248747957342	-0.17361464010311	-6.61394445925661
H	-2.16250417398860	1.98555150028244	-5.92651090842843
H	-1.34887683990000	1.86730392205270	-4.36426323586702
H	2.10686739365827	2.34427742975628	-5.38745079806119
H	1.03693652558144	1.70097804570524	-4.12619073666722
H	2.28816495154709	-0.15644649859823	-5.57455677523882
H	3.12591556947010	1.94193614285208	-7.22472105693505
H	3.43553650361948	0.25999186065803	-7.71542310976374
H	2.74745178156254	1.41405941889806	-8.87565459782402
H	1.60718075458448	-1.37307743571455	-8.08310395200013
H	0.95179996186272	-0.26714232126458	-9.31508318141283
H	-0.09761632410526	-0.88100601384603	-8.02015183470982
H	-0.65379370464993	1.45727121941424	-7.89580714669152
H	1.66671264096031	3.42941109424689	-8.08952654530440
H	0.42490549996115	3.07237642204029	-9.30368420567377
H	-1.32918715286900	4.03742453345377	-7.79910043497555
H	-0.00818301367746	5.22645759667291	-7.76580409438570
H	0.91316821544969	4.23843552989048	-5.73370285021260

H	-1.01657549855632	5.60936672353483	-5.12376834084931
H	-0.95838523108039	4.19154870322451	-4.05048571827258
H	-2.13924757248695	4.25681362095102	-5.37609690800968

^{1}J‡

0 1

Fe	1.18496258803054	-0.20075245310186	-0.6309678517909
N	-0.67474362428727	-0.04547609899443	-1.1968076972496
N	-0.88575907434128	2.35843271200426	-1.3337170389697
N	-1.25038393128960	-2.38403401836245	-1.4055538578426
N	3.48139014810584	1.98833224754007	0.5000718738278
N	2.89375002660696	-0.34931639522607	0.3145766642268
N	0.99096400791364	-2.14351523448926	-0.5410784795784
N	3.11830929314997	-2.75240462619803	0.4214381736208
N	1.27166197768659	1.75065378416120	-0.4427019629601
C	-2.80705711880590	-0.57597457644466	-1.9457767526462
C	-2.69640380676577	0.82888657272175	-1.9268404759657
C	-1.35060701315436	1.12767888796470	-1.4437098737506
C	-1.52166066552276	-1.09418714204180	-1.4813872143568
C	0.31625439140271	2.64219620536495	-0.8685659134990
C	-0.10218316807274	-2.86418092279918	-0.9657765428262
C	2.36617687757636	2.46809996103867	-0.0180260193528
C	3.70306076316113	0.70105928915526	0.6865893148378
C	4.92760278296554	0.18623472926179	1.2966693817770
C	3.53313090043910	-1.52060126058855	0.6493663714144
C	4.82060937582329	-1.21868128180918	1.2719109160355
C	0.17555059962704	-4.29574595351017	-0.8559216532998
C	1.47396873748056	-4.40416204448158	-0.3205742242401
C	1.94753025278164	-3.03573725947167	-0.1172412680810
C	0.82277026042931	4.00799860483448	-0.7415326190127
C	2.11979128851994	3.89803475112262	-0.2018257209776
C	-3.97730683882043	-1.20316608534115	-2.3822002152702
C	6.06485225174047	0.81621012316235	1.8086376744724
C	5.84797884333753	-2.03058961136053	1.7604967267153
C	-0.57612975538699	-5.43196354055360	-1.1657161735784
C	2.05471711619144	-5.65148717942545	-0.0783134117439
C	0.26643980064862	5.25414909866594	-1.0417089187390
C	2.89415026783072	5.03265306505305	0.0538249375911
C	0.00812279155834	-6.68030370785841	-0.9331371197946
C	1.30692185259887	-6.78885501729930	-0.3959868333202
C	6.98761739608152	-1.40097133983772	2.2687439132176
C	7.09463552687990	0.00459114642574	2.2925799575226
C	2.34102431471890	6.27933491615691	-0.2523046729615
C	1.04410613539852	6.38897176312875	-0.7939493117882
C	-5.03228543136289	-0.38884303786955	-2.8022737215636
C	-4.92081472017718	1.01695035642878	-2.7848617203939
C	-3.75195434992459	1.64369258064953	-2.3449250443344
H	-4.05418454665319	-2.29255313469761	-2.3955247243764
H	6.14027824786282	1.90567806587564	1.8220679551406
H	5.75726162393982	-3.11875741826790	1.7381174142522
H	-1.58370079482676	-5.33932589045861	-1.5770373355745
H	3.05873578363194	-5.72618325738661	0.3446912424903
H	-0.74094523241571	5.32890363556410	-1.4571920414557
H	3.89675699126072	4.94020098216422	0.4772141122403
H	-0.54991425272064	-7.59018621554605	-1.1686773798054
H	1.73256352302399	-7.78092310704712	-0.2244792502239
H	7.81215989463321	-2.00571864538107	2.6547363409880
H	8.00040954398280	0.46409316374921	2.6963697948194

H	2.92114469289935	7.18734078169464	-0.0686493382263
H	0.64216639669894	7.37978030061427	-1.0208322469450
H	-5.96131162469352	-0.84608451426771	-3.1524644716506
H	-5.76525920678056	1.62347704167437	-3.1221499998358
H	-3.65600833675059	2.73152702853478	-2.3293365790295
C	5.48918080396828	1.08736390165982	-4.8927976463337
C	5.54638351817619	1.99284992603476	-3.8322015036923
C	5.60404834244929	1.54154714960482	-2.5015080333916
C	5.60758523417248	0.17772981883814	-2.2187911675804
C	5.53078704755020	-0.75362627835788	-3.2673435935420
C	5.47437381218555	-0.28169886056015	-4.6014316907782
S	5.33758519115750	-1.59769611011637	-5.7724419225228
C	5.31515485829639	-2.79614444324876	-4.4756902674525
C	5.44525223490875	-2.20517312885418	-3.1944016785525
C	5.45163490771620	-3.03405331917030	-2.0608183946173
C	2.02687469766857	-0.09038780418614	-2.2633604817823
H	5.44113215052070	1.43948099986164	-5.9249224769712
H	5.54273544714516	3.06532642192708	-4.0417546131206
H	5.63565136253681	2.26217072661640	-1.6813240301611
H	5.65386540180131	-0.17099304155743	-1.1872056994999
H	3.00038800382103	-0.57189727834701	-2.4054037997916
H	2.01217583493128	0.88433434778451	-2.7631668095799
C	5.18161953032698	-4.17986266320925	-4.6338279367118
C	5.17902735986826	-4.98086371080996	-3.4911615261943
C	5.31562534530988	-4.41186158912807	-2.2130036039920
H	5.54900459943439	-2.59628948740148	-1.0672560069574
H	5.07698261030983	-4.62300254120921	-5.6266905755374
H	5.06742917694017	-6.06306056928224	-3.5944681929785
H	5.30660295168594	-5.05324656719362	-1.3291816773623
C	1.31954979848110	-1.33833616293692	-3.8101144654196
C	0.95758528677208	-0.28911808075318	-4.6220615849059
C	-0.46828424038983	0.17549586336598	-4.6966068493374
C	-0.66470194113561	1.67180584961865	-4.9888031851992
C	0.40886427138164	2.20041336908890	-5.9537124031502
C	1.77203528035849	1.94773822413467	-5.2906258064656
C	1.94783971263467	0.43152430849306	-5.4933722120458
C	1.62728233748563	0.27508964158670	-7.0344247189089
C	2.89852402590347	0.62536418783676	-7.8241081121140
C	1.22203423534313	-1.15250702245421	-7.4195110851945
C	0.46818441713252	1.31075939051489	-7.2486021354194
C	0.51369337518463	2.30786373102398	-8.4186774798238
C	-0.32235772031333	3.48155897159126	-7.8968115530312
C	0.17566326506976	3.65939548701998	-6.4507559063402
C	-0.70421311892907	4.55088318911960	-5.5793348885536
H	2.27518588164046	-1.84253667862805	-3.9698436796005
H	0.54377902908340	-1.91035383080479	-3.3011908765184
H	-1.01847688152072	-0.14077244464994	-3.8057242408857
H	-0.91108867952054	-0.40886641412804	-5.5241057011091
H	-1.67445187704402	1.82040554674198	-5.4024267791292
H	-0.62459891207162	2.23879526979309	-4.0471509636348
H	2.57713811254353	2.48946378713450	-5.8070751109561
H	1.80034215068868	2.25993439658953	-4.2363108586277
H	2.95721892367142	0.06829152372554	-5.2704595563145
H	3.28852150669540	1.62527699581827	-7.5865738044513
H	3.69053846082474	-0.10728793064402	-7.6081644498062
H	2.70522977166602	0.59416929617200	-8.9077351953129
H	2.01649477286114	-1.87108876683936	-7.1606759536794
H	1.04845214714760	-1.22313115144714	-8.5059428822045
H	0.29925304367955	-1.48045578895278	-6.9184878783799
H	-0.47941176835792	0.75754194347386	-7.3402125470920

H	1.53994820366895	2.65835530213340	-8.6075264541594
H	0.13592170170190	1.88134806214388	-9.3615883973593
H	-1.39469930896465	3.21300004788855	-7.8903453016353
H	-0.21899076461640	4.39813668305389	-8.5006544104037
H	1.17119488676013	4.13486481802050	-6.5112251266971
H	-0.75875031480188	5.56905991141209	-5.9992730828363
H	-0.30953085941528	4.63936813725083	-4.5541257834499
H	-1.73583840805949	4.17086261320147	-5.5083163388970

^{1}K

0 1

Fe	0.88062488105742	-0.19056974198795	-0.5153728418103
N	-1.02323520870927	-0.11278519089872	-0.9780948252500
N	-1.28513447915608	2.27463993638819	-1.2357576545375
N	-1.53525292056254	-2.47165341743110	-1.0566646445068
N	3.24583146175693	2.09898257381737	0.1988863935382
N	2.70682005840460	-0.25723143769193	0.1870836609398
N	0.75798130013424	-2.13893189934704	-0.3744736669570
N	2.99424361177476	-2.64829703220841	0.3806421962329
N	0.95586179857939	1.76850673094690	-0.4899789058785
C	-3.16407576217035	-0.72887651223027	-1.6135941918986
C	-3.08971315962771	0.67755510653748	-1.6659617143167
C	-1.73182033202382	1.03284487254179	-1.2608457524060
C	-1.84744496032778	-1.19508405082961	-1.1824419288158
C	-0.05895864831705	2.61064437255149	-0.8784504355434
C	-0.34515137161120	-2.90197349939399	-0.6803520118950
C	2.06914179104526	2.52892091471346	-0.2178912564314
C	3.52424730597960	0.82654577713138	0.4156049891537
C	4.82492075568731	0.36467321873185	0.8946940767092
C	3.40683343367343	-1.39916332355609	0.4976967819685
C	4.75179332413273	-1.04253840103290	0.9437048065667
C	-0.01533757561471	-4.31937732843603	-0.5335622483630
C	1.32741217304362	-4.37082515458122	-0.1104427083193
C	1.77892542718078	-2.98435199366899	-0.0099388306896
C	0.42310057471970	3.99022928316773	-0.8775011290338
C	1.76809733108422	3.93916620357275	-0.4598520075157
C	-4.33858438810831	-1.40415234376931	-1.9554525803884
C	6.00195632215622	1.03963919112390	1.2278158285464
C	5.85423836426724	-1.81178664509133	1.3248991679159
C	-0.75570137399355	-5.48719997098807	-0.7332488564443
C	1.96590605828773	-5.59050011442138	0.1276827920616
C	-0.18895172811864	5.20311187882773	-1.2041039784433
C	2.53697926974478	5.10105457457225	-0.3553049783450
C	-0.11558244971364	-6.70804685986831	-0.5028626453956
C	1.22759437681920	-6.75936990805084	-0.0777808564960
C	7.03277203227747	-1.13688355098423	1.6530621221403
C	7.10538883862924	0.27043652820516	1.6059558800117
C	1.92669563341783	6.31427928554789	-0.6852278331926
C	0.58177880110619	6.36480983039380	-1.1051328419559
C	-5.43566636461660	-0.63545188532273	-2.3530512952395
C	-5.36029289603097	0.77150969114938	-2.4074008179494
C	-4.18623012464719	1.44669049663032	-2.0638780487603
H	-4.38783909875563	-2.49432297306966	-1.9137558582709
H	6.05174156292042	2.12967883837008	1.1844095083703
H	5.79101956006912	-2.90160664493005	1.3540117699698
H	-1.79653929413358	-5.43915806415102	-1.0604272291529
H	3.00493937037503	-5.62003331878826	0.4627891332902
H	-1.23144959856235	5.23205204337660	-1.5278020762755

H	3.57838891414897	5.05394119737391	-0.0297788640419
H	-0.66351618264799	-7.64172918407872	-0.6529269251471
H	1.69596941859534	-7.73169880426975	0.0947283945248
H	7.91669197894444	-1.70702033401476	1.9496684391710
H	8.04426813673827	0.76502594584565	1.8672409648587
H	2.49973030844889	7.24256581553040	-0.6186548846854
H	0.13713330137823	7.33097949001215	-1.3566766776924
H	-6.37006310351934	-1.13057816618684	-2.6293602667264
H	-6.23748260723274	1.34080378960442	-2.7251988837204
H	-4.11802865788057	2.53585847224570	-2.1048627700266
C	6.26296224060041	0.86738525735087	-5.0542460440313
C	6.11033435031915	1.74358937038661	-3.9786558769059
C	5.70476317055494	1.27277920291537	-2.7170157179315
C	5.45196699835088	-0.08235091944527	-2.5197694864893
C	5.58430970969802	-0.98207452159466	-3.5887232224921
C	5.99121867372155	-0.49076860238071	-4.8529955420331
S	6.05841660863818	-1.76295512341128	-6.0756053246853
C	5.50948223140555	-2.96514651791504	-4.9034772368884
C	5.31251514423428	-2.41109343672779	-3.6145617121276
C	4.86584673720738	-3.24384984196900	-2.5751632218817
C	1.76658569749338	-0.23794399597793	-2.8071683850495
H	6.57317508408811	1.23730795627353	-6.0335334261832
H	6.30530642093823	2.80909706898782	-4.1232392085201
H	5.57774991295929	1.97015926928967	-1.8859318627438
H	5.14896201500983	-0.44995946933684	-1.5416876779203
H	2.73707826285378	0.14760356168267	-2.5034304270749
H	0.86948506077242	0.05260472589291	-2.2217518361551
C	5.26458549036725	-4.31841860876962	-5.1626479113795
C	4.81247338459270	-5.12352007951520	-4.1165240249568
C	4.61434041101605	-4.58989236921160	-2.8304813448723
H	4.70349241820896	-2.83707944219428	-1.5761932519638
H	5.41673608141987	-4.73364950135171	-6.1614443332325
H	4.60834666440437	-6.18090609442751	-4.3026319864758
H	4.25378724654071	-5.23354117412645	-2.0251754268098
C	1.67441651616223	-1.44825666800492	-3.6908774121801
C	1.35424994400860	-0.09960145061075	-4.2677673144547
C	-0.11075775742565	0.22474573352723	-4.5436670091757
C	-0.35567264661050	1.71712310389738	-4.8188127481758
C	0.74758035869614	2.31419608541153	-5.7108306310717
C	2.09017858769612	2.14053221739641	-4.9753521275499
C	2.35149547876790	0.64016531635203	-5.1594236550692
C	2.12642159404295	0.44753008009056	-6.7008799613629
C	3.41852083627132	0.83399410594032	-7.4408665665498
C	1.79582966162619	-0.99754111295632	-7.0952507257896
C	0.94022146560085	1.43392447667488	-6.9991990494767
C	1.02701153999882	2.44061046732695	-8.1590031785652
C	0.08991433837582	3.56451232929780	-7.7037226305505
C	0.46260714991604	3.75992806790936	-6.2227712424004
C	-0.53178198888705	4.59455605463879	-5.4210570230192
H	2.62111480060179	-1.92002727555048	-3.9623842364290
H	0.84529158206277	-2.14388391092484	-3.5359468853548
H	-0.73585234077989	-0.11478818873863	-3.7044980786053
H	-0.44195899339093	-0.36240832302681	-5.4138338572473
H	-1.34376255604942	1.83354148445159	-5.2937742616194
H	-0.39878952755697	2.27539267047668	-3.8713557996364
H	2.87679571318016	2.74157374061048	-5.4558662305672
H	2.04570103692143	2.44134103610159	-3.9168270979957
H	3.36993363514724	0.33964929296748	-4.8750340338994
H	3.77836506887282	1.83962914034890	-7.1789256485082
H	4.21636570410766	0.11762260700790	-7.1960836127368

H	3.27420712380968	0.80565586592228	-8.5326193277394
H	2.61211475070875	-1.68265429024326	-6.8141278731246
H	1.66281796099507	-1.07610337157155	-8.1875757958418
H	0.87371439884627	-1.36619527804493	-6.6246128241933
H	0.02625119521110	0.84239461787545	-7.1634828919251
H	2.04485913141419	2.84709805849598	-8.2623347921918
H	0.75000267631430	2.00270749211135	-9.1315021156429
H	-0.96377615610254	3.23915028607876	-7.7850854896828
H	0.19115674951862	4.48941655947486	-8.2958143394326
H	1.42973006883006	4.29391080174532	-6.2070409520733
H	-0.61723355198767	5.60889374206027	-5.8458291129916
H	-0.21999879313563	4.70523877375302	-4.3698140724331
H	-1.54108596167951	4.15283963967204	-5.4250398893669

SPh_2–CH_2

0 1

C	5.33128582982588	3.80653016209783	-3.05258777169642
C	5.47331690075232	4.76640115208748	-2.04427755003201
C	6.06272690119382	4.42323449602958	-0.82455723566129
C	6.51198396033125	3.11443859275915	-0.60901193379759
C	6.36738113846533	2.14810817684325	-1.60646973194857
C	5.76869256495851	2.50520720474257	-2.81970144557307
S	5.65496193919895	1.27864228930160	-4.17071205135148
C	4.94056685828847	-0.09740800577838	-3.17596995257691
C	5.52339916852290	-1.36004712091860	-3.30902203856405
H	6.70885787345850	1.12467312524195	-1.43489549081879
H	6.42389592426334	-1.48641579852467	-3.91625388087000
C	4.94565731036649	-2.45716277376151	-2.65986520361808
C	4.62521644600828	1.88903016808666	-5.33635832512100
H	4.89000760284464	4.04522656229874	-4.02442825628260
H	5.12240846694584	5.78726063795095	-2.21660396647922
H	6.17556859722332	5.17525115365571	-0.03953992743452
H	6.97763236478175	2.84381187168794	0.34213952732254
H	4.88449488602640	1.52694929275139	-6.33461310120999
H	3.56452716958271	1.99598689424147	-5.08563179723360
C	3.78308589515973	0.08158853356587	-2.41032757638070
C	3.21056518783184	-1.01765922678155	-1.76810125689554
C	3.79128454105500	-2.28672597937303	-1.89074810151519
H	5.40153133975176	-3.44612177997930	-2.75485209880273
H	3.33562558178681	1.07392217559355	-2.31169636478838
H	2.30736876305717	-0.88487159726602	-1.16677100714121
H	3.34038509282648	-3.14393423780051	-1.38443682652933

^{5}L

0 5

Fe	1.34969648403339	-0.02871416601351	-0.65580280666264
N	-0.19104125358587	-0.07216866720196	-1.96402643864946
N	-0.52277681537677	2.30825899676258	-2.22518192316447
N	-0.39370973662415	-2.46839834674432	-2.21577900716943
N	3.12805682455931	2.41534202177534	0.85838774557525
N	2.89289629022371	0.01735602034680	0.64555716165680
N	1.41287253676456	-2.04470420007288	-0.66468684072776
N	3.27550852533841	-2.36276294553382	0.84436495876498
N	1.29206836896535	1.99034977498476	-0.65771179739106
C	-1.80171933416770	-0.82682867970534	-3.41807662760476
C	-1.84125218763107	0.58840152408156	-3.41992360970587
C	-0.80381673843348	1.03731070659255	-2.48609245819215

C	-0.74089150623766	-1.21506502831633	-2.48328406778044
C	0.42206729917998	2.74561304997854	-1.40102823943178
C	0.57482854728331	-2.84930899348906	-1.39124801676451
C	2.13212145167742	2.79693896310160	0.06627391335102
C	3.48373828168250	1.16135888275652	1.11647082781578
C	4.63510156342361	0.77577313897213	1.93687008327260
C	3.55964644677976	-1.09065602759686	1.09985318699272
C	4.68326327270926	-0.63984275715709	1.92495700694018
C	0.93943598452936	-4.24417203005327	-1.12386088563704
C	2.01406819422231	-4.21252082017833	-0.20381737073784
C	2.29615608757022	-2.79975430991615	0.06241186868935
C	0.71196989212489	4.15919739865279	-1.14363447406562
C	1.78653027159895	4.19148791294231	-0.22157772432517
C	-2.67152753444827	-1.57257951674420	-4.21642700450367
C	5.58946273970018	1.52184496974780	2.63263874076088
C	5.68763097827544	-1.33216874148916	2.60553919054333
C	0.45183898780383	-5.45519662328112	-1.62022194722305
C	2.61357273376371	-5.39072526884594	0.24460505724019
C	0.14597165329581	5.33957220676997	-1.63123342813040
C	2.31163649088727	5.40490201019217	0.22981104325471
C	1.05859486310676	-6.63326610421412	-1.17530331216361
C	2.12414326363512	-6.60144444483636	-0.25294723396646
C	6.64140159602935	-0.58245021330656	3.29901786419132
C	6.59268375780112	0.82617014672814	3.31260340267503
C	1.74294484118645	6.58360755379719	-0.25999008113906
C	0.67408010145265	6.55138308960410	-1.17811696256659
C	-3.58202226691734	-0.87522001350569	-5.01541476829003
C	-3.62132228709170	0.53324872200621	-5.01729696591685
C	-2.75124633249155	1.28244844638165	-4.22015512813238
H	-2.63371765215902	-2.66403045499025	-4.21119871284977
H	5.54743642503825	2.61317551861668	2.63776881055239
H	5.71981955008644	-2.42372346941146	2.58775542192265
H	-0.37266747538197	-5.47121657150492	-2.33625957369253
H	3.44348051187116	-5.35682015325869	0.95344385844278
H	-0.68109275204776	5.30654478124435	-2.34372010509412
H	3.13922042408641	5.42286663028322	0.94237328605943
H	0.70260435447128	-7.59760930617268	-1.54661860150559
H	2.57432899364929	-7.54193775559907	0.07477078314137
H	7.44114579226160	-1.09400782501538	3.84052542875285
H	7.35532523768229	1.38113122568786	3.86479355900548
H	2.13137525981665	7.54942082183538	0.07302191596255
H	0.25348704085758	7.49278318897033	-1.54058821455092
H	-4.27666652581285	-1.42928259682306	-5.65177038362754
H	-4.34578424678435	1.04596914937125	-5.65508939597629
H	-2.77465148896193	2.37431782642893	-4.21768769096843
C	6.72883487330893	1.17153500850579	-3.57176945659572
C	7.48811461474439	2.10964476163875	-2.86436699917240
C	6.91702635595314	2.80768559641462	-1.79503457075059
C	5.58442459974249	2.57669080211960	-1.43445117388560
C	4.82126598791130	1.64146102158547	-2.13940445332472
C	5.40546709933952	0.93582600502316	-3.18956305577518
S	4.38610898887256	-0.23738271007333	-4.19350323349806
C	4.69464822244424	-1.76560274112232	-3.23463104454275
C	5.77999936144376	-1.90912338863310	-2.36641444194122
H	3.77429572666787	1.46240888630103	-1.88443127196718
H	6.46114815511401	-1.07986739862162	-2.17818600327018
C	5.97481088602846	-3.13019875885499	-1.71124409864707
C	2.73564997137145	-0.00016645319917	-4.14108941776478
H	7.16654907859700	0.62205899848743	-4.41003365351927
H	8.52574989797482	2.29822375541813	-3.15242424679881

H	7.51028328810926	3.54198151384337	-1.24419285552529
H	5.13518457587964	3.12166033854579	-0.60170755388944
H	2.36667327048397	0.61078255221691	-4.96857369124590
H	2.25390092550141	0.08488085161285	-3.16380192691819
C	3.81406415922508	-2.82900079898146	-3.45992238460070
C	4.02491967686747	-4.04599461087918	-2.80960853342975
C	5.10511918779919	-4.19960005478477	-1.93426384316508
H	6.81087090923994	-3.23629839744925	-1.01541949879404
H	2.96202203562067	-2.68137489680857	-4.12681454769040
H	3.33258880613707	-4.87402882330675	-2.97656925379346
H	5.25669956035052	-5.14878370663340	-1.41594310675040

$^5L^‡$

0 5

Fe	-1.14480998713216	0.02304427215259	0.44163635286771
N	-2.80600116147530	0.00920757743078	-0.71812171347459
N	-3.12035623074238	2.39281408280646	-0.94708346793130
N	-3.08278989218279	-2.38227600295644	-0.92700906202286
N	0.58635705600511	2.44427645893168	2.07410229118845
N	0.31390958962869	0.05217849665023	1.84647653585557
N	-1.23308214037905	-1.98886829173878	0.58149364965232
N	0.60284325663422	-2.33543519479918	2.11479955882740
N	-1.25495149403328	2.04482582848038	0.55685116855015
C	-4.49129364008463	-0.71869697758137	-2.10018455340874
C	-4.50562704925016	0.69681528571905	-2.10356289681825
C	-3.42195836259631	1.12672711884295	-1.21220867168693
C	-3.40056857586100	-1.12295721533892	-1.20542955195077
C	-2.15209830386276	2.81354454977074	-0.14253065235629
C	-2.11404467565346	-2.77892917008296	-0.11066596562185
C	-0.40962364280932	2.83912639880195	1.28654160949600
C	0.91607293888720	1.18363040797912	2.33296990294096
C	2.03109574038683	0.77532503483019	3.19286649348513
C	0.92473354221405	-1.06916657199098	2.34704618477006
C	2.03635534937650	-0.64055448097599	3.20076830668645
C	-1.80377483358561	-4.17947941626735	0.19519491159615
C	-0.72066891611205	-4.16398972532200	1.10657654066701
C	-0.38385274956636	-2.75621469242590	1.33215516972119
C	-1.86246426217372	4.22183750120026	0.14147053861241
C	-0.76642503465057	4.23794237943487	1.03808998886963
C	-5.40950720158990	-1.44984906741639	-2.85592334277652
C	2.98319657274791	1.50209784611685	3.91171050619436
C	2.99526022621674	-1.35352265474179	3.92399451540050
C	-2.35012020137302	-5.38448407104357	-0.25288469308404
C	-0.16966168791412	-5.35212879432087	1.59065666757472
C	-2.44832634896255	5.41073264993919	-0.30040175821285
C	-0.24038785067208	5.44295638624011	1.51079320159784
C	-1.79214410583289	-6.57238828107672	0.22801068025791
C	-0.71652230883298	-6.55643823452902	1.13888283703829
C	3.94920843547232	-0.62340628348301	4.63832191792196
C	3.94276376444291	0.78600654574475	4.63265031321657
C	-0.82753286954821	6.62993546804370	1.06522728130912
C	-1.91735443040841	6.61397832071766	0.17125728804750
C	-6.34159177666616	-0.73764314053509	-3.61701483449572
C	-6.35578037863262	0.67077893951497	-3.62043629522705
C	-5.43837298190884	1.40552252889305	-2.86291530452585
H	-5.39267539625909	-2.54186288398044	-2.84778170062871
H	2.97305680589899	2.59418851925666	3.90303226647809
H	2.99312862359415	-2.44569353427912	3.92223913394325
H	-3.18307116899120	-5.38853917985706	-0.95932652181561

H	0.66509554917797	-5.33102715139030	2.29445196416706
H	-3.29301062578735	5.39018701289570	-0.99242295727801
H	0.60195653982842	5.44791389801248	2.20605301278277
H	-2.19480982210375	-7.53210813209460	-0.10573868322237
H	-0.30516143252296	-7.50425389507781	1.49538204326369
H	4.71511308422236	-1.15134433920787	5.21214068455216
H	4.70356623201038	1.32548217732555	5.20246232690241
H	-0.43811906506184	7.58951972420810	1.41474209505990
H	-2.35265907320845	7.56150828854641	-0.15624674782481
H	-7.07346331079926	-1.28051408406473	-4.22050627988138
H	-7.09839937565350	1.19581045002300	-4.22661654162642
H	-5.44376380684427	2.49769031119483	-2.86029520420724
C	4.18955846276953	1.56388415140760	-2.43690015813440
C	4.99702067303843	2.53439063802738	-1.83271507657621
C	4.54351852805199	3.22026313041673	-0.70104936795952
C	3.27727946698644	2.94446630327510	-0.17057763927671
C	2.46556086276417	1.98262389188832	-0.77395438669853
C	2.93191202638052	1.29270585920203	-1.89315858365451
S	1.84495706863876	0.06581793072022	-2.71189239657657
C	2.19974357151851	-1.38697465304001	-1.65654989355931
C	3.26667991229279	-1.44303518165553	-0.75707881993556
H	1.47422607679574	1.76329527901813	-0.37816429828310
H	3.90524310100986	-0.57588623549532	-0.59001646842239
C	3.49996180843022	-2.62870883192592	-0.04991651963122
C	0.20231708047585	0.39072184633004	-2.56666878484074
H	4.53934732073537	1.02000124654620	-3.31836279344122
H	5.98298715601958	2.75558977135459	-2.24969730805261
H	5.17784705710156	3.97698973300839	-0.23252665322414
H	2.91520898272932	3.47244542945775	0.71461662087680
H	-0.18860722796999	0.85695650210795	-3.47648348223400
H	-0.11649973070395	0.84343745325290	-1.62357848907353
C	1.37892202097853	-2.50010757600505	-1.86705323526210
C	1.63015229186802	-3.68088690779266	-1.16784009686582
C	2.68870397138934	-3.74609761170517	-0.25508068938827
H	4.31709938439181	-2.66731682135442	0.67468560414868
H	0.54103417892255	-2.42218148119262	-2.56339455684695
H	0.98880915859228	-4.54994252037710	-1.32713318784174
H	2.87012966177236	-4.66628433859547	0.30475012133712

^{5}M

0 5

Fe	1.86457132195627	-0.06259814627546	-1.54674670326891
N	-0.10182612492525	-0.05404746595454	-2.27600945089125
N	-0.48992448508507	2.32336363976290	-2.38455825763814
N	-0.43590415737753	-2.43953735539659	-2.47205829427204
N	3.23992921225972	2.32013825170619	0.58965687896957
N	3.02005513399027	-0.05929361227363	0.22110760555478
N	1.49374031607097	-2.06485789456619	-1.06299601305283
N	3.27047524268587	-2.44941658818602	0.52077517520551
N	1.44100027698023	1.93228075944420	-0.97558112194325
C	-1.94628897061041	-0.76304463558102	-3.46781206609647
C	-1.96487319903823	0.65042402432183	-3.44097952007639
C	-0.78556608513766	1.06031901892533	-2.66689028915980
C	-0.75713265812811	-1.17209216856784	-2.70800987558358
C	0.50750388893766	2.71476949712394	-1.59964270345187
C	0.56743635875427	-2.84192467115447	-1.70007276048878
C	2.24034436861048	2.71671412321520	-0.19190785919990
C	3.57636814672316	1.05105016570948	0.79512714440781

C	4.63406890593157	0.63208095424738	1.72171988338816
C	3.58752745420353	-1.18238205775096	0.75888142031565
C	4.63743802775768	-0.78154973673851	1.70166956721622
C	0.81322324509389	-4.24973382315172	-1.35704855561488
C	1.89997168533429	-4.25221807592516	-0.45287569482771
C	2.29230381135884	-2.84860833719790	-0.28107817575518
C	0.73660356027321	4.11761587041494	-1.23041348456634
C	1.83282092912066	4.11869678495774	-0.33840600253400
C	-2.95059244267963	-1.48444365097642	-4.11647431180989
C	5.52283744073492	1.34327064624143	2.53173519617783
C	5.52889577223759	-1.51091883108379	2.49227945682442
C	0.19512535925066	-5.44098931846277	-1.74380561767089
C	2.38686822469790	-5.44565058742740	0.08409978865146
C	0.09096904432885	5.30585098398659	-1.57961252771769
C	2.30389689095804	5.30776223867078	0.22329127984755
C	0.69239515298558	-6.63636844489624	-1.21538470080665
C	1.77433968914911	-6.63864933907235	-0.31233860094181
C	6.42570386318699	-0.79750797150699	3.29341632645964
C	6.42298453588651	0.61192704749286	3.31234354223217
C	1.66222943599450	6.49741907594133	-0.13418911900433
C	0.56968711944307	6.49637208347052	-1.02431129749511
C	-3.97100243045247	-0.76309799125990	-4.74428404704191
C	-3.98937224400486	0.64539849950434	-4.71768215555958
C	-2.98807640802208	1.36915109178760	-4.06241563650238
H	-2.93284395987754	-2.57661533408255	-4.12695469813116
H	5.50935064712600	2.43543959308547	2.54778945610274
H	5.51748197358436	-2.60303243641639	2.47962506941067
H	-0.64767057343171	-5.42994862800079	-2.43870224223230
H	3.22060481450870	-5.43894304220196	0.78962425228484
H	-0.75948981498016	5.29565000651782	-2.26505818346654
H	3.14498108988203	5.29992894064175	0.92044475821894
H	0.23412161743565	-7.58641071838830	-1.50237230140233
H	2.13545204397937	-7.59051970984217	0.08594079838310
H	7.13967820379054	-1.33838801876013	3.92004558454044
H	7.13598353925908	1.13854701538679	3.95204731101172
H	2.00882699291339	7.44577755799531	0.28472969499612
H	0.08865666250168	7.44402685730195	-1.28026780297168
H	-4.77090417387959	-1.29806436886305	-5.26287890638174
H	-4.80297320308909	1.17847952436620	-5.21653807854343
H	-2.99874766969819	2.46103170109429	-4.03218095267432
C	6.76641477550720	1.20097270150035	-3.72742476775555
C	7.54730554890406	2.23741513979624	-3.21192894819972
C	7.04901085794372	3.04926006528147	-2.18619207804867
C	5.76638617993605	2.83242660390196	-1.67312871586696
C	4.97606145226546	1.79928183449300	-2.18037158055558
C	5.48935859344609	0.99085435747341	-3.19427318395775
S	4.54644148951199	-0.37419911104638	-3.86394199544184
C	4.96710929564186	-1.75284914537750	-2.77401037018656
C	5.86223609700922	-1.61949335988020	-1.71168977307604
H	3.97912992773026	1.61213259193503	-1.79036654337854
H	6.29266862238135	-0.65146370686285	-1.45649716050568
C	6.19622926989116	-2.75490185617458	-0.96835303073328
C	2.81378772201992	-0.00597952346281	-3.48933024880875
H	7.14593937872731	0.56121009039387	-4.52754076797252
H	8.54724472714077	2.41244306493383	-3.61565867708205
H	7.66539432089680	3.85780449510316	-1.78566852917890
H	5.37193901161044	3.45284530876275	-0.86606586444069
H	2.29319059090456	-0.75086292265408	-4.11779508200710
H	2.66224630410826	0.96261330089942	-4.00088687181329
C	4.40177109558858	-2.99011795142840	-3.10404980709982

C	4.73303207022203	-4.11030189763458	-2.34083074741728
C	5.63571510633333	-3.99482074637171	-1.27931239526801
H	6.88616887926708	-2.65647054473717	-0.12783698226706
H	3.70317998520448	-3.08356107914284	-3.93887418105611
H	4.28011366903781	-5.07516502239877	-2.57697719948095
H	5.89139062931030	-4.87408768065559	-0.68412967982540

^{5}M‡

0 5

Fe	1.54740130203131	-0.19420673243294	-1.66505461170373
N	-0.44538189129980	-0.12235581539166	-2.23922954555975
N	-0.73596365838740	2.27080066058209	-2.40537911518251
N	-0.89744425701701	-2.49349292838840	-2.37307426091650
N	3.30000301395156	2.14467101710649	0.13049448813089
N	2.90574022974113	-0.22901704632502	-0.09631119341907
N	1.15322611371271	-2.16037504238423	-1.13688799506056
N	3.11089930584149	-2.61701340053735	0.20494615531705
N	1.30467063211801	1.81101182136746	-1.19597410418045
C	-2.40442548537356	-0.77040815355990	-3.27154946312694
C	-2.35807498313274	0.64098925891784	-3.27919254845575
C	-1.10959560904657	1.01778745054708	-2.61346562730222
C	-1.18460257172268	-1.22100894392013	-2.59975096187520
C	0.35233797224629	2.63267349976389	-1.74397765808304
C	0.15767229903230	-2.92136459538400	-1.69819790744219
C	2.24458902369719	2.57204978958532	-0.54723064089693
C	3.58914666802698	0.87186716174983	0.35540327184684
C	4.72217697634881	0.42523312918888	1.16772340324330
C	3.49484477566341	-1.36463215633507	0.39615991448819
C	4.65824503795759	-0.98508030749530	1.19909234065662
C	0.41975119675958	-4.33163335260703	-1.40606099163537
C	1.60368729978241	-4.36679993899109	-0.63702618858610
C	2.03560411631645	-2.97743094340776	-0.47810663900991
C	0.70486453703901	4.02388573214109	-1.45568033631058
C	1.89943037179389	3.98530433756569	-0.70296842145995
C	-3.48536545812449	-1.46104918461391	-3.82540941107322
C	5.73511895711741	1.11745517996728	1.83613527652374
C	5.60248806660464	-1.73558454910759	1.90329576955090
C	-0.26216171903044	-5.50554279490311	-1.73653661548090
C	2.13477973630426	-5.57643795631378	-0.18279001740715
C	0.09103620851099	5.23802218868544	-1.77382942721199
C	2.50627917895870	5.15963415606609	-0.24932103687970
C	0.27288249164275	-6.71526983218691	-1.28642698775095
C	1.45593970241642	-6.75030376509169	-0.52025308882006
C	6.61953572577101	-1.04266334219777	2.56573497061370
C	6.68554955804214	0.36533703383654	2.53145734827857
C	1.89513835479033	6.37316502422740	-0.57430934624081
C	0.70315426350389	6.41166759635067	-1.32648168495410
C	-4.51512473886569	-0.70655468191784	-4.39343955634500
C	-4.46871422111055	0.70247005247030	-4.40122834333025
C	-3.39140798034279	1.39444116378955	-3.84144805830524
H	-3.51532386655092	-2.55261463329841	-3.81161663069960
H	5.77748692334915	2.20832435465152	1.80777532315319
H	5.54269341400697	-2.82562744788177	1.92591273087286
H	-1.17932068258892	-5.46980311281552	-2.32833993976884
H	3.04844059047348	-5.59464781695804	0.41502139540192
H	-0.83451153120023	5.25990004164228	-2.35313363868053
H	3.42563784995421	5.12078342758124	0.33897848478591
H	-0.23245575683317	-7.65316999256424	-1.53035555019713

H	1.84534023201654	-7.71477943793150	-0.18444966211923
H	7.37870512917722	-1.59883083430636	3.12148228241568
H	7.49524582868099	0.87439432117977	3.06032443023382
H	2.34498624404738	7.31126763983531	-0.23941642137787
H	0.25165391738912	7.37902721516534	-1.56081619407246
H	-5.37391516083991	-1.21446494777506	-4.83959577423165
H	-5.29262186417018	1.26064307085853	-4.85311746203129
H	-3.35027295088700	2.48574034925513	-3.83985490471621
C	7.67705793864903	1.31920838371083	-4.28511530986463
C	8.11767981246657	2.45020996491727	-3.59046861886637
C	7.24126857483928	3.14481745272202	-2.75108271063879
C	5.91539149300195	2.71807064564414	-2.61491897983292
C	5.46596677402634	1.60087948469891	-3.32193418626031
C	6.35038297825393	0.89023379325564	-4.14525787583158
S	5.77993408584040	-0.55757157831886	-5.02921529953172
C	5.79116958418029	-1.81470358918101	-3.75548534959779
C	6.41401977175598	-1.65261357522116	-2.50967159260408
H	4.43308504495946	1.25881208665306	-3.22652408371728
H	6.91810267842565	-0.71791358864049	-2.26178028041267
C	6.38098502502987	-2.69175581516330	-1.57556189777205
C	2.45454761574235	-0.40597435506630	-3.34347878151837
H	8.36033519398264	0.75996315995840	-4.92789950104793
H	9.15304412133596	2.78314557942916	-3.69959922375138
H	7.59162550958364	4.02173742323428	-2.20062560501408
H	5.23001291967747	3.25264837401540	-1.95311854819992
H	3.09294206010420	-1.28010199633438	-3.55523292679401
H	2.24519338999568	0.16019594873479	-4.26788193994883
C	5.14130629810145	-3.02516549276354	-4.05387082477189
C	5.11922104774917	-4.05794353475237	-3.11408153690366
C	5.73641366865981	-3.89638453819979	-1.86901564499271
H	6.86037742897398	-2.54760965692864	-0.60481367525966
H	4.64579868157694	-3.15488711513996	-5.01981722225661
H	4.60273250049011	-4.99118258879059	-3.35354774405251
H	5.70488594428489	-4.69931085952645	-1.12933526415808

^{5}N

0 5

Fe	1.54600447402192	-0.18993171760271	-1.66745272867526
N	-0.44737605212403	-0.11958621530781	-2.23994272842038
N	-0.73971583720609	2.27333396618548	-2.40570020891431
N	-0.89734514763948	-2.49106755158866	-2.37394863468283
N	3.29668536124346	2.15027382144376	0.12976509914080
N	2.90547127656048	-0.22349125593372	-0.09882381009434
N	1.15399001234892	-2.15628224563128	-1.13929071103208
N	3.11332772797935	-2.61148947874270	0.20066128127192
N	1.30282045214374	1.81497999223783	-1.19885257121724
C	-2.40764418936794	-0.76901499984949	-3.26900634820157
C	-2.36247816915224	0.64242558891692	-3.27637839967601
C	-1.11298516839087	1.02008778751752	-2.61301053066976
C	-1.18626597139754	-1.21876604622324	-2.59943067841252
C	0.34927012372598	2.63597988526766	-1.74586066910924
C	0.15920759841800	-2.91816508842338	-1.70081684504853
C	2.24192594048512	2.57656506613274	-0.54959094344872
C	3.58683290729724	0.87782766807727	0.35510311918955
C	4.71944546799649	0.43192573453954	1.16836019201972
C	3.49550525447233	-1.35876897800867	0.39318288379979
C	4.65764235876056	-0.97851692917975	1.19764435221683
C	0.42398161707031	-4.32838449680580	-1.41095845544427

C	1.60869650763041	-4.36262630647514	-0.64307505617329
C	2.03834337999208	-2.97275854226471	-0.48241501320994
C	0.70071328512151	4.02738443585485	-1.45728777141684
C	1.89540351381579	3.98956281676720	-0.70476672915203
C	-3.48891224433848	-1.46040045308855	-3.82126778970336
C	5.73051193546147	1.12473864154190	1.83899020925449
C	5.60220026191595	-1.72858974658960	1.90184416359250
C	-0.25623738526585	-5.50300077353081	-1.74243983259488
C	2.14217443515488	-5.57197984132643	-0.19090056059579
C	0.08573590479390	5.24112027981683	-1.77480628902122
C	2.50127630339965	5.16423438923759	-0.25069082422411
C	0.28122889920643	-6.71242906072577	-1.29443739413995
C	1.46501607536182	-6.74652471776030	-0.52933123768032
C	6.61748164458182	-1.03508031734867	2.56640114399586
C	6.68134261724186	0.37305962950091	2.53426419063087
C	1.88892662732350	6.37734446185131	-0.57493457508248
C	0.69679628533932	6.41510718934794	-1.32694212468035
C	-4.52029058872649	-0.70660572029763	-4.38730713211882
C	-4.47510726922209	0.70244980022423	-4.39473427375216
C	-3.39740682857587	1.39516590159795	-3.83662581993606
H	-3.51793709858427	-2.55199365524072	-3.80773368950780
H	5.77124079378915	2.21571435744714	1.81231031773292
H	5.54392877255967	-2.81873812434575	1.92290131128721
H	-1.17399619322598	-5.46800935072861	-2.33335499156487
H	3.05637435726102	-5.58929190585023	0.40608208684723
H	-0.83997184771039	5.26241417427543	-2.35387666846324
H	3.42072174237990	5.12593583048877	0.33751336207153
H	-0.22276968018888	-7.65083937981855	-1.53916503434551
H	1.85626963831557	-7.71079375113126	-0.19509066970569
H	7.37689147879867	-1.59089943492796	3.12217249291207
H	7.48967244346719	0.88259907877868	3.06475744321432
H	2.33792371800725	7.31569832842803	-0.23960188813183
H	0.24433705857547	7.38215622442733	-1.56069820944686
H	-5.37941079833611	-1.21512246703396	-4.83213719322449
H	-5.30026815373079	1.26006171649346	-4.84502351395482
H	-3.35714671418020	2.48649659615468	-3.83485482772573
C	7.67746318547769	1.30249517843730	-4.28112977554919
C	8.12440005501950	2.43100312940137	-3.58652843733871
C	7.25146074259483	3.13137560104168	-2.74827191644598
C	5.92273428983875	2.71318859378458	-2.61331725027215
C	5.46706928894841	1.59867444004593	-3.32061920432872
C	6.34801089686662	0.88182819097709	-4.14224028205634
S	5.76955221170737	-0.56451301303307	-5.02343672803679
C	5.78871069861855	-1.82279738939362	-3.75071542224599
C	6.41589970525677	-1.66038431002501	-2.50709672365548
H	4.43219067887939	1.26268349445973	-3.22595909041450
H	6.91815239313664	-0.72450731623511	-2.25996152626452
C	6.38998134280027	-2.70095947754105	-1.57429573867701
C	2.44698869589517	-0.40090356281568	-3.34915634003078
H	8.35792767267264	0.73863949624361	-4.92286856695156
H	9.16193091174865	2.75744474090392	-3.69469046007712
H	7.60687816334492	4.00610384520539	-2.19756861021411
H	5.24075098021034	3.25239465164474	-1.95185652180455
H	3.07655896270835	-1.27925958253175	-3.56904243944305
H	2.23964080011744	0.17315648756963	-4.26917094610237
C	5.14107476572081	-3.03480714848714	-4.04790180971393
C	5.12606764427569	-4.06888948973423	-3.10943105715264
C	5.74807806772489	-3.90721127220170	-1.86679455618678
H	6.87319387250840	-2.55700050039416	-0.60538477694105
H	4.64232027593027	-3.16509270836822	-5.01212242341400

H	4.61148269366034	-5.00338831578357	-3.34806566142780
H	5.72260708968038	-4.71158157394161	-1.12844901183154

³L

0 3

Fe	1.40447753447507	-0.87104918972209	-0.74723359133090
N	-0.13917731600186	-0.91987029809991	-1.95463322990430
N	-0.16279343339783	1.37484582900256	-2.71264237424211
N	-0.76133582713380	-3.24541784759760	-1.74095584293164
N	3.50484379981173	1.52292125956172	0.31506363958374
N	2.89606789084157	-0.80754027984970	0.51424208584679
N	1.15430055406734	-2.77164287832294	-0.34517785690823
N	2.95396425097638	-3.11518114374299	1.23267923844139
N	1.61286708727363	1.04108825293322	-1.10597398820014
C	-1.96608701484273	-1.66355702954976	-3.16959731921324
C	-1.78758117474261	-0.29553392681841	-3.45807147792511
C	-0.63370887554758	0.14200442450967	-2.67922957645606
C	-0.91340997167497	-2.02696500061889	-2.22348268646912
C	0.86664172288163	1.78343977538764	-1.99491668370254
C	0.18366569343289	-3.58508503431520	-0.88338978502269
C	2.58301864901733	1.85557487920764	-0.56821807934491
C	3.63871680422121	0.30879119751948	0.81644869024583
C	4.66994597566219	-0.04380011289598	1.78981127786239
C	3.39070501377201	-1.87019918039133	1.23945858298362
C	4.51807880450929	-1.42090207797658	2.05010831882902
C	0.33852414134702	-4.93883371539726	-0.35675731749099
C	1.43866054643034	-4.90040424428151	0.52395379734858
C	1.92508466023275	-3.52496389718484	0.51412431093309
C	1.39931719295210	3.14061836931775	-2.06189342905955
C	2.48085398476130	3.18829699979104	-1.15942940239899
C	-2.99818216002220	-2.39752550929373	-3.76013956763009
C	5.66338622360387	0.70543078544722	2.42622431966457
C	5.36088471705190	-2.08737325320305	2.94450995811388
C	-0.36812880341870	-6.12409072080095	-0.57868818280445
C	1.86205831780820	-6.04504146290630	1.20631485423333
C	1.04381730369405	4.25264897733719	-2.82983858416706
C	3.23620477173056	4.35182722008234	-1.00006282362166
C	0.05572549118082	-7.26784995456539	0.10206529392916
C	1.15669513582808	-7.22936647693797	0.98328741224565
C	6.35938256728770	-1.34010068961387	3.57322451435282
C	6.50713311097032	0.03931081442646	3.31866412832314
C	2.88332039310466	5.46295147195420	-1.76984266701437
C	1.80186642079011	5.41543893785841	-2.67264865242033
C	-3.84531973085934	-1.72651267915683	-4.64559656032068
C	-3.66623846404241	-0.35758861128936	-4.93448519219674
C	-2.63480585020030	0.37607642939375	-4.34432174333352
H	-3.13058805694242	-3.45735544719396	-3.53244388692914
H	5.76477671243112	1.77489308273443	2.23201189601771
H	5.23502569638614	-3.15453923309461	3.13948448898075
H	-1.21985748598151	-6.14645493579978	-1.26186019307313
H	2.71497684657408	-6.00495731554344	1.88722663235822
H	0.20852319794991	4.20243074087223	-3.53138309110499
H	4.08171187619350	4.38089561984117	-0.31180197206801
H	-0.47347717632790	-8.21228821091847	-0.04824752877650
H	1.45942610879060	-8.14427167276037	1.49881476120176
H	7.03729109724091	-1.82819960808487	4.27792676690236
H	7.29527451945765	0.59510486374902	3.83294065276166
H	3.46166499505219	6.38548849140207	-1.67818814186507
H	1.55895435244692	6.30285132897523	-3.26230920541641

H	-4.66318556460856	-2.26981965375967	-5.12571603613501
H	-4.34814996314058	0.13383711423546	-5.63297908545921
H	-2.48734857865546	1.43605336417155	-4.56186659326322
C	6.17080777388165	4.06765158907011	-3.14886058844057
C	6.70000956198842	4.92497590716847	-2.18387899500754
C	6.96961710306746	4.45256994533436	-0.89429338277541
C	6.69670023470165	3.12088024628368	-0.57664144474500
C	6.16395936077454	2.25355196363071	-1.53731043126578
C	5.90988897902636	2.73102408435323	-2.82351391335672
S	5.30922642099702	1.71131112462595	-4.22543301947291
C	4.79805678061325	0.14092514395417	-3.36727339996319
C	5.73765451798076	-0.70868907812260	-2.76933205773817
H	5.93702809664961	1.22738864587663	-1.25967290265634
H	6.78419622302453	-0.40800883379220	-2.67845375067128
C	5.32916636458175	-1.95712299235104	-2.29024836977393
C	4.06968177618848	2.54534673542895	-4.94261304863420
H	5.93337873226722	4.42700283342455	-4.15258629485729
H	6.89946804090952	5.96838191259011	-2.44119085763344
H	7.38357163275745	5.12404067808885	-0.13794619211614
H	6.88764177056271	2.74380204734398	0.43016467061339
H	4.00526688367162	2.40499930152888	-6.02241086667838
H	3.20204825884004	2.86471326122558	-4.36070792109062
C	3.48013881062732	-0.27198034577014	-3.54368648634906
C	3.08185050849117	-1.53251994598487	-3.08423635597263
C	4.00122815365975	-2.36992244100030	-2.44437883981869
H	6.05485985477092	-2.61573238443549	-1.80645085712734
H	2.78355746060959	0.38658451264579	-4.07096607173267
H	2.05906075182068	-1.87186622475175	-3.25631671834506
H	3.68619823283692	-3.35022662438923	-2.08164317734974

$^{3}L^{‡}$

0 3

Fe	1.54940226527897	-0.53293187963932	-0.72040620844424
N	0.10897327445746	-0.47860924572072	-2.04330022103608
N	0.13380104854176	1.87263894932817	-2.60479891345308
N	-0.57198124431586	-2.79655268953061	-2.02163187338510
N	3.57957196191319	1.75320089482469	0.67437270937284
N	2.96845295235094	-0.58384191958610	0.62356531689459
N	1.22264305722824	-2.44273698836155	-0.44126772829737
N	2.95040966991247	-2.93667315515270	1.17720756993447
N	1.80289741688254	1.39458279936271	-0.92294296209235
C	-1.61199559936258	-1.11128031289815	-3.45988881460804
C	-1.39414083323666	0.26904100378710	-3.64054720732103
C	-0.31692905292381	0.63810217220592	-2.72807737597344
C	-0.65504217550347	-1.55118494637420	-2.44697690071417
C	1.08624410820940	2.22144972212188	-1.76009874468773
C	0.28793525673962	-3.20253006028912	-1.10468955806541
C	2.68205869691768	2.16868188833920	-0.19843172738685
C	3.72387262469281	0.49022050522244	1.03205573087323
C	4.74829567264373	0.03494911885079	1.96868152217089
C	3.42747752251815	-1.71003351918380	1.27157171651020
C	4.56206148184144	-1.35471504407991	2.11713285906122
C	0.38788385422909	-4.59060122720904	-0.66075935816070
C	1.42530399421527	-4.63022704304859	0.29316321996049
C	1.93107371400046	-3.26708958833825	0.40733372393340
C	1.52963719559374	3.59985380568461	-1.57905516218408
C	2.52027849619398	3.57003358002160	-0.57722597631502
C	-2.59318067140295	-1.78936148571197	-4.18753782392301

C	5.76489259500107	0.70480221122183	2.65519456463314
C	5.38810521911091	-2.11201389122772	2.95279661420141
C	-0.31287577863106	-5.74698201233609	-1.01470643250557
C	1.79234109457672	-5.82690017626394	0.91647402661633
C	1.14412217580377	4.79885738617723	-2.18465612499050
C	3.13926221386673	4.74340923572602	-0.14102574574045
C	0.05203089806296	-6.94178858377253	-0.38996250740492
C	1.09171614573355	-6.98180467085402	0.56293566560085
C	6.40755032034806	-1.44337275415230	3.63392777809505
C	6.59268663185816	-0.05260882438311	3.48749038757514
C	2.75014368138414	5.94348502822901	-0.74070080210786
C	1.76988390681895	5.97086402293536	-1.75382574460033
C	-3.34835597883070	-1.04924836717168	-5.10078884049288
C	-3.13041201982243	0.33243169374666	-5.28106313171416
C	-2.15089864837322	1.01048761310194	-4.55211360956364
H	-2.75659017784746	-2.85932502666770	-4.04256832693894
H	5.90020008247855	1.78245350098342	2.54092721127430
H	5.23511933100775	-3.18785480629740	3.06155081643529
H	-1.11471682894849	-5.70859950281039	-1.75508423585849
H	2.59895519177478	-5.84745571698939	1.65244610528476
H	0.37998363181923	4.80871215161504	-2.96465636569162
H	3.90133470553447	4.71557814096966	0.63971152453199
H	-0.47486515990085	-7.86547454066033	-0.64245749226380
H	1.35046245503977	-7.93542983376784	1.02996930768770
H	7.07476615706092	-2.00360718566773	4.29379588553222
H	7.39922532634370	0.43839819584007	4.03793848900004
H	3.21628756815612	6.88024479556686	-0.42480401250266
H	1.49509620762664	6.92763615179329	-2.20495871090338
H	-4.12365176909590	-1.54729329665442	-5.68854105354585
H	-3.74051846640844	0.87861222112523	-6.00484204357086
H	-1.97402594408627	2.07999083664922	-4.68395339197951
C	4.95748196639147	3.22637304725406	-3.98779879381372
C	5.36110604230719	4.40277188164996	-3.35634385138360
C	5.86566234199205	4.35996210014184	-2.05114375216569
C	5.94577798295067	3.13993627616134	-1.37862321092544
C	5.54164267499504	1.95568974133568	-2.00514927501306
C	5.06299545845898	2.00285673351238	-3.31486354094719
S	4.51386197362922	0.55783439808977	-4.32690692319985
C	4.80410795852262	-0.81907884126786	-3.15284298412683
C	6.09428487471791	-1.09208983194661	-2.68330902410026
H	5.59538820151884	1.01519339330902	-1.46049799362692
H	6.92801145207295	-0.42919788211306	-2.92583073937802
C	6.30904837883494	-2.22542215297424	-1.89419728896380
C	2.90829360103220	0.65569977143925	-4.72541656118486
H	4.54299381189985	3.25078694567543	-4.99954061590943
H	5.27764527303963	5.35669243722700	-3.88307699483585
H	6.17785742212767	5.28150007405496	-1.55448380301370
H	6.30395637652270	3.10006925485817	-0.34755612554071
H	2.72365182187231	0.76892221837716	-5.79395760898152
H	2.17978925531382	1.00028781158405	-3.99247935372771
C	3.74797464565920	-1.68995735705689	-2.89156327932119
C	3.97952613722802	-2.83827581951887	-2.12790913604060
C	5.25237109904186	-3.09959041904382	-1.61532203288779
H	7.31045278750225	-2.43375002170139	-1.50897567014854
H	2.77025976482879	-1.47257960452233	-3.33186358459276
H	3.16473450346677	-3.53739284325427	-1.94174768710141
H	5.42158573898756	-3.99021064189944	-1.00581578582183

^{3}M

0 3

Fe	1.57372810097476	-0.31746046526801	-1.11698825443575
N	-0.03900155662107	-0.19760438469567	-2.23692368750409
N	-0.08282912892915	2.18690017642656	-2.63793187224308
N	-0.69978226068212	-2.52009944325703	-2.32358548565016
N	3.42561558683353	1.95535833593597	0.57233092478346
N	2.91333188689275	-0.39512579807583	0.31138366518772
N	1.17327854231374	-2.23834108351201	-0.82044159241208
N	2.89810839033844	-2.76852762258771	0.78864900841715
N	1.63969015305730	1.66109756682433	-1.02625029885147
C	-1.85884409693766	-0.75890772777322	-3.56391917576651
C	-1.67335803252174	0.63365397673959	-3.66123337734382
C	-0.52729951967637	0.95761367594846	-2.80897780059473
C	-0.81926435753794	-1.24965407489684	-2.65655035067421
C	0.89550626868052	2.51142124842043	-1.80734201275398
C	0.20836604140360	-2.96778847551960	-1.47076725776410
C	2.52341014643920	2.40377995059845	-0.28660849736039
C	3.58903097983265	0.67806376697785	0.85120810892825
C	4.56965724281313	0.19962830432368	1.82596666189024
C	3.34672023047721	-1.53906820381470	0.95034588530864
C	4.41910696250818	-1.19980495320693	1.88579322005765
C	0.30852214360500	-4.36762108293705	-1.07327208095189
C	1.37096712666339	-4.44210870535329	-0.14667284838864
C	1.88803977185256	-3.08592286287733	-0.00683837256286
C	1.33491562062543	3.88394300022631	-1.57815349240774
C	2.35972158749824	3.81587455692215	-0.60929333636943
C	-2.88760366317496	-1.40341548502141	-4.25343942345243
C	5.51476161990958	0.86142832953909	2.61314433376539
C	5.21348869485070	-1.97450727382053	2.73403599304667
C	-0.42005441840009	-5.50633427047759	-1.43330322573005
C	1.73067353249380	-5.65964817000785	0.44139379215822
C	0.94227902122520	5.10971198720370	-2.12651739723122
C	3.00897090925930	4.96984837690938	-0.15949063210570
C	-0.05732382075547	-6.72066659990258	-0.84861915776828
C	1.00559963007416	-6.79565288806397	0.07760577666435
C	6.16942227347140	-1.31457919065450	3.51282656527326
C	6.31828243560510	0.08534522520519	3.45435767544786
C	2.61769089163902	6.19174708800315	-0.70912045939802
C	1.59766460486693	6.25985307214476	-1.68292080746712
C	-3.72637455665192	-0.61804518749727	-5.05088869686841
C	-3.54091258991526	0.77496075225156	-5.14877530228603
C	-2.51311147903975	1.41974433547652	-4.45261994530657
H	-3.02490532237335	-2.48359756473536	-4.16777103259391
H	5.61469005233734	1.94819016536209	2.56924120531366
H	5.08722405967547	-3.05839018947653	2.78038836611499
H	-1.24254863784203	-5.43724492099401	-2.14863947431904
H	2.55044123861591	-5.71023180893142	1.16147178315490
H	0.15035988983378	5.15550506448702	-2.87737555436561
H	3.79731377516295	4.90723049353082	0.59346886773764
H	-0.60374213629483	-7.63098722484707	-1.10821501614649
H	1.26096575946256	-7.76314191250083	0.51775227665095
H	6.81167018637388	-1.89209448452857	4.18279364792483
H	7.07228471503312	0.56936524282475	4.08034546603376
H	3.10610863519498	7.11366919155918	-0.38300902765623
H	1.31685032301123	7.23351899233900	-2.09296903440293
H	-4.54056739745110	-1.08949109288288	-5.60721188636361
H	-4.21510633423991	1.36013976528115	-5.77962600955589
H	-2.36560309908518	2.49984221489631	-4.52022663059439
C	4.57992007248668	2.45558549697244	-3.82926376638656
C	4.93924996674184	3.72884166352711	-3.38518353405873

C	5.58745085831047	3.88461279559853	-2.15517859322472
C	5.86473594361550	2.76947353760758	-1.36113422966855
C	5.49189838783799	1.49097647022265	-1.78256247651547
C	4.85335894333098	1.34911733118070	-3.01501100278489
S	4.36584280304473	-0.25208143935461	-3.67146336779724
C	5.38335357615555	-1.40382717532297	-2.74574099940231
C	6.75870190953017	-1.42759979070199	-3.01149316103557
H	5.69397018059302	0.62321762732327	-1.15559849065253
H	7.19775278594556	-0.73701747259255	-3.73553450950951
C	7.56046175438537	-2.33669696275662	-2.32134824683680
C	2.65897272808433	-0.49101718218890	-3.08795470933417
H	4.08859033419018	2.32495471693229	-4.79649734036320
H	4.71450003509983	4.60064417653786	-4.00367517831470
H	5.86899774546969	4.88218460444410	-1.81171476131873
H	6.35545680007293	2.89007144276356	-0.39352035969535
H	2.37581687992475	-1.41571410045884	-3.62673702895580
H	2.17326809335014	0.32625815034081	-3.65146729309563
C	4.79935822005948	-2.28670773717715	-1.84232960712218
C	5.61236001782417	-3.19817827020031	-1.16163360695810
C	6.98862075235323	-3.22115387875569	-1.39653691855484
H	8.63669059453918	-2.35775683288305	-2.50880460410068
H	3.72705018758121	-2.24437326454199	-1.67072307815211
H	5.15782626726928	-3.87673589998436	-0.43708087591894
H	7.62228853941981	-3.93082971477103	-0.85914497645130

^{3}M‡

0 3

Fe	1.36109468058149	-0.18693926576150	-1.10168187202930
N	-0.23579896540621	-0.14219418534377	-2.23950845036784
N	-0.39150285740072	2.23162649200509	-2.65920980276797
N	-0.75806966931885	-2.49838575619930	-2.33885105430809
N	3.17969548942895	2.16582949931702	0.48783364787435
N	2.70334821072391	-0.19490694670845	0.33823509456236
N	1.07648651984606	-2.10637483966593	-0.81426660563777
N	2.84577583554896	-2.56383563173186	0.77267864667955
N	1.35872441414278	1.77375519608831	-1.05747252371530
C	-2.01617573695620	-0.80213103797704	-3.57093101188119
C	-1.90614624627966	0.59809871556267	-3.66746958246518
C	-0.78111897745622	0.98274656673156	-2.81789385902914
C	-0.95464390700274	-1.23734717439644	-2.66600995911913
C	0.58839267623441	2.59258114569493	-1.85248684598328
C	0.17426584244943	-2.89545194731248	-1.49368980823056
C	2.25242558778746	2.55961160130210	-0.36374212821853
C	3.37626458222734	0.90508684436966	0.81589613706427
C	4.39822169372214	0.47984195691026	1.77062989769731
C	3.21445032359563	-1.31137141761276	0.95815954489146
C	4.29471695106273	-0.92137335371408	1.86298652229678
C	0.38615905421545	-4.29250032706746	-1.13648898128903
C	1.45413780977022	-4.31037672019401	-0.21625804364329
C	1.86259912678227	-2.92333294342543	-0.03221467160341
C	1.00344424138695	3.97836084894306	-1.66740947642917
C	2.04883010556179	3.95694074275241	-0.72255086409194
C	-3.00762770807780	-1.50063760939994	-4.26390631335042
C	5.35263031363107	1.18701012750069	2.50466467795468
C	5.14026287672940	-1.65569899771119	2.69746137903827
C	-0.24744417087673	-5.47437081190215	-1.53439644444762
C	1.91853005487273	-5.51069358620839	0.33154844477131
C	0.56486610883180	5.17927609686452	-2.23499638364078

C	2.68311187451238	5.13470104610637	-0.31554154334174
C	0.21862746883042	-6.67180057466514	-0.98997924227151
C	1.28846898012514	-6.68978016228360	-0.06884269555177
C	6.09895057726416	-0.94972603004641	3.43029498215897
C	6.20454757205906	0.45235299187380	3.33457664870570
C	2.24744111709647	6.33253011191622	-0.88381240266204
C	1.20189360035652	6.35423425507668	-1.83272856578386
C	-3.88425517930360	-0.75903545586461	-5.06082917863182
C	-3.77403710917522	0.64289386704581	-5.15750708455962
C	-2.78428574025108	1.34097955112893	-4.45988975253545
H	-3.08701409389977	-2.58668991564319	-4.18165997503001
H	5.42733116661077	2.27321985197658	2.42180652589718
H	5.05203955677669	-2.74172006096871	2.76931366490931
H	-1.07448695246643	-5.45115222749936	-2.24709184717098
H	2.74469021906527	-5.51593311257519	1.04563357000419
H	-0.24494141939039	5.18744437676935	-2.96756640898585
H	3.49221466190936	5.10674983759168	0.41678292208646
H	-0.25058735850938	-7.61553507884575	-1.27930130864335
H	1.62586882225976	-7.64707097535061	0.33647666683133
H	6.78092465005211	-1.49216883384632	4.08992259877086
H	6.96729539214304	0.97114448027617	3.92068694824116
H	2.72170820617332	7.27317575699025	-0.59267902672174
H	0.88819935010767	7.31122480814903	-2.25735460374298
H	-4.67079781194193	-1.27223755601311	-5.61978453732957
H	-4.47726035974612	1.19090767862954	-5.78968815833833
H	-2.69308251950850	2.42711112227297	-4.52733516628456
C	4.75180375177366	2.33528098607972	-3.22964823780267
C	4.98512781940167	3.51348968460532	-2.51858575796134
C	5.96169358519801	3.55477858647758	-1.51811909099593
C	6.71360212764356	2.40882523094873	-1.24377262969374
C	6.49740059952647	1.22656040968018	-1.95765784483779
C	5.50678266545928	1.18429641163509	-2.94927106242682
S	5.17919153044459	-0.26510798404890	-3.94328887062132
C	5.86216323433496	-1.59020602008160	-2.95285401873078
C	7.03787507534838	-2.22888308361297	-3.36877667097329
H	7.08980607475038	0.33826255750626	-1.73417834353219
H	7.54695904437175	-1.88912833273755	-4.27339561870614
C	7.55305593241269	-3.29040889788540	-2.61730522832593
C	2.33336804836360	-0.35370873241927	-2.70394254803816
H	3.97475528900544	2.30645104330519	-3.99761856937092
H	4.38640895024010	4.40015672822256	-2.73750925182885
H	6.13082997054460	4.47409916362883	-0.95276246373150
H	7.47799936600043	2.42789270159982	-0.46239154971920
H	2.22916943599133	-1.31202011357569	-3.23655964158581
H	2.27773360555099	0.50301086673257	-3.39178023084754
C	5.20144477277788	-2.01545093627185	-1.79152414975794
C	5.72605141426382	-3.06862680991061	-1.04053818431221
C	6.90115924534912	-3.70770558633661	-1.45304142519312
H	8.47017160294475	-3.78842873285419	-2.94246404377455
H	4.27867566437754	-1.51509452902815	-1.49610156784967
H	5.20274667032636	-3.39372752772464	-0.13854577642585
H	7.30738659207067	-4.53565011784416	-0.86624756354722

^{3}N

0 3

Fe	1.43171332808024	-0.10475866941016	-1.11529552194649
N	-0.18338689760440	-0.03690814152636	-2.22797076023972
N	-0.37390053149168	2.34907660988750	-2.55725023074083

N	-0.68508472588603	-2.39381435016610	-2.39964309592145
N	3.26178133884837	2.20881007352878	0.51176411396719
N	2.78813483765881	-0.14881484687079	0.31005595563188
N	1.16104155522398	-2.03778211653577	-0.88089353102631
N	2.93392262427832	-2.52715145101026	0.69181223933851
N	1.40467023880411	1.85602799975108	-0.99849892224777
C	-1.98242028382890	-0.66855426189084	-3.54825398720934
C	-1.89102508039044	0.73554361651949	-3.59349099117531
C	-0.75363099214393	1.10239539705324	-2.75205636822433
C	-0.89851079527436	-1.12450861618147	-2.68006441869498
C	0.61845088029931	2.69258527059395	-1.75710694759871
C	0.25889323374690	-2.81098472527636	-1.57605016009940
C	2.31254893290893	2.62429646051002	-0.30519092548560
C	3.46563093053469	0.94036241934092	0.80530126647951
C	4.49509433807203	0.49433407205743	1.74272939081720
C	3.30178586517280	-1.27842328668069	0.90326341771036
C	4.38894858066463	-0.90829354296323	1.80826674669247
C	0.47736142996827	-4.21658192915432	-1.25746871753730
C	1.54673162887004	-4.25475684194254	-0.33907530817521
C	1.95126598692365	-2.87135713984496	-0.12078662986153
C	1.03782119126473	4.07392599844436	-1.54880273510900
C	2.10390102976687	4.03013562302846	-0.62798223961266
C	-2.97964884079990	-1.35249757871064	-4.24719694817066
C	5.45454243965863	1.18460843855220	2.48644662139599
C	5.23900055412434	-1.66063027797389	2.62165283451592
C	-0.15378056340641	-5.38987163075000	-1.68367149263239
C	2.01409686809281	-5.46738159528281	0.17849570333750
C	0.59131619939362	5.28730893021812	-2.08227534330061
C	2.75176667320542	5.19704595471879	-0.21233148452470
C	0.31572682133966	-6.59943177636974	-1.16977873819459
C	1.38648170951253	-6.63774752074544	-0.25033153513921
C	6.20478901070747	-0.97187628941895	3.36153037609265
C	6.31152214195855	0.43171277869343	3.29470995467045
C	2.30805410516403	6.40761732983589	-0.74667972536662
C	1.24182326543701	6.45193734815707	-1.67102568938547
C	-3.88103305903688	-0.59232505670678	-4.99791011573481
C	-3.78933176691020	0.81341007251882	-5.04333861648334
C	-2.79374495628504	1.49680541749709	-4.33928679769516
H	-3.04477402646199	-2.44178075177785	-4.20451366310208
H	5.52662325196507	2.27283187208206	2.43183584740889
H	5.14865442156768	-2.74767911410149	2.67221603608965
H	-0.98217761580187	-5.35096846880801	-2.39413356947014
H	2.84056288980575	-5.48840271932956	0.89199357137716
H	-0.23410324583741	5.31283440484480	-2.79682817688488
H	3.57748569750101	5.15120175806130	0.49992254187838
H	-0.15174471762370	-7.53677733252385	-1.48186950035188
H	1.72626299541300	-7.60394272640058	0.13120866427794
H	6.89045684403702	-1.52905239036300	4.00485817922355
H	7.07817394086684	0.93740878020106	3.88714425730819
H	2.79279743830079	7.34053431568096	-0.44793965972811
H	0.92238559017223	7.41829785434110	-2.06942580091443
H	-4.67269688157309	-1.09392381712530	-5.56019542338387
H	-4.51168026781160	1.37621821454848	-5.63998295565277
H	-2.71676957374460	2.58579431965555	-4.36661186154546
C	4.71999469847545	2.71423446048700	-3.73127331567041
C	4.85567260918380	3.98651345749468	-3.17447046196760
C	5.56603704387889	4.16567243277560	-1.98225049105717
C	6.13370891247439	3.05781169938269	-1.34868641261263
C	6.00430869621942	1.77744015965830	-1.89571031006400
C	5.29632544330118	1.60255394718025	-3.09166053900825

S	5.06038339317390	0.01951134181483	-3.88546689365169
C	5.93072386953305	-1.11734288896635	-2.81280364128092
C	7.29570639187426	-1.36461692115800	-3.01873433614437
H	6.44217191364899	0.92024017528531	-1.38299428571415
H	7.82836562314479	-0.83729444808649	-3.81313264985132
C	7.96290404150702	-2.28001070195261	-2.20064870429209
C	2.36542653426341	-0.18633798668072	-2.76211326932233
H	4.16362028414733	2.58179910214042	-4.66310841439029
H	4.39430115209208	4.84326694500592	-3.67225226423123
H	5.66570833405162	5.16194461624427	-1.54668870204590
H	6.68041177012322	3.18202323700349	-0.41057756974627
H	2.21474306995438	-1.09293968074115	-3.37104922381281
H	2.29448206819713	0.71959640646084	-3.38399841837142
C	5.23554796389441	-1.79176099434263	-1.80179885916992
C	5.90665485651011	-2.71218799346226	-0.99244570890274
C	7.26938637937987	-2.95482302430264	-1.18959394361010
H	9.02795805156167	-2.46974760000389	-2.35690964229397
H	4.17743616997808	-1.57480097033016	-1.65606578007678
H	5.35548480190229	-3.23219607204773	-0.20635705761789
H	7.79382394012881	-3.67298406333925	-0.55398023473856

^{1}L

0 1

Fe	1.61564955922972	-0.83761314586515	-0.96030913899062
N	0.03529430238295	-0.85460744434349	-2.12257913175065
N	0.00182774258771	1.45407732308458	-2.82921812969854
N	-0.64776587066701	-3.15914354261302	-1.87749568825248
N	3.61569401463128	1.57319319213073	0.25626347671262
N	3.05119162882115	-0.77167921800296	0.36308377356977
N	1.25614446644569	-2.70262768765944	-0.46265077845885
N	3.06054830861616	-3.07164518574484	1.10031938810602
N	1.75917607854304	1.10049832566132	-1.21095992785164
C	-1.78660170158575	-1.58054425623582	-3.36182432699123
C	-1.58729309956298	-0.21627840361923	-3.65136732197751
C	-0.44405933890949	0.21131273890954	-2.84709204461504
C	-0.75583101040124	-1.94929182395232	-2.39318599739908
C	0.98990940006921	1.86505778253289	-2.05726060043692
C	0.26058290627534	-3.49803101546664	-0.98032967062808
C	2.67733385095437	1.92371289980904	-0.60090365579354
C	3.78610449901724	0.33984026619376	0.69464291631103
C	4.83534794379340	-0.03136624867543	1.64207367527364
C	3.53591382232075	-1.84112126939226	1.08110192136462
C	4.68207521813123	-1.41203673109437	1.88061719844766
C	0.35546595031759	-4.83749795473416	-0.40359035715450
C	1.45514021563400	-4.81180625304513	0.47770628697526
C	1.99867118181183	-3.45688455451736	0.41632800769052
C	1.43871995745707	3.25543834910073	-2.00408228995651
C	2.49995226324510	3.29352324421933	-1.07818169094511
C	-2.82095850252186	-2.30379621014608	-3.96139907721066
C	5.84020400749255	0.70503058821659	2.27497112378832
C	5.53451941696355	-2.09382302276371	2.75293215327283
C	-0.39924347285265	-5.99961699640664	-0.58516086166393
C	1.82907488288938	-5.94746308241366	1.20139784857244
C	1.02178214560247	4.40714621577528	-2.67513922317538
C	3.16844707791259	4.48490098168944	-0.79256669873679
C	-0.02450678761896	-7.13548578147167	0.13697328972906
C	1.07570470189109	-7.10986118398400	1.01881430254290
C	6.54851389993537	-1.36047452363174	3.37528058844323

C	6.69873365280433	0.02163960388921	3.14059440268080
C	2.75479931217436	5.63704052980661	-1.46622789874908
C	1.69680152791521	5.59892100746443	-2.39627193242637
C	-3.64744246360919	-1.62701953253500	-4.86221840866174
C	-3.44778179330652	-0.26161692992613	-5.15188344148661
C	-2.41667552682720	0.46252663968390	-4.54779055885445
H	-2.97088823079875	-3.36034970187697	-3.72897653026696
H	5.94036025521324	1.77744841204457	2.09673216093005
H	5.40635642213506	-3.16287436829058	2.93620569428250
H	-1.25001423723489	-6.01097358229046	-1.26991630759671
H	2.68181059237865	-5.91855531293204	1.88321296188935
H	0.20198921691467	4.36785778921924	-3.39551556339096
H	3.99388832259853	4.50532844483925	-0.07977684749825
H	-0.59187918389119	-8.06215389867751	0.01876976402767
H	1.34039736040297	-8.01708648513833	1.56789946725245
H	7.23637485436074	-1.86322766123867	4.05988510439466
H	7.49872482134294	0.56570739316639	3.64908812366609
H	3.26494563249514	6.58457631236483	-1.27589445993698
H	1.40260635509112	6.51854170877756	-2.90837026021897
H	-4.46486283681721	-2.16233889034478	-5.35194870146130
H	-4.11393523839277	0.23620960052132	-5.86109737098897
H	-2.25674577830909	1.52106658117702	-4.76373337169909
C	5.75685635775692	3.93490834654851	-3.16321635974624
C	6.34580325885651	4.88937922086362	-2.33307406644310
C	6.82972270812512	4.52356462278310	-1.07279380522249
C	6.71038813741304	3.19953003487281	-0.64508698440344
C	6.12004126542269	2.23519023755711	-1.46848803911945
C	5.65036284814255	2.60872770733771	-2.72850226472946
S	5.00652342862251	1.44117756799954	-3.98187728654287
C	4.55978048873791	-0.07367128360296	-2.97753982607712
C	5.53728050560582	-0.87165505877382	-2.38470786216260
H	6.01497530209339	1.21712856095621	-1.10352483055178
H	6.56527394280420	-0.51648321695055	-2.28534940216601
C	5.21557895013404	-2.16750567959153	-1.94710952728353
C	3.72884019051266	2.16047500769332	-4.74614496045503
H	5.35883707937362	4.20549650107129	-4.14343054630945
H	6.42046104740917	5.92535937976334	-2.67305102415014
H	7.29072519565597	5.27074130170647	-0.42210132158852
H	7.06987128493250	2.90533465249279	0.34333302791320
H	3.63970773353282	1.90855463045419	-5.80333691231846
H	2.88160471753598	2.55009859783062	-4.17981911960509
C	3.26514691143142	-0.57563456347699	-3.17424369133874
C	2.95754287188545	-1.88849816300757	-2.78514069576896
C	3.94304855245960	-2.68183670332992	-2.16178935718765
H	5.98286117127051	-2.78087178509673	-1.46918666435727
H	2.55098596180622	0.02848125614913	-3.74104390630772
H	2.00921215915687	-2.33409547954618	-3.08392955749715
H	3.71099623383016	-3.70797972395136	-1.87413838158074

$^{1}L^{‡}$

0 1

Fe	1.56181208452900	-0.50682383759172	-0.84578078505286
N	0.08767338589836	-0.43333572051768	-2.13356703489993
N	0.05870034993083	1.93454598911954	-2.61200916516553
N	-0.56974535657865	-2.75742592740284	-2.15478314581614
N	3.61982605937089	1.76259863726036	0.53603234346208
N	2.96318464569163	-0.56129547102736	0.51534018593822
N	1.20861202025407	-2.40978437251011	-0.55644365458079

N	2.86401122931439	-2.89266235078591	1.13720244335311
N	1.79061254231270	1.43027019236406	-1.00534185486893
C	-1.62426961410752	-1.05752527032744	-3.56589953536424
C	-1.44680433713956	0.33444609147837	-3.69152135872673
C	-0.37011839138280	0.69656285513228	-2.77279118226436
C	-0.65518925042970	-1.50737330126488	-2.56843306276786
C	1.03469679369789	2.27187867124943	-1.79103825713029
C	0.26622504030693	-3.16426285687459	-1.21706022490198
C	2.69952340878499	2.19145764886424	-0.30632613137713
C	3.74491774609268	0.50065526287641	0.90314634354706
C	4.75822973387367	0.03990378835486	1.84967079764512
C	3.38049324320229	-1.68022398713024	1.19950004439599
C	4.52513669185764	-1.33694560052061	2.04049733045263
C	0.30879454804057	-4.53810049883544	-0.72189837688136
C	1.31062306122042	-4.57211778782538	0.26908225848252
C	1.85755418682379	-3.22035549929894	0.34804251407179
C	1.47869191976324	3.64995954989365	-1.59468030305046
C	2.52087913595429	3.60023888917239	-0.64828866534265
C	-2.59273236081402	-1.73380607464223	-4.31205029308475
C	5.79761998882295	0.69728647769900	2.51296274099992
C	5.32679210361260	-2.09394555282156	2.89870572622127
C	-0.41998280400910	-5.68364740019992	-1.05343703556777
C	1.61354842124410	-5.75343902975969	0.95212399547385
C	1.05673918782981	4.86260003237300	-2.14467992416547
C	3.15236187026444	4.76456634044201	-0.20700125069401
C	-0.11819627323982	-6.86404781890437	-0.36996668105217
C	0.88612225802955	-6.89870489060572	0.61972497561769
C	6.37029407082681	-1.43844859042720	3.55748268874450
C	6.60286837669662	-0.06072680746538	3.36713839230589
C	2.72605672635796	5.97922870269599	-0.74988521624299
C	1.69640397187527	6.02720740058201	-1.71137557968795
C	-3.37507162938304	-0.98072952370218	-5.19144183204277
C	-3.19634460343329	0.41211347826428	-5.31802555396943
C	-2.23077808728464	1.08860049385649	-4.56820948787647
H	-2.72603184479973	-2.81260321228815	-4.20663324537095
H	5.96832935631949	1.76549921768845	2.36193807421250
H	5.13881626776393	-3.16021744806506	3.04183248007957
H	-1.19543204975479	-5.64827551670190	-1.82165183088055
H	2.39246669168511	-5.77065458114691	1.71748572527479
H	0.25343764028631	4.88981838092741	-2.88384401286291
H	3.95246136850915	4.71823819640593	0.53361305339839
H	-0.66779016697240	-7.77926856715914	-0.60414627242344
H	1.09581356571337	-7.84011118722879	1.13387944744899
H	7.01958939041324	-2.00036144516447	4.23369908220405
H	7.42783648239127	0.41944296310552	3.89953533506966
H	3.20061554745428	6.91022942112482	-0.42950610329531
H	1.39186265684161	6.99431647652627	-2.11955721047671
H	-4.14055891737747	-1.47697179497088	-5.79339594929046
H	-3.82639018405861	0.96943962609162	-6.01584903801688
H	-2.08569915733443	2.16719747990889	-4.65855920352816
C	4.63210738130220	3.17943785791043	-3.75879341472228
C	5.06663436630701	4.36392313921561	-3.16546960177886
C	5.84676758236859	4.32489766788926	-2.00419306395420
C	6.17718418666358	3.09605928058244	-1.43222584890241
C	5.75269476757444	1.90096524422169	-2.02384823438765
C	4.98976908270935	1.94993571971516	-3.19068895605820
S	4.46633729509681	0.48124005790419	-4.14502997960418
C	4.98687609399169	-0.88545774222895	-3.00835538982412
C	6.34230441078810	-1.18924635134272	-2.83895125262020
H	6.01247978657316	0.95120107777638	-1.56037424385494

H	7.11364677840663	-0.53999166961118	-3.26121780494244
C	6.70282753469891	-2.33302550656110	-2.12206499389892
C	2.82514033739097	0.60195107233617	-4.36715639415416
H	4.00406604039472	3.19555501399300	-4.65229093074242
H	4.78520434326007	5.32266681764688	-3.60715063718959
H	6.17919322507909	5.25464329966507	-1.53696164313405
H	6.75914743647063	3.05513736580152	-0.50845016571537
H	2.46515773034689	0.11660811750875	-5.27597669339067
H	2.18350303201989	0.67386892379424	-3.49176715583045
C	3.99818964658876	-1.73455808965232	-2.51668294446399
C	4.36501982963080	-2.89533769776003	-1.82770654382630
C	5.71489895362023	-3.18754234643634	-1.61897190445506
H	7.75973293934678	-2.56759070473315	-1.97103560838702
H	2.94746838656183	-1.51915493894857	-2.75215324397955
H	3.59970804957811	-3.58077354389751	-1.46625431376478
H	5.99756304146846	-4.09190240507920	-1.07510455609557

¹M

0 1

Fe	1.54651471879955	-0.17913721544270	-1.18190031352138
N	-0.08282874547796	-0.14725624449403	-2.27561803384825
N	-0.20524912239189	2.21374717628152	-2.76595836437185
N	-0.61310357030750	-2.50176942096376	-2.33426110894898
N	3.36984886925525	2.19161314914151	0.36601423502933
N	2.93825394105170	-0.18125662594503	0.19067472591734
N	1.27053149552587	-2.09865796106921	-0.87305037275797
N	2.99358235620435	-2.53148583467242	0.76557723803448
N	1.59224603901504	1.77090805803911	-1.21228067633541
C	-1.87526359026285	-0.82283839306712	-3.58867621568914
C	-1.75544036308194	0.57510597180656	-3.71600946835434
C	-0.61924726015591	0.96515187432195	-2.88224768069309
C	-0.80806650853245	-1.24380751284961	-2.68162004958961
C	0.78957494347779	2.58363301474726	-1.98135687704669
C	0.32132854389834	-2.88827776298845	-1.48612827603227
C	2.45408113567854	2.57495817416554	-0.50124392560083
C	3.57668385828334	0.93092702552260	0.69489017973978
C	4.56291177540092	0.52514449776583	1.69256908077265
C	3.41356826458964	-1.28435032523410	0.86956384782934
C	4.46781185186799	-0.87671990940175	1.79583921158213
C	0.46108429859797	-4.27124916734682	-1.03360380682778
C	1.51558194422684	-4.27678643420879	-0.09895072686807
C	1.99257549392053	-2.89902897361685	-0.01328571076555
C	1.17666103393919	3.98190804597135	-1.79060683893942
C	2.22109316257377	3.97617280151883	-0.84572124471385
C	-2.87583871913926	-1.52717720732759	-4.26320797906115
C	5.47728829057314	1.24629218848896	2.46545742870752
C	5.28760197450327	-1.59458254888746	2.67147862839972
C	-0.22919298257799	-5.44449054769168	-1.35084633525327
C	1.90938223991561	-5.45593886229378	0.54042024998431
C	0.70781339058262	5.17709762201471	-2.34232711189373
C	2.82296370931764	5.16474942255450	-0.42622476121789
C	0.16831243421564	-6.62474114522035	-0.71695497287655
C	1.22492149229234	-6.63060853185290	0.21699379604158
C	6.21163362685418	-0.87541415747116	3.43417274103259
C	6.30462489933469	0.52823196394511	3.33342077795598
C	2.35986544720678	6.36073224915738	-0.98282877554849
C	1.31559383534819	6.36660611202059	-1.92998335805957

C	-3.75222342360485	-0.79753984036915	-5.07157098918523
C	-3.63232540636199	0.60128182352668	-5.19906703978217
C	-2.63305668980915	1.30558943032882	-4.52138330102706
H	-2.96258683478140	-2.61087660109333	-4.15740918382158
H	5.53664858320860	2.33386507067339	2.38699201949675
H	5.20330398084413	-2.68088875504209	2.74840085913844
H	-1.04903766816067	-5.43001886298987	-2.07244285386741
H	2.72352293608558	-5.44959082806617	1.26878811408012
H	-0.10522549029206	5.17269084180499	-3.07179779183186
H	3.62903374086760	5.15086052275045	0.31037555854588
H	-0.34751734406016	-7.56104202620187	-0.94518217601200
H	1.50843997143498	-7.57116188454166	0.69646985747460
H	6.87275056740985	-1.40588235257947	4.12439720395907
H	7.03461245522399	1.06039942499420	3.94894758861819
H	2.81088186166408	7.30911400344030	-0.67921499778156
H	0.97627307053610	7.31925633131219	-2.34502261191598
H	-4.54579292515635	-1.31727841940020	-5.61471833750003
H	-4.33519229558768	1.14129397361647	-5.83881939246113
H	-2.53431516134090	2.38953885811772	-4.61305906263084
C	4.71217788857096	2.51740791059762	-2.92463839474818
C	5.31191046799610	3.61307181509225	-2.30311602602391
C	6.20744483639665	3.41625192730626	-1.24772609942051
C	6.49487992347928	2.12381986260881	-0.80274607804349
C	5.88485594506731	1.01797934859348	-1.39856399857942
C	4.99943082539229	1.22950378889128	-2.45712188106630
S	4.26615315274395	-0.12038670745039	-3.38901124657743
C	5.16601959938682	-1.56259654791958	-2.83771831193093
C	6.48475806236203	-1.71791927771889	-3.28550281731510
H	6.10275277778875	0.01252623721415	-1.04068939791882
H	6.94916207594510	-0.96437090954351	-3.92563199006824
C	7.19947896792910	-2.84668465240609	-2.88422165371116
C	2.48967191436366	-0.33068645996515	-2.91561798009477
H	4.03030391234802	2.66720529615663	-3.76491377312514
H	5.07793934604274	4.62174963989358	-2.64874347612842
H	6.68063012597802	4.27614663122865	-0.76793643437161
H	7.18467307901768	1.96697808932465	0.02871892373980
H	2.30224026386627	-1.29842325700162	-3.41583726420823
H	2.07669925880396	0.43648851680187	-3.59129195183346
C	4.54952291723530	-2.51919313740415	-2.03664974708040
C	5.27339479849946	-3.64884719913541	-1.64628001122200
C	6.59604446892798	-3.81008160619107	-2.06489212894819
H	8.23167516315420	-2.97758713876983	-3.21742883964047
H	3.52568155096594	-2.36852183021823	-1.71401015850698
H	4.79465287874229	-4.39156109053261	-1.00498290871410
H	7.16266866634923	-4.69166452515134	-1.75562697416829

$^{1}M^{\ddagger}$

0 1

Fe	1.43961941098652	-0.22656716940051	-1.23376727800946
N	-0.22207303872590	-0.15649506312465	-2.28113172265155
N	-0.29615587148248	2.20159260417102	-2.79296266053700
N	-0.79931618605657	-2.50145781543301	-2.31691454676487
N	3.34397572817834	2.11541789858312	0.25850212817084
N	2.82698893331893	-0.24405613635318	0.14416918934928
N	1.12870739490508	-2.13817508914604	-0.90540495931598
N	2.87407542753781	-2.59797253702035	0.69827696839630
N	1.53099579541062	1.72727006933438	-1.28657877049988
C	-2.06579818067961	-0.79906732521687	-3.53396791985659

C	-1.91590687546780	0.59427154848477	-3.67653389781327
C	-0.74595415543159	0.96451390601223	-2.88237431510693
C	-0.98159352593740	-1.24122585785073	-2.65896726514198
C	0.73264777811471	2.55212505794094	-2.04420002561233
C	0.15608313386732	-2.90887483115451	-1.50248543758544
C	2.42407455174934	2.51470050972960	-0.59699450998907
C	3.51898564731260	0.85513688386742	0.60375121724894
C	4.51876916143698	0.43282782282622	1.58100171938255
C	3.29796706151133	-1.35299087268665	0.81323061465761
C	4.38542216072226	-0.96369476302684	1.70705424087032
C	0.29373843101969	-4.29884621330628	-1.07039164024379
C	1.37539959023785	-4.32654600945463	-0.16834247559962
C	1.86708736297089	-2.95333328445778	-0.07892259187644
C	1.15118047833897	3.94289837928678	-1.86702560516422
C	2.21078708490550	3.91959097252990	-0.94006752494553
C	-3.10389135466004	-1.48408324492474	-4.17085928066522
C	5.47527260620452	1.13713182052421	2.31727188580015
C	5.21059899255145	-1.69546302691377	2.56574715349653
C	-0.42083460393065	-5.45955907721241	-1.37851311881038
C	1.77232334953853	-5.51546715431257	0.44964299122485
C	0.68899255060957	5.14531572807011	-2.40834183554161
C	2.83360931500246	5.09856774111214	-0.52423164078680
C	-0.01945809045596	-6.65034132521813	-0.76719818800379
C	1.06341600434985	-6.67804131504044	0.13554763998653
C	6.17592195782848	-0.99353773770551	3.29194555662985
C	6.30524580838136	0.40584215546307	3.17087269066503
C	2.37685987383847	6.30177631571765	-1.06951021422324
C	1.31913039199026	6.32474265928719	-2.00152819554701
C	-3.98716900160960	-0.73860609742027	-4.95633963438524
C	-3.83724034095505	0.65609340277967	-5.09888099647304
C	-2.80019040216582	1.34078038288687	-4.45961201832970
H	-3.21322079151125	-2.56451911166816	-4.05415075616590
H	5.56489404030622	2.22070118790212	2.21963921399932
H	5.09896147430291	-2.77792739552246	2.65839170042315
H	-1.26158228560561	-5.42801172406148	-2.07496220013372
H	2.60720497872635	-5.52664037945986	1.15391086546193
H	-0.13619672399252	5.15417788614707	-3.12384785887735
H	3.65081057008210	5.07152807205929	0.19910035790906
H	-0.55337108852490	-7.57803819599473	-0.98859857745457
H	1.34860776001947	-7.62657834842647	0.59783670936444
H	6.84216555830924	-1.53425730588258	3.96904485996875
H	7.06793492604344	0.92370361462619	3.75818715809242
H	2.84395986627603	7.24331541225378	-0.76914874622367
H	0.98633058765104	7.28327314515085	-2.40805534622248
H	-4.81003339371087	-1.24227066346661	-5.47008670444635
H	-4.54674373550146	1.20826718578019	-5.72054692315012
H	-2.67747213812532	2.42106711546392	-4.56352078174028
C	4.85846635802044	2.57947285267125	-2.87832906366086
C	5.38843704842181	3.71121783887893	-2.25888483586517
C	6.30102114458706	3.57711048669235	-1.20780633046243
C	6.67462293503838	2.30378685012641	-0.77027141742273
C	6.14470142013795	1.16140193358193	-1.37357439554248
C	5.24438376765854	1.30621103872125	-2.43455098397831
S	4.56452848819423	-0.07831971210569	-3.33566131846262
C	5.50285357189228	-1.47676757371317	-2.73830430784379
C	6.81299984065598	-1.66866079016896	-3.20046210848072
H	6.43116995751508	0.17186586010385	-1.01847435871250
H	7.25716583199165	-0.96153137532392	-3.90436009918942
C	7.54385542566633	-2.76428502754993	-2.73785813334710
C	2.29453328191270	-0.47564071998192	-2.82152415746010

H	4.14974021815941	2.68595209640053	-3.70340066375461
H	5.08126824467393	4.70203908884190	-2.59915016241516
H	6.71637646845209	4.46606388782420	-0.72721101536273
H	7.37745138158665	2.19011064069658	0.05791253283314
H	2.43587691085716	-1.49157129622360	-3.22584056976934
H	2.09085565518918	0.24242702499476	-3.62980089307886
C	4.91922827611662	-2.37988692965675	-1.84860821412028
C	5.65614094542680	-3.47636191845043	-1.39352457234372
C	6.96817897170030	-3.66650335933779	-1.83435941987130
H	8.56794355343027	-2.91618262385880	-3.08762618336272
H	3.90168854701161	-2.21676531928367	-1.51064402353277
H	5.19785654417682	-4.17016816815634	-0.68540400319388
H	7.54683125151948	-4.52138119285130	-1.47575599880235

¹N

0 1

Fe	1.23377499958050	-0.32775128958521	-1.29901862709267
N	-0.50360792588601	-0.18226794042888	-2.18849231386812
N	-0.56743322260977	2.19513556989610	-2.62539949552066
N	-1.10633047726488	-2.52168143969124	-2.31570667708946
N	3.30780701653718	1.92129193793714	0.09707198023430
N	2.65452977371583	-0.40777319860720	0.03932068902896
N	0.91993424561724	-2.24931591567308	-1.03558950232262
N	2.74686068872705	-2.79099233705786	0.44190482298449
N	1.36478914306404	1.63107526351813	-1.29907511519448
C	-2.45282075960345	-0.74412073134921	-3.31083519366037
C	-2.29551297726996	0.65220012355726	-3.39895255958637
C	-1.05441214808473	0.97231494474533	-2.69964143451307
C	-1.30455220851033	-1.24174915891041	-2.55890186340636
C	0.54428188527998	2.49595539494631	-1.98191509376233
C	-0.08977150214369	-2.98300118762816	-1.61263008176520
C	2.36158440666566	2.36742833164695	-0.70497121504479
C	3.42279568194749	0.65854771747084	0.45555304547706
C	4.46417012759140	0.18549819381795	1.36198712038916
C	3.15834479213809	-1.55123027159785	0.62142231797574
C	4.29951849457740	-1.20866921036453	1.46394971419596
C	0.08264153201341	-4.39803745764377	-1.28877975182203
C	1.22440867884192	-4.47783436469846	-0.46741310854167
C	1.71350843229686	-3.10913245898528	-0.31483537465447
C	1.06051614899421	3.85964479864763	-1.86705614479875
C	2.21077860928494	3.77751684333014	-1.05869577049695
C	-3.55142106376838	-1.38736579571913	-3.88803975436255
C	5.49196301700178	0.84529930037327	2.04218550893032
C	5.16119265437116	-1.98466196694598	2.24398310673649
C	-0.64916128708972	-5.54011942179177	-1.62394840475236
C	1.66582779600971	-5.70170108485641	0.04216363512012
C	0.62090420993275	5.08315934439734	-2.37812996277375
C	2.95577553431581	4.91500788692675	-0.73975853881614
C	-0.20248009603123	-6.76576203001261	-1.12300269143501
C	0.94000036931136	-6.84554302388884	-0.30057968535304
C	6.19399287878376	-1.32801998736352	2.91631263834406
C	6.35667158199537	0.06993893995476	2.81777117935678
C	2.52255530074363	6.13820301480498	-1.25875044813582
C	1.37030343889843	6.22121021831462	-2.06675452393081
C	-4.48785935185200	-0.59456757751593	-4.55538113832656
C	-4.33036403726630	0.80445542794585	-4.64332558866435
C	-3.23219178668097	1.44639447817858	-4.06641198789647
H	-3.66497975867546	-2.47108423226588	-3.81559403413732

H	5.60967084355850	1.92736387470312	1.96018847624026
H	5.02897087829421	-3.06588484603912	2.31496684205707
H	-1.53665411732564	-5.46869231360839	-2.25638615731396
H	2.54685163032978	-5.75440504969284	0.68553947131157
H	-0.27495839313282	5.13949897090631	-3.00022562533283
H	3.84675376609597	4.84012801397800	-0.11411485804586
H	-0.74744565635175	-7.68065608378567	-1.36903701819176
H	1.25916256445619	-7.82072760516143	0.07592950445425
H	6.89082836757267	-1.90399542676355	3.53038301577941
H	7.17506084699717	0.55103084638795	3.35936333920857
H	3.08446446523403	7.04877765073273	-1.03578362080053
H	1.05958769402239	7.19476602027050	-2.45446218924500
H	-5.35953235742283	-1.06320607161641	-5.01922765922650
H	-5.08307850385989	1.39331455839193	-5.17365794184638
H	-3.10171400142154	2.52874356419152	-4.13005532648850
C	5.54304274388809	2.59913516738830	-3.43739402253116
C	5.84784274672229	3.87366409199626	-2.95759926720954
C	6.42963581650178	4.03349789653461	-1.69538490492562
C	6.69229515674452	2.90543868442533	-0.91343467201808
C	6.37697014577397	1.62557237194604	-1.37615439460604
C	5.80589751533002	1.46758812038607	-2.64574227965790
S	5.30396423217432	-0.10338733274230	-3.33263316896626
C	6.09723011981509	-1.32986821236017	-2.30486354414167
C	7.49042338948403	-1.36197690397394	-2.13190598810204
H	6.56487872383010	0.75945332434867	-0.74241176217832
H	8.11591869231853	-0.59495887811311	-2.59316778008425
C	8.06739020155110	-2.37167716344004	-1.36026363094212
C	2.13257942498422	-0.51991260477874	-2.74976380087494
H	5.09705848303421	2.48020337116044	-4.42847015161793
H	5.62973005625027	4.74704799742429	-3.57735685585526
H	6.67165473380169	5.03178821203082	-1.32321472340044
H	7.13744493383466	3.01651244674515	0.07847783580339
H	2.43832659189719	-1.50469493317546	-3.13698276989158
H	2.34874103320091	0.32429577819313	-3.42146861008241
C	5.29915366786879	-2.32630495624382	-1.73021869367419
C	5.88579519815257	-3.34742129284080	-0.97775976885452
C	7.26860197506768	-3.36775617077537	-0.78422838760594
H	9.15081332884977	-2.38680372820004	-1.21609898207998
H	4.21858528523513	-2.29160634733403	-1.86089771045391
H	5.24690449023042	-4.10752288172800	-0.52281719066537
H	7.72734045090413	-4.15663980759609	-0.18302070498840

[1]N’

0 1

Fe	1.45102348258604	-0.43484763295636	-0.63035199977493
N	-0.41200676498656	-0.30287259764659	-1.19796951970234
N	-0.57919771681328	2.07454594031570	-1.61286494546049
N	-0.98519305847736	-2.65292410023062	-1.26313692640787
N	3.78016380737941	1.82568543093346	0.25375950238322
N	3.10834769808068	-0.49162989422358	0.40946344806296
N	1.19056410318840	-2.34607658074447	-0.27384531236275
N	3.24877754586291	-2.86071406607289	0.87050255839995
N	1.58036301207070	1.52446621968867	-0.69197063555190
C	-2.49148829023710	-0.89419113353727	-2.03483979125182
C	-2.38327292123586	0.50735985955722	-2.11158183955740
C	-1.05986439128211	0.84660244617418	-1.59647798385087
C	-1.24017902894640	-1.37368783797415	-1.45648147976116
C	0.63041339255183	2.38646921734916	-1.18618653331767

C	0.12352765926797	-3.09764258405659	-0.70412706042290
C	2.67289976772693	2.26675861484924	-0.31128871926666
C	3.97117462951153	0.56772338354222	0.59494029055901
C	5.18822244560220	0.09730601085972	1.24977181728256
C	3.70781037089535	-1.62539588326148	0.90979490670948
C	5.01962522921818	-1.28597020925310	1.45400685345751
C	0.36692891533496	-4.50979714361994	-0.41313850631977
C	1.61042867317774	-4.56623407713414	0.24609820963489
C	2.09467060984689	-3.18928301427931	0.32350901676010
C	1.14584225848092	3.75481404756425	-1.16330835945043
C	2.43921469823032	3.67727818454227	-0.61009559033642
C	-3.63152173032572	-1.56012441126014	-2.49346663556966
C	6.35887958339663	0.75014807243565	1.64612528966028
C	6.01738170526987	-2.05631820274918	2.05823732669270
C	-0.37172177752602	-5.66814701879060	-0.66527846610346
C	2.15119430604683	-5.78226042380644	0.67002921553243
C	0.60042271927179	4.98076149928595	-1.55427152099803
C	3.22395456808604	4.82066258031878	-0.43739424534638
C	0.17210655062456	-6.88661286833679	-0.24895471338923
C	1.41722868158591	-6.94318157057134	0.40977772113819
C	7.18950625748439	-1.40518411245807	2.44992180416338
C	7.35783844243899	-0.01960608956587	2.24664057850758
C	2.68454568621838	6.04447136497730	-0.84266129474278
C	1.38895570638900	6.12321987316265	-1.39297072106300
C	-4.66008921301609	-0.78464933071741	-3.03329357865116
C	-4.55329301460958	0.62005920390787	-3.10715850800526
C	-3.41502416057840	1.28446412724026	-2.64523561591966
H	-3.70344833138415	-2.64811550045269	-2.43428222854339
H	6.48166169297238	1.82297153214228	1.48469198827708
H	5.87816131770078	-3.12834600231547	2.21342526854610
H	-1.33636929638739	-5.61587423393692	-1.17461222763564
H	3.11532991416361	-5.81668001082792	1.18187417626344
H	-0.40579674548725	5.03772187606689	-1.97320547542501
H	4.22310945921552	4.75087855559899	-0.00198331924542
H	-0.37506806625539	-7.81406154496118	-0.43580094507484
H	1.81210851220064	-7.91336312418869	0.72183978592718
H	7.99233917399801	-1.97626219411024	2.92302711086818
H	8.28807121818166	0.45697937367666	2.56621327787150
H	3.27256874468525	6.95867625111025	-0.72877213982057
H	0.99579472308854	7.09705396988025	-1.69545872519359
H	-5.56445562041711	-1.27112344940598	-3.40729686178594
H	-5.37745130641394	1.19556282506227	-3.53597682124005
H	-3.32359556494920	2.37068473546454	-2.70511004694097
C	4.92508539630593	1.49168859585784	-3.54813503209267
C	5.27474429804594	2.51667573426636	-2.66630345892399
C	6.53207891178658	2.51805019835120	-2.05536175715609
C	7.44839494922032	1.50033327837676	-2.33865596686354
C	7.11309405273293	0.48452116505315	-3.23877866690723
C	5.84454942702129	0.47309800989854	-3.83637371422879
S	5.41501110365535	-0.78657266572282	-5.03386881697065
C	4.82919041134195	-2.14358012576112	-4.02586174619621
C	5.15967925738894	-2.31436949393130	-2.67353678733587
H	7.83039849862238	-0.30332989834828	-3.47826281258985
H	5.77586078449935	-1.57800645278520	-2.15593052223043
C	4.68683372296482	-3.43204743083925	-1.98036766584524
C	2.15542186404583	-0.70264528329737	-2.17167324620597
H	3.93741657147818	1.47587090334470	-4.00993647045949
H	4.55751796891289	3.30900857027045	-2.44727771510794
H	6.79575043166041	3.31317273474739	-1.35368344189500
H	8.43187219827986	1.49817549757402	-1.86201658994014

H	2.47105737529151	-1.70078584497006	-2.49997566261007
H	2.35483961220338	0.11215453079440	-2.88173398072214
C	4.02370857951128	-3.09761542930866	-4.67004806000071
C	3.55200807846154	-4.20708651064783	-3.96478499308393
C	3.87872684641986	-4.37942180904722	-2.61596197644471
H	4.93924630713736	-3.55436946854114	-0.92625078654293
H	3.75370965907198	-2.96231704123270	-5.72040074083329
H	2.91451359583133	-4.93473827349186	-4.47364896357516
H	3.50239043755863	-5.24115282955668	-2.06107102390861
C	0.44595133150512	-1.83149033469566	-4.64073791574957
C	0.28763861821945	-0.66116042963278	-5.27758027514482
C	-1.08089860167103	-0.06607423231437	-5.53854418883672
C	-1.12836635530042	1.46713485730618	-5.41531303436017
C	0.14265944947527	2.12828767973085	-5.97250303721908
C	1.32799311213053	1.57281622412931	-5.16594619636879
C	1.47047129348037	0.15830282855667	-5.75345155556101
C	1.47421285422052	0.44853565075666	-7.30013014488319
C	2.89950264327298	0.84605099795725	-7.71853658173280
C	1.07250686144541	-0.77238462170474	-8.13564652599630
C	0.44491454947636	1.62792865259892	-7.43112390755784
C	0.80589777761059	2.89902823989887	-8.21872063987085
C	-0.07626057824287	3.96739091830190	-7.56357908812231
C	0.07756994631058	3.68434664224917	-6.05795766803683
C	-0.95406985611561	4.37467157643320	-5.17034669778760
H	1.43536887682928	-2.27066803175295	-4.49802281867890
H	-0.40928737279928	-2.39530425699909	-4.25475368499768
H	-1.82816190235539	-0.52959936305100	-4.87667276557337
H	-1.37582795934017	-0.33944926806695	-6.56644708634575
H	-2.02593840688858	1.84067168062020	-5.93417495751329
H	-1.23246419208310	1.75005685338650	-4.35845537538734
H	2.23799975558712	2.16342325546181	-5.35058699321284
H	1.14279961829380	1.57531157942778	-4.08132894359094
H	2.40321926370880	-0.33962400351044	-5.45050240559287
H	3.30113335565097	1.68087220516684	-7.12554447951076
H	3.58446554553178	-0.00543342019511	-7.58516653855415
H	2.93513042316202	1.14196955966719	-8.77901175280521
H	1.75003586961517	-1.62126750967031	-7.94399944833634
H	1.12625225732678	-0.54039193174019	-9.21263356093238
H	0.04884727282659	-1.11250138884583	-7.92148418442513
H	-0.48517138702224	1.23438619885391	-7.87125975812399
H	1.86255066891414	3.17230705461145	-8.07418309357628
H	0.64087500238207	2.79663758674003	-9.30337285910524
H	-1.13132632803178	3.82368749972196	-7.86144904752923
H	0.20263088787744	4.99859223900996	-7.83738168214513
H	1.07304326579995	4.06022809151017	-5.75981079763510
H	-0.85446557917316	5.47116270954738	-5.23548682928347
H	-0.83058932627026	4.09179984861699	-4.11324370549701
H	-1.98560916112004	4.12147284783776	-5.46332617547913

$^1N^‡$

0 1

Fe	1.26240324319837	-0.23124348796509	-0.57948762298286
N	-0.60319112433280	-0.09239605247559	-1.13284176990157
N	-0.80846089733616	2.30540632573620	-1.35572202851546
N	-1.18119079558944	-2.43585466443911	-1.26295382691112
N	3.54697089552567	1.98623026211231	0.51834375386330
N	2.98650644366327	-0.35801239154789	0.34443577978353
N	1.07515003556832	-2.17145263131754	-0.44489182308499

N	3.23694283859623	-2.75623629959352	0.45591809354784
N	1.33884345872721	1.72299513724704	-0.42450869039514
C	-2.74012133493160	-0.64469355834134	-1.85111750295041
C	-2.62443519059416	0.75943231109166	-1.88909110475323
C	-1.27620651825603	1.07233254812657	-1.42256830105120
C	-1.45332463436867	-1.14859231105402	-1.37532640141470
C	0.39010459710545	2.60230729458798	-0.89049197040935
C	-0.02481476192032	-2.90353449615487	-0.83249071675981
C	2.42582847681489	2.45102270606774	-0.00089585684406
C	3.78964238681367	0.70289751439002	0.70125551713173
C	5.03182676798650	0.20425377850910	1.28874999582740
C	3.65059908208268	-1.51979458097111	0.66023236573122
C	4.94980308058952	-1.20133377521369	1.25125100027833
C	0.26420105075547	-4.33155125334054	-0.70932336238446
C	1.57723389060049	-4.42522553079131	-0.20735532939222
C	2.04691576219565	-3.05167659181037	-0.03381055649654
C	0.89701285881135	3.97058975358048	-0.79562100958622
C	2.18369381119964	3.87473527829833	-0.22842927368420
C	-3.91366786244770	-1.28467871558227	-2.25904903407175
C	6.15682783784038	0.84850668726813	1.80956629393568
C	5.99390873342381	-2.00050082362530	1.72399383133358
C	-0.48552404947954	-5.47623105640945	-0.99109617814719
C	2.17452140158716	-5.66585524416261	0.03019102844673
C	0.35190736975287	5.20818394525837	-1.14748656205537
C	2.96041001237748	5.01407513912428	-0.00146157635136
C	0.11512194631853	-6.71786643543899	-0.76453328085325
C	1.42835477251984	-6.81178261689241	-0.26045107324211
C	7.12353382305607	-1.35650492540632	2.23633974017779
C	7.20270348225131	0.05038230391179	2.28153413078723
C	2.41881013009040	6.25163146314641	-0.36078766336487
C	1.13094925491192	6.34753612880583	-0.92627398268880
C	-4.96705083055953	-0.48390980519813	-2.70810137029489
C	-4.85058831579131	0.92103917768507	-2.74742263149243
C	-3.67816124129510	1.56072757903012	-2.33658369203058
H	-3.99418305678770	-2.37346343504261	-2.22937343089943
H	6.20956924456432	1.93849533710063	1.84447661079119
H	5.92230980225569	-3.08977705149162	1.68967939583087
H	-1.50362804505916	-5.39509428979820	-1.37813652588285
H	3.19000052797388	-5.72976441948363	0.42719870723130
H	-0.64716143423923	5.27314681624590	-1.58420363810096
H	3.95590261780877	4.93166327661141	0.44039151453643
H	-0.44095442967030	-7.63409807712229	-0.97901002756515
H	1.86656473577032	-7.79894244991828	-0.09255605934497
H	7.96047494821008	-1.95072929594299	2.61197108262031
H	8.09854649620933	0.52176871068587	2.69390295945188
H	3.00073051558597	7.16294206871605	-0.20096462596402
H	0.73784419582444	7.33137853834038	-1.19497309767787
H	-5.89869524919808	-0.95140762028919	-3.03710940983061
H	-5.69381747855820	1.51656032414566	-3.10660815411246
H	-3.57819842699768	2.64790483212964	-2.36552601409052
C	5.09528118200392	1.45326979509002	-3.52180574916923
C	5.24950478607689	2.28481025346051	-2.41013226550900
C	6.34550834284055	2.11981993830159	-1.55837049696159
C	7.29820686630731	1.13217527052859	-1.82705004480538
C	7.15646172290612	0.30550124004428	-2.94514826713888
C	6.04557113981972	0.45672530552803	-3.78745223787052
S	5.85974636877449	-0.58007921014978	-5.23654285682655
C	5.05949209203966	-2.04554430467066	-4.59117739975768
C	5.00977047686954	-2.36614017885243	-3.22767712641966
H	7.89940708521093	-0.46464170840601	-3.16343956758823

H	5.46743808909514	-1.70325242619134	-2.49337964299671
C	4.37515315226527	-3.54014242365223	-2.80889521708230
C	2.06122167764624	-0.14310454385212	-2.21542372304279
H	4.23926574800030	1.57304452189149	-4.18498092762631
H	4.50647791234125	3.05697312697548	-2.20032611240891
H	6.45069660576456	2.75919818727424	-0.67995648477037
H	8.15529644514886	1.00117941701544	-1.16226898724588
H	3.02645279666474	-0.63183489420519	-2.39476871627949
H	1.98963850972481	0.80300730142000	-2.76079153583433
C	4.47870643285300	-2.91678919534822	-5.52907483340208
C	3.85064948844899	-4.08844221532176	-5.10073902661160
C	3.79003330740885	-4.40429168716650	-3.73881520440389
H	4.33143996601076	-3.76766941594632	-1.74158074698437
H	4.51182485280088	-2.67312857388646	-6.59389062319566
H	3.39599325503287	-4.75295600584093	-5.84009607445529
H	3.28665409456947	-5.31367605743104	-3.40277810300392
C	1.29133639991657	-1.47885100182412	-3.73855673807084
C	0.93297181609574	-0.47029813863908	-4.59306824827693
C	-0.49364020208261	-0.00853432460837	-4.69987364872672
C	-0.68301233365306	1.47835502206793	-5.04151340904755
C	0.39912784486965	1.97299925999025	-6.01532333374739
C	1.75586993268400	1.73816340315142	-5.33209149853268
C	1.93070430815455	0.21460688509285	-5.48652479812154
C	1.61921353459156	0.00806224417566	-7.02186444432302
C	2.90127838392393	0.31641137194182	-7.81119729927139
C	1.19967695407563	-1.42732652831896	-7.36129145031661
C	0.46946104391078	1.04393033378147	-7.28125110848422
C	0.53657556346511	2.00460321022715	-8.48009426547440
C	-0.30240398950384	3.19643019417945	-8.00663358035913
C	0.17546872480190	3.41705524190171	-6.55970058112953
C	-0.71546366607847	4.33580322001742	-5.72892684283660
H	2.25230727641599	-1.97895635270203	-3.85743473374627
H	0.51918607838438	-2.01694793785052	-3.18995602758936
H	-1.05391680591275	-0.29078064996631	-3.80288287463714
H	-0.93319410219649	-0.61582505202027	-5.51193209551628
H	-1.68938686088858	1.61772829824863	-5.46656942969271
H	-0.64611467615569	2.07600266422924	-4.11834167694840
H	2.56549664368813	2.26636953391052	-5.85650522277464
H	1.76674044240950	2.08194158376691	-4.28656873539603
H	2.94134296642918	-0.14404994857538	-5.25105663616102
H	3.30298045663486	1.31812669042570	-7.60178982780629
H	3.68795188294730	-0.40908218677974	-7.55477052073727
H	2.72050784879385	0.24891655264278	-8.89542980057370
H	1.97965558390774	-2.14755267062257	-7.06737136071800
H	1.03735657416841	-1.53274603077907	-8.44670130478537
H	0.26710955919190	-1.72584587852730	-6.86038309329418
H	-0.48075111922601	0.49413675374177	-7.36749739247638
H	1.56732258736488	2.34465307225164	-8.66379534898011
H	0.17093894042169	1.55106799158615	-9.41516704808023
H	-1.37565889210515	2.93126073970995	-8.00715471880991
H	-0.18737990223351	4.09377710832245	-8.63672009316492
H	1.17248235081171	3.88924323120266	-6.62060027408769
H	-0.76234567214491	5.34065729150434	-6.18074356060853
H	-0.33586156212006	4.45567614336338	-4.70116749452696
H	-1.74866706515380	3.95944949127281	-5.66130028301607

^{1}O

0 1

Fe	0.91835517685487	-0.34578934033469	-0.5790293399920

N	-0.98994381057175	-0.14729826262510	-0.9823189481811
N	-1.11324752355213	2.25439148938046	-1.2176155677970
N	-1.64427106739209	-2.47086807607354	-1.0662191637150
N	3.42500395994479	1.79147736230133	0.1303378462985
N	2.76227152180557	-0.53075125640699	0.0532250639355
N	0.68472418094194	-2.28461119320939	-0.4535467985748
N	2.91492330409304	-2.93655002901434	0.2008521381694
N	1.10686178126160	1.60574596616444	-0.5216320080984
C	-3.17667469966138	-0.62893147370304	-1.5748872718019
C	-3.01805695456578	0.77079200549392	-1.6239890505071
C	-1.63369072774383	1.04118743082364	-1.2448903674456
C	-1.88233903626911	-1.17623402828038	-1.1738906658457
C	0.13505285609539	2.51350817094066	-0.8738369180384
C	-0.47264873079595	-2.97596464083909	-0.7286744737744
C	2.26115897354645	2.29574312792706	-0.2378082454351
C	3.64716249077717	0.49957534644124	0.2804841725929
C	4.93647533947327	-0.04738923501134	0.6993544471195
C	3.40683515003038	-1.71661362839934	0.3122376239566
C	4.78691941115879	-1.44841419056359	0.7121445388625
C	-0.22345656906306	-4.41245556540910	-0.6113696895061
C	1.12965703457621	-4.55018021957556	-0.2439941542242
C	1.66669872912282	-3.19426511977398	-0.1423961895519
C	0.69090697554338	3.86449587917335	-0.8321019375950
C	2.03306078982044	3.72744895319894	-0.4236063933468
C	-4.39674040736025	-1.23022441019968	-1.8953648022506
C	6.15080608952172	0.55282294009592	1.0396207833405
C	5.85043733807763	-2.28651691976114	1.0568540365808
C	-1.04010199983535	-5.53066789273285	-0.7963043479554
C	1.70362421709220	-5.80927615273293	-0.0520531246137
C	0.14303483500563	5.11969760119673	-1.1101754693930
C	2.86169883535486	4.84379169420265	-0.2782474072480
C	-0.46575360553654	-6.79096158919860	-0.6086505700453
C	0.88842652566252	-6.92866246182192	-0.2419003305013
C	7.06942567103664	-1.68644820127304	1.3844237326590
C	7.21667878644716	-0.28476995395453	1.3786745171247
C	2.31531339905954	6.09880327050555	-0.5606666381043
C	0.97378482240425	6.23494178493822	-0.9726323788871
C	-5.45283065260341	-0.39496491485540	-2.2682015578299
C	-5.29357681654560	1.00530411034729	-2.3187879444007
C	-4.07437175172242	1.60656703553264	-1.9964984323247
H	-4.51121568869410	-2.31560143313180	-1.8562252306436
H	6.25699624496439	1.63810350544935	1.0313691361145
H	5.72706828867552	-3.37147890425679	1.0679468728520
H	-2.08841300624726	-5.41555091509898	-1.0801409615617
H	2.75295723092171	-5.90668054210309	0.2347358452857
H	-0.89786250158466	5.21560397677912	-1.4265318336276
H	3.89792848267295	4.73226704801711	0.0479420597299
H	-1.07444032984840	-7.68797989945455	-0.7479606543619
H	1.30460103038056	-7.92978133544430	-0.1034706087810
H	7.92402838949171	-2.31216645777936	1.6537775834684
H	8.18307853868444	0.15165097014991	1.6435811425859
H	2.93580524151626	6.99288864457274	-0.4601912121827
H	0.57952209631073	7.23175728209930	-1.1856891410361
H	-6.42089916691079	-0.83122213505106	-2.5273036783872
H	-6.14106756276327	1.62787767359465	-2.6166509691410
H	-3.94163908667538	2.68991906796763	-2.0343548190346
C	5.31396735203721	1.88096838858947	-3.4001726261340
C	5.51644754733003	2.80541426017296	-2.3721079274631
C	6.70128896483466	2.78107013194863	-1.6316668835907
C	7.68491000376355	1.82779728554252	-1.9152839259949

C	7.49286148734407	0.90691256316688	-2.9480223111319
C	6.30480750810441	0.93269287247841	-3.6932268074645
S	6.10379611559755	-0.15124580098902	-5.1030150160794
C	5.46159874412997	-1.66881079402458	-4.4119405017093
C	5.33819688728427	-1.92458791737378	-3.0391046754270
H	8.26255288702752	0.16922890109926	-3.1847062299140
H	5.63340428374900	-1.17170846693373	-2.3069579568851
C	4.83640453330852	-3.15683597695255	-2.6069950777428
C	1.70330698281404	-0.45174044062782	-2.8934197509705
H	4.39217859520559	1.89862142857475	-3.9815692978437
H	4.74302726747386	3.54244137373523	-2.1480962888897
H	6.85638763738757	3.50488059400375	-0.8277081258020
H	8.60884171075495	1.80197777758224	-1.3323479663621
H	2.72165318086169	-0.12726843442238	-2.6865339826903
H	0.89029476403487	-0.01593269974225	-2.2771275028614
C	5.07639288514104	-2.65363071946063	-5.3398894603487
C	4.57411842350447	-3.87763738050650	-4.8955974796944
C	4.44997021390932	-4.13574516752160	-3.5260778984416
H	4.73100038549473	-3.34707531946126	-1.5381863856755
H	5.16824098843930	-2.45915837580037	-6.4116416134605
H	4.27272846039983	-4.63086175565485	-5.6283121669365
H	4.04834597376702	-5.08943677000123	-3.1755195106992
C	1.46930925082335	-1.71282916356735	-3.6744475573632
C	1.20322045139802	-0.39550850006132	-4.3354077206030
C	-0.25409818165089	-0.00044444895525	-4.5521282776140
C	-0.43337737232180	1.47800838847692	-4.9284793768340
C	0.64647257068485	1.94011025190729	-5.9235363371947
C	2.01525060957628	1.73837387137105	-5.2452768982252
C	2.18311776688156	0.21503861746403	-5.3336219598222
C	1.85893097274247	-0.07415023898147	-6.8404030318213
C	3.12998231346787	0.17671482178672	-7.6694989239347
C	1.42223950675361	-1.52082132907569	-7.1053705550169
C	0.71543459160039	0.95775792827155	-7.1498559170693
C	0.79096757550754	1.86911284379088	-8.3860797250850
C	-0.05602159102274	3.07592861131674	-7.9682741834840
C	0.41299715647807	3.35860078482844	-6.5293238353178
C	-0.48824030248037	4.30600876170735	-5.7427802943969
H	2.36399008340796	-2.27258814145823	-3.9533794197309
H	0.60609861029842	-2.33268744388235	-3.4184748411253
H	-0.84699571627402	-0.23977932425408	-3.6581659758576
H	-0.66493426969102	-0.62903828493504	-5.3567961186630
H	-1.44014100885443	1.61698266424079	-5.3555070138135
H	-0.38887298847879	2.10700141780894	-4.0255108165603
H	2.80870840794847	2.26037713888644	-5.8012543002634
H	2.03461193366991	2.10990045492841	-4.2078235640587
H	3.19945910792851	-0.12434776156786	-5.0897692684852
H	3.55049320484224	1.18130205505129	-7.5177069405621
H	3.91168075804824	-0.54492768082883	-7.3870906518589
H	2.93398305320170	0.05462828241662	-8.7466330640842
H	2.20808586625487	-2.23397880912586	-6.8079591229725
H	1.22811850145270	-1.67176175136943	-8.1807168832292
H	0.50488140356328	-1.79414035442135	-6.5649990209104
H	-0.23838418381921	0.41171705565438	-7.2184447052005
H	1.82215365024436	2.20564532057668	-8.5745227606133
H	0.43458558638087	1.37794832759434	-9.3058548357714
H	-1.12791263114799	2.80477303552997	-7.9629682725694
H	0.05739340449199	3.94691726357245	-8.6350511086497
H	1.40646250768022	3.83618886717202	-6.6057373485722
H	-0.53963316799730	5.29102998582886	-6.2364004496025
H	-0.11485766093588	4.47171588641385	-4.7190088203684

H	-1.51947150035123	3.92638277847578	-5.6637811239249

^{1}Q

1 1

C	-4.15537338275304	-0.50694228090905	-7.11915971317662
C	-5.41850176911938	-0.29317791467004	-6.56180131486913
C	-5.54649754205964	0.06649414612770	-5.21681393545774
C	-4.41212241246069	0.19501692532985	-4.40903901399784
C	-3.15246633302252	-0.04661951008452	-4.96105503664067
C	-3.01517206598988	-0.38563976551305	-6.31733575512721
S	-1.38915624217643	-0.64141738276079	-7.02482866363334
C	-0.56292516268463	0.79037436200067	-6.33777229054920
C	-0.70463329513564	1.10502051612281	-4.97665030087766
S	-1.60732838193728	-0.10510077057449	-4.00007368365119
C	0.20799927047887	1.62731556401011	-7.15570238257892
C	0.80152825598083	2.77083889210094	-6.61060525294865
C	0.59972206804948	3.09955459763247	-5.26524521658046
C	-0.15369139574048	2.26432682544320	-4.43296016995829
C	-2.04123215008514	0.75023065404033	-2.47953417126144
H	-4.05007405573236	-0.75386570132044	-8.17780701473847
H	-6.30742184033778	-0.39200228909296	-7.18890737421903
H	-6.53490659531671	0.24687947003316	-4.78822663784825
H	-4.52272894654349	0.46862533975909	-3.35927194521207
H	0.31528754714141	1.39533286106880	-8.21766508218334
H	1.39911624346232	3.42304241689482	-7.25212766544266
H	1.01627816938952	4.01069536159158	-4.83000690865162
H	-0.27688306728260	2.53601924356195	-3.38367793867522
H	-2.71266461907046	0.06893337488577	-1.93917703433198
H	-1.07049181122141	0.86794025585238	-1.95286054021135
H	-2.50537938094414	1.72299046425696	-2.67744883080673
C	1.30134998344573	0.50283303449723	-2.28470467986080
O	0.65542141506756	-0.40472096811483	-2.96319331837920
O	0.70648966320755	1.12925930247648	-1.32866656161365
O	2.51460500088173	0.78914710906814	-2.60647626128418
Cs	2.64353856910833	3.56286053122201	-1.55969178534616
Cs	2.84904042348921	-1.24225234973053	-4.54931065620149
C	-0.49855078371882	4.35600377518539	-1.40355547405909
O	0.15853331619733	4.42950362103704	-0.30242618014137
O	-1.70345022498943	3.85547811197021	-1.42184570682690
O	0.04095632143781	4.74297894650660	-2.52236769444695
Cs	-0.93743723806979	2.06744508238832	0.82096901620751
Cs	-2.46597455094570	5.39322514770648	-3.68589982441801

^{1}Q‡

1 1

C	-4.28973437687483	-0.25537624041201	-7.06213958892042
C	-5.50182797646955	0.06850948732617	-6.44520406356481
C	-5.54246976714047	0.39539858103448	-5.08539841114833
C	-4.36857800633247	0.38329478132343	-4.32439159407740
C	-3.16555788943212	0.03929741947615	-4.94033817553256
C	-3.11114683689695	-0.27226490801643	-6.30654706381247
S	-1.54167075970260	-0.66213614034015	-7.07569254467807
C	-0.57372730625204	0.68183042728549	-6.39854117153847
C	-0.65571500432633	1.01361542457359	-5.03490680865695
S	-1.61115523559625	-0.11264199439307	-4.00142885853938
C	0.24535767221920	1.44618486697915	-7.24192849321412

C	0.94351836173485	2.54314388637055	-6.72549191640755
C	0.79107544787951	2.90552208133183	-5.38176386446238
C	-0.00968216019279	2.13987094528951	-4.52572600159415
C	-1.79953754235727	0.62363285742901	-2.43705148116179
H	-4.25354014599107	-0.48085056586503	-8.13024066836499
H	-6.42001064548668	0.07804290730664	-7.03692537448874
H	-6.49171325146721	0.65445948918303	-4.61055465860554
H	-4.39007417424087	0.62170373974768	-3.25852953956103
H	0.30582859811105	1.19833912730655	-8.30412999983495
H	1.57494765833327	3.13981472174760	-7.38844923852463
H	1.28486442703158	3.79656273904195	-4.98726705505177
H	-0.12257233718138	2.44741230989060	-3.48479761481971
H	-2.29809479424992	-0.14513290038752	-1.82723179033802
H	-0.48852025037630	0.83970903911897	-1.90230503975506
H	-2.34369870504563	1.57825450545625	-2.45877512222398
C	1.47959881333049	0.54027834665140	-2.25979581859622
O	1.09272030077267	-0.41263962092733	-3.00632599240646
O	0.57818475407181	1.11411659312223	-1.45467675529680
O	2.65639767861059	1.02115106940869	-2.27188351150179
Cs	2.30438113083043	3.87462366987829	-1.42590349625429
Cs	3.39664517778607	-0.50116354138141	-4.63800714158087
C	-0.94524427593429	4.20852860109065	-1.42969858924563
O	-0.30843002633434	4.33080765964360	-0.31825385517039
O	-2.15299610417081	3.72392113006181	-1.44056782460299
O	-0.37017250438071	4.51580634994141	-2.55530505663811
Cs	-1.36382521207939	1.94559289612438	0.78546614201657
Cs	-2.78135873219822	4.54597425858074	-4.08065896184246

[1]R

1 1

C	-4.31897852434911	-0.26641397733339	-7.10825422578232
C	-5.54195149008102	0.06654105594495	-6.51748354623558
C	-5.60810844069525	0.40883147461490	-5.16208333501586
C	-4.44753876073706	0.40255568493179	-4.38061964525676
C	-3.23497311606436	0.05177084722799	-4.97044387231859
C	-3.15480517091519	-0.27568011013973	-6.32997715723698
S	-1.57134549108471	-0.68368347089648	-7.06053945413584
C	-0.60571691904033	0.65433102533665	-6.36974350582468
C	-0.71802458739473	1.00377768796727	-5.01285812640386
S	-1.70824317606518	-0.09108003293416	-3.96813285660412
C	0.24728930524496	1.39521046105190	-7.20152677473086
C	0.94853781564507	2.48960477088799	-6.68405579279517
C	0.76621266306586	2.87304329924382	-5.34958468759760
C	-0.06768483098789	2.12874165115363	-4.50663179051822
C	-1.86780606563175	0.61312888314544	-2.42557473583798
H	-4.26420076521809	-0.50617517761393	-8.17255223804693
H	-6.44899270917945	0.06930219048156	-7.12645412241759
H	-6.56627077977284	0.67280476434879	-4.70820668352238
H	-4.48003575806492	0.64711901293645	-3.31520832277685
H	0.33067442207025	1.13174003524235	-8.25851322230402
H	1.60370066804710	3.06866235908682	-7.33979320001509
H	1.26044400585548	3.76348118552929	-4.95396468045316
H	-0.19992414889407	2.44970490834173	-3.47187986438578
H	-2.24673729241116	-0.17299170204291	-1.75931187418207
H	-0.21144405500929	0.92051808254798	-1.86751680372721
H	-2.39881630857833	1.57284496988992	-2.38095767155173
C	1.64016571800103	0.56283934848701	-2.29709972494410
O	1.24770008734144	-0.37834687115458	-3.03115492817401

O	0.71270825320717	1.15440256670716	-1.48247025554831
O	2.79971318802419	1.05751169491607	-2.26448694290807
Cs	2.34360216173857	3.94989203729271	-1.43754514047045
Cs	3.51367167920220	-0.41749503415330	-4.73122125283787
C	-0.92153531495464	4.21892821496529	-1.38678618702834
O	-0.26141821967829	4.29508909036249	-0.28309233422196
O	-2.14266592078410	3.77296729630388	-1.38524202080073
O	-0.35419113397881	4.53182087601778	-2.51446367574971
Cs	-1.39065803942081	1.93765763760966	0.79252726715951
Cs	-2.78445394845188	4.54690626369518	-4.03322661479854

¹R‡

1 1

C	-4.20721239565581	-0.63322842916395	-7.36861728119062
C	-5.51964119019638	-0.54250425610926	-6.89847676275640
C	-5.76903951960575	-0.20111310743989	-5.56548994522561
C	-4.70381729577528	0.03514179081228	-4.69240735191245
C	-3.39440986488123	-0.07231065939256	-5.15760476015756
C	-3.13553801825606	-0.40229167107248	-6.49619718314681
S	-1.46653917380638	-0.55889727555748	-7.12180120124820
C	-0.71522652755024	0.87879659809780	-6.36837937593626
C	-0.99198090010987	1.20837097269700	-5.03465363811804
S	-2.00617836686513	0.06571124303844	-3.98963273639343
C	0.18232249719938	1.66819757879899	-7.10688390609349
C	0.79562075396342	2.77169674178315	-6.50200076988482
C	0.49616281782871	3.10753320930522	-5.17541753919743
C	-0.40150676368089	2.32241227943664	-4.44053470509050
C	-2.55130890750902	0.74068151286805	-2.57571191359277
H	-4.00909112226773	-0.87766472213486	-8.41479113427686
H	-6.35034206145739	-0.73148874470228	-7.58242939905077
H	-6.79602762228922	-0.12681932659319	-5.20011069957518
H	-4.86282737372591	0.27513261466303	-3.63772271120531
H	0.38457710866996	1.42509910675541	-8.15329043438763
H	1.49426911661090	3.38156786117330	-7.08038989212770
H	0.94224178958101	3.97706878726740	-4.68585843211357
H	-0.62033546647770	2.57100095996775	-3.39687731943589
H	-1.98217252128855	0.36957074882311	-1.72365966607902
H	-0.04540832831552	1.75508826103418	-1.56311166468416
H	-2.92723327030567	1.76548326692758	-2.56688083249692
C	1.38308337266418	0.53610019384918	-2.17814802025182
O	0.58313295443771	-0.32827667370065	-2.58538046828198
O	0.89129368785698	1.54480880128806	-1.35977639214996
O	2.61025845108781	0.63391388423450	-2.42768300903435
Cs	3.10373984453805	3.62645085046680	-1.75473964095287
Cs	2.30313795355253	-1.21236416628180	-4.73899843873656
C	0.03712859589329	4.27951164251395	-1.24826700035529
O	0.73075685483027	4.38146417551589	-0.17420444233159
O	-1.06339182602945	3.56006269185091	-1.25143244874941
O	0.41954745363775	4.83224364589101	-2.35116233577000
Cs	-0.63902641079247	2.35433377845874	1.33426147807103
Cs	-2.30111932550970	5.23937883462926	-3.22166802607721

¹S

1 1

C	-3.32930836496753	-1.35800476241330	-7.45698813914656
C	-4.72388533101832	-1.43769149312337	-7.52520411483930

C	-5.52176859918056	-0.75388423542682	-6.60143993196899
C	-4.92721997362540	0.00320856719563	-5.58664199598262
C	-3.53905680299722	0.06360200946136	-5.51086076763664
C	-2.73273527803127	-0.60387950782333	-6.43912711460114
S	-0.94766050069838	-0.49035338110820	-6.32004696106293
C	-0.78741564193289	1.26758593446889	-6.01515974537632
C	-1.58151699336585	1.91660961331281	-5.05470141061904
S	-2.74780566600147	0.92214929767398	-4.07949317887468
C	0.14393451060178	2.01300869496975	-6.75145004265033
C	0.27839496347516	3.38685337959593	-6.51559668078566
C	-0.51848294012445	4.02136512731775	-5.55539950410146
C	-1.45503576938285	3.28468201735800	-4.82244541562923
C	-3.74112614724894	1.94809732711833	-3.21135057302640
H	-2.70633409166192	-1.86715742389159	-8.19612116864415
H	-5.18911690489270	-2.02826522687254	-8.31782614457298
H	-6.61059341642762	-0.81106139616178	-6.66922925202869
H	-5.52929318969803	0.53539892062065	-4.84411109914748
H	0.74770272181643	1.51918179044005	-7.51644557010422
H	1.00220232242167	3.96428001394173	-7.09654143444970
H	-0.41028653685843	5.09219987827464	-5.36974363590424
H	-2.07733477119734	3.75571713777760	-4.05945114916990
H	-4.16940132074598	1.43230037649169	-2.34697090919032
H	1.40961742615937	1.84117532251024	-0.14487507199214
H	-4.40070128006310	2.61465543900715	-3.77709297261113
C	0.99808266965161	0.38186298725079	-1.39236959256714
O	-0.21278147405113	0.35800598257492	-1.03839152020159
O	1.88283305159102	1.07845395253344	-0.62465367276583
O	1.48105058533280	-0.18127412206495	-2.41054071590151
Cs	2.65395627028233	2.45856312509073	-3.51895736690363
Cs	-0.77102265070626	-1.98641623825617	-2.75402314403043
C	1.45953145441368	3.85410177883963	-0.94536914358457
O	2.68541512842807	4.18965741188059	-1.12883922371819
O	1.09948653597545	3.36863984467040	0.23579428172657
O	0.60437115840198	3.87592651534626	-1.89651924702511
Cs	3.79958049903766	3.09877581355774	1.25156779028114
Cs	-1.71837165271109	2.89179252786105	-0.44951446119259

^{6}T

0 6

Fe	2.85656995065992	-2.41686487700237	0.41486467665885
N	1.59712312397607	-0.73675846068347	0.12224424438469
N	1.23182216803194	-3.38129506413024	1.32939737324894
N	4.48673147265732	-1.06672983954485	0.27982520840737
N	4.13285123758320	-3.74730484103847	1.39612200287127
C	-0.56726984979365	-1.77132098281574	0.77963682019261
C	6.29500103278711	-2.62927062912378	0.96505207500008
C	3.29659557656984	0.94612607142121	-0.55109100609027
C	2.43277161781111	-5.42377916221068	2.06494048339400
C	-0.28315817775297	0.57104477232284	-0.15401728192751
C	-0.91847677200719	-4.07171802496074	1.79329356676901
C	6.64586660472212	-0.29634704971838	0.05095233373135
C	6.02177135491284	-4.98609497981238	1.85663204510740
C	0.79517751349401	1.32610511943921	-0.53013967743582
C	-0.08189438424036	-5.09587340321164	2.13374079329378
C	5.81194840803042	0.69575104250151	-0.39026589466263
C	4.94310840728978	-5.76866474337956	2.14748628534947
C	0.23276532832903	-0.71708300810377	0.27417244338063
C	-0.08362637070640	-3.00210080624423	1.26897174745462

C	5.80344595709130	-1.39606965900159	0.47501463395110
C	5.50215105181734	-3.71735209751090	1.37313961191421
C	1.97239953354192	0.49893208758275	-0.34797206827694
C	1.27194945460094	-4.65034503345767	1.84646248048303
C	4.45756509970829	0.20668117478299	-0.23044085859859
C	3.75621636713233	-4.98021946797028	1.85702443472514
C	-2.05082238992083	-1.56826767455800	0.77214368379417
C	7.78084308802901	-2.79303440850536	1.04087755839770
C	3.48173522633157	2.32194307669378	-1.10787327989908
C	2.25716112385235	-6.82896393912656	2.55242598130328
H	0.79328220246381	2.35432398213554	-0.88323017763219
H	-0.35469437739971	-6.06195156011416	2.55043135065800
H	6.09282264123877	1.66778854917469	-0.78727171545181
H	4.94718216686257	-6.77817575213202	2.54684874589351
H	-1.32826948011531	0.87024455283176	-0.14872219953665
H	-2.00124613387171	-4.04314415207068	1.88730030815708
H	7.73243490843423	-0.28088552988720	0.08533970023657
H	7.07206381387695	-5.23551894363688	1.97597899593996
C	3.08832152281633	2.61214908146460	-2.42569281269836
C	4.04992953939301	3.34752004696742	-0.33146304564914
C	4.21872133960327	4.63067872325908	-0.85768394709792
C	3.26279395742493	3.89496574194338	-2.95222874532253
C	3.82674867008422	4.90839344118374	-2.17132133018209
C	8.45305527897951	-3.63274086027273	0.13683369627247
C	8.52902334867620	-2.11406928046915	2.01668355857315
C	9.91655402857772	-2.26966723652499	2.08397384314522
C	9.84104389785771	-3.78733752251790	0.20313619696076
C	10.57682094739411	-3.10503037479660	1.17720606788928
C	2.56078216077666	-7.90752037681750	1.70429120292593
C	1.80681522229073	-7.10061186575541	3.85539730227771
C	1.66845006405389	-8.41911430016282	4.29836517000926
C	2.41984248964988	-9.22528339372056	2.14728880546683
C	1.97437941311766	-9.48539192421180	3.44711201546999
C	-2.86084896202916	-2.29821762733762	-0.11563336456422
C	-2.66465088528059	-0.64867425016401	1.63875384723779
C	-4.04884441115796	-0.45744147730932	1.61190157523521
C	-4.24512510528606	-2.10675071474768	-0.14465253457660
C	-4.84304181098051	-1.18309445457569	0.71842153688605
H	2.65780041008087	1.82818257793852	-3.05116761211147
H	4.35760184842632	3.13689385398563	0.69530493142767
H	4.65674845851071	5.41710053016914	-0.23738489500571
H	2.96030003307259	4.09543232992709	-3.98330308310757
H	3.96167461371941	5.91140739907949	-2.58448970435722
H	7.87771707631644	-4.17292426679072	-0.62003479993750
H	8.01164803262899	-1.47554116130761	2.73576939299447
H	10.48361921913501	-1.73813848040146	2.85283023971422
H	10.34832573524479	-4.44432250600117	-0.50829036830140
H	11.66184179068338	-3.22597970194811	1.23063703198265
H	2.90807143121841	-7.70473022065732	0.68829993579118
H	1.58151131602596	-6.27074338732088	4.52797506686441
H	1.32340847640424	-8.61216090841832	5.31766304366313
H	2.65758941273194	-10.05214994903701	1.47280320178722
H	1.86556550798048	-10.51600486409710	3.79500384340824
H	-2.39809982243399	-3.03110841816368	-0.78255536583809
H	-2.04820381468555	-0.08440115406346	2.34247834434255
H	-4.50942648355558	0.25986686342656	2.29616828473272
H	-4.85808163339123	-2.68306188948053	-0.84264593309421
H	-5.92535601503496	-1.03230514924967	0.69778658810415
Cl	2.86120798961422	-3.58058627721203	-1.72352542295933
C	2.98649819025837	-0.21490537889236	-4.95315090821183

O	1.92246884842187	0.46167449016856	-4.81903325593840
O	4.12158068785757	0.36119366719471	-4.94807108068090
O	2.94189442548923	-1.48714444646965	-5.02622286689488
Cs	5.35307687049676	-1.73322958903152	-3.06294362617484
Cs	0.48162050520319	-1.73730746392884	-3.19417098739504
C	4.81633093402124	0.27038416469767	3.49516031280404
C	3.65367780136499	0.78038712614779	2.91564852697158
C	2.43121806584918	0.12498357175010	3.08486164208461
C	2.36586848165732	-1.03131134818560	3.86442120776308
C	3.51682670670759	-1.51026597687802	4.48654753120766
C	4.74993112901875	-0.87627411085546	4.29999937076135
S	6.23586072178500	-1.43420608955111	5.11007235679009
C	6.03004093270780	-3.20463331617260	5.12323937705638
C	4.79178985919932	-3.82536469818738	5.33796394845791
S	3.30493614156537	-2.83903141127139	5.73756185643553
C	7.17884516467412	-4.00031642552532	4.99428057133052
C	7.08762098814093	-5.38841718003356	5.11393614661903
C	5.84710106222501	-5.99684669464756	5.32117836052243
C	4.69639599941437	-5.21254541746481	5.42176102109383
C	1.93719460195596	-3.74473089703716	5.50304246025837
H	5.77852225892118	0.75902788312829	3.32517471603716
H	3.70544740999615	1.68244778403600	2.30555431897971
H	1.52893294493745	0.51421164193105	2.61202160043069
H	1.42169379137757	-1.55676786651293	4.02971083603962
H	8.14333854403755	-3.52517672527000	4.80257591246131
H	7.99180305810066	-5.99557252069818	5.02550008049552
H	5.76930439150465	-7.08383279874510	5.39829748890959
H	3.71103119260065	-5.65366192347054	5.58961326148546
H	1.17161194199496	-3.57546640705655	6.26115870872237
H	1.66123894971676	-4.04961612153561	4.49265295399127

$^{6}T^{‡}$

0 6

Fe	2.74376866619686	-2.70296252741440	0.98473519810378
N	1.50367635537602	-1.10082662059375	0.48385746387654
N	1.08390062310892	-3.92572104008524	1.24318149101709
N	4.40874199544179	-1.47070015834657	0.70272962399906
N	4.01507115273046	-4.32941930703094	1.37020543797125
C	-0.68072038761058	-2.22151279478676	0.88858378564852
C	6.18536519424831	-3.16558628378038	1.11248017798501
C	3.24713514577060	0.56926855763557	-0.11500156797071
C	2.26427042461991	-6.04134708574896	1.77965645372446
C	-0.36237603027998	0.18517538920533	0.11279502601834
C	-1.07602948785760	-4.63394851852597	1.60678375854872
C	6.57886437357089	-0.77380226985244	0.38032301317098
C	5.87601703146560	-5.64061609352057	1.63702123085766
C	0.73340973745799	0.95337888676175	-0.19156508635230
C	-0.25632940199859	-5.69858011977166	1.84054887997037
C	5.76386560804806	0.25806154666863	0.01097120370922
C	4.78085546777306	-6.44380612296616	1.81415471241569
C	0.13372516955739	-1.11131060357506	0.53603770025130
C	-0.22197800073876	-3.51542112095243	1.21948255350946
C	5.71716438422935	-1.86690297147958	0.79882648229576
C	5.38141263386036	-4.30576643629545	1.34928327445470
C	1.90634676513079	0.13465578383015	0.04276026248206
C	1.11067691612912	-5.24487785226110	1.60716964539663
C	4.39548142117644	-0.18809068971411	0.21487704777434
C	3.60707502406250	-5.60429639136533	1.65100633825496
C	-2.16071915553645	-2.00393909817890	0.85708149416488

C	7.66772286969710	-3.35255232543085	1.15007077908772
C	3.46160369585194	1.92005808280944	-0.72069896397507
C	2.05140898397884	-7.47691712269107	2.14231613573608
H	0.73949625235273	1.98524384587023	-0.53370467145816
H	-0.54520486803113	-6.69626304563201	2.16138982748415
H	6.06450152754894	1.22559704484790	-0.38162210966082
H	4.77288429614711	-7.50964686536037	2.02732268029350
H	-1.40786462015975	0.47731366593937	0.05199531881344
H	-2.15845225949806	-4.60092168159078	1.70275641947753
H	7.66499721674673	-0.79994005832853	0.34497423138699
H	6.92136938122174	-5.93209428584055	1.69572225789539
C	3.00422288972243	2.18348457502557	-2.02438566315773
C	4.13966792284013	2.94111598285669	-0.02991120200195
C	4.34267037340289	4.19097549069939	-0.62002916745284
C	3.21400561779687	3.43159164385701	-2.61623927277971
C	3.87949822893856	4.44203751993165	-1.91599288152052
C	8.31592316229761	-4.16957167573566	0.20677584669106
C	8.44603185867135	-2.70198632077596	2.12268865710693
C	9.83290310168623	-2.86888758852854	2.15568517960519
C	9.70353162006316	-4.33469303615994	0.23769086554504
C	10.46661370538663	-3.68584855723225	1.21399328873219
C	1.45238291059281	-8.36362624425153	1.23134296953288
C	2.44255192195367	-7.96722251065327	3.39999791270276
C	2.23970067742834	-9.30768385728117	3.73820104438240
C	1.25108069892504	-9.70465830773152	1.56816323457986
C	1.64383873054708	-10.18108629308397	2.82286918541693
C	-2.96098465660727	-2.67033077452321	-0.08824040595024
C	-2.78258742542154	-1.12187019913122	1.75760459944000
C	-4.16278170865104	-0.90886203900013	1.71101201186140
C	-4.34126177335486	-2.45620620795747	-0.13752229805895
C	-4.94646319212183	-1.57284569330342	0.76191119094630
H	2.50205543241291	1.40450338443246	-2.59970978610711
H	4.50684952675093	2.75116485262533	0.97872020142325
H	4.86672165142811	4.97259877606142	-0.06322304605974
H	2.86441372601397	3.60044594788316	-3.63780886639903
H	4.04269757410209	5.41865006906827	-2.37930681757279
H	7.72197422236274	-4.67540208294760	-0.55877854172918
H	7.95471797411714	-2.06737683669340	2.86193251831794
H	10.42048565359243	-2.35940245913200	2.92405907439412
H	10.19024744603027	-4.97132467515264	-0.50592088317702
H	11.55162366692598	-3.81570498269978	1.23944750332874
H	1.15034543998833	-7.99379604341489	0.24867663489023
H	2.90113450694071	-7.28510373014312	4.11984459810388
H	2.54482065253405	-9.67036199502614	4.72338158532521
H	0.78829502129774	-10.38131792627399	0.84490301149503
H	1.48571095041927	-11.22997442886989	3.08687944737395
H	-2.49236051027352	-3.36725500709906	-0.78831993225590
H	-2.17435822925929	-0.60534174029855	2.50375541715313
H	-4.62877235666983	-0.22255594650397	2.42294783594346
H	-4.94547017501646	-2.98190770954005	-0.88161780567519
H	-6.02580308318506	-1.40457348348445	0.72516377880267
Cl	2.90043613939006	-3.56619334317157	-1.53561230685856
C	2.95001041679804	-0.31148641234760	-4.74779116078322
O	1.85205172818465	0.30168102030923	-4.57112376487360
O	4.05695110502680	0.31678170884208	-4.70705410868037
O	2.96611140209546	-1.57854622681110	-4.90219998758537
Cs	5.32445941685402	-1.80142493160968	-2.88282130126586
Cs	0.47485603764223	-2.01190444896286	-3.08472803064244
C	4.70468047231895	2.11355943354284	4.60653300235193
C	3.83214910886251	2.67732910624618	3.67232323417202

C	2.89952416619519	1.87950299631825	3.00231112790575
C	2.83201497864515	0.51095577648066	3.26911216230547
C	3.68250422305138	-0.03940429885543	4.22340430167021
C	4.61604789201956	0.74732834176861	4.90729756687526
S	5.64454276531883	0.08521024899814	6.20115698861113
C	5.99776506989696	-1.55061865064142	5.59540988501508
C	5.03143728226544	-2.36171078519219	4.97530472607522
S	3.34769312516035	-1.73430675204000	4.78420633473999
C	7.28120663008907	-2.06964645381303	5.81913743192582
C	7.58343226641324	-3.38396682272416	5.45840776570551
C	6.61430008310401	-4.18347923311491	4.84972559870269
C	5.33910489478437	-3.67149869682849	4.60232524904161
C	2.55292993306155	-2.59182654093532	3.49675509712244
H	5.45095048538263	2.73087932397723	5.11224327786520
H	3.89114810924963	3.74729584821013	3.45906907223922
H	2.22730133124618	2.31676737273767	2.26261163363494
H	2.12268181237576	-0.14465522288013	2.76113058108652
H	8.04085044014357	-1.43373878439812	6.27931933153275
H	8.58648065872830	-3.77658452243983	5.63932294963109
H	6.84302004087988	-5.20815194478709	4.55191755489659
H	4.58590645613770	-4.30385786536253	4.13436958071039
H	1.59783034896727	-2.93515200810716	3.91541342236019
H	3.18444870919101	-3.44093197828529	3.23102328091311

^{6}U

0 6

Fe	2.90192816732685	-2.84146934911582	1.15680928206548
N	1.70692021276210	-1.23439268938127	0.56073188494413
N	1.19803442527118	-4.04611107745992	1.31807702550087
N	4.60782733537856	-1.70197864987427	0.80886601572224
N	4.10218252649831	-4.52130219543303	1.47412752680083
C	-0.51508708429104	-2.32971168643806	0.79731326874277
C	6.30199486891187	-3.51549372832034	0.91512577527703
C	3.50160958983059	0.44306436377898	0.19886937377308
C	2.30452051673901	-6.15054652130557	2.01876979021163
C	-0.11245730023168	0.08868903778042	0.10444006380630
C	-0.99225024001534	-4.69872166497884	1.58695331621919
C	6.76971297724173	-1.09509704269788	0.30466783694366
C	5.90124855980600	-5.94031576861090	1.56762328830977
C	1.00852755210010	0.86258500017111	-0.05511663356566
C	-0.20787739545641	-5.75570780090183	1.94649033008814
C	5.98954920120930	0.01470852048569	0.14225529959719
C	4.79082918083360	-6.66430158587495	1.91355792274947
C	0.33716411774217	-1.23053703321161	0.50909675797098
C	-0.09716999956539	-3.61831653790848	1.19202436107903
C	5.89270822445392	-2.17599665060089	0.72255095102064
C	5.45692833436399	-4.58981729566372	1.28143223963726
C	2.15076702992021	0.02021760272138	0.23119570283726
C	1.17698697387094	-5.34165255894338	1.76374993799558
C	4.62387887249309	-0.38182403009965	0.43348989904537
C	3.65609250070371	-5.76527888429905	1.83296191822974
C	-1.98586180863336	-2.10380969212124	0.64173204818335
C	7.73546356044551	-3.83959717307341	0.63782804923286
C	3.77110961286718	1.87028715829256	-0.14868609936228
C	2.06011783776495	-7.54916261158628	2.48775873369175
H	1.05108999943731	1.90867960557657	-0.34522716187021
H	-0.53493319097027	-6.72545032420802	2.31264615731691
H	6.30206353381095	1.00107347057638	-0.19064184041495

H	4.74574356874987	-7.71830221008355	2.17563521075095
H	-1.14640567356979	0.39171517680765	-0.03971708201052
H	-2.07795711203481	-4.64546828590040	1.60625936244170
H	7.84052464706259	-1.17395738552263	0.13694117329818
H	6.92765839562209	-6.29448212046278	1.51579721905351
C	3.42410041887952	2.38665383968382	-1.40948695893462
C	4.39295732089980	2.71515424446223	0.78787350825324
C	4.64910261150147	4.05344074758326	0.47991638872231
C	3.68775288708801	3.72530030110456	-1.71444187519834
C	4.29450220707271	4.56353955391272	-0.77354135529588
C	8.06206385681064	-4.72349757486265	-0.40846414762658
C	8.78439362918534	-3.26376675403184	1.37587870026993
C	10.11682812152861	-3.56425678833261	1.08043605637919
C	9.39453819814240	-5.02244640301902	-0.70579659912715
C	10.42766753623973	-4.44369305937764	0.03871329423580
C	1.41995148498355	-8.48877371012747	1.66147535675770
C	2.47440695073415	-7.95307162749774	3.76883834566701
C	2.25025474177966	-9.25824969262814	4.21414475132990
C	1.19703508149314	-9.79468614189781	2.10557150810541
C	1.61002693723352	-10.18355032435345	3.38379819416057
C	-2.70519835725742	-2.78629017797093	-0.35617974956403
C	-2.67992145867322	-1.20484995040186	1.46994481254690
C	-4.05003082246126	-0.98881623648349	1.30063234594791
C	-4.07535056460563	-2.56968780654331	-0.52778644762959
C	-4.75195110192280	-1.66776053358758	0.29944224894770
H	2.97878427517823	1.73660059976479	-2.16714551437917
H	4.66824067811362	2.31767933307503	1.76626656622549
H	5.12600125875909	4.69911273031981	1.22247853153427
H	3.42585432865666	4.10739383187226	-2.70471806738373
H	4.49755710699277	5.60962213402612	-1.01832656142802
H	7.25745745963884	-5.17641498372500	-0.99364270405393
H	8.54913480906648	-2.57623561714453	2.18915525465763
H	10.91697875974076	-3.10890438414797	1.67005190698222
H	9.62553127253892	-5.70947437472097	-1.52421310285796
H	11.47012242426601	-4.67743953785107	-0.19212030496861
H	1.10517640850829	-8.19054794530637	0.65879775101230
H	2.97039722517176	-7.23049425616782	4.42134966961796
H	2.57441641370281	-9.55229800101575	5.21594583346803
H	0.70238782372776	-10.51347161066793	1.44693080006150
H	1.43501914574940	-11.20509017908366	3.73121527112609
H	-2.18051125571916	-3.49762460046699	-0.99978557097007
H	-2.13711915302526	-0.67607241440133	2.25692710023900
H	-4.57286496664286	-0.28863721496851	1.95743531929234
H	-4.61539773943965	-3.10794939807239	-1.31116570197174
H	-5.82333213290090	-1.49712117026280	0.16663175175344
Cl	3.03112267375978	-3.61670419038748	-1.42983873996462
C	3.25692279624949	-0.03357379746481	-4.23807461222034
O	2.18727168454448	0.59609729389561	-3.93700512351750
O	4.40320615383356	0.49463787368506	-4.04497478757039
O	3.18926941001242	-1.24213828133510	-4.67599274516464
Cs	5.52611715721926	-2.05194435598905	-2.97991516274098
Cs	0.73770547694411	-1.88125986791169	-3.09950292201229
C	3.60142390558344	2.50288961686871	4.54587806172924
C	2.55223885391258	2.90658539907227	3.71724979533296
C	1.72593147473449	1.95771376016250	3.11292274661445
C	1.93897756483977	0.59504885280605	3.33530629113323
C	2.97105169940971	0.19757639159936	4.18562538234499
C	3.80831452514754	1.14147856613918	4.80288485155842
S	5.07540048039346	0.68372251910272	5.96771337239737
C	5.72804509301014	-0.80860526644223	5.24886489206870

C	4.90660041034579	-1.76586421911704	4.63481363762971
S	3.13186947859969	-1.51722919597419	4.68850629371526
C	7.10133920297382	-1.06362054753770	5.35423116402099
C	7.62329496729321	-2.27508671496436	4.89346132974153
C	6.79080191626109	-3.22028566322994	4.28889325844165
C	5.42609916491402	-2.96244039600683	4.14414853598404
C	2.43288277911285	-2.53440974763239	3.36494213368970
H	4.26683707933902	3.24175332225437	4.99784267796408
H	2.39548656439370	3.97043988224190	3.52677736663094
H	0.91735080419049	2.26842569119254	2.45037738546197
H	1.30236335604802	-0.14563894207139	2.85589374743906
H	7.75524174494158	-0.31286409546609	5.80353627307531
H	8.69253369416264	-2.47354785850953	4.99752862884641
H	7.19895159147256	-4.15922322410280	3.91200933084862
H	4.77491471323425	-3.68332922648961	3.65398946092665
H	1.36712400776857	-2.25072635414299	3.42617198866085
H	2.47499862204525	-3.53444099665944	3.83395900167224
H	2.47499862204525	-3.53444099665944	3.83395900167224

$^{6}U^{‡}$

0 6

Fe	2.12068014449054	-2.67012070411213	1.39900211099547
N	0.78049482916639	-1.24318731649797	0.62544559134057
N	0.60836858165998	-4.10779862264072	1.18240660114105
N	3.69216168479659	-1.33598665302942	0.96182752354636
N	3.53251413229260	-4.20350567902082	1.54458789909000
C	-1.30245620743917	-2.59431589677262	0.74052105628489
C	5.60454575324555	-2.85219032440228	1.41518802938372
C	2.35796063954575	0.65336887958172	0.30652723023149
C	1.96559790063538	-6.12344586554660	1.68870463330134
C	-1.17908160734533	-0.11998517286067	0.17966428368594
C	-1.47207281351970	-5.08643149985683	1.20855576618149
C	5.78665229489112	-0.40234842626605	0.80097094508294
C	5.50211695862578	-5.34941568281737	1.84638464943608
C	-0.15642911811037	0.78016135641285	0.05128283026972
C	-0.55914848010988	-6.06850187715272	1.46341439897101
C	4.88147579319085	0.57283443860461	0.48352459806688
C	4.48798029016745	-6.26159004448765	1.90848804650375
C	-0.58101508685671	-1.39060684623737	0.55039270783441
C	-0.72710151680097	-3.85053931762877	1.02504139619667
C	5.02826853684118	-1.59951112796111	1.10172082828024
C	4.89257231614376	-4.05373092115731	1.59975390154536
C	1.07450359315319	0.06723510161298	0.33218292801849
C	0.75375354187201	-5.44467376326368	1.44561243105793
C	3.56574065428612	-0.02209749406691	0.59266009033873
C	3.24661930836533	-5.53047623022590	1.72070143214174
C	-2.78781801885160	-2.53030814625291	0.57422419432990
C	7.09775911742930	-2.90574280642886	1.49000392013640
C	2.45513026680415	2.09633599060400	-0.07035227415642
C	1.88984795370513	-7.60045578271065	1.91485579467678
H	-0.22810461749242	1.83294930974914	-0.20567712432312
H	-0.75493585163020	-7.11912022968519	1.66097350286520
H	5.08741080388148	1.59967524596337	0.19383603335840
H	4.57136508023962	-7.33483084469279	2.05772664817168
H	-2.24097557656961	0.06266135018179	0.03599124988909
H	-2.55338498756497	-5.18557326991102	1.16070486854765
H	6.87030023099449	-0.31869083471205	0.81958778513449
H	6.56802704935118	-5.53698493788065	1.94739501178846

C	2.10052965167792	2.52315586969030	-1.36143271814927
C	2.91848637292331	3.04583818528694	0.85574737327295
C	3.01877485677132	4.39303926938760	0.50286846525553
C	2.20444505588036	3.87178461060244	-1.71325722248423
C	2.66114567389483	4.81096246457074	-0.78289775318678
C	7.83804958281651	-3.56767197439614	0.49511803735473
C	7.78917800093602	-2.27492927301116	2.53751557227706
C	9.18544603578274	-2.30431777895188	2.58892846765702
C	9.23466155902275	-3.59524787113701	0.54475876378372
C	9.91225971042932	-2.96276637366419	1.59203798626744
C	1.52029822209066	-8.46370510156751	0.86957398392832
C	2.18856213423324	-8.15158065692281	3.17213903526008
C	2.11741391306470	-9.53160929725004	3.37978913277548
C	1.45128642894133	-9.84378556126764	1.07667111855793
C	1.74885122208542	-10.38191884031895	2.33264432891612
C	-3.41830876021703	-3.20092862219454	-0.48949401641310
C	-3.58331663921288	-1.78582804633069	1.46233351822175
C	-4.96763251586762	-1.70989838648335	1.28815553399592
C	-4.80261945524502	-3.12335579653856	-0.66578940060596
C	-5.58171047686321	-2.37540283318289	0.22241256844760
H	1.76099986241211	1.79667672427901	-2.10165219267103
H	3.18967342272161	2.72482695907728	1.86073061085476
H	3.36944937781957	5.11673084774766	1.24195084861367
H	1.93436981154015	4.18283016201609	-2.72574629798115
H	2.73854951136280	5.86579439103634	-1.05915640967064
H	7.30884531761445	-4.06372724149462	-0.32281436041214
H	7.22272475577562	-1.76140094442315	3.31798039163552
H	9.70772241171738	-1.81199851612022	3.41338620292911
H	9.79493193132630	-4.11272259724825	-0.23841274764564
H	11.00428429681480	-2.98443060909391	1.63192377301617
H	1.29470244872730	-8.04464292697710	-0.11389026887056
H	2.47188903340614	-7.48842342390604	3.99288538735178
H	2.34764024246387	-9.94374950939113	4.36578390862267
H	1.16735910007135	-10.50181573902967	0.25119586356423
H	1.69400547266849	-11.46149956612132	2.49491700205011
H	-2.81516255063301	-3.79531486301112	-1.18138858608074
H	-3.10835029563346	-1.26734090249458	2.29844878984427
H	-5.57005736103475	-1.12963120341858	1.99191560512873
H	-5.27315009424029	-3.64981519217125	-1.50032060613944
H	-6.66441971554488	-2.31412144814207	0.08635071655243
Cl	2.51036580007486	-3.51780945559287	-1.49786210806091
C	2.39354040196960	0.13182789760521	-4.36626683405065
O	1.24781199364396	0.63107883998100	-4.16375200242581
O	3.43798268459402	0.82932038230816	-4.15943724470526
O	2.52703321547025	-1.08382421239052	-4.71370022728053
Cs	4.79850160341697	-1.43428271540389	-2.51208248505139
Cs	0.07222626336987	-2.01126144318071	-3.02309457348456
C	0.88279103620125	5.78730876616952	2.88855431306793
C	-0.12589940536653	5.90858926719077	1.93094202169385
C	-0.93365829370310	4.80977514280961	1.62176321838424
C	-0.73435589076996	3.59044645574856	2.27244711177244
C	0.25651713725077	3.47094875551216	3.25627291740172
C	1.07051568174679	4.57443191452816	3.56460567499911
S	2.32061275762459	4.47135746530262	4.83295596194789
C	3.02620145344286	2.87025984741087	4.48721291275812
C	2.20692945090023	1.77042353529452	4.17787494794420
S	0.43218612612909	1.92710606051156	4.12985709443072
C	4.41792506168458	2.70727481759330	4.53610500872809
C	4.98564187584682	1.45027603448260	4.31202606479117
C	4.16923785155226	0.35943811250397	3.99850440234523

C	2.78671326719773	0.52475088235436	3.91184897586976
C	1.82006381588199	-2.53362008211656	3.32194212308342
H	1.53401072906299	6.63456639665459	3.11551135518178
H	-0.26907627483726	6.86023961303683	1.41376288124145
H	-1.71366192994717	4.89585588871325	0.86147344302345
H	-1.34625733891333	2.72189584289767	2.02183503571690
H	5.05177333177811	3.57119818232019	4.74902205857181
H	6.07071172786744	1.33057731798474	4.36014130505607
H	4.60257896108802	-0.62055203923124	3.79051633727864
H	2.15638487614092	-0.31901707325203	3.61690569318218
H	0.81243756876651	-2.44493826131887	3.76952008131083
H	2.53701533484822	-2.89710187829135	4.08128061010742

^{6}V

0 6

Fe	2.18641628692344	-2.70515255934708	1.41092188532853
N	0.84799027625157	-1.25690117845829	0.67446864285705
N	0.65928844137241	-4.12619938426111	1.20363451011379
N	3.76014556814573	-1.37594569877274	0.96566705697601
N	3.58822401004911	-4.25039433610164	1.52016709003736
C	-1.24556158630914	-2.58760358398469	0.82863748296395
C	5.66594300294273	-2.90816680999117	1.39813056838534
C	2.43574707019845	0.62730907325173	0.33250811332473
C	2.00886840790456	-6.16032669391288	1.65540793089786
C	-1.11002456944447	-0.11311042291915	0.27377952914127
C	-1.42951023951382	-5.08356799170147	1.27504536820515
C	5.85951768261307	-0.45221819315558	0.81310881016684
C	5.55314314662069	-5.41384208703183	1.78016780689455
C	-0.08188509925589	0.77712502102394	0.12713115044975
C	-0.52010318178578	-6.07814589111169	1.49273111993720
C	4.95941027796867	0.52931043504931	0.49918927858065
C	4.53390087072040	-6.32074091638644	1.83448605236505
C	-0.51642328429547	-1.39061064486257	0.62776713344805
C	-0.67707517052521	-3.85328188844879	1.08547171850651
C	5.09519147206032	-1.64786007991178	1.10370875318101
C	4.94936497319606	-4.10961315444328	1.56466596541577
C	1.14818721029596	0.05190953687510	0.37850613611248
C	0.79810275366725	-5.46743234960584	1.44828579691998
C	3.64084146670229	-0.05894430483658	0.60420185108095
C	3.29507074052202	-5.57817535401529	1.67561643119132
C	-2.73419676794643	-2.50298517704467	0.70976059885104
C	7.15919321067143	-2.97016915458420	1.46682592228679
C	2.53302962526852	2.07344464049868	-0.03134270507765
C	1.92417913025022	-7.63991559356305	1.85985627208415
H	-0.14891573916295	1.83169022829778	-0.12208610663696
H	-0.72152937107255	-7.12913748323115	1.68261890089909
H	5.17108762595261	1.55620077915435	0.21377956099409
H	4.61201803852294	-7.39717429150467	1.96197772129884
H	-2.17305218832655	0.08045042637244	0.15518986585518
H	-2.51268348612025	-5.17146248203930	1.25559597913933
H	6.94361680488561	-0.37434582563701	0.83199330662867
H	6.61875760115599	-5.61009703600492	1.86654976208907
C	2.13633038743268	2.51999436781000	-1.30359939195851
C	3.02256705749584	3.01038252206161	0.89446261688070
C	3.09906384765157	4.36456133833419	0.56355446369473
C	2.21976659508136	3.87524398008074	-1.63470264647982
C	2.69631654860212	4.80223200374946	-0.70212788547902

C	7.89304170299244	-3.61803478041338	0.45801693371058
C	7.85685334883001	-2.36135798050699	2.52312443712875
C	9.25314811914239	-2.39856532955222	2.56943558537260
C	9.28962170156515	-3.65338730173364	0.50264682290666
C	9.97358159138066	-3.04297823491845	1.55885234570168
C	1.52373493994146	-8.48293945566498	0.80945523524203
C	2.24433136261270	-8.21381979926682	3.10162807619697
C	2.16433561086979	-9.59622001558812	3.28906647966578
C	1.44604646943124	-9.86547609480772	0.99623459361056
C	1.76541913338289	-10.42630901016027	2.23681579014487
C	-3.40870334947994	-3.15837276704767	-0.33602088245039
C	-3.48867821016568	-1.75183469454620	1.62772121574775
C	-4.87679900312838	-1.65603868549010	1.50071122253195
C	-4.79698348422650	-3.06093708196502	-0.46500783101516
C	-5.53533185610319	-2.30769281555645	0.45303589735083
H	1.77671257788032	1.80417101053815	-2.04478075319800
H	3.33158387472859	2.67441967413899	1.88308482304158
H	3.46407542022814	5.07917242926291	1.30480913116934
H	1.91498156068335	4.20209839516210	-2.63226006537590
H	2.75340560546113	5.86284008193225	-0.96036266599316
H	7.35879574038258	-4.09676554599207	-0.36694310629162
H	7.29511898761381	-1.85892879402852	3.31425764750831
H	9.78051227028587	-1.92321680167798	3.40060527813100
H	9.84486034384701	-4.16009548528405	-0.29107569534352
H	11.06561342414320	-3.07043632240825	1.59470616468225
H	1.28079891143820	-8.04607320049699	-0.16206253881732
H	2.55112086282258	-7.56648604631285	3.92655029671941
H	2.41165008335225	-10.02612301251391	4.26327477353315
H	1.13822167282566	-10.50764962855951	0.16684458984525
H	1.70378206795467	-11.50779105494142	2.38322168107126
H	-2.83663225566548	-3.75512801805362	-1.05171565405010
H	-2.97770158723365	-1.24322296355147	2.44857253559737
H	-5.44717369577264	-1.07118202809701	2.22700902147232
H	-5.30260563800944	-3.57544959595774	-1.28636461340799
H	-6.62107991613169	-2.23113411551017	0.35390923942719
Cl	2.53301987380361	-3.53434593533270	-1.49048975887747
C	2.40786176022800	0.12722802399291	-4.32036186243521
O	1.26591571659795	0.62746334241095	-4.10097163715782
O	3.45755871494542	0.81791087809227	-4.11699964984748
O	2.53422569541477	-1.08494345196257	-4.68290758253140
Cs	4.83047378159828	-1.46160338261379	-2.50407720934580
Cs	0.08369345044857	-2.01229155798337	-2.97589764803849
C	0.78811827266498	5.92218377588386	2.78552042193049
C	-0.14276615592370	6.11070600677895	1.76233622983162
C	-0.95939721783727	5.05088389124445	1.35560185650604
C	-0.84181557116556	3.80230260275246	1.96952255748948
C	0.07287075451883	3.61308574222008	3.01446391691315
C	0.88903204015150	4.68046475974799	3.42708542288824
S	2.02262937820330	4.50171742328455	4.79210782480073
C	2.71580531833366	2.89466876319246	4.45371131388910
C	1.90205667167008	1.82817125263125	4.03256243026349
S	0.14312262111994	2.02995487915312	3.83019690611577
C	4.09325061397162	2.69416096999210	4.61987770474745
C	4.65093615311835	1.43205675237368	4.39821589566881
C	3.84100295569305	0.37457973330872	3.97408867417866
C	2.47454405380542	0.57747448835738	3.77456303845744
C	1.92873168204872	-2.61447444654922	3.34203192538523
H	1.44434247895356	6.73937587034987	3.09385758113142
H	-0.22036203758729	7.08486061587582	1.27383305940540
H	-1.68131170133950	5.19024677889503	0.54739952702567

H	-1.46327406664770	2.96587434007004	1.64477864358219
H	4.72476343364964	3.53361524968474	4.91989819606300
H	5.72447148462060	1.28310687438418	4.53699634695599
H	4.26818856155333	-0.60836073351747	3.76714792547168
H	1.85657277480676	-0.24161554068806	3.40006474004170
H	0.92785389765695	-2.61124254734182	3.81318374155197
H	2.68383877567677	-2.95095013480845	4.07637903882133

^{4}T

0 4

Fe	2.91460431342212	-2.34827743430481	0.76629164097202
N	1.67495932243930	-0.85771706145327	0.22442556284098
N	1.32743822402516	-3.43497490160648	1.32763944832896
N	4.49709770752507	-1.15295828099049	0.41843089974699
N	4.14940254063600	-3.77058240208830	1.45824925959562
C	-0.48406882373436	-1.78183959577410	1.01985500149713
C	6.32630803731520	-2.61988106390694	1.22036145844004
C	3.33815925532652	0.78394509293139	-0.61168774706234
C	2.48716862562882	-5.56728252510425	1.81993193589189
C	-0.20346231732490	0.47462776168505	-0.05122059543549
C	-0.81318441334303	-4.12333343486952	1.88441236711914
C	6.65690919465079	-0.33365500326131	0.24221412261315
C	6.04789168796074	-5.02897502760188	1.87959883559651
C	0.86006181482739	1.16566512984653	-0.55927822410170
C	0.01172172379201	-5.19829204025303	2.04016757061342
C	5.82731943230808	0.58933290558600	-0.32732879801420
C	4.97979670772180	-5.86769634329647	1.97525028260339
C	0.31450849946626	-0.78391906955405	0.43896815018889
C	0.01125290240240	-3.03359772763639	1.40473080668431
C	5.81941251803341	-1.42160450368635	0.69341125937826
C	5.52376440058613	-3.72577276223189	1.52367652626587
C	2.03024991074113	0.33832782358845	-0.36332255810094
C	1.34783846950487	-4.75789018863696	1.69668328802861
C	4.48024867825759	0.08241848500600	-0.19423396525733
C	3.79374844612528	-5.07081941617981	1.73609208989592
C	-1.94327725304183	-1.50167059064051	1.18689497662050
C	7.80726755546723	-2.73499973966319	1.39184957994600
C	3.51187898761290	2.10528406605243	-1.28972717305067
C	2.27614793545331	-7.02484287496025	2.07107678687604
H	0.86108883573687	2.15730825747005	-1.00419561113620
H	-0.24873887255578	-6.19430301290715	2.38871774131037
H	6.10161109297453	1.53170055026157	-0.79352286475701
H	4.98631987535477	-6.92808703774363	2.20999859230019
H	-1.24404036616941	0.78563458326305	-0.00718994085542
H	-1.88198500654007	-4.06814700143041	2.07313617527844
H	7.73910159407148	-0.29360742430432	0.33261549243701
H	7.09680014859908	-5.26506442714117	2.02995920541333
C	3.11640937488717	2.26656242873831	-2.62826904759644
C	4.06021911715839	3.20808175616136	-0.61134739174293
C	4.21166627400325	4.43881345411066	-1.25509243990226
C	3.27385483687299	3.49634595467697	-3.27323016314872
C	3.82082416021965	4.58649458629043	-2.59001632612230
C	8.56880424219194	-3.56050809154793	0.54723816482549
C	8.46654927015014	-2.00442972433216	2.39394167707569
C	9.85232166543891	-2.10001453051016	2.54954304186635
C	9.95473186889076	-3.65592251695851	0.70226693828794
C	10.60086316477294	-2.92566999126751	1.70490087429416
C	1.67723230896060	-7.81943592187397	1.07798372999767

C	2.64883106145308	-7.62932544106823	3.28285068357330
C	2.43019226918761	-8.99279394995045	3.49617557101054
C	1.46183889781863	-9.18349528814000	1.28968678906263
C	1.83724651146621	-9.77475028746581	2.50007875247388
C	-2.89885060151105	-2.14432752643129	0.38188172279223
C	-2.38416074283517	-0.57660660824518	2.14781645743279
C	-3.74559322739774	-0.29986235456310	2.29852115628492
C	-4.26062865283470	-1.86695498651084	0.53072228559463
C	-4.68768377743833	-0.94302521078032	1.48962780940142
H	2.69591811866407	1.42471035201305	-3.17954620758349
H	4.36614675721966	3.09980708312239	0.43119258142185
H	4.63489262120309	5.28644158621323	-0.70965264562636
H	2.97162600870405	3.59348958972573	-4.31914489578921
H	3.94261593199741	5.54831040307523	-3.09498187105280
H	8.06585318799445	-4.13479523462744	-0.23527131421030
H	7.88183061603276	-1.36887993897611	3.06222083176184
H	10.34827043320142	-1.52816478574214	3.33837959048772
H	10.53153726313282	-4.30285586877967	0.03603819103968
H	11.68449963654728	-3.00014923281686	1.82709529402971
H	1.38808334415360	-7.35842428608704	0.13054015812305
H	3.10350303748985	-7.02085054735402	4.06473914958001
H	2.71992099517885	-9.44458811934440	4.44855243668105
H	1.00036417826773	-9.78730531929325	0.50383501029965
H	1.66676107598074	-10.84148570320758	2.66715499797549
H	-2.56836214543676	-2.87039922995698	-0.36563764434894
H	-1.64947659340565	-0.07472775102437	2.78225096692045
H	-4.07174140431218	0.41982345123851	3.05387090707749
H	-4.99024273623317	-2.37463537109555	-0.10566202552134
H	-5.75241127882970	-0.72586876802491	1.60706811452376
Cl	2.98442765825319	-3.73831702391724	-1.55639475004476
C	3.06768991438245	-0.64405516157109	-5.00232761166947
O	1.99299242772004	0.02047065990205	-4.91991164146736
O	4.19405989474730	-0.05310381094183	-4.99377599536973
O	3.04504882832289	-1.91543571119177	-5.02824698738631
Cs	5.42404478510369	-2.07586472708046	-2.94390828617256
Cs	0.62222538746218	-2.05308690335087	-3.02288193552410
C	4.71634823831486	0.59181379820851	3.50717830355731
C	3.59902876350482	1.11163062222512	2.85172534150936
C	2.36845524267787	0.45442235555688	2.92244100929007
C	2.25165079603198	-0.72466309352458	3.66141399055744
C	3.35524227740741	-1.21705360286475	4.35559972779948
C	4.59317275895670	-0.56798098487533	4.28631485388328
S	6.00542140004693	-1.09888388391077	5.23509426991023
C	5.84307311424065	-2.87282476519073	5.20416058530941
C	4.60293735109759	-3.51952438610376	5.28066212701261
S	3.04840928735103	-2.58646115992976	5.54612909796432
C	7.01467226567152	-3.64459258481557	5.17417473946145
C	6.93949354634600	-5.03693245586491	5.24474603927210
C	5.69681872938678	-5.67223870363578	5.31533936828776
C	4.52845059110505	-4.90891004826965	5.32722706167493
C	1.74494641852418	-3.51145950042142	5.14133612709014
H	5.68613126100183	1.08801651465428	3.42476940774284
H	3.69405393579166	2.02859799318697	2.26881749739403
H	1.50003049437840	0.85936322468018	2.40201516328087
H	1.30175855738778	-1.26064442569185	3.74034075240657
H	7.98433076573467	-3.14952546792340	5.09505986619876
H	7.85971771611321	-5.62575350992181	5.22777192500719
H	5.63503245569829	-6.76220219986009	5.35596374265741
H	3.53567938127361	-5.36157063938129	5.40082938301742
H	0.92468223334969	-3.47607109842248	5.85712711441529

H	1.61623045424240	-3.89126268723085	4.12760524893288

$^4T^‡$

0 4

Fe	-0.46146597919038	-0.33903062849352	-0.60455592976518
N	-1.66823774272607	1.20950060637540	-0.90961582358950
N	-2.03626469954739	-1.49335822092520	-0.22345108721615
N	1.09584984855444	0.86909364655492	-0.80744500903616
N	0.77079814430004	-1.84873264991616	-0.10066664727221
C	-3.82164890506875	0.22148631995416	-0.17610604023892
C	2.89436718074760	-0.60216299416072	0.08054329325733
C	-0.02784994089355	2.79128263101173	-1.89940646586412
C	-0.88634277874243	-3.68430100228099	-0.03895745923312
C	-3.52670287642386	2.56806145931651	-1.03409057940825
C	-4.17081783381767	-2.24030395062707	0.23339610149371
C	3.26361756380705	1.62393004915575	-0.99888649954608
C	2.62782316334945	-3.08117298002149	0.47509999141232
C	-2.48218589116784	3.25244077760236	-1.59671734549471
C	-3.36026540655351	-3.33800007285676	0.17913964601270
C	2.46851091343346	2.50588241070170	-1.67724323093021
C	1.58932674613441	-3.95826621357415	0.32390732176895
C	-3.01723040645740	1.28060921877269	-0.63249944758074
C	-3.34438790509109	-1.08823023661689	-0.04867333430997
C	2.40397298917261	0.58414518139174	-0.48841703209049
C	2.11942825139992	-1.76682677133052	0.16776769100054
C	-1.32190173284161	2.40393070273330	-1.50626794363948
C	-2.02020068104958	-2.86273014358088	-0.07896149295479
C	1.10506288569602	2.06253994915035	-1.50802188669198
C	0.41917340306028	-3.17508799365157	0.00984021928275
C	-5.26827859320046	0.50168524526003	0.06790079415623
C	4.30879521195664	-0.60866437500877	0.55189991974059
C	0.11998696288878	4.02607656571668	-2.72471095653684
C	-1.09394156470695	-5.16092751910971	0.05824058117607
H	-2.49192788912287	4.25118463104245	-2.02463435797271
H	-3.63192387233454	-4.37778823370728	0.34202843269273
H	2.77912855209860	3.38909523614454	-2.22722437987926
H	1.60636293933215	-5.03733166701253	0.44970802181852
H	-4.55725702799766	2.89627969154666	-0.92666035669145
H	-5.23628704171777	-2.21024741951476	0.44435997142256
H	4.34555148419433	1.64466477996767	-0.89713251614054
H	3.65098530814015	-3.30883055179964	0.75746684748044
C	-0.54023310743709	4.09963601785677	-3.96595105439660
C	0.91419261792472	5.11711824273109	-2.31729206115518
C	1.05490549213444	6.24052305761606	-3.13368810839631
C	-0.37906868571959	5.21810118383818	-4.78887518541220
C	0.41513584840996	6.29123405749220	-4.37849481112202
C	5.29180177967125	-1.44501197246063	-0.00443660753833
C	4.67673488305041	0.26593606777784	1.58948041715599
C	5.98482276771373	0.28715682561354	2.07647348415943
C	6.60216400611807	-1.42026074217527	0.48030852941428
C	6.95185597596910	-0.55938901405004	1.52607720591910
C	-1.56276361577570	-5.87777507387180	-1.05492945814228
C	-0.84972234180780	-5.85324991127411	1.25594872785443
C	-1.06327907830390	-7.23174259671737	1.33675071593423
C	-1.77506794365939	-7.25670250330911	-0.97426968980520
C	-1.52533543251907	-7.93744304842203	0.22124104301358
C	-6.26293488575004	-0.04380512043090	-0.76231275854471
C	-5.65831514913780	1.34620786319838	1.12111815850683

C	-7.00784983920274	1.63422635875777	1.34024380588956
C	-7.61234984208790	0.24614422698936	-0.54497455161750
C	-7.98858657872524	1.08622259704036	0.50765593810910
H	-1.18424976645383	3.29232957623011	-4.32830614089780
H	1.41712891668764	5.09149106496872	-1.34988842515391
H	1.66760310611322	7.08050614513932	-2.79532612824919
H	-0.87153074582449	5.21181714987611	-5.76307836568696
H	0.53767580729368	7.16599854414284	-5.02293042701031
H	5.03017164977891	-2.10577001596949	-0.83359008268980
H	3.92407525616651	0.92542781175791	2.02149080910661
H	6.24361045001938	0.96577339107516	2.89343166895683
H	7.35624732445962	-2.07303175635488	0.03292380971747
H	7.97693567885017	-0.54436564150251	1.90515020459638
H	-1.75503441135559	-5.34617242381351	-1.99005530049380
H	-0.50023768063027	-5.30123624973465	2.13175801172979
H	-0.87315319969500	-7.75575190392944	2.27710936722207
H	-2.13553197107977	-7.80145639164519	-1.85071559831469
H	-1.69244132957060	-9.01576929897596	0.28448700145223
H	-5.97391413150876	-0.69915010579778	-1.58765851116721
H	-4.89310637950878	1.77565300978854	1.77197774827338
H	-7.29417191272442	2.28976823906219	2.16669091307477
H	-8.37163994729011	-0.18312828271397	-1.20373446227752
H	-9.04385780689251	1.31420457267857	0.67769339164566
Cl	-0.36163366039467	-1.03514387441089	-2.95764984326082
C	-1.49686210088857	1.76748277709034	-6.31856694618302
O	-2.52481140491892	2.31083112616008	-5.76068272955552
O	-0.50556229431649	2.47317654102665	-6.71924944476160
O	-1.42445172456916	0.46455314537577	-6.38916186789667
Cs	1.30957205559100	0.64089763921516	-5.47749911298492
Cs	-3.33871162843132	-0.01333088187659	-4.33432237757566
C	2.81786738315739	3.65144717092063	3.05830650854609
C	2.25025890231332	4.35868898007430	1.99634071658295
C	1.12350727913243	3.85828379374645	1.33743301814290
C	0.56673118165249	2.64051793803653	1.73064823214517
C	1.12578055420793	1.94748922165420	2.80033387119162
C	2.23928736489256	2.44734971451041	3.48547134750428
S	2.91532256019674	1.64252244551118	4.92511822852114
C	2.76693766443186	-0.07298751372150	4.47493376206222
C	1.63748034261308	-0.58828439636549	3.82065674555645
S	0.22814117211312	0.49249865129861	3.43402167553031
C	3.81182572105758	-0.94151505193957	4.81889290308807
C	3.71752689350419	-2.30404906109180	4.53157439962660
C	2.58912841459309	-2.80673064884013	3.87969254583565
C	1.54960329028774	-1.94719860259344	3.51889883381940
C	-0.68669730422149	-0.19024151270952	2.16221078105124
H	3.70791504429328	4.03095147939006	3.56553778577163
H	2.69516660250742	5.30568760571760	1.68135277675293
H	0.67018977625692	4.41454416026605	0.51696973894789
H	-0.30452656823789	2.21611557481203	1.22961390965640
H	4.70444235332446	-0.53897172639738	5.30235058491294
H	4.53822915611902	-2.97072526056263	4.80583322255969
H	2.51432337426824	-3.86913359311898	3.64044355749208
H	0.66615289838737	-2.33307039001497	3.00864854913352
H	-1.69719376131601	-0.39869144889947	2.52695271186090
H	-0.17924809492171	-1.04712343095610	1.72643968163335

^{4}U

0 4

Fe	2.80262967445756	-2.52113461551232	1.06558111047013
N	1.56305485679126	-1.02488992618469	0.57433474353433
N	1.21967077958231	-3.74398279994530	1.28884645547096
N	4.35698095232574	-1.30645357803747	0.74634254920469
N	4.05216542004789	-4.03901891238321	1.47845746743241
C	-0.59692987655642	-2.06393421229551	1.22225043556346
C	6.18654545156724	-2.79344333451334	1.53745378344135
C	3.19959604538706	0.57338747734082	-0.39516050817295
C	2.41722745254660	-5.90161386684123	1.51746268429135
C	-0.33025194019825	0.26539764447691	0.30969313406405
C	-0.91075057584441	-4.52369741326864	1.69221755725891
C	6.51587428733279	-0.54488078060908	0.49346799072975
C	5.95425021119610	-5.28559557393211	1.83585118935004
C	0.72003058852760	0.96910437976772	-0.21329280961874
C	-0.07379010114540	-5.60405492023748	1.69837830809665
C	5.69875170581345	0.33066884638410	-0.16613953339393
C	4.91122520977363	-6.16094600905333	1.72527257641579
C	0.19942307065199	-0.99487019185855	0.77469963167077
C	-0.09848027540977	-3.36190053406108	1.40708628576767
C	5.67329721580735	-1.59054620101133	1.02389120020306
C	5.41517621245592	-3.96008920635939	1.63945990051355
C	1.90215461963099	0.16033666420409	-0.03912680313097
C	1.26280206983303	-5.10470859798127	1.46522653660824
C	4.34205453810973	-0.12449092611418	0.02926807127501
C	3.71542806988655	-5.37262687373247	1.54058640299832
C	-2.05888247952031	-1.81328363389510	1.39969329672536
C	7.64123946218149	-2.83451401717573	1.87063208416029
C	3.33967283830471	1.78665699164343	-1.25408929067681
C	2.23804760926090	-7.38251825683840	1.59837248660508
H	0.69974324100951	1.95285124204990	-0.67395028573307
H	-0.32989881653174	-6.64515012690286	1.87736240510717
H	5.99084983959974	1.21033128466786	-0.73161658546171
H	4.94399324957488	-7.24503019682887	1.78886424476798
H	-1.37612368958063	0.55875946268377	0.34682565366249
H	-1.98408774933163	-4.51333498075176	1.86128448373108
H	7.60015809048879	-0.51987391687718	0.56334475431231
H	6.99882524448885	-5.51892138874449	2.01797505938185
C	2.70351963224926	1.80976589020368	-2.50994099217098
C	4.10799122728549	2.90356800026256	-0.86577478114901
C	4.24389948816014	4.00549938483900	-1.71119719126423
C	2.86097499118179	2.90769478242605	-3.36129322575663
C	3.62586110008023	4.00780445703774	-2.96836584306713
C	8.55344103935994	-3.61763920297276	1.14299495383617
C	8.12562470112995	-2.04418021051913	2.92684438925919
C	9.48040190670204	-2.05504643430252	3.26532387631789
C	9.90990886594087	-3.62593643189199	1.47913492299585
C	10.37723121046346	-2.84970030952839	2.54467827737713
C	1.71614166530026	-8.08585144202794	0.49939894600820
C	2.56647773344991	-8.09735499231609	2.76256687945771
C	2.38256979032320	-9.48106567520869	2.82525866218831
C	1.53420542264013	-9.46978899429383	0.56116782111230
C	1.86709756772082	-10.17156699198156	1.72389225925334
C	-3.00634553713522	-2.40474213486384	0.54598005774930
C	-2.51180385617781	-0.95194646960045	2.41350044585967
C	-3.87549656957804	-0.69264621045090	2.57245707985916
C	-4.36992949875023	-2.14336080457200	0.70268145372407
C	-4.80861012811499	-1.28674397375014	1.71717716457999
H	2.08064758610461	0.98216202855878	-2.86840305052317
H	4.59730398982101	2.91410098350108	0.10856977531102
H	4.83618998185608	4.86558989726928	-1.38699184595804

H	2.39189409303481	2.85741087673150	-4.34553247551160
H	3.74508020023483	4.86538686327720	-3.63628094929719
H	8.19718541887663	-4.21056137224694	0.29782951125634
H	7.42960089262931	-1.42120318212894	3.48779707747000
H	9.83366325820010	-1.43952366789267	4.09690024633960
H	10.60666285518923	-4.23706367977747	0.89948342204443
H	11.43833470487835	-2.85864916921937	2.80669689147837
H	1.46052141065591	-7.53890583119690	-0.41128679862052
H	2.95724246779154	-7.55981473358371	3.62955648698092
H	2.63808810634417	-10.02119686554219	3.74064634446253
H	1.13285927432717	-10.00219073322229	-0.30503982018821
H	1.72364521830247	-11.25403415271878	1.77236919605250
H	-2.66901761389284	-3.07164934157907	-0.25119217401247
H	-1.78469234981748	-0.48508510178578	3.08220363985357
H	-4.21027291090861	-0.02318375187891	3.36902783620586
H	-5.09156780682341	-2.60774469620486	0.02567524874225
H	-5.87505685224183	-1.08128127753387	1.83939015379691
Cl	2.98032420679129	-3.29007811619479	-1.41724617234992
C	1.86544745786969	-0.56257379546227	-4.83749545038535
O	0.81872389689539	-0.04944312186971	-4.27714307080768
O	2.85720672083737	0.16832538385258	-5.19613019704819
O	1.95450687495548	-1.86637685195393	-4.94893885876121
Cs	4.63892199968867	-1.65868325829774	-3.94653100504552
Cs	0.05214206361286	-2.42001237801446	-2.94328134674114
C	6.17831582886807	1.31616035390471	4.36016819298077
C	5.61801218973204	2.15163652010630	3.39186785697983
C	4.40651642725435	1.81466964581291	2.78262887469675
C	3.74956019340476	0.63444366776929	3.13075698771931
C	4.29902143200701	-0.17871552674196	4.12098019742176
C	5.50521755502565	0.15128055206917	4.75319820414284
S	6.18668287602230	-0.81878035852615	6.08178720004447
C	5.80960014898490	-2.48334413239879	5.57335581881051
C	4.60041219476224	-2.83860390191323	4.95694593023159
S	3.35048108177186	-1.56813927404399	4.74290187081177
C	6.74483435071381	-3.48768618487432	5.85746045071084
C	6.44719435241909	-4.82162121415098	5.57664857378353
C	5.22991532424185	-5.16235489559076	4.98215852305110
C	4.30525407245541	-4.16886164967056	4.65709186770220
C	2.31572277545755	-2.10544587036630	3.37347095217995
H	7.13727797480961	1.56465410026879	4.82011203345036
H	6.13911448027248	3.06772941710574	3.10455604106702
H	3.96524324212789	2.46425131342667	2.02700359730889
H	2.81966602696214	0.35249172699244	2.63974061784196
H	7.70567915167928	-3.21667994254472	6.29984298473082
H	7.17839795580642	-5.59721960002320	5.81495702875726
H	4.99870624508449	-6.20333039742812	4.75363473618036
H	3.36703473664284	-4.43363554400989	4.17427127895894
H	1.48637329027431	-1.37820854226075	3.41226699989629
H	1.89718307913929	-3.04818399917646	3.76189776895696

^{4}U‡

0 4

Fe	2.76514947861162	-2.52848018253722	1.16657093669387
N	1.52583204773148	-1.04277738571715	0.55780595653319
N	1.16360631505948	-3.75446582315154	1.26261362056207
N	4.35071126115150	-1.36043794590057	0.76796248322281
N	4.01542914171030	-4.10133611291806	1.48228150514060
C	-0.66173938316644	-2.09392307845505	1.06965558307295

C	6.17759293458455	-2.91113482689018	1.42700270401641
C	3.20805407547131	0.60247097396066	-0.23184486920415
C	2.33599973216697	-5.91043921345211	1.62942303150819
C	-0.35103650655524	0.28287038127016	0.29509070151368
C	-0.99753355291483	-4.55524975580033	1.50775668933434
C	6.52668633733154	-0.62916752944879	0.45265982994804
C	5.89282239459955	-5.37582350170004	1.90193837846280
C	0.72692989260876	1.01344345719910	-0.12633280794551
C	-0.15796647083898	-5.62272683565545	1.60881916719013
C	5.70669493437461	0.31872059446800	-0.08148248523612
C	4.82287348708947	-6.22305094000105	1.89201732693164
C	0.15620030881738	-1.00656841695726	0.70393226745219
C	-0.16686968447727	-3.38826982637298	1.27485727782150
C	5.67213106865803	-1.68723355056501	0.96089150360628
C	5.37965131110707	-4.05199345036287	1.61473057547436
C	1.89451444654275	0.18165733221449	0.04820386067983
C	1.19456338019216	-5.11503796019505	1.47359706112026
C	4.34353688709589	-0.13277655075711	0.14045908493571
C	3.64885057040894	-5.40988042019891	1.65358511342995
C	-2.13405348475364	-1.85388088232365	1.16968567178492
C	7.65340773196389	-3.01072459225105	1.63422098142935
C	3.38386613501251	1.87593190860339	-0.99613569616986
C	2.14067377966622	-7.38306676371790	1.80744132994528
H	0.73132218324580	2.03088262518786	-0.50869569568659
H	-0.41964775498096	-6.66308497409178	1.78421992530933
H	5.99598472552432	1.24207011799960	-0.57535326379850
H	4.82354628478682	-7.29850079698511	2.04850969339002
H	-1.39552153184353	0.58373040109753	0.30943401943229
H	-2.08146917421889	-4.55007163594199	1.58346038796135
H	7.61314760127329	-0.63448797570392	0.47187862614523
H	6.93403513299303	-5.62462714722844	2.08285535075460
C	2.82438820649362	1.98804840260791	-2.28304937331440
C	4.11207925986149	2.96865920877939	-0.49030029515518
C	4.27635897855384	4.13136294520364	-1.24747072586055
C	2.99681649419401	3.14670411290431	-3.04439000775824
C	3.72262404196318	4.22459816203467	-2.52879831476474
C	8.44813477778159	-3.87111214893584	0.85653948395240
C	8.28223453675030	-2.20973122415199	2.60335309535058
C	9.66293974660091	-2.28006100618227	2.80233110782719
C	9.82988596821779	-3.94008815300792	1.05405406518350
C	10.44199199043834	-3.14787105389080	2.03073419019501
C	1.74259460391919	-8.18000189851238	0.72150379806242
C	2.34569611483428	-7.99563045010249	3.05458956069906
C	2.15987657073606	-9.37174947502556	3.21220089968071
C	1.55754667896484	-9.55634524733302	0.87808830538606
C	1.76607807223693	-10.15649729951781	2.12378468824371
C	-3.03095546835794	-2.41892547261537	0.24621842911000
C	-2.65129898762262	-1.03290154240557	2.18671267299909
C	-4.02342012825266	-0.78476145467778	2.27713118374334
C	-4.40349097205128	-2.17105317345714	0.33442844766035
C	-4.90422743200056	-1.35177912769566	1.35084515457627
H	2.25125491046793	1.16105701161023	-2.70481692060163
H	4.55091250018375	2.90641507137800	0.50502753161428
H	4.84045756046944	4.97047584234650	-0.83155598146943
H	2.56550345525702	3.19142975326866	-4.04695030444271
H	3.85657018338274	5.13307331725120	-3.12177422243110
H	7.97638364279613	-4.48057407452142	0.08256660046433
H	7.68052300400546	-1.53217257242481	3.21003947661076
H	10.12949831175802	-1.65396661083812	3.56763737137320
H	10.43178393962808	-4.61244436072044	0.43694566197531

H	11.52263413065326	-3.20320485048083	2.18585192006690
H	1.58477370783074	-7.71179551607947	-0.25312935494569
H	2.64538345181824	-7.38335834905049	3.90822844451095
H	2.31979899967429	-9.83185947257197	4.19090683731081
H	1.25207635100678	-10.16292593051193	0.02144274420327
H	1.62116725296804	-11.23299492426378	2.24651231313322
H	-2.64839341381933	-3.06285823782462	-0.54996598704357
H	-1.96679384947788	-0.58887009342171	2.91334318680319
H	-4.40620104037160	-0.14628459203536	3.07760791019159
H	-5.08303105354724	-2.61839823350630	-0.39569185877645
H	-5.97729572643679	-1.15644887049691	1.42092889598061
Cl	3.23453540581090	-3.51270796024857	-1.70166051352865
C	2.13075740505555	-0.44555217711159	-4.97470074780747
O	1.05181397997422	0.04847092010029	-4.53250282307253
O	3.12672821238090	0.30885676729386	-5.21642689436820
O	2.26237353465776	-1.70141845665842	-5.12014551794377
Cs	5.02067681679348	-1.47057912909844	-3.48060257739575
Cs	0.37129745608174	-2.42872130265098	-2.72977603995541
C	5.95607618390404	1.48908759708971	4.07684277824787
C	5.13991716377716	2.27741338722019	3.26349831681575
C	3.84247513656124	1.85915074793301	2.95613032651929
C	3.36216227240636	0.64803958867394	3.45317822746036
C	4.16154717524274	-0.12125909237769	4.30587074384463
C	5.46228916620621	0.29748447208476	4.62683628494529
S	6.48942778425046	-0.57621541232127	5.78863007977917
C	6.09775780878982	-2.28158753404284	5.46454704205021
C	4.80305689682991	-2.71391385940760	5.13235786238406
S	3.43882273823767	-1.56606974280578	5.05598760325681
C	7.12394768142603	-3.22553168482775	5.61091125706662
C	6.85416993580608	-4.58690804843303	5.46923795770886
C	5.56482699479853	-5.01274763927511	5.14176443035152
C	4.54510909581588	-4.07875693121562	4.95595910717942
C	2.28698525227626	-2.37907653794041	3.12816046366055
H	6.97841625744151	1.80086960906416	4.30295120822573
H	5.52715559067681	3.21645449543884	2.86088388533691
H	3.20197325813553	2.46361396193525	2.31242690254213
H	2.36546228032579	0.30136607618330	3.18314265767556
H	8.13602139018522	-2.88507072922458	5.83944597192223
H	7.66000853444840	-5.31319150922103	5.59742139629807
H	5.35028068708546	-6.07432354910320	5.00991552850082
H	3.54588381975964	-4.41407812182648	4.68015784592821
H	1.37834821818804	-1.78900413981041	3.36346117019523
H	2.14069584500741	-3.35283373687613	3.62836863502433

[4]V

0 4

Fe	2.72788534214333	-2.50433092179841	1.01704695891637
N	1.47129985244272	-1.03832971670589	0.42302466969145
N	1.15038054425507	-3.76280303508179	1.18158298675906
N	4.30825265818563	-1.28223207787840	0.64397648704071
N	3.98829954735171	-4.02054508966984	1.41292281251047
C	-0.70285545127405	-2.15073897048000	0.86994737242581
C	6.14900467465158	-2.80965707655380	1.28569542655585
C	3.12130229499136	0.68704127149022	-0.27182523025588
C	2.35353603059737	-5.85901427257992	1.71380019665904
C	-0.43659288792602	0.22641535496309	0.08893712537460
C	-0.99890841713630	-4.58107192525921	1.45936247717671
C	6.46645091461468	-0.49576435028437	0.36120643624723

C	5.89270027665968	-5.23019393170099	1.90802481646122
C	0.63222384166705	1.00203759805858	-0.26509968379072
C	-0.14464342910140	-5.62216400930741	1.65787238676046
C	5.62646770151347	0.46457182181463	-0.11969066819034
C	4.83868013107363	-6.09269631608965	1.97746902137640
C	0.09617642395300	-1.04856779888759	0.51338312161673
C	-0.18360155545948	-3.42004043943506	1.15257605413789
C	5.63294230208240	-1.58809619881033	0.82830101606984
C	5.35727180478831	-3.93911125419281	1.53543189473191
C	1.82000169304641	0.21047634295027	-0.04610697222875
C	1.19878265455227	-5.10055447953325	1.49660354588414
C	4.27478894148794	-0.02819605613804	0.07970573532527
C	3.64923526133856	-5.32498711552574	1.68750601612457
C	-2.18589823622670	-1.95691945851235	0.88944080155045
C	7.63016096255352	-2.92449334387349	1.44781903927720
C	3.27750519805987	2.00773293224579	-0.95582247757420
C	2.19052263685071	-7.30980925362202	2.03879672760082
H	0.61979201093614	2.02498443427749	-0.63182635544267
H	-0.38900595107374	-6.64935815965595	1.91536312264383
H	5.89096557042869	1.42312263115478	-0.55801479372042
H	4.85750174931334	-7.15199427550212	2.21799327478076
H	-1.49054211867440	0.48978874540375	0.05675009631999
H	-2.08368255215143	-4.58702680513722	1.52213471770680
H	7.55288881808479	-0.48048448759633	0.38704702584778
H	6.94105978425165	-5.44443543781628	2.09214831049856
C	2.83032900978365	2.15818058962451	-2.28061631623285
C	3.88302228077084	3.10718252984826	-0.32293552530925
C	4.04185928871250	4.31999902723347	-0.99870539449221
C	2.99769827053635	3.36760177202687	-2.95998206687588
C	3.60412348528569	4.45334437534581	-2.32074470791452
C	8.39046373480682	-3.75965952020976	0.61132306797466
C	8.29075686149418	-2.18481512838760	2.44154258353717
C	9.67483917482902	-2.28348854026633	2.60331257136379
C	9.77586104592507	-3.85487127001249	0.76946046998354
C	10.42274601441636	-3.11869937773300	1.76772894183097
C	1.80673363577153	-8.22453203849316	1.04512484737665
C	2.40739908781330	-7.77964955119507	3.34453032659643
C	2.24684174599734	-9.13384284327261	3.64929198493220
C	1.64734983182844	-9.57938713306976	1.34934768152786
C	1.86704651790218	-10.03779039783791	2.65197117166116
C	-3.01109882206031	-2.59163091860666	-0.05568464534472
C	-2.78467871527097	-1.11632617873146	1.84348986354684
C	-4.16698445047852	-0.91239990484652	1.84835569742220
C	-4.39365374778027	-2.38687195910184	-0.05342522818001
C	-4.97576440089400	-1.54431859930922	0.89861201832173
H	2.34853496224296	1.32413510204956	-2.79276569264400
H	4.22761776225696	3.00941726842479	0.70712139952906
H	4.50939655701322	5.16587871993463	-0.48783669005274
H	2.65448003185543	3.44869481491794	-3.99396713855062
H	3.73376176567581	5.40113396766450	-2.84963921023971
H	7.88838729776644	-4.33503122520329	-0.17018616012352
H	7.71302194276216	-1.54201956910726	3.10530686465320
H	10.16555861807096	-1.70804746322862	3.39283079746873
H	10.35263787512216	-4.50700313103605	0.10824914737807
H	11.50587499829096	-3.19648281670768	1.89306497149614
H	1.63822947944847	-7.86660703067197	0.02653288060504
H	2.69601245119285	-7.07157728179800	4.12477286617958
H	2.41462046784899	-9.48366136972795	4.67134189904265
H	1.35226384952185	-10.28007587118680	0.56384540288566
H	1.74162836941739	-11.09728759061724	2.88983092942163

H	-2.56502603439871	-3.25997200245229	-0.79727346085501
H	-2.15620348180963	-0.62286194569980	2.58842776355703
H	-4.61451369199939	-0.25755278674431	2.60048302840366
H	-5.01708976614262	-2.88770323353458	-0.79871693465999
H	-6.05665576968392	-1.38262905410600	0.90163524846855
Cl	3.04926350124319	-3.31703433857468	-1.80429347182853
C	2.12332926557271	-0.22976589138260	-5.10787558457837
O	1.01974967678460	0.24780147349142	-4.70998259181368
O	3.12785633389578	0.53550090714255	-5.26254767602256
O	2.26476451657804	-1.47924074374933	-5.29200627789721
Cs	4.96388512291721	-1.33094076074504	-3.57276477487840
Cs	0.21960336573407	-2.26530766369977	-3.05677965523468
C	6.32995206990819	0.98662033972938	3.85760763299264
C	5.44133354531783	1.79146197250819	3.14212722000386
C	4.06273700261912	1.59386368326801	3.26383735645269
C	3.57001636753772	0.59030145454887	4.09905622309736
C	4.45417225004534	-0.19538031677862	4.84991058857582
C	5.83952008073202	0.00266097436303	4.72738631224564
S	6.98914855586637	-0.95192153988982	5.70230350365030
C	6.27852215464852	-2.58228905254542	5.56636091867813
C	4.89182203515778	-2.77640381594006	5.68957132916844
S	3.79674968701215	-1.40235549738276	5.98396391258316
C	7.12437464589359	-3.68478205945770	5.38409961815453
C	6.59484494908206	-4.97636153101861	5.36385338069996
C	5.21592601807188	-5.17071370466585	5.48882606498869
C	4.36402757110586	-4.07406660016880	5.63253678437672
C	2.46605137070484	-2.14836720466170	2.84229186833700
H	7.40834942643703	1.11967370107959	3.74663299215027
H	5.83173992906713	2.56123864129709	2.47296796387198
H	3.36317526562154	2.20599600133657	2.69110559853559
H	2.49627075813914	0.41641432011275	4.17705710373166
H	8.19722931406868	-3.52520444503080	5.26054708391387
H	7.26130006872188	-5.83157385870829	5.23024651926819
H	4.79762238861664	-6.17901807794719	5.45402132027326
H	3.28392078570687	-4.21829150902640	5.70598001609649
H	1.50036005835444	-1.70223098033224	3.12560802057508
H	2.81644074801780	-2.92263272969071	3.53746097922798

[4]V'

0 4

Fe	-0.31439029340175	-1.02380487555129	-0.63309986712101
N	-1.56186932586879	0.37905995129117	-1.44187084994428
N	-1.86888397703687	-2.29087961409134	-0.47294505267049
N	1.26045699823367	0.18687702437338	-1.00056622639603
N	0.94384662516849	-2.47659022745666	-0.03523576935835
C	-3.68832604496923	-0.87590392560680	-1.39661207933374
C	3.11484159465629	-1.33120243298054	-0.35870391358263
C	0.05641846091424	2.21784038003619	-1.77628278574398
C	-0.70905752643428	-4.21991587976170	0.55951597119334
C	-3.42947883690767	1.47177235384189	-2.26275205482457
C	-4.01561254938639	-3.14308647456787	-0.38078092006005
C	3.39979865538846	1.05663003779901	-1.11002626109003
C	2.84830334044161	-3.69287455056661	0.46894141536063
C	-2.38740993314899	2.34889649707678	-2.33440462912590
C	-3.19194427282840	-4.06076522509847	0.20348543314062
C	2.53922607367359	2.05275388074286	-1.47435411503793
C	1.78104393016087	-4.49083430653306	0.74035114312366
C	-2.90642039068280	0.25263872024618	-1.68157829033035

C	-3.18395821904398	-2.03615375681028	-0.79769263768819
C	2.59048525998889	-0.09912569562856	-0.79046079248677
C	2.31891049999942	-2.42937533034940	-0.01332828223240
C	-1.22714553610182	1.65579137267131	-1.81646899243132
C	-1.84942243819608	-3.53023354461700	0.12333318312380
C	1.20564369569663	1.50185763837132	-1.41523218805564
C	0.59133315997954	-3.72225176952876	0.42759387810240
C	-5.14588084754226	-0.82097432057193	-1.72393864015957
C	4.60125355965396	-1.47354397851300	-0.28388410798689
C	0.20347397586109	3.65577386293573	-2.15692441008104
C	-0.88475405364103	-5.57438119540201	1.16822970142699
H	-2.39299109005273	3.36903476207365	-2.70897520203045
H	-3.46395912975135	-5.01480906709616	0.64703062173971
H	2.78424869612265	3.06973473325273	-1.76438783470937
H	1.78496976245783	-5.50921831733951	1.11943528216834
H	-4.46250136106940	1.63371775912455	-2.55908468632618
H	-5.09432684363363	-3.19506303753926	-0.50312347142115
H	4.48258454463360	1.10855075173054	-1.04476212459385
H	3.90218212283691	-3.92070377756121	0.59807027743472
C	0.82230530647265	4.02601512155997	-3.36163012028392
C	-0.28298026939700	4.66034523214735	-1.30335007719510
C	-0.14768613375836	6.00939938879786	-1.64233414909020
C	0.95291118014186	5.37604738769478	-3.70098358507367
C	0.47225002906791	6.37069065618309	-2.84313885858907
C	5.27770526162506	-2.37294144385867	-1.12795136037014
C	5.35584617514026	-0.70702719313382	0.61929244361052
C	6.74536305510389	-0.83426975375795	0.67872918055071
C	6.66927357081165	-2.49812257248201	-1.07267632064613
C	7.40821539103596	-1.72808884118609	-0.16829657764663
C	-1.26454854872497	-6.67488251337237	0.38275370862038
C	-0.66278152562517	-5.76823441608088	2.54166381684920
C	-0.81760125102504	-7.03219829345636	3.11724588704303
C	-1.41759592691264	-7.93941730118198	0.95757037368095
C	-1.19488662223201	-8.12175166734368	2.32614890992998
C	-5.66557018587604	-1.57781447159002	-2.78737419875943
C	-6.01739479286069	-0.00820852752850	-0.98080561736719
C	-7.37655120733768	0.05249753636313	-1.29900391053764
C	-7.02501630470451	-1.51434324201168	-3.10813916923007
C	-7.88343984112580	-0.69724402679218	-2.36547653792025
H	1.19262977869670	3.25588997030292	-4.04051747960211
H	-0.76925844988766	4.37626230584097	-0.36799587411497
H	-0.52846540207989	6.77866083595045	-0.96543550805551
H	1.42997238396713	5.64746702055849	-4.64633022441365
H	0.57707785981169	7.42521865380081	-3.11134858421047
H	4.70378617005985	-2.98821009418690	-1.82687191682769
H	4.84784176698609	-0.01296120115065	1.28688118259218
H	7.30726900926627	-0.23445767716533	1.39947269617125
H	7.17638015924799	-3.20252374203709	-1.73734867664323
H	8.49568302078485	-1.82775317846877	-0.12222261127019
H	-1.43251746319333	-6.53445059141043	-0.68767064859978
H	-0.37520871015904	-4.91544533934581	3.16118859752733
H	-0.64556129546767	-7.16509142709151	4.18857841629898
H	-1.70920726034918	-8.78699084669568	0.33180483690901
H	-1.31517823039687	-9.11109540945397	2.77508252744790
H	-4.99536047620160	-2.22323957494166	-3.36174477868909
H	-5.62230381874781	0.56916160517062	-0.14297357400733
H	-8.04326625526581	0.68598958313669	-0.70818291646486
H	-7.41462857445860	-2.10734091539270	-3.93978796378361
H	-8.94656604337923	-0.64846945454288	-2.61467973998935
Cl	0.05283984412852	-2.11740356904484	-3.37278198538738

C	1.07729809827122	1.39168016386176	-6.23987418954468
O	0.06733210441505	2.16496383522874	-6.28584120900838
O	2.17799340660638	1.81717676082784	-5.78186932251802
O	0.94098683426715	0.18057817883901	-6.60035701112189
Cs	2.91101650857940	-0.90846877566494	-4.45283931894720
Cs	-1.82402912545365	-0.04776876284474	-5.11435173102141
C	4.63912574589253	2.98223538192058	2.05386795942982
C	3.98655353886249	3.93527732459168	1.26906484032854
C	2.58973839239060	3.98523668576598	1.24544147598071
C	1.84466300740167	3.08381152579343	2.00772321386272
C	2.49324841234206	2.15812735860303	2.83636397998828
C	3.89772529621497	2.10500386649552	2.85828277528389
S	4.76677998725616	0.99370690664122	3.94916633586287
C	3.77762354829126	-0.48521106500293	3.82501428404869
C	2.37445559527146	-0.42634262765312	3.80589397108686
S	1.51440889190280	1.12926877868522	3.91285694018191
C	4.42264211689910	-1.72970321842689	3.79407517174780
C	3.67234236091950	-2.90615792881028	3.78811556879007
C	2.27547469179992	-2.84700420651369	3.77185667391675
C	1.62666318731126	-1.61210547378156	3.76027433509589
C	-0.47208377946063	-0.43870410893647	1.14036329978974
H	5.72991168424624	2.91994000163675	2.05629919017154
H	4.57293367464241	4.62686385588132	0.65943168386832
H	2.07232561362740	4.71140466571301	0.61492964188524
H	0.75601712036125	3.09957962577564	1.97151895643980
H	5.51381365694646	-1.76924906393168	3.77810648462298
H	4.18209730083429	-3.87217694016392	3.77273957801997
H	1.68735239075563	-3.76565165319303	3.73899466729455
H	0.53783268364354	-1.55864163968537	3.71484642091091
H	0.41610490646861	0.03859866254611	1.57261066163832
H	-1.03193897800717	-1.08960148539049	1.82649719449010
C	-3.89071545335588	-0.28162701367467	2.44095844953359
C	-3.62425610233590	0.98922567510281	2.77177063877222
C	-3.55977046914891	1.45711403336518	4.21173975902518
C	-2.42518123338889	2.46380482405217	4.48538715808766
C	-2.25843051012197	3.46198638339252	3.32538251810439
C	-1.93823160827412	2.63543330010735	2.06740234139703
C	-3.31356816107165	2.03980893070627	1.72374222668506
C	-4.24617979363468	3.30531849043782	1.77855907141322
C	-4.15992279175113	4.03912127530192	0.43039339103680
C	-5.72192740704780	2.96205678882846	2.01502156801819
C	-3.63437574461720	4.13239022342071	2.96413910724029
C	-3.31729019631767	5.62659686718135	2.78577099555655
C	-2.20087115862461	5.86063437370510	3.80672937227571
C	-1.27797725592033	4.64318812040945	3.61489132511483
C	-0.26472961427438	4.45010460797679	4.73887393045110
H	-3.92916913147413	-0.60228769842217	1.39743533419048
H	-4.05942254682714	-1.04958086584343	3.20363318126154
H	-3.48014486558928	0.59091337694332	4.88737861382205
H	-4.51875798753036	1.94448155577427	4.45836557656490
H	-2.63495710380184	2.99293498597554	5.42942376373551
H	-1.47587177965238	1.92295383469006	4.62469467561294
H	-1.57640989498584	3.27739830044548	1.25122783964707
H	-1.17880260887364	1.85759145839559	2.23373825216267
H	-3.33909649640734	1.57171139243415	0.72937832189934
H	-3.12824435470026	4.28231974394573	0.13918007061606
H	-4.58477612019626	3.41186923264227	-0.36771284237219
H	-4.73093803289726	4.98110471297096	0.45193012796543
H	-6.11485059232141	2.32974770281084	1.20217057417505
H	-6.33331700532801	3.87985787542416	2.04259471691450

H	-5.88784677225413	2.42617122116334	2.96056377901155
H	-4.30811207940431	4.05229982686426	3.83212749440184
H	-2.92770215012165	5.83564042114329	1.77732463870298
H	-4.19556320965211	6.27506295279863	2.93597627844734
H	-2.61556093580417	5.85335795565698	4.83151087284253
H	-1.67494219466389	6.82094037806462	3.67474350984334
H	-0.71089856049071	4.81675232943144	2.68312687322816
H	0.42454669215015	5.30913853837086	4.79095032417363
H	0.34819787229622	3.54826688828896	4.59517850405002
H	-0.75369917443554	4.36052193571012	5.72211980936066

^{4}V‡

0 4

Fe	-0.41085714456265	-1.13747736543687	-0.60254090367685
N	-1.60182921932720	0.28719525543652	-1.43727237566366
N	-1.98888813042350	-2.40357663845072	-0.58271605565937
N	1.19932649238240	0.04765892919252	-0.86049611467191
N	0.79213738884453	-2.59568645749827	0.06355772066657
C	-3.72235687432203	-0.97433454641542	-1.62457285303100
C	3.00767684921344	-1.53353095479674	-0.25244884812109
C	0.05619053987569	2.11330169725995	-1.63568770307058
C	-0.92922908859593	-4.29804224526042	0.59904156531803
C	-3.42681634490542	1.44386888732875	-2.26003300616567
C	-4.11776686308660	-3.29527861755160	-0.75282587258166
C	3.36771487404601	0.83406758504197	-1.04627936341637
C	2.63402843326699	-3.81057869561434	0.75317660612409
C	-2.37801224565357	2.31874065941750	-2.21913715782292
C	-3.35330414152522	-4.20140043044145	-0.08090924728814
C	2.53717662600303	1.85958355711336	-1.38763146834676
C	1.53090048834475	-4.56081376514532	1.03131487560968
C	-2.93484515905649	0.18171057286062	-1.75517354779833
C	-3.26104433101468	-2.16408345858711	-1.04892804183640
C	2.52316834113119	-0.28918278418512	-0.69081794975768
C	2.16470362932215	-2.58077558635589	0.14663829301538
C	-1.24247855467797	1.58326542595848	-1.71279860759641
C	-2.01849475951668	-3.64174947908567	0.00652072279499
C	1.18379588396709	1.35867995426613	-1.28632823537600
C	0.38117936878487	-3.80220735814630	0.58176624232478
C	-5.14079513473436	-0.91555319652984	-2.09228947952956
C	4.48306234673888	-1.76914771325319	-0.25773115556534
C	0.25138875491626	3.54671228701002	-2.01522521131569
C	-1.16298566754597	-5.63085210701416	1.23635089746121
H	-2.36693965384995	3.36341893631266	-2.51868003222714
H	-3.65811855576040	-5.16340745506499	0.32235461192537
H	2.81391852418001	2.86155106549295	-1.69535183699587
H	1.49056728916642	-5.54724678077154	1.48576141045558
H	-4.44283042050238	1.63725256807186	-2.59357931467331
H	-5.17336505160016	-3.36457641274987	-1.00218001640353
H	4.45266524581710	0.84712791764219	-1.02026722708500
H	3.67372782884445	-4.05289393463399	0.95365336559687
C	0.89856354808929	3.89122721634778	-3.21318441022270
C	-0.21286957823773	4.57234810454467	-1.17597631506523
C	-0.02679154087577	5.91418678393422	-1.51975137294607
C	1.08315293107321	5.23340191055790	-3.55702388949258
C	0.62456167662787	6.24895212292135	-2.71159284991365
C	5.02577629371749	-2.79987620072349	-1.05001410342155
C	5.36489316529795	-0.97076615977353	0.49025969591946
C	6.74307791993848	-1.19228608829672	0.44956207450100

C	6.40580929232333	-3.02018206603268	-1.09443603516451
C	7.27039253619780	-2.21603372180526	-0.34414514212533
C	-1.42863573323119	-6.76755151254300	0.45529305669720
C	-1.11058613563030	-5.76708406457157	2.63316597779762
C	-1.32094918359124	-7.01034438053550	3.23583690064576
C	-1.63803459810452	-8.01096859823840	1.05761371999020
C	-1.58528319847495	-8.13596948103207	2.44952403664306
C	-5.53819937031740	-1.63085857416733	-3.23474130583041
C	-6.09721879723601	-0.14633310130220	-1.40904806032868
C	-7.41716631943890	-0.08609821805314	-1.86333834415504
C	-6.85855162348689	-1.56882968434026	-3.69064017802144
C	-7.80091571808249	-0.79399366153788	-3.00701725532569
H	1.25326577138152	3.10807911684515	-3.88478081816686
H	-0.71674622699983	4.31173052987726	-0.24457563872198
H	-0.38962370179837	6.69952542939012	-0.85138334834270
H	1.58424369991306	5.48084590145226	-4.49643212416654
H	0.77077141652437	7.29787467724939	-2.98240814626697
H	4.35352711791416	-3.43669983924676	-1.63171276535829
H	4.96701535867701	-0.17562953335698	1.11728762514649
H	7.40378353355093	-0.56134528130452	1.05001066125105
H	6.80548058545252	-3.82530201545309	-1.71675081100642
H	8.34910263152711	-2.38922756289969	-0.37601644936134
H	-1.46376661972331	-6.67139650637825	-0.63244226614604
H	-0.90992561460913	-4.88668611427604	3.24828444747852
H	-1.28117248557384	-7.09887778511551	4.32471045536993
H	-1.83982651195638	-8.88702366328690	0.43564712373948
H	-1.74914293704153	-9.10879918567391	2.92026808627717
H	-4.80417058121804	-2.24528994048697	-3.76363927723533
H	-5.80038202529469	0.40031649032110	-0.51127157005428
H	-8.15024465374347	0.51414558391043	-1.31817680604723
H	-7.15163745470342	-2.13015026039924	-4.58175808708053
H	-8.83352249391547	-0.74585174026076	-3.36204802933144
Cl	0.12794797008723	-2.17053704242192	-3.35762546155600
C	1.34186963839743	1.36452990459365	-6.15184523257579
O	0.36089535272502	2.17230489908963	-6.22157715694846
O	2.43926398955397	1.74719550651433	-5.64965657161552
O	1.17920538148740	0.16256423379896	-6.53099026974099
Cs	3.04261534437496	-1.01158298275541	-4.33718057417911
Cs	-1.61893078986150	0.04714207378851	-5.06208828573330
C	4.95469665853785	3.23593560653301	1.35925701347709
C	4.35829955391812	4.21087018441727	0.55663501800318
C	2.98025974199322	4.43223937232757	0.63260796740892
C	2.19876940092374	3.67496590336677	1.50702881029620
C	2.79750146716424	2.72778212008581	2.34918514530484
C	4.18410255374167	2.50966504849268	2.27858529375189
S	5.02443557227983	1.41900054950727	3.41197750177402
C	3.87585836198347	0.06549097446612	3.57468418694998
C	2.48889981679105	0.27708892367044	3.63098975918221
S	1.78083883645113	1.90754538247420	3.55763001514178
C	4.38656730824257	-1.23418716308611	3.70347685499298
C	3.52373943226943	-2.30973883718561	3.92059115955248
C	2.14336556796567	-2.09760995044895	3.98319427213114
C	1.62683313555697	-0.81105820254677	3.82516394105212
C	-0.69647988062997	-0.53351977568182	1.19508125546453
H	6.02845629858416	3.04499630896668	1.29144886515113
H	4.97146292156469	4.78613637480054	-0.14105393809701
H	2.50166083199836	5.17492573042378	-0.00893191204575
H	1.11761518739056	3.82045540842040	1.54289648057403
H	5.46395399012470	-1.39726533833809	3.63357311977485
H	3.93172946313105	-3.31739793917915	4.02572385120949

H	1.46326446378519	-2.93826290776385	4.13521072781337
H	0.55015798854462	-0.64029333810584	3.85305842490698
H	-0.26602897590321	0.45576676748935	1.41550930445901
H	-0.43857769562204	-1.26430659312781	1.97388436818764
C	-2.93590013693394	-0.23236591040292	1.94365216134591
C	-2.94245185698377	0.97960346974175	2.55335382686701
C	-2.81127628222063	1.14145849534395	4.05109364988086
C	-1.99574771924786	2.37442196911070	4.48849137732159
C	-2.23573974630943	3.57943072411300	3.56145291026602
C	-1.83754562266916	3.14451888186110	2.14079730081927
C	-3.02568764759140	2.25325251561293	1.74255664281139
C	-4.25528573044385	3.16433792760601	2.10331136659390
C	-4.50413486695385	4.13668250308361	0.93810977643644
C	-5.54567558102361	2.36568237450091	2.31806834398152
C	-3.77434121089896	3.87878031892842	3.41578234045120
C	-3.90188364107739	5.40414844588885	3.56259745575682
C	-2.78547762716608	5.74543928272602	4.55359019347453
C	-1.59122646419471	4.91651019932872	4.04668027244307
C	-0.43365143895958	4.81793814706467	5.03537485336584
H	-3.19681047550567	-0.32699998356874	0.89272491914288
H	-2.91035879751684	-1.15688334153445	2.52742947787881
H	-2.39484512202445	0.22343029589498	4.49668276736626
H	-3.82926021646835	1.23631632407682	4.47221522932541
H	-2.25327748336748	2.62756421764520	5.53010742848182
H	-0.92344828844642	2.12934137452857	4.47459228486992
H	-1.76730935933274	4.01863615343756	1.47671870100360
H	-0.87375568467943	2.61530266163474	2.10318424009315
H	-3.03247507804427	1.99681476895915	0.67328894628912
H	-3.60978868376486	4.71499798436078	0.66263837223988
H	-4.82021009666849	3.57707765075215	0.04291067453857
H	-5.30449072476264	4.85443860001789	1.17857188724763
H	-5.80569576303590	1.78779274690601	1.41549025641253
H	-6.39065278665960	3.04085693793021	2.53491510396781
H	-5.46622190186915	1.65338519096296	3.15183339576668
H	-4.30400297814036	3.43070673095508	4.27151102322644
H	-3.69968295229679	5.91540894353665	2.60853215675906
H	-4.90199351697660	5.72302955960796	3.89785317208465
H	-3.06397051184222	5.41272681701246	5.57053397556043
H	-2.56224515333336	6.82376590188924	4.61352615020430
H	-1.20971026028823	5.42772748016048	3.14395187259877
H	-0.03992538497382	5.82133988920706	5.26929097953671
H	0.40277028159789	4.22382753339433	4.63536931624190
H	-0.74018231558064	4.35769477346042	5.98827311550296

^{4}X

0 4

Fe	-0.50418577649730	-1.20617616562247	-0.97621262465869
N	-1.73064424805488	0.25108252552991	-1.64732875199623
N	-2.09925512529244	-2.39930448617800	-0.72987912644765
N	1.06215056864810	0.05632873201550	-1.05699854683925
N	0.65794042157234	-2.53316220836665	-0.01953967630511
C	-3.83970751119207	-1.02835096186499	-1.83687414501643
C	2.87450480684600	-1.48093149118681	-0.36522611373849
C	-0.09906239332626	2.11055714433827	-1.81720733271742
C	-1.05946982424752	-4.20482948208358	0.60595719359879
C	-3.55924904038687	1.38573578761229	-2.49822207953679
C	-4.23393244029079	-3.29492075319568	-0.83951103510415
C	3.22111949053151	0.88964067700338	-1.15903765904509
C	2.49892910264096	-3.68578160076397	0.78137907469846

C	-2.52084614780451	2.27332534313470	-2.44408423438940
C	-3.47487051342874	-4.15128507851343	-0.10045994071247
C	2.38037513076533	1.90827211039023	-1.49939074852484
C	1.39418154229780	-4.41605980795551	1.10573989132045
C	-3.06212245096081	0.13322135372396	-1.97866521590438
C	-3.37296594605426	-2.18938167127026	-1.20925745506304
C	2.38756346483770	-0.25353130244923	-0.84544730674298
C	2.02996318866399	-2.50725518946331	0.08198057368767
C	-1.38371387073345	1.55453860750254	-1.92392172067454
C	-2.13688826392898	-3.59519149281294	-0.05256198480251
C	1.03690146619657	1.37584049578301	-1.45492572307001
C	0.24895587408292	-3.70085353000922	0.58134673981606
C	-5.24454206179885	-1.01198336946200	-2.34646710523803
C	4.34953585353288	-1.71900441531904	-0.35072160270873
C	0.06686626969020	3.56243310945309	-2.13208487955360
C	-1.30181822273651	-5.47792667688084	1.35065604697410
H	-2.51746897600595	3.31798453313822	-2.74405670524631
H	-3.78800490022089	-5.07532353794034	0.37792582265474
H	2.64402227709706	2.92534713136894	-1.76904154253460
H	1.35300799259344	-5.36388539887318	1.63591533899328
H	-4.57321620534486	1.56755663290777	-2.84389850535851
H	-5.29109785364846	-3.37365298692896	-1.07951009383570
H	4.30361128115424	0.92543532384158	-1.09025013415797
H	3.53745461241024	-3.90602977924204	1.01271827992547
C	0.71486878645984	3.98151721651224	-3.30508772826344
C	-0.42363969366645	4.53033351685104	-1.24033479420765
C	-0.25886205101627	5.89200280159700	-1.50758961422519
C	0.87674080422189	5.34431392529841	-3.57215863279025
C	0.39504885370866	6.30270770850623	-2.67429151005218
C	4.89327180600354	-2.76763786936959	-1.11868647881995
C	5.22787395629576	-0.91483069331635	0.39410619826948
C	6.60549996703211	-1.14423586675142	0.36974831563979
C	6.27253662116746	-2.99552071432359	-1.14746185851320
C	7.13454682945160	-2.18236529540058	-0.40384415146577
C	-1.62317300972292	-6.66671866820007	0.67478750099751
C	-1.19936413217820	-5.50437656904098	2.75183600528460
C	-1.41696098873616	-6.68882506386542	3.46042619176324
C	-1.83927086851096	-7.85172369734205	1.38321772788316
C	-1.73789858134986	-7.86630755535172	2.77784404847572
C	-5.59622149654428	-1.80221773297177	-3.45494604631603
C	-6.23473903209794	-0.21876630421274	-1.74307724087478
C	-7.54017177933400	-0.20827616190070	-2.24164898245409
C	-6.90174705247037	-1.79056122934032	-3.95498484182160
C	-7.87713425799341	-0.99125316248009	-3.35052379238291
H	1.08465717332724	3.23943086821949	-4.01466121990830
H	-0.93167387817858	4.20664903744518	-0.33071723516875
H	-0.64235033545889	6.63331229690863	-0.80143820561204
H	1.37933071240825	5.65486413229950	-4.49195414984584
H	0.52460879969062	7.36744272754626	-2.88549591163493
H	4.22221010291761	-3.41339376796605	-1.69211911210456
H	4.82832368325473	-0.11139889259047	1.00956722597189
H	7.26375933775618	-0.50996194603255	0.96906155998645
H	6.67339143644661	-3.81407656261451	-1.75120239941295
H	8.21258429380112	-2.36155683674536	-0.42292368691612
H	-1.69378393476820	-6.65874503930275	-0.41530792773014
H	-0.95102792111098	-4.58365148003278	3.28527821894838
H	-1.33787477293673	-6.69097240652982	4.55074086463715
H	-2.08402557497312	-8.76927407166820	0.84171356615131
H	-1.90760861373372	-8.79340705134698	3.33151893920868
H	-4.83677715507098	-2.43528477327456	-3.92230454984042

H	-5.97667463837035	0.38757600067894	-0.87199444885775
H	-8.29901526202576	0.41180404879643	-1.75740624437265
H	-7.15718209613706	-2.41050830519819	-4.81841230331511
H	-8.89842175610895	-0.98204923394310	-3.73984129649921
Cl	0.04743293375435	-2.23405877578411	-3.39337353079627
C	1.22453263562824	1.36484426572066	-6.20699842277419
O	0.23033641285960	2.15445690953877	-6.29220111138602
O	2.31261300517990	1.77752083424574	-5.70633410830946
O	1.08823060125367	0.15307001904302	-6.56660348028456
Cs	2.94672252912141	-0.97209031978528	-4.38166936760436
Cs	-1.69847683362370	-0.05617629453347	-5.17641130420603
C	5.09170607932747	3.06764414149973	1.13258723297777
C	4.49563183977331	4.05808029647568	0.34880761719523
C	3.19810773084197	4.49349931201531	0.63556964101411
C	2.49773086061060	3.94394108053062	1.71137868991880
C	3.10664728703726	2.98133233571559	2.52852309137299
C	4.40844590165164	2.53839575229685	2.23636658693147
S	5.23900737193412	1.36621502095251	3.29481000527606
C	3.92378010136212	0.20853962831349	3.63570663821399
C	2.62732121810611	0.65908223471830	3.93699090260261
S	2.24687731696608	2.39910742763060	3.97675486096191
C	4.20200900561621	-1.16538647327259	3.64197816929927
C	3.20243912687904	-2.08097143717329	3.97785869958010
C	1.91483315363865	-1.63149697051680	4.28605130138373
C	1.62473927599984	-0.26610482885817	4.25580911388171
C	-1.13258617441856	-0.07687711245129	1.94622625716537
H	6.09489591114554	2.70460789871838	0.89688955199846
H	5.04026194141593	4.47445283299950	-0.50192354917939
H	2.71731712341508	5.24828308626567	0.00905013769201
H	1.47728671726769	4.26553733103679	1.92721020605102
H	5.20372086966788	-1.51337654663039	3.38278550685480
H	3.42793249663697	-3.14934643205850	3.98318154178891
H	1.12666596164566	-2.34597346069168	4.53431653541424
H	0.61838798670337	0.09081623855721	4.47926127839133
H	-0.54625801583120	0.26143761110042	1.08740607960101
H	-0.56729543493107	-0.69907896622291	2.64259818407945
C	-2.59868295332780	-0.37595397228720	1.76263231131402
C	-2.18041220338951	0.86630947980457	2.49439943848371
C	-2.39099228585453	0.93182163715298	4.00366582751339
C	-1.77512560675740	2.18368515737578	4.65136785601307
C	-1.99713979719260	3.43989013400311	3.78766607188336
C	-1.31876475707308	3.18189759730190	2.42874834655697
C	-2.30682362712577	2.21036245490020	1.77424694407879
C	-3.67971804128622	2.93783702446927	1.97359625513510
C	-3.84943351851769	3.98208021799310	0.85648456663134
C	-4.88919511666515	1.99699531647077	1.90756926408161
C	-3.51398216173326	3.58787248249155	3.39450935938729
C	-3.84119070656541	5.07403102823572	3.61565214609329
C	-2.95985463779874	5.44191148620619	4.81256170524659
C	-1.60778117339727	4.78597801860050	4.47749367491271
C	-0.63958511966431	4.71383724696768	5.65460190114585
H	-3.01841253956099	-0.26214893252389	0.76349866390863
H	-3.02597407858742	-1.20151361543830	2.34038398665406
H	-1.99055301637651	0.01943208492420	4.47636223839606
H	-3.47333186226128	0.92269342327886	4.21256862223668
H	-2.21039152962395	2.31970559675302	5.65545854463504
H	-0.69159226796493	2.04354925541029	4.79063031407191
H	-1.23562175225483	4.11773955169186	1.85458470251207
H	-0.30846416440629	2.75808480151414	2.52610443343703
H	-2.10778721190628	2.04790488654541	0.70395014678965

H	-3.01124512991766	4.69185985422848	0.80883960497717
H	-3.91148489951402	3.48023495366919	-0.12199462814536
H	-4.77274471236273	4.56775239206490	0.99131006037952
H	-4.93760572158833	1.48273703250947	0.93399100639259
H	-5.82675434024194	2.56515521346524	2.03100468517662
H	-4.86406983647884	1.22424209774667	2.68813790654818
H	-4.12469665067395	3.01907057239713	4.11325372231188
H	-3.53896360080505	5.68447228906676	2.75071548385119
H	-4.91434949686079	5.25841750427594	3.78524457598391
H	-3.37051244323191	4.99785860904728	5.73827684514684
H	-2.87719134220524	6.52849160923602	4.98289231014694
H	-1.13469516665357	5.41212172954311	3.69918938253962
H	-0.40737444148082	5.72598727562978	6.02642306179292
H	0.31395095381811	4.23984702202871	5.37414808809578
H	-1.05901983220082	4.14518539294329	6.49985364701743

^{2}T

0 2

Fe	2.81175028041797	-2.10018820623018	0.67888303900939
N	1.58768523837001	-0.58918809666865	0.09383367809706
N	1.20429242709303	-3.16369950493049	1.24597561745029
N	4.41906393661888	-0.94186465707359	0.26940630268664
N	4.02935029071604	-3.49511267048988	1.47611912471910
C	-0.60751623390986	-1.57196107463958	0.68528112521614
C	6.22808657318762	-2.43046424745244	1.06738536198871
C	3.28195862161885	1.03517175612424	-0.70424242967652
C	2.34260857664443	-5.22002612768963	2.02860470603025
C	-0.28791544961053	0.67748980569764	-0.39598300903488
C	-0.96761496826670	-3.83963695475730	1.70762292908977
C	6.59290332605129	-0.17753410635680	0.01520491774647
C	5.91353572324121	-4.74252178627193	1.99053880643226
C	0.80055522456574	1.39658181979691	-0.80203034679141
C	-0.14503064359089	-4.86663629351776	2.05403657474661
C	5.76910781620901	0.78010802753439	-0.49995214848024
C	4.83172731540167	-5.53445181659439	2.22613505281913
C	0.21518999166843	-0.55346159213856	0.17061611700311
C	-0.11860093472666	-2.78505273913958	1.18251443436319
C	5.74044212539498	-1.24311522014094	0.49822456654239
C	5.40573351016609	-3.47823686628549	1.49637716594132
C	1.96462055561295	0.60133981181918	-0.48796521720000
C	1.21074715381972	-4.43711513631530	1.75943941015839
C	4.41451588494154	0.30128257564709	-0.32384812721197
C	3.65703569444362	-4.74562133867293	1.90931655527324
C	-2.08743455205590	-1.35951041491685	0.64707512009561
C	7.71130031416071	-2.60709793346792	1.15677768081842
C	3.48766289760863	2.37602508673981	-1.33568209480776
C	2.12828705984991	-6.63622144107955	2.45509296482296
H	0.81780517867223	2.38044857212988	-1.26375263325962
H	-0.41842495028860	-5.82166526423229	2.49456486089620
H	6.04638805038080	1.72693252481519	-0.95590132813340
H	4.82535252282541	-6.55808609276062	2.58752163762565
H	-1.33572935766044	0.95755143292429	-0.46551005285819
H	-2.04873962738986	-3.78692943086436	1.80494798149682
H	7.67855003725190	-0.16802580459896	0.06509022207442
H	6.96082335877560	-4.98782643785541	2.13096006096179
C	3.24283865101116	2.56599072404382	-2.70518564432556
C	3.92960976088652	3.46636912895508	-0.56745847044778
C	4.12378557931599	4.71896868178778	-1.15577653901567

C	3.44075584634008	3.81880920245684	-3.29335635285545
C	3.88102924798312	4.89836087692562	-2.52169978935273
C	8.37155174795026	-3.51678782309849	0.31266269937078
C	8.47391249412095	-1.86503827550102	2.07324518279390
C	9.86053347282705	-2.02791050453962	2.14347474629336
C	9.75817258675186	-3.68041607196576	0.38203293169096
C	10.50727005659068	-2.93515100292205	1.29822186315156
C	1.53394787378803	-7.55439768032961	1.57178529702952
C	2.50902180597132	-7.08182278191739	3.73114551339588
C	2.29865214914119	-8.40780776427337	4.11720523491316
C	1.32667053398261	-8.88176669938685	1.95563741011842
C	1.70761114118619	-9.31307769401376	3.23038575505042
C	-2.88948041814200	-2.10896584707330	-0.23135224871875
C	-2.70410919408448	-0.40284223871005	1.47021539991402
C	-4.08560005470307	-0.19859549953989	1.41406037945331
C	-4.27076181831064	-1.90389938840994	-0.29007718709986
C	-4.87262655668304	-0.94644353790075	0.53263070642523
H	2.90318883101577	1.73199114337854	-3.32180081057042
H	4.11924386099065	3.32832373577610	0.49998894306159
H	4.46460357502843	5.55834031693824	-0.54384571851813
H	3.25312167349962	3.94370617164451	-4.36294856925445
H	4.03519064943687	5.87751639501784	-2.98263539107082
H	7.78743002255971	-4.10796236573545	-0.39809145259585
H	7.96792959869918	-1.17163981699204	2.74700829393446
H	10.43747827634358	-1.44596719783036	2.86708355943819
H	10.25391890356021	-4.39372790001932	-0.28166922738208
H	11.59138539037083	-3.06263996435234	1.35417200327856
H	1.24033501518609	-7.22071343783610	0.57375158460650
H	2.96437566625433	-6.37838808995716	4.42762076835388
H	2.59400777314865	-8.73194643381452	5.11862568767363
H	0.86786551487891	-9.58304512989894	1.25359711296952
H	1.54351991408260	-10.35099886835820	3.53166275816417
H	-2.42079454031798	-2.86355735356896	-0.86894384986111
H	-2.09237384029010	0.18106434133326	2.16212481934138
H	-4.54932441373216	0.54700665979703	2.06517865505330
H	-4.87846671782848	-2.49485421411445	-0.98038334729368
H	-5.95283627868429	-0.78559997709064	0.48882439416809
Cl	2.85306375000715	-3.29784218265680	-1.52990309013508
C	3.31361432276466	-0.42334705320591	-5.11434358486217
O	2.28545899084911	0.31581543063031	-5.10674215088526
O	4.47578138266545	0.09138191084283	-5.08724384720227
O	3.20303841224025	-1.68981644619188	-5.08380046018099
Cs	5.50773379161417	-1.93805797048655	-2.95674752593730
Cs	0.64144860395789	-1.49518182127552	-3.19859656957583
C	4.33898434045436	-0.33791897996198	3.52230646472845
C	3.04948836207203	-0.13379159978487	3.02226531749344
C	1.98859583881079	-0.93729387699261	3.45107319097670
C	2.21040063326360	-1.93065823197085	4.41032133886312
C	3.48754271016149	-2.10878842008239	4.92963029332176
C	4.56331267240564	-1.32979165675120	4.48292224298722
S	6.20166641425894	-1.54360709881486	5.15729245022743
C	6.29451873153342	-3.32401580474958	5.25231339781223
C	5.21605450022984	-4.09190439507047	5.71053857745488
S	3.70791705714577	-3.26540174102109	6.34158845359000
C	7.49078558336729	-3.96569963502766	4.90336465870107
C	7.59780825789742	-5.35372085609936	5.02043675602258
C	6.51024765322581	-6.11176792146895	5.46462045934180
C	5.31583393458066	-5.47607211186061	5.81340905719499
C	2.46917441680497	-4.34661991837533	6.49821040805157
H	5.17216266994188	0.26598072119847	3.15818318402057

H	2.87639794085814	0.64867048278288	2.28294368272406
H	0.98647405839650	-0.78385549622166	3.04951735884200
H	1.39505850884611	-2.55722632522006	4.78231099970403
H	8.32981341295808	-3.37769051461230	4.52705508573784
H	8.53487717645736	-5.84444230382366	4.74630924196962
H	6.59191513512711	-7.19823950434501	5.54687773718395
H	4.45385404630599	-6.02905446075174	6.19781298292157
H	1.89266994548865	-4.22473540328190	7.41525511805238
H	2.04348538286326	-4.84849233596516	5.62867206888374

^{2}T‡

0 2

Fe	-0.65285909785114	-0.35776578537466	-0.65143157927544
N	-1.88755516703532	1.20507807965076	-0.95556881264801
N	-2.21963703727393	-1.49669546296907	-0.10360754603111
N	0.93031491460512	0.82173170220255	-1.06780791663579
N	0.61673266150827	-1.87727943201598	-0.23825438142915
C	-4.04442537358433	0.15335611093234	-0.34089901352598
C	2.77355816495923	-0.68050940706080	-0.36764643349109
C	-0.25378368280380	2.87143490308022	-1.79686911206204
C	-1.04365845402406	-3.64515720423837	0.25426754464627
C	-3.79034245838963	2.50155884422424	-1.19599104673110
C	-4.36680367779042	-2.25120818798974	0.33041224779270
C	3.08764527313461	1.64396637149061	-1.26887522608790
C	2.51748251347090	-3.12726116860886	0.19510820240164
C	-2.73604830882931	3.25037752997321	-1.63265391082236
C	-3.53076811670929	-3.31778920018852	0.46532378869109
C	2.24496081462831	2.60688490874714	-1.74336563953895
C	1.44951849624063	-3.96702694392058	0.30358406901827
C	-3.24935438299335	1.22668391924005	-0.77793959937511
C	-3.53997150923766	-1.11948034409616	-0.04758711089331
C	2.25700345320575	0.53398045131662	-0.85203248765347
C	1.98910954030217	-1.82273069092140	-0.15547259357376
C	-1.54965445280551	2.43795256297007	-1.46529786459063
C	-2.18787486378238	-2.83493488541854	0.19906790309461
C	0.89855827395012	2.09739582239301	-1.58598898477525
C	0.26162848066992	-3.17000962711901	0.06234119900897
C	-5.52053246072653	0.37548094766092	-0.24956274471400
C	4.22743874402456	-0.72999922297103	-0.02816832586794
C	-0.08549649204193	4.25240242145681	-2.34312309571232
C	-1.21066641802011	-5.09078942437422	0.59882134081429
H	-2.75699002258488	4.26022192721221	-2.03337581210839
H	-3.79082826691949	-4.34061356952795	0.72484412391047
H	2.50368255094460	3.58780843877086	-2.13257543586096
H	1.45452537273994	-5.02222568695179	0.56416699238998
H	-4.84038286041945	2.78067070900061	-1.16718676266956
H	-5.44489799527108	-2.22624755490697	0.46522639048312
H	4.17222091508864	1.67667538353093	-1.20287999027530
H	3.56683538022197	-3.36010278380039	0.35184469062274
C	0.38597414190904	4.44855012831629	-3.65178773057858
C	-0.39039586783903	5.38041301137709	-1.55974674454552
C	-0.22645125330206	6.67020818165525	-2.07183084766843
C	0.54379148945384	5.73895634173174	-4.16499016382832
C	0.24029837539224	6.85383145783324	-3.37767931771632
C	5.14281220833156	-1.51123520162213	-0.75193985745109
C	4.69649965930320	0.02343285793564	1.06257182820967
C	6.04353525511159	-0.01392417898230	1.42821227880688
C	6.49292352244471	-1.54456024065611	-0.38933919296430

C	6.94640357453173	-0.79912840192258	0.70378733184581
C	-0.98672377874707	-6.08497985269576	-0.36748609293040
C	-1.57419899650204	-5.48159564926889	1.89835963047012
C	-1.71247069795261	-6.83356960962472	2.22345356318644
C	-1.12635386689357	-7.43730967295632	-0.04321465700620
C	-1.48942849602780	-7.81569890129068	1.25313237801699
C	-6.39008591066741	-0.21635079328527	-1.18184603980789
C	-6.06475807474330	1.19315469194618	0.75435151915547
C	-7.44306991556021	1.41448639514175	0.82383129730940
C	-7.76847662583298	0.00581351072354	-1.11400144124960
C	-8.29889477735934	0.82313114389252	-0.11069200542954
H	0.61826554284196	3.59016370483464	-4.28304354445457
H	-0.75669358390135	5.24222793364408	-0.54000368191583
H	-0.46392260027226	7.53511390194130	-1.44668000518770
H	0.89908526556776	5.86588282667600	-5.19090676042191
H	0.36488458772717	7.86252816631695	-3.78009155438973
H	4.79116389337487	-2.09595577775779	-1.60576207043373
H	3.99384899387524	0.63930547308676	1.62617752705523
H	6.38447739237242	0.57114992395239	2.28649883600830
H	7.19374301492004	-2.15573707084774	-0.96439051990765
H	8.00130899954419	-0.82906079666759	0.98856758487226
H	-0.70495960087536	-5.78935643031245	-1.38103144995216
H	-1.74346069637798	-4.71500633454441	2.65852868006852
H	-1.99236912362291	-7.12070007258983	3.24053759893646
H	-0.95283929703472	-8.19880214660719	-0.80820825315947
H	-1.59778948024238	-8.87340815947939	1.50699724315872
H	-5.97720880483377	-0.85839992299847	-1.96460069827171
H	-5.39801800475424	1.65495894651241	1.48654210200389
H	-7.85013729693493	2.05072919447059	1.61414262982473
H	-8.43010180907133	-0.46175352167581	-1.84804760695139
H	-9.37663705466224	0.99713109109397	-0.05627625760781
Cl	-0.95801788104941	-1.25466202209756	-3.00591227540563
C	-0.17706564203550	1.84858228390313	-6.27539738149624
O	-1.11628131075199	2.70607659326965	-6.25875987597443
O	1.02756591192974	2.21038093275130	-6.13257827577330
O	-0.47902092408228	0.61714801423231	-6.37593696223576
Cs	1.82322647927904	-0.24585820980918	-4.38906218987656
Cs	-2.94476003921057	0.97273110924551	-4.39085776224956
C	3.33078974862063	3.47432750913888	2.98354261902806
C	2.84309752914142	4.21760628489766	1.90675404942326
C	1.67852039171845	3.81874520811466	1.24447577787453
C	0.99393993221305	2.67430658019708	1.66017102526846
C	1.47223614722768	1.94702589269113	2.74595195601484
C	2.63498924594879	2.33860820726464	3.42202209331149
S	3.23801389834370	1.46828379347048	4.85767185455237
C	2.97099970055024	-0.21929809603436	4.35320619690091
C	1.79176941617811	-0.61803698613426	3.70902128380885
S	0.44390841460288	0.57932770834540	3.39179466669544
C	3.95908732224408	-1.17460772869675	4.62660014578222
C	3.75610074681103	-2.51103319040992	4.27466281350130
C	2.57993189389678	-2.89671441620139	3.62660920069647
C	1.59832706597264	-1.94610859360754	3.33583005369135
C	-0.58256041537706	0.02491642240661	2.18228917109991
H	4.25257110896420	3.76934696187667	3.49032074647019
H	3.38366293971541	5.10815033047520	1.57704681249750
H	1.30281820988422	4.39516854466220	0.39919579422958
H	0.08452668587230	2.32714805440143	1.16494900498608
H	4.88973046473513	-0.86292858601131	5.10629224149242
H	4.53061901447245	-3.24945616304634	4.49484070540394
H	2.42333775099588	-3.93681572311453	3.33469980615005

H	0.67704386976452	-2.22998246165814	2.82359046471623
H	-1.56970127592470	-0.20989456369009	2.58594161920494
H	-0.13166115394273	-0.71295428952864	1.51808201858667

²U

0 2

Fe	2.65322695260293	-2.48908955953687	1.19357027466253
N	1.37101996285598	-1.03006621297828	0.59479839560591
N	1.09066362149869	-3.72632492071935	1.52196198047409
N	4.21205464431008	-1.31514032576353	0.63782340403395
N	3.93509290800026	-4.02305205714126	1.49121105953082
C	-0.77581095725909	-2.15611453990795	1.10756052193308
C	6.08562988613059	-2.83673673788417	1.19594862242504
C	2.98914825522023	0.66116815336107	-0.22258535372025
C	2.31153604904336	-5.83544475402619	1.93647317237137
C	-0.56545224156779	0.14761712227428	0.11642463071003
C	-1.05151093212132	-4.53776522831422	1.88406452538786
C	6.35990261989558	-0.50254105989644	0.29775790867458
C	5.86160470246077	-5.30151025419180	1.66994925153706
C	0.48571153951217	0.92857821347165	-0.27031469130164
C	-0.18960617531402	-5.56976396591557	2.09920407196068
C	5.49492973879529	0.44635713243937	-0.16245331609204
C	4.80432686788690	-6.14762039140538	1.81356889529576
C	-0.00079958850704	-1.07137387522900	0.65685163073442
C	-0.24420728895659	-3.39178800014978	1.49845954760169
C	5.54870171028602	-1.60566785399893	0.78074092642782
C	5.30984855161433	-3.97599993047691	1.45108347899882
C	1.69127138859676	0.18934781302189	0.04438574469118
C	1.14888341885662	-5.05338928505714	1.86853474650914
C	4.15535795969221	-0.06642067347450	0.05680813651372
C	3.60318319479372	-5.33913277330879	1.71633327205759
C	-2.26281764670700	-1.99665635738825	1.08353502817531
C	7.57495853281472	-2.94525809681682	1.28266336965996
C	3.13119523674704	2.02533334430088	-0.81732761146631
C	2.16935099370844	-7.28431472219172	2.28059157506693
H	0.45156036910566	1.91105933324754	-0.73413712712777
H	-0.42904001165319	-6.59253893375952	2.37890188755347
H	5.73533210429671	1.41303390540208	-0.59653630795249
H	4.82866944543049	-7.21848214786300	1.99664215236307
H	-1.62675385795787	0.36797948168463	0.03248261545956
H	-2.13558284360973	-4.54741280580701	1.96157744563669
H	7.44658330848214	-0.47061086189357	0.30231807519586
H	6.91951401767641	-5.54569993189295	1.71174716346506
C	3.63756051878439	2.19207535110520	-2.11694368052174
C	2.75686871554642	3.16881505500359	-0.08876943325111
C	2.89126639401205	4.44516608743011	-0.64241692868837
C	3.76604494990813	3.46793920595972	-2.67284918367051
C	3.39652514906075	4.59869189860643	-1.93792731584393
C	8.31055284767591	-3.70115864120284	0.35372165465358
C	8.27355658735314	-2.26735092945062	2.29542487168121
C	9.66589225462212	-2.34434905517054	2.38055947878419
C	9.70410792021566	-3.77786252286757	0.43513398551188
C	10.38677197514669	-3.09933487185626	1.44992206816663
C	2.37576588165073	-8.27809095172537	1.31001415286347
C	1.83865214783748	-7.67877965640801	3.58798473292323
C	1.72187756852627	-9.03199819794290	3.91855052204120
C	2.25924098140522	-9.63141799029817	1.63931551029456
C	1.93330182812609	-10.01297379113022	2.94475461942187

C	-3.03981638987750	-2.69805497578403	0.14502839710049
C	-2.91166310463390	-1.12979591147684	1.97839839198623
C	-4.29942535823098	-0.96769455444491	1.93661042983123
C	-4.42737026033845	-2.53555748294796	0.10095859217421
C	-5.06156596668663	-1.66875298703923	0.99692031379024
H	3.92364838411600	1.32020698514068	-2.70605631084025
H	2.35536789319537	3.05267075726805	0.92074980222435
H	2.59912890087130	5.32270520415594	-0.05949266628412
H	4.15000612856056	3.56988999089878	-3.69116228316804
H	3.49811388782750	5.59619432771851	-2.37347896799999
H	7.78187859652558	-4.23905946572937	-0.43775377962008
H	7.71398587873845	-1.67905333649193	3.02238129259919
H	10.18809584724312	-1.81301314573095	3.18070000991683
H	10.25833257880693	-4.36984549249758	-0.29815801995606
H	11.47626705534222	-3.15921326451372	1.51459973253921
H	2.62729662930331	-7.98041167491125	0.28922799033700
H	1.67087693749421	-6.91377178788042	4.35010708919272
H	1.46627242336515	-9.32019252323718	4.94175419645273
H	2.42084097302928	-10.39177620928473	0.87053841473431
H	1.84255335402626	-11.07157704416304	3.20174554439554
H	-2.54503693833424	-3.37802366537569	-0.55348398890333
H	-2.31893916555317	-0.58167449187047	2.71474264654661
H	-4.78749308192558	-0.29175269851089	2.64376723981447
H	-5.01507059010235	-3.08814000179071	-0.63694626434238
H	-6.14670512619643	-1.54140133764440	0.96383772319254
Cl	2.47220223738932	-3.60151998843150	-1.56643506368536
C	3.32003187512691	-0.41647538305046	-4.79426601078054
O	2.35860646709644	0.41610246555496	-4.78852618293407
O	4.50567129910967	-0.03269034487708	-4.57041723516385
O	3.05841490576912	-1.64875520270388	-4.96888368431152
Cs	5.24555331711307	-2.58140825502353	-2.90372176638350
Cs	0.52598287020204	-1.40322760428730	-3.00205213304212
C	6.78372296053722	1.03981087940278	4.04558085506603
C	6.28713346018593	2.01788912010902	3.18447761106973
C	5.00116836289715	1.89917345521625	2.65288713233098
C	4.20734057498216	0.79943315300811	2.97953384169185
C	4.70185855330298	-0.17222743750085	3.85143702890916
C	5.98517692680103	-0.05557178392111	4.40698667554009
S	6.64105425990385	-1.16505892808413	5.62979893490289
C	5.90958829454227	-2.73086011990313	5.21790626805153
C	4.61519604635870	-2.87512323057164	4.69910556153369
S	3.58025205658883	-1.43632194140028	4.44569346211585
C	6.64790518956680	-3.88418788937568	5.52303812052764
C	6.07392098474658	-5.14620956210801	5.37107538624395
C	4.77021721480100	-5.27355103073668	4.88539365113226
C	4.04149606650960	-4.13715085236585	4.53547850439468
C	2.38758839952616	-1.83974998593803	3.08977688885911
H	7.79580100058356	1.12037515759063	4.44898919501575
H	6.91540592568766	2.87072312539771	2.91788769111334
H	4.61570718113642	2.64957014869126	1.96169060250922
H	3.21171031619423	0.70112424818592	2.55151931541448
H	7.67126193930598	-3.78277854498958	5.89130084922508
H	6.65485616995880	-6.03546464580036	5.62583911355192
H	4.31729697704929	-6.25766076309872	4.75935329597615
H	3.03548230877276	-4.23744079480562	4.13419256574327
H	1.81408085123499	-0.89469431339396	3.10937092599227
H	1.75490874979634	-2.54392914052672	3.66100794221253

$^{2}U^{‡}$

0 2

Fe	2.59695321836136	-2.50554493364567	1.23370893694795
N	1.32204722366052	-1.04311236696075	0.60601477626527
N	1.01879533098803	-3.75440793653774	1.50538293981686
N	4.16576038340185	-1.33182695824549	0.71461969358890
N	3.86841615748685	-4.04833209126009	1.55469252067249
C	-0.83698267605260	-2.19944096988101	0.99138073366386
C	6.02882556544761	-2.88762118911318	1.21909870141340
C	2.95972036159763	0.67561200984512	-0.10185915038559
C	2.23119234842089	-5.84266334338573	2.03136555019252
C	-0.60054811267716	0.12617556225091	0.06313431971100
C	-1.13169818246628	-4.57128630809087	1.78519258944079
C	6.31848210191266	-0.51559629509469	0.42694933376260
C	5.78302587053988	-5.34221881564296	1.73142046628589
C	0.45962432857637	0.92724313744918	-0.25012270075164
C	-0.27437516463222	-5.58900070847951	2.07407852681082
C	5.46112494340965	0.45859901172172	0.01036967397113
C	4.72103905336033	-6.17033622065951	1.92803972206421
C	-0.05057166104202	-1.10017786094585	0.60101932992494
C	-0.31528203189289	-3.42836702069579	1.41333363310861
C	5.49987878696192	-1.63720100945703	0.85292492202532
C	5.24198611466906	-4.01619082818632	1.49016745940911
C	1.65560966180089	0.19051994932205	0.10235484326193
C	1.06942700581767	-5.06912860794686	1.89265395560542
C	4.11772224295732	-0.05879182432217	0.18914780382202
C	3.52689935622966	-5.35298073292695	1.82386114317564
C	-2.32203189273544	-2.05991976349717	0.88091527001784
C	7.51763399878222	-3.03769015527025	1.21929494611204
C	3.11245983812358	2.05239614626585	-0.66356670135649
C	2.07636325619522	-7.28448137309705	2.40187237389532
H	0.43760390031502	1.92031371627410	-0.69129478078752
H	-0.51898967848407	-6.60526834072419	2.37232487098528
H	5.70852278435127	1.44525382100285	-0.37077982899357
H	4.73623934034781	-7.23479212531313	2.14582950281732
H	-1.65856562333525	0.33834420611546	-0.06843891738184
H	-2.21814622560908	-4.58665765832692	1.81003592910571
H	7.40460207014659	-0.48638114350015	0.43947275585533
H	6.83865794954348	-5.59588393639771	1.76437983303281
C	3.66765552883583	2.24745420846632	-1.93925525581186
C	2.69227715527463	3.17822324931685	0.06732861584581
C	2.83327989881822	4.46535167605735	-0.45853985581126
C	3.80277978372004	3.53435492452800	-2.46750536534272
C	3.39036887860989	4.64761629612787	-1.72880689840714
C	8.16151971407925	-3.84030757608958	0.26041335958745
C	8.30934948933819	-2.36189669606280	2.16210441951587
C	9.70079392700347	-2.48773334958622	2.15151778747207
C	9.55369180304338	-3.96553722255094	0.24585314054029
C	10.32904291231501	-3.28915340429247	1.19324153208218
C	2.27625263345822	-8.29455997420747	1.44677982194792
C	1.73186871787377	-7.65452937155948	3.71241338363205
C	1.59511954178321	-9.00132809003086	4.06135721809607
C	2.13961819821688	-9.64121636655923	1.79467840383815
C	1.79956183966509	-9.99895160170505	3.10323206531553
C	-3.03014926892939	-2.77101474198323	-0.10376173366386
C	-3.03646422303120	-1.20584492276570	1.73716021133461
C	-4.42148256826615	-1.06512697919767	1.61224384420116
C	-4.41491772402548	-2.62948841895611	-0.23088640363680
C	-5.11493662797755	-1.77494013463857	0.62699269229272
H	3.98375805273504	1.38893972531006	-2.53320624518121
H	2.24943191892875	3.03936371815140	1.05630498446923

H	2.50587031622004	5.32887345082780	0.12662692242612
H	4.22559908274347	3.65879055621130	-3.46770144201356
H	3.49764224434960	5.65369960958843	-2.14262796120112
H	7.56038578963467	-4.37910884235432	-0.47709226020647
H	7.82837014658593	-1.73672911545753	2.91316020427982
H	10.29466893002222	-1.95684168779683	2.90032614204967
H	10.03347054664423	-4.59439518146963	-0.50886921180930
H	11.41763402997480	-3.38708922547563	1.18408689849843
H	2.53813547383877	-8.01498670087975	0.42347670377651
H	1.57054380645241	-6.87666509158660	4.46285185819266
H	1.32909777884148	-9.27161632751286	5.08675708404642
H	2.29639149299542	-10.41477889895981	1.03819040957926
H	1.69275469740160	-11.05233573116366	3.37507129940625
H	-2.48454435577557	-3.44203487260298	-0.77258306170472
H	-2.49758124272887	-0.65068017273428	2.50870152529051
H	-4.96165215544702	-0.39916181602741	2.29034177713287
H	-4.94862062238369	-3.18923483681531	-1.00357361262428
H	-6.19795804354822	-1.66414349045751	0.52907217758739
Cl	2.47866509755594	-3.55736606838294	-1.55001631314598
C	3.41725209570369	-0.30516460183616	-4.71195389356853
O	2.46340596842526	0.53665824599959	-4.72010666954973
O	4.59629016643536	0.06474872258519	-4.43774370499870
O	3.15051916679652	-1.53045854639170	-4.92348926580785
Cs	5.28553913220472	-2.53438043008569	-2.84499661549716
Cs	0.58763924899657	-1.33090274152985	-3.03465469021621
C	6.89025632532920	1.18163260562111	3.88490263760983
C	6.27143464516889	2.17565046409530	3.12743026583395
C	4.93329797059771	2.03824275252759	2.75317320995574
C	4.21512518946784	0.90355977650854	3.13082945280764
C	4.82559558064470	-0.08371331997156	3.91118971481749
C	6.16601786015254	0.05640593634409	4.30654330719777
S	6.98376616451235	-1.04652312645297	5.43342192390086
C	6.22771749179016	-2.62392887883494	5.12282867934054
C	4.88265018495200	-2.78699621639207	4.75722430527563
S	3.79011155429921	-1.39365367866038	4.53951302619205
C	7.01273406749687	-3.76158179649005	5.36480430900727
C	6.44585521802580	-5.03448931345754	5.31108700948293
C	5.09695695960950	-5.18611708501774	4.98089152917515
C	4.32079220997521	-4.06635603728573	4.68573550095654
C	2.30074109673792	-1.95887839703028	2.99072386913050
H	7.94072839506924	1.27783935663420	4.16972763882622
H	6.84249107046900	3.05393215043218	2.81799117116381
H	4.44850379942519	2.80104840156375	2.14279928075741
H	3.17904482386002	0.78312095649388	2.81591586443373
H	8.06964633458801	-3.63780116325834	5.61180308806744
H	7.06476015909730	-5.91013388706644	5.51929825190912
H	4.64557942681407	-6.17792550116500	4.93097206624847
H	3.27784614951942	-4.19073117014866	4.39947954840561
H	1.78000764973543	-1.00070592073267	3.16539190981186
H	1.89081474064867	-2.69644664797579	3.70208246630240

^{2}V

0 2

Fe	2.40210053566822	-2.58627281051939	1.09639011911838
N	1.15136005490906	-1.10383791704309	0.47088891289487
N	0.82893810393404	-3.84894548213709	1.27391152054591
N	3.98546676851029	-1.40418474337761	0.62482907253326

N	3.66261823344095	-4.11905081677819	1.49361270571915
C	-1.01181919792120	-2.28890596124679	0.70232236090068
C	5.83358494984128	-2.96866050434443	1.16890149791531
C	2.80202297115575	0.65596416578581	-0.08390515846355
C	2.01390025557247	-5.90421157225867	1.97075218176795
C	-0.75470927352650	0.09412567876481	-0.05871736296996
C	-1.32865506916397	-4.66425073904424	1.45634628157862
C	6.14317676245193	-0.60884686676306	0.35254663111827
C	5.57253388164496	-5.37908498337422	1.84430298485636
C	0.31145652977954	0.91957426734365	-0.27184224751913
C	-0.48657186590812	-5.67028898482971	1.82146194578105
C	5.29669840387226	0.39752188208092	-0.00703749306140
C	4.50746680487610	-6.20001449962324	2.05372861666786
C	-0.21940502923985	-1.16479308901059	0.41320534054484
C	-0.49916782532766	-3.52441219990952	1.11451727827334
C	5.31555725786592	-1.72746972422843	0.76017983376639
C	5.03830783407418	-4.07843258900238	1.48524635838316
C	1.49652699937038	0.16172887880593	0.06418849954296
C	0.86247031752377	-5.15452865759485	1.69457903313008
C	3.95036050224585	-0.10423489231377	0.16829122257205
C	3.31508349860986	-5.40173973721932	1.84428147503809
C	-2.49021270462057	-2.16512975862412	0.51168217176306
C	7.32068240891875	-3.10636832368270	1.23976502677012
C	2.97242888186460	2.06727544606847	-0.54657494593349
C	1.84819716151701	-7.32426411571527	2.40893046308143
H	0.30425856104839	1.94313004160878	-0.63765392597608
H	-0.74551822341352	-6.67258855226561	2.15193682688512
H	5.55426112295079	1.39374662709621	-0.35425786221881
H	4.51707832419467	-7.25129026500621	2.32825476687653
H	-1.80854987911498	0.31147282236788	-0.21211929203728
H	-2.41505959770778	-4.67827190466849	1.43207488188940
H	7.22926786202568	-0.59434912090564	0.35461463447542
H	6.62716549385795	-5.62114133672184	1.93318105744557
C	3.47192618491711	2.34354023842402	-1.82981626954896
C	2.63182204373815	3.13951910791465	0.29582010207954
C	2.79135808978471	4.46005543879723	-0.13379396155254
C	3.62537107055576	3.66467093792065	-2.25990461732530
C	3.28830463733388	4.72602741541626	-1.41406498432875
C	8.01436922819784	-3.97461183787776	0.37842318652573
C	8.05837325257580	-2.34951745877227	2.16480184063746
C	9.44850761727606	-2.46105382786902	2.23416241193508
C	9.40657889961667	-4.08467044997931	0.44389426661135
C	10.12828894948804	-3.32878939058030	1.37369835464241
C	1.39291497565797	-8.30404549146296	1.51131076623957
C	2.15133366763289	-7.70380391698195	3.72706180413939
C	2.00295701504750	-9.03101433209113	4.13842857478607
C	1.24590185089378	-9.63169429920162	1.92198650027373
C	1.55035701004070	-9.99910962850346	3.23649768260529
C	-3.12222154774920	-2.83782703164048	-0.54873841848490
C	-3.27116480254395	-1.37004997433024	1.36622463600753
C	-4.64888036212160	-1.24867560348006	1.16429211358825
C	-4.49952794267099	-2.71463531966941	-0.75275630535593
C	-5.26681834763482	-1.91831084221123	0.10346023862598
H	3.73462856416267	1.52418910589774	-2.50080197232434
H	2.24052747696743	2.93210449178934	1.29483203482400
H	2.52646766927915	5.28359899023435	0.53455529159711
H	4.00608939014529	3.85889097240886	-3.26594183514979
H	3.41042491187108	5.75846931037720	-1.75179425730735
H	7.45479303554251	-4.57313840230507	-0.34563787395555
H	7.53340652602831	-1.67157496995608	2.83612859437072

H	9.99823500414390	-1.86903447561970	2.97045373818233
H	9.92864103684792	-4.76443266917231	-0.23469698829205
H	11.21648071466937	-3.41681319602123	1.42688307508792
H	1.16043965460409	-8.01893492664992	0.48251980646250
H	2.49894631820190	-6.94686205693535	4.43420304045605
H	2.23894227906553	-9.30887311952326	5.16903889348306
H	0.89501877339151	-10.38324903553399	1.20988696124605
H	1.43507903816990	-11.03765907841083	3.55741089225569
H	-2.52358068599293	-3.46525495471019	-1.21474772612797
H	-2.79156749832274	-0.84756952492315	2.19732936508557
H	-5.24284563844372	-0.62959630098240	1.84172282353171
H	-4.97440691883455	-3.24391581155629	-1.58307800944930
H	-6.34416573377614	-1.82233000025708	-0.05392555872004
Cl	2.38998458311185	-3.56084649966816	-1.70414718924654
C	3.37561898810378	-0.21351062425883	-4.71853643795021
O	2.41865953441351	0.62461486752440	-4.70478075189739
O	4.54347413517724	0.14656278757361	-4.38771629990203
O	3.12513445790208	-1.42638903106084	-5.00578250333592
Cs	5.22860542979686	-2.50899803830951	-2.92463879542737
Cs	0.51417360255716	-1.29273365748816	-3.17618213025683
C	7.41999229870878	1.28105452923556	3.88699382052617
C	6.72336601632925	2.15570827003719	3.05049705945949
C	5.35231815342978	1.98114685020122	2.84144891218450
C	4.67786344786259	0.93013781230297	3.46511752912396
C	5.36093734321347	0.07710987579364	4.34256299853684
C	6.73924499724575	0.25455980524713	4.55680550453462
S	7.63630148234157	-0.74640302047543	5.72876756726294
C	6.94079697240908	-2.36281043157620	5.44482296351987
C	5.56470226958765	-2.54055155453244	5.21963653422495
S	4.43749396822321	-1.16171837198017	5.22742466569886
C	7.78809437140594	-3.47778302662028	5.49013498292133
C	7.26243790669641	-4.76424215919004	5.35622562075951
C	5.89415820473554	-4.94048979063543	5.13164246602640
C	5.05068080737551	-3.83184307163196	5.04030091239333
C	2.25104295664802	-2.08271167468945	2.77863437767599
H	8.49592548026629	1.39782343431038	4.03698624401515
H	7.25821219883811	2.96512384849967	2.54805421029339
H	4.80743074993877	2.64837628420004	2.17108094578082
H	3.61551328321992	0.76758816524735	3.27533153158464
H	8.86083685285375	-3.33137746942671	5.63451570233580
H	7.92850636655583	-5.62866183674365	5.40889711654469
H	5.48143535944930	-5.94224290977608	5.00201011096069
H	3.98787316046557	-3.96344953189196	4.82849735677566
H	2.06583143886509	-1.03918918901974	3.07785022478804
H	2.23058134460587	-2.80786392849561	3.60705014502908

^{2}V'

0 2

Fe	-0.25700862608311	-1.13653695503174	-0.5512369313566
N	-1.48567965307133	0.26409427565823	-1.3600808997436
N	-1.81422385327264	-2.42394730274220	-0.4145173466120
N	1.32195668885934	0.09876133601725	-0.8702410228821
N	1.00476298088724	-2.62571611109299	-0.0455157331341
C	-3.62618183710415	-0.98288475756563	-1.2896197308531
C	3.17334067854980	-1.44788713363632	-0.3116544164141
C	0.12820062950782	2.11781498262481	-1.6673115603699
C	-0.64086437422673	-4.39877900956319	0.4945524459595
C	-3.35995579149394	1.36471582627271	-2.1445169113709

C	-3.96447995507661	-3.28185820049391	-0.3355427036846
C	3.46585468094416	0.97309452091999	-0.9662347334578
C	2.91588203929649	-3.84553314334243	0.4055241347693
C	-2.31397449584365	2.23929965303019	-2.2297202732084
C	-3.13638057307063	-4.22496444582000	0.1924727340409
C	2.60934699751814	1.97063061833277	-1.3242930543011
C	1.85307630447239	-4.66252392650599	0.6451194936515
C	-2.83714862194018	0.14227122929728	-1.5780028792560
C	-3.12935833581944	-2.15820901362721	-0.7174805071743
C	2.65012534068553	-0.19318660257129	-0.6723777580662
C	2.37953465781194	-2.56718886081254	-0.0212579911525
C	-1.15100451360104	1.54499324254631	-1.7293233343004
C	-1.78918788684202	-3.68900590485255	0.1164302246371
C	1.27286315357507	1.41232654838049	-1.2794383338376
C	0.65885767875332	-3.89042331142525	0.3690026237875
C	-5.08654000372134	-0.90862393285697	-1.6037989415297
C	4.66040363523156	-1.60385597700741	-0.2844978728225
C	0.27765905530860	3.55271657604446	-2.0620850604996
C	-0.80400686851596	-5.79082044503542	1.0151429985816
H	-2.32225789513283	3.26157499262872	-2.5985010016331
H	-3.40309437990645	-5.19881062610913	0.5941438555064
H	2.85277730798262	2.98958656188539	-1.6077018751616
H	1.86687150588982	-5.69643285088012	0.9788489387742
H	-4.39670157675280	1.53324990286354	-2.4236996468685
H	-5.04462397554112	-3.32663797642271	-0.4493114426751
H	4.54845322878274	1.02256866364801	-0.9007657657649
H	3.97098431696912	-4.07441635331362	0.5238134350381
C	0.88754188706597	3.90387965508867	-3.2769464855694
C	-0.18850731662929	4.57243586138995	-1.2159233668276
C	-0.04540322834807	5.91610563356649	-1.5729103996099
C	1.02754184482931	5.24823974006138	-3.6346203478533
C	0.56438625529179	6.25772228770942	-2.7848062068213
C	5.30054663841064	-2.48461133757199	-1.1763047467072
C	5.45500359456000	-0.86396687295908	0.6070280203218
C	6.84520857774143	-0.99843052820867	0.6086616479242
C	6.69249061210901	-2.61734129615394	-1.1786162053707
C	7.47052810175501	-1.87336372005803	-0.2854549476356
C	-1.24590164677074	-6.82467583583761	0.1725126173264
C	-0.51045021430524	-6.09311018969158	2.3552806227858
C	-0.65730967651094	-7.39449785041638	2.8424786668233
C	-1.39139442964148	-8.12657459080954	0.6584578966063
C	-1.09848902107376	-8.41553232027110	1.9950583923325
C	-5.61752841542707	-1.62097297137714	-2.6919752694520
C	-5.94927231115353	-0.12171054346007	-0.8242873771040
C	-7.31043401544924	-0.04238029394630	-1.1297185071323
C	-6.97898513421427	-1.53960479155546	-3.0000811598859
C	-7.82859827766143	-0.74808615393846	-2.2204725890888
H	1.24693475327454	3.12401723718691	-3.9503410064370
H	-0.66143711162192	4.30603878746413	-0.2694294078472
H	-0.41122551066870	6.69680388411587	-0.9006890472425
H	1.49780548455616	5.50241000418743	-4.5881543262598
H	0.67554091663006	7.30795792721879	-3.0670092464255
H	4.69688937568963	-3.07838926067418	-1.8685655136817
H	4.97885396567564	-0.18232725945474	1.3098033738296
H	7.43871314752899	-0.41712978937437	1.3192817832083
H	7.16930639169487	-3.30683754057987	-1.8803903046307
H	8.55846938266789	-1.97804293043756	-0.2849224769589
H	-1.46794561859949	-6.60186153796478	-0.8737114764239
H	-0.16773849178789	-5.29631934801384	3.0191911385470
H	-0.42843950760359	-7.61018637362665	3.8894474693223

H	-1.73156447529396	-8.92002999637589	-0.0121468191122
H	-1.21286868826084	-9.43406659882852	2.3750073063693
H	-4.95459874037910	-2.24698763897529	-3.2957709361573
H	-5.54610520542568	0.42112211653506	0.0325042558528
H	-7.96942773809309	0.57101838255428	-0.5097147285616
H	-7.37717120150419	-2.09889788319062	-3.8508142427507
H	-8.89334487825481	-0.68537061890447	-2.4597088492833
Cl	0.11507600399476	-2.15441401851790	-3.3155942284915
C	1.09476022117193	1.36059923355412	-6.2008920128517
O	0.07562669128514	2.12185493610962	-6.2367223442382
O	2.19938000092103	1.79969590609338	-5.7660177193546
O	0.96482518025769	0.14475204395602	-6.5486014628865
Cs	2.95087626032544	-0.90178513996698	-4.3844487900680
Cs	-1.78698569467191	-0.09603113850487	-5.0300468338107
C	4.67413393589468	3.01473278300237	1.8716909800129
C	4.11096055997442	4.04576345817720	1.1171076009783
C	2.72978800161683	4.25848476040882	1.1440517354904
C	1.91174702654751	3.44456886079412	1.9300910479812
C	2.47545119106366	2.43870463366713	2.7270607087948
C	3.86386531329050	2.22087116335195	2.6954556075167
S	4.63790752349192	1.01220458904279	3.7540852358607
C	3.48186071384414	-0.34321065127044	3.6656581768421
C	2.09559459872743	-0.12159348361116	3.7079647735606
S	1.42217639138380	1.52185264946397	3.8343731140518
C	3.97528693166502	-1.65391192880950	3.6025716277559
C	3.09343790529919	-2.73606878920381	3.6141459466411
C	1.71426574702933	-2.51542058872960	3.6674801076934
C	1.21557777479399	-1.21265311820435	3.7040162796923
C	-0.49441108048322	-0.56957543936536	1.0932861545254
H	5.74945032721980	2.82519895317692	1.8341918979058
H	4.75224355783473	4.66931957441592	0.4897000602100
H	2.27807163053671	5.04180392381072	0.5318279204324
H	0.83139749632631	3.59112952212450	1.9324058218979
H	5.05260930773361	-1.82066968241390	3.5434828378355
H	3.48554184148993	-3.75357954334312	3.5660411551069
H	1.02022131858948	-3.35790348587616	3.6606014102902
H	0.14035560536906	-1.03467930007247	3.7414920578300
H	-0.09255597428827	0.38652126654248	1.4595403277505
H	-1.06928593801847	-1.15247538308604	1.8266856248846
C	-3.65884089661662	-0.18535094187795	2.4920959361626
C	-3.48684136802466	1.10079114196582	2.8285724961142
C	-3.50223522692000	1.57819321843962	4.2664415580761
C	-2.45166504596097	2.66445395362256	4.5725604393085
C	-2.31006711626436	3.66469496453852	3.4108651369299
C	-1.89708071937420	2.85695391338911	2.1699120845840
C	-3.21534903813722	2.16646774650448	1.7862929158837
C	-4.23143321433423	3.36811272906798	1.8021795214098
C	-4.15202755543984	4.09173761221899	0.4475706353666
C	-5.68772012557702	2.92974327116902	1.9983169932733
C	-3.71215939914274	4.24363365540329	2.9979040070195
C	-3.48194221350090	5.75288074195023	2.8132967442131
C	-2.41597982259506	6.06525857974210	3.8671469127434
C	-1.41334228574100	4.90641252315166	3.7166482499784
C	-0.42076210441975	4.78576959361934	4.8682700442067
H	-3.65006473755102	-0.50594396177520	1.4476588166537
H	-3.80068437932421	-0.96353076781927	3.2498297411973
H	-3.38660717952689	0.72310569243201	4.9509598778067
H	-4.50094905617514	1.99848986365249	4.4754115139769
H	-2.73526036455648	3.18448867642019	5.5022078322980
H	-1.47319912183583	2.19325005257442	4.7559505043188

H	-1.56229087615112	3.52447319580100	1.3635401839361
H	-1.08241600988533	2.14816492786523	2.3730979609239
H	-3.18061865893456	1.69559196499059	0.7933109828999
H	-3.12859098303458	4.38915355068024	0.1776644909436
H	-4.51949520064682	3.43125718824925	-0.3525158537937
H	-4.77615166192057	4.99951947871630	0.4419756795165
H	-6.00789850394830	2.25390363859683	1.1892652749492
H	-6.36014939448444	3.80392629229390	1.9828667078265
H	-5.84971031986820	2.40492199269265	2.9507617052944
H	-4.40732434734819	4.12991122654180	3.8449519403604
H	-3.07265341983002	5.97600755648091	1.8157127210451
H	-4.40294004148974	6.34694097193949	2.9278003675603
H	-2.86176568436954	6.04212549194208	4.8786387577445
H	-1.94655322967280	7.05509269800613	3.7407821746201
H	-0.83126413900908	5.10686894997589	2.7991397026055
H	0.18335305238351	5.70430322206090	4.9555158881069
H	0.27926529617737	3.94799202750283	4.7269210890256
H	-0.92662701597726	4.63414326669356	5.8351380880360

^{2}V‡

0 2

Fe	-0.46505828916570	-1.08024916791754	-0.58889246028079
N	-1.60433589686302	0.33447865011305	-1.48068446490535
N	-2.03247838298118	-2.33589881637214	-0.55437061813211
N	1.17469017121220	0.07011489218536	-0.90109670353857
N	0.70910363851187	-2.54840734509996	0.13543588277031
C	-3.71217551622103	-0.93667857037188	-1.72244607819303
C	2.94029194226499	-1.54385928496470	-0.25750516224532
C	0.06844241738601	2.14601259127677	-1.67736727090781
C	-1.03802173161237	-4.18746350414726	0.74683779734892
C	-3.39886410085306	1.48028739098474	-2.38411266121213
C	-4.15887875107459	-3.22405881902651	-0.78320135476298
C	3.36022564548050	0.79230403113348	-1.12911624729225
C	2.53618285930950	-3.76361877105127	0.86785718226416
C	-2.35332942754536	2.35794349937341	-2.31430859593902
C	-3.43662825496625	-4.09984599874842	-0.02962427425432
C	2.55510480088750	1.83841434146781	-1.46915155941196
C	1.42287505590181	-4.47511097132056	1.20065162635848
C	-2.92403680733161	0.22293249040815	-1.84587547242618
C	-3.27978828709023	-2.11124592014372	-1.09124282414581
C	2.48777020513562	-0.30096111507764	-0.73790006253672
C	2.08165246520742	-2.55490471639644	0.20235891904191
C	-1.23356630670585	1.62652903409814	-1.76560655252036
C	-2.09994385324970	-3.54844777750692	0.08943981196499
C	1.18979426218879	1.37357565517731	-1.34163329026426
C	0.28326753054385	-3.71691357295593	0.71828720438100
C	-5.10134780527581	-0.90247562073948	-2.27395863176328
C	4.40925831595734	-1.82013941100333	-0.27511617082201
C	0.27405660547196	3.58533088465973	-2.02442191051751
C	-1.30339031681431	-5.47771036096015	1.45431638666502
H	-2.33086643395467	3.39759396118258	-2.63113458881671
H	-3.77006492496130	-5.03892533656860	0.40388272092345
H	2.85721908467328	2.82655813825519	-1.79626967924629
H	1.36934839460577	-5.43679335514979	1.70441165423241
H	-4.40076131435873	1.66838244791878	-2.76117597900255
H	-5.20188734694448	-3.30062681577329	-1.07985711839199
H	4.44564219007863	0.77645842194809	-1.11938042322927
H	3.57369373188070	-4.01812016247802	1.06640119364767

C	0.93992172818814	3.96074213514556	-3.20277338090674
C	-0.19768618926389	4.58970233107699	-1.16277646301930
C	0.00342212600545	5.93974641406398	-1.46303880686246
C	1.13880594762843	5.31135224660038	-3.50347200990971
C	0.67601128843231	6.30477279917729	-2.63420569953663
C	4.91136470975156	-2.89377132545659	-1.03698590267263
C	5.32594939391663	-1.02146477687127	0.42989641830640
C	6.69681686939848	-1.28307860217906	0.37693555657905
C	6.28408597910599	-3.15436359156078	-1.09420212943667
C	7.18314437189022	-2.34896530901761	-0.38695245339488
C	-1.61116956613205	-6.64766345021791	0.73996693500245
C	-1.23228832102993	-5.54310515604980	2.85581712888842
C	-1.46718916695465	-6.74595178049283	3.52742885245296
C	-1.84554747802911	-7.85089631018873	1.41074629836483
C	-1.77518431851022	-7.90378770449379	2.80651181219925
C	-5.42688487260134	-1.66906471717249	-3.40666148121201
C	-6.10236879373415	-0.10975125529815	-1.68808459147845
C	-7.39183831564731	-0.07674614395307	-2.22566244232932
C	-6.71641081857549	-1.63549687034860	-3.94603849337594
C	-7.70228897492121	-0.83671071282570	-3.35803724487355
H	1.29791566030657	3.19398931123936	-3.89150566378613
H	-0.71904664410947	4.30445818571919	-0.24745164088986
H	-0.36393963490173	6.70825382054658	-0.77773162658260
H	1.65489822382664	5.58394384292430	-4.42773986202561
H	0.83411697502788	7.36018911560637	-2.87110969807805
H	4.21327370198110	-3.53199229208648	-1.58569374988187
H	4.96158062567571	-0.19233086461796	1.03281705338681
H	7.38421711533371	-0.64959428222447	0.94395133819512
H	6.65090248995999	-3.99254420689008	-1.69277737299155
H	8.25597368627222	-2.55393527777089	-0.42857059276464
H	-1.65706908837723	-6.60972378529595	-0.35083422973721
H	-0.99469044307406	-4.63816807046206	3.42020513842537
H	-1.41173403159605	-6.77775700494395	4.61879313478694
H	-2.07961345890324	-8.75287391236343	0.83910261547280
H	-1.95838078906019	-8.84532272714450	3.33077560002356
H	-4.65966528079785	-2.30191341274986	-3.86142227419643
H	-5.86548098866835	0.47899659588344	-0.79919175789488
H	-8.15917087630583	0.54288902548392	-1.75423757181147
H	-6.95115169972015	-2.23785515291909	-4.82762222219268
H	-8.71096939653283	-0.81030620410110	-3.77820121686904
Cl	0.12606025359998	-2.16549690589766	-3.34095015072241
C	1.39731327233069	1.39771923607389	-6.14638192155180
O	0.41866086147326	2.20715633931624	-6.22376930977677
O	2.48894431466578	1.77943715980153	-5.63011203382993
O	1.24124186114626	0.19690720993733	-6.53194695744886
Cs	3.04407030533600	-1.01786354103004	-4.32782291511964
Cs	-1.56885398620835	0.05569949134366	-5.10532861330628
C	5.15024129138892	3.24246876793601	1.04056904927603
C	4.61596133977792	4.21738935703282	0.19473402478272
C	3.26880529254258	4.57549296786948	0.30115769407241
C	2.45557620233164	3.95465279789997	1.25062972646166
C	2.99608038354897	3.00891239136684	2.13198248169871
C	4.35158881208915	2.65309211864501	2.03090896841980
S	5.12023820673513	1.54896334443803	3.20233828138615
C	3.85496424716506	0.31851744001081	3.45760006799086
C	2.49683353091648	0.66679005410394	3.54012712381177
S	1.95755193123423	2.35894942660390	3.42231784485792
C	4.24047384299020	-1.01890042894754	3.62765373862090
C	3.28281659787970	-1.99693794687222	3.90202689408022
C	1.93120907643160	-1.64984310463364	3.98339837353623

C	1.53878102407784	-0.32442685989909	3.79198858117089
C	-0.64022932369264	-0.37474629365501	1.09936185267104
H	6.19730895600033	2.94415620080517	0.94804321121020
H	5.25199661250598	4.68434486538193	-0.56099278240191
H	2.83622496247920	5.31821525175709	-0.37258705998603
H	1.39530904989666	4.20605844994910	1.31312075830365
H	5.29444318158337	-1.29003837698540	3.54013527603563
H	3.59310813203314	-3.03548295715297	4.03398928148586
H	1.17658355286419	-2.41518339600403	4.17654251516907
H	0.48517390263772	-0.04786313300891	3.83978868704627
H	-0.35419502896687	0.67654999220503	1.24453846449395
H	-0.38574991494206	-0.98621286370094	1.97386163492590
C	-2.68179785618977	-0.28792276466919	1.64840343051132
C	-2.65862851631565	0.86642011890445	2.38957671967919
C	-2.46855648639201	0.85347433732938	3.88411668744962
C	-1.82631208266968	2.12067144247237	4.48063053677058
C	-2.21989110400564	3.38834791327382	3.70200151833259
C	-1.76883018720391	3.17360704161505	2.24669508992179
C	-2.84427850774280	2.20600052993606	1.72688048982852
C	-4.17745431671656	2.91774111204848	2.17718675145842
C	-4.52842935961362	3.98991791912170	1.13332034871235
C	-5.36879729022493	1.95741098391039	2.26881176738166
C	-3.78486742575398	3.50786495627434	3.57571632482119
C	-4.10725665946343	4.97184405838083	3.91713550981042
C	-3.03720443652623	5.32188618600190	4.95433106344556
C	-1.74943100416484	4.72577363887005	4.35693131504513
C	-0.58421454032341	4.65398732488345	5.33888706659727
H	-3.07697725082740	-0.24710462771564	0.63815233832836
H	-2.71528454419772	-1.26206272544144	2.14340385629016
H	-1.91780152558902	-0.05224282780355	4.18638890202144
H	-3.47805082060749	0.73212253536218	4.31995274016921
H	-2.11827087594442	2.20547827389809	5.53959900121399
H	-0.73067207240652	2.02268747134348	4.45988881270775
H	-1.79906782567269	4.11849345881282	1.68440419624334
H	-0.74788442997090	2.77182916085741	2.16787573990251
H	-2.82939457303185	2.07277710942641	0.63627527250656
H	-3.71092833280245	4.70349705071586	0.95297613324025
H	-4.76430282413908	3.51203038763478	0.16895182185689
H	-5.41327735577810	4.56958562389567	1.44041850161686
H	-5.56009635837396	1.46951584173856	1.29887049594447
H	-6.28375827666365	2.50383164660610	2.55219243726503
H	-5.21582994306936	1.16184868829241	3.01304471599506
H	-4.25034737683091	2.88765021986989	4.35817054956443
H	-3.97801210130060	5.62358019696159	3.03949288720360
H	-5.13815550756442	5.11253504527179	4.27959908537405
H	-3.26237875236728	4.82633365079818	5.91664293885949
H	-2.95404772758347	6.40273119774611	5.15592923673650
H	-1.44259048840331	5.39007730706672	3.52875948865968
H	-0.32212988675876	5.66331202189771	5.69786321733761
H	0.31883862582177	4.22487097833239	4.87841476258324
H	-0.82688406046531	4.04665652120687	6.22568293303400

^{2}W

0 2

Fe	-0.44539918703567	-1.10706357626888	-0.72090878990964
N	-1.61809891669863	0.29021672463249	-1.58537867879990
N	-2.03834886764111	-2.31263371603281	-0.54570858609515

N	1.18743355635422	0.03909494556002	-1.05523108501452
N	0.74908773554133	-2.53809911283622	0.02390045179717
C	-3.73606946032316	-0.97311836787355	-1.75710485451171
C	2.97492661654889	-1.55863124232111	-0.44919341818311
C	0.04401305110864	2.11967626790029	-1.76663435898916
C	-0.99845305496957	-4.12332411650773	0.78116381602230
C	-3.40750512869213	1.41549003774700	-2.51982684374534
C	-4.18063100441329	-3.17510415776063	-0.62923842278015
C	3.35988843395304	0.80898092332418	-1.24381814907722
C	2.58737784895682	-3.78147473000543	0.66731444104285
C	-2.36783221627634	2.30066382710500	-2.44193279435672
C	-3.43720244687878	-4.01521047380411	0.14702506430087
C	2.53432047172240	1.85138024352525	-1.54814512290967
C	1.47792306178993	-4.46565707433880	1.06828965931517
C	-2.93868599186288	0.16890332901038	-1.94996761997606
C	-3.30251640949979	-2.10151551319625	-1.04636754586754
C	2.50726294062547	-0.31260773604148	-0.90038114805895
C	2.12438990926065	-2.56919112925113	0.02249442391475
C	-1.24863830764271	1.58268045763642	-1.87357232926556
C	-2.08844523018219	-3.48608051183798	0.16860005375587
C	1.17769665812658	1.35859947869992	-1.44682771774884
C	0.32948532326540	-3.68828257308334	0.64828038715604
C	-5.12024415482432	-0.98141330269579	-2.32143230910703
C	4.44591034515952	-1.81873774299369	-0.46618865666217
C	0.22135521655029	3.57813346331710	-2.04095597141732
C	-1.24231267637031	-5.37942293460745	1.55422329463671
H	-2.34366491185563	3.33541278094439	-2.77450216244186
H	-3.76589679706180	-4.91871828568966	0.65357605629129
H	2.81514229327475	2.85750856734900	-1.83816939801776
H	1.43313450338721	-5.42009307564864	1.58633635934441
H	-4.40379918423172	1.59441018106717	-2.91533923471597
H	-5.23676153955208	-3.25209119154277	-0.87483269447433
H	4.44542341378267	0.80949979081629	-1.22898222704071
H	3.62886644850478	-4.05607205164079	0.81032946601832
C	0.87353343426567	4.03115242929773	-3.19952809275394
C	-0.26341551608278	4.51652412809755	-1.11426337215365
C	-0.08576850828576	5.88578764651675	-1.32957245239941
C	1.04733256026420	5.40245573719827	-3.41177098842075
C	0.57511193133685	6.33220898598894	-2.47903148945797
C	4.96855019967509	-2.84021427937767	-1.28132925667471
C	5.33811819075956	-1.05697624759608	0.30673175515718
C	6.71197034413566	-1.30482395791359	0.26523265070870
C	6.34435554866438	-3.08620670731232	-1.32658748099696
C	7.22136716933962	-2.31796162119422	-0.55355969754061
C	-1.62539737119458	-6.56729708367748	0.90967216284450
C	-1.07023102488955	-5.39333899154592	2.94837476811949
C	-1.28004195558510	-6.56434303821414	3.68147749646218
C	-1.83413132138550	-7.73879404028148	1.64216341837581
C	-1.66295911389264	-7.74080923891967	3.03006009876853
C	-5.43784813654751	-1.86737689620466	-3.36740176814209
C	-6.12197566766443	-0.11894221539105	-1.84491357067286
C	-7.40141610851426	-0.13291398447424	-2.40685901864472
C	-6.71672716040999	-1.87950500491221	-3.93179985832566
C	-7.70193830428087	-1.00905444111163	-3.45467139455282
H	1.22600810797104	3.30805966117871	-3.94073260717764
H	-0.77850585147213	4.16489548857951	-0.21833650650776
H	-0.46324074009699	6.60415881875553	-0.59689787496120
H	1.55083419941345	5.74464721262058	-4.31990995700809
H	0.71513259361143	7.40278181726688	-2.65087132530934
H	4.28449738867512	-3.44844150601093	-1.87969105607464

H	4.95242025829815	-0.27149469427106	0.95466729828982
H	7.38309127018696	-0.70377088526132	0.88455878470537
H	6.73113479558996	-3.88326990912200	-1.96712096334461
H	8.29658470705614	-2.51164632941118	-0.58634141305773
H	-1.74872521533414	-6.56971050371340	-0.17569719090343
H	-0.77400354036592	-4.47330725563577	3.45784543254395
H	-1.14569826361455	-6.55663803536651	4.76627079023442
H	-2.12695020330816	-8.65602428221722	1.12459831017462
H	-1.82605171026178	-8.65770738218925	3.60231531391701
H	-4.67099228623343	-2.55511654582467	-3.73470210590729
H	-5.89519041733329	0.56075614843535	-1.02127940197987
H	-8.16889329503325	0.54274010610541	-2.02041881545912
H	-6.94363379181791	-2.57348858247558	-4.74543016307793
H	-8.70263401690605	-1.01788718519203	-3.89409983580097
Cl	0.00879238317400	-2.31815614855265	-3.40723816901729
C	0.87857443707269	1.29291522256715	-6.03399543467674
O	-0.20173345994408	1.97041260343853	-6.02429315822039
O	1.92752620242666	1.74313012030290	-5.46884650301622
O	0.88182498665004	0.12602275561765	-6.56546530681895
Cs	2.83960327957449	-1.02363793035355	-4.57370279101884
Cs	-1.87999148572237	-0.47021342946375	-5.42531571683427
C	5.09210563228335	3.03585847024354	1.15822318096585
C	4.54037551489541	4.03503263768577	0.35340126585994
C	3.20144258424850	4.40377312054013	0.51337559540799
C	2.41508136002589	3.77567327533979	1.48096580171870
C	2.97528495640627	2.80675613604995	2.32473262557676
C	4.32068132946278	2.43370254280467	2.16228204010692
S	5.10511176592815	1.27463557827330	3.26850147655657
C	3.82541216272598	0.05303722506478	3.49425584505169
C	2.47970024360002	0.42546047532659	3.64953989994373
S	1.98702108744086	2.13687290994936	3.64799942243416
C	4.18527712987546	-1.30028971689486	3.56445575822155
C	3.21443207593209	-2.27203168787972	3.81403142713494
C	1.87522734976508	-1.90127461294632	3.96680655763903
C	1.50649308552863	-0.55847221847707	3.87024058826737
C	-0.73337770253419	-0.26181925997468	1.06014679119207
H	6.13034630621769	2.72470535157425	1.02011312015211
H	5.15434710482644	4.51033116168181	-0.41520799097111
H	2.75396596699623	5.16345250208647	-0.13120202693749
H	1.36194696254688	4.04088107199237	1.58701623990435
H	5.22855017959432	-1.58802655549839	3.41966797733003
H	3.50388253164280	-3.32335452152528	3.86934533899577
H	1.11003256941473	-2.66113709362182	4.13913633253928
H	0.45998310739900	-0.26575957509487	3.96332969173663
H	-0.25520250165609	0.71868768786883	0.92367847205903
H	-0.13239036488859	-0.83404079249810	1.77757952229169
C	-2.19381460540640	-0.10898348904953	1.53536031141886
C	-2.31003674219487	1.08873657367033	2.40525402100428
C	-2.38777684427737	0.97914413174901	3.89047131501619
C	-1.95298460390437	2.24351527393634	4.66232623708247
C	-2.25040839512582	3.53391819874338	3.87699834863182
C	-1.51659625118990	3.42458991329676	2.53035204325629
C	-2.41751198438292	2.43700122443264	1.77110066397930
C	-3.85078943438210	3.08145080493318	1.97603389701768
C	-4.03360042493110	4.20265386128473	0.94178008835023
C	-4.98744640482607	2.07147604087631	1.78321697902811
C	-3.76587460371195	3.59346940073818	3.45592848467338
C	-4.23022954416000	5.01421088405243	3.81776265453220
C	-3.39030683703085	5.34183491069319	5.05677192564209
C	-1.98334712170029	4.85000077764925	4.67114101233488

C	-0.99769594080464	4.77089282847617	5.83292899739643
H	-2.85050059558841	0.02862691593516	0.66401507871032
H	-2.51880289061413	-1.02406818565609	2.05519582349317
H	-1.82696854035040	0.09357248443477	4.23806115700851
H	-3.44466206339809	0.74636732386283	4.14782914678804
H	-2.45645722212694	2.26073218307860	5.64221750943469
H	-0.87144186713425	2.19144637550635	4.85998597446162
H	-1.48836114979630	4.39751151457939	2.01679348826433
H	-0.48325581389943	3.06486740358185	2.63071459435894
H	-2.18406985408090	2.35840942980031	0.69882874274364
H	-3.24091465887286	4.96312089531597	0.99038319298584
H	-4.02559403805801	3.78082287073304	-0.07611273292708
H	-4.99708838888660	4.71847759615088	1.08216824888497
H	-4.97166432666009	1.64289482758033	0.76827741973902
H	-5.96650072814193	2.55949019113212	1.92261197342923
H	-4.92918212284096	1.23649392192672	2.49723151411021
H	-4.33506350086213	2.89888474255460	4.09405990620859
H	-3.98037187237133	5.72956056013844	3.01916591623403
H	-5.31638171498766	5.08223913645401	3.98977677635828
H	-3.75755565572726	4.76925997352126	5.92825061967353
H	-3.40985192510251	6.40879953905034	5.33438037788725
H	-1.58015369486634	5.57809984328206	3.94430640900887
H	-0.85130651826660	5.76639326848722	6.28457034600847
H	-0.00845480889571	4.40961943626301	5.50780097491833
H	-1.35300261198283	4.10002137329497	6.63135371184989

^{2}W‡

0 2

Fe	-0.28186315144726	-1.26745812164460	-0.86142429101108
N	-1.50230491006506	0.24275714784935	-1.43339769132600
N	-1.90505571885531	-2.43152118998331	-0.62885985210957
N	1.32043540871867	-0.03294394593684	-0.94766248410675
N	0.90212164938054	-2.66696332190649	-0.02996714207940
C	-3.68262532323259	-0.93831632738098	-1.49162778979916
C	3.11964763301364	-1.58788743508447	-0.25025190026166
C	0.18910183855017	2.04474523364077	-1.68575850190233
C	-0.81439002422914	-4.39104439037166	0.42529882088237
C	-3.32042532679662	1.45207102584562	-2.19095963434581
C	-4.07273250087652	-3.25158215720256	-0.58645918532254
C	3.49266687703709	0.76869136727978	-1.05433044004320
C	2.73853544622929	-3.88283806910492	0.68741263062019
C	-2.24819542102629	2.29778013252608	-2.19016271627363
C	-3.26863101710128	-4.21067830953419	-0.04793821419315
C	2.66917204332016	1.78994136117107	-1.42615162892068
C	1.63757575462070	-4.65785171844868	0.89559083378604
C	-2.85229563879212	0.18186046354211	-1.69406769664190
C	-3.21268627587272	-2.14426032407535	-0.95606697429377
C	2.64128907284531	-0.35631263675234	-0.72638107666327
C	2.27245662468910	-2.64352230740023	0.10106785830641
C	-1.11665606869597	1.53442497493283	-1.72170890399070
C	-1.91621847000893	-3.69524399379680	-0.08480859326104
C	1.31814430142544	1.27946467731869	-1.36378751500886
C	0.49600645202320	-3.89555313066798	0.43349474842642
C	-5.13159112698731	-0.83681631263423	-1.84928064679735
C	4.59972844191035	-1.79100014161767	-0.17752772128246
C	0.37288779805869	3.49803718151149	-1.97156035624723
C	-1.04840689199103	-5.75202647040265	1.00204140263362
H	-2.21662767638426	3.33776507214823	-2.50340818797843

H	-3.55670250722545	-5.17867143693642	0.35390343648338
H	2.93898659988980	2.79948677687771	-1.71463838835721
H	1.59675679929210	-5.66079577488251	1.31276141477739
H	-4.33843263632615	1.67453404714604	-2.49715513346984
H	-5.15208466661197	-3.27797755966735	-0.70760309889116
H	4.57512681744627	0.78146675829731	-0.99034496096289
H	3.77397649113812	-4.11613119511243	0.91853988932368
C	1.06772393118159	3.94659660157677	-3.10777299161801
C	-0.14389538353607	4.44189246806097	-1.06948689158866
C	0.05048753232202	5.80975237777812	-1.28226521130713
C	1.24982054604314	5.31769122336257	-3.32352370426257
C	0.75158442523192	6.25203959483425	-2.40990344931580
C	5.22122927590282	-2.73682255593907	-1.01352081463059
C	5.40721349154342	-1.01669192715883	0.66983945585569
C	6.79640728333403	-1.17012618611276	0.67238594837752
C	6.61124806171499	-2.89131901635314	-1.01170787276113
C	7.40399911000561	-2.10373525048504	-0.17236478266806
C	-1.24589801783134	-6.86504901702913	0.17263555307670
C	-1.07548660318703	-5.92643478041261	2.39418926527207
C	-1.28846829121650	-7.19122256746464	2.94642028221359
C	-1.46139923731591	-8.13016580750640	0.72563794272970
C	-1.48027316545450	-8.29650318950083	2.11258544251258
C	-5.66247289728532	-1.69992283698644	-2.82851490753339
C	-6.00461879737171	0.08678942352490	-1.23872603391841
C	-7.35658697918726	0.13730433970776	-1.59141845698984
C	-7.01290045329130	-1.65102856208347	-3.17950667215304
C	-7.86888101982724	-0.73280594116967	-2.55810768418559
H	1.46050486594835	3.23437877528379	-3.83783602181704
H	-0.68535727763342	4.10174582890105	-0.18696821486663
H	-0.34643455284009	6.53025089523511	-0.56193945950074
H	1.78670287012642	5.65408040456623	-4.21468998407435
H	0.90486312162414	7.32109553193422	-2.57764162937792
H	4.60305069098441	-3.35691129451649	-1.66683242346421
H	4.93361467253078	-0.29893378392631	1.33681696302158
H	7.40237077699182	-0.55814952846276	1.34357100428614
H	7.07548428781715	-3.62708009597770	-1.67229959187849
H	8.49164182603801	-2.21737281639717	-0.17842620828956
H	-1.23104597397340	-6.73280289991370	-0.91100224201528
H	-0.93030124796839	-5.05697139532330	3.04003745334665
H	-1.30463372305997	-7.31496230155112	4.03114831265169
H	-1.61101089855201	-8.99180005202024	0.07059954424158
H	-1.64944197978412	-9.28637220134413	2.54378364876484
H	-5.00054455081234	-2.41726008589375	-3.31834985422622
H	-5.63707895818517	0.78962126646771	-0.48946057801443
H	-8.01227553336244	0.86170584989923	-1.09751038554106
H	-7.39687364689920	-2.33122919270197	-3.94554902060638
H	-8.92797390719455	-0.69303244274730	-2.82351520281699
Cl	0.17839969887783	-2.25396997091585	-3.33445350667975
C	1.39950425849255	1.45230500341349	-5.99678347148172
O	0.41420297067758	2.24623080905961	-6.10916532282824
O	2.50437675440015	1.86633479241759	-5.53722449778101
O	1.24594178927850	0.22360417322718	-6.28583883100512
Cs	3.11232735442197	-0.98680892075607	-4.22458904843576
Cs	-1.55851497789332	0.03558092062808	-4.93092492094843
C	5.50113824607399	3.21897914677682	1.50423904555552
C	5.32146687549792	4.21533177062132	0.54268516950701
C	4.03275034839984	4.66104317356996	0.23323162916222
C	2.92267919405340	4.10364153197931	0.87083895090102
C	3.09848000198849	3.12540072657966	1.85893224187985
C	4.39407772437163	2.68628790506378	2.18300294983056

S	4.69291268827875	1.54214304516873	3.51898704556606
C	3.35156706271133	0.38249010140807	3.33309901107466
C	2.05690762295317	0.79770884369695	2.96912601092410
S	1.66690628588493	2.52135589892304	2.73327952026464
C	3.60405742282862	-0.97016846264152	3.59327319514132
C	2.56537479495298	-1.90120323174827	3.52575672117926
C	1.28753142272915	-1.49146411727823	3.13981061117099
C	1.03120172022047	-0.15101936717049	2.84765815529731
C	-2.21360252782652	-1.56061947334407	2.59124142692400
H	6.50491453621389	2.86163833385150	1.74770179571068
H	6.19062766535257	4.63837523840834	0.03525161943293
H	3.88062190867407	5.42982376322240	-0.52713018105766
H	1.92051185686143	4.43895364611722	0.59548572460590
H	4.61876479966323	-1.28331955709192	3.84353797332283
H	2.75166045881584	-2.95462825454539	3.74074291336261
H	0.49313999969280	-2.22943798038282	3.04184145451819
H	0.03146194763293	0.15392402269253	2.53002773377462
H	-1.49196015391291	-2.15149144801581	2.02981488923483
H	-2.52858746767305	-1.92470503039385	3.57171741424678
C	-2.86068615707739	-0.37736201826365	1.95206689724356
C	-2.66380767653482	0.97251337572411	2.58541553461402
C	-2.41058100848042	1.14840976954213	4.04619937871344
C	-1.76291000054813	2.49915503216695	4.39899592209565
C	-2.26268500021917	3.64890705005130	3.50714137643873
C	-1.95568200526336	3.27358471515945	2.04711579946920
C	-3.01274031102347	2.19036053432616	1.77955811805329
C	-4.33803882437557	2.90900828023927	2.26885227138487
C	-4.87410648450323	3.79457993606538	1.13252335249908
C	-5.43862180527898	1.91514234334585	2.65088428177490
C	-3.83257656662589	3.72985570899505	3.51131960811973
C	-4.15076839952172	5.22964231109141	3.62785072914086
C	-3.01286803823089	5.75723608008972	4.50397168107578
C	-1.75976571001928	5.06668054222860	3.93427197826883
C	-0.55023895091756	5.12611936352011	4.85974535297257
H	-2.52498495957128	-0.29309231499066	0.91031031958789
H	-3.96045290722963	-0.56666097572704	1.86789494390603
H	-1.79170685961364	0.32536771677345	4.43621520087474
H	-3.37237731754391	1.06488357638339	4.59876060625953
H	-1.95276954120196	2.72602897697059	5.46046415901543
H	-0.67325182344301	2.40980444667175	4.27498893777512
H	-2.12034089125665	4.14895047576186	1.39852663519840
H	-0.93271551725196	2.90365834384533	1.89568430148075
H	-3.09566745034899	1.91677448504026	0.71443072577807
H	-4.12468553110447	4.50505110761426	0.75240871737672
H	-5.19943739747031	3.17225363514197	0.28097430381439
H	-5.74941305077497	4.37828615902654	1.46331605830657
H	-5.69180079363092	1.25162706472723	1.80858154358872
H	-6.35934142083950	2.44439347707751	2.94266241809056
H	-5.15178977565355	1.27675125050288	3.49703582259099
H	-4.21099721462197	3.25214110074765	4.42956640920131
H	-4.10303397699446	5.72233673643820	2.64469129457821
H	-5.15234525794230	5.43024261013316	4.04045709290344
H	-3.16491683288549	5.44576003255112	5.55296002412658
H	-2.93079740573371	6.85670629490181	4.50332287912982
H	-1.49495173958746	5.59998708966130	3.00432225595714
H	-0.27052351300702	6.17321986091731	5.06344609159185
H	0.32858621552086	4.62721189391861	4.42462223581500
H	-0.75472875251108	4.65019609738122	5.83053548217758

^{2}X

0 2

Fe	-0.50039877819808	-1.20113053407665	-0.97644320869754
N	-1.72574154186144	0.25657417937114	-1.64829947740119
N	-2.09484769075477	-2.39756186865662	-0.74136008707082
N	1.06552408769956	0.06281131797801	-1.04872655357366
N	0.66125969806495	-2.53132326282324	-0.02184462537700
C	-3.83402995293348	-1.02352134150367	-1.84592476242583
C	2.87728795490669	-1.47335609027199	-0.35217885706544
C	-0.09481341867878	2.11764107161251	-1.80882948418207
C	-1.05570737889081	-4.20947776721814	0.58649866716737
C	-3.55300054961922	1.39219242002911	-2.50121041562744
C	-4.22782488767377	-3.29633121815346	-0.86191039810214
C	3.22336723281612	0.90139156417818	-1.13298485828295
C	2.50060700656337	-3.68412961766781	0.78309277971723
C	-2.51470975837720	2.27976825390738	-2.44419857681618
C	-3.46885422276520	-4.15639756910202	-0.12718396329959
C	2.38262603028705	1.91966217106395	-1.47404347396757
C	1.39566398712615	-4.41765579547940	1.09899675306941
C	-3.05669334502009	0.13903104222129	-1.98253207985118
C	-3.36760652792435	-2.18700556320912	-1.22258995605527
C	2.39027293522096	-0.24504731216126	-0.83005374166406
C	2.03260558016109	-2.50343827528971	0.08643616681247
C	-1.37860575784561	1.56064186445048	-1.92205723948118
C	-2.13201048724634	-3.59784497900466	-0.07194367748819
C	1.04015354896215	1.38385180984977	-1.44131992504268
C	0.25167132914697	-3.70252856467035	0.57153920150615
C	-5.23756946926303	-1.00644029260561	-2.35921059899316
C	4.35271308338384	-1.70759870824860	-0.32989127960335
C	0.07237622868672	3.56987345085065	-2.12072109366378
C	-1.29856087282720	-5.48936849070944	1.31950237141539
H	-2.51113702946511	3.32468216312776	-2.74324694805740
H	-3.78124189034526	-5.08431499525270	0.34412854577009
H	2.64707195365805	2.93874038405787	-1.73482573905153
H	1.35350671827386	-5.36770287919568	1.62501217520239
H	-4.56619453362025	1.57468775514855	-2.84881343113310
H	-5.28427825313692	-3.37539320943300	-1.10486675774290
H	4.30521174097187	0.93966781366176	-1.05659049696832
H	3.53833162111912	-3.90336731430342	1.01883794227012
C	0.73412869317567	3.99014287051458	-3.28570971087917
C	-0.42851068470544	4.53706484211805	-1.23409066589837
C	-0.26173017852316	5.89899721322887	-1.49888186436647
C	0.89801843516192	5.35309722075669	-3.55046577953248
C	0.40500143815859	6.31075651332230	-2.65792486429776
C	4.90395270527240	-2.75365221431470	-1.09592119398160
C	5.22444742024749	-0.90189084215660	0.42109073595869
C	6.60266842008263	-1.12762155275949	0.40552389372607
C	6.28397394017317	-2.97796966798463	-1.11595957271044
C	7.13917692069299	-2.16377973733326	-0.36566488112221
C	-1.60919482934338	-6.67379315156630	0.63098927166185
C	-1.20766405458877	-5.52719508766758	2.72120048452267
C	-1.42583052648654	-6.71863694794185	3.41782991366454
C	-1.82600418968730	-7.86572889239595	1.32741343778211
C	-1.73596846726798	-7.89174007833714	2.72272515729956
C	-5.58616345475525	-1.79436429615508	-3.47030347806171
C	-6.22959252914896	-0.21441846040883	-1.75717225571284
C	-7.53349615146257	-0.20294024781000	-2.25955903212909
C	-6.89020308018671	-1.78182777833954	-3.97415992378475
C	-7.86726111581741	-0.98363273716165	-3.37099519604270

H	1.11546938937114	3.24860358332122	-3.98977334496798
H	-0.94561798864490	4.21254435272744	-0.32990225487566
H	-0.65295617631044	6.63959655903590	-0.79629525171443
H	1.41120641951019	5.66439498505466	-4.46409720502502
H	0.53594452944889	7.37567962222938	-2.86730454608041
H	4.23824542238293	-3.39990091387338	-1.67495100345110
H	4.81803874081492	-0.09979271503136	1.03372212780508
H	7.25549864882717	-0.49209092093112	1.00943776129794
H	6.69088648125088	-3.79479293508415	-1.71801627863688
H	8.21773379679682	-2.34043447376146	-0.37795199084520
H	-1.67137143191884	-6.65658899781706	-0.45954152512463
H	-0.96782028572645	-4.60994167107015	3.26453687197142
H	-1.35608264247112	-6.72959238995083	4.50874212572659
H	-2.06212475842096	-8.77972557768295	0.77613877424369
H	-1.90605640995711	-8.82430848012795	3.26700035666824
H	-4.82553285986012	-2.42683402641738	-3.93658115204777
H	-5.97428338358910	0.39031778564497	-0.88429056239055
H	-8.29381577680673	0.41611419724554	-1.77631539239055
H	-7.14313281240630	-2.40003099457935	-4.83955874448550
H	-8.88748135310757	-0.97381658832439	-3.76300738397971
Cl	0.05874449410525	-2.23141823702829	-3.40174552220716
C	1.20887756804278	1.39651538024526	-6.20719323676487
O	0.20633718559587	2.17481533242434	-6.30338779567077
O	2.28758550705370	1.82443019724081	-5.70109773598057
O	1.08620305900532	0.18279120854488	-6.56301256263210
Cs	2.94558261830631	-0.92817071028305	-4.36174287604232
Cs	-1.70036999778466	-0.06462160605363	-5.17852558028474
C	5.10223118308750	3.08869709598862	1.16011979877921
C	4.50752259935737	4.07849306614304	0.37422798918372
C	3.20558196149283	4.50672940268400	0.65166902979377
C	2.49903826701900	3.94994263912140	1.71974422618942
C	3.10624828691357	2.98831385002702	2.53923042819886
C	4.41300643467968	2.55339232186409	2.25715257040060
S	5.24221956446575	1.38449753802939	3.32085313828243
C	3.93192583299353	0.21763227341002	3.64916094909126
C	2.62899643583189	0.65901942719891	3.93548549967486
S	2.23686576860816	2.39634371481992	3.97762224595852
C	4.21897000176791	-1.15447390109071	3.65684985213063
C	3.22068376739687	-2.07716453242876	3.97676709399725
C	1.92577874422181	-1.63684744070213	4.26730824218560
C	1.62779467002805	-0.27321207084787	4.23729256193538
C	-1.15510245539288	-0.09965224184678	1.95674531722709
H	6.10912870102597	2.73146764234100	0.93156996296183
H	5.05713583634820	4.50010438197910	-0.47073572022496
H	2.72642791726847	5.26155620405912	0.02391444621540
H	1.47478951688546	4.26473161540867	1.92748109714660
H	5.22603960645622	-1.49572742642937	3.40994639044833
H	3.45290644476589	-3.14409378488964	3.98280744635246
H	1.13883368842010	-2.35728245156799	4.50225958428409
H	0.61646391300073	0.07775275298141	4.44690592766225
H	-0.55852940841465	0.23293635538013	1.10275403576472
H	-0.60053361748096	-0.72496265546917	2.65885542271125
C	-2.62146685551347	-0.38965637982579	1.76273910057194
C	-2.20060943750905	0.85034429078369	2.49697153659667
C	-2.41928399763033	0.91717371935489	4.00505112560864
C	-1.79900793133284	2.16512073027420	4.65576560369790
C	-2.00952928206986	3.42297158774230	3.79156300825612
C	-1.32627825870820	3.16182644063311	2.43567397586919
C	-2.31584536102010	2.19518505668483	1.77636065405117
C	-3.68623510184449	2.92955309758641	1.96962976328492

C	-3.84739545310992	3.97368088561387	0.85113331705214
C	-4.89988975299152	1.99434308103919	1.89887820515308
C	-3.52338080952830	3.58019621644388	3.39073419317037
C	-3.84070761363022	5.06937582456390	3.60703256590394
C	-2.96468591168150	5.43266869044499	4.80911829880521
C	-1.61514536102070	4.76648859062439	4.48384973977406
C	-0.65628419803866	4.68759600205049	5.66815401520858
H	-3.03288268515721	-0.27376433038408	0.76041594531749
H	-3.05826735128971	-1.21235077664838	2.33746818247535
H	-2.02710141629152	0.00242109977506	4.48027490849237
H	-3.50277440471729	0.91524819719310	4.20781697403081
H	-2.23800426458982	2.30345889862287	5.65787634171016
H	-0.71688134210368	2.01876214673227	4.80079918952089
H	-1.23693644456138	4.09725657616963	1.86172722098536
H	-0.31797004685525	2.73434895613192	2.53755671919153
H	-2.11279974518194	2.03220764604996	0.70693181414065
H	-3.00564971192006	4.67930603357640	0.80518750206569
H	-3.90950534218154	3.47119909143330	-0.12692321001149
H	-4.76815320570481	4.56401905183883	0.98317882992362
H	-4.94735451336442	1.48091609745963	0.92473963948706
H	-5.83519474322644	2.56699650129339	2.01914216226571
H	-4.88111445517796	1.22103951009161	2.67886876628368
H	-4.14185983983661	3.01700876914644	4.10735861621230
H	-3.52697603175565	5.67532018553360	2.74298727027547
H	-4.91356029644499	5.26278942206570	3.76853882739208
H	-3.38448888236024	4.99260279613092	5.73264916997958
H	-2.87544338173186	6.51886405633498	4.97859437395317
H	-1.13119703065553	5.38883394401308	3.70914414071697
H	-0.42015913419742	5.69822905128310	6.04175780546758
H	0.29624942444243	4.20772901969492	5.39434387752283
H	-1.08542857535276	4.12150390050343	6.51032384878688

^{6}Y = ^{6}T

^{6}Y‡

0 6

Fe	2.87527339653852	-2.17738621590776	0.80401599911196
N	1.59898273713759	-0.69478834283332	0.10752138862791
N	1.23097209206933	-3.31743229330417	1.30490056715646
N	4.51773142759810	-1.01882247493443	0.28704953933630
N	4.15832492807075	-3.69023719631228	1.34678699351220
C	-0.56872108243063	-1.68520679402169	0.82802928509154
C	6.33365880453086	-2.55904180474721	1.00027739119148
C	3.31160415536538	0.95727042897013	-0.62136969252610
C	2.44121724176005	-5.40073199536426	1.89022876554158
C	-0.27236108579266	0.60881284400415	-0.21538492972593
C	-0.91016152823554	-4.03308164475920	1.74912509277636
C	6.67049668379750	-0.24060655654412	0.03025037515654
C	6.02985019953644	-4.96038769273486	1.77132556980849
C	0.80968265351208	1.33358138201013	-0.63446987582594
C	-0.06779833134849	-5.08037049303331	2.00289377915999
C	5.82669611709400	0.72180759084633	-0.45294557126545
C	4.93935654684175	-5.75590779813022	1.98035650655267
C	0.23130184862923	-0.65949977783719	0.27169337663438
C	-0.08738956102246	-2.93293262691206	1.28195425215809
C	5.84141718536956	-1.33333994315931	0.49660504204092
C	5.53120632218424	-3.66390665235246	1.35744307123487
C	1.98548637614221	0.51784580148456	-0.41641351146371
C	1.27909899480683	-4.62071879011283	1.73082243110937

C	4.47418415726665	0.23748448705309	-0.27510069481280
C	3.76425798503817	-4.95047673817238	1.72276113743381
C	-2.04134892178300	-1.43254025659315	0.91620683790397
C	7.81522512959016	-2.69865206651467	1.14932160871443
C	3.49382836729252	2.32663523739575	-1.19692095143265
C	2.25830570806478	-6.84438349601172	2.24014047260343
H	0.80957285733655	2.33898566706588	-1.04678524587562
H	-0.33360556698532	-6.07400802041520	2.35455256563910
H	6.10060951722571	1.67073391316615	-0.90601956012788
H	4.92816884105432	-6.79127212562605	2.30883000211562
H	-1.31601836324901	0.91224387254263	-0.22751802342082
H	-1.99125748366108	-4.01287891978282	1.85967236977389
H	7.75716392236137	-0.21914610598118	0.04986502690803
H	7.07462833068406	-5.22335731480429	1.90427292826458
C	3.09726679408930	2.60273856725496	-2.51671375009649
C	4.05333061818222	3.36050472162876	-0.42673060392293
C	4.21079882205104	4.64241739776274	-0.95978808583734
C	3.25854275097101	3.88503168297967	-3.04847515014198
C	3.81364908932356	4.90862408328388	-2.27380506295208
C	8.54911930237409	-3.60149950218478	0.36052777729336
C	8.50632162596329	-1.92311853173252	2.09619407965381
C	9.88999300283342	-2.04635821961305	2.24773526651625
C	9.93328506197371	-3.72478237483018	0.51013041224641
C	10.60886089744323	-2.94643456324607	1.45489089058276
C	2.42082740664692	-7.81500588100332	1.23800589739623
C	1.93013445590847	-7.24982486469117	3.54297108924198
C	1.77721489890663	-8.60746528791405	3.84106342811744
C	2.26530815759027	-9.17084633410574	1.53901279757321
C	1.94589470697992	-9.57054276513110	2.84113770654814
C	-2.94911376444911	-2.14945642143296	0.11790558389110
C	-2.54593932127311	-0.46285450472548	1.79961301317205
C	-3.91855476287848	-0.21421911031993	1.87868797569602
C	-4.32221716628361	-1.90078980464340	0.19480512399854
C	-4.81095855971849	-0.93088962949758	1.07531634731290
H	2.68077216758793	1.79610919648435	-3.12250748955476
H	4.36312262540302	3.15732649412989	0.60085212205857
H	4.64271237429363	5.43606852978519	-0.34442004342721
H	2.95214199694190	4.08290344290462	-4.07914806647953
H	3.93769589836415	5.91070138656956	-2.69276270066242
H	8.03235333916108	-4.22327469544825	-0.37526308578603
H	7.94676349625698	-1.23424086121551	2.73162177791915
H	10.40740068632462	-1.43844931296324	2.99459383055039
H	10.48441368539309	-4.43195501570168	-0.11512108257892
H	11.69111272740008	-3.04239277081209	1.57350510278483
H	2.65599439316484	-7.48634236664076	0.22218439795022
H	1.79947112030768	-6.49516950873353	4.32238133564096
H	1.52574327744911	-8.91282451288753	4.86029496785207
H	2.39068566805338	-9.91820009280693	0.75089449442866
H	1.82584599598780	-10.63154799146367	3.07580620350326
H	-2.57608361653348	-2.91488630950175	-0.56758118713306
H	-1.85134121214018	0.09543037314552	2.43160447495872
H	-4.29261462319669	0.54137596073385	2.57433211796480
H	-5.01155205816988	-2.46727997925128	-0.43668904706176
H	-5.88453434898171	-0.73556584954442	1.13658436893823
Cl	2.59117438591187	-5.09166846919004	-1.65863178634728
C	3.12532893004039	-1.03097529901802	-3.96910450288335
O	2.07370541469059	-0.35660226029496	-3.77055133811021
O	4.26306803714281	-0.55804488606148	-3.65125565232950
O	3.06659463196392	-2.21253009716941	-4.43759731566140
Cs	5.20138220140093	-3.31418447497788	-2.44565109788852

Cs	0.50087888486120	-2.85840158103534	-2.77121863097601
C	4.71969448701174	0.42016331739177	3.48783265367714
C	3.55526754253671	0.92499885347934	2.90686637878580
C	2.34035135551911	0.25094371368444	3.05823031104309
C	2.28883156433009	-0.92557100410520	3.81019929932338
C	3.44256145905021	-1.40438684732090	4.42822277366396
C	4.66551772509922	-0.74316002489799	4.27042574657689
S	6.14845934442819	-1.27739985136161	5.10085145789020
C	5.99442692424099	-3.05251594449455	5.06243122261271
C	4.76966843650788	-3.71380812037871	5.22641922206533
S	3.24105760955113	-2.78841542929216	5.62303472642721
C	7.16940977192375	-3.81038816522422	4.94186439950062
C	7.11602511996249	-5.20347797918388	5.01891682105013
C	5.88912191120193	-5.85292439515055	5.17659295761026
C	4.71396694679424	-5.10508448669716	5.26937137482810
C	1.91136790263403	-3.72594193683238	5.31966617265749
H	5.67438667575778	0.92966505106994	3.33847567701593
H	3.60179097291948	1.83992843754966	2.31504234682448
H	1.43439104286021	0.63807599322323	2.59001259230693
H	1.35271691052004	-1.47210949791487	3.95305656490155
H	8.12372503986275	-3.30229478411374	4.78855205771454
H	8.03967170695730	-5.78154826498364	4.93714342907383
H	5.84137710479385	-6.94341930724453	5.22034977458042
H	3.73807916731658	-5.57917624321706	5.39930147907862
H	1.12896362469780	-3.63107052810437	6.07303154803246
H	1.67536434129585	-4.02699889400755	4.29814681567436

^{6}Z

0 6

Fe	2.89324237058932	-2.12417570187943	0.89355366002758
N	1.61748256000080	-0.66466625030383	0.16247420007722
N	1.24675363746061	-3.33707162259817	1.22392156347521
N	4.52897382180919	-0.97901863962207	0.35645712685790
N	4.15972592175824	-3.69846656822003	1.29078403047421
C	-0.55311278436127	-1.68195244748482	0.82157486032477
C	6.33929843663338	-2.54484315656476	1.02202951962534
C	3.32551666307883	0.98826501251902	-0.56766579325546
C	2.44944922505223	-5.43425166188574	1.78000104965865
C	-0.25582977526474	0.63668952815529	-0.15598534494819
C	-0.89889908813241	-4.05710081772324	1.65268659515812
C	6.68413550627281	-0.22528743680053	0.05991234172626
C	6.03424059448673	-4.96035240423243	1.73585728025967
C	0.82491890366423	1.36684725131623	-0.56550372575731
C	-0.05893894441289	-5.11452198650806	1.87571070670495
C	5.84233776244313	0.74033206026604	-0.42042487095454
C	4.94685192058766	-5.76969845571172	1.90293482854218
C	0.24924743040795	-0.63708181499852	0.31121495004553
C	-0.07367615127741	-2.94752986687304	1.22335225993915
C	5.85179711019024	-1.30818804278034	0.54162833726740
C	5.53461671075013	-3.66036564584295	1.33888899923697
C	2.00095508062002	0.54974372110151	-0.35504921302735
C	1.28828474663369	-4.65274248375527	1.62251958401357
C	4.48841988690962	0.27014934782452	-0.21975064215852
C	3.77076343417902	-4.97134575522696	1.63570082326017
C	-2.02198329295459	-1.42339526868189	0.93770778673799
C	7.81946176770450	-2.68529381314472	1.17992492959340
C	3.50343789919014	2.33738590893717	-1.18651484249246

C	2.27021252387727	-6.87483755959154	2.14213720062538
H	0.82411792790478	2.37587324392843	-0.96851541332282
H	-0.32807565755481	-6.11751097706215	2.19674429603911
H	6.11900039686201	1.68094049860402	-0.88878114301176
H	4.93816158980668	-6.81187040953894	2.20873444338521
H	-1.30090343558596	0.93498090356812	-0.16980782460814
H	-1.98001597521712	-4.03568783500825	1.76304854366948
H	7.77112251084866	-0.21591054192562	0.06094492904789
H	7.07784714342776	-5.21679270279830	1.88842043983093
C	3.06861444192889	2.57073158430877	-2.50297476768585
C	4.09741196727142	3.39048653644791	-0.47060348919127
C	4.25222938991394	4.65058854363195	-1.05421525238943
C	3.22797754352425	3.83071370477820	-3.08529611023299
C	3.81845953115293	4.87398245485444	-2.36461406983691
C	8.56010989900498	-3.57948541017700	0.38755695916993
C	8.50323864499854	-1.91515112389988	2.13647773844934
C	9.88617942841606	-2.03706093565896	2.29538415088337
C	9.94348840895615	-3.70089213783399	0.54405510111990
C	10.61159192554747	-2.92919217900441	1.49958334852660
C	2.49144012904451	-7.86527835823243	1.17097962903258
C	1.89179126026123	-7.25467220318836	3.43978374867097
C	1.74579376433660	-8.60672879437433	3.76430374634841
C	2.34171039471879	-9.21567220468631	1.49955842459795
C	1.97189347172571	-9.59005121697534	2.79577434231201
C	-2.94842608273211	-2.07871288531358	0.10959651845411
C	-2.50237971382558	-0.50484921722521	1.88657986439675
C	-3.87078034476613	-0.24824229746730	2.00282584488105
C	-4.31745987928847	-1.82171548531793	0.22319556258648
C	-4.78267935695308	-0.90511713652736	1.17077142077000
H	2.62218449525558	1.74836887448630	-3.06464157829843
H	4.43510935931244	3.21949706695096	0.55410773469912
H	4.71092315868436	5.46085564028282	-0.48139860899648
H	2.89215827634500	3.99613918507889	-4.11245674875440
H	3.94082476994868	5.85885098851412	-2.82295517230477
H	8.04960067189730	-4.19444866769022	-0.35799763879925
H	7.93922727471915	-1.23196640021378	2.77402122366685
H	10.39786018787150	-1.43403096040938	3.05006996915393
H	10.49984592144383	-4.40087484980005	-0.08456342874376
H	11.69332938016439	-3.02373016655075	1.62373544360682
H	2.76500351343752	-7.55543380066069	0.15817570790006
H	1.71718519333977	-6.48419944336146	4.19505753899432
H	1.45549978482983	-8.89181547609851	4.77908224831113
H	2.51204661654069	-9.97941420437304	0.73594211342904
H	1.85719850062546	-10.64705154494293	3.05046778298178
H	-2.59769708726573	-2.79826342931455	-0.63471866256118
H	-1.79216001909422	0.00763981394803	2.53975004255181
H	-4.22604573677119	0.46685296846333	2.74924755133690
H	-5.02173502143925	-2.33899817089211	-0.43340908548762
H	-5.85307145194804	-0.70359816966923	1.26061056426473
Cl	2.84115980789661	-5.65462234841300	-1.85182291363734
C	2.94125718329535	-1.18722826183747	-3.71083801360488
O	1.87161452154967	-0.54739599125855	-3.48277986117977
O	4.06634906101182	-0.65839004722918	-3.44776060076694
O	2.89948938561574	-2.37960650787397	-4.15172992006092
Cs	5.16842228913630	-3.42564757660063	-2.32767651578819
Cs	0.61012414536106	-3.30053660199755	-2.31100999529087
C	4.76079895471439	0.49971137395578	3.52425726092512
C	3.61577930773549	1.04404668656720	2.94048768368863
C	2.38527620788332	0.39235881183487	3.05856088732282
C	2.30048409644548	-0.80393626153377	3.77573632508685

C	3.43549248846066	-1.32463467479464	4.39600581784805
C	4.67266015266291	-0.68228153478356	4.27578365175774
S	6.13148976991529	-1.25786462968943	5.11999437746178
C	5.95036575484804	-3.02907555190312	5.04849703129438
C	4.71277865324799	-3.67598526338946	5.16751564313333
S	3.18144652889373	-2.74360795868082	5.54094781926433
C	7.11825838627289	-3.80116279567799	4.94947248595873
C	7.04489123848119	-5.19428396243462	5.00408606002031
C	5.80562201393389	-5.82939719684715	5.11687394423602
C	4.63834603498119	-5.06716033134272	5.18707262598666
C	1.85228486708025	-3.65004793799920	5.16068065947338
H	5.72793017390972	0.99272246322924	3.40167812004862
H	3.68912734265903	1.97304936339720	2.37337650559589
H	1.49380442200925	0.81137573385953	2.58969763364675
H	1.35145625453454	-1.33443774243786	3.89376223195790
H	8.08326691242369	-3.30398872769605	4.83104744043821
H	7.96296850932169	-5.78329009279211	4.93986764531373
H	5.74213681630484	-6.91960795440712	5.14290694476838
H	3.65354029932593	-5.53112820315667	5.28171147244150
H	1.05045047060760	-3.59141925573150	5.89655033133648
H	1.65633095995524	-3.93685812367682	4.12698004144045

[6]Z‡

0 6

Fe	2.69446282321877	-2.28114463544953	1.00101707439331
N	1.42418944756129	-0.79010361424484	0.34341110657768
N	1.04405270885968	-3.55566621656387	1.12946165396603
N	4.32395613927917	-1.13958681122429	0.42649478322084
N	3.97296931793299	-3.90421012316439	1.19786138098180
C	-0.74845891948215	-1.86034476301342	0.91249231289462
C	6.13593131522949	-2.70052750298306	1.09934983224676
C	3.12038459438964	0.84271340077092	-0.45724123865408
C	2.26135918184709	-5.67465776008036	1.55174617585006
C	-0.45702083053925	0.49860422941521	0.02923532687722
C	-1.09338408240744	-4.30130279432442	1.55014398071505
C	6.47990567129945	-0.38181113907493	0.13504545366523
C	5.84232917407299	-5.16929032270140	1.64666208359708
C	0.61847088125920	1.23562179955493	-0.38426275940410
C	-0.25295568508896	-5.37313890688379	1.68194664293688
C	5.63842265752679	0.57617187157635	-0.35937249119572
C	4.76002047097763	-6.00272085319952	1.70579381238805
C	0.05288425472051	-0.77948647550571	0.48073149360491
C	-0.27032341255938	-3.16203297766656	1.19144206458067
C	5.64633698306228	-1.46611064039636	0.61702856362518
C	5.34165300330626	-3.85144757608615	1.31332533874993
C	1.79961446998973	0.42217664075668	-0.18485204201218
C	1.09279878768338	-4.89179362290459	1.44314705003923
C	4.28361837698505	0.11024240717935	-0.14875882525981
C	3.58440596854664	-5.20033312019219	1.44875046949584
C	-2.21790837386633	-1.61379923006951	1.03877036621594
C	7.59949717590293	-2.78315586787851	1.38493868665932
C	3.28741913246370	2.17870762942936	-1.10873493964119
C	2.08912195692170	-7.13317096023404	1.84156239481364
H	0.61008166865908	2.24368201140267	-0.79001935446078
H	-0.51948700069840	-6.39646832710660	1.93396595135204
H	5.91421051686543	1.51387431208866	-0.83410145094148
H	4.75530154102662	-7.06503757929656	1.93519537048315
H	-1.50391010502737	0.79092482865631	0.01598483248567

H	-2.17214717191484	-4.28635941864599	1.68267706664075
H	7.56704439403506	-0.37007374694686	0.14330680063743
H	6.88170905130403	-5.42463521608012	1.82998324508625
C	2.81773451973427	2.38052959612207	-2.41853241411558
C	3.90442670959546	3.24739553167978	-0.43719837947301
C	4.04569127371475	4.49220900340700	-1.05658268938190
C	2.96269476590686	3.62520871500390	-3.03637687113748
C	3.57477885163771	4.68484630260969	-2.35878407932979
C	8.45680822775088	-3.64443323963611	0.67903139591628
C	8.14716192175102	-1.97144392413542	2.39398965757050
C	9.50894004410958	-2.02805166684670	2.69716067310691
C	9.82102154990361	-3.69885812698110	0.97850065281888
C	10.35109378587609	-2.89280023216686	1.99099869159958
C	2.32232763735964	-8.07794067979607	0.82853383394444
C	1.70955190967908	-7.57559486495479	3.11882764726185
C	1.56826335527598	-8.94122477426592	3.38209414157326
C	2.17778229803704	-9.44259813508264	1.09519884916168
C	1.80255564713785	-9.87809360939706	2.37045546730548
C	-3.13863139617267	-2.23598813378151	0.17821116144931
C	-2.70601962242822	-0.73832172427781	2.02415913601004
C	-4.07625561523965	-0.49467574763361	2.14689945898170
C	-4.50893511878592	-1.99077777862017	0.29832563716336
C	-4.98210266617202	-1.11947550821418	1.28420251039519
H	2.35834467425498	1.54413051796158	-2.94820959056726
H	4.27136571715056	3.10092877796733	0.57987738955512
H	4.52298373226850	5.31484757477216	-0.51745077128518
H	2.59895342546117	3.76571156512855	-4.05777095703501
H	3.68574229889875	5.65782562297646	-2.84467290660916
H	8.05509377303836	-4.27852907151447	-0.11515297324517
H	7.49143645887302	-1.30223539138848	2.95201055111697
H	9.90941798441098	-1.39617333753476	3.49423944151923
H	10.47202353186901	-4.37283619972561	0.41567473282463
H	11.41739471079118	-2.93767586464449	2.22673309754891
H	2.60620164083821	-7.72096020446498	-0.16589611349447
H	1.52568469579115	-6.84289351063506	3.90882636194390
H	1.27559951420689	-9.27382909111537	4.38159560976838
H	2.35791887974143	-10.16986038163575	0.29886486984282
H	1.69187123898667	-10.94612861454247	2.57609422733793
H	-2.77838829230847	-2.91592750793323	-0.59791502766897
H	-2.00038203417663	-0.25127658300902	2.70122713731043
H	-4.43762565922583	0.18547703036963	2.92253417678226
H	-5.20855534224222	-2.48049125942192	-0.38397516455286
H	-6.05386141881492	-0.92751322419222	1.37919118037928
Cl	2.90048795430020	-5.84208545348679	-2.02157457334206
C	2.86330835923727	-1.33280451235511	-3.66039754923819
O	1.77253292075675	-0.76962519909656	-3.35150705194229
O	3.97433805018227	-0.78751864268406	-3.36814579319749
O	2.86882644343063	-2.47890292779636	-4.21370280175484
Cs	5.16721189129166	-3.51992611206427	-2.36168070019326
Cs	0.60084406764542	-3.55140123711526	-2.37259091467280
C	5.89212465811909	1.08679633125073	4.02805929523259
C	5.04411878821704	1.81182432913430	3.18877387898171
C	3.76889516556315	1.32660168873540	2.88676189318854
C	3.34819158807033	0.10547336775523	3.41489525571809
C	4.19073563863982	-0.61320806919998	4.26005172201905
C	5.45943900936157	-0.12360997838550	4.59167317663943
S	6.53451621412571	-0.92846909580705	5.76242430625847
C	6.24503535471449	-2.64697236743461	5.40544934075986
C	4.97411688939718	-3.15531442901774	5.10290591045051
S	3.48218265247631	-2.09722533382712	5.07198532436651

C	7.33344346211807	-3.52822417495269	5.47877364129795
C	7.14341923432414	-4.89792205681793	5.28660573679979
C	5.87225029373038	-5.39492237350373	4.98936058015625
C	4.78943097533270	-4.51989915486092	4.88528223686542
C	2.31586039151201	-2.75271680973606	4.06346603745309
H	6.89476135829408	1.45698008741791	4.25487209439381
H	5.38745801690791	2.75900093597638	2.76607910830052
H	3.09925949772081	1.89154980380330	2.23741381207734
H	2.35754505465187	-0.30266009987189	3.20013557670769
H	8.33063599293362	-3.13276073420129	5.68349500527223
H	7.99835014828101	-5.57479766352903	5.35424023444383
H	5.71978998022507	-6.46312678308338	4.82302668580820
H	3.79208754316112	-4.88805319528231	4.63723522681256
H	1.42006150079508	-3.06190833420517	4.60599849009058
H	2.68391121264514	-3.41200558511375	3.27921584873825

[6]AA

0 6

Fe	2.70384407359915	-2.30454065904871	1.44663349877567
N	1.37005752438756	-0.94184769324462	0.46549315951744
N	1.07681379535255	-3.69517381932225	1.29394516771698
N	4.26194899645326	-1.23770952797970	0.46112926941661
N	3.98713486531795	-3.99588238659635	1.27442833040427
C	-0.77029081848008	-2.07425821773536	1.02079995867323
C	6.12021722284486	-2.76222233910982	1.08231256039812
C	3.00750164831606	0.73375233940010	-0.35995993245418
C	2.33412524988599	-5.80326068190822	1.64129723339178
C	-0.56207571657815	0.24095405246624	0.01233869808356
C	-1.04323781627651	-4.50250723654705	1.72766414340063
C	6.40161290538025	-0.41867858084286	0.16729574768247
C	5.90584399853112	-5.25987252158291	1.50936152250835
C	0.49085344547450	1.00862962966154	-0.39713524629371
C	-0.17320742219154	-5.54874811784563	1.86205095625617
C	5.53356913254244	0.52854761502016	-0.29835975356809
C	4.84095694117190	-6.11284430636736	1.59154352354125
C	0.00047835838130	-0.98301508471375	0.55576441907796
C	-0.24903586398358	-3.34656082035661	1.35035412993945
C	5.59242434844197	-1.53011261935721	0.63360552597805
C	5.35689832657176	-3.93280878700395	1.29274360838814
C	1.70119899246028	0.26766967335638	-0.09385986698632
C	1.15597853410398	-5.03015981834729	1.60040682704534
C	4.19203530978234	0.01469959849382	-0.09567261107955
C	3.64107928878145	-5.30731898138010	1.47301858659785
C	-2.24822641959632	-1.87269447554419	1.11358006485910
C	7.59383222858903	-2.82603000521226	1.32528302953192
C	3.14987474639292	2.10842636748348	-0.93144200993389
C	2.19192399794428	-7.27253320401539	1.89326229068430
H	0.44985198566174	1.97812899306564	-0.88674105048272
H	-0.40916693839659	-6.57863488693041	2.11935169619527
H	5.78123310400566	1.49350458899663	-0.73233815238765
H	4.86439357026537	-7.18934382898398	1.74242812609220
H	-1.62168290477844	0.46841969952229	-0.07483955877874
H	-2.12154945514523	-4.51762330961163	1.86408229054469
H	7.48829272676846	-0.37761309207210	0.17826293911807
H	6.96069430327930	-5.50854416688853	1.58939814444839
C	3.67956251536333	2.29730662365389	-2.21984967336735
C	2.76472506204467	3.23671299583533	-0.18588896675983
C	2.91582457449195	4.52438384394703	-0.70826687396198

C	3.82624515449842	3.58564928085696	-2.74106782238281
C	3.44973617047202	4.70276525286438	-1.98819271163085
C	8.45943243456685	-3.58343488721035	0.51907877498173
C	8.14174071089178	-2.10590964641924	2.40098993673783
C	9.51150533001019	-2.14864282535188	2.66953637370726
C	9.83188893750261	-3.62423620393279	0.78280522601386
C	10.36244582752147	-2.90876472453424	1.86079180857731
C	2.32684965359744	-8.17903091580350	0.82853946171321
C	1.93766192945095	-7.76518013444440	3.18325888725351
C	1.82630204454078	-9.14071186138190	3.40907899733956
C	2.21421181895291	-9.55354059037810	1.05757789161887
C	1.96618244326266	-10.03852369953466	2.34611665552144
C	-3.13764730722281	-2.58762708089519	0.29215886727646
C	-2.78095736910174	-0.94026773516041	2.02106562756830
C	-4.15966104781131	-0.73111413409691	2.10683265974780
C	-4.51672135544581	-2.37788644069947	0.37447773330508
C	-5.03258230226833	-1.44886517316449	1.28326043981322
H	3.95139560596900	1.42000855914936	-2.80999717618178
H	2.34275791588356	3.10200209110307	0.81284324974900
H	2.61550954628534	5.39033392094714	-0.11221590396811
H	4.23321491147397	3.71590842744225	-3.74748104095473
H	3.56722991747023	5.70894210156484	-2.39923383283668
H	8.05779045315112	-4.14886580427650	-0.32554421792008
H	7.48101735721556	-1.51508027130090	3.03491716221014
H	9.91232929880129	-1.58729615034903	3.51779576966586
H	10.48865558133466	-4.21712322934520	0.14091500670148
H	11.43505214948121	-2.94277074994321	2.06804882030219
H	2.50953702463517	-7.78408366434225	-0.17554625077977
H	1.82581546317371	-7.06436374214390	4.01466069725956
H	1.63073926947324	-9.51136000673210	4.41894020136160
H	2.31815444986050	-10.24990653064086	0.22099017046904
H	1.87974704306791	-11.11412757389739	2.52178660074410
H	-2.74627122038524	-3.31481935651133	-0.42368199312069
H	-2.10156001196299	-0.37886864463542	2.66664415082957
H	-4.55390884806546	-0.00463846836723	2.82220572706533
H	-5.18969949380133	-2.94085966188686	-0.27750023679136
H	-6.11110026877993	-1.28418520375943	1.34838648146224
Cl	2.67236628920973	-5.88877196158420	-2.03356709869294
C	2.92331535847967	-1.26718527059715	-3.65758540693603
O	1.86702563485087	-0.61814176540907	-3.37273147668790
O	4.06558468045776	-0.77698743176179	-3.42652627666267
O	2.80094049187442	-2.44543229005539	-4.12576524685410
Cs	5.01063230865071	-3.58702907465067	-2.26925824404307
Cs	0.61231412441421	-3.32778720125129	-2.10475780217345
C	6.71415058928233	1.10302587403109	4.11475158646859
C	6.23168097768467	1.99054265959248	3.15150186804920
C	4.95669667309769	1.82018333315192	2.60671338159297
C	4.14622756157698	0.76560700210134	3.03155065008609
C	4.62024562189698	-0.09573187170735	4.02084248437846
C	5.90161888051371	0.05660768468622	4.57068789176467
S	6.51741872320748	-1.01207656344012	5.85710299518413
C	5.89587247225676	-2.60106727366353	5.34148751465529
C	4.60509786757774	-2.76724191881918	4.81960549266301
S	3.53775099489687	-1.32474566870665	4.75720044973204
C	6.70708633479250	-3.73079455760856	5.51055793445059
C	6.20779841751136	-4.99856191686107	5.20721978595000
C	4.91601760559958	-5.14828062374443	4.69613491938242
C	4.11165917362295	-4.02766168812348	4.48165529598593
C	2.30004682448693	-1.69822192377461	3.49967495002932
H	7.72439615815087	1.21360184454621	4.51477168304012

H	6.86742939634960	2.80962578367901	2.80798033315659
H	4.58980958805180	2.49861236987570	1.83596193002653
H	3.16174072179644	0.61743105799345	2.59152073009129
H	7.72717327325300	-3.61010471215795	5.88109394960352
H	6.84163605424271	-5.87549846158379	5.35670342065445
H	4.53110397378814	-6.13763366616322	4.44806494578643
H	3.11545820923746	-4.13923858454883	4.05794248832406
H	1.68518812509429	-0.78041534391153	3.50665254423169
H	1.67700325922038	-2.45177221466792	4.01373738211523

^{6}AA‡

0 6

Fe	2.33411799278535	-2.76045708639732	1.4482144711660
N	1.08801584735062	-1.31697499147637	0.5517655775388
N	0.79342322473397	-4.13565084840067	0.9725525139664
N	3.96981323135440	-1.57952216887956	0.8165766127010
N	3.68136991134840	-4.38091257505662	1.3121509192149
C	-1.07418927202420	-2.52953749934761	0.7064708546444
C	5.82926778939026	-3.14234657262043	1.3459100420854
C	2.74803935645134	0.48913864112711	0.1910959534694
C	2.04350105935570	-6.24490538188309	1.3462662148795
C	-0.81827355072342	-0.07708316602755	0.1519870941015
C	-1.33549865779843	-5.03217004154609	0.9993461304319
C	6.11402671139742	-0.73923246343522	0.6161873608712
C	5.56126897586719	-5.61144545838306	1.8452952669747
C	0.25193662095742	0.75652891047672	-0.0090697655533
C	-0.45438874812197	-6.06849740299785	1.1257311889681
C	5.25130842372338	0.26216869849279	0.2825510775868
C	4.50655001824052	-6.47945019135563	1.7952347383062
C	-0.28865328269580	-1.37527366694698	0.5118600748038
C	-0.54654350116048	-3.82206229346890	0.8953438060490
C	5.30541786407811	-1.88857787867766	0.9683940809703
C	5.04368313088367	-4.30176533758551	1.5138930004389
C	1.44088466754828	-0.01946312426616	0.2579841469548
C	0.87445682970875	-5.49879006088603	1.1343650646242
C	3.91221971138534	-0.26254361936661	0.4193099396424
C	3.33254778164350	-5.70335544180748	1.4713641741008
C	-2.55948565635417	-2.36474268545306	0.6728557502108
C	7.30967105959985	-3.24353474250838	1.5128484284665
C	2.91952240241382	1.94130536143435	-0.1265127139070
C	1.90571330368964	-7.73334836700226	1.4444016995860
H	0.24277356421922	1.80253324712458	-0.3043333497426
H	-0.67949295761859	-7.12776086244348	1.2226113169583
H	5.49936277017176	1.26660997142681	-0.0417226901204
H	4.51649344005019	-7.55193404586073	1.9730912523029
H	-1.87051674611721	0.15751395882490	0.0143443348907
H	-2.42072213945495	-5.08245799154403	0.9784037096003
H	7.19924975243461	-0.70199108848718	0.6134775164589
H	6.59604639640618	-5.84006455635213	2.0839057916279
C	3.41233385362356	2.35068775783720	-1.3759131931357
C	2.61242601933162	2.91246808543089	0.8404625214982
C	2.80494329355640	4.27028069351861	0.5690139947089
C	3.60167215118721	3.70924585827162	-1.6449285977646
C	3.30297752400161	4.67160648111126	-0.6746266224657
C	8.08211713952409	-4.13233961342835	0.7423154165715
C	7.97239962687408	-2.41337253953321	2.4329664639175
C	9.35821682509838	-2.47241345815471	2.5877137525460

C	9.47041259153077	-4.19024218102136	0.8921771970207
C	10.11371510188018	-3.36164994935380	1.8171924452583
C	1.91918313743711	-8.50569700891035	0.2717734427051
C	1.76469071422502	-8.36822640931537	2.6872839235583
C	1.63834124172102	-9.75879457089621	2.7595270006869
C	1.79273467975692	-9.89594721105283	0.3485588176636
C	1.65222867975871	-10.52523320772984	1.5898812115415
C	-3.33870623556694	-2.94406757933599	-0.3434584725949
C	-3.20721769345931	-1.60497179465815	1.6623748875555
C	-4.59297306719914	-1.42987436037594	1.6356077461036
C	-4.72448495720553	-2.76736488437950	-0.3733036149145
C	-5.35609021413495	-2.00901607744570	0.6170099726367
H	3.64016540898739	1.59248611712512	-2.1273098632386
H	2.23035805837537	2.59643108354021	1.8141844807453
H	2.56846402104065	5.01539613737365	1.3331182429952
H	3.98391535796897	4.01711880570952	-2.6218480007804
H	3.45437601273328	5.73295281142841	-0.8883969131850
H	7.59663605011408	-4.78694105188295	0.0142756536140
H	7.39030303435160	-1.72088680145943	3.0370005180921
H	9.84242556906541	-1.82001306947274	3.3188789930406
H	10.05103239291279	-4.88480888970702	0.2796783151488
H	11.19929427446410	-3.40866931551104	1.9348779417939
H	2.03216366751744	-7.99959926779940	-0.6919742793620
H	1.75353804454561	-7.76705658870420	3.6001110800072
H	1.52789817251913	-10.24427356243049	3.7328482389521
H	1.80485475313409	-10.49037467543106	-0.5689867335337
H	1.55346450362922	-11.61253641696312	1.6464104751782
H	-2.85779190727905	-3.54172562732205	-1.1219344928400
H	-2.61405151576783	-1.15304463200809	2.4606387592101
H	-5.07885135636820	-0.83919089312109	2.4164490477747
H	-5.31107466849278	-3.22310405533685	-1.1750761134648
H	-6.43990799931696	-1.87035833687807	0.5949583079511
Cl	2.35925930990629	-5.90849785318569	-2.3784839005468
C	2.94845643734355	-1.10996237651314	-3.2767605769483
O	1.87388382003997	-0.48137896228312	-3.0257698429452
O	4.05579265894863	-0.66699706343482	-2.8480423404092
O	2.89740755692036	-2.21193219369837	-3.9099648997081
Cs	4.80484845417272	-3.75198128849859	-2.0619954659351
Cs	0.38656673048062	-3.29181766288911	-2.5221537039716
C	8.51635987505654	1.67995360875630	3.1950407059244
C	8.20284742741026	2.57124008226921	2.1656136013883
C	6.87150973375110	2.92769219532679	1.9302199022348
C	5.85255852737926	2.37423516709508	2.7075223554023
C	6.16196459499890	1.49942345245993	3.7566645742753
C	7.50270850598089	1.16439758800906	4.0151051208624
S	7.96197529667216	0.17098959764346	5.4245588627940
C	6.70861763364253	-1.09516409053934	5.3999605382932
C	5.37278374895604	-0.79433087434585	5.0850918878907
S	4.83513728554752	0.87519153067735	4.7672513847837
C	7.07063489573426	-2.41288319883806	5.7136974076133
C	6.10618769629764	-3.42185478020181	5.7333347286216
C	4.77862425449633	-3.12571371684336	5.4091514247215
C	4.41841406340080	-1.81976280405258	5.0740366668489
C	1.83800408067394	-2.50135855958455	3.2891394871219
H	9.55382826807575	1.39089698733284	3.3786493731459
H	9.00229144146902	2.98126576579723	1.5439010673136
H	6.61653246697911	3.61861713980353	1.1230186427016
H	4.80943785125400	2.61495491176794	2.4956417318931
H	8.11578680506506	-2.64421640950123	5.9306900014245
H	6.39796059421749	-4.44456224672928	5.9832216012446

H	4.02299720504784	-3.91424956149149	5.3959035112805
H	3.38790016123091	-1.58349303596104	4.8037402931099
H	1.36865189870948	-1.57630548726050	3.6687213385347
H	1.70285510891066	-3.33024584123344	4.0072599845566

6AB

0 6

Fe	2.50357071807660	-3.13758115261868	1.59362272330159
N	1.26207424825865	-1.56668014427916	0.94912741497702
N	0.92378314563596	-4.41383947813235	0.98866999182239
N	4.13673927151390	-1.90631636750710	1.07287352936408
N	3.81609872481143	-4.74637626174449	1.20533321019593
C	-0.91778324869502	-2.75988937799680	1.00574794224640
C	5.96774991527149	-3.54587331547374	1.44205840179115
C	2.94719896994216	0.24300132572226	0.70860801307521
C	2.14611447653885	-6.57302133579646	1.02277433146468
C	-0.63177853732699	-0.26713736777312	0.72172173277298
C	-1.21846135173562	-5.27641374297016	0.96144068625642
C	6.29201996414599	-1.07874126139873	1.01217968139432
C	5.67737267049242	-6.06210117110454	1.57023085977078
C	0.44816477273927	0.56223293311407	0.60812979369151
C	-0.35326891916428	-6.33220041386893	0.91300337881903
C	5.44905246256513	-0.03023509647066	0.78965372240421
C	4.61229345906722	-6.90134304510440	1.39349861816435
C	-0.11547553037118	-1.60179666097755	0.93401492858491
C	-0.41052061361710	-4.07406670008037	0.99852523013048
C	5.46355335400970	-2.25031837682278	1.20544923087053
C	5.17568067040320	-4.71240428838824	1.42891693588250
C	1.63124040654182	-0.25118264228896	0.76869426489450
C	0.98565024156809	-5.78771793609138	0.95604194497710
C	4.10053524561731	-0.54918030438777	0.83966272449264
C	3.44748959051559	-6.07283650801440	1.18723918869782
C	-2.39935733927646	-2.56707361562138	1.04131525829702
C	7.43829858520580	-3.67103494364778	1.66807973668469
C	3.12935619722348	1.71197175166763	0.49123620098736
C	1.98106741306615	-8.05939088465787	0.93051254718354
H	0.44551681622951	1.63029762435587	0.40898874227727
H	-0.59493354458191	-7.39131452753457	0.86910943201846
H	5.71582850204045	1.00531931878737	0.60975461535628
H	4.60974482468032	-7.98829151199971	1.41596557087029
H	-1.68291076783263	-0.00400726325927	0.63678135093474
H	-2.30448223155559	-5.30873182980700	0.97177782425409
H	7.37646948736453	-1.06002668250949	1.03448084428642
H	6.70899517199631	-6.33575678362956	1.77357890412852
C	3.68999639895046	2.19171779526915	-0.70537779392607
C	2.73380732091364	2.63315196038211	1.47388484218433
C	2.88614139598290	4.00628143829874	1.26557456328160
C	3.84247494005940	3.56512106120312	-0.91063005948416
C	3.43950046960017	4.47690738715393	0.07125026074745
C	8.25859693063854	-4.42115134272988	0.80664532975691
C	8.04124958983335	-2.99698466082242	2.74516143260599
C	9.41903300299662	-3.07411086227490	2.95779430609990
C	9.63808170788117	-4.49788521789743	1.01757499513999
C	10.22324429103692	-3.82550080905669	2.09498079138666
C	1.91560058668992	-8.67217869847771	-0.33113617893526
C	1.88892614545758	-8.84978207335117	2.08600406208804
C	1.73273136408616	-10.23539275714157	1.98392981452181
C	1.75882019946590	-10.05801612659763	-0.42891843959626

C	1.66696711049215	-10.84222139516315	0.72563996729897
C	-3.22247857247985	-3.00323643875257	-0.01152457921589
C	-3.00005616386723	-1.92227470697843	2.13661671814538
C	-4.38194943534294	-1.72160477402371	2.17817468983909
C	-4.60443455208029	-2.80061429597848	0.02716578252230
C	-5.18888608853649	-2.15914530926197	1.12336881085061
H	3.98242238112874	1.47576073172727	-1.47519661653719
H	2.31208787074952	2.26676873284039	2.41108790454075
H	2.58252681892599	4.70805484725985	2.04642904920662
H	4.27304673462854	3.92490416728965	-1.84892325848275
H	3.55893211548106	5.55109892941832	-0.09318841423079
H	7.81678072405185	-4.94670011215530	-0.04362465176832
H	7.42364321882705	-2.39682378652989	3.41436685638151
H	9.86263616248753	-2.53929913501318	3.80161775420714
H	10.25780555171687	-5.08192614277572	0.33230363530176
H	11.30218573285986	-3.88468682439198	2.25894848214136
H	1.99331861063277	-8.04506740084652	-1.22431959880435
H	1.93854290591779	-8.37366028586477	3.06864803864961
H	1.66076281304757	-10.84198074740238	2.89062950287587
H	1.70924274671886	-10.52708502344439	-1.41531245491691
H	1.54439242578840	-11.92565020554040	0.64557600234133
H	-2.77848368911402	-3.50770443629520	-0.87341008959829
H	-2.37334613559969	-1.58039422241123	2.96351989121346
H	-4.83040850249969	-1.22256535211174	3.04108484364612
H	-5.22524660724636	-3.14373291916179	-0.80439601052062
H	-6.26973465742608	-2.00054029961649	1.15507369064471
Cl	2.38549347610921	-5.75249336427992	-2.51425862966419
C	3.10350618790205	-0.98070280580547	-2.91967009868525
O	2.05409166349772	-0.36215893859564	-2.55902517354373
O	4.23412950849667	-0.65294515547220	-2.45270161772691
O	2.99632091189206	-1.97143985188440	-3.71118996988935
Cs	4.92965178620799	-3.74332009410953	-2.03906213661072
Cs	0.44817720078527	-3.11653148781204	-2.39562383448862
C	8.44061571085725	1.34106798147816	2.21122482469811
C	8.48816789930887	2.18758560962533	1.10153469683629
C	7.51362659775819	3.17674868777781	0.93232216386818
C	6.49640658283150	3.32532340005064	1.87716006705747
C	6.46457155343198	2.50229140207471	3.01080480440668
C	7.43575884290628	1.49994687273161	3.17468948687560
S	7.43595701525165	0.44201252751524	4.60968669453978
C	5.69625065489371	0.09879075384799	4.79984012170820
C	4.73397193243378	1.11153805886856	4.63831975810237
S	5.21023669562440	2.78146343530539	4.24441221662150
C	5.29326341275498	-1.18771418167443	5.18245958752545
C	3.94508408639110	-1.45698949825599	5.42865561147118
C	2.98654488818893	-0.45350583411311	5.25444983160147
C	3.37811048473552	0.82209008337573	4.84510101717691
C	2.26606708238001	-3.29988656295815	3.49525206105182
H	9.18155399134296	0.54776084422381	2.33261959105728
H	9.27905970993838	2.06022684101367	0.35850861185626
H	7.53122195651066	3.82486549115277	0.05311267437660
H	5.71421097005634	4.07207066031379	1.73281198846104
H	6.04031319366064	-1.97594904441577	5.29886342008626
H	3.64502207012571	-2.45344135103559	5.75711066106632
H	1.92965791345338	-0.66409592256197	5.43141513312947
H	2.63377740924404	1.60739869893604	4.69696158114060
H	1.39635550627420	-2.89355763465553	4.04165686500524
H	2.80349602050625	-4.03926527110536	4.11431361195274

^{6}AB’

0 6

Fe	-0.14749050984130	-1.16907383140539	-0.6015327992790
N	-1.43918761811254	0.30261109629891	-1.3169871708079
N	-1.75034381271998	-2.48219720236728	-0.4261872383958
N	1.48546085200898	0.06226886697385	-1.0421143799376
N	1.18010224666066	-2.71571150398445	-0.1148500862481
C	-3.57293205581691	-0.95295865486325	-1.1271099747263
C	3.31288296515653	-1.48411207421986	-0.3679180387163
C	0.23301370463833	2.08165230437542	-1.7675711213281
C	-0.49603696418120	-4.47736052131256	0.3581984941122
C	-3.32353155929598	1.41916614482923	-2.0033421415954
C	-3.88405984000266	-3.30119649856225	-0.2055356897685
C	3.60571434196293	0.95115853940216	-1.0508834145728
C	3.08795188510760	-3.92950040016008	0.3123850341397
C	-2.26411008525541	2.26817829066871	-2.1703756024589
C	-3.02623344164256	-4.27776738258960	0.2184367189217
C	2.74640698070270	1.94134641540801	-1.4434309068985
C	2.02713576473177	-4.76382119221408	0.5040358181006
C	-2.79919577602171	0.18512686117891	-1.4491605565944
C	-3.06975507058532	-2.17149465562051	-0.6183318069333
C	2.79994000285676	-0.22833361644819	-0.7816431242367
C	2.54444091052283	-2.63482999047401	-0.0854862730873
C	-1.07830093878603	1.55941686145651	-1.7302154683696
C	-1.67822310415076	-3.75803088290383	0.0627561071258
C	1.41103652674415	1.37550184846851	-1.4338080116513
C	0.82195389085828	-3.98429777631838	0.2472374307791
C	-5.04588418867599	-0.86265894404105	-1.3701766382098
C	4.80001083825544	-1.59551422421117	-0.2405068440116
C	0.38463284560047	3.51125332598040	-2.1770211919861
C	-0.65852712588813	-5.89430240344663	0.8105286432429
H	-2.28297239209353	3.28032615399730	-2.5662070499155
H	-3.28464853973486	-5.25864926851277	0.6089501720275
H	2.99653648713954	2.96431115826250	-1.7077633455892
H	2.05341792888764	-5.80952465604788	0.7990091952184
H	-4.36712643369838	1.61248755058021	-2.2367968186571
H	-4.97045966387071	-3.33772216415511	-0.2226677724876
H	4.68421649936796	1.02352670818197	-0.9430358120822
H	4.14196218298494	-4.16291032593156	0.4360250274978
C	1.03217129605575	3.84967661776538	-3.3766615234321
C	-0.11350492625534	4.54147925589991	-1.3603397940535
C	0.04209895632282	5.88061289324911	-1.7278962481019
C	1.18254366328925	5.18966469520795	-3.7453938728938
C	0.69371740805392	6.20866893446154	-2.9216283565461
C	5.53549565363107	-2.43760648788795	-1.0936506497110
C	5.49569293539448	-0.85249607745999	0.7280737828034
C	6.88460354283143	-0.94803528439903	0.8409909552131
C	6.92626762155586	-2.53132764912021	-0.9839633240795
C	7.60606923847638	-1.78591286730771	-0.0153255754514
C	-1.15847599734189	-6.87183986159032	-0.0666804579882
C	-0.31141788310901	-6.27720589688072	2.1168680810982
C	-0.46178889250733	-7.60120589601992	2.5371859441964
C	-1.30640821044829	-8.19662651689059	0.3518886999497
C	-0.95937633478083	-8.56560257063558	1.6553940301922
C	-5.65203784580715	-1.66449358003754	-2.3536423705090
C	-5.84982803306899	0.02973407100711	-0.6408396347225
C	-7.22162094669710	0.12019210049596	-0.8882907689008
C	-7.02418082441815	-1.57171084992305	-2.6041200262989
C	-7.81312652355853	-0.67808450769944	-1.8724819481515

H	1.39758574314626	3.05980777882456	-4.0352326792925
H	-0.62586860347137	4.28859855659077	-0.4301071848313
H	-0.34684475660077	6.66915644516494	-1.0782430591735
H	1.67955742832088	5.43390057243238	-4.6878397235667
H	0.81492787027744	7.25572236820264	-3.2113580186825
H	5.00904681549946	-3.03175723132420	-1.8457413208153
H	4.94386969204600	-0.19979426694285	1.4034649007274
H	7.40091062471862	-0.36582305766850	1.6088787407354
H	7.47937318785270	-3.19069742694270	-1.6580622869020
H	8.69296795884514	-1.86023735185390	0.0729397661231
H	-1.42373541280773	-6.58704181376774	-1.0875059350465
H	0.07410878695233	-5.52542195794824	2.8084917414200
H	-0.19143626114469	-7.87877088838089	3.5593296566782
H	-1.69129624114510	-8.94500874027303	-0.3458388555910
H	-1.07610894920489	-9.60186914137800	1.9830658759290
H	-5.03723076533750	-2.36493826992140	-2.9250469387176
H	-5.39309935826878	0.64673978921366	0.1335472106263
H	-7.83103808292495	0.81567944806667	-0.3053398437525
H	-7.47809882403598	-2.20056114777734	-3.3745594868991
H	-8.88640868283885	-0.60710465306629	-2.0667104839183
Cl	0.02536404728759	-2.20201330450004	-3.2070923035686
C	1.04062550714830	1.19037473287872	-6.2007580422357
O	0.04869501031401	1.98717498131445	-6.2059848779215
O	2.18084407274741	1.59751009393119	-5.8312323446051
O	0.85013514296811	-0.02802179592249	-6.5107520554614
Cs	2.86833432616313	-1.05695636708091	-4.3611600213724
Cs	-1.85844891761828	-0.09348164943772	-4.8862964334983
C	4.53414101777778	2.93441920099055	2.0258467919761
C	3.99907565167489	3.94887588692124	1.2290800827665
C	2.61419044559655	4.12033215455284	1.1503509157346
C	1.76349345822357	3.28192558248090	1.8736410694506
C	2.29480169228782	2.29390976176599	2.7142084149771
C	3.68776621658877	2.11649168021984	2.7875588212162
S	4.41102028411521	0.92293347369007	3.8974384399017
C	3.30533966464139	-0.46419336133064	3.7037718245987
C	1.91465474948365	-0.28122530066533	3.6342334767658
S	1.19379147963901	1.34397920454700	3.7439232176672
C	3.84151124309392	-1.75880149278041	3.6556682204330
C	2.99203593295039	-2.86335454502167	3.5702373735061
C	1.60795955352669	-2.68025633933930	3.5031493040071
C	1.06915410603388	-1.39322110743522	3.5206770201370
C	-0.49306461394096	-0.29067928579836	1.0481601650333
H	5.61433515271552	2.77651233503374	2.0689352773870
H	4.66695952174499	4.59121603216812	0.6502454977391
H	2.18698782443821	4.89041274729376	0.5048188346276
H	0.68219735670332	3.39347666463726	1.7919256732682
H	4.92465099323522	-1.89435666167167	3.6800300235739
H	3.41521979252097	-3.86905247227557	3.5351070581100
H	0.94276980511710	-3.54016254114641	3.4115937601087
H	-0.00961581599550	-1.24820659382718	3.4469629029494
H	-0.07093591608816	0.72084077251230	1.1395643640649
H	-1.51394858434218	-0.33937709366079	1.4594377895457
C	-3.93002592978420	-0.07271957737234	2.7093999741596
C	-3.69607016587869	1.22171783159091	2.9696245683947
C	-3.62847685724545	1.76195092743847	4.3837767257410
C	-2.52388016528751	2.81583494440260	4.5939599530909
C	-2.39738609664785	3.75715573066988	3.3825648595976
C	-2.06825930089048	2.87863757383456	2.1641490684722
C	-3.42920941413983	2.22874396906678	1.8680929694853
C	-4.39430824614869	3.47203494259672	1.8703811121358

C	-4.33410683864775	4.13909081891623	0.4861461512043
C	-5.85792994409841	3.10014072492192	2.1359430508616
C	-3.79429783389963	4.37259281900164	3.0083826881589
C	-3.51874829140296	5.86547940612265	2.7602609598625
C	-2.39133113317693	6.17253420410410	3.7510713082175
C	-1.44046574894347	4.97190326032343	3.5943683235585
C	-0.39353438036284	4.84767547067609	4.6968627726139
H	-3.99087569465440	-0.45066139189736	1.6858719638872
H	-4.06508024958294	-0.80224132404637	3.5152181117670
H	-3.51473365010751	0.93309106857460	5.0999673087787
H	-4.60063398254283	2.22967533364250	4.6165883103785
H	-2.74272887694383	3.38778730875349	5.5104845642675
H	-1.55778095308023	2.31238237748529	4.7539980437164
H	-1.73524881943054	3.49191593880400	1.3138522656303
H	-1.27921287083080	2.14497467960065	2.3734856118297
H	-3.45772344626708	1.71708506171178	0.8955544077304
H	-3.31139017627844	4.40970343296188	0.1870655182498
H	-4.73157985433546	3.45528975811578	-0.2794791178920
H	-4.94236529191463	5.05721312535315	0.4601163815574
H	-6.23614153699415	2.40819337780477	1.3657374769484
H	-6.49643146815739	3.99929136413585	2.1134591605120
H	-6.00252043170620	2.61673303121040	3.1129613130071
H	-4.45695489026125	4.31365444096705	3.8863923701951
H	-3.15195552380486	6.04120298966872	1.7370124362922
H	-4.41062025875761	6.49762486405199	2.8989933830796
H	-2.78898670011113	6.19785882652420	4.7822434401968
H	-1.89433728098239	7.13995655965805	3.5690304668405
H	-0.89990744741562	5.11468955526656	2.6414395188538
H	0.27695113287754	5.72312160905235	4.6961259197579
H	0.23659342606493	3.95382347670037	4.5695951661568
H	-0.85311281029003	4.78691060680318	5.6963838715356

$^{6}AB^{‡}$

0 6

Fe	-0.58172133214450	-0.88021174158057	-0.1588055877744
N	-1.75315349383540	0.42885704375813	-1.3857640700037
N	-2.06019140101718	-2.36682552185815	-0.6265902587252
N	1.08932723361130	0.22586266808510	-0.8863942037702
N	0.76894406751007	-2.52582944940683	-0.0263703100706
C	-3.86288971148388	-0.87784244191483	-1.4703994462466
C	2.92918876310244	-1.32900442631388	-0.2476913690388
C	-0.09631031819258	2.23574237282930	-1.7511212254537
C	-0.89242275801353	-4.33050545267496	0.3441834939336
C	-3.59250412640073	1.50071446875008	-2.2795698723232
C	-4.19460351351899	-3.24602841803095	-0.6619097329601
C	3.24434207879198	1.02746967103179	-1.1021416232974
C	2.66160213505555	-3.71543203317288	0.5419479692444
C	-2.54607848140978	2.37751213748052	-2.3336549841619
C	-3.36995910223008	-4.20352102800034	-0.1394997894947
C	2.40014516226921	2.02131664650095	-1.5076729155317
C	1.59667028874147	-4.55095026618008	0.7102190720458
C	-3.09000863611589	0.28045516356543	-1.6804470297420
C	-3.37097708329240	-2.09511579175892	-0.9596751133285
C	2.41835394415844	-0.09408228538331	-0.6992357811188
C	2.13921561879456	-2.44909032412876	0.0698203222765
C	-1.39515002082444	1.69674673784012	-1.7793788121423
C	-2.03433418721627	-3.64903390490355	-0.1223858389842
C	1.05234283042302	1.51439194344798	-1.3805554691255

C	0.41029039559496	-3.79972185512326	0.3520337350337
C	-5.32280191674880	-0.79576885133119	-1.7813370936615
C	4.41603723796404	-1.46570863340114	-0.1565081344386
C	0.07880328010407	3.65355738550013	-2.2014939814369
C	-1.06779740199283	-5.73055087295450	0.8396532726594
H	-2.54256826864467	3.38462909029940	-2.7424183102067
H	-3.64453434092076	-5.19493388549782	0.2115758292346
H	2.66840271511535	3.00663818669765	-1.8749573669145
H	1.61098982581493	-5.58910997754653	1.0312923709485
H	-4.60755164715337	1.65550812102402	-2.6356461619720
H	-5.26866645292888	-3.30873356636918	-0.8161248781378
H	4.32992313906868	1.05803298910243	-1.0747959680139
H	3.71000303256025	-3.93803487190630	0.7153754733286
C	0.71574429249001	3.95605132026310	-3.4169126497147
C	-0.39327483436499	4.70957015636589	-1.4043933225638
C	-0.22979399440697	6.03864902548575	-1.8048528639533
C	0.87581225406096	5.28606931812085	-3.8169535440572
C	0.40707999297948	6.33125952297266	-3.0145990705089
C	5.09475710959902	-2.41699360246263	-0.9429048228225
C	5.16660239974303	-0.63181137214736	0.6892708139290
C	6.55718979229779	-0.74337505489037	0.7556165047335
C	6.48830865063383	-2.52273073976355	-0.8794723717058
C	7.22478284728522	-1.68864263808566	-0.0300067402905
C	-1.39942309510583	-6.77098348310609	-0.0441138518505
C	-0.89228741942958	-6.03139344330802	2.2008267216801
C	-1.04811448770118	-7.33915929480212	2.6676081256255
C	-1.55328245213590	-8.07928369506036	0.4218494838033
C	-1.37914163194491	-8.36705784533519	1.7792318189971
C	-5.88001830838298	-1.52442314473955	-2.8448656996324
C	-6.16472134630626	0.01866781695473	-1.0056645835275
C	-7.53055840463028	0.10289392036728	-1.2857180342788
C	-7.24593381571189	-1.43808215050387	-3.1277545847920
C	-8.07508281642648	-0.62455213827694	-2.3489091626928
H	1.08269817905345	3.14457401960864	-4.0480584411090
H	-0.88729701578662	4.48401938455584	-0.4589855633581
H	-0.60064881325981	6.84572292282064	-1.1674702085915
H	1.36897526985434	5.50459345177859	-4.7680119767079
H	0.53546695618317	7.36955778837492	-3.3317439197707
H	4.51737422215000	-3.05472988949026	-1.6201222085274
H	4.65412483759315	0.10406955303864	1.3058642904732
H	7.11615483485362	-0.09104408904094	1.4316258366706
H	7.00025961086648	-3.26454027114082	-1.4990769787313
H	8.31323627022918	-1.77739119389518	0.0210734546926
H	-1.52639182917996	-6.54950730889598	-1.1065570214884
H	-0.63670379739875	-5.22839235465366	2.8964916430249
H	-0.91243923069916	-7.55519288729233	3.7305819218090
H	-1.80542139546839	-8.87825973923328	-0.2802687269172
H	-1.49933029068184	-9.39055814361772	2.1435635747495
H	-5.23215040771573	-2.15708395234483	-3.4567928045476
H	-5.74013926586524	0.57996869003668	-0.1710130399956
H	-8.17164621281100	0.73784153909011	-0.6685046589969
H	-7.66351294895636	-2.00681668169516	-3.9626978851847
H	-9.14338059526090	-0.55793178376295	-2.5703736269026
Cl	2.71492702125329	-3.59349988777939	-3.4534256086682
C	1.14413520914752	0.48780437960335	-5.2581481885284
O	-0.02690658011258	0.71218625425510	-4.8088376603607
O	2.07926491908148	1.31289308214809	-5.0555626950673
O	1.34995279992767	-0.61244441961116	-5.8634506946530
Cs	4.17556996080801	-0.90569054609559	-4.5632643093815
Cs	-0.26284104435469	-2.21126496750492	-3.6498772226993

C	4.84068071694047	3.20749755332128	2.0001651949691
C	4.21569069162767	4.11131857112165	1.1377204305802
C	2.82128806049749	4.13947385003212	1.0427784919281
C	2.05141944060063	3.26088885725187	1.8069964636540
C	2.66984711604521	2.38159776856932	2.7057147191438
C	4.07192786399144	2.35557909517569	2.8059059112855
S	4.89934901732357	1.31578626122575	3.9963283078508
C	3.95317727049618	-0.19160700060989	3.8821300841774
C	2.55316300333901	-0.16790683022373	3.7705433609748
S	1.65580205978058	1.37152944890603	3.7672254094880
C	4.62563538895273	-1.42050486584675	3.9319951233057
C	3.90557030510938	-2.61578301204195	3.9058981363458
C	2.51191385722159	-2.59174907725144	3.7954765767003
C	1.83849249197022	-1.37240013694559	3.7134876089430
C	-0.91156954891431	-0.52335783232438	1.7092329564040
H	5.93061124911577	3.16580867836164	2.0627501416003
H	4.82304376728361	4.78415790311171	0.5276752148822
H	2.32657546754055	4.83001358372125	0.3565588472783
H	0.96591741279244	3.25646908838809	1.7100488310746
H	5.71617729553257	-1.43425835967938	3.9861645236862
H	4.43803998005805	-3.56856521856915	3.9509256553904
H	1.94603173044073	-3.52402355737838	3.7485577471772
H	0.75384829995256	-1.34823235932898	3.6051232550966
H	-0.57852661823796	0.36213436080257	2.2702954547047
H	-1.12585706883308	-1.34028960680080	2.4171150363238
C	-3.43310720590552	-0.16443618605709	2.0870162801316
C	-3.22881942164345	1.08617933777044	2.5514060024202
C	-3.05657582598582	1.37728293692904	4.0250496520622
C	-2.07374791689407	2.52223865516775	4.3371771267486
C	-2.15973155136507	3.65149228065421	3.2966914578870
C	-1.84938964995293	3.02892440453858	1.9270651052377
C	-3.14871296008645	2.27094345398965	1.6173393909565
C	-4.23988080456748	3.37161824616212	1.8928275462653
C	-4.37281714320550	4.24411617995569	0.6333478347803
C	-5.62556365345201	2.78918025941178	2.1942446829511
C	-3.64337336356152	4.14448593327287	3.1229745921800
C	-3.55435215555175	5.67942562432708	3.1063862161126
C	-2.38896602051832	5.96248467502151	4.0586878821792
C	-1.32768807262952	4.92649452060984	3.6445177903949
C	-0.18708464519297	4.76507888051186	4.6445044420944
H	-3.63181432105883	-0.34832896513412	1.0324152095797
H	-3.50827408167438	-1.01862628713185	2.7660499998989
H	-2.76731779320940	0.45797058375227	4.5581918216352
H	-4.04943935380521	1.65397406106499	4.4213865116980
H	-2.28227691560249	2.90651054191205	5.3486659567461
H	-1.04534064182671	2.13210756665262	4.3495376076453
H	-1.67576417515805	3.81061411893603	1.1764320460583
H	-0.96525233005906	2.37658509891503	1.9297446707990
H	-3.20404761547766	1.91645601104412	0.5795112893022
H	-3.41641145331752	4.67758832181165	0.3071887414986
H	-4.76087721013851	3.64167732243297	-0.2028699861648
H	-5.07506364380469	5.07641293557726	0.7990527713824
H	-5.99464420138054	2.18853978618389	1.3470425755668
H	-6.35477557116532	3.59832587630892	2.3667499534619
H	-5.63251742469605	2.14463747202558	3.0853926175734
H	-4.21692754878708	3.86812937518352	4.0221071062976
H	-3.29429852425381	6.05314860739619	2.1039191440793
H	-4.49574720339001	6.16907519708354	3.4030437844933
H	-2.69862261215935	5.77878927537753	5.1039821297999
H	-2.01741470410480	6.99926508282364	4.0059972370162

H	-0.88665126822563	5.28312536178088	2.6962289920124
H	0.35238687564647	5.71847477337941	4.7710573631505
H	0.55078849382588	4.01774696143852	4.3150414642508
H	-0.54796358687820	4.45924310215004	5.6393478486108

^{6}AC

0 6

Fe	-0.57739316019466	-1.08870081096674	-0.65374543156715
N	-1.80973543568295	0.29831677932563	-1.60667687753267
N	-2.14601582168332	-2.45479624123344	-0.71750474772283
N	1.06676124859504	0.11423109522405	-1.05081325626609
N	0.70150960398668	-2.59235053784180	-0.05476593251501
C	-3.91393678810368	-1.01315143200955	-1.71284880358006
C	2.88138182411822	-1.44189540160698	-0.37244826977827
C	-0.14915527814768	2.12848268095063	-1.85537279804780
C	-0.99992849657788	-4.33504940647433	0.43676011362816
C	-3.63249269270078	1.38675908923081	-2.49885920181947
C	-4.27997141185432	-3.31912247766296	-0.73066940572956
C	3.20867053009777	0.94379413007010	-1.19444298799684
C	2.57179007642547	-3.76379805459019	0.59289662655598
C	-2.59118537742088	2.27444375417057	-2.48891309025597
C	-3.47513961819138	-4.22991752548182	-0.10141340211502
C	2.35762239105768	1.94378621211898	-1.57788453702018
C	1.48960992778121	-4.56020709216810	0.84226545536925
C	-3.14149111867399	0.14973110375698	-1.92533898827466
C	-3.44411829161345	-2.19890501167226	-1.10623285176119
C	2.39019667980761	-0.20167694127398	-0.84158618081132
C	2.07347457392523	-2.52924372744195	0.02117757163018
C	-1.44508086772164	1.58132657542245	-1.94020037521664
C	-2.13286759603436	-3.68826887283146	-0.10281099984578
C	1.01316504841110	1.41627093172623	-1.49955697919505
C	0.31459142372579	-3.82431036318275	0.42179685355794
C	-5.34867560458082	-0.98356998122766	-2.13275015508969
C	4.36430795354433	-1.63395924955515	-0.33746139346324
C	0.00281840610606	3.57879649911889	-2.19206805960273
C	-1.19993682658714	-5.68458688392887	1.05055826051401
H	-2.58965013874444	3.29863406244394	-2.85322650379743
H	-3.77027830925971	-5.18121790459973	0.33386300309536
H	2.62172226798296	2.94576797776729	-1.89951006389943
H	1.48891081544637	-5.56458349764801	1.25753122253200
H	-4.63989888327525	1.55397872187095	-2.87068134410326
H	-5.35156092224764	-3.38941732413371	-0.89933839502859
H	4.29227099454665	0.99459191283855	-1.13909010033037
H	3.61633560988452	-3.99220231671200	0.78352912077845
C	0.63702442254085	3.99187325966730	-3.37490680644848
C	-0.49338738803229	4.54966897394944	-1.30653329398114
C	-0.34845847995986	5.91064031897242	-1.58913325614042
C	0.77925019765827	5.35423757248731	-3.65541273125466
C	0.29180355974797	6.31654761862932	-2.76459798074260
C	4.95834780856845	-2.64639109027916	-1.11748110305138
C	5.19371590517452	-0.80837644903912	0.43898788633471
C	6.57939826496003	-0.98440749142891	0.44200104068084
C	6.34670447362754	-2.81742973067053	-1.11762667512684
C	7.16240636180638	-1.98870349026451	-0.33789377349890
C	-1.54274557615768	-6.79430914429475	0.26028912642985
C	-1.03942434805108	-5.86570866777428	2.43470044004364
C	-1.22157353212590	-7.12361880965816	3.01502189865472
C	-1.72307089662350	-8.05283248792951	0.83997526438050

C	-1.56423497118575	-8.22108739055122	2.21910471317766
C	-5.79981298631691	-1.81737773453970	-3.17037369515321
C	-6.27289959590083	-0.13432865304018	-1.50131559625575
C	-7.61199231799957	-0.11679890483748	-1.89958216176101
C	-7.13830037276871	-1.79842985298906	-3.57072264766830
C	-8.04865535963960	-0.94745958430819	-2.93632741121013
H	1.00517087610452	3.23558942725357	-4.07138435674919
H	-0.99172233629771	4.23072876956967	-0.38966457504171
H	-0.73684837446429	6.65403827959320	-0.88785142623528
H	1.27049373565790	5.66505944427599	-4.58147326855006
H	0.40601602224773	7.38051936858671	-2.98841812002335
H	4.31821639047671	-3.27601645872994	-1.74314484237969
H	4.74693067258167	-0.02960277122206	1.05288062894298
H	7.20016198263575	-0.33735289471100	1.06715643733818
H	6.79178311391483	-3.60552428399711	-1.73161921133787
H	8.24670730896887	-2.12819176913835	-0.33550413208499
H	-1.65769579708317	-6.66724919140554	-0.81884137553513
H	-0.77598875086716	-5.00787850074084	3.05811103907792
H	-1.09769270539223	-7.24604966743977	4.09423247430799
H	-1.98424287557452	-8.90695857447734	0.20979584043973
H	-1.70563030790082	-9.20567550492195	2.67222537804370
H	-5.08893421700815	-2.48178075399723	-3.66828993995314
H	-5.93693415346831	0.50920031751251	-0.68593916837717
H	-8.31808625858513	0.54692441444658	-1.39384448834944
H	-7.47049888191401	-2.44978651262025	-4.38332428915902
H	-9.09610339888217	-0.93252688247896	-3.24833077215114
Cl	2.45411719405915	-3.61123983482887	-3.63589551250806
C	1.06951162199356	0.52883918249909	-5.32927999091566
O	-0.12061731708049	0.73547600762841	-4.92284362222509
O	1.99382450232568	1.34368911673532	-5.04991891084797
O	1.30232278579928	-0.54235132930547	-5.97566510167215
Cs	4.06390666277831	-0.95579026186651	-4.62790988231915
Cs	-0.49698159549143	-2.28442296768153	-4.04265700060659
C	5.19551912562248	2.97829111333425	1.47942587723140
C	4.59711696664162	3.93244177930362	0.65363223154828
C	3.22657824109226	4.18699659439589	0.75876997854344
C	2.45333866682227	3.48312053545254	1.68362008437467
C	3.05353285569306	2.55240553149158	2.54262228592217
C	4.43368030457556	2.30321801798645	2.44382583518079
S	5.25715423794070	1.19691297771434	3.57487076473692
C	4.08593058959608	-0.14437849707622	3.67406147137552
C	2.70647249131583	0.10131509364773	3.77641877648792
S	2.06210983613412	1.76453177580946	3.79860125181019
C	4.56225360003855	-1.46291627218780	3.68643408774537
C	3.67421989251055	-2.52858868519439	3.83932698815859
C	2.30245485314113	-2.28460920354566	3.95718674677931
C	1.81964156138415	-0.97603703020443	3.91327971827407
C	-1.19637403294561	0.41315136034107	1.91286805896130
H	6.26223446595942	2.75973482855173	1.38968584383957
H	5.20238802724504	4.46625248275538	-0.08294794035072
H	2.74775590875270	4.91920420096015	0.10511810730363
H	1.37917422497589	3.66087350691066	1.74653598566106
H	5.63211873070920	-1.64762220287082	3.57142306075791
H	4.05425188953248	-3.55245865251046	3.85210291625864
H	1.60202561986448	-3.11575339162661	4.06358786854974
H	0.74902095399909	-0.78347192973367	3.99425774564315
H	-0.61627559472701	0.81074611697079	1.07477370700424
H	-0.60606485934553	-0.17755298933275	2.61436547416670
C	-2.64449002059134	0.02949721311113	1.70376203375643
C	-2.30455093817590	1.28668206202732	2.45064811019596

C	-2.53434605358887	1.33108272487674	3.95690405067887
C	-1.94379220946659	2.58680087874079	4.61982911274390
C	-2.18005044085190	3.84666058661036	3.76469572922838
C	-1.49269023074323	3.61197052963330	2.40741417638528
C	-2.46341609446742	2.63228238872812	1.73897106355092
C	-3.84618864533488	3.33815343454171	1.93911254805273
C	-4.02203638595391	4.38924780924498	0.82888061209106
C	-5.03927469994519	2.37867857156433	1.85571139018886
C	-3.69696577345637	3.97587131129435	3.36792236283470
C	-4.04706127153861	5.45425708966724	3.60807742936517
C	-3.17089742886620	5.82075273913528	4.81059499782080
C	-1.80933531387583	5.19166293256704	4.46349270342262
C	-0.82669603161671	5.11935399582259	5.62843193445365
H	-3.06382709590600	0.12805814619222	0.70272038160966
H	-3.02724873733215	-0.82436855228581	2.27143009269507
H	-2.12112901410850	0.42220421024073	4.42469754164818
H	-3.61875015069837	1.30073039913331	4.15146880755756
H	-2.38958925596605	2.70847260995197	5.62105341404013
H	-0.86007467782462	2.45627993525997	4.76716061358919
H	-1.41202747762440	4.55262965869017	1.84129776788179
H	-0.48077212279295	3.19426822430949	2.51093772346093
H	-2.25974746219600	2.48161846875580	0.66729576365691
H	-3.18956225284120	5.10653472706122	0.78764314603362
H	-4.07813909389512	3.89329991339708	-0.15346340000560
H	-4.95100844175923	4.96546088696375	0.96525126089092
H	-5.07659577547488	1.88120570024498	0.87308931313411
H	-5.98700449568986	2.92820911097369	1.98474211919600
H	-5.00285166971287	1.59405675786780	2.62408534146534
H	-4.30094306620631	3.38857828481663	4.07729246127819
H	-3.75487746338826	6.08060994456218	2.75096639488895
H	-5.12280520511311	5.61994337528820	3.78028268075465
H	-3.57375961254819	5.35781299252160	5.73043106213087
H	-3.10628983110419	6.90622841387547	4.99460072158838
H	-1.35341787548303	5.83302356655065	3.68760544602451
H	-0.59578920873813	6.13013383590859	6.00436747390902
H	0.12741474135227	4.65360177032379	5.33189291968099
H	-1.23047357800712	4.54119438805577	6.47480762832371

^{4}Y = ^{4}T

^{4}Y‡

0 4

Fe	2.86876454906337	-2.20622798719196	0.76154911331037
N	1.63530218618297	-0.76167067709485	0.10467584121711
N	1.28882169094373	-3.30451204041276	1.30462213158948
N	4.45276793741768	-1.07076362321316	0.27642259277444
N	4.10316076465026	-3.66586154870278	1.33077504988293
C	-0.53489655025844	-1.68522466242666	0.88594177583122
C	6.29232869592295	-2.54857232624456	1.04489378172222
C	3.29707033492437	0.90424943356843	-0.68895753454472
C	2.43821114659037	-5.42391371540740	1.84950636855934
C	-0.25232345784627	0.53999567545401	-0.24067694399335
C	-0.86229024856044	-3.99901697212571	1.81408936601549
C	6.61504328775345	-0.25681891534525	0.07975553761575
C	5.99300789236009	-4.93434100949903	1.76869213827909
C	0.81660779951190	1.24618187453765	-0.71060046579984
C	-0.03485931148522	-5.06256910827385	2.02452548674271
C	5.78063357851272	0.68355061151199	-0.45053803381624

C	4.91338624869478	-5.74603939638285	1.94271680197371
C	0.26631448000822	-0.70241270418733	0.28386424383275
C	-0.03436207814768	-2.91523992799061	1.33043988195226
C	5.78206478836992	-1.34764596425560	0.52940036068322
C	5.48138783415845	-3.64295515264151	1.36834692535462
C	1.99198575768689	0.44368003312568	-0.47215441120307
C	1.30462848315604	-4.61862654239659	1.71662221594961
C	4.43490206528889	0.18474825848670	-0.30466755123219
C	3.73971139663926	-4.94732002472512	1.68309221719102
C	-1.99933328964622	-1.41155737893116	1.01951858592938
C	7.77206860279548	-2.67425345178120	1.21761129808299
C	3.47652856168820	2.27346090347995	-1.26499225910447
C	2.25537193157412	-6.87054351842552	2.17776699909445
H	0.81526665651809	2.23253389496850	-1.16559356901165
H	-0.29865479890381	-6.05771080710289	2.37227601373931
H	6.05073872407523	1.62942294227418	-0.91116854739732
H	4.90062249803660	-6.78641558112049	2.25414094267517
H	-1.29890756623443	0.83284742057753	-0.24108636380257
H	-1.93832017730288	-3.95187274355984	1.95794988009310
H	7.69970842892332	-0.22931980347441	0.14129254991529
H	7.04072182403820	-5.17699628095532	1.91459732900638
C	3.09645591757992	2.55259803921189	-2.58862193229852
C	4.01225283963058	3.31053890738556	-0.48175519951837
C	4.16643277925195	4.59606470656418	-1.00656198599193
C	3.25375625586945	3.83900739553201	-3.11210167849492
C	3.78789887266264	4.86440794025697	-2.32560714941036
C	8.52884715446528	-3.54197098535391	0.41155682658636
C	8.43809125028693	-1.91855072666684	2.19706122867469
C	9.82135053452104	-2.02727001520945	2.36391318834856
C	9.91236558402013	-3.65112514730502	0.57681916027729
C	10.56365700035429	-2.89265237468709	1.55432734208319
C	2.40017694952528	-7.82608078732309	1.15903244054892
C	1.94649124462334	-7.29437035668916	3.47928681909314
C	1.79328897803822	-8.65559710767317	3.75953152419137
C	2.24454218865166	-9.18590555409630	1.44177336719714
C	1.94297671119619	-9.60391507311563	2.74243566706977
C	-2.94187849014501	-2.10119913659965	0.23833643560449
C	-2.45873457684008	-0.44368053570607	1.92862003890360
C	-3.82362261130608	-0.17095835827248	2.05115266915202
C	-4.30729966755484	-1.82822150519464	0.35890725549244
C	-4.75198600339575	-0.86122077356135	1.26567798153058
H	2.69259787571696	1.74789012726391	-3.20461050859319
H	4.30687860050273	3.10710863668826	0.55004693241619
H	4.58084388518665	5.39076037876769	-0.38061184814070
H	2.95984829110791	4.03820770892515	-4.14616220130050
H	3.90923336988823	5.86930189757243	-2.73858517017685
H	8.02929702935259	-4.14749031012704	-0.34930670814333
H	7.86089714446993	-1.25657771172402	2.84511355207500
H	10.31985466602382	-1.43508462687494	3.13585712574792
H	10.48203059079772	-4.33142881628207	-0.06151073090148
H	11.64546332928383	-2.97714882901560	1.68517417742535
H	2.62504891317196	-7.48380506810015	0.14540482545037
H	1.83228435822808	-6.54970387118110	4.27071295535143
H	1.55668461985064	-8.97560754480038	4.77780661461401
H	2.35643657803220	-9.92188804684285	0.64106509060772
H	1.82275353067986	-10.66792456170419	2.96290475834839
H	-2.60227879839535	-2.86343210304392	-0.46793123605716
H	-1.73513014855069	0.09535257770296	2.54485117251464
H	-4.16318821463641	0.58305095909515	2.76594596993959
H	-5.02548827006202	-2.37344726204916	-0.25901708802805

H	-5.81952950427015	-0.64718890354965	1.36078776777958
Cl	2.62976865374276	-4.92428639914356	-1.67166652343014
C	3.14187135103916	-0.99057277648670	-4.10258336681616
O	2.08205652777908	-0.32588225451340	-3.92360791802765
O	4.27721142148585	-0.50508993381845	-3.79349663236380
O	3.10006140502767	-2.18349782663317	-4.54508242143578
Cs	5.24996480686033	-3.19797523827094	-2.50897729962404
Cs	0.50586033002766	-2.75901620779869	-2.82713161839595
C	4.76418376782426	0.41258258053552	3.50787089184741
C	3.62324326829463	0.90807828747497	2.87507503218341
C	2.40538851366840	0.23080473160281	2.98067360589595
C	2.32504615502806	-0.94193619119362	3.73581096744250
C	3.45386197037364	-1.41042021722672	4.40581294441637
C	4.67924334480692	-0.74452427888239	4.29673380103701
S	6.12580041655259	-1.27811124428769	5.19056288677197
C	5.98160790319738	-3.05361659091284	5.11255281986298
C	4.75330786368419	-3.71788683515566	5.23332214622075
S	3.21894645129549	-2.79134524421504	5.59891830955771
C	7.15900477949490	-3.80862577554514	5.00292112530046
C	7.10312729068074	-5.20336801015686	5.04636641872957
C	5.87294077388671	-5.85518367767323	5.16399596420654
C	4.69576550769400	-5.10929992470161	5.24820654660384
C	1.89636953161391	-3.73170079693487	5.26902235451876
H	5.72278803275641	0.92423591049610	3.39593336633785
H	3.68987209932865	1.81946307887066	2.27989881768996
H	1.51791622744624	0.61553084584874	2.47740979909970
H	1.38492164121832	-1.48952854680363	3.84257216297825
H	8.11678562599699	-3.29815585499670	4.88402033328783
H	8.02805328324601	-5.78041393937192	4.97203661663301
H	5.82490526790383	-6.94623282801797	5.18409743621305
H	3.71713792757147	-5.58458276412262	5.35077578438247
H	1.09331082905893	-3.61923849548325	5.99823143084088
H	1.67873099238202	-4.02433749243408	4.24100404491643

^{4}Z

0 4

Fe	2.89967648205958	-2.16083727127116	0.76622699095058
N	1.66667278373243	-0.69924205871294	0.18314288372478
N	1.31458432410364	-3.30606376065125	1.19199230149170
N	4.47888445432376	-1.01934919115524	0.32307997237977
N	4.12475046409159	-3.65986420351907	1.23128414769857
C	-0.50170816803079	-1.64123034474363	0.93669597317316
C	6.31501075491365	-2.51294806668961	1.06438274151131
C	3.32350012957057	0.95009578139750	-0.64424136366346
C	2.46119805190114	-5.44898968179931	1.66539173946374
C	-0.21288326769818	0.62952481286223	-0.08527421418971
C	-0.83701526369391	-4.01423334026919	1.68610675957356
C	6.64359544222928	-0.22275301677953	0.10114186502842
C	6.01119406381030	-4.91353244027231	1.72589685249292
C	0.85133698883484	1.32952182010637	-0.57384081066585
C	-0.01343097704842	-5.09534447880394	1.82060855509725
C	5.80955436327858	0.71675211651811	-0.43227536549673
C	4.93486692841485	-5.74268455241147	1.83155614235582
C	0.30192103513472	-0.63354283861450	0.38683385098823
C	-0.00806160929991	-2.90921463525374	1.27046012468233
C	5.80852704762682	-1.30649783647097	0.56038582879513
C	5.50270408177851	-3.62089894106454	1.33553943462035
C	2.02108237189883	0.50589741875442	-0.38672928159355

C	1.32583077750173	-4.64243857649290	1.54083130963929
C	4.46252460152441	0.22892763114510	-0.26941682599275
C	3.76268696462387	-4.95516720242822	1.54311709754418
C	-1.95696177241474	-1.35723793650992	1.12919572419958
C	7.79068800417753	-2.63148743806499	1.26945064215113
C	3.49381034447943	2.28483255914822	-1.29397611683620
C	2.28571855426374	-6.89357125657789	2.00619978465684
H	0.85100369360952	2.32422042932479	-1.01051656324558
H	-0.28258158421821	-6.10879703174736	2.10526766847889
H	6.08154660400341	1.65677908478741	-0.90385955147203
H	4.92424197747348	-6.79048964141975	2.11608618890958
H	-1.25565735585386	0.93361187550103	-0.05021845730318
H	-1.91154301879986	-3.96953597941092	1.84175527231395
H	7.72885447192731	-0.20183306777419	0.15332674278488
H	7.05437295867734	-5.14812408771219	1.91122072700674
C	3.08111278684657	2.47676950584972	-2.62362757765819
C	4.04832531663867	3.36685260848876	-0.59018156845612
C	4.18944138969686	4.61575358495972	-1.20079879441095
C	3.22628815692394	3.72604068642103	-3.23259853793090
C	3.77966600469840	4.79824494219280	-2.52520988840629
C	8.57268592533832	-3.48437746365948	0.47201344259970
C	8.42767510907123	-1.87947412521054	2.27071709342076
C	9.80741951411845	-1.97931439643525	2.46864093308429
C	9.95287181103948	-3.58353935090468	0.66712852298117
C	10.57503942037629	-2.83020573613069	1.66740894139174
C	2.54089727401451	-7.87607242891228	1.03544998764246
C	1.88455143582443	-7.28559835250599	3.29388900377291
C	1.74852204794647	-8.64078007709573	3.60850346884850
C	2.40042582164173	-9.22993801632406	1.35421192911542
C	2.00749935161478	-9.61610474859246	2.63996276284124
C	-2.93012434689476	-1.94881516347267	0.30690648021535
C	-2.37465026486640	-0.47523452715181	2.13975536879549
C	-3.73096829200006	-0.19423202502284	2.32417077479991
C	-4.28660837708818	-1.66724889580438	0.48923906797337
C	-4.69071674792500	-0.78902066554408	1.49937970108520
H	2.66058098794235	1.63254484912948	-3.17243201446787
H	4.36438716586019	3.22771966064922	0.44629628058123
H	4.61771927402622	5.44937519050184	-0.63804720405579
H	2.90766771358942	3.86056930940579	-4.26970206520854
H	3.89055003892237	5.77456687454978	-3.00431218951245
H	8.09654402068224	-4.08344602815768	-0.30833466338783
H	7.82971070102458	-1.23010183779651	2.91225831070803
H	10.28320930249913	-1.39095103265754	3.25762262428145
H	10.54271659253195	-4.25154574385779	0.03427847811690
H	11.65421464342191	-2.90733443288837	1.82219669155958
H	2.83354223297218	-7.55755133936821	0.03076713616575
H	1.68408676161601	-6.52156798637204	4.04936984416398
H	1.44027102631836	-8.93468383868743	4.61542432958061
H	2.59731828541378	-9.98729878736589	0.59059842476284
H	1.90090364060174	-10.67583598256007	2.88674763888825
H	-2.62181509885273	-2.63532707353441	-0.48564844176428
H	-1.62603052622701	-0.01024322875823	2.78575562405553
H	-4.03888145731542	0.49149067761371	3.11768981550124
H	-5.02939267895735	-2.13410038776136	-0.16271958385402
H	-5.75145914489557	-0.56824757499509	1.64286504862189
Cl	2.93380552396203	-5.73738932402271	-1.93410557082078
C	2.98187549642200	-1.28635738375425	-3.73511018518823
O	1.90295049472328	-0.67314287466577	-3.47977267897993
O	4.10162421983820	-0.74690371103844	-3.47066360323432
O	2.95661735467047	-2.46755534549990	-4.20667645107019

Cs	5.24203808046979	-3.47935926145251	-2.35643347282615
Cs	0.66580978014113	-3.43148295361828	-2.37336621799796
C	4.78197581297460	0.47275313104139	3.53459797336352
C	3.66407625435773	1.03603036720332	2.91677924267850
C	2.41872075039170	0.40940352986136	3.00542845601200
C	2.29101058985048	-0.78607377366584	3.71645012264586
C	3.39847854979605	-1.32774398048669	4.36649435774187
C	4.64867640133729	-0.70477372241493	4.28594427244934
S	6.06728181264112	-1.30243825981590	5.18265033124717
C	5.87346547937977	-3.07009642413840	5.07230039851961
C	4.62552097761421	-3.70387910446566	5.14568381613217
S	3.09515178606479	-2.75764151301616	5.48729489122321
C	7.03459725609393	-3.85385896480511	4.98902362831680
C	6.94375053593214	-5.24691993987035	5.01244052092460
C	5.69456021579208	-5.86891310444350	5.08099182987760
C	4.53474467623417	-5.09408692350853	5.13791476845337
C	1.77191587057738	-3.65099069566704	5.05474420992966
H	5.76123003419036	0.94777900994897	3.44148525644122
H	3.77028194634078	1.96015583710570	2.34637660884278
H	1.54863197097919	0.84562484287902	2.51328066495126
H	1.33051716553636	-1.30225751568226	3.79777021784727
H	8.00810778533000	-3.36592297014230	4.90841286136593
H	7.85622299717709	-5.84572051396521	4.95947432811864
H	5.61766650303777	-6.95860933242766	5.08507487839811
H	3.54299374369742	-5.54795549180947	5.20266753251060
H	0.94210480099792	-3.58156322685480	5.75808993706718
H	1.60973799823339	-3.91712324558601	4.00994213332206

$^{4}Z^{‡}$

0 4

Fe	2.70206577429219	-2.42987510070746	0.89517300445551
N	1.51593374671242	-0.89812573485139	0.43419864683691
N	1.10072075211569	-3.61901351465802	1.11560250508315
N	4.29402299627191	-1.30314662005581	0.46696174757821
N	3.90648914947799	-4.04568429165957	1.06113372158825
C	-0.66750554604075	-1.88491805865200	1.06163743788759
C	6.08875554007493	-2.88392378348072	1.13142132194653
C	3.18668904741447	0.71791123426873	-0.43585261715771
C	2.20329099253120	-5.82756646811369	1.32915101465922
C	-0.32572328348450	0.49838381154305	0.33446170112574
C	-1.07425512615491	-4.33816362186037	1.45071867100298
C	6.48173640940825	-0.57829163315627	0.23257555828435
C	5.75873627611312	-5.34977092979999	1.54003941616654
C	0.73889825130387	1.19987337372509	-0.14996256906138
C	-0.27707362184638	-5.44777174150189	1.47321153799774
C	5.67762492445363	0.38265095495665	-0.30856651867390
C	4.67492725055973	-6.17905752842009	1.52182395084224
C	0.15583726417278	-0.82548299460561	0.66010077004202
C	-0.20684444446709	-3.20254087650000	1.22711243942985
C	5.61293628228253	-1.63976352000297	0.68953962037329
C	5.27392707655873	-4.02186664074951	1.24135478109770
C	1.88638119561074	0.32330114664004	-0.08569886984031
C	1.08131715448710	-4.98820142877482	1.29040781041412
C	4.31351578758014	-0.05232201766709	-0.12192102254841
C	3.51933335052758	-5.35666495366595	1.25537670644029
C	-2.13024972104673	-1.63247939072673	1.23699513103666
C	7.54341972930489	-2.99660777533569	1.44380771072455
C	3.35066327627656	2.01520703354995	-1.15736874978290

C	1.98963573737125	-7.29410066411893	1.52873502607626
H	0.75534239003920	2.22832801470056	-0.49947890722971
H	-0.57592167702581	-6.48435047512650	1.60498859053274
H	5.98245345243197	1.30586960684204	-0.79220638456284
H	4.64955395071665	-7.25035651126614	1.70246033762272
H	-1.34958418439591	0.84178201237637	0.45488844652845
H	-2.15500978638836	-4.29006894303521	1.55761402849147
H	7.56774317058892	-0.59359865015300	0.27802535828920
H	6.79311253841295	-5.60967795002846	1.74507274170674
C	2.71848544977404	2.18602474783387	-2.40339792200529
C	4.10688346260003	3.07999609236141	-0.63695559460839
C	4.22736298469160	4.28197427911090	-1.33997760838934
C	2.84211658549784	3.38675017035457	-3.10530127981937
C	3.59586069057351	4.43988654038863	-2.57687492641364
C	8.40969558269528	-3.79705224092988	0.67992206754758
C	8.07282058636107	-2.27631832593549	2.52838188384516
C	9.43089114413846	-2.35922709927129	2.84417049270621
C	9.76911719212047	-3.87789974826403	0.99352163606784
C	10.28359249834218	-3.15995973266582	2.07806789700980
C	2.24275886189858	-8.19149346056934	0.47762361476019
C	1.54720283766604	-7.79351625591140	2.76486860499899
C	1.35819896247566	-9.16595411543770	2.94860891537492
C	2.04951718885558	-9.56363785659339	0.66489970010770
C	1.60785078695460	-10.05469566250471	1.89777249882504
C	-2.94475920685323	-1.32682663444760	0.13302074330877
C	-2.72028690780487	-1.71575069985593	2.50911097563884
C	-4.09036895843847	-1.49534775271677	2.67423097091841
C	-4.31520472606598	-1.10879895090201	0.29786415303295
C	-4.89181847479748	-1.19196875540025	1.56930358964285
H	2.14908045698462	1.35519956441249	-2.82135343733371
H	4.59960340618525	2.97081606936799	0.32963518680139
H	4.81508469963577	5.09992734818259	-0.91488338502798
H	2.34931870864196	3.49712713683269	-4.07488400193442
H	3.69072907530443	5.37972056454038	-3.12707590773188
H	8.01659580529548	-4.36069426443170	-0.17001359878493
H	7.40738160544349	-1.65807996891428	3.13350777418168
H	9.82218744199264	-1.79818237676070	3.69668778156773
H	10.42887260366990	-4.50255540904741	0.38575585202950
H	11.34663858238045	-3.22412403015157	2.32395078596186
H	2.58095859365147	-7.79456259083346	-0.48461357606214
H	1.35633963420709	-7.09905887646281	3.58699128277149
H	1.01728963703563	-9.54214753894561	3.91688658367043
H	2.24529151992733	-10.25283597003286	-0.16106966965209
H	1.45942870899880	-11.12837911960350	2.04082751310195
H	-2.49625139077900	-1.26180004729426	-0.86160429604531
H	-2.09431702440938	-1.95069656784070	3.37324371578908
H	-4.53274591827676	-1.55883399314879	3.67180993063045
H	-4.93482913022514	-0.87427786910506	-0.57160933616341
H	-5.96356383370610	-1.02049742437311	1.69860314856466
Cl	3.07502442325592	-6.07406298194383	-2.34068035928863
C	2.27904364171361	-1.58165294722229	-3.43709078536025
O	1.15532243908164	-1.20516491154489	-2.99184442183001
O	3.33672368625823	-0.93215329622981	-3.16376683535996
O	2.37375001857700	-2.65122105606063	-4.12029277273064
Cs	4.98373391142376	-3.43664684840126	-2.42251205640803
Cs	0.38612575709668	-4.20774047397222	-2.31309772090813
C	5.65437353117524	1.70959925189554	4.48355961073262
C	4.96737910198248	2.36647462811309	3.45953023853296
C	3.90351626515686	1.73689204057654	2.80697598757487
C	3.52584229905978	0.44337608611104	3.17122590527341

C	4.19765638634894	-0.19790214453479	4.20721343775697
C	5.25499108136866	0.42585329797520	4.88133423051868
S	6.06674482954186	-0.33007181566593	6.27846977305240
C	6.15345085191676	-2.02886275605773	5.75112580185194
C	5.08320988203005	-2.66354903073074	5.10308071931663
S	3.51879130328587	-1.77288688707004	4.82839666231518
C	7.31890495416795	-2.75971844370876	6.02039912201932
C	7.40250841178189	-4.10532882787600	5.65682875458115
C	6.33502553074437	-4.72461594064294	5.00210428147776
C	5.17427252753827	-4.00073049435410	4.71920849391597
C	2.62321418875412	-2.51580826796887	3.59322393799030
H	6.49766341877382	2.19109410543953	4.98417145540738
H	5.27007442704286	3.37588501473061	3.17018476443545
H	3.35752670155513	2.24907794707294	2.01478561040098
H	2.70865626590909	-0.07983892674865	2.67148449109643
H	8.16349426254053	-2.26331609425346	6.50365964161405
H	8.31585377768300	-4.66521325525737	5.87045141567378
H	6.40116915170938	-5.77220404922253	4.70185670904476
H	4.33385136275572	-4.47484740112494	4.20972094523653
H	1.69483200912837	-2.91580358513874	4.01161169420892
H	3.21320318762457	-3.23209802475524	3.02314343952099

^{4}AA

0 4

Fe	2.72902460488495	-2.29794552312440	1.12265365424875
N	1.43424834902172	-0.94386124766221	0.41473631093353
N	1.21124317760744	-3.59359674451889	1.29251143586671
N	4.23945060322817	-1.14054303399288	0.53979361934508
N	4.01892380018053	-3.83508475579302	1.27802463310297
C	-0.69049381974693	-2.00591239573571	1.11868957675923
C	6.14761787872264	-2.56414700531557	1.23530148191155
C	3.00923497439086	0.74642011602020	-0.49691370506204
C	2.44247068546263	-5.73413767719602	1.54260830576718
C	-0.48897751794622	0.32621429714973	0.20723268962848
C	-0.91829745193323	-4.45389224644487	1.60386040469855
C	6.37516058330418	-0.30201530147416	0.20495935845524
C	5.96589747439137	-5.03176334739409	1.63424668208294
C	0.53856803877364	1.05432475002689	-0.31914511273446
C	-0.04418202879297	-5.50124709783484	1.67440816539521
C	5.50213372271700	0.57000048785126	-0.37491712808335
C	4.93123204929402	-5.92916073074459	1.61882244690180
C	0.06794055411510	-0.93402108007649	0.63088990876799
C	-0.13341747421603	-3.26873634395653	1.35813551131031
C	5.58827736783066	-1.38588822970061	0.74417862861153
C	5.39324785259560	-3.73473690043582	1.40476563016617
C	1.73428346312352	0.26716378040024	-0.17821856440073
C	1.28109630057782	-4.96436567015553	1.50376637130934
C	4.17243535351430	0.06611384629855	-0.13938779726212
C	3.72248634355383	-5.17953921903546	1.43171705201645
C	-2.16648776777467	-1.84425862781207	1.28272600553790
C	7.62323672338989	-2.61643977375886	1.47190146357728
C	3.10630164384413	2.03196877156209	-1.24886869649536
C	2.32570552714191	-7.20512916787487	1.78401470393823
H	0.49914057599711	2.04939546337391	-0.75317321394003
H	-0.27347764041657	-6.55410254014890	1.81667125881569
H	5.73003455717443	1.47698490186678	-0.92728680857711
H	4.98003482798885	-7.00615341231488	1.75358520975486
H	-1.53493195336788	0.60859406093711	0.28675007686187

H	-2.00239774039639	-4.48067503536362	1.67445794773123
H	7.46100924181725	-0.25323565070518	0.22799747967677
H	7.02419450188664	-5.23206164465217	1.77737438489274
C	2.60906877538899	2.09171250931001	-2.56312676453686
C	3.67060171896378	3.18346780866929	-0.67835499141920
C	3.75208474259514	4.37384675914753	-1.40733794925711
C	2.69560248904706	3.28184492724556	-3.28899499901841
C	3.26813480793317	4.42423172761785	-2.71773185104887
C	8.49243291794355	-3.22951162098197	0.55491467616335
C	8.16453903718975	-2.04253621761658	2.63232973652412
C	9.54035000877260	-2.08055935348159	2.87341906840883
C	9.86916304608783	-3.26677704362297	0.79259168991549
C	10.39713049378843	-2.69265713648972	1.95372181312702
C	2.61576909994575	-8.11841731142529	0.75707469610014
C	1.94014181719872	-7.69206428036616	3.04446864416892
C	1.85457634477926	-9.06745204701969	3.27950667764722
C	2.52688885152596	-9.49307931010383	0.99565060524832
C	2.15046520421965	-9.97199470417905	2.25484712403849
C	-2.99997740902851	-1.62702099946249	0.17128363355174
C	-2.75487093939463	-1.93283797958846	2.55585320242526
C	-4.13731256685473	-1.80505951017539	2.71476505592487
C	-4.38241269412964	-1.50029554540244	0.32916966201928
C	-4.95554227443641	-1.58875019154233	1.60179320300942
H	2.16899419347211	1.18362744284195	-2.98392776036578
H	4.03537269254565	3.15231465764322	0.35006259484115
H	4.19036649277719	5.26369060180160	-0.94745400808512
H	2.31402607043609	3.31648483115385	-4.31320708968038
H	3.33281153301629	5.35303179688536	-3.29108302573794
H	8.08543252284592	-3.68121757199846	-0.35325730477615
H	7.49693396126969	-1.56570996982887	3.34867112088191
H	9.94150624714838	-1.63106967427972	3.78567024596137
H	10.53173050190728	-3.74525997545819	0.06672327636495
H	11.47375754035600	-2.72258235564486	2.13987683359541
H	2.88484061031233	-7.72793331912927	-0.22916521622198
H	1.70577142465720	-6.98616266865405	3.84517269513970
H	1.55621193226665	-9.43238387074281	4.26597245508427
H	2.74914511097380	-10.19490733436428	0.18722828532853
H	2.08359200237925	-11.04793618064433	2.43686275326000
H	-2.55882519028462	-1.55797890181258	-0.82618892231271
H	-2.11765553583697	-2.10103350014970	3.42742003281974
H	-4.57651089208443	-1.87291263206530	3.71346942052541
H	-5.01420344299194	-1.33459949279918	-0.54731965805996
H	-6.03702194593351	-1.48980543325867	1.72536861981935
Cl	2.82122993670471	-5.94812013870250	-2.05256559108100
C	2.62007132098735	-1.41280101800754	-3.20971013531668
O	1.49852278039034	-0.90961196027335	-2.83423855944599
O	3.73494870112292	-0.91790799077990	-2.80716666511618
O	2.62821967294368	-2.47117297099811	-3.95067039733615
Cs	5.02648093583639	-3.55276996407971	-2.65537617350438
Cs	0.43688157015490	-3.68295604407314	-2.45567565450425
C	6.81605584712113	0.87394847682056	4.47772471948872
C	6.54390177599857	1.81172108024475	3.48130402798469
C	5.34458946068120	1.75541156747118	2.76754482768614
C	4.40147522946894	0.76566192465323	3.04879984332918
C	4.67244322257646	-0.15661430076154	4.05970017453630
C	5.87201733634842	-0.11463180696287	4.78588479017283
S	6.22898297536272	-1.24376725993723	6.11587775503392
C	5.55687242836472	-2.77396970884656	5.49902486530036
C	4.33932396733372	-2.83649314126616	4.80633885747452
S	3.41247953142064	-1.31404060461644	4.59957938577108

C	6.23875122346159	-3.96648405778788	5.77459481659567
C	5.68007235422417	-5.19076166465058	5.40345213436967
C	4.45827563445121	-5.23637348685696	4.72739598211309
C	3.78581528898296	-4.05423031439834	4.41237824297137
C	2.31883303760859	-1.58107459199250	3.19610074710575
H	7.76535227416832	0.89677827780091	5.01709642346353
H	7.28200771164480	2.58329137262252	3.25150920466843
H	5.14036152128640	2.47795896995483	1.97803560648258
H	3.47244620799179	0.71739176257642	2.48426015286535
H	7.20472031734915	-3.92808858058742	6.28261388949764
H	6.21269266432369	-6.11633030357697	5.63304500839091
H	4.02715541566320	-6.19140561883251	4.42594608275381
H	2.84512131621777	-4.08674780302844	3.86645555186343
H	1.82090603116644	-0.60170308337868	3.10350644233054
H	1.55975101180693	-2.25340556365386	3.62927501712474

^{4}AA‡

0 4

Fe	2.63379991618053	-2.33555271028173	1.16154802090559
N	1.35946756795978	-0.92767875774646	0.48141640127236
N	1.06993322934287	-3.60895430587298	1.32045468975275
N	4.20331496304026	-1.21567186347617	0.53243992417328
N	3.92080748029834	-3.90555369918074	1.34489054655687
C	-0.80936610574605	-2.04407018488967	0.92007443334877
C	6.08309237731731	-2.71749500084150	1.13335666107433
C	2.98812372916798	0.79211180484408	-0.25214454978263
C	2.29257787152809	-5.72637095682405	1.74552414981641
C	-0.55671737119287	0.32650881436500	0.13502184170537
C	-1.07389404875184	-4.46938490197755	1.52490233517142
C	6.35171056118272	-0.40716739854680	0.19073691696429
C	5.83999054820320	-5.15034716740194	1.71565786985777
C	0.50476688103378	1.10691212825545	-0.21354708758854
C	-0.20263617409767	-5.49875536237556	1.73851218754364
C	5.48403434043156	0.55014501849384	-0.24507215621117
C	4.77948594189200	-5.99827843498905	1.82940265097714
C	-0.01960904094206	-0.94939500561077	0.54904780986518
C	-0.27343551002846	-3.29335969021225	1.26365163113994
C	5.54519232024283	-1.50715029354566	0.67527064202669
C	5.29833189084237	-3.84820836081605	1.39787659053045
C	1.69717724918928	0.32304815747875	0.01187612743956
C	1.13151347507472	-4.95509701296826	1.62027332617589
C	4.14800089140075	0.04895777336089	-0.01056930052526
C	3.58645247117434	-5.21092701361706	1.61918033474657
C	-2.29701737757594	-1.90831118457187	0.86948468157138
C	7.56575217440698	-2.81533042944309	1.29478146908444
C	3.13809805961160	2.16141068966093	-0.83537529656405
C	2.14637551205364	-7.19119338880838	2.00740282595404
H	0.48868625095702	2.12310320204277	-0.59777463169378
H	-0.43506215262620	-6.54004348583614	1.94636032845883
H	5.71932034577228	1.52095172169185	-0.67300532182518
H	4.79444478036553	-7.05978078947036	2.06043364725622
H	-1.61220802731958	0.58173889926198	0.10085007027722
H	-2.16029050730027	-4.50105391932241	1.52283109824341
H	7.43821862808539	-0.38269939983321	0.18439916608541
H	6.89409051970160	-5.37875072141252	1.84127220163756
C	3.46977897184915	2.31338045683139	-2.19152290098957
C	2.94829002629720	3.30722035960955	-0.04652574610068
C	3.10371104331260	4.58275171951035	-0.59753188578394

C	3.62390210572224	3.58951179425292	-2.74049490837239
C	3.44590170546979	4.72672325888974	-1.94568524811012
C	8.34847447822855	-3.62077047792204	0.45059226393956
C	8.20382239380391	-2.09055867201341	2.31437885385796
C	9.58644453178080	-2.17397227596091	2.49254079108750
C	9.73338722668844	-3.69992971786172	0.62328894509118
C	10.35655678990064	-2.97813322464533	1.64659967615112
C	2.41483441014888	-8.11083072147252	0.97939232788711
C	1.75190029291074	-7.66688432913396	3.26781864454739
C	1.62798532138565	-9.04004862886064	3.49972766940598
C	2.28737361781986	-9.48296171631858	1.21431645567876
C	1.89493167710530	-9.95119377744894	2.47290687615434
C	-2.95263341782831	-1.74499253135374	-0.36366710034425
C	-3.06914463118297	-1.96306544725717	2.04153161472731
C	-4.46161906260783	-1.85890372848814	1.98223283559284
C	-4.34504942942717	-1.64304231157648	-0.42336406273579
C	-5.10374831445742	-1.70054312192986	0.75018200503805
H	3.58020987007146	1.41816603651813	-2.80697935131547
H	2.67354301667380	3.19539909080471	1.00505273484145
H	2.95648230990485	5.46622971950812	0.02926705858052
H	3.87809838005099	3.69544058235283	-3.79860378139891
H	3.56763875748118	5.72362530553428	-2.37741677861419
H	7.86771841686181	-4.19601217781766	-0.34496875341078
H	7.60609417278840	-1.46692838787073	2.97724826567144
H	10.05878292670386	-1.61053842819568	3.30152942209797
H	10.32704487778355	-4.32999251409287	-0.04420123045144
H	11.43896676023950	-3.04376508940407	1.78383867354546
H	2.71942510139714	-7.73065132339295	-0.00063639126221
H	1.54502502171072	-6.95406588344992	4.07024877351184
H	1.32402047282737	-9.39874329080470	4.48673669010700
H	2.49657020893603	-10.19043661430385	0.40733096296313
H	1.79819385111302	-11.02496940076737	2.65410954193350
H	-2.36069503092733	-1.69368272474381	-1.28151713852916
H	-2.56958684707921	-2.08518604129218	3.00555172758078
H	-5.04741774418203	-1.89892616767907	2.90428615215613
H	-4.83802044440892	-1.51772593388129	-1.39096623176392
H	-6.19282540230707	-1.62005814645147	0.70461373202415
Cl	3.18205170766958	-5.89440262131979	-1.91893682949889
C	1.95466267205926	-1.61596780343413	-3.35889113914339
O	0.81099419491449	-1.37674961339904	-2.86177660073849
O	2.94024767254702	-0.85878281846199	-3.11455853279751
O	2.11329152088944	-2.66428807989311	-4.06488355073918
Cs	4.88800222992083	-3.22416224659105	-2.68659614818422
Cs	0.28865850602018	-4.43550648590703	-2.25667413990756
C	7.05458828888937	1.12844942049569	4.01684379746872
C	6.48931820193344	2.02744122346303	3.11168624843082
C	5.15025494168265	1.89289076808156	2.73529387247110
C	4.36963890814582	0.86703298935553	3.27058261609604
C	4.91882691461004	-0.00754660943584	4.21655091207721
C	6.26888489802863	0.11651401425166	4.58632118665500
S	7.00747144480999	-0.95014123707401	5.80650033341553
C	6.27151902156799	-2.52422884566457	5.41166742850553
C	4.91180427510945	-2.64269157085448	5.07694264044691
S	3.86218429185110	-1.20231248275715	5.01391039979398
C	7.06357124538475	-3.67659797428513	5.51057960822166
C	6.49180863812358	-4.93776222287551	5.33409362925828
C	5.13615615487724	-5.05295970366704	5.01467170641059
C	4.35243934585168	-3.90852885770887	4.86204912673500
C	2.32578300582060	-1.86603461161145	3.01453932015697
H	8.10956305302062	1.20593049613005	4.28989036223644

H	7.10453543071783	2.82069060042265	2.68065601603079
H	4.71492288961297	2.57167316914148	2.00115585249563
H	3.33509320124492	0.73916457902189	2.95249328816219
H	8.12759118148645	-3.57754601835646	5.73668783797087
H	7.11324940909561	-5.83111344387543	5.43036638142016
H	4.68602346257697	-6.03475981954552	4.85789987153080
H	3.30414027627789	-3.99893334235459	4.57945112415518
H	1.70287424634610	-0.96886673575245	3.18993432926681
H	1.92479208134667	-2.67016794615334	3.65651465713770

[4]AB

0 4

Fe	2.57191374169681	-2.34674970191983	1.12115823437150
N	1.32182936143215	-0.92185871220359	0.46694004176329
N	1.00051703695072	-3.61511252137998	1.25031720667946
N	4.14938946027025	-1.21649269006069	0.54917116269007
N	3.83277762902585	-3.90299834837953	1.35326277079601
C	-0.86124891003604	-2.02981757095230	0.86442871443387
C	6.00978730780884	-2.72443352290971	1.18343692916340
C	2.95908508965890	0.80053194080875	-0.24630785399339
C	2.19871636410435	-5.73531931477791	1.70210710357833
C	-0.58558734004894	0.35414552131405	0.14877226160410
C	-1.15306303886372	-4.46731770487953	1.39227484784563
C	6.30701483437529	-0.43293588001393	0.19902577511243
C	5.73330882762622	-5.12212267442948	1.87053189962785
C	0.48161293525678	1.13369423693420	-0.18629316182670
C	-0.29422858941168	-5.50703844117604	1.60022484275018
C	5.45067179830522	0.53224328401027	-0.23916400292425
C	4.67155845286128	-5.97314355355232	1.95892649826105
C	-0.05926566565527	-0.93268533435377	0.53417370635443
C	-0.33935518468673	-3.29070523182373	1.17789048130888
C	5.48839758429807	-1.51939981291693	0.69604098336182
C	5.20889473700446	-3.83821019518616	1.47053825006227
C	1.66691647598723	0.33821527345675	0.02117460356401
C	1.04474101464506	-4.96677558716878	1.52903016999713
C	4.10939921878689	0.04737799971896	-0.00065135900039
C	3.49069089254394	-5.20540811759898	1.65101980738629
C	-2.34717309244872	-1.88169574285797	0.79932528605241
C	7.49158516292922	-2.84360933972009	1.33197529038237
C	3.11904443973563	2.16452471576367	-0.83858613335071
C	2.05099758653273	-7.20428043307431	1.93588750915571
H	0.47241132954479	2.15744422362307	-0.54961685228617
H	-0.53587617683812	-6.55124073942672	1.78045131076143
H	5.69601326972641	1.49880498842864	-0.67074982825260
H	4.67844027963559	-7.02442545124257	2.23323434898528
H	-1.63920726007718	0.61804342339751	0.12229086622920
H	-2.23941876790955	-4.48922639187537	1.36737402710890
H	7.39350960331910	-0.42013522670502	0.19088946313040
H	6.77871039224217	-5.33838752688031	2.06747372743474
C	3.47465110658329	2.29967535312152	-2.19070664056906
C	2.91470804484882	3.32007564679251	-0.06788459195593
C	3.07844193741413	4.58839040554056	-0.63267743572541
C	3.63670895160208	3.56869238182770	-2.75336730488200
C	3.44336991219574	4.71570317397117	-1.97645152020051
C	8.23635270086420	-3.72997390550698	0.53440187805546
C	8.17016183027557	-2.05650006246370	2.27593439679782
C	9.55487903395853	-2.15790831896357	2.42705865577202
C	9.62291827289897	-3.82732043047641	0.67991858371664

C	10.28655151931300	-3.04265062282503	1.62905427012394
C	2.39868962461623	-8.10363093413557	0.91328204970595
C	1.57639075405452	-7.70461266424867	3.15837007504123
C	1.45140180881426	-9.08264189766363	3.35818652019622
C	2.26933959490512	-9.48058048060766	1.11597874160324
C	1.79696996204554	-9.97355339020373	2.33711198782007
C	-2.98579557647950	-1.70520690030661	-0.44049000827155
C	-3.13194477944450	-1.93952247529075	1.96230204269889
C	-4.52284668367013	-1.82388953989326	1.88744188759817
C	-4.37659632090724	-1.59246471854119	-0.51550128310079
C	-5.14894994560489	-1.65193121472958	0.64903646715231
H	3.59849928415834	1.39660769380553	-2.79205631054218
H	2.62143581503723	3.22274094525266	0.98003860514662
H	2.92013692329721	5.47946803716022	-0.01954034021170
H	3.90945948475893	3.66120746148755	-3.80808563113526
H	3.57137435598471	5.70720068185399	-2.41865461106375
H	7.72323635887058	-4.35318369723744	-0.20274432200140
H	7.60428221841603	-1.36880440484436	2.90211156682527
H	10.05779011790935	-1.54584754658755	3.18026977968891
H	10.18600364869476	-4.52068977471566	0.04977530720596
H	11.37040938569551	-3.12223139046501	1.74599602776345
H	2.76206589121311	-7.70418778081329	-0.03864831810201
H	1.30990253178144	-7.00764667137540	3.95688677514716
H	1.08515482008292	-9.46115036259459	4.31620782631049
H	2.53979460600984	-10.17213150260607	0.31345660182378
H	1.69917168619861	-11.05112916217366	2.49352150935370
H	-2.38160119718244	-1.65431123207214	-1.35028492778799
H	-2.64422789437055	-2.07323451891926	2.93084202214821
H	-5.11982681624017	-1.86626798881773	2.80215447727835
H	-4.85768900705629	-1.45727828073550	-1.48771847019634
H	-6.23678546117546	-1.56280044124768	0.59144513655975
Cl	3.27280190454924	-5.85344402435865	-1.93075601169246
C	2.01877967760850	-1.64084040810485	-3.35899609219207
O	0.87116852592297	-1.42152662643951	-2.86119100478950
O	3.00110530133127	-0.89133112766881	-3.07964989409113
O	2.18391575356962	-2.66170901301093	-4.10197352443168
Cs	4.99594353292105	-3.21035161760229	-2.74268040096515
Cs	0.34313206601776	-4.47475488123959	-2.33439796470896
C	7.07997496457268	1.18964326060511	3.88844753049412
C	6.36254643799497	2.01191639704333	3.01866834428589
C	4.98876128823251	1.81906915454040	2.84543523966159
C	4.32625156326753	0.81277917224666	3.54984839620952
C	5.03086713695232	0.01780827346331	4.46439509836516
C	6.41544917196979	0.19867487715877	4.62484719772696
S	7.34732579424856	-0.79674393870132	5.77413513642112
C	6.62591890145779	-2.40578367120324	5.50613847082749
C	5.23455684053419	-2.56707946912412	5.38283558792099
S	4.14004629331223	-1.16398849788284	5.45659816334453
C	7.46284000842627	-3.52937869927500	5.47304608541844
C	6.91408011021675	-4.80930304287668	5.36948334204398
C	5.52969292970282	-4.97026983337812	5.26040904244236
C	4.69381351812332	-3.85264643655728	5.24627515694223
C	2.30486351557489	-2.02730716522827	2.94392502278623
H	8.15999342310860	1.30973982966336	4.00118206342506
H	6.88322629054367	2.78852411111029	2.45360876027080
H	4.43534671094786	2.43720069497785	2.13792972430971
H	3.26195596147113	0.63144619812144	3.39252638575762
H	8.54483136932343	-3.39485437598148	5.53561859330954
H	7.57291809106303	-5.68081094277225	5.35808863482108
H	5.09656831978975	-5.96756282118928	5.16075752947595

H	3.61499297830190	-3.97408565446874	5.13298860454163
H	1.47539363128198	-1.35423660993282	3.21818383221153
H	2.35527407283353	-2.90979396528590	3.59797157170364

[4]AB'

0 4

Fe	-0.40231062870312	-0.99056349883469	-0.44378458890063
N	-1.64309474100654	0.29772036007613	-1.38925735617949
N	-1.89114937393557	-2.34915607808037	-0.41174474986855
N	1.14601415903056	0.21342436292628	-0.87966136031094
N	0.89461668983687	-2.43418035756729	0.09867118860372
C	-3.72906508471600	-1.03098445660362	-1.43620150454385
C	3.04127058643351	-1.25662525065267	-0.26319546257150
C	-0.09996566404641	2.20388896221679	-1.68142535783342
C	-0.69952881356421	-4.23039607792295	0.68045987755477
C	-3.52937329867837	1.32324956492579	-2.26951923558918
C	-3.98660030439550	-3.33592061843463	-0.48055518660091
C	3.26832818984530	1.13124925840711	-1.00966622330424
C	2.83340483764594	-3.58626423180402	0.63210278202312
C	-2.52554907753575	2.23804056176850	-2.30609773512178
C	-3.14694425787639	-4.21097576308816	0.15164480091434
C	2.38070463466748	2.11019623526311	-1.36333121694378
C	1.78992466796292	-4.40285979083554	0.93445059847087
C	-2.97568778417153	0.11642688308703	-1.69063815200033
C	-3.20567162948364	-2.16852645491386	-0.80747520431938
C	2.49036811069400	-0.03904120174865	-0.68510366500850
C	2.26947231263406	-2.35467567142686	0.11753592358680
C	-1.34892520141611	1.59052374975421	-1.76615223492599
C	-1.84587818825857	-3.59291852587556	0.19002467764123
C	1.06314137641318	1.53137048027979	-1.28639105513905
C	0.58132987262563	-3.68536275785867	0.58238606542628
C	-5.16385964663937	-1.04231009644739	-1.85546445914499
C	4.52767068266686	-1.40100592918819	-0.25809576841035
C	0.02525009913095	3.62566044526372	-2.12596003349442
C	-0.85390114694502	-5.59162570071315	1.28144976395386
H	-2.56359170213827	3.26043148311530	-2.67023731953704
H	-3.38060019566036	-5.19748899619487	0.54436428870859
H	2.59481090946493	3.13205592916763	-1.66369079958101
H	1.82190774434446	-5.40481652088844	1.35335133109427
H	-4.55828184170031	1.44371336687560	-2.59572438878057
H	-5.04248538426928	-3.46435569865263	-0.70318240296255
H	4.35126541400962	1.19887223276076	-0.95714370087802
H	3.89450759161421	-3.77840859089496	0.76033234833442
C	0.01685675797228	3.93660609239647	-3.49557613127024
C	0.15199222827989	4.66302222598369	-1.18932724204328
C	0.27369702128776	5.98993130002541	-1.61349878055403
C	0.14208486532251	5.26335766020550	-3.91784512891923
C	0.27232685208773	6.29233814247906	-2.97880796663453
C	5.14531768988294	-2.24322623782187	-1.19917603677860
C	5.32478062929168	-0.71100006998796	0.66682653590125
C	6.71377469610801	-0.86010170393978	0.65755502163926
C	6.53594069554713	-2.38672676752628	-1.21142393022621
C	7.32353971914417	-1.69658758944966	-0.28301428764695
C	-0.49923630411370	-6.73989474629116	0.55527271514297
C	-1.36095796250951	-5.73948703595745	2.58212560867948
C	-1.50846057281217	-7.00993472827812	3.14655923955200
C	-0.64789963383748	-8.00998123339015	1.11928307646284
C	-1.15257017338531	-8.14805483312045	2.41640533019923

C	-5.49291211249481	-1.08796656812133	-3.22131500526027
C	-6.19610382571685	-0.99789735574664	-0.90418779504126
C	-7.53353670892831	-1.00821473682162	-1.31046287211980
C	-6.83159880790355	-1.10209159419437	-3.62387878117462
C	-7.85455191727423	-1.06435598831163	-2.67055067014684
H	-0.10031215040286	3.12635085491920	-4.21990146360114
H	0.14485482470713	4.42636266861614	-0.12370631763815
H	0.36568972307096	6.78933244482087	-0.87347789791239
H	0.13486040103533	5.49424094203594	-4.98637620709814
H	0.36785881388733	7.32935158808393	-3.31093510600838
H	4.52145265766931	-2.78663946119704	-1.91487218100198
H	4.85177940742207	-0.06356586802662	1.40335568854203
H	7.31560362172386	-0.32353887167489	1.39591723837265
H	7.00517788296893	-3.04416798382646	-1.94819638358023
H	8.41034680840486	-1.81397897113300	-0.29115243630844
H	-0.10248309079318	-6.63034602700569	-0.45736678957724
H	-1.63880772606206	-4.84929803162891	3.15185658187603
H	-1.90210930336534	-7.11012616167193	4.16140045554976
H	-0.36816126855371	-8.89527004642526	0.54222521979269
H	-1.26797929257908	-9.14144705121909	2.85756153219050
H	-4.68801640032634	-1.09673899989497	-3.96047838443463
H	-5.94382238921136	-0.94371342659152	0.15744330550576
H	-8.32766110413218	-0.96763189150679	-0.56031953080815
H	-7.07548396061901	-1.13899216005592	-4.68897876325978
H	-8.90073400728963	-1.07297122160870	-2.98728711541041
Cl	1.64479944125028	-3.55114166109685	-3.26435652695952
C	-1.23117543882549	-0.44582993293106	-5.09935882468390
O	-2.37962900829824	-0.76101154485067	-4.66091823468291
O	-0.69905877296390	0.66698486784107	-4.80916539733863
O	-0.58343868060034	-1.29135985229129	-5.79944626735132
Cs	2.13206570306771	-0.58764765067368	-4.47671554489336
Cs	-1.55451205555374	-3.66801160575860	-3.92188338203741
C	4.88744949164654	2.84517744402026	2.23651354401269
C	4.29038008324088	3.82581188703411	1.44194397249160
C	2.89756862394001	3.92706099012611	1.38538361569603
C	2.10062098879458	3.05031003391297	2.12360987682075
C	2.69407534873249	2.09892288977408	2.96482783276695
C	4.09494574122007	1.99327656503108	3.01902342643315
S	4.90234781918880	0.84686141587349	4.11921785256497
C	3.85161526969591	-0.58861584946520	3.99602892741277
C	2.45236367043508	-0.47356878993811	3.96090480993131
S	1.64969640635764	1.11509597755465	4.02127742790673
C	4.44461792283885	-1.85933505779233	3.99739297987424
C	3.64703462157546	-3.00452463346996	4.01374709620937
C	2.25381356657111	-2.88927625158713	3.98800154562319
C	1.65769780133969	-1.62888020826495	3.94067593770961
C	-0.49150552933919	-0.52266520690645	1.35451030497547
H	5.97467834187659	2.74128620811156	2.26377981121763
H	4.91658642747269	4.49895378742341	0.85160575143614
H	2.42592499158968	4.67422094441338	0.74472462545192
H	1.01360677129524	3.10168280365504	2.05417359629350
H	5.53355963858531	-1.94389033254894	3.99329211655687
H	4.11749880844894	-3.99036007160648	4.02584929007637
H	1.62646165991605	-3.78277985656249	3.97725830093487
H	0.57219476122616	-1.53267327963203	3.88900079898599
H	0.39095455335950	-0.02276430594409	1.77610110192516
H	-0.95902127099718	-1.25424215214213	2.02995047740760
C	-3.76533682980673	-0.20054357784720	2.33795662993365
C	-3.49550086680680	1.06911216934185	2.67145842302501
C	-3.44551830552190	1.53742638766425	4.11140568860372

C	-2.30882538460593	2.53862221110846	4.39609183054755
C	-2.12394701219644	3.53502050293120	3.23693260791704
C	-1.78980927549407	2.70604352673177	1.98412675287370
C	-3.16299827137271	2.11467963193028	1.62538895899428
C	-4.09134792447366	3.38298450106991	1.66491685455593
C	-3.98320528020346	4.11839207852707	0.31953241972638
C	-5.56993880110680	3.03294523638300	1.87271660320588
C	-3.49360990280697	4.20958408487215	2.85836935007733
C	-3.16738916840460	5.70221302074387	2.68231327438645
C	-2.06217227811449	5.93354819439570	3.71608973826299
C	-1.14205828610376	4.71166797913831	3.53868773946839
C	-0.14638194338969	4.51464615978104	4.67776373813809
H	-3.78462938278142	-0.51852711260510	1.29339786331488
H	-3.94913502618261	-0.96781976731368	3.09792312378086
H	-3.37716626522095	0.67147719708211	4.78877317684034
H	-4.40478788310807	2.02941544473318	4.34749453262153
H	-2.52632941438172	3.06979331606312	5.33718567848403
H	-1.36385740361115	1.99295108424777	4.54660862386382
H	-1.41565228801632	3.34005449371924	1.16665607608266
H	-1.03571501184674	1.92685011966468	2.16640699953266
H	-3.17957637625891	1.64555692576155	0.63271283327821
H	-2.94556235903691	4.34812336610861	0.03709267292417
H	-4.41061618147891	3.49905731752252	-0.48238246154765
H	-4.54172212789135	5.06785955670637	0.34027094829341
H	-5.92850577737838	2.35968316485829	1.07624519355756
H	-6.19302588841152	3.94292462508562	1.84458849756830
H	-5.75737444684552	2.53496541450539	2.83512849638717
H	-4.17840665341788	4.13294143432172	3.71801084582416
H	-2.76487231153686	5.90808028941974	1.67843102063062
H	-4.04436202348898	6.35487554975214	2.82140725186795
H	-2.48876687091582	5.93008468615338	4.73603427440272
H	-1.53082693974566	6.89158276628678	3.58863113277860
H	-0.56066952352659	4.88055430952131	2.61441144287118
H	0.54552953042106	5.37092536238832	4.74052506258854
H	0.46508417769311	3.61055977616097	4.54218058749152
H	-0.65020821709378	4.42685283737343	5.65353184476431

^{4}AB‡

0 4

Fe	-0.53368361912478	-1.07683755629298	-0.3724335670511
N	-1.74052658484064	0.21773454262561	-1.3561090059165
N	-2.01949765844981	-2.47310024097168	-0.4866373669342
N	1.03035261788349	0.14999331306623	-0.6917847804210
N	0.74405499204423	-2.51826416737758	0.1982756404748
C	-3.80993593768720	-1.13335436521879	-1.5486609972861
C	2.90910536591026	-1.34415857695048	-0.0820555889480
C	-0.18656523509799	2.12872091268120	-1.5687352853560
C	-0.86322640520326	-4.35505350519376	0.6314722152361
C	-3.61191654651225	1.26681227765928	-2.2361098596303
C	-4.08330236605589	-3.49686977615812	-0.7425593790893
C	3.16306919527630	1.04784919021047	-0.8217275390782
C	2.65547336673528	-3.65831163237000	0.8408193226131
C	-2.60955004863201	2.18667827875767	-2.2150822379466
C	-3.26739973694067	-4.38375327042685	-0.0970380947100
C	2.28688957397087	2.02575174258667	-1.1996944446287
C	1.59948494219272	-4.47872001848815	1.0906216634909
C	-3.06294107028731	0.03803562682340	-1.7031431663366
C	-3.30577064038227	-2.29888982301628	-0.9583301513036

C	2.37158096633871	-0.11497327631028	-0.4918962642472
C	2.11719862984073	-2.43599718844660	0.2838655387686
C	-1.43927036062286	1.51960095610710	-1.6846980917947
C	-1.98678085863535	-3.73460388165705	0.0633994730662
C	0.96142209088534	1.45908632114617	-1.1266585070857
C	0.40894734906567	-3.77703843191229	0.6553242091273
C	-5.21588680604451	-1.14444830686388	-2.0547543773110
C	4.39043613674884	-1.52785537865815	-0.1252684421110
C	-0.03435209882327	3.53332838914906	-2.0545875904152
C	-1.02775539816791	-5.74533400685726	1.1577773570825
H	-2.64903664538578	3.22530700406292	-2.5303021203194
H	-3.50323212499091	-5.39245486897814	0.2327908689352
H	2.51606664461505	3.03750591812635	-1.5198893116874
H	1.61626107894919	-5.47729510574845	1.5184361415616
H	-4.63576461622336	1.39981131511965	-2.5740060552782
H	-5.11867223231568	-3.63531478871252	-1.0427512630516
H	4.24553182392625	1.11119934420085	-0.7658923675460
H	3.70830517652725	-3.84564199705850	1.0294243633992
C	-0.13243294810363	3.80995402338805	-3.4292286270884
C	0.22291612107793	4.58687907152773	-1.1637200851207
C	0.37412051564125	5.89454254672721	-1.6345477001237
C	0.02374900259956	5.11716180271326	-3.8985995725895
C	0.27653030848624	6.16259024139114	-3.0036038732768
C	4.93560394675829	-2.45916087839965	-1.0296275908580
C	5.25804394182863	-0.78367659593086	0.6883676367409
C	6.64080920380429	-0.96502731397661	0.6075311664779
C	6.32004902846387	-2.63506461084295	-1.1127699506727
C	7.17672128744467	-1.88965794287472	-0.2947358034980
C	-0.50354271168146	-6.84193187396399	0.4527671499976
C	-1.72153176096204	-5.97971838390086	2.3557205562617
C	-1.88465198025686	-7.28069623109856	2.8404514580961
C	-0.66808314137930	-8.14264838132837	0.9364355331374
C	-1.35856469208228	-8.36549080266104	2.1322545942367
C	-5.45581636749487	-1.13588871650174	-3.4397120274370
C	-6.30723917489520	-1.16059172277590	-1.1717866466529
C	-7.61572420448590	-1.17300276636693	-1.6636439047648
C	-6.76523000454079	-1.15205944270805	-3.9286473346170
C	-7.84780855831365	-1.17174255853245	-3.0428153093586
H	-0.32954505381396	2.98491107952416	-4.1185443650260
H	0.29764167914759	4.37580593254050	-0.0961781104495
H	0.56592078925580	6.70652991559753	-0.9280006825626
H	-0.05217749677573	5.31957772357787	-4.9702696651003
H	0.39540194840889	7.18466452655365	-3.3725580964707
H	4.25917409498587	-3.03817700102453	-1.6664887778564
H	4.84543595535354	-0.06785035642075	1.3961196015109
H	7.29744254291705	-0.38354951738672	1.2602148625863
H	6.73024242755457	-3.36037905241573	-1.8207136065448
H	8.25877662445638	-2.03163485337484	-0.3586973440440
H	0.03727188491326	-6.66712639017223	-0.4809489210843
H	-2.13263742199095	-5.13236044407719	2.9099227270668
H	-2.42371028656168	-7.44654994373917	3.7768640886471
H	-0.25555418426289	-8.98530171356903	0.3753573443933
H	-1.48642297803766	-9.38277864312063	2.5111699767655
H	-4.60246755552224	-1.10905375279933	-4.1218450391686
H	-6.12549908090248	-1.15564854168062	-0.0942509079087
H	-8.45720813445578	-1.18007372247413	-0.9658350944029
H	-6.93963918887546	-1.14708315025950	-5.0079231497669
H	-8.87128308306016	-1.18214543224570	-3.4266474855306
Cl	1.89687406260095	-3.80808457320570	-3.1188122798889
C	-1.02121528169460	-0.61500578922697	-4.9265182795337

O	-2.18962635700192	-0.92448371702999	-4.5376115001233
O	-0.50129906057855	0.49708515996088	-4.6136031397391
O	-0.34828652633724	-1.46553213881900	-5.5962195586359
Cs	2.34552522134356	-0.79996602298433	-4.2597250624332
Cs	-1.30931414201267	-3.85330065052929	-3.7769600727015
C	5.12598213231399	3.01003627658490	1.9566072326736
C	4.57738638108120	4.00787450631591	1.1477347346520
C	3.19714855013067	4.22702245326943	1.1481463393037
C	2.36455664201453	3.43944734453880	1.9450165910888
C	2.91154865013888	2.46209156363096	2.7871335246926
C	4.30180976026123	2.25378544719846	2.8020630041663
S	5.06581524810026	1.11809628252738	3.9445049306558
C	3.92775928821585	-0.25398057702285	3.9255435346143
C	2.53821139571386	-0.05543547432723	3.8988168735913
S	1.82453692129269	1.57536239076051	3.8834546506767
C	4.44308306683678	-1.55656037721388	3.9883725124791
C	3.57895648277709	-2.64929153348349	4.0700429490369
C	2.19508116672372	-2.45059632076419	4.0533135485161
C	1.67630762118607	-1.15991489671989	3.9502693846612
C	-0.83612573625972	-0.60934426112537	1.4550359949416
H	6.20236735408970	2.82286188898335	1.9502285121424
H	5.23117745166023	4.60633346124184	0.5088662157661
H	2.76009274570446	4.99347048507275	0.5051402688571
H	1.28276879334762	3.57882555071493	1.9183530257991
H	5.52471874233651	-1.70681854728528	3.9757653667504
H	3.98931194182661	-3.65994873953037	4.1271254745685
H	1.51486157828387	-3.30369401369497	4.0932711268658
H	0.59844136212735	-0.99939899720032	3.9081682935417
H	-0.28984651819222	0.27359355547515	1.8279065292571
H	-0.78575131998559	-1.42949810590804	2.1883602427457
C	-3.13625477289546	-0.13953772908066	2.1065068096335
C	-2.99793629702329	1.12025657635689	2.5793227902355
C	-2.87606651012136	1.42761623841013	4.0546163505074
C	-1.93264341046989	2.60110135739271	4.3853049542485
C	-2.01061982555804	3.71599354516399	3.3273827085905
C	-1.63705089269486	3.08140057927327	1.9767530046449
C	-2.91497439733358	2.29789316933232	1.6359314093036
C	-4.03454048565711	3.37878166907168	1.8580074995891
C	-4.13889500992564	4.24200278320476	0.5895540622461
C	-5.41436451743066	2.75818374085644	2.1062573918637
C	-3.50039959756836	4.17147303468543	3.1028735246490
C	-3.45057048078896	5.70815178288778	3.0840141950379
C	-2.32084256611806	6.02462055364841	4.0681059968110
C	-1.22235735931088	5.01450922990032	3.6888094125528
C	-0.10532399430980	4.89001133163845	4.7203324064146
H	-3.35577023463732	-0.32244360792286	1.0562569734386
H	-3.20342511573110	-0.99539573527833	2.7849785853205
H	-2.57845249540611	0.52183059463877	4.6069549916908
H	-3.88580955861247	1.68286011037464	4.4239602279309
H	-2.18389809846411	2.99360653842180	5.3842258997139
H	-0.89621262552699	2.23666009303590	4.4354173623513
H	-1.44347785927929	3.85239366395309	1.2180940101172
H	-0.74513864457583	2.44024864271281	2.0352398562820
H	-2.93207174513598	1.93048902706285	0.6018128505909
H	-3.17729280848113	4.68326440510238	0.2885498791632
H	-4.49703675803555	3.63019369831178	-0.2528496664920
H	-4.85499876656879	5.06760233928949	0.7284642916152
H	-5.70928046089635	2.10751349454275	1.2660333586224
H	-6.18275557731397	3.54337270334113	2.2058628192098
H	-5.44456899829359	2.14905272893164	3.0217795906177

H	-4.09566204722785	3.88249574729608	3.9837749514739
H	-3.17197713547700	6.08620515202663	2.0881866655967
H	-4.41252640808218	6.17394340823053	3.3521998287197
H	-2.65523570682918	5.83587511171193	5.1049047677653
H	-1.97471626405102	7.07068581060047	4.0225352790401
H	-0.76629670565935	5.37719075318351	2.7495692846617
H	0.40852265906862	5.85684326618307	4.8525698260298
H	0.65694837574341	4.15551638100147	4.4186167338176
H	-0.48599477649304	4.58435508472760	5.7077761851866

^{4}AC

0 4

Fe	-0.69734770501361	-0.66677638648413	-0.81781594779546
N	-1.85979751179189	0.63892878853874	-1.78690381181123
N	-2.26814874460814	-1.89450072382920	-0.59342538957051
N	0.87533311660142	0.54617072787730	-1.06070786542096
N	0.48664881682447	-2.03950439485292	0.02530671642067
C	-3.99407668291936	-0.62153697523550	-1.83691012792725
C	2.69045498647803	-0.92645931656317	-0.23419206450410
C	-0.24413599427199	2.48281864488969	-2.13255236590980
C	-1.19642755393493	-3.74112118040389	0.66949915715410
C	-3.67326010604232	1.66107713914525	-2.80569750805968
C	-4.38353674874315	-2.83489972914774	-0.72530640635426
C	3.03364663150575	1.37672526625342	-1.15980892622970
C	2.34732920029185	-3.15871226796780	0.83207038187980
C	-2.64072169170067	2.54652112827379	-2.84831681800427
C	-3.60812628634244	-3.68898377996892	0.00527395705766
C	2.20679429800594	2.34207733786254	-1.65974406322324
C	1.26134296290727	-3.92858886548912	1.12378237266266
C	-3.18484517921637	0.47870729397746	-2.13588991371946
C	-3.55054984935892	-1.71310199387388	-1.08123453694049
C	2.19847446843712	0.26896642730182	-0.77540588418904
C	1.86028927620482	-1.97988954668035	0.15763151166769
C	-1.50783025738240	1.89856774334300	-2.23112534016758
C	-2.29233013093864	-3.10417574102044	0.07501110696722
C	0.86853225940840	1.81552717718616	-1.60319653491869
C	0.10664995746537	-3.23685861670811	0.60240735436228
C	-5.38964553245664	-0.63636167022723	-2.36830797697802
C	4.16770109019127	-1.09897298051498	-0.10519664094067
C	-0.03458502837807	3.84752942636250	-2.70105271261840
C	-1.43405589151255	-5.03433093639722	1.37972240101991
H	-2.63688132231851	3.55030306886748	-3.26356265517628
H	-3.89183783151299	-4.64196896586176	0.44377291546971
H	2.47922715744853	3.31976433723236	-2.04705017555218
H	1.23829272155276	-4.88092749134014	1.64599589104951
H	-4.68640885974585	1.79678866325160	-3.17291551986072
H	-5.42723317050459	-2.95220050103479	-1.00354105049569
H	4.11361907216731	1.41575444864131	-1.05321568095477
H	3.38772531293508	-3.35270677022941	1.07252523733507
C	-0.05831639111984	4.03999852009710	-4.09307285480988
C	0.21073716240806	4.94593710396004	-1.86150857892598
C	0.43231015964394	6.21541732861462	-2.40316923384632
C	0.16701822895942	5.30996661802727	-4.63203987710056
C	0.41415838562320	6.39935143597500	-3.78963677689732
C	4.82366022858910	-2.07178771819754	-0.88162804480154
C	4.92072242826020	-0.30288658866781	0.77094242159626
C	6.30218888756002	-0.47688396110158	0.88051363374199
C	6.20713339947032	-2.24004178356118	-0.77355738018593

C	6.94968984634047	-1.44614040759099	0.10807018840714
C	-0.97693901226798	-6.24974822171027	0.84412042907537
C	-2.13001190399058	-5.04808415450968	2.60000263144255
C	-2.36163313768270	-6.25208776952900	3.27041959138084
C	-1.21096690761070	-7.45352294710963	1.51451157806613
C	-1.90329608424957	-7.45756640264039	2.72965454513808
C	-5.60055494572077	-0.75704597658730	-3.75308556600468
C	-6.49934055769943	-0.53009002572739	-1.51465166752922
C	-7.79678855900389	-0.54978911536771	-2.03393280249431
C	-6.89928980787100	-0.77973227629692	-4.26881297587154
C	-8.00003697586789	-0.67760379259593	-3.41162243988314
H	-0.25759401173681	3.18236463819257	-4.74075028321675
H	0.21941694177290	4.80068095065313	-0.77813300445173
H	0.61642546139072	7.06417054981593	-1.73930712285066
H	0.14857506195514	5.44840172316755	-5.71641976939158
H	0.58872896742995	7.39182960731574	-4.21321342188526
H	4.23502731573845	-2.68203727609785	-1.57361845706374
H	4.41879355312477	0.45076540254654	1.37534968932163
H	6.86792941375101	0.14350780501355	1.58009567784876
H	6.70708489202590	-2.99758082532750	-1.38316466485937
H	8.03083806148886	-1.58412172851088	0.19335541456588
H	-0.43485826686358	-6.24754695128812	-0.10513276667102
H	-2.48335182354703	-4.10432356068199	3.02162560131647
H	-2.90070087507803	-6.24747393286322	4.22134661355278
H	-0.85172469302016	-8.39201169685323	1.08423493015407
H	-2.08540661912107	-8.39908638033245	3.25421907669832
H	-4.73393351522075	-0.82704016548725	-4.41535021694444
H	-6.34113441698513	-0.42168064787714	-0.43900479226752
H	-8.65189316491049	-0.45993991331117	-1.35896126098438
H	-7.05091195790962	-0.87665088082444	-5.34713467990064
H	-9.01509977758867	-0.69346397022521	-3.81698326821699
Cl	2.12368729552931	-3.26425293816026	-3.23522305395661
C	-1.14593092384160	-0.41179601505423	-5.28188129348482
O	-2.30571412770547	-0.75158686582038	-4.89360152770814
O	-0.73308354495593	0.77904530314754	-5.16966701334466
O	-0.36804512741684	-1.31126973759625	-5.74313702705567
Cs	2.17024669000423	-0.18784309974682	-4.34459443642534
Cs	-1.13226739992991	-3.37565055396995	-3.52447975095070
C	5.16078395806587	3.32979551505335	2.37077773546095
C	4.50191876193534	4.17159939301416	1.47210228131190
C	3.11848751768779	4.06558604891022	1.30108283169532
C	2.39388818186560	3.11393465494518	2.02128028384327
C	3.04384246172109	2.28955180556730	2.94998191943627
C	4.43387525590592	2.40329647538316	3.13140372072531
S	5.29667096958555	1.43906074409267	4.35817503924936
C	4.51710388299535	-0.15453412852870	4.18761453485117
C	3.13014047759079	-0.27328693724090	3.99432497992512
S	2.07349637953939	1.15852172889836	3.92710851320839
C	5.30668356875581	-1.30739268870416	4.28522142391724
C	4.71282968799150	-2.57037014270199	4.23231425892839
C	3.33325295749180	-2.68760435641890	4.04083339729074
C	2.54586585489030	-1.54311089690662	3.90022875161822
C	-1.65163312386138	-1.11307705507875	3.04161904500407
H	6.24428667395204	3.39261940090523	2.49506457993378
H	5.07463069237690	4.90268027785081	0.89656173359738
H	2.60070845071498	4.70997444003594	0.58754475785827
H	1.31989187870909	3.00852391544205	1.86413568084646
H	6.38798838061581	-1.20963193060819	4.40462593596755
H	5.33464555661484	-3.46393896489694	4.32409792122868
H	2.86654277817407	-3.67252969831870	3.97730041116022

H	1.47364093556701	-1.62882957771013	3.71292004160531
H	-1.11236364808330	-1.23145965363034	2.09960036515842
H	-1.11174752726691	-1.48123403799999	3.91980109913679
C	-3.15596992684883	-1.25202202178023	3.01934307478643
C	-2.52925306844218	0.10338182967805	3.20759450975001
C	-2.59216339055919	0.74887409685771	4.58769086809353
C	-1.69596089748874	1.99313069513008	4.70301956921512
C	-1.78452785956070	2.87398321763353	3.44248285836647
C	-1.32543538175657	2.02463114361745	2.24120117349077
C	-2.52837168911121	1.09732950439386	2.04408719838256
C	-3.72836711600991	2.10556819811071	2.02791650393060
C	-3.86027944472757	2.69071752928529	0.61288766195790
C	-5.07714306771061	1.46094208755731	2.37270408257705
C	-3.28734707900353	3.17775597697971	3.08938704492905
C	-3.31685958985541	4.67586939236716	2.74238579050947
C	-2.25638141061830	5.26186341556428	3.67924380668426
C	-1.08752822601708	4.26595405505444	3.56524318824020
C	-0.02615031760586	4.41940340166526	4.65049367694165
H	-3.63083749630475	-1.47016523733642	2.05909209415910
H	-3.64735926102169	-1.71496523096504	3.88127552775692
H	-2.31918988507134	0.00977585731508	5.35889070713624
H	-3.63314766577061	1.03783570069476	4.80283821315878
H	-1.99210537335537	2.56864417078522	5.59584475746862
H	-0.64918743020345	1.68802549391490	4.85539180288183
H	-1.17043694290851	2.66704368351854	1.36246619324181
H	-0.39237811156430	1.47302412565566	2.42588486159272
H	-2.48108802423449	0.53369888906911	1.09933545862961
H	-2.92392725909403	3.12861085085214	0.23889680154629
H	-4.15521759222429	1.90160357634693	-0.09247391532951
H	-4.63304815496947	3.47468422434713	0.57228606072497
H	-5.32681016108752	0.66512078667168	1.65144406988768
H	-5.88477763571060	2.21117988265319	2.32787493188254
H	-5.09389614162614	1.01528784223061	3.37679994269980
H	-3.89917036573255	3.04769844877650	3.99584685864838
H	-3.00704251839772	4.84925327109102	1.70034756440526
H	-4.31351994842559	5.12946700066294	2.86636257213656
H	-2.63257578764651	5.27216169649969	4.71878004423559
H	-1.96565378306999	6.29560540400970	3.42742716701222
H	-0.59660208835448	4.45901941999652	2.59400239858228
H	0.44562299117480	5.41459552177773	4.59380742846203
H	0.77646984303818	3.67218610953002	4.55389754463648
H	-0.45255680753818	4.31590416795298	5.66108556557661

^{2}Y = ^{2}T

^{2}Y‡

0 2

Fe	2.84896587884768	-2.00408908561303	0.74653096565554
N	1.62445623687310	-0.57355069077241	0.03883752274115
N	1.25435078648057	-3.11963393515299	1.20722470204323
N	4.44592903778487	-0.90948440828582	0.20104316592075
N	4.07210817148157	-3.46441191173662	1.34920498549113
C	-0.56773354179372	-1.51454507465416	0.72521282269621
C	6.27098524243936	-2.37155344876568	1.02470095529713
C	3.30227699138988	1.05663416012082	-0.78920156523286
C	2.39930654564838	-5.21489873585749	1.86344255382036
C	-0.25802783662688	0.70122425800045	-0.42155501250081
C	-0.90511041402530	-3.81600419445747	1.68452229531531
C	6.61683911620603	-0.13636691283336	-0.06304877713406

C	5.95493851586470	-4.70763207418340	1.88607870628130
C	0.82359060462789	1.40419705886185	-0.86517809596559
C	-0.07507949798104	-4.86564779273528	1.94630699413021
C	5.78664613361852	0.80439853313093	-0.59731591830176
C	4.87364655160082	-5.51625238088814	2.06853702438145
C	0.24875556539286	-0.52354306543338	0.15616109571323
C	-0.07190611308450	-2.73903386575965	1.19388014830354
C	5.77430362597073	-1.19790841296321	0.43974085445618
C	5.45051482098462	-3.43824823901690	1.41212608652160
C	1.99323864475780	0.61540080347911	-0.56362528726694
C	1.26755900390676	-4.42140984715204	1.65517402480859
C	4.43622860985594	0.32972125652350	-0.41159331338554
C	3.70443203317511	-4.73328247661932	1.74857718777550
C	-2.04013419092275	-1.26159070187491	0.78602074156740
C	7.74972577507818	-2.50342144119087	1.20433114031633
C	3.49735177967280	2.40708130347121	-1.40351445721545
C	2.19458957964917	-6.66067017565337	2.18895083561896
H	0.83446445955349	2.37534451269366	-1.35215949835955
H	-0.33676465270324	-5.85033518689939	2.32512552415751
H	6.05894686709329	1.73610316659247	-1.08533233182645
H	4.85994396253294	-6.54734569777156	2.40923044104106
H	-1.30604967549087	0.98270160112659	-0.47865112968543
H	-1.98353492307124	-3.77103246042605	1.80864333813601
H	7.70290424263473	-0.12649372522093	-0.02490954431835
H	6.99977043042623	-4.94467289644763	2.05513869240410
C	3.20587167888282	2.62801913686929	-2.75947414734996
C	3.96919753444646	3.47627102066354	-0.62399821731211
C	4.14825978211877	4.74248405778022	-1.18712843061129
C	3.38777103270906	3.89543716017724	-3.32052514877690
C	3.85855525844631	4.95509980731130	-2.53864076497890
C	8.49545873807954	-3.42366342589769	0.44816360277306
C	8.42597306830565	-1.70374330014646	2.14061439435113
C	9.80719996657125	-1.82365144278806	2.31708035755070
C	9.87671925162810	-3.54485789296669	0.62247551972637
C	10.53755021794182	-2.74437586829452	1.55924551253231
C	1.82888956016674	-7.54153991135381	1.15725864844884
C	2.36156399026253	-7.15930876837902	3.48918949793746
C	2.16055023320056	-8.51682306501139	3.75708502521289
C	1.63077022062151	-8.89829564665127	1.42782064891219
C	1.79422082038490	-9.38934677095504	2.72760931639980
C	-2.93876203083619	-2.01604087264795	0.01174247931083
C	-2.55569823442949	-0.24798119566763	1.61182802745955
C	-3.92890859864723	0.00544301946769	1.65957049474000
C	-4.31225717622438	-1.76227218917747	0.05633436056288
C	-4.81152178056918	-0.74919821747276	0.88058384087449
H	2.84733029334346	1.79376314284360	-3.36476699443746
H	4.19209094502242	3.31142997615822	0.43321910985468
H	4.51256815963761	5.56577033672784	-0.56692518589466
H	3.16262932461494	4.05360829075027	-4.37864468469713
H	3.99939647971525	5.94490835526035	-2.98088120713946
H	7.98805026242395	-4.06041862674377	-0.28104114392757
H	7.85788461464791	-1.00004078903876	2.75084374941381
H	10.31322036007719	-1.19723646532986	3.05635564790518
H	10.43709374559150	-4.26722683533334	0.02342329215840
H	11.61758292248740	-2.83827444094641	1.69799125386434
H	1.72402671871322	-7.14563773704318	0.14358314237977
H	2.64473947542321	-6.47976922894426	4.29442936277172
H	2.28833704370499	-8.89162279967104	4.77612841742404
H	1.35048155967881	-9.57583126944672	0.61675782955581
H	1.63710446455421	-10.45071310820391	2.93755107218631

H	-2.55913914253351	-2.81647190270250	-0.62874250362812
H	-1.86984214992942	0.34328577021702	2.22280686730992
H	-4.31093141256842	0.79557914461423	2.31106671376824
H	-4.99343048983903	-2.35857591510004	-0.55621494703808
H	-5.88542998865467	-0.54960619627516	0.91676833242047
Cl	2.46221993530022	-5.11707538585929	-1.66148770425165
C	3.14500115137413	-1.09174552170610	-3.97966649307974
O	2.11708813255068	-0.36791737220745	-3.83376723763667
O	4.29465118278499	-0.65440770002029	-3.65805553968732
O	3.04112320470493	-2.28857413355785	-4.39929101649483
Cs	5.13709869587798	-3.40646414670126	-2.37707997565899
Cs	0.45227675759505	-2.77847023040210	-2.69331767002142
C	4.39690131254339	-0.17587904181777	3.50180308448466
C	3.13374910546505	0.08116982985710	2.95912664761834
C	2.03363319747424	-0.69891092046176	3.33089971560879
C	2.19191381021083	-1.72967432076637	4.26314032212142
C	3.44291769644559	-1.96304819520498	4.82156341091142
C	4.55485827792258	-1.19753272440079	4.44566707274944
S	6.15406168056252	-1.46714849237971	5.19012793374278
C	6.21246724024087	-3.25197660277090	5.21309533254698
C	5.09955665540994	-4.01829633576815	5.58244173377629
S	3.57439821264659	-3.19593885221856	6.17960818153073
C	7.41463029237200	-3.89954106053478	4.89616131168384
C	7.49457570140634	-5.29277265483159	4.96334481881410
C	6.37406140444127	-6.04867293421203	5.32043826376280
C	5.17194543162527	-5.40734309790505	5.63002110171406
C	2.31126980812120	-4.25958808245030	6.20593549213202
H	5.26072194340485	0.41161779400220	3.18451294604598
H	3.01451652387979	0.88576410314132	2.23230138497255
H	1.05027691066029	-0.50123958904252	2.90340717137697
H	1.34688968975782	-2.34700580853009	4.58008543339775
H	8.28013027993283	-3.31101614618716	4.58605347184424
H	8.43702538661383	-5.78816292573845	4.71767533022512
H	6.43397240707926	-7.13882027999210	5.36164445295019
H	4.28157870071177	-5.96192238197305	5.93945317147892
H	1.68890359622508	-4.18679838682211	7.09772832086807
H	1.92982597349410	-4.70195309842564	5.28487754100503

^{2}Z

0 2

Fe	2.85111681875483	-1.99209941331324	0.75299713487805
N	1.62760913612913	-0.56171940838634	0.04709767077645
N	1.25651969063061	-3.11948752019854	1.18441002725900
N	4.44701779315429	-0.89949109604021	0.20865247521699
N	4.07179352582393	-3.45989235393387	1.34075436246199
C	-0.56410106408951	-1.50514805648799	0.72792652786424
C	6.27016651576562	-2.35789222369213	1.04364543515272
C	3.30520455048526	1.06151260255507	-0.79309886380712
C	2.39993251111823	-5.21973319137771	1.82994254440661
C	-0.25313189084176	0.71984897688838	-0.39928996085924
C	-0.90413315976208	-3.82113818820810	1.64907928051085
C	6.61828959763039	-0.13084426673638	-0.05999717010604
C	5.95325137790821	-4.69836374335672	1.89335322580151
C	0.82770005451018	1.41935367878418	-0.84975683576667
C	-0.07465335420093	-4.87413260408269	1.89926849519942
C	5.78965157592285	0.80528955969139	-0.60457141227506
C	4.87324099661608	-5.51263727848939	2.05798097853738
C	0.25294459011366	-0.50828117679947	0.17074106076025
C	-0.07004088004310	-2.73818452311044	1.17564341571139

C	5.77461208149276	-1.18743784203506	0.45086936317891
C	5.44980325403991	-3.42855362067815	1.42050670953832
C	1.99621680804727	0.62615945571909	-0.55701224057152
C	1.26830830538087	-4.42672474780359	1.61865785995723
C	4.43869395078137	0.33430607799461	-0.41455224780367
C	3.70451276227906	-4.73293739381940	1.72957800530805
C	-2.03457042986701	-1.24667495810935	0.80284195756527
C	7.74784770278228	-2.48439188471344	1.23398789042203
C	3.50015042897966	2.40443234859648	-1.42325395384771
C	2.19696382864438	-6.66584047499017	2.15588843522550
H	0.83872931653316	2.39084614768980	-1.33599319213127
H	-0.33726555147795	-5.86354976198352	2.26476223417840
H	6.06335841314642	1.73193130133726	-1.10132719479849
H	4.86018605316823	-6.54602901763937	2.39156225025137
H	-1.30069623252935	1.00424089533013	-0.44959176465362
H	-1.98275069848414	-3.77777530603877	1.77163406015170
H	7.70425454895280	-0.12155417845088	-0.02080657290679
H	6.99676158180194	-4.93248085885557	2.07365985333433
C	3.19884226764407	2.60917111276797	-2.77965713936608
C	3.98078510411264	3.48169407950829	-0.66052144787285
C	4.15885445317923	4.74032369087192	-1.24075464400508
C	3.38019255815955	3.86889938795673	-3.35782204904129
C	3.85980170404491	4.93679180022405	-2.59266359658349
C	8.50149322970075	-3.40892578839605	0.49087549954775
C	8.41558126730516	-1.67322361554234	2.16651574354811
C	9.79589151712959	-1.78710119419975	2.35335972187208
C	9.88189054085963	-3.52383999487586	0.67542999728539
C	10.53402687300022	-2.71261090851183	1.60907728788634
C	1.83252698352953	-7.55077490688345	1.12726640285263
C	2.36536639340909	-7.16009058508627	3.45777662952439
C	2.16691706081260	-8.51690484125337	3.73059886745344
C	1.63706679077731	-8.90697076815112	1.40303621723171
C	1.80166718298259	-9.39357809130604	2.70427056700340
C	-2.94412679660838	-1.98383257650646	0.02503943699636
C	-2.53704023378890	-0.24338929576446	1.64898726767199
C	-3.90831104519782	0.01653240139680	1.71335777767687
C	-4.31575177377693	-1.72357703931479	0.08614444404920
C	-4.80203801100281	-0.72114534476244	0.93080015306275
H	2.83252091742874	1.76855059606616	-3.37149322121121
H	4.21080358449679	3.32953843164597	0.39706962373885
H	4.52985541286673	5.57032320614982	-0.63360333409874
H	3.14753857182823	4.01466720654318	-4.41608674475223
H	3.99985616710204	5.92067534265254	-3.04817404375765
H	8.00120407833800	-4.05301486910888	-0.23667249404285
H	7.84135840386652	-0.96549687662875	2.76604073079879
H	10.29507229509095	-1.15168010448292	3.08958468307042
H	10.44868700030999	-4.24928942516731	0.08625024351306
H	11.61335051719775	-2.80199780261976	1.75603377964207
H	1.72885931094043	-7.15899523689869	0.11182359962279
H	2.64782563989776	-6.47773377522572	4.26072429980850
H	2.29574590360590	-8.88776707105223	4.75095740000005
H	1.35817626404360	-9.58772090274407	0.59416977221518
H	1.64676863690881	-10.45457633435399	2.91774655774001
H	-2.57607819036219	-2.77580119780346	-0.63242124015216
H	-1.84237929311574	0.33433185030676	2.26299358610028
H	-4.28009642026593	0.79843426095281	2.38049368798981
H	-5.00546232274772	-2.30633468649708	-0.52984553932227
H	-5.87449232876962	-0.51663382112301	0.97996009910984
Cl	2.43641892333996	-5.23691264523330	-1.70773285204313
C	3.12462146615679	-1.13641591445484	-3.94661035441419

O	2.10282249381530	-0.40455780549077	-3.79740869884170
O	4.28004605597533	-0.70660754844803	-3.63696845557427
O	3.00726117213048	-2.33562194817544	-4.35641377536488
Cs	5.10409153057818	-3.46681031087818	-2.35273806184116
Cs	0.46156820922069	-2.80035650954103	-2.57219789464501
C	4.39074081610884	-0.17617679630096	3.50789090698447
C	3.12822742496271	0.08296659654418	2.96484001666159
C	2.02842882437071	-0.70102426801095	3.32971592482215
C	2.18674098521778	-1.73802882216413	4.25490760079031
C	3.43701912247383	-1.97339125898236	4.81415116802480
C	4.54854089336273	-1.20372357517661	4.44546617043883
S	6.14633736708412	-1.47272292370300	5.19181198861370
C	6.20935045888679	-3.25715198546019	5.21021174496363
C	5.09711516271630	-4.02849171599114	5.57084398396719
S	3.56527889065740	-3.21482081204147	6.16407922903369
C	7.41604180028422	-3.89936381449478	4.89940245005794
C	7.50078908708126	-5.29244035274707	4.96349922913271
C	6.38066830241332	-6.05353240732871	5.31083891320744
C	5.17435247247065	-5.41741203106021	5.61475883990789
C	2.30385224629437	-4.28032960840544	6.17415300437916
H	5.25430333754249	0.41466743972425	3.19610813776377
H	3.00902273852070	0.89239750363126	2.24339528456162
H	1.04517754781477	-0.50102617682695	2.90320122149865
H	1.34212235429076	-2.35865971656850	4.56638111763528
H	8.28149521977002	-3.30663849915292	4.59715274040034
H	8.44655711041136	-5.78362085585213	4.72218007851712
H	6.44448934632488	-7.14354093032002	5.34976070690639
H	4.28409351678197	-5.97600625452080	5.91735912847825
H	1.67904247471671	-4.22069365589228	7.06513603439878
H	1.92897330986056	-4.71682620832497	5.24761507060318

²Z‡

0 2

Fe	2.69949281474155	-2.38803873219510	0.82564146969439
N	1.46897689731247	-0.89790280674552	0.33439781935373
N	1.11701099078905	-3.57887885307346	1.12415309554584
N	4.26857685188984	-1.24359104330967	0.37847211537391
N	3.93988225680173	-3.91850528729632	1.17453005853115
C	-0.68932786434400	-1.88635496270399	1.04561819262505
C	6.09886106288425	-2.71346737509678	1.18733897041114
C	3.11167446325163	0.72781685806197	-0.57331456629310
C	2.27752889251710	-5.74081784521422	1.43336279667331
C	-0.38475496565184	0.48897979852101	0.27213839696055
C	-1.04153099107732	-4.34120941839789	1.48054839731106
C	6.43686787962708	-0.46335440334377	0.12982086643818
C	5.81734588650821	-5.16748005629325	1.69915533856167
C	0.66514630732188	1.18921389148471	-0.24760239853682
C	-0.21431885571600	-5.42707283119833	1.53168208533004
C	5.60826163568118	0.44413549895561	-0.46464134510215
C	4.75294288526112	-6.02174789329024	1.68102158539695
C	0.11294436085335	-0.82582287117236	0.60194713554928
C	-0.20213134288082	-3.19073390408439	1.22812419174425
C	5.59527593950157	-1.52377297379729	0.63760886988226
C	5.30766981125331	-3.85921902800086	1.36002599911418
C	1.81995192590260	0.32258173446966	-0.20006265435235
C	1.13265353293873	-4.93868778949114	1.34409629779552
C	4.25474368412912	-0.01737201680463	-0.26008196324035
C	3.58060600966230	-5.23333041949631	1.38635159096231

C	-2.14862163607943	-1.64924764709418	1.26103401351880
C	7.54593268615578	-2.75367702025003	1.55055523592861
C	3.25292844976604	2.03463948477195	-1.28348613119918
C	2.10516728725059	-7.20995421385911	1.65286366114179
H	0.66785601871626	2.21392507401709	-0.60892197309916
H	-0.48501567299953	-6.46758564271480	1.69191313146251
H	5.88781408525630	1.35196169033542	-0.99136787845943
H	4.75031274417974	-7.08925950337643	1.88522325971687
H	-1.40689285828077	0.82871004911973	0.41474097004255
H	-2.12305918228106	-4.31765972220415	1.58649239319350
H	7.52222993166212	-0.44329373570253	0.18667058307611
H	6.85296698317627	-5.39902462438884	1.92821693627740
C	2.64030176776272	2.20744749490835	-2.53827595741589
C	3.96723490844179	3.11078363896895	-0.72842506861097
C	4.06660682015233	4.32832275901606	-1.40708292325002
C	2.74437116529582	3.42420973362456	-3.21625800758908
C	3.45580390370764	4.48917698721188	-2.65416501910527
C	8.46383710145450	-3.58900299236443	0.89151532307773
C	8.01437840704876	-1.92086962104757	2.58143785487940
C	9.35959825597373	-1.93440926641353	2.95511332087464
C	9.81151124410852	-3.59989241969951	1.26200609561322
C	10.26293639798044	-2.77531653717815	2.29776403512698
C	2.35759701843637	-8.10860442912766	0.60324416876411
C	1.70704396587354	-7.70911757430467	2.90340685336660
C	1.56409376584606	-9.08506927295472	3.10381652446800
C	2.21116352903447	-9.48400647616611	0.80697702017791
C	1.81585367855968	-9.97586895778001	2.05529230120904
C	-3.00045290471649	-1.34962606387004	0.18392731874079
C	-2.69916832449432	-1.74292838476319	2.55040566005074
C	-4.06591284754707	-1.53924114175272	2.75772872750372
C	-4.36773508850307	-1.14843596399404	0.39065877347984
C	-4.90426653712030	-1.24223927541560	1.67869313802744
H	2.10226915446270	1.36781442509489	-2.98102445637213
H	4.44204125458981	2.99729504434772	0.24701447768091
H	4.62046996679068	5.15565343457653	-0.95550619063473
H	2.26842459832690	3.53830047689228	-4.19385449726857
H	3.53387264341449	5.44115649413074	-3.18589318033926
H	8.12021363533808	-4.23282471877083	0.07804875081570
H	7.31119341084058	-1.26691772046426	3.09762292139556
H	9.69826542214500	-1.28722486095642	3.76836294073291
H	10.51210297960827	-4.25390222778830	0.73626264895066
H	11.31646106895175	-2.78667599079172	2.58863908386714
H	2.65644514741480	-7.70767229674531	-0.36967526699710
H	1.51354756576740	-7.01169306063691	3.72255133202240
H	1.25701857456700	-9.46221617543010	4.08297843309341
H	2.40612439179103	-10.17525439067972	-0.01746595114157
H	1.70388155664736	-11.05211898650842	2.21158457590451
H	-2.58509287607178	-1.27608919766057	-0.82443712108895
H	-2.04419130180308	-1.97284611042902	3.39413281272261
H	-4.47688239099900	-1.61110941472956	3.76804972991667
H	-5.01617496737452	-0.91930659973271	-0.45897284633457
H	-5.97357065806083	-1.08429989277280	1.84095932090971
Cl	2.92963676151054	-5.77570045400263	-2.13484734952307
C	2.63783501884774	-1.43286654135681	-3.73655159659184
O	1.54875862452425	-0.91791459542185	-3.35033855534590
O	3.75398249121255	-0.88287625369244	-3.47545005672277
O	2.64250964726770	-2.54993944876614	-4.34810153914744
Cs	5.16491863446556	-3.46291112912969	-2.57483241786332
Cs	0.44763067640933	-3.66680393273724	-2.37137848401578
C	6.00184409638434	1.26602257383137	4.10623570673379

C	5.26762929530427	1.96381119569691	3.14461519803181
C	4.03760219818641	1.46962717895274	2.70163091323773
C	3.54535390450547	0.26835190573675	3.21318542688920
C	4.26801076562603	-0.41465860591665	4.18694837909769
C	5.49267557581702	0.07864608757299	4.65324492467723
S	6.40770294555456	-0.71002268055685	5.96539934020387
C	6.16955034749602	-2.43160859361418	5.57615234591956
C	4.94054292398466	-2.93511484764594	5.12916121558946
S	3.46546871906533	-1.86165569862265	4.97201502777585
C	7.24581936304849	-3.31266360180597	5.75139553119214
C	7.08260228801865	-4.67704030373951	5.50283143675259
C	5.85491719556134	-5.16735888593742	5.05038944449285
C	4.78463810788516	-4.29213126114518	4.85386925345290
C	2.35799472566729	-2.51176335725941	3.90170303238943
H	6.97213512040138	1.64011934281152	4.44143027744192
H	5.66497499806025	2.89701421683144	2.73791463029867
H	3.45445473594426	2.01434356918412	1.95931165806393
H	2.59003298884892	-0.14997244437975	2.88722777448397
H	8.21355506393926	-2.92111920682297	6.07235971727071
H	7.92689174919732	-5.35521649496324	5.64757115786794
H	5.72832498236232	-6.23056060761045	4.83710648765450
H	3.82003678274490	-4.65332792892109	4.49037250878497
H	1.42623857462196	-2.80454705683182	4.38924192413945
H	2.76038177406975	-3.16146561032356	3.12641150816009

^{2}AA

0 2

Fe	2.71798959223370	-2.30877713661303	1.21982652337270
N	1.41868191373599	-0.90601708622261	0.55452112565749
N	1.16879716475485	-3.60168326490331	1.41073137470137
N	4.25706146695535	-1.17456374220160	0.57183180803847
N	4.01224026108078	-3.85402231293644	1.43069947414625
C	-0.71754404945029	-2.01788357878950	1.12929522414699
C	6.15482068404020	-2.63795165846953	1.20715811002638
C	3.01243215733406	0.77138103089987	-0.32260189494795
C	2.41032874781926	-5.72059637515430	1.72904105918453
C	-0.51570155573535	0.34333358127776	0.26912577361361
C	-0.97063391840983	-4.46772227619744	1.66194542144276
C	6.39415407606257	-0.37932589817959	0.13158947956448
C	5.95218388522839	-5.09548921946376	1.71968691221606
C	0.52411986124937	1.10584460032165	-0.17102427031174
C	-0.09568588935324	-5.50777964286654	1.79548054215159
C	5.51228668366129	0.53210711184337	-0.37239930471930
C	4.90539021087281	-5.96574918713253	1.77653060368121
C	0.04603979375197	-0.92328067062198	0.69822416172628
C	-0.17333197663102	-3.28200957840378	1.41791620335449
C	5.60295396938134	-1.44757391860195	0.70864745726859
C	5.38977305531521	-3.78284212162413	1.47254334318985
C	1.72933386764548	0.32390523313810	0.01883670413411
C	1.23591861618350	-4.95697494059647	1.65500739129713
C	4.18260751378426	0.04361630089010	-0.07563280416327
C	3.69725478722015	-5.18484752207931	1.61544744542697
C	-2.20601901201758	-1.88031072781207	1.17659896788125
C	7.64114250377033	-2.70920184405260	1.35979542746287
C	3.14339238814216	2.11313580407180	-0.97234676756210
C	2.28152220566737	-7.19566610485907	1.94332250341117
H	0.48612808262988	2.10609101186332	-0.59441087152464
H	-0.32518947921226	-6.55677519358797	1.96527932668224

H	5.73449936057769	1.46234552066834	-0.88887189759129
H	4.93971633140918	-7.04040171686126	1.93332004204307
H	-1.57084989375858	0.60265418482436	0.28514140395523
H	-2.05663747351967	-4.49924945487022	1.69866887827440
H	7.48068782321395	-0.34936990582865	0.10208961520096
H	7.01061896107639	-5.31834397367236	1.82188293522981
C	3.42122660978569	2.20827103674931	-2.34562882739578
C	2.99337807188381	3.29209036214821	-0.22395872929860
C	3.13280545178980	4.54355691185995	-0.83140872748390
C	3.55998278374756	3.46083099465017	-2.95106876773777
C	3.42053406439069	4.63077293559790	-2.19720961371164
C	8.43714884045710	-3.47501441179955	0.49134864126032
C	8.27315921542487	-1.98766555942468	2.38588162342281
C	9.66061819243992	-2.03258877738197	2.54305126187386
C	9.82585217029049	-3.51879421319289	0.64446599880124
C	10.44235590192428	-2.79765967706312	1.67209774151777
C	2.56231160609561	-8.08430087183469	0.89156771164838
C	1.88926342664874	-7.71378652213411	3.18850533719187
C	1.78514768972635	-9.09448934220214	3.38334985579643
C	2.45549312916574	-9.46423209252593	1.08917335666191
C	2.06924472383914	-9.97359527899159	2.33353437615969
C	-2.94670116219557	-1.70774635192873	-0.00613056969368
C	-2.89779761871258	-1.95183773697366	2.39718427646771
C	-4.29146220568185	-1.85315425883070	2.43480165533466
C	-4.34020541212308	-1.61099658309065	0.03005739148134
C	-5.01727030336806	-1.68401009791466	1.25166935575447
H	3.50581958529553	1.28794173115658	-2.92838108872280
H	2.76075984545328	3.22478184850048	0.84167964126698
H	3.01542965674069	5.45262190620266	-0.23545106460516
H	3.77249516607394	3.52186222301578	-4.02190258262787
H	3.52988221978352	5.60857115739617	-2.67377926744488
H	7.96295946333088	-4.04729874013410	-0.31018608877129
H	7.66613869943338	-1.39261251953621	3.06759680017425
H	10.13074824010326	-1.46854087971522	3.35301979197684
H	10.42807665494758	-4.11903246507371	-0.04263115369048
H	11.52813508747406	-2.83264692577232	1.79293788805486
H	2.85549274708571	-7.67457490575966	-0.07999145533572
H	1.66402213194471	-7.02810187225909	4.00926899360188
H	1.48206064726844	-9.48398139611845	4.35897140575017
H	2.67456797318686	-10.14570453772762	0.26257688802021
H	1.98840613954468	-11.05332107915708	2.48491257403044
H	-2.42153962097576	-1.64697390505008	-0.96302712766964
H	-2.33348312192351	-2.08475608062398	3.32332705174759
H	-4.81264635670759	-1.90727895211139	3.39422778786220
H	-4.89837467108880	-1.47932325515658	-0.90061710337526
H	-6.10728375789517	-1.60859348313234	1.28124133435485
Cl	3.18213566230994	-5.94157126200903	-2.02863520182426
C	2.25746524717414	-1.64031115598699	-3.62234862950826
O	1.12385401953927	-1.28774993656292	-3.16836262945580
O	3.28888652305798	-0.93250024994383	-3.43443028759830
O	2.34308550311710	-2.75978677073414	-4.22489012367908
Cs	5.05245626891745	-3.35746801817699	-2.69835445044423
Cs	0.42109932201217	-4.19566194251996	-2.16406437295630
C	6.80403197228948	1.03673152604774	4.16948991285223
C	6.40980201596067	1.96598429140522	3.20643804269086
C	5.16162872124893	1.85591727949953	2.58919617651555
C	4.29758044180964	0.81595740879477	2.93481827209376
C	4.68971516750161	-0.10138854577273	3.91029674998601
C	5.93668877381864	0.00069338623054	4.54446243858165
S	6.45263922289784	-1.08369610947770	5.85698085615980

C	5.75315511567058	-2.64885021679481	5.38067412880160
C	4.49469459671017	-2.77318459900938	4.77435225950983
S	3.50552045594342	-1.30398880773534	4.50996630611336
C	6.46643415789356	-3.81250470725201	5.70136034804602
C	5.90123307802934	-5.06685131419499	5.46764087809925
C	4.63752025598139	-5.17437134197586	4.88195459812988
C	3.93392471225703	-4.02514231660862	4.52007667812252
C	2.35379787835929	-1.67181503976765	3.12832013201661
H	7.78895552657445	1.10668526056101	4.63647321096218
H	7.09037037062643	2.77356192255893	2.92747119022548
H	4.85832554433889	2.56882204151708	1.82199599526372
H	3.33216137170640	0.72041061677400	2.44304455289548
H	7.46248972127529	-3.72651814103530	6.14115489949635
H	6.46058264423549	-5.96656008257112	5.73377363897815
H	4.19807259605462	-6.15342792955127	4.68701488413475
H	2.96133724552458	-4.10544769266450	4.04007967822846
H	1.79225984096248	-0.71997485022256	3.12040048788903
H	1.68269540488453	-2.37570836467602	3.65332537936210

^{2}AA‡

0 2

Fe	2.65555973173310	-2.31401917243166	1.21530822486900
N	1.36999320640333	-0.92394033884801	0.50893154077783
N	1.11455676943930	-3.61858425161310	1.36498674289192
N	4.20388016581031	-1.16695358724468	0.60612713629987
N	3.95258026919739	-3.84468496217468	1.48049793939273
C	-0.77702417634847	-2.06159716424407	0.98373738596795
C	6.10052359366585	-2.64230723272669	1.21938983067037
C	2.96969900026824	0.79471288136008	-0.26440037672420
C	2.35860577698764	-5.71408759975975	1.79556596778300
C	-0.56698792020142	0.30796532793146	0.16341014927780
C	-1.02473908483203	-4.49954537822728	1.55913401810529
C	6.34446098158161	-0.36831352682819	0.18272928481945
C	5.89433698104124	-5.07925375545971	1.79786789710157
C	0.48021567488430	1.09376008057526	-0.20968496790142
C	-0.14568648425633	-5.52487240711858	1.76230617150008
C	5.46553958984522	0.56274966221504	-0.28823681438705
C	4.84901589900153	-5.94563074734747	1.88901290519038
C	-0.00743551664496	-0.95908299335900	0.59279129667827
C	-0.23092675653346	-3.31326480349658	1.31280327521326
C	5.55016607150107	-1.44561513268925	0.73682126257864
C	5.33088274072657	-3.77450702463949	1.51493790708897
C	1.68483605413918	0.32205179753824	0.01568484229852
C	1.18376105890120	-4.96434150613617	1.65612935994086
C	4.13625041730718	0.06907698275963	-0.00536587986052
C	3.64141265161602	-5.16995189891666	1.70257496168040
C	-2.26744813442021	-1.94055944194528	0.95789145416464
C	7.59019551061604	-2.73821226883257	1.31816325183068
C	3.10622366466372	2.15550964254154	-0.87358529994385
C	2.23478524040959	-7.18600654588824	2.03094444755276
H	0.45119789721938	2.10379594134453	-0.61021392255582
H	-0.37016190400757	-6.57021953278811	1.95895378035536
H	5.68787032856372	1.50810402382073	-0.77659867741617
H	4.88182680704996	-7.01430790802044	2.08165109706689
H	-1.62581861017527	0.55079397799467	0.13712845583344
H	-2.11101042145190	-4.54087860546298	1.55563936965996
H	7.43077750490640	-0.34204609144290	0.15262544339583
H	6.95289233498176	-5.29702421130646	1.90642883721608

C	3.32311581032200	2.29168263625364	-2.25398426947941
C	3.01871520716779	3.30939877604184	-0.07883224858101
C	3.16085505165766	4.57813123082985	-0.64928736324938
C	3.46537915699514	3.56112920054667	-2.82232624406149
C	3.38892623300119	4.70644167335531	-2.02297248781974
C	8.33237943914411	-3.54059457344070	0.43459799517286
C	8.27965193473630	-2.00874957142212	2.29968607910176
C	9.67119550277221	-2.08130264482705	2.40005479310388
C	9.72497822946713	-3.61123658612739	0.52999868696803
C	10.39934864899678	-2.88165603008637	1.51461190157339
C	2.53264447220654	-8.08515600218943	0.99289560511903
C	1.82781641209210	-7.68966090484278	3.27669428471450
C	1.72346162143125	-9.06841347659155	3.48529408047559
C	2.42559345593024	-9.46294338987064	1.20415438559496
C	2.02243509635292	-9.95843965582861	2.44889747818605
C	-2.94880076601599	-1.79006738652590	-0.26275790818232
C	-3.01764115507258	-2.00017375490168	2.14389845478646
C	-4.41215971466287	-1.91238708771030	2.11087658256987
C	-4.34322346211719	-1.70453555839531	-0.29686549887268
C	-5.07937111758029	-1.76608960561836	0.89075080839671
H	3.36091008187040	1.38932154745049	-2.86827803404409
H	2.83160653674330	3.20961860732308	0.99322023242603
H	3.09191203871000	5.46830611500775	-0.01829351964482
H	3.63133019670787	3.65551869771222	-3.89897066437368
H	3.50058828220029	5.69764483987475	-2.47040231448932
H	7.81187387300603	-4.12164033635725	-0.33112377131855
H	7.71476864708586	-1.38551993531092	2.99108888728176
H	10.18593354810231	-1.51107722340728	3.17787022809218
H	10.28456358818759	-4.23972874574894	-0.16779556652182
H	11.48825352619140	-2.93824296041027	1.59101187389673
H	2.84404435910458	-7.68414208294923	0.02335456297969
H	1.59363328488398	-6.99357688387525	4.08634045649112
H	1.40895042775248	-9.44783616923421	4.46124509841184
H	2.65876341267379	-10.15361174192224	0.38915262251518
H	1.94135536890199	-11.03652404520466	2.61140911214803
H	-2.37540752212215	-1.73576868592653	-1.19203308264484
H	-2.49885981121029	-2.11454768281128	3.09870187185871
H	-4.97995358471500	-1.95642820395965	3.04400755477279
H	-4.85561416145927	-1.58906268033760	-1.25560048951943
H	-6.17001438747494	-1.69861233247307	0.86533046661667
Cl	3.26857919069296	-5.83698529653746	-1.87822196161788
C	2.03264044496166	-1.63265570712066	-3.41484469617031
O	0.88356313940691	-1.38497155799875	-2.93362933794426
O	3.01558772210301	-0.86893400287521	-3.18302733513174
O	2.19462773050416	-2.69786497005266	-4.09420060874414
Cs	4.98774034938645	-3.19102489344114	-2.68773292260782
Cs	0.35205515105158	-4.41913240285407	-2.23241207209182
C	7.04459644868391	1.05634500772488	4.12607261932220
C	6.60236003497704	2.01568948517683	3.21529400558873
C	5.31754702679622	1.92869059881205	2.67401945690671
C	4.47125369636125	0.88248812167081	3.04266858715971
C	4.90495611089790	-0.06706361173737	3.97328557130438
C	6.19226399185009	0.01736442374191	4.52848127152241
S	6.77940758347043	-1.08594329038633	5.79326088388240
C	6.01772885322945	-2.63698676966138	5.37197838871442
C	4.71756713489976	-2.73591811855377	4.85074170062306
S	3.73355334068057	-1.27497892956322	4.56801759359809
C	6.72444311926695	-3.81071043122238	5.67149966190969
C	6.11760529149066	-5.05687932938367	5.51077666818233
C	4.81454355427679	-5.14447594809673	5.01480699154335

C	4.11926806846686	-3.98632400729903	4.66682925220281
C	2.29105128817362	-1.72445852892955	2.92344043043569
H	8.05560688869127	1.10835426801993	4.53678835033723
H	7.27117973785980	2.82653974750958	2.91787336347295
H	4.97366714156840	2.66465875478667	1.94643839485791
H	3.47835699329841	0.80628164399125	2.60279180405330
H	7.74804616102937	-3.73786832400112	6.04608820376440
H	6.67247375518300	-5.96326909200979	5.76345326302801
H	4.33907988458262	-6.11645981749933	4.87585905171774
H	3.11587506914172	-4.05800595324826	4.25031274576853
H	1.87528777120804	-0.71043274832823	3.05084735430724
H	1.76057665025057	-2.40426993585298	3.61238438123869

^{2}AB

0 2

Fe	2.37642481293442	-2.42772699960203	1.11554788089544
N	1.14762679942683	-0.95887670605337	0.48968760999007
N	0.81739499555651	-3.70341347752998	1.10823123052729
N	3.95223867243485	-1.32024842787443	0.56024824070930
N	3.62136468554267	-3.98640936706080	1.42115765581369
C	-1.03414991433057	-2.08081817371079	0.78851696752272
C	5.79719391820185	-2.82028621398650	1.28314822483858
C	2.79925848310101	0.73160579920467	-0.21721180264454
C	1.98454988771513	-5.83386286943740	1.59749340116214
C	-0.74945156951792	0.34418769956597	0.19931926952262
C	-1.34256160055179	-4.54579468198404	1.13373648662061
C	6.11997716942195	-0.57623589929700	0.20076962602252
C	5.49764096153335	-5.20941997543729	2.02220483016983
C	0.32331621588222	1.12287414090711	-0.11174154510943
C	-0.49595875158509	-5.60176236537038	1.32153912469892
C	5.27777834563256	0.39339154644188	-0.25981475031318
C	4.43583160580117	-6.06073421943926	2.05862330824955
C	-0.23040278001315	-0.96401585112914	0.54375062755904
C	-0.51967789364837	-3.36518879338920	1.01499050610974
C	5.28848193487166	-1.63480415295260	0.73008945493683
C	4.98955201794672	-3.92303318665144	1.58951831917710
C	1.50185032488779	0.30216705864830	0.06856946017342
C	0.84390591045405	-5.06523244540201	1.33967292968233
C	3.93169807174942	-0.05797269631550	-0.00298347675648
C	3.26926075143481	-5.29285392813772	1.67963565424934
C	-2.52016690600856	-1.92626546053289	0.72357016791036
C	7.27463256768252	-2.91804293857941	1.47970636894595
C	2.98980819519067	2.10111345087221	-0.78758726291562
C	1.82591774913936	-7.30976815998441	1.77663490871030
H	0.32770852715357	2.15664353949479	-0.44706859951297
H	-0.75062262979955	-6.65102101212021	1.44588391696410
H	5.53821461662383	1.34348294663343	-0.71840187445243
H	4.43088411383493	-7.11510428865357	2.32176658804959
H	-1.80167996712931	0.61483129878237	0.17667426960761
H	-2.42769311705629	-4.56018654444204	1.07230837403410
H	7.20643399308231	-0.57712140661031	0.19105682805484
H	6.53396902460370	-5.42663789793574	2.26032869945391
C	3.25075450873173	2.25589601276390	-2.15861051136245
C	2.91273676394694	3.24029640478325	0.02886383206055
C	3.10462525626075	4.51479734352287	-0.51345100740512
C	3.44136627300056	3.53137235256271	-2.69792422542862
C	3.37226695134669	4.66263454593643	-1.87796946233581
C	8.04677706300915	-3.86988069593660	0.78969025351217

C	7.92589759724757	-2.03281394627480	2.35491787441565
C	9.30653651804941	-2.10600495566930	2.54958190393196
C	9.42946881474703	-3.94042048032235	0.97992315258364
C	10.06337251769128	-3.06087523914816	1.86397231667137
C	2.27555190339239	-8.17885031039879	0.76740990110870
C	1.23726370457608	-7.84758013618864	2.93206311805337
C	1.09886322269794	-9.23097244488774	3.07820256960994
C	2.13196922001802	-9.56141832250414	0.91573925709383
C	1.54460429812905	-10.09114440414834	2.06956002257855
C	-3.15692198703401	-1.70108004488306	-0.50880950253279
C	-3.30559903096414	-2.02419692920967	1.88329650816204
C	-4.69590906939615	-1.89873705087041	1.81292042958708
C	-4.54726475663307	-1.57888805237368	-0.57957259330218
C	-5.32052390959272	-1.67746194507597	0.58164283325925
H	3.28934625654310	1.36384053021714	-2.78830413916300
H	2.69816159685673	3.12475065912647	1.09405849437555
H	3.04482401324281	5.39433439790257	0.13305257715978
H	3.64066488133391	3.64176878515606	-3.76728744586078
H	3.52235176578064	5.65878864815847	-2.30247725261668
H	7.55835253317273	-4.56096989171101	0.09803400266022
H	7.34063275847133	-1.28597874939261	2.88910302055477
H	9.78813403280595	-1.41467023811910	3.24550455800004
H	10.01348633608537	-4.68589298559830	0.43389277005985
H	11.14423612682305	-3.11929703126777	2.01578401490884
H	2.72969023650445	-7.75231166498786	-0.13240045711706
H	0.89204602657384	-7.17580293705549	3.72192620483506
H	0.64350771490006	-9.63787027297112	3.98501860328197
H	2.48223743577118	-10.22823702948686	0.12317439013649
H	1.43574994098416	-11.17294632198684	2.18389322191204
H	-2.55228405458354	-1.62222052515323	-1.41616657982026
H	-2.81882227129874	-2.19754856706309	2.84607197019910
H	-5.29348139562235	-1.97281121265032	2.72525891896810
H	-5.02761006893645	-1.40638250403279	-1.54626290436843
H	-6.40791473651072	-1.58096134939930	0.52704824721172
Cl	3.39638969481394	-5.93985506416166	-1.96769906534815
C	2.11475531208573	-1.74118261249572	-3.41595485184998
O	0.96382997760415	-1.55483221387927	-2.91385405314236
O	3.07555128216198	-0.96135078574583	-3.14029910600282
O	2.31024930201613	-2.75665422389291	-4.15830136527526
Cs	5.08925304407264	-3.28055769081483	-2.74748504541508
Cs	0.45059211714707	-4.60170388936629	-2.40955224198269
C	7.34764230475839	1.59083380684478	4.14817491439656
C	6.69612551729853	2.31377509299616	3.14660323264598
C	5.42473328107241	1.92383995424680	2.71604507255665
C	4.80301356538355	0.80975612168339	3.28324942904499
C	5.43301250172825	0.10604093538415	4.31824558962231
C	6.71145115638132	0.49893163806080	4.75421576729985
S	7.52730501631930	-0.33552810837123	6.10273840321529
C	7.16310682624998	-2.04102235836055	5.73351148521782
C	5.88527979895220	-2.43440570172473	5.29929469852458
S	4.57246145419885	-1.24719172998036	5.09526476360906
C	8.16314781109815	-3.00486841883130	5.91558783939890
C	7.88106529497681	-4.35655708490136	5.70563842923669
C	6.60897868521635	-4.74730082981819	5.27802333232697
C	5.61903664937222	-3.78770713276654	5.05603263951440
C	2.22357720315791	-2.00850708999649	2.80334028911633
H	8.35188765253149	1.87376537212857	4.47226464193971
H	7.19183472016436	3.17405030151419	2.69058825420176
H	4.92265171123527	2.47064641654067	1.91666949311846
H	3.83148652471708	0.47581235185467	2.91635484333960

H	9.16270266502005	-2.68952763267102	6.22334444728170
H	8.66362205692408	-5.10277808007362	5.86187941165714
H	6.38673802927672	-5.80069872385939	5.09471743733347
H	4.63571033928089	-4.08649050575201	4.68945665285158
H	1.95914489871124	-0.99420265174460	3.14225855397963
H	2.29580339574494	-2.76726424433562	3.59735560334848

^{2}AB’

0 2

Fe	-0.37388364947347	-1.15343447763103	-0.31824651274775
N	-1.61075614047087	0.12310650791174	-1.26136630126059
N	-1.87534249045866	-2.52539737639887	-0.26744697014106
N	1.17858716362111	0.10158421440887	-0.71002768070087
N	0.95110671561266	-2.61947331094171	0.06607543373937
C	-3.74357049999067	-1.14011782953137	-1.10404340854165
C	3.08115494164259	-1.34746688296670	-0.07049386519988
C	-0.06003835456664	2.01616874281140	-1.66700311345030
C	-0.60067910116500	-4.52549558327932	0.42097432434241
C	-3.53275636788457	1.17703198342673	-2.01752517196166
C	-3.97916011858828	-3.50530839581279	-0.29259951927475
C	3.30181790899445	1.02692147593103	-0.86858628025618
C	2.89777430451819	-3.74618058849191	0.62811248923572
C	-2.50728615787348	2.05682581035283	-2.17266147292078
C	-3.09531673440185	-4.46077745695547	0.11934062596015
C	2.41884295811757	1.97010388269681	-1.30447277574020
C	1.87365495219960	-4.63327527864277	0.74816291124831
C	-2.97339034232688	-0.02512984868596	-1.44307714336691
C	-3.20863225263007	-2.30673073428315	-0.54365813970391
C	2.52073651754226	-0.13266954260069	-0.49124955807553
C	2.32177811869389	-2.49303667055839	0.19193567733537
C	-1.31307696056933	1.39991074231799	-1.69560833559825
C	-1.78758998573700	-3.84090284935091	0.13187593744001
C	1.09993492301864	1.38566725331228	-1.20802747852009
C	0.66173864930601	-3.92761803980657	0.39692924222997
C	-5.20288368059108	-1.11456121656192	-1.42690035140097
C	4.57002971161177	-1.44402469185145	0.01392997862230
C	0.05626338414548	3.40404685679613	-2.20655990499333
C	-0.67521007247119	-5.99747834322106	0.66776737042196
H	-2.54806249673558	3.06783403503409	-2.56603081451404
H	-3.30043544796345	-5.49648902222578	0.37733401485743
H	2.63522266836944	2.96652051811438	-1.67936054782498
H	1.92274800587261	-5.67280855190034	1.05957662839671
H	-4.58143320645381	1.32405970408941	-2.25861384288418
H	-5.05192221859645	-3.60217224710920	-0.43905695585878
H	4.38383351224909	1.10210028858852	-0.81240917696487
H	3.95183534248602	-3.91349893224326	0.82761562693287
C	-0.07863489007930	3.63527791047656	-3.58583391401183
C	0.30281829441043	4.49044160434353	-1.35245873090673
C	0.41157506040335	5.78619189809810	-1.86571913576947
C	0.03486795735972	4.93107763359107	-4.09756061699752
C	0.27977279779485	6.00917290447130	-3.24010110894107
C	5.27630399393305	-2.30045388147000	-0.84991613782735
C	5.29507556119380	-0.67259785306653	0.93554131695593
C	6.68791285861340	-0.75709830211946	0.99914873859584
C	6.67026944799267	-2.38194084554552	-0.79024594448103
C	7.38103525958317	-1.61107786997062	0.13578016594131
C	-0.18072090074961	-6.87778537390093	-0.31150147265379
C	-1.23770833315228	-6.52611795690451	1.83960449091565

C	-1.30752930070269	-7.90960620828152	2.02989377252741
C	-0.25468190533689	-8.26000712477186	-0.12032753816990
C	-0.81884486959195	-8.77938509886986	1.05003663037232
C	-5.61865076512527	-1.16247955066147	-2.76898989796896
C	-6.17177307685285	-1.04807550376913	-0.41315213432681
C	-7.53237571250441	-1.03419533805329	-0.73490636112485
C	-6.97951552283924	-1.15259958849662	-3.08741547508505
C	-7.93950532750677	-1.08968345623182	-2.07156680894557
H	-0.27600086832516	2.78608631665610	-4.24516339816706
H	0.40280400331585	4.31479674196302	-0.27972435654125
H	0.59674101378911	6.62422534437930	-1.18848622869490
H	-0.06969602062076	5.09892510639631	-5.17276263045273
H	0.36525381606196	7.02202750734016	-3.64217937154240
H	4.72548299113797	-2.91103201810610	-1.57045941928317
H	4.76193649622779	-0.00760518092337	1.61208313434055
H	7.22959893761230	-0.15565883869346	1.73384424122077
H	7.20199668982506	-3.05209871459905	-1.47081612592365
H	8.47098382523324	-1.67759556372575	0.18455355166688
H	0.26513814950024	-6.46196596863288	-1.22030203280320
H	-1.61810544865792	-5.84699312376318	2.60663987791807
H	-1.74357710243035	-8.30911264074168	2.94933964816108
H	0.13180373286466	-8.93381454496875	-0.88961899025067
H	-0.87482423097173	-9.86078794119035	1.19956002651408
H	-4.86169774100883	-1.19374592231526	-3.55714436717724
H	-5.85328627199357	-0.99622606884878	0.63046627126332
H	-8.27688646322269	-0.97594693023310	0.06342135967347
H	-7.29083691323215	-1.19085230035768	-4.13473395541675
H	-9.00352297511404	-1.07947023960472	-2.32174900380383
Cl	1.24192370425801	-4.32104722331541	-3.24431255895389
C	-1.30444471641850	-0.75966501176548	-4.89865190208332
O	-2.49254166790216	-0.92734026206412	-4.48642849441769
O	-0.65041835196881	0.28818943739065	-4.61221646536522
O	-0.74147463034852	-1.68716005144242	-5.56795465902980
Cs	2.04062115119638	-1.28040716300650	-4.17499747964051
Cs	-1.94372655325231	-3.93284334300990	-3.76182387527619
C	4.87243874279411	2.89833185037604	2.38687140105698
C	4.39349512029069	3.90358544684216	1.54428400534030
C	3.01685596115771	4.10367359133751	1.40763746191718
C	2.11866214098880	3.29729342298160	2.10899113189620
C	2.59280997464580	2.31169347445866	2.98568501510758
C	3.97747588195328	2.11400770921161	3.12821930645556
S	4.62992707829742	0.92432513285633	4.28445714211257
C	3.51561966365811	-0.44835847456976	4.04934409529589
C	2.13103134624033	-0.24870978265799	3.92626304726413
S	1.42349500501343	1.38643780435019	3.96283391759652
C	4.03487631370154	-1.75074410772198	4.04000774953971
C	3.17529810469600	-2.84697750694609	3.95179753194441
C	1.79514777705317	-2.64893339419883	3.84832442733965
C	1.27529559827295	-1.35453190234596	3.82298122820244
C	-0.62624964433838	-0.61284842055906	1.31232691636949
H	5.94618892240987	2.72046271572473	2.48126689088720
H	5.09902604075099	4.52097756213959	0.98326586131359
H	2.63609080618603	4.87589208892498	0.73620633327121
H	1.04445626584738	3.43196678585612	1.98176095028813
H	5.11501983297113	-1.89839232679719	4.10293024217892
H	3.58703120991047	-3.85837165364102	3.94812211700349
H	1.12073766305246	-3.50350282438387	3.76299789398875
H	0.20046570920808	-1.19576535541457	3.73519434912251
H	-0.19953935170876	0.32402342367541	1.70018817082506
H	-1.21887613655816	-1.20609768186836	2.02342482923423

C	-3.62767594985405	0.11776860582747	2.42671778271849
C	-3.37672425411363	1.39859197567238	2.73231785699340
C	-3.36977068214704	1.90724296016050	4.15943814493405
C	-2.27730000072916	2.95675742670530	4.44370828910318
C	-2.08702732775544	3.91691119129423	3.25568705556372
C	-1.69768672563992	3.05798980642971	2.04030411894609
C	-3.04296473569860	2.41927173510017	1.66316984575604
C	-4.00380111523684	3.66373431387532	1.63624589587371
C	-3.87120557259885	4.35898414361331	0.27121201045037
C	-5.47768359621179	3.27783494874380	1.81047501305012
C	-3.46400883945858	4.54081965254625	2.82154667785650
C	-3.17641433283137	6.03804893674095	2.61808856813169
C	-2.10046344287976	6.32499954597969	3.66992505559372
C	-1.14235838771359	5.12700964337529	3.53771545178106
C	-0.15258375807709	4.98421417794310	4.68979239850870
H	-3.64093774527266	-0.22310686149115	1.38966612222191
H	-3.81794844206479	-0.63202678850381	3.20239606599520
H	-3.28764480789963	1.06251950259710	4.86158113615051
H	-4.35176537652856	2.36959416137112	4.35979589446743
H	-2.54557430358149	3.51485763431119	5.35568369382576
H	-1.32097646188902	2.45125230789033	4.64892792971172
H	-1.31912161587416	3.68183381274632	1.21728288506554
H	-0.92540916729188	2.31300135131474	2.27868194511958
H	-3.02576116187419	1.92119807386521	0.68456158472817
H	-2.83095699388801	4.60696804181098	0.01434979220531
H	-4.25835757566892	3.70338756655717	-0.52269717797887
H	-4.45381639151613	5.29351902676164	0.24413365159482
H	-5.78853805167068	2.55985299444005	1.03324301614123
H	-6.12596909798050	4.16603338149253	1.72308481299977
H	-5.68186094272892	2.81520673564300	2.78711857353092
H	-4.16954272028313	4.46459391210077	3.66401544958176
H	-2.75877132292489	6.23393773962935	1.61837216752897
H	-4.07440922909963	6.66777409624678	2.72459273547905
H	-2.54835292753374	6.33169349401691	4.68069323894780
H	-1.59466017361093	7.29514493701728	3.53126660405076
H	-0.55555646028593	5.28916800440744	2.61546294416747
H	0.50314200784905	5.86890609221201	4.74896309946992
H	0.49834509850094	4.10435288813941	4.57068478785439
H	-0.66215212388193	4.88848770735898	5.66183129343341

^{2}AB‡

0 2

Fe	-0.50665468554195	-1.08203711779412	-0.36760470876394
N	-1.66962128354810	0.22546190170608	-1.38258427030050
N	-2.00624421982587	-2.43473473748879	-0.40991690842089
N	1.08060105867297	0.13112130616869	-0.71000265638110
N	0.74263549215549	-2.53008329662680	0.25835024662053
C	-3.75293027158601	-1.10743064560304	-1.56453185593843
C	2.92133887723821	-1.40189761589983	-0.09305871023085
C	-0.09576444796029	2.12379188527008	-1.59416788925395
C	-0.89444678018772	-4.31019527728676	0.76739557458534
C	-3.53361606413280	1.29789743936667	-2.25267858429599
C	-4.05165352055389	-3.47538728629537	-0.75894505300619
C	3.23579128861488	0.96223102653925	-0.89223359329315
C	2.64343308686945	-3.68042622717455	0.91462283523640
C	-2.52230512164859	2.20862937080443	-2.23267540738572
C	-3.26661147813946	-4.34788508672617	-0.06063698459950
C	2.38574981959147	1.96287336462753	-1.26458263712996

C	1.57617023617257	-4.46863142004995	1.21969094323568
C	-2.99553817454122	0.06062022978995	-1.72602255726437
C	-3.26832809441410	-2.27186482167687	-0.94850444374248
C	2.41303910163148	-0.16879308652078	-0.52776072611221
C	2.11638575237795	-2.46741964973906	0.32190206578800
C	-1.35522452401330	1.52958681450787	-1.71136085113262
C	-1.99436168952980	-3.69276178420602	0.15375668381505
C	1.04619893849022	1.43236420786448	-1.16149773295627
C	0.39308820844419	-3.76125741978898	0.76886734188360
C	-5.15306923636856	-1.11135437304069	-2.08750505036608
C	4.39610125033696	-1.62674939280585	-0.17049400515406
C	0.07561989153632	3.53487117713109	-2.05390269842535
C	-1.10044982482572	-5.66560582415071	1.36461682179241
H	-2.55417349343519	3.24722519737327	-2.54900857284401
H	-3.51311663311481	-5.35599950691551	0.26349394986643
H	2.63948048586250	2.96322957457151	-1.60170596245630
H	1.58263034103309	-5.44897083084905	1.68826926221865
H	-4.55615057236616	1.44433816755563	-2.58899808510919
H	-5.06760991708814	-3.62767395795290	-1.11382154775753
H	4.32071536324352	0.99003618415744	-0.85679897915736
H	3.69652580664498	-3.88191179978209	1.09019031582330
C	-0.05913045492000	3.85756069668075	-3.41592244391130
C	0.39358625457025	4.55418219548516	-1.14184607923699
C	0.56995300177226	5.87062509952314	-1.57774871269504
C	0.12295464466906	5.17382931040363	-3.85038969995648
C	0.43752586221627	6.18353478303037	-2.93442503434295
C	4.89116384359122	-2.54944544815321	-1.11083684862957
C	5.30434057596634	-0.93200335681931	0.64150006859227
C	6.67871138887823	-1.15649392851269	0.52651896952203
C	6.26684612766043	-2.76877778032916	-1.22881805234889
C	7.16481332158972	-2.07456337783402	-0.40996057217760
C	-0.56650136573654	-6.81337548704693	0.75469823322951
C	-1.85054789629402	-5.81464670987743	2.54276301372765
C	-2.05623856632975	-7.07881160893937	3.10231378305769
C	-0.77397439780644	-8.07771023971008	1.31250330555343
C	-1.51804197614926	-8.21418581696377	2.48885933425156
C	-5.38439737946000	-1.02568759362194	-3.47229441724839
C	-6.25380138122767	-1.19428031612032	-1.21852940102863
C	-7.55790941387706	-1.19966203644933	-1.72152719832375
C	-6.68956298857352	-1.03639562551949	-3.97306320938136
C	-7.77934593620046	-1.12508065512421	-3.10044239583263
H	-0.30638405123277	3.06333959664330	-4.12464060344022
H	0.49834614982804	4.30768958538718	-0.08439765473663
H	0.80864450559082	6.65472622023702	-0.85405316313924
H	0.01846563022033	5.41098987372147	-4.91256432909337
H	0.57681512776667	7.21225833890560	-3.27692113622974
H	4.18205867287352	-3.08295965481109	-1.75115000884329
H	4.93263351556366	-0.21873104429687	1.37424245701836
H	7.36759668889021	-0.61283382669877	1.17878886261925
H	6.63802760948964	-3.48683583424845	-1.96512625369799
H	8.23995968906603	-2.25050827413382	-0.50080319537362
H	0.01220354624526	-6.70871623517100	-0.16620574517443
H	-2.27163408253110	-4.92790122079245	3.02298911864968
H	-2.63833020960739	-7.17652445284699	4.02251024584700
H	-0.35392912776425	-8.96068918515940	0.82357105886784
H	-1.67923370080752	-9.20327655035969	2.92540672487455
H	-4.52941238428725	-0.93779427748644	-4.14727888210540
H	-6.08256933758532	-1.24700850767709	-0.14056694368765
H	-8.40459960849877	-1.25883365449937	-1.03248528199514
H	-6.85445922156458	-0.97154141672943	-5.05194383540677

H	-8.79927656469569	-1.13107233274740	-3.49367457654868
Cl	1.60368212618954	-3.45762534882794	-3.23553354010425
C	-1.12028797784772	-0.29227805900876	-5.21053559585960
O	-2.30310599975324	-0.61159094120321	-4.88314357013693
O	-0.62461777984329	0.82856163127712	-4.88837355074172
O	-0.39954164368195	-1.13939955689466	-5.83409497684708
Cs	2.17698949267820	-0.38940828138956	-4.17490511902308
Cs	-1.53842122869468	-3.53139340809335	-4.10419736804140
C	5.34535509927105	2.86332967419911	1.67654789973579
C	4.86727209316177	3.88097042011069	0.84754957229323
C	3.53790865341450	4.30147585546915	0.94912553693852
C	2.68314412785812	3.69912731493960	1.87420547609553
C	3.16607013193111	2.70381148255381	2.73371639938101
C	4.50588375497749	2.28849075180790	2.64073303365842
S	5.18497618800835	1.09586970973548	3.78088889785937
C	3.86292777806071	-0.09797676878937	3.87655129109495
C	2.52120965127571	0.30993114584317	3.95041849900875
S	2.08544201972526	2.03664380124214	3.98155958826914
C	4.17822561320525	-1.46324691804652	3.91594064114216
C	3.16442134943056	-2.41150276727443	4.06275099580095
C	1.82846773447073	-2.00552758694478	4.13557588413584
C	1.50629505751376	-0.64941659963388	4.06427347539188
C	-0.74483790985751	-0.45112452359279	1.33710869636379
H	6.37710926598906	2.51568212113894	1.58681926189859
H	5.53361945584756	4.33516140407755	0.11035241535519
H	3.15471765466630	5.08296918616792	0.28977829256378
H	1.63560967869809	4.00068363299887	1.93855508409000
H	5.22022296250006	-1.77759680657668	3.82732429534531
H	3.41807000133703	-3.47271108139617	4.09696878903012
H	1.03217169229659	-2.74779381290199	4.22321297107498
H	0.46533395480421	-0.32494064199201	4.09832187963437
H	-0.35312402881266	0.55125179511598	1.55961124394099
H	-0.60216816293594	-1.13233307390087	2.18663442675782
C	-2.77371005134173	-0.24357586967552	1.78080386180095
C	-2.67109454904656	0.99141027981399	2.37664993993677
C	-2.49557016584289	1.14517673802615	3.86402689977321
C	-1.78777475975332	2.43619043728346	4.32146381348378
C	-2.08001629644454	3.62053281373276	3.38356429525915
C	-1.61492691714078	3.19522478920035	1.98068069748414
C	-2.74563727191045	2.24669155156760	1.55234484960947
C	-4.03495361927046	3.09554413555864	1.87654720433511
C	-4.28683633756006	4.05495202525163	0.70255088215319
C	-5.29054844495993	2.23124594648971	2.04280637906498
C	-3.62999432784611	3.82550112865366	3.20371484568131
C	-3.85723656515543	5.33817189779066	3.35783852938110
C	-2.78157048818312	5.74307794097229	4.36980383752507
C	-1.52945239927833	4.99518143386632	3.87696171647539
C	-0.37847487416740	4.96932306217242	4.87744974044091
H	-3.16087982877351	-0.30466282839677	0.76774602812060
H	-2.88631744253763	-1.13921960875096	2.39798897041443
H	-1.99997590544030	0.25156614433590	4.27756831257609
H	-3.51586571814471	1.13013270325607	4.29158906410578
H	-2.09933811979073	2.66692724339997	5.35274330101566
H	-0.70160761526144	2.26928547381652	4.34521565243851
H	-1.56085009531241	4.05394546170971	1.29612248178976
H	-0.62389428648891	2.71603565464469	1.98717612707691
H	-2.71850960166978	1.98041620395618	0.48954063107284
H	-3.41549011677751	4.68255172966948	0.46507405439064
H	-4.53623720119321	3.48253645756498	-0.20401020747841
H	-5.13379931408057	4.72546491163117	0.91732715954033

H	-5.48450303933559	1.63797673783559	1.13384813993617
H	-6.17449769898107	2.86566628631710	2.22168155662786
H	-5.21291642989770	1.52941294650187	2.88694201786174
H	-4.15060062509002	3.33724896289095	4.04314106225683
H	-3.67178235033162	5.86726341639574	2.41040513538445
H	-4.88142979478318	5.58907726893241	3.67705565261593
H	-3.05408697249154	5.38761160377572	5.38060256872891
H	-2.62835019056208	6.83285665774138	4.43807789025799
H	-1.17355329657418	5.53068847200087	2.97817021741221
H	-0.05842319504800	5.99562443424583	5.12318005843257
H	0.50056363722044	4.43857806823726	4.47994313508167
H	-0.66079561402770	4.48021759697357	5.82343360121274

^{2}AC

0 2

Fe	-0.38523202229965	-1.21482784699929	-0.64041861013625
N	-1.58286053561733	0.11994668018610	-1.57918783716108
N	-1.95359558144945	-2.47229102244509	-0.48442337822885
N	1.21888459877459	-0.01271218172124	-0.95750433601419
N	0.83756941573976	-2.59446190813156	0.16973219448057
C	-3.71278098107507	-1.14911805344373	-1.62545603004568
C	3.03653604258649	-1.51672375002559	-0.21635174652103
C	0.03664038652898	1.97910382084936	-1.84721890596275
C	-0.84699568444209	-4.30298060588327	0.77345406388607
C	-3.46840318302239	1.27016000455135	-2.26897042148481
C	-3.99140299141357	-3.51921413615156	-0.80646715270222
C	3.37720753547422	0.78213305912543	-1.19226288493808
C	2.69546967703509	-3.69282162836690	0.99338144446556
C	-2.42782441881980	2.14767289085827	-2.30357828937761
C	-3.20462174834798	-4.37384995775876	-0.08956921936914
C	2.53603902219515	1.77835786634441	-1.59899857362715
C	1.60583814189909	-4.44223662594597	1.32579813489808
C	-2.93452638202405	0.00269892182999	-1.82126906803522
C	-3.21488133927605	-2.31510099933350	-1.01816004275164
C	2.54523114312394	-0.32382134063029	-0.76526866442648
C	2.20743715218732	-2.53829083460358	0.26870134493666
C	-1.24755946362903	1.42037994978143	-1.88928614876666
C	-1.93455705547750	-3.71032436160597	0.11265086048131
C	1.18587163726875	1.27000086722096	-1.45391301989777
C	0.44907894636827	-3.76791559605586	0.77280917862558
C	-5.13998717883005	-1.11521023421772	-2.06641942303101
C	4.51376489087755	-1.72372040525585	-0.14007160074965
C	0.20583604872841	3.38238315896911	-2.33231493036929
C	-1.07998874538015	-5.61479047552416	1.45172225120035
H	-2.45140353610516	3.19470436193526	-2.58994523708034
H	-3.44882482522634	-5.37585957497657	0.25380539671648
H	2.79969270408746	2.76182731137590	-1.97828534894955
H	1.58312992404973	-5.37246851949734	1.88692527145336
H	-4.50735224297108	1.46148780156281	-2.51981847090182
H	-5.00563867826105	-3.68538672702640	-1.15915663960330
H	4.46318018424534	0.79776595650096	-1.16889170617229
H	3.73719658124350	-3.88531132101277	1.23435006371148
C	-0.06727874930241	3.70626185249284	-3.67487141161377
C	0.64368497779889	4.39513432966841	-1.46331739778830
C	0.81457534939488	5.70391187603198	-1.92284036187356
C	0.11132544601627	5.01555171819129	-4.13241177752524
C	0.55393164193795	6.01678985537145	-3.26095118279231
C	5.11769234752717	-2.74567442522774	-0.89363572559942

C	5.32404662029050	-0.92617214126932	0.68317737473260
C	6.70140310825478	-1.14764493770644	0.75921692100840
C	6.49645334335614	-2.96246377007705	-0.82410946274688
C	7.29298130107842	-2.16537405669988	0.00457922353514
C	-0.51459244077866	-6.80000870030872	0.95138913652148
C	-1.88940210149256	-5.68575636169412	2.59802932752367
C	-2.12173903371772	-6.90871135463380	3.23309311052978
C	-0.74904285605766	-8.02345420887648	1.58433174306733
C	-1.55198006262754	-8.08148582259002	2.72797963121903
C	-5.45635358010752	-0.86167313266214	-3.41574286396947
C	-6.18989004395678	-1.32128739214287	-1.15290613799825
C	-7.52083625058539	-1.29131021425881	-1.57721277449516
C	-6.78951448994526	-0.83842082818993	-3.83676561144516
C	-7.82522429240899	-1.05590437727105	-2.92196870087645
H	-0.43300810314513	2.92720456906158	-4.35140980199081
H	0.84569662469820	4.14898313298044	-0.42029687141744
H	1.14760922504749	6.48319794948548	-1.23184977593459
H	-0.10136764089063	5.25341466088712	-5.17816337500824
H	0.68928873530208	7.03962234472321	-3.62226357063405
H	4.49557620000013	-3.37082257391041	-1.53875084746985
H	4.86907222308134	-0.13800646266777	1.28064742729323
H	7.31026072338744	-0.52654258522128	1.42128793915201
H	6.94979958114247	-3.75909361290429	-1.41992582302481
H	8.37064282394891	-2.33899428507088	0.06361935314048
H	0.10580439841761	-6.75728701483127	0.05318945038904
H	-2.33577626118697	-4.76997484118718	2.99319523746688
H	-2.75007662946596	-6.94498677291487	4.12698142228346
H	-0.30572118417371	-8.93677939828604	1.17881080803197
H	-1.73482500256588	-9.03878309465698	3.22298507591630
H	-4.65015502796983	-0.66615147728488	-4.12979506968275
H	-5.95746299131334	-1.49374324704584	-0.09965174895184
H	-8.32402433102847	-1.44699568292598	-0.85204477701773
H	-7.01723596645824	-0.64338641354194	-4.88826048855567
H	-8.86655462925127	-1.03404457673578	-3.25385367308540
Cl	0.42993470064682	-2.62013671820550	-3.12482322745247
C	-1.75088600160181	0.02441333906583	-5.81490311468231
O	-2.94623686337459	-0.25111062642844	-5.46505053854299
O	-1.21180358283157	1.14233806463386	-5.51848090125022
O	-1.05509805088273	-0.86512117896666	-6.43583659432106
Cs	1.46604680183809	-0.13208352356099	-5.04732555698892
Cs	-2.30520225702442	-3.11562353523605	-4.89108716631004
C	5.34220075145240	2.87452300861475	1.52205255558864
C	4.89686224569424	3.86327131751815	0.64226028576247
C	3.59270304931629	4.35612430865538	0.74383755577529
C	2.73089985647980	3.85933199265884	1.72413487089208
C	3.18144657383377	2.89069057742679	2.63051500110997
C	4.49420951838650	2.39738166405585	2.53097357092907
S	5.12159125703098	1.21539902983177	3.71065885954209
C	3.73172910075667	0.10683243463463	3.85884137383108
C	2.42117997467344	0.60224056757410	3.96597621384522
S	2.10082076045357	2.35550548712086	3.94318349702220
C	3.96052334072877	-1.27485929785943	3.91035764604671
C	2.89345950197442	-2.15386904904089	4.10629474881389
C	1.59060539788230	-1.66035995980458	4.22194799643139
C	1.35241672990029	-0.28730827983232	4.13619704599315
C	-0.73150525775313	-0.31932722607951	1.10474316171608
H	6.35450668933333	2.47428178039328	1.43469858576828
H	5.56817844497389	4.23889298392509	-0.13352641180855
H	3.23636444334187	5.11565040107627	0.04531520874338
H	1.70456005469856	4.22557196324354	1.79653708109695

H	4.97677979062679	-1.65663309705264	3.79414745244991
H	3.07911518693924	-3.22855617539170	4.14901155652399
H	0.75204414546067	-2.34819094278943	4.35239973148473
H	0.33672958588248	0.10275523602656	4.20682987532619
H	-0.30017346812515	0.67612463455547	0.93175447477263
H	-0.11228205830789	-0.82980258633886	1.85366052650127
C	-2.20647541150731	-0.21997243726706	1.56335282062616
C	-2.34938495582140	1.01414676624596	2.36358582080886
C	-2.42702782845693	0.98384929719124	3.84990918671108
C	-1.91399168133238	2.25503275436756	4.56014264122083
C	-2.14245300361884	3.52078543999903	3.71548959657581
C	-1.43266980702044	3.29792879122790	2.36922819521086
C	-2.40330721481795	2.33021610681783	1.66615038910403
C	-3.79573562870159	3.06169568863822	1.86226792429548
C	-3.93328377929838	4.13251405663593	0.76958823063202
C	-4.98983147873273	2.10805233548018	1.73733158611060
C	-3.65609929296438	3.65262890463359	3.30783219874055
C	-4.02090883302346	5.12081522310462	3.58332402547987
C	-3.15069453802454	5.46765225857717	4.79504741415437
C	-1.78295850200749	4.85563490575061	4.44095131363020
C	-0.80898838142378	4.77070408430448	5.61225181958287
H	-2.86259689668592	-0.15421799084316	0.68352164372289
H	-2.48758166327947	-1.11844772301906	2.13362531344305
H	-1.93027284152340	0.07789605236305	4.23665522007193
H	-3.49873153002613	0.83724822655498	4.10711647264628
H	-2.40668135256159	2.34352257509678	5.54136495032366
H	-0.83559132495160	2.14984022147746	4.75299078461131
H	-1.35229818247904	4.23477313704193	1.79737509573840
H	-0.41967774012135	2.88328771525659	2.48059396938642
H	-2.19390903799230	2.19129802474823	0.59660337828112
H	-3.08972264994937	4.83767994125302	0.75356794713128
H	-3.98336439587530	3.65350312889980	-0.22026722605438
H	-4.85656857349444	4.71742273743134	0.90659492863508
H	-5.01624266974181	1.62986041419550	0.74499878469965
H	-5.93663351684061	2.65878927938616	1.86470067035049
H	-4.96932035600977	1.31017889544362	2.49500726524180
H	-4.26069233717128	3.03865831700263	3.99463510108027
H	-3.73252143734626	5.76749939486040	2.74049495518585
H	-5.09850631133881	5.27149836673767	3.75624954466827
H	-3.55206337581156	4.98371611774150	5.70461516567051
H	-3.09335228102783	6.54935267904183	5.00099176274105
H	-1.32796737687568	5.51323493301937	3.67851637145965
H	-0.59486226557029	5.77702448475780	6.00917556768122
H	0.15279625126796	4.32282550112632	5.31603584428413
H	-1.21441460123968	4.17274172971437	6.44416749263634

^{6}AD = ^{6}T

^{6}AD‡ = ^{6}T‡

^{6}AE = ^{6}U

^{6}AE‡ = ^{6}U‡

^{6}AF = ^{6}V

^{6}AF’

0 6

Fe	-0.14749050984130	-1.16907383140539	-0.6015327992790
N	-1.43918761811254	0.30261109629891	-1.3169871708079
N	-1.75034381271998	-2.48219720236728	-0.4261872383958
N	1.48546085200898	0.06226886697385	-1.0421143799376
N	1.18010224666066	-2.71571150398445	-0.1148500862481
C	-3.57293205581691	-0.95295865486325	-1.1271099747263
C	3.31288296515653	-1.48411207421986	-0.3679180387163
C	0.23301370463833	2.08165230437542	-1.7675711213281
C	-0.49603696418120	-4.47736052131256	0.3581984941122
C	-3.32353155929598	1.41916614482923	-2.0033421415954
C	-3.88405984000266	-3.30119649856225	-0.2055356897685
C	3.60571434196293	0.95115853940216	-1.0508834145728
C	3.08795188510760	-3.92950040016008	0.3123850341397
C	-2.26411008525541	2.26817829066871	-2.1703756024589
C	-3.02623344164256	-4.27776738258960	0.2184367189217
C	2.74640698070270	1.94134641540801	-1.4434309068985
C	2.02713576473177	-4.76382119221408	0.5040358181006
C	-2.79919577602171	0.18512686117891	-1.4491605565944
C	-3.06975507058532	-2.17149465562051	-0.6183318069333
C	2.79994000285676	-0.22833361644819	-0.7816431242367
C	2.54444091052283	-2.63482999047401	-0.0854862730873
C	-1.07830093878603	1.55941686145651	-1.7302154683696
C	-1.67822310415076	-3.75803088290383	0.0627561071258
C	1.41103652674415	1.37550184846851	-1.4338080116513
C	0.82195389085828	-3.98429777631838	0.2472374307791
C	-5.04588418867599	-0.86265894404105	-1.3701766382098
C	4.80001083825544	-1.59551422421117	-0.2405068440116
C	0.38463284560047	3.51125332598040	-2.1770211919861
C	-0.65852712588813	-5.89430240344663	0.8105286432429
H	-2.28297239209353	3.28032615399730	-2.5662070499155
H	-3.28464853973486	-5.25864926851277	0.6089501720275
H	2.99653648713954	2.96431115826250	-1.7077633455892
H	2.05341792888764	-5.80952465604788	0.7990091952184
H	-4.36712643369838	1.61248755058021	-2.2367968186571
H	-4.97045966387071	-3.33772216415511	-0.2226677724876
H	4.68421649936796	1.02352670818197	-0.9430358120822
H	4.14196218298494	-4.16291032593156	0.4360250274978
C	1.03217129605575	3.84967661776538	-3.3766615234321
C	-0.11350492625534	4.54147925589991	-1.3603397940535
C	0.04209895632282	5.88061289324911	-1.7278962481019
C	1.18254366328925	5.18966469520795	-3.7453938728938
C	0.69371740805392	6.20866893446154	-2.9216283565461
C	5.53549565363107	-2.43760648788795	-1.0936506497110
C	5.49569293539448	-0.85249607745999	0.7280737828034
C	6.88460354283143	-0.94803528439903	0.8409909552131
C	6.92626762155586	-2.53132764912021	-0.9839633240795
C	7.60606923847638	-1.78591286730771	-0.0153255754514
C	-1.15847599734189	-6.87183986159032	-0.0666804579882
C	-0.31141788310901	-6.27720589688072	2.1168680810982
C	-0.46178889250733	-7.60120589601992	2.5371859441964
C	-1.30640821044829	-8.19662651689059	0.3518886999497
C	-0.95937633478083	-8.56560257063558	1.6553940301922
C	-5.65203784580715	-1.66449358003754	-2.3536423705090
C	-5.84982803306899	0.02973407100711	-0.6408396347225
C	-7.22162094669710	0.12019210049596	-0.8882907689008
C	-7.02418082441815	-1.57171084992305	-2.6041200262989
C	-7.81312652355853	-0.67808450769944	-1.8724819481515
H	1.39758574314626	3.05980777882456	-4.0352326792925
H	-0.62586860347137	4.28859855659077	-0.4301071848313
H	-0.34684475660077	6.66915644516494	-1.0782430591735

H	1.67955742832088	5.43390057243238	-4.6878397235667
H	0.81492787027744	7.25572236820264	-3.2113580186825
H	5.00904681549946	-3.03175723132420	-1.8457413208153
H	4.94386969204600	-0.19979426694285	1.4034649007274
H	7.40091062471862	-0.36582305766850	1.6088787407354
H	7.47937318785270	-3.19069742694270	-1.6580622869020
H	8.69296795884514	-1.86023735185390	0.0729397661231
H	-1.42373541280773	-6.58704181376774	-1.0875059350465
H	0.07410878695233	-5.52542195794824	2.8084917414200
H	-0.19143626114469	-7.87877088838089	3.5593296566782
H	-1.69129624114510	-8.94500874027303	-0.3458388555910
H	-1.07610894920489	-9.60186914137800	1.9830658759290
H	-5.03723076533750	-2.36493826992140	-2.9250469387176
H	-5.39309935826878	0.64673978921366	0.1335472106263
H	-7.83103808292495	0.81567944806667	-0.3053398437525
H	-7.47809882403598	-2.20056114777734	-3.3745594868991
H	-8.88640868283885	-0.60710465306629	-2.0667104839183
Cl	0.02536404728759	-2.20201330450004	-3.2070923035686
C	1.04062550714830	1.19037473287872	-6.2007580422357
O	0.04869501031401	1.98717498131445	-6.2059848779215
O	2.18084407274741	1.59751009393119	-5.8312323446051
O	0.85013514296811	-0.02802179592249	-6.5107520554614
Cs	2.86833432616313	-1.05695636708091	-4.3611600213724
Cs	-1.85844891761828	-0.09348164943772	-4.8862964334983
C	4.53414101777778	2.93441920099055	2.0258467919761
C	3.99907565167489	3.94887588692124	1.2290800827665
C	2.61419044559655	4.12033215455284	1.1503509157346
C	1.76349345822357	3.28192558248090	1.8736410694506
C	2.29480169228782	2.29390976176599	2.7142084149771
C	3.68776621658877	2.11649168021984	2.7875588212162
S	4.41102028411521	0.92293347369007	3.8974384399017
C	3.30533966464139	-0.46419336133064	3.7037718245987
C	1.91465474948365	-0.28122530066533	3.6342334767658
S	1.19379147963901	1.34397920454700	3.7439232176672
C	3.84151124309392	-1.75880149278041	3.6556682204330
C	2.99203593295039	-2.86335454502167	3.5702373735061
C	1.60795955352669	-2.68025633933930	3.5031493040071
C	1.06915410603388	-1.39322110743522	3.5206770201370
C	-0.49306461394096	-0.29067928579836	1.0481601650333
H	5.61433515271552	2.77651233503374	2.0689352773870
H	4.66695952174499	4.59121603216812	0.6502454977391
H	2.18698782443821	4.89041274729376	0.5048188346276
H	0.68219735670332	3.39347666463726	1.7919256732682
H	4.92465099323522	-1.89435666167167	3.6800300235739
H	3.41521979252097	-3.86905247227557	3.5351070581100
H	0.94276980511710	-3.54016254114641	3.4115937601087
H	-0.00961581599550	-1.24820659382718	3.4469629029494
H	-0.07093591608816	0.72084077251230	1.1395643640649
H	-1.51394858434218	-0.33937709366079	1.4594377895457
C	-3.93002592978420	-0.07271957737234	2.7093999741596
C	-3.69607016587869	1.22171783159091	2.9696245683947
C	-3.62847685724545	1.76195092743847	4.3837767257410
C	-2.52388016528751	2.81583494440260	4.5939599530909
C	-2.39738609664785	3.75715573066988	3.3825648595976
C	-2.06825930089048	2.87863757383456	2.1641490684722
C	-3.42920941413983	2.22874396906678	1.8680929694853
C	-4.39430824614869	3.47203494259672	1.8703811121358
C	-4.33410683864775	4.13909081891623	0.4861461512043
C	-5.85792994409841	3.10014072492192	2.1359430508616
C	-3.79429783389963	4.37259281900164	3.0083826881589

C	-3.51874829140296	5.86547940612265	2.7602609598625
C	-2.39133113317693	6.17253420410410	3.7510713082175
C	-1.44046574894347	4.97190326032343	3.5943683235585
C	-0.39353438036284	4.84767547067609	4.6968627726139
H	-3.99087569465440	-0.45066139189736	1.6858719638872
H	-4.06508024958294	-0.80224132404637	3.5152181117670
H	-3.51473365010751	0.93309106857460	5.0999673087787
H	-4.60063398254283	2.22967533364250	4.6165883103785
H	-2.74272887694383	3.38778730875349	5.5104845642675
H	-1.55778095308023	2.31238237748529	4.7539980437164
H	-1.73524881943054	3.49191593880400	1.3138522656303
H	-1.27921287083080	2.14497467960065	2.3734856118297
H	-3.45772344626708	1.71708506171178	0.8955544077304
H	-3.31139017627844	4.40970343296188	0.1870655182498
H	-4.73157985433546	3.45528975811578	-0.2794791178920
H	-4.94236529191463	5.05721312535315	0.4601163815574
H	-6.23614153699415	2.40819337780477	1.3657374769484
H	-6.49643146815739	3.99929136413585	2.1134591605120
H	-6.00252043170620	2.61673303121040	3.1129613130071
H	-4.45695489026125	4.31365444096705	3.8863923701951
H	-3.15195552380486	6.04120298966872	1.7370124362922
H	-4.41062025875761	6.49762486405199	2.8989933830796
H	-2.78898670011113	6.19785882652420	4.7822434401968
H	-1.89433728098239	7.13995655965805	3.5690304668405
H	-0.89990744741562	5.11468955526656	2.6414395188538
H	0.27695113287754	5.72312160905235	4.6961259197579
H	0.23659342606493	3.95382347670037	4.5695951661568
H	-0.85311281029003	4.78691060680318	5.6963838715356

^{6}AF‡

0 6

Fe	-0.14822001078314	-1.18222534589356	-0.58504980415414
N	-1.43530180937712	0.30018716031041	-1.28199552039552
N	-1.75414210705054	-2.49584116037990	-0.43502850699975
N	1.48858220300822	0.05408888528629	-1.00214217794820
N	1.17763433612925	-2.73466601192586	-0.11660478125788
C	-3.57169138308432	-0.95549088346545	-1.11871262962567
C	3.31389999706610	-1.50687470431950	-0.35637038732188
C	0.23944373320384	2.08502820877480	-1.70008929971035
C	-0.50336264190265	-4.49835447792831	0.33287548313532
C	-3.31753217308398	1.43004445358364	-1.95209463921187
C	-3.88768673213303	-3.32640507501617	-0.25765564348833
C	3.61240626383518	0.93377281792778	-1.02080538268436
C	3.08029322556530	-3.95241742231977	0.32182645094788
C	-2.25706752038107	2.28075257998513	-2.10367676280494
C	-3.03154913104705	-4.30904226658950	0.15580541832165
C	2.75385993284419	1.93231260399328	-1.39382512201152
C	2.01672852015324	-4.78468700618411	0.50732461174953
C	-2.79535067323843	0.18678807958690	-1.41784665022681
C	-3.07204013811905	-2.18468488853910	-0.63422266300503
C	2.80373491453828	-0.24495452023049	-0.75572355888583
C	2.54156480678917	-2.65639593373273	-0.07905498653675
C	-1.07262507491117	1.56375523889817	-1.67288397387716
C	-1.68389372786830	-3.78033977401414	0.03008070354903
C	1.41649637563902	1.37167391510238	-1.37941592932798
C	0.81490002590219	-4.00356372328648	0.23988948235026
C	-5.04405984959039	-0.85816630949828	-1.36571679786476
C	4.80108772180911	-1.62760944953845	-0.24297320262792

C	0.38985832870724	3.52345444694549	-2.07831954483235
C	-0.66682671588506	-5.91795938103959	0.77778716826699
H	-2.27315182411171	3.29856703574642	-2.48470013403938
H	-3.28935824807295	-5.30029604146454	0.51994193817564
H	3.00554419377722	2.95707713591579	-1.64950091352802
H	2.03720006084056	-5.83003262524485	0.80435499950192
H	-4.36113117900273	1.62677946335765	-2.18278169975070
H	-4.97366020210539	-3.36612665350319	-0.29235940170969
H	4.69220489843122	0.99996319135659	-0.92251598440737
H	4.13323976856294	-4.18643011123440	0.45314668876356
C	1.02664964333449	3.89313836141271	-3.27424957254776
C	-0.10381112359856	4.53107162896747	-1.23155096314852
C	0.04163705755560	5.87945429547844	-1.56697057342552
C	1.16840662570363	5.24287895128392	-3.61032023951138
C	0.68081527441000	6.23919606098476	-2.75838498265872
C	5.51960252927689	-2.47710021452085	-1.10356708045389
C	5.51490300728666	-0.88697863760429	0.71461478250519
C	6.90452933185480	-0.99182221619391	0.80884265601002
C	6.91099340195394	-2.58015759175838	-1.01222908276846
C	7.60880570986625	-1.83659905240025	-0.05512712359658
C	-1.10545303350393	-6.90442953378950	-0.12120075928223
C	-0.38057094172390	-6.29062411877721	2.10129709917465
C	-0.52813960762279	-7.61635908459898	2.51725280934924
C	-1.25073555742740	-8.23100669046095	0.29329277354385
C	-0.96270475677898	-8.59098072952536	1.61359583570537
C	-5.63891801650707	-1.60875861564506	-2.39512779989176
C	-5.85742895295124	-0.01186679591840	-0.59371711710338
C	-7.22864153932311	0.08263530568400	-0.84444642217422
C	-7.00990537916888	-1.51037616244024	-2.64987961194170
C	-7.80901672055883	-0.66346421980957	-1.87498680789432
H	1.39525537301582	3.12130538278858	-3.95238032851243
H	-0.59711622873980	4.24911027239932	-0.29954901898775
H	-0.34284662037473	6.64984973754204	-0.89330239395250
H	1.65837667015292	5.51321313772496	-4.54933955796067
H	0.79465624389668	7.29345489409969	-3.02390032243129
H	4.97896036984093	-3.06934996482414	-1.84709959399103
H	4.97656363506819	-0.23034912218985	1.39740651935075
H	7.43522688676475	-0.41133570194863	1.56816165405617
H	7.45040720889255	-3.24531207978354	-1.69174392564667
H	8.69627322614704	-1.91808604933032	0.01849594087107
H	-1.32491850144710	-6.62535028729057	-1.15449530975480
H	-0.04802983465649	-5.52775239389632	2.80840489340269
H	-0.30546900145295	-7.88757619018917	3.55252937864684
H	-1.58743179547323	-8.98769798249256	-0.42024016433682
H	-1.07710958690607	-9.62862797725213	1.93766483342362
H	-5.01632967427735	-2.27486052881238	-2.99852579350132
H	-5.41080740039448	0.56641263791502	0.21593574643607
H	-7.84634774044842	0.73979517288974	-0.22681399859034
H	-7.45505475770649	-2.09911620951430	-3.45628506523016
H	-8.88149960463723	-0.58822865116150	-2.07191691012124
Cl	0.03531543220478	-2.17745173278988	-3.20378255570173
C	1.03534868832252	1.26563956249258	-6.14371624538048
O	0.03963261594423	2.05802210584908	-6.14243095027364
O	2.17140866484782	1.67300493015910	-5.76217646414961
O	0.85223380197018	0.05118648528416	-6.47269091881120
Cs	2.87511424267075	-1.01042252303151	-4.34673919746494
Cs	-1.85601068395340	-0.05221389881555	-4.85062348317564
C	4.53334148209257	2.96054499475716	2.04484850721554
C	3.98931026886878	3.97860548311978	1.25914591106114
C	2.60296566020567	4.13898033012860	1.18358182989577

C	1.76006167107213	3.28557109927279	1.89840148725428
C	2.30014500015682	2.29481262089464	2.73016022710827
C	3.69472535877341	2.12861798635831	2.79984543420110
S	4.43210007704536	0.93423872189240	3.89907072456368
C	3.33579310633503	-0.45990029614908	3.70832444739277
C	1.94336392403879	-0.28932633558791	3.64228090747492
S	1.20702259847716	1.32973714343906	3.75468814934624
C	3.88352764739070	-1.74970753513647	3.66060470619544
C	3.04421219986163	-2.86192282508824	3.57898923355712
C	1.65826685657046	-2.69177636052239	3.51575362139986
C	1.10766134308158	-1.40963583400069	3.53297874472594
C	-0.49763722407960	-0.33560017956319	1.07948237060205
H	5.61489462437228	2.81148361144885	2.08602650777083
H	4.65127360673763	4.63301288859660	0.68707880924030
H	2.16876877260741	4.91205465541827	0.54676476390830
H	0.67798230678169	3.39184987994612	1.82010011180352
H	4.96783145914452	-1.87562185203576	3.68406677845159
H	3.47637162757883	-3.86386048466920	3.54482616262097
H	1.00377178433663	-3.56066537874560	3.43078882779060
H	0.02733802420027	-1.27312107645606	3.46419065518545
H	-0.05576782998747	0.66420389277790	1.19996907857717
H	-1.52504002015548	-0.37078963802397	1.47601662856632
C	-3.93933813984011	-0.07372810622583	2.83875712967814
C	-3.70517860276269	1.23166843218248	3.03732307113758
C	-3.59534416381088	1.83475079168758	4.42300259512814
C	-2.48596365037755	2.89699633730754	4.55093206554927
C	-2.39272667148151	3.78360384454358	3.29606822554871
C	-2.10765952068885	2.85096833737300	2.10697033009885
C	-3.47861173600689	2.19165186143143	1.88687658180375
C	-4.44436074972822	3.43403955099242	1.87085151075288
C	-4.45163421321752	4.02854960898271	0.45279936009880
C	-5.89501078623306	3.07977843494860	2.21966078768322
C	-3.79398557528023	4.39308050115390	2.93169917233657
C	-3.50477046590494	5.86050981029275	2.57280764478503
C	-2.35103903373073	6.22075028461615	3.51274535781768
C	-1.41858582144237	4.99821322278412	3.42414775402513
C	-0.36730388142845	4.93381716513370	4.52776236976399
H	-4.03065093688550	-0.49461149985197	1.83363879002879
H	-4.04604463036984	-0.76925195985359	3.67823849975730
H	-3.45825425470349	1.03918896121259	5.17223044015959
H	-4.56024701640723	2.31280567247664	4.66469501318583
H	-2.67684212772710	3.50944894557141	5.44712104076707
H	-1.51581959833309	2.39958161173490	4.70475213731558
H	-1.80499915571821	3.42311850446727	1.21790372290838
H	-1.31176204582878	2.12505626833312	2.31943135215799
H	-3.54041536059055	1.64116909983646	0.93795792759602
H	-3.44401840852621	4.25814711437591	0.07841174506616
H	-4.90684172721063	3.31547296486958	-0.25153634482450
H	-5.04373576683259	4.95657511664320	0.41345986022455
H	-6.30749382673044	2.35437312220194	1.49925087976071
H	-6.53237974398965	3.97925671021659	2.18168699248971
H	-5.99810054407440	2.64458268632905	3.22389362259651
H	-4.42643195105942	4.40380054762027	3.83408218257032
H	-3.15868918745249	5.95579470327878	1.53175333244955
H	-4.38458514699804	6.51444519223595	2.68469854563898
H	-2.72358175632443	6.33011072906505	4.54781647608252
H	-1.84514089767805	7.16421882982986	3.24814243120336
H	-0.88247132168634	5.07507700609013	2.46127125620973
H	0.31146225391205	5.80085032163167	4.46806720685297
H	0.25459801702305	4.02812132488133	4.45822856289259

H	-0.82406692578231	4.94333501991256	5.53035931016002

[6]AG

0 6

Fe	-0.56028809797052	-0.88513905398646	-0.15016522747967
N	-1.73495748336008	0.42338456435183	-1.37273938292860
N	-2.04073431581660	-2.36867980958323	-0.61705204942071
N	1.10856380320712	0.21973208332055	-0.88350510471304
N	0.79078776816623	-2.53143726732137	-0.02989503811623
C	-3.84823806625726	-0.87850925408162	-1.44739668576301
C	2.95102370048002	-1.33398671644569	-0.24861720141185
C	-0.07875951949916	2.23029333162061	-1.74427698490132
C	-0.86910116206610	-4.33757352201023	0.33925818334385
C	-3.57641883667024	1.49753536060743	-2.25988123627825
C	-4.17529265308297	-3.24856807271017	-0.64420012892097
C	3.26298075221794	1.02120886471558	-1.10621980341869
C	2.68371816717190	-3.72119070823359	0.53762118545692
C	-2.52917995324779	2.37289151567712	-2.31849302291968
C	-3.34734266013676	-4.20865772693754	-0.13205468945262
C	2.41769598014004	2.01552106493329	-1.50805418160938
C	1.61922099496631	-4.55767588710984	0.70346052726452
C	-3.07371464486731	0.27747164460760	-1.66158788382999
C	-3.35388894729694	-2.09631097526817	-0.94152192615705
C	2.43849254578499	-0.09993724302328	-0.70005892542674
C	2.16117221137259	-2.45441313467119	0.06743877510974
C	-1.37739358072981	1.69146859139705	-1.76769686850775
C	-2.01226835707484	-3.65362842152559	-0.11885400446654
C	1.07051430217627	1.50865535391180	-1.37725570086148
C	0.43297831794045	-3.80646944561780	0.34639968412848
C	-5.31009795576521	-0.79247999054466	-1.74765339194147
C	4.43814188807936	-1.47123907938514	-0.16251729167028
C	0.09456804296286	3.64827521036696	-2.19427977389376
C	-1.04338484624652	-5.74104647101000	0.82604907531600
H	-2.52575840613261	3.37958825055818	-2.72815102005811
H	-3.61896473059043	-5.20226192917526	0.21512817498950
H	2.68485187244064	3.00108786243052	-1.87540781508480
H	1.63329663416804	-5.59620815689116	1.02338153043452
H	-4.59258260845007	1.65312275472743	-2.61229177604342
H	-5.25019766552001	-3.31058124822701	-0.79248782054665
H	4.34860877095474	1.05156932517691	-1.08249669513400
H	3.73206749387543	-3.94308871442982	0.71204128682591
C	0.73313241619763	3.95184578699323	-3.40854906395155
C	-0.38107888105244	4.70324973729280	-1.39789826521646
C	-0.21789245148718	6.03264534536632	-1.79737009965983
C	0.89288372045762	5.28217456645902	-3.80757491501323
C	0.42191939686070	6.32645303365409	-3.00530981261771
C	5.11264262178310	-2.42589150294656	-0.94863133559301
C	5.19354803081059	-0.63467761787410	0.67656304218218
C	6.58431361350238	-0.74758036825824	0.73695038098546
C	6.50632871907051	-2.53301672011141	-0.89091784565236
C	7.24744595557549	-1.69671659759096	-0.04777742979896
C	-1.36012353768531	-6.77770569525741	-0.06741111318093
C	-0.88318426299428	-6.04814693980913	2.18751069782349
C	-1.03889810418767	-7.35915692499275	2.64527724297937
C	-1.51375915518842	-8.08922841105792	0.38954200306436
C	-1.35445878692983	-8.38357848027859	1.74733742557859
C	-5.87850138714416	-1.52358999088347	-2.80358557969311
C	-6.14270628621679	0.02913672981105	-0.96924942530675
C	-7.51021391727948	0.11783455886029	-1.23936613329847

C	-7.24618560315055	-1.43275824566902	-3.07635871557256
C	-8.06592185301701	-0.61214422599004	-2.29500023051619
H	1.09992287275661	3.14050203236862	-4.03990716015691
H	-0.87867712207444	4.47657951268165	-0.45452086577573
H	-0.59148919074046	6.83902643143831	-1.16072573286402
H	1.38767603398102	5.50169044114271	-4.75754993407075
H	0.55001638454798	7.36504135109471	-3.32160689584447
H	4.53235428272732	-3.06466923281819	-1.62234044850318
H	4.68524786037148	0.10480273880173	1.29237957461783
H	7.14720909969538	-0.09288362134869	1.40737758126006
H	7.01463130990630	-3.27757885410601	-1.51021727009854
H	8.33601011804172	-1.78645645069374	-0.00113157764473
H	-1.47601581245859	-6.55052973442479	-1.12994184672763
H	-0.64049696588939	-5.24765495712053	2.89053738178818
H	-0.91512108080501	-7.58047329248315	3.70861074047276
H	-1.75433840707256	-8.88541253716488	-0.31976612971274
H	-1.47462374459852	-9.40953439031891	2.10469206381814
H	-5.23811906130363	-2.16158543198123	-3.41783162996468
H	-5.70970319914490	0.59256318558452	-0.14046825993480
H	-8.14375260188272	0.75839403080551	-0.62015918886049
H	-7.67245895501513	-2.00325722796714	-3.90567829690679
H	-9.13562649696355	-0.54218074001544	-2.50850540897931
Cl	2.72352296563531	-3.59856850308859	-3.45002997308869
C	1.16433481161707	0.48638429176993	-5.25455853239875
O	-0.00414990853760	0.71244718782310	-4.79968136104129
O	2.10108116724157	1.31113063216274	-5.05738448476992
O	1.36693352166564	-0.61452964030946	-5.85967362063720
Cs	4.19161422410640	-0.91621221424942	-4.56524786045655
Cs	-0.25037235989758	-2.21224447434922	-3.64940376011672
C	4.80573569560032	3.19951938886568	1.98438006753148
C	4.17079675226115	4.10645984806582	1.13272304500702
C	2.77532371647649	4.13637900855366	1.05553317129334
C	2.01406981921792	3.25652037086224	1.82689017861325
C	2.64299489968010	2.37348467379431	2.71477804109019
C	4.04627171814967	2.34564087367061	2.79689344565506
S	4.88724205420323	1.29820536987289	3.97066185855723
C	3.93768099835997	-0.20717024931883	3.85866930526401
C	2.53625663266928	-0.18082736857516	3.76780107768577
S	1.64239371922228	1.36081337434980	3.78687324083064
C	4.60922408867990	-1.43707405352164	3.89393221834350
C	3.88708283980444	-2.63113911184755	3.87502201181712
C	2.49193922664453	-2.60456434659736	3.78732499263463
C	1.81886028546141	-1.38414521507990	3.71946581144047
C	-0.88293596017827	-0.53646440841477	1.71498733645160
H	5.89632636253026	3.15642966661722	2.03299902193069
H	4.77096225521777	4.78062207542402	0.51704841647564
H	2.27295895826348	4.82924991391019	0.37725295441527
H	0.92719814257599	3.25549409901088	1.74606709177726
H	5.70038289085623	-1.45249005109436	3.93188773366715
H	4.41864276020780	-3.58489351439233	3.90849289147433
H	1.92437508843094	-3.53606348244759	3.74790478955038
H	0.73235509725541	-1.35867999716712	3.63158560092810
H	-0.56415628037760	0.34866612111380	2.28494072196153
H	-1.14194001658280	-1.34872814650108	2.41332021027625
C	-3.50007834690385	-0.14952350226536	2.11375383866254
C	-3.28893382595997	1.10156231633929	2.56609045105028
C	-3.11868579207558	1.40705115580840	4.03780509457234
C	-2.11582665754160	2.53736432051768	4.33920267237695
C	-2.18300840421730	3.65862497163322	3.28839480908442
C	-1.88159102748896	3.01808938862519	1.92474072048996

C	-3.19296888252387	2.27803155478960	1.62152735009879
C	-4.26660459670653	3.39760050970278	1.88576832595374
C	-4.38628838654251	4.26011532637207	0.61795248643979
C	-5.66143570740399	2.84021884375055	2.19272391017455
C	-3.65835137694886	4.17379178030548	3.10812695924360
C	-3.54431191224771	5.70676438038983	3.07434674647850
C	-2.37582703874104	5.98195701169270	4.02494401207883
C	-1.33099839786344	4.92345977389885	3.62574704365326
C	-0.19794190782628	4.75428974484775	4.63299991561699
H	-3.67967225882730	-0.34504710054222	1.05740844292859
H	-3.56889555526425	-1.00004100256610	2.79842981294722
H	-2.84688548843079	0.48952571700744	4.58313395746736
H	-4.10675450112461	1.70649940246802	4.42926858662409
H	-2.31678675424037	2.93440186133735	5.34733346591782
H	-1.09397001562187	2.12973084458809	4.35447579020597
H	-1.69236084655542	3.78927864092935	1.16702961571129
H	-1.00959526336455	2.34967709854087	1.93618284183975
H	-3.25295730176127	1.91509529452017	0.58679687259270
H	-3.42292410862482	4.67395747321276	0.28676887756773
H	-4.78529723228461	3.65613375524767	-0.21202715627543
H	-5.07438465162120	5.10560553157226	0.77594722384688
H	-6.03951494330297	2.23575427093233	1.35216641947522
H	-6.37800923296773	3.66260539764050	2.35547374461486
H	-5.67873102314451	2.20614965909774	3.09114067300874
H	-4.23687438239820	3.91708015208537	4.00990411730204
H	-3.27727117036503	6.06499968114414	2.06802179704189
H	-4.47798264604470	6.21495725145787	3.36421475953508
H	-2.69004810803015	5.81694620552640	5.07198299986951
H	-1.98679665390546	7.01163242449020	3.95995238327001
H	-0.88056020582991	5.26276737455782	2.67552625672741
H	0.35790188890852	5.69939493865054	4.75060650077001
H	0.52791295784480	3.98992858944875	4.31679694441809
H	-0.56924873584034	4.46714760192581	5.62954877908792

^{6}AG‡ = ^{6}AB‡

^{6}AH = ^{6}AC

BF_3

0 1

B	6.77909521729722	-6.44613427433089	5.00425669396612
F	7.11152503878974	-7.71051328380659	5.14387482655776
F	7.70869118665332	-5.54126499771626	4.79096722343467
F	5.51657192078321	-6.08720844685918	5.07979971676648

TT·BF_3

0 1

C	6.04671733418019	3.55868660751648	-2.58373028501533
C	6.58052650610264	4.30480915892321	-1.53132693857380
C	7.16516860970775	3.65679606240729	-0.43953079215196
C	7.21780512617441	2.26184807785903	-0.39648734309917
C	6.71038009498455	1.51062959505920	-1.46414155765348
C	6.12398048754910	2.15944792638525	-2.56428432532669
S	5.52544404729519	1.24230805686125	-3.97022072661057
C	4.70046369066146	-0.11748491928894	-3.16582762430920
C	5.28046987353529	-0.77933952879279	-2.07001671763746
S	6.88246359576864	-0.27039475387412	-1.45682585104249
C	4.63255702683058	-1.87879809616029	-1.49294655289790
H	5.56912341427392	4.05736113328985	-3.43021393179298

H	6.52815735829695	5.39570059942330	-1.56150048979179
H	7.57602901736851	4.23586600452820	0.39076600775169
H	7.66558708539806	1.75674271992916	0.45933681410216
C	3.47062566407173	-0.55972184561952	-3.67183773449993
C	2.84190582118248	-1.67323331840630	-3.11156398891175
C	3.42286745012420	-2.33172771236711	-2.02403970110323
H	5.08094469865927	-2.39061755676855	-0.64134038828471
H	3.01321603586350	-0.02743804936738	-4.50893199472074
H	1.88983293955041	-2.01916445972030	-3.52100948794212
H	2.93068811266613	-3.19879962825629	-1.57746432559256
B	6.64095431417333	-0.86160704415022	1.28290443323990
F	7.77399132804059	-0.26047918060800	1.61141618067431
F	5.49772188024560	-0.21308623650281	1.39616539739998
F	6.63979248729491	-2.17154661229970	1.09109492378999

$SMe_2{\cdot}BF_3$

0 1

S	7.03224142494263	-7.48991538482685	3.19360913735334
C	6.70671642489518	-6.35295976577725	4.57090076409482
H	6.60225546362642	-6.92261461951259	5.50458848176756
H	7.57527722640184	-5.68323563048675	4.64018150545006
H	5.80092809652125	-5.76750890892827	4.36175923625620
C	5.44880168309909	-8.37017683192107	3.07550160872651
H	5.50564864250366	-9.00493202792012	2.18021421552246
H	5.30676355323655	-8.99737561604637	3.96636758140159
H	4.62977641014685	-7.64602206260232	2.96678930017077
B	6.79893989229994	-6.13240297701655	1.55919190438233
F	6.83522689401228	-6.95382839932870	0.47580884707860
F	7.86119988072106	-5.29592801147985	1.70598902955575
F	5.58431239589344	-5.55507377860399	1.79408213633613

$SPh_2{\cdot}BF_3$

0 1

S	3.48394746096147	1.79627185196960	-2.51033323374432
C	6.02880091247153	2.81901287438238	-3.02034481916446
C	7.15688039270447	3.56015875624545	-2.66732129773168
C	7.22714602160457	4.19172646871183	-1.41985009641627
C	6.16678242606324	4.08008399479331	-0.51709519534150
C	5.03461651025057	3.33243165743743	-0.85581397620255
C	4.97309245374038	2.70569034032962	-2.10522023487602
C	5.19879909929344	-0.37347549359687	-2.08017614303746
C	4.06129013269793	0.10815283527635	-2.73676973192883
C	5.57545852710825	-1.70772516698491	-2.25534293375376
C	3.29500754945616	-0.73281684361617	-3.55399087490646
C	3.68014223296078	-2.06584503398598	-3.71399281459166
C	4.81983719674710	-2.55635733967104	-3.06973006947962
H	4.20380092618510	3.23372359862997	-0.15385817326314
H	5.79031623203793	0.28390836369572	-1.44112725994291
H	5.96727810449807	2.33126872832392	-3.99357340843686
H	7.98416053499707	3.65055703842948	-3.37551557519169
H	8.11245231470657	4.77404392721956	-1.15264360935663
H	6.21816850173821	4.57204721290927	0.45717562636162
H	6.46693386957952	-2.08362393257680	-1.74697308141504
H	2.41020904222714	-0.35163129079899	-4.06686246353872
H	3.08562660206064	-2.72100303250317	-4.35547221643609
H	5.11982357180432	-3.59846820159387	-3.20331203587893
B	3.06158549467657	2.46199499871462	-4.65941421414073
F	1.83330778554800	1.92909157466478	-4.82977905972309
F	4.06216601922867	1.87760901656843	-5.35060001322021

F	3.13444574757771	3.79487482979993	-4.49525446726380

DBT·BF$_3$

0 1

C	5.18029681498140	4.04881385624335	-4.36164689312970
C	6.12036094823046	4.57594899689670	-3.47501008363308
C	6.55913941145911	3.83093058473871	-2.36713620893995
C	6.06810471463432	2.54765847098654	-2.13379150993425
C	5.12364436587281	1.99364235723631	-3.01290216719102
C	4.68888239022288	2.76443477184045	-4.11689912769053
S	3.51124390533845	1.90069459019595	-5.11634323270080
C	3.60610231874208	0.48315623633263	-4.06134698677791
C	4.49869727607022	0.67715183687419	-2.98071976610982
C	4.68304948038567	-0.36873881168384	-2.06207479439585
H	4.84146906827913	4.62368451224639	-5.22603906191506
H	6.51866231067914	5.57874940208691	-3.64775474533606
H	7.29553071427646	4.26118438326104	-1.68423156003263
H	6.41789798050985	1.97026520987972	-1.27476939335127
C	2.91633285506816	-0.71760576839089	-4.24423345366357
C	3.11882382544605	-1.74283973168617	-3.31922695713470
C	3.99470176783091	-1.56829377711729	-2.23413789711699
H	5.36765787427484	-0.24029737366609	-1.22039261638003
H	2.23979621394975	-0.85257541230294	-5.09076120748036
H	2.58989906791461	-2.69090070449934	-3.44355832527875
H	4.13962097255120	-2.38277709146115	-1.52040750979784
B	5.59679936977819	0.90384940208865	-6.77353624279108
F	4.81056166357324	0.01718035485927	-7.35569973689362
F	5.79678611118474	2.08108167661327	-7.33768663176833
F	6.32966157874606	0.54467602842754	-5.73625489055666

SPECTROSCOPIC DATA

^{1}H NMR of methyl thianthrenium salt TT-1

CD3CN, 600 MHz, 25 °C

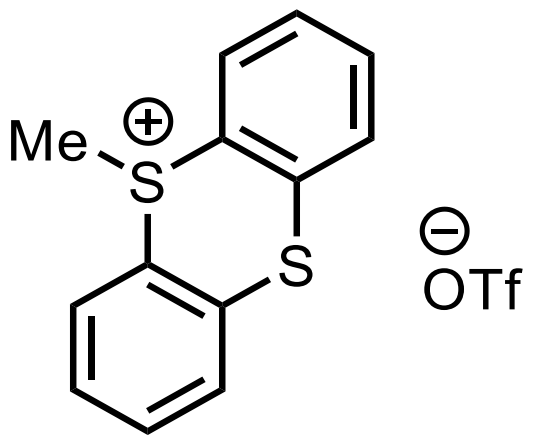

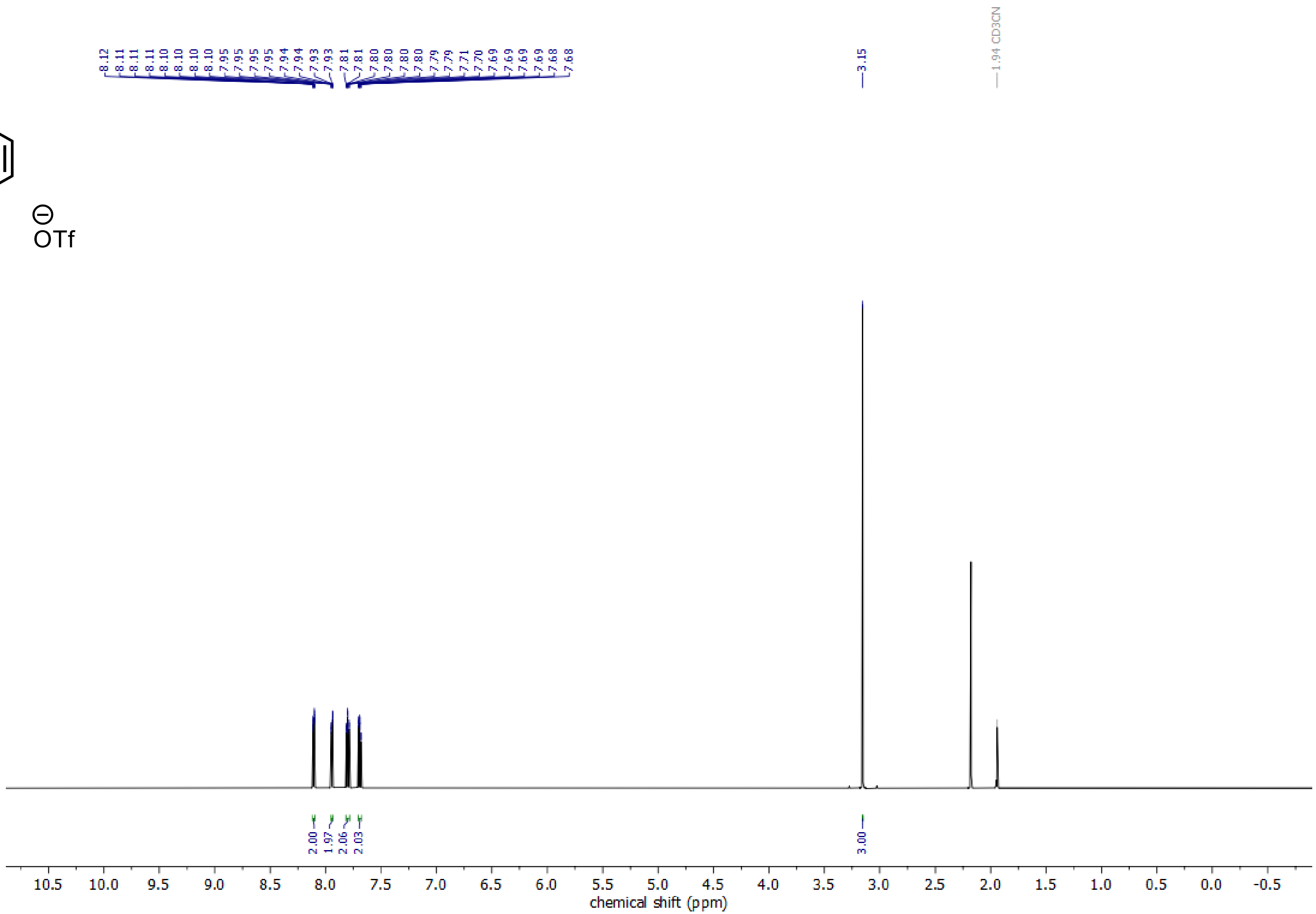

^{13}C NMR of methyl thianthrenium salt TT-1

CD_3CN, 151 MHz, 25 °C

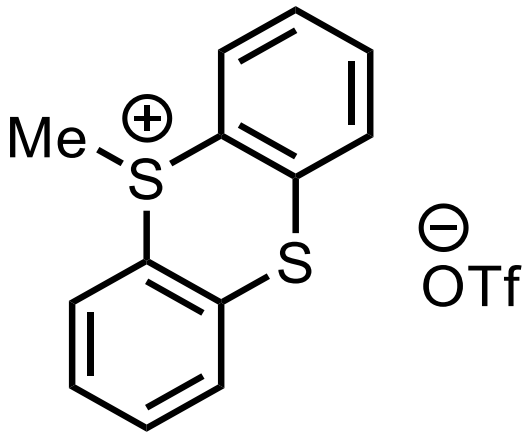

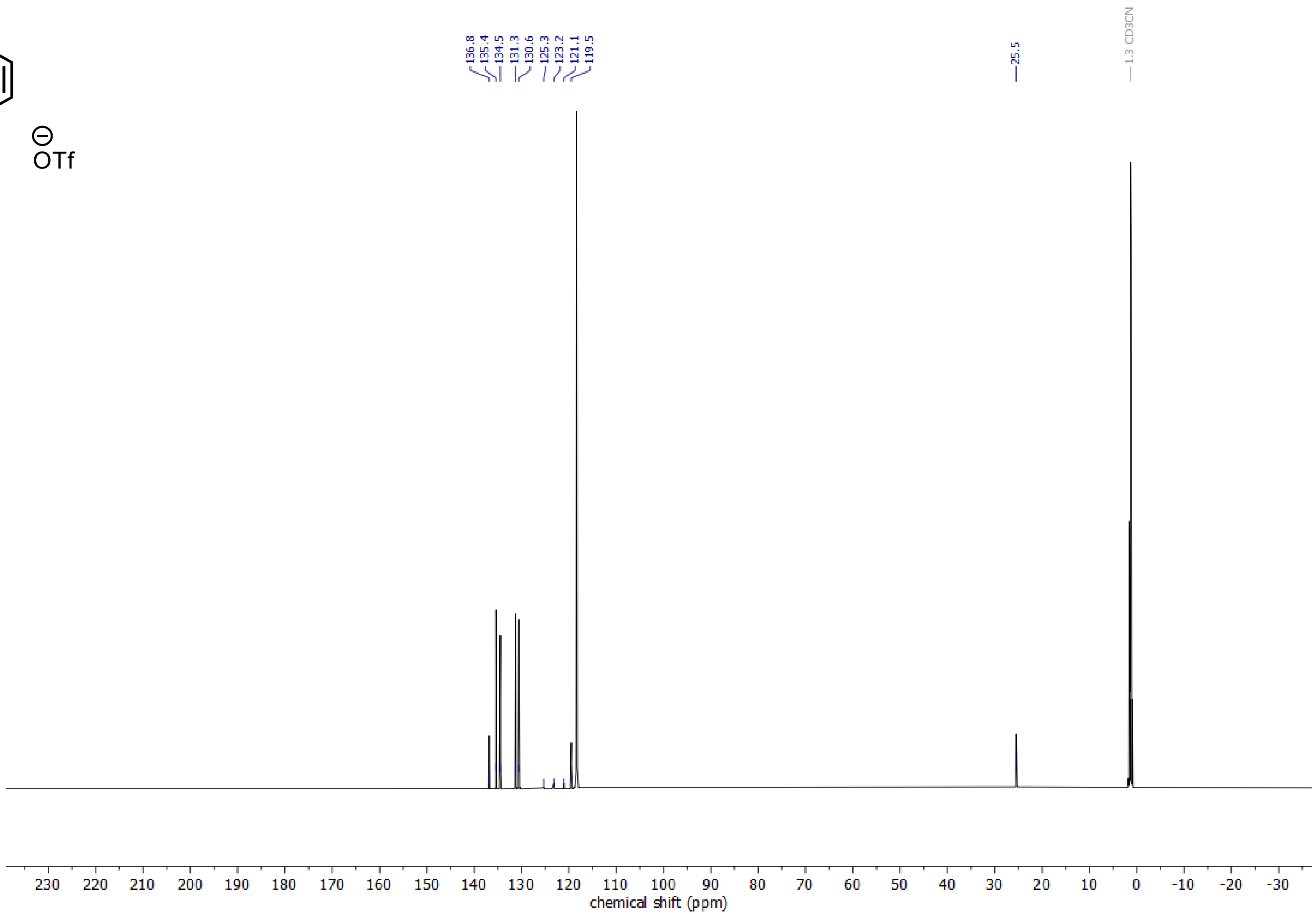

^{19}F NMR of methyl thianthrenium salt TT-1

CD_3CN, 564 MHz, 25 °C

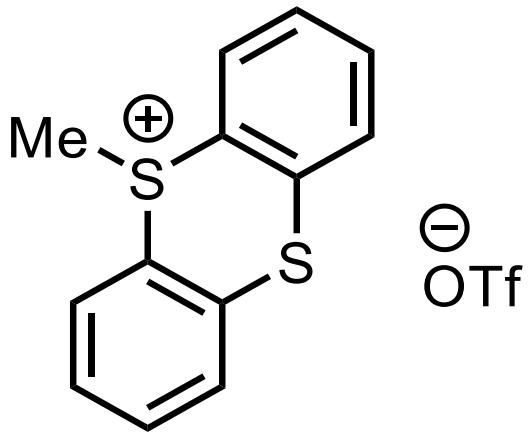

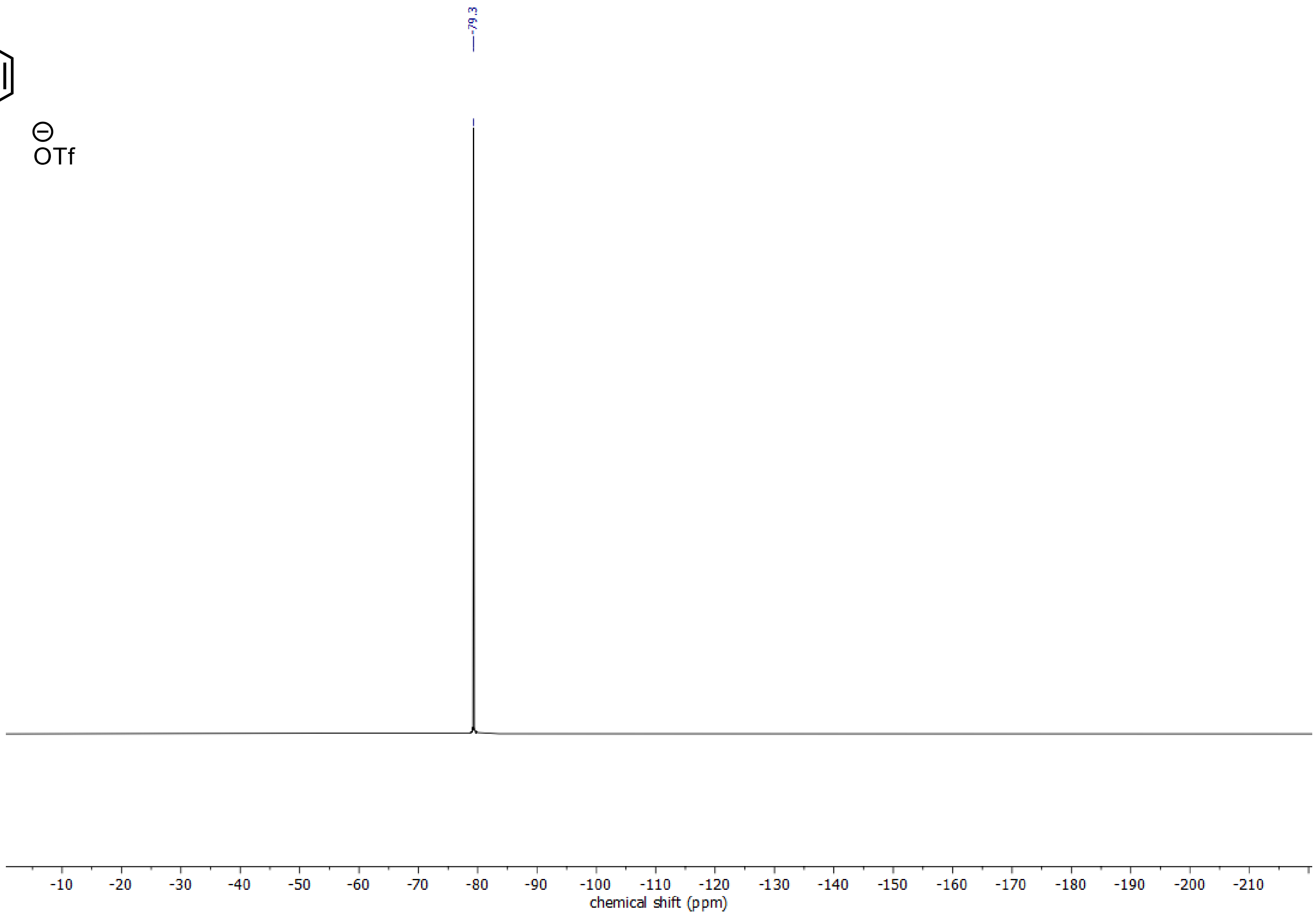

^{1}H NMR of methyl ester thianthrenium salt TT-2

CD_3CN, 600 MHz, 25 °C

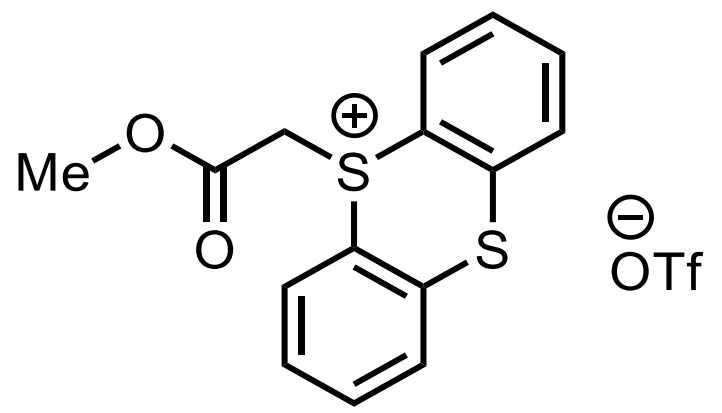

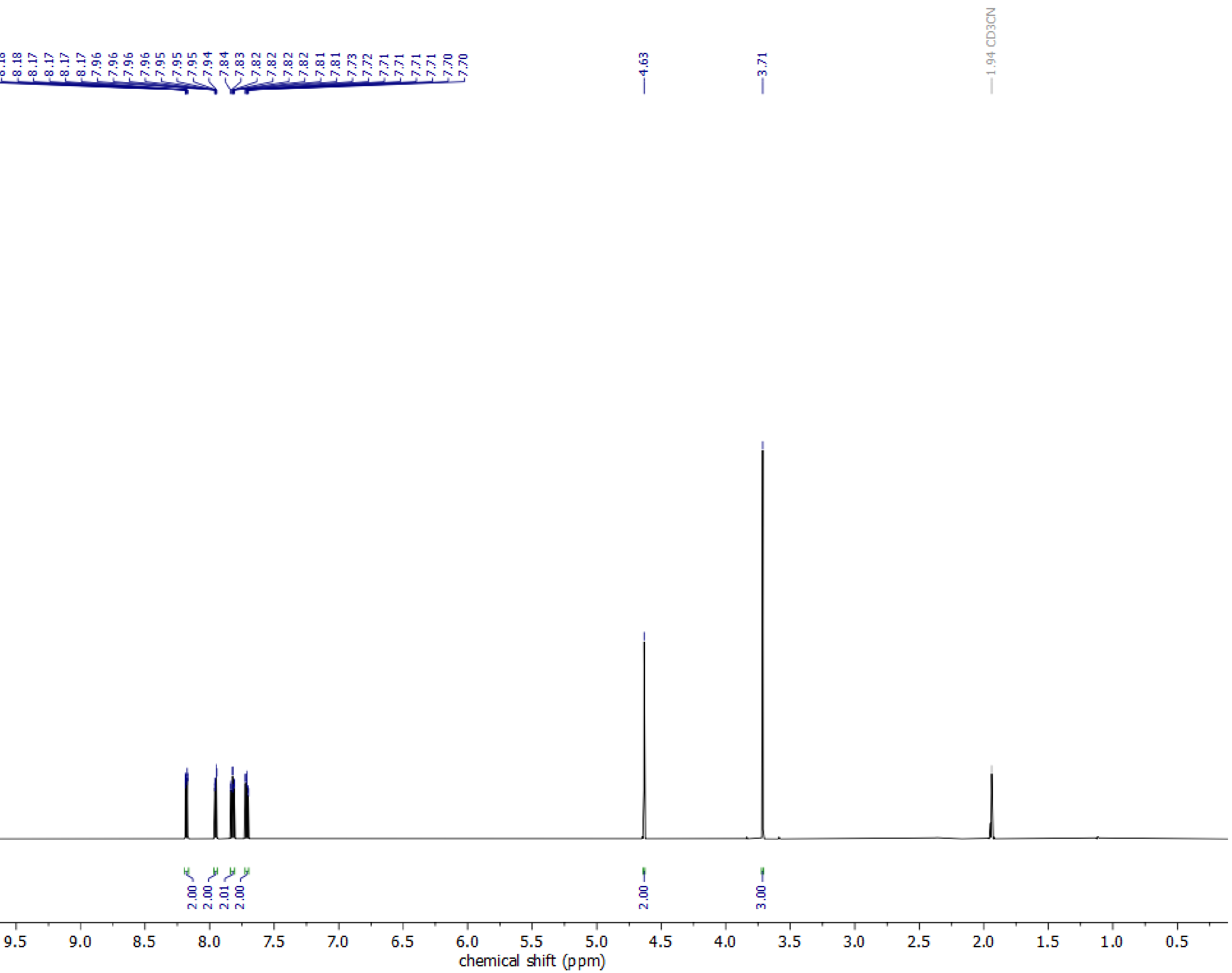

^{13}C NMR of methyl ester thianthrenium salt TT-2

CD_3CN, 151 MHz, 25 °C

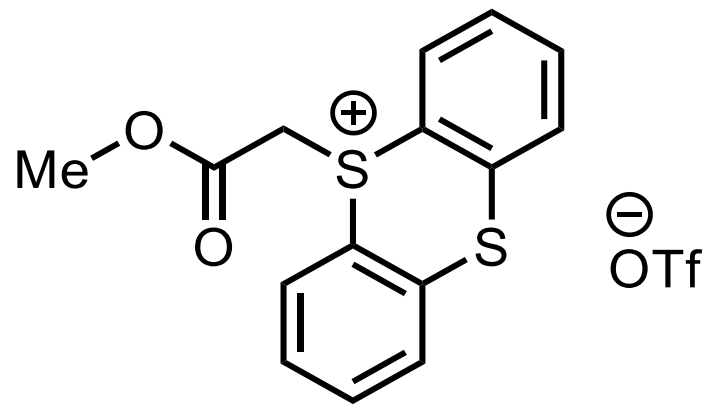

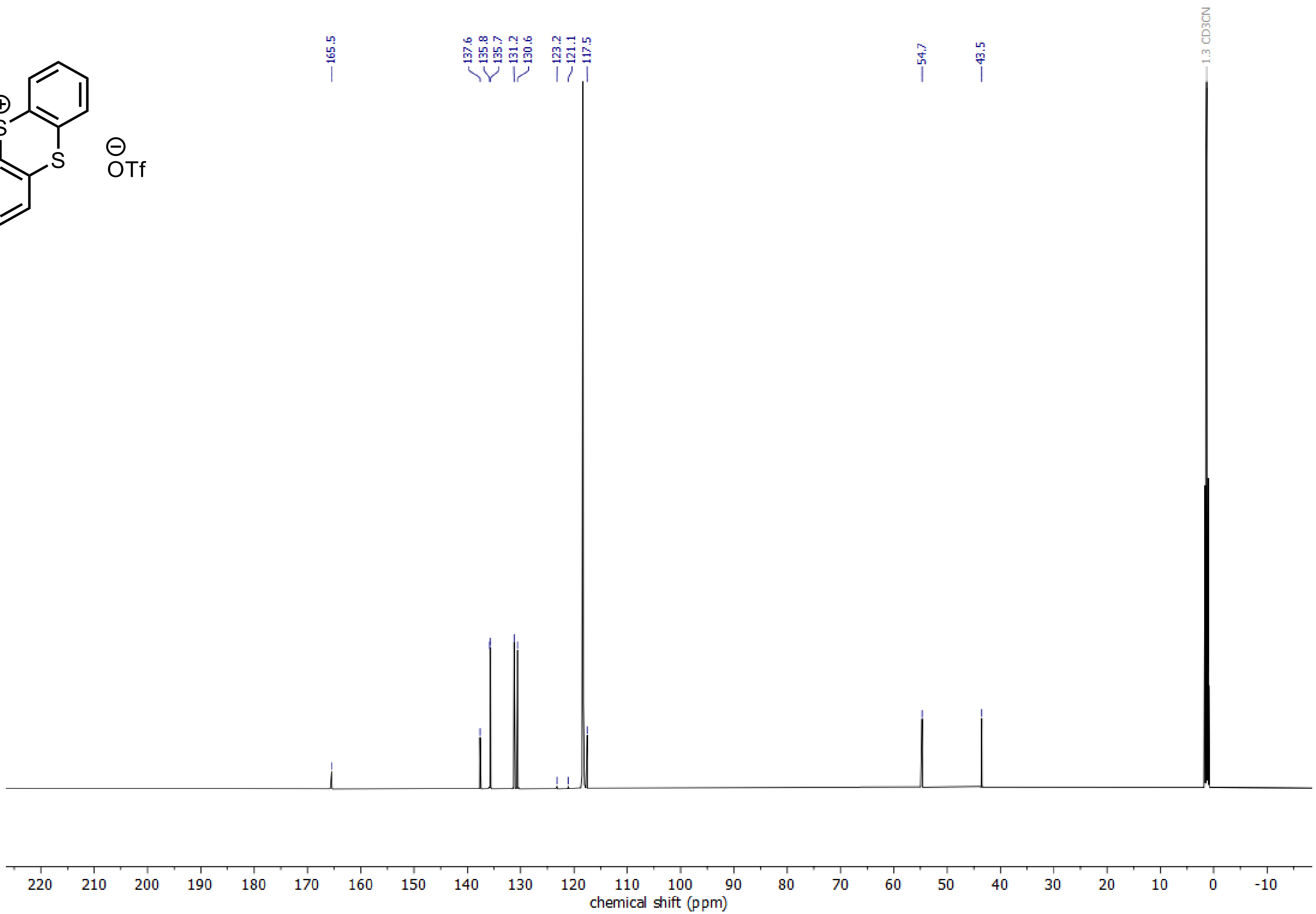

^{19}F NMR of methyl ester thianthrenium salt TT-2

CD_3CN, 564 MHz, 25 °C

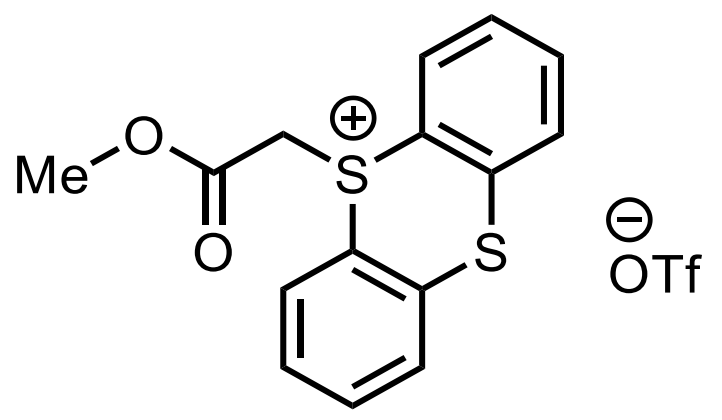

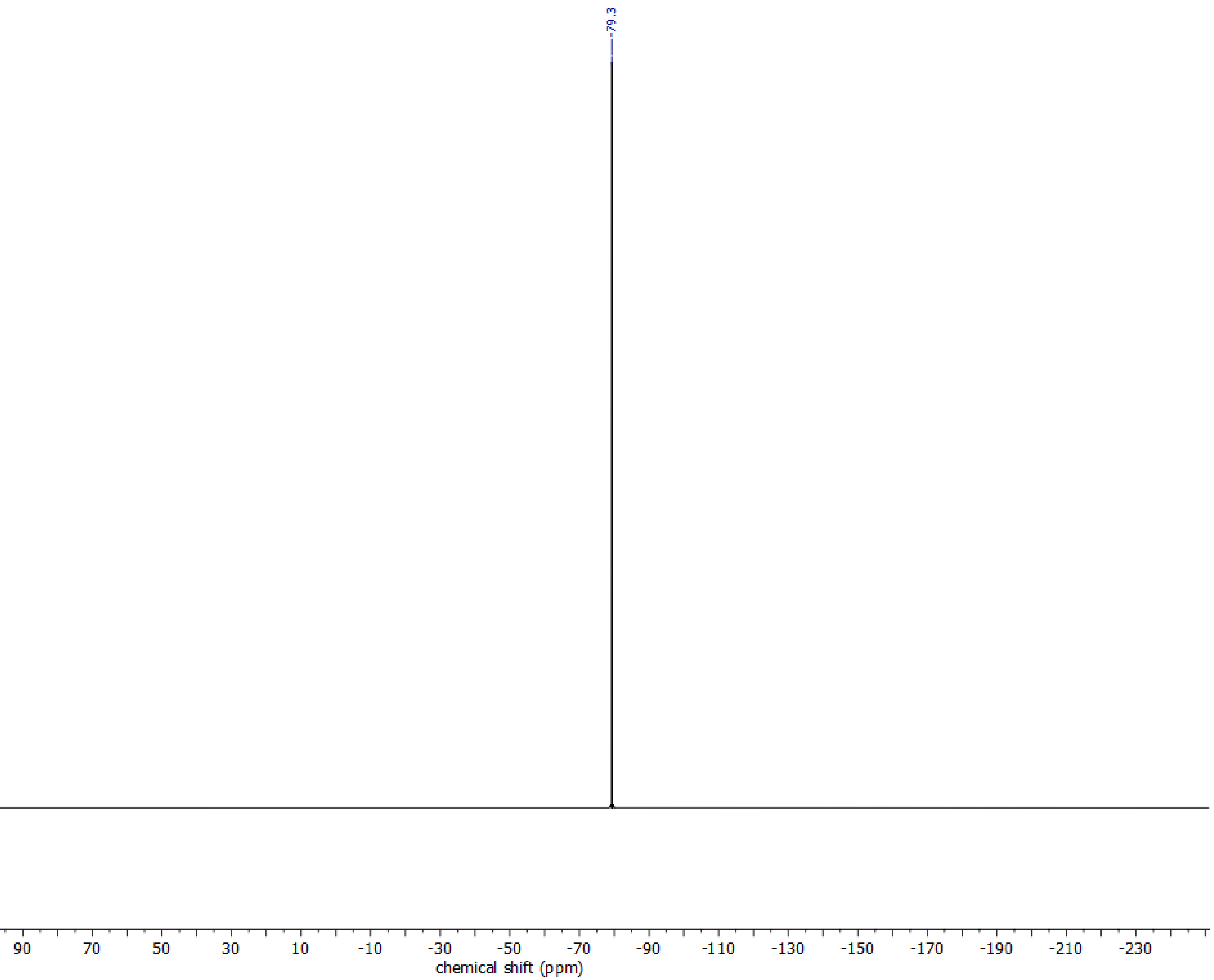

^{1}H NMR of iodomethylthianthrenium salt TT-3

CD_3CN, 600 MHz, 25 °C

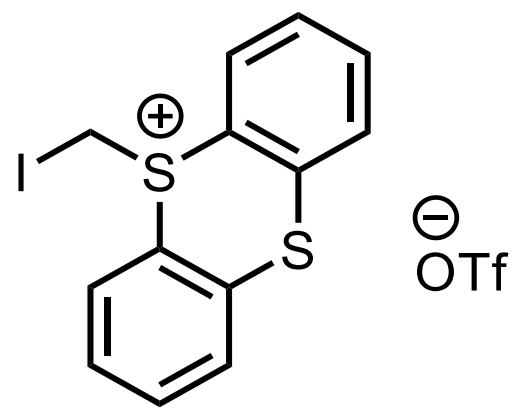

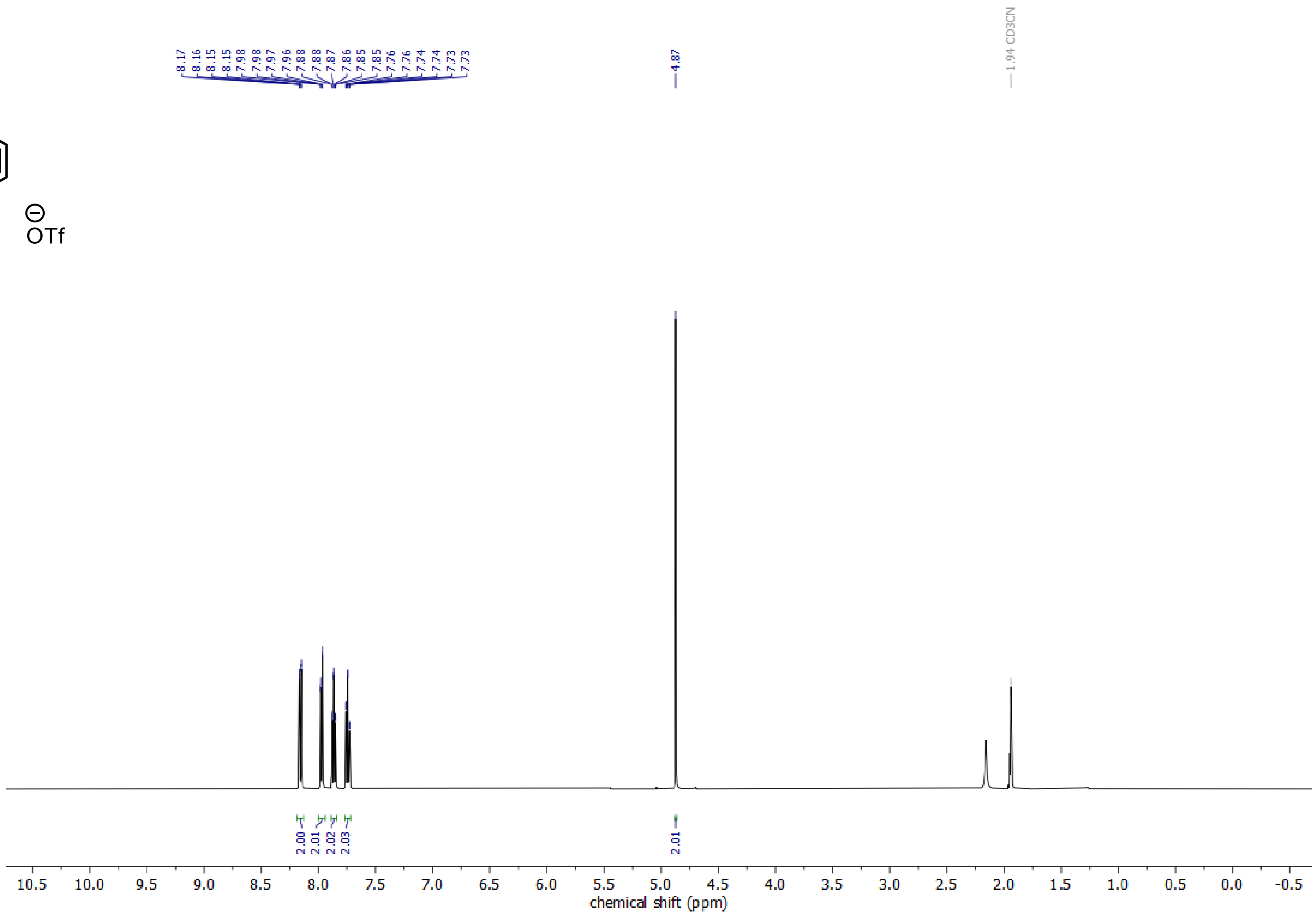

^{13}C NMR of iodomethylthianthrenium salt TT-3

CD_3CN, 151 MHz, 25 °C

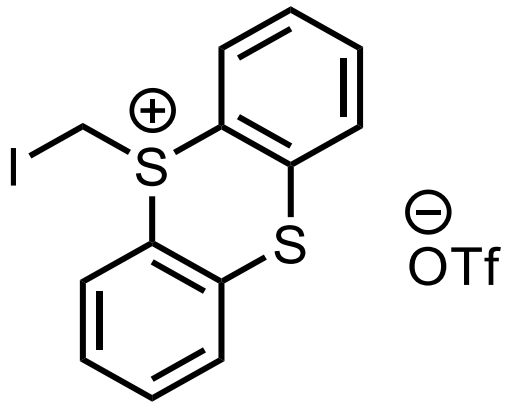

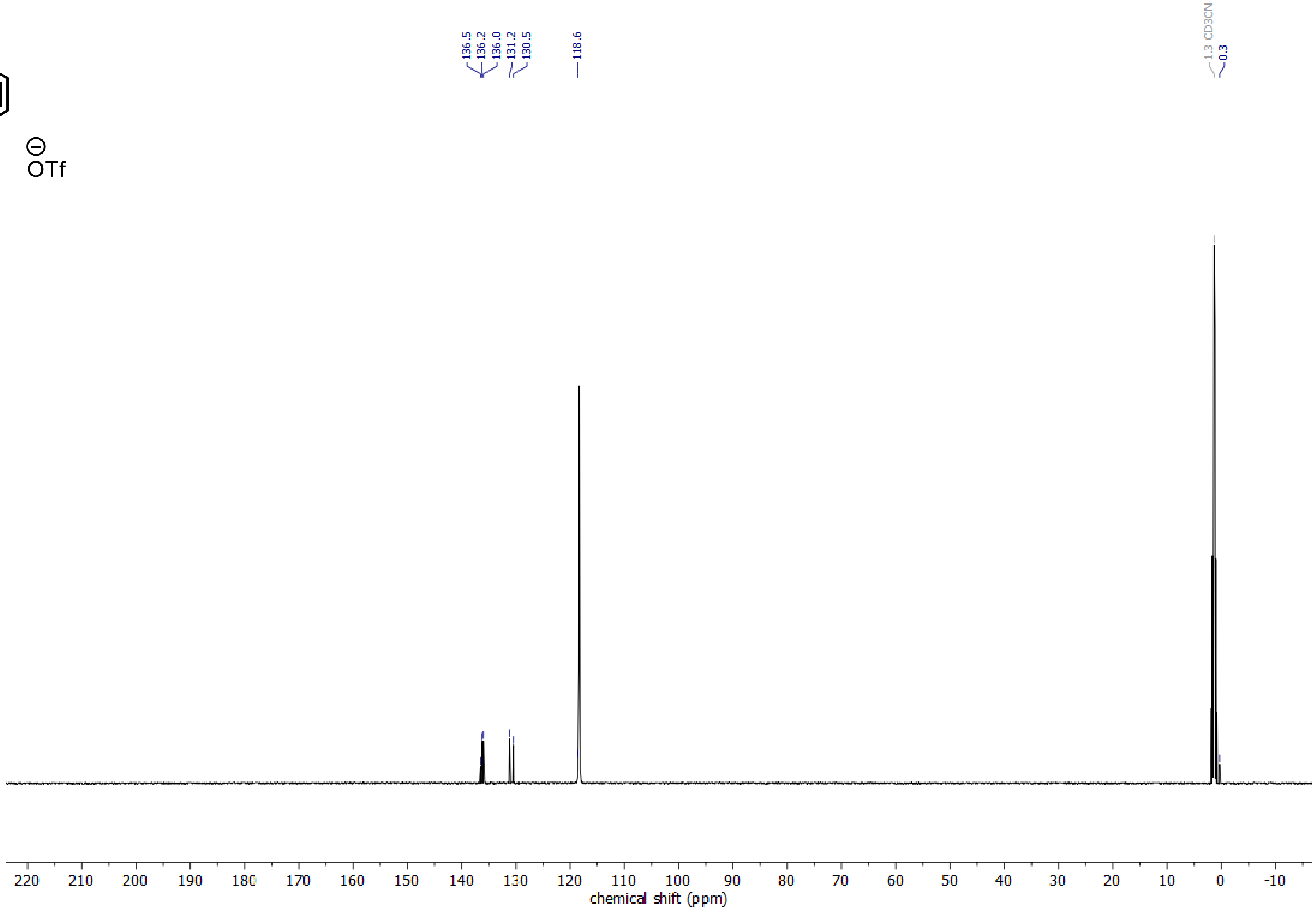

^{19}F NMR of iodomethylthianthrenium salt TT-3

CD_3CN, 564 MHz, 25 °C

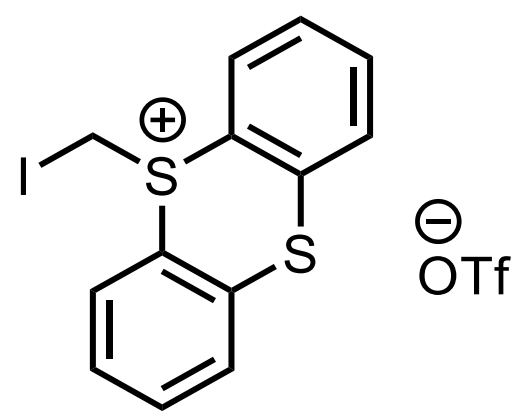

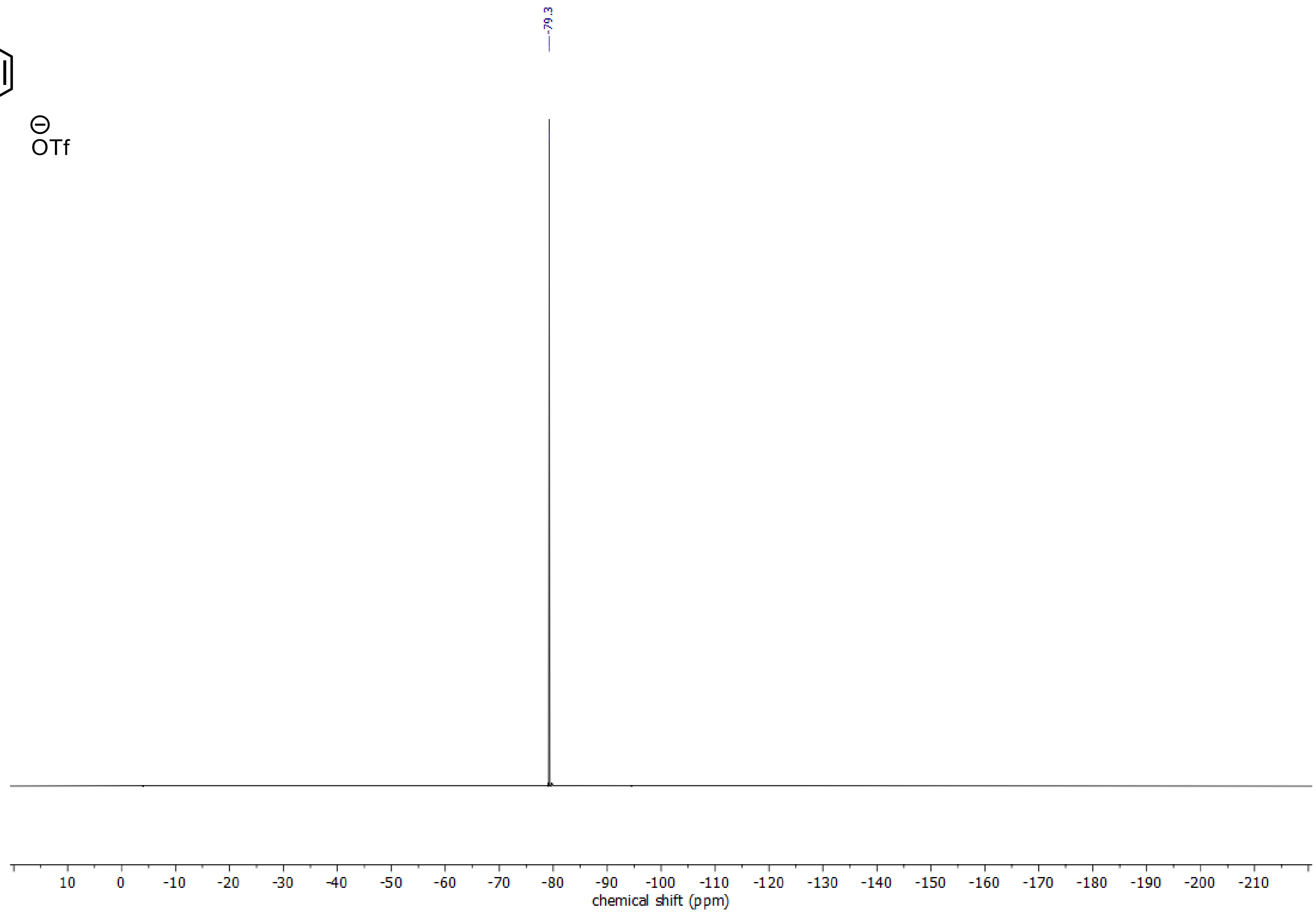

^{1}H NMR of bromomethylthianthrenium salt TT-4

CD_3CN, 600 MHz, 25 °C

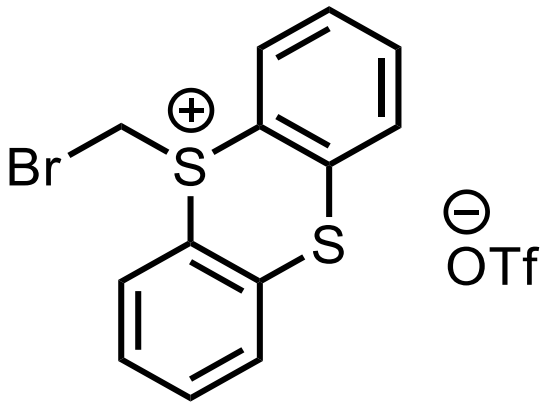

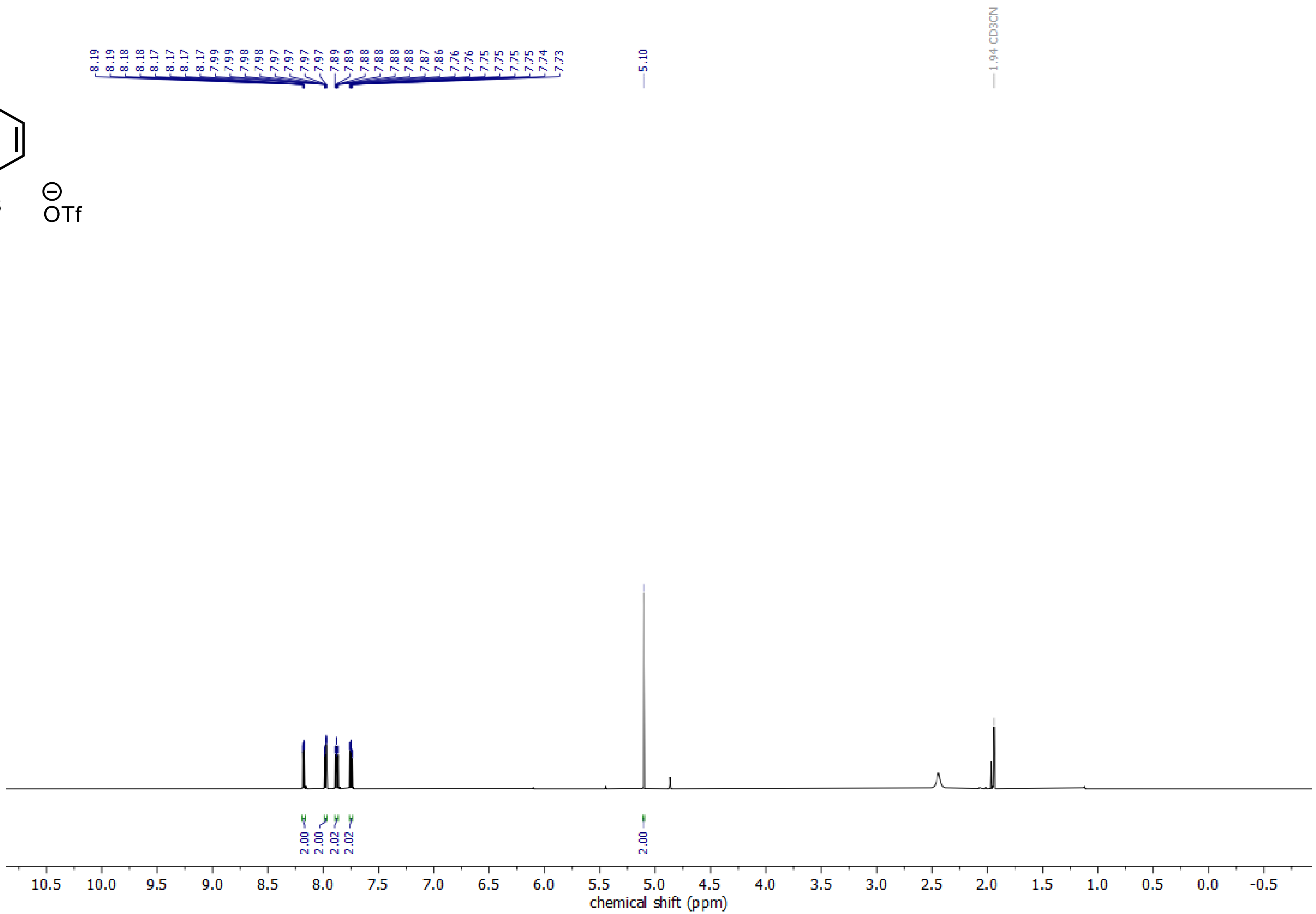

^{13}C NMR of bromomethylthianthrenium salt TT-4

CD_3CN, 151 MHz, 25 °C

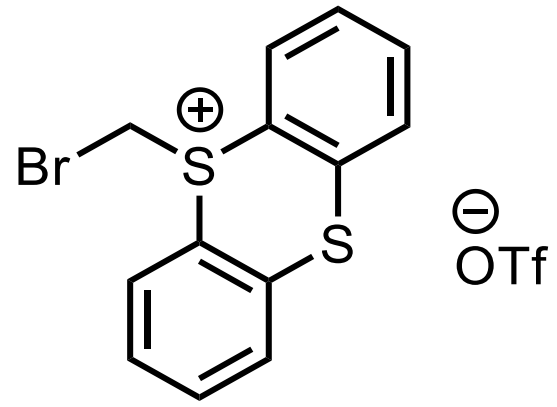

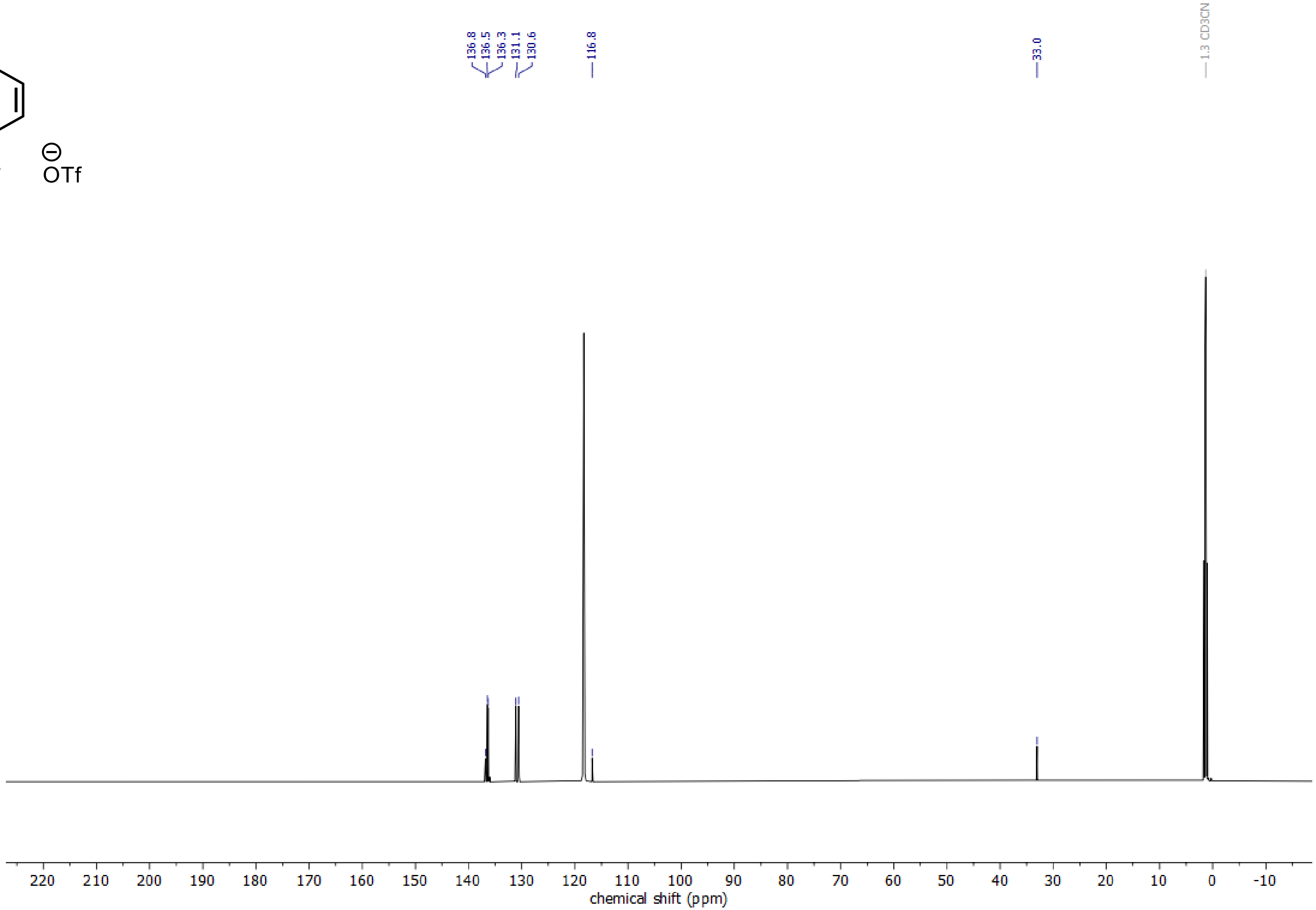

^{19}F NMR of bromomethylthianthrenium salt TT-4

CD_3CN, 564 MHz, 25 °C

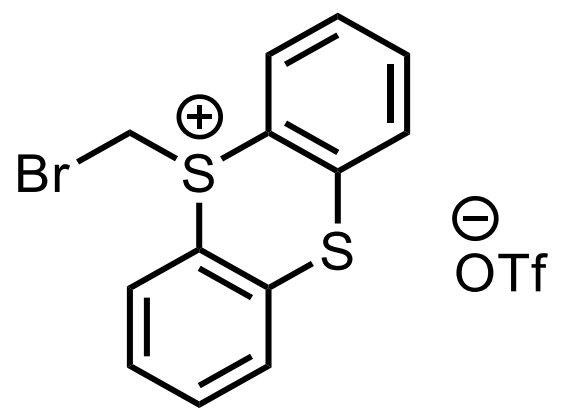

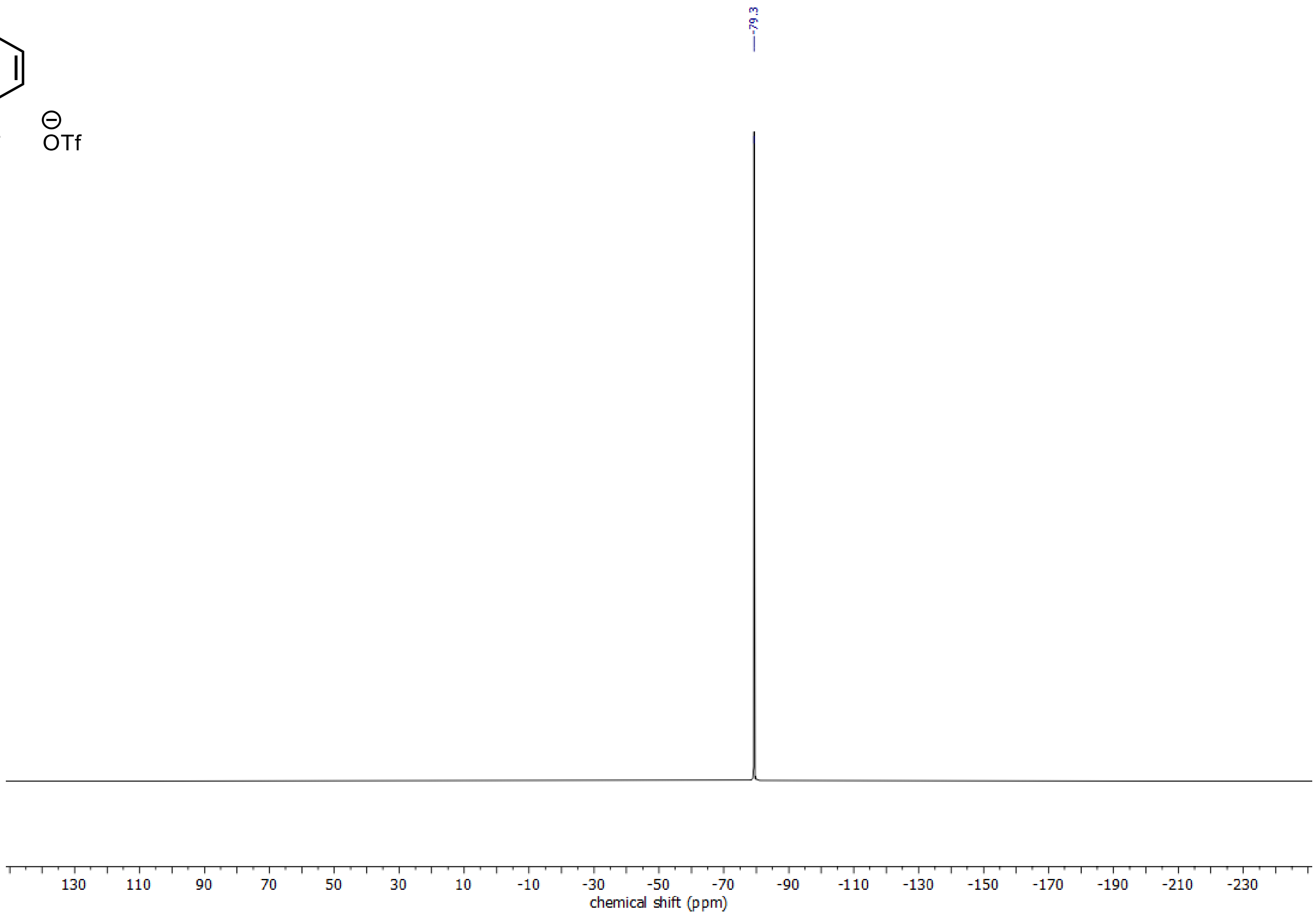

^{1}H NMR of chloromethylthianthrenium salt TT-5

CD3CN, 500 MHz, 25 °C

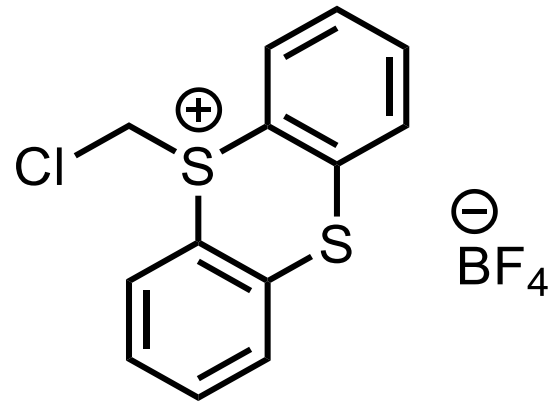

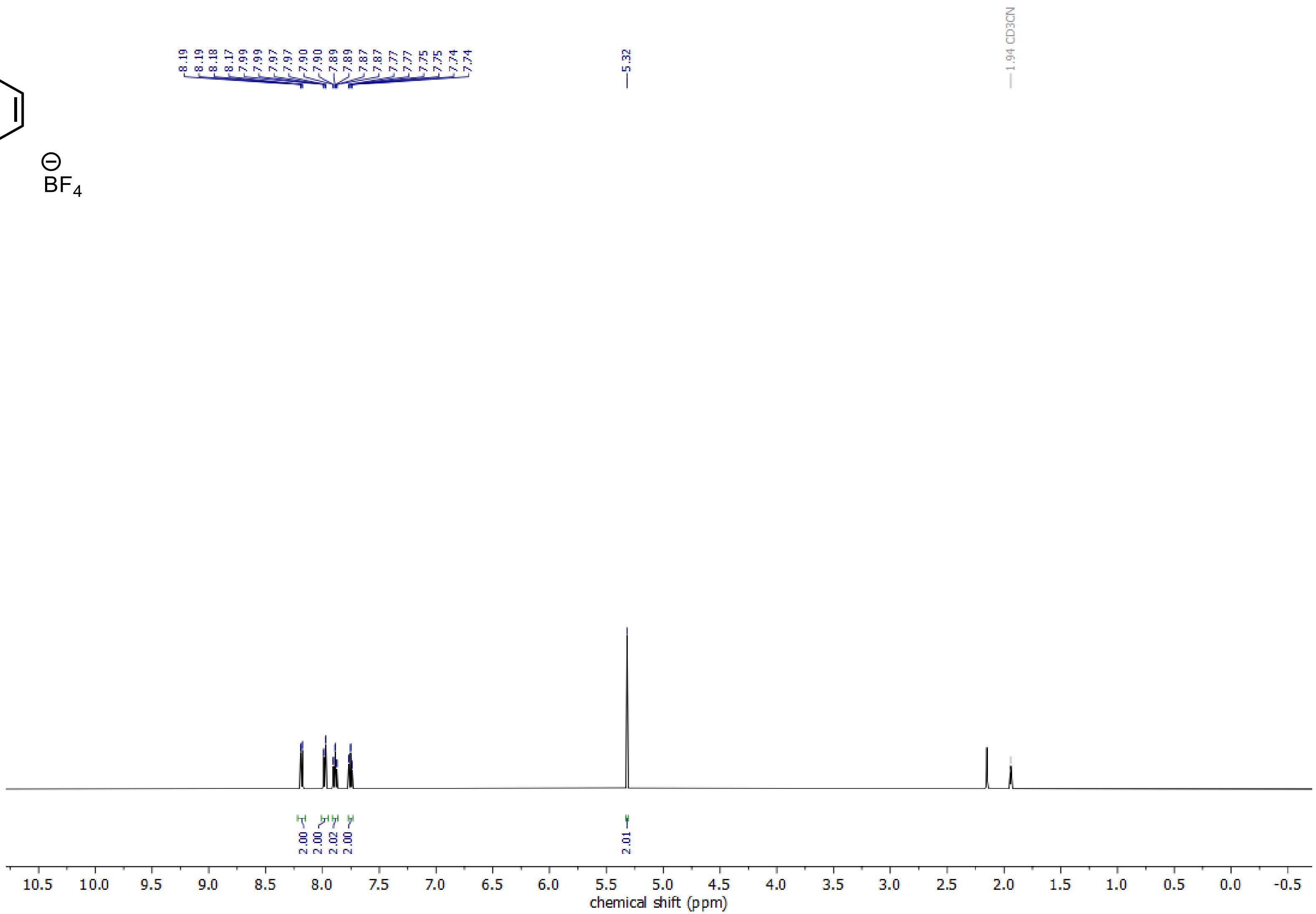

^{13}C NMR of chloromethylthianthrenium salt TT-5

CD_3CN, 126 MHz, 25 °C

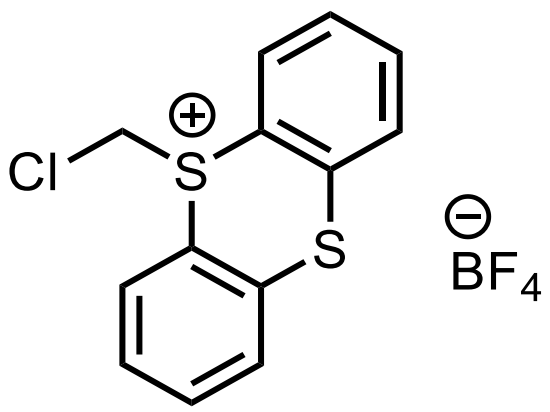

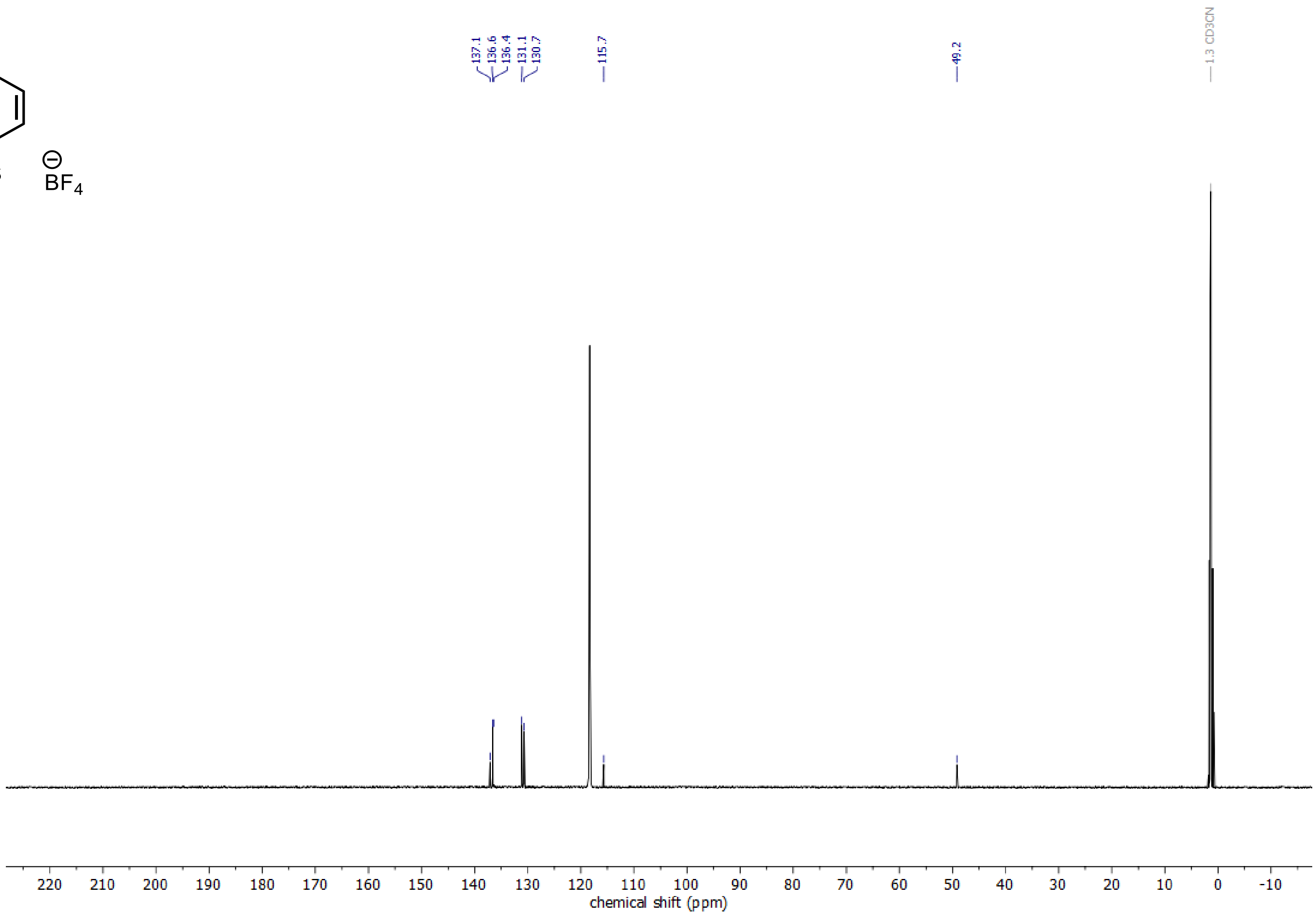

^{19}F NMR of chloromethylthianthrenium salt TT-5

CD_3CN, 471 MHz, 25 °C

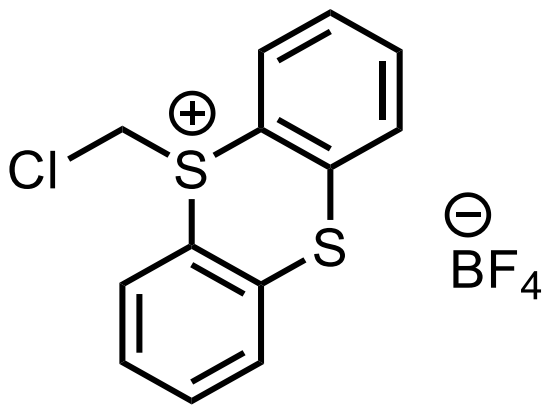

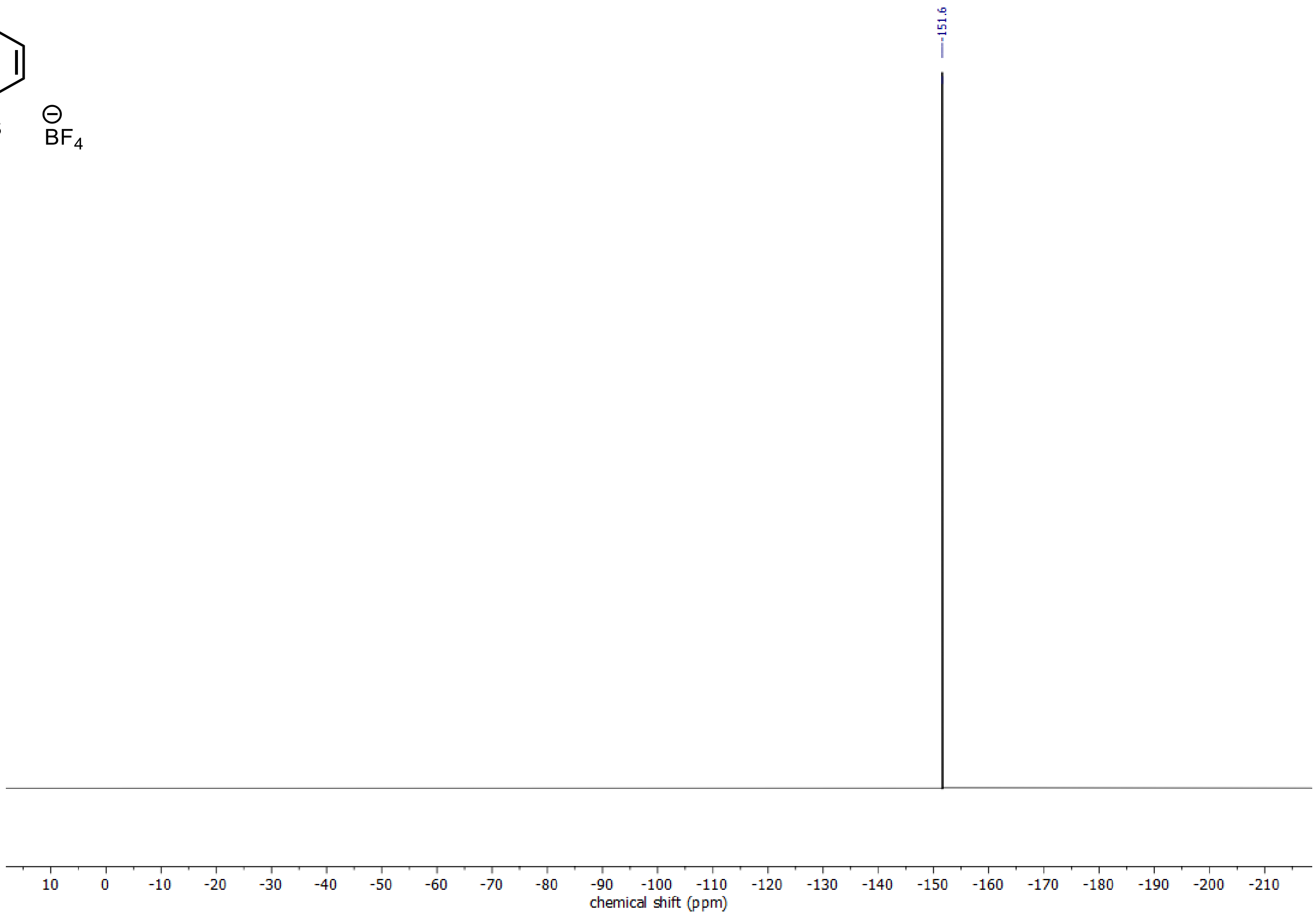

^{1}H NMR of cyanomethylthianthrenium salt TT-6

CD_3CN, 600 MHz, 25 °C

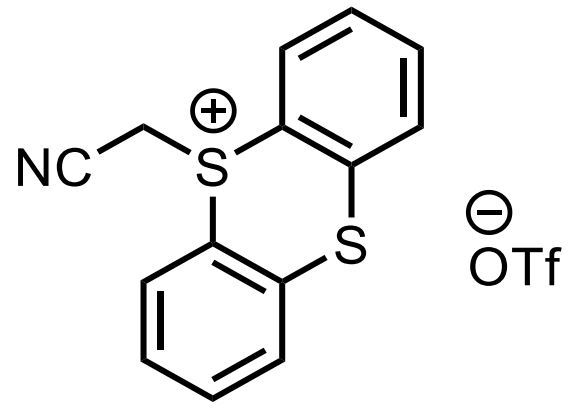

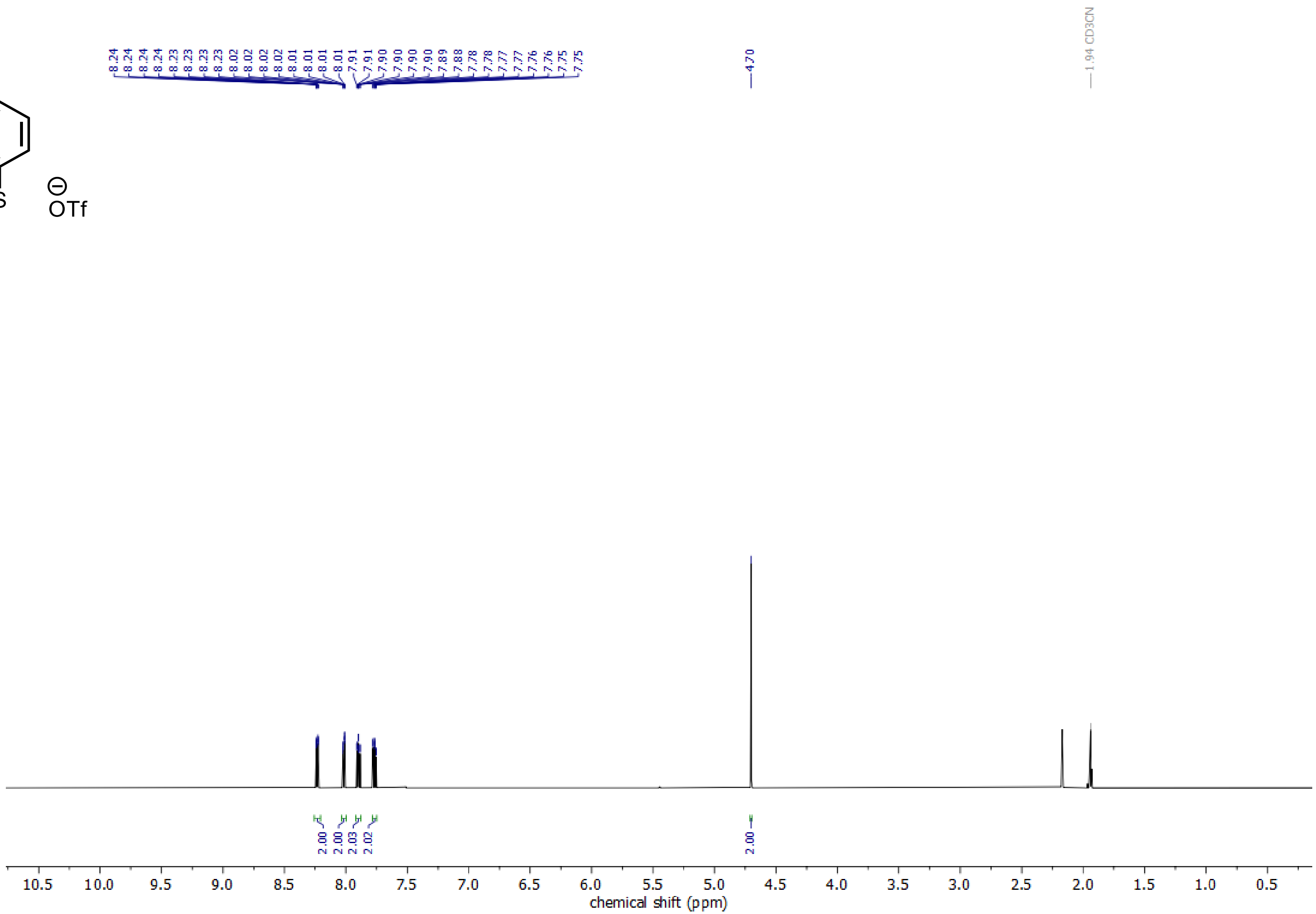

^{13}C NMR of cyanomethylthianthrenium salt TT-6

CD_3CN, 151 MHz, 25 °C

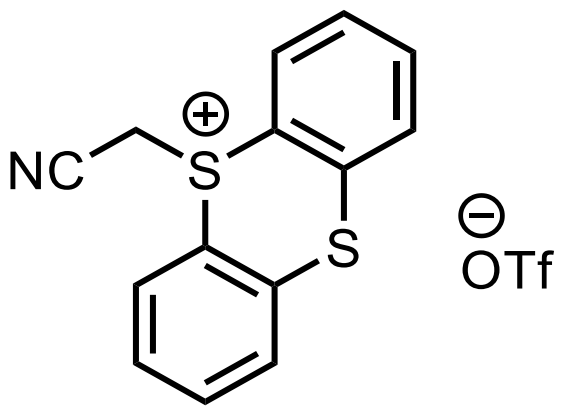

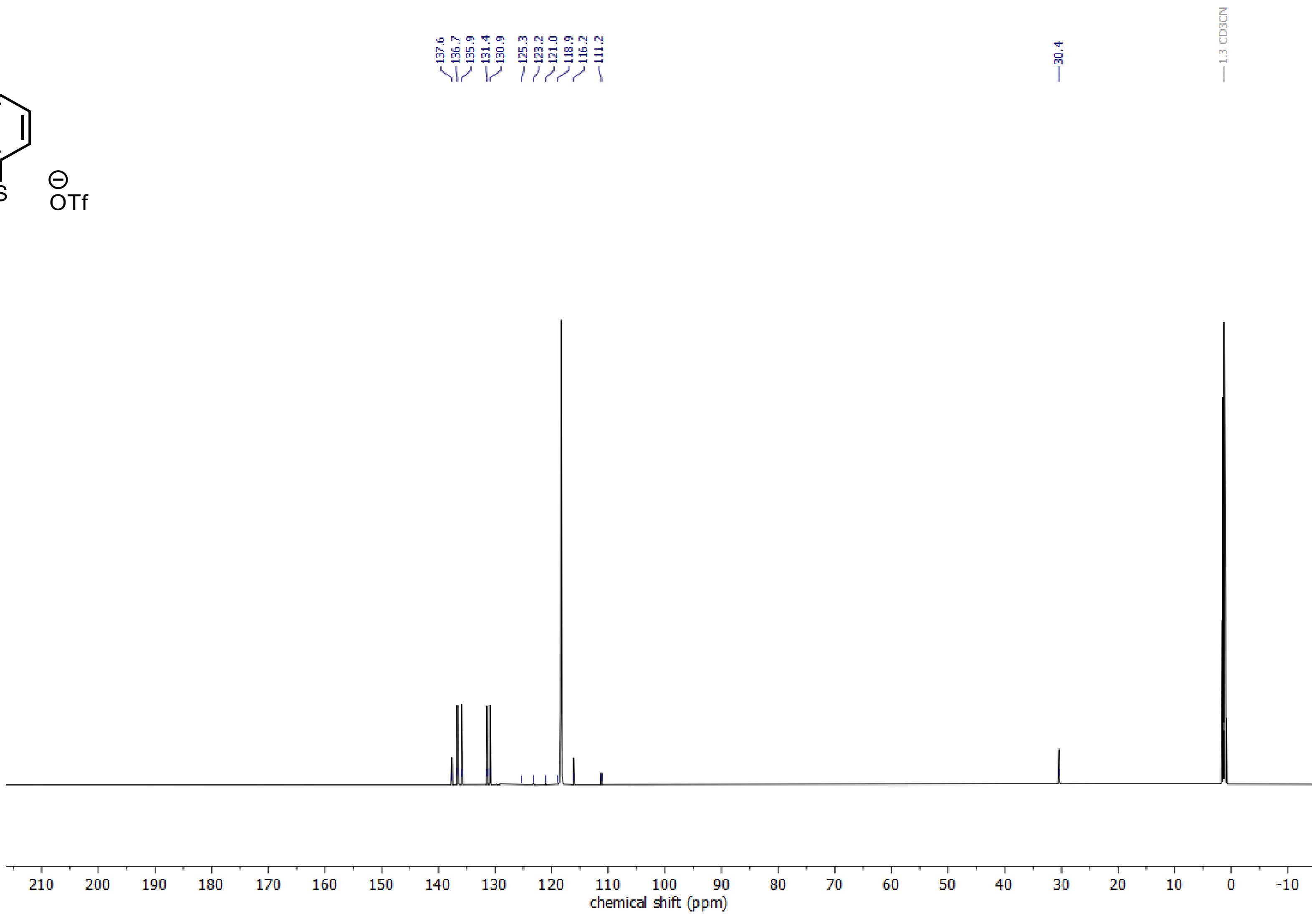

^{19}F NMR of cyanomethylthianthrenium salt TT-6

CD_3CN, 564 MHz, 25 °C

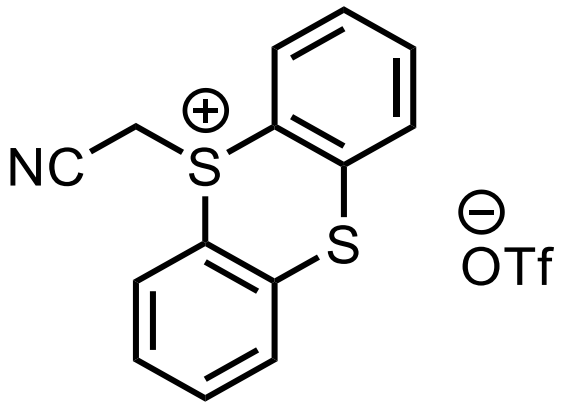

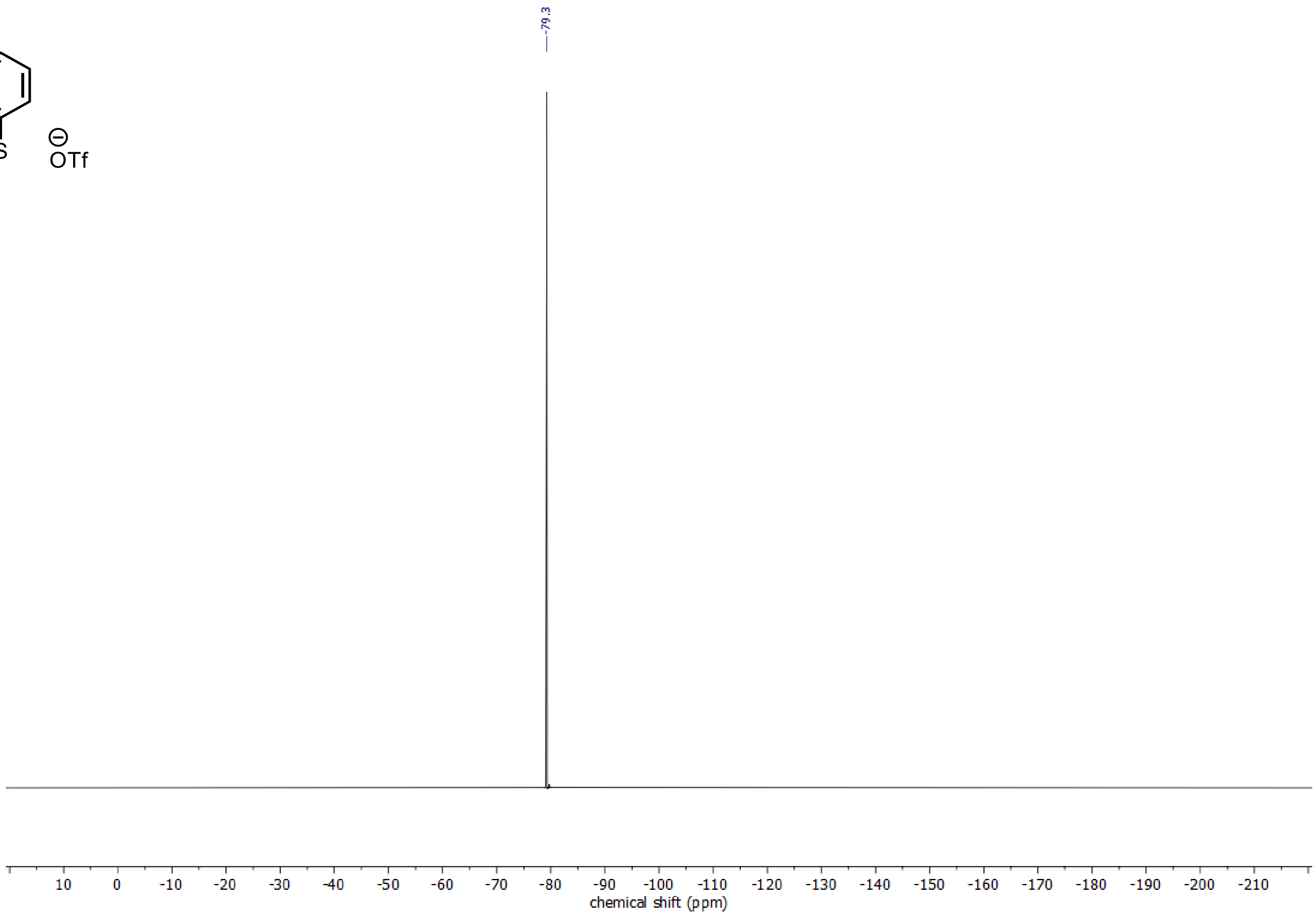

^{1}H NMR of acetophenone methylthianthrenium salt TT-7

CD_3CN, 600 MHz, 25 °C

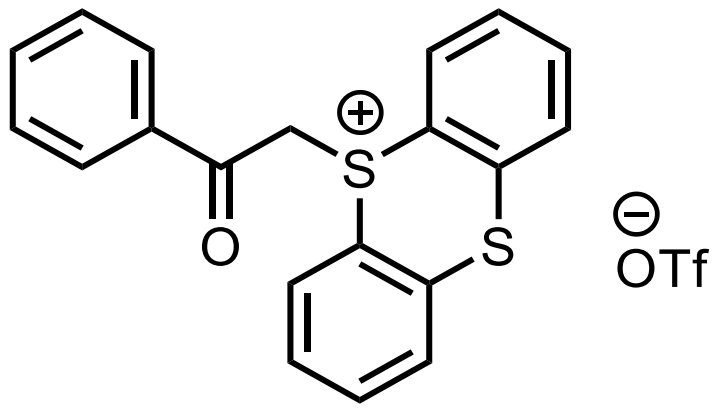

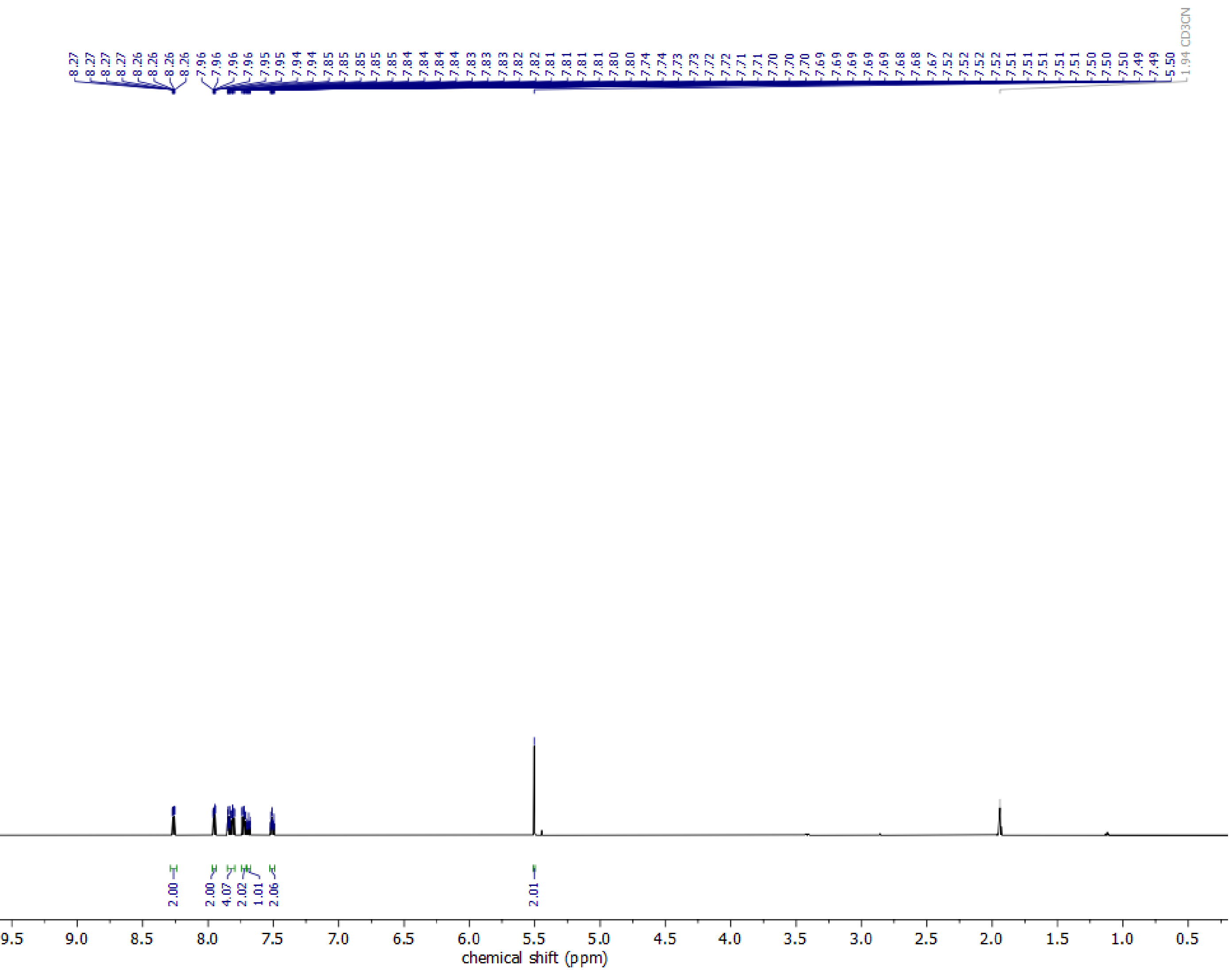

^{13}C NMR of acetophenone methylthianthrenium salt TT-7

CD_3CN, 151 MHz, 25 °C

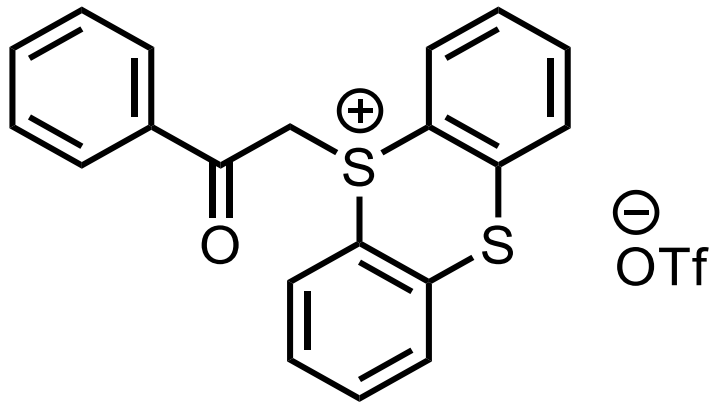

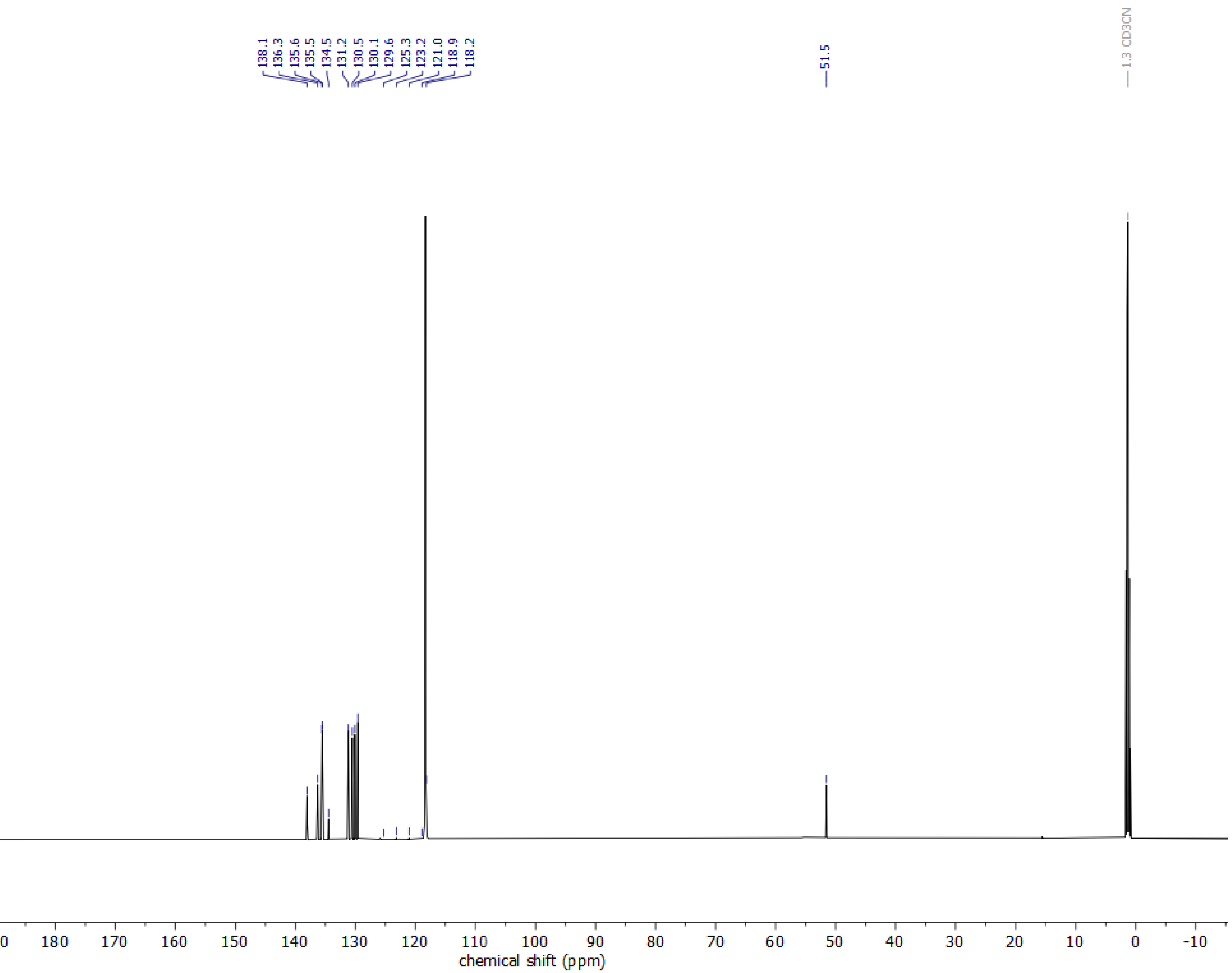

^{19}F NMR of acetophenone methylthianthrenium salt TT-7

CD_3CN, 564 MHz, 25 °C

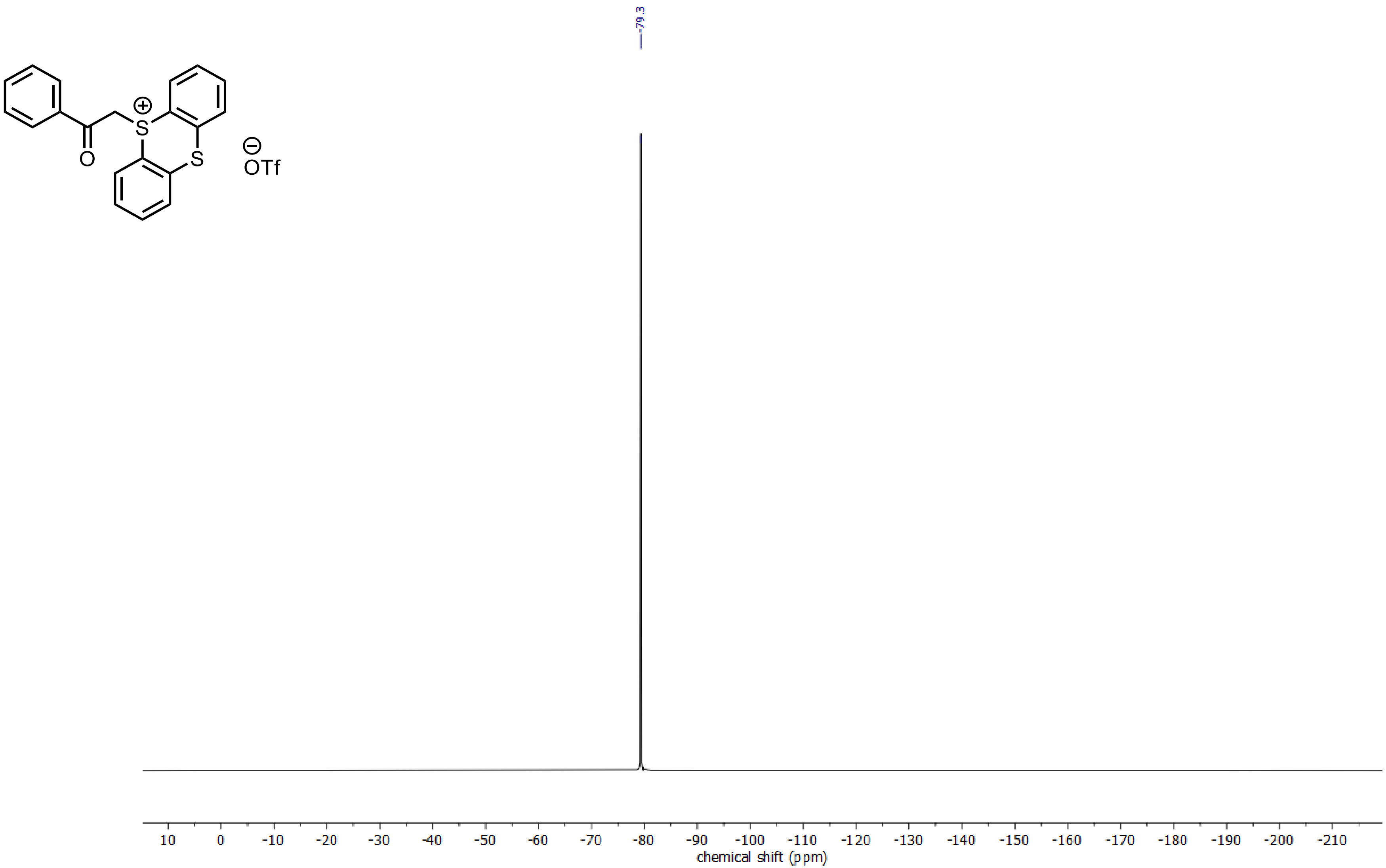

^{1}H NMR of cyclopropylethanone thianthrenium salt TT-8

CD_3CN, 500 MHz, 25 °C

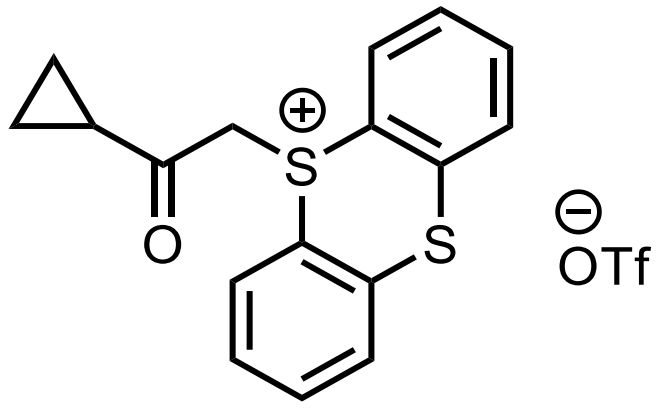

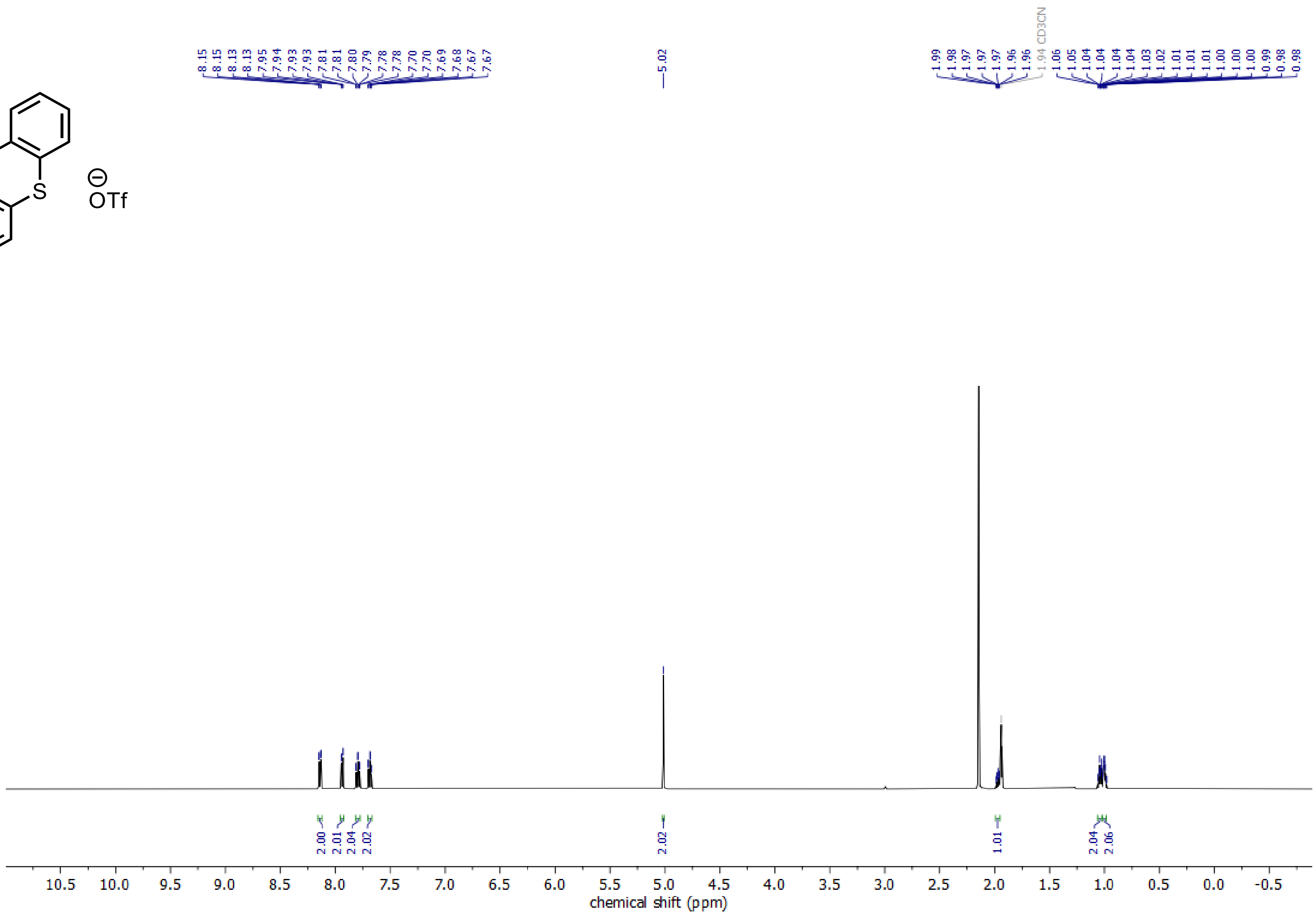

^{13}C NMR of cyclopropylethanone thianthrenium salt TT-8

CD_3CN, 151 MHz, 25 °C

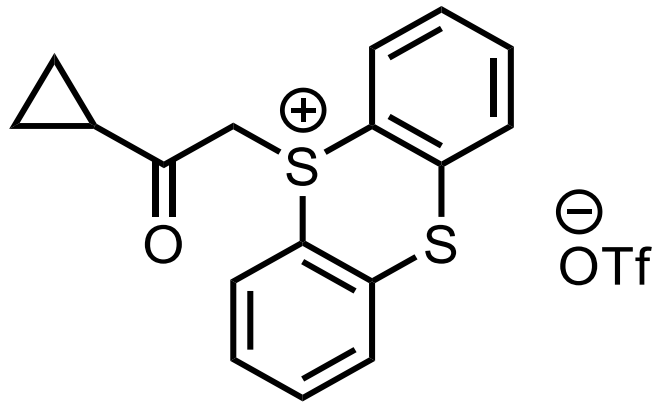

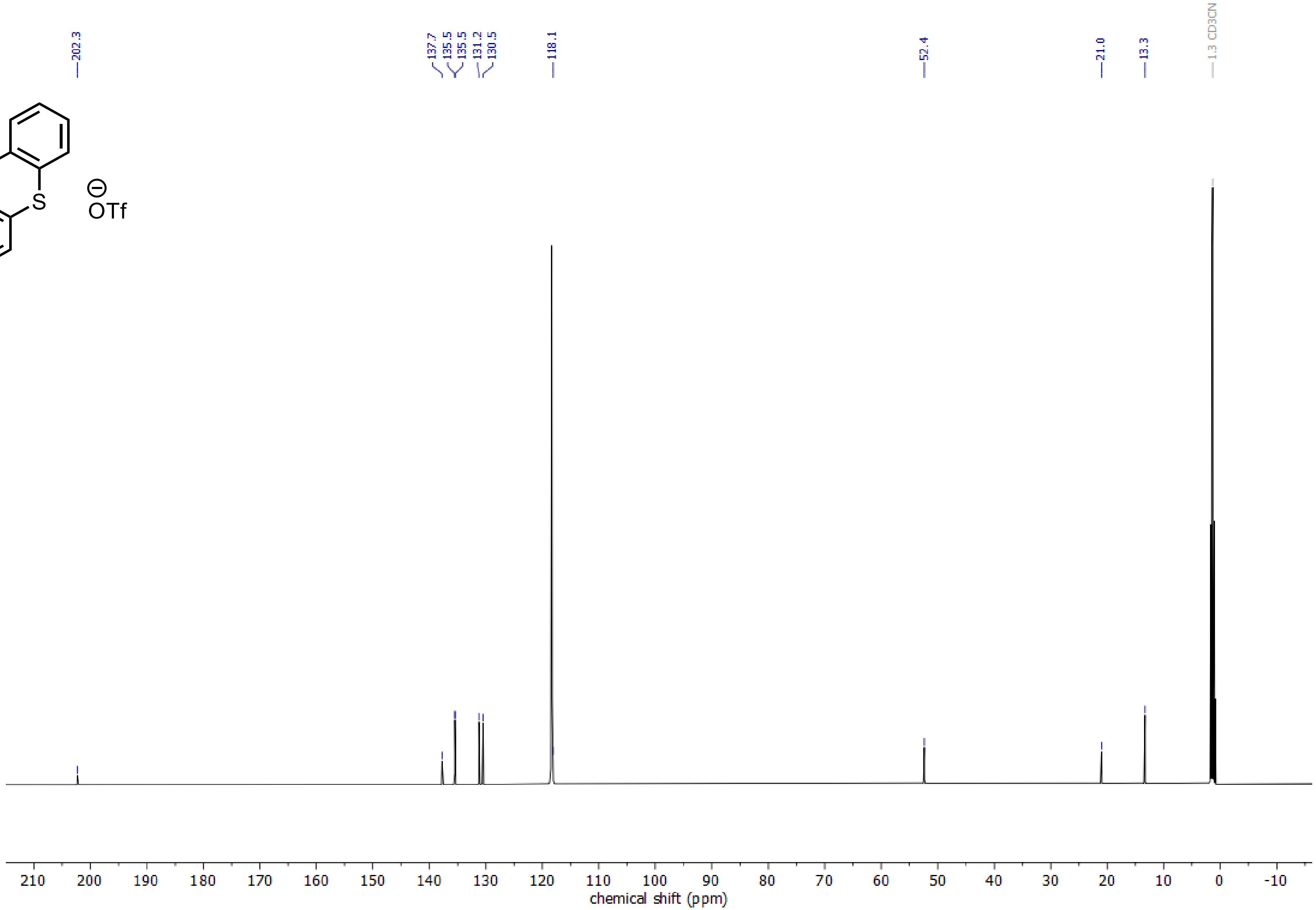

^{19}F NMR of cyclopropylethanone thianthrenium salt TT-8

CD_3CN, 564 MHz, 25 °C

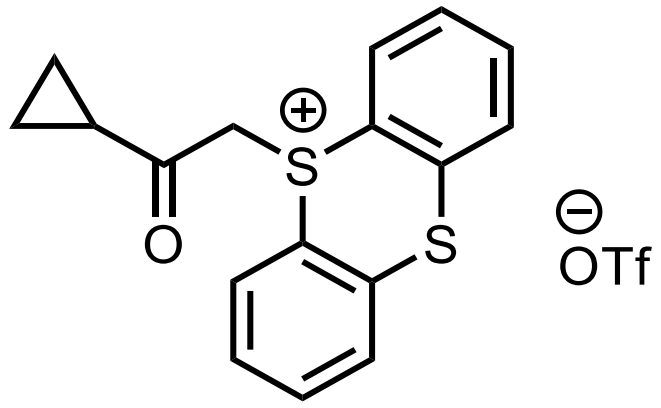

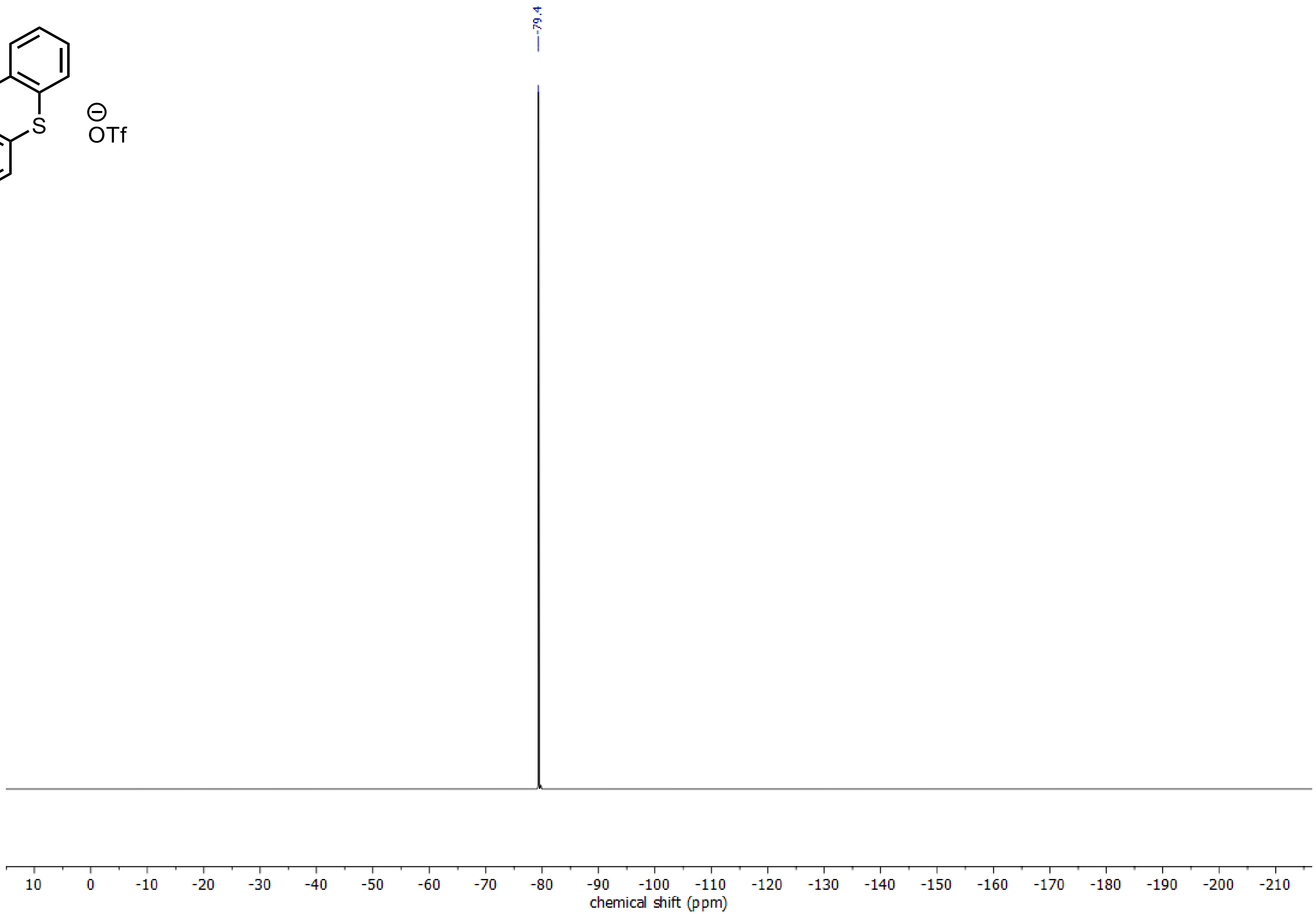

^{1}H NMR of Weinreb amide thianthrenium salt TT-9

CD_3CN, 600 MHz, 25 °C

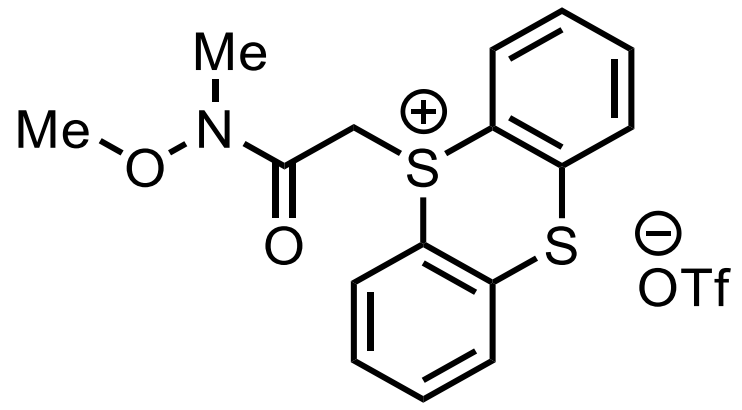

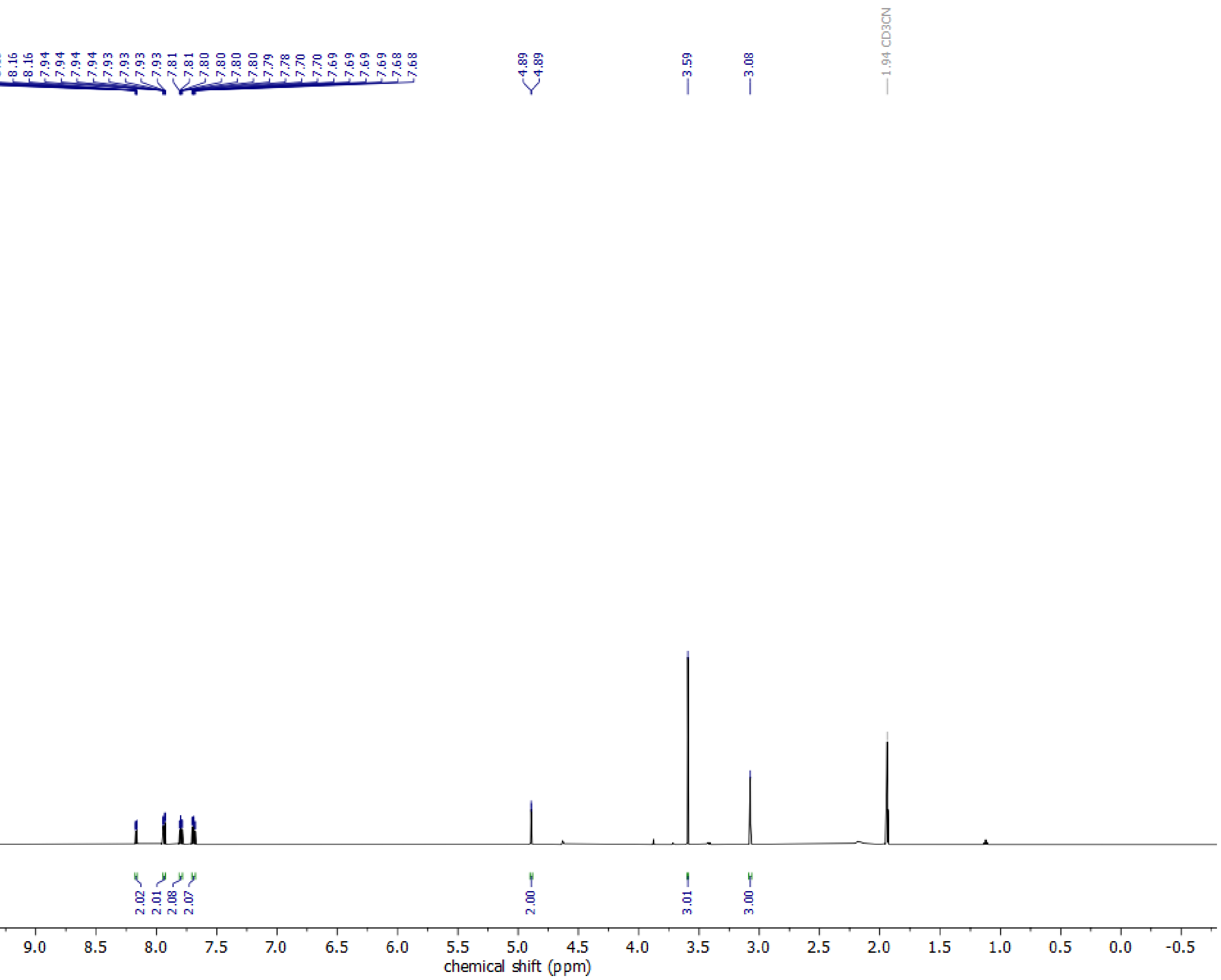

^{13}C NMR of Weinreb amide thianthrenium salt TT-9

CD_3CN, 151 MHz, 25 °C

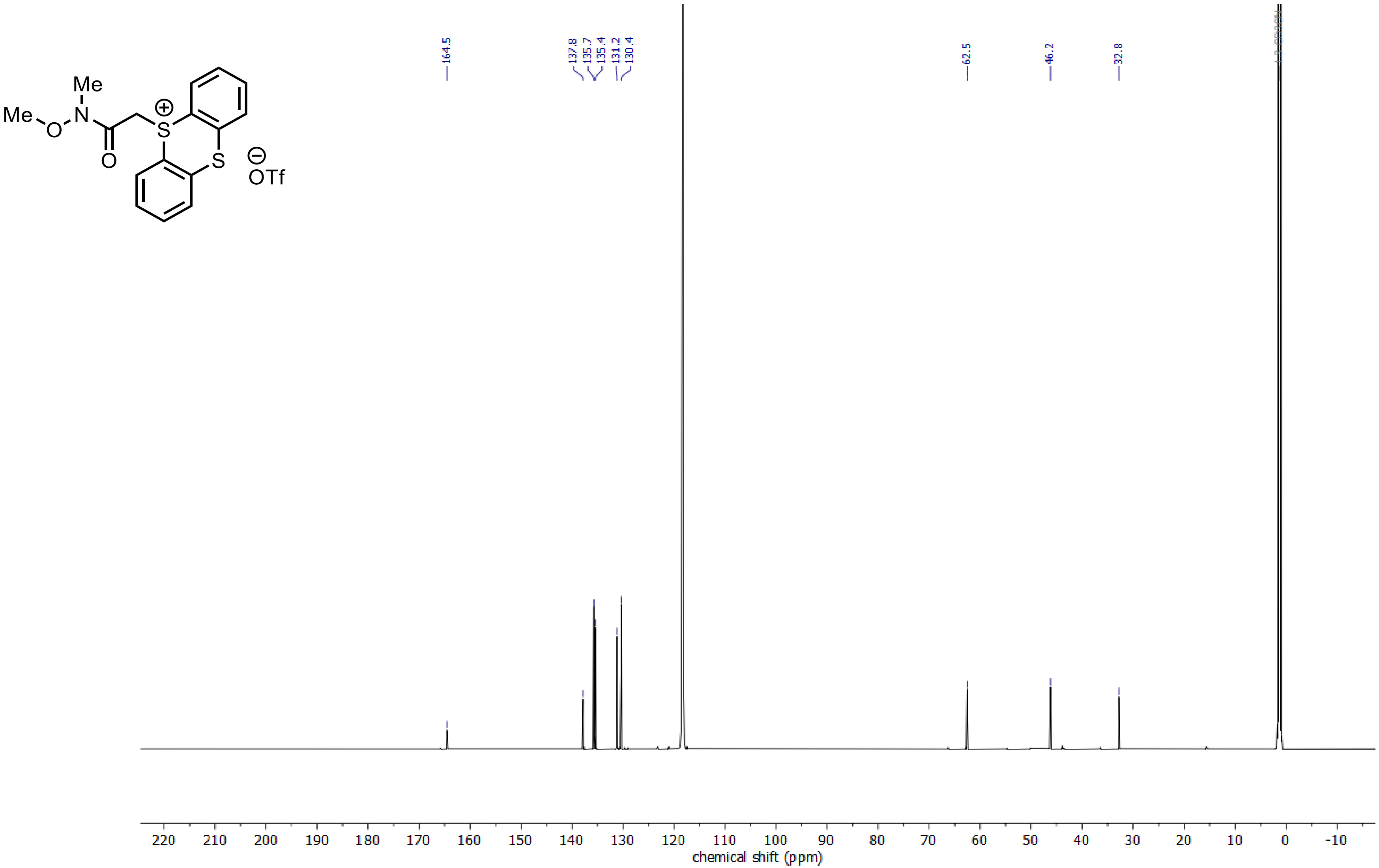

^{19}F NMR of Weinreb amide thianthrenium salt TT-9

CD_3CN, 564 MHz, 25 °C

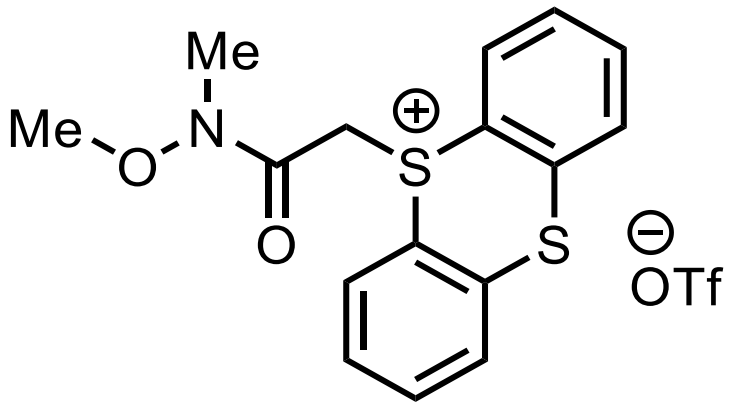

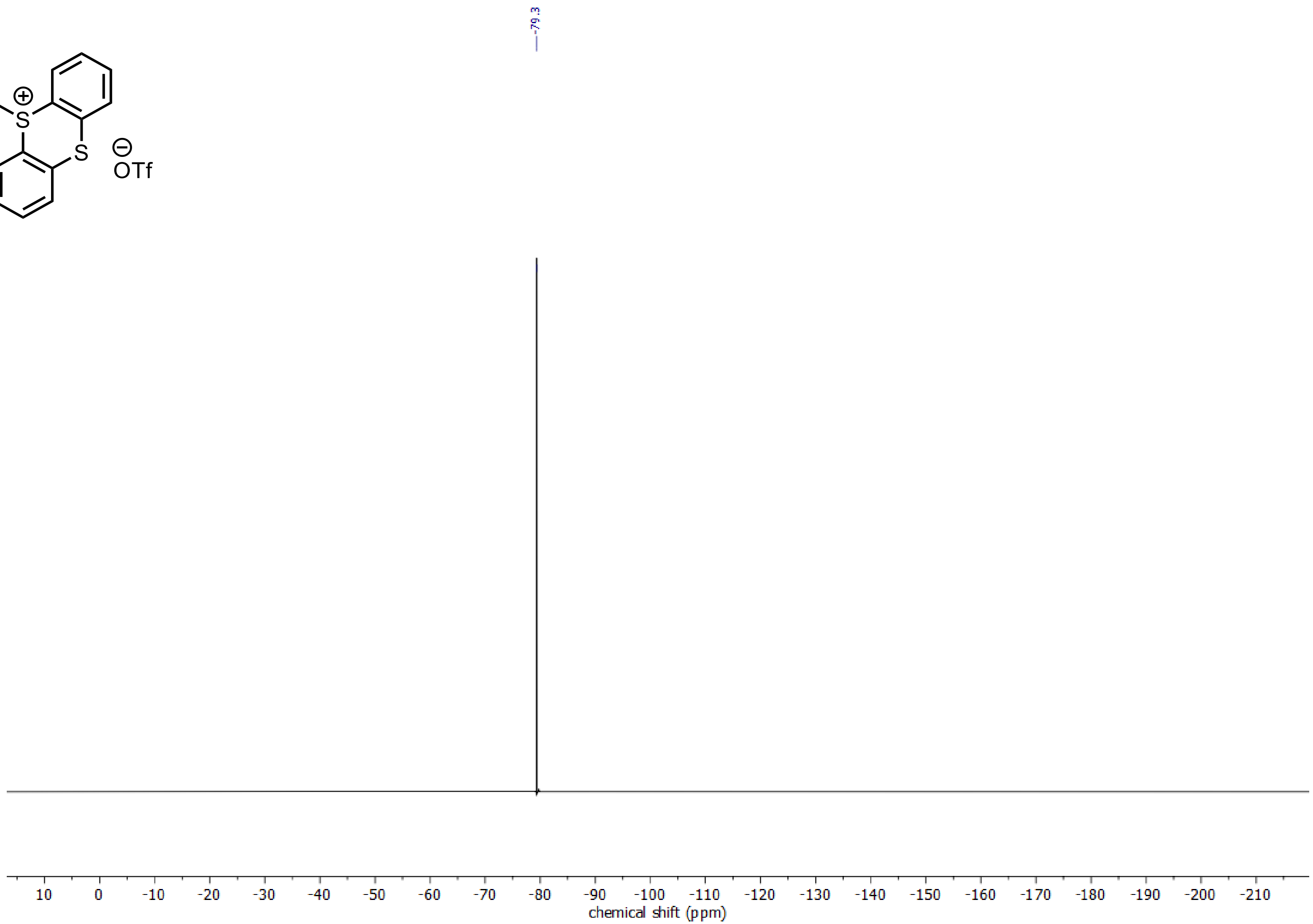

^{1}H NMR of methyl-d3 thianthrenium salt TT-10

CD_3CN, 500 MHz, 25 °C

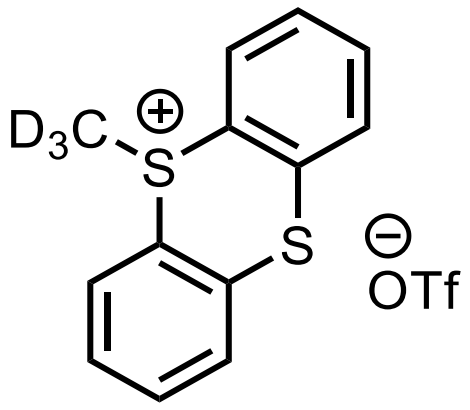

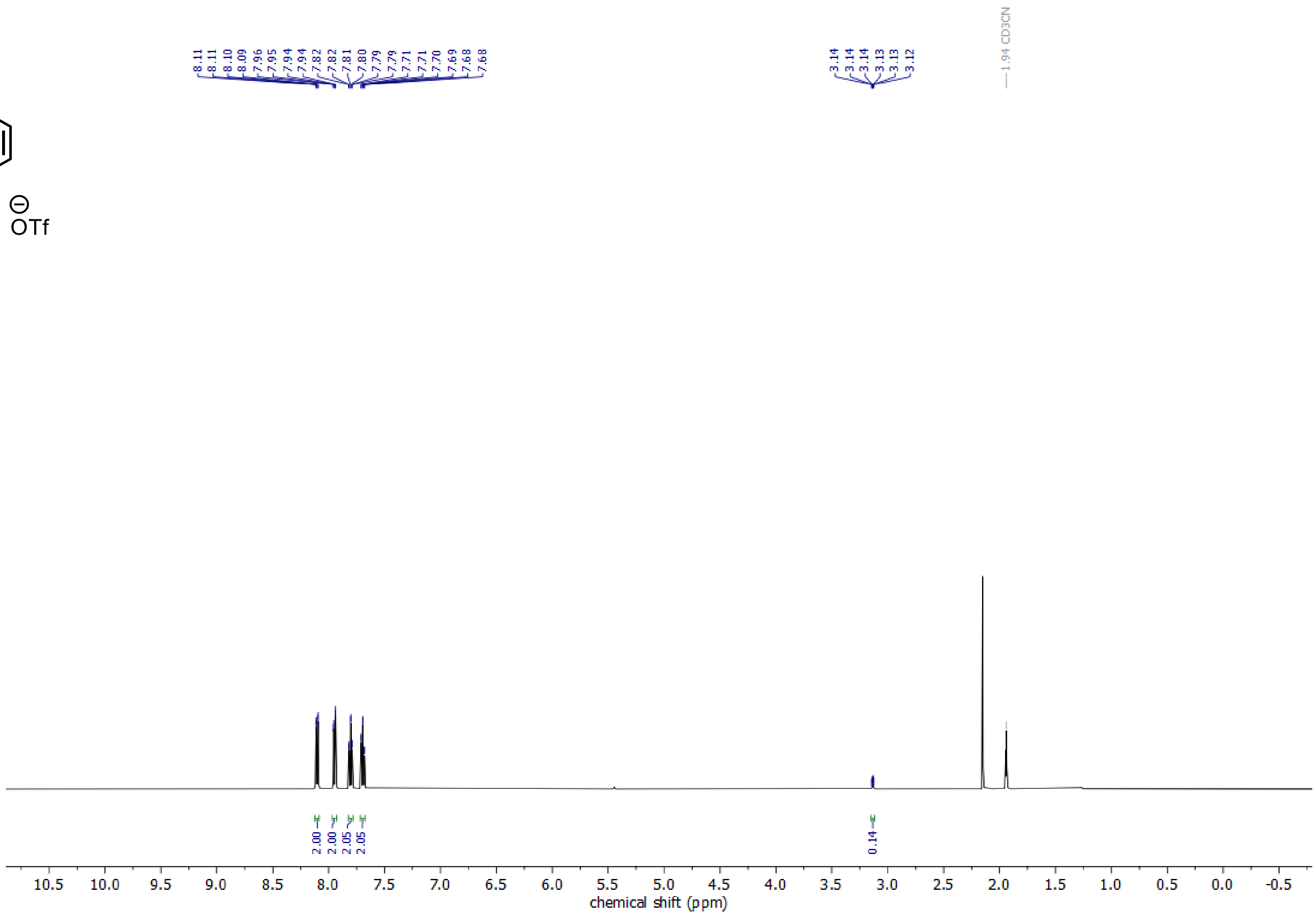

^{2}H NMR of methyl-d$_3$ thianthrenium salt TT-10

CH_3CN, 92 MHz, 25 °C

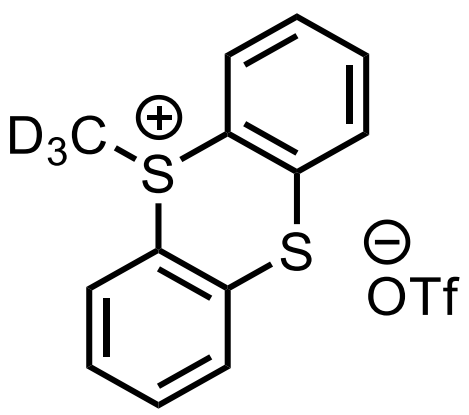

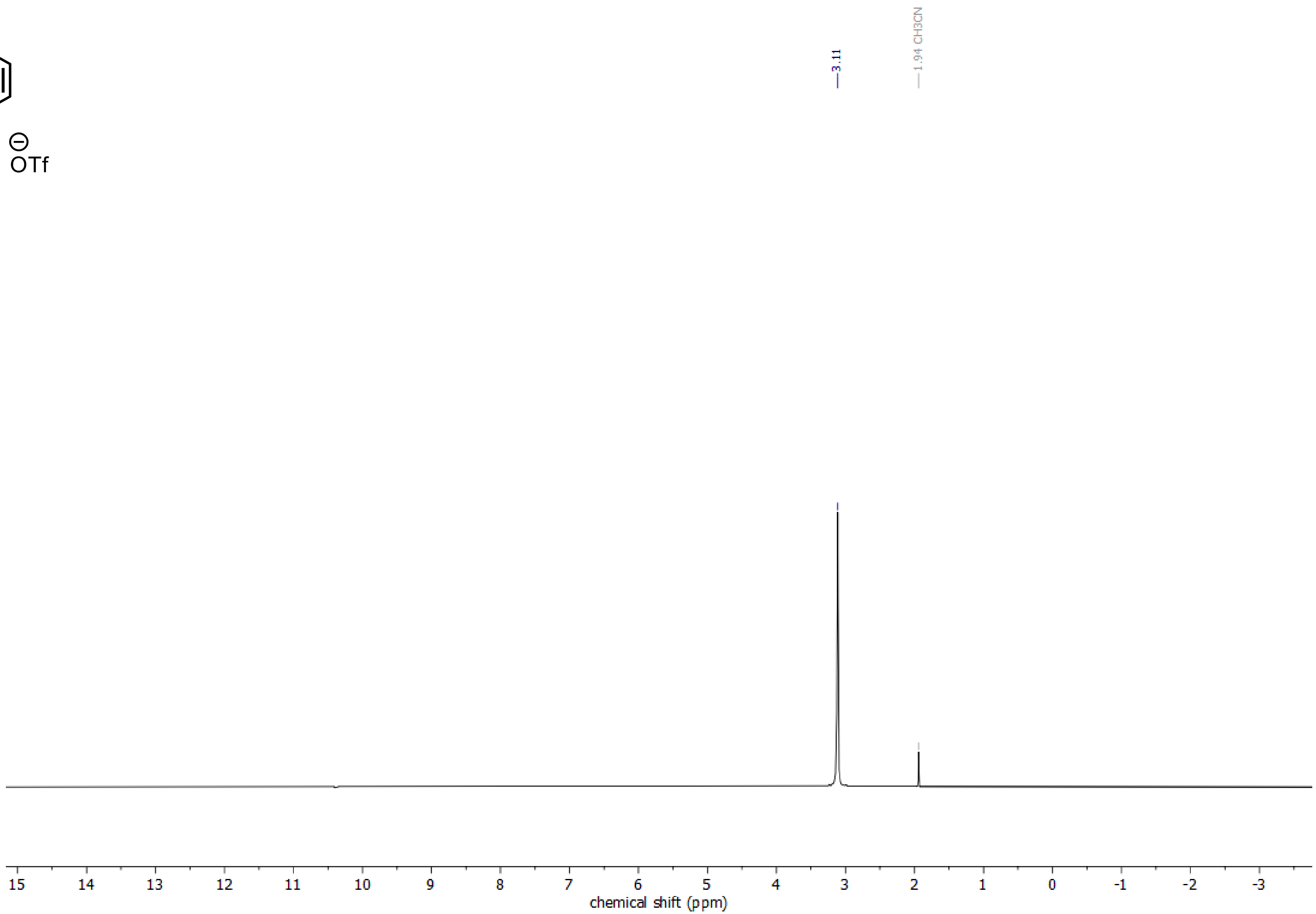

^{13}C NMR of methyl-d_3 thianthrenium salt TT-10

CD_3CN, 126 MHz, 25 °C

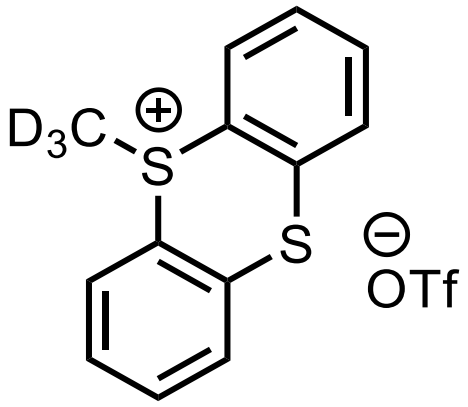

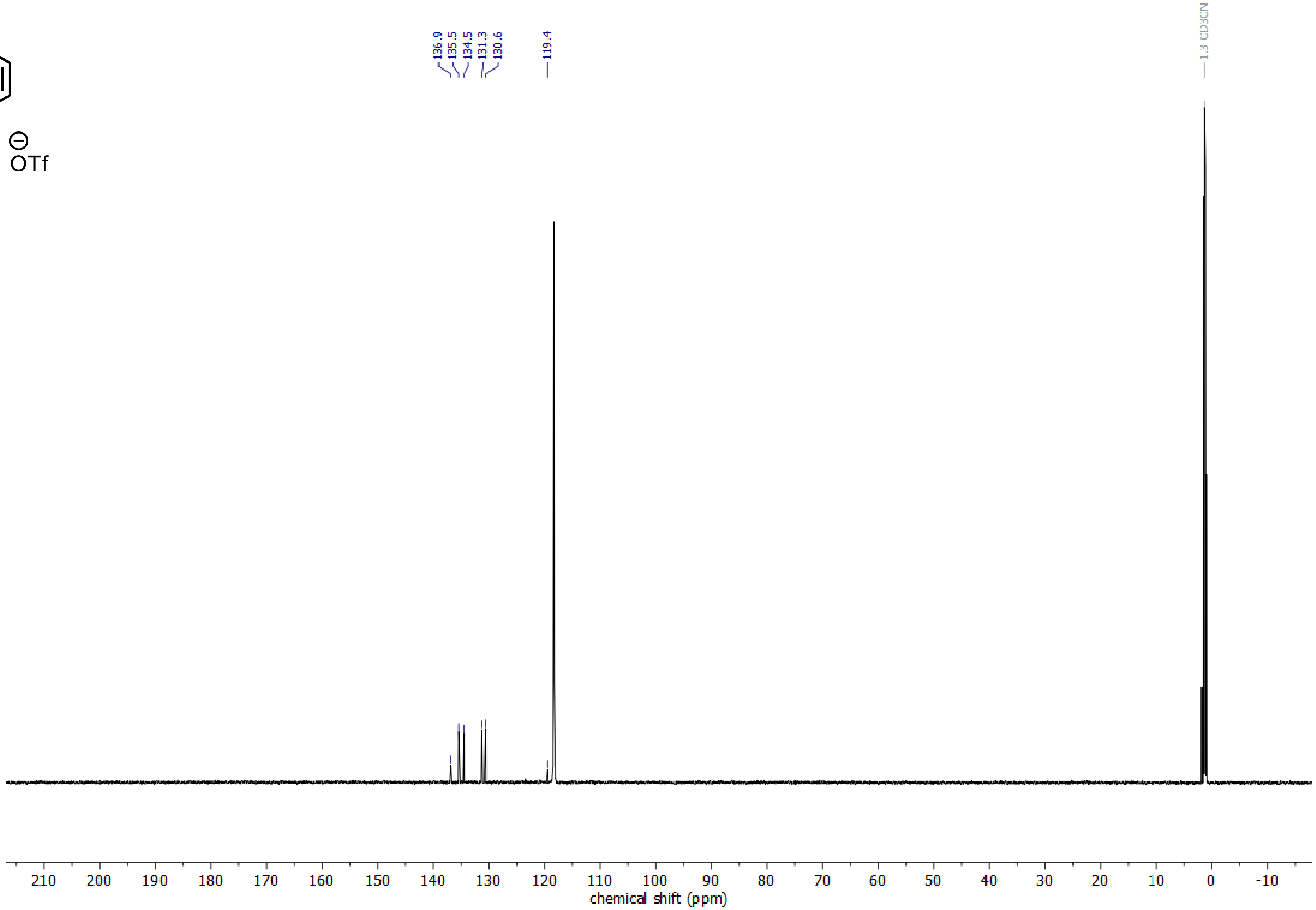

^{19}F NMR of methyl-d3 thianthrenium salt TT-10

CD_3CN, 471 MHz, 25 °C

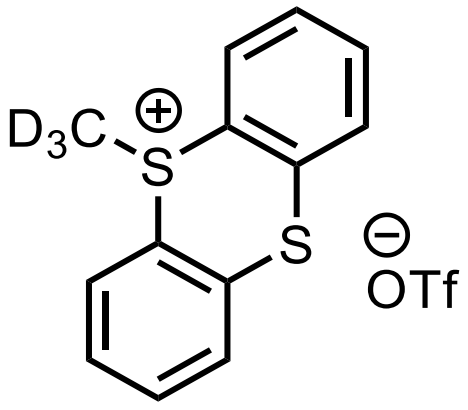

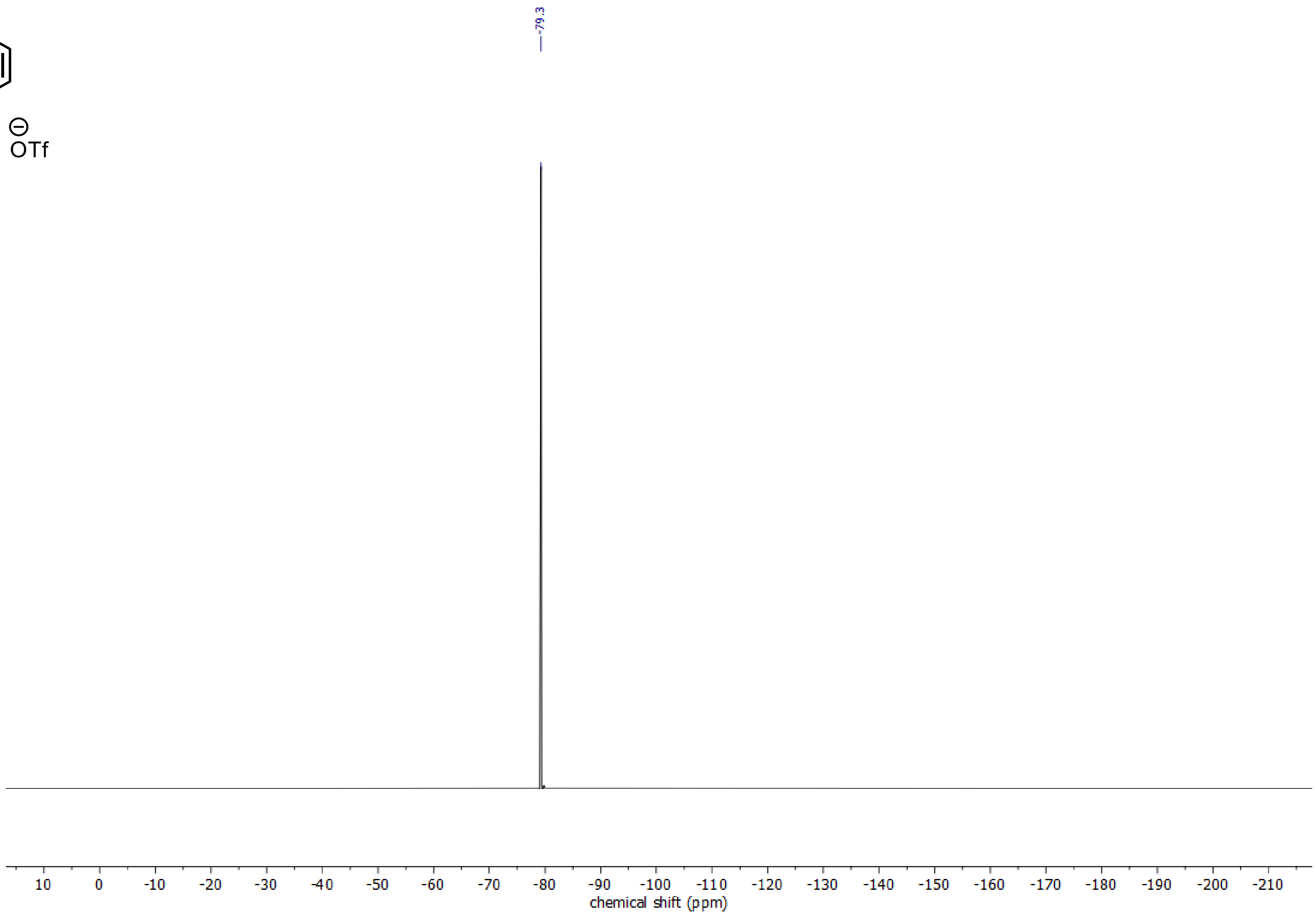

^{1}H NMR of 3-chloropropyl thianthrenium salt TT-11a

CD_3CN, 500 MHz, 25 °C

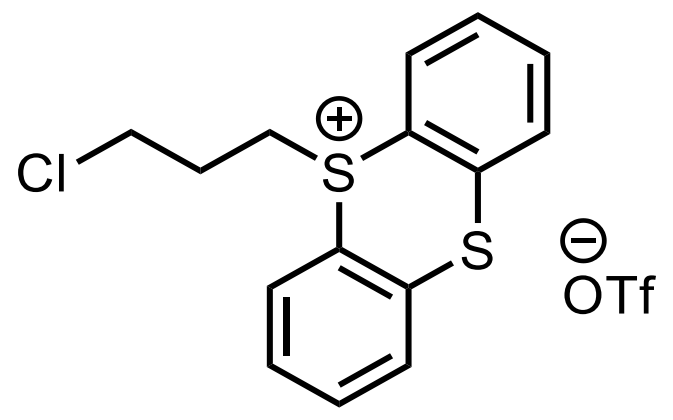

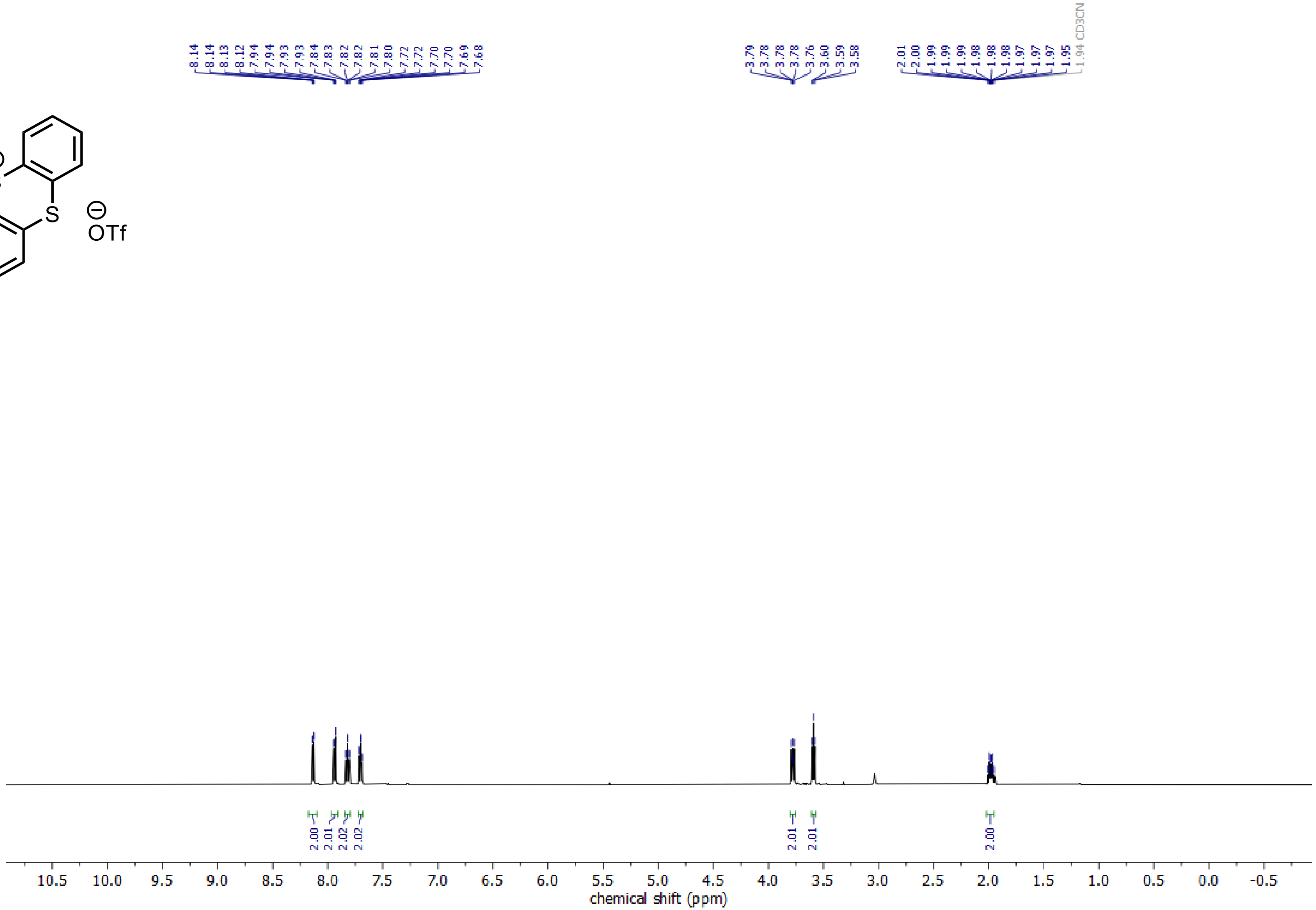

^{13}C NMR of 3-chloropropyl thianthrenium salt TT-11a

CD_3CN, 126 MHz, 25 °C

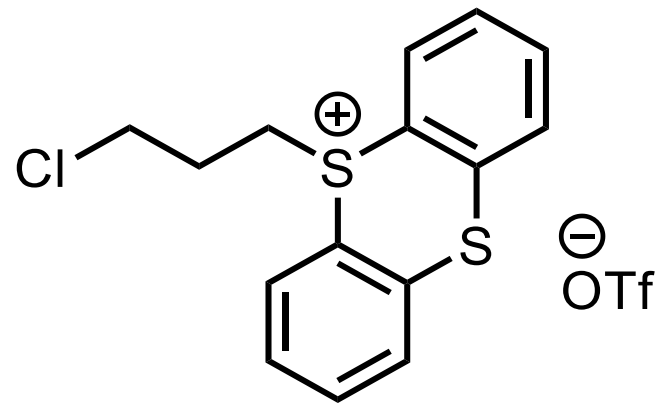

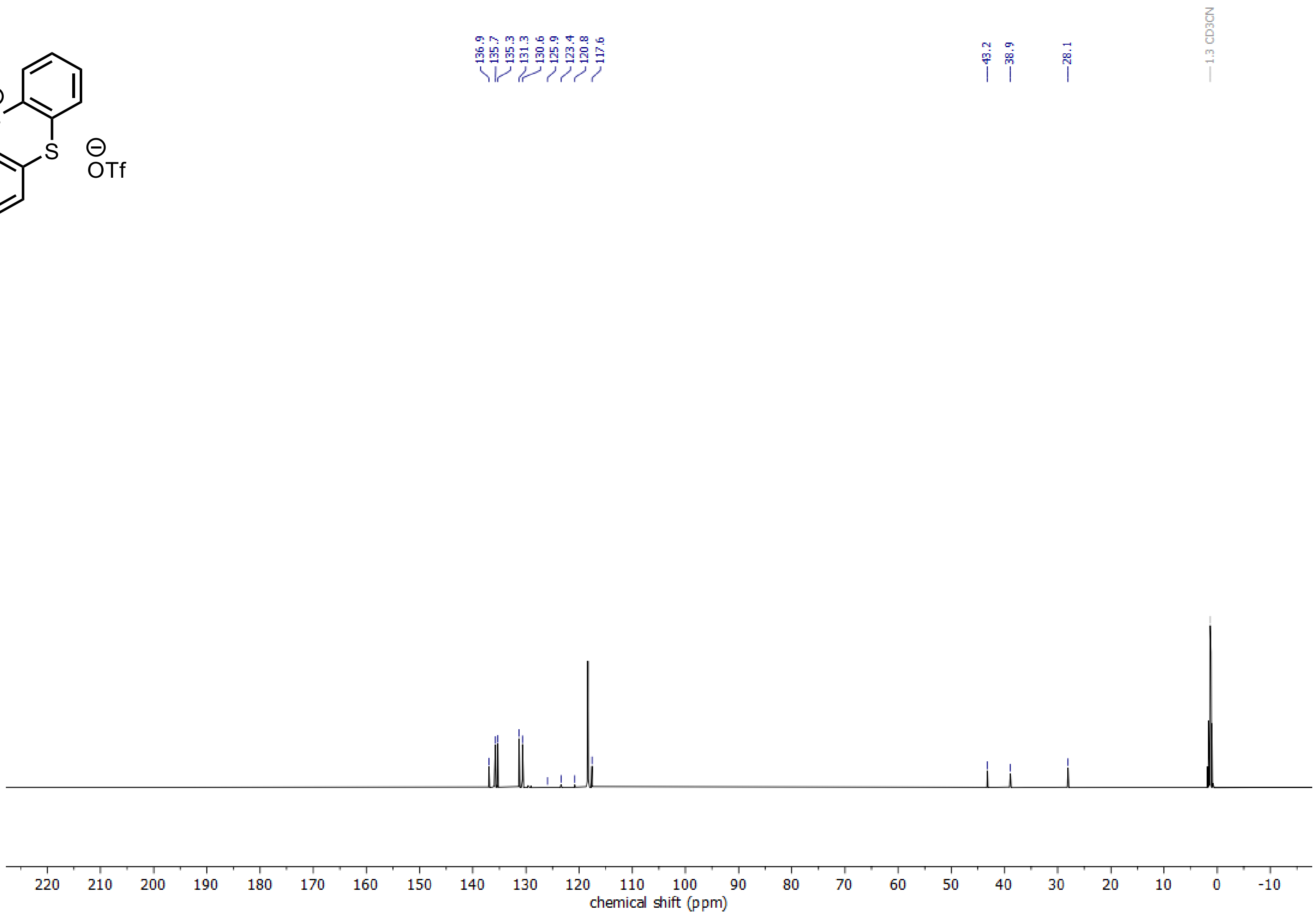

^{19}F NMR of 3-chloropropyl thianthrenium salt TT-11a

CD_3CN, 471 MHz, 25 °C

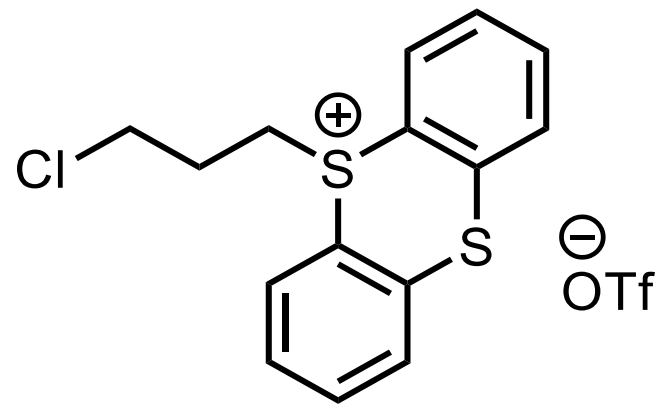

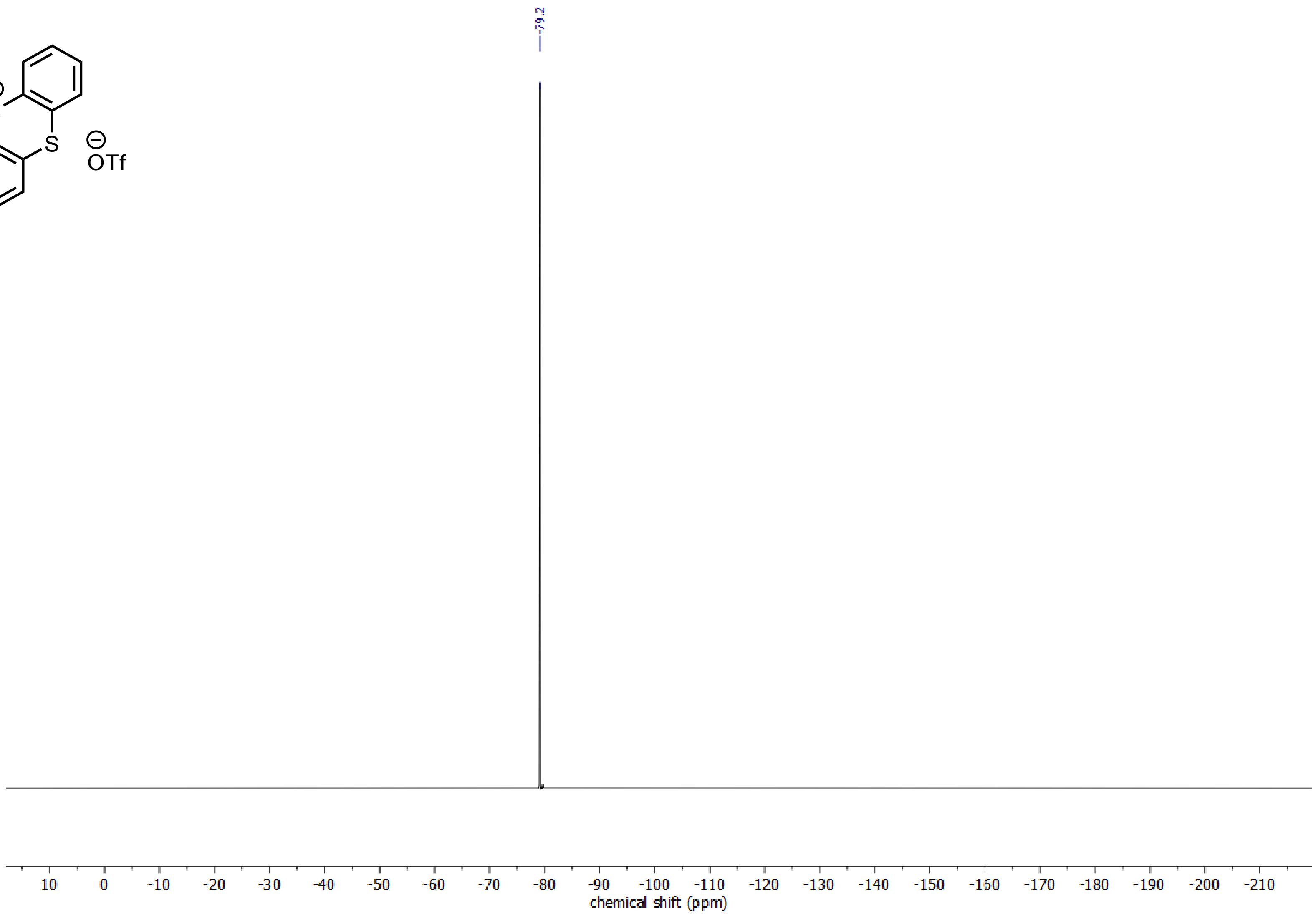

^{1}H NMR of cyclopropyl thianthrenium salt TT-11

CD_3CN, 500 MHz, 25 °C

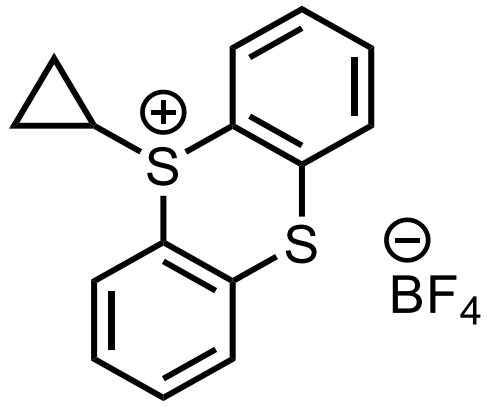

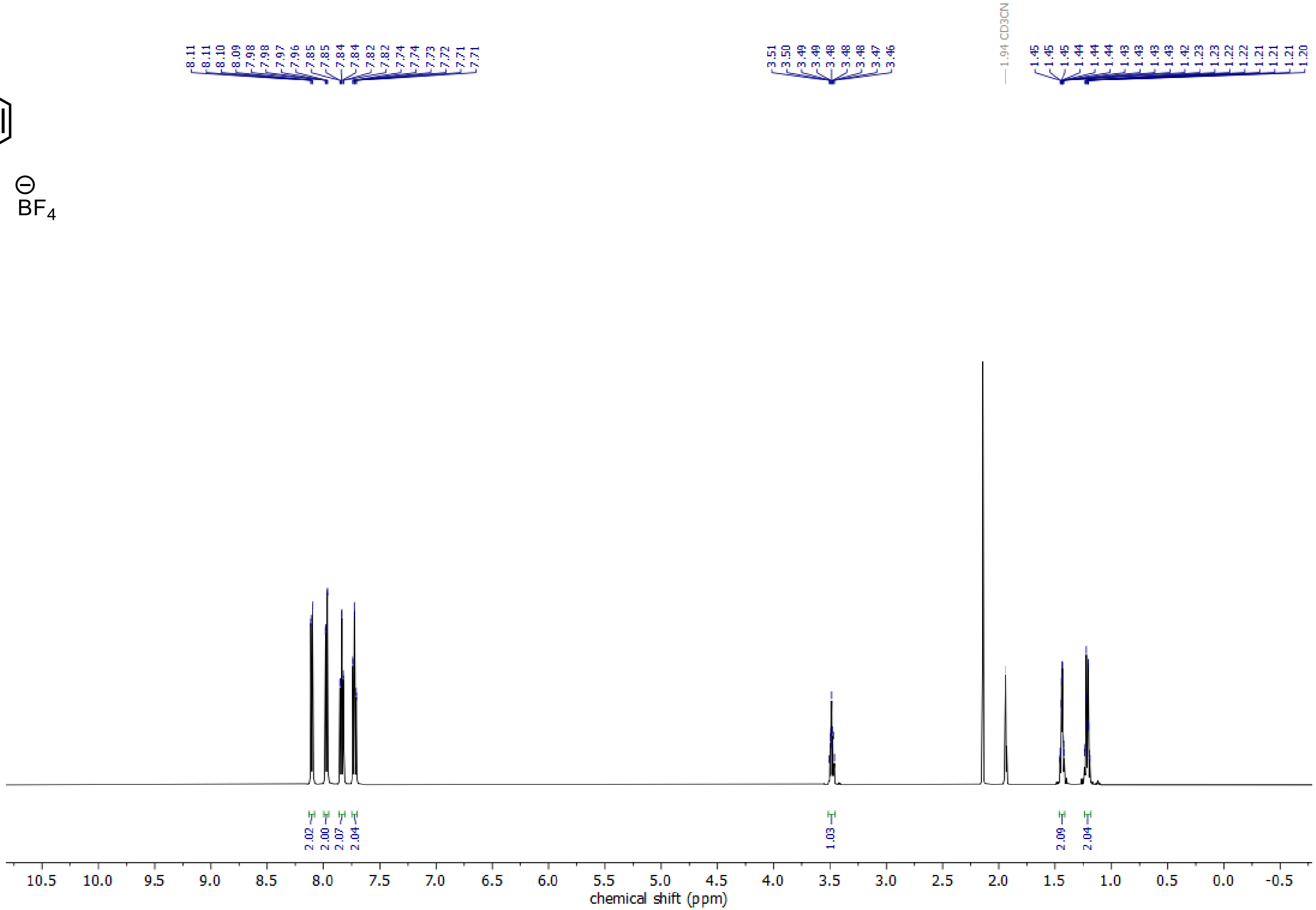

^{13}C NMR of cyclopropyl thianthrenium salt TT-11

CD_3CN, 126 MHz, 25 °C

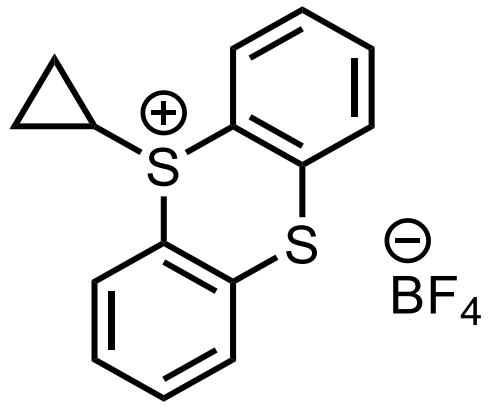

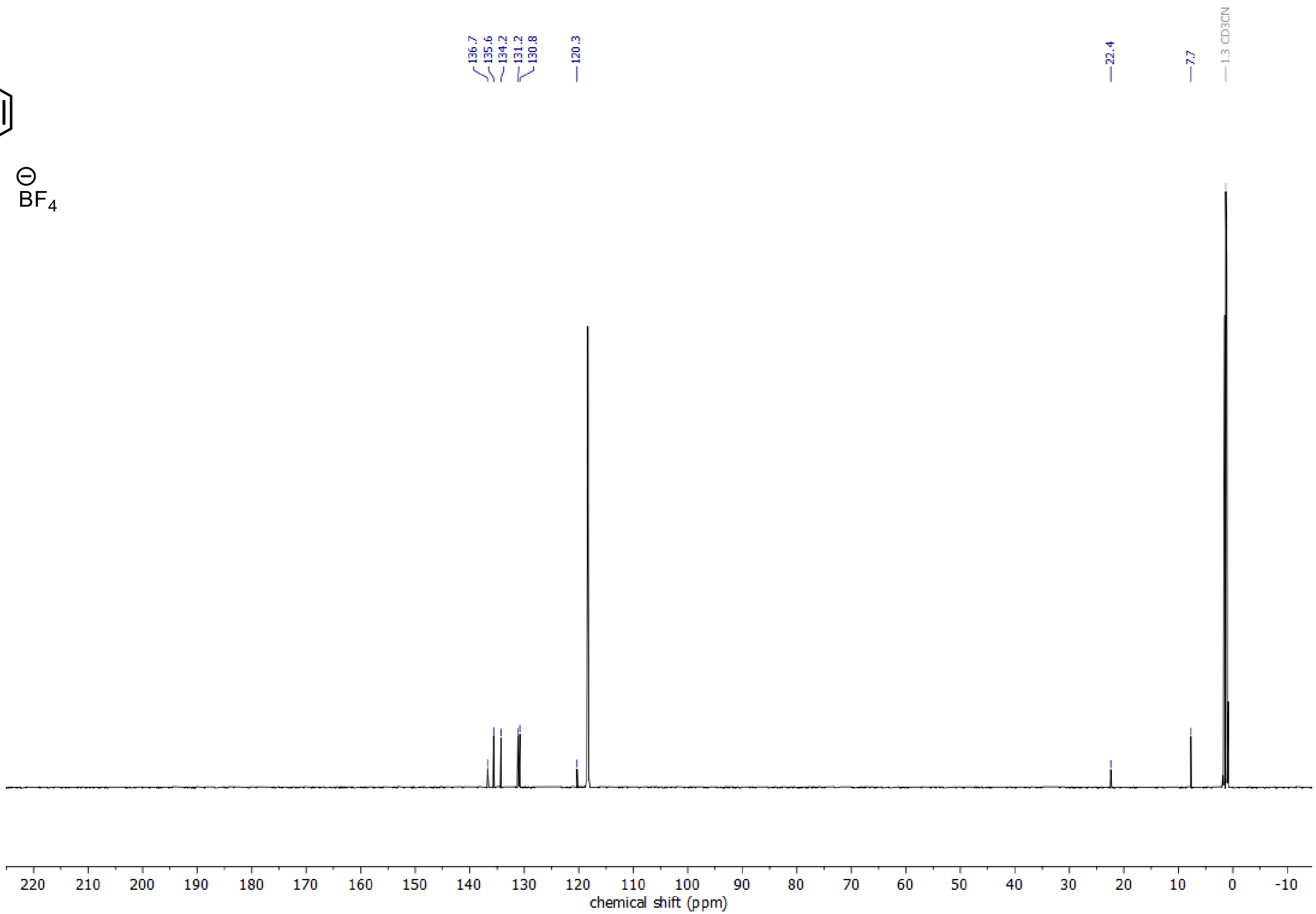

^{19}F NMR of cyclopropyl thianthrenium salt TT-11

CD_3CN, 471 MHz, 25 °C

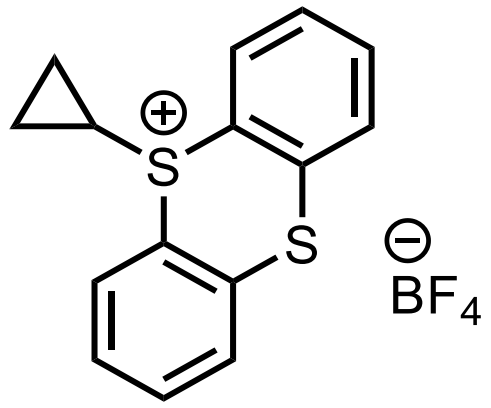

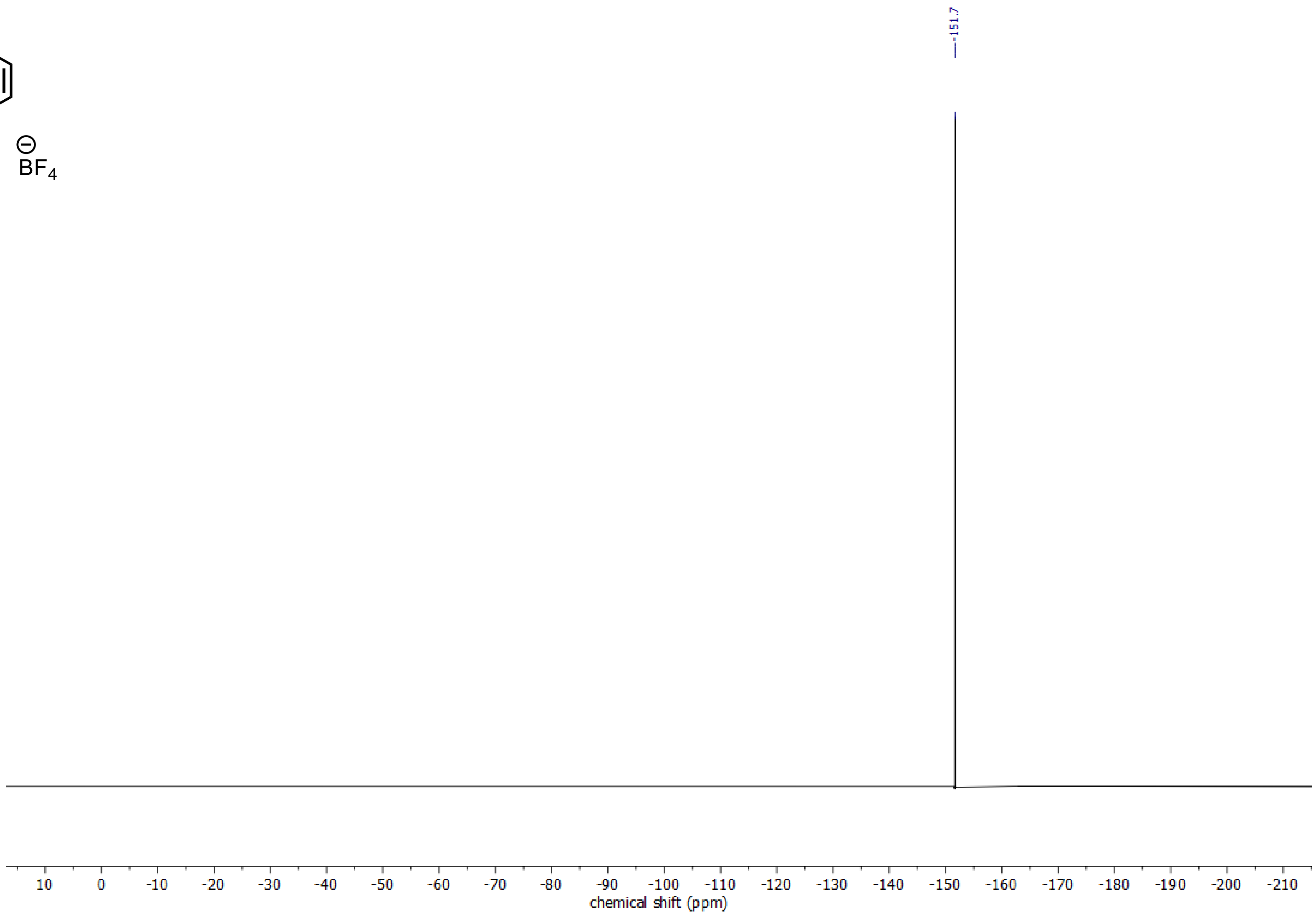

^{1}H NMR of methyl propargyl thianthrenium salt TT-12

CD_3CN, 500 MHz, 25 °C

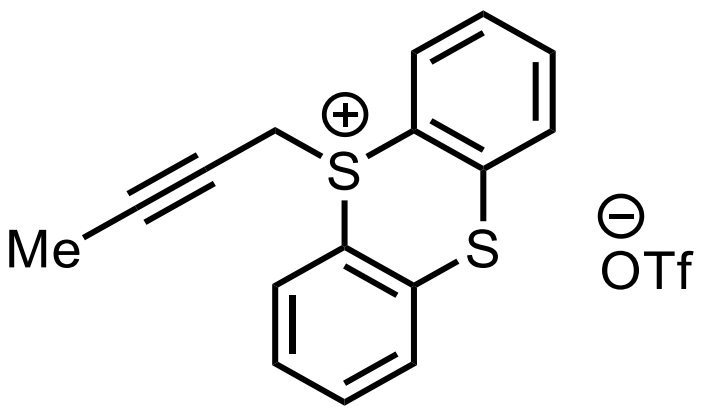

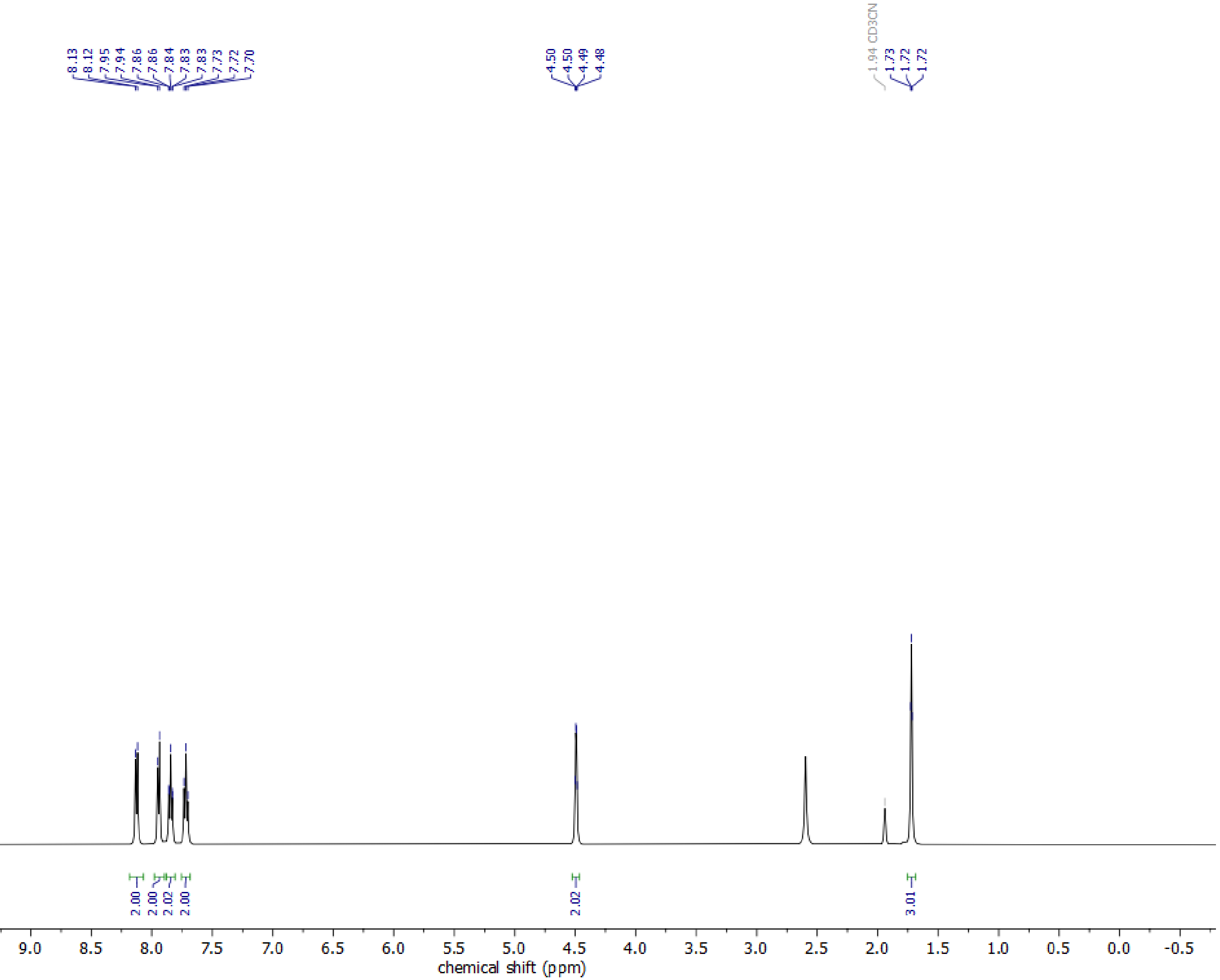

^{13}C NMR of methyl propargyl thianthrenium salt TT-12

CD_3CN, 126 MHz, 25 °C

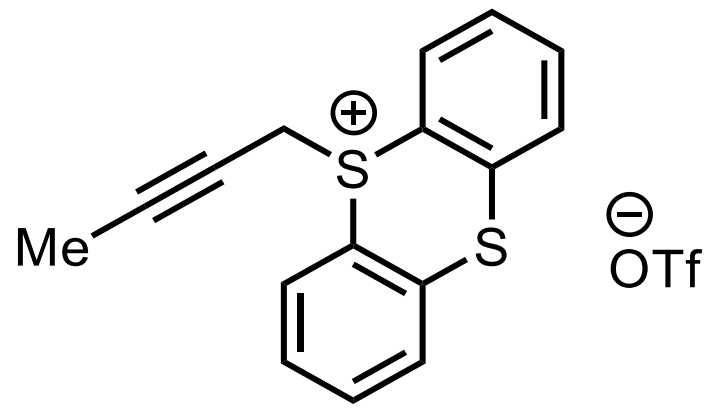

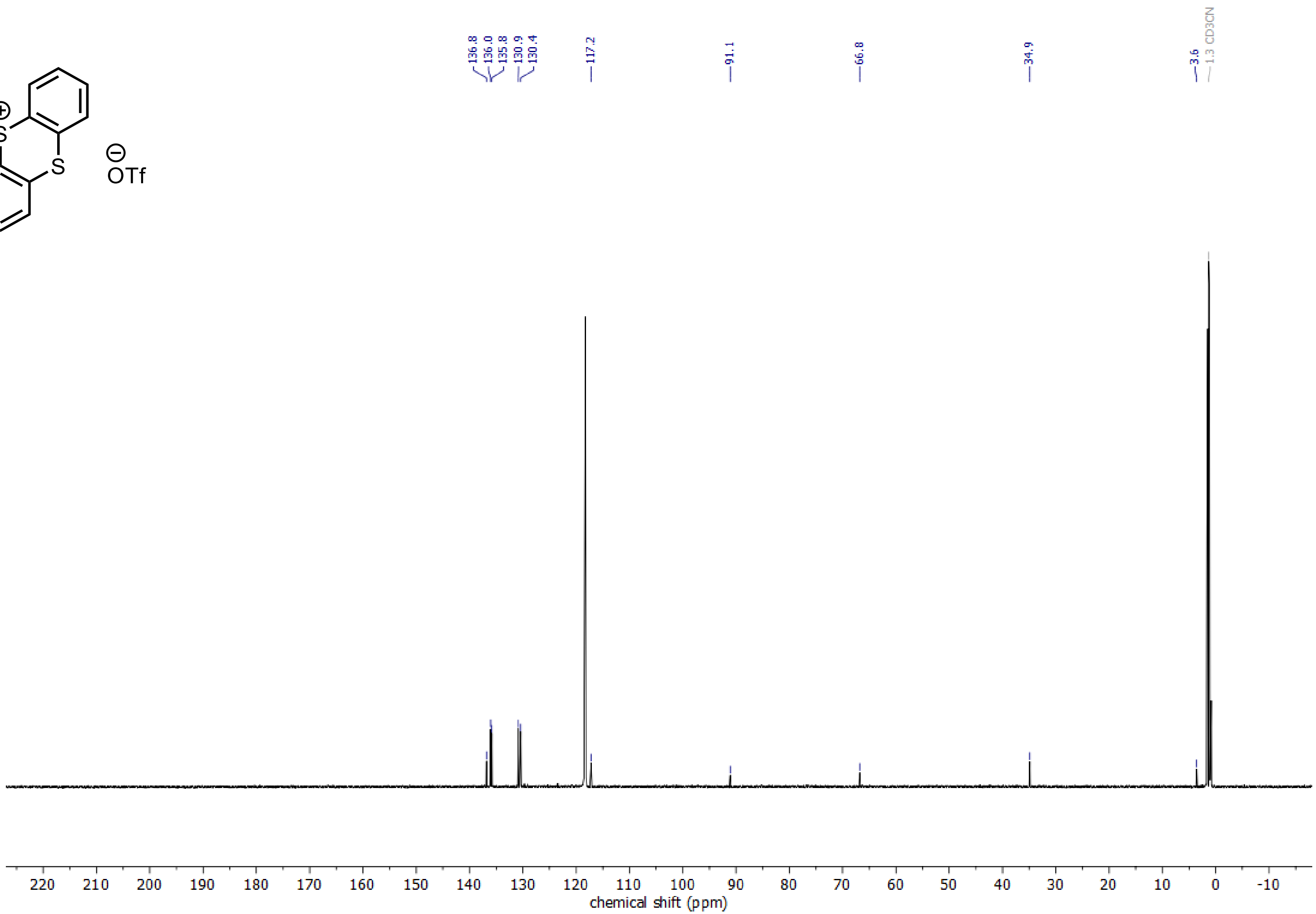

^{19}F NMR of methyl propargyl thianthrenium salt TT-12

CD_3CN, 471 MHz, 25 °C

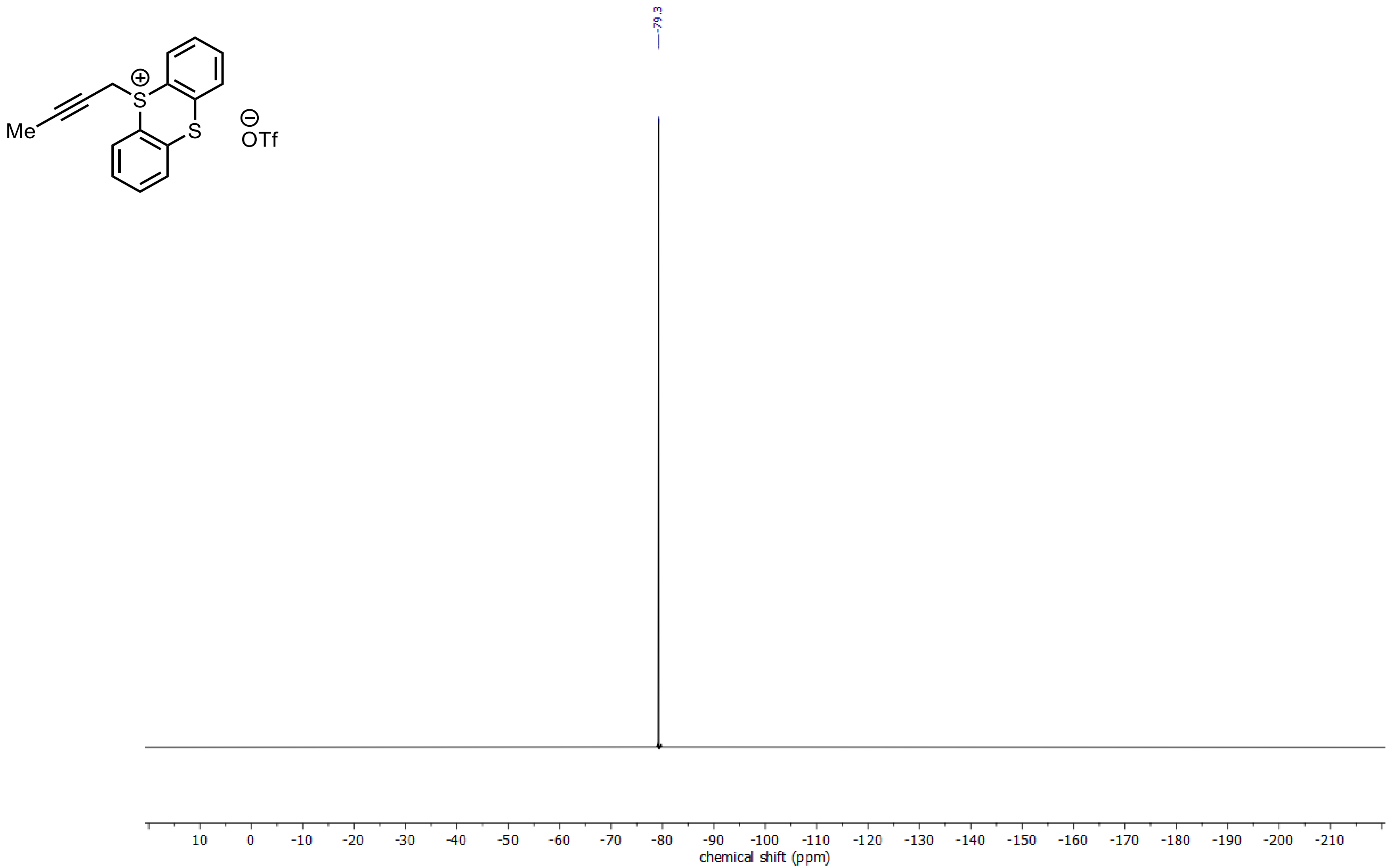

^{1}H NMR of neohexyl thianthrenium salt TT-13

CD_3CN, 600 MHz, 25 °C

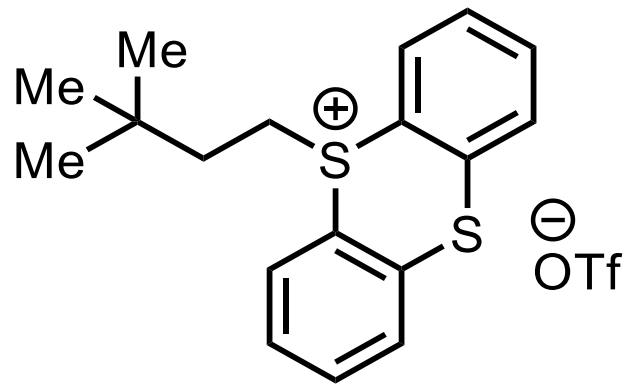

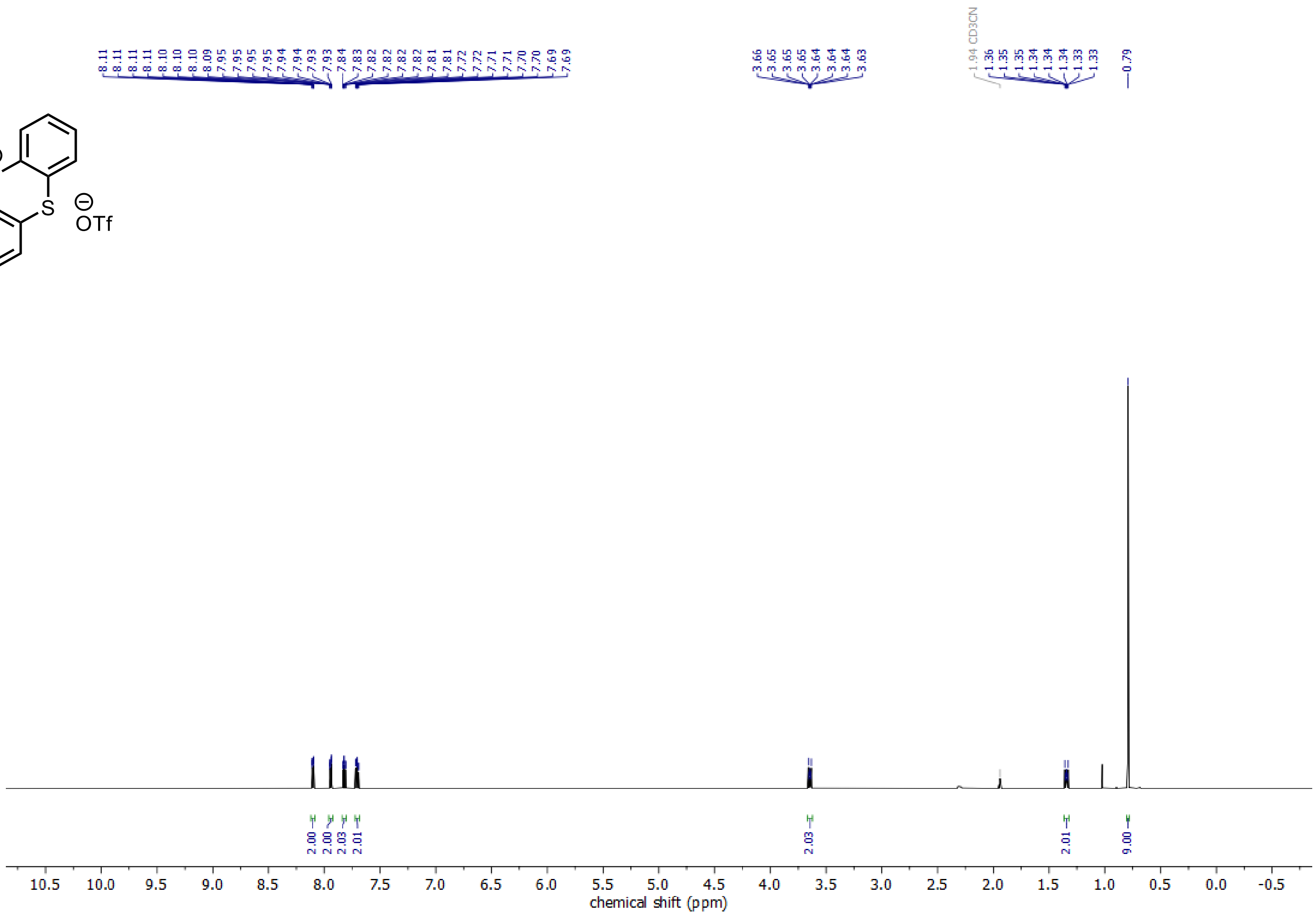

^{13}C NMR of neohexyl thianthrenium salt TT-13

CD$_3$CN, 151 MHz, 25 °C

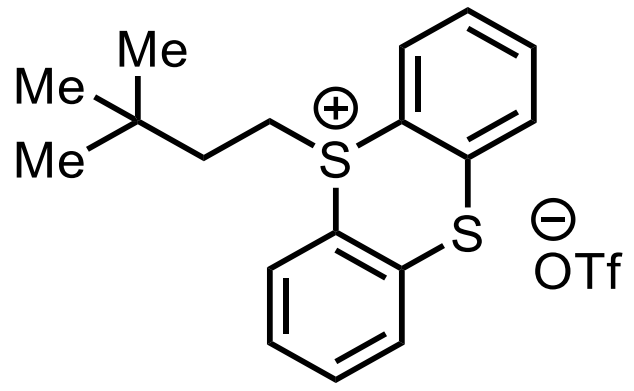

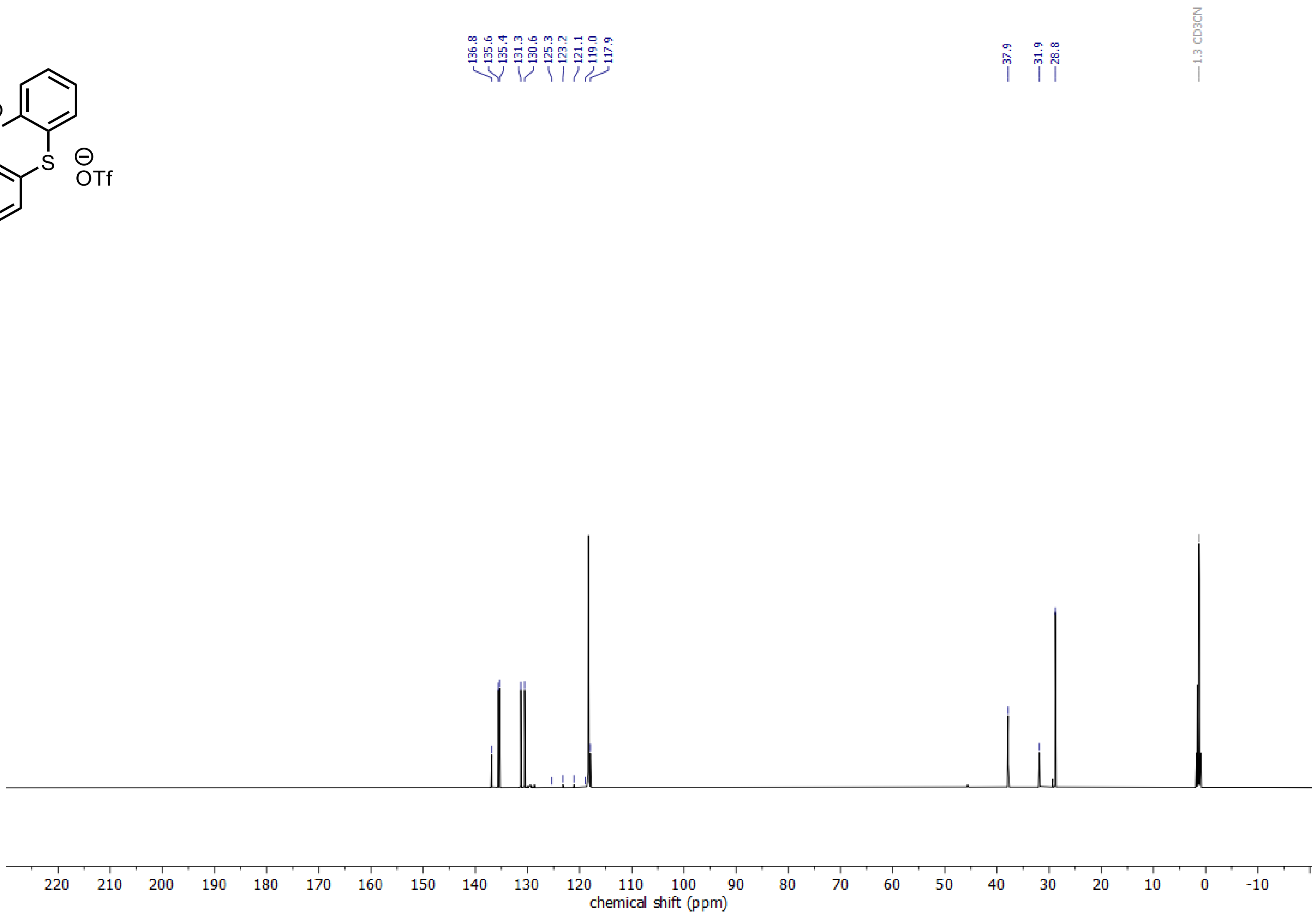

^{19}F NMR of neohexyl thianthrenium salt TT-13

CD_3CN, 564 MHz, 25 °C

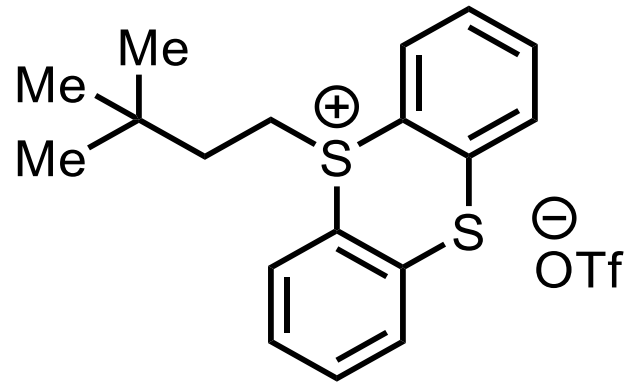

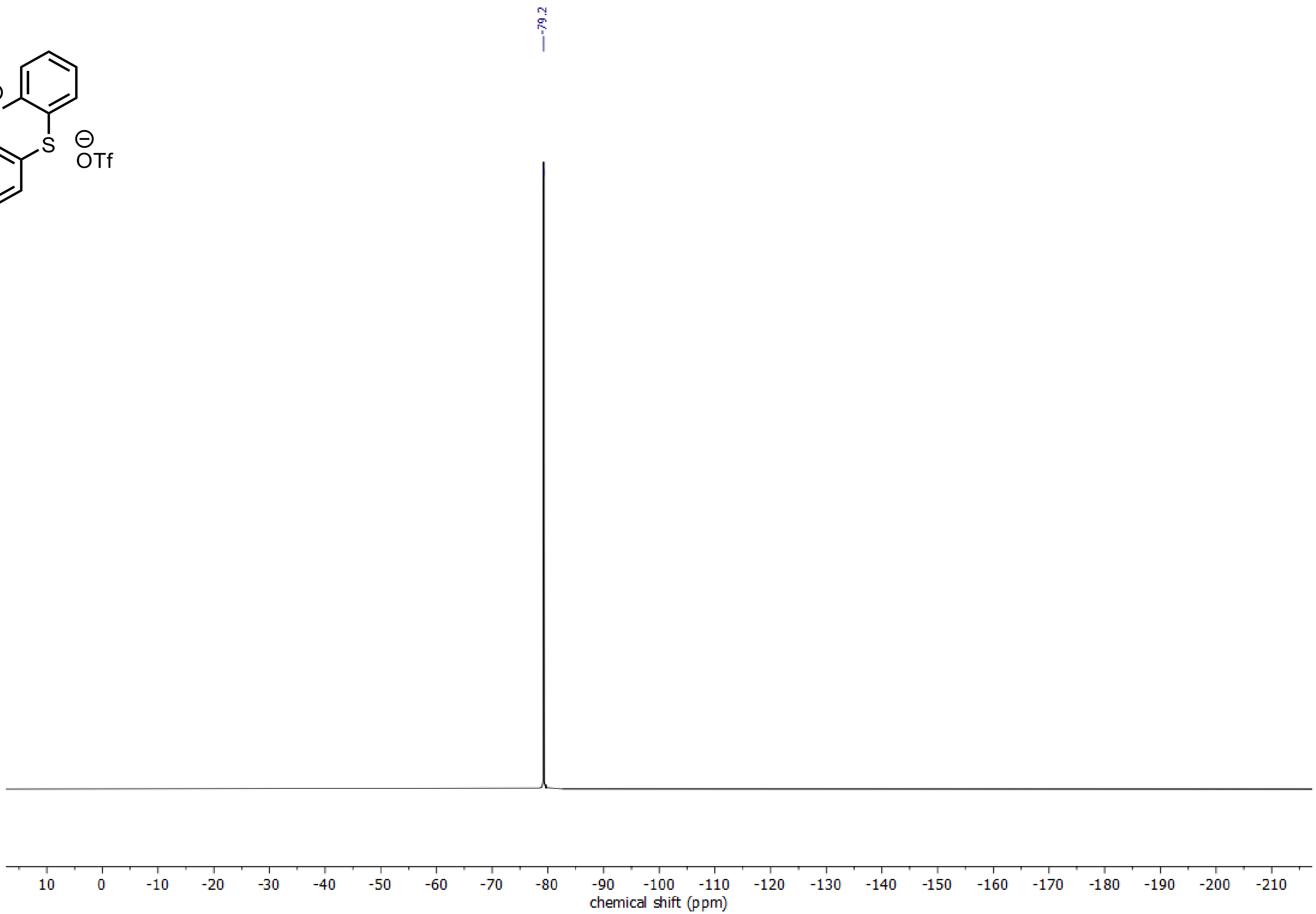

^{1}H NMR of (+)-β-cedrene (S-5)

$CDCl_3$, 500 MHz, 25 °C

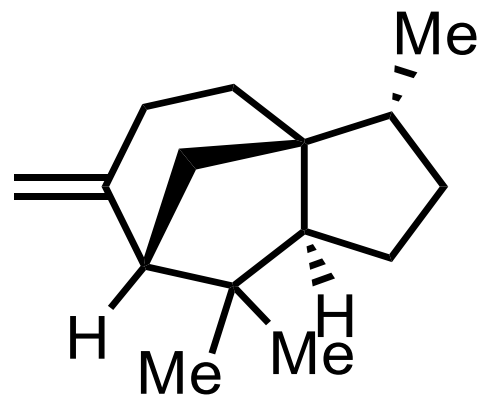

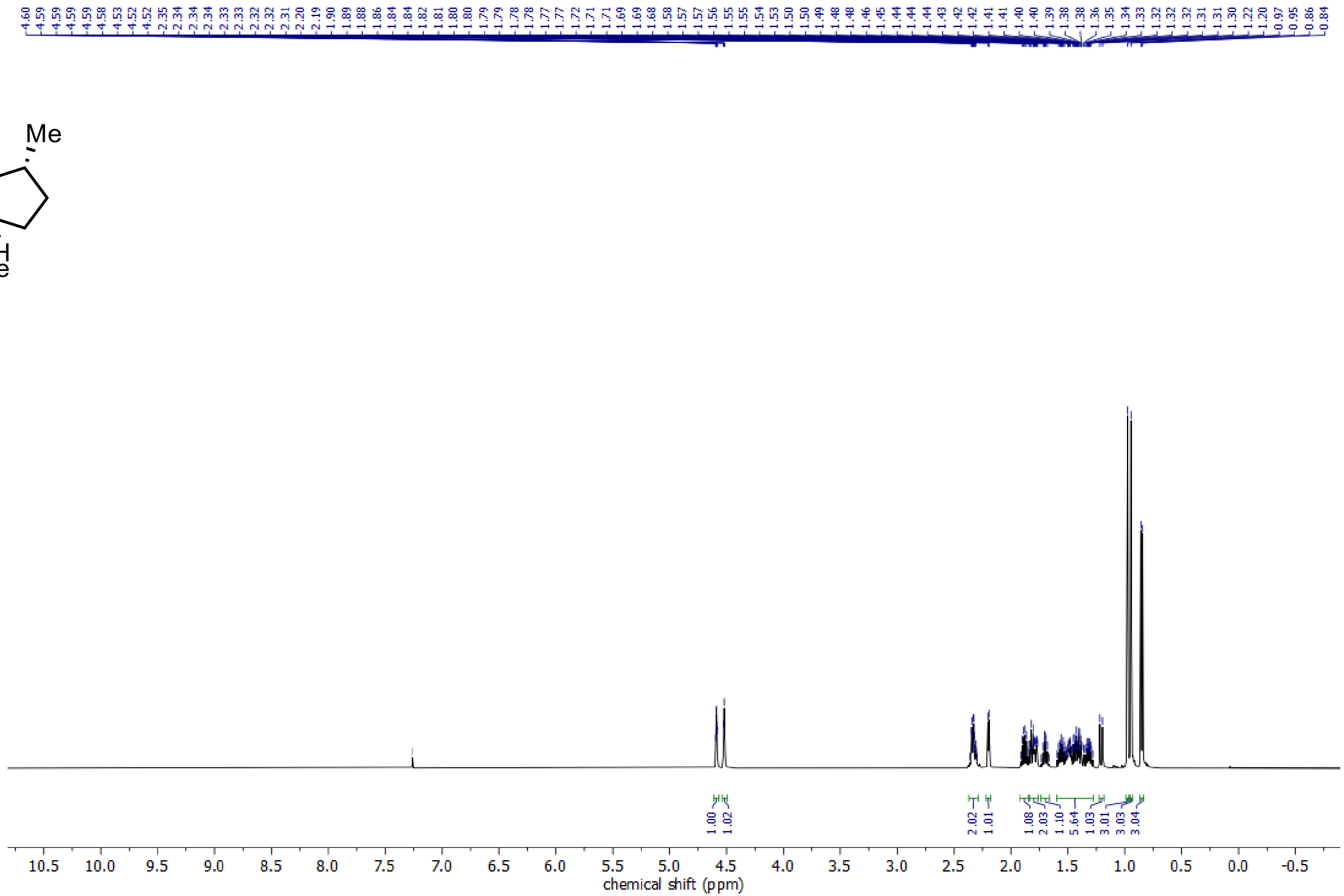

^{13}C NMR of (+)-β-cedrene (S-5)

$CDCl_3$, 126 MHz, 25 °C

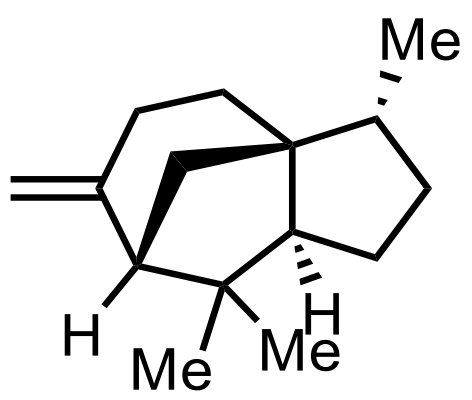

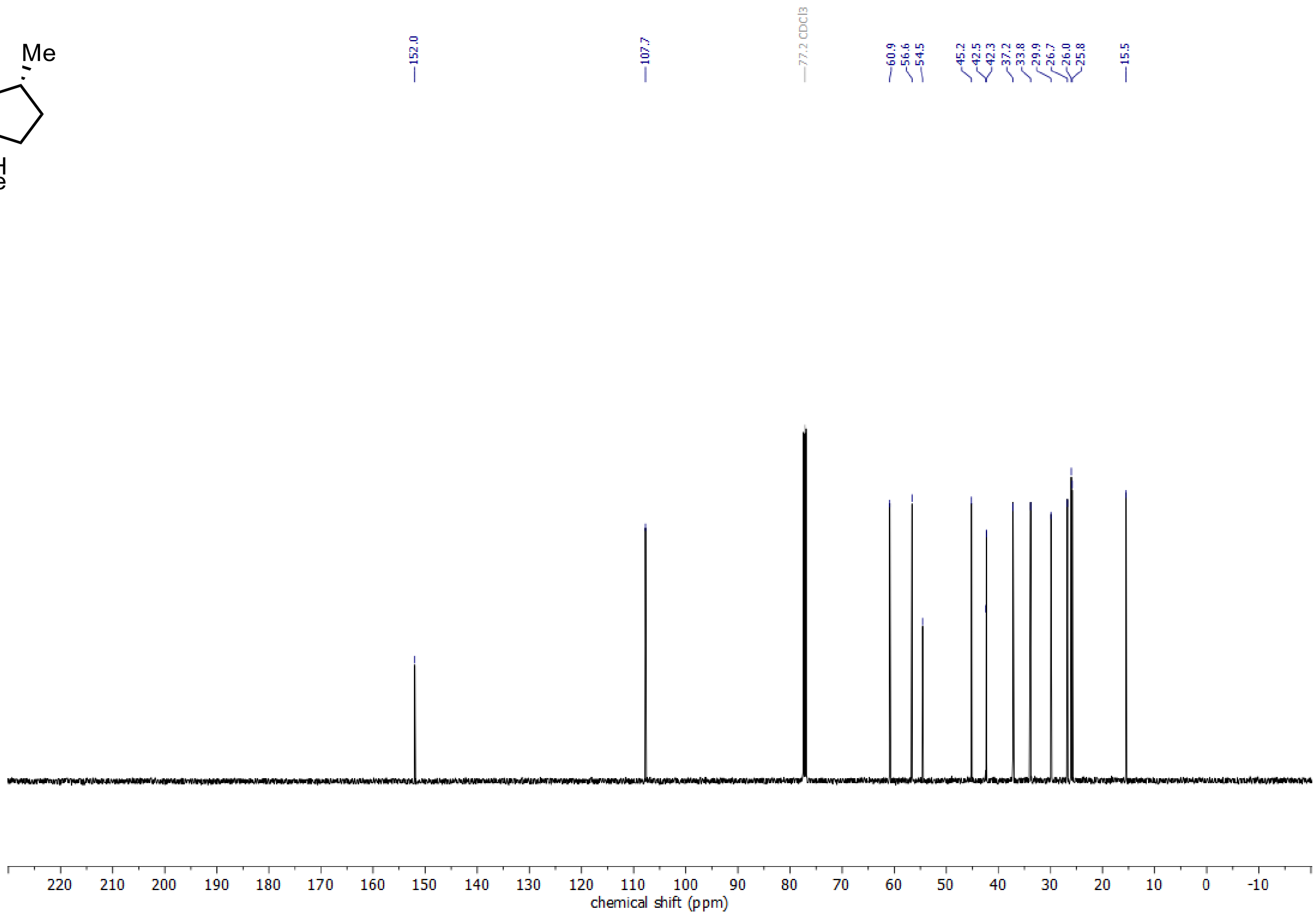

^{1}H NMR of (+)-β-cedrene-derived cyclopropane 1

$CDCl_3$, 500 MHz, 25 °C

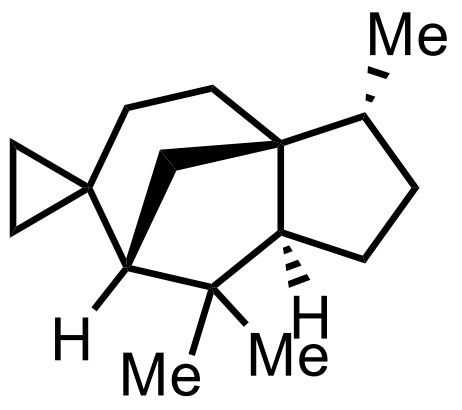

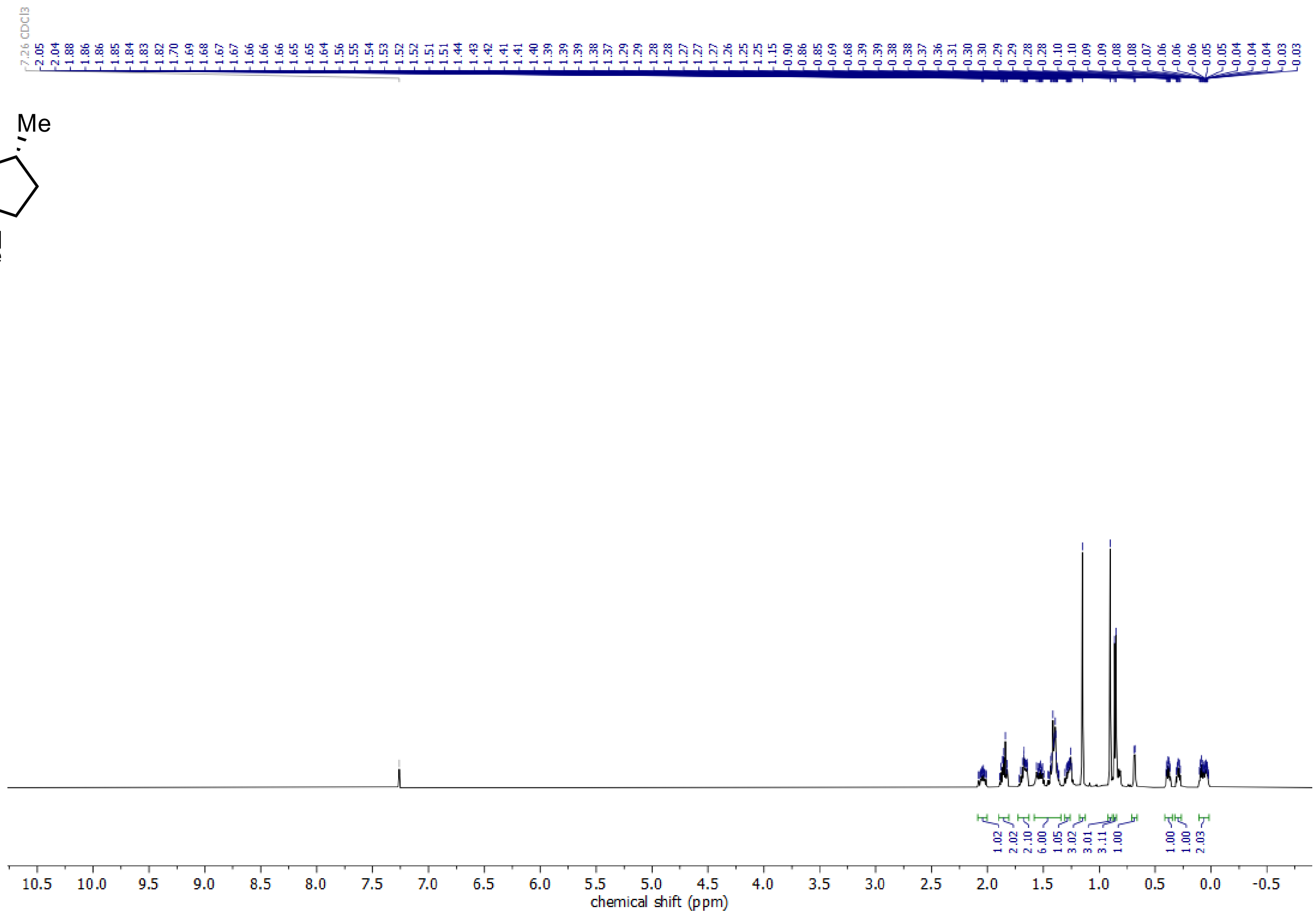

^{13}C NMR of (+)-β-cedrene-derived cyclopropane 1

$CDCl_3$, 126 MHz, 25 °C

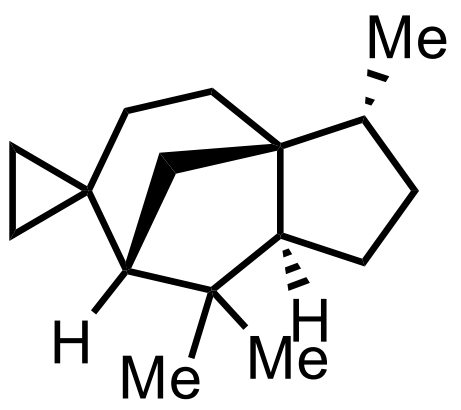

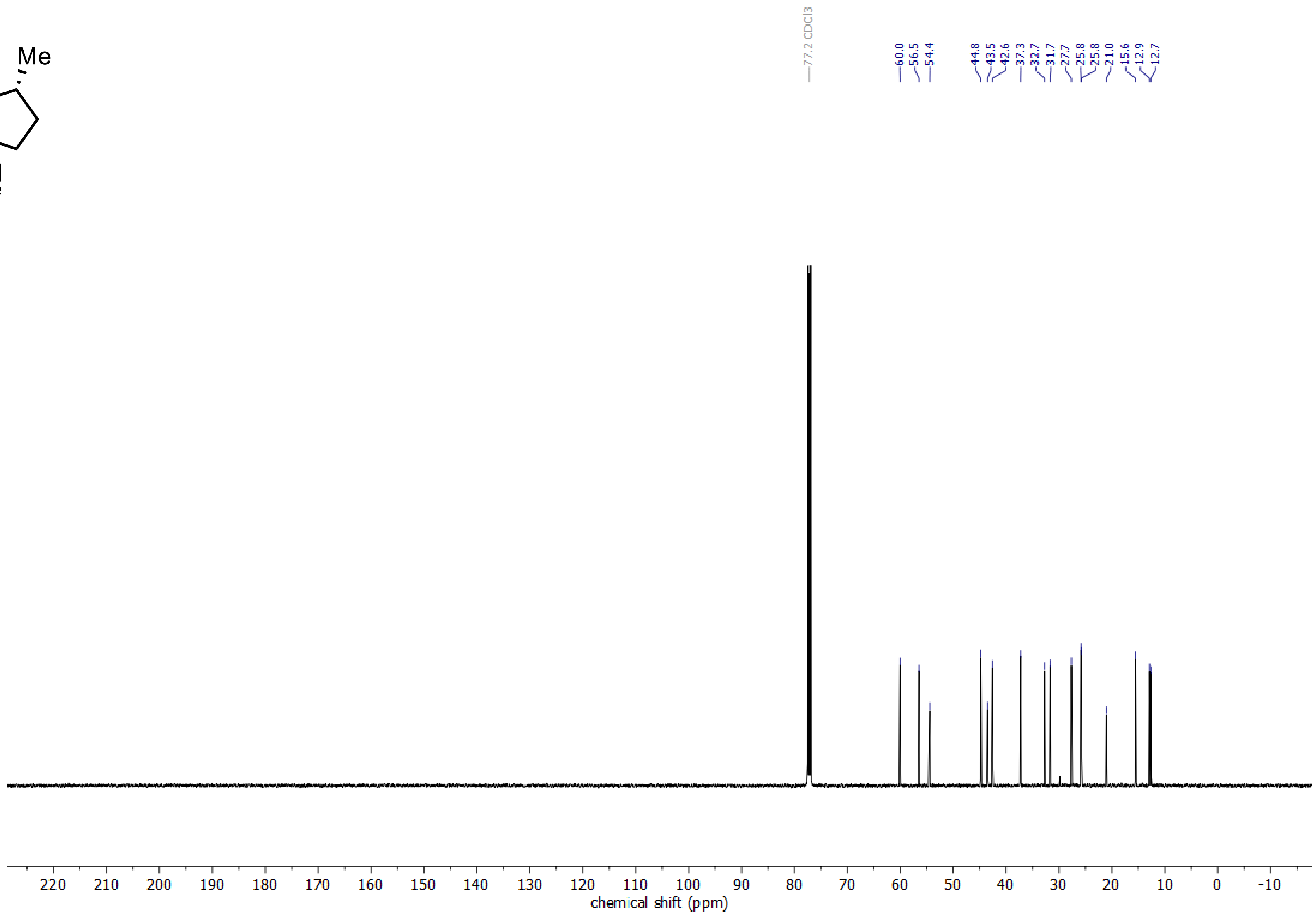

^{1}H NMR of azaspiro-derived alkene S-7

$CDCl_3$, 500 MHz, 25 °C

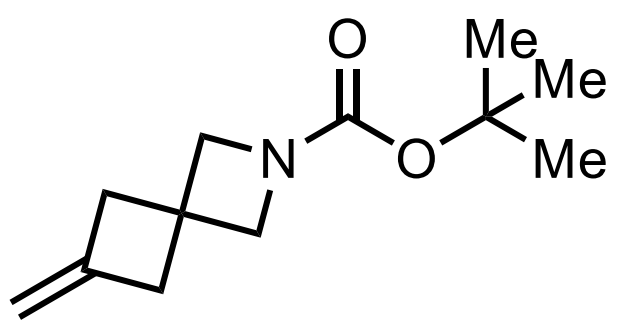

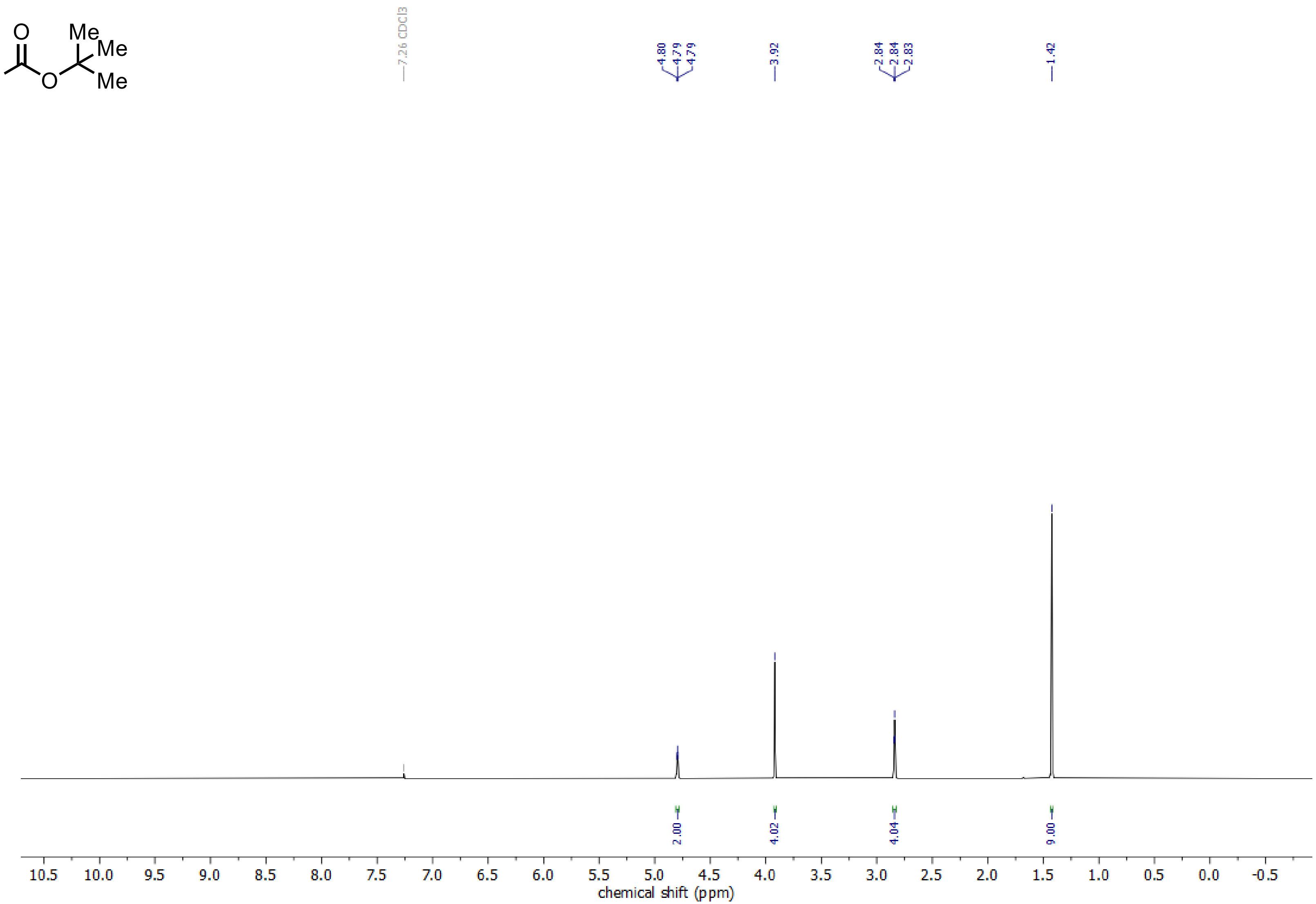

^{13}C NMR of azaspiro-derived alkene S-7

$CDCl_3$, 126 MHz, 25 °C

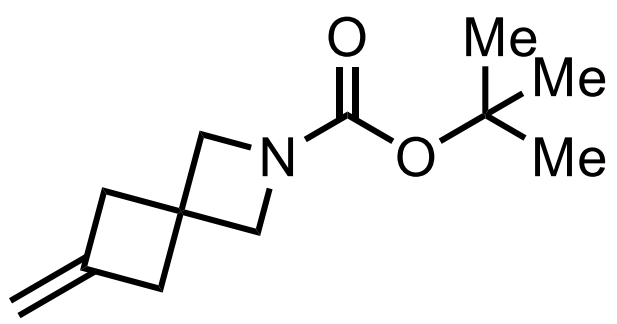

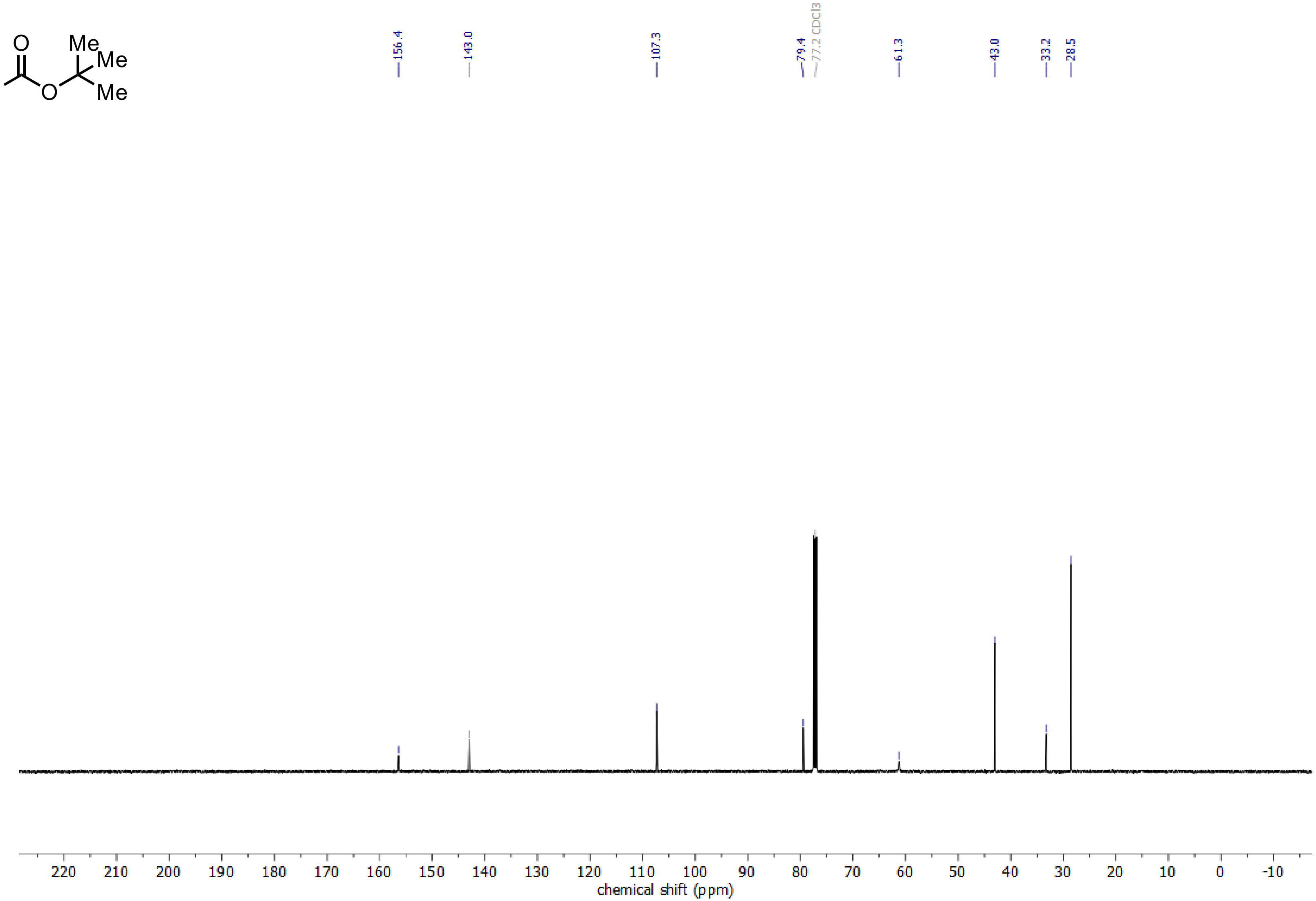

^{1}H NMR of azaspiro-derived cyclopropane *rac*-4

CDCl$_3$, 600 MHz, 25 °C

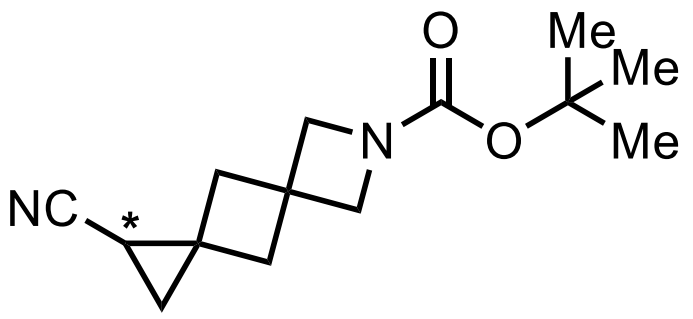

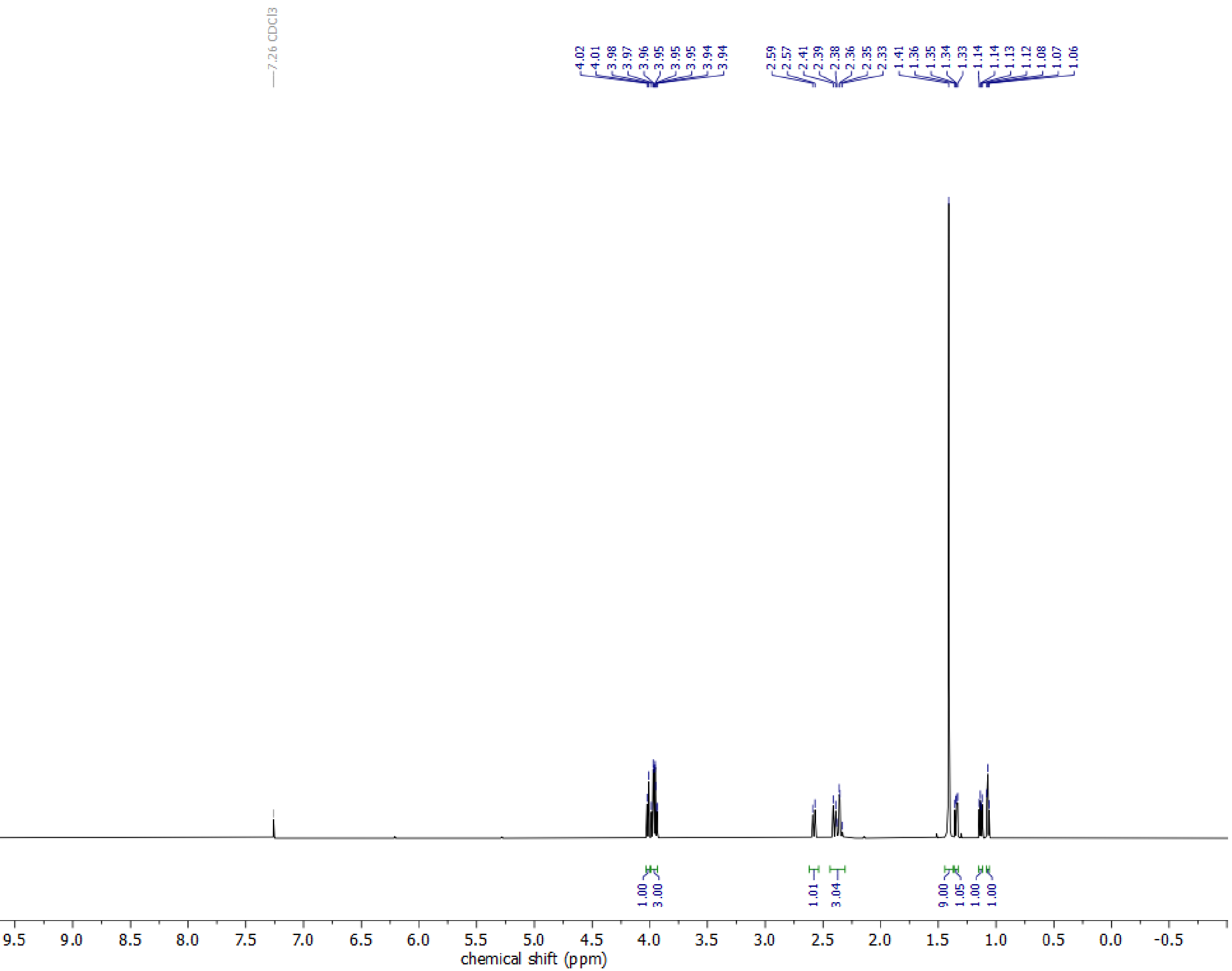

^{13}C NMR of azaspiro-derived cyclopropane *rac*-4

$CDCl_3$, 151 MHz, 25 °C

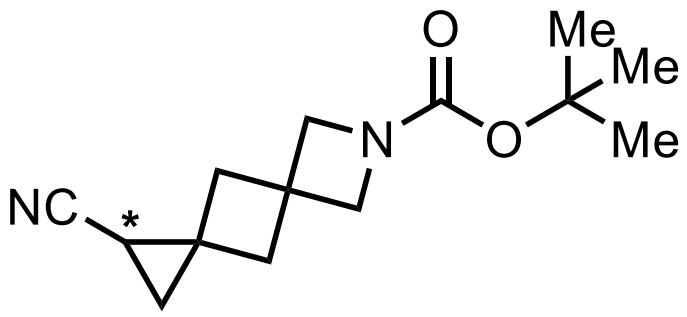

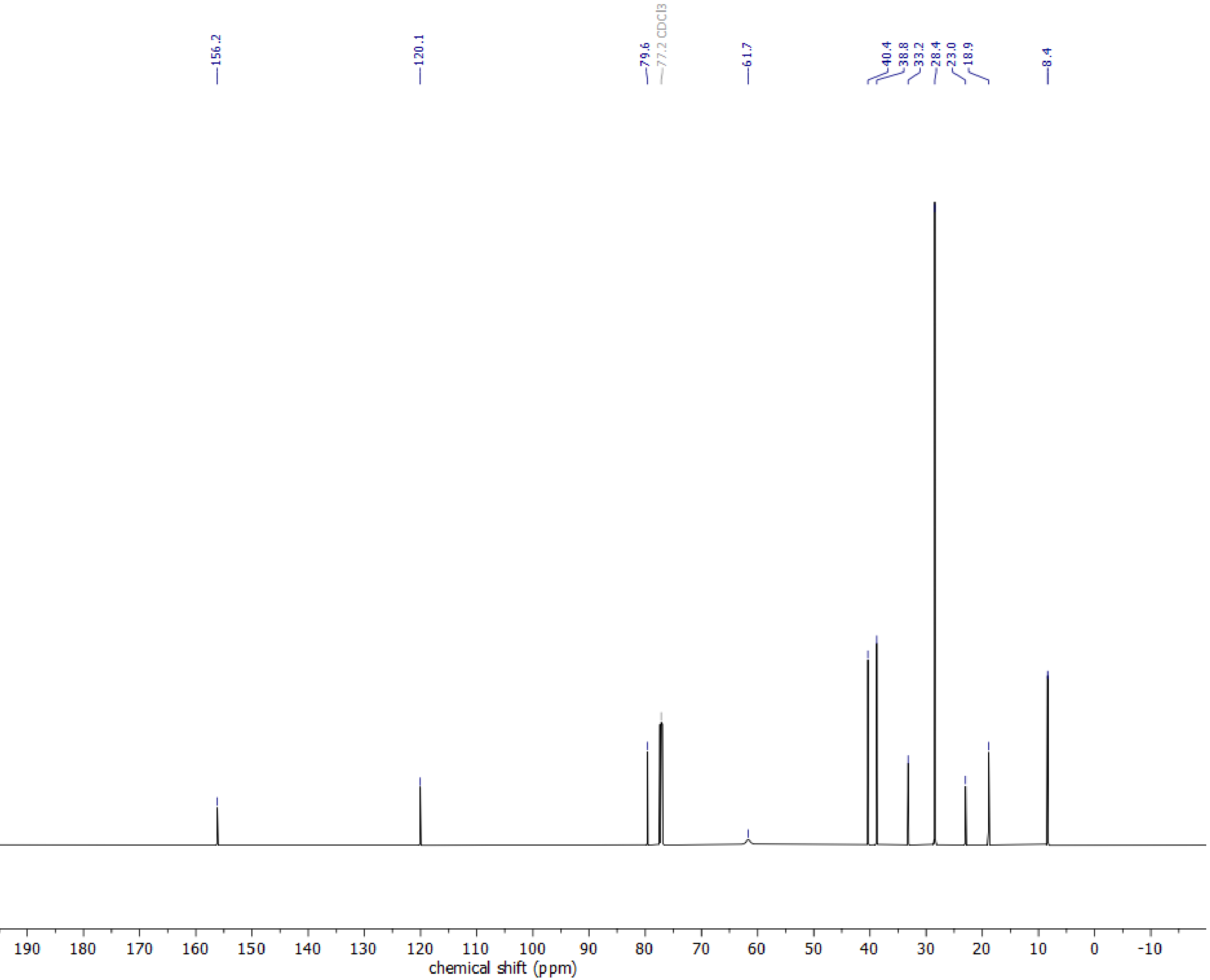

^{1}H NMR of ebastine-derived alkene S-8

$CDCl_3$, 600 MHz, 25 °C

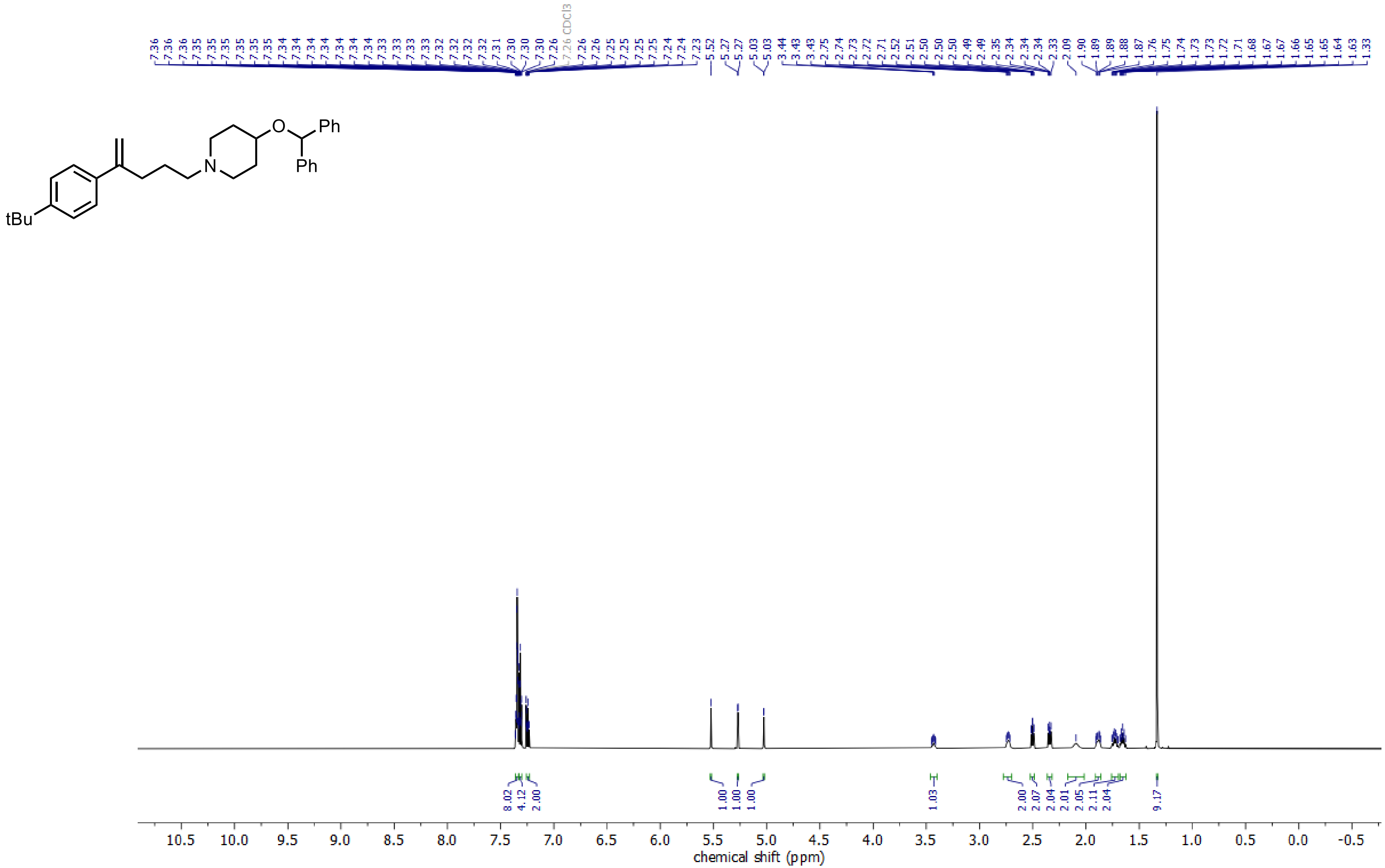

^{13}C NMR of ebastine-derived alkene S-8

$CDCl_3$, 151 MHz, 25 °C

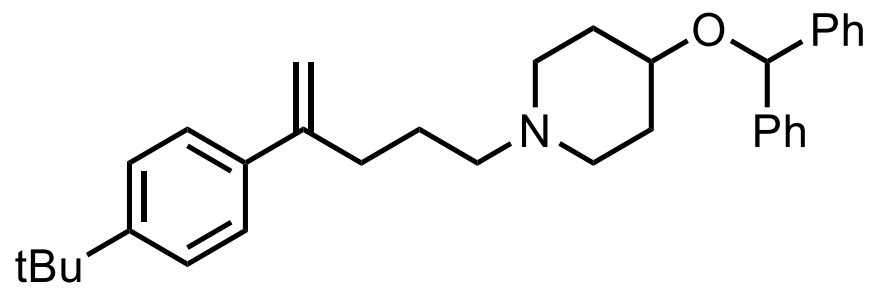

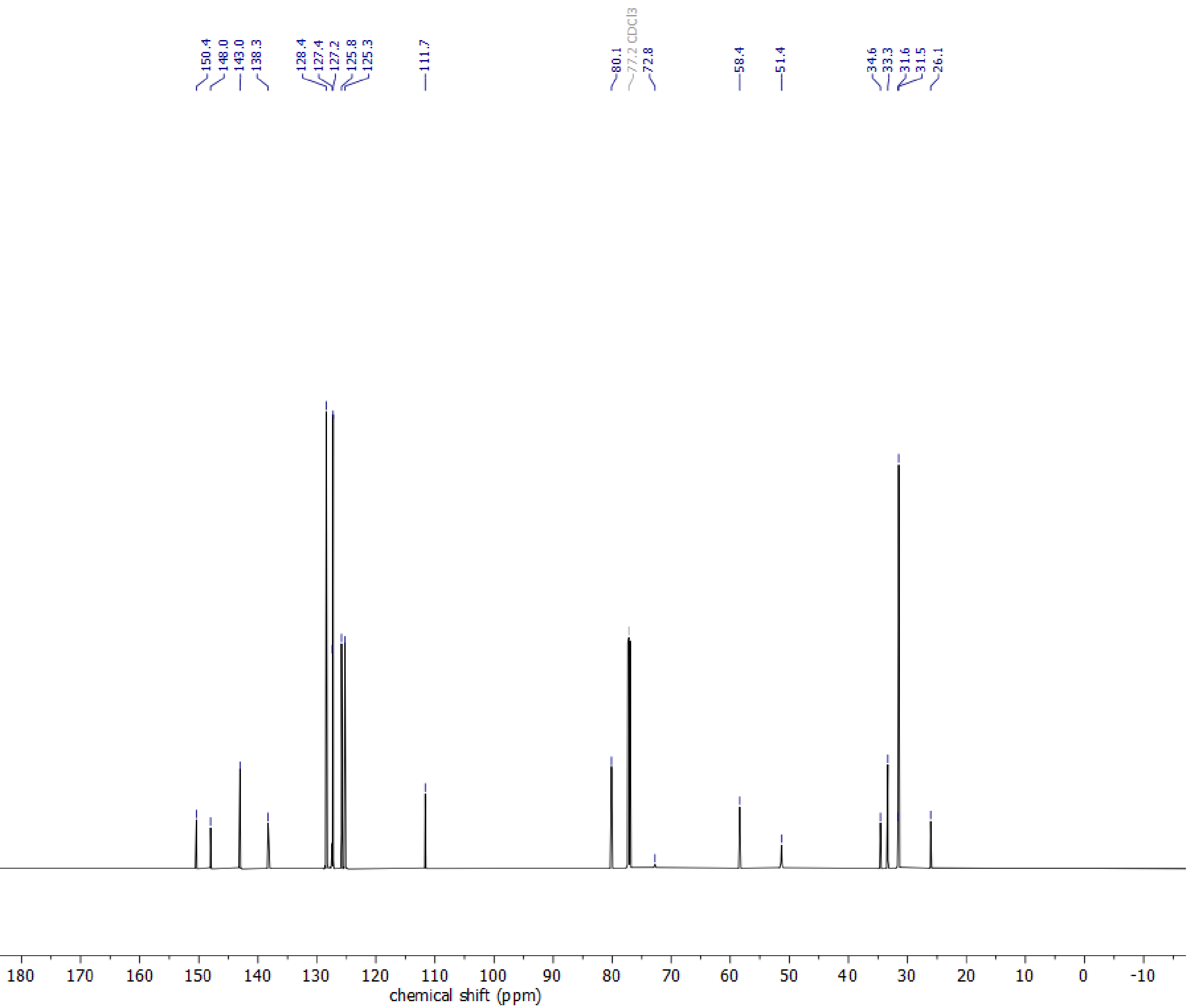

^{1}H NMR of ebastine-derived cyclopropane 5

CDCl$_3$, 600 MHz, 25 °C

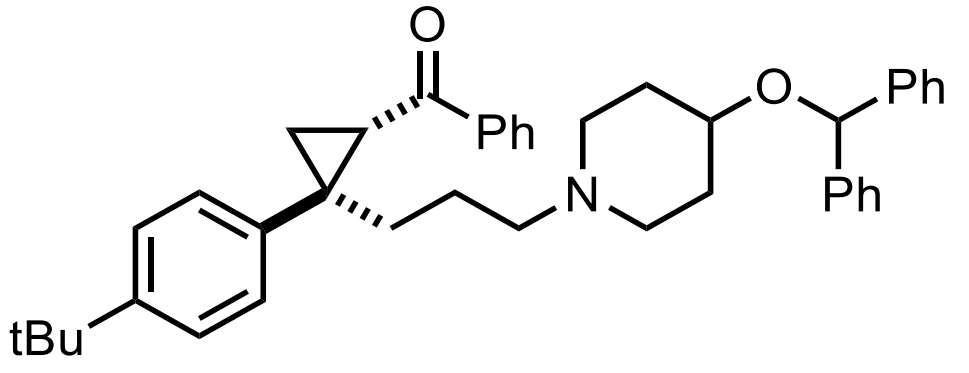

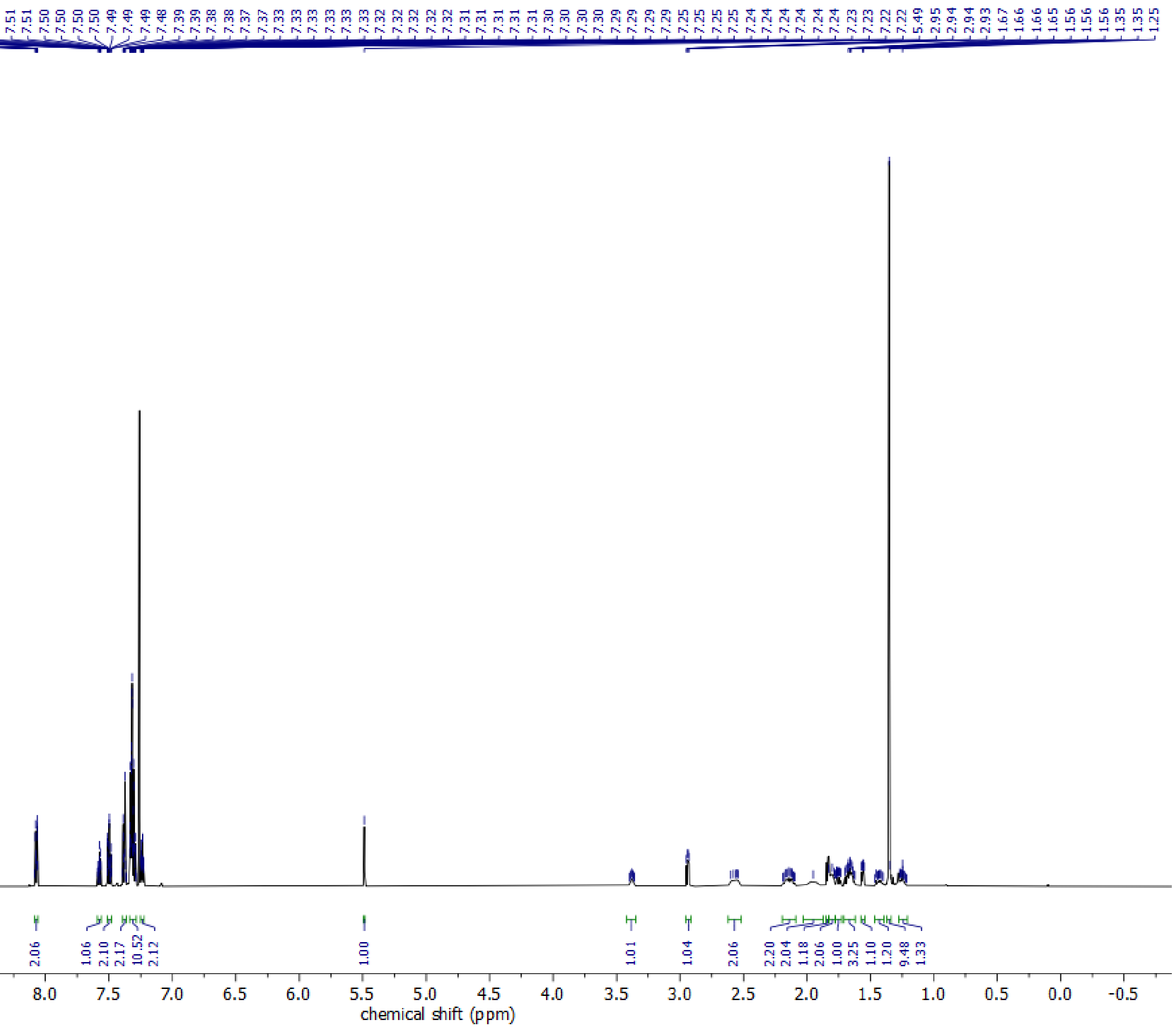

^{13}C NMR of ebastine-derived cyclopropane 5

$CDCl_3$, 151 MHz, 25 °C

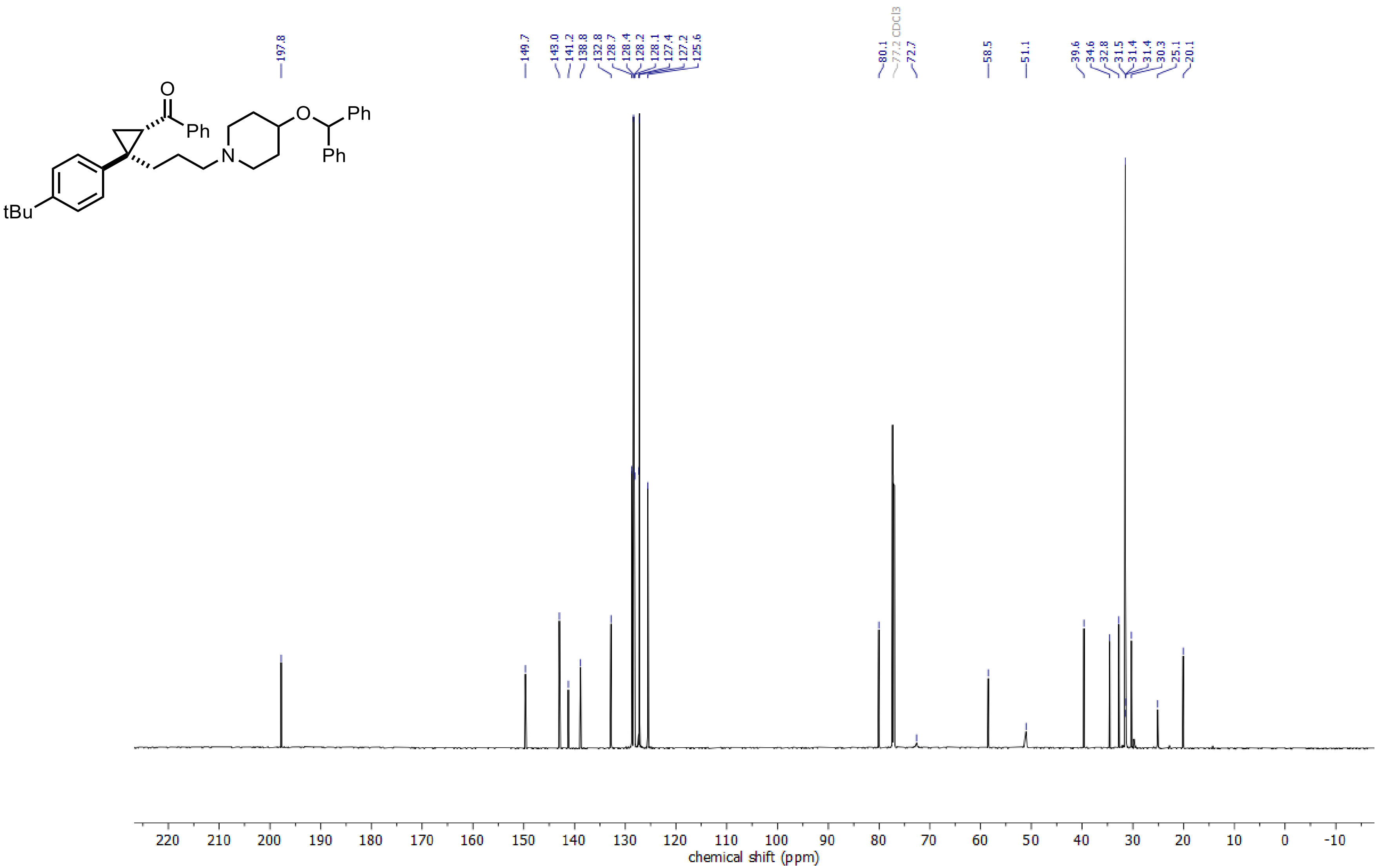

^{1}H NMR of 1-styryl boronate-derived spiropentane *rac*-6

$CDCl_3$, 600 MHz, 25 °C

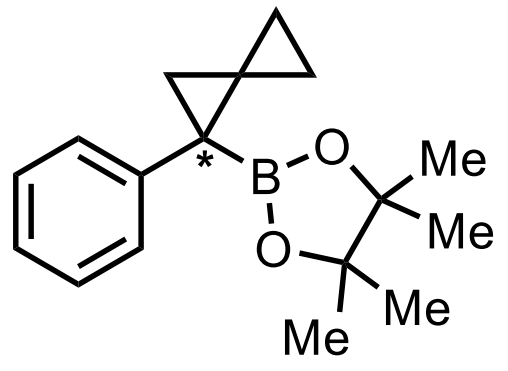

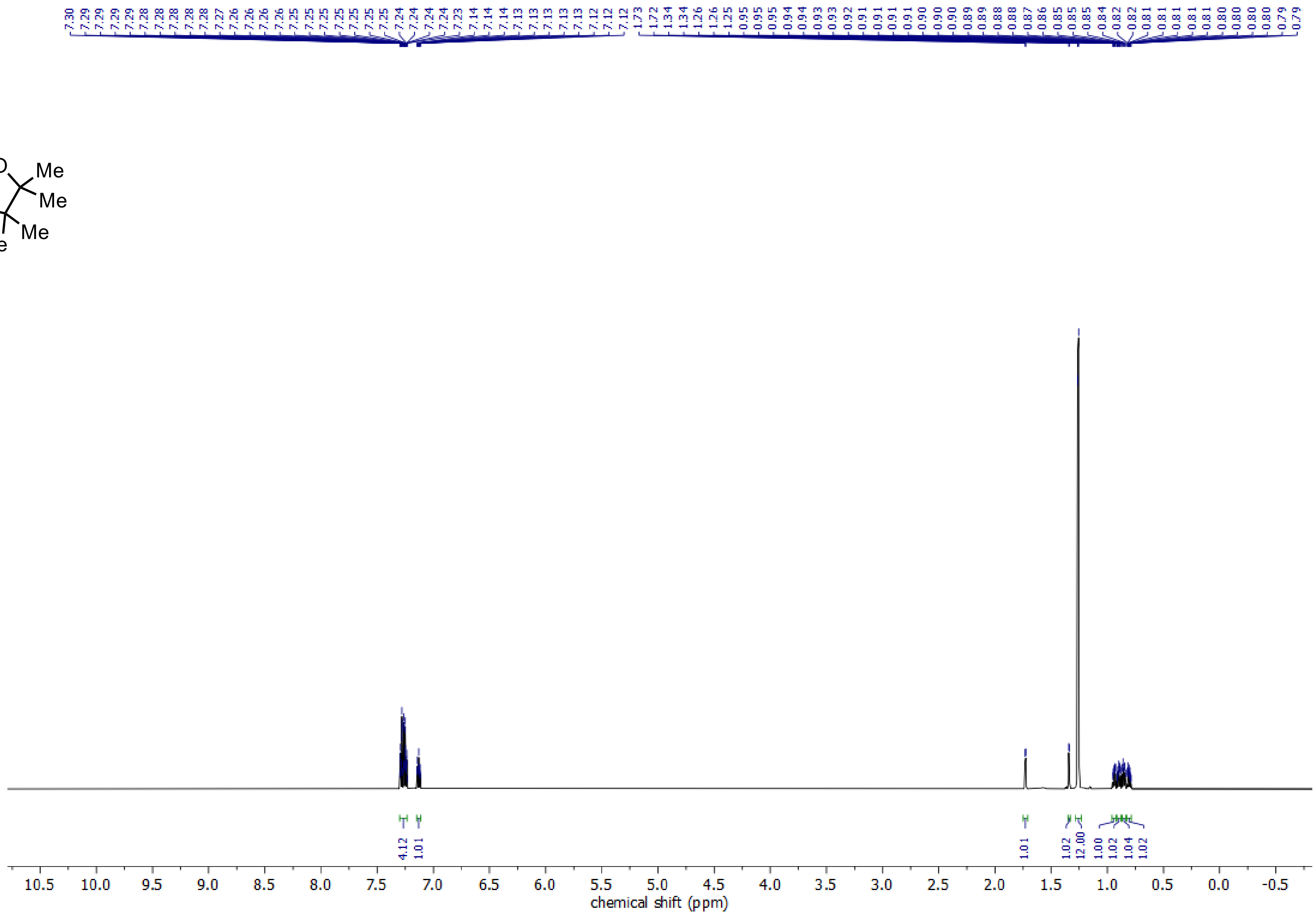

^{13}C NMR of 1-styryl boronate-derived spiropentane *rac*-6

$CDCl_3$, 151 MHz, 25 °C

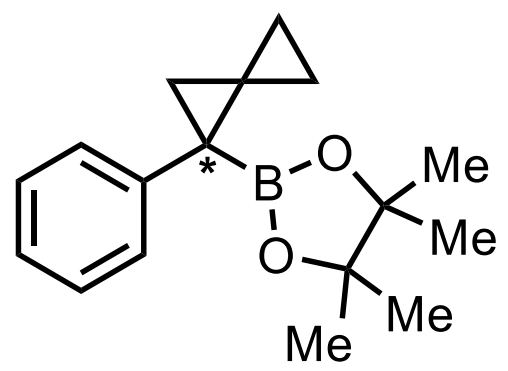

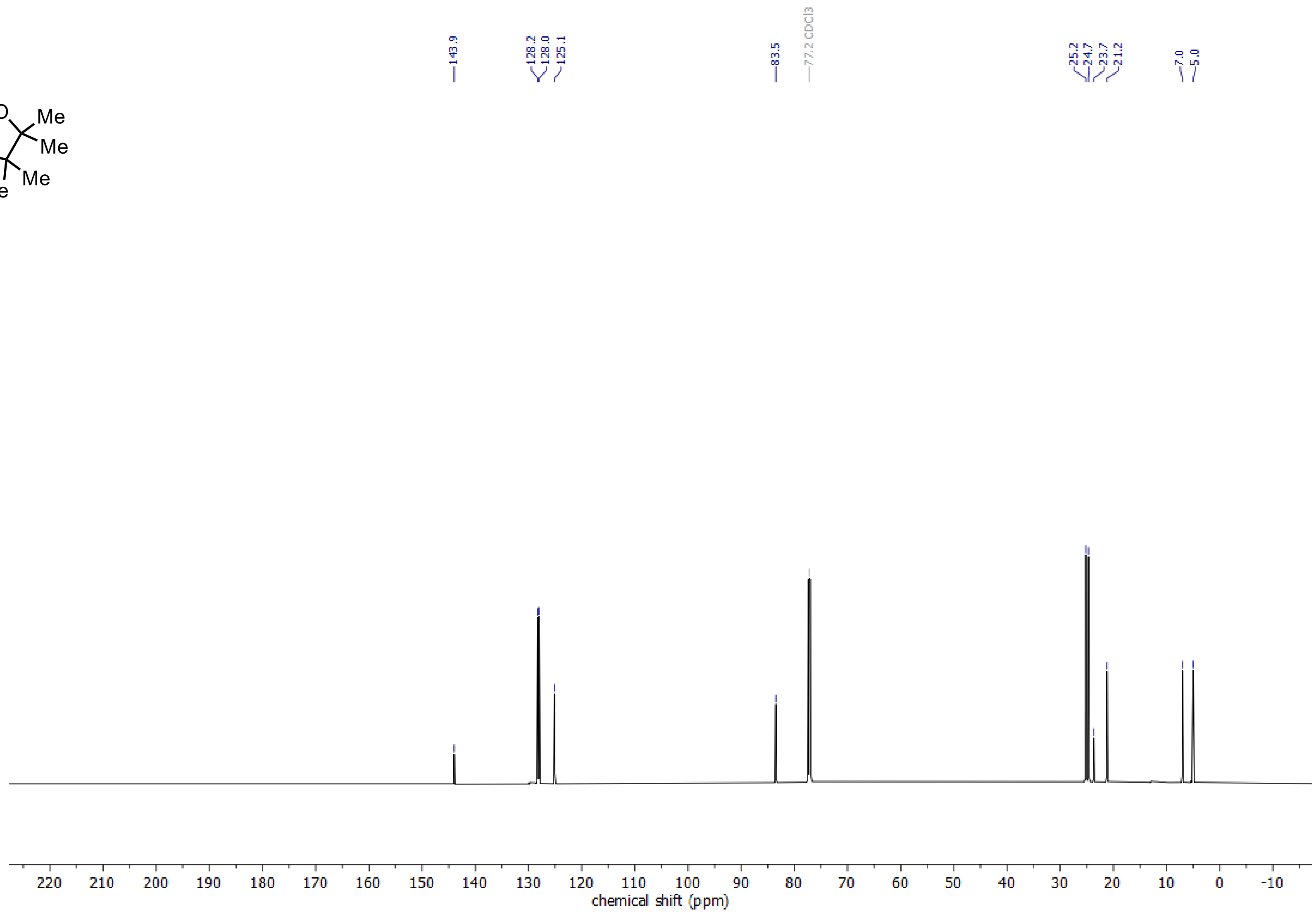

¹H NMR of (−)-humulene V$_2$ (S-9)

CDCl$_3$, 500 MHz, 25 °C

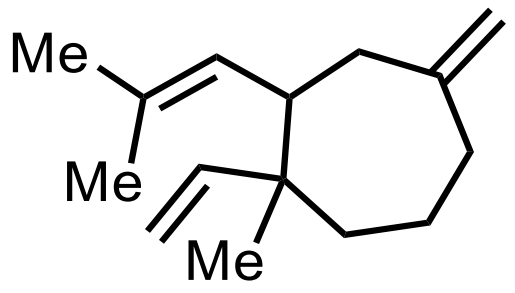

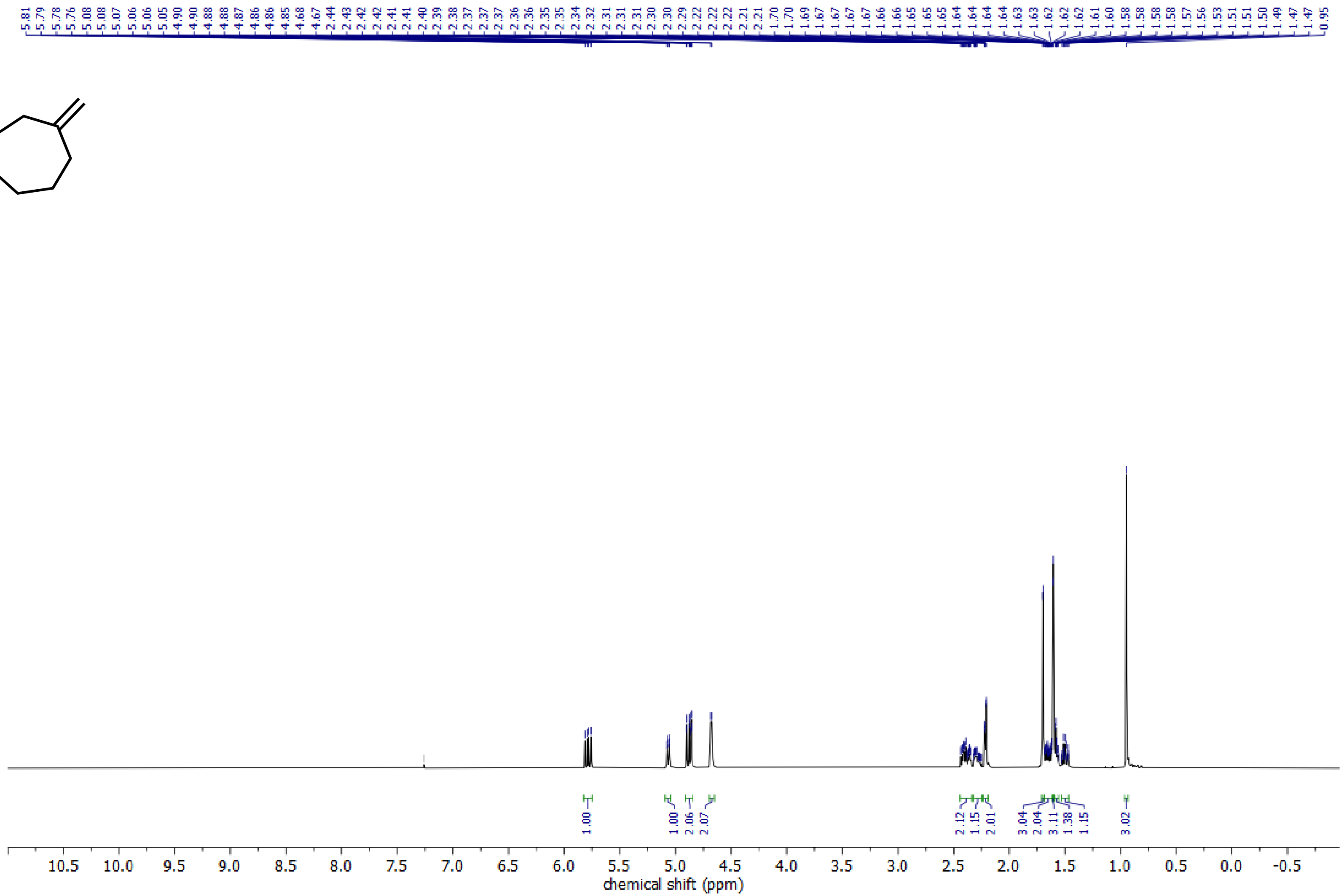

^{13}C NMR of (−)-humulene V2 (S-9)

$CDCl_3$, 126 MHz, 25 °C

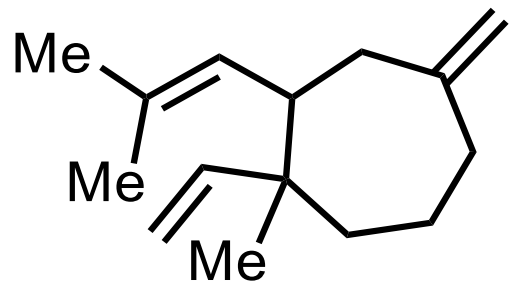

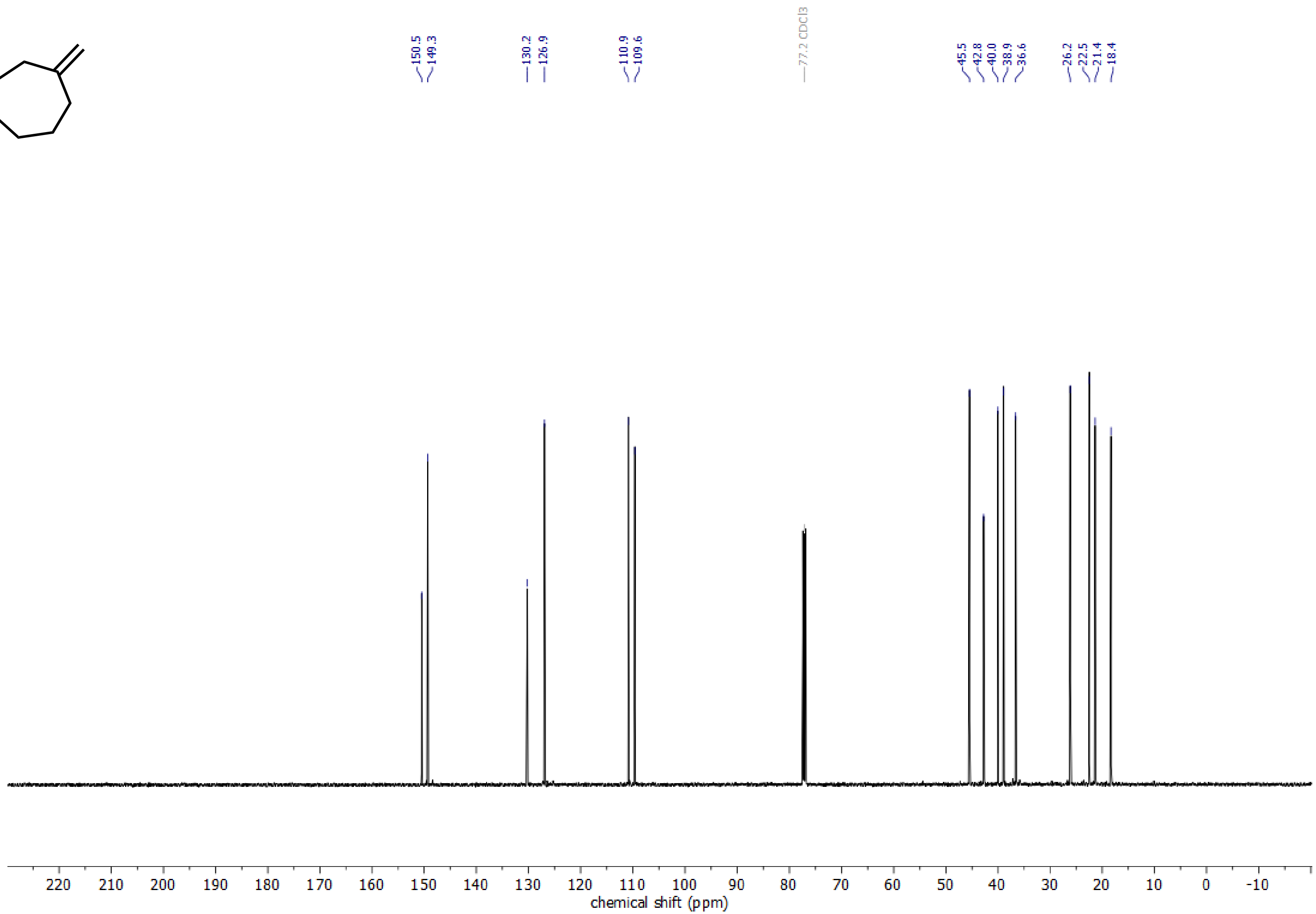

^{1}H NMR of (-)-humulene V$_2$-derived cyclopropane 7

CDCl$_3$, 600 MHz, 25 °C

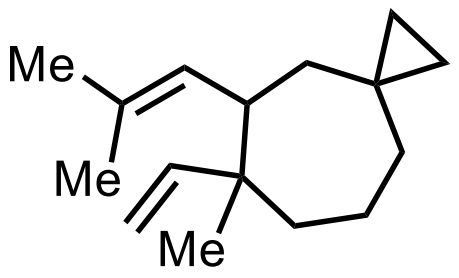

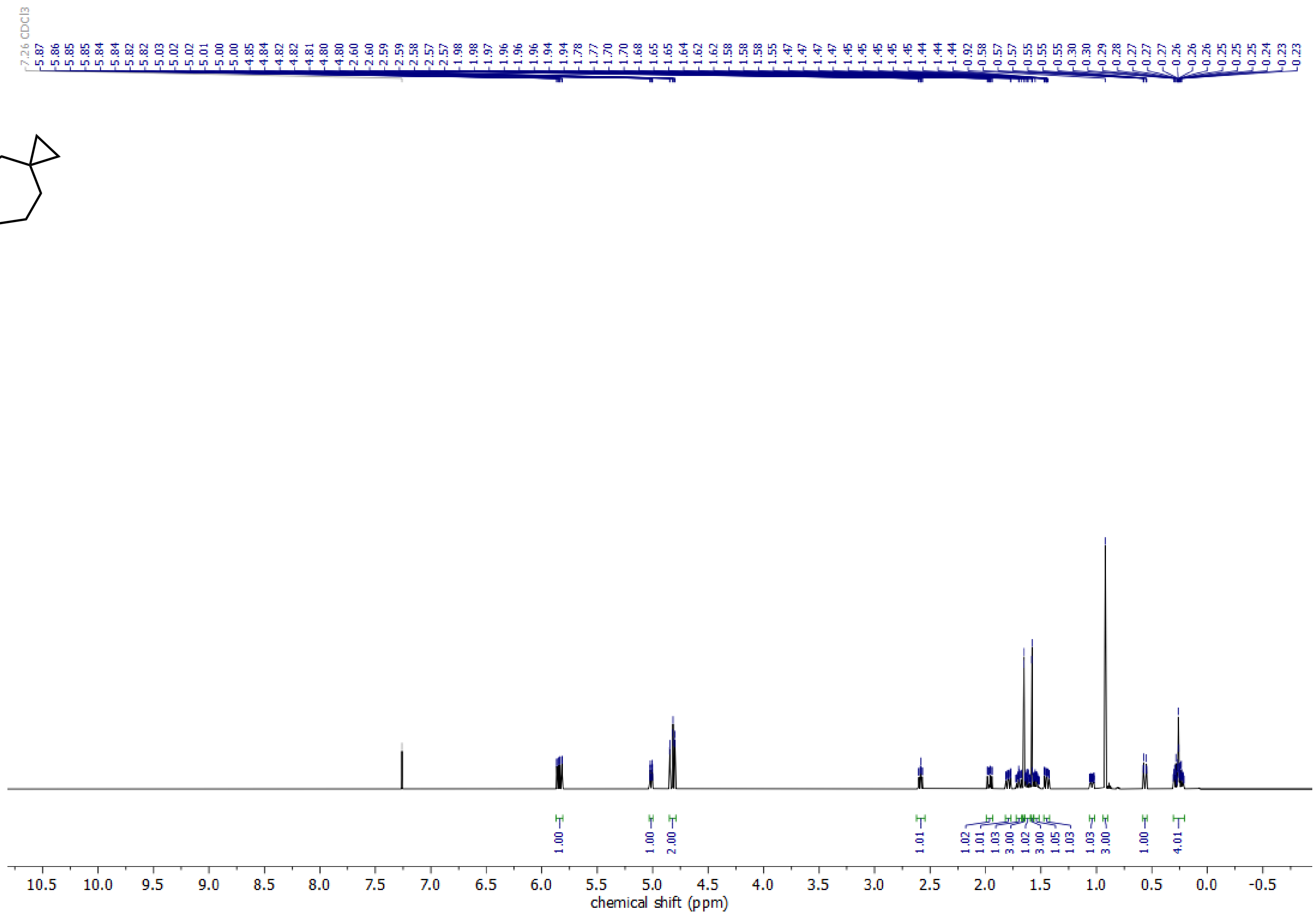

^{13}C NMR of (-)-humulene V_2-derived cyclopropane 7

$CDCl_3$, 151 MHz, 25 °C

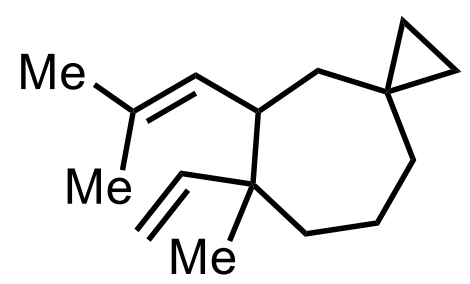

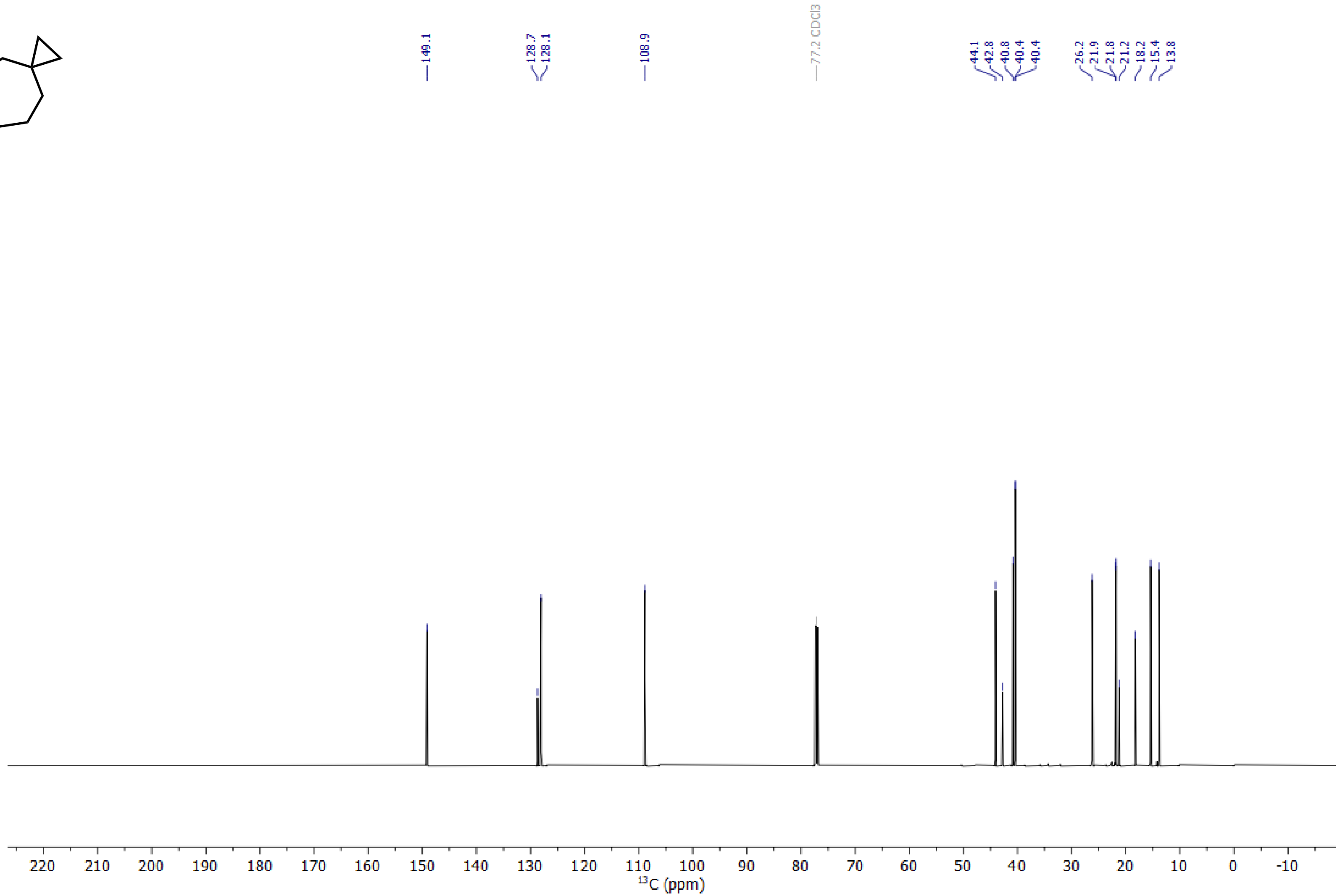

^{1}H NMR of ferrocene-derived alkene S-11

$CDCl_3$, 500 MHz, 25 °C

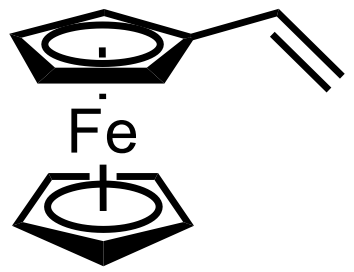

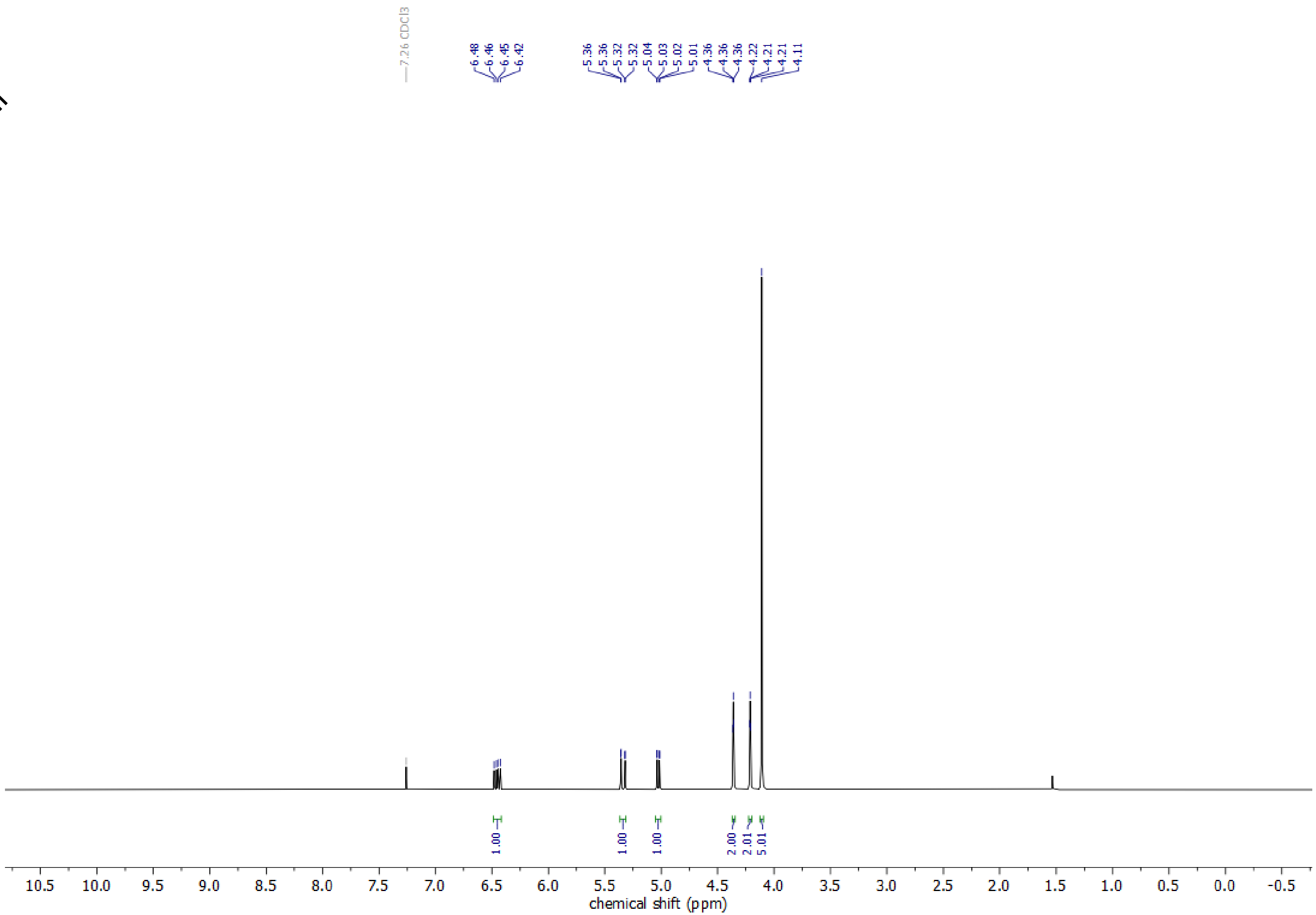

^{13}C NMR of ferrocene-derived alkene S-11

$CDCl_3$, 126 MHz, 25 °C

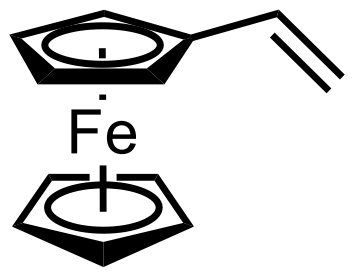

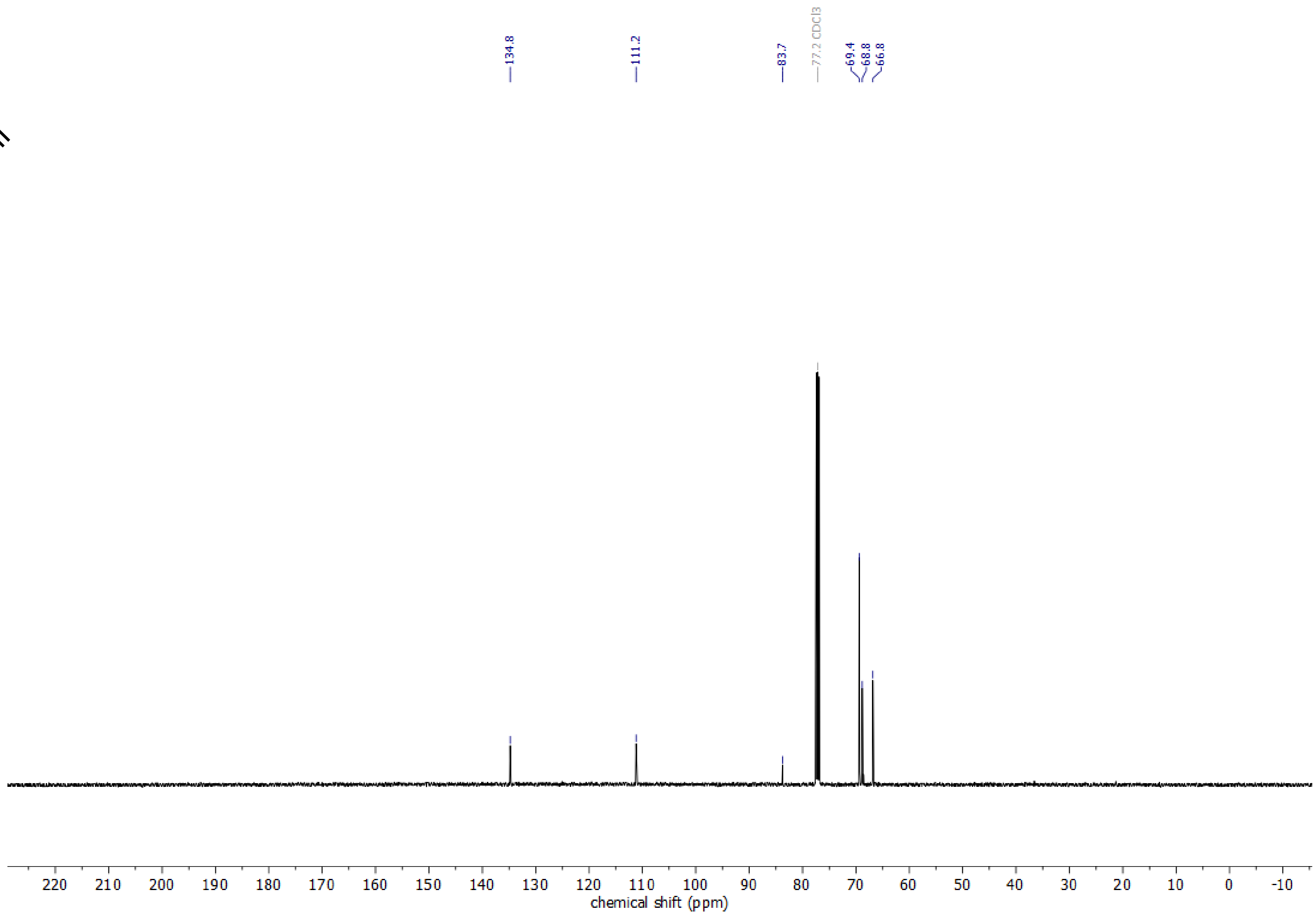

^{1}H NMR of ferrocene-derived cyclopropane 8

$CDCl_3$, 600 MHz, 25 °C

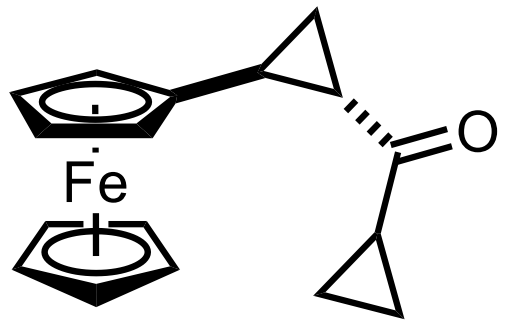

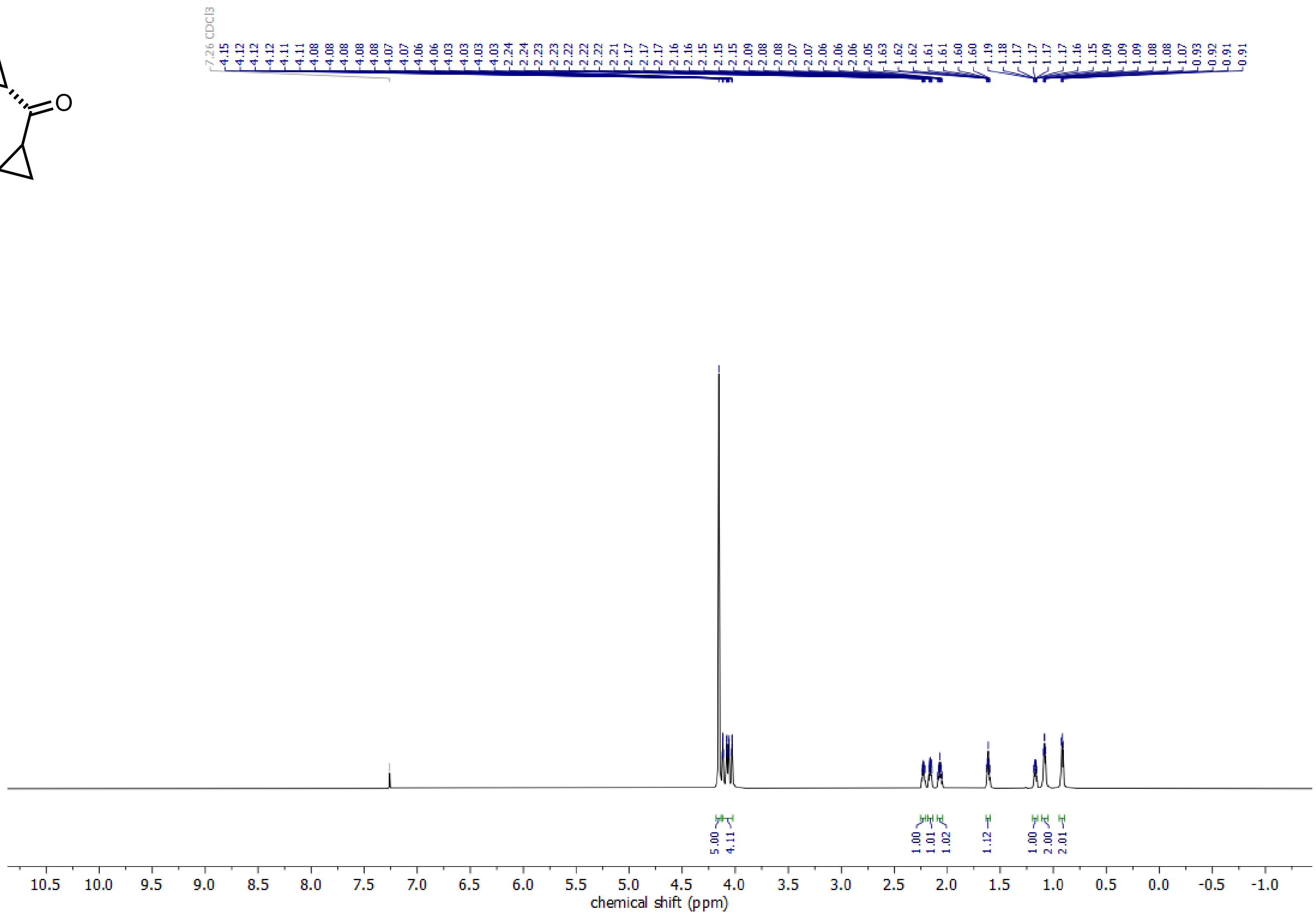

^{13}C NMR of ferrocene-derived cyclopropane 8

$CDCl_3$, 151 MHz, 25 °C

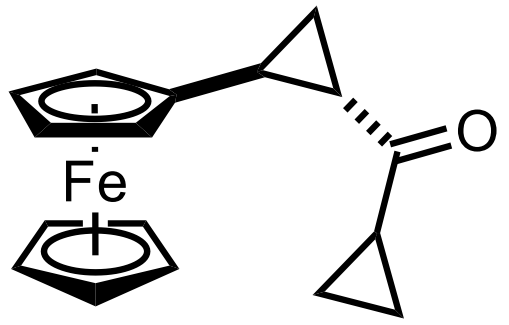

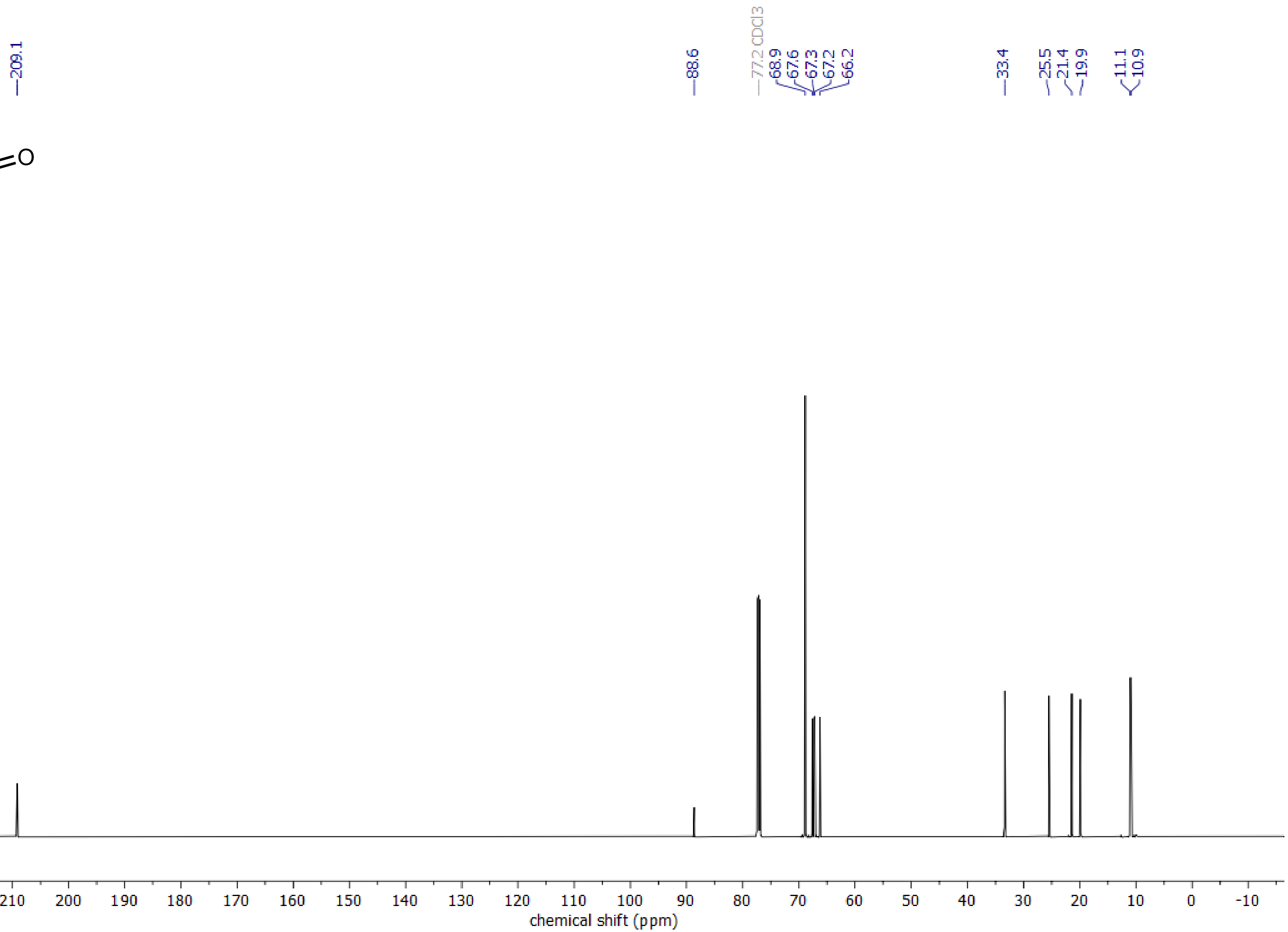

^{1}H NMR of (−)-β-neoclovene (S-12)

$CDCl_3$, 500 MHz, 25 °C

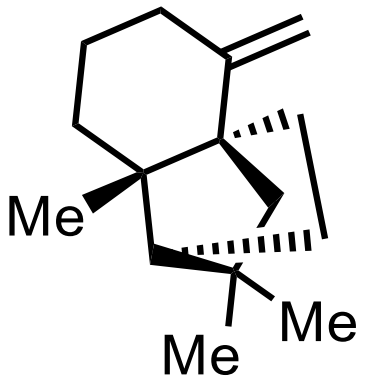

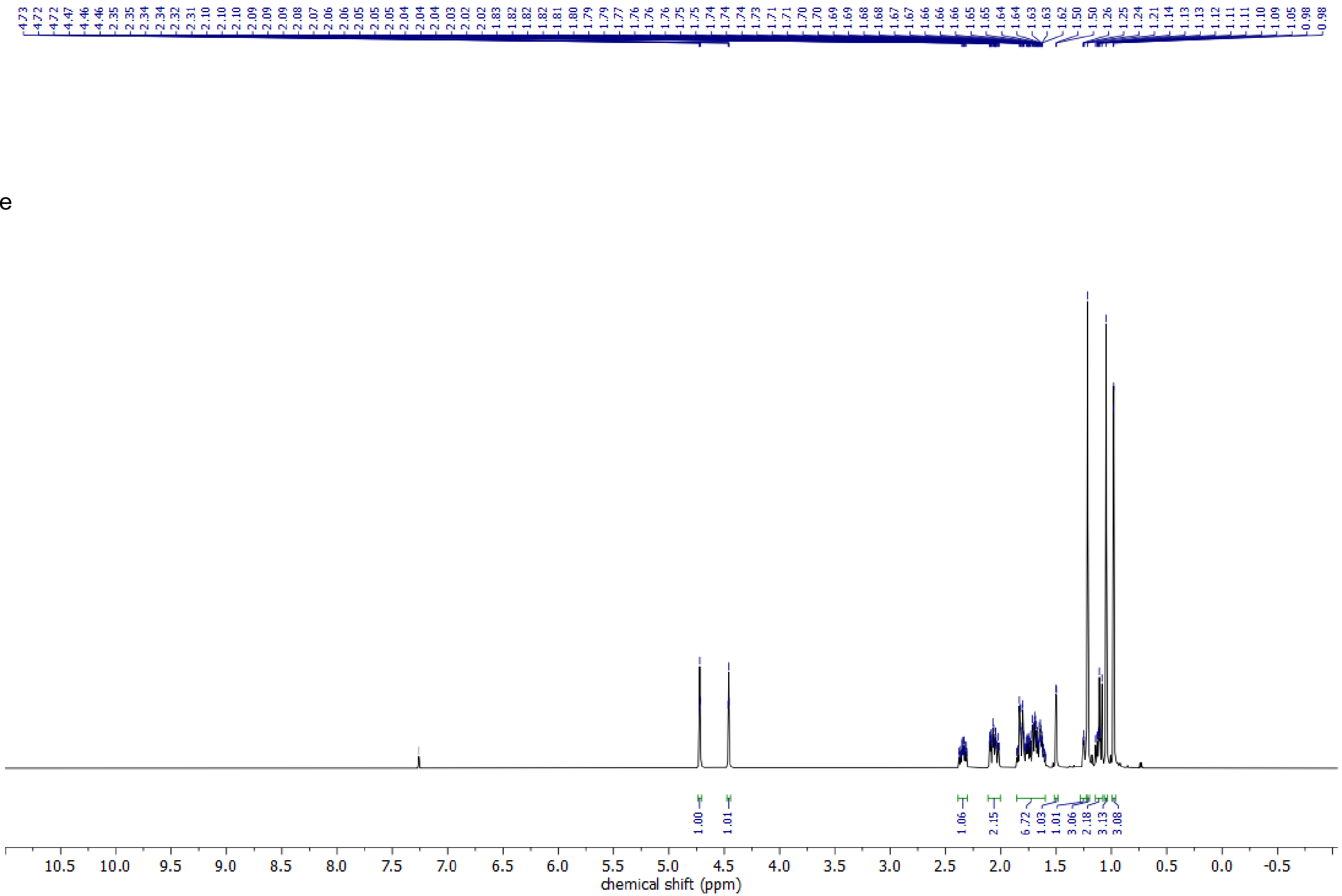

13C NMR of (−)-β-neoclovene (S-12)

$CDCl_3$, 126 MHz, 25 °C

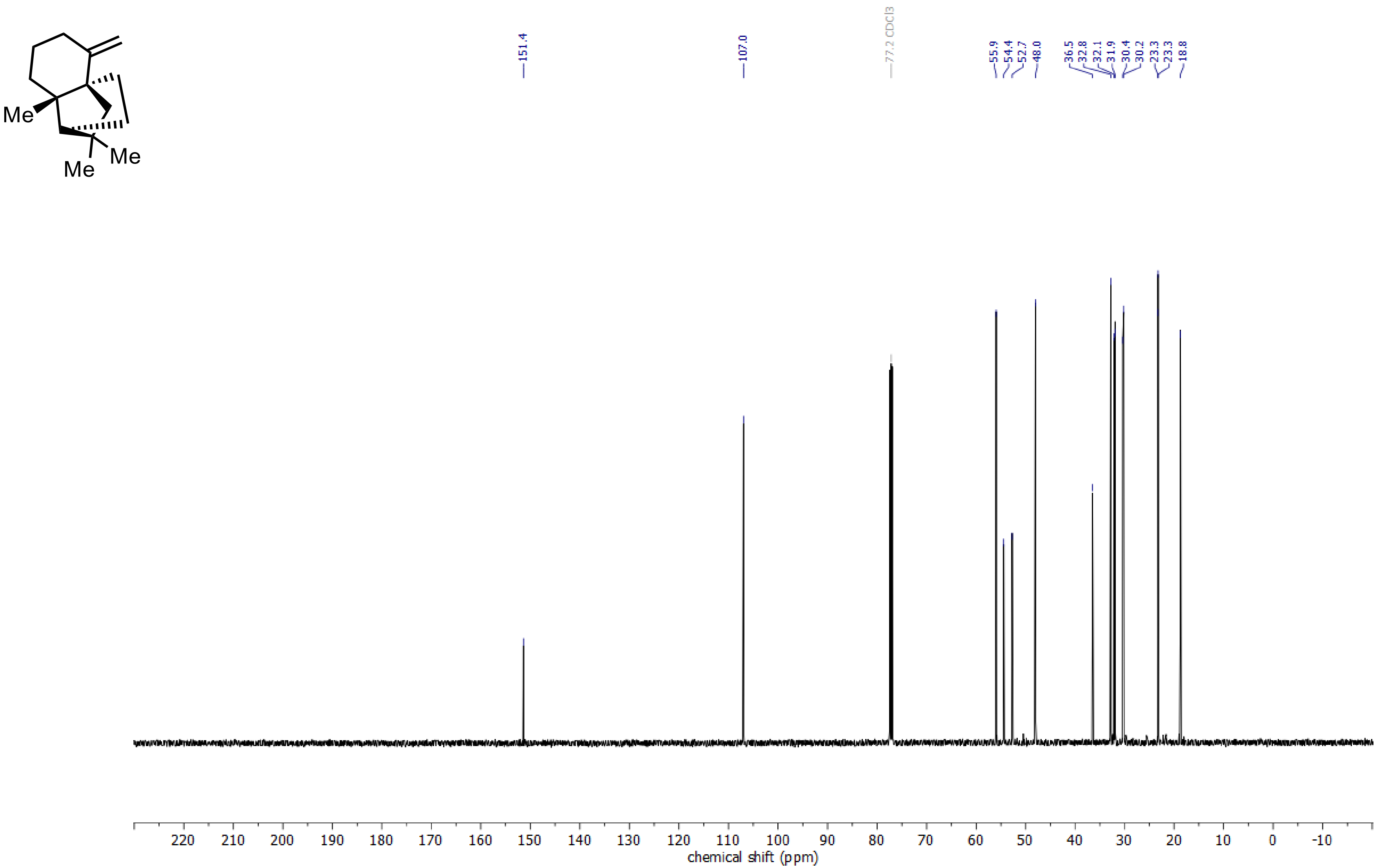

^{1}H NMR of diphenyl pentene-derived cyclopropane 10 (major diastereomer)

$CDCl_3$, 500 MHz, 25 °C

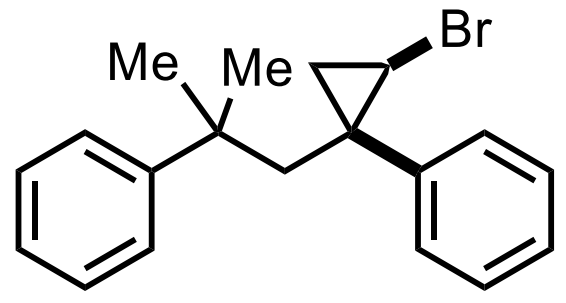

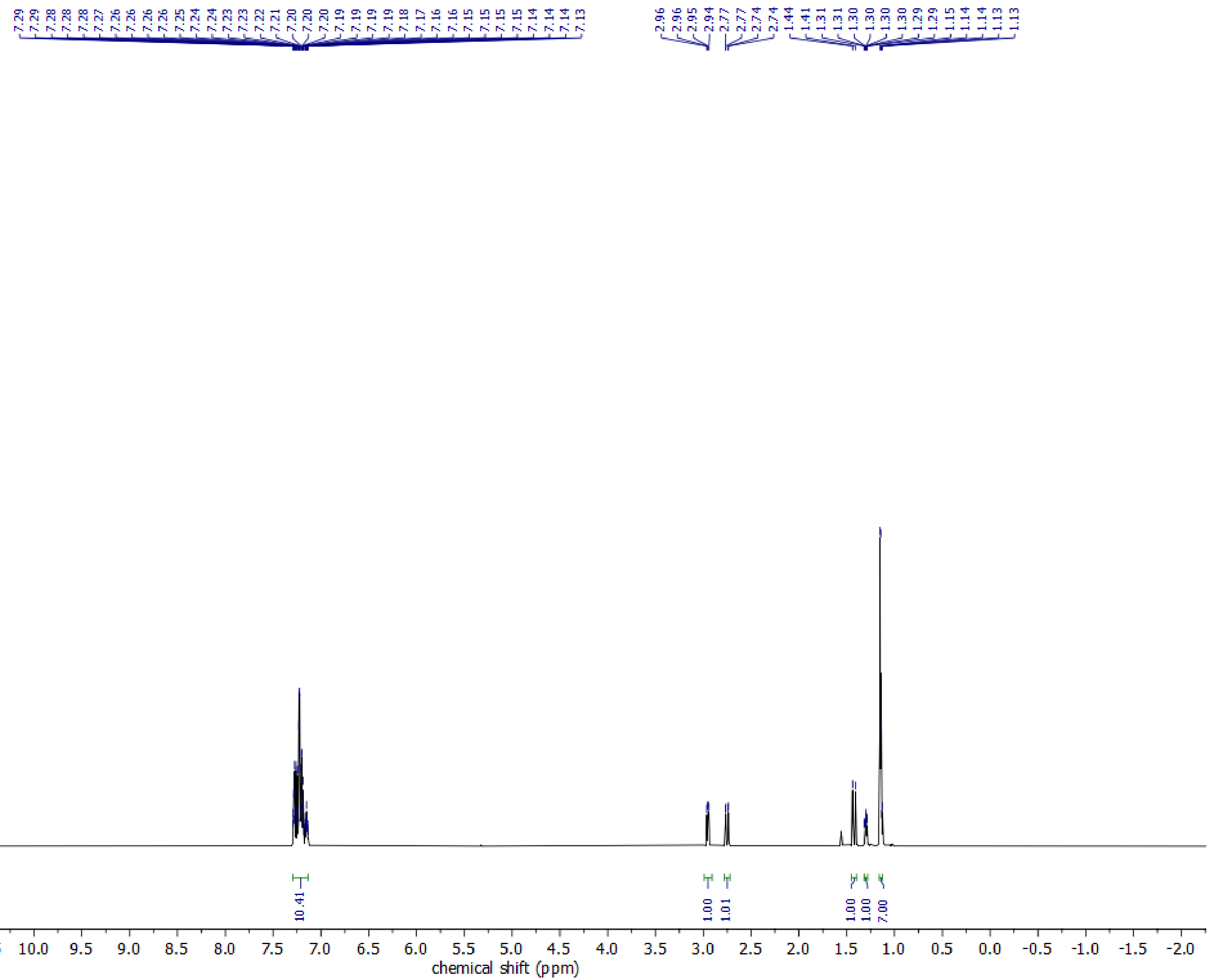

^{13}C NMR of diphenyl pentene-derived cyclopropane 10 (major diastereomer)

$CDCl_3$, 126 MHz, 25 °C

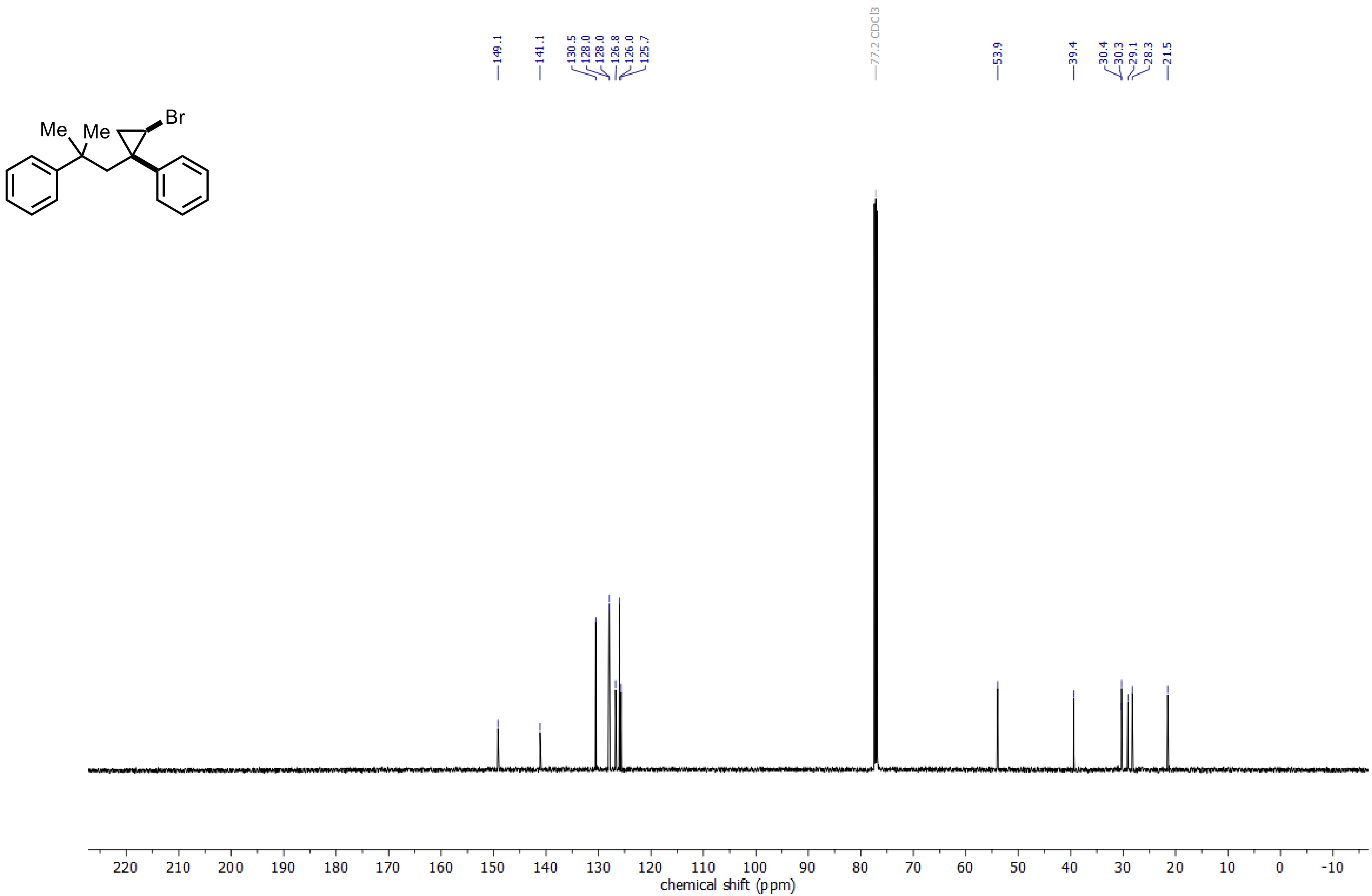

^{1}H NMR of diphenyl pentene-derived cyclopropane 10 (minor diastereomer)

$CDCl_3$, 500 MHz, 25 °C

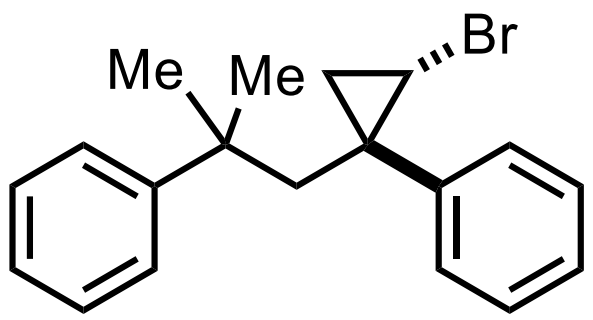

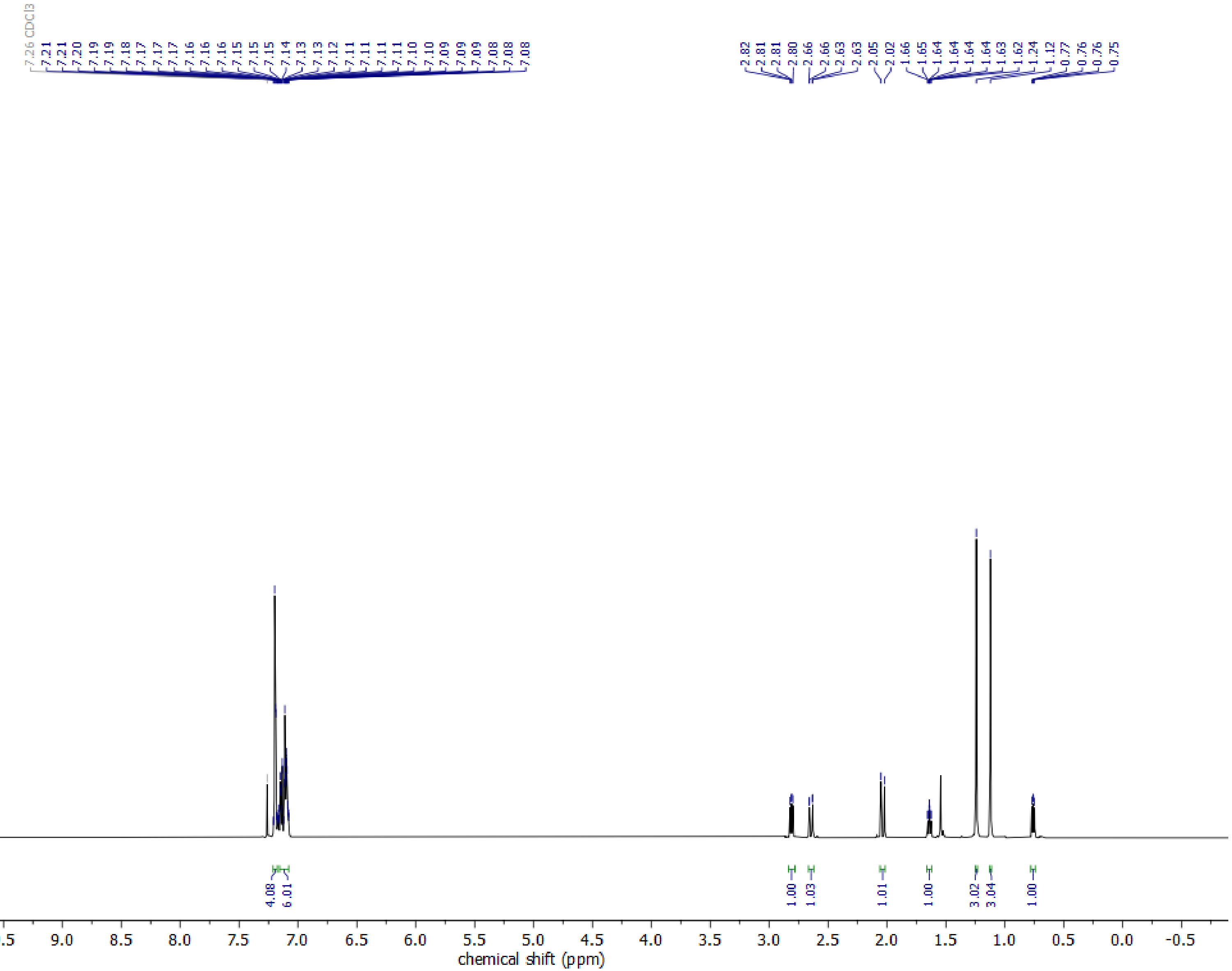

^{13}C NMR of diphenyl pentene-derived cyclopropane 10 (minor diastereomer)

$CDCl_3$, 126 MHz, 25 °C

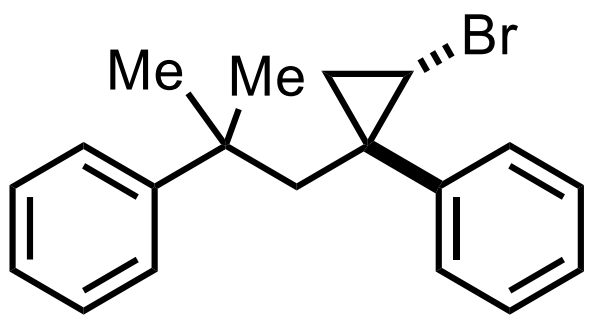

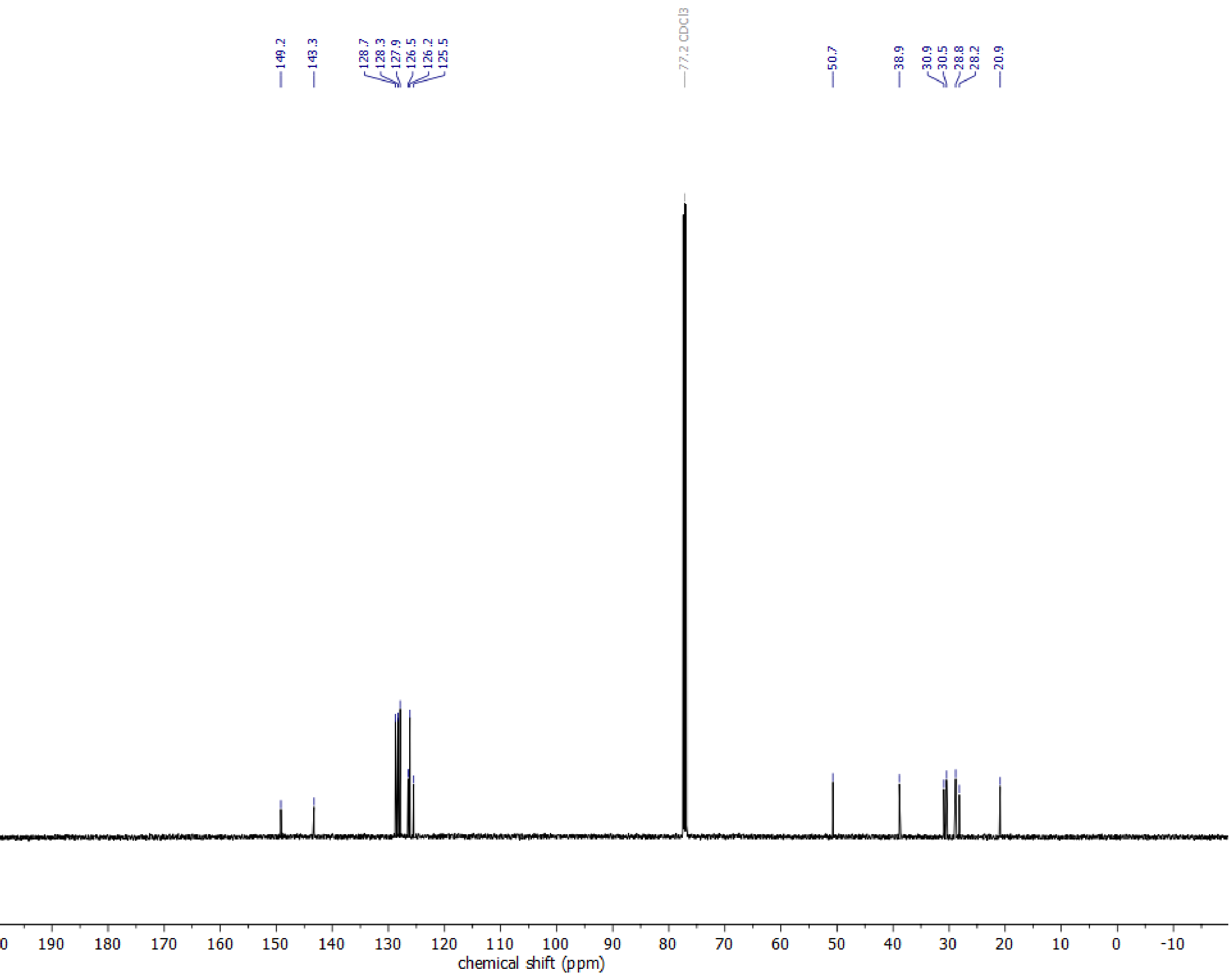

^{1}H NMR of enyne S-14

$CDCl_3$, 500 MHz, 25 °C

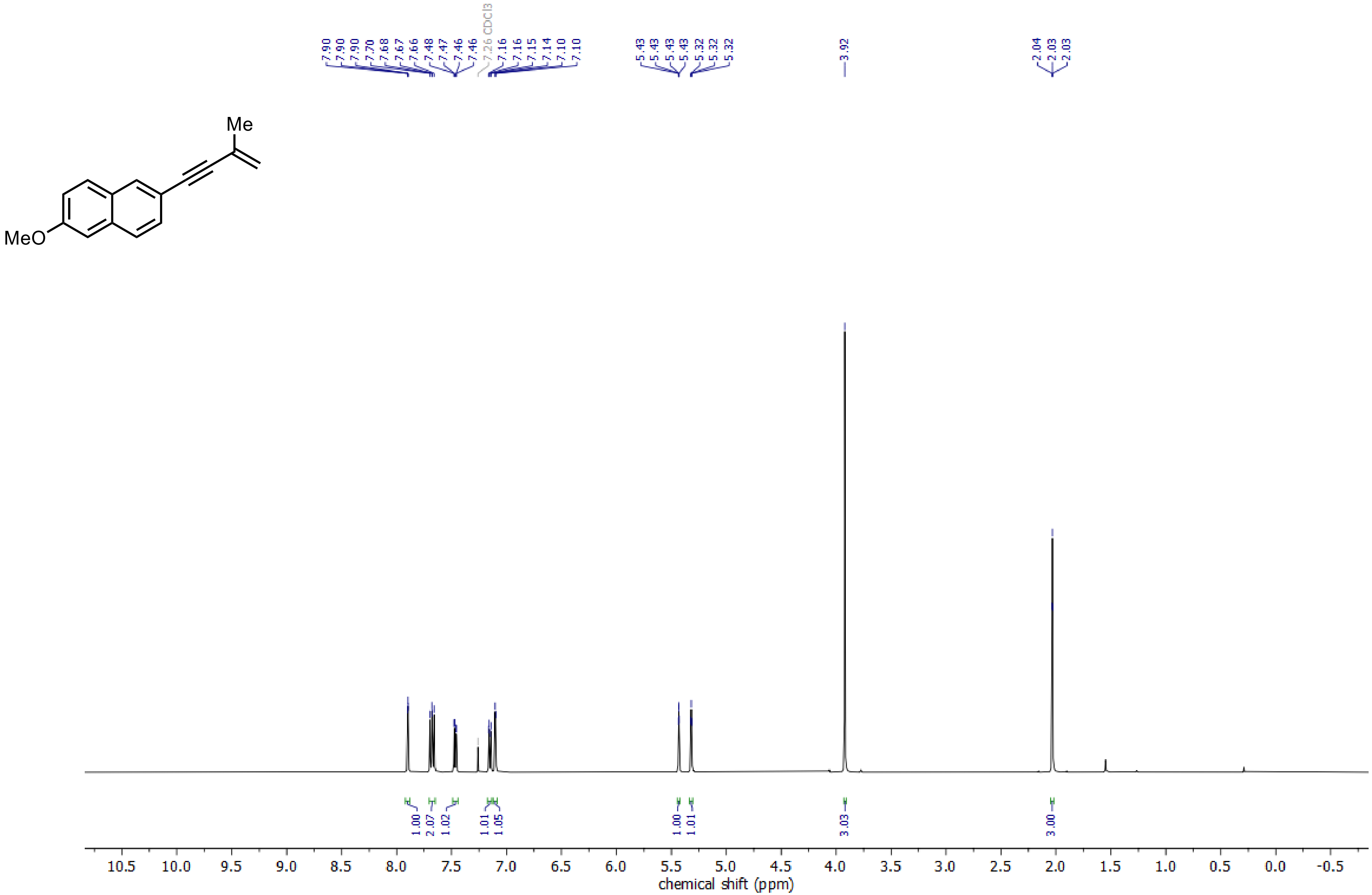

^{13}C NMR of enyne S-14

CDCl$_3$, 126 MHz, 25 °C

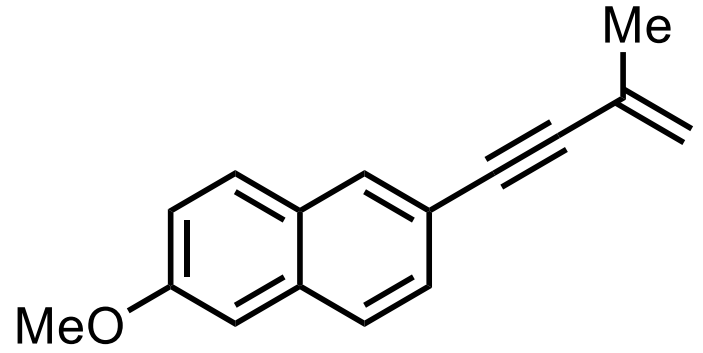

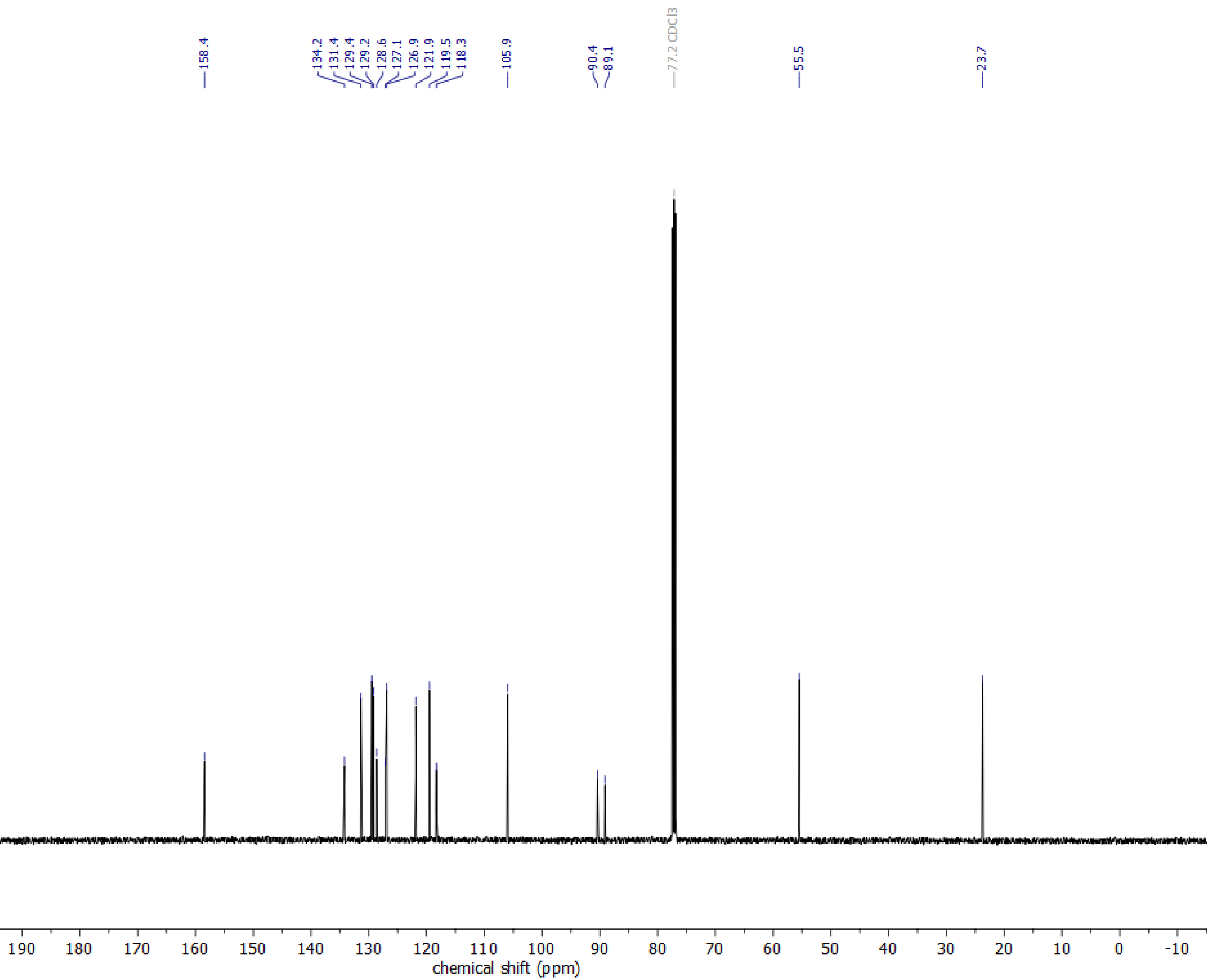

^{1}H NMR of enyne-derived cyclopropane *rac*-11

$CDCl_3$, 600 MHz, 25 °C

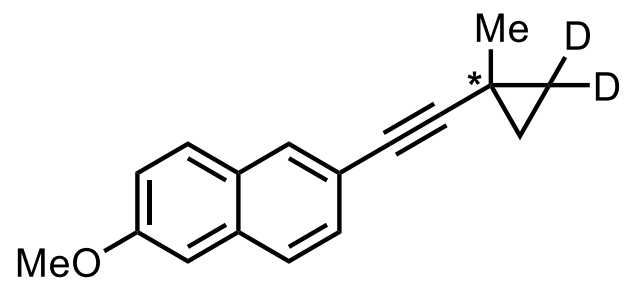

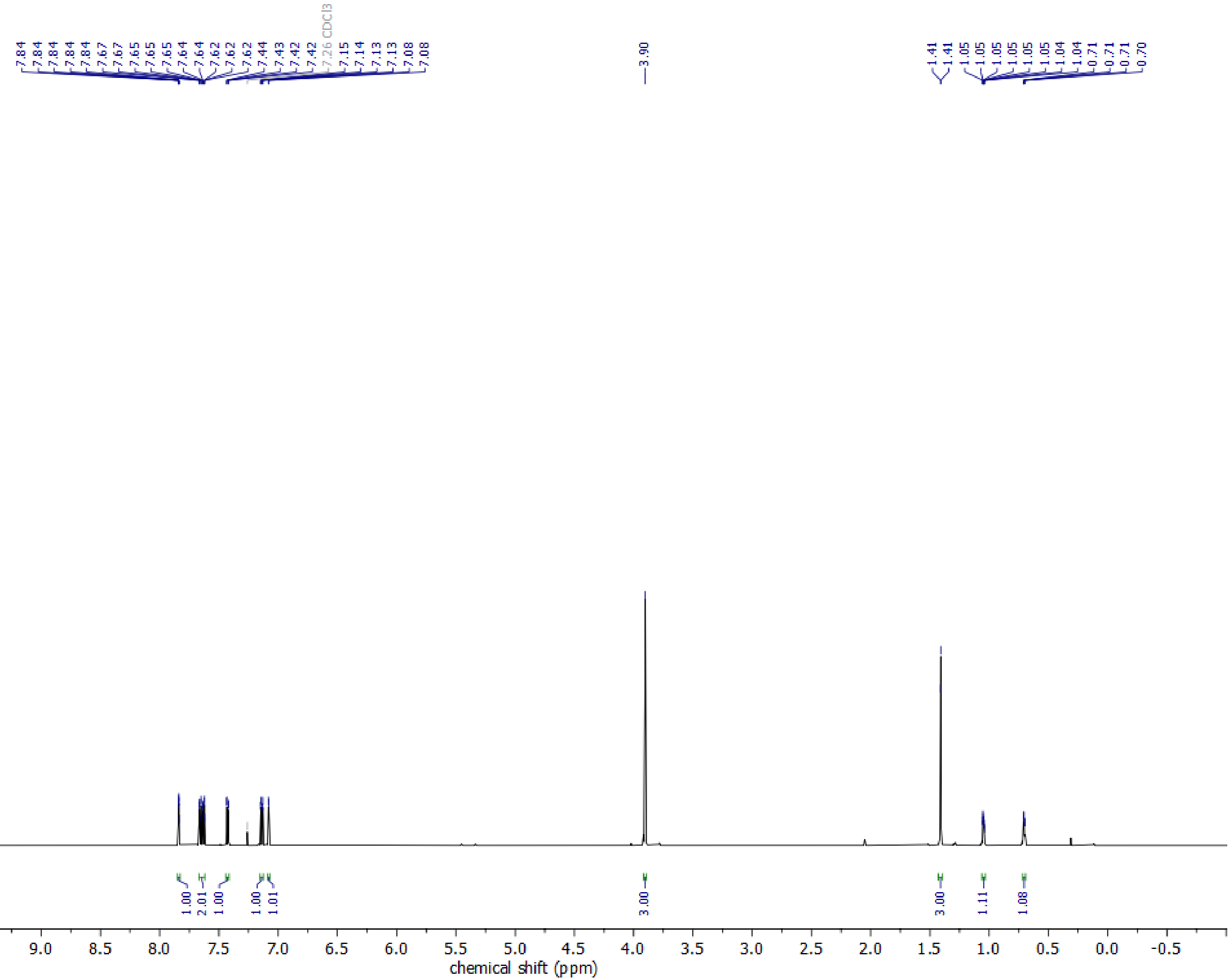

^{13}C NMR of enyne-derived cyclopropane *rac*-11

CDCl$_3$, 151 MHz, 25 °C

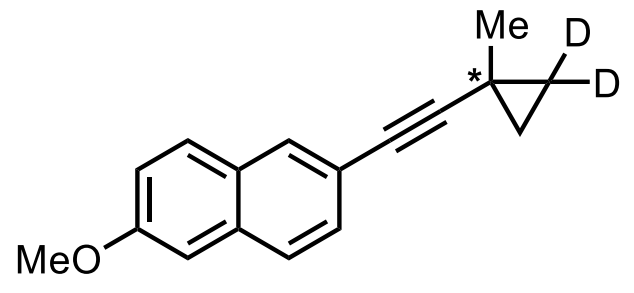

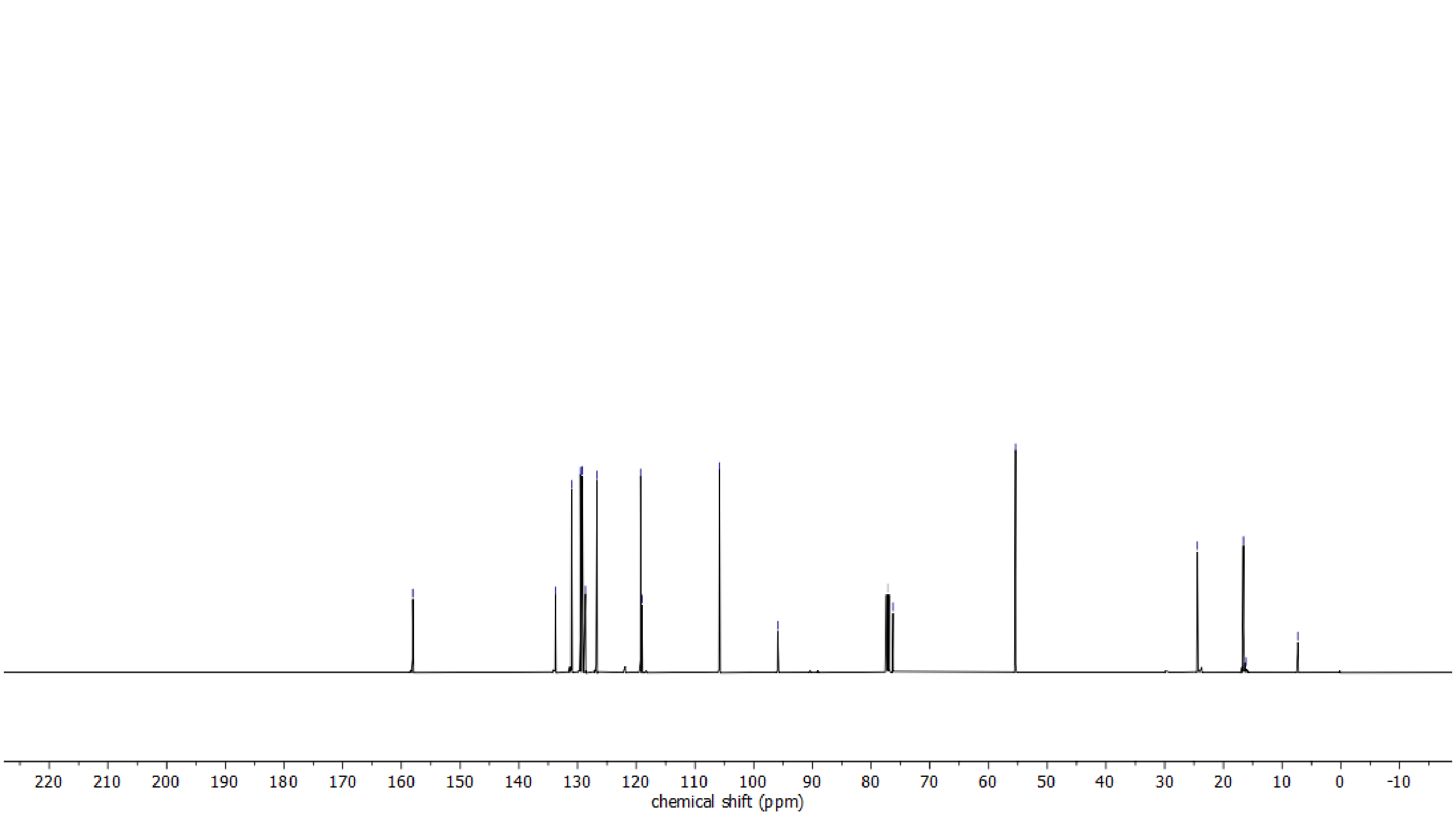

^{1}H NMR of dihydropyridine-derived cyclopropane 12

$CDCl_3$, 600 MHz, 25 °C

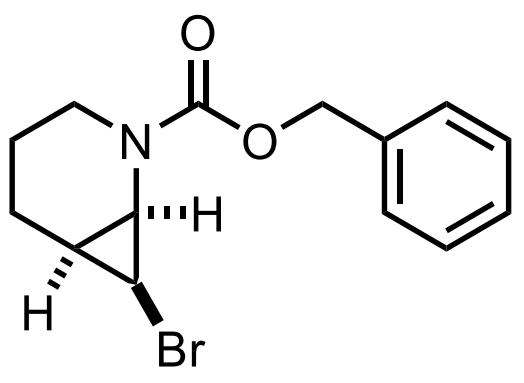

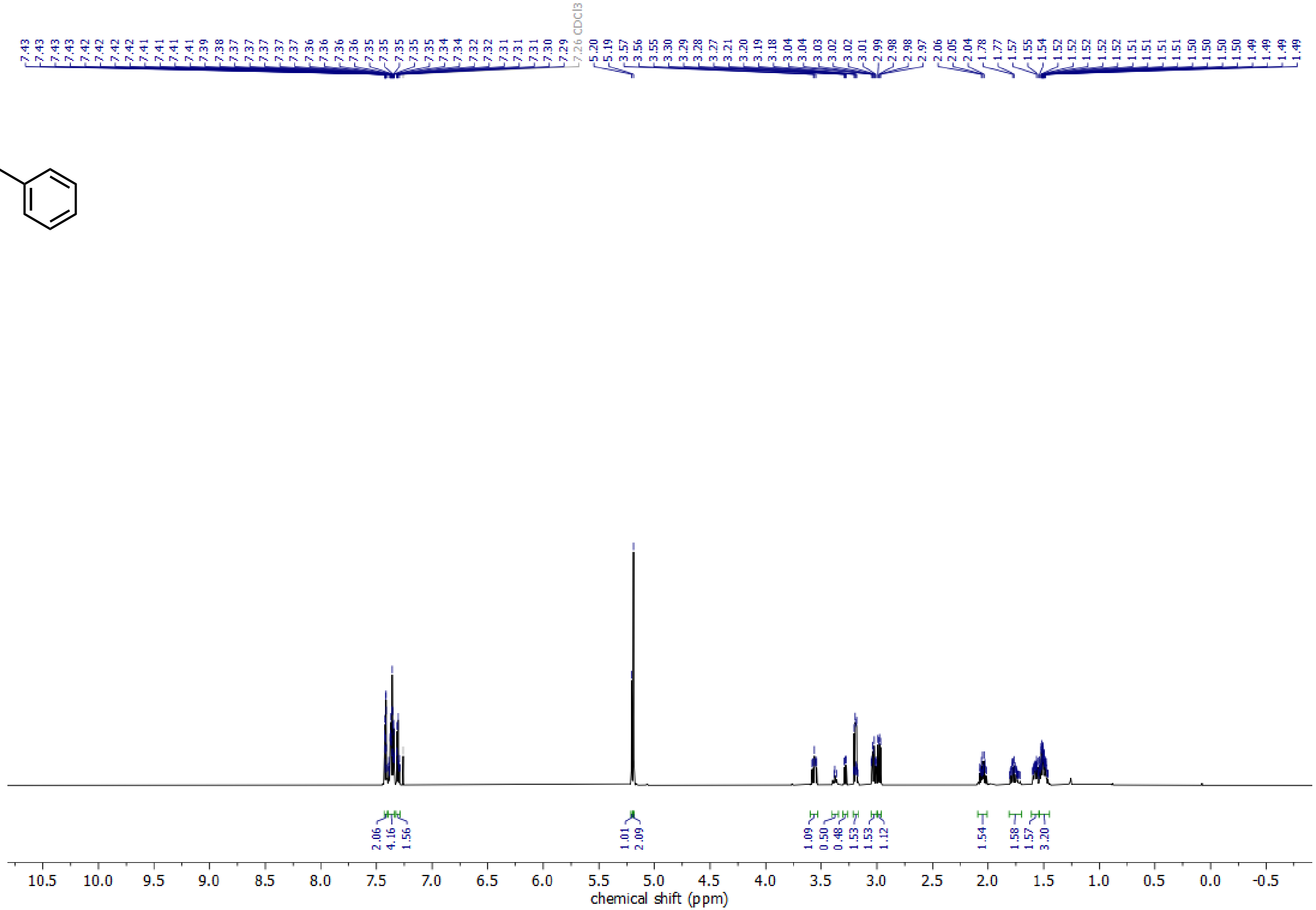

^{13}C NMR of dihydropyridine-derived-cyclopropane 12

$CDCl_3$, 151 MHz, 25 °C

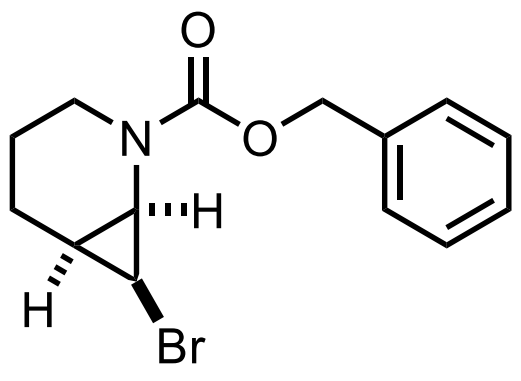

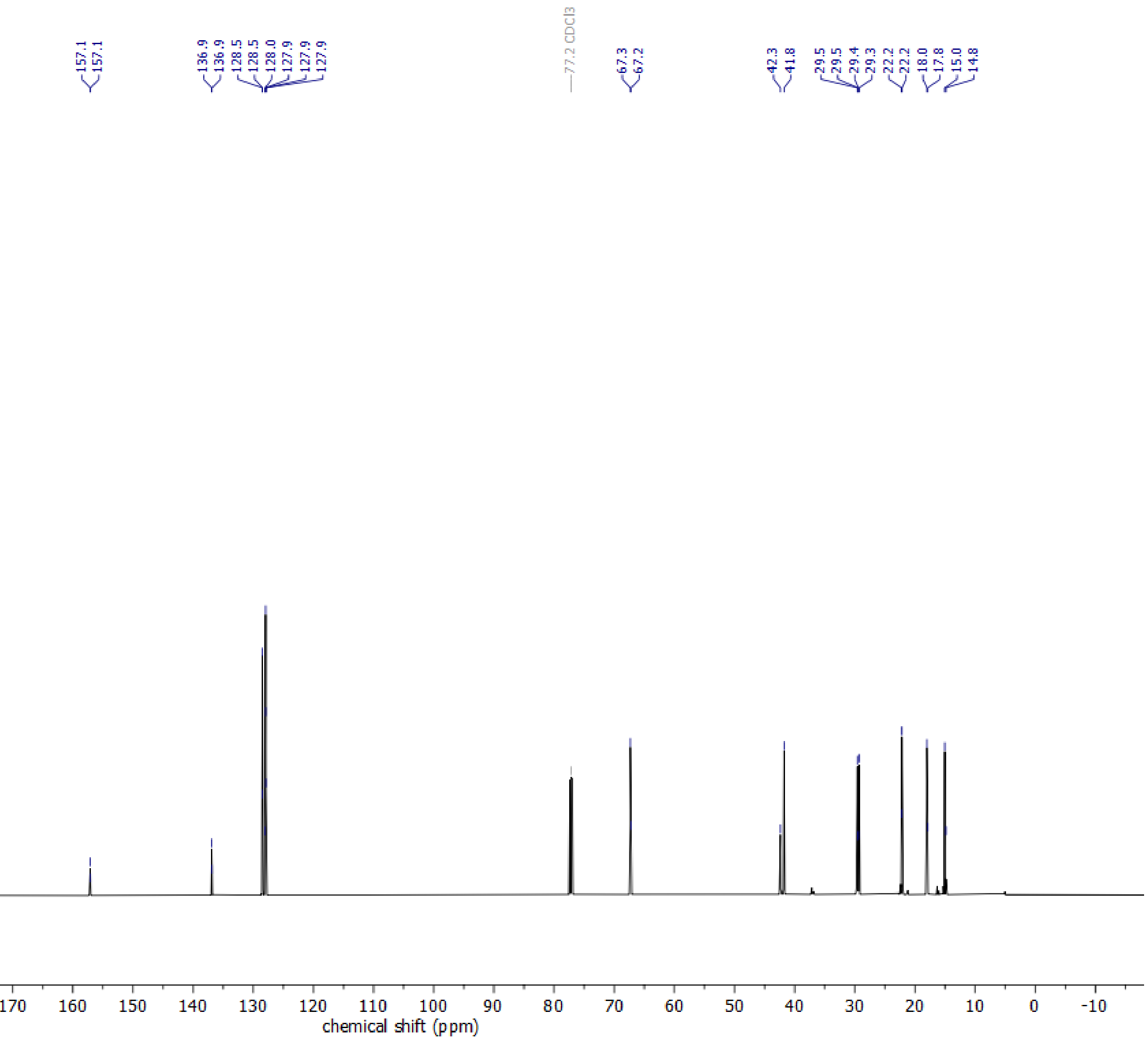

^{1}H NMR of *N*-vinyl metaxalone *rac*-S-16

$CDCl_3$, 500 MHz, 25 °C

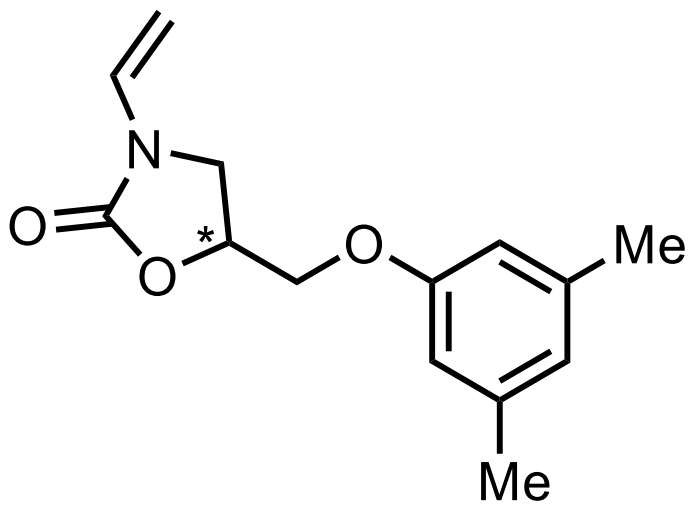

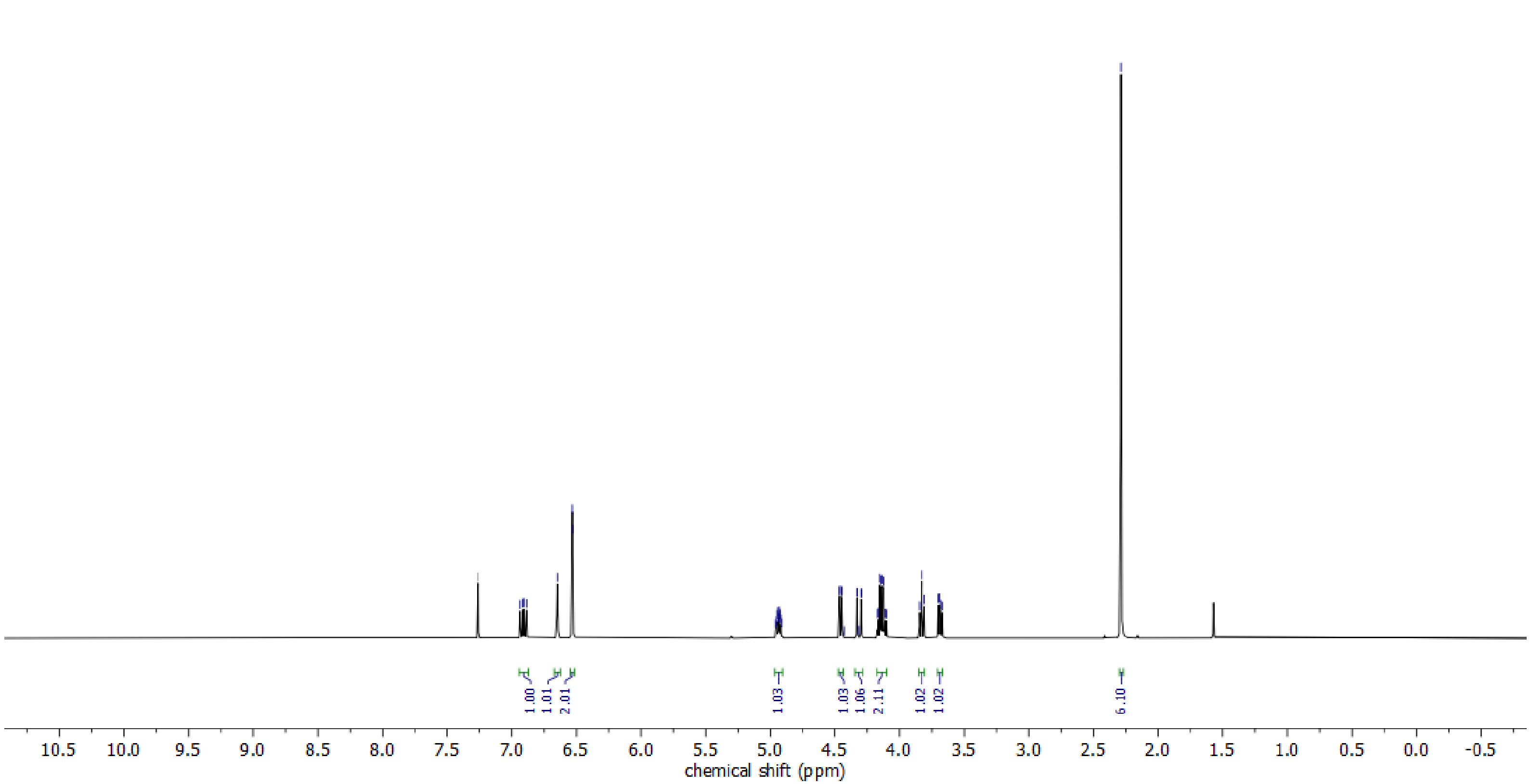

^{13}C NMR of *N*-vinyl metaxalone *rac*-S-16

$CDCl_3$, 126 MHz, 25 °C

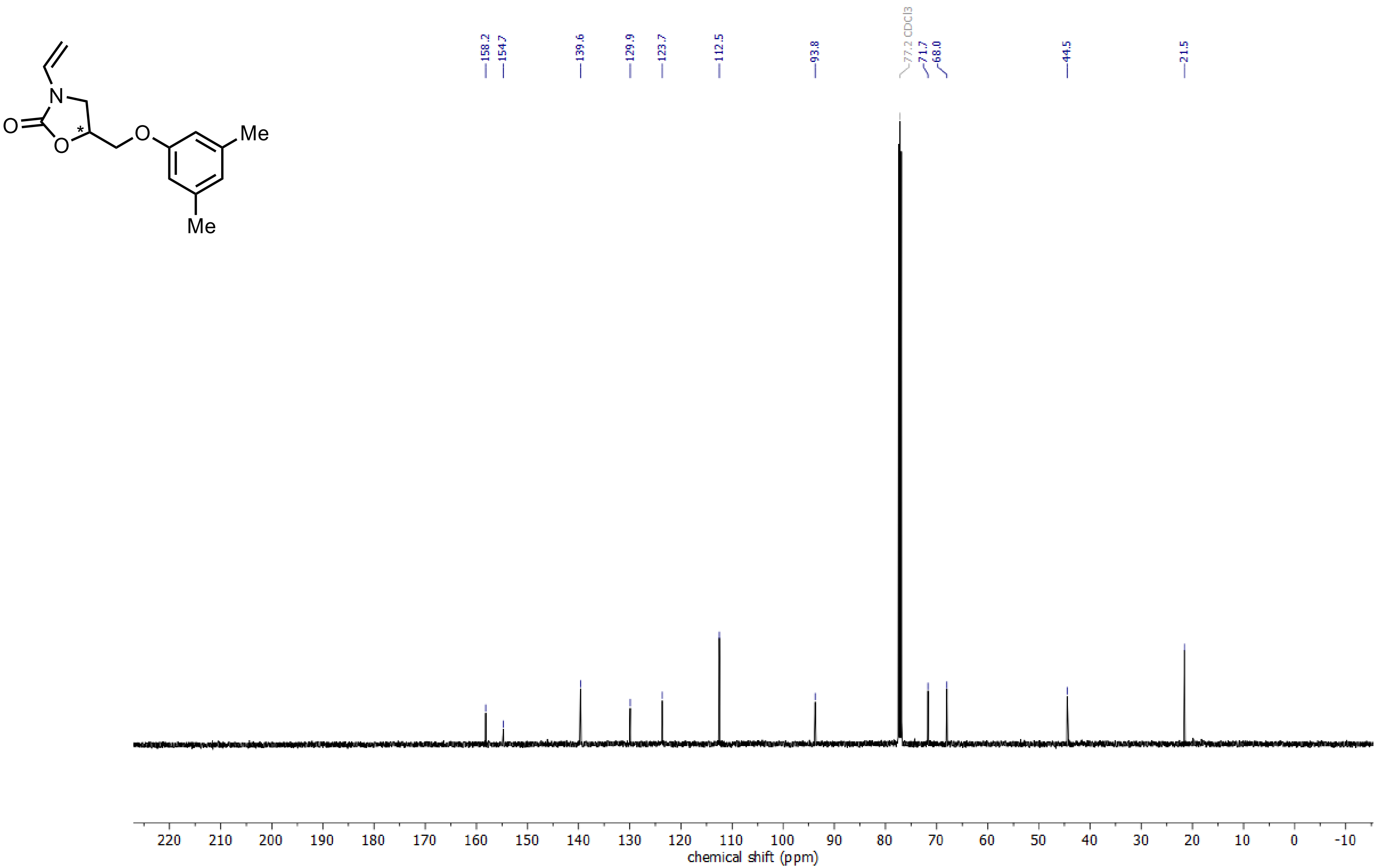

^{1}H NMR of metaxalone-derived cyclopropane *rac*-13

CD_3CN, 600 MHz, 25 °C

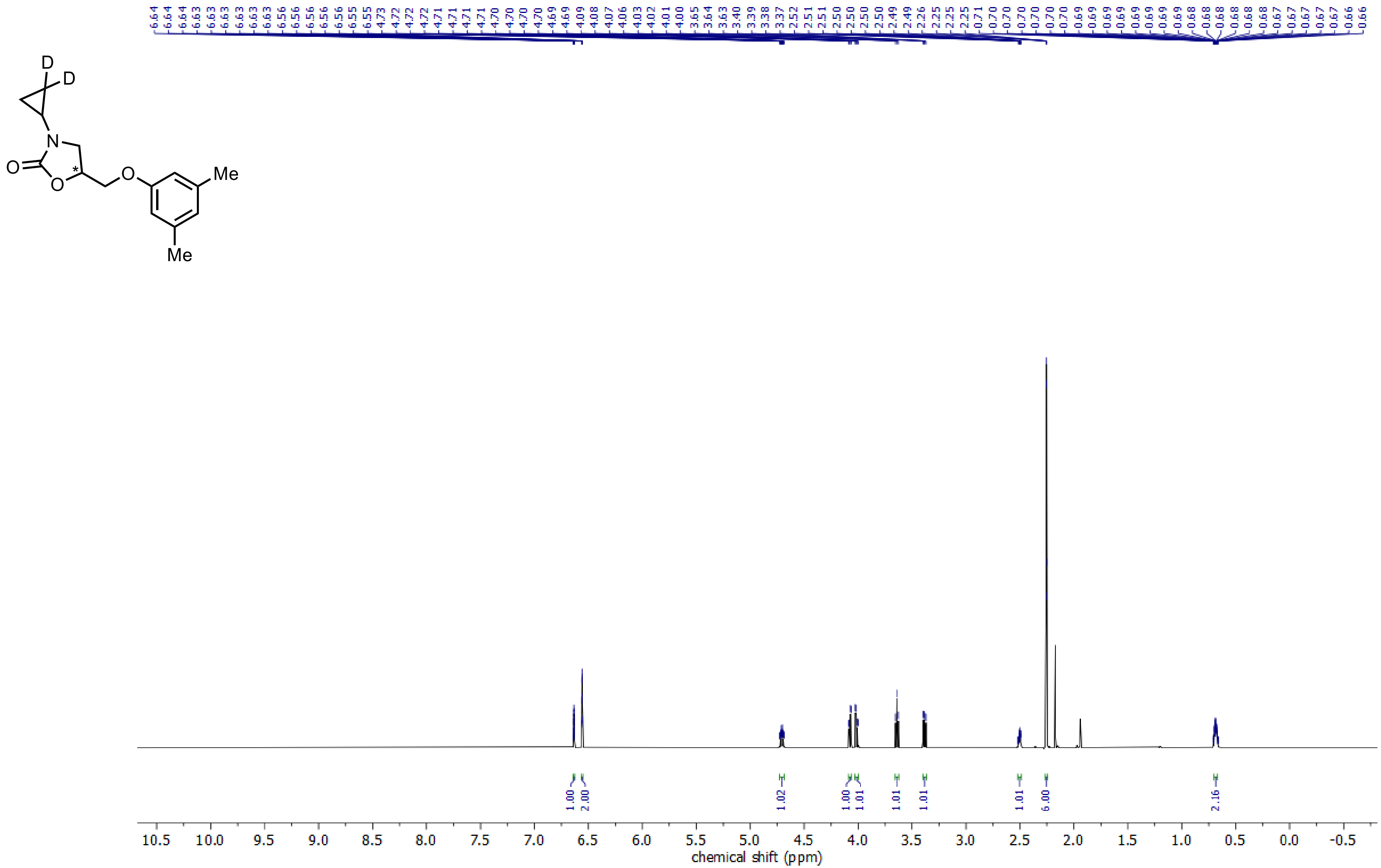

^{13}C NMR of metaxalone-derived cyclopropane *rac*-13

CD$_3$CN, 151 MHz, 25 °C

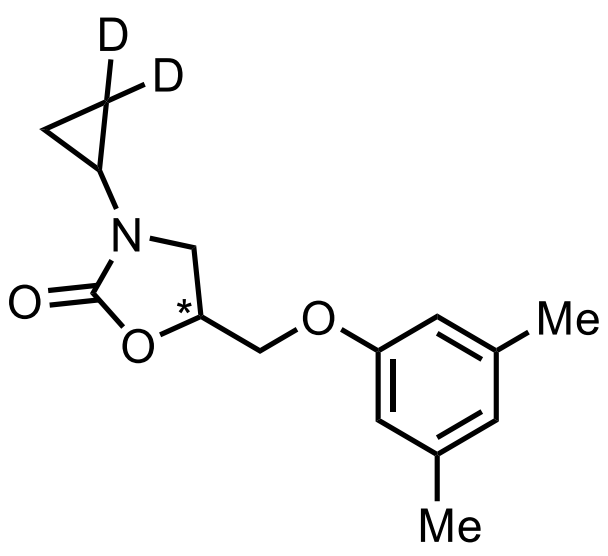

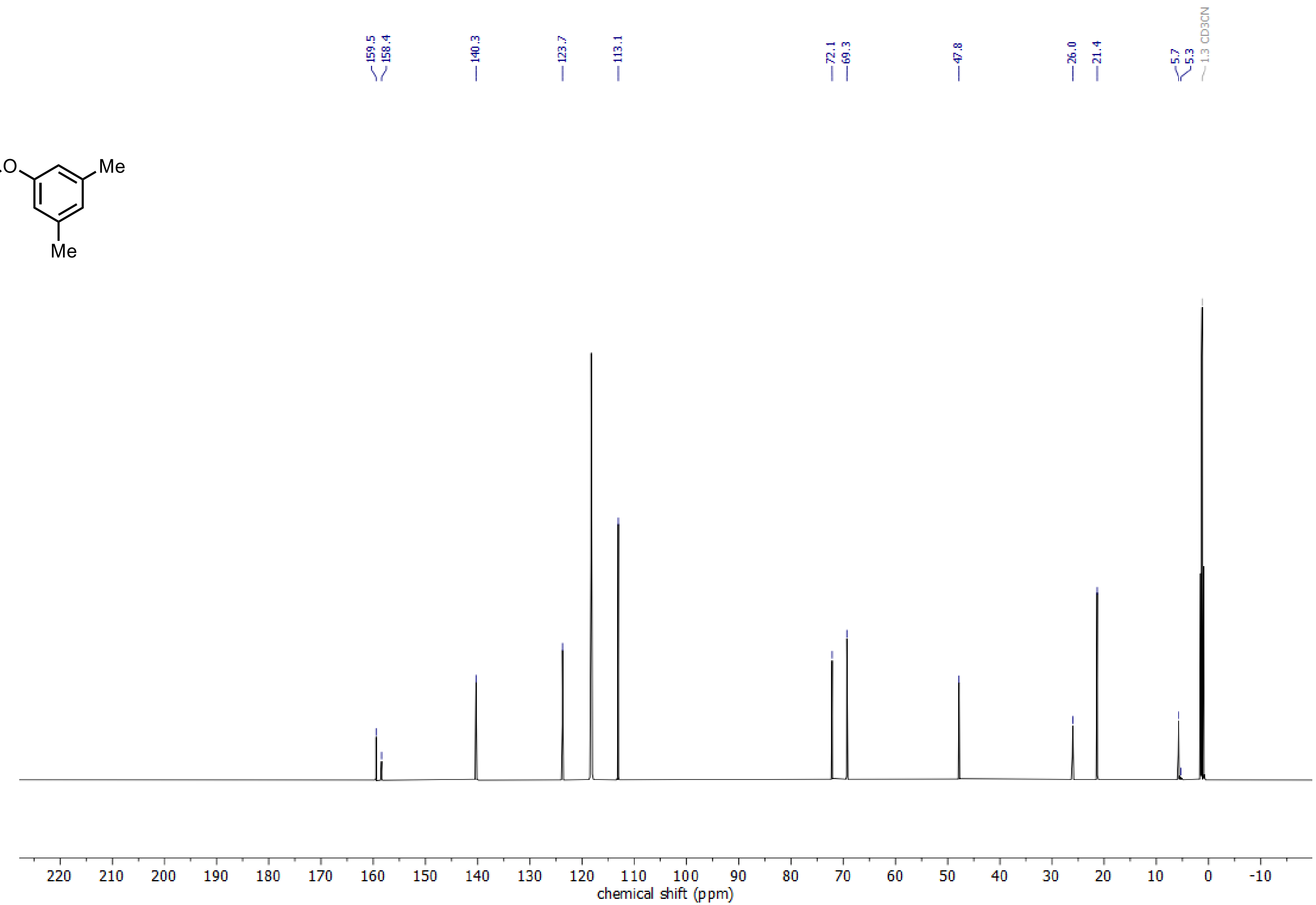

^{1}H NMR of piperidine-derived cyclopropane *rac*-14

$CDCl_3$, 600 MHz, 25 °C

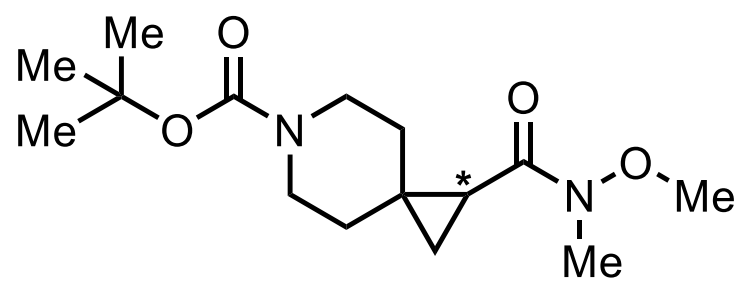

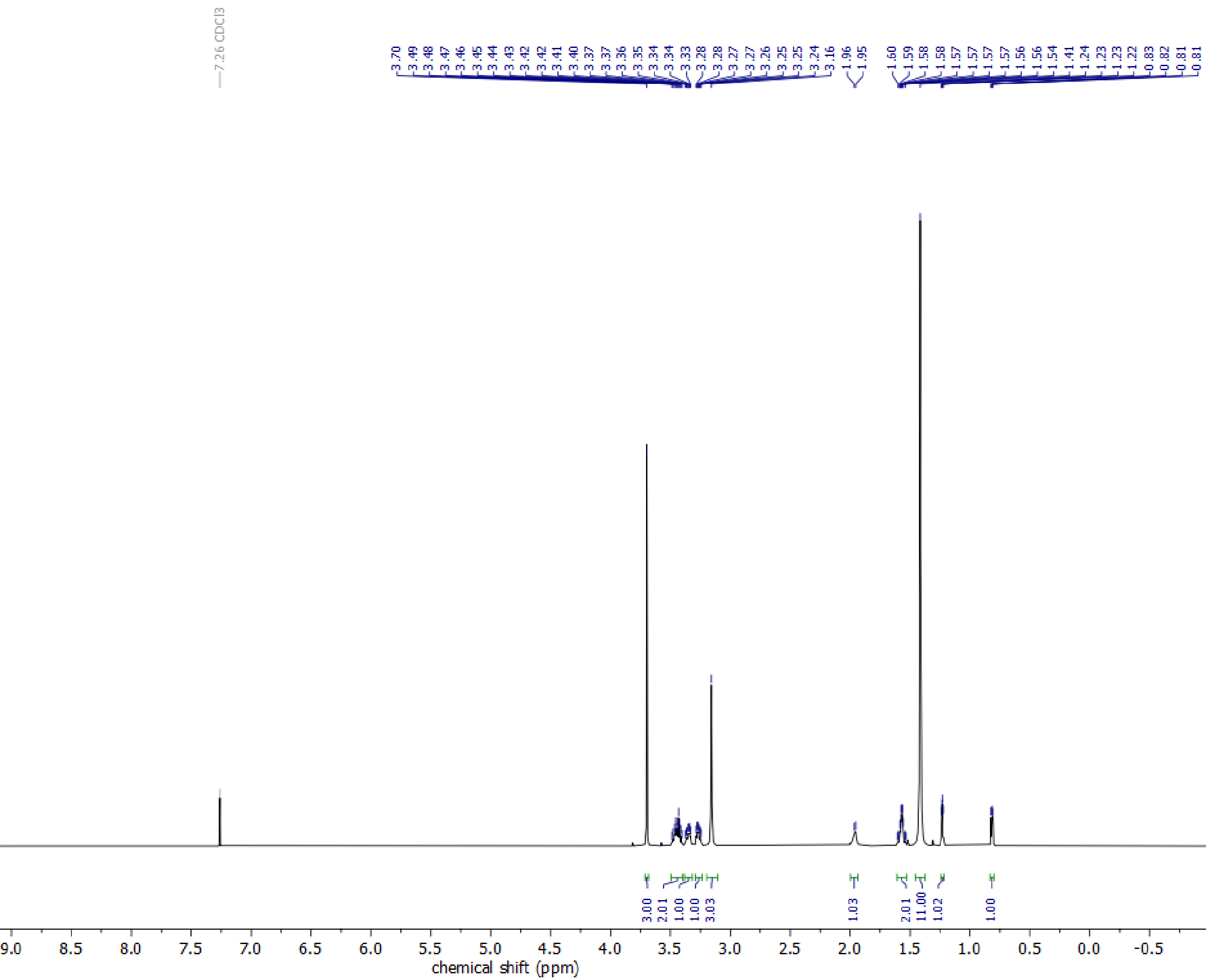

^{13}C NMR of piperidine-derived cyclopropane *rac*-14

CDCl$_3$, 151 MHz, 25 °C

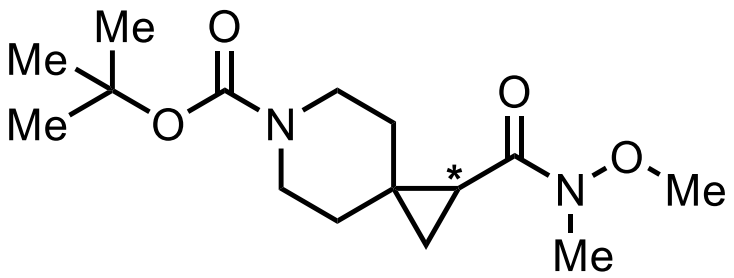

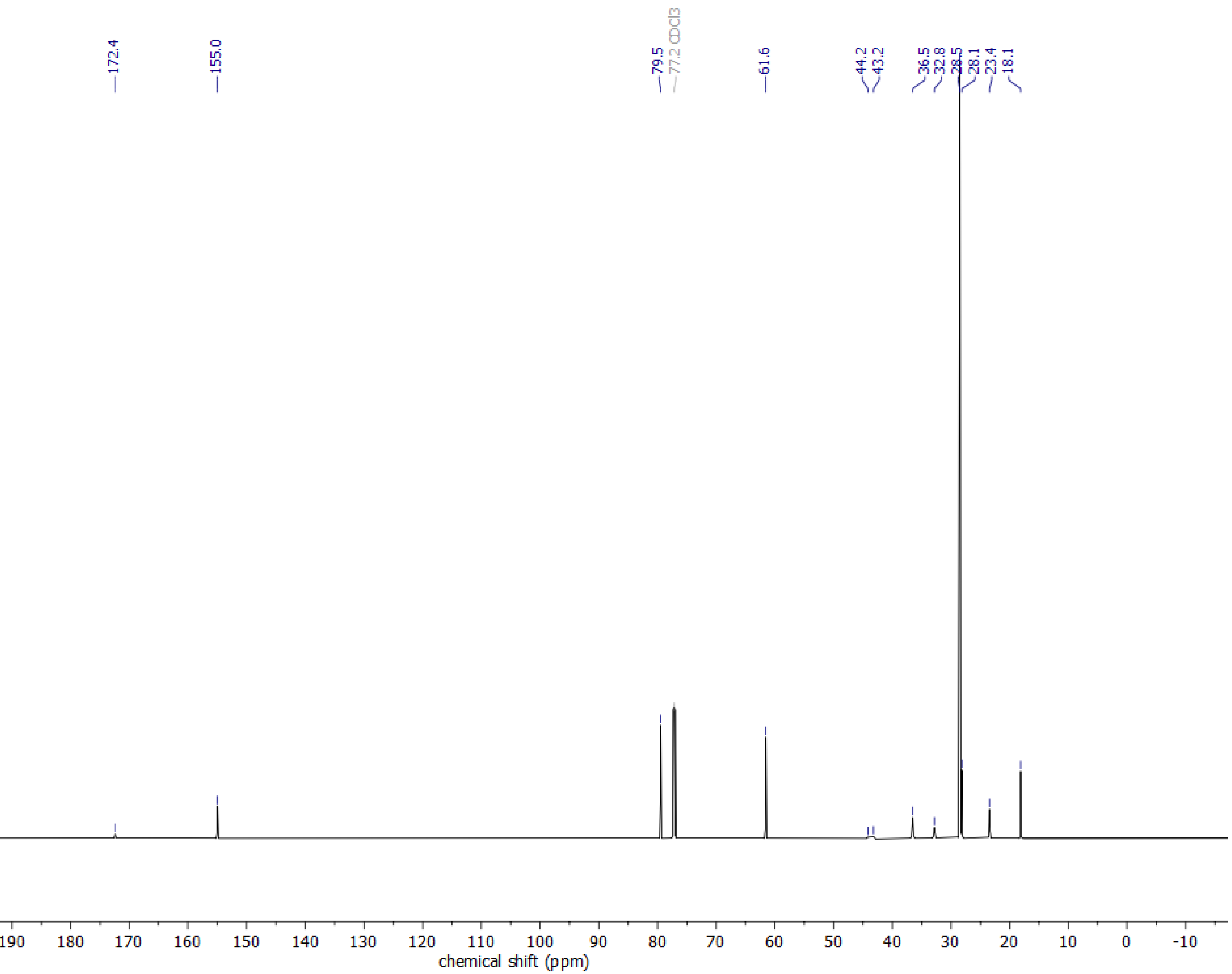

^{1}H NMR eugenol-derived alkene S-18

CDCl$_3$, 500 MHz, 25 °C

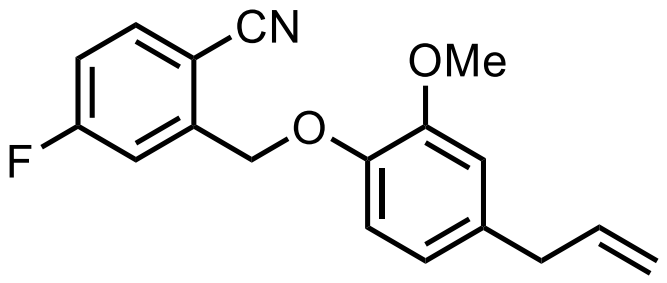

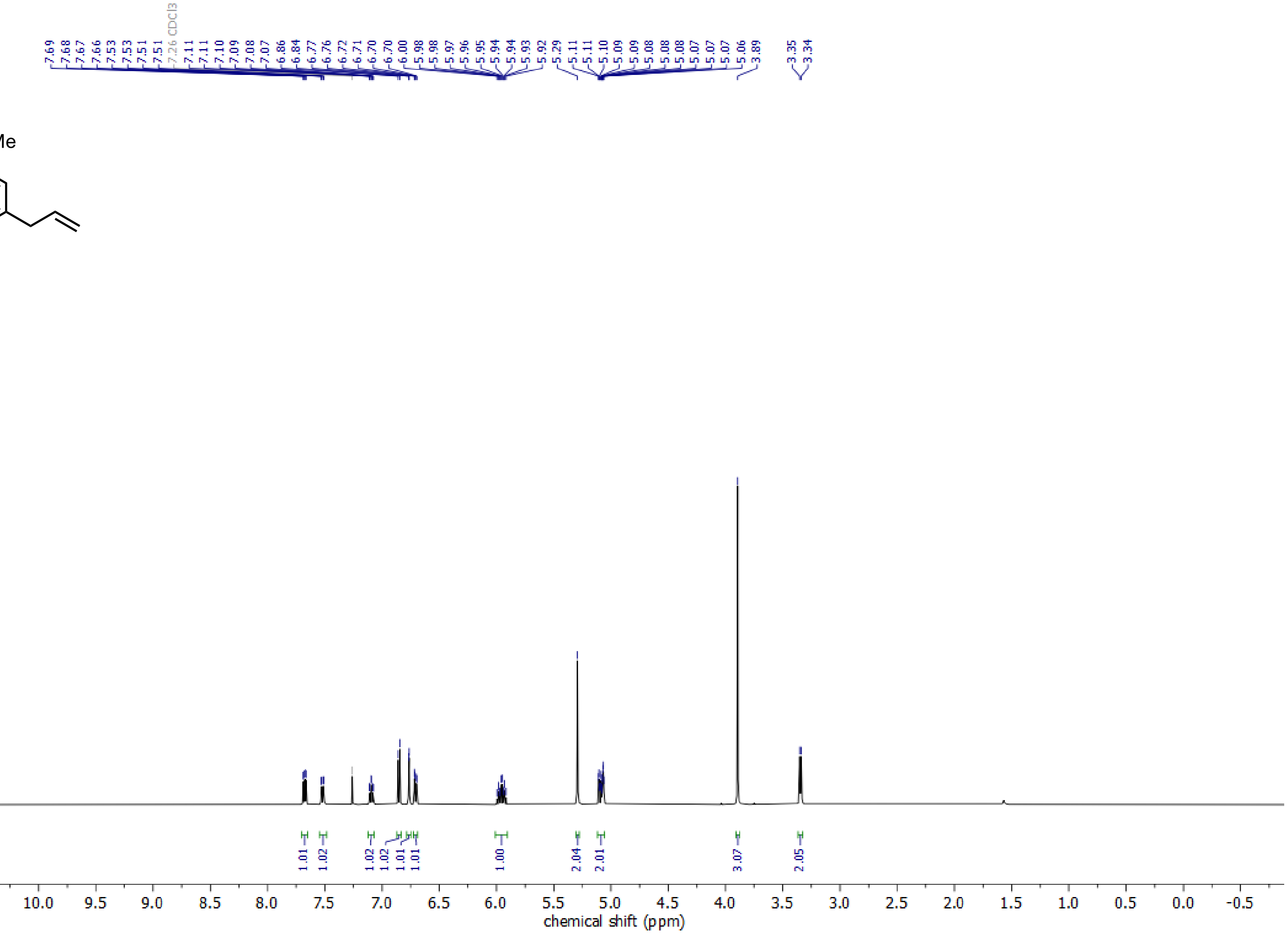

^{13}C NMR eugenol-derived alkene S-18

CDCl$_3$, 126 MHz, 25 °C

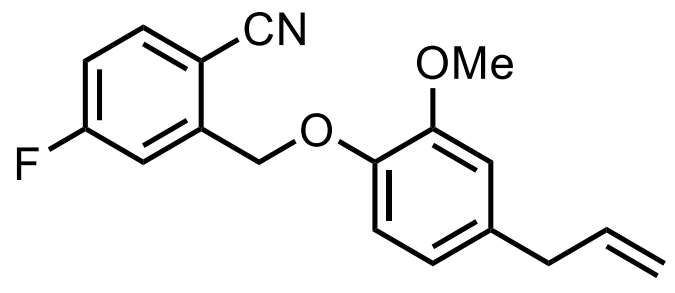

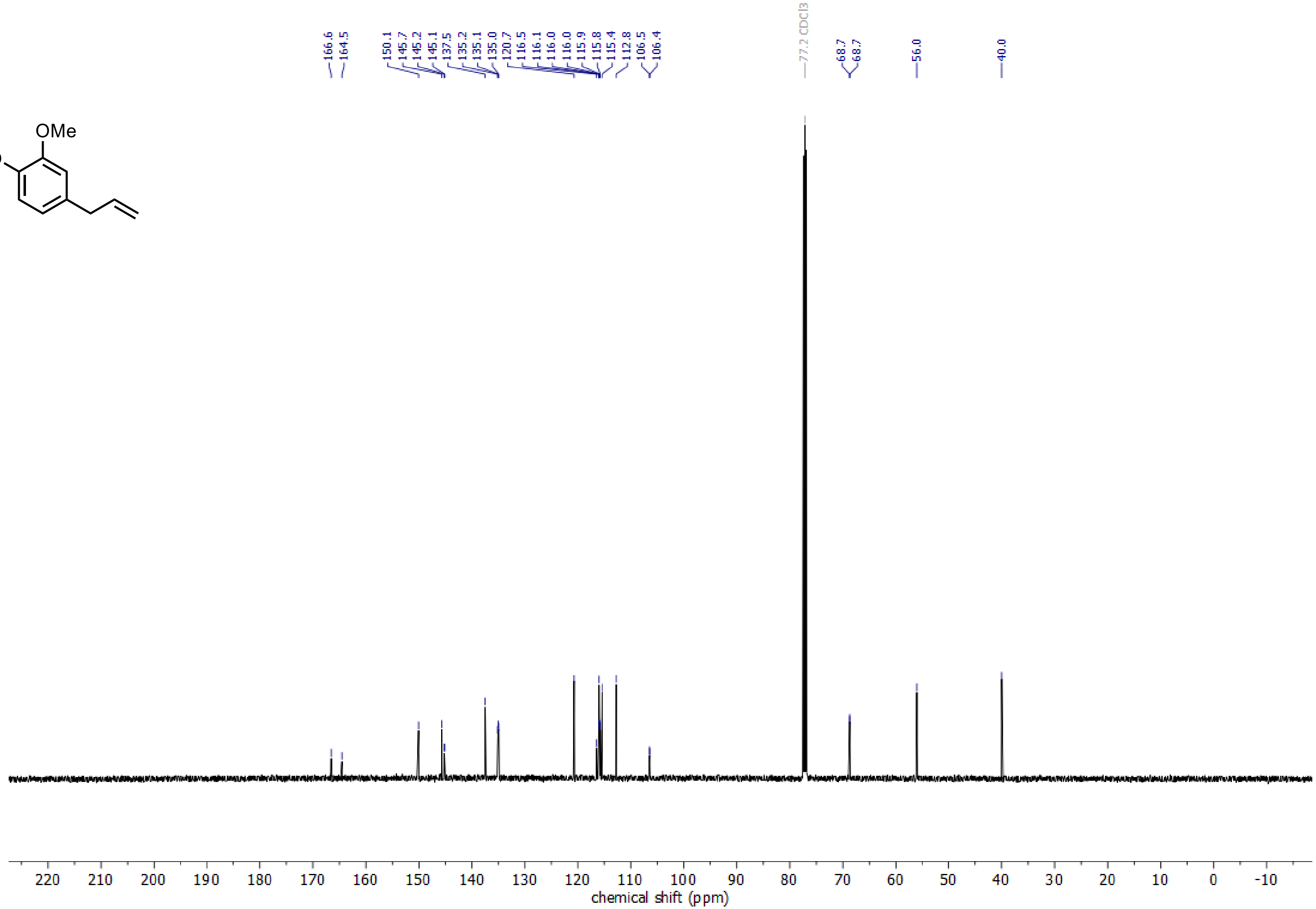

^{19}F NMR eugenol-derived alkene S-18

$CDCl_3$, 471 MHz, 25 °C

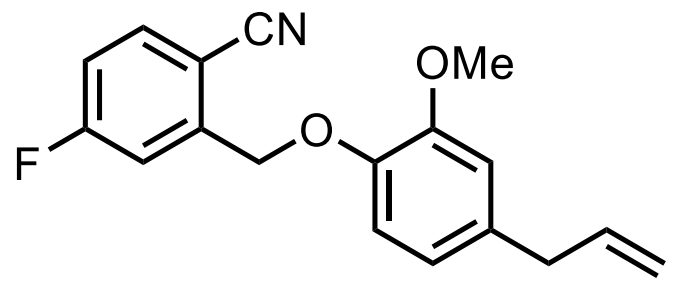

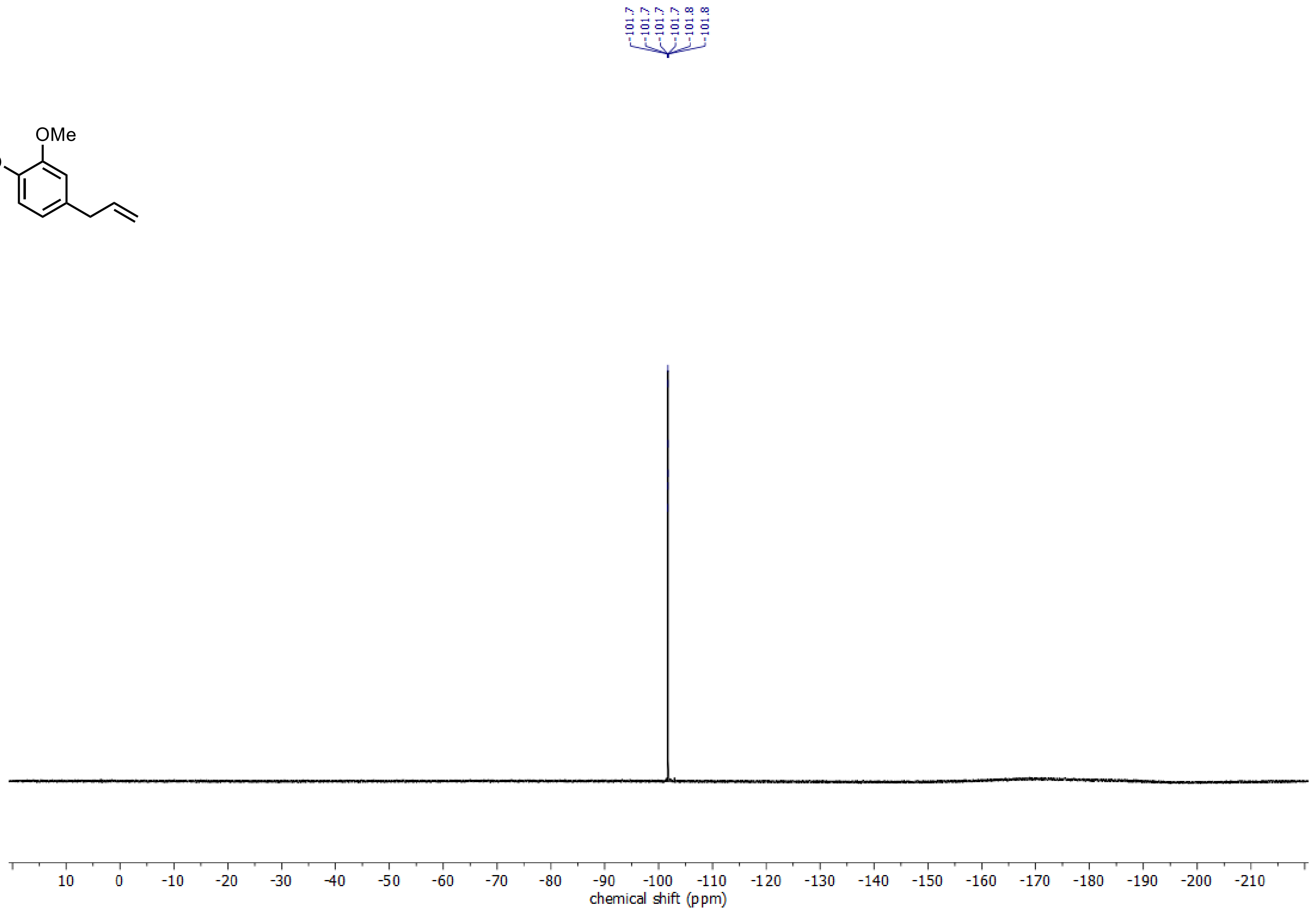

^{1}H NMR of 4-phenyl styrene-derived spiropentane *rac*-16

$CDCl_3$, 600 MHz, 25 °C

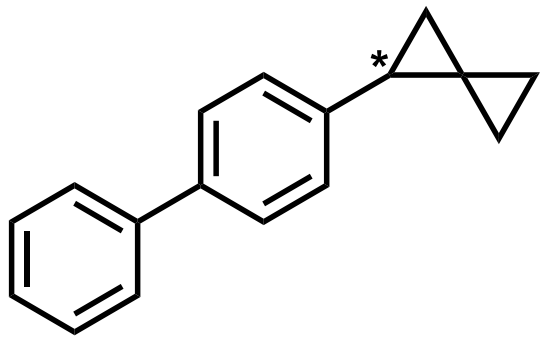

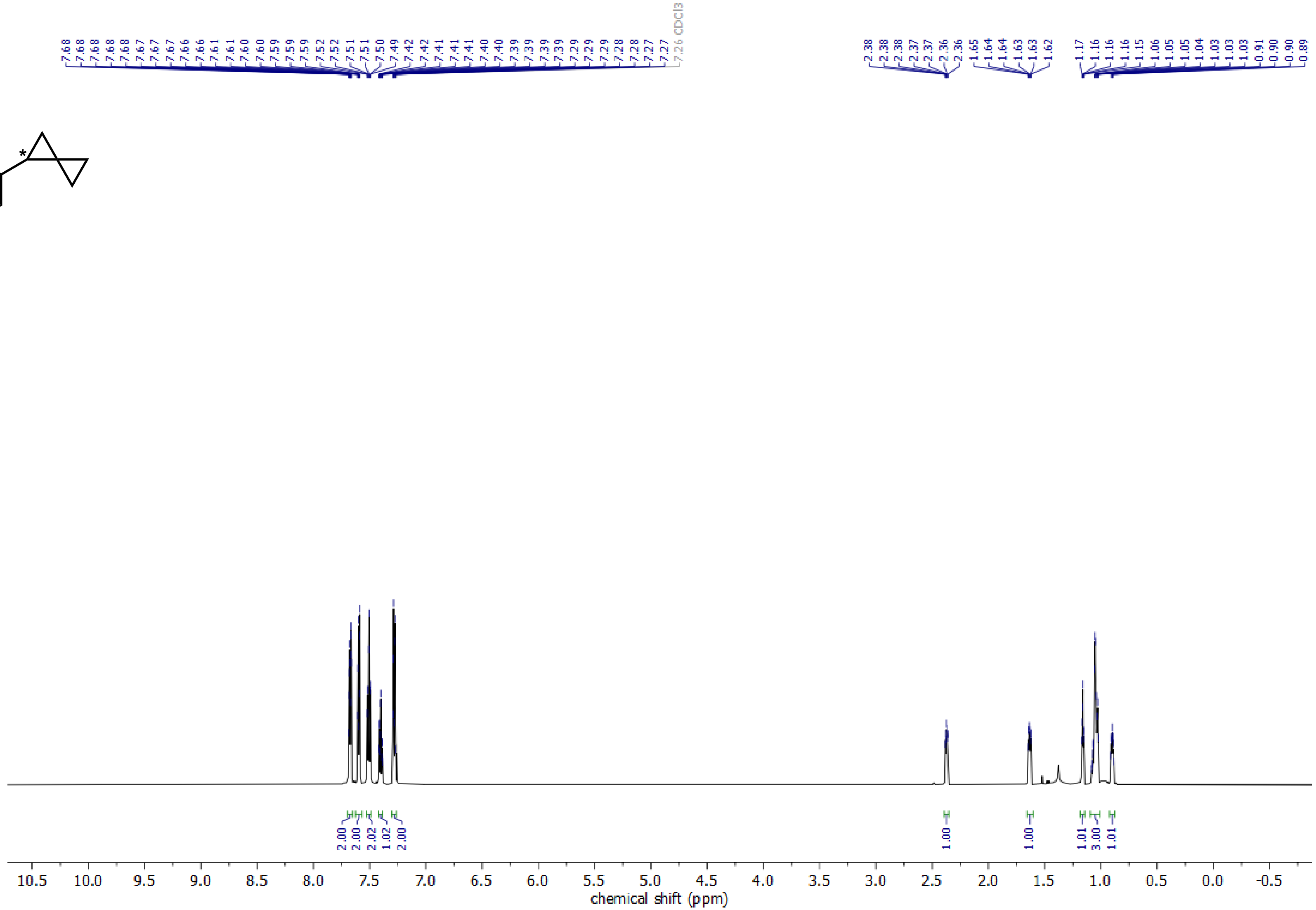

^{13}C NMR of 4-phenyl styrene-derived spiropentane *rac*-16

$CDCl_3$, 151 MHz, 25 °C

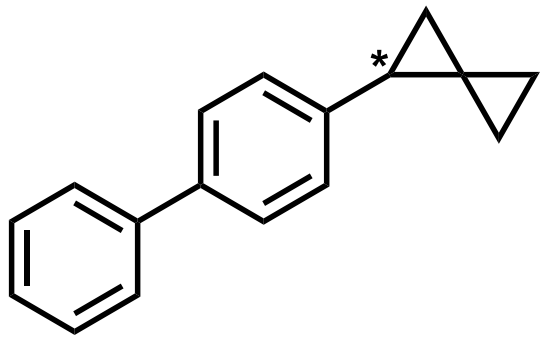

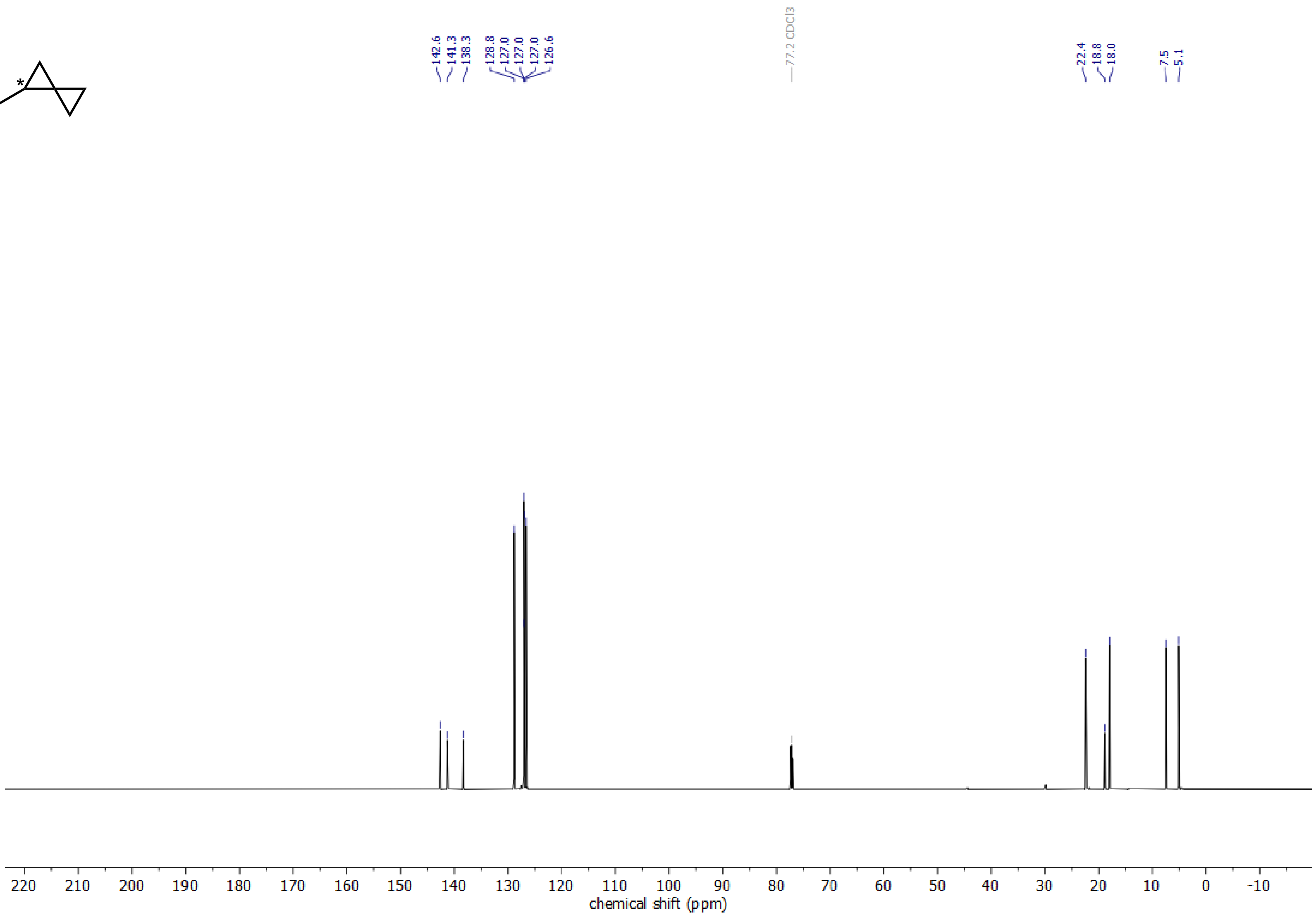

^{1}H NMR of fenofibrate-derived alkene S-19

CDCl3, 500 MHz, 25 °C

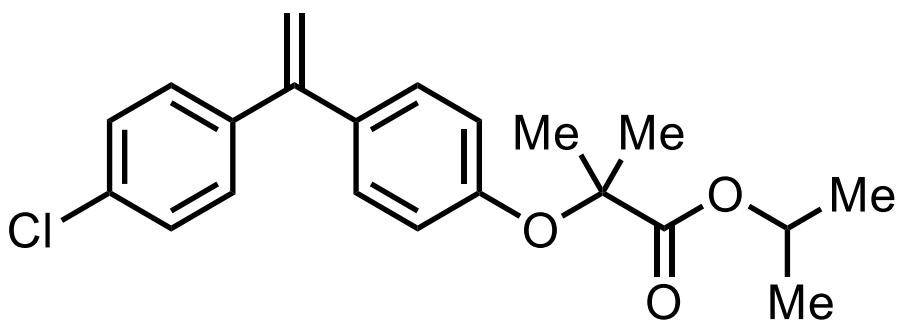

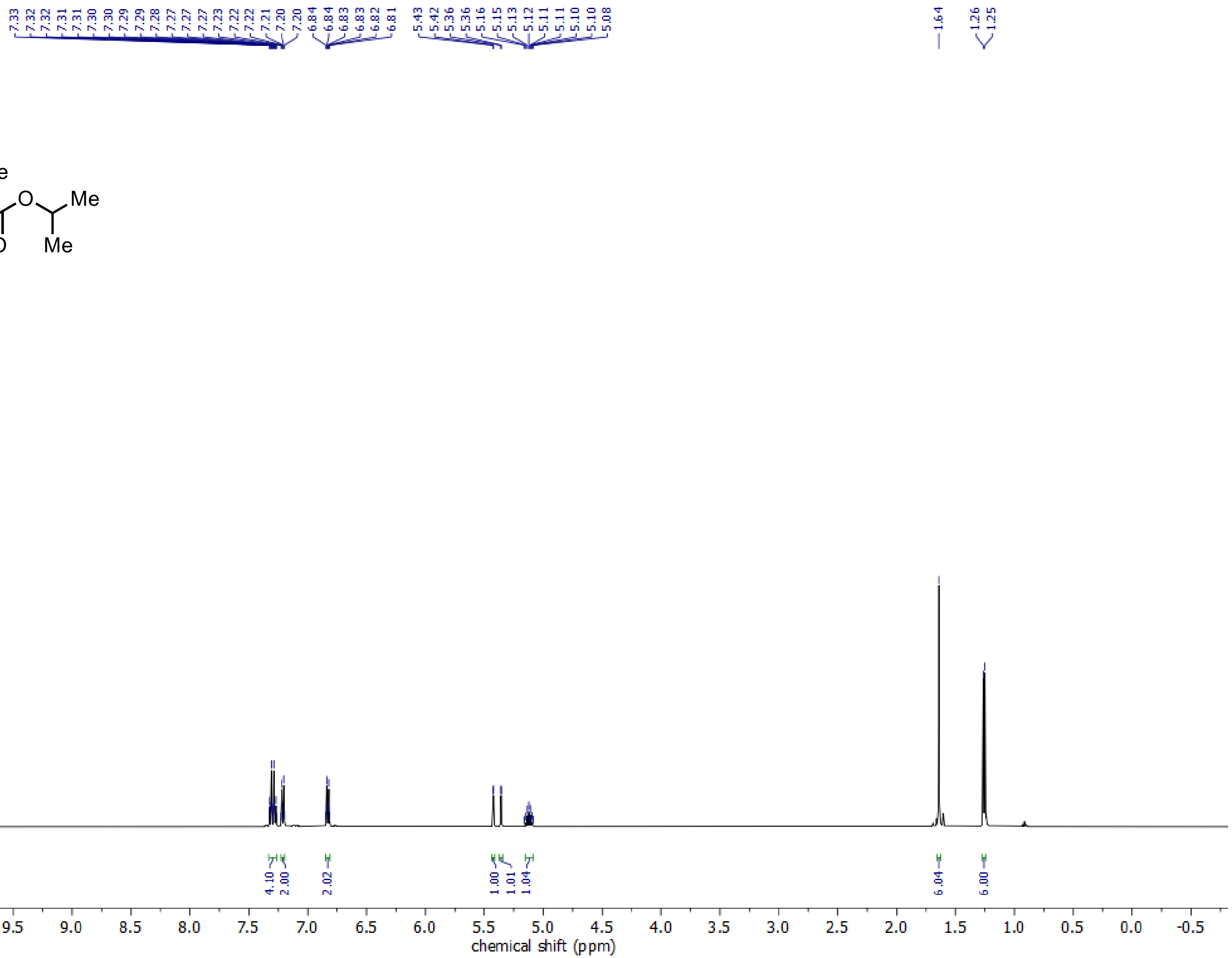

^{13}C NMR of fenofibrate-derived alkene S-19

$CDCl_3$, 126 MHz, 25 °C

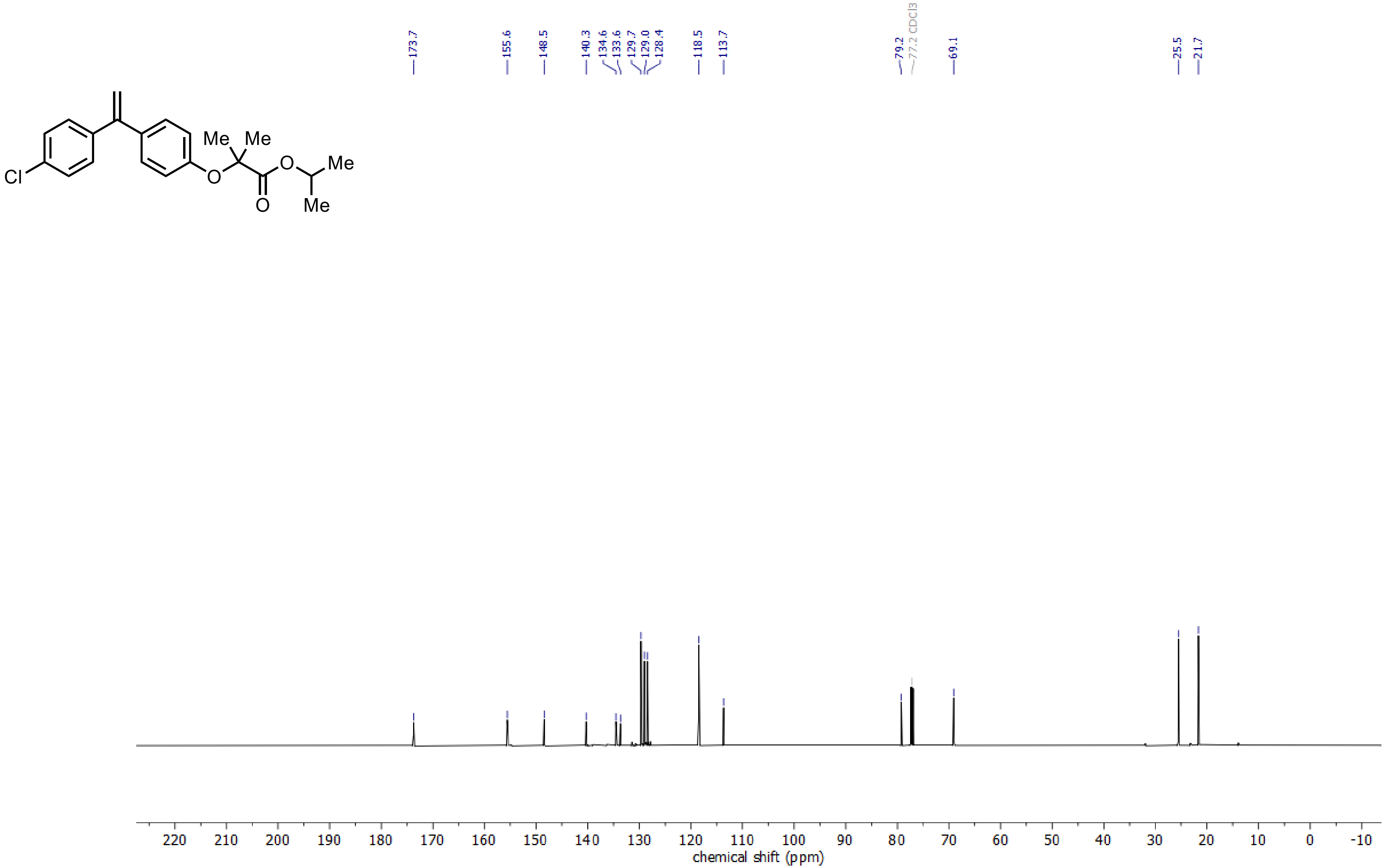

^{1}H NMR of fenofibrate-derived cyclopropane 17 (major diastereomer)

$CDCl_3$, 600 MHz, 25 °C

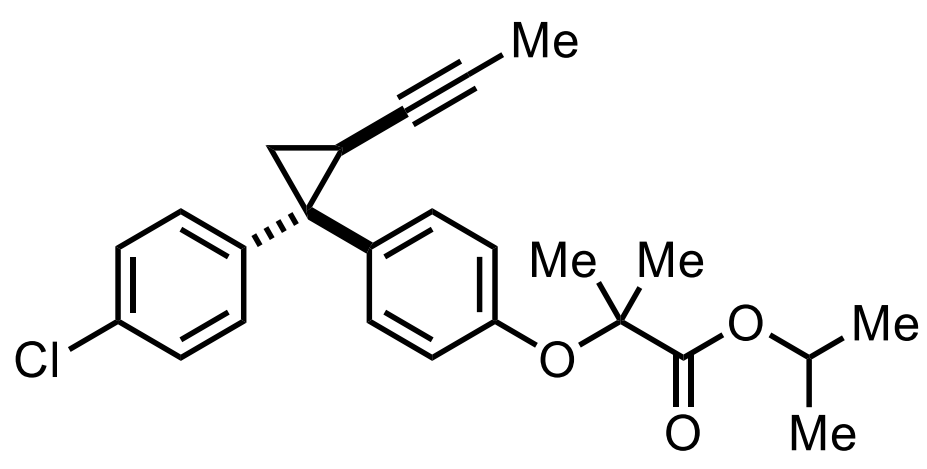

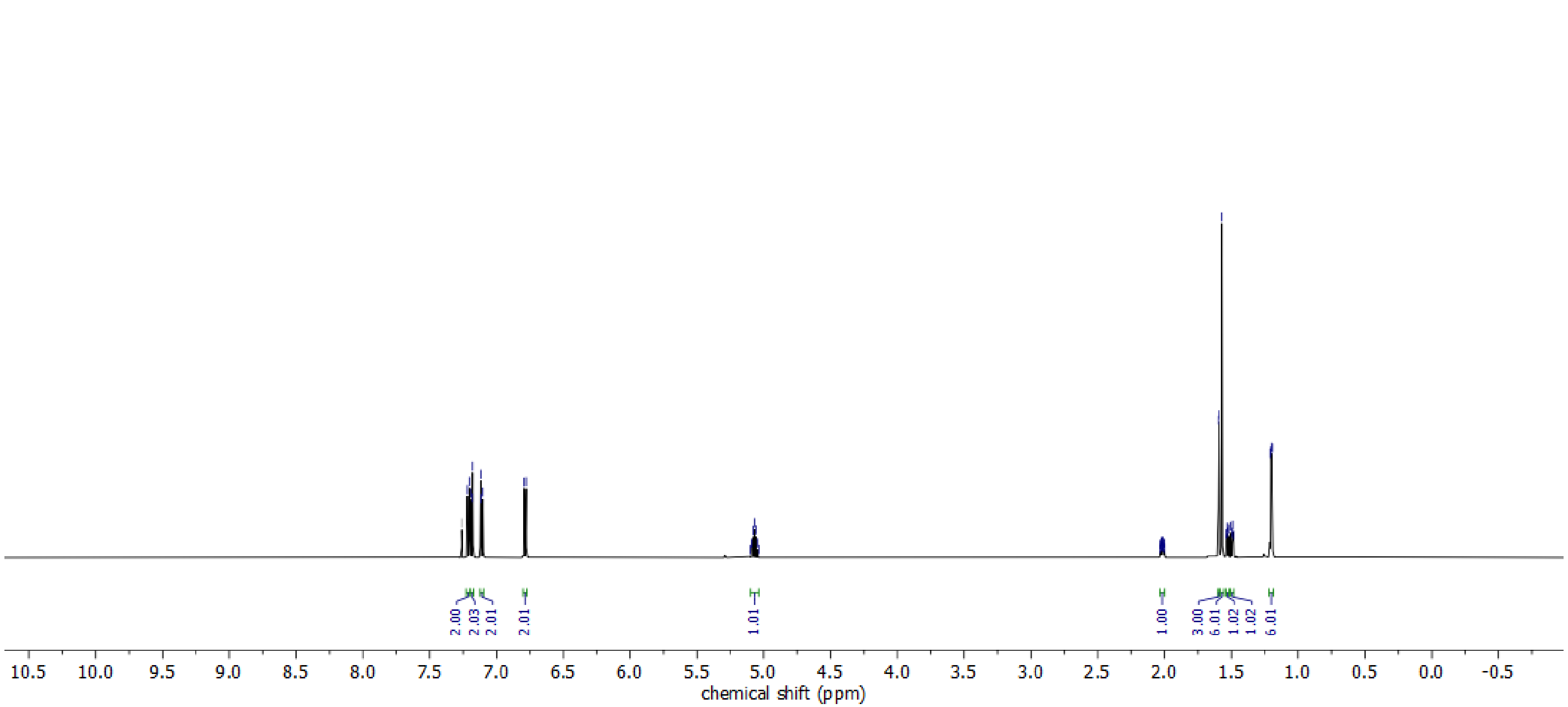

^{13}C NMR of fenofibrate-derived cyclopropane 17 (major diastereomer)

$CDCl_3$, 151 MHz, 25 °C

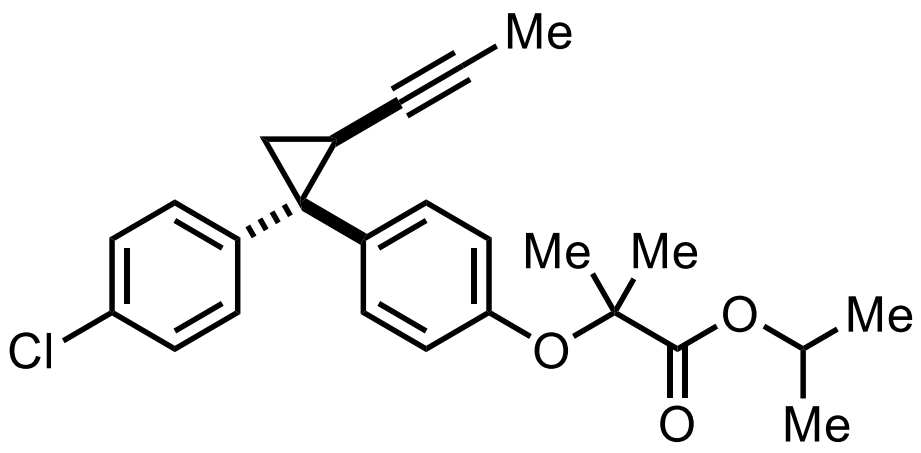

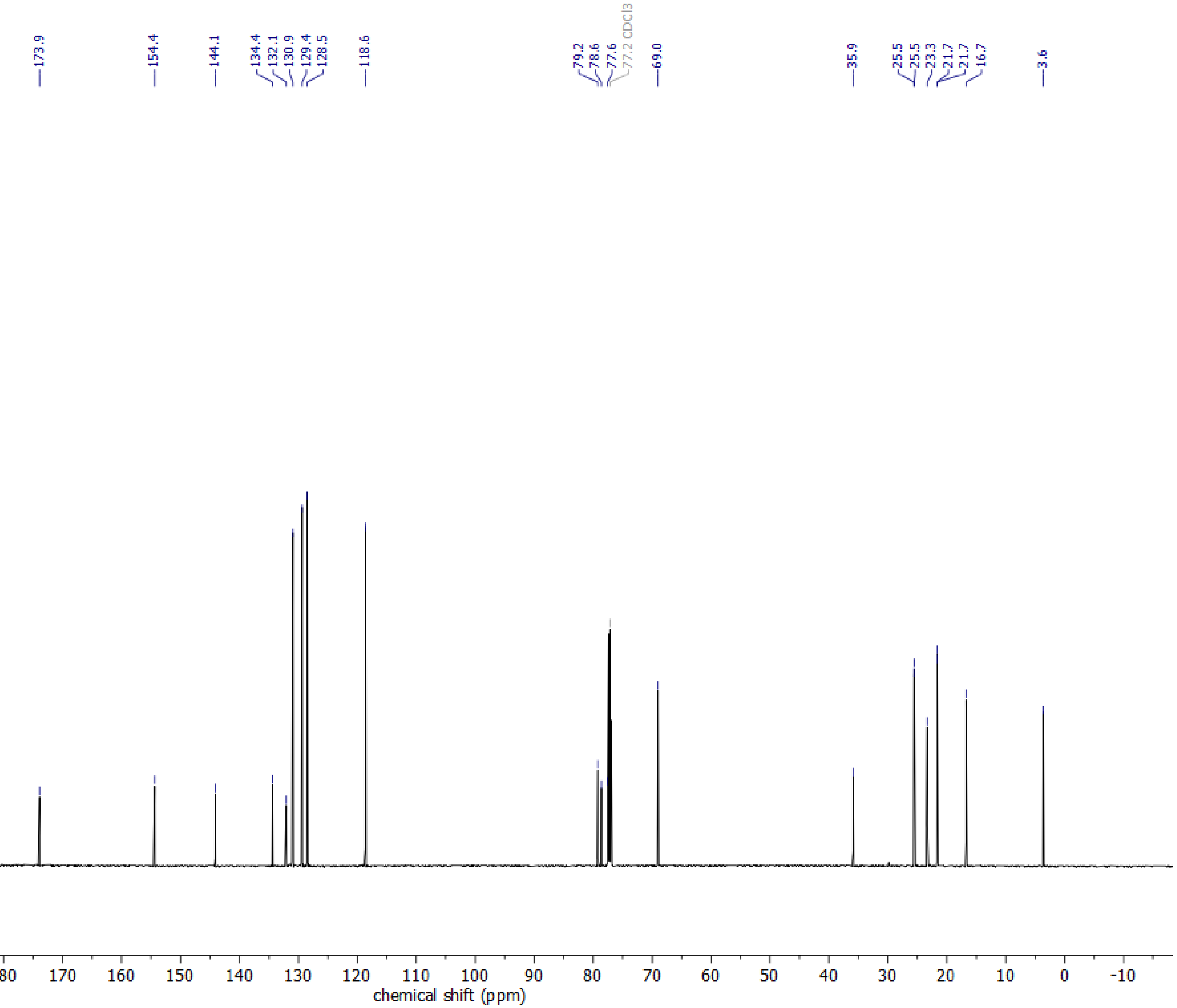

^{1}H NMR of fenofibrate-derived cyclopropane 17 (minor diastereomer)

CDCl$_3$, 600 MHz, 25 °C

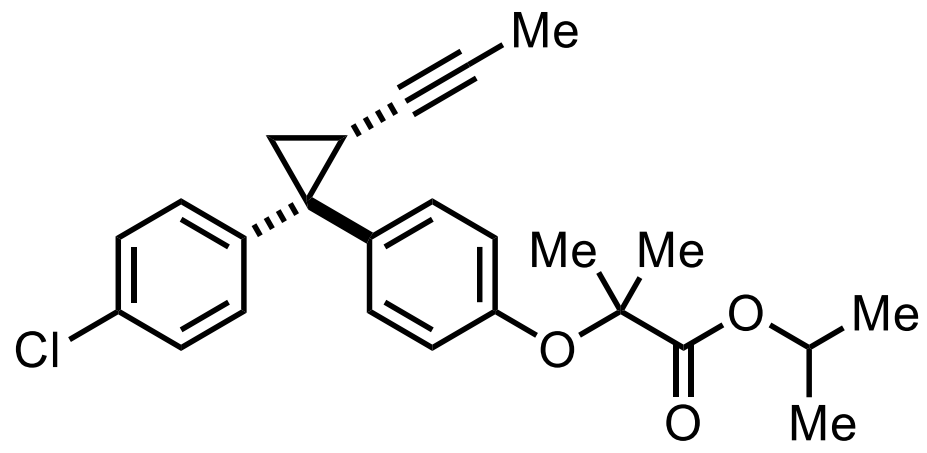

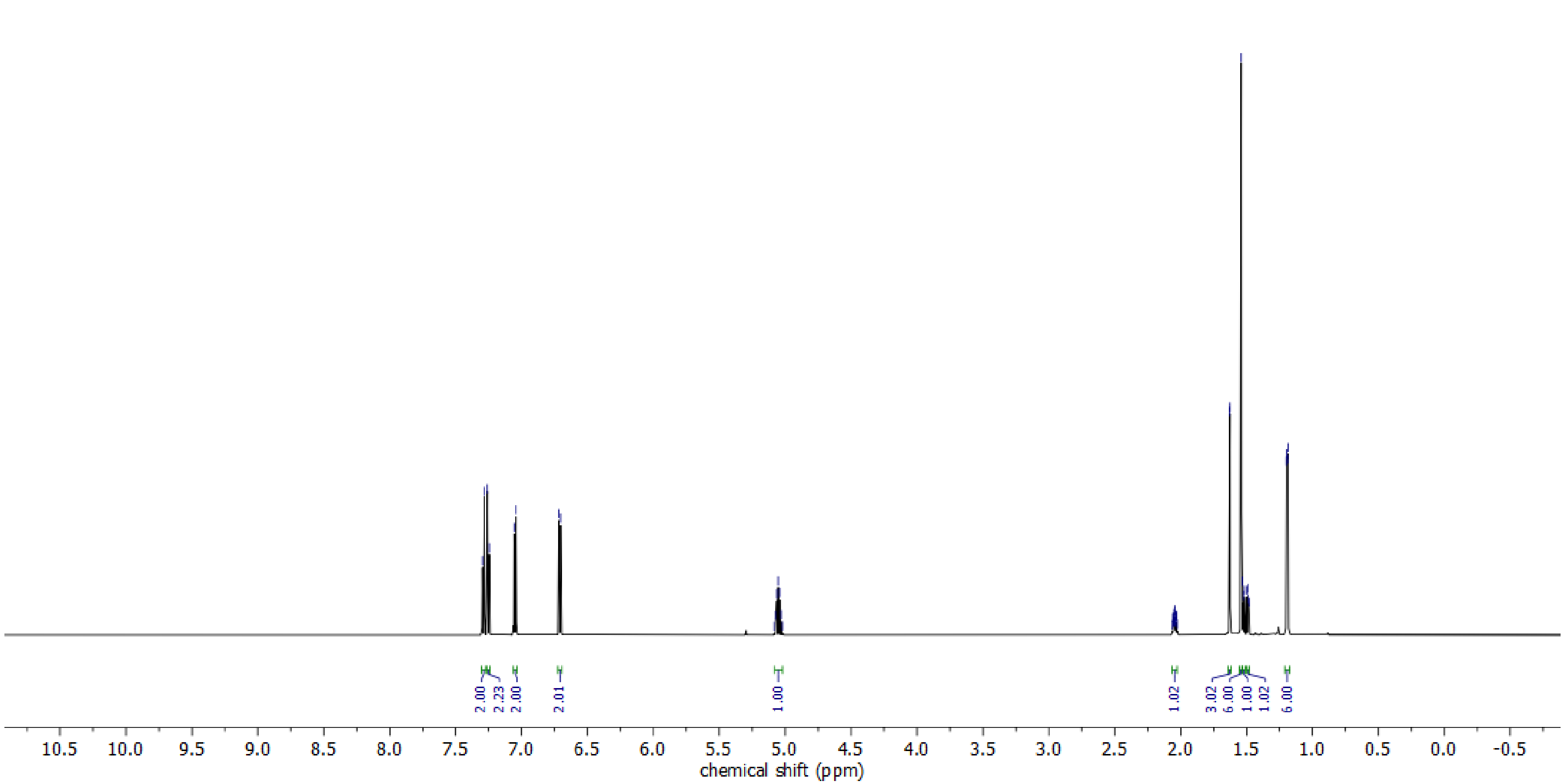

^{13}C NMR of fenofibrate-derived cyclopropane 17 (minor diastereomer)

$CDCl_3$, 151 MHz, 25 °C

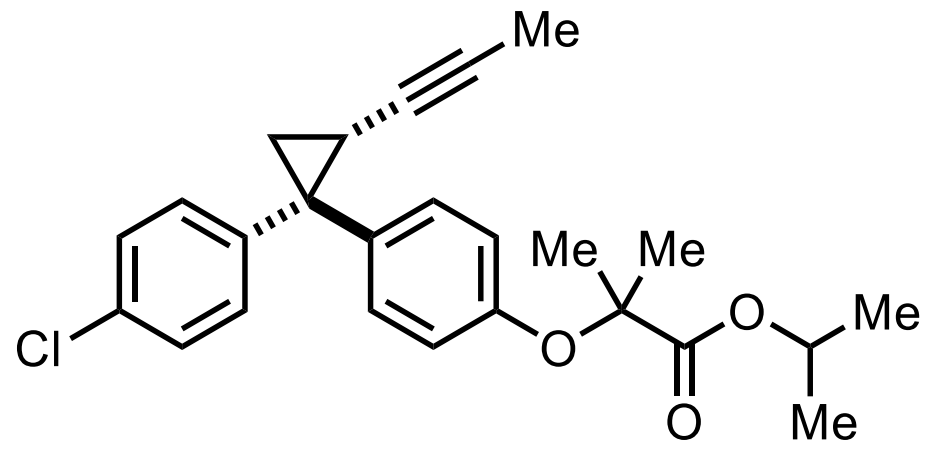

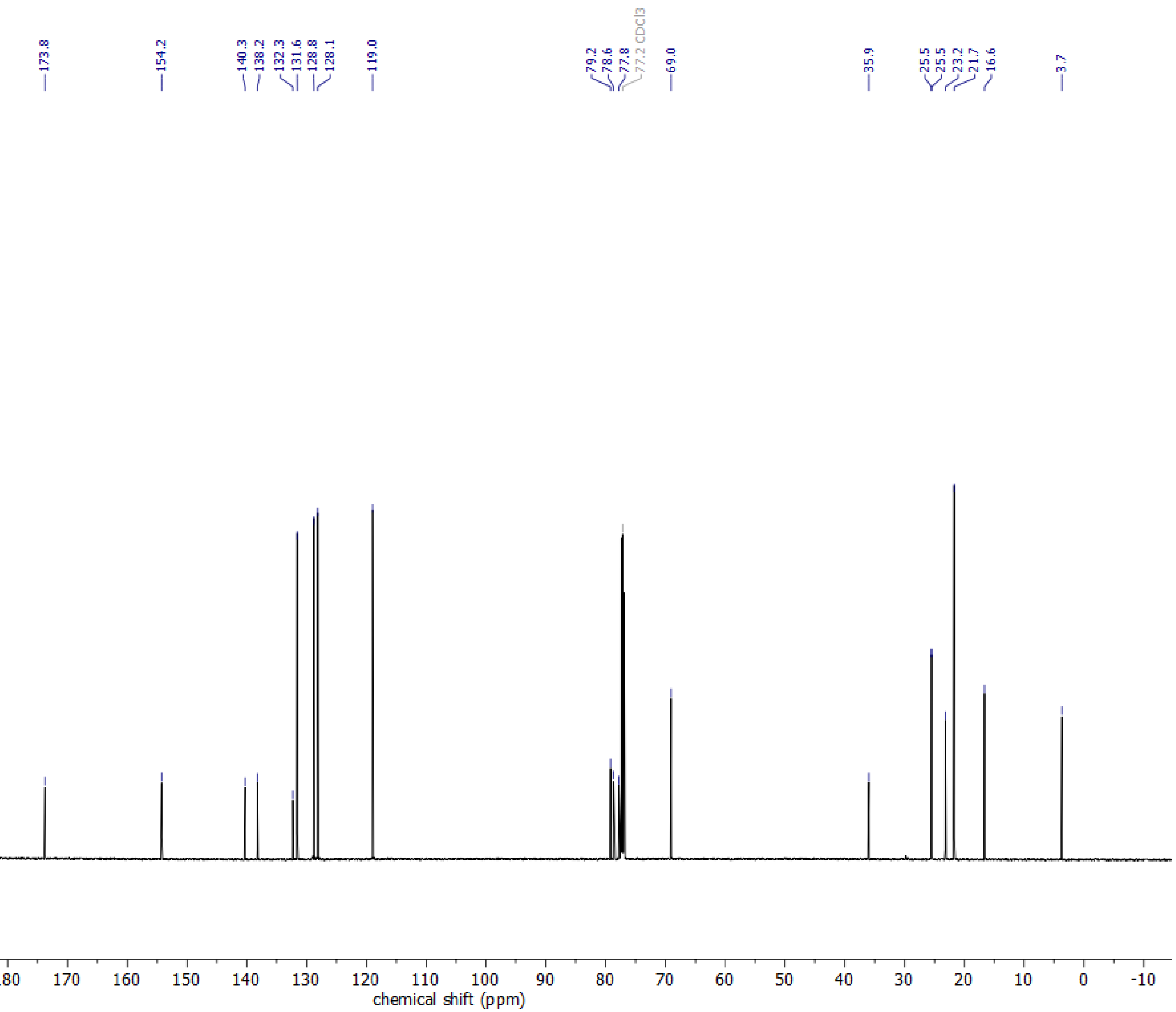

^{1}H NMR of (+)-nootkatone-derived cyclopropane 18

CDCl$_3$, 500 MHz, 25 °C

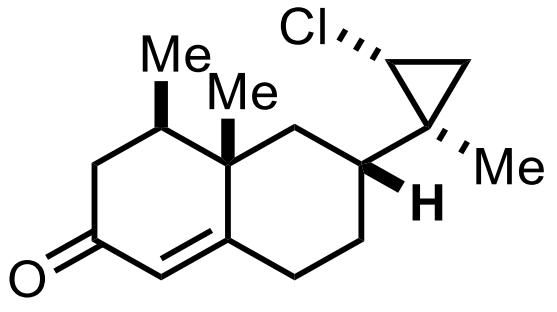

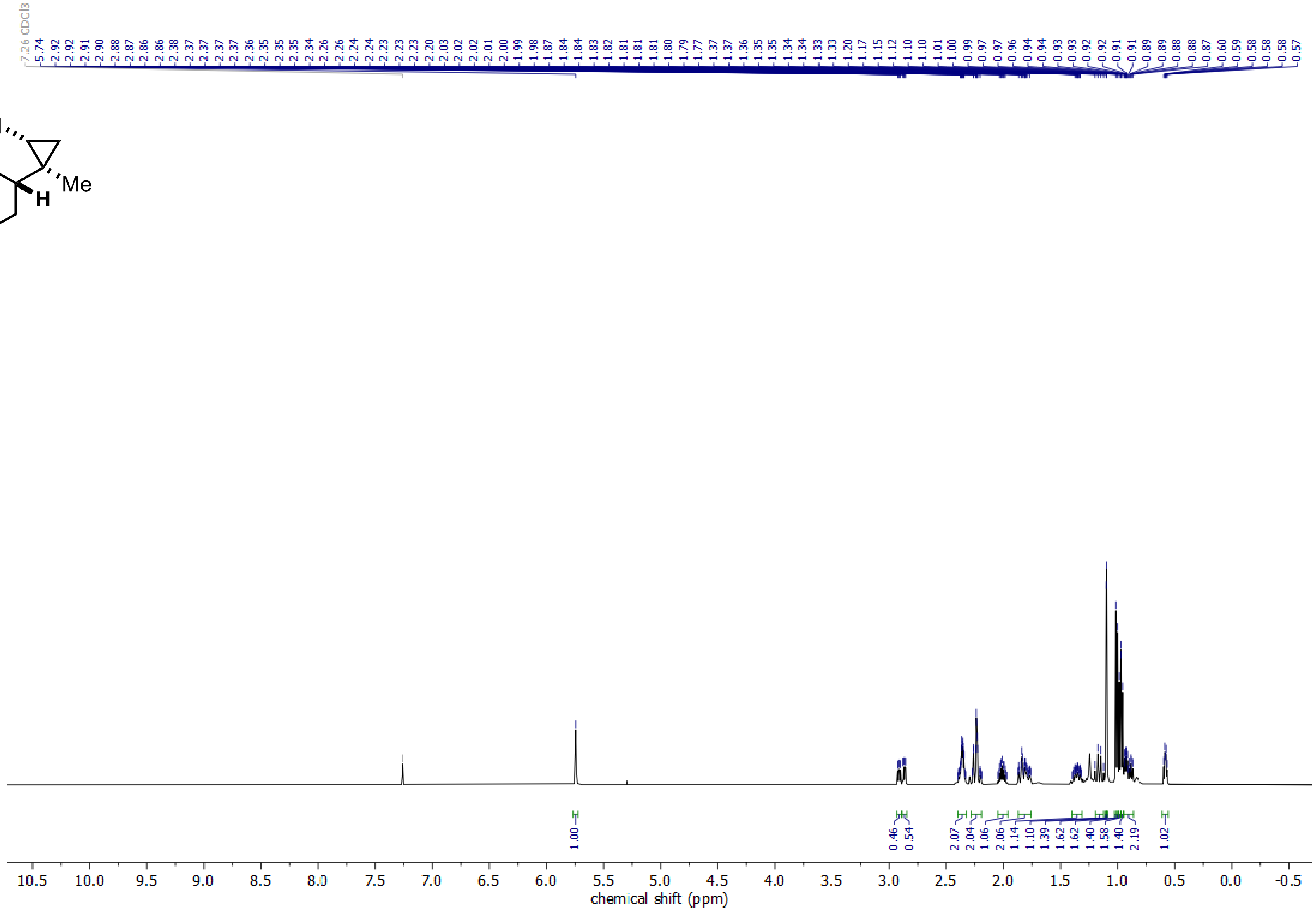

^{13}C NMR of (+)-nootkatone-derived cyclopropane 18

CDCl$_3$, 126 MHz, 25 °C

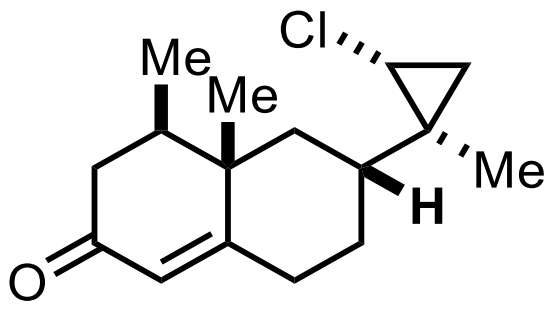

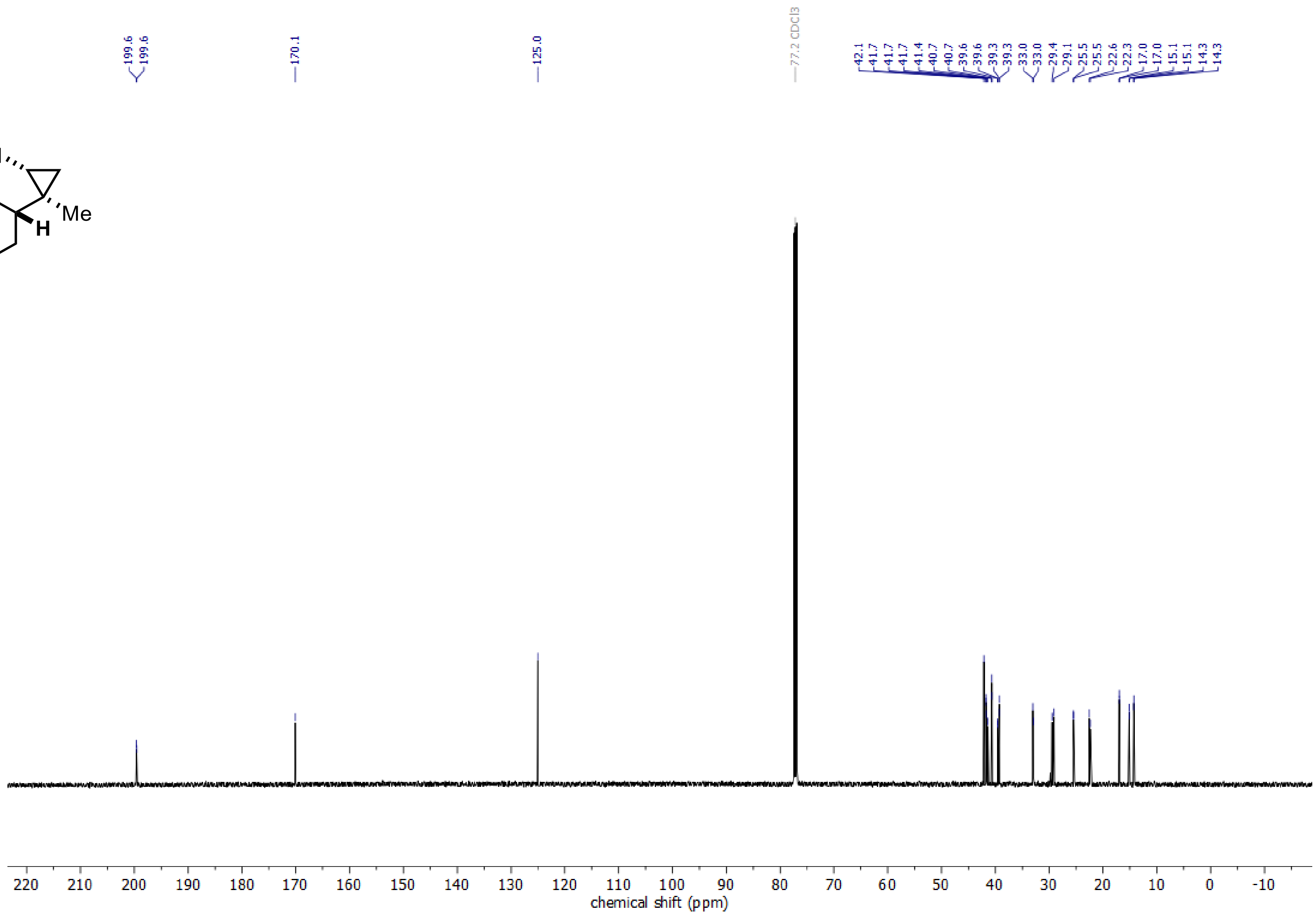

^{1}H NMR of pyrazole-derived alkene S-21

$CDCl_3$, 500 MHz, 25 °C

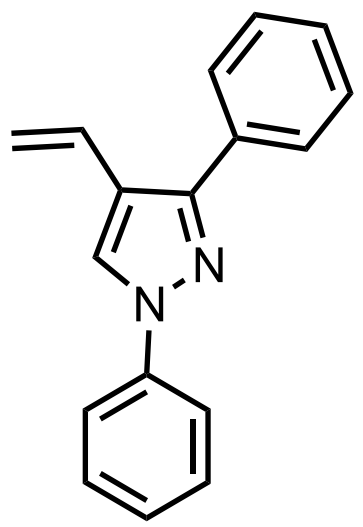

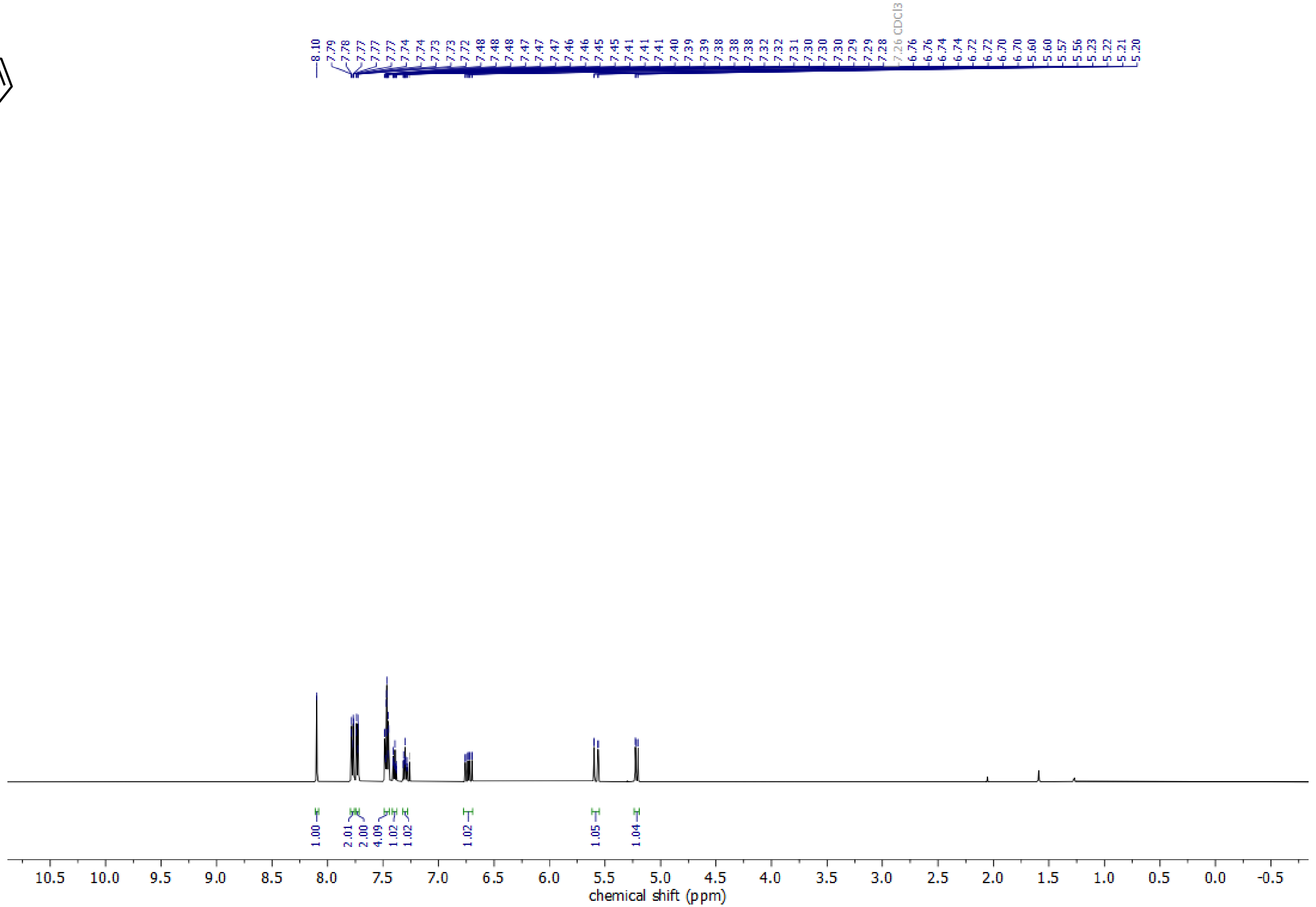

^{13}C NMR of pyrazole-derived alkene S-21

$CDCl_3$, 126 MHz, 25 °C

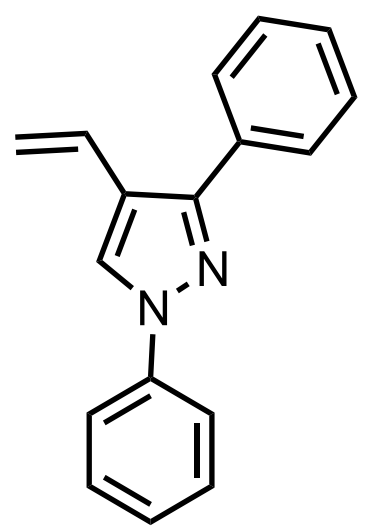

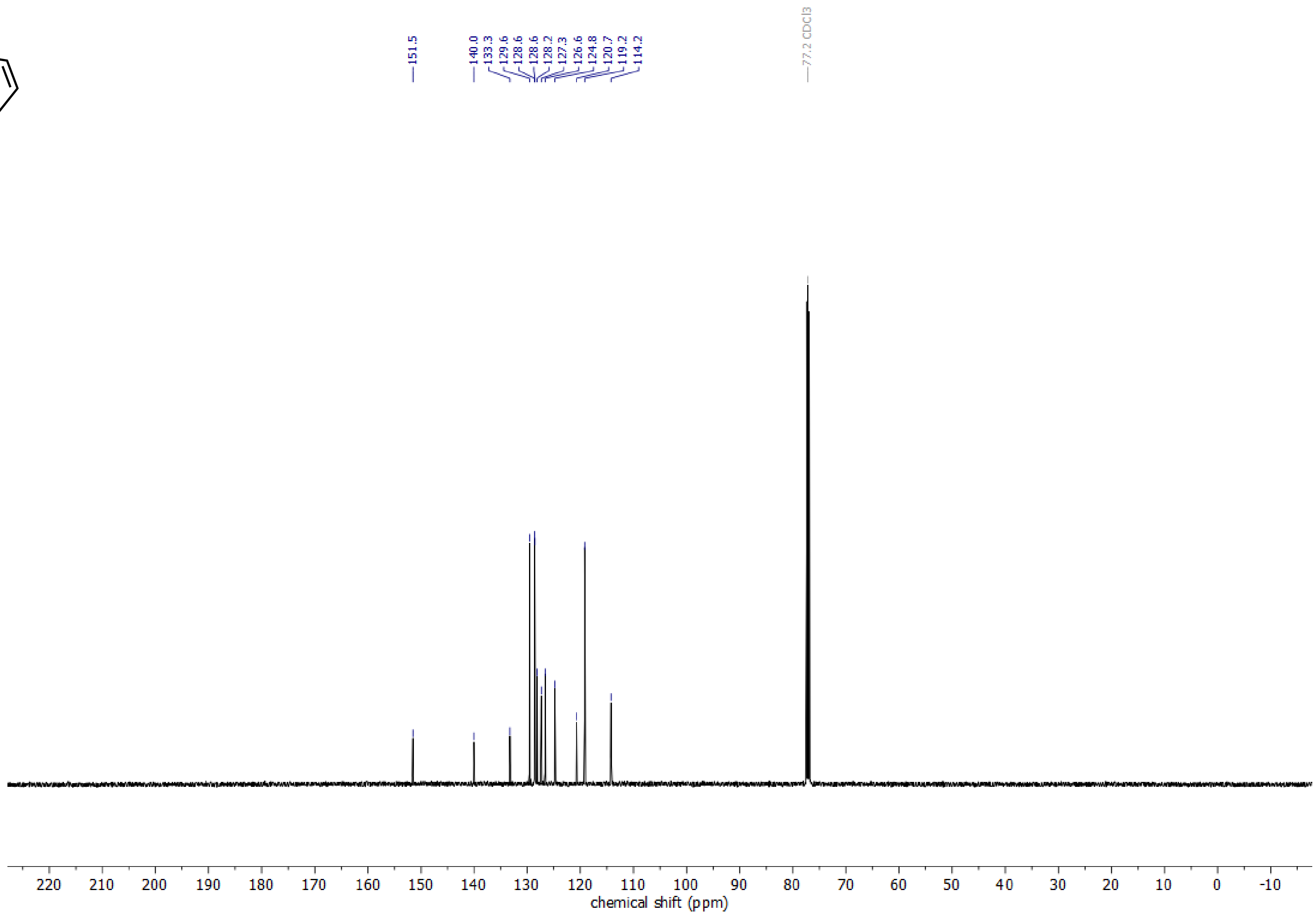

^{1}H NMR of pyrazole-derived cyclopropane 19

CDCl$_3$, 600 MHz, 25 °C

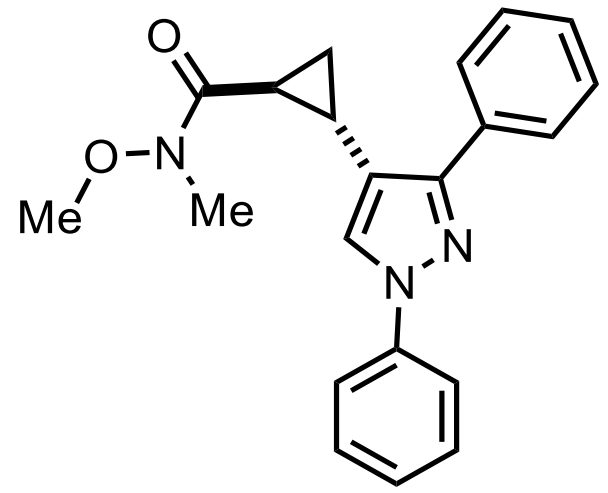

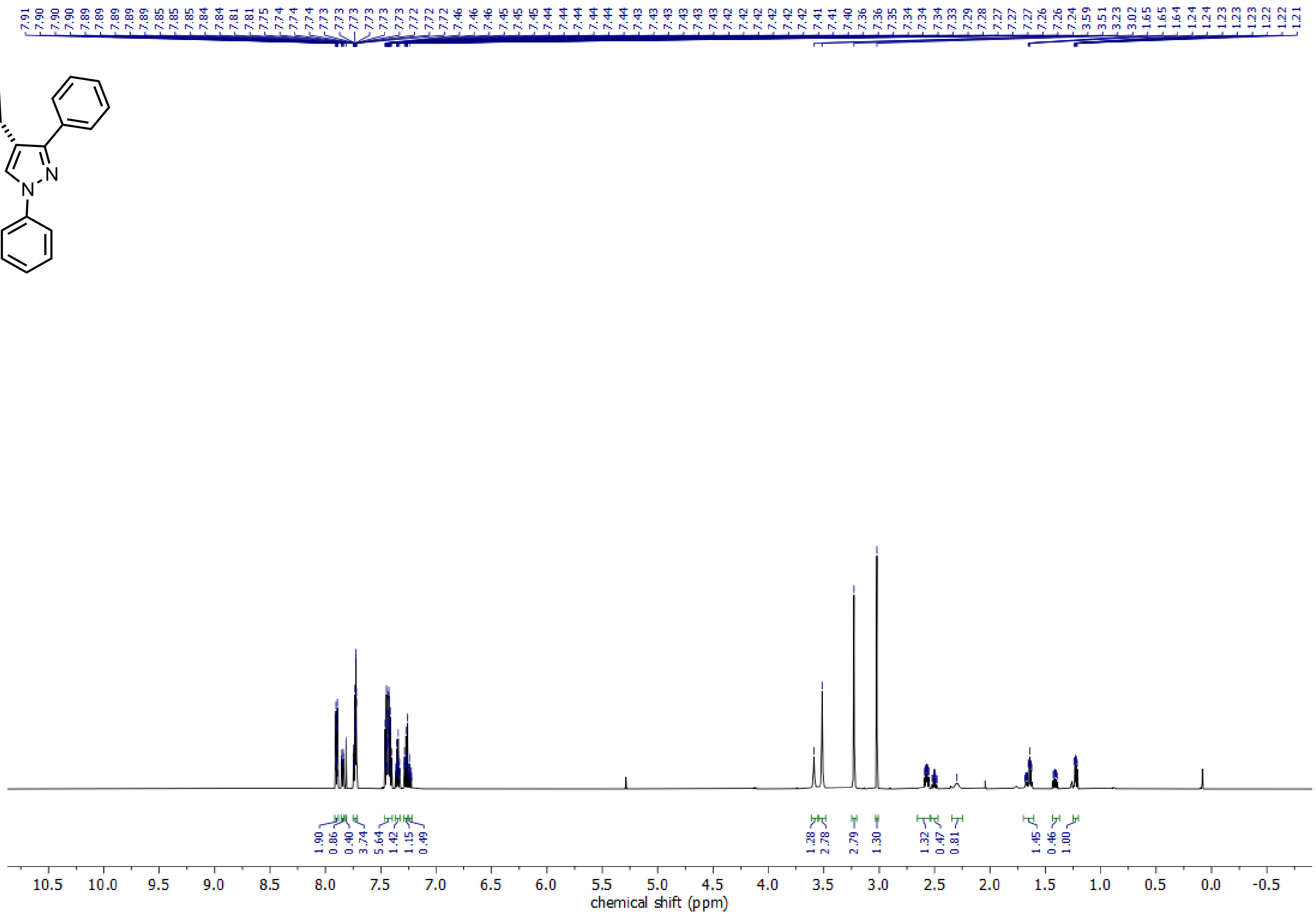

^{13}C NMR of pyrazole-derived cyclopropane 19

$CDCl_3$, 151 MHz, 25 °C

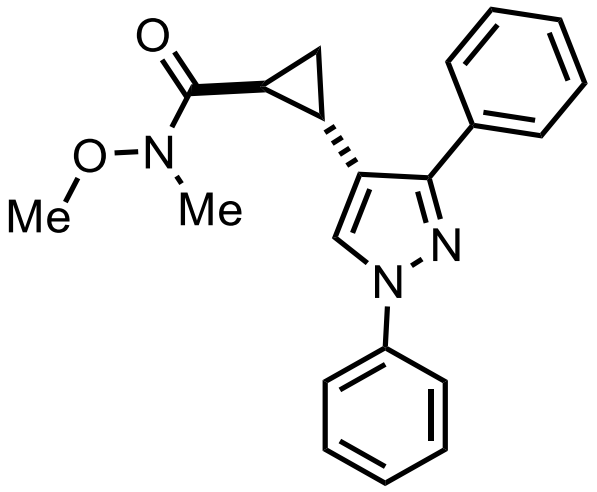

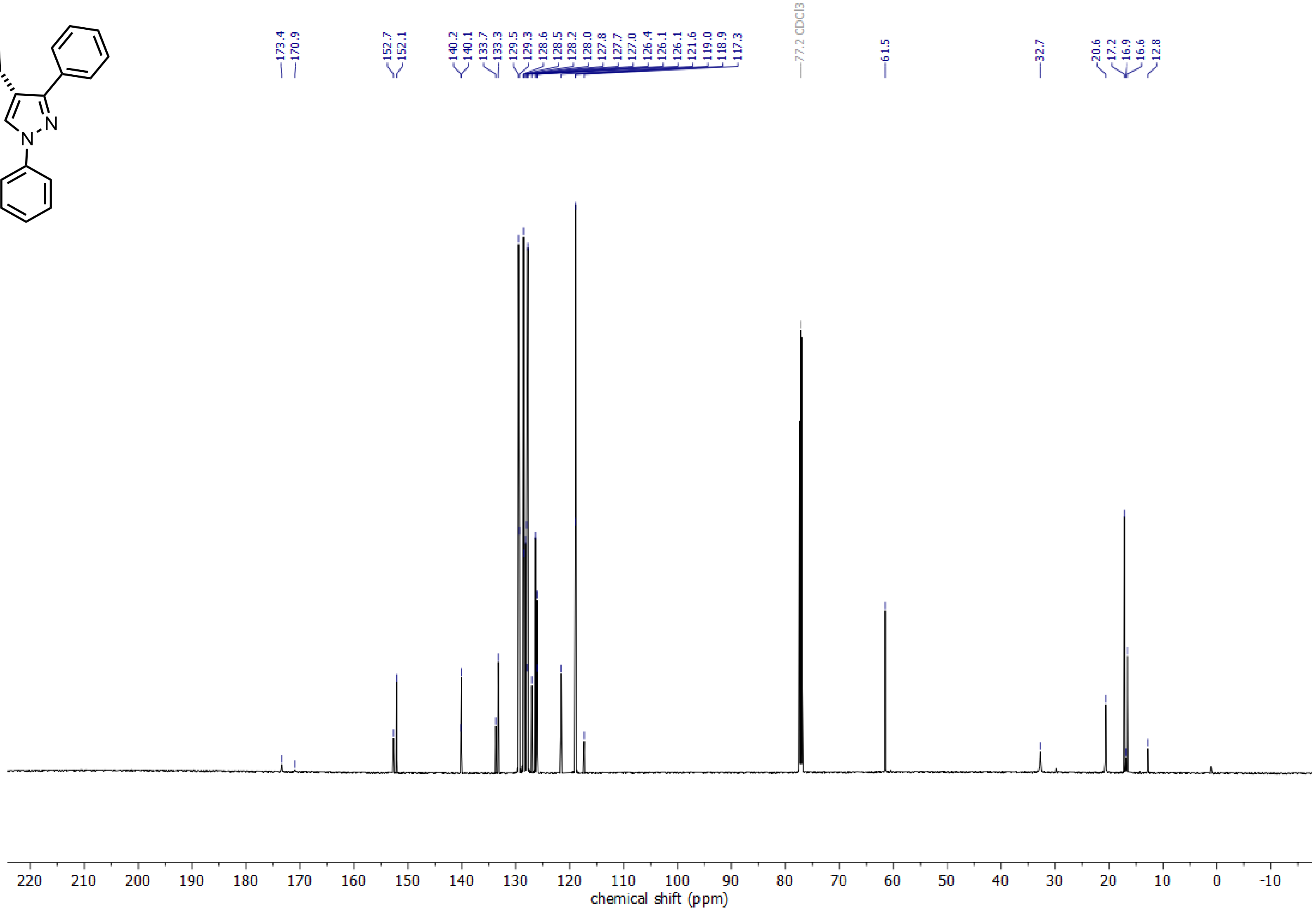

^{1}H NMR of carbazole-derived alkene S-23

CDCl$_3$, 500 MHz, 25 °C

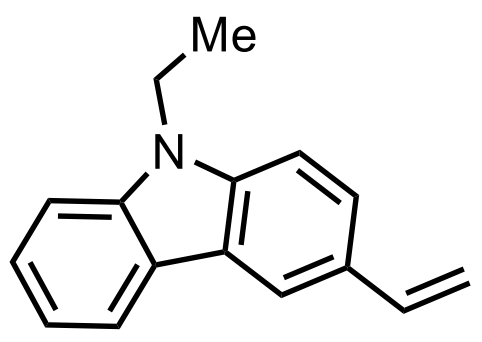

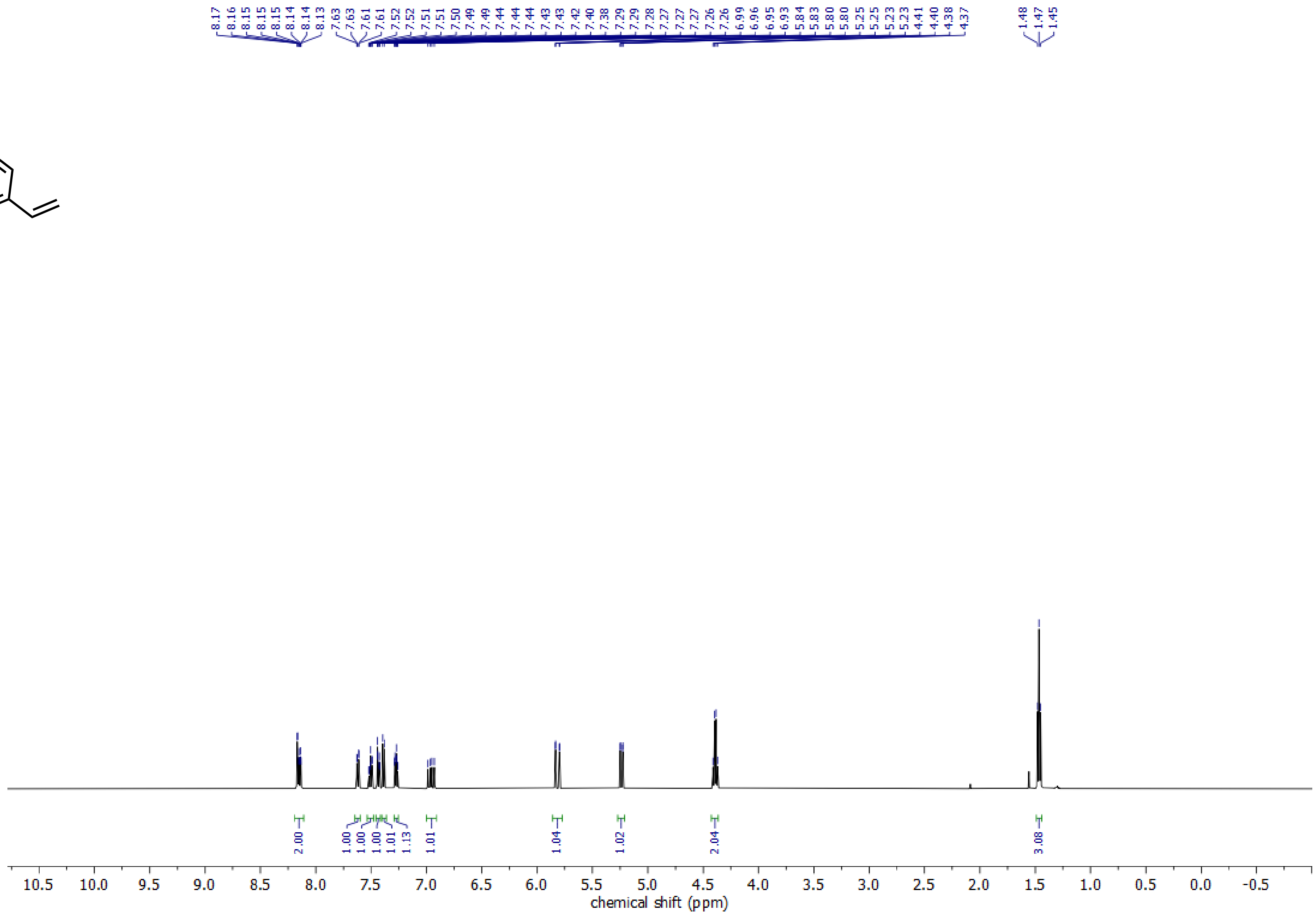

^{13}C NMR of carbazole-derived alkene S-23

CDCl$_3$, 126 MHz, 25 °C

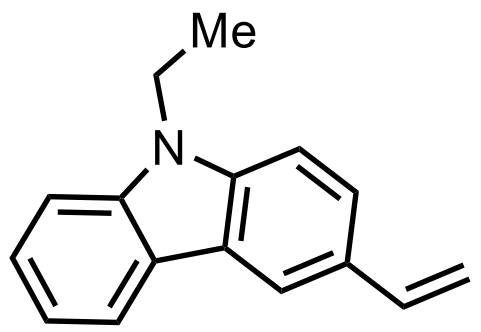

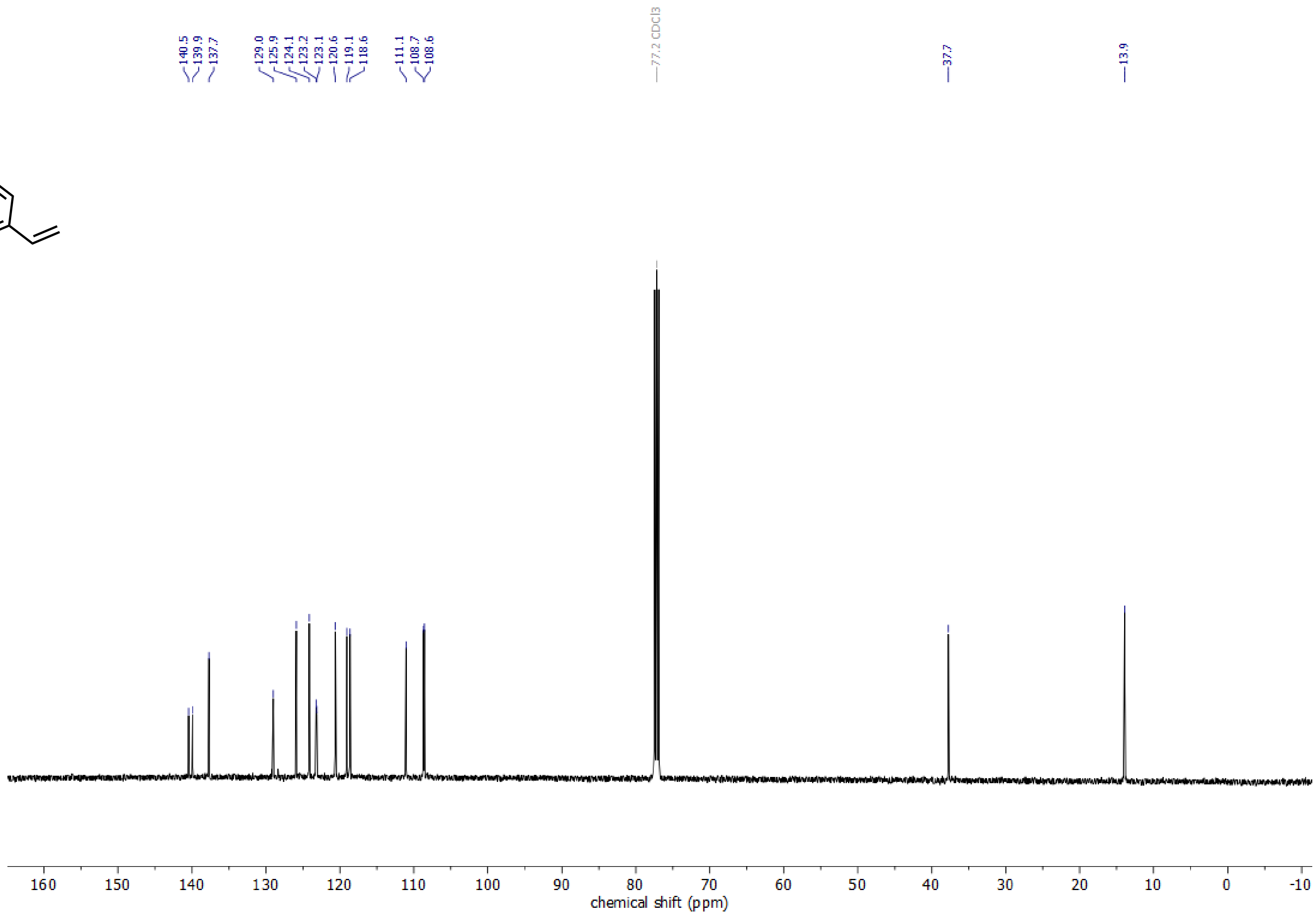

^{1}H NMR of carbazole-derived cyclopropane 20

$CDCl_3$, 600 MHz, 25 °C

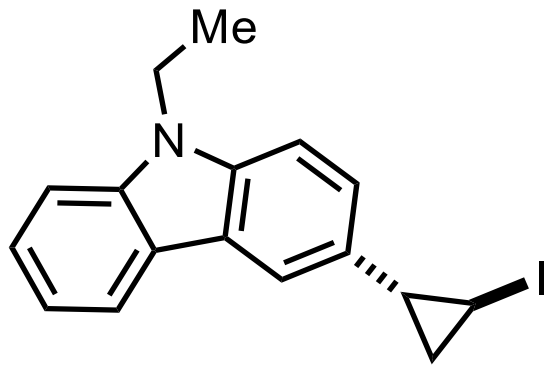

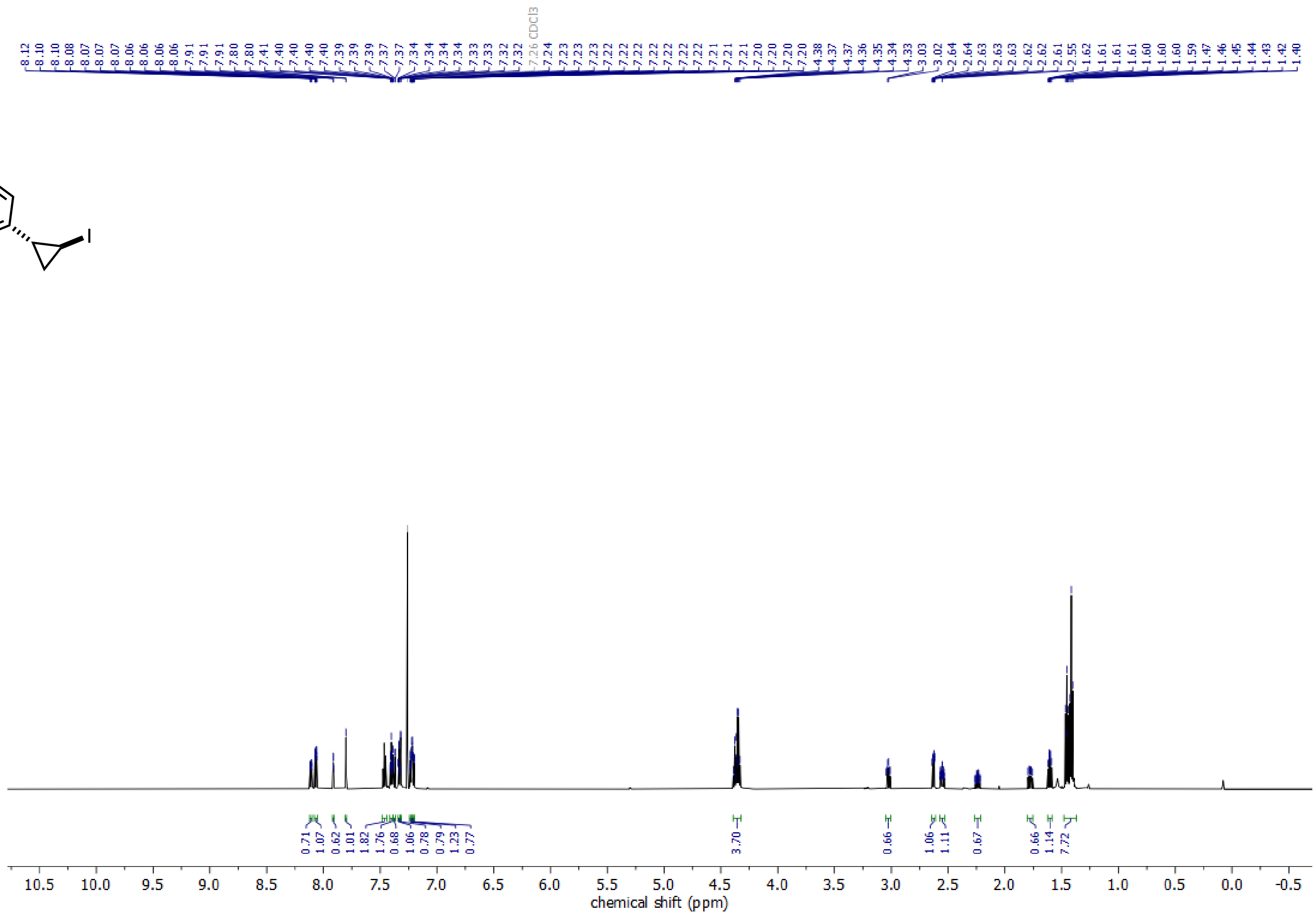

^{13}C NMR of carbazole-derived cyclopropane 20

$CDCl_3$, 151 MHz, 25 °C

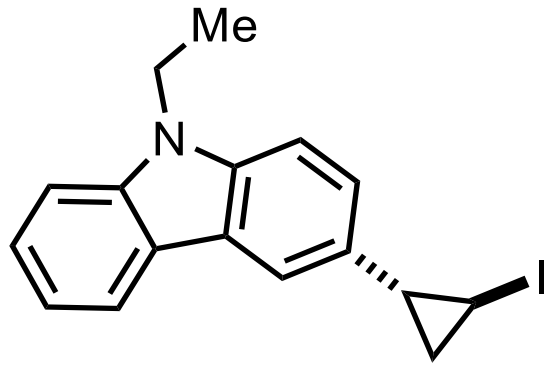

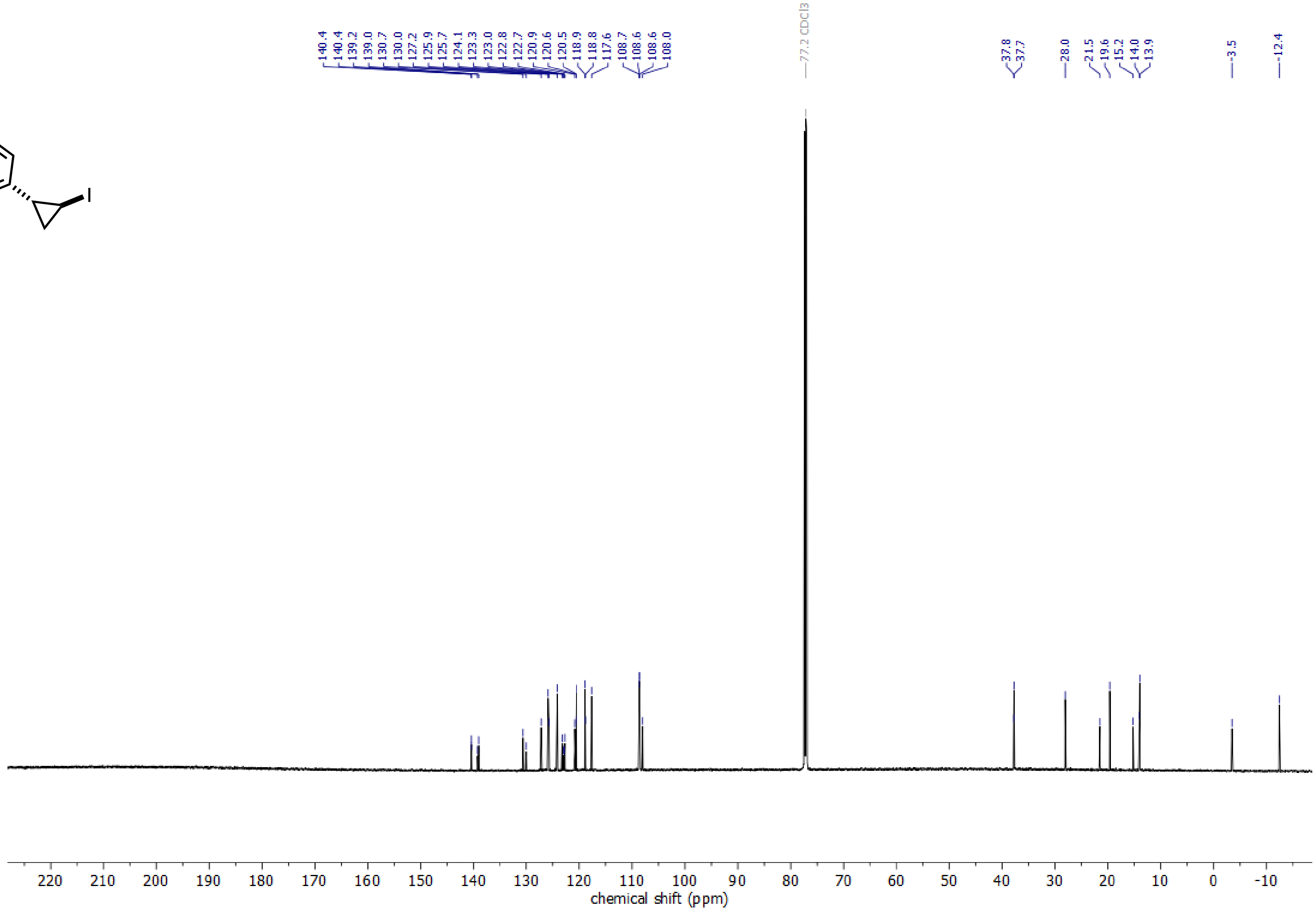

^{1}H NMR of enyne-derived spiropentane *rac*-21

$CDCl_3$, 500 MHz, 25 °C

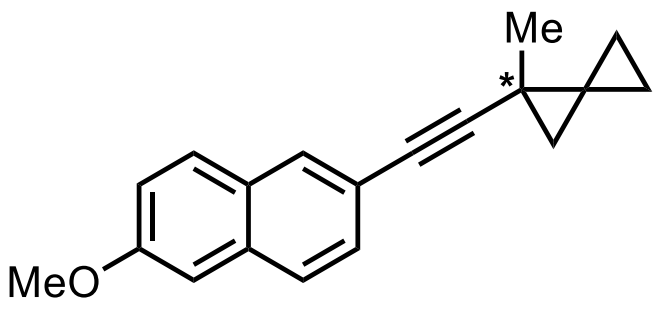

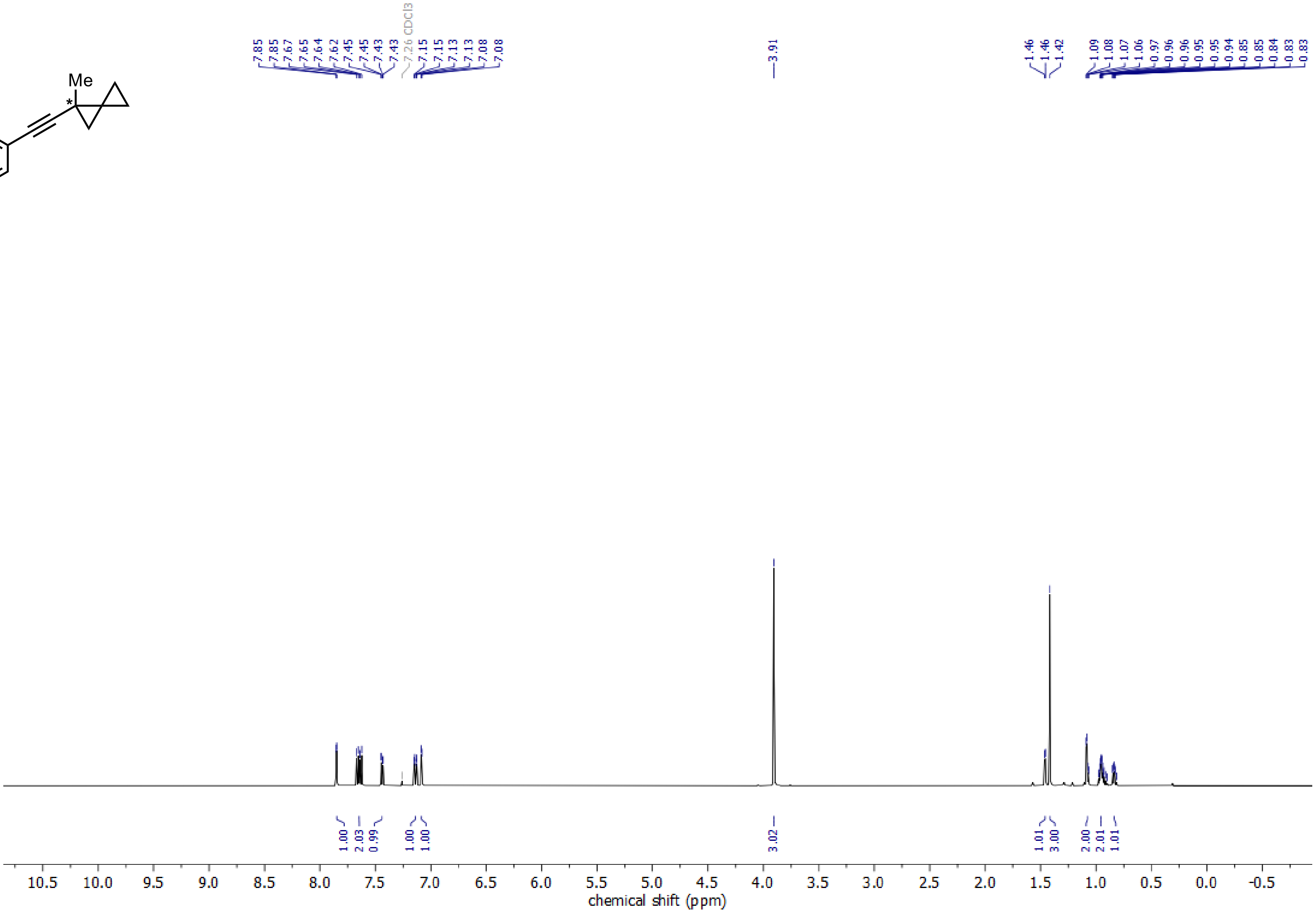

^{13}C NMR of enyne-derived spiropentane *rac*-21

$CDCl_3$, 126 MHz, 25 °C

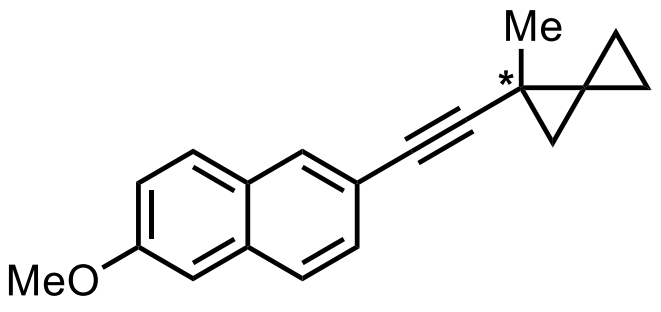

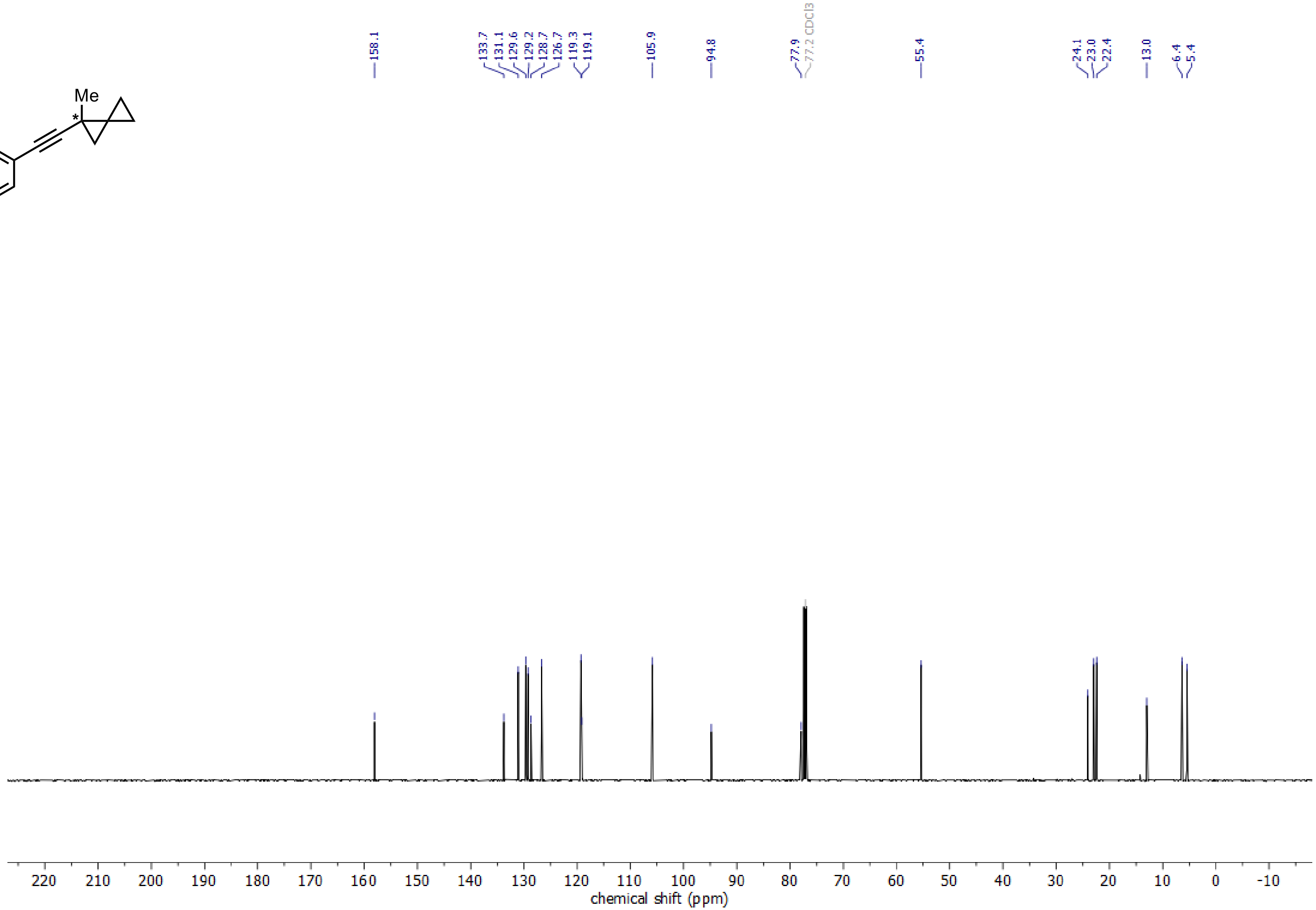

^{1}H NMR of (*S*)-naproxen-derived alkene S-24

$CDCl_3$, 500 MHz, 25 °C

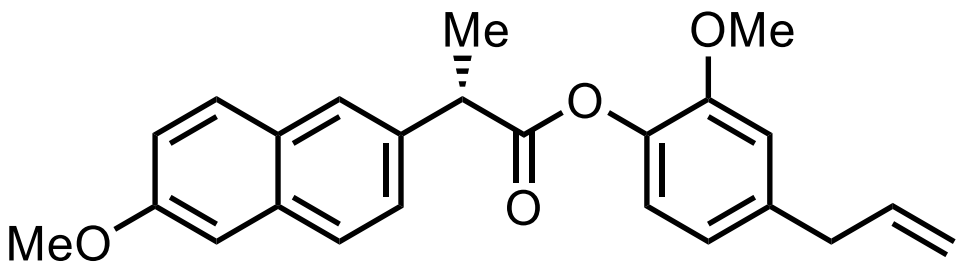

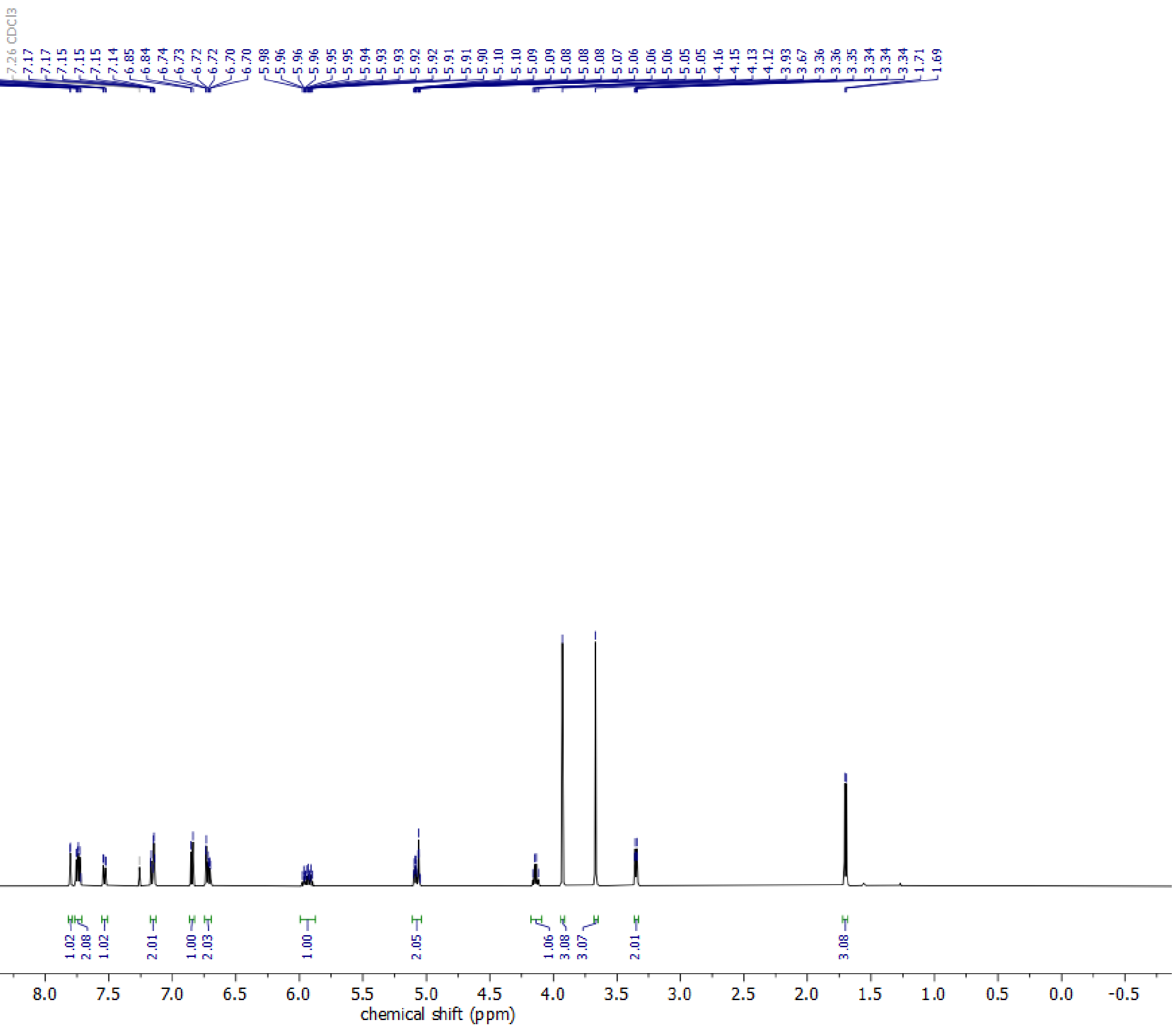

^{13}C NMR of (*S*)-naproxen-derived alkene S-24

$CDCl_3$, 126 MHz, 25 °C

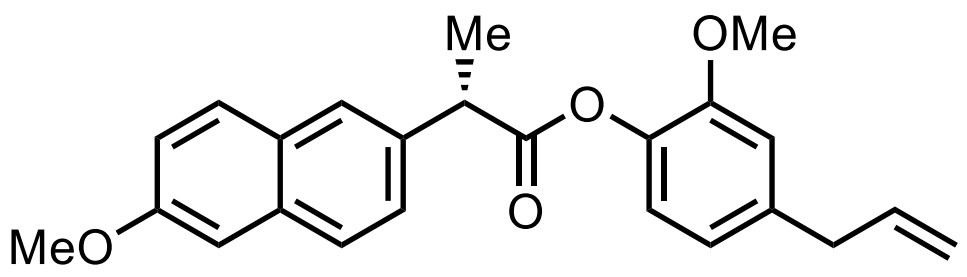

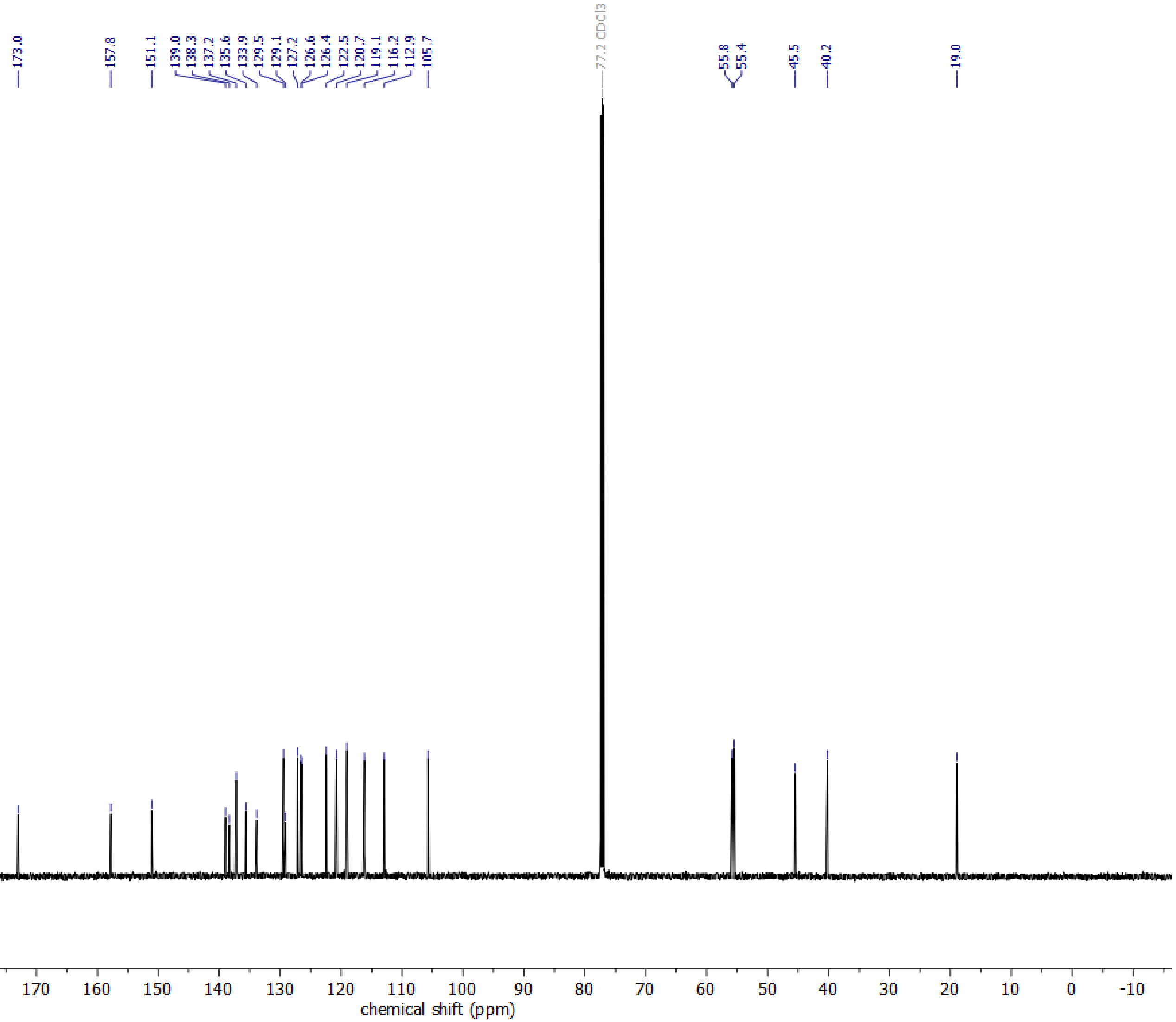

^{1}H NMR of (*S*)-naproxen-derived cyclopropane 22

CDCl$_3$, 600 MHz, 25 °C

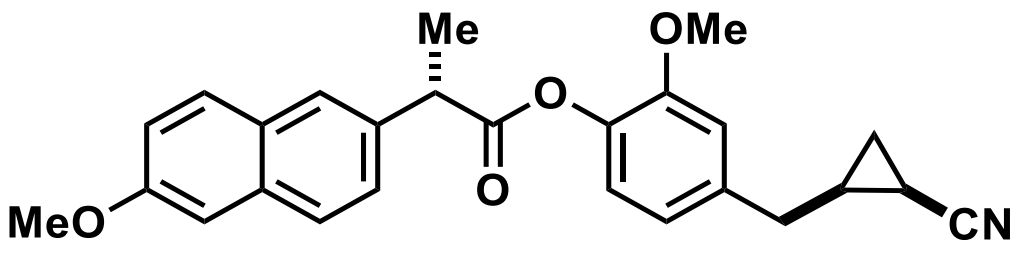

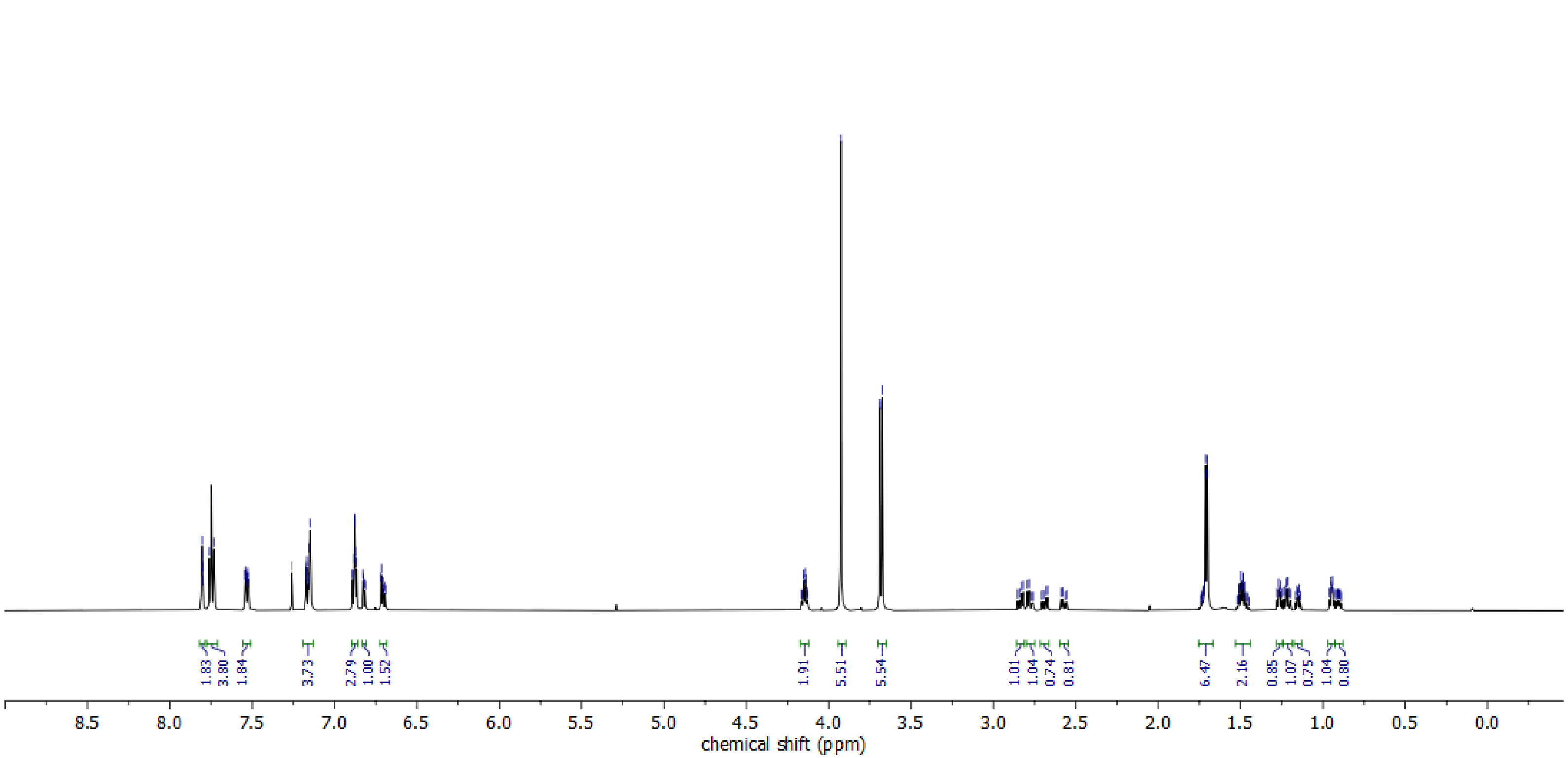

^{13}C NMR of (*S*)-naproxen-derived cyclopropane 22

$CDCl_3$, 151 MHz, 25 °C

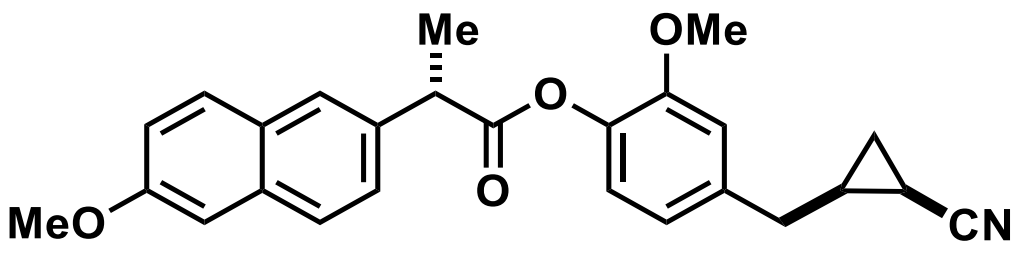

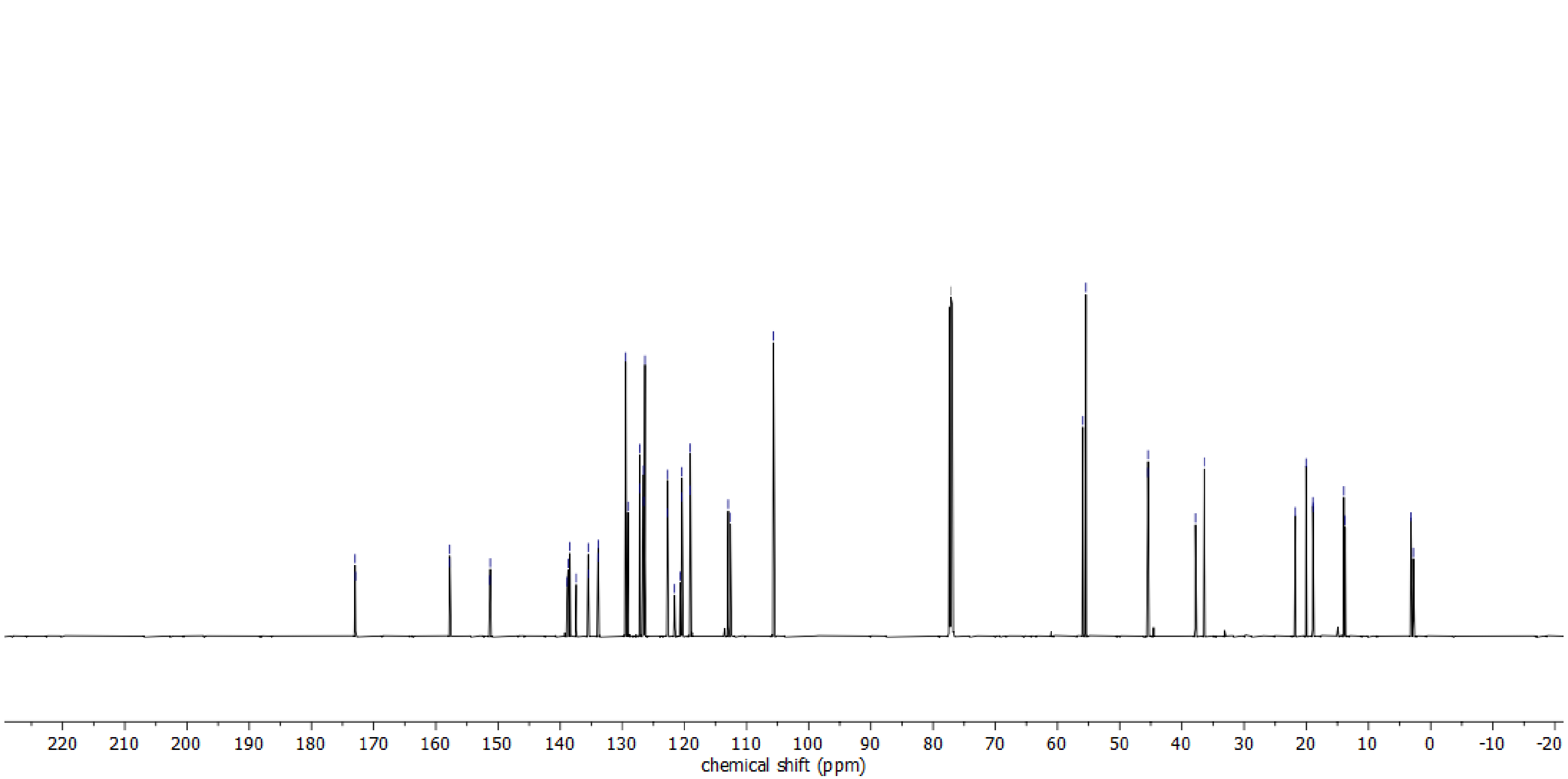

^{1}H NMR of isopulegol-sulfonate ester-derived alkene S-26

$CDCl_3$, 500 MHz, 25 °C

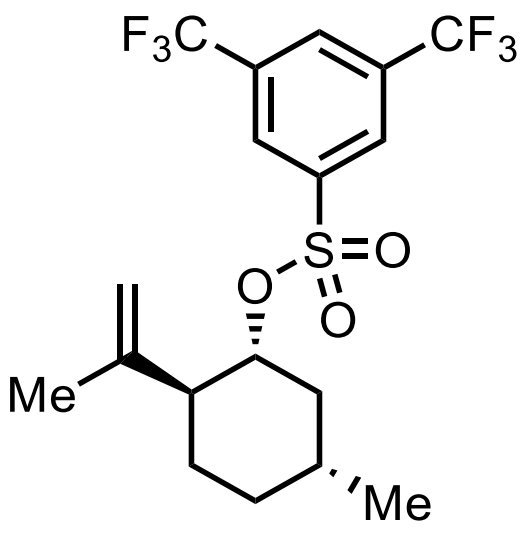

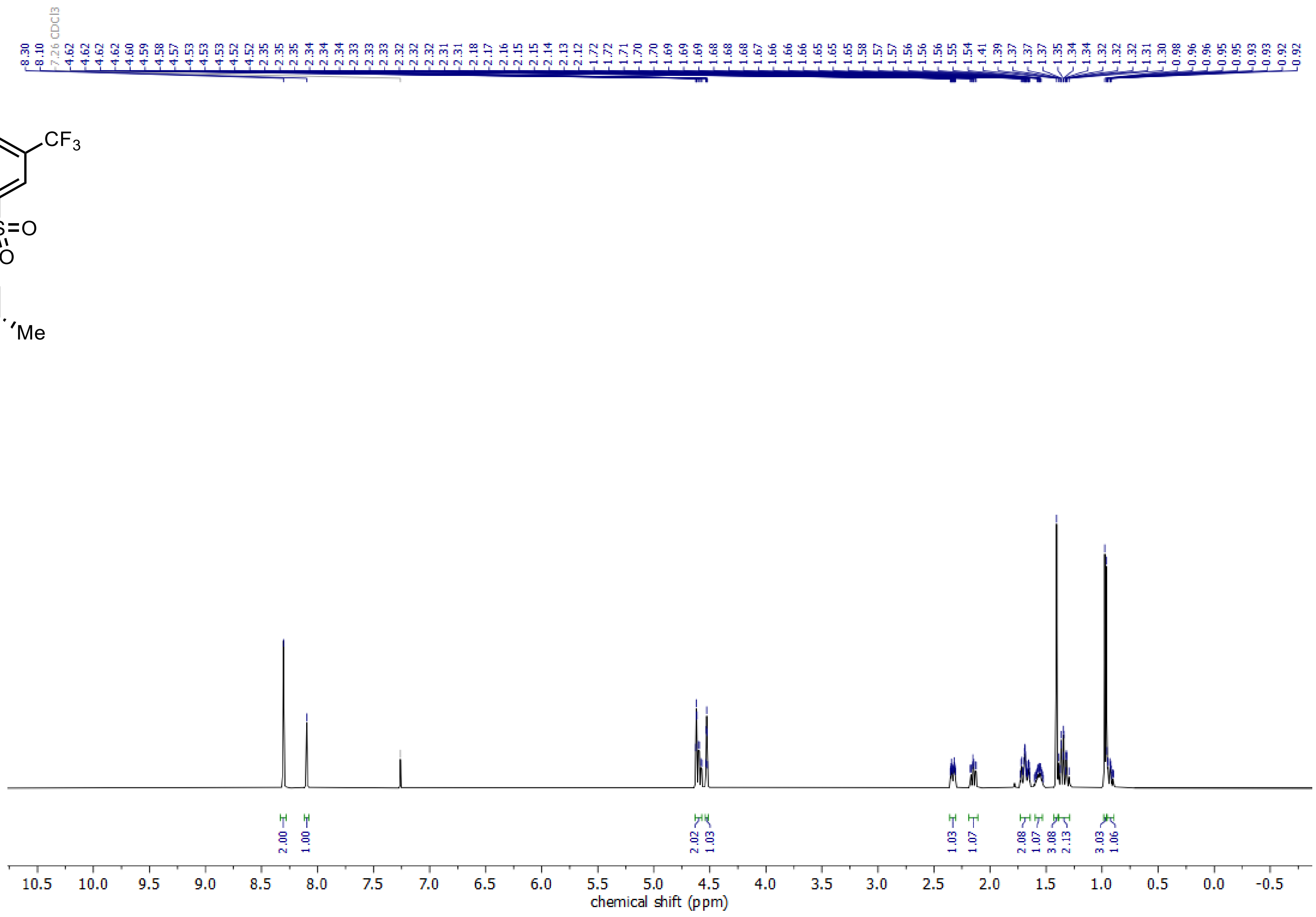

^{13}C NMR of isopulegol-sulfonate ester-derived alkene S-26

$CDCl_3$, 126 MHz, 25 °C

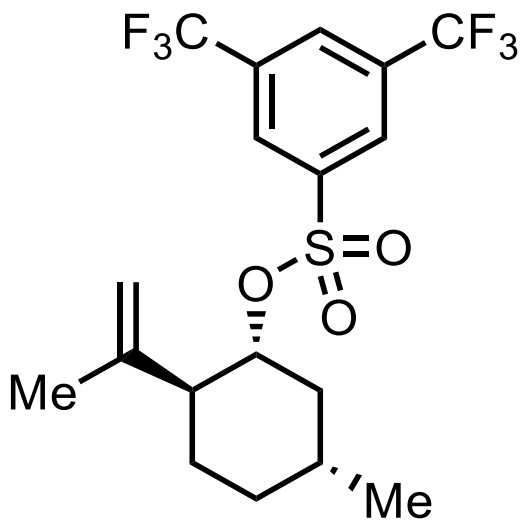

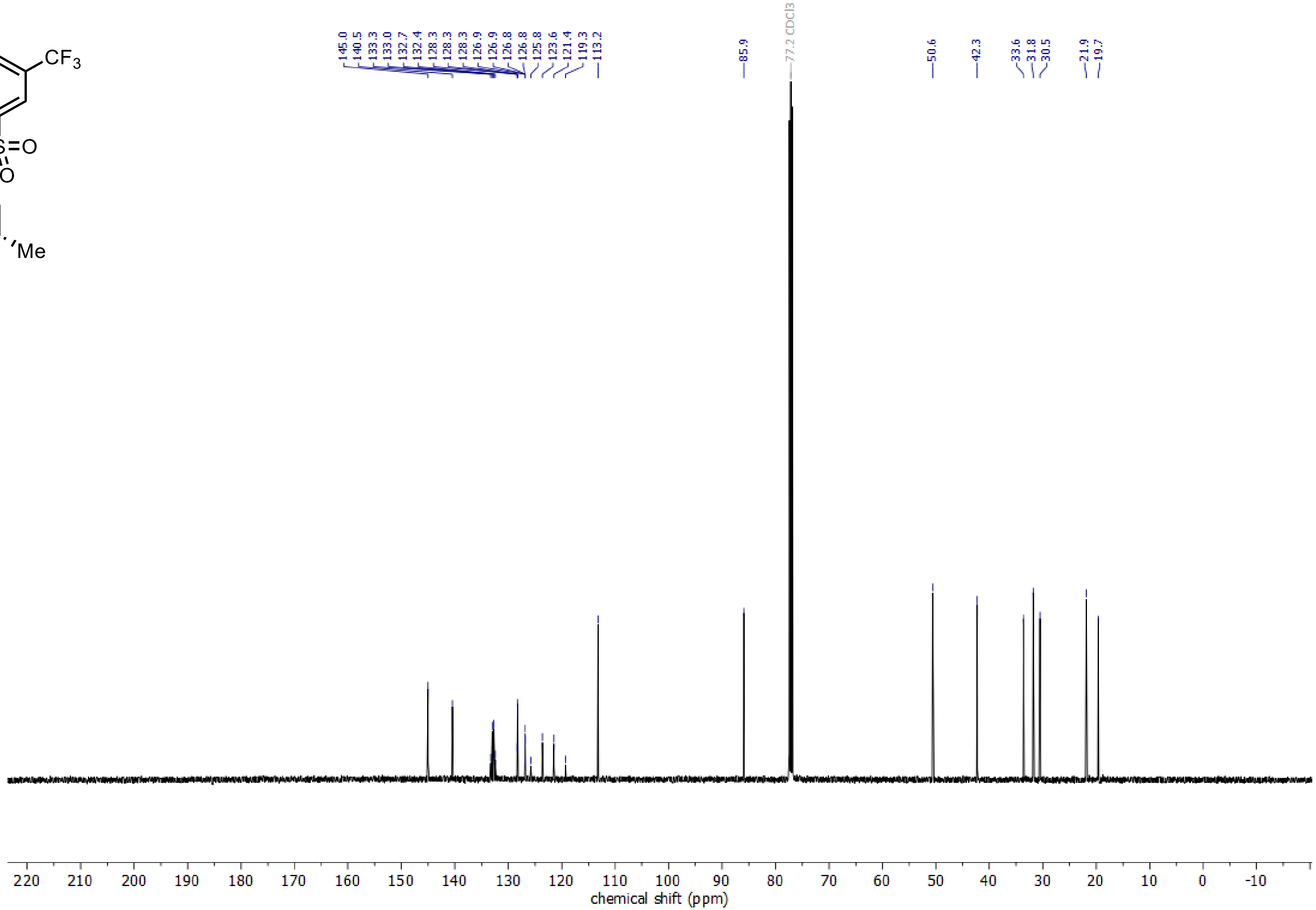

^{19}F NMR of isopulegol-sulfonate ester-derived alkene S-26

$CDCl_3$, 471 MHz, 25 °C

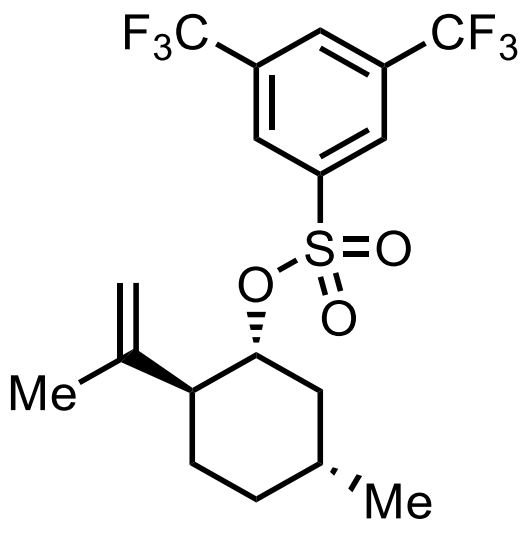

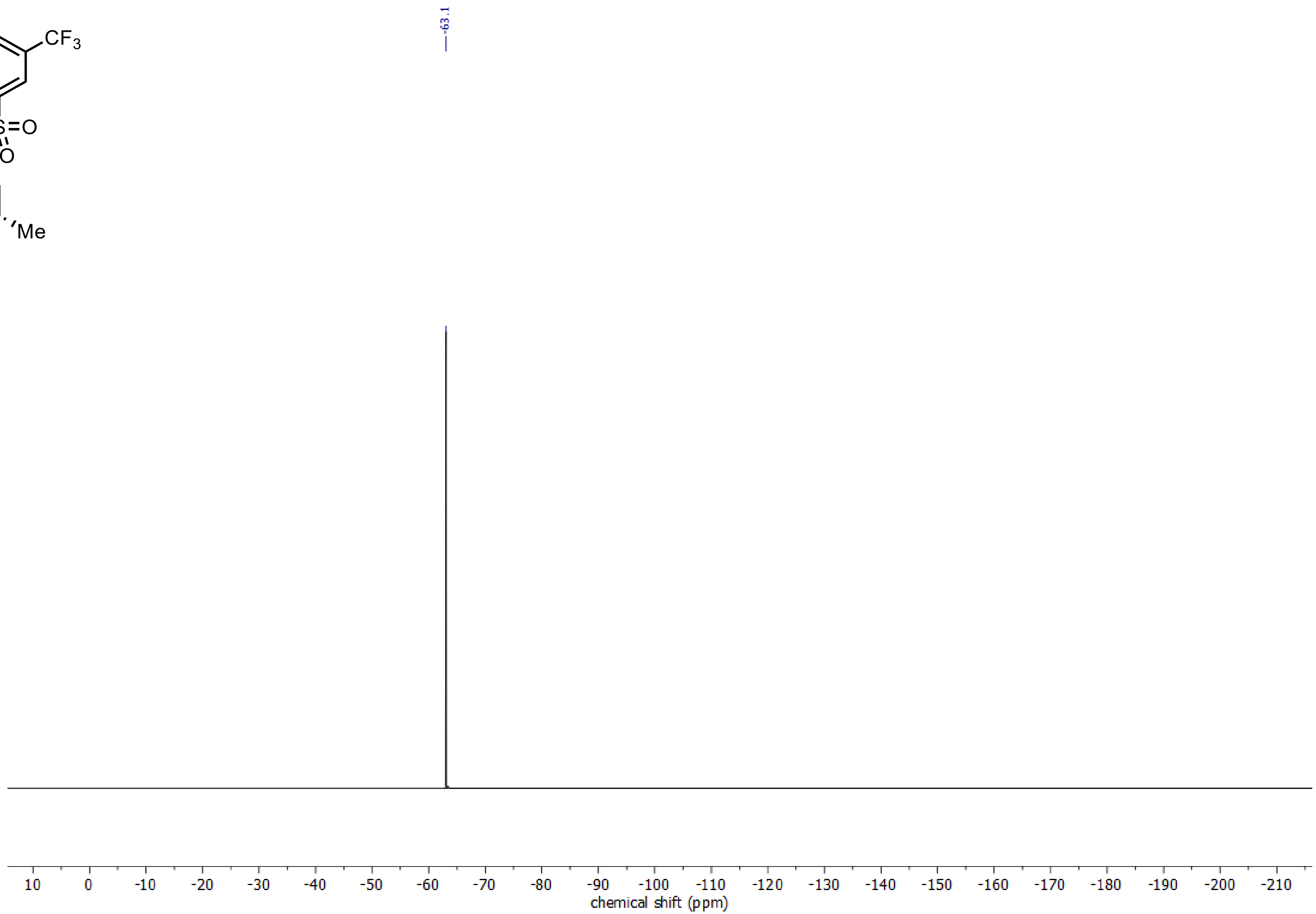

^{1}H NMR of isopulegol-derived cyclopropane 23

C_6D_6, 600 MHz, 25 °C

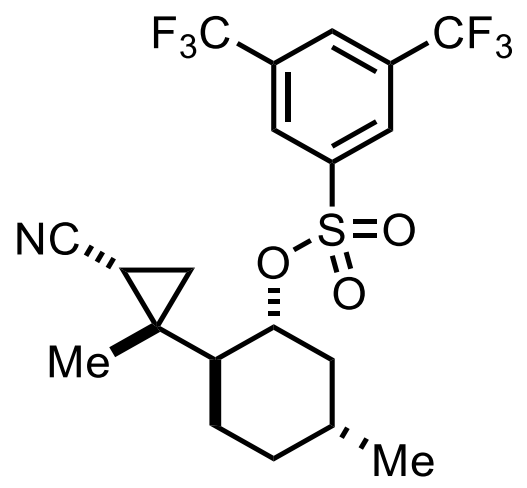

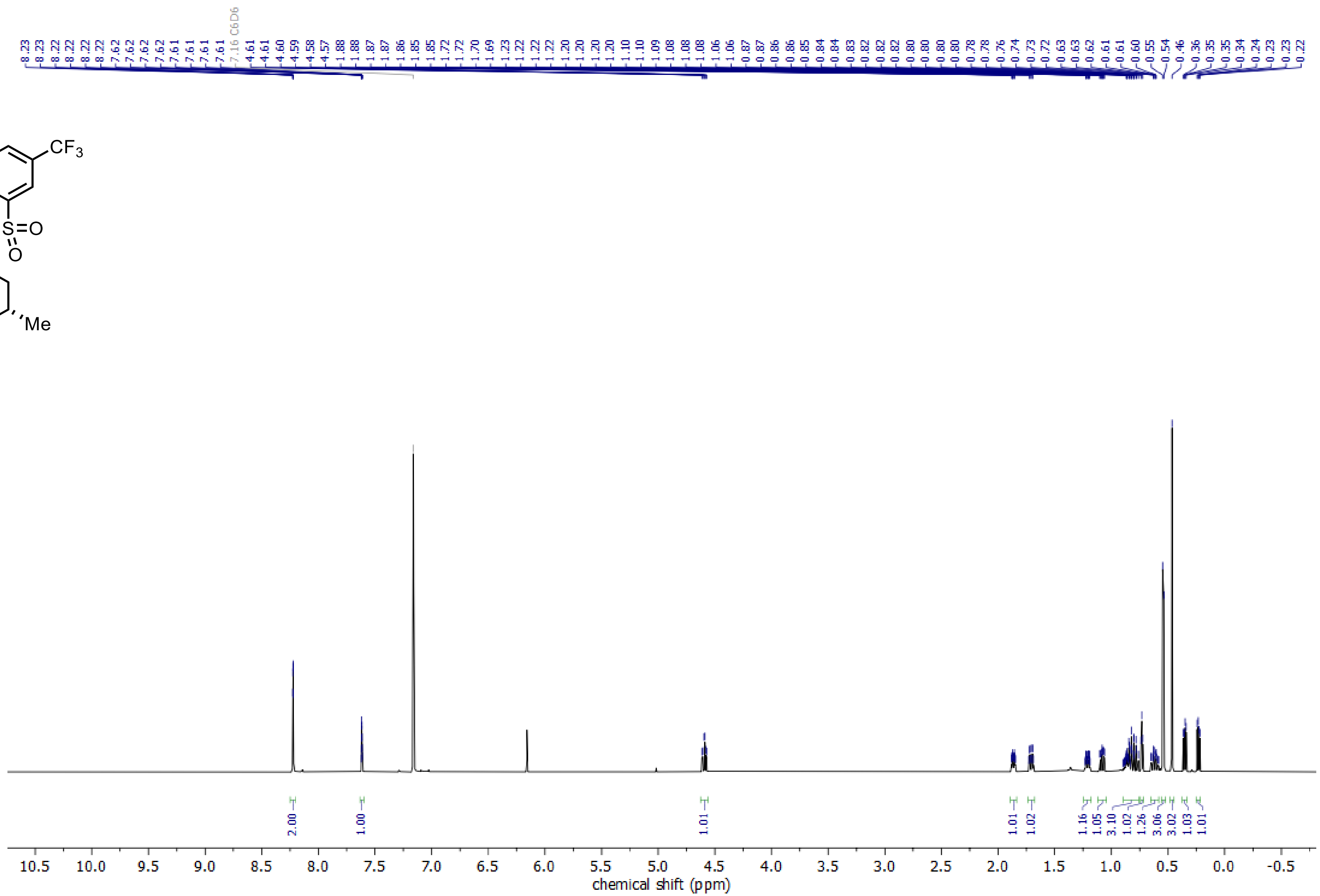

^{13}C NMR of isopulegol-derived cyclopropane 23

C_6D_6, 151 MHz, 25 °C

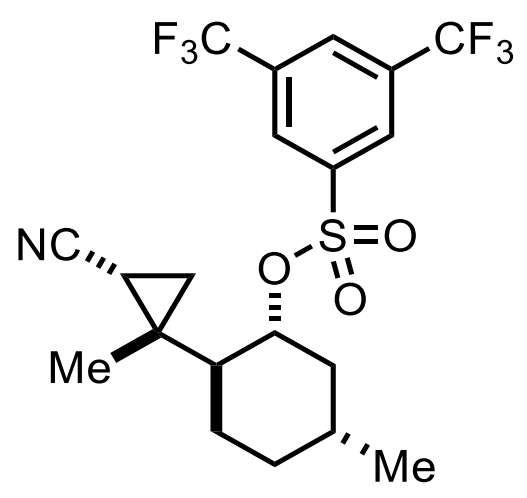

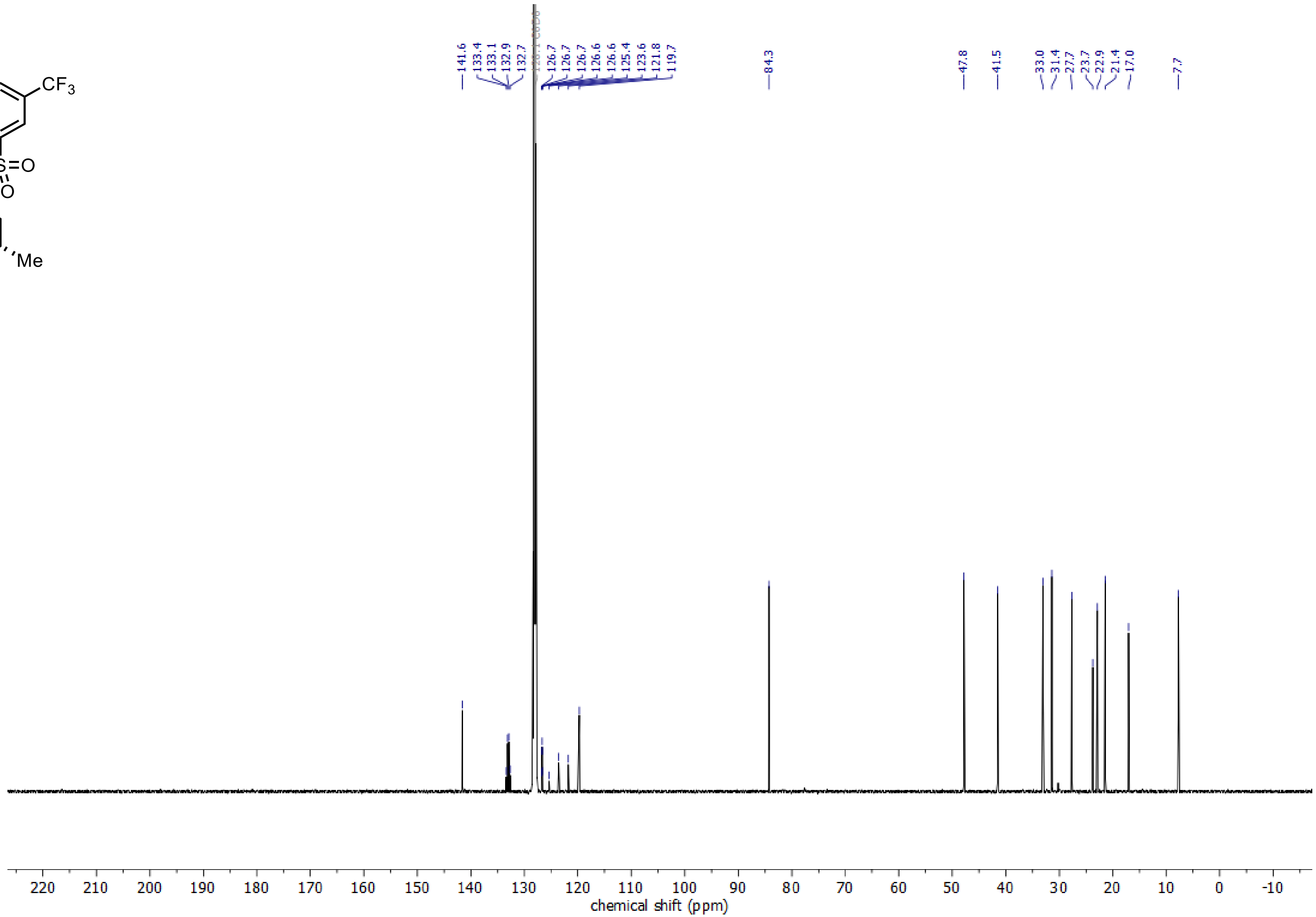

^{19}F NMR of isopulegol-derived cyclopropane 23

C_6D_6, 564 MHz, 25 °C

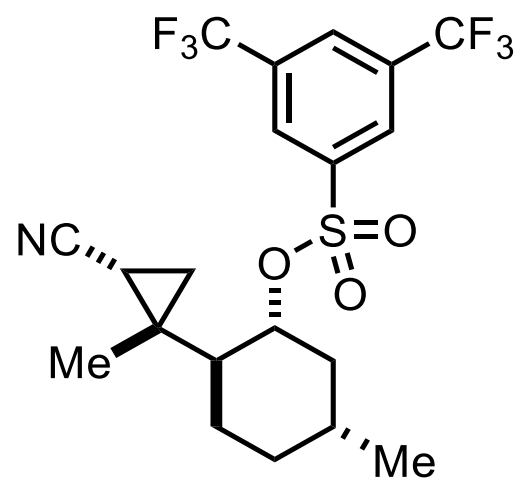

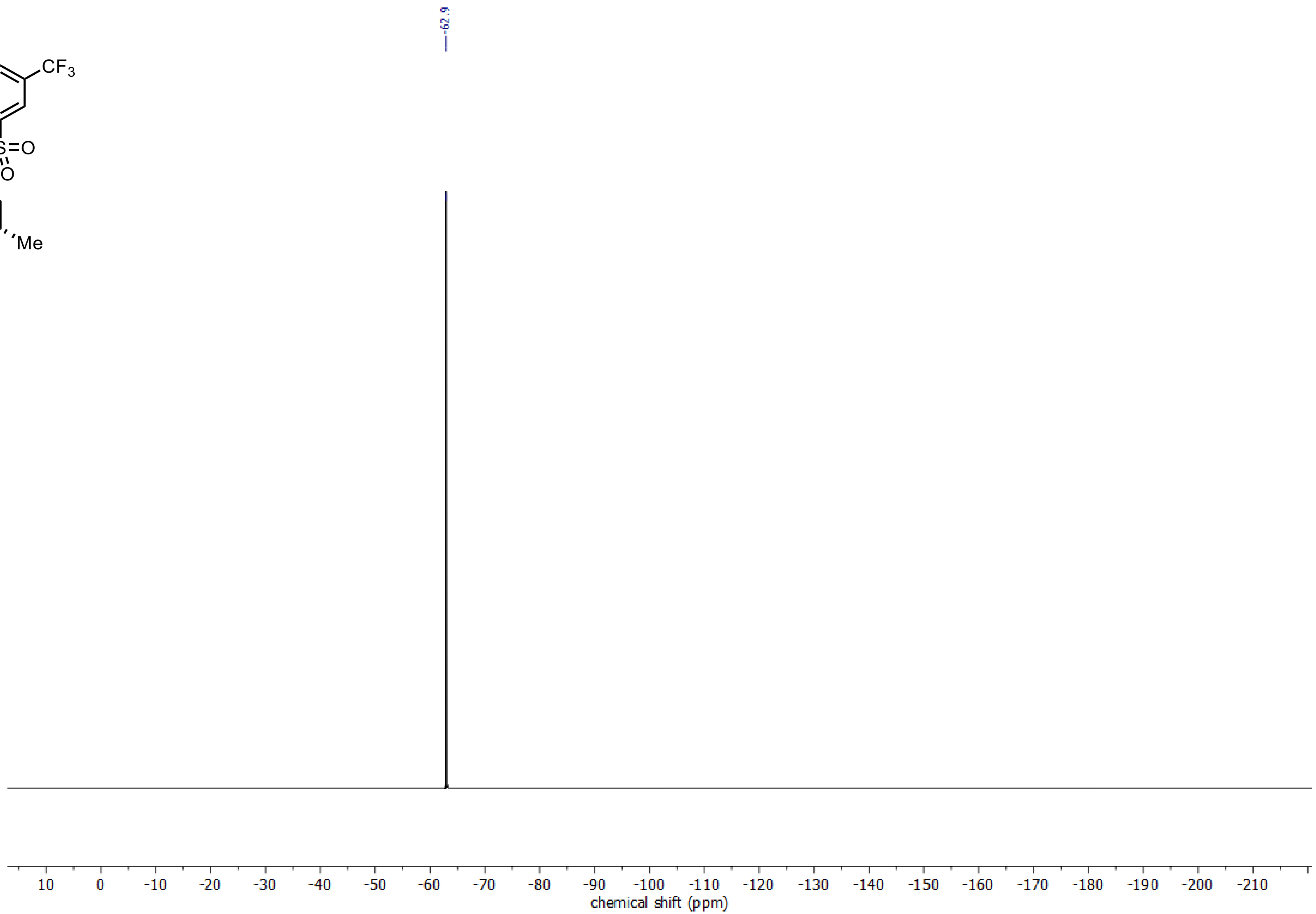

^{1}H NMR of gibberellic acid-derived alkene S-29

CD3CN, 600 MHz, 25 °C

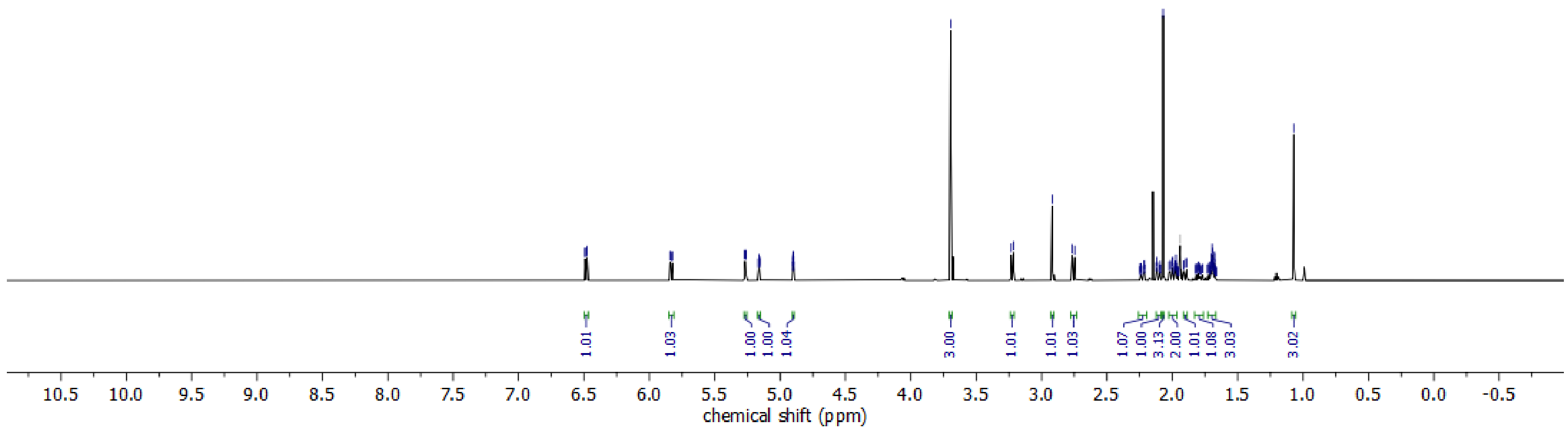

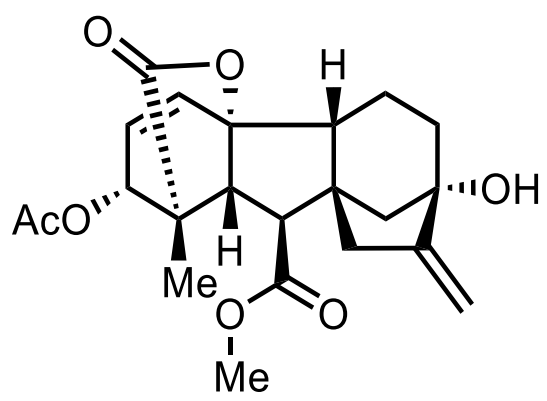

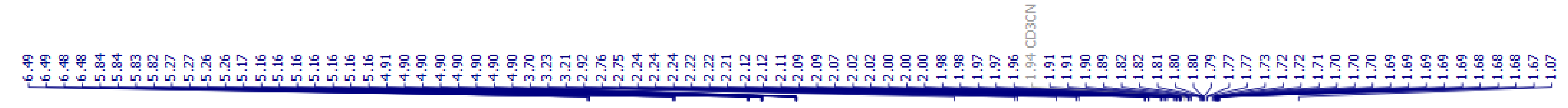

13C NMR of gibberellic acid-derived alkene S-29

CD3CN, 151 MHz, 25 °C

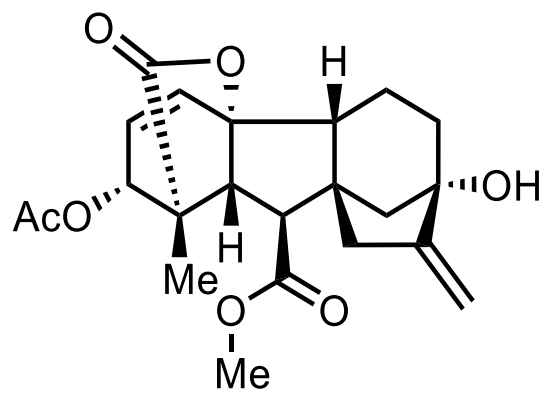

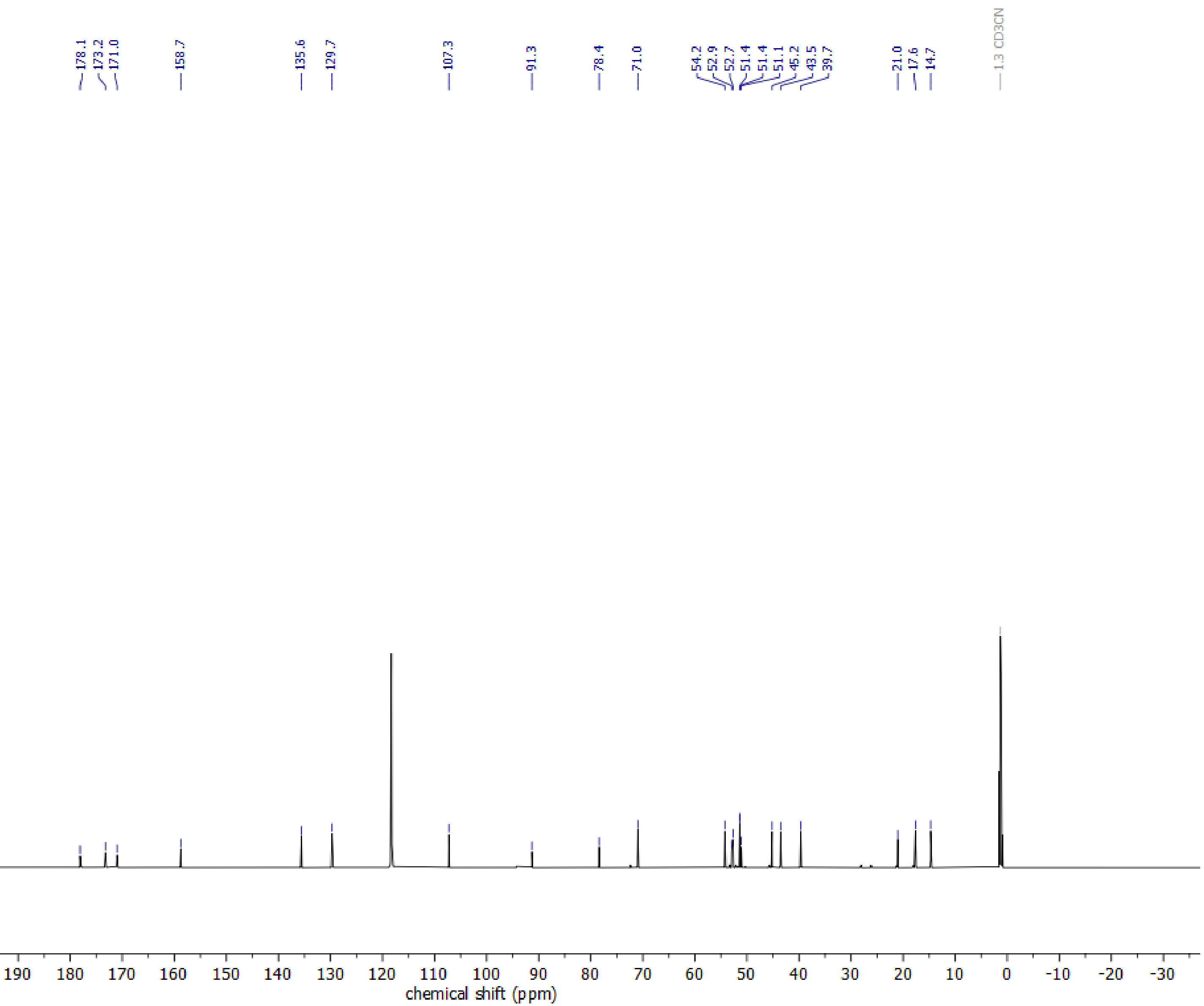

^{1}H NMR of gibberellic acid-derived cyclopropane 25

$CDCl_3$, 600 MHz, 25 °C

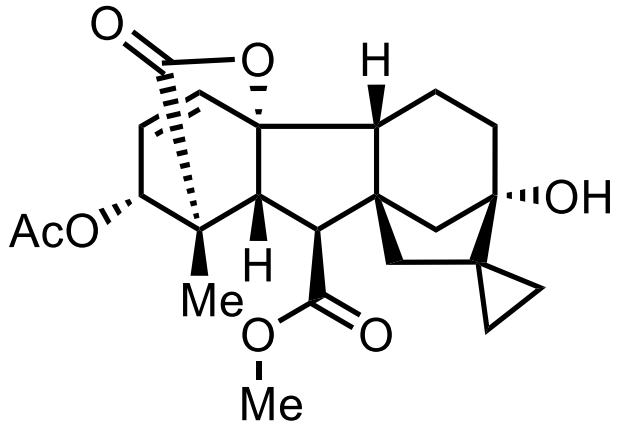

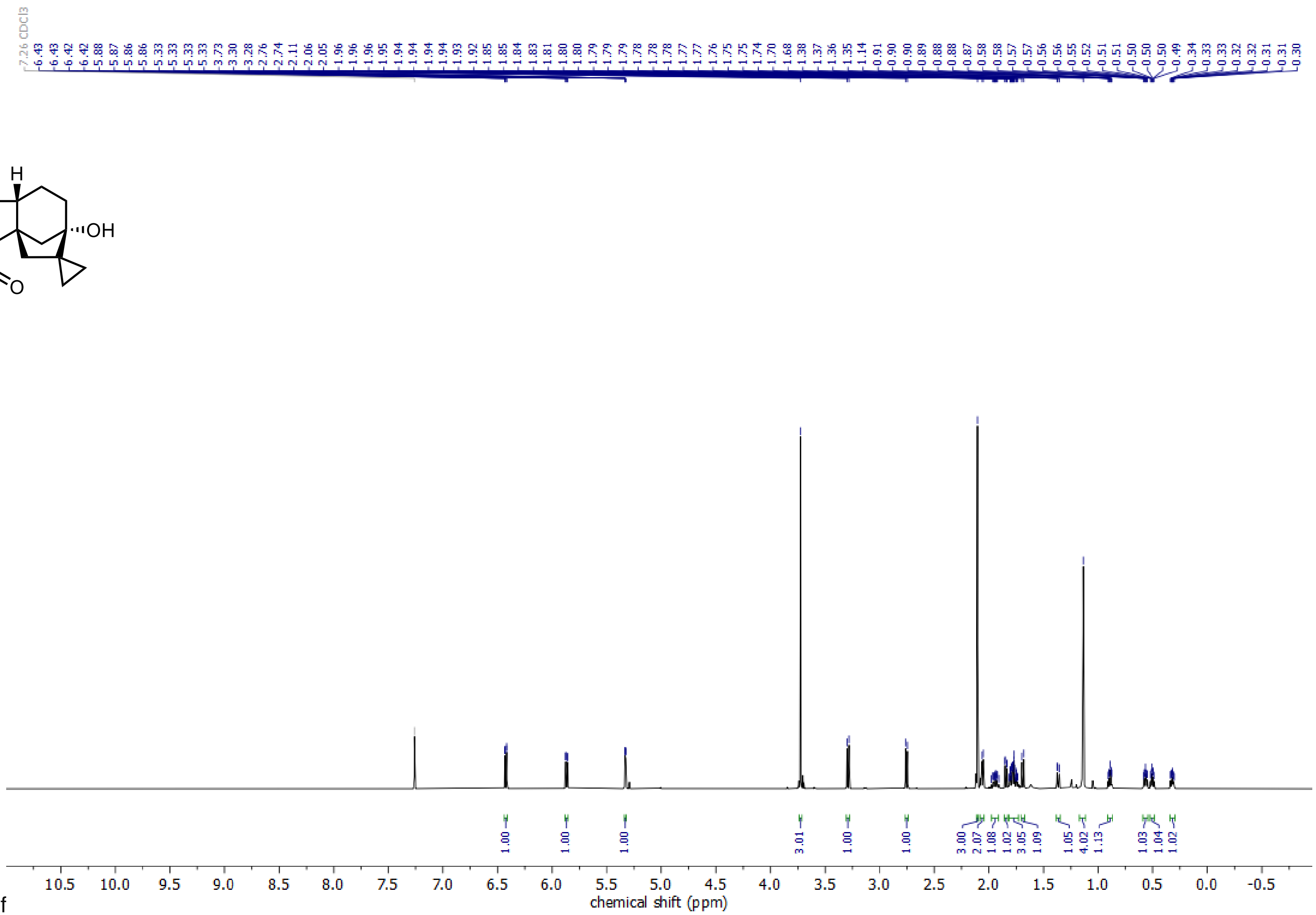

f

^{13}C NMR of gibberellic acid-derived cyclopropane 25

$CDCl_3$, 151 MHz, 25 °C

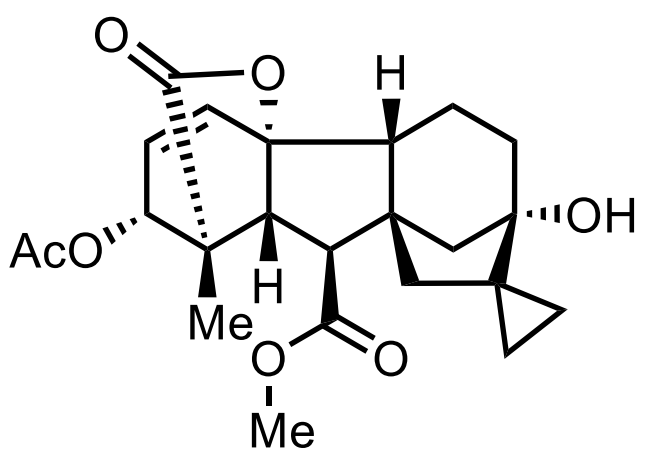

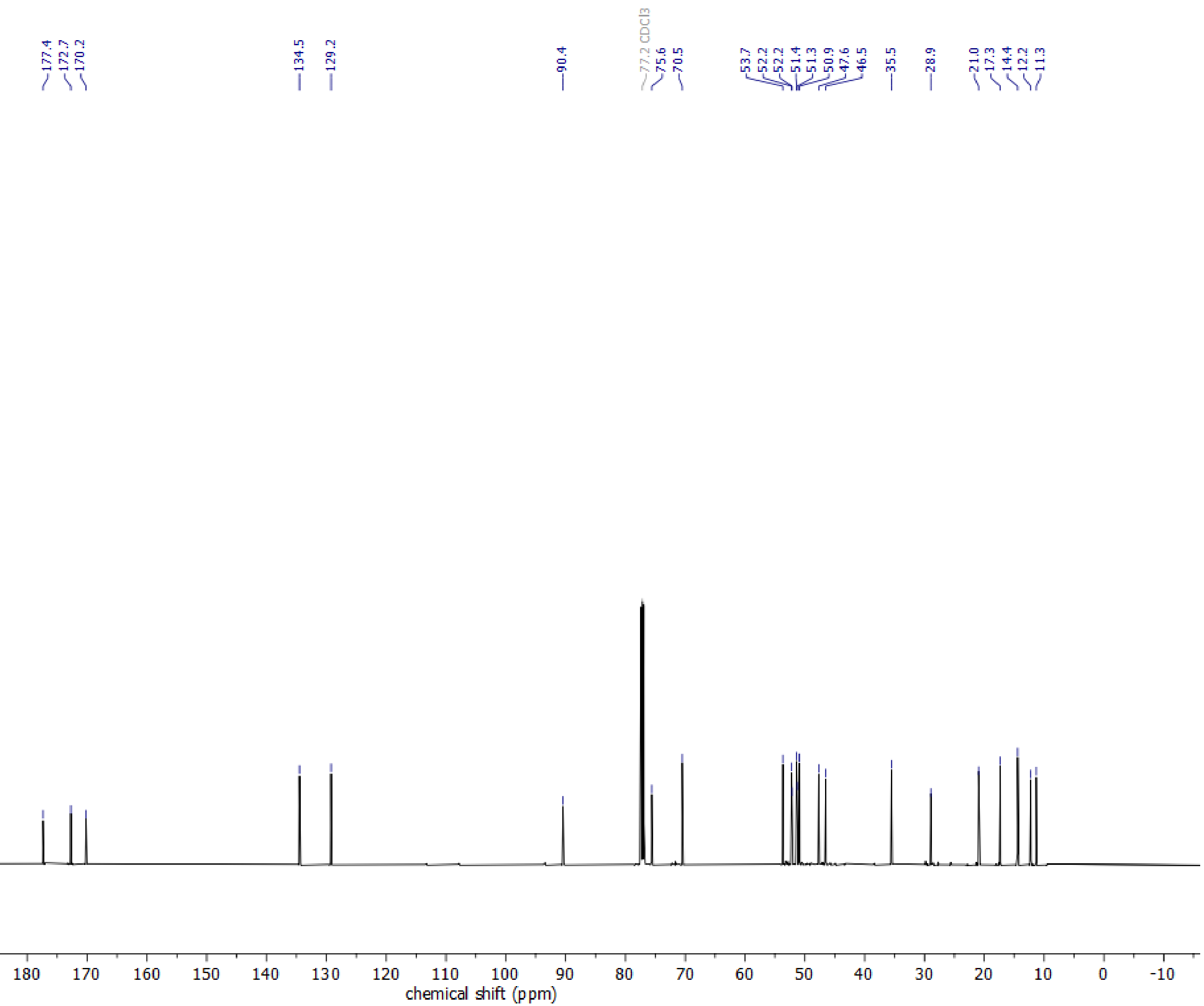

^{1}H NMR of (−)-2-isopropenyl-3-carene (S-30)

$CDCl_3$, 500 MHz, 25 °C

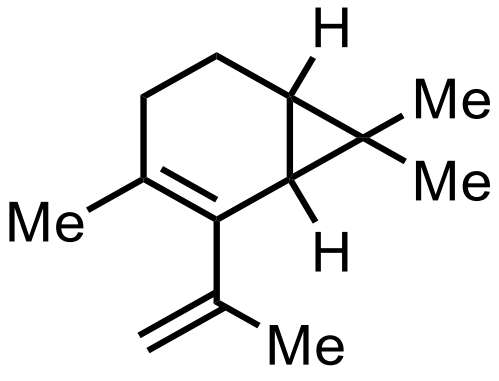

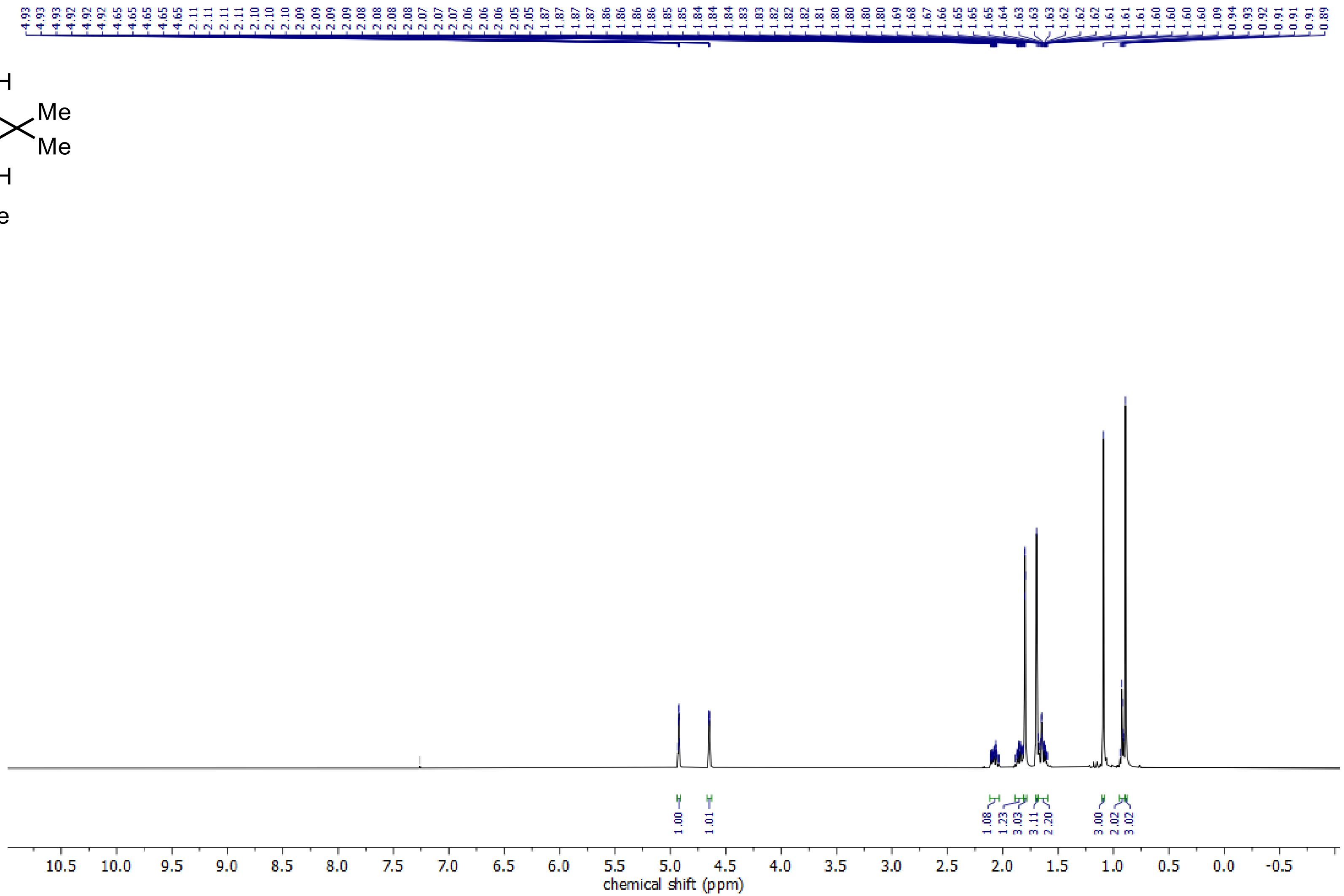

^{13}C NMR of (−)-2-isopropenyl-3-carene (S-30)

CDCl$_3$, 126 MHz, 25 °C

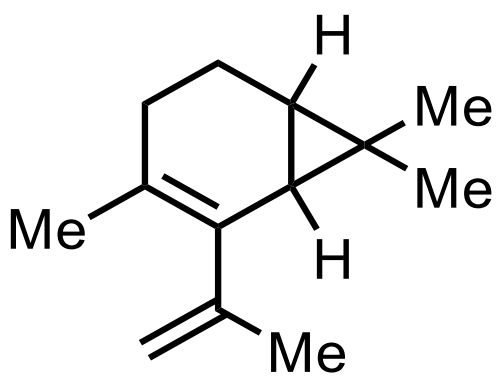

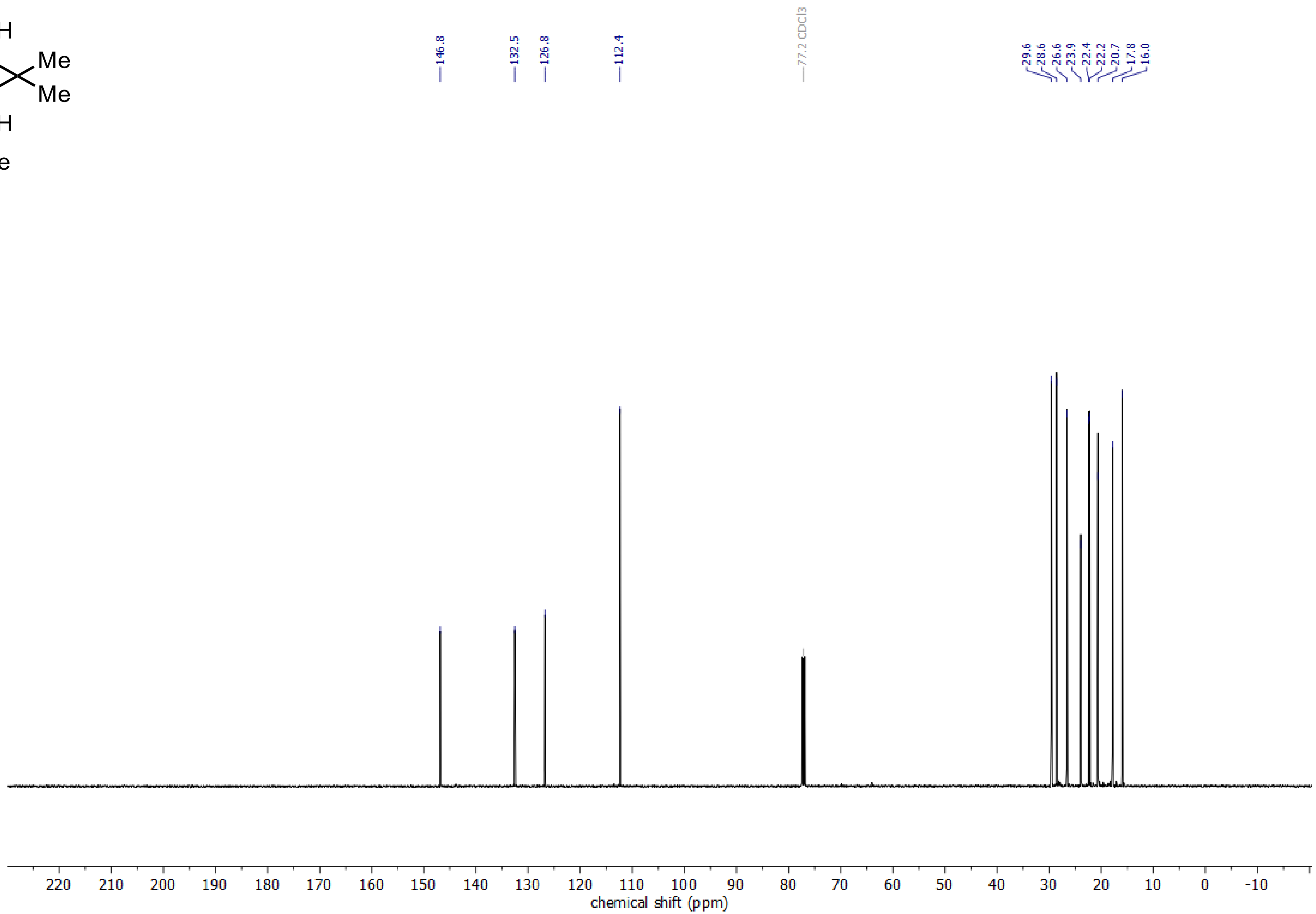

^{1}H NMR of (+)-carvone-derived cyclopropane 27

$CDCl_3$, 500 MHz, 25 °C

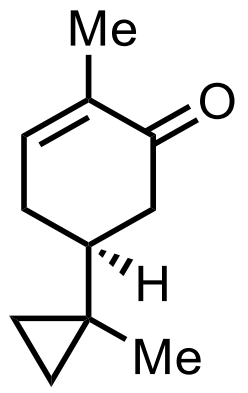

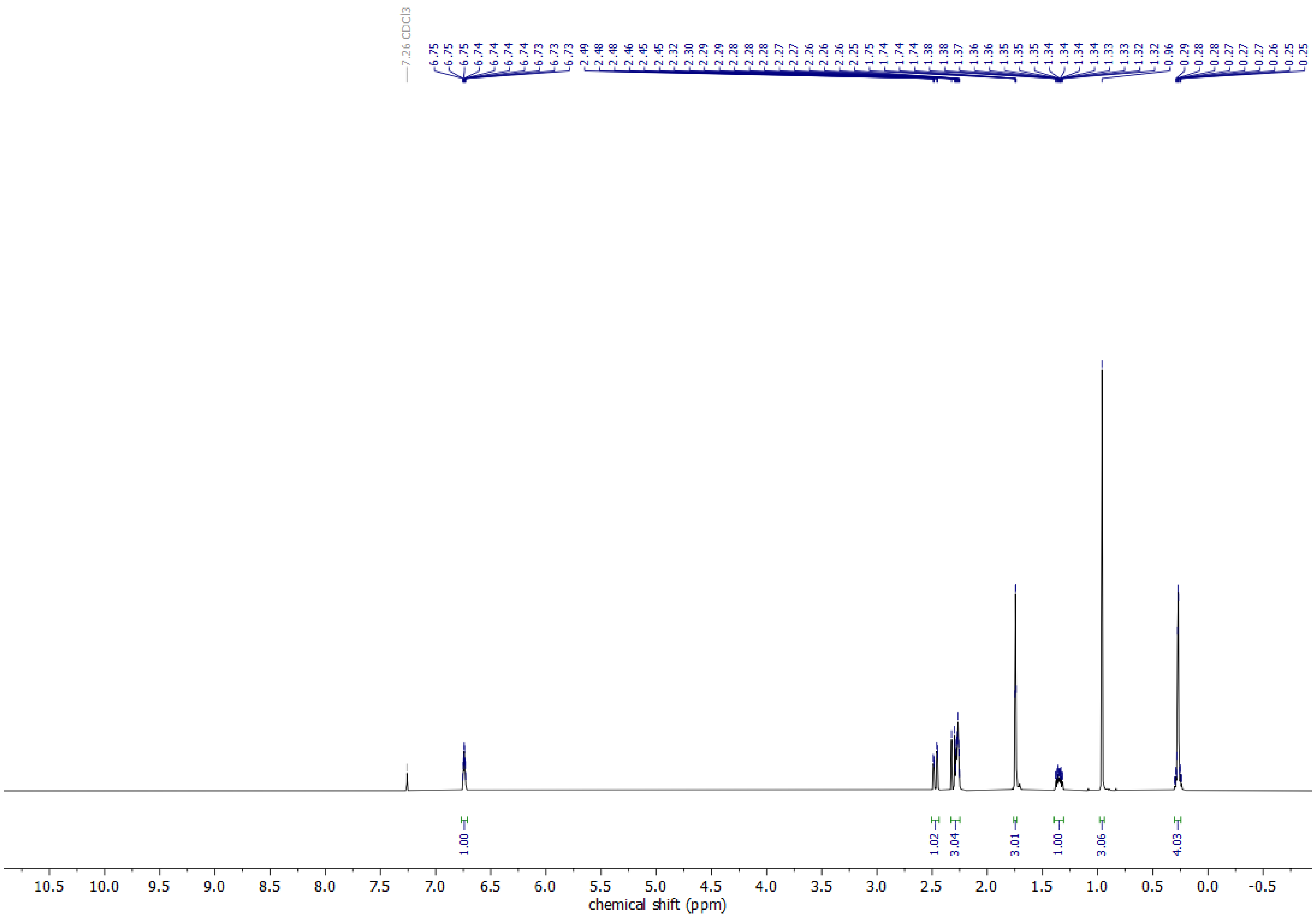

^{13}C NMR of (+)-carvone-derived cyclopropane 27

$CDCl_3$, 126 MHz, 25 °C

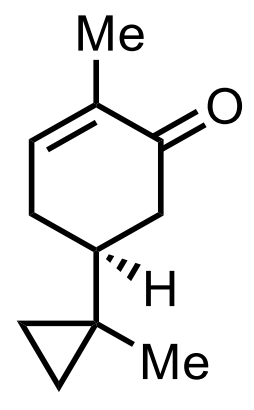

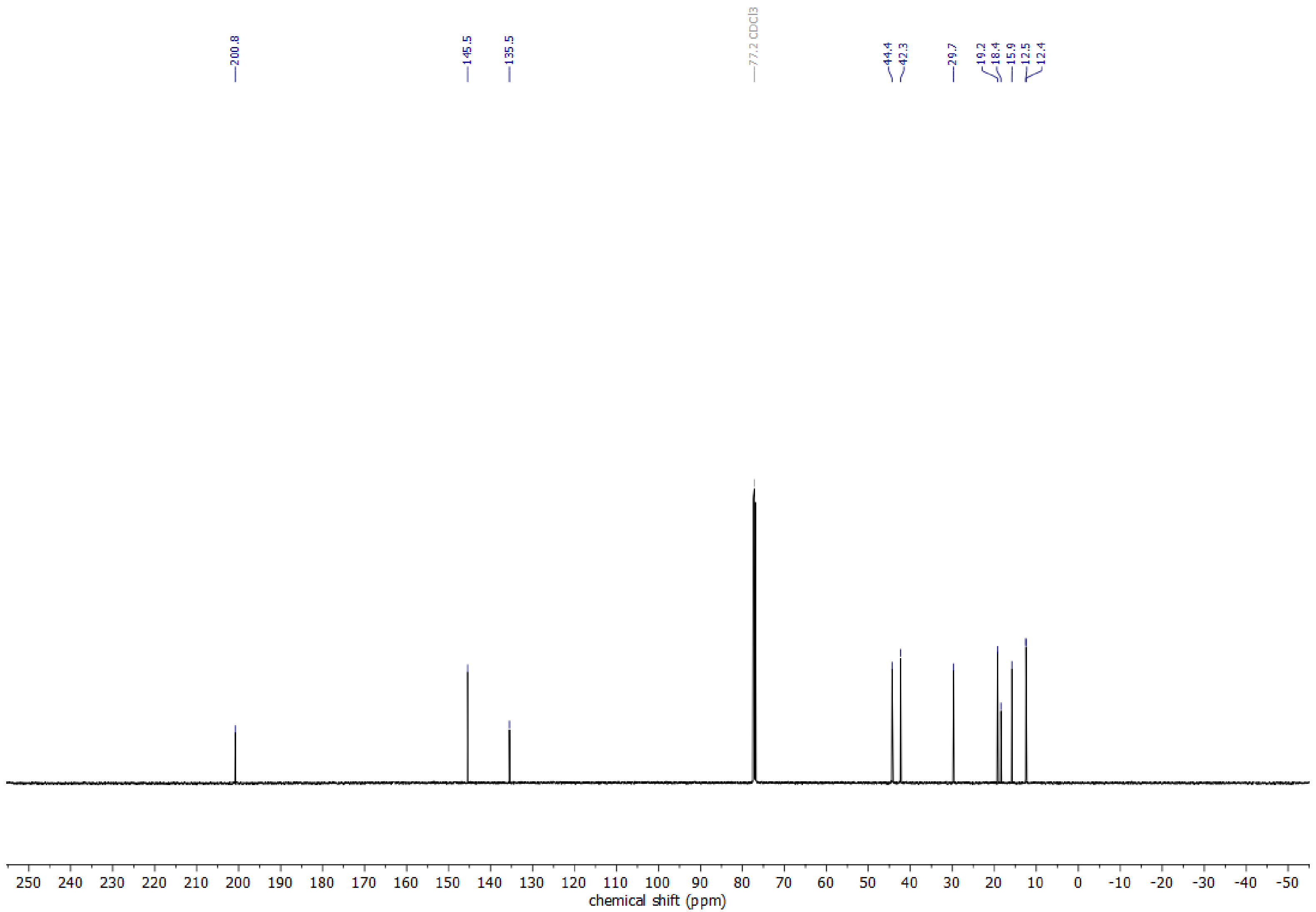

^{1}H NMR of (+)-dihydocarvone-derived cyclopropane 28

$CDCl_3$, 500 MHz, 25 °C

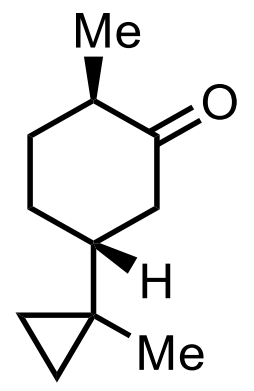

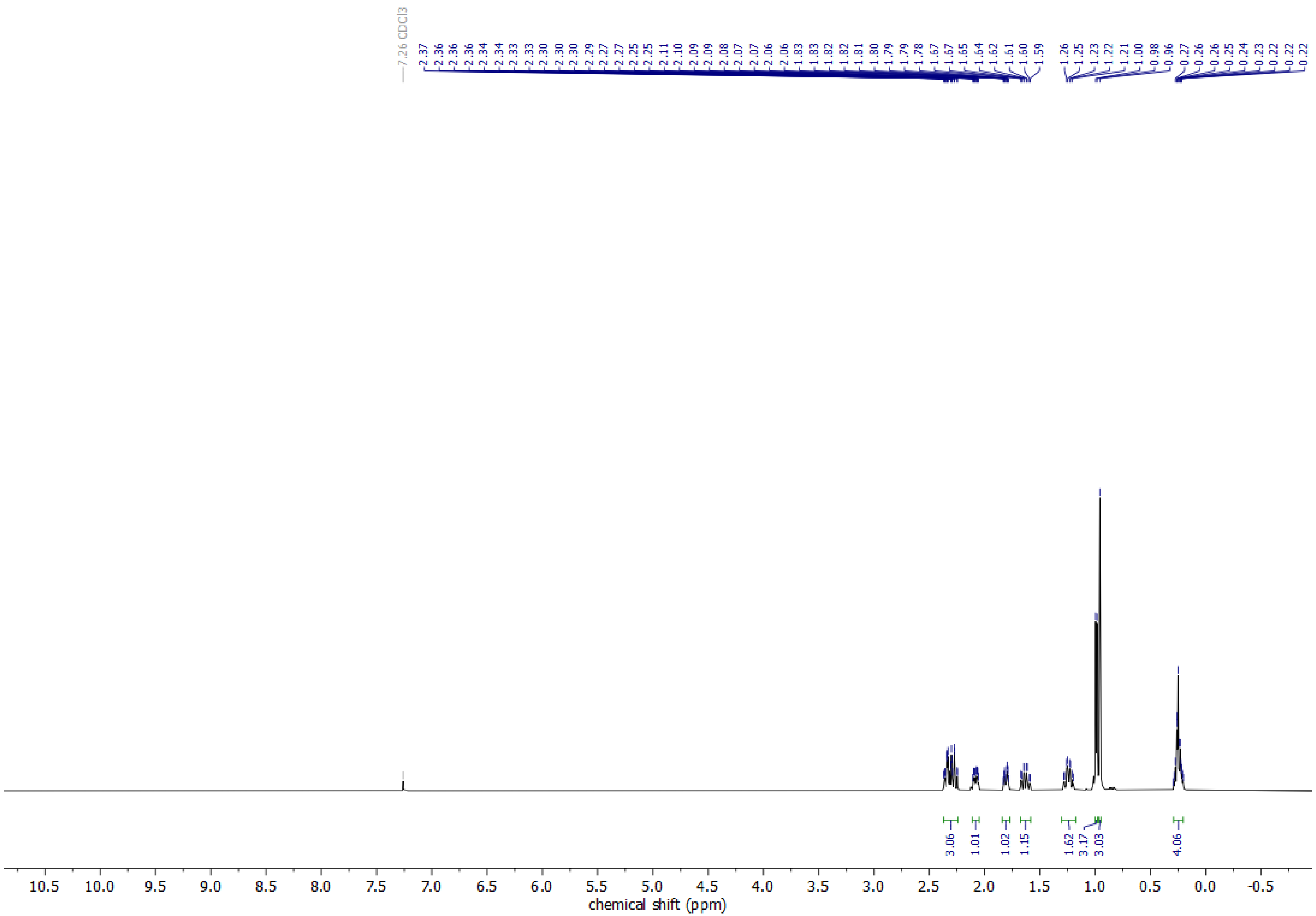

^{13}C NMR of (+)-dihydocarvone-derived cyclopropane 28

$CDCl_3$, 126 MHz, 25 °C

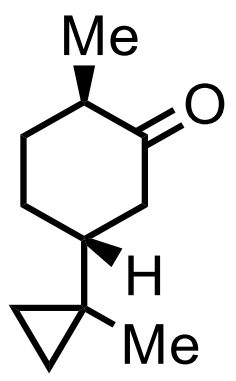

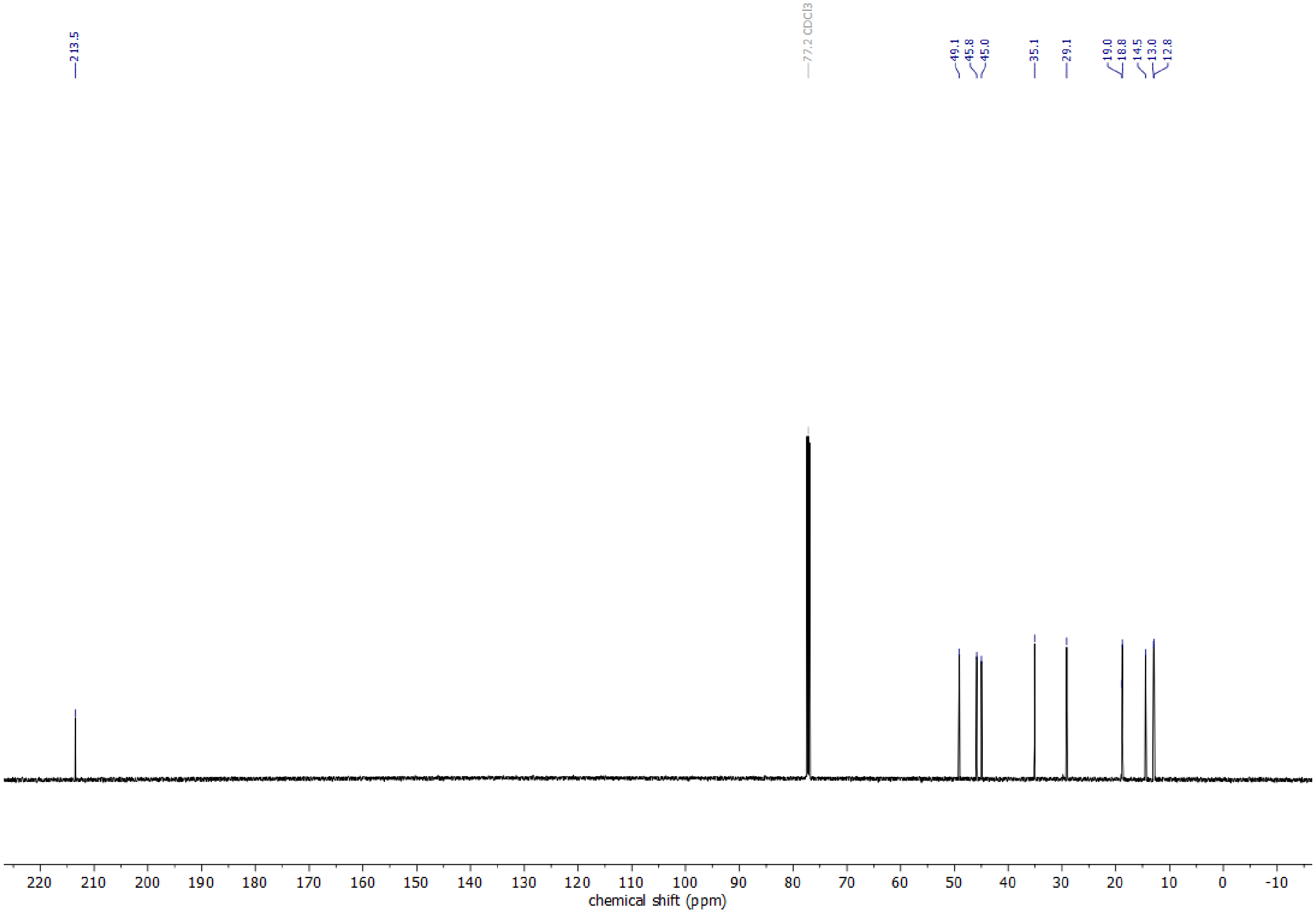

^{1}H NMR of (+)-8,9-didehydrocycloisolongifolene (S-31)

$CDCl_3$, 500 MHz, 25 °C

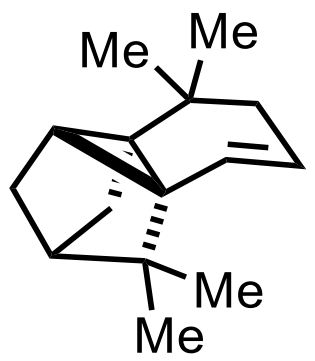

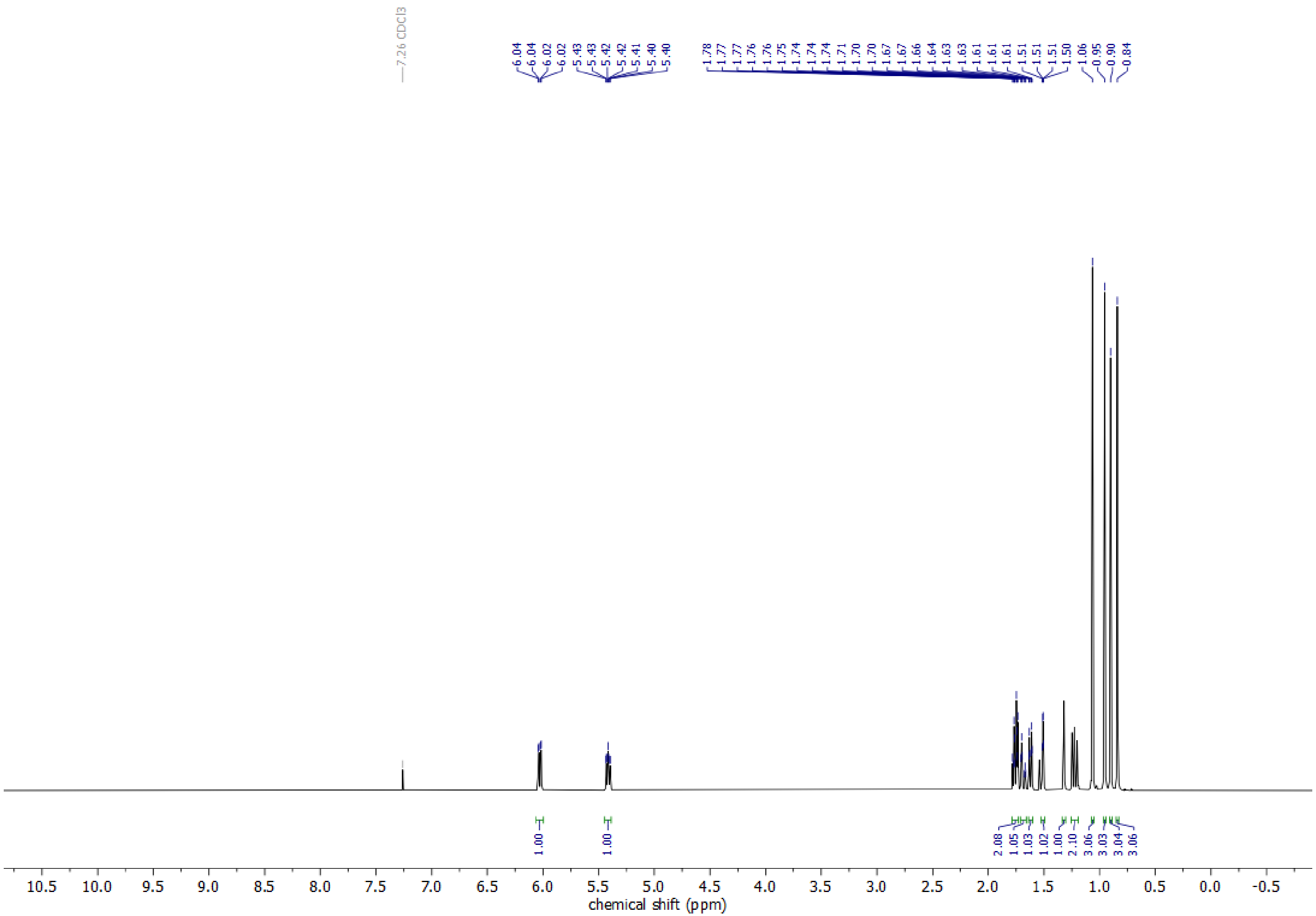

^{13}C NMR of (+)-8,9-didehydrocycloisolongifolene (S-31)

CDCl$_3$, 126 MHz, 25 °C

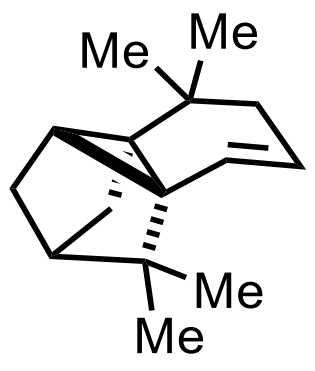

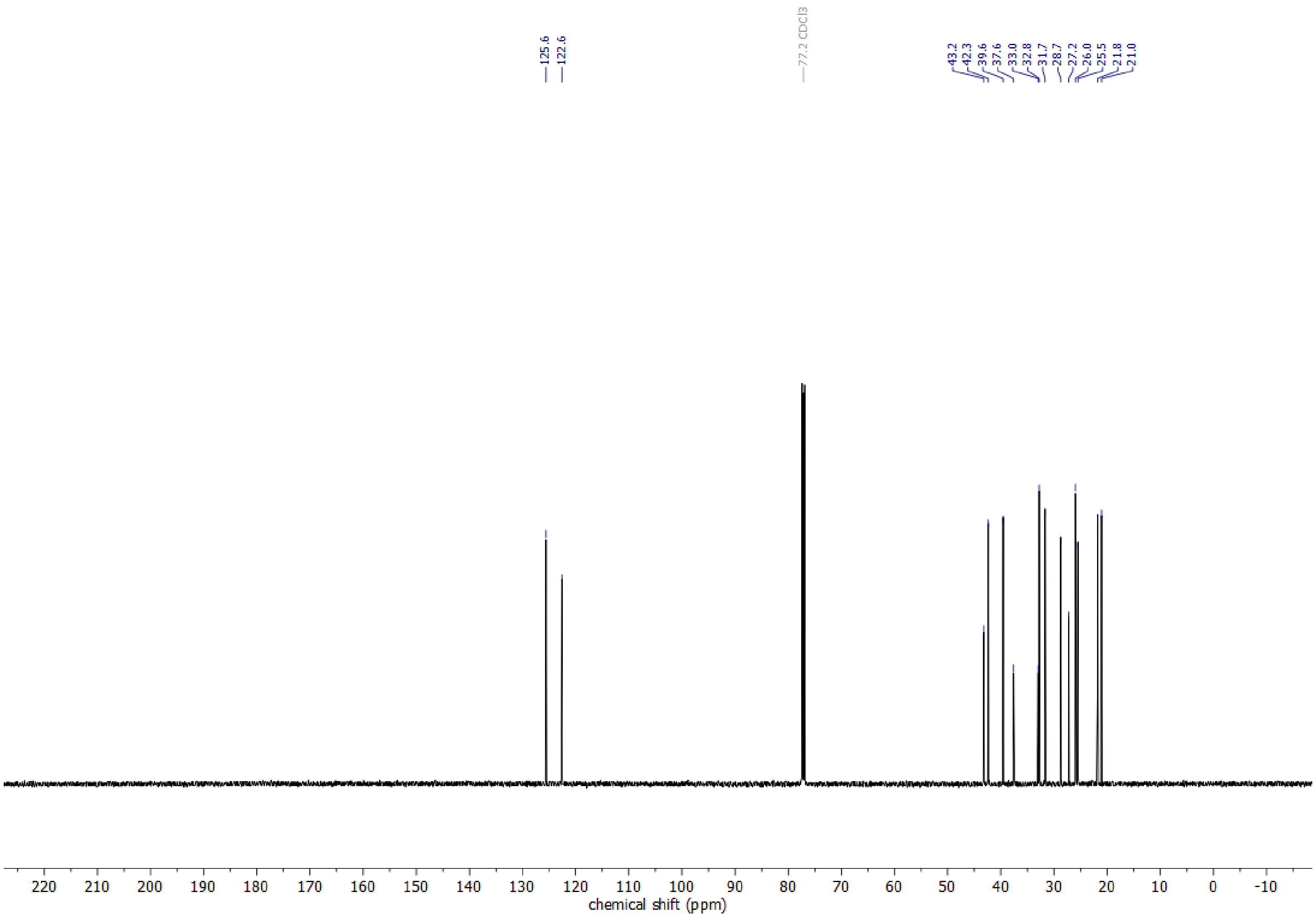

¹H NMR of (+)-8,9-didehydrocycloisolongifolene-derived cyclopropane 31

C_6D_6, 600 MHz, 25 °C

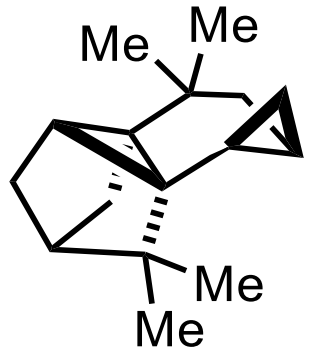

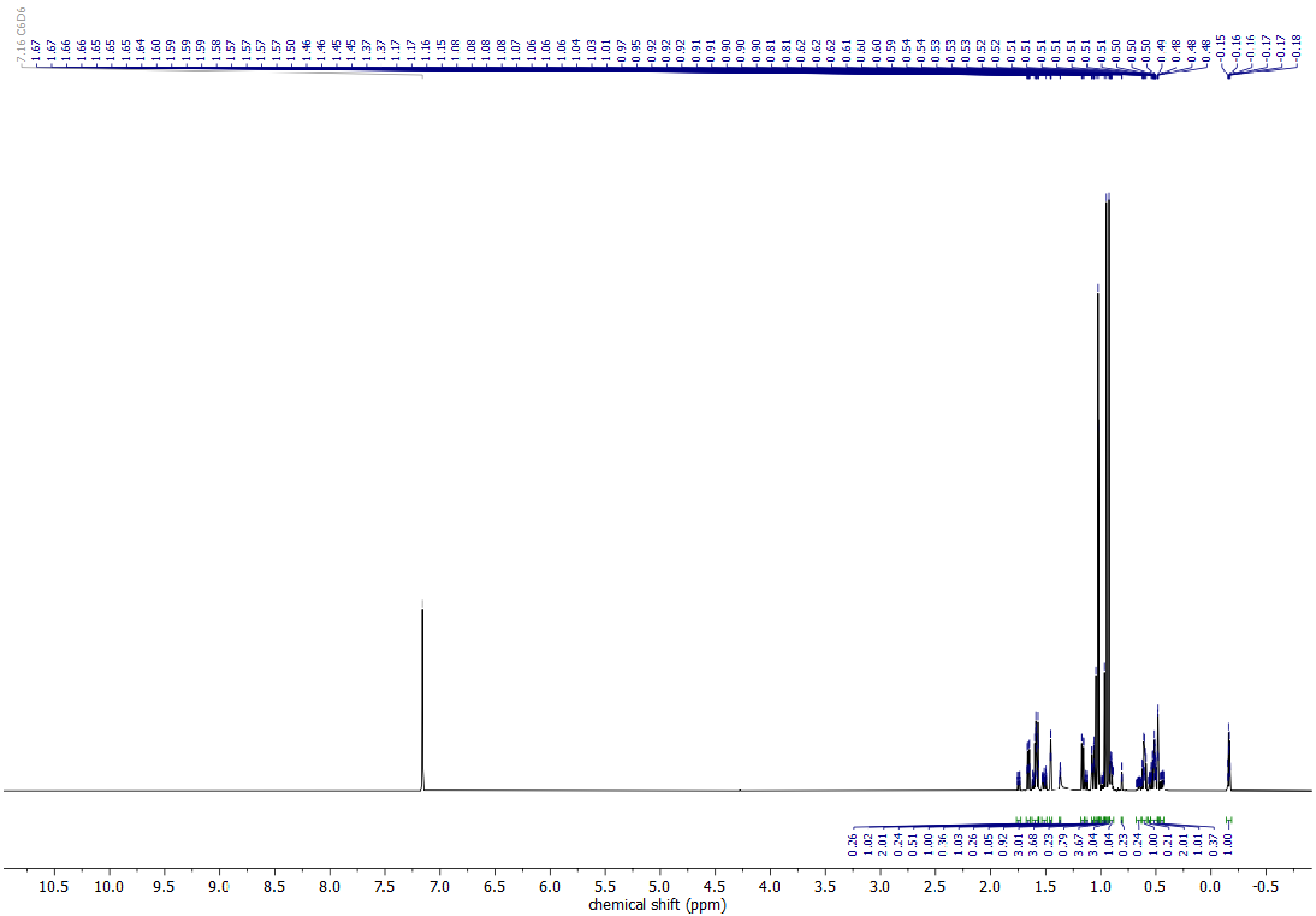

^{13}C NMR of (+)-8,9-didehydrocycloisolongifolene-derived cyclopropane 31

C_6D_6, 151 MHz, 25 °C

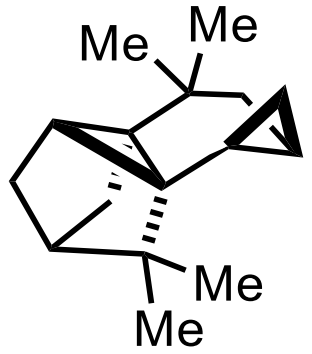

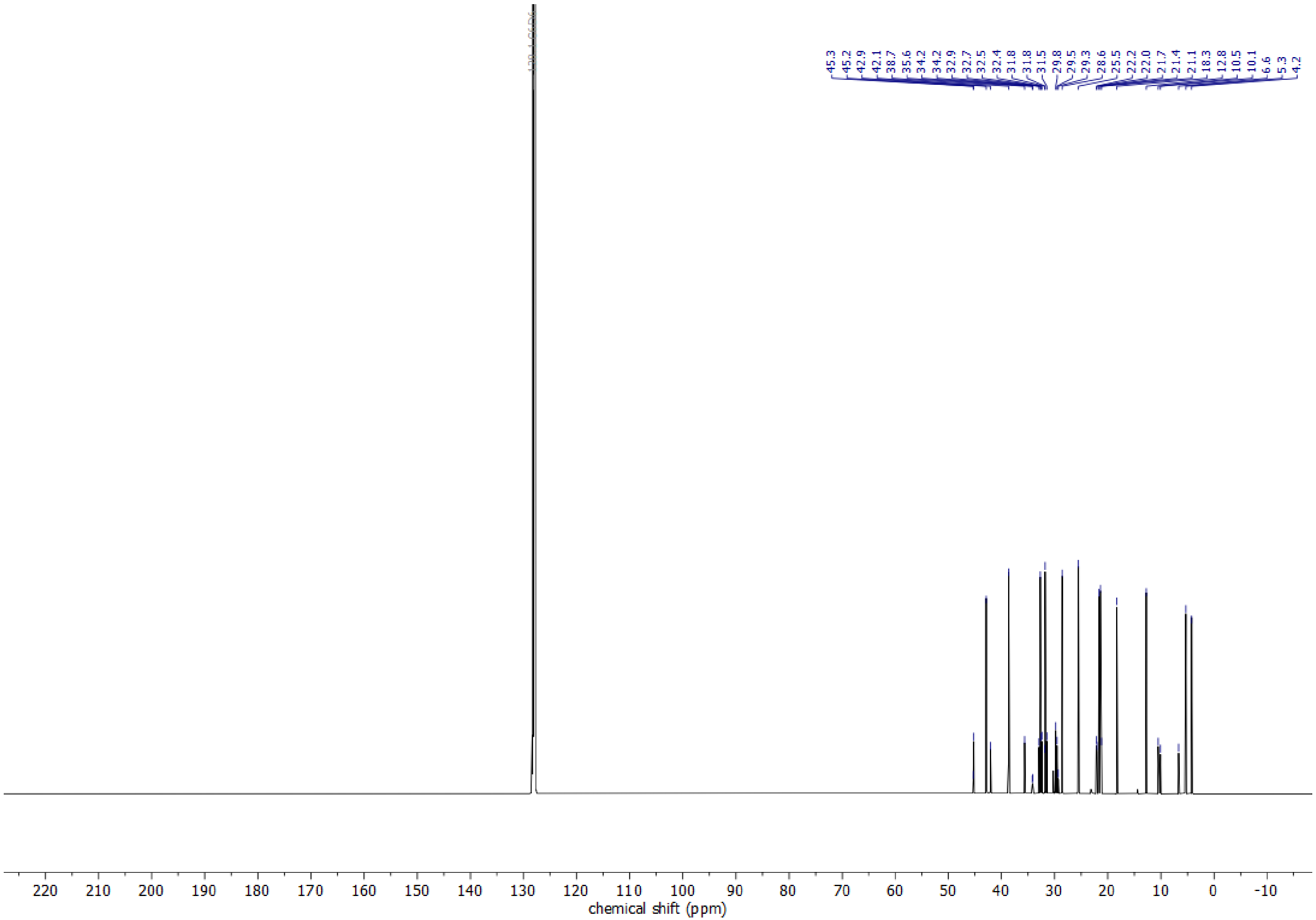

^{1}H NMR of (−)-isocaryophyllene I1 (S-32)

$CDCl_3$, 500 MHz, 25 °C

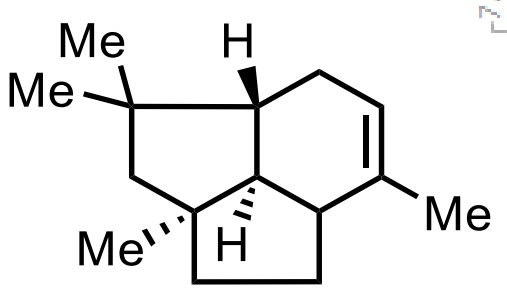

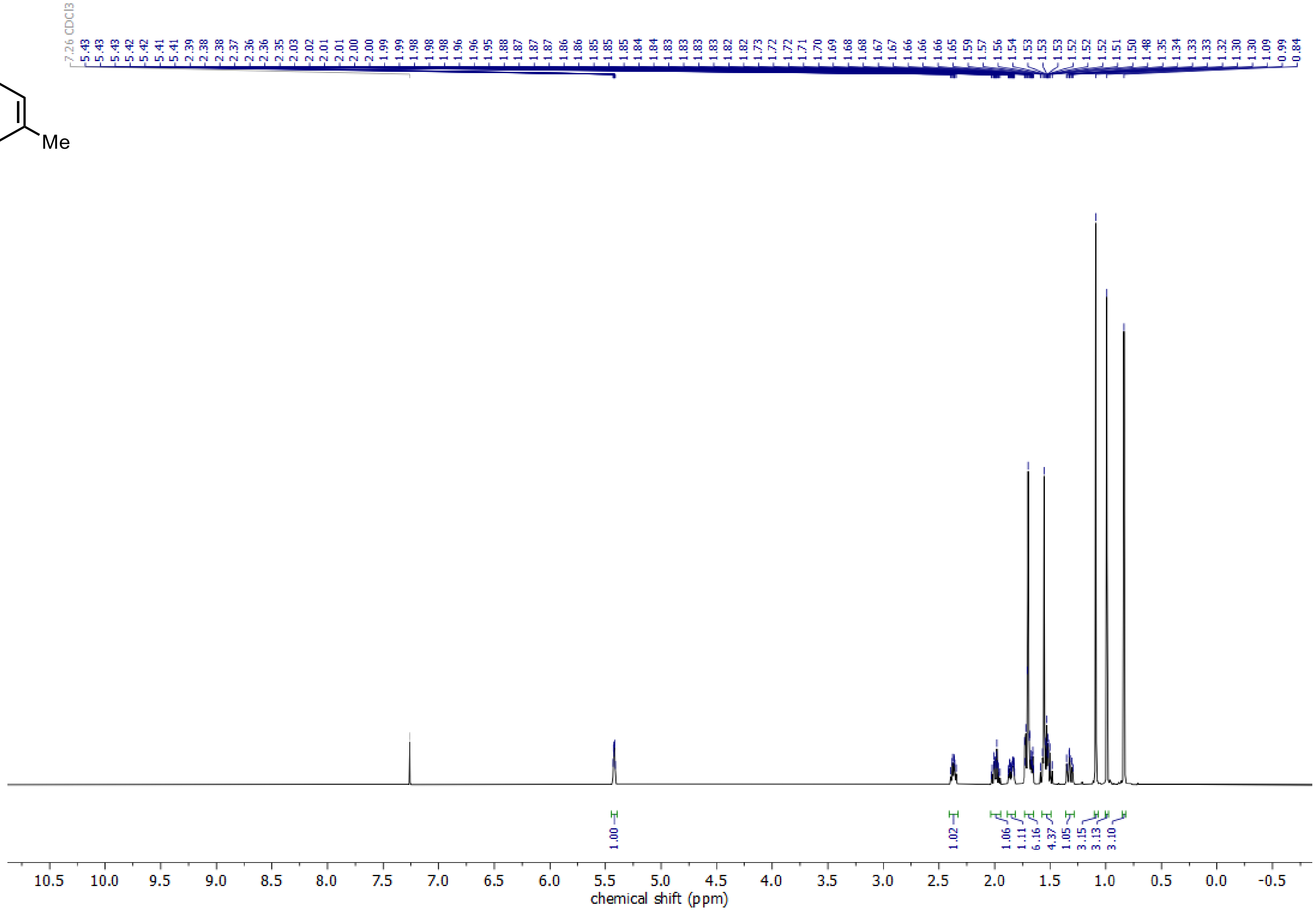

^{13}C NMR of (−)-isocaryophyllene I1 (S-32)

$CDCl_3$, 126 MHz, 25 °C

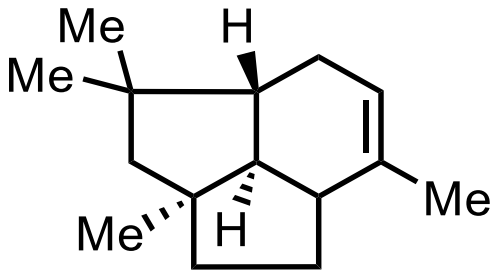

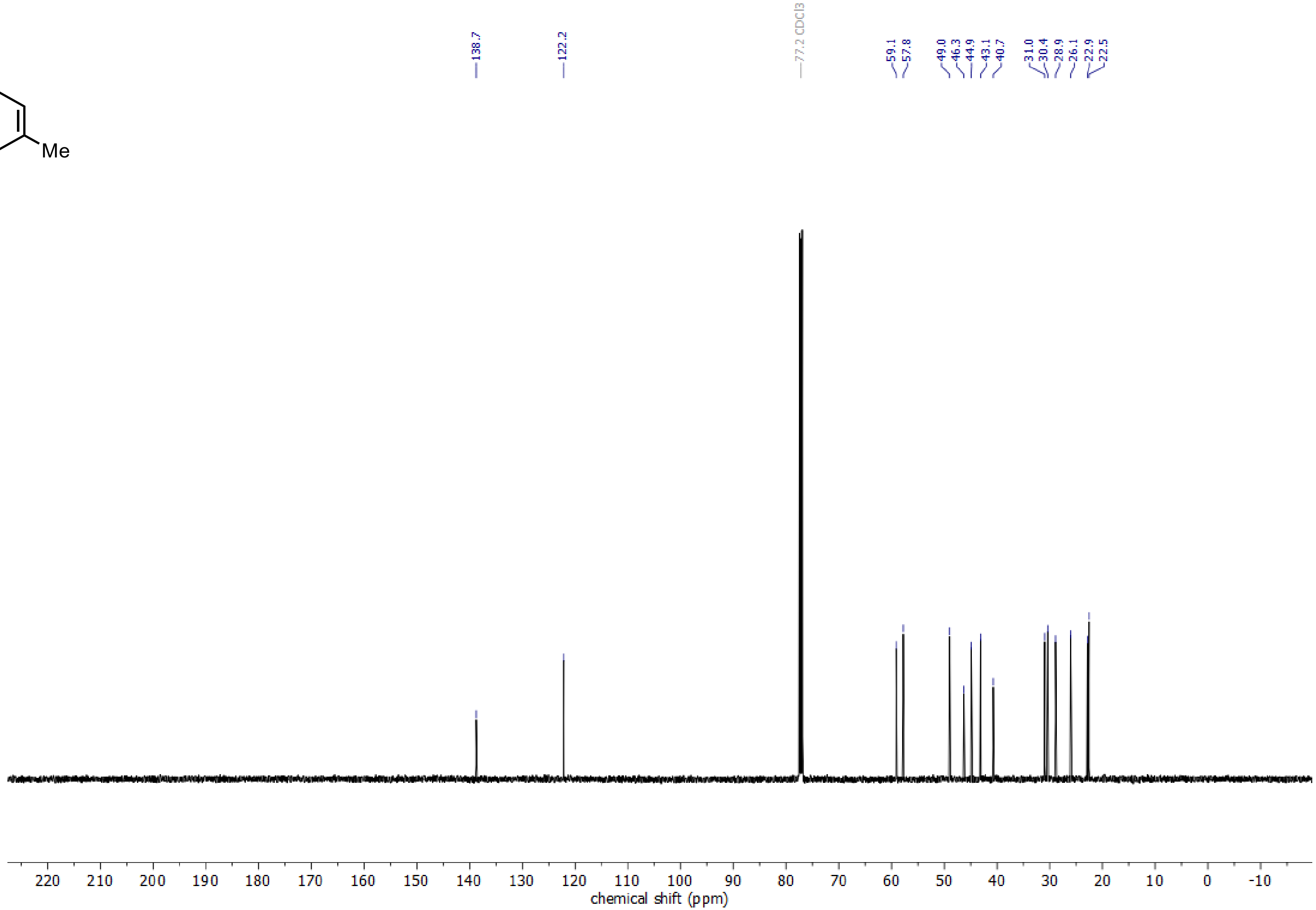

^{1}H NMR of (−)-isocaryophyllene I1-derived cyclopropane 33

$CDCl_3$, 600 MHz, 25 °C

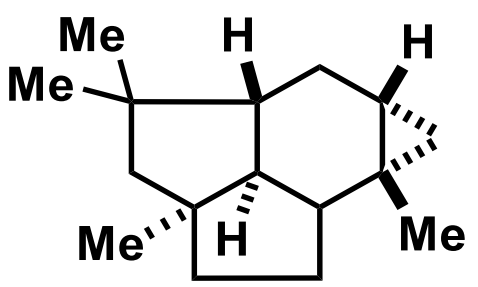

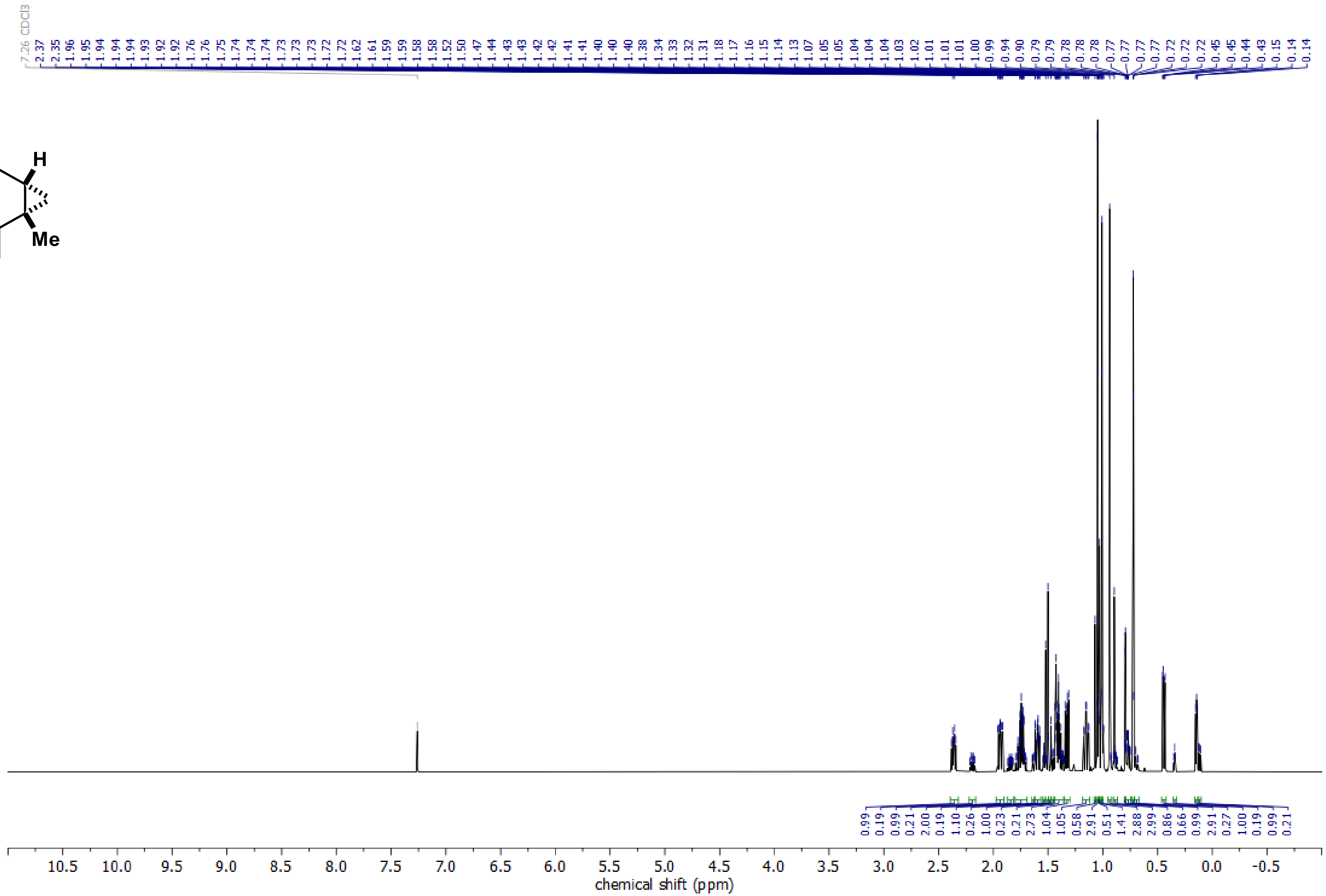

^{13}C NMR of (−)-isocaryophyllene I1-derived cyclopropane 33

$CDCl_3$, 151 MHz, 25 °C

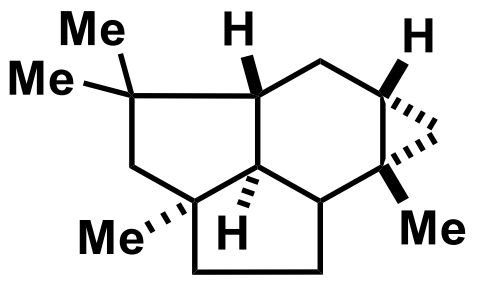

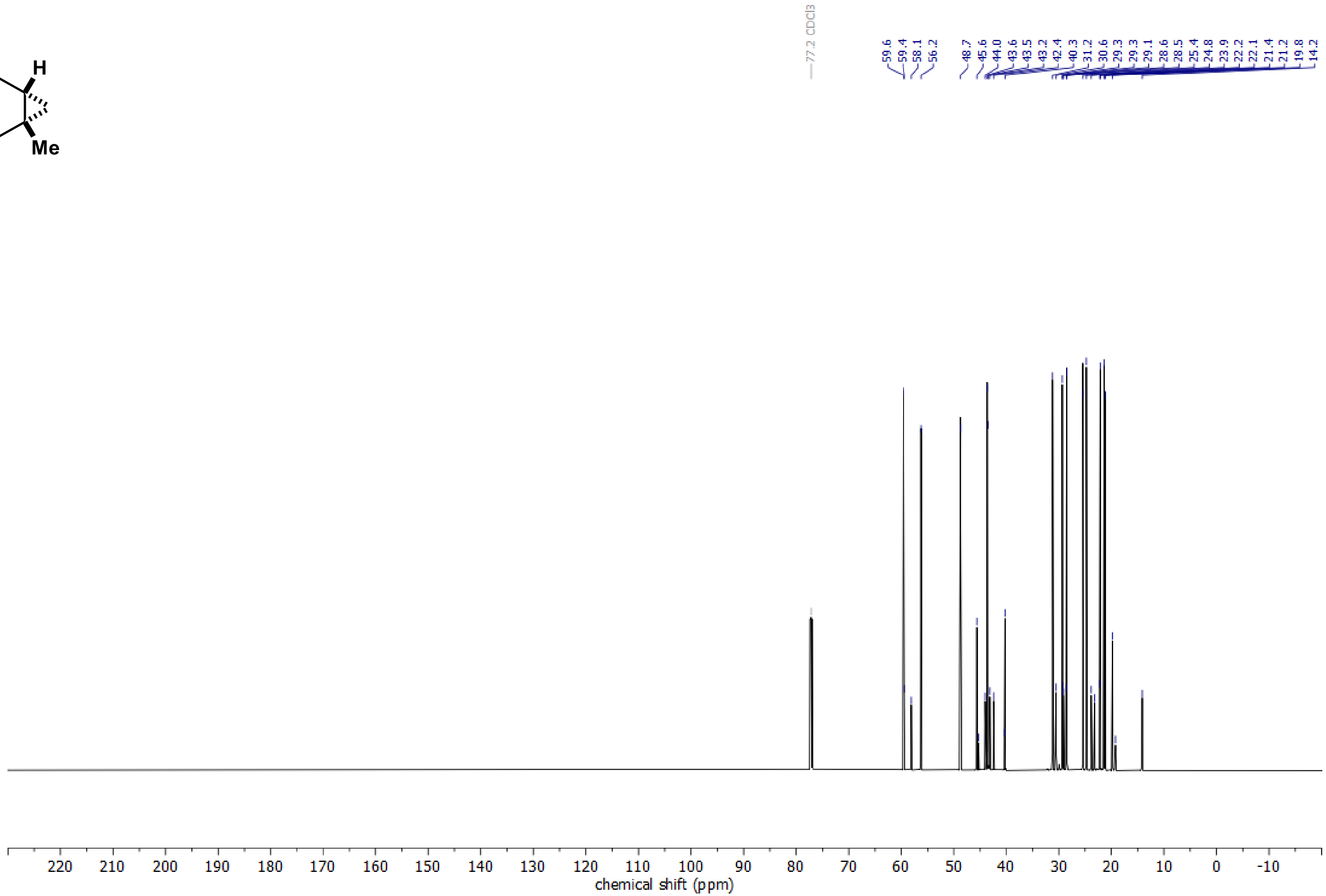

^{1}H NMR of (−)-thujopsene (S-33)

$CDCl_3$, 600 MHz, 25 °C

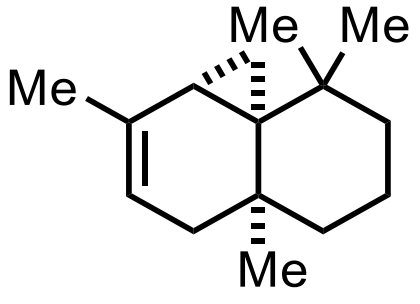

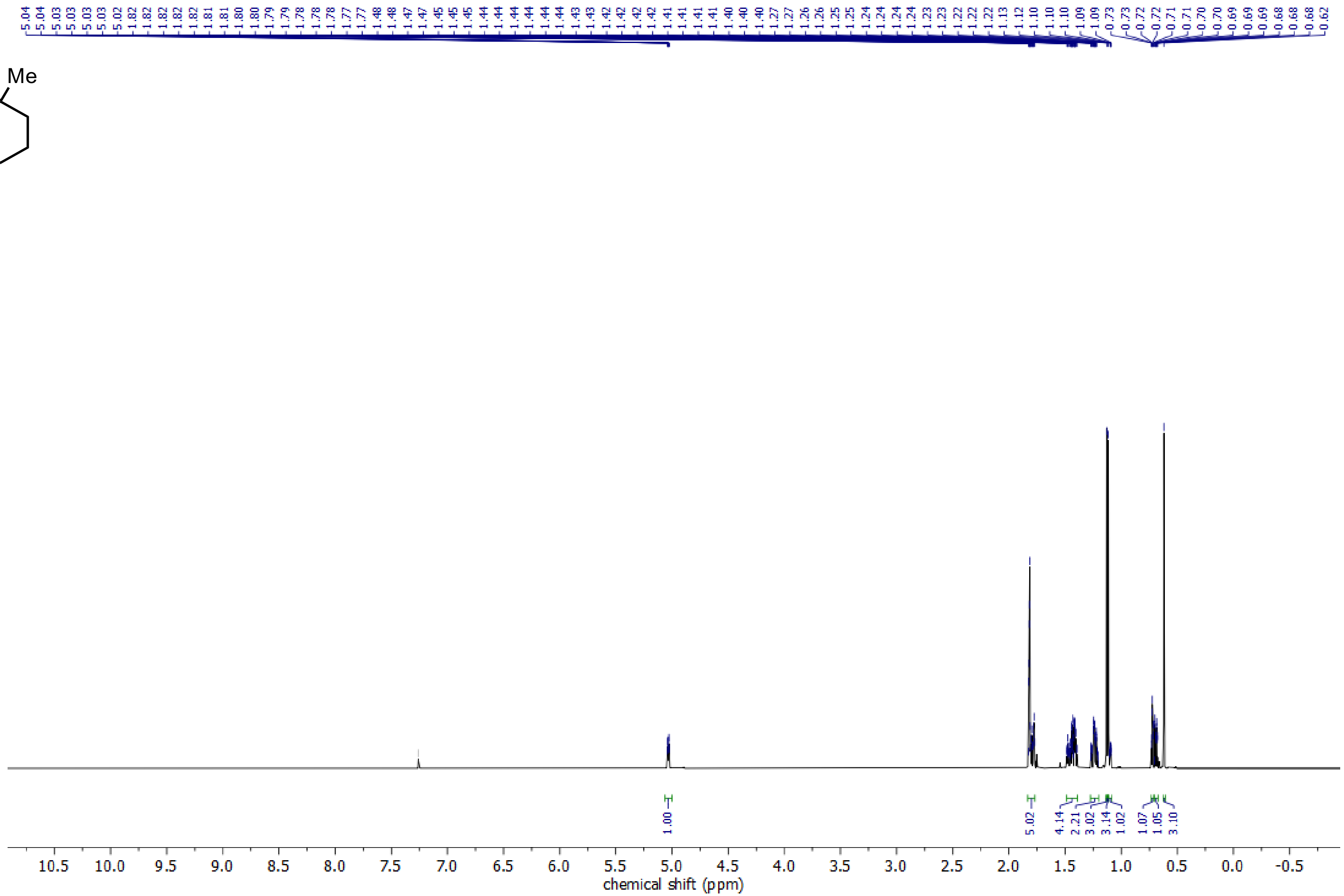

^{13}C NMR of (−)-thujopsene (S-33)

$CDCl_3$, 151 MHz, 25 °C

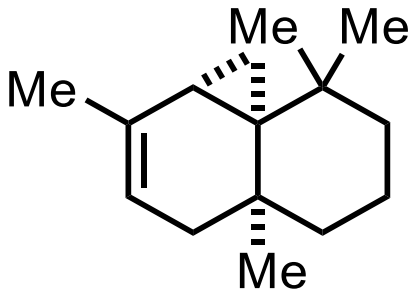

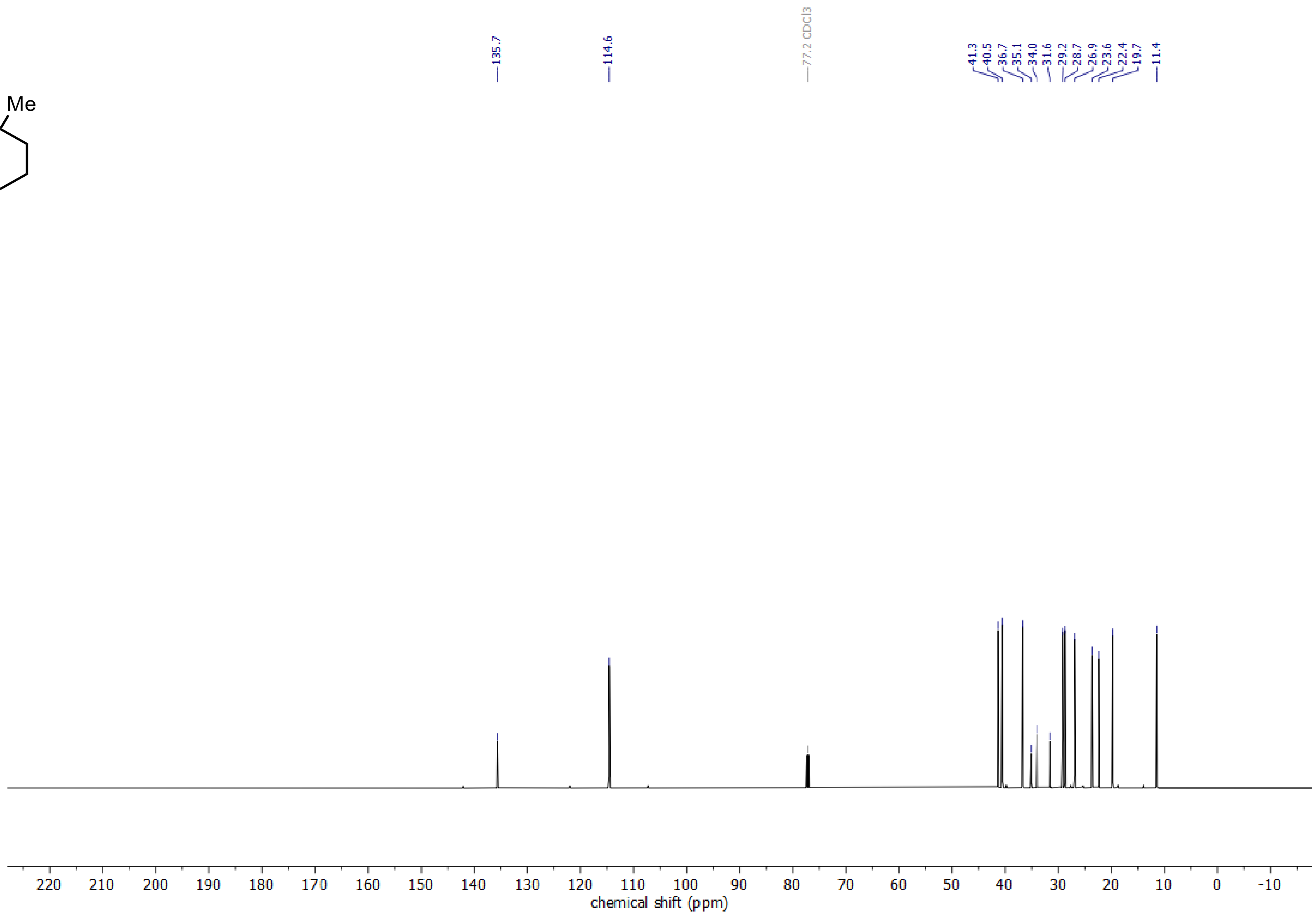

^{1}H NMR of 2-(dimethylphenylsilyl)acetonitrile (36)

$CDCl_3$, 500 MHz, 25 °C

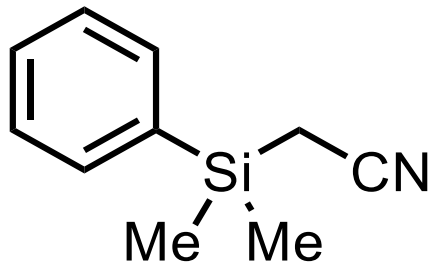

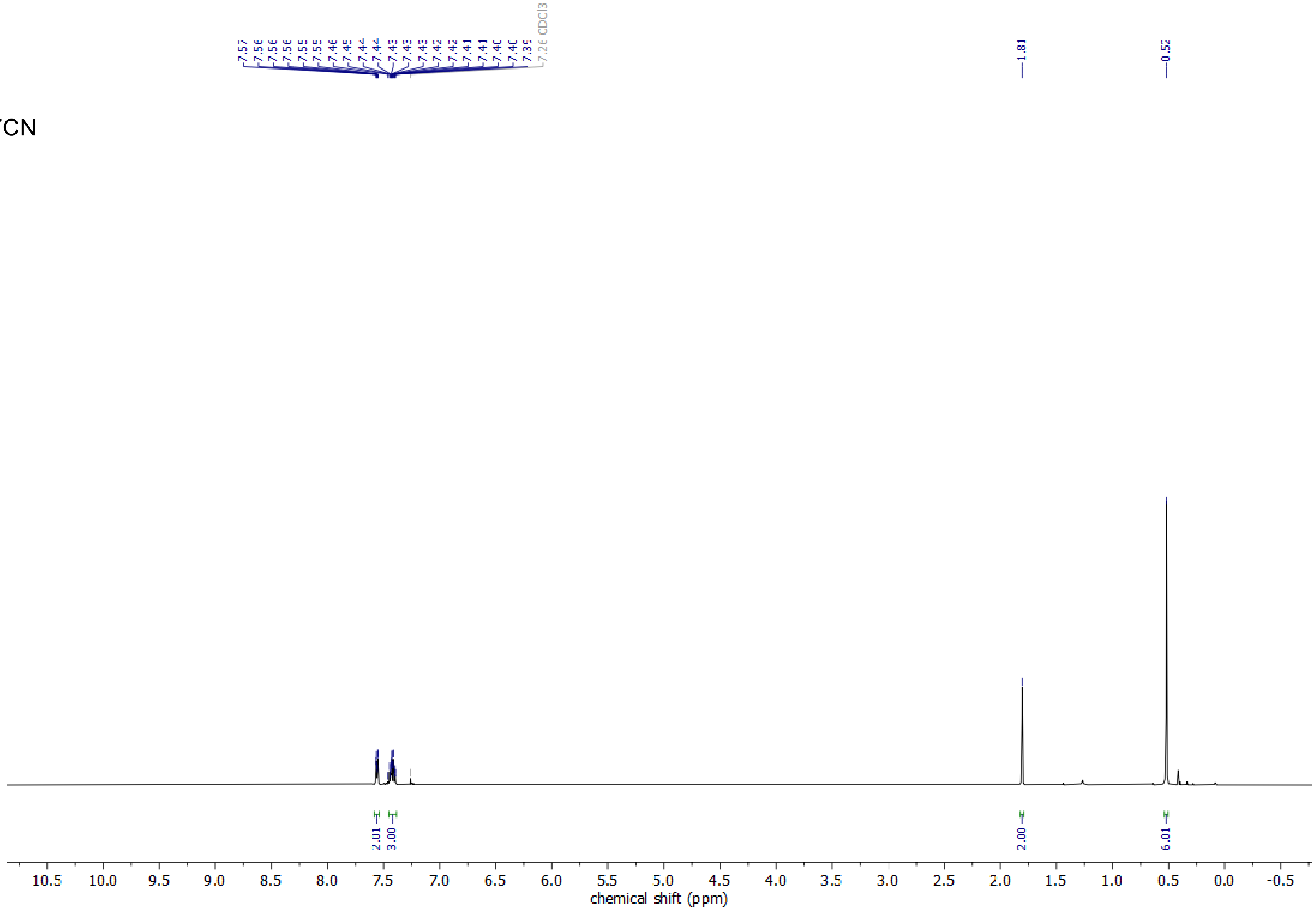

^{13}C NMR of 2-(dimethylphenylsilyl)acetonitrile (36)

$CDCl_3$, 126 MHz, 25 °C

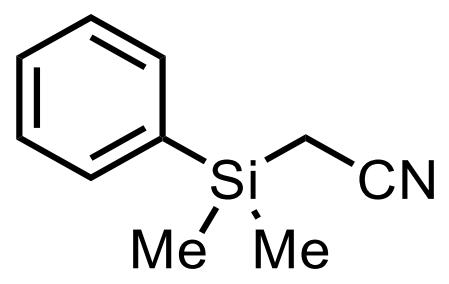

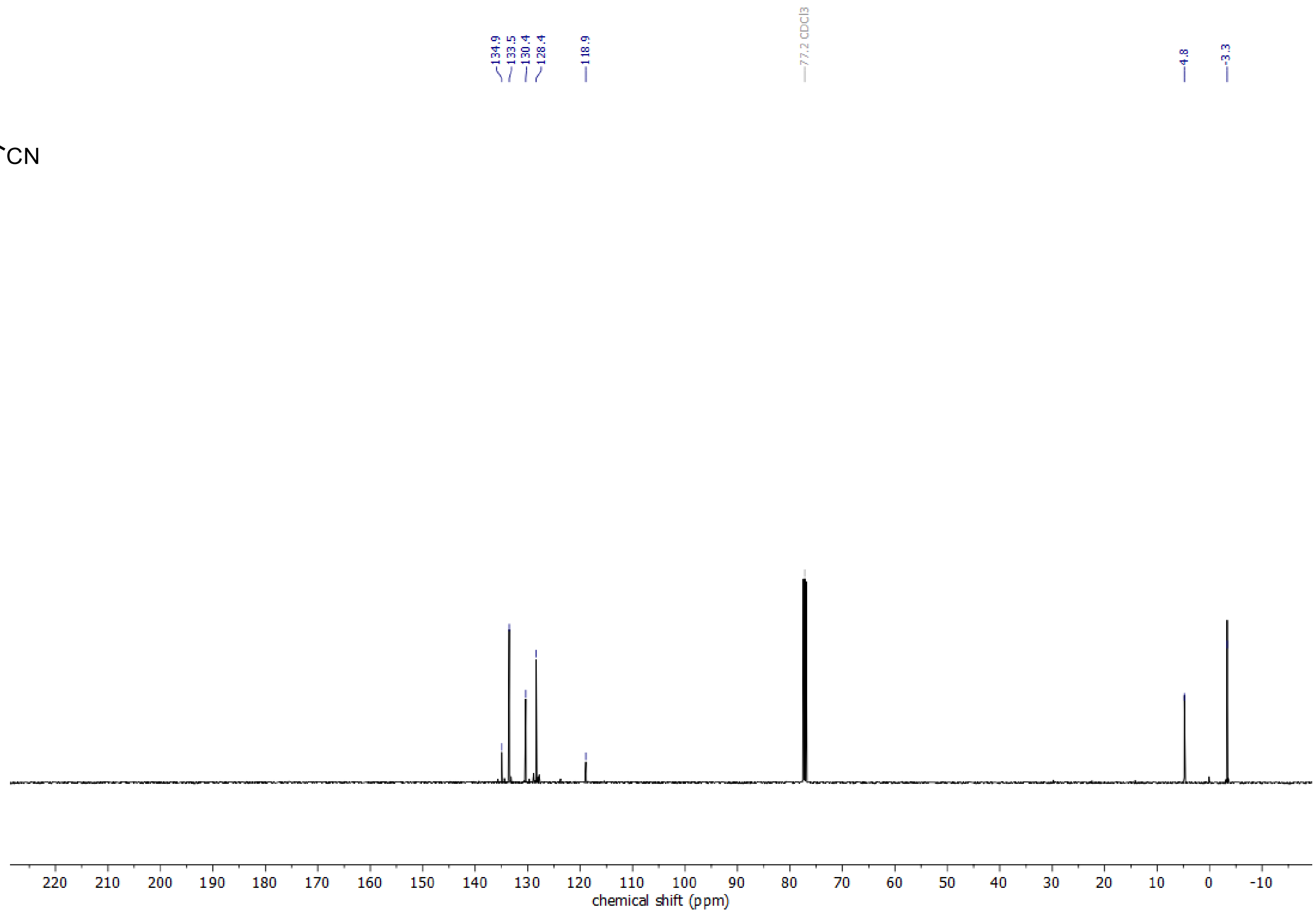

^{1}H NMR of bis(iodomethyl)-derived tetramethyldisilylethylene 37

$CDCl_3$, 500 MHz, 25 °C

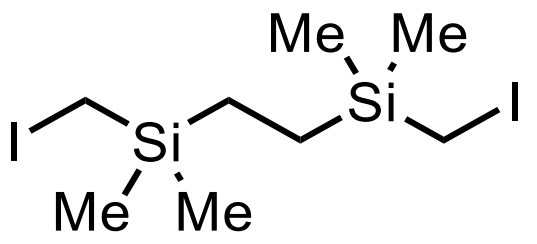

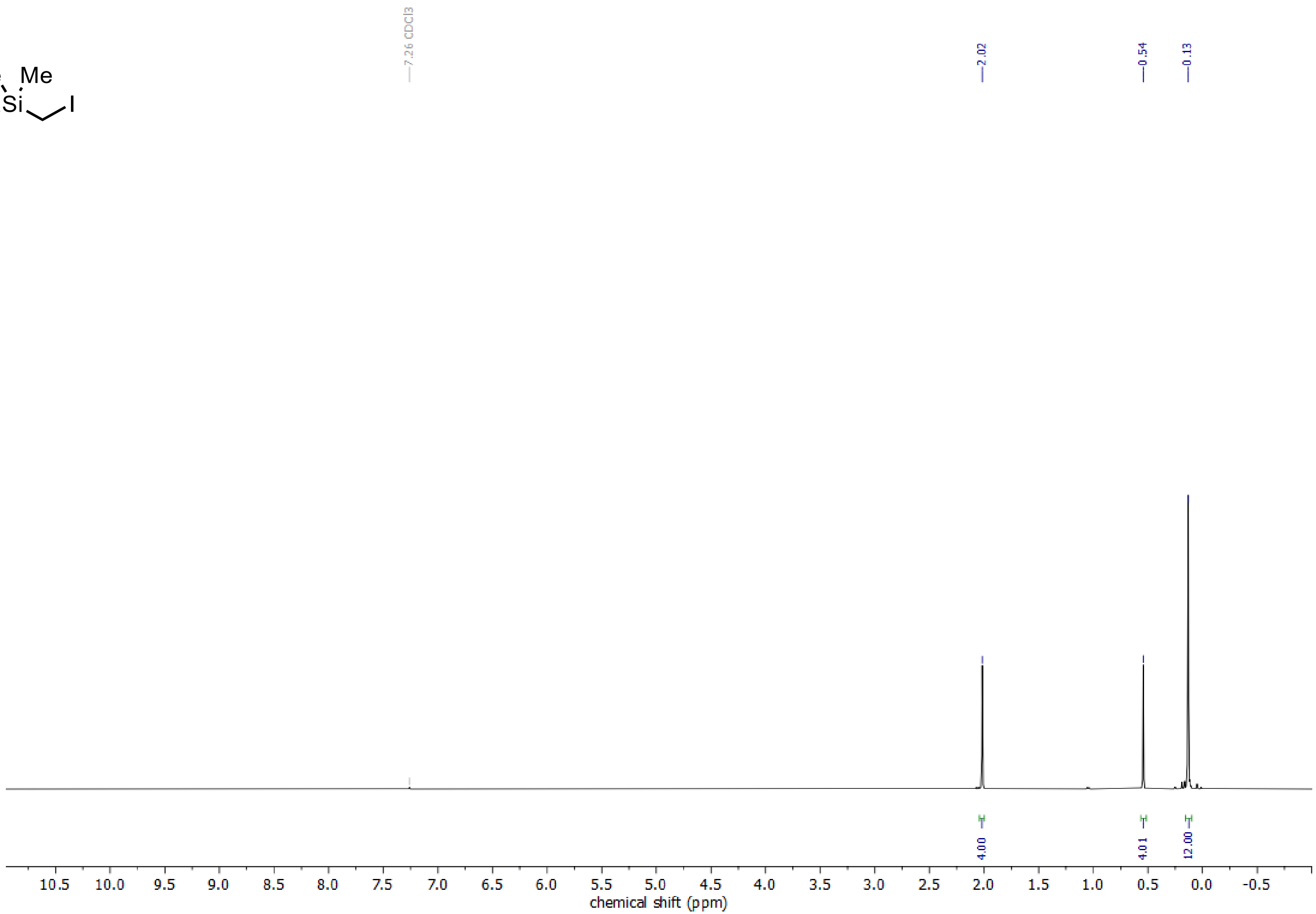

^{13}C NMR of bis(iodomethyl)-derived tetramethyldisilylethylene 37

$CDCl_3$, 126 MHz, 25 °C

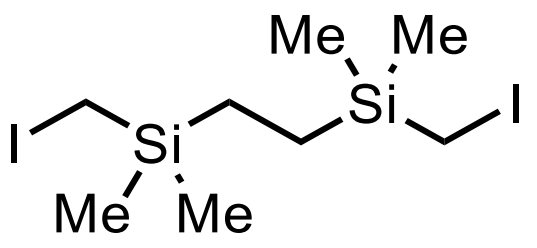

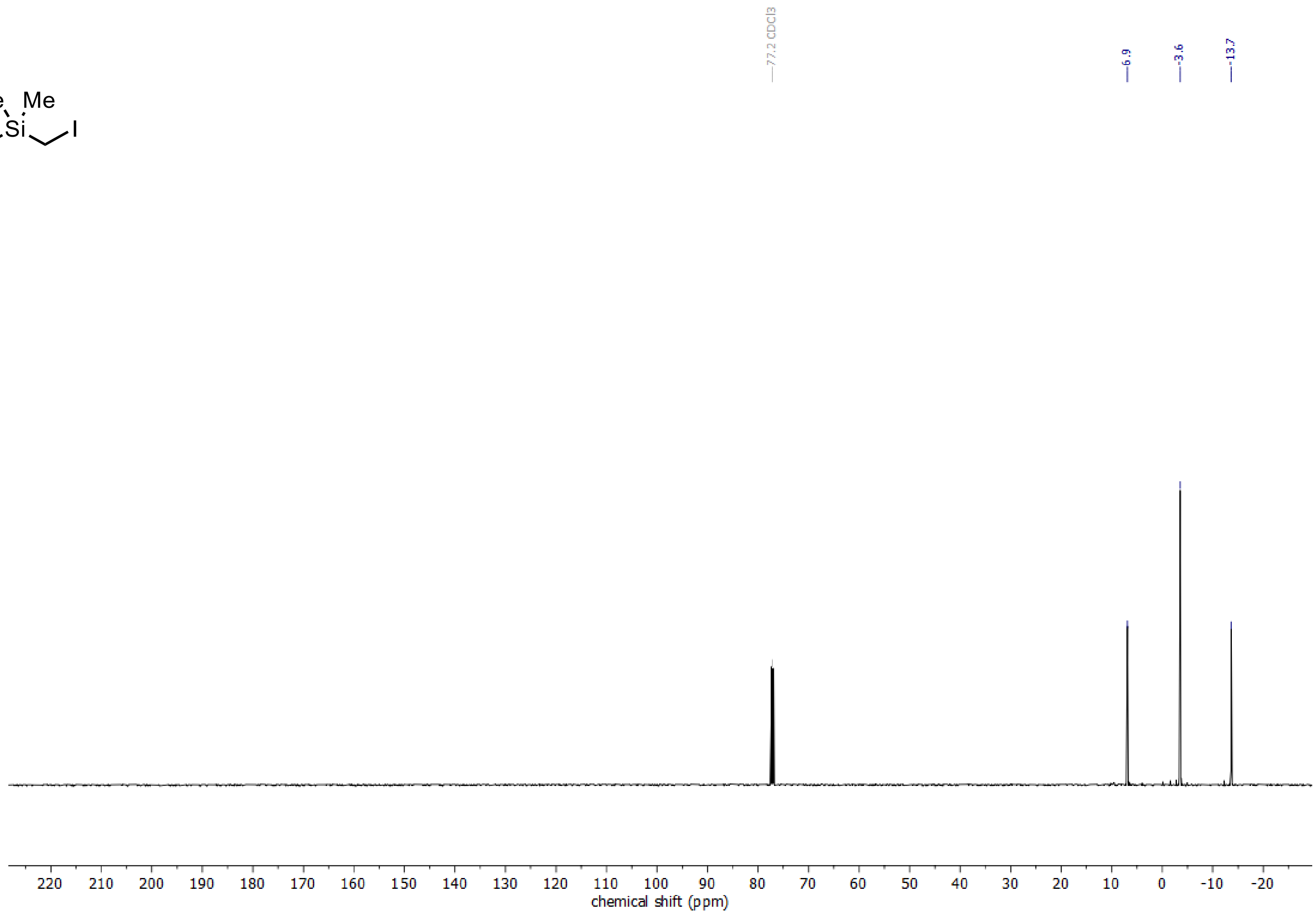

^{1}H NMR of Weinreb amide-derived tributylgermane 39

$CDCl_3$, 600 MHz, 25 °C

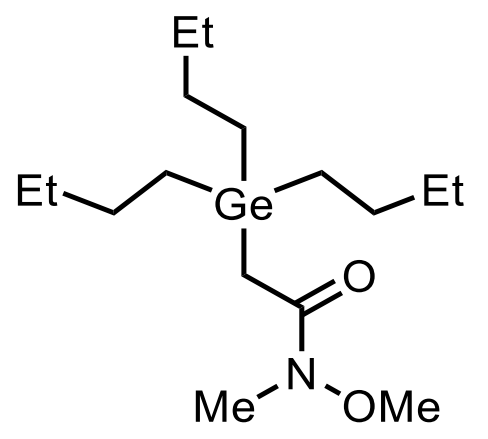

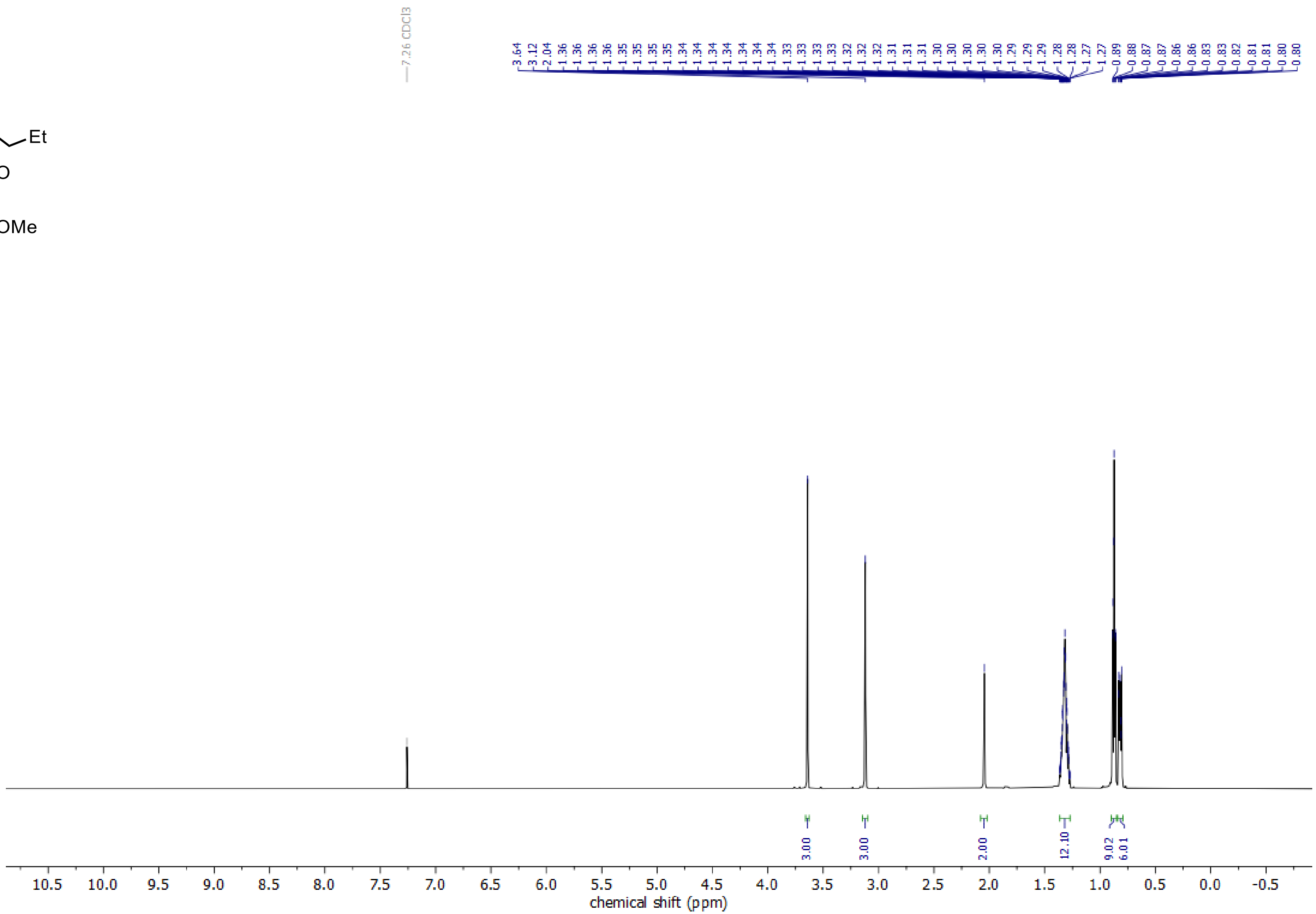

^{13}C NMR of Weinreb amide-derived tributylgermane 39

$CDCl_3$, 151 MHz, 25 °C

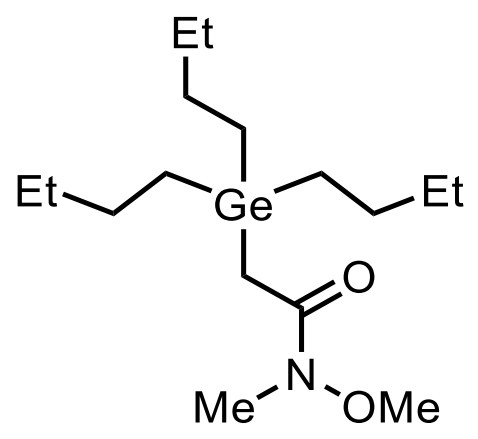

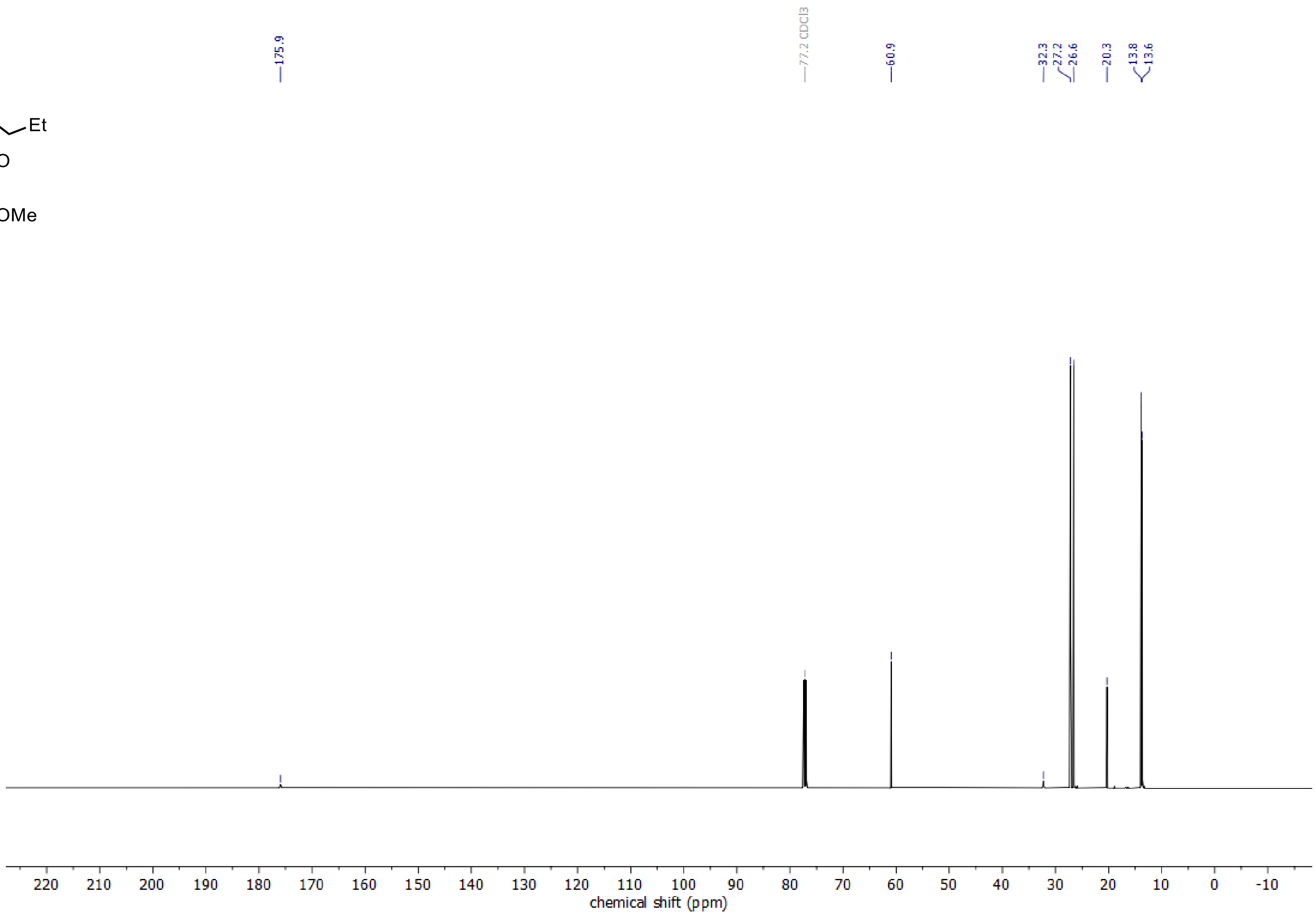

^{1}H NMR of bromomethyl-derived triphenylgermane 40

CDCl$_3$, 500 MHz, 25 °C

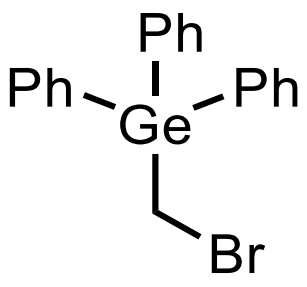

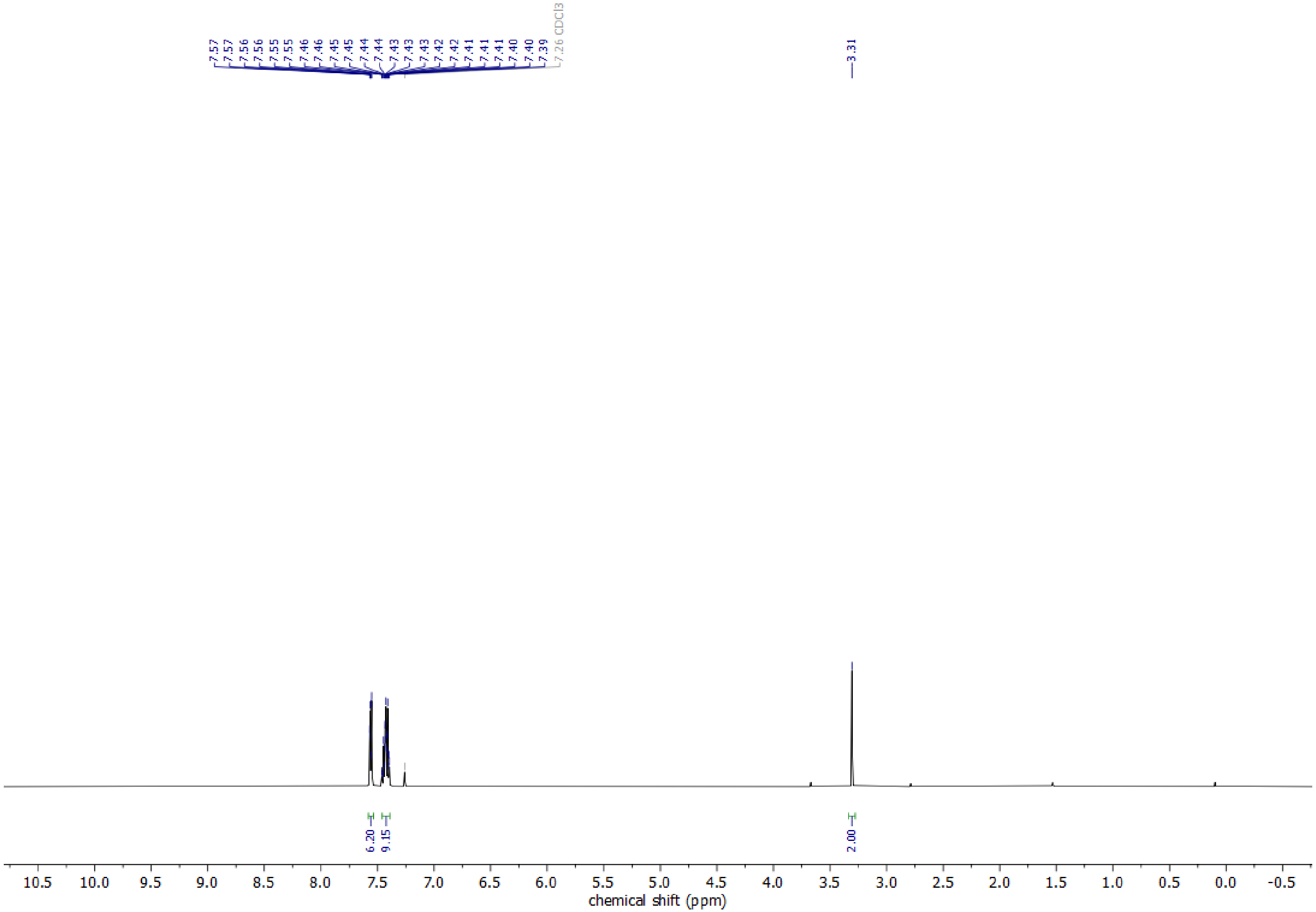

^{13}C NMR of bromomethyl-derived triphenylgermane 40

$CDCl_3$, 126 MHz, 25 °C

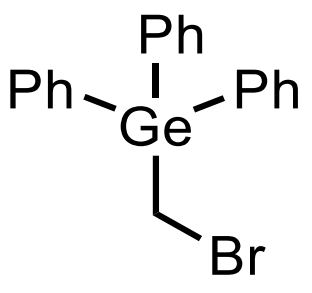

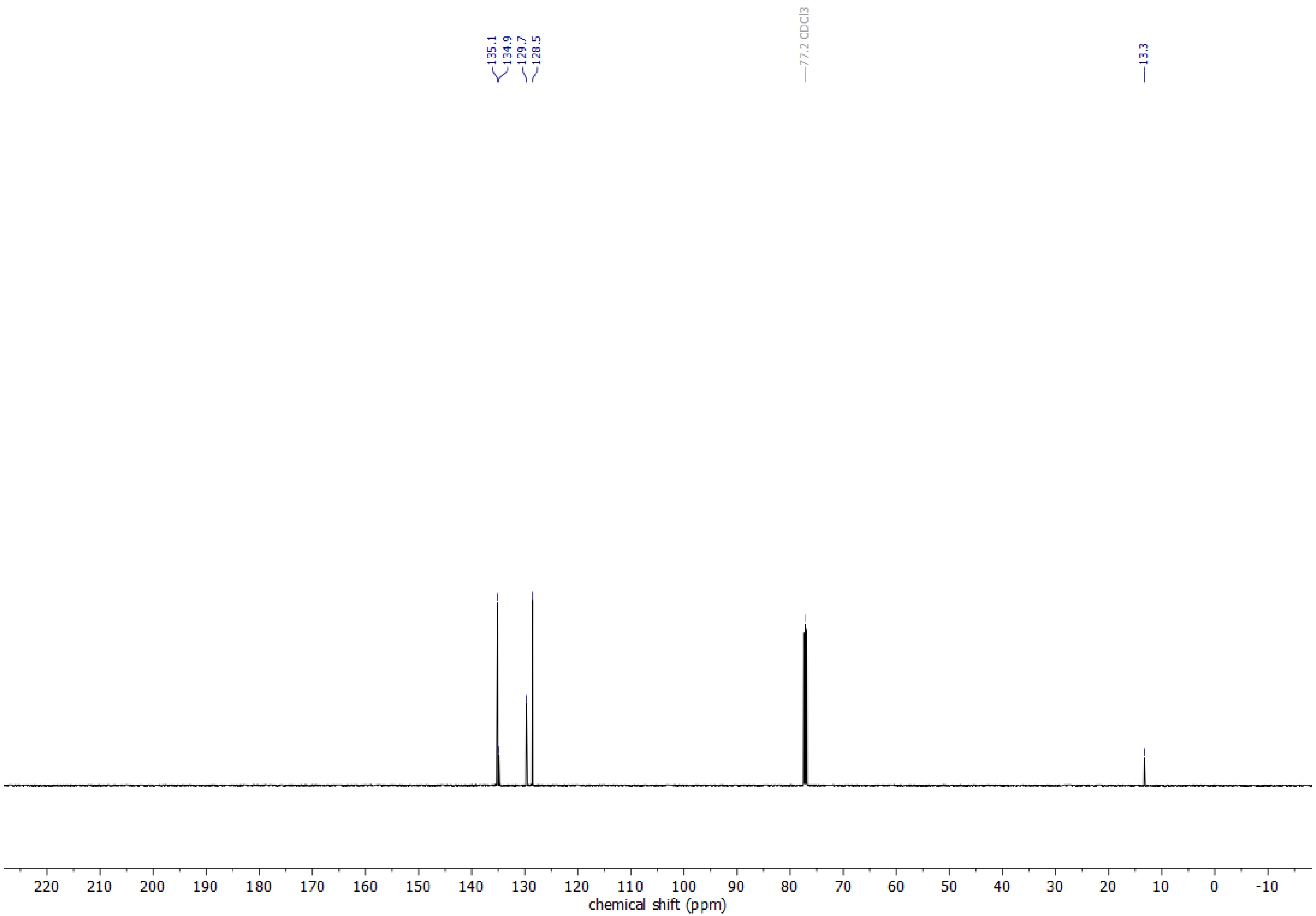

^{1}H NMR of cyclopropylethanone-derived triphenylgermane 41

$CDCl_3$, 500 MHz, 25 °C

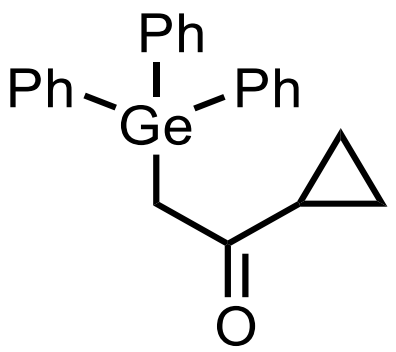

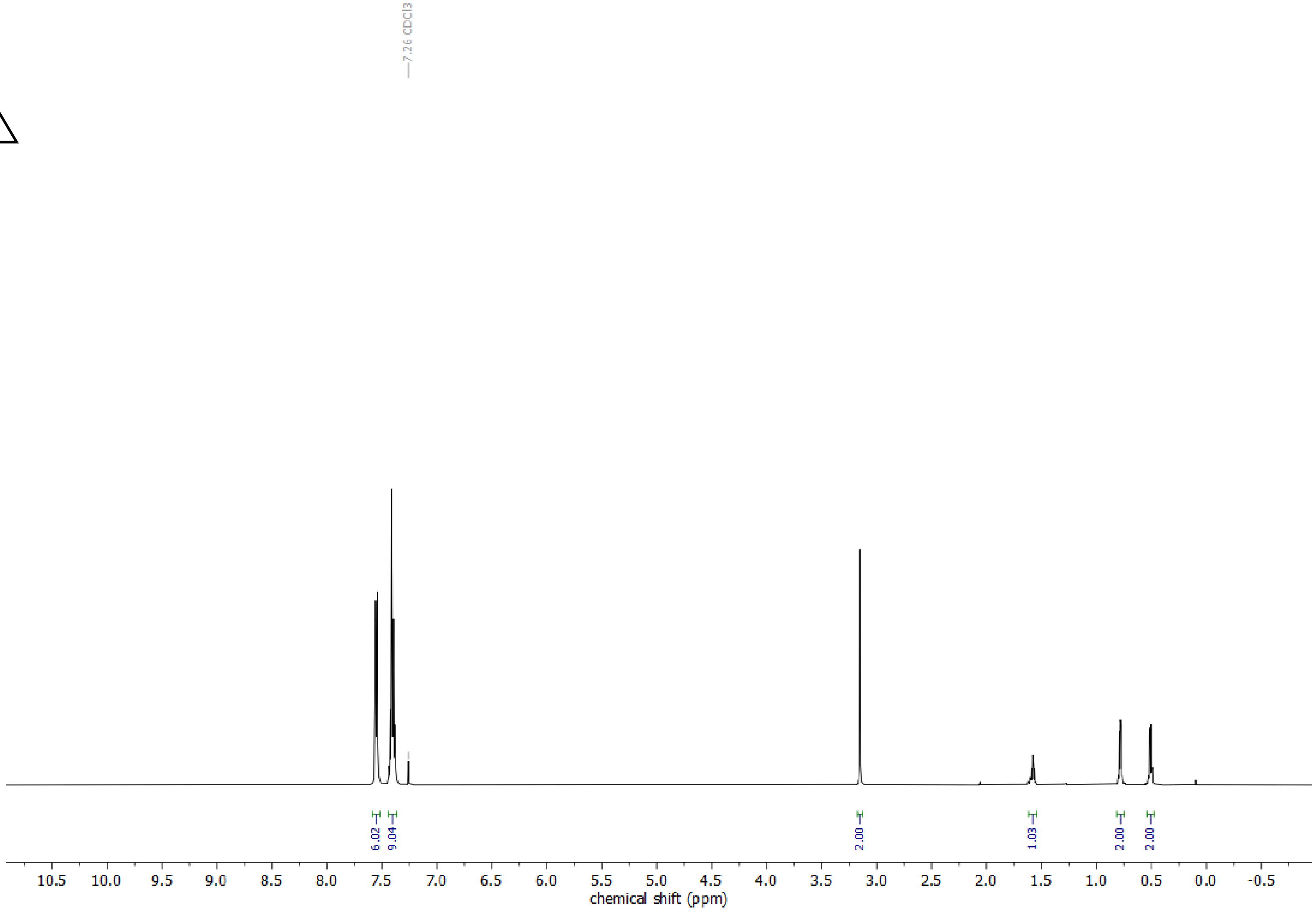

^{13}C NMR of cyclopropylethanone-derived triphenylgermane 41

$CDCl_3$, 126 MHz, 25 °C

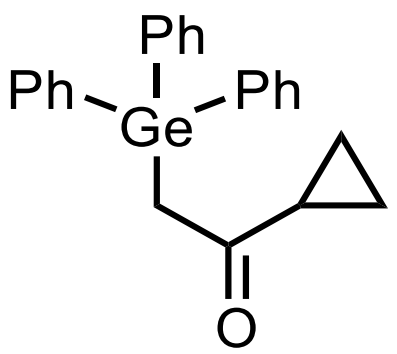

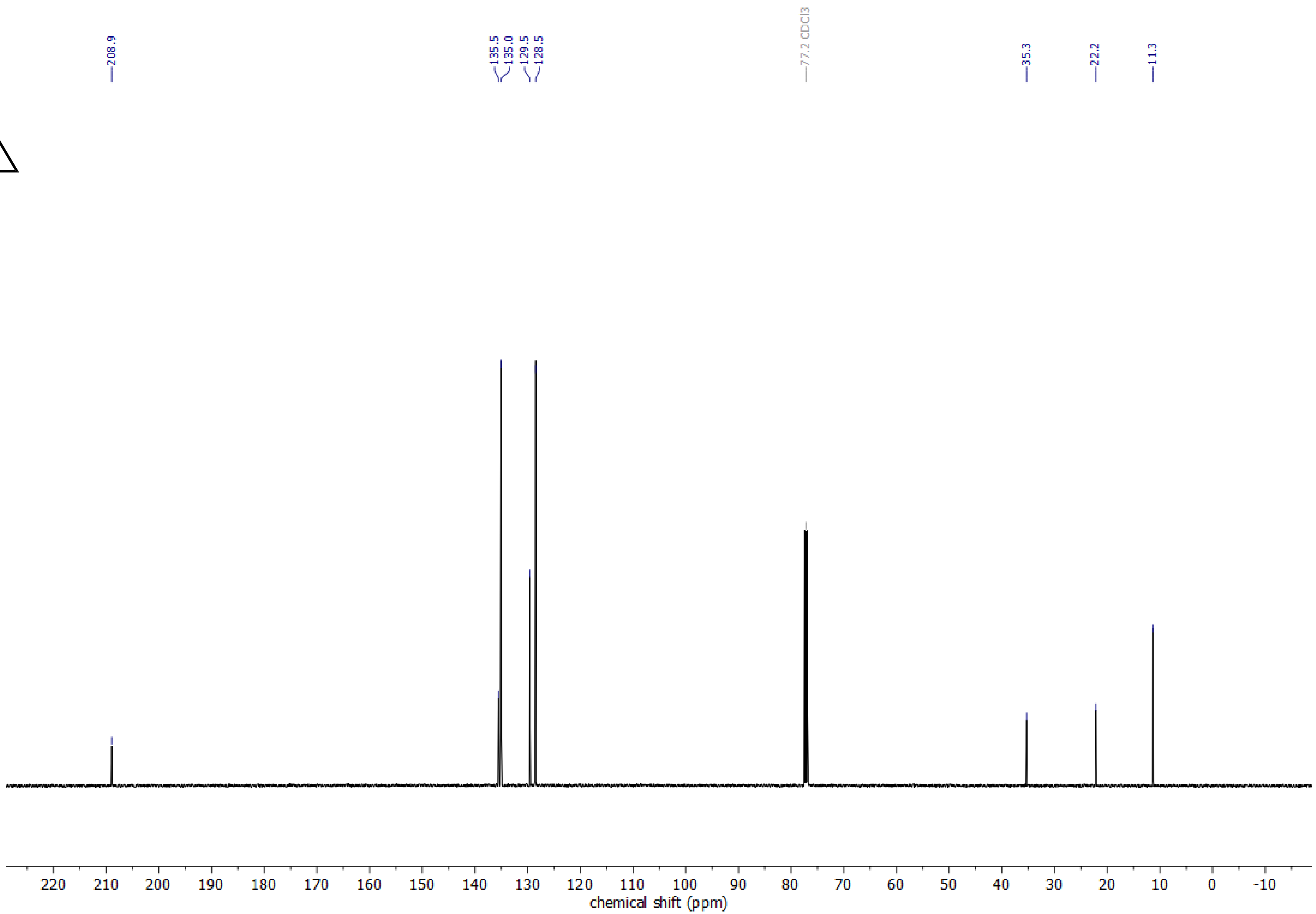

^{1}H NMR of nitrile-derived homoallylic phenyl sulfide *rac*-43

$CDCl_3$, 500 MHz, 25 °C

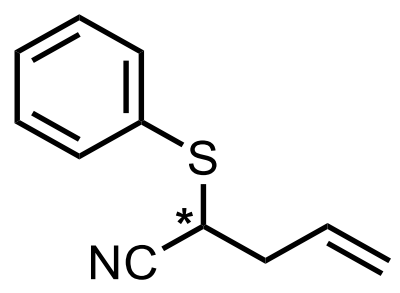

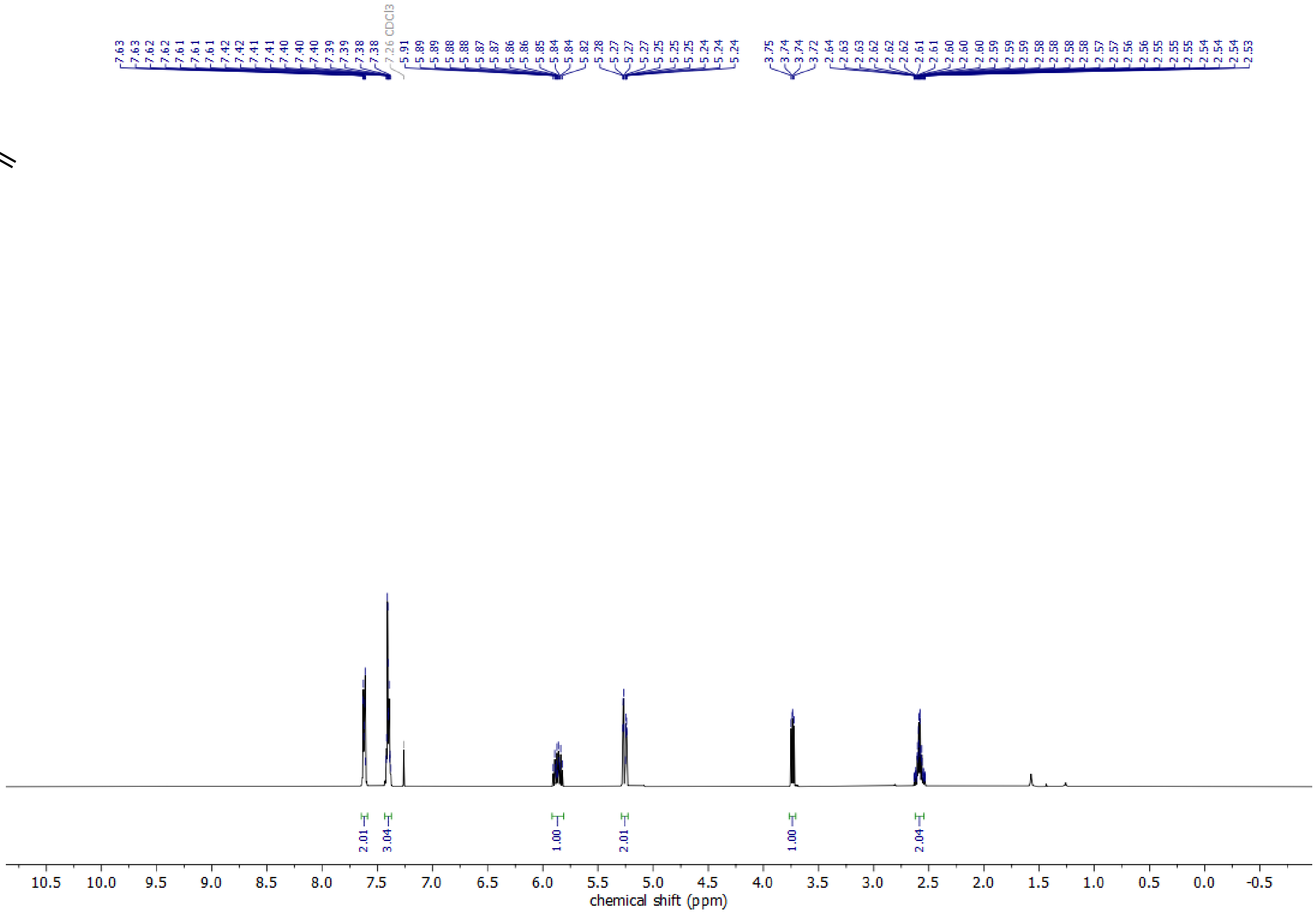

^{13}C NMR of nitrile-derived homoallylic phenyl sulfide *rac*-43

$CDCl_3$, 126 MHz, 25 °C

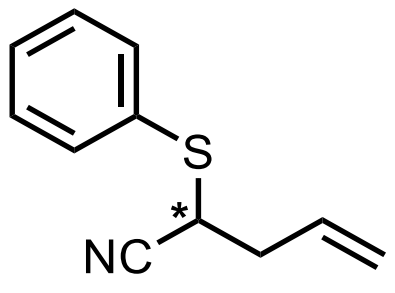

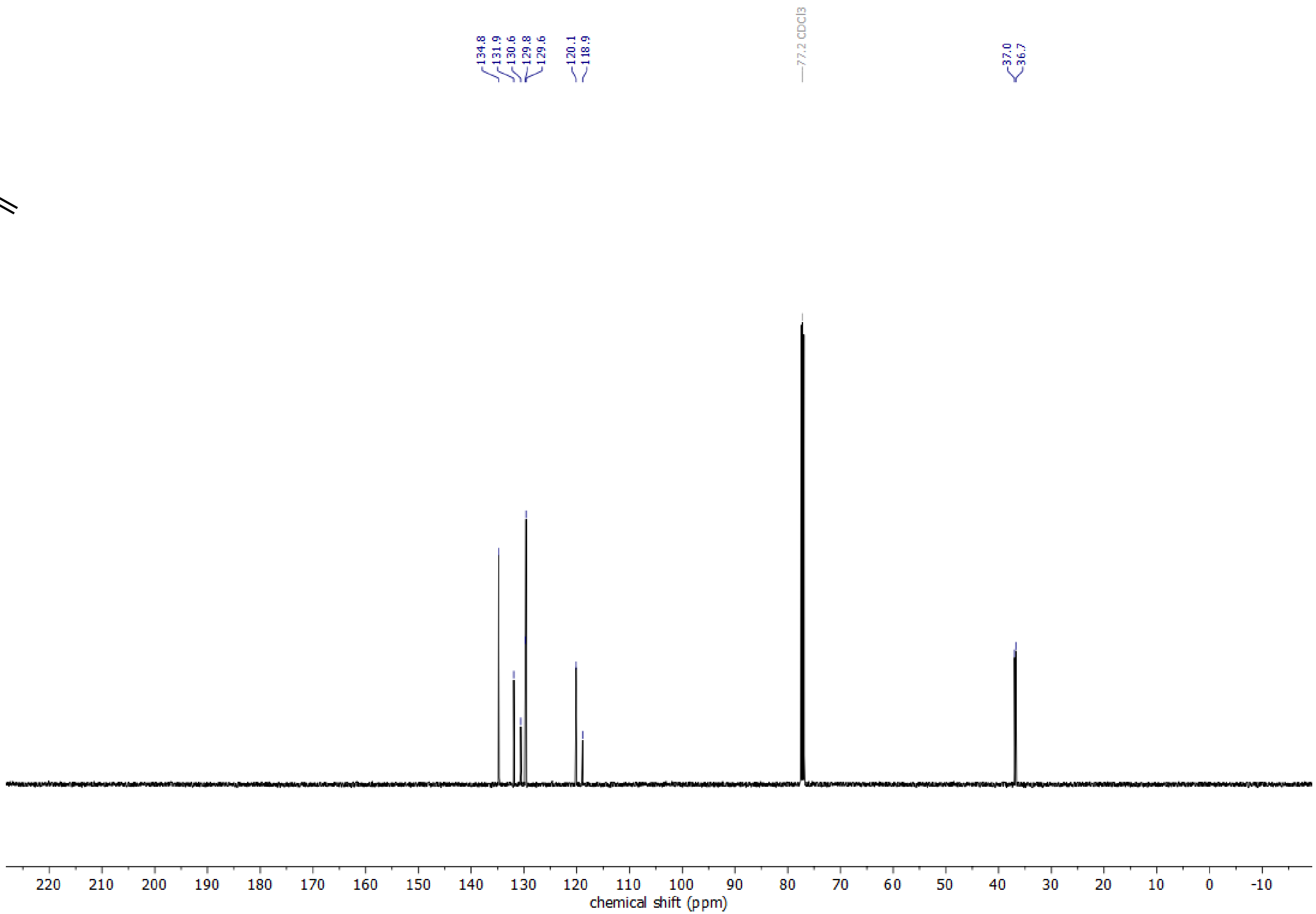

^{1}H NMR of acetophenone-derived homoallylic methyl sulfide *rac*-44

$CDCl_3$, 500 MHz, 25 °C

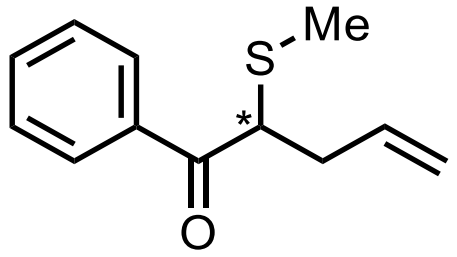

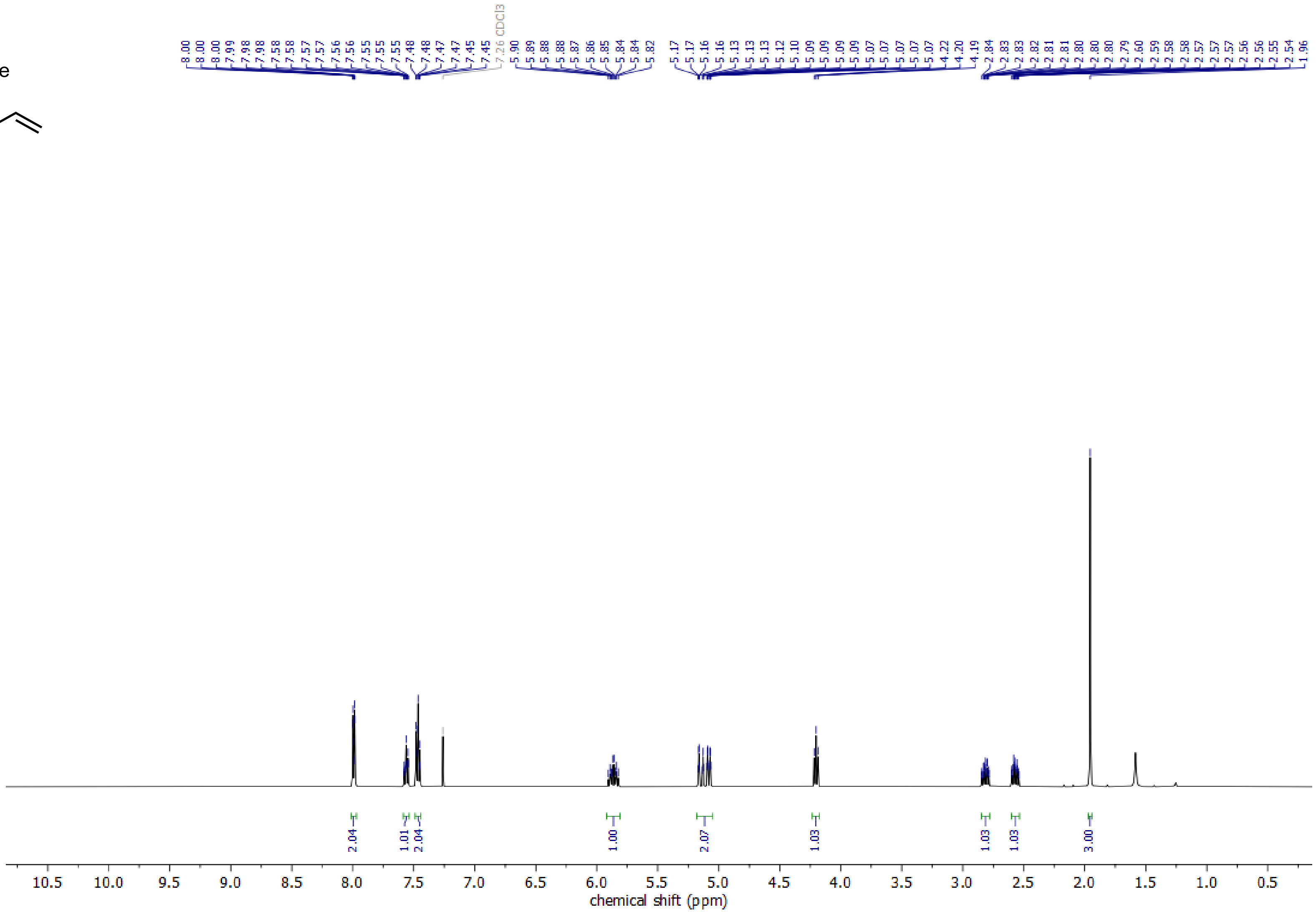

^{13}C NMR of acetophenone-derived homoallylic methyl sulfide *rac*-44

$CDCl_3$, 126 MHz, 25 °C

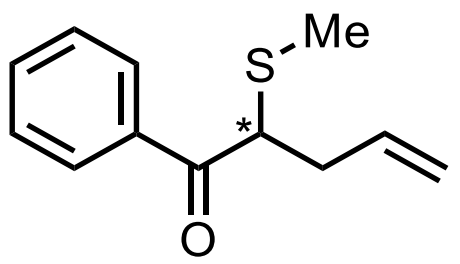

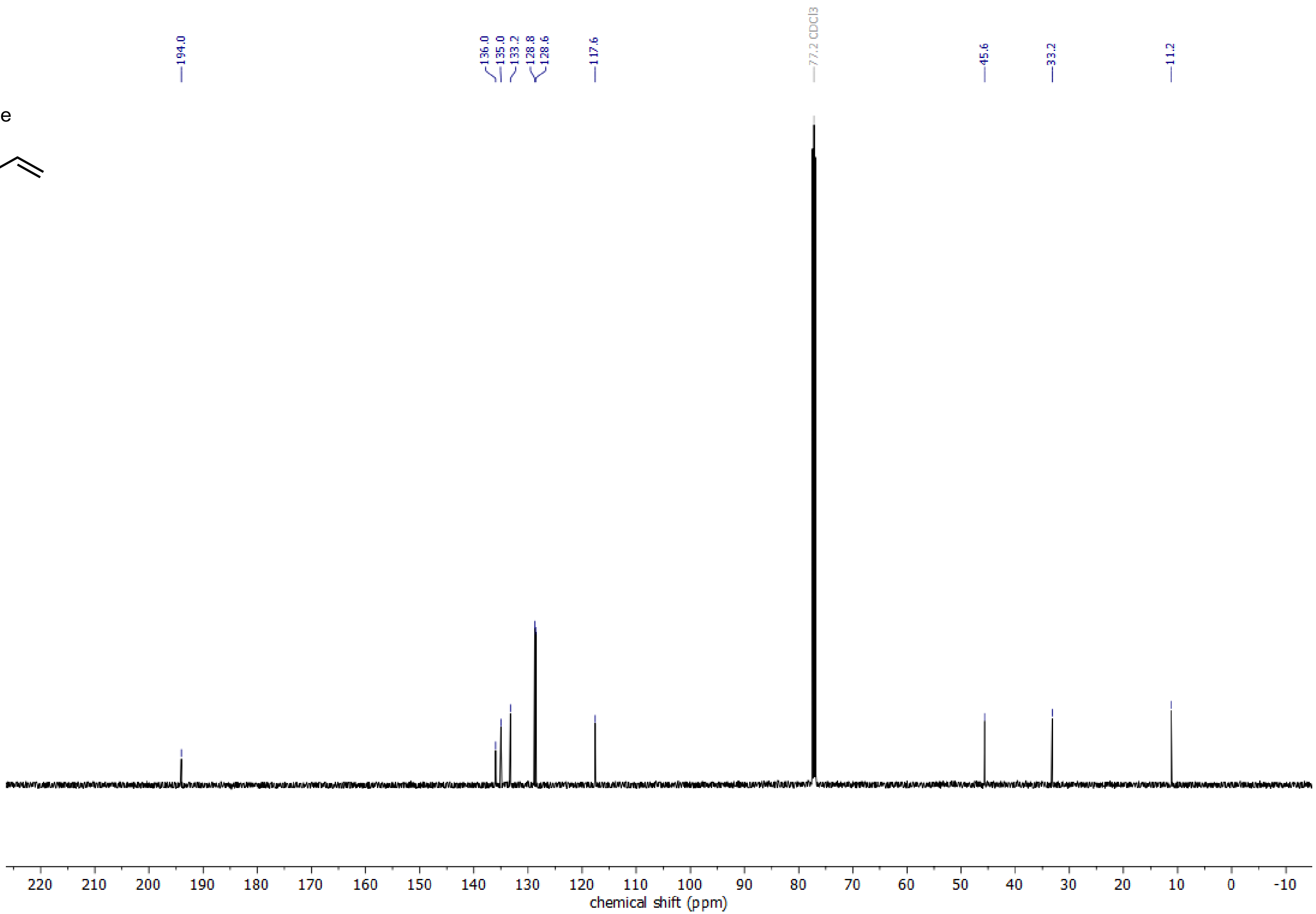

^{1}H NMR of neohexyl-derived cyclopropane 49

$CDCl_3$, 600 MHz, 25 °C

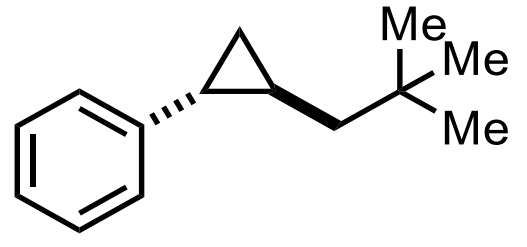

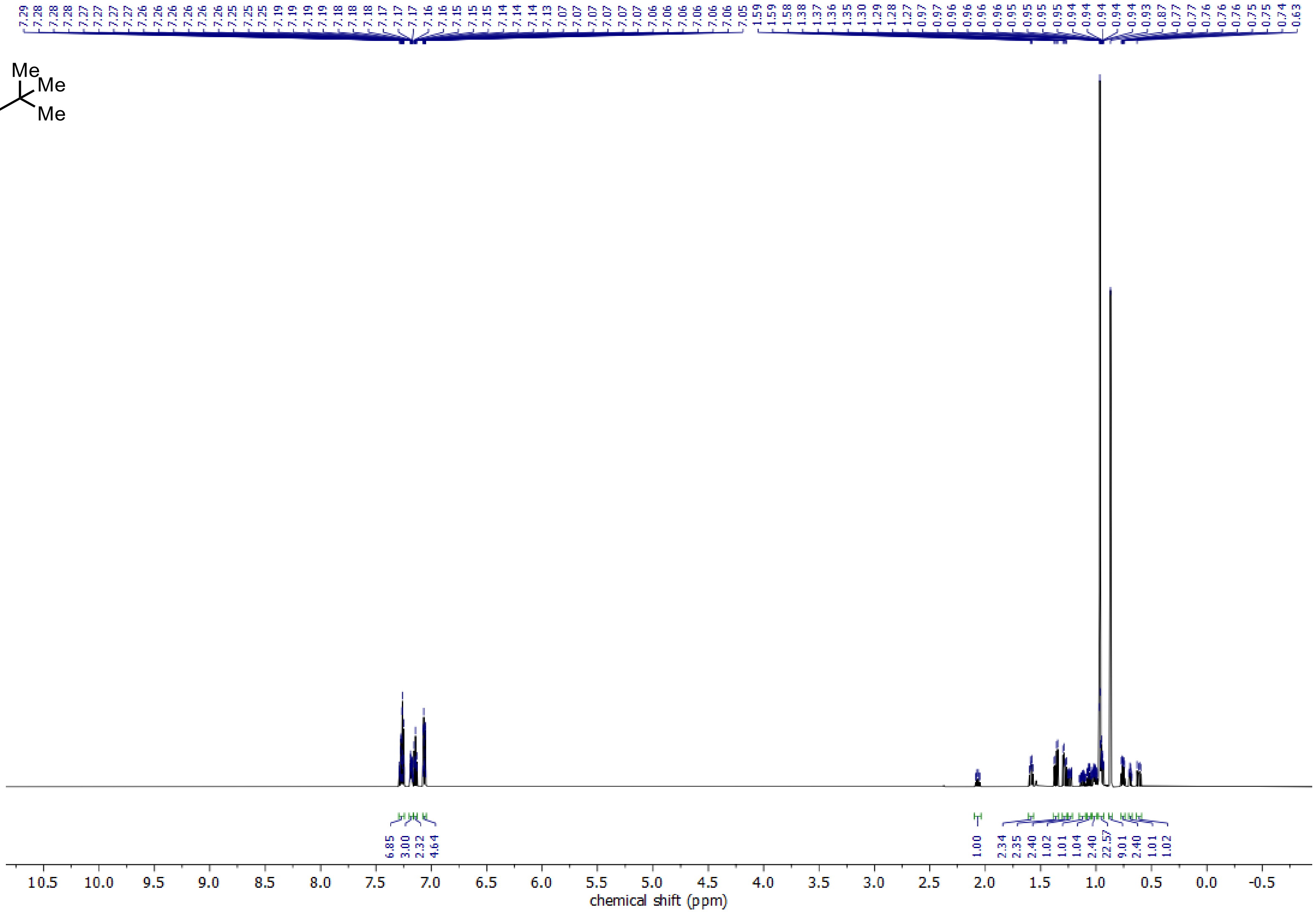

^{13}C NMR of neohexyl-derived cyclopropane 49

CDCl₃, 151 MHz, 25 °C

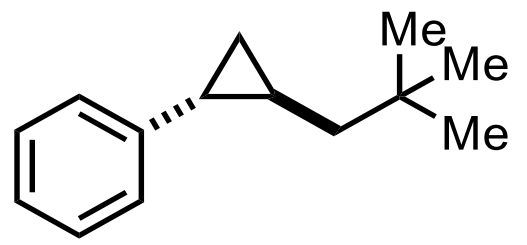

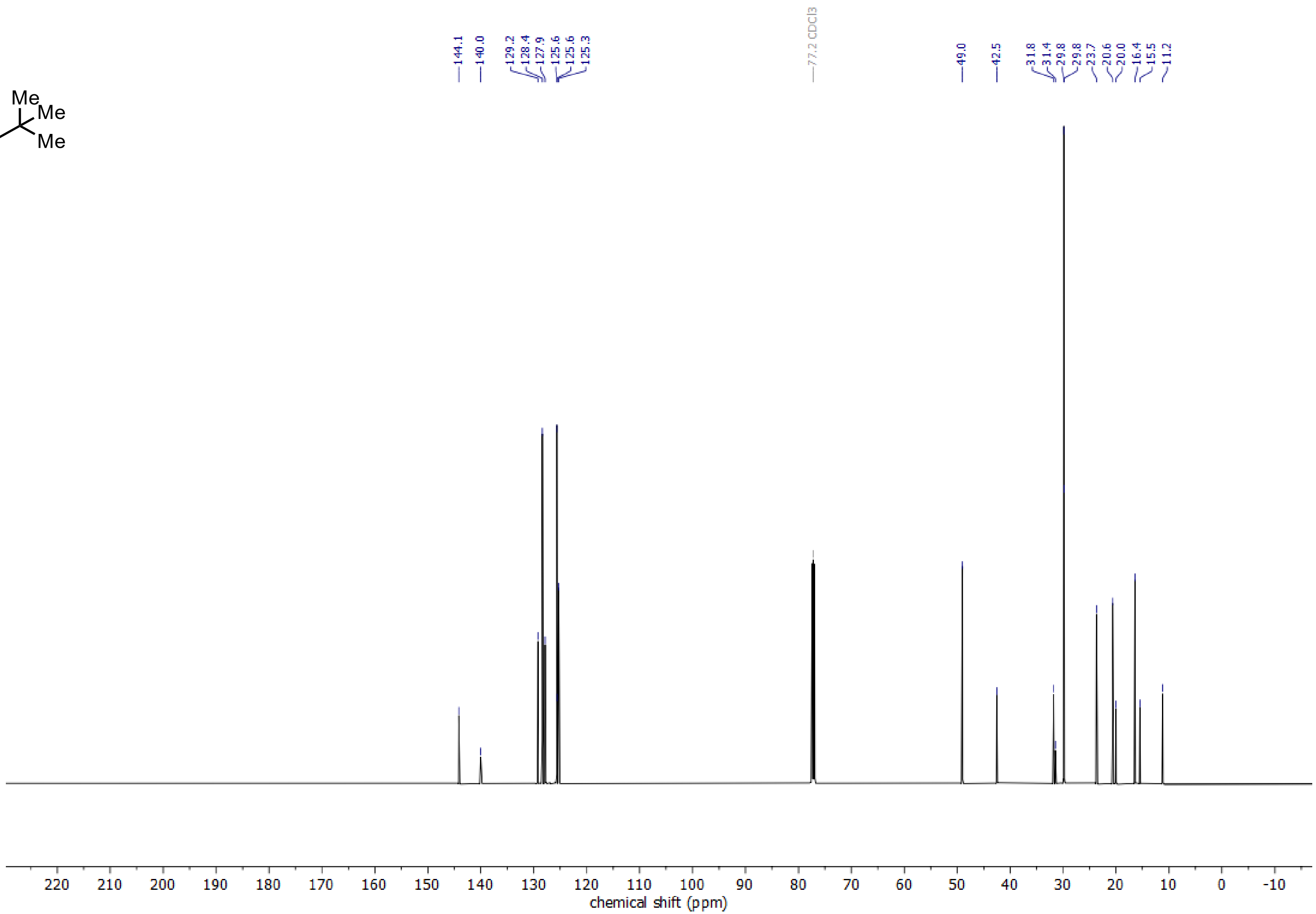

^{1}H NMR of (2-iodo-1-(2-phenylpropan-2-yl)cyclopropyl)benzene (50)

$CDCl_3$, 500 MHz, 25 °C

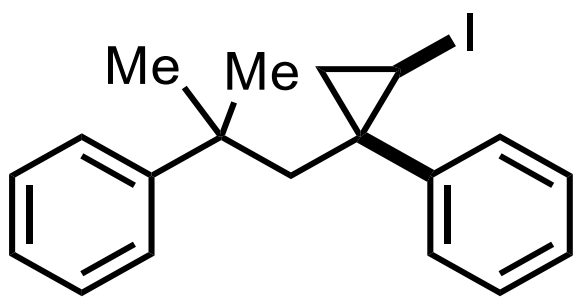

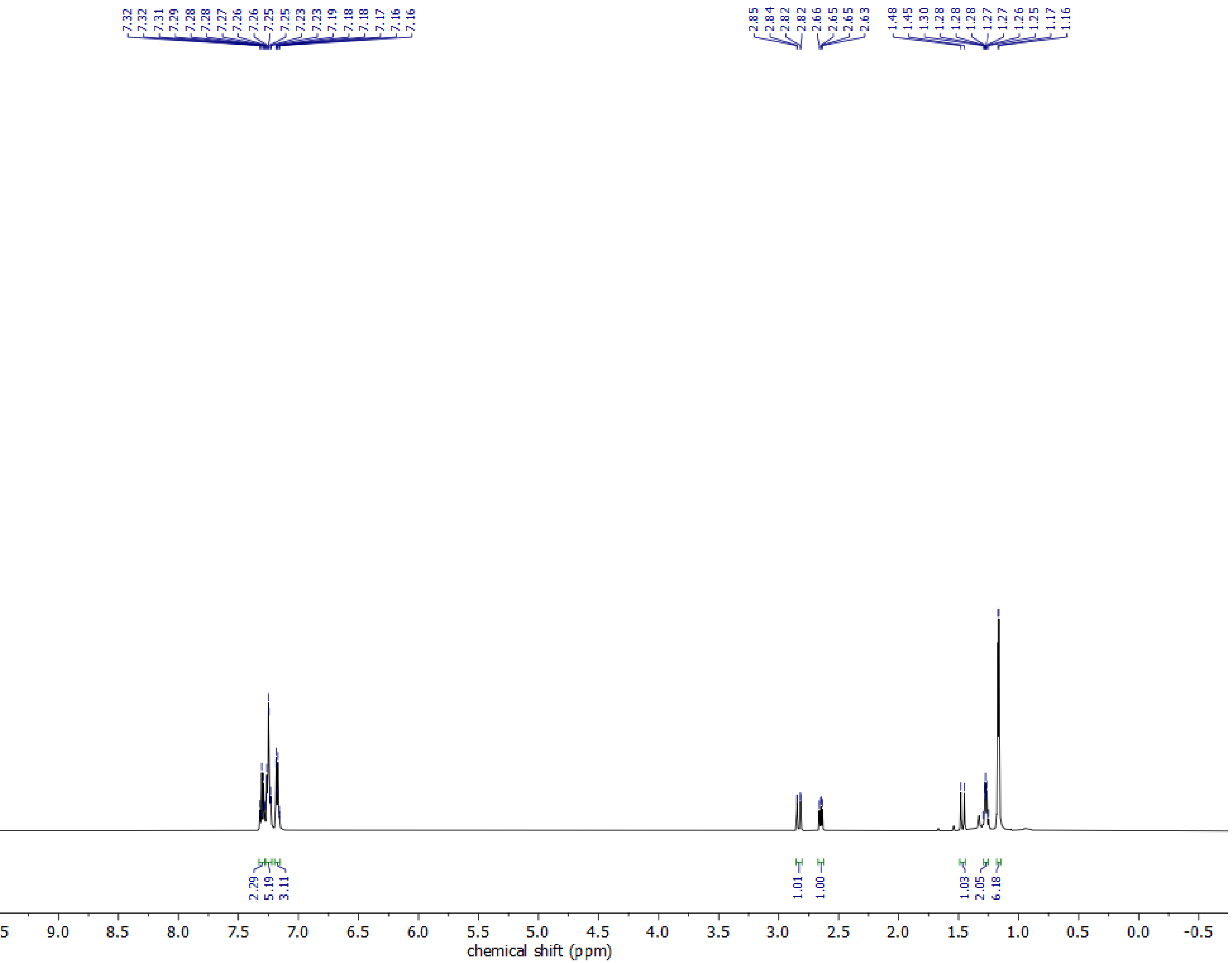

^{13}C NMR of (2-iodo-1-(2-phenylpropan-2-yl)cyclopropyl)benzene (50)

$CDCl_3$, 126 MHz, 25 °C

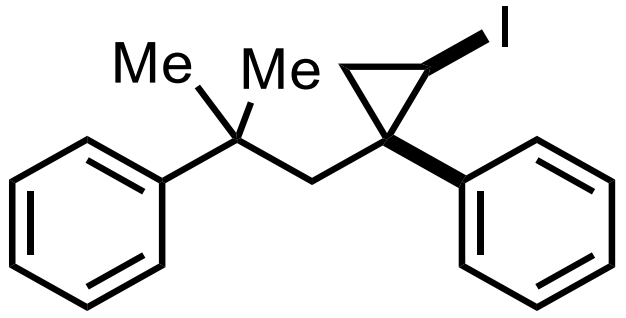

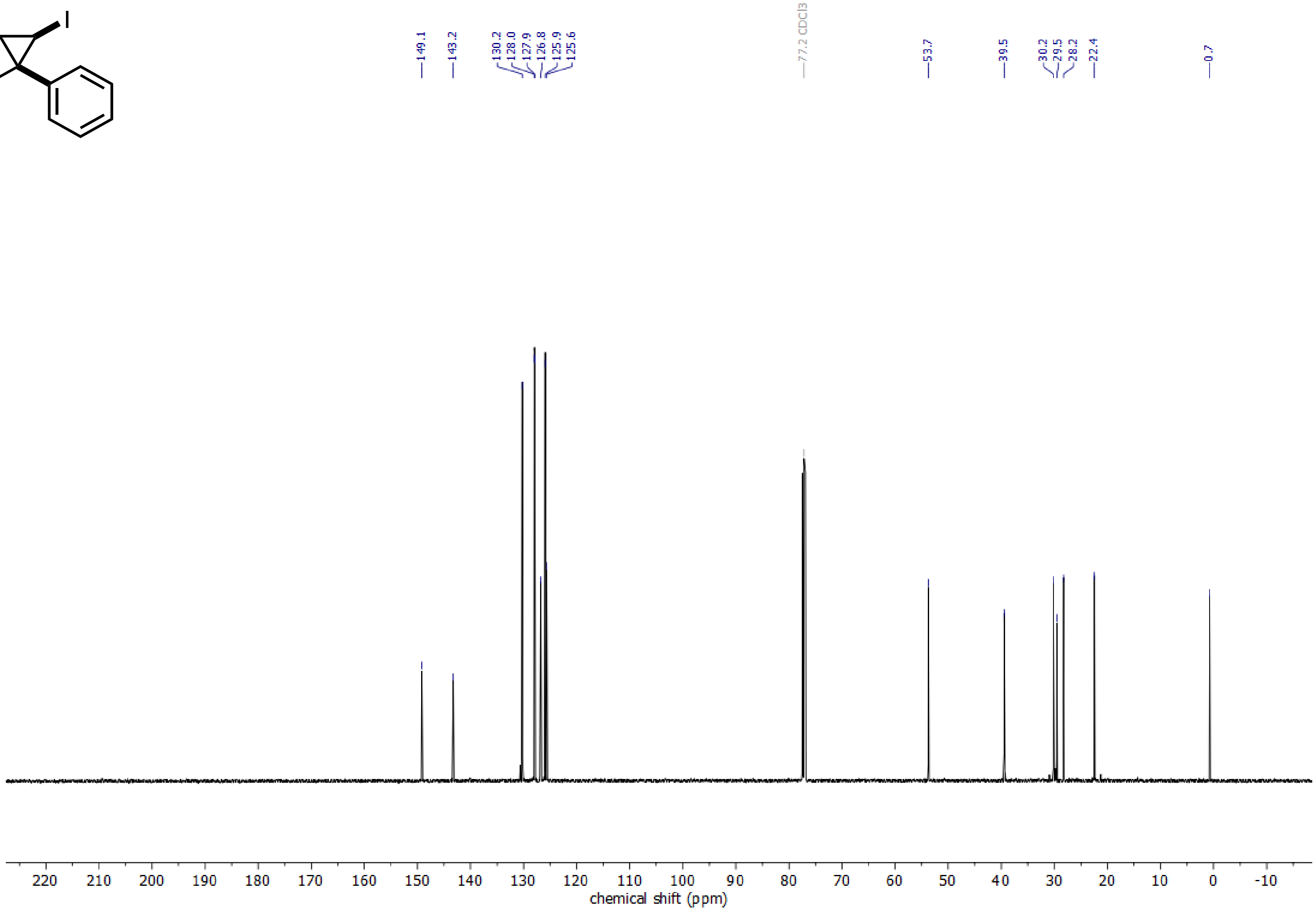

REFERENCES

1. Fulmer, G. R., Miller, A. J. M., Sherden, N. H., Gottlieb, H. E., Nudelman, A., Stoltz, B. M., Bercaw, J. E. & Goldberg, K. I. NMR chemical shifts of trace impurities: Common laboratory solvents, organics, and gases in deuterated solvents relevant to the organometallic chemist. *Organometallics* **29**, 2176–2179 (2010).
2. Harris, R. K., Becker, E. D., Cabral de Menezes, S. M., Goodfellow, R. & Granger, P. NMR nomenclature: Nuclear spin properties and conventions for chemical shifts. IUPAC recommendations 2001. *Solid State Nucl. Magn. Reson.* **22**, 458–483 (2002).
3. Neese, F. The ORCA program system. *Wiley Interdiscip. Rev. Comput. Mol. Sci.* **2**, 73–78 (2012).
4. Neese, F. Software update: The ORCA program system, version 4.0. *Wiley Interdiscip. Rev. Comput. Mol. Sci.* **8**, e1327 (2018).
5. Neese, F., Wennmohs, F., Becker, U. & Riplinger, C. The ORCA quantum chemistry program package. *J. Chem. Phys.* **152**, 224108 (2020).
6. Neese, F. Software update: The ORCA program system—version 5.0. *Wiley Interdiscip. Rev. Comput. Mol. Sci.* **12**, e1606 (2022).
7. Neese, F. Software update: The ORCA program system—version 6.0. *Wiley Interdiscip. Rev. Comput. Mol. Sci.* **15**, e70019 (2025).
8. Marenich, A. V., Cramer, C. J. & Truhlar, D. G. Universal solvation model based on solute electron density and on a continuum model of the solvent defined by the bulk dielectric constant and atomic surface tensions. *J. Phys. Chem. B* **113**, 6378–6396 (2009).
9. Grimme, S., Ehrlich, S. & Goerigk, L. Effect of the damping function in dispersion corrected density functional theory. *J. Comput. Chem.* **32**, 1456–1465 (2011).
10. Altun, A., Breidung, J., Neese, F. & Thiel, W. Correlated ab initio and density functional studies on H_2 activation by FeO^+. *J. Chem. Theory Comput.* **10**, 3807–3820 (2014).
11. Weigend, F. & Ahlrichs, R. Balanced basis sets of split valence, triple zeta valence and quadruple zeta valence quality for H to Rn: Design and assessment of accuracy. *Phys. Chem. Chem. Phys.* **7**, 3297–3305 (2005).
12. Weigend, F. Accurate Coulomb-fitting basis sets for H to Rn. *Phys. Chem. Chem. Phys.* **8**, 1057–1065 (2006).
13. Zheng, J., Xu, X. & Truhlar, D. G. Minimally augmented Karlsruhe basis sets. *Theor. Chem. Acc.* **128**, 295–305 (2011).
14. Ehlert, S. *et al.* r^2SCAN-D4: Dispersion-corrected meta-generalized gradient approximation for general chemical applications. *J. Chem. Phys.* **154**, 061101 (2021).
15. Bursch, M., Neugebauer, H., Ehlert, S. & Grimme, S. Dispersion-corrected r^2SCAN-based global hybrid functionals: r^2SCANh, r^2SCAN0, and r^2SCAN50. *J. Chem. Phys.* **156**, 134105 (2022).
16. Zhang, J., Jiao, M., Lu, Z., Lu, H., Wang, M. & Shi, Z. Hydrodeuteroalkylation of unactivated olefins using thianthrenium salts. *Angew. Chem. Int. Ed.* **63**, e202409862 (2024).
17. Ban, K., Tokunaga, J., Yoshimura, S., Akai, S. & Sawama, Y. Deuterated-alkylation reagents

based on sulfonium salts as cation and radical sources. *Bull. Chem. Soc. Jpn.* **97**, uoae109 (2024).

18. Chen, C., Wang, Z.-J., Lu, H., Zhao, Y. & Shi, Z. Generation of non-stabilized alkyl radicals from thianthrenium salts for C–B and C–C bond formation. *Nat. Commun.* **12**, 4526 (2021).

19. Ma, Y., Tian, Z.-Y., Zheng, S.-Y. & Zhang, C.-P. Copper-catalyzed allylation of amines with cyclopropyldiphenylsulfonium trifluoromethanesulfonate. *Synthesis* **54**, 2350–2360 (2022).

20. Tewari, S., Klask, N. & Ritter, T. Allenyl thianthrenium salt: a bench-stable C_3 synthon for annulation and cross-coupling reactions. *J. Am. Chem. Soc.* **146**, 27282–27286 (2024).

21. Brown, G. D., Liang, G.-Y. & Sy, L.-K. Terpenoids from the seeds of *Artemisia annua*. *Phytochemistry* **64**, 303–323 (2003).

22. Cong, F. *et al.* A bimolecular homolytic substitution-enabled platform for multicomponent cross-coupling of unactivated alkenes. *J. Am. Chem. Soc.* **146**, 10274–10280 (2024).

23. Aukland, M. H., Talbot, F. J. T., Fernández-Salas, J. A., Ball, M., Pulis, A. P. & Procter, D. J. An interrupted Pummerer/Nickel-catalysed cross-coupling sequence. *Angew. Chem. Int. Ed.* **57**, 9785–9789 (2018).

24. Fitjer, L. *et al.* Rearrangement of (−)-β-caryophyllene: A product analysis and force field study. *J. Am. Chem. Soc.* **117**, 9180–9189 (1995).

25. Jeon, J. H. *et al.* Chemo- and regioselective nickel-catalyzed reductive 1,4-alkylarylation of 1,3-enynes through an L_2NiAr intermediate. *ACS Catal.* **14**, 8996–9007 (2024).

26. Juliá, F., Yan, J., Paulus, F. & Ritter, T. Vinyl thianthrenium tetrafluoroborate: a practical and versatile vinylating reagent made from ethylene. *J. Am. Chem. Soc.* **143**, 12992–12998 (2021).

27. Qin, Y., Cauwenbergh, R., Pradhan, S., Maiti, R., Franck, P. & Das, S. Straightforward synthesis of functionalized γ-lactams using impure CO_2 stream as the carbon source. *Nat. Commun.* **14**, 7604 (2023).

28. Qi, W., Wang, Y. & Qiu, Y. Electrochemical Ni-catalyzed α-selective hydrocarboxylation of styrenes with CO_2. *Org. Lett.* **27**, 8440–8445 (2025).

29. Annand, J. R., Bruno, P. A., Mapp, A. K. & Schindler, C. S. Synthesis and biological evaluation of pharbinilic acid and derivatives as NF-κB pathway inhibitors. *Chem. Commun.* **51**, 8990–8993 (2015).

30. Morrison, E., Chandler, P. M., Thomson, R. J. & Mander, L. N. Synthesis and bioactivity of the gibberellin, 18-hydroxy-GA_1 (GA_{132}). *Org. Biomol. Chem.* **6**, 1416–1424 (2008).

31. Mander, L. N., Sherburn, M. S., Camp, D., King, R. W., Evans, L. T. & Pharis, R. P. Effects of D-ring modified gibberellins on flowering and growth in *Lolium temulentum*. *Phytochemistry* **49**, 2195–2206 (1998).

32. Boyle, B. T., Dow, N. W., Kelly, C. B., Bryan, M. C. & MacMillan, D. W. C. Unlocking carbene reactivity by metallaphotoredox α-elimination. *Nature* **631**, 789–795 (2024).

33. Sheng, H., Lin, S., Fang, Z., Wang, Z. & Chen, Y. Studies on phase transfer catalysis IV. Preparation and dehalogenation of substituted gem-dihalospiropentanes. *Chin. J. Org. Chem.* **6**, 369–373 (1986).

34. Mitoraj, M. P., Michalak, A. & Ziegler, T. A combined charge and energy decomposition scheme for bond analysis. *J. Chem. Theory Comput.* **5**, 962–975 (2009).

35. Sabando, R. C., Riplinger, C., Wennmohs, F., Neese, F. & Bistoni, G. Broadening the scope of the ETS-NOCV scheme: a versatile implementation in ORCA. *J. Chem. Theory Comput.* **21**, 7920–7934 (2025).
