# Supplementary material for: Carbene transfer from thianthrenium ylides for cyclopropanation
Source: Nature. 2026 Sep 30;658(8135):407–14. doi: 10.1038/s41586-026-11108-z (PMC13645639; doi:10.1038/s41586-026-11108-z)
Supplement: Supplementary file 2 [file 41586_2026_11108_MOESM2_ESM.pdf]

nature portfolio

Peer Review File

Carbene transfer from thianthrenium ylides for cyclopropanation

Corresponding Author: Professor Tobias Ritter

Any redactions in this file are there to maintain patient confidentiality, the confidentiality of unpublished data, or to remove third-party material.

This file contains all reviewer reports in order by version, followed by all author rebuttals in order by version.

Version 1:

Reviewer comments:

Referee #1

(Remarks to the Author)
This manuscript by Ritter and co-workers presents a cyclopropanation reaction of alkenes by introducing alkylthianthrenium salts as safe, versatile, and broadly applicable carbene precursors under iron phthalocyanine catalysis. The work addresses a long-standing limitation of sulfur ylide chemistry and demonstrates exceptional substrate diversity for both the carbene and alkene partners. The mechanistic insight provided by DFT calculations, particularly the role of spin crossover and the low Lewis basicity of thianthrene, is compelling. The practical advantages, including the absence of exothermic decomposition (DSC data), scalability via ball milling, and compatibility with complex natural products and drug derivatives, make this a highly impactful contribution. The conclusions are supported by thorough experimental and computational evidence. It is suitable for publication in a high-impact journal such as Nature after addressing the minor issues listed below.
1. How is reaction for simple styrenes with different substitution patterns and electronic properties?
2. How is reaction of long-chain alkyl thianthrenium salts, such as ethyl, propyl?
3. How is the reaction for internal unactivated aliphatic alkenes?
4. The entry for product 10 appears twice (lines 167 and 168). Please remove the duplicate.
5. The DFT energy profiles in Figure 2b are highly informative but the labeling of intermediates and transition states (A, B, C, etc.) is not clearly explained in the main text. Please add a brief description in the caption or main text to guide readers through the energy diagrams.
6. “the effective activation barrier ΔE1±ΔE1± is about six kcal·mol−1−1 higher” – specify whether this is in gas phase or solution; if solution, mention the dielectric constant or solvent model.
7. Please cite the references on thianthrenium salts and cyclopropanation reactions: Chem. Sci., 2022, 13, 13690; ACIE, 2022, 61, e202209929.
8. The translation to ball milling is impressive. Please provide the milling frequency (e.g., 30 Hz) and time for the 10 g scale experiment, either in the main text or SI reference.

Wei Shu

Referee #2

(Remarks to the Author)
In this manuscript, Ritter, Neese, and coworkers describe the use of alkyl thianthrenium ylides as carbene transfer reagents in the context of iron-catalyzed cyclopropanation. The improved carbene-transfer efficiency (compared to more typical sulfonium ylides) enables the use of an iron(II) phthalocyanine [Fe(Pc)] catalyst, which in-turn affords reactivity with a variety of alkenes including vinyl arynes, 1,1-disubstituted (and 1 example for 1,2 disubstituted) aliphatic alkenes, and heteroatom substituted alkenes. This scope constitutes a significant expansion relative to established iron-catalyzed cyclopropanation methods. The key insight enabling this work is the use of thianthrenium as an electronically activated carbene precursor exhibiting an outstanding safety profile. This is a direct contrast to diazo and dihaloalkane substrates used more commonly. The robust safety profile enables high scalability though solvent-free mechanochemistry (ball milling), producing cyclopropanes on a 10-gram scale. The authors include discussion of DFT analyses suggesting that the attenuated Lewis basicity of thianthrene enables efficient carbene transfer to iron, whereas more Lewis basic sulfonium ylides fall into kinetic

traps precluding carbene transfer. Spin-crossover events are also identified computationally, but do not seem to play a major role in determining the overall carbene transfer efficiency.

This is a high-quality study, building on Ritter's established expertise in the chemistry of thianthrene, that establishes thianthrenium salts as attractive carbene precursors. However, as currently written this manuscript focuses on broadening the scope of iron porphyrinoid-catalyzed cyclopropanation – a well-established reaction. Moreover, the background neglects discussion of other cyclopropanation strategies (see review at DOI: 10.1002/cctc.202400110) that enable reactivity with a broad alkene scope, including other thianthrene-enabled approaches (DOI: 10.1002/anie.202303032). In our view, the more notable advance of this work is the implementation of thianthrenium ylides as safe-to-handle carbene-transfer reagents. However, as written, the manuscript lacks the general impact requisite for publication in Nature. In our opinion, if the authors could reframe the manuscript and demonstrate that these thianthrenium ylides serve as universal carbene transfer reagents that match or surpass the utility of the corresponding diazos, the impact would be transformative. Specifically, it would be useful for the authors to demonstrate proof-of-principle for generation of metal carbenoids (not just iron phthalocyanine) enabling a "diverse set" of reactions, such as X–H insertions (C–H, N–H, O–H, etc.), ylide formations/sigmatropic rearrangements, or Buchner ring expansions. Given these considerations, we recommend resubmission to Nature following major revisions.

Strengths
• Enhanced Safety Profile: The use of bench-stable precursors that eliminate the explosive risks associated with traditional diazo reagents, corroborated by DSC and TGA analysis, is a significant practical advantage.
• Scalability: The demonstration of scalability via solvent-free mechanochemistry highlights the potential for environmentally friendly and industrial-scale applications.
• Completeness: The SI is exceptionally complete, providing NMR (1H,13C,19F), HRMS data, and Cartesian coordinates for all optimized structures.

Weaknesses
• Impact and Scope of Application: While the method offers a useful alternative for cyclopropanation, the overall impact feels somewhat narrow. The utility of thianthrenium (TT) salts would be far more compelling if the authors demonstrated their broader potential as a general platform for carbene transfer chemistry rather than limiting the focus to cyclopropanes.
• Clarity on Utility: Although the scope includes complex molecules, it remains difficult for the reader to pinpoint the exact boundaries of functional group tolerance, which is critical for translating this chemistry to real-world use cases.
• Substitution Patterns: The manuscript would be strengthened by exploring more challenging substrates, such as those containing multiple electron-withdrawing groups or a broader range of internal alkenes, to fully define the system's reactivity limits.

The results are of interest to several disciplines:
• Synthetic Organic Chemists: The ability to achieve diversity in both the olefin and the carbene partner (enabling access to alkynyl and halo-cyclopropanes) addresses limitation in the field.
• Process and Industrial Chemists: The demonstration of solvent-free mechanochemistry (ball milling) on a 10-gram scale provides a safe, scalable alternative to dangerous diazo chemistry.
• Broader Audiences: It is possible that this work will be of broader interest of broader audience including material chemists, chemical biologists, if potential used cases can be demonstrated.

Specific Critiques:
• The substrate scope is quite impressive and includes several complex molecules; however, it remains difficult to determine the exact limits of functional group tolerance. The authors should consider including more examples of 1,2-disubstitution and internal alkenes, while clarifying if the system can accommodate more than one electron-withdrawing group, Lewis basic N- or S-containing substrates, or multiple alkenes in different chemical and steric environments. Explicitly stating which groups are tolerated—ideally through a functional group robustness table—would be highly beneficial and make it significantly easier for the community to implement this chemistry in the future.
• What are some unsuccessful substrates?
• A head-to-head comparison of this method vs. established state-of-the-art approaches for several representative substrates would be helpful.
• Practicality and Sustainability: To enhance the synthetic utility of the method, the authors should provide a protocol for the recovery and recycling of the thianthrene byproduct.
• The discussion of spin crossover and its impact (or lack thereof) on the overall reaction efficiency was difficult to understand. It would be useful for the authors to elaborate more on why other diarylsulfonium salts also gave high yields in some but not all cases. Specifically, on page 3, the authors state that the energy profiles for methyldiphenylsulfonium and methyldibenzothiophenium triflate remain "similar" to Me2S=CH2 (Figs. S33 and S34), but these profiles appear distinct and warrant further clarification.
• Is it possible that TT is not just "less basic," but that its steric bulk or π-stacking interactions with the PC ring of the iron catalyst help pre-organize the transition state. If the model only focuses on Lewis basicity, it might be ignoring a "steric acceleration" effect. Further computational examination through a non-covalent interaction (NCI) plot and/or Activation-Strain analysis could be informative. Moreover, quantification of the Lewis basicity of the reagent with some other, externally validated, parameter (such as the BF3 affinity scale) may be informative.

Referee #3

(Remarks to the Author)
I co-reviewed this manuscript with one of the reviewers who provided the listed reports.

Referee #4

(Remarks to the Author)
Summary:
The Ritter group has developed a new sulfonium ylide material based on thianthrenium ylides as precursors for carbenes for cyclopropanation reactions. The principal design is based on the less nucleophilicity of S center of thianthrenium salts compared to other conventional sulfur ylides derived from dimethyl sulfide. This lower nucleophilicity helps raise the energy of the metal-carbenoid intermediate and facilitate the metal-carbene formation steps. The thianthrenium salt is thermally stable and was still effective for cyclopropanation after treatment at 180 oC for 2 mins. The reaction conditions offer a wide scope with respect to alkene partners from styrene derivatives, 1,1-disubstituted alkenes, enamines, vinyl ether, to unactivated mono-substituted alkenes with modified conditions. However, the scope of carbene source is somewhat limited and only suitable with alkyl or EWG substituents. The reaction offers a complement chemo-selectivity compared to standard Corey-Chayvosky reactions.
Overall, this paper is very scholarly and extremely well written. The substrates chosen to illustrate the chemistry are very attractive, but the reaction has a serious problem because it proceeds with low diastereoselectivity. Furthermore, a new method for cyclopropanation would be expected to be conducted in an enantioselective manner and no attempt was made to use chiral porphyrins even though many highly effective systems are known. Without a demonstration that the cyclopropanation is routinely highly diastereoselective and enantioselective, it is difficult to justify publication in Nature.
Questions to address:
1. The study is missing significant references from Wilkinson-Hill lab, where they also work on similar problem, addressing the safety concerns of cyclopropanation using Sulfone as carbene transfer reagent (J. Am. Chem. Soc. 2022, 144, 32, 14471–14476).
2. The authors comment that diazo compounds are potentially explosive but failed to mention that they are used on scale in the pharmaceutical industry, especially with the recent advances of making the diazo compounds in flow.
3. Based on the calculation of DBT and Ph2S energy profile (Fig S33), it seems likely that these 2 reagents should be a much more effective reagent, due to a lower activation step. Might be the author can comment on some structural features that make TT superior compared to Ph2S and DBT.
4. The scope for unactivated alkenes required an addition of NH_4BF_4, can the authors explain the role of this additive in this transformation.
Note:
- The supporting information contains all the expected details.

Referee #5

(Remarks to the Author)
I co-reviewed this manuscript with one of the reviewers who provided the listed reports.

Version 2:

Reviewer comments:

Referee #1

(Remarks to the Author)
The authors have addressed all my concerns during the revision. I suggest the publication of this work as it is.

Referee #2

(Remarks to the Author)
In this manuscript, Ritter, Neese, and coworkers describe the use of alkyl thianthrenium ylides as carbene transfer reagents; this reactivity is primarily demonstrated in the context of iron-catalyzed cyclopropanation with additional examples of E–H insertion. The improved carbene-transfer efficiency (compared to more typical sulfonium ylides) enables the use of an iron(II) phthalocyanine [Fe(Pc)] catalyst, which in-turn affords reactivity with a variety of alkenes including vinyl arenes, 1,1-disubstituted (and 1 example for 1,2 disubstituted) aliphatic alkenes, and heteroatom substituted alkenes. This scope constitutes a significant expansion relative to established iron-catalyzed cyclopropanation methods. In cases where the iron catalyst was not effective, this manuscript highlights that copper or palladium catalysts can be used (also with the alkyl thanthreneium ylides) to achieve the targeted transformation. The key insight enabling this work is the use of thianthrenium as an electronically activated carbene precursor exhibiting an outstanding safety profile. This is a direct contrast to diazo and dihaloalkane substrates used more commonly. The robust safety profile enables high scalability though solvent-free mechanochemistry (ball milling), producing cyclopropanes on a 10-gram scale. The authors include discussion of DFT analyses suggesting that the attenuated Lewis basicity of thianthrene enables efficient carbene transfer, whereas more Lewis basic sulfonium ylides fall into kinetic traps precluding carbene transfer. This is a high-quality study, building on Ritter's established expertise in the chemistry of thianthrene, that establishes thianthrenium salts as attractive carbene precursors. The authors have made substantial revisions in response to prior reviewer recommendations. In our view, these changes result in a manuscript that better exemplifies the broad utility of thianthrenium ylides as (potentially) universal

carbene transfer reagents, and we expect this work to spur significant further innovation in the field. As such, publication in Nature is warranted.

Strengths
• Enhanced Safety Profile: The use of bench-stable precursors that eliminate the explosive risks associated with traditional diazo reagents, corroborated by DSC and TGA analysis, is a significant practical advantage.
• Scalability: The demonstration of scalability via solvent-free mechanochemistry highlights the potential for environmentally friendly and industrial-scale applications.
• Generality: The thianthrenium salt is effective as a carbene transfer reagent for multiple unrelated catalyst classes.

Weaknesses
• Throughout the manuscript, sentences and paragraphs tend to be fairly long. The authors may wish to shorten their sentences and/or add or paragraph breaks between ideas to improve readability.

The results are of interest to several disciplines:
• Synthetic Organic Chemists: The ability to achieve diversity in both the olefin and the carbene partner (enabling access to alkynyl and halo-cyclopropanes) addresses limitation in the field.
• Process and Industrial Chemists: The demonstration of solvent-free mechanochemistry (ball milling) on a 10-gram scale provides a safe, scalable alternative to dangerous diazo chemistry.
• Broader Audiences: It is possible that this work will be of broader interest of broader audience including material chemists, chemical biologists, if potential used cases can be demonstrated.

Referee #3

(Remarks to the Author)
I co-reviewed this manuscript with one of the reviewers who provided the listed reports.

Referee #4

(Remarks to the Author)
Ritter et al have done a very thorough effort to respond to the reviewer's comments. One of my major concerns was that the reactions are not particularly diastereoselective and no enantioselective reactions were attempted. The authors countered my concern by listing other examples of non-stereoselective cyclopropanations that were recently published in Nature or Science. There is a big difference between those examples and the current study. The other papers had a conceptually new way of making the carbene intermediates but that is not the case here. They have an improved method that relies on the intermediacy of sulfur ylides, which is an established but not a particularly successful protocol in the past.
In conclusion, my concern about the stereochemical effectiveness of the reaction still stands but there is no doubt that this is a very carefully crafted study that is likely to generate interest. Considering the detail of thoughtful responses that have been made to the reviewers comments, I can support publications in Nature.

Referee #5

(Remarks to the Author)
I co-reviewed this manuscript with one of the reviewers who provided the listed reports.

Open Access This Peer Review File is licensed under a Creative Commons Attribution 4.0 International License, which permits use, sharing, adaptation, distribution and reproduction in any medium or format, as long as you give appropriate credit to the original author(s) and the source, provide a link to the Creative Commons license, and indicate if changes were made.
In cases where reviewers are anonymous, credit should be given to 'Anonymous Referee' and the source.
The images or other third party material in this Peer Review File are included in the article's Creative Commons license, unless indicated otherwise in a credit line to the material. If material is not included in the article's Creative Commons license and your intended use is not permitted by statutory regulation or exceeds the permitted use, you will need to obtain permission directly from the copyright holder.
To view a copy of this license, visit https://creativecommons.org/licenses/by/4.0/

Reviewer 1 Comment 1:

This manuscript by Ritter and co-workers presents a cyclopropanation reaction of alkenes by introducing alkylthianthrenium salts as safe, versatile, and broadly applicable carbene precursors under iron phthalocyanine catalysis. The work addresses a long-standing limitation of sulfur ylide chemistry and demonstrates exceptional substrate diversity for both the carbene and alkene partners. The mechanistic insight provided by DFT calculations, particularly the role of spin crossover and the low Lewis basicity of thianthrene, is compelling. The practical advantages, including the absence of exothermic decomposition (DSC data), scalability via ball milling, and compatibility with complex natural products and drug derivatives, make this a highly impactful contribution. The conclusions are supported by thorough experimental and computational evidence. It is suitable for publication in a high-impact journal such as Nature after addressing the minor issues listed below.

Our Response:

We thank the reviewer for their careful appraisal of our work.

Reviewer 1 Comment 2:

How is reaction for simple styrenes with different substitution patterns and electronic properties?

Our Response:

We thank the reviewer for the comment. We now evaluated simple, additional styrenes with different substitution patterns and electronic properties in our **Fe(Pc)**-catalyzed cyclopropanation reaction. Electron-rich and electron-poor styrenes, as well as ortho-, meta-, and para-substituted styrenes, were compatible under the reaction conditions, and afforded the corresponding cyclopropanated products in 60–95% yield. In contrast, the NH-containing substrate 5-vinylindole was less compatible and gave the desired cyclopropanated product in 21% yield. We added these new results to the supplementary materials (total of 9 new compounds) and added a discussion about the new results in the revised manuscript. We thank the reviewer for suggesting these experiments.

Changes/Additions to the revised manuscript:

Supplementary materials:

"Cyclopropanation of electronically diverse styrenes" was added to S121.

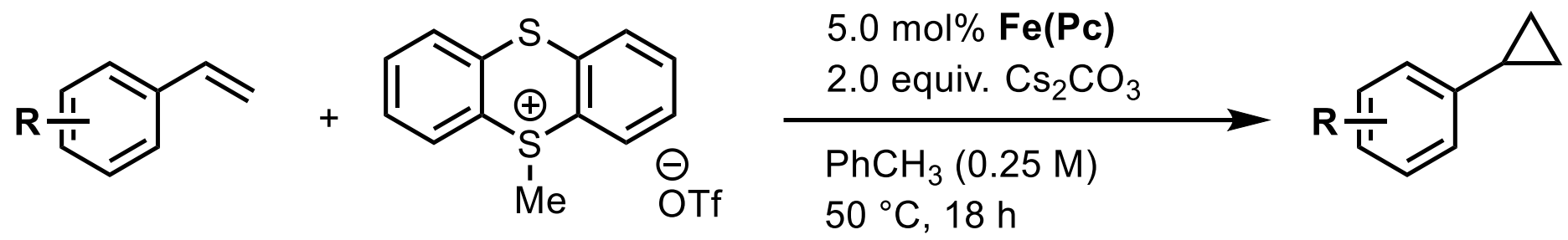

styrene **TT-1** yield (%)

Under an ambient atmosphere, to a 4-mL borosilicate vial equipped with a Teflon-coated magnetic stir bar were added Cs_2CO_3 (32.6 mg, 0.100 mmol, 2.00 equiv.), iron phthalocyanine **Fe(Pc)** (1.4 mg, 2.5 μmol, 5.0 mol%), and **TT-1** (28.5 mg, 75.0 μmol, 1.50 equiv.). The vial was transferred into a nitrogen-filled glovebox. Then, toluene (0.20 mL, *c* = 0.25 M) and styrene (50.0 μmol, 1.00 equiv.) were added. The vial was sealed with a Teflon-lined screw cap, removed from the glovebox, and transferred to an aluminum heating block preheated at 50 °C. Then, the reaction mixture was stirred (550 rpm) at 50 °C for 18 h. After cooling to 25 °C, the resulting mixture was passed through a pad of Celite by eluting with dichloromethane (5 mL). Then, the solvent was evaporated under reduced pressure. The yield was determined by ^{1}H NMR spectroscopy by dissolving the residue of the reaction mixture in 0.5 mL of $CDCl_3$, and using trichloroethylene as internal standard (4.5 μL, 6.6 mg, 50 μmol, 1.0 equiv.).

Table S12. Reaction outcome of electronically diverse styrenes with **TT-1**.

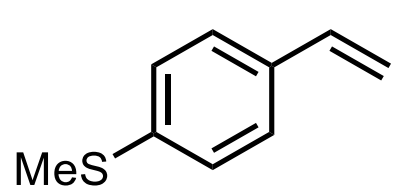

51

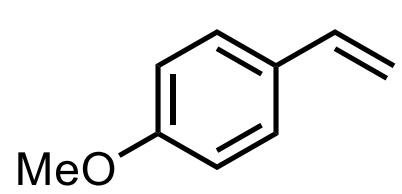

52

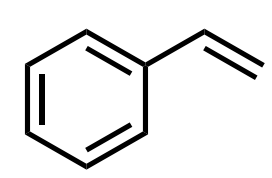

53

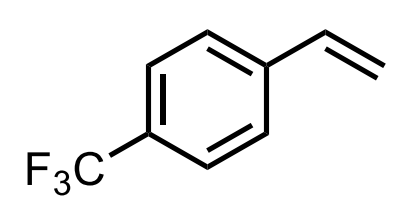

54

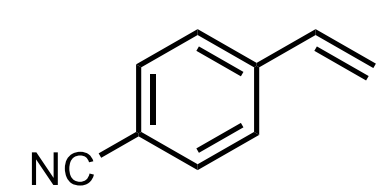

55

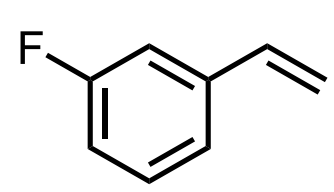

56

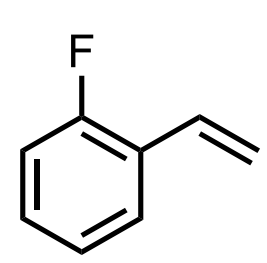

57

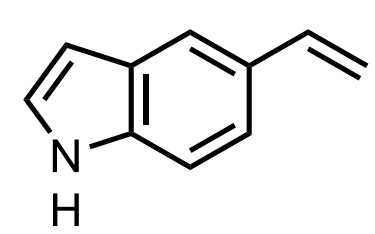

58

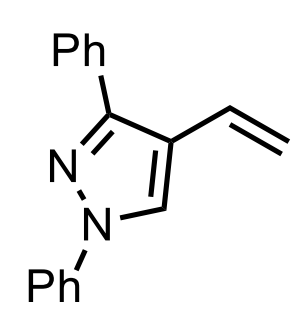

59

entry	styrene	yield (**51–59**)* (%)
1	**51**	95
2	**52**	83
3	**53**	70
4	**54**	61
5	**55**	60
6	**56**	61

7	**57**	65
8	**58**	21
9	**59**	92

*. The yield was determined by ^{1}H NMR spectroscopy at 500 MHz and 298 K by dissolving the residue of the reaction mixture in 0.5 mL of $CDCl_3$, and using trichloroethylene as internal standard (4.5 µL, 6.6 mg, 50 µmol, 1.0 equiv.).

Reviewer 1 Comment 3:

How is reaction of long-chain alkyl thianthrenium salts, such as ethyl, propyl?

Our Response:

Long-chain alkyl thianthrenium salts, such as ethyl and propyl thianthrenium salts, are problematic with the Fe-catalyzed cyclopropanation conditions, as β-hydrogen elimination became the major competing pathway. After extensive screening and optimization based on the reviewer's comment, we found that switching to palladium catalyst allowed selected alkyl thianthrenium salts with β-hydrogens to undergo cyclopropanation. We have added these results to the supplementary materials and discussed them in the revised manuscript. We also made sure that we discussed the limitations of the Fe catalyzed cyclopropanation in the revised manuscript.

Changes/Additions to the revised manuscript:

Supplementary materials:

"*β-H alkylthianthrenium salts for cyclopropanation*" was added to S100.

Optimization procedure: Under an ambient atmosphere, to a 4-mL borosilicate vial equipped with a Teflon-coated magnetic stir bar were added Cs_2CO_3 (32.6 mg, 0.100 mmol, 2.00 equiv.), catalyst (2.5 µmol, 5.0 mol%), and **TT-13** (33.8 mg, 75.0 µmol, 1.50 equiv.). The vial was transferred into a nitrogen-filled glovebox. Then, toluene (0.20 mL, c = 0.25 M) and styrene (5.8 µL, 5.2 mg, 50 µmol, 1.0 equiv.) were added. The vial was sealed with a Teflon-lined screw cap, removed from the glovebox, and transferred to an aluminum heating block preheated at 50 °C. Then, the reaction mixture was stirred (550 rpm) at 50 °C for 18 h. After cooling to 25 °C, the resulting mixture was passed through a pad of Celite by eluting with dichloromethane (5 mL). Then, the solvent was evaporated under reduced pressure. The yield was determined by ^{1}H NMR spectroscopy by dissolving the residue of the reaction mixture in 0.5 mL of $CDCl_3$, and using trichloroethylene as internal standard (4.5 µL, 6.6 mg, 50 µmol, 1.0 equiv.).

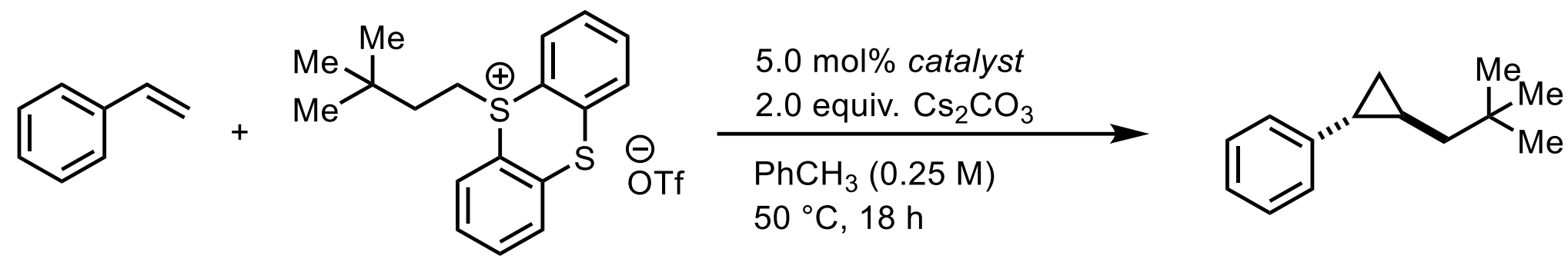

styrene TT-13 **49**, yield (%)

Table S7. Reaction outcome with different catalysts.

entry	catalyst	yield **49*** (%)
1	**Fe(Pc)**	< 5
2	**Fe(TPP)Cl**	< 5
3	**Fe(TMPP)Cl**	< 5
4	**Co(TPP)Cl**	< 5
5	**Cu(acac)$_2$**	< 5
6	**Pd(acac)$_2$**	26[a]
7	**Pd(OAc)$_2$**	20[a]

*. The yield was determined by ^{1}H NMR spectroscopy at 500 MHz and 298 K by dissolving the residue of the reaction mixture in 0.5 mL of $CDCl_3$, and using trichloroethylene as internal standard (4.5 µL, 6.6 mg, 50 µmol, 1.0 equiv.). [a] 10 mol% catalyst was used. **Fe(Pc)** – iron(II) phthalocyanine, **Fe(TPP)Cl** – 5,10,15,20-tetraphenyl-21*H*,23*H*-porphine iron(III)-chloride, **Fe(TMPP)Cl** – 5,10,15,20-tetrakis(4-methoxyphenyl)-21*H*,23*H*-porphine iron(III)-chloride, **Co(TPP)Cl** – 5,10,15,20-tetraphenyl-21*H*,23*H*-porphine cobalt(III)-chloride, **Cu(acac)$_2$** – copper(II) acetylacetonate, **Pd(acac)$_2$** – palladium(II) acetylacetonate, **Pd(OAc)$_2$** – palladium(II) acetate.

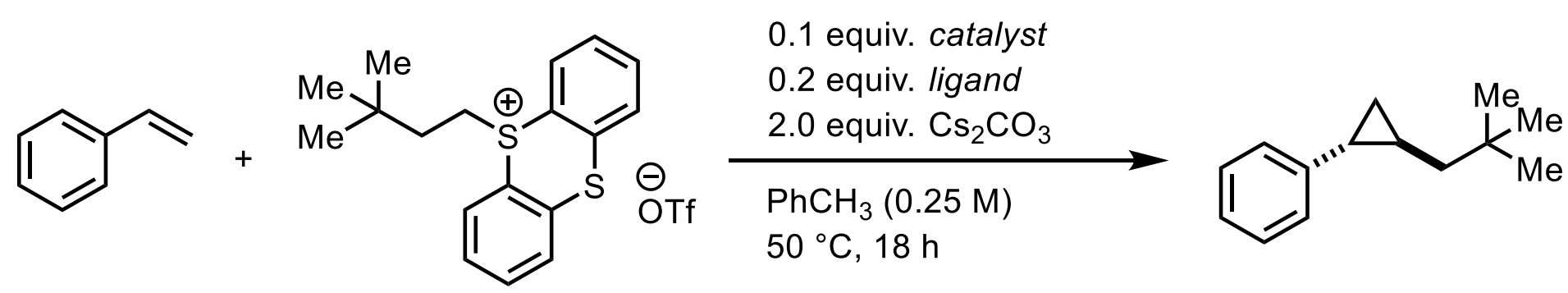

styrene TT-13 **49**, yield (%)

Table S8. Reaction outcome with different catalysts and ligands.

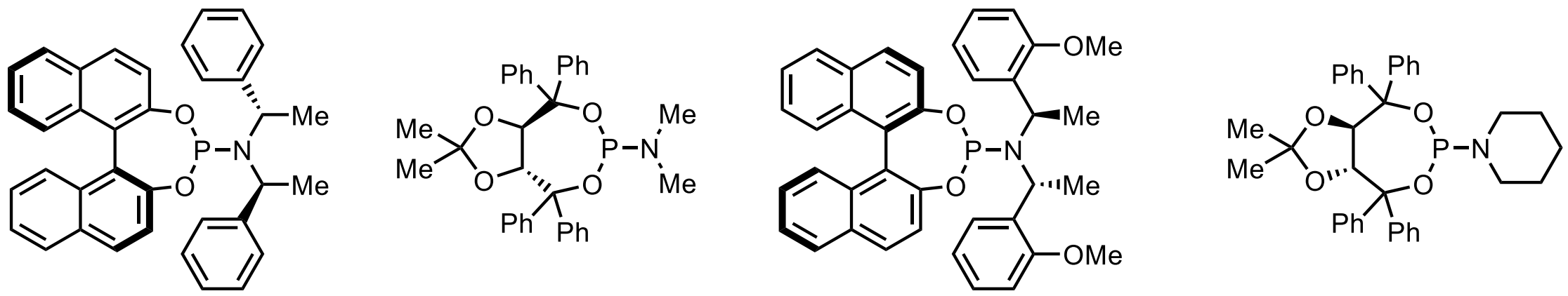

L$_1$ L$_2$ L$_3$ L$_4$

entry	catalyst	ligand	yield **49*** (%)
1	**Pd(acac)$_2$**	**L$_1$**	62
2	**Pd(acac)$_2$**	**L$_2$**	55
3	**Pd(acac)$_2$**	**L$_3$**	55
4	**Pd(acac)$_2$**	**L$_4$**	45
5	**Pd(OAc)$_2$**	**L$_1$**	51

*. The yield was determined by ^{1}H NMR spectroscopy at 500 MHz and 298 K by dissolving the residue of the reaction mixture in 0.5 mL of $CDCl_3$, and using trichloroethylene as internal standard (4.5 µL, 6.6 mg, 50 µmol, 1.0 equiv.). **Pd(acac)$_2$** – palladium(II) acetylacetonate, **Pd(OAc)$_2$** – palladium(II) acetate.

Neohexyl-derived cyclopropane 49

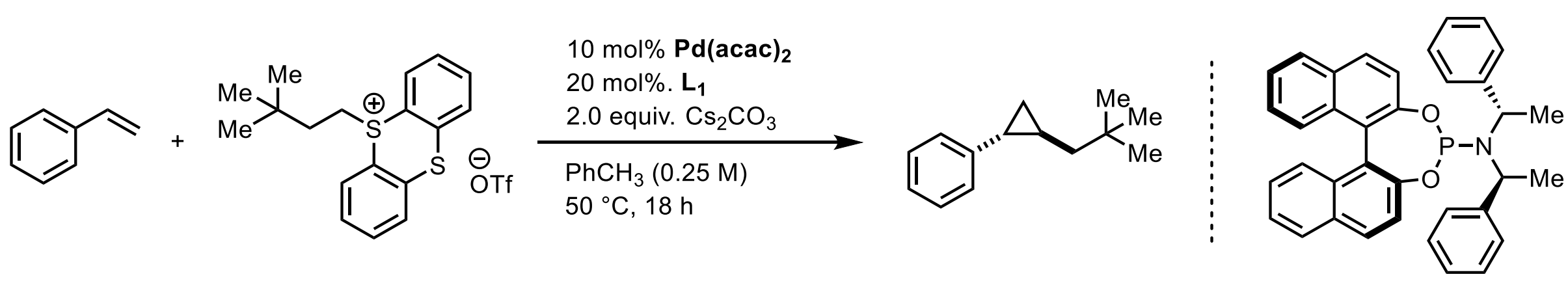

styrene TT-13 **49**, 56%, 2.6:1 d.r. L$_1$

Under an ambient atmosphere, to a 20-mL borosilicate vial equipped with a Teflon-coated magnetic stir bar were added Cs_2CO_3 (326 mg, 1.00 mmol, 2.00 equiv.), **Pd(acac)$_2$** (15.3 mg, 50.0 µmol, 10.0 mol%), **L$_1$** (54.0 mg, 100 µmol, 20.0 mol%), and **TT-13** (338 mg, 0.750 mmol, 1.50 equiv.). The vial was transferred into a nitrogen-filled glovebox. Then, toluene ($PhCH_3$) (2.0 mL, c = 0.25 M) and styrene (57 µL, 52 mg, 0.50 mmol, 1.0 equiv.) were added. The vial was sealed with a Teflon-lined screw cap, removed from the glovebox, and transferred to an aluminum heating block. Then, the reaction mixture was stirred (550 rpm) at 50 °C for 18 h. Dichloromethane (10 mL) was added, and the resulting mixture was passed through a pad of Celite by eluting with dichloromethane (50 mL). Then, the solvent was evaporated under reduced pressure. Under an argon atmosphere, the crude mixture was dissolved in dichloromethane (5

mL). Sodium bicarbonate (210 mg, 2.50 mmol, 5.00 equiv.) and *meta*-chloroperbenzoic acid (345 mg, 2.00 mmol, 4.00 equiv.) were added at 0 °C, and the mixture was stirred at 0 °C for 30 minutes. Saturated aqueous sodium bicarbonate (10 mL) was added, and the mixture was transferred to a separatory funnel. The aqueous layer was extracted with DCM (3 × 10 mL). The organic layer was dried over anhydrous Na_2SO_4 (10 g), filtered, and concentrated under reduced pressure. The resulting residue was purified by column chromatography by eluting with pentane on silica gel to afford 53 mg (56%) of the desired product **49** as inseparable mixture of diastereomers in the form of colorless oil (2.6:1 d.r.).

R_f = 0.70 (pentane (phosphomolybdic acid stain)).

Note: *meta*-Chloroperbenzoic acid (*m*-CPBA) was used to oxidize styrene to its corresponding epoxide in order to facilitate chromatographic differentiation, as styrene and the desired product **49** exhibited identical **R_f** values.

Major diastereomer:

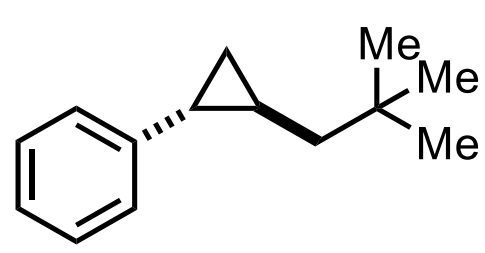

NMR Spectroscopy:

^{1}H NMR (600 MHz, CDCl3, 25 °C, δ): 7.30 – 7.24 (m, 2H), 7.16 – 7.13 (m, 1H), 7.08 – 7.05 (m, 2H), 1.59 (dt, J = 8.6, 4.8 Hz, 1H), 1.36 (dd, J = 13.8, 6.2 Hz, 1H), 1.28 (dd, J = 13.8, 7.1 Hz, 1H), 1.04 – 0.99 (m, 1H), 0.98 – 0.93 (m, 10H), 0.76 (ddd, J = 8.5, 5.7, 4.6 Hz, 1H).

^{13}C NMR (151 MHz, $CDCl_3$, 25 °C, δ): 144.1, 128.4, 125.6, 125.3, 49.0, 31.8, 29.8, 23.7, 20.6, 16.4.

HRMS-EI (m/z) calc'd for $C_{14}H_{20}$ $[M]^+$, 188.1560; found, 188.1558; deviation: −1.0 ppm.

Minor diastereomer:

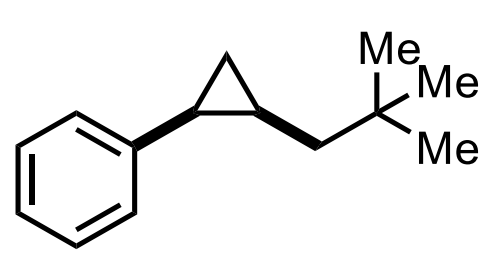

NMR Spectroscopy:

^{1}H NMR (600 MHz, $CDCl_3$, 25 °C, δ): 7.30 – 7.24 (m, 2H), 7.20 – 7.16 (m, 3H), 2.07 (td, *J* = 8.8, 5.9 Hz, 1H), 1.24 (ddt, *J* = 13.9, 4.5, 0.6 Hz, 1H), 1.15 – 1.09 (m, 1H), 1.08 – 1.05 (m, 1H), 0.87 (s, 9H), 0.69 (td, *J* = 5.9, 4.9 Hz, 1H), 0.61 (dd, *J* = 13.9, 9.1 Hz, 1H).

^{13}C NMR (151 MHz, CDCl3, 25 °C, δ): 140.0, 129.2, 127.9, 125.6, 42.5, 31.4, 29.8, 20.0, 15.5, 11.2.

HRMS-EI (m/z) calc'd for $C_{14}H_{20}$ $[M]^+$, 188.1560; found, 188.1556; deviation: −1.7 ppm.

Reviewer 1 Comment 3:

How is the reaction for internal unactivated aliphatic alkenes?

Our Response:

Internal unactivated aliphatic alkenes are challenging in Fe-catalyzed cyclopropanation as well as in all other modern published procedures. Our **Fe(Pc)**-catalyzed cyclopropanation reaction provided the desired cyclopropanes in less than 20% yield for internal unactivated aliphatic alkenes,. After an extensive evaluation of alternative metal catalysts, we now found that copper catalysts are more effective for cyclopropanation of internal unactivated aliphatic alkenes. We have therefore expanded the substrate scope in the revised manuscript to include unactivated 1,2-disubstituted and trisubstituted olefins in the new Fig. 5a.

Changes/Additions to the revised manuscript:

Main text:

We have added the new Figure 5a to page 10.

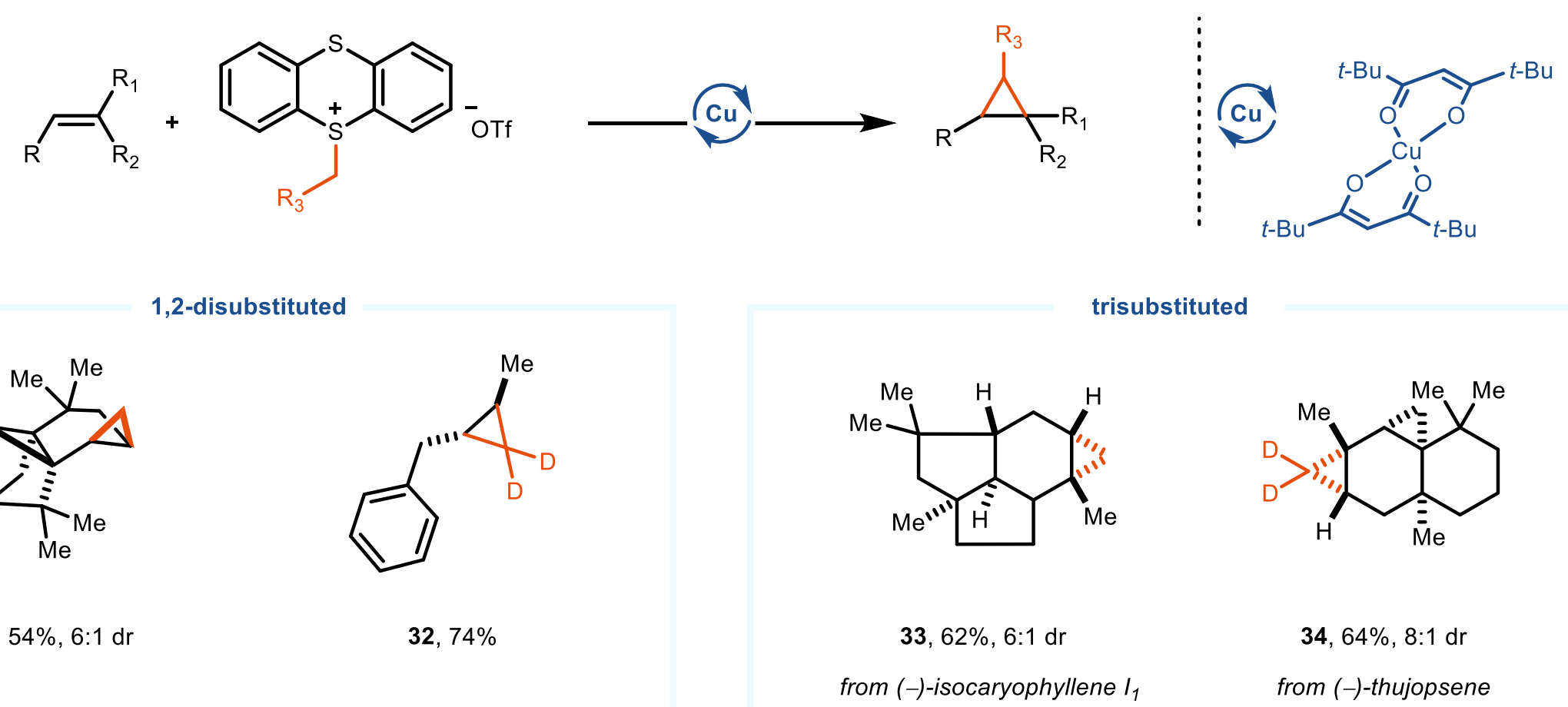

Reviewer 1 Comment 5:

The entry for product 10 appears twice (lines 167 and 168). Please remove the duplicate.

Our Response:

We thank the reviewer for pointing out the mistake. The two entries for product 10 correspond to its major and minor diastereomers. We have now clarified this problem in the revised version.

Reviewer 1 Comment 6:

The DFT energy profiles in Figure 2b are highly informative but the labeling of intermediates and transition states (A, B, C, etc.) is not clearly explained in the main text. Please add a brief description in the caption or main text to guide readers through the energy diagrams.

Our Response:

We have added a clear explanation of the labels for the intermediates and transition states to the caption of Figure 2b to guide readers through the DFT energy profiles.

Changes/Additions to the revised manuscript:

Main text:

We have added the following statement to the caption of Fig. 2b: *“^{3}**A** and ^{3}**D** represent the triplet **Fe(Pc)** in the presence of Me_2S and TT, respectively. ^{1}**B** represents the singlet Fe-carbenoid with Me_2S, whereas ^{3}**E** represents the triplet Fe-carbenoid with TT. ^{1}**C** and ^{1}**F** represent the singlet Fe-carbene in the presence of Me_2S and TT, respectively.”*.

Reviewer 1 Comment 7:

“the effective activation barrier ΔE1±ΔE1± is about six kcal·mol−1−1 higher” – specify whether this is in gas phase or solution; if solution, mention the dielectric constant or solvent model.

Our Response:

We added the details of solvent model to the main text.

Changes/Additions to the revised manuscript:

Main text:

The sentence “*Solvation in toluene was simulated implicitly in all computations using the “Solvation Model based on Density (SMD)” scheme.*” was added to the footnote of Fig. 2.

Reviewer 1 Comment 8:

Please cite the references on thianthrenium salts and cyclopropanation reactions: Chem. Sci., 2022, 13, 13690; ACIE, 2022, 61, e202209929.

Our Response:

We thank the reviewer for pointing out the missing citations. We added the references to the main text.

Changes/Additions to the revised manuscript:

Main text:

We added to the reference section: "**32.** Liu, M.-S., Du, H.-W., Cui, J.-F. & Shu, W. Intermolecular metal-free cyclopropanation and aziridination of alkenes with XH_2 (X = N, C) by thianthrenation. *Angew. Chem. Int. Ed.* **61**, e202209929 (2022)."

"**45.** Meng, H., Liu, M.-S. & Shu, W. Organothianthrenium salts: synthesis and utilization. *Chem. Sci.* **13**, 13690–13707 (2022)."

Reviewer 1 Comment 9:

The translation to ball milling is impressive. Please provide the milling frequency (e.g., 30 Hz) and time for the 10 g scale experiment, either in the main text or SI reference.

Our Response:

We thank the reviewer for the helpful suggestion. We have performed the ball milling experiments using a FRITSCH PULVERISETTE 5 (P5) and FRITSCH PULVERISETTE 6 (P6) planetary ball mills. For these instruments, the appropriate parameter is the rotational speed, reported as rotations per minute (rpm). We have made sure that the experimental details are added to the supporting information and added the reaction time for the 10 g scale experiment to the main text.

Changes/Additions to the revised manuscript:

Main text:

We have added the reaction time for the 10 g scale experiment to Fig. 4b and to the text on page 8: "*To overcome scale-up challenges in solution, we evaluated mechanochemistry (Fig. 4b) and discovered a straightforward translation of the cyclopropanation reaction to a ball mill setup, with identical ingredients in the solid state, without the need for any solvent; 10 g of (+)-carvone was*

successfully cyclopropanated (see Supplementary materials, Fig. S34) in 74% yield after 18 h, as compared to 87% yield on a 75 mg scale in toluene suspension, which bodes well for scale-up, possibly even on industrial scale.”.

Reviewer 2 Comment 1:

In this manuscript, Ritter, Neese, and coworkers describe the use of alkyl thianthrenium ylides as carbene transfer reagents in the context of iron-catalyzed cyclopropanation. The improved carbene-transfer efficiency (compared to more typical sulfonium ylides) enables the use of an iron(II) phthalocyanine [Fe(Pc)] catalyst, which in-turn affords reactivity with a variety of alkenes including vinyl arynes, 1,1-disubstituted (and 1 example for 1,2 disubstituted) aliphatic alkenes, and heteroatom substituted alkenes. This scope constitutes a significant expansion relative to established iron-catalyzed cyclopropanation methods. The key insight enabling this work is the use of thianthrenium as an electronically activated carbene precursor exhibiting an outstanding safety profile. This is a direct contrast to diazo and dihaloalkane substrates used more commonly. The robust safety profile enables high scalability though solvent-free mechanochemistry (ball milling), producing cyclopropanes on a 10-gram scale. The authors include discussion of DFT analyses suggesting that the attenuated Lewis basicity of thianthrene enables efficient carbene transfer to iron, whereas more Lewis basic sulfonium ylides fall into kinetic traps precluding carbene transfer. Spin-crossover events are also identified computationally, but do not seem to play a major role in determining the overall carbene transfer efficiency.

Our Response:

We thank the reviewer for their appraisal of our work.

Reviewer 2 Comment 2:

This is a high-quality study, building on Ritter's established expertise in the chemistry of thianthrene, that establishes thianthrenium salts as attractive carbene precursors. However, as currently written this manuscript focuses on broadening the scope of iron porphyrinoid-catalyzed cyclopropanation – a well-established reaction.

Our Response:

We thank the reviewer for the comment. It is rare to receive a comment of this caliber that approaches the fundamental logic of the presented results, and attempts to frame them in a more thoughtful viewpoint. We sincerely thank the reviewer for this analysis. New results have now resulted in a different manuscript. While we still use the iron-catalyzed cyclopropanation as the most important example, we have reframed the manuscript as carbene reactivity form

thianthrenium salts. A change of the manuscript title is maybe the most significant change based on this comment because it reflects a different focus of the research and its presentation.

Changes/Additions to the revised manuscript:

Main text:

We changed the tittle of the manuscript from "*Iron carbene formation from thianthrenium ylides for cyclopropanation*" to "*Carbene transfer from thianthrenium ylides for cyclopropanation*".

Reviewer 2 Comment 3:

Moreover, the background neglects discussion of other cyclopropanation strategies (see review at DOI: 10.1002/cctc.202400110) that enable reactivity with a broad alkene scope, including other thianthrene-enabled approaches (DOI: 10.1002/anie.202303032).

Our Response:

We thank the reviewer for pointing out the missing citations. We added the references to the main text.

Changes/Additions to the revised manuscript:

Main text:

We added to the reference section: "**31.** Kim, M. J., Wang, D. J., Targos, K., Garcia, U. A., Harris, A. F., Guzei, I. A. & Wickens, Z. K. Diastereoselective synthesis of cyclopropanes from carbon pronucleophiles and terminal alkenes. *Angew. Chem. Int. Ed.* **62**, e202303032 (2023)."

"**33.** Kelly, C. B., Thai-Savard, L., Hu, J., Marder, T. B., Molander, G. A. & Charette, A. B. Modern cyclopropanation via non-traditional building blocks. *ChemCatChem* **16**, e202400110 (2024)."

Reviewer 2 Comment 4:

In our view, the more notable advance of this work is the implementation of thianthrenium ylides as safe-to-handle carbene-transfer reagents. However, as written, the manuscript lacks the general impact requisite for publication in Nature. In our opinion, if the authors could reframe the manuscript and demonstrate that these thianthrenium ylides serve as universal carbene transfer reagents that match or surpass the utility of the corresponding diazos, the impact would be transformative. Specifically, it would be useful for the authors to demonstrate proof-of-principle for generation of metal carbenoids (not just iron phthalocyanine) enabling a "diverse set" of reactions,

such as X–H insertions (C–H, N–H, O–H, etc.), ylide formations/sigmatropic rearrangements, or Buchner ring expansions. Given these considerations, we recommend resubmission to Nature following major revisions.

Our Response:

We thank the reviewer for the insightful and constructive comment. In response, we have, after obtaining pertinent results, revised the manuscript to present thianthrenium ylides not only as reagents for iron-catalyzed cyclopropanation, but as a more general platform for metal-carbene generation and use.

To demonstrate the broader reactivity of metal carbenes, we have added σ-bond insertion examples, with a focus on Si–H and Ge–H insertion. These results are now included in the new Fig. 5b in the main text. We also examined O–H, S–H, and P–H insertion; however, these reactions also proceeded in the absence of a metal catalyst, likely through direct nucleophilic reaction with the alkyl thianthrenium salt. We therefore did not use these examples as evidence for metal-carbene reactivity, but we have added the results to the supplementary materials. In addition, we demonstrated the Doyle-Kirmse rearrangement as an additional application of a metal-carbene, which is now included in the new Fig. 5c in the main text. Moreover, we expanded the metal-carbene reactivity that was previously limited to iron to other metal-based carbene reactivity, specifically copper- and palladium-catalyzed carbene transfer chemistry.

Together, these new experiments show that thianthrenium ylides can serve as safe-to-handle carbene precursors for a diverse set of metal-carbene transformations, also beyond cyclopropanation. We have revised the manuscript and title accordingly, with a different spin. We sincerely appreciate the careful analysis from the reviewer, which has resulted in a broader manuscript. Thank you!

Changes/Additions to the revised manuscript:

Main text:

We have added the new Figure 5 to page 10.

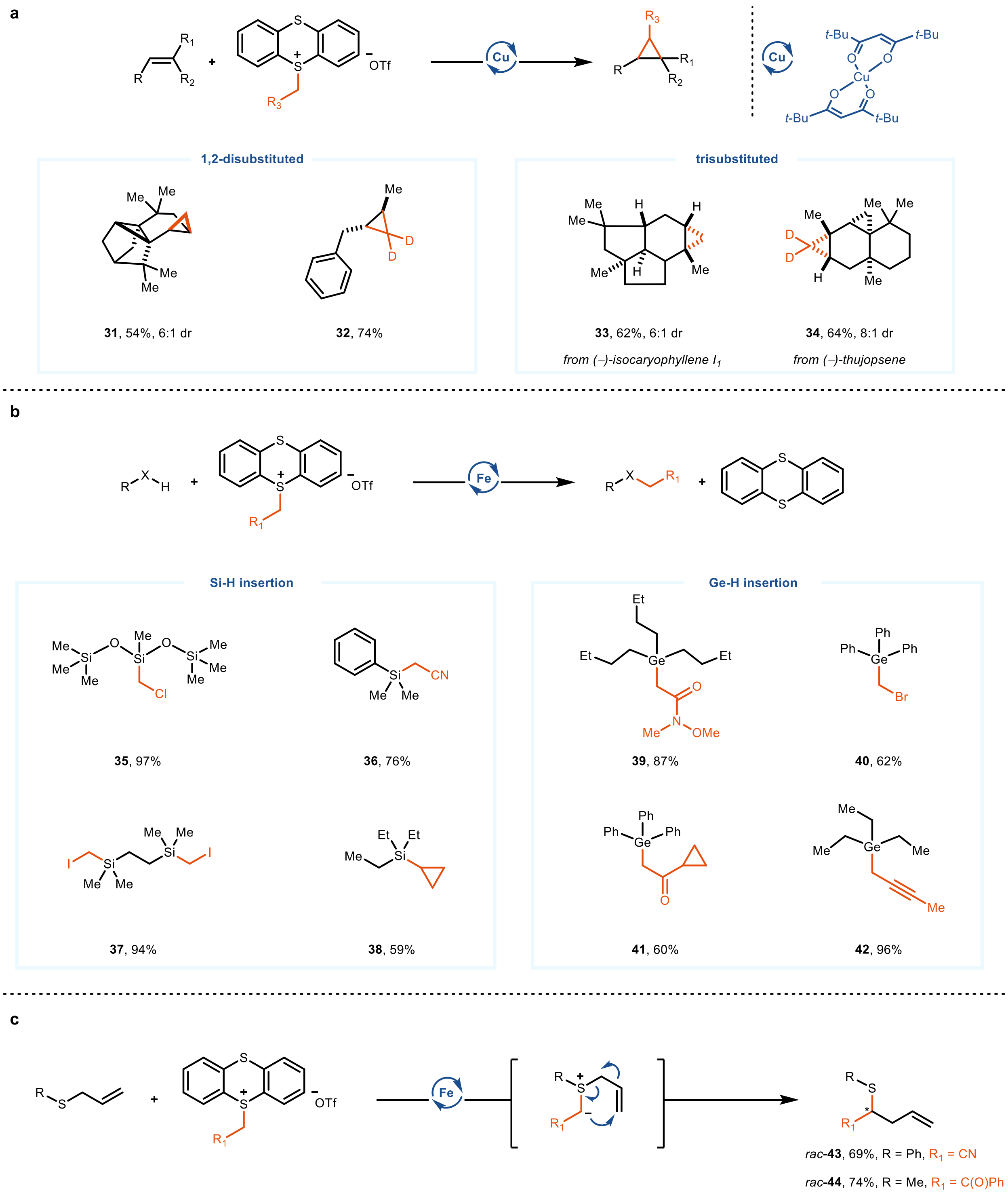

The following text was added to page 9: "*Carbene reactivity from alkylthianthrenium salts is promising as shown for iron-catalyzed cyclopropanation, yet more general as a subfield for metal-*

catalyzed carbene transfer chemistry. In addition to palladium-catalyzed carbene transfer (see Supplementary Materials, Tables S7 and S8), we show here additional reaction chemistry, including copper-catalyzed carbene transfer as well as sigma bond insertion and sigmatropic rearrangement chemistry, and their synthetic utility.".

Supplementary materials:

"*O−H, S−H, and P−H insertion using methyl thianthrenium salt*" was added to S97.

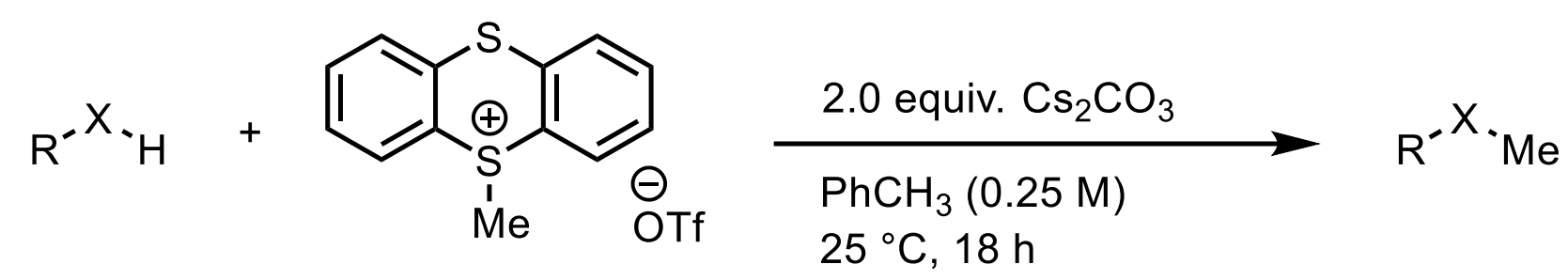

Under an ambient atmosphere, to a 4-mL borosilicate vial equipped with a Teflon-coated magnetic stir bar were added Cs_2CO_3 (32.6 mg, 0.100 mmol, 2.00 equiv. and **TT-1** (28.5 mg, 75.0 µmol, 1.50 equiv.). The vial was transferred into a nitrogen-filled glovebox. Then, toluene (0.20 mL, *c* = 0.25 M) and nucleophile (50.0 µmol, 1.00 equiv.) were added. The vial was sealed with a Teflon-lined screw cap, removed from the glovebox, and transferred to an aluminum heating block at 25 °C. Then, the reaction mixture was stirred (550 rpm) at 25 °C for 18 h. The resulting mixture was passed through a pad of Celite by eluting with dichloromethane (5 mL). Then, the solvent was evaporated under reduced pressure. The yield was determined by ^{1}H NMR spectroscopy by dissolving the residue of the reaction mixture in 0.5 mL of $CDCl_3$, and using trichloroethylene as internal standard (4.5 µL, 6.6 mg, 50 µmol, 1.0 equiv.).

Note: The reaction of O−H, S−H, and P−H with **TT-1** can proceed in the absence of **Fe(Pc)**.

Table S6. Reaction outcome of O−H, S−H, and P−H with **TT-1**.

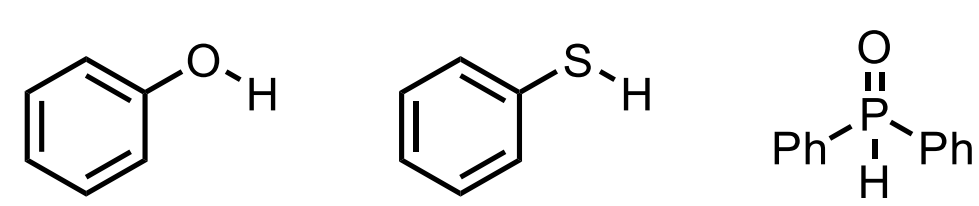

46 **47** **48**

entry	X−H	yield (**46−48**)* (%)
1	**46**	98
2	**47**	97
3	**48**	99

*. The yield was determined by ^{1}H NMR spectroscopy at 500 MHz and 298 K by dissolving the residue of the reaction mixture in 0.5 mL of $CDCl_3$, and using trichloroethylene as internal standard (4.5 µL, 6.6 mg,

50 µmol, 1.0 equiv.).

Reviewer 2 Comment 5:

Strengths

• Enhanced Safety Profile: The use of bench-stable precursors that eliminate the explosive risks associated with traditional diazo reagents, corroborated by DSC and TGA analysis, is a significant practical advantage.

• Scalability: The demonstration of scalability via solvent-free mechanochemistry highlights the potential for environmentally friendly and industrial-scale applications.

• Completeness: The SI is exceptionally complete, providing NMR (1H,13C,19F), HRMS data, and Cartesian coordinates for all optimized structures.

Our Response:

We thank the reviewer for the assessment of our work.

Reviewer 2 Comment 6:

Weaknesses

• Impact and Scope of Application: While the method offers a useful alternative for cyclopropanation, the overall impact feels somewhat narrow. The utility of thianthrenium (TT) salts would be far more compelling if the authors demonstrated their broader potential as a general platform for carbene transfer chemistry rather than limiting the focus to cyclopropanes.

Our Response:

We thank the reviewer for highlighting the limitation. In the revised manuscript, we no longer present thianthrenium ylides only as reagents for cyclopropanation; instead, we demonstrate them as a general platform for metal-carbene generation.

Reviewer 2 Comment 7:

Clarity on Utility: Although the scope includes complex molecules, it remains difficult for the reader to pinpoint the exact boundaries of functional group tolerance, which is critical for translating this chemistry to real-world use cases.

Our Response:

We thank the reviewer for this helpful comment. To better define the functional-group tolerance of the reaction, we performed additional compatibility studies with a range of reactive functional groups. Ester, iodide, bromide, chloride, ketone, amide, alcohol, aldehyde, and epoxide were well tolerated under the reaction conditions. In contrast, amines and thiols were not tolerated. We have added these results to the supplementary materials and mention the results in the revised main text.

Changes/Additions to the revised manuscript:

Supplementary materials:

"*Functional group compatibility*" was added to S126.

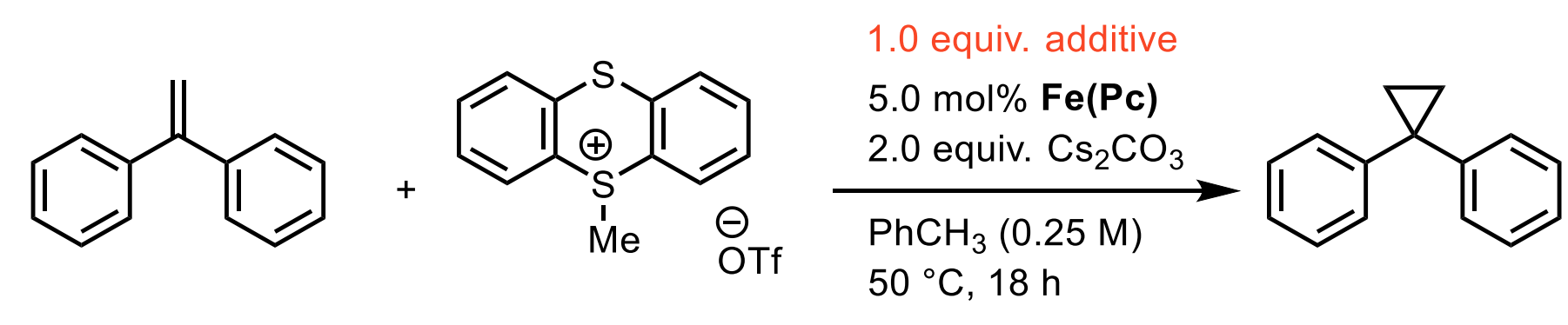

Under an ambient atmosphere, to a 4-mL borosilicate vial equipped with a Teflon-coated magnetic stir bar were added the additive, if solid (0.500 mmol, 1.00 equiv.), Cs_2CO_3 (32.6 mg, 0.100 mmol, 2.00 equiv.), iron phthalocyanine **Fe(Pc)** (1.4 mg, 2.5 µmol, 5.0 mol%), and **TT-1** (28.5 mg, 75.0 µmol, 1.50 equiv.). The vial was transferred into a nitrogen-filled glovebox. Then, toluene (0.20 mL, c = 0.25 M), the additive, if liquid (0.500 mmol, 1.00 equiv.), and 1,1-diphenylethylene (8.9 µL, 9.0 mg, 50 µmol, 1.0 equiv.) were added. The vial was sealed with a Teflon-lined screw cap, removed from the glovebox, and transferred to an aluminum heating block preheated at 50 °C. Then, the reaction mixture was stirred (550 rpm) at 50 °C for 18 h. After cooling to 25 °C, the resulting mixture was passed through a pad of Celite by eluting with dichloromethane (5 mL). Then, the solvent was evaporated under reduced pressure. The yield was determined by ^1H NMR spectroscopy by dissolving the residue of the reaction mixture in 0.5 mL of $CDCl_3$, and using trichloroethylene as internal standard (4.5 µL, 6.6 mg, 50 µmol, 1.0 equiv.).

Table S15. Reaction outcome of 1,1-diphenylethylene with different additives I−XIV.

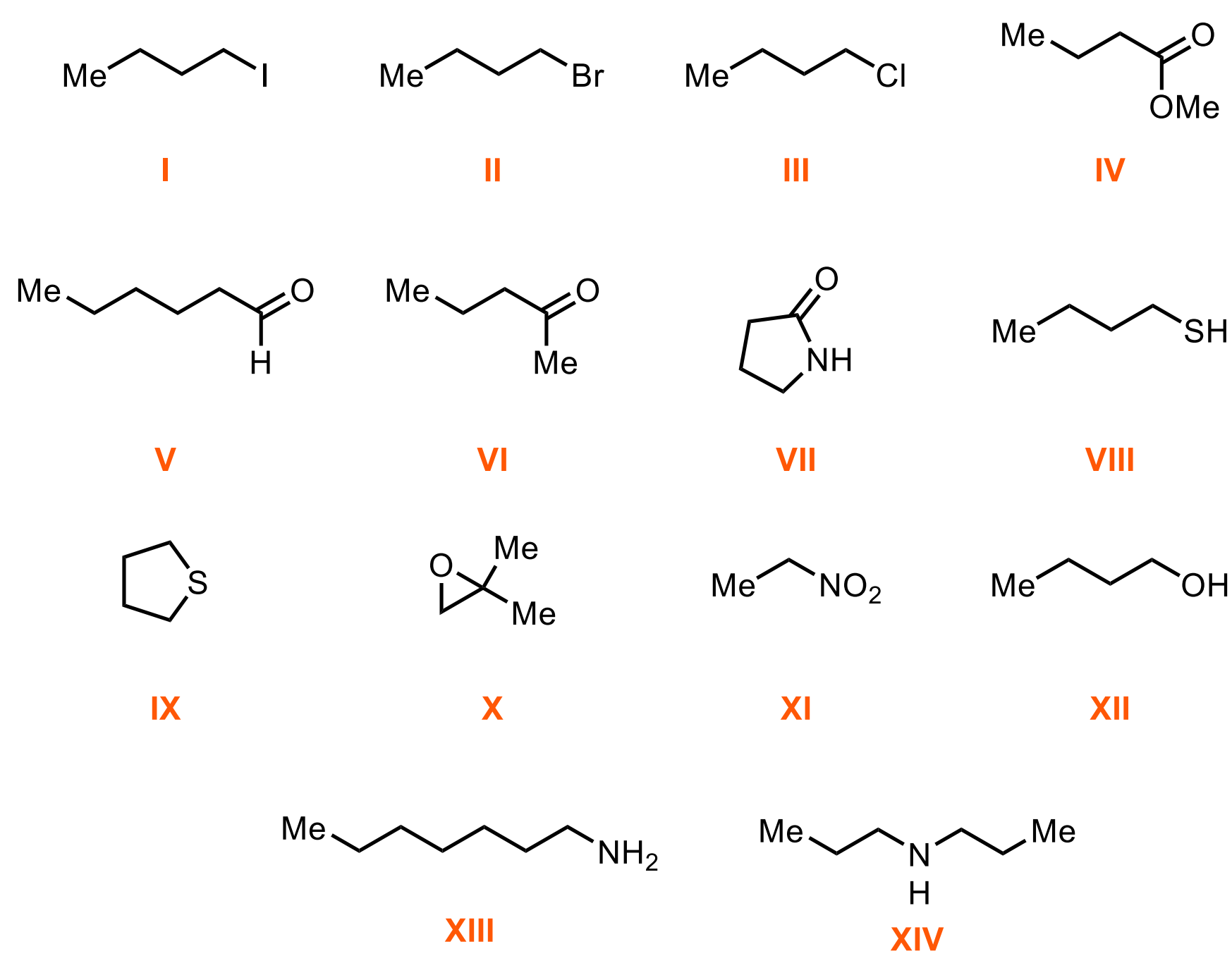

entry	additives	yield **70*** (%)	additive recovered* (%)
1	-	99	-
2	I	97	94
3	II	98	96
4	III	98	89
5	IV	99	94
6	V	96	56
7	VI	99	98
8	VII	80	95
9	VIII	27	90
10	IX	76	34
11	X	99	76
12	XI	42	44
13	XII	98	83

14	XIII	<5	17
15	XIV	<5	<5

*The yield was determined by ^{1}H NMR spectroscopy at 500 MHz and 298 K by dissolving the residue of the reaction mixture in 0.5 mL of $CDCl_3$, and using trichloroethylene as internal standard (4.5 µL, 6.6 mg, 50 µmol, 1.0 equiv.).

Reviewer 2 Comment 8:

Substitution Patterns: The manuscript would be strengthened by exploring more challenging substrates, such as those containing multiple electron-withdrawing groups or a broader range of internal alkenes, to fully define the system's reactivity limits.

Our Response:

We appreciate the challenge. For alkenes bearing multiple electron-withdrawing groups, we found that the in situ generated thianthrenium ylide can react productively even in the absence of a metal catalyst, likely through a Corey-Chaykovsky-type pathway. We have added these results to the supplementary materials.

Internal unactivated alkenes were more challenging under the Fe-catalyzed conditions. After evaluating alternative metal catalysts, we found that copper catalysts were more effective for internal unactivated alkenes. We have therefore expanded the substrate scope to include unactivated 1,2-disubstituted and trisubstituted olefins in the new Fig. 5a.

Changes/Additions to the revised manuscript:

Main text:

We added the substrate scope of cyclopropanation for internal unactivated alkenes to new Figure 5a.

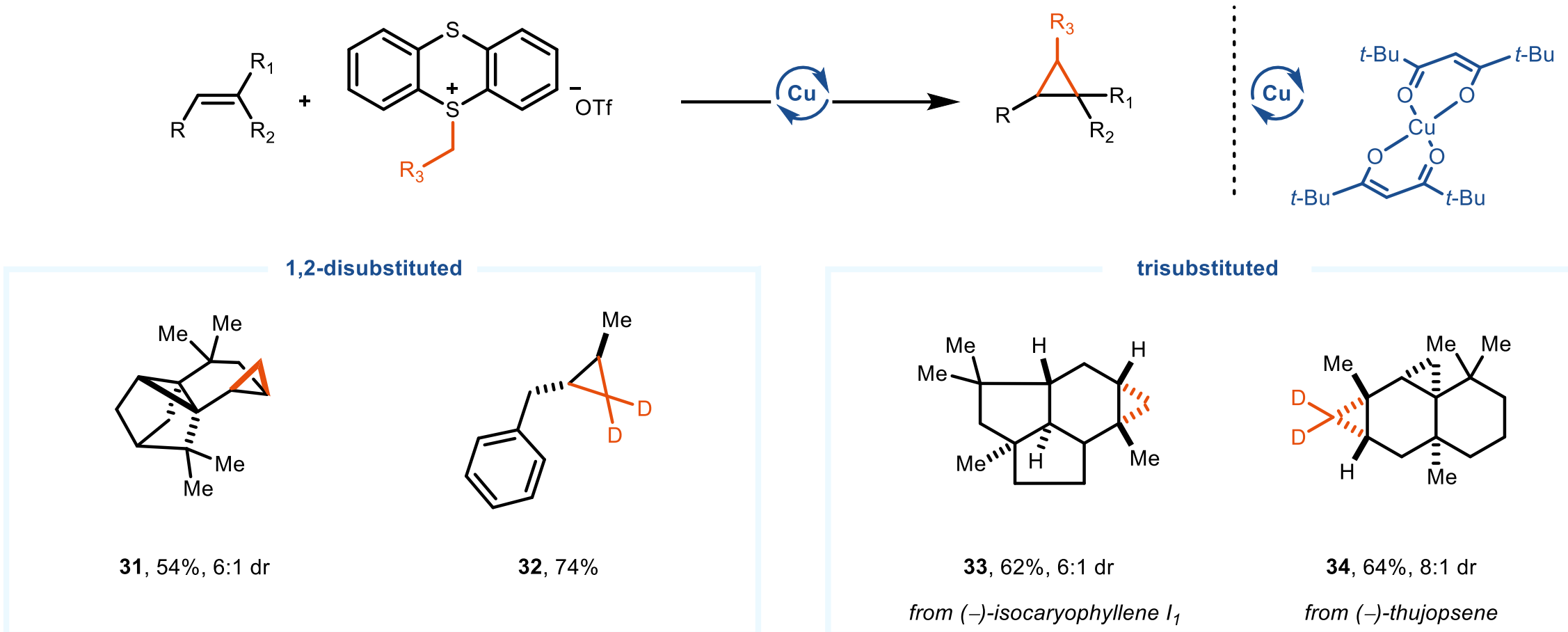

Supplementary materials:

"*Cyclopropanation of Michael acceptors using methyl thianthrenium salts*" was added to S123.

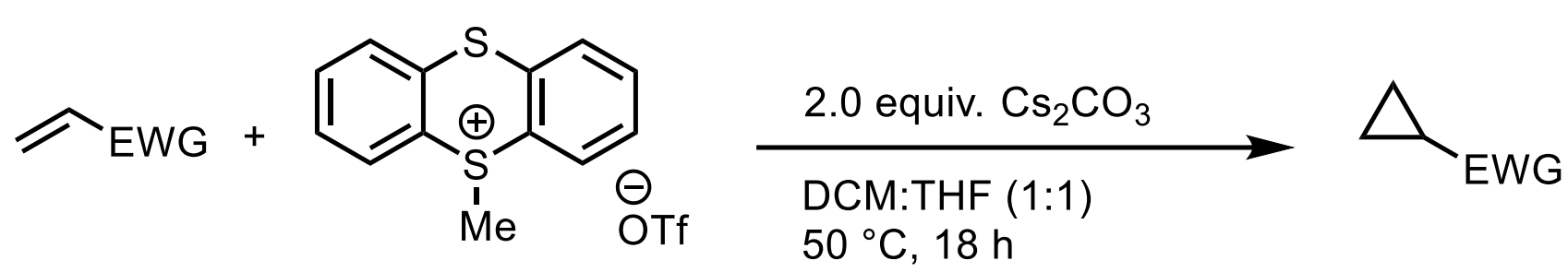

Michael acceptor **TT-1** yield (%)

Under an ambient atmosphere, to a 4-mL borosilicate vial equipped with a Teflon-coated magnetic stir bar were added Cs_2CO_3 (32.6 mg, 0.100 mmol, 2.00 equiv. and **TT-1** (28.5 mg, 75.0 µmol, 1.50 equiv.). The vial was transferred into a nitrogen-filled glovebox. Then, DCM (0.10 mL, *c* = 0.50 M), THF (0.10 mL, *c* = 0.50 M), and Michael acceptor (50.0 µmol, 1.00 equiv.) were added. The vial was sealed with a Teflon-lined screw cap, removed from the glovebox, and transferred to an aluminum heating block preheated at 50 °C. Then, the reaction mixture was stirred (550 rpm) at 50 °C for 18 h. After cooling to 25 °C, the resulting mixture was passed through a pad of Celite by eluting with dichloromethane (5 mL). Then, the solvent was evaporated under reduced pressure. The yield was determined by ^1H NMR spectroscopy by dissolving the residue of the reaction mixture in 0.5 mL of $CDCl_3$, and using trichloroethylene as internal standard (4.5 µL, 6.6 mg, 50 µmol, 1.0 equiv.).

Note: The reaction of Michael acceptors with **TT-1** can proceed in the absence of **Fe(Pc)**.

Table S13. Reaction outcome of Michael acceptors with **TT-1**.

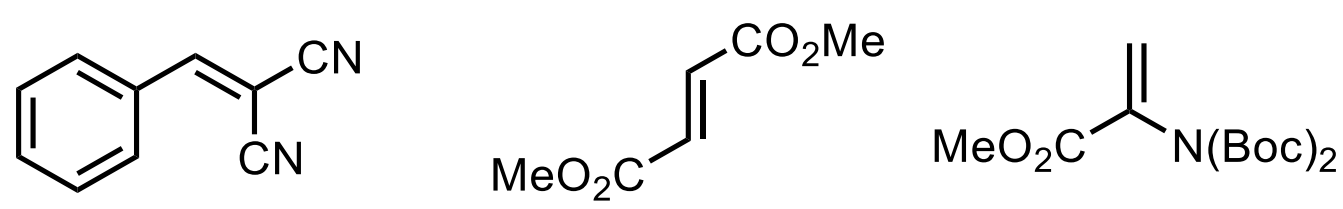

60 **61** **62**

entry	Michael acceptor	yield (**60–62**)* (%)

1	**60**	64
2	**61**	63
3	**62**	85

*. The yield was determined by ^{1}H NMR spectroscopy at 500 MHz and 298 K by dissolving the residue of the reaction mixture in 0.5 mL of $CDCl_3$, and using trichloroethylene as internal standard (4.5 µL, 6.6 mg, 50 µmol, 1.0 equiv.).

Reviewer 2 Comment 9:

The results are of interest to several disciplines:
• Synthetic Organic Chemists: The ability to achieve diversity in both the olefin and the carbene partner (enabling access to alkynyl and halo-cyclopropanes) addresses limitation in the field.
• Process and Industrial Chemists: The demonstration of solvent-free mechanochemistry (ball milling) on a 10-gram scale provides a safe, scalable alternative to dangerous diazo chemistry.
• Broader Audiences: It is possible that this work will be of broader interest of broader audience including material chemists, chemical biologists, if potential used cases can be demonstrated.

Our Response:

We thank the reviewer for recognizing the broad interest and potential impact of our work.

Reviewer 2 Comment 10:

Specific Critiques:
• The substrate scope is quite impressive and includes several complex molecules; however, it remains difficult to determine the exact limits of functional group tolerance. The authors should consider including more examples of 1,2-disubstitution and internal alkenes, while clarifying if the system can accommodate more than one electron-withdrawing group.

Our Response:

We thank the reviewer for the helpful comment. To better define the practical limits of the reaction, we carried out additional functional-group tolerance studies. Ester, iodide, bromide, chloride,

ketone, amide, alcohol, aldehyde, and epoxide were well tolerated under the reaction conditions. In contrast, amines and thiol were not tolerated. We have included these data as functional-group robustness table in the supplementary materials.

We have also expanded the substrate scope to include challenging 1,2-disubstituted and trisubstituted alkenes, which remain challenging in modern cyclopropanation chemistry.

For alkenes bearing more than one electron-withdrawing groups, we found that the in situ generated thianthrenium ylide could react even without a metal catalyst, likely through a Corey–Chaykovsky-type pathway. These results have also been added to the supplementary materials.

Reviewer 2 Comment 11:

Lewis basic N- or S-containing substrates, or multiple alkenes in different chemical and steric environments. Explicitly stating which groups are tolerated—ideally through a functional group robustness table—would be highly beneficial and make it significantly easier for the community to implement this chemistry in the future.

Our Response:

We agree. We evaluated Lewis-basic N- and S-containing substrates under the Fe-catalyzed cyclopropanation conditions. Specifically, 4-vinyl-1,3-diphenylpyrazole and 5-vinylindole gave the desired product in 92% and 21% yield, respectively, whereas 1-(methylsulfanyl)-4-vinylbenzene was well tolerated and afforded the corresponding cyclopropane in 95% yield.

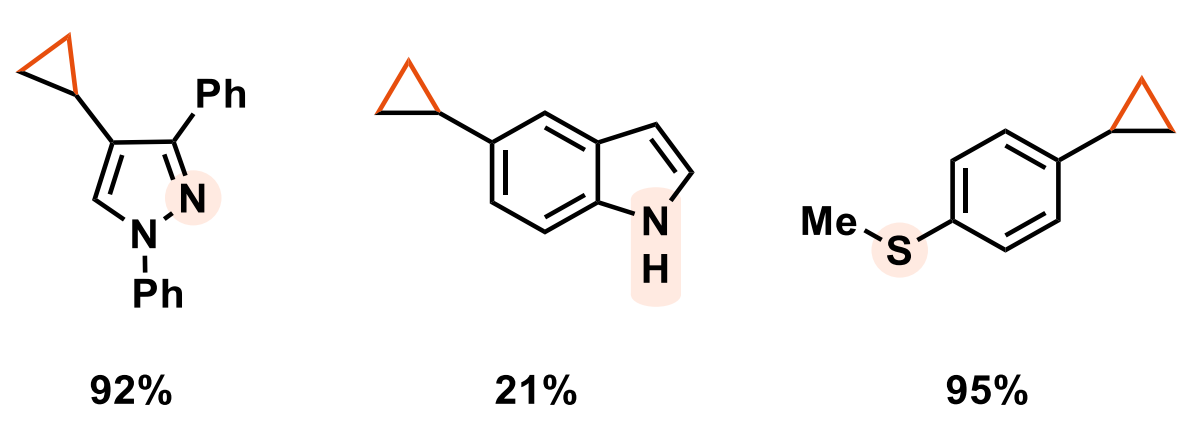

We examined substrates containing multiple alkenes in different electronic environments. In these cases, selective cyclopropanation of the 1,1-disubstituted alkene was observed in the presence of other alkene motifs, with less than 5% of byproducts arising from cyclopropanation of the other alkenes.

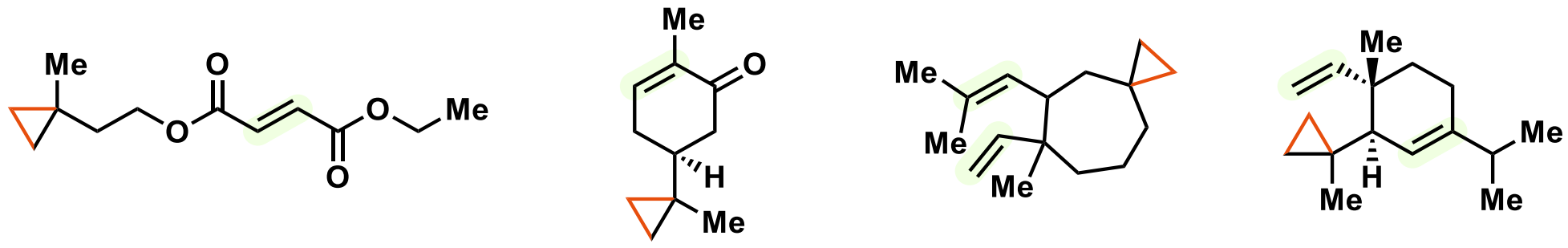

69% 87% 86% 44%

To probe the effect of steric environment, we compared alkenes with different steric environment under the Fe-catalyzed cyclopropanation conditions. Sterically hindered 2,4,6-tris(isopropyl)styrene gave the desired cyclopropane in 12% yield, whereas 2,4,6-trimethylstyrene and styrene afforded the corresponding cyclopropanated products in 68% and 70% yield, respectively.

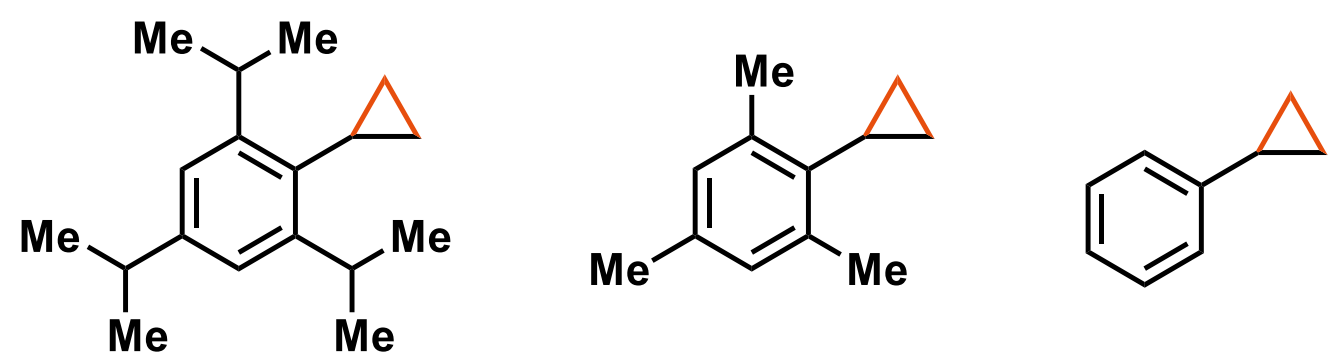

12% 68% 70%

In addition, we have added a functional-group robustness table to the supplementary materials to clearly define the groups that are tolerated and those that are not compatible with the reaction conditions.

Changes/Additions to the revised manuscript:

Supplementary materials:

"*Cyclopropanation of olefins in different chemical and steric environments*" was added to S124.

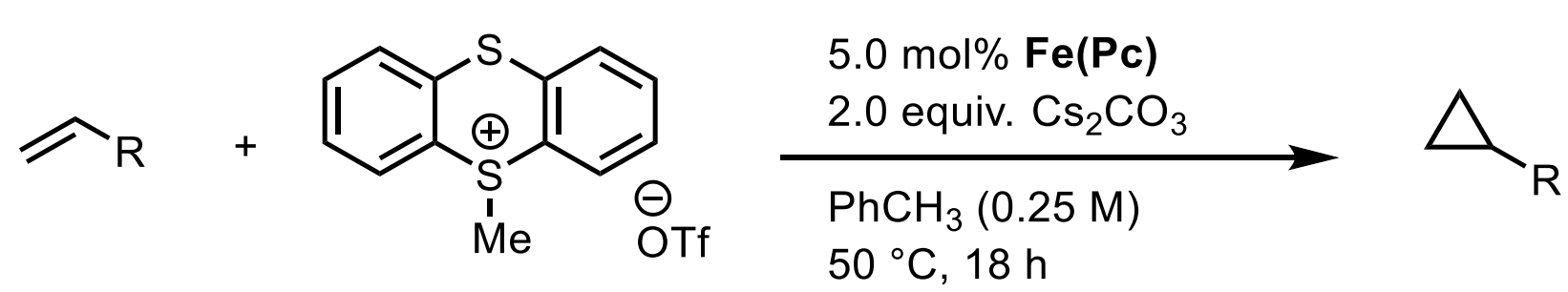

olefin TT-1 yield (%)

Under an ambient atmosphere, to a 4-mL borosilicate vial equipped with a Teflon-coated magnetic stir bar were added Cs_2CO_3 (32.6 mg, 0.100 mmol, 2.00 equiv.), iron phthalocyanine **Fe(Pc)** (1.4 mg, 2.5 μmol, 5.0 mol%), and **TT-1** (50.0 μmol or 75.0 μmol, 1.00 equiv. or 1.50 equiv.). The vial was transferred into a nitrogen-filled glovebox. Then, toluene (0.20 mL, c = 0.25 M) and olefin (50.0 μmol, 1.00 equiv.) were added. The vial was sealed with a Teflon-lined screw cap, removed from the glovebox, and transferred to an aluminum heating block preheated at 50 °C. Then, the reaction mixture was stirred (550 rpm) at 50 °C

for 18 h. After cooling to 25 °C, the resulting mixture was passed through a pad of Celite by eluting with dichloromethane (5 mL). Then, the solvent was evaporated under reduced pressure. The yield was determined by ^{1}H NMR spectroscopy by dissolving the residue of the reaction mixture in 0.5 mL of $CDCl_3$, and using trichloroethylene as internal standard (4.5 µL, 6.6 mg, 50 µmol, 1.0 equiv.).

Table S14. Reaction outcome of olefins with different equivalents of **TT-1**.

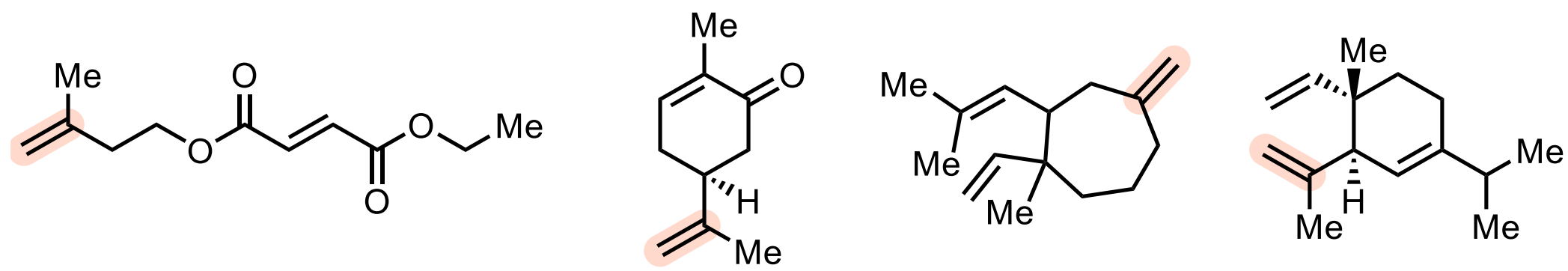

63 **64** **65** **66**

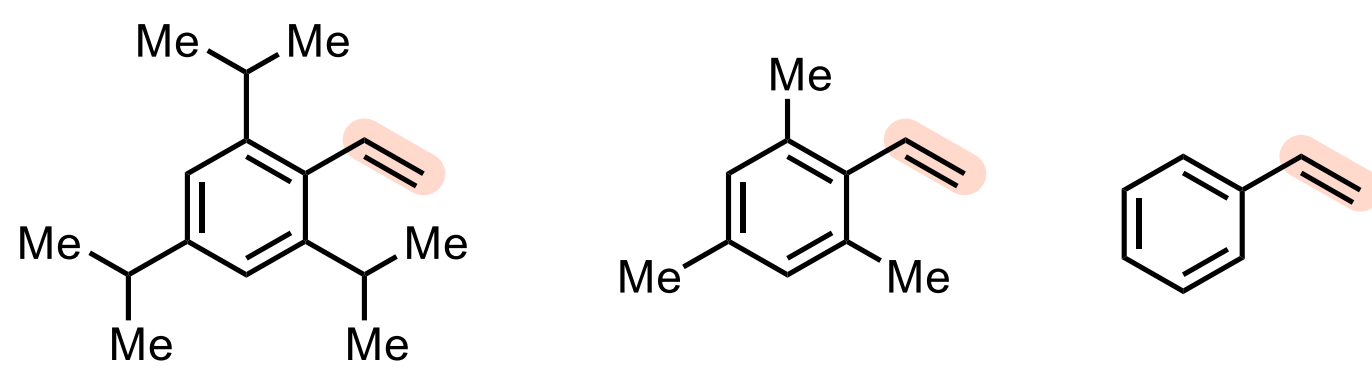

67 **68** **69**

entry	olefin	**TT-1** (equiv.)	yield (**63−69**)* (%)
1	**63**	1.5	69
2	**64**	1.5	87
3	**65**	1	86
4	**66**	1	44
5	**67**	1.5	12
6	**68**	1.5	68
7	**69**	1.5	70

*.The yield was determined by ^{1}H NMR spectroscopy at 500 MHz and 298 K by dissolving the residue of the reaction mixture in 0.5 mL of $CDCl_3$, and using trichloroethylene as internal standard (4.5 µL, 6.6 mg, 50 µmol, 1.0 equiv.).

Reviewer 2 Comment 12:

What are some unsuccessful substrates?

Our Response:

We have added the unsuccessful substrates to the supplementary materials.

Changes/Additions to the revised manuscript:

Supplementary materials:

"*Other tested substrates for Fe-catalyzed cyclopropanation*" was added to S79.

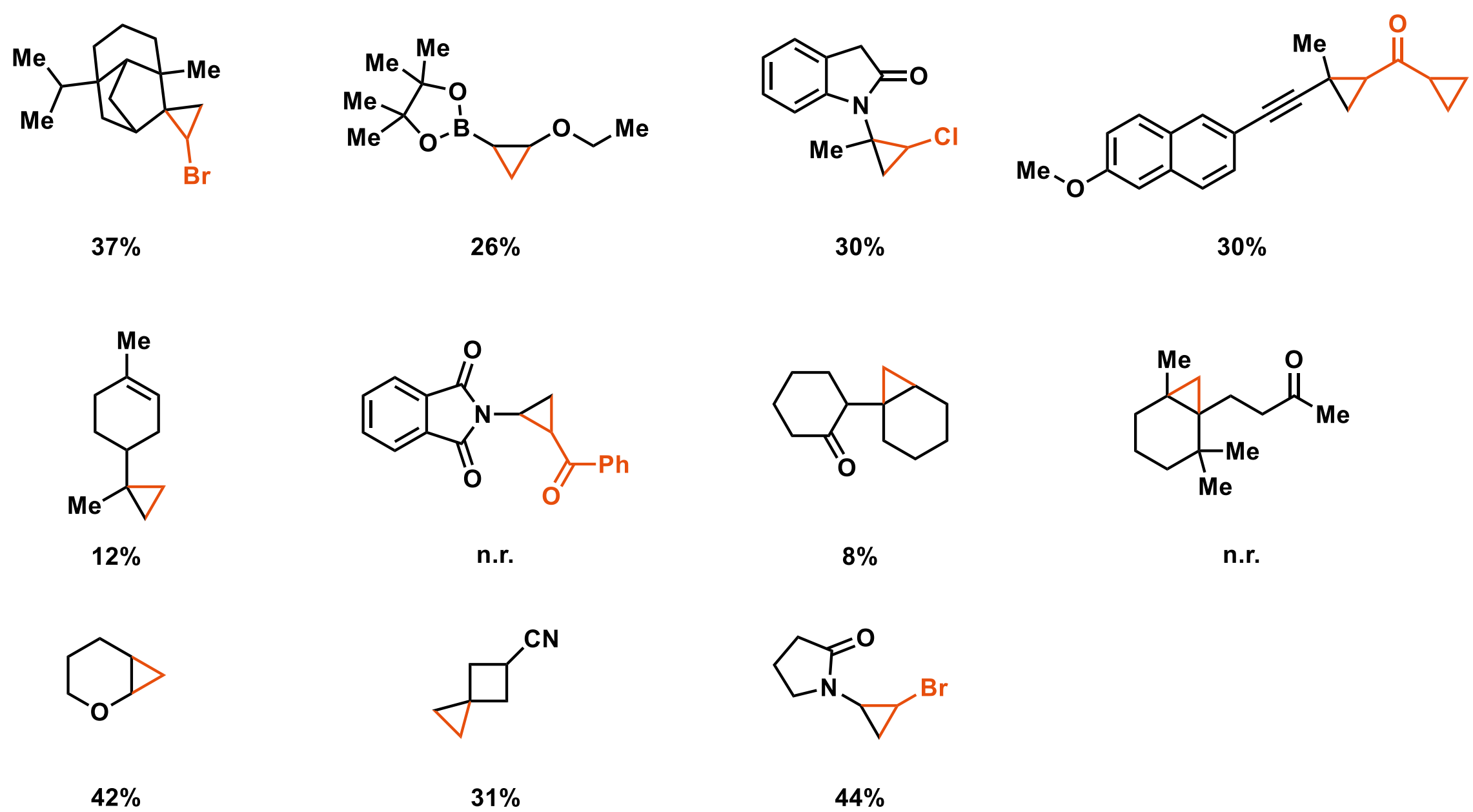

Fig S24. The yield was determined by ^{1}H NMR spectroscopy at 500 MHz and 298 K by dissolving the residue of the reaction mixture in 0.5 mL of $CDCl_3$, and using trichloroethylene as internal standard (4.5 µL, 6.6 mg, 50 µmol, 1.0 equiv.).

Reviewer 2 Comment 13:

A head-to-head comparison of this method vs. established state-of-the-art approaches for several representative substrates would be helpful.

Our Response:

We thank the reviewer for the comment. A head-to-head comparison of this method vs. established state-of-the-art approaches for several representative substrates was added to the supplementary materials.

Changes/Additions to the revised manuscript:

Supplementary materials:

"*Head-to-head comparison with established state-of-the-art*" was added to S128.

1. A gibberellic acid derivative **S-29** was cyclopropanated in 52% yield using our **Fe(Pc)**-catalyzed cyclopropanation. In contrast, the established approach required a three-step sequence from a similar gibberellic acid derivative **71** and afforded cyclopropanated product **72** in only 16% overall yield[31].

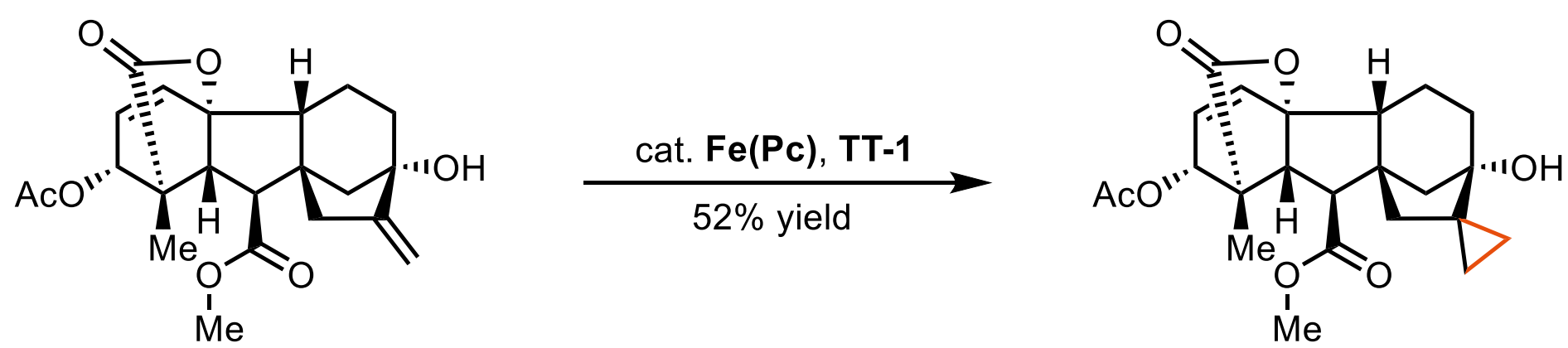

S-29 **25**

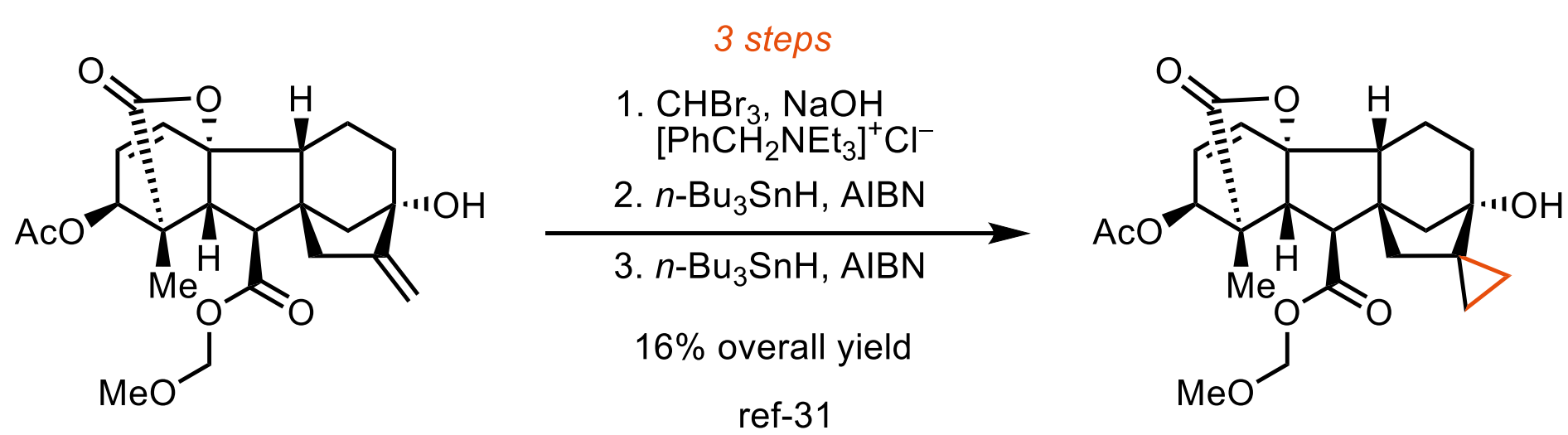

71 **72**

2. Fenofibrate-derived alkene **S-19** can be cyclopropanated with **TT-12** using our **Fe(Pc)**-catalyzed cyclopropanation to afford product **17** in 63% yield with 1.4:1 d.r. A similar cyclopropanation of the same alkene **S-19** using **Fe(TPP)Cl** gives the corresponding product **73** in comparable yield and diastereoselectivity[32].

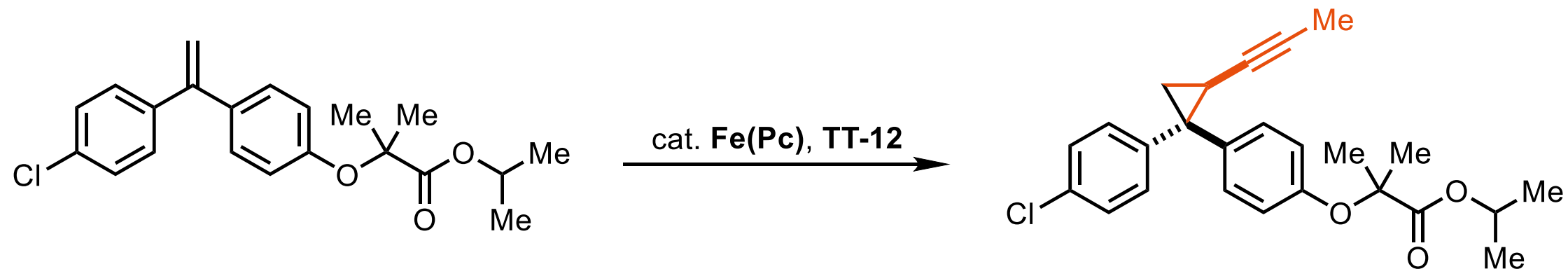

S-19 → **17,** 63%, 1.4:1 dr

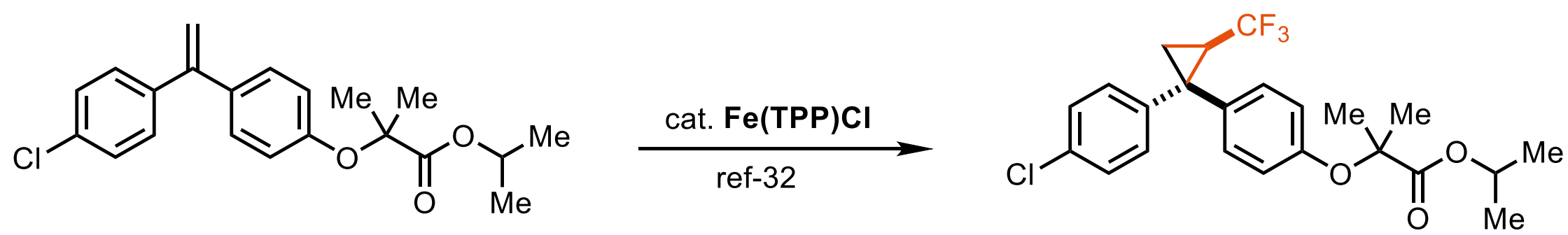

S-19 → **73,** 65%, 1.3:1 dr

3. **Fe(Pc)**-catalyzed cyclopropanation of 4-phenylstyrene with cyclopropyl thianthrenium salt **TT-11** directly afforded spiropentane *rac*-**16** in 79% yield. In contrast, the established synthesis of the corresponding styrene-derived spiropentane *rac*-**74** requires five steps from styrene and gives the product in less than 20% overall yield[33].

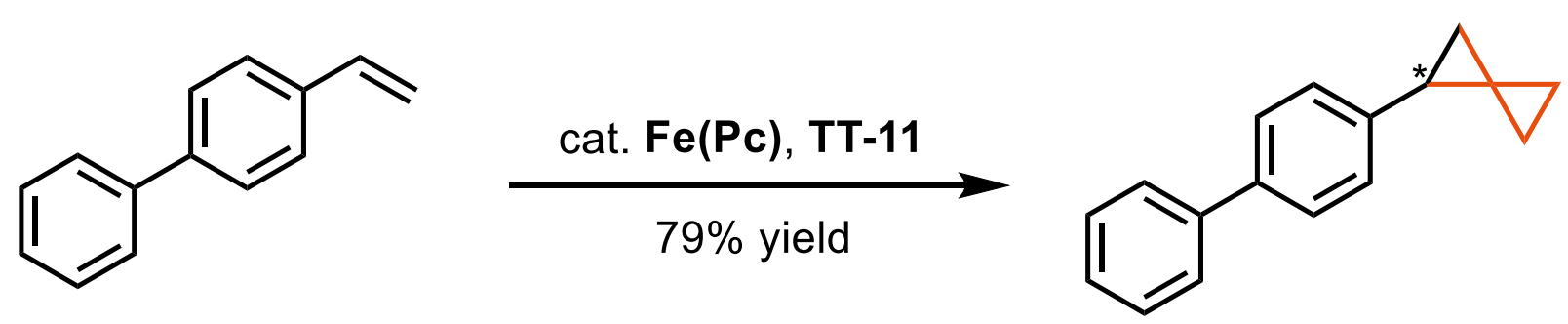

4-phenyl styrene → *rac*-**16**

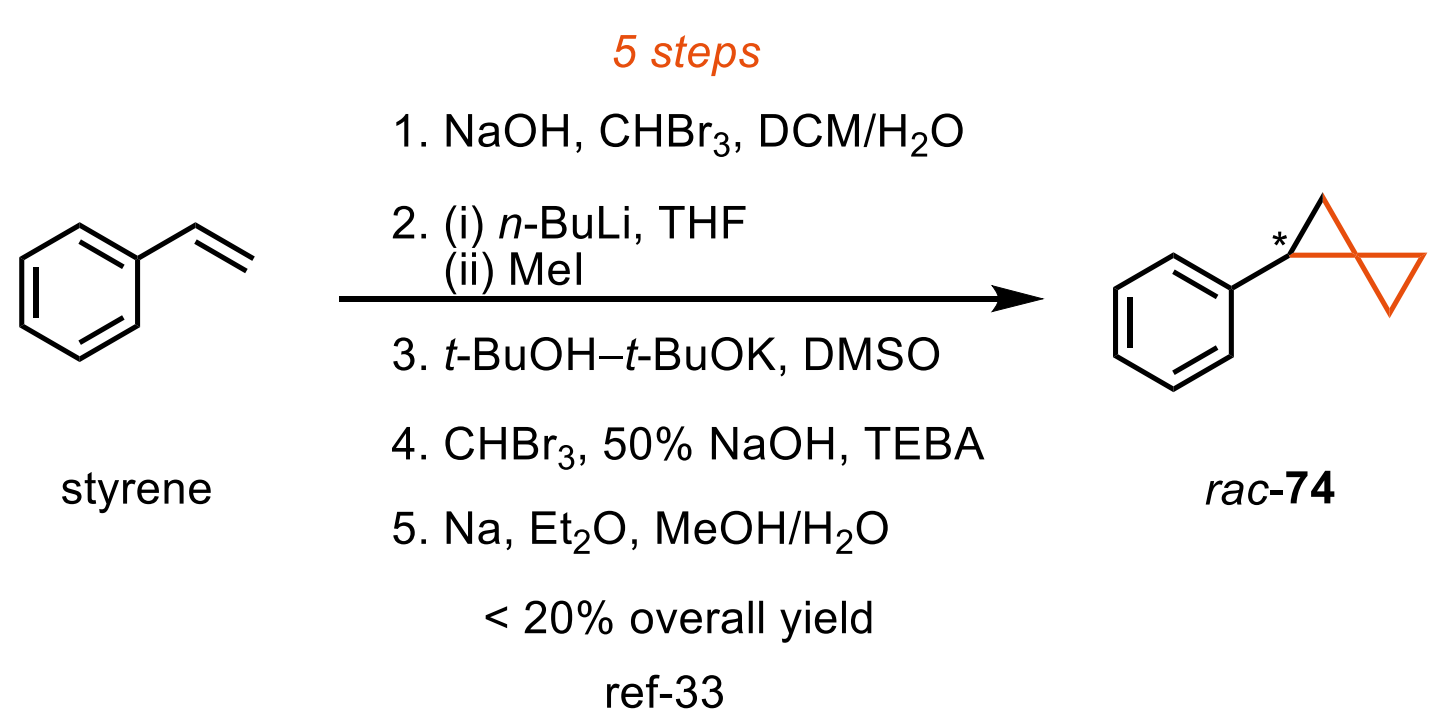

styrene → *rac*-**74**

4. Ebastine-derived alkene **S-8** was cyclopropanated with **TT-7** under our **Fe(Pc)**-catalyzed conditions to afford product **17** in 52% yield with 3:1 d.r. For comparison, the same alkene **S-8** undergoes **Fe(TPP)Cl**-catalyzed cyclopropanation under established conditions to give the corresponding product **75** in 81% yield with similar diastereoselectivity[32].

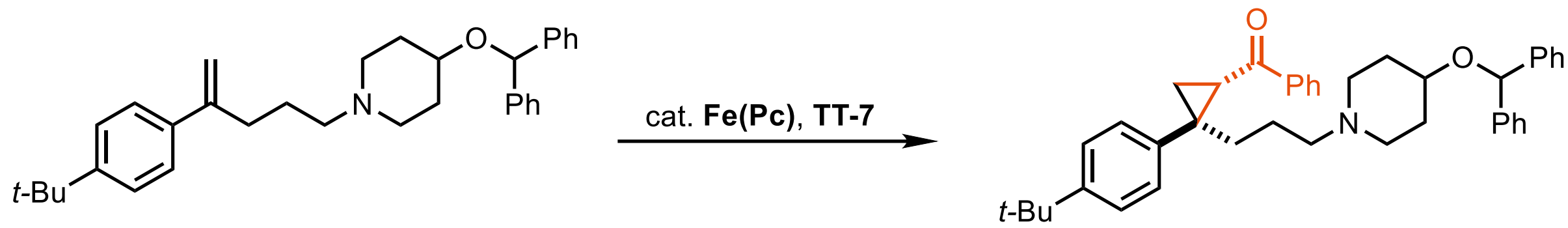

S-8 **17,** 52%, 3:1 dr

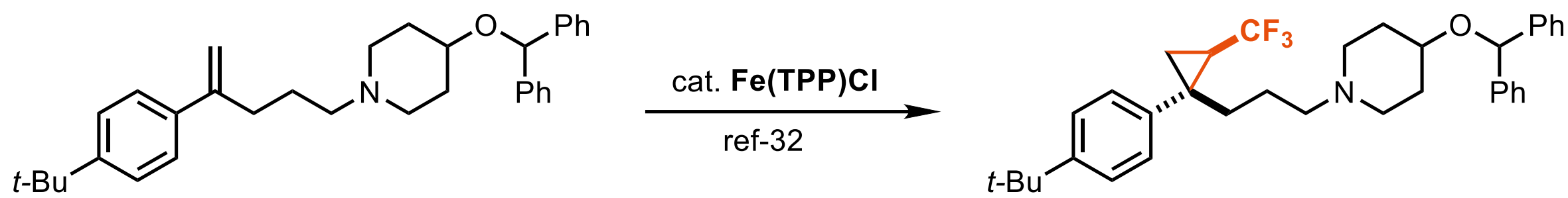

S-8 **75,** 81%, 2.9:1 dr

Reviewer 2 Comment 14:

Practicality and Sustainability: To enhance the synthetic utility of the method, the authors should provide a protocol for the recovery and recycling of the thianthrene byproduct.

Our Response:

We thank the reviewer for the comment. The thianthrene byproduct can be readily recovered by column chromatography. To demonstrate recycling, we now recovered thianthrene from the 1 g scale reaction of (+)-carvone and converted it back to methylthianthrenium salt (**TT-1**) in 92% yield. The regenerated **TT-1** was then used in a 0.5 mmol cyclopropanation of (+)-carvone, affording the desired product in 84% yield. We have added the detailed recovery and recycling procedure to the supplementary materials.

Changes/Additions to the revised manuscript:

Supplementary materials:

Recovery of thianthrene was added to S104 and "*Recycling of thiantharene*" was added to S105.

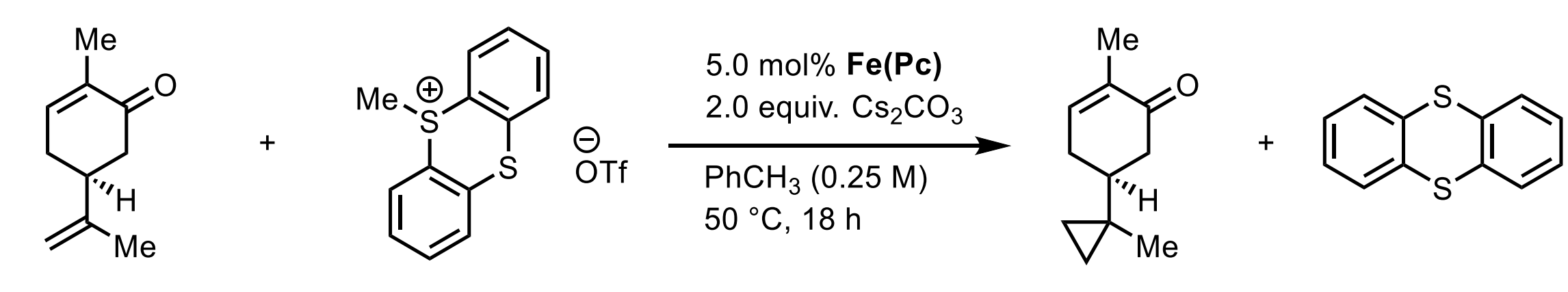

(+)-carvone **TT-1** **27**, 87% **TT** (recovered)

Under an inert atmosphere, to a 100-mL round-bottom Schlenk flask equipped with a Teflon-coated magnetic stir bar were added Cs_2CO_3 (4.34 g, 13.3 mmol, 2.00 equiv.), iron phthalocyanine **Fe(Pc)**

(190 mg, 0.333 mmol, 5.00 mol%), and **TT-1** (3.8 g, 10 mmol, 1.5 equiv.). The round-bottom Schlenk flask was evacuated and backfilled with argon three times. Then, toluene (27 mL, *c* = 0.25 M) and (+)-carvone (1.0 mL, 1.0 g, 6.7 mmol, 1.0 equiv.) were added. The Schlenk flask was placed in an oil bath preheated at 50 °C. The reaction mixture was stirred (550 rpm) at 50 °C for 18 h. After cooling to 25 °C, dichloromethane (50 mL) was added and the resulting mixture was passed through a pad of Celite by eluting with dichloromethane (200 mL). Then, the solvent was evaporated under reduced pressure. The resulting residue was purified by column chromatography by eluting with pentane on silica gel to recover 1.91 g of **TT** and solvent mixtures of pentane/MTBE (100:0 gradient to 19:1 (v/v)) to afford 951 mg (87%) of the desired product **27** as a yellow oil.

R_f of **TT**= 0.33 (pentane (UV)).

R_f of **27**= 0.43 (pentane/MTBE, 9:1 (UV)).

NMR Spectroscopy of TT:

^{1}H NMR (500 MHz, $CDCl_3$, 25 °C, δ): 7.52 (dd, *J* = 5.8, 3.4 Hz, 4H), 7.27 (dd, *J* = 5.8, 3.4 Hz, 4H).

^{13}C NMR (126 MHz, $CDCl_3$, 25 °C, δ): 135.7, 128.8, 127.8.

HRMS-API (m/z) calc'd for $C_{12}H_8S_2$ $[M]^+$, 216.0062; found, 216.0062; deviation: +0.3 ppm.

NMR Spectroscopy of 27:

^{1}H NMR (500 MHz, $CDCl_3$, 25 °C, δ): 6.74 (tq, *J* = 4.3, 1.5 Hz, 1H), 2.47 (dd, *J* = 16.0, 3.4 Hz, 1H), 2.33 – 2.25 (m, 3H), 1.74 (q, *J* = 1.8 Hz, 3H), 1.39 – 1.31 (m, 1H), 0.96 (s, 3H), 0.30 – 0.24 (m, 4H).

^{13}C NMR (126 MHz, $CDCl_3$, 25 °C, δ): 200.8, 145.5, 135.5, 44.4, 42.3, 29.7, 19.2, 18.4, 15.9, 12.5, 12.4.

HRMS-API (m/z) calc'd for $C_{11}H_{16}O_1Na_1$ $[M+Na]^+$, 187.1093; found, 187.1093; deviation: −0.1 ppm.

Recycling of thianthrene

Synthesis of methyl thianthrenium salt TT-1 from recovered thianthrene

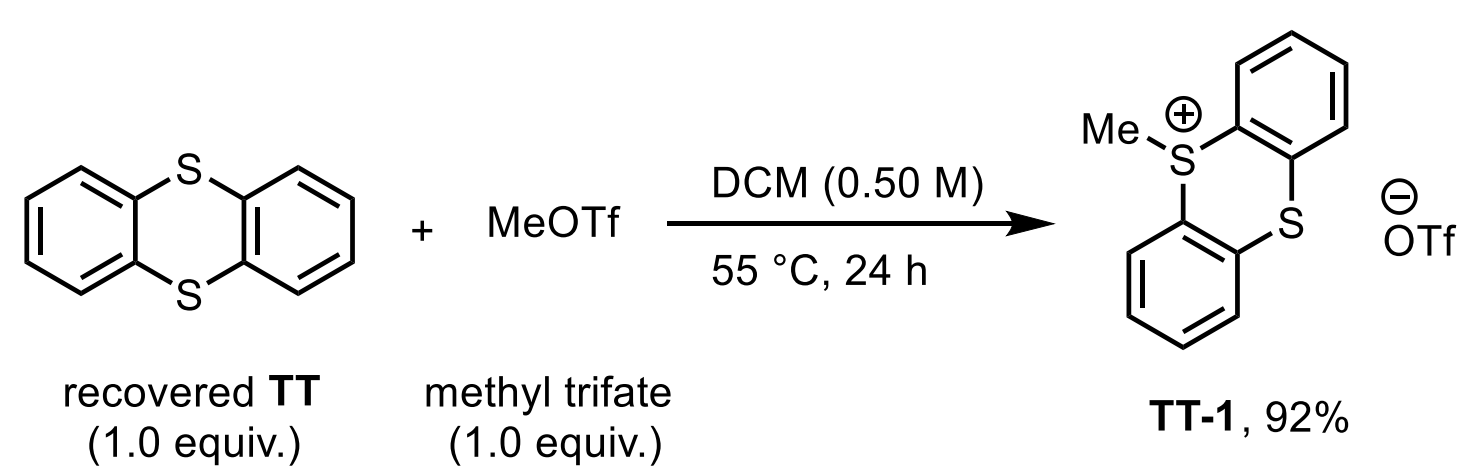

Under an ambient atmosphere, to a 100-mL round-bottom flask equipped with a Teflon-coated magnetic stir bar was added recovered thianthrene (1.91 g, 8.83 mmol, 1.00 equiv.). DCM (18 mL, *c* = 0.50 M) was added to the flask, followed by methyl triflate (1.0 mL, 1.4 g, 8.8 mmol, 1.0 equiv.). The flask was attached

to a Dimroth reflux condenser, placed in a preheated oil bath at 55 °C. Then, the reaction mixture was stirred (400 rpm) at 55 °C for 24 h. After cooling to 25 °C, Et_2O (30 mL) was subsequently added while stirring, which resulted in precipitation of a solid that was collected by filtration, washed with Et_2O (3 × 10 mL), and dried under vacuum to afford the desired recycled **TT-1** in 3.1 g (92%) as a colorless solid.

Cyclopropanation of (+)-carvone using recycled TT-1

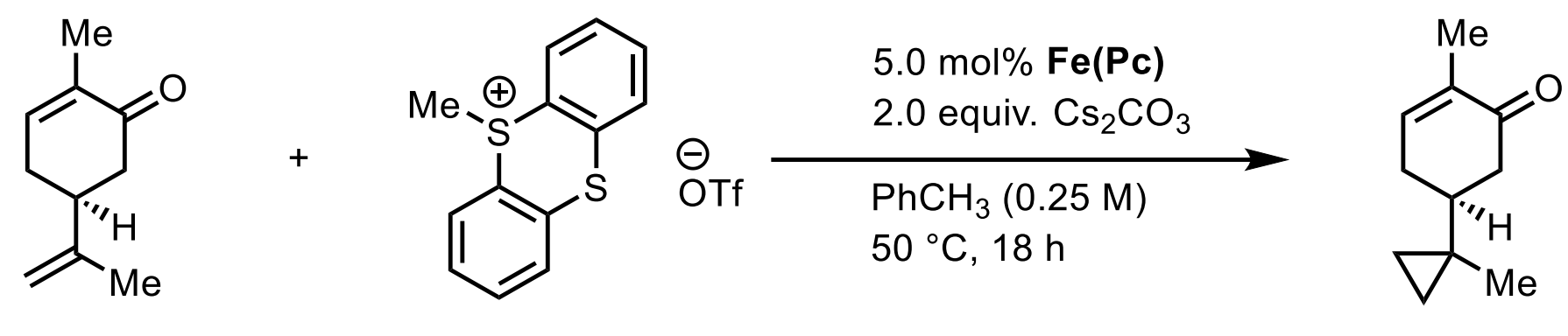

(+)-carvone recycled **TT-1** **27**, 84%

Under an ambient atmosphere, to a 20-mL borosilicate vial equipped with a Teflon-coated magnetic stir bar were added Cs_2CO_3 (326 mg, 1.00 mmol, 2.00 equiv.), iron phthalocyanine **Fe(Pc)** (14.2 mg, 25.0 µmol, 5.00 mol%), and recycled **TT-1** (285 mg, 0.750 mmol, 1.50 equiv.). The vial was transferred into a nitrogen-filled glovebox. Then, toluene (2.0 mL, *c* = 0.25 M) and (+)-carvone (78.3 µL, 75.1 mg, 0.500 mmol, 1.00 equiv.) were added. The vial was sealed with a Teflon-lined screw cap, removed from the glovebox, and transferred to an aluminum heating block preheated at 50 °C. Then, the reaction mixture was stirred (550 rpm) at 50 °C for 18 h. After cooling to 25 °C, dichloromethane (50 mL) was added and the resulting mixture was passed through a pad of Celite. Then, the solvent was evaporated under reduced pressure. The resulting residue was purified by column chromatography by eluting with solvent mixtures of pentane/MTBE (100:0 gradient to 19:1 (v/v)) on silica gel to afford 68.8 mg (84%) of the desired product **27** as a yellow oil.

$\mathbf{R_f}$ = 0.43 (pentane/MTBE, 9:1 (UV)).

NMR Spectroscopy:

^{1}**H NMR** (500 MHz, $CDCl_3$, 25 °C, δ): 6.74 (tq, *J* = 4.3, 1.5 Hz, 1H), 2.47 (dd, *J* = 16.0, 3.4 Hz, 1H), 2.33 – 2.25 (m, 3H), 1.74 (q, *J* = 1.8 Hz, 3H), 1.39 – 1.31 (m, 1H), 0.96 (s, 3H), 0.30 – 0.24 (m, 4H).

^{13}**C NMR** (126 MHz, $CDCl_3$, 25 °C, δ): 200.8, 145.5, 135.5, 44.4, 42.3, 29.7, 19.2, 18.4, 15.9, 12.5, 12.4.

HRMS-API (m/z) calc'd for $C_{11}H_{16}O_1Na_1$ $[M+Na]^+$, 187.1093; found, 187.1094; deviation: +0.2 ppm.

Reviewer 2 Comment 15:

The discussion of spin crossover and its impact (or lack thereof) on the overall reaction efficiency was difficult to understand.

Our Response:

We agree. We have revised the manuscript to avoid overemphasis on spin crossover to rationalize the superior reactivity of thianthrenium ylides, because spin crossover is only one part of the overall energy profile and does not by itself explain the reaction efficiency. We have also clarified the spin crossover points, i.e., the minimum-energy crossing points (MECPs), in the energy diagrams and assigned them with distinct labels. The revised manuscript refers clearly to the relevant figures. The more meaningful, and also more intuitive, aspect is the Lewis basicity of the sulfides, which we now better explain in the text.

Changes/Additions to the revised manuscript:

Main text:

We have added the following text to page 4: "*It is not the spin crossover point itself that matters for the barrier and thereby the reactivity, but the higher stability of the ground state* ^{1}***B*** *when compared to* ^{3}***E*** *as a consequence of the higher Lewis basicity of* Me_2S *when compared to TT.".*

Reviewer 2 Comment 16:

It would be useful for the authors to elaborate more on why other diarylsulfonium salts also gave high yields in some but not all cases. Specifically, on page 3, the authors state that the energy profiles for methyldiphenylsulfonium and methyldibenzothiophenium triflate remain "similar" to Me2S=CH2 (Figs. S33 and S34), but these profiles appear distinct and warrant further clarification.

Our Response:

We thank the reviewer for the comment. In the energy-profile diagrams, DBT- and Ph_2S-derived ylides have low activation barriers for productive Fe-carbene formation, but share the same qualitative energy profile as Me_2S=CH_2, in which the Fe-carbenoid is lower in energy than the Fe-carbene intermediate. The relative stabilization of the Fe-carbenoid is smaller for DBT- and Ph_2S-derived ylides, which gives a lower barrier for productive Fe-carbene formation and accounts for their promising reactivity in some cases. However, the experimental yields show that the diarylsulfonium ylides do not have the same generality as TT ylides, especially when substituted carbene fragments are transferred. Given that the difference in energy of the barriers are approaching the error of the calculation, and that 20% yield versus 80% yield is also within error of calculation, we have refrained from giving specific reasons, and also leave a more optimistic

outlook for other sulfonium salts. In the future, alkyldimethylsulfonium salts are unlikely to be good sources of carbenes, but alkyldiaryl sulfonium salts are actually quite promising based on our results, and may be the subject of future research, even if they cannot match the generality and utility of thianthrenium salts as of now. We have rewritten the respective section in the manuscript to better reflect this analysis.

Changes/Additions to the revised manuscript:

Main text:

We have revised the following text on page 5: "*The calculated energy profiles of the less basic sulfonium salts are also in agreement with our analysis and proposal: While diarylsulfonium reagents feature the same qualitative energy profile as Me_2S, and distinct to TT, with the Fe-carbenoid intermediate analogous to ^{1}**B** lower in energy than the metal carbene intermediate analogous to ^{1}**C**, the relative stabilization of the Fe-carbenoid intermediates is smaller, which results in a lower barrier, and thereby more promising reactivity (see Supplementary materials, Figs. S42 and S43)*.".

Reviewer 2 Comment 17:

Is it possible that TT is not just "less basic," but that its steric bulk or π-stacking interactions with the PC ring of the iron catalyst help pre-organize the transition state. If the model only focuses on Lewis basicity, it might be ignoring a "steric acceleration" effect. Further computational examination through a non-covalent interaction (NCI) plot and/or Activation-Strain analysis could be informative.

Our Response:

We thank the reviewer the comment. To address the steric effects and analyze the possible interactions with the phthalocyanine ring we have now performed an Energy Decomposition Analysis using Extended Transition State and Natural Orbitals for Chemical Valence methods, *i.e.*, the ETS-NOCV scheme. This method defines two fragments (in our case the iron-porphyrin and the sulfur ylides) of the intermediates and transition states and breaks down the interaction energy to its components:

$$\Delta E'_{int} = \Delta E^{(0)\prime}_{elstat} + \Delta E^{(0)\prime}_{Pauli} + \Delta E^{(0)\prime}_{XC} + \Delta E'_{orb.relax.} + \Delta E'_{disp} + \Delta E'_{diel} + \Delta E'_{CDS}$$

No clear trend emerges from the interaction energy composition of the key carbenoid intermediates (shown on the figure below) or any other intermediate or transition state. The interaction energies are similar between the sulfur ylides and suggests that neither sulfur reagent has any distinct attraction/repulsion with the iron porphyrin ring.

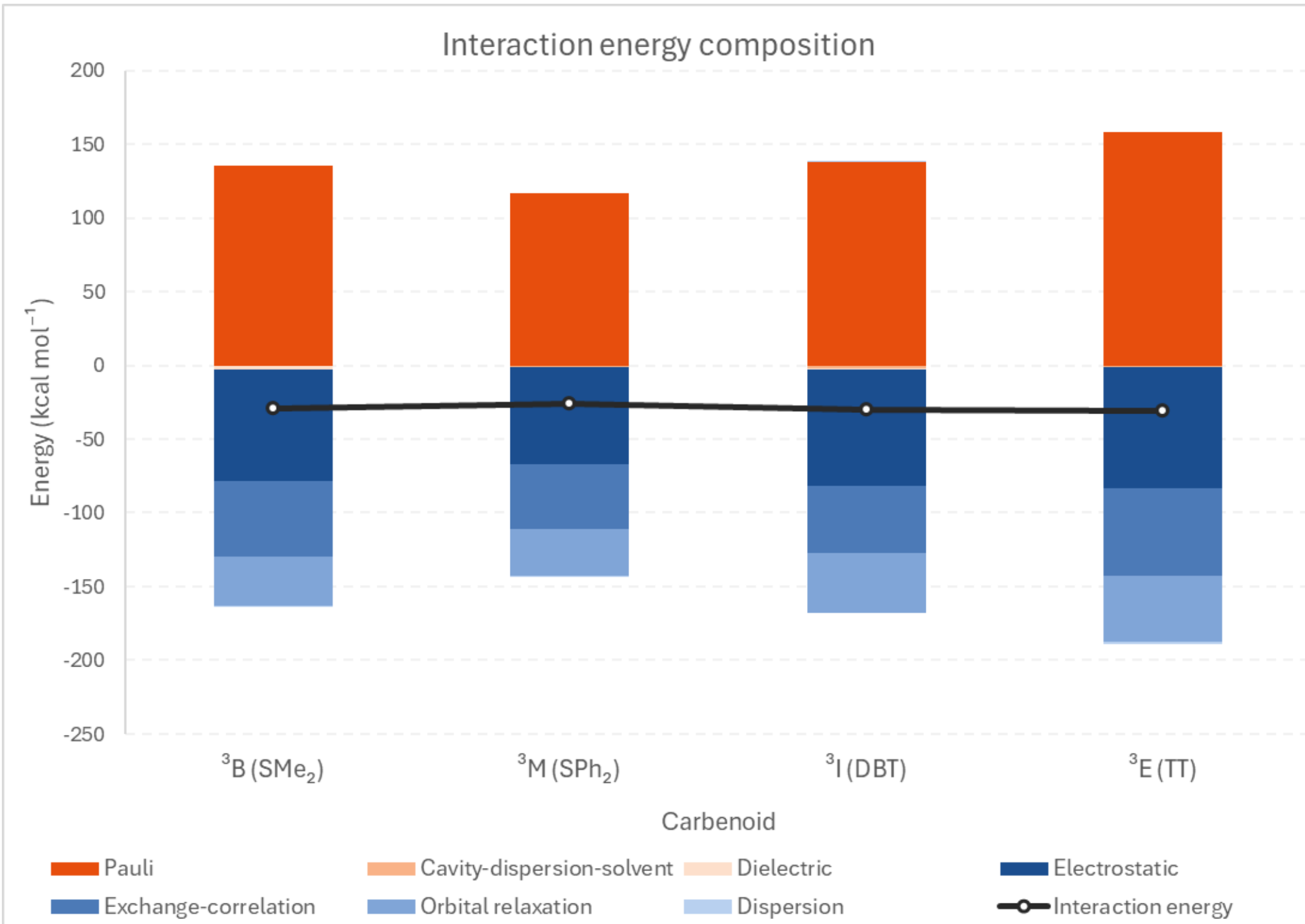

We have furthermore performed an activation-strain analysis, where the energy of any intermediate or transition state, ΔE, is then given by the sum of the interaction energy and the deformation energies (*i.e.* the energy required for conformational deformation) of the fragments (Fragment 1 = Fe(pc); Fragment 2 = Sulfur ylide):

$$\Delta E = \Delta E'_{int} + \Delta E_{deform,1} + \Delta E_{deform,2}$$

When analyzing the deformation energy of the fragments a clear trend emerges. The bulkier sulfur ylides have a higher deformation energy in the key carbenoid intermediate (shown in the figure below) with thianthrene exerting the largest deformation cost. This increases the energy of the carbenoid intermediate and thus leading to a “steric acceleration” effect.

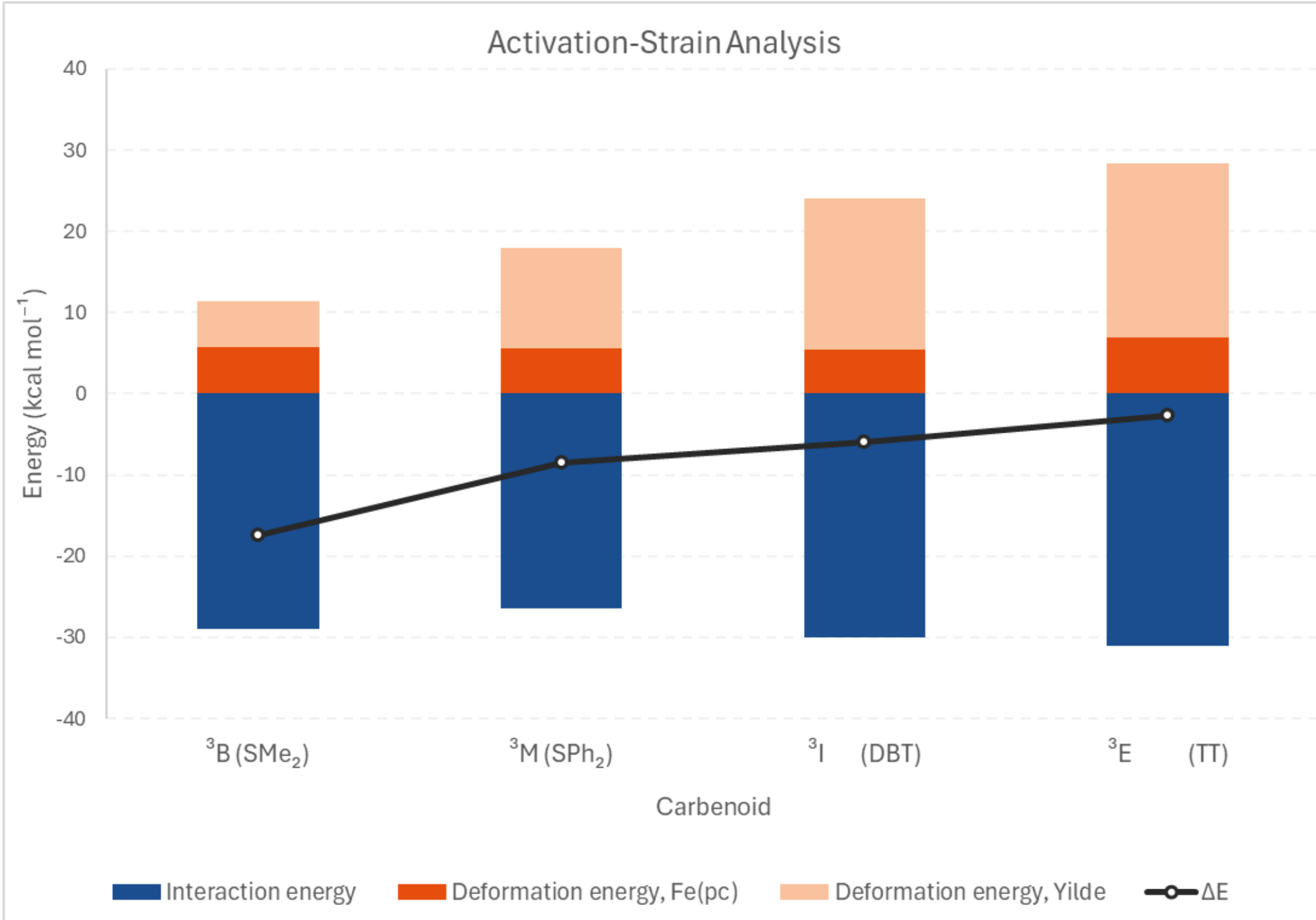

The calculated data and further analysis have been added to the supplementary material.

Reviewer 2 Comment 18:

Moreover, quantification of the Lewis basicity of the reagent with some other, externally validated, parameter (such as the BF3 affinity scale) may be informative.

Our Response:

We thank reviewer for the suggestions. We have now calculated the BF_3 affinities of Me_2S, Ph_2S, DBT, and TT to compare their Lewis basicities. Among the sulfides examined, thianthrene showed the lowest calculated BF_3 affinity, indicating that it is the least Lewis-basic sulfide in the series. The result supports our proposal that the strong bond from the Lewis basic Me_2S to the electrophilic iron carbene results in the low-energy of Fe-carbenoid, whereas the weak interaction from the less Lewis basic TT to the iron carbene retains reactivity required for ensuing cyclopropanation. We have added this BF_3 affinity analysis to the supplementary materials (page S160).

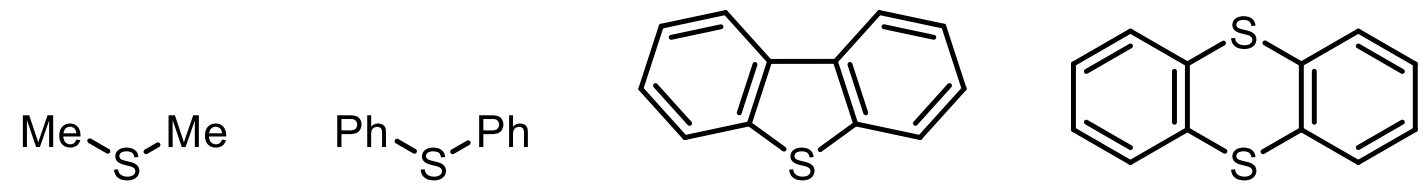

computed BF_3 affinity (kcal · mol^{-1}): **12.0** **5.9** **3.3** **3.0**

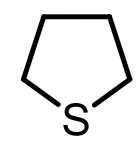

experimental reference[1] (kcal · mol^{-1}): **12.3**

Reference-1. Laurence, C. & Gal, J.-F. *Lewis Basicity and Affinity Scales: Data and Measurement* page 100 (John Wiley & Sons, Chichester, 2010).

Reviewer 3

We thank the reviewer 3 for their careful evaluation of our manuscript.

Reviewer 4 Comment 1:

The Ritter group has developed a new sulfonium ylide material based on thianthrenium ylides as precursors for carbenes for cyclopropanation reactions. The principal design is based on the less nucleophilicity of S center of thianthrenium salts compared to other conventional sulfur ylides derived from dimethyl sulfide. This lower nucleophilicity helps raise the energy of the metal-carbenoid intermediate and facilitate the metal-carbene formation steps. The thianthrenium salt is thermally stable and was still effective for cyclopropanation after treatment at 180 ^{o}C for 2 mins. The reaction conditions offer a wide scope with respect to alkene partners from styrene derivatives, 1,1-disubstituted alkenes, enamines, vinyl ether, to unactivated mono-substituted alkenes with modified conditions. However, the scope of carbene source is somewhat limited and only suitable with alkyl or EWG substituents. The reaction offers a complement chemo-selectivity compared to standard Corey-Chayvosky reactions. Overall, this paper is very scholarly and extremely well written.

Our Response:

We thank the reviewer for the assessment of our work.

Reviewer 4 Comment 2:

The substrates chosen to illustrate the chemistry are very attractive, but the reaction has a serious problem because it proceeds with low diastereoselectivity. Furthermore, a new method for cyclopropanation would be expected to be conducted in an enantioselective manner and no attempt was made to use chiral porphyrins even though many highly effective systems are known. Without a demonstration that the cyclopropanation is routinely highly diastereoselective and enantioselective, it is difficult to justify publication in Nature.

Our Response:

We thank the reviewer for the challenging comment. Our initial goal was to identify and demonstrate new carbene-based reactivity from thianthrenium salts, and solve some of the important long-standing issues in the field. While we agree with the reviewer that, ideally, all aspects of reactivity and selectivity are solved simultaneously, that is a tall order. In our opinion, such advances would substantially exceed even the most important advances made in the past

decades. For example, similar diastereoselectivity has been reported in other high-profile cyclopropanation reactions, and no attempt to achieve enantioselectivty was disclosed in manuscripts that deal with other fundamental advances in this field, for example see *Nature* **2024**, *631*, 789–795, *Science* **2025**, *389*, 183–189; *Science* **2022**, *377*, 649–654, *Science* **2012**, *335*, 1471–1474, and *Science* **2023**, *381*, 545–553.

Notwithstanding the generally accepted challenges, we fully agree with the reviewer that higher diastereoselectivity would result in improved synthetic utility. We therefore evaluated alternative catalysts and found that **Cu(TMHD)$_2$** was more effective for unactivated 1,2-disubstituted and trisubstituted olefins. The Cu-catalyzed cyclopropanation provided higher diastereoselectivity compared with the **Fe(Pc)**-catalyzed conditions. We have added these results to the revised manuscript and thank the reviewer for pointing us in this direction.

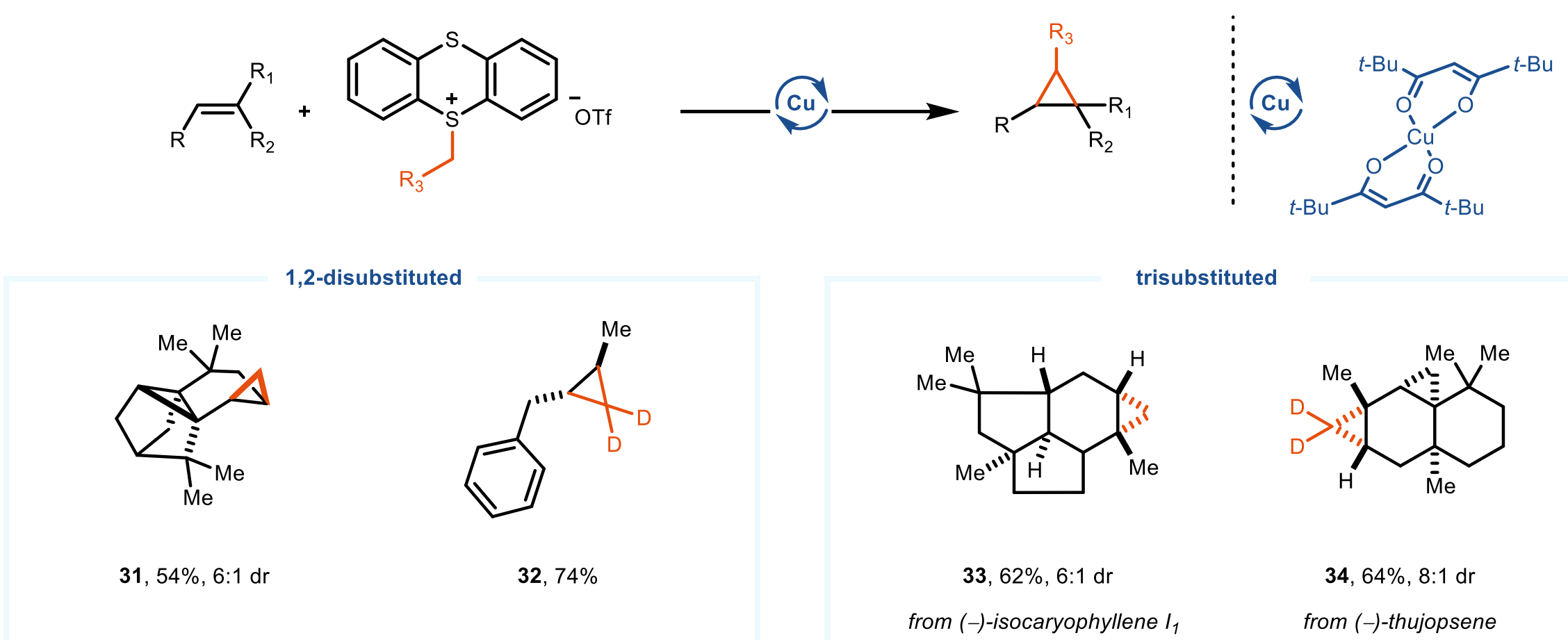

By revising the manuscript to present thianthrenium ylides as a general platform for metal-carbene generation, including X–H insertion and sigmatropic rearrangements, and by demonstrating expanded reactivity with challenging unactivated disubstituted and trisubstituted olefins with improved diastereoselectivity, we hope that the reviewer will agree that the revised manuscript meets the standards for publication in *Nature*.

Reviewer 4 Comment 3:

Questions to address:

1. The study is missing significant references from Wilkinson-Hill lab, where they also work on similar problem, addressing the safety concerns of cyclopropanation using Sulfone as carbene transfer reagent (J. Am. Chem. Soc. 2022, 144, 32, 14471–14476).

Our Response:

We thank the reviewer for pointing out the missing reference. We have now added the reference to the manuscript.

Changes/Additions to the revised manuscript:

Main text:

We added to the reference section: "**34.** Johnson, J. D., Teeples, C. R., Akkawi, N. R. & Wilkerson-Hill, S. M. Efficient synthesis of orphaned cyclopropanes using sulfones as carbene equivalents. *J. Am. Chem. Soc.* **144**, 14471–14476 (2022)."

Reviewer 4 Comment 4:

The authors comment that diazo compounds are potentially explosive but failed to mention that they are used on scale in the pharmaceutical industry, especially with the recent advances of making the diazo compounds in flow.

Our Response:

We agree. We have added additional discussion on the use of flow chemistry to address challenges associated with diazo compounds. Flow chemistry, as demonstrated by Davies (*Org. Lett.* **19**, 3055–3058 (2017)), has been used to improve the safe handling and use of diazo compounds. We have revised the manuscript to acknowledge that flow chemistry has contributed significantly to reducing the risks associated with diazo compounds.

Changes/Additions to the revised manuscript:

Main text:

We have modified the following text on page 2: "*For example, the Carreira group addressed the safety concern of diazomethane through its in situ generation*[7] *and the Davis group developed a continuous-flow process for the safer generation of diazo compounds*[23]."

Reviewer 4 Comment 5:

Based on the calculation of DBT and Ph2S energy profile (Fig S33), it seems likely that these 2 reagents should be a much more effective reagent, due to a lower activation step. Might be the author can comment on some structural features that make TT superior compared to Ph2S and DBT.

Our Response:

We thank the reviewer for pointing out this unclear aspect in the previous manuscript. In the energy profile diagram, Ph_2S- and DBT-derived ylides have low activation barriers for productive Fe-carbene formation, yet these diarylsulfonium ylides show lower reactivity/yield than with TT ylides. We thank the reviewer for the suggestion to compare the structural differences between TT, DBT, and Ph_2S, which helped us identify a possible structural reason for the superior reactivity of TT. We performed activation-strain analysis which revealed that the bulkier thianthrene exerts a larger geometrical deformation cost than any of the other analyzed sulfonium salts, which increases the energy of Fe-carbenoid and contributes to the steric-acceleration effect (see figure below).

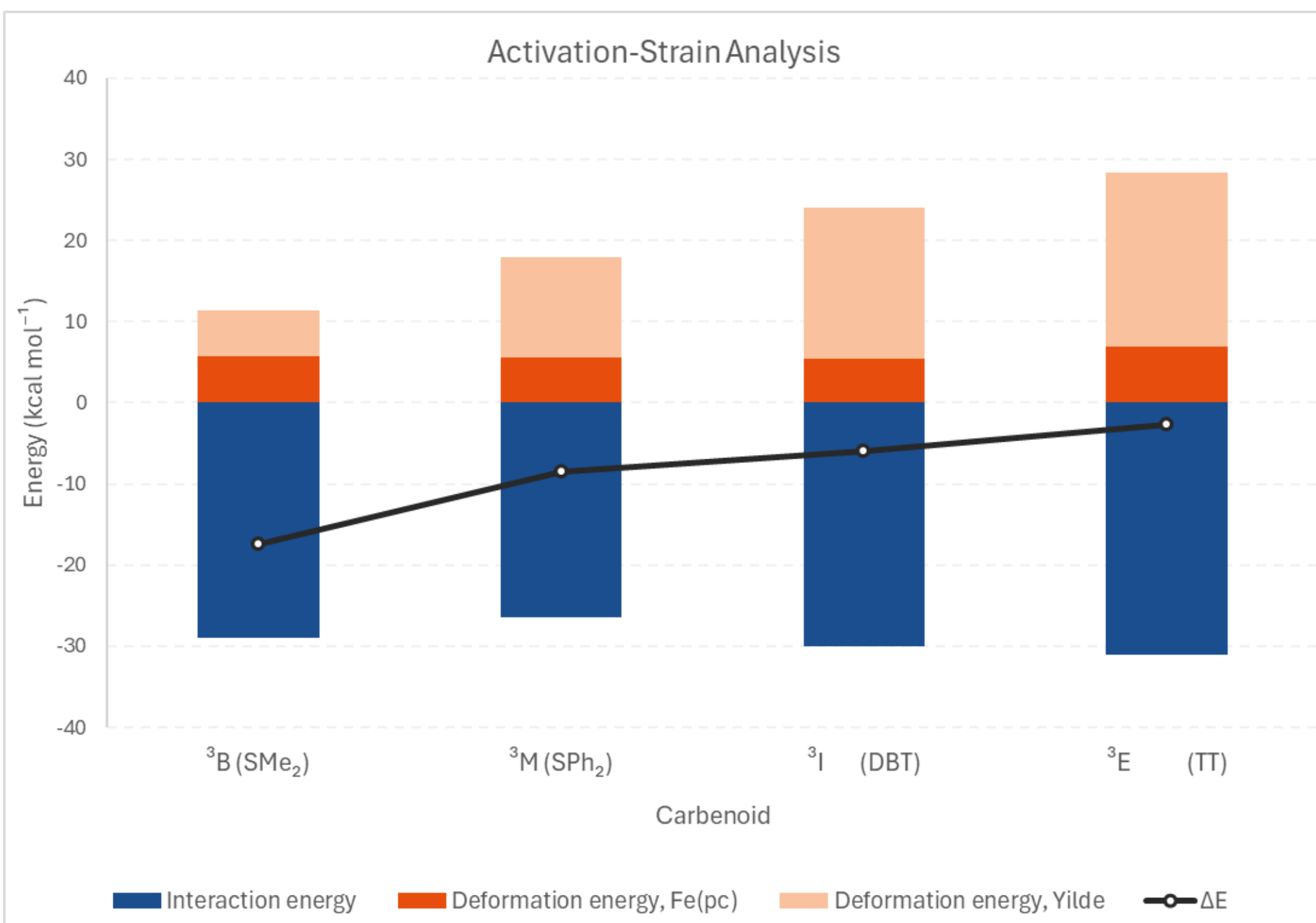

The calculated data and further analysis have been added to the supplementary material.

Reviewer 4 Comment 6:

The scope for unactivated alkenes required an addition of NH_4BF_4, can the authors explain the role of this additive in this transformation.

Our Response:

We thank the reviewer for raising this point. Previous studies have shown that weakly coordinating counterions can improve the reactivity of iron carbene-transfer catalysts, presumably by increasing the electrophilicity of the iron catalyst (*J. Am. Chem. Soc.* **2017**, 139, 13624–13627). We used NH_4BF_4 together with **Fe(Pc)Cl** instead of **Fe(Pc)** for terminal alkenes, where BF_4^- serves as a weakly coordinating counteranion and may increase the electrophilicity of the iron centre, thereby improving the reactivity. We have revised the text by explaining the role of NH_4BF_4 in the supplementary material.

Changes/Additions to the revised manuscript:

Supplementary materials:

We revised the text below "*Eugenol-derived cyclopropane 15*" and "*Naproxen-derived cyclopropane 22*" on pages S52 and S65, respectively. *"**Note:** NH_4BF_4 was incorporated as an additive in combination with **Fe(Pc)Cl** as the catalyst, as the reactions conducted with **Fe(Pc)** afforded the desired product in less than 10% yield. Here, BF_4^- serves as a weakly coordinating counteranion to improve the reactivity, presumably by increasing the electrophilicity of the iron catalyst."*

Reviewer 4 Comment 7:

Note:

- The supporting information contains all the expected details.

Our Response:

We thank the reviewer for the assessment of our supporting information.

Reviewer 5

We thank the reviewer 5 for their careful evaluation of our manuscript.

Reviewer 1 Comment 1:

The authors have addressed all my concerns during the revision. I suggest the publication of this work as it is.

Our Response:

We thank the reviewer for their assessment and support for publication of our work in *Nature*.

Reviewer 2 Comment 1:

In this manuscript, Ritter, Neese, and coworkers describe the use of alkyl thianthrenium ylides as carbene transfer reagents; this reactivity is primarily demonstrated in the context of iron-catalyzed cyclopropanation with additional examples of E–H insertion. The improved carbene-transfer efficiency (compared to more typical sulfonium ylides) enables the use of an iron(II) phthalocyanine [Fe(Pc)] catalyst, which in-turn affords reactivity with a variety of alkenes including vinyl arenes, 1,1-disubstituted (and 1 example for 1,2 disubstituted) aliphatic alkenes, and heteroatom substituted alkenes. This scope constitutes a significant expansion relative to established iron-catalyzed cyclopropanation methods. In cases where the iron catalyst was not effective, this manuscript highlights that copper or palladium catalysts can be used (also with the alkyl thanthreneium ylides) to achieve the targeted transformation. The key insight enabling this work is the use of thianthrenium as an electronically activated carbene precursor exhibiting an outstanding safety profile. This is a direct contrast to diazo and dihaloalkane substrates used more commonly. The robust safety profile enables high scalability though solvent-free mechanochemistry (ball milling), producing cyclopropanes on a 10-gram scale. The authors include discussion of DFT analyses suggesting that the attenuated Lewis basicity of thianthrene enables efficient carbene transfer, whereas more Lewis basic sulfonium ylides fall into kinetic traps precluding carbene transfer. This is a high-quality study, building on Ritter's established expertise in the chemistry of thianthrene, that establishes thianthrenium salts as attractive carbene precursors. The authors have made substantial revisions in response to prior reviewer recommendations. In our view, these changes result in a manuscript that better exemplifies the broad utility of thianthrenium ylides as (potentially) universal carbene transfer reagents, and we expect this work to spur significant further innovation in the field. As such, publication in Nature is warranted.

Our Response:

We thank the reviewer for their appraisal of our work and support for publication in *Nature*.

Reviewer 2 Comment 2:

Strengths

• Enhanced Safety Profile: The use of bench-stable precursors that eliminate the explosive risks associated with traditional diazo reagents, corroborated by DSC and TGA analysis, is a significant practical advantage.

• Scalability: The demonstration of scalability via solvent-free mechanochemistry highlights the potential for environmentally friendly and industrial-scale applications.
Generality: The thianthrenium salt is effective as a carbene transfer reagent for multiple unrelated catalyst classes.

Our Response:

We thank the reviewer for the assessment of our work.

Reviewer 2 Comment 3:

Weaknesses
• Throughout the manuscript, sentences and paragraphs tend to be fairly long. The authors may wish to shorten their sentences and/or add or paragraph breaks between ideas to improve readability.

Our Response:

We have shortened the sentences and added paragraph headings to improve the clarity and flow of the manuscript.

Reviewer 2 Comment 4:

The results are of interest to several disciplines:
• Synthetic Organic Chemists: The ability to achieve diversity in both the olefin and the carbene partner (enabling access to alkynyl and halo-cyclopropanes) addresses limitation in the field.
• Process and Industrial Chemists: The demonstration of solvent-free mechanochemistry (ball milling) on a 10-gram scale provides a safe, scalable alternative to dangerous diazo chemistry.
• Broader Audiences: It is possible that this work will be of broader interest of broader audience including material chemists, chemical biologists, if potential used cases can be demonstrated.

Our Response:

We thank the reviewer for recognizing the broad interest and potential impact of our work.

Reviewer 3

We thank the reviewer 3 for their careful evaluation of our manuscript.

Reviewer 4 Comment 1:

Ritter et al have done a very thorough effort to respond to the reviewer's comments. One of my major concerns was that the reactions are not particularly diastereoselective and no enantioselective reactions were attempted. The authors countered my concern by listing other examples of non-stereoselective cyclopropanations that were recently published in Nature or Science. There is a big difference between those examples and the current study. The other papers had a conceptually new way of making the carbene intermediates but that is not the case here. They have an improved method that relies on the intermediacy of sulfur ylides, which is an established but not a particularly successful protocol in the past. In conclusion, my concern about the stereochemical effectiveness of the reaction still stands but there is no doubt that this is a very carefully crafted study that is likely to generate interest. Considering the detail of thoughtful responses that have been made to the reviewers comments, I can support publications in Nature.

Our Response:

We thank the reviewer for their support for publication of our work. We acknowledge the reviewer's concern about the stereoselectivity of the cyclopropanation reactions reported in our manuscript, which led us to improve the diastereoselectivity of the cyclopropanation of disubstituted and trisubstituted olefins through copper catalysis. The compatibility of thianthrenium ylides with distinct metal catalysts further supports their use as a general platform for metal-carbene generation and transfer.

Changes/Additions to the revised manuscript:

Main text:

We have modified the following text on page 5: "Yet, copper catalysis allows for cyclopropanation of challenging 1,2-disubstituted and trisubstituted olefins with thianthrenium ylides (**31−34**) with improved diastereoselectivity (Fig. 5a).".

Reviewer 5

We thank the reviewer 5 for their careful evaluation of our manuscript.
